# Low Intensity Pulsed Ultrasounds Modulate Adipose Stem Cells Differentiation

**DOI:** 10.1007/s12015-025-10896-7

**Published:** 2025-05-23

**Authors:** Tamara Fernández-Marcelo, Alba Calero, Beatriz de Lucas, María Garrido, Raúl L. Arregui, Paulina Sury, Elena Quintana, Laura M. Pérez, Elisa Fernández-Millán, Beatriz G. Gálvez

**Affiliations:** 1 Department of Biochemistry and Molecular Biology, Faculty of Pharmacy, Universidad Complutense de Madrid, Madrid, Spain; 2 Faculty of Sports Sciences, Universidad Europea de Madrid, Madrid, Spain; 3 CIBER de Diabetes y Enfermedades Metabólicas Asociadas (CIBERDEM), Instituto de Salud Carlos III (ISCIII), 28029 Madrid, Spain

**Keywords:** Adipose-derived stem cells, Inflammation, Low-intensity pulsed ultrasound, Adiponectin, Obesity

## Abstract

LIPUS, low intensity pulsed ultrasounds, are considered a safe and non-invasive tool that have been used extensively in medicine for chronic diseases. We evaluated the effects produced by LIPUS on the physiological behaviour of mouse and human adipose stem cells (ASCs) as well as on adipose tissue explants. LIPUS stimulation for one minute did not affect mouse or human ASC proliferation or apoptosis, with no evident changes in morphology or cell growth. Further, RNA-seq analysis revealed more than 200 genes differentially expressed after ultrasound stimulation of mouse and human ASCs. Among them, the changes in gene expression mainly belong to the canonical pathways of stemness, energy metabolism or chemokine signalling. On the other hand, the migration ability of mouse and human ASCs was partially affected by the ultrasound protocol, slightly increasing their capacity to migrate, while maintaining their stemness properties. Major affection was detected on the adipocyte differentiation capacity. Indeed, LIPUS-treated ASCs were unable to differentiate into mature adipocytes and their inflammatory secretion profile was reduced in both mouse and human ASCs. These effects were confirmed on an ex vivo model of adipose tissue explants, demonstrating that LIPUS also provoked an anti-inflammatory profile in the adipose tissue while reducing adipocytes differentiation and lipids content. Overall, the strong adipogenesis blockade, the reduction of their secretion profile and the RNA-seq analysis suggest that LIPUS-treated ASCs may be prevented from differentiating into mature adipocytes and from exacerbating inflammation, thus limiting their contribution to obesity.

**Graphical Abstract:**

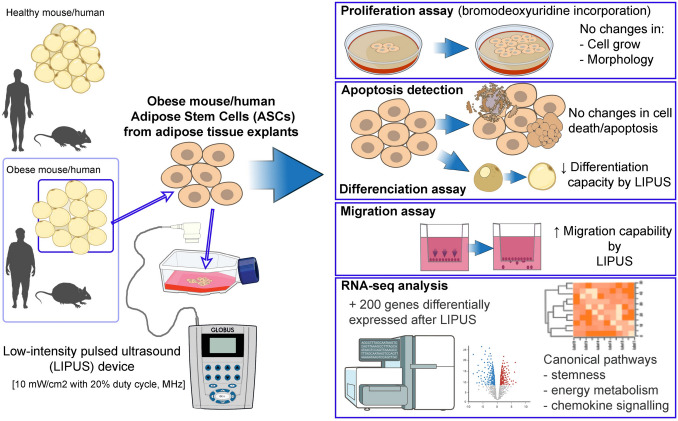

**Supplementary Information:**

The online version contains supplementary material available at 10.1007/s12015-025-10896-7.

## Introduction

The escalating global obesity epidemic represents one of the most important challenges facing public health today as it plays a role in the increasing occurrence of associated comorbidities. World Health Organisation (WHO) defines obesity as a body mass index (BMI) > 30 kg/m^2^ and overweight as a BMI between 25 and 30 kg/m^2^. The prevalence of obesity is increasing in every single country of the world [1–3]. By 2035, the global overweight and obesity prevalence is projected to reach 51%, with South Pacific Islands leading the course of the obesity epidemic [3]. Conversely, while the overall obesity rates are leveling off, there is a noticeable increase in the number of individuals falling into higher obesity categories, which is associated with a rise in various health conditions [1, 2] and significant economic consequences [4].

Obesity is a complex, multifactorial condition associated with an increase in several inflammatory markers. [5] but also variable across species and tissue types influenced by a variety of factors including gender, race, ethnicity, age of onset and genetics [6–9]. High fat feeding and other metabolic stressors disrupt corporal energy balance and metabolic homeostasis, causing dramatic changes in adipose morphology, physiology, and cellular composition [10] as well as an altered cytokine profile, leading to systemic inflammation and dyslipidemia [3, 11]. All these changes cause an increase in related health complications such as insulin resistance, type 2 diabetes, joint problems, sleep and breathing disorders, some types of cancer as well as cardiovascular, liver or neurodegenerative and mental diseases among other conditions [12–19].

In humans, WAT becomes the predominant adipose tissue type in adulthood [20–23], composed of a heterogeneous group of cell types, including preadipocytes, fibroblasts, endothelial cells, pericytes, immune cells, and stem cells, that collectively form the adipose tissue stromal vascular fraction (SVF) [10, 21, 24]. The proportions of these cellular subtypes can vary based on pathological and physiological metabolic processes [11, 25].

During periods of caloric excess, increased energy intake results in energy being stored in adipose tissue and in non-adipose tissues (ectopic lipids) [8]. Excesses of lipids in adipocytes usually are stored intracellularly within lipid droplets (LDs), that are a hallmark of differentiated and functional adipocytes [22].

Adipose tissue demonstrates significant adaptability [26–28]; however, it can be influenced by what some researchers refer to as an “*obesogenic environment*" [29]. In response to excessive and chronic state of overnutrition, adipocytes primarily undergo hypertrophy (instead of hyperplasia process) and preferentially localized in visceral depots [30, 31], ultimately fall under a hypoxic state [32] after expanding to a limited size, [33], probably due to anomalous lipid accumulation that exceeds the storage capacity of lipid droplets. As consequence, this can lead to endoplasmic reticulum stress and ultimately alters the internal balance of fatty acids within the cell, resulting in the release of harmful lipid species in a phenomenon known as lipotoxicity [22, 23, 26, 27, 29, 34].

Adipose tissue also secretes a macrophage chemoattractant that can accelerate the migration of macrophages and promote infiltration into adipose tissue, such as chemokines like chemoattractant protein-1 (MCP-1/CCL2) and HP (haptoglobin) [22, 27, 35]. This promotes more and more macrophage accumulation because of an inefficient and dysfunctional adipocyte elimination [22]. Inflammation promotes infiltration of other types of immune cell, like C–C motif chemokine ligand 5 (CCL5), induced by TNF-alpha via the NF-kB pathway, that initiates the infiltration of T cells [36, 37].

Adipocytes also lead to fibrotic remodelling of the extracellular matrix. Mature adipocytes and adipose stem cells transdifferentiate into myofibroblasts that aggregate, and a large amount of extracellular matrix is deposited in adipose tissue [38]. These events ultimately lead to ectopic lipid deposition, glucotoxicity, capillary rarefaction [39], and other metabolic complications with life-threatening consequences like insulin resistance [27, 40, 41].

In addition, autocrine and paracrine regulatory loops involving angiotensin as proinflammatory and adiponectin as an anti-inflammatory signal can further modulate the cross talk between adipocytes and adipose tissue macrophages [29, 42] that decreased under conditions of obesity, insulin resistance, and type II diabetes [42, 43]. Obesity is also well-associated with the production of leptin [42, 44], a pleiotropic adipokine, that has a pivotal role in energy homeostasis and produces various effects throughout the body [45]. It has also been identified as an important immune modulator with a wide range of functions, many of which are pro-inflammatory [44, 46] and exacerbates immune dysfunction [34, 47, 48]. As a result, there is a generalized and chronic elevated level of inflammatory markers such as IL-6, TNF-alpha, IL-8 or IL-1beta [5, 49, 50] that can drive tissues into a senescent state, characterized by reduced proliferation and altered secretory profiles, also named as *adipoaging* [51].

Adipose stromal/stem cells (ASCs) are multipotent mesenchymal stromal/stem cells that represent a promising tool for tissue regeneration applications as well as for cell-based treatment of inflammatory and autoimmune conditions [52]. ASCs have been widely studied due to their important properties that have made them suitable candidates for use in different therapies. They are relatively easy to obtain from many cell depots and are endowed with important therapeutic properties including immunomodulatory potential, regeneration, and secretory and migratory functions [53, 54]. It is already known that the complex cellular and molecular environment that occurs in obesity can also alter the functionality of the ASC reservoir, giving rise to an inflammatory phenotype and a decrease in its regenerative potential [55]. Due to the changes undergone by adipocytes in the obese environment ASCs attempt to counteract it by differentiating into mature adipocytes, although they are also affected by this environment [56].

In fact, ASCs obtained from obese donors would not be good candidates for use in therapies [57, 58]. A limitation for the use of ASCs as a therapy is their propensity to differentiate to adipocytes, in some cases with abnormal metabolism, and provoke malfunctioning of the system and drive to obesity development. It is then a priority to development new strategies to overcome these challenges.

**Fig. 1:**
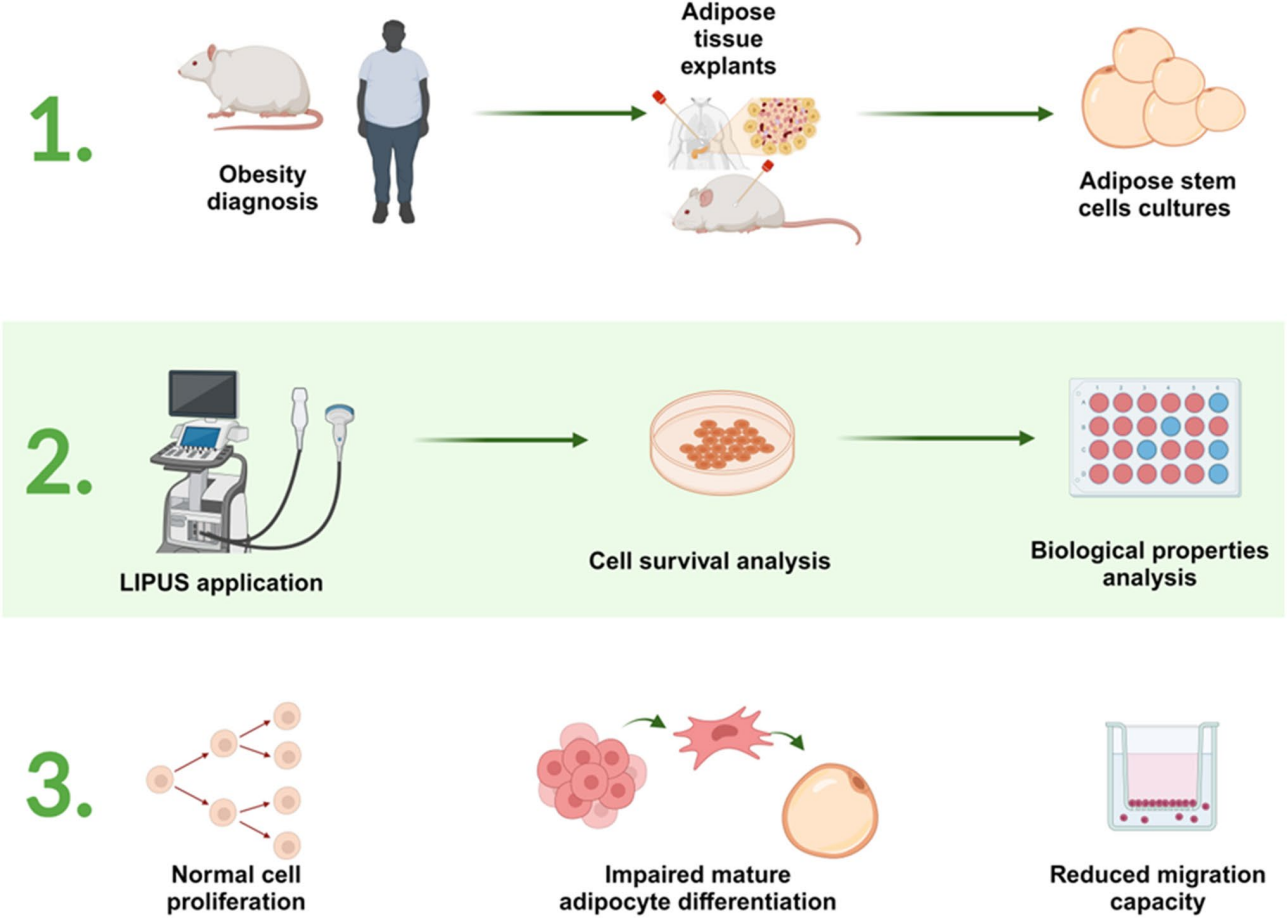
The use of LIPUS on ASCs and the impact on their physiological properties

Low-intensity pulsed ultrasound (LIPUS), a specific form of ultrasound, has been approved for over two decades for bone fracture healing [59, 60]. LIPUS have been shown to stimulate cell proliferation [61, 62], migration [63, 64], production of extracellular components [65] and angiogenesis [66] in different soft tissues with minimal secondary effects [5, 67, 68]. More recently, LIPUS have also been used as a therapeutic strategy for various disorders (bone fractures, kidney stone ablation among others) [59, 69, 70]. Indeed, new applications for ultrasound are continually evolving not only in the biomedical field but also in others such as the food industry [71–73]. Due to the great potential of LIPUS, we aimed to study whether ultrasound waves generated by a conventional device, at the lowest intensity setting, could impact on the main physiological functions ASCs and in the future being developed as a biomedical tool to fight against obesity and metabolic diseases (Fig. 1).

## Methods

### Culture of Mouse and Human Adipose Stem Cells

Mouse stable cell lines of ASCs, previously derived from adipose tissue explants of obese mice by our group as [49] and in accordance with all the approved ethics committee, were used for all the experiments. Commercial human adipose stem cells vials (Ref: PT-5006) were acquired from Lonza (Switzerland), which counts with the corresponding ethical approval and informed consents to be used for research purposes only. Both mouse and human ASCs were incubated and maintained at 37 ºC with DMEM (Gibco, USA) + 10% FBS (Gibco, USA) and then they were sub-cultured under the same conditions. Cultures up to 20 passages were used in the experiments.

### Reagents

The cells were grown in Dulbecco’s modified Eagle’s medium from Sigma (St Louis MO) supplemented with 10% FBS from Sigma (St Louis, MO), Pen/Strep, L-glutamine and HEPES from Lonza (Basel, Switzerland). Matrigel Basement Membrane Matrix Phenol Red Free was purchased from BD biosciences (Franklin Lakes, NJ). TRANSWELL filters were from Corning Incorporated (Acton, MA) and all the cytokines used were obtained from Peprotech Inc. (Rocky Hill, NJ).

### Adipose Tissue Explants

To isolate adipose tissue explant from mouse WAT discarded tissue we followed previously published protocol [74]. Briefly, the adipose tissue was dissected into small, uniformly sized pieces. Each piece was plated onto a well of p24w plate containing 500 ml of complete DMEM. Culture dishes were incubated at 37ºC and 5% CO2 for 1–2 h and experiments were performed after checking integrity under a microscope.

### Ultrasound Application

Application of therapeutic ultrasounds LIPUS to cells was performed using the Medisound 3000 device (Globus, Codognè, Italy), which is approved by the EU for use in hospitals and physiotherapy clinics. A total of 1.5 × 10^4^ cells were seeded in each well of a 24-well plate and were maintained for 24 h in the humidified incubator before stimulation. Application of LIPUS to ASCs was performed using the following parameters: 10 mW/cm^2^ intensity and 3 MHz frequency. The LIPUS protocol consisted of 1 min exposure with an ultrasound pulsed at 20% duty-cycle. LIPUS was applied outside of the incubator at room temperature on the bottom of the LIPUS-treated plates Control cultures grown on different plates were treated identically (without LIPUS stimulation). Once the daily application was completed, the cells were returned to the incubator and the next day the different experiments were performed.

Application of LIPUS to adipose tissue explants was performed using similar parameters to cell cultures: 10 mW/cm2 intensity and 3 MHz frequency for 1 min (20% duty-cycle). LIPUS was applied outside of the incubator at room temperature on the bottom of a p24 wells containing adipose tissue explants to be stimulated. In parallel, a p24 wells plate with adipose tissue explants without LIPUS stimulation was processed.

### Proliferation Assay–bromodeoxyuridine Incorporation

The bromodeoxyuridine (BrdU) assay (Merck KGaA, Darmstadt, Germany) was used for proliferation analysis. Briefly, 1 × 10^4^ cells were seeded in each well of a 24-well plate. The following day, LIPUS stimulation was applied to the corresponding wells in contrast to control wells, and BrdU (1:2000 dilution) was added to the cultures and cells were maintained for 24 h. Cells were then fixed and washed and incorporated BrdU was detected with an anti- BrdU antibody (1:100, 1 h incubation at room temperature), which was visualized with an HRP-conjugated secondary antibody (1:1000, 30 min at room temperature). Finally, after washing, the chromogen substrate was added for 30 min in the dark for the development of the peroxidase reaction. Once the STOP solution was added, the optical density was read in a spectrophotometer (SPECTROstarNano; BMG LABTECH, Aylesbury, UK) at 450 nm.

### Apoptosis Detection

Annexin V Alexa Flour 488 and PI Dead Cell Apoptosis Kit (Thermofisher Scientific) were used to detect apoptosis, cell death and live cells following the manufacturer instructions. Briefly, LIPUS-stimulated and control mouse and human ASCs were harvested and resuspended in 1 × annexin-binding buffer containing 5 µL of Alexa Fluor™ 488 Annexin V (Component A) and 1 µL 100 µg/mL PI working solution. Cells were incubated at room temperature for 15 min and after the incubation period, 400 µL of 1 × annexin-binding buffer were mixed gently, and samples were kept on ice before being analyzed by flow cytometry (FACsScan Beckon Dickinson).

### RNA-Sequencing

RNA-sequencing library preparation and sequencing of the mouse and human cell samples was carried out by STABVida Lda (Caparica, Portugal). RNA integrity was checked on a Bioanalyzer 2100 (Agilent Technologies, Santa Clara, CA, USA). The Kapa Stranded Total RNA and Ribo-Zero Library Preparation Kit were employed for library construction, and sequencing was performed using the HiSeq 4000 Illumina Platform with 2 × 150 bp paired end reads. The bioinformatics analysis of the generated raw sequence data was carried out using CLC Genomics Workbench 11.0.1. Further quality control was performed by principal component analysis (PCA), hierarchical clustering (considering Manhattan distance), and heat map analysis. Differential expression was then calculated using multi-factorial statistical analysis based on a negative binomial model that used a generalized linear model approach influenced by the multi-factorial EdgeR method. The differentially expressed genes were filtered using standard conditions, a *p*-value less than 0.05 and fold change over 3 or under −3. Microarray data were analyzed using Ingenuity Pathway Analysis software for detection of canonical pathways or tissue’s function.

### *In vitro* Scratch Healing Assay

To evaluate collective migration, we used the in vitro scratch healing assay. Confluent mouse and human ASCs cultured on 6-well plates were stimulated with LIPUS or not (control cells) and immediately after scratch-wounded with a sterile micropipette tip, washed with PBS, 0,1 M, pH 7,5 (Gibco, USA) to remove cellular debris, and replenished with complete medium. Cells were maintained in culture and images were captured at different times using a Motic AE31 microscope (Motic, Hong Kong, China) to follow the closure of the scratch. The calculation of the wound area was performed with ImageJ software (Bethesda, MD, USA). The results were expressed as size of wound width during time.

### TRANSWELL Migration Assay

To evaluate individual migration, we used TRANSWELL chambers (Corning Inc., MA, USA) with 6.5 mm-diameter permeable membranes and 8-μm pore size filters. Murine (2 × 10^4^) and human (5 × 10^4^) ASCs, previously LIPUS-stimulated or not (control cells), were plated in 80 μl of medium in the upper chamber of the TRANSWELL chamber (placed on 24-well plates) and complete culture medium was placed in the lower chamber. After 8 h, chambers were fixed with 4% glutaraldehyde for 1 h and then stained overnight with 1% toluidine blue, both from (St Louis, MO). Cells on the lower side of the membrane were visualized with a Motic AE31 microscope and counted in five randomly- selected 10 × fields using ImageJ software (Bethesda, MD, USA). The results were expressed as migrated cells per field.

### Differentiation Protocol

To induce adipogenic differentiation, mouse and human ASCs were cultured in serum-free DMEM/F12 medium (GIBCO, USA) (1:1) supplemented with 10 mg/mL transferrin, 15 mmol/L NaHCO3, 15 mmol/L HEPES (GIBCO, USA), 33 mmol/L biotin, 17 mmol/L pantothenate, 1 nmol/L insulin, 20 pmol/L triiodothyronine, and 1 mmol/L cortisol, plus antibiotics. Accumulation of triglycerides in adipocytes was visualized by staining formalin-fixed cells with Oil Red O from Sigma (St Louis, MO). Triglyceride accumulation was assessed microscopically, and Oil Red O concentration was quantified spectrophotometrically at 510 nm. Lipid content was also analysed enzymatically with a triglyceride determination kit both in ASCs and in adipose tissue explants (St Louis, MO).

### Quantitative PCR

Total RNA was extracted from mouse and human ASCs, LIPUS stimulated and unstimulated cells, using the Easy-spin Total RNA Extraction Kit (iNtRON Biotechnology, Sangdaewon-Dong, South Korea) and its concentration was quantified in a spectrophotometer (ND1000 NanoDrop, Thermofisher Scientific, Rockford, IL, USA). RNA was reverse transcribed to cDNA using PrimeScript™ RT Master Mix (TAKARA Bio. Inc., Kusatsu, Japan). Quantitative PCR (qPCR) was performed using SYBER1 Green PCR Master Mix (Premix Ex Taq™, TAKARA Bio. Inc.) on the CFX96 Touch Deep Well™ Real-Time PCR Detection System (Bio-Rad Laboratories, Richmond, CA, USA). Thermal cycling parameters were as follows: first step of 94˚C for 10 min, then 40 cycles of 94˚C for 15 s and the primer- specific annealing temperature for 1 min (56˚C). The last step was the melting curve analysis. qPCR was performed using the following primers: *IRS1* (forward [Fw], CTTCTGTCAGGTGTCCATCC; reverse [Rv], CTCTGCAGCAATGCCTGTTC), *IRS-2* (Fw, ACAATGGTGACTACACCGAG; Rv, CTGCTTTTCCTGAGAGAGAC), *C/EBP-a* (Fw, TTACAACAGGCCAGGTTTCC; Rv, CTCTGGGATGGATCGATTGT), *C/EBP-b* (Fw, ACCGGGTTTCGGGACTTGA; Rv, GTTGCGTAGTCCCGTGTCCA), *Pref-1* (Fw, AGCTGGCGGTCAATATCATC; Rv, AGCTCTAAGGAACCCCGGTA), *PPAR-g* (Fw, ATTGACCCAGAAAGCGATTC; Rv, CAAAGGAGTGGGAGTGGTCT), *aP2* (Fw, AACCTTAGATGGGGGTGTCCTG; Rv, TCGTGGAAGTGACGCCTTTC).

### Preparation of Adipocyte-Conditioned Media and Measurement of Adipokine Release

Mouse and human ASCs, LIPUS stimulated and unstimulated cells, after seven days of differentiation into mature adipocytes were cultured overnight in serum-free medium, and the medium was retained as adipocyte-conditioned media. Adipokines were measured using Luminex xMAP technology with multiplex immunoassays (LincoPlex) for simultaneous quantitative determination of adiponectin, Il-6 and TNF-alpha (Millipore), with detection limits of 75.2, 0.8, and 0.09 pg/mL, respectively.

Mouse adipose tissue explants media derived from LIPUS stimulated and unstimulated explants, was collected after 24 h and simultaneous quantitative determination of adiponectin, Il-6 and TNF-alpha (Millipore) was also performed.

### Data Analysis

Statistical analysis and graphical representation of the results were performed using GraphPad Prism software (GraphPad Software Inc., San Diego, CA, USA). Values are expressed as mean ± standard deviation (SD) from 3 independent experiments. Data were checked for normality using the D’Agostino-Pearson test. Comparisons between groups were performed with one-way or two-way analysis of variance (ANOVA). The multiple comparisons test used for one-way ANOVA was Bonferroni’s and for two one-way ANOVA we used Tukey’s. Student’s t test was used when there was only one variable to consider. The specific analysis used is specified in the figure legends. Data were considered significantly different when *p < 0.05.

## Results

### Low-Intensity Ultrasound Stimulation does not Affect Cell Proliferation and Viability

**Fig. 2:**
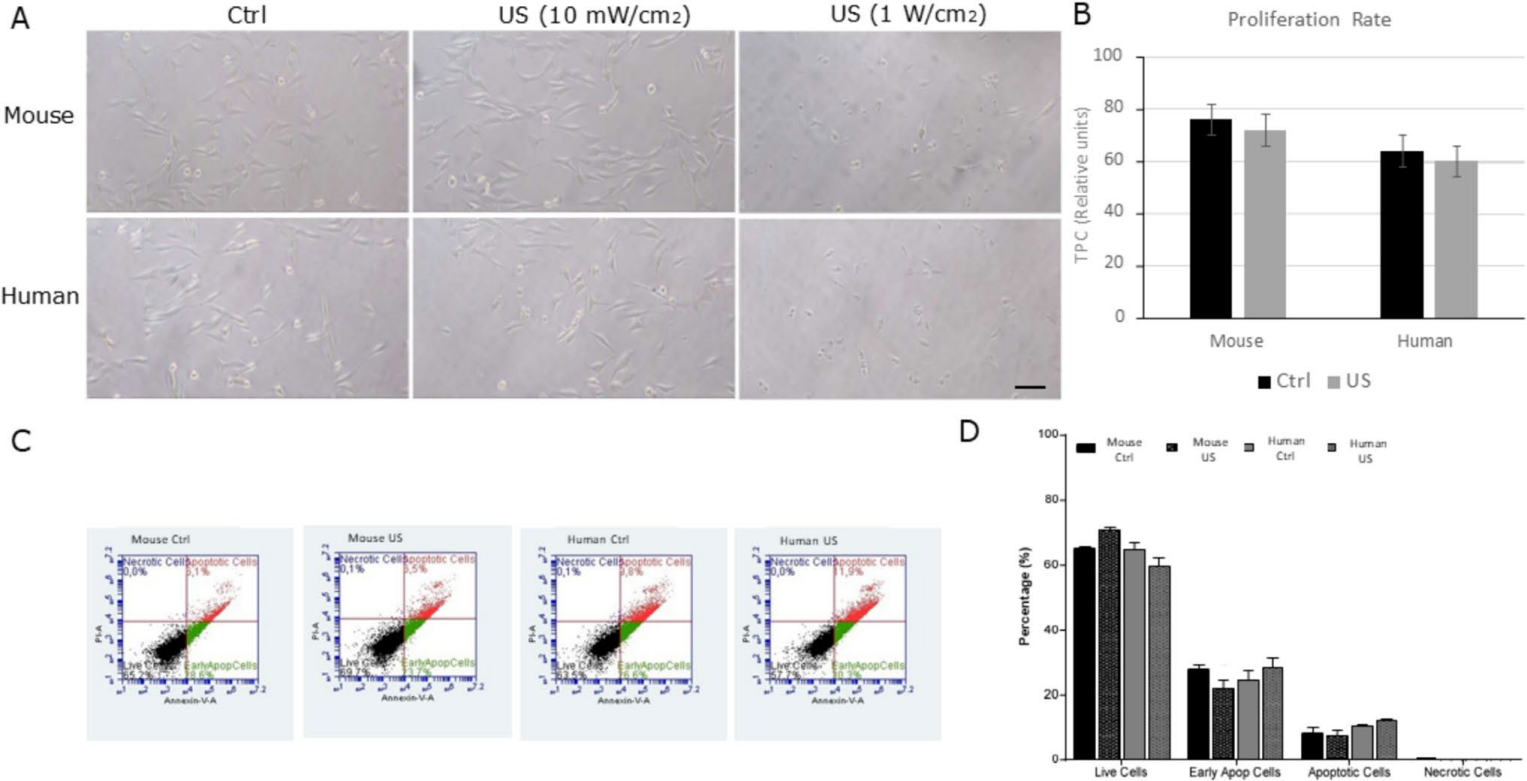
Application of LIPUS. (**A**) Representatives phase contrast images of mouse and human ASCs showing control cells and LIPUS- stimulated (US) cells with two different dosages before and after 3 days of treatment. (**B**) Cell proliferation was evaluated by BrdU incorporation. Data are shown from a representative experiment out of three performed and denote mean ± SD. No significant differences were found between control conditions or treated with ultrasound (US). (**C**) Cell apoptosis, cell death and live cells were measured by flow cytometry with the Annexin V Alexa Flour 488 and PI Dead Cell Apoptosis Kit. The graphs show a representative experiment of three different performed with mouse and human cells. (**D**) No significant differences were found between treated (dose) and untreated ASC for the parameters shown in C) (two-way ANOVA with Tukey’s multiple comparisons test)

Ultrasound exposure (Medisound 3000) was performed on mouse and human ASCs adhered to tissue culture plates at confluence prior to experimental testing. Attached cells were stimulated at 10 mW/cm2 with 3 MHz for 1 min, and a 20% duty-cycle. Cells receiving the ultrasound stimulation were morphologically indistinguishable from control cells (Fig. 2A). As a positive control of LIPUS stimulation, the increase of the intensity to 1 W/cm2 caused the death of the cells (Fig. 2A, right panel).

For cell counting experiments, 1 × 10^4^ cells were seeded in each well of a 24-well plate and counted over three days by measuring bromodeoxyuridine incorporation. The results were expressed as cell proliferation rate representing the total number of cells present on the plate at the final point. No changes in proliferation were observed either on mouse or human ASCs (Fig. 2B).

To further clarify the effect of LIPUS on cell survival, the levels of apoptosis and necrosis were measured by flow cytometry (Fig. 2C) and quantified (Fig. 2D). In this case, LIPUS stimulation did not increase the rates of apoptosis or necrotic mouse or human cells, which were similar to the levels of live cells.

### ASCs Gene Expression Pattern Changes after LIPUS Stimulation

**Fig. 3:**
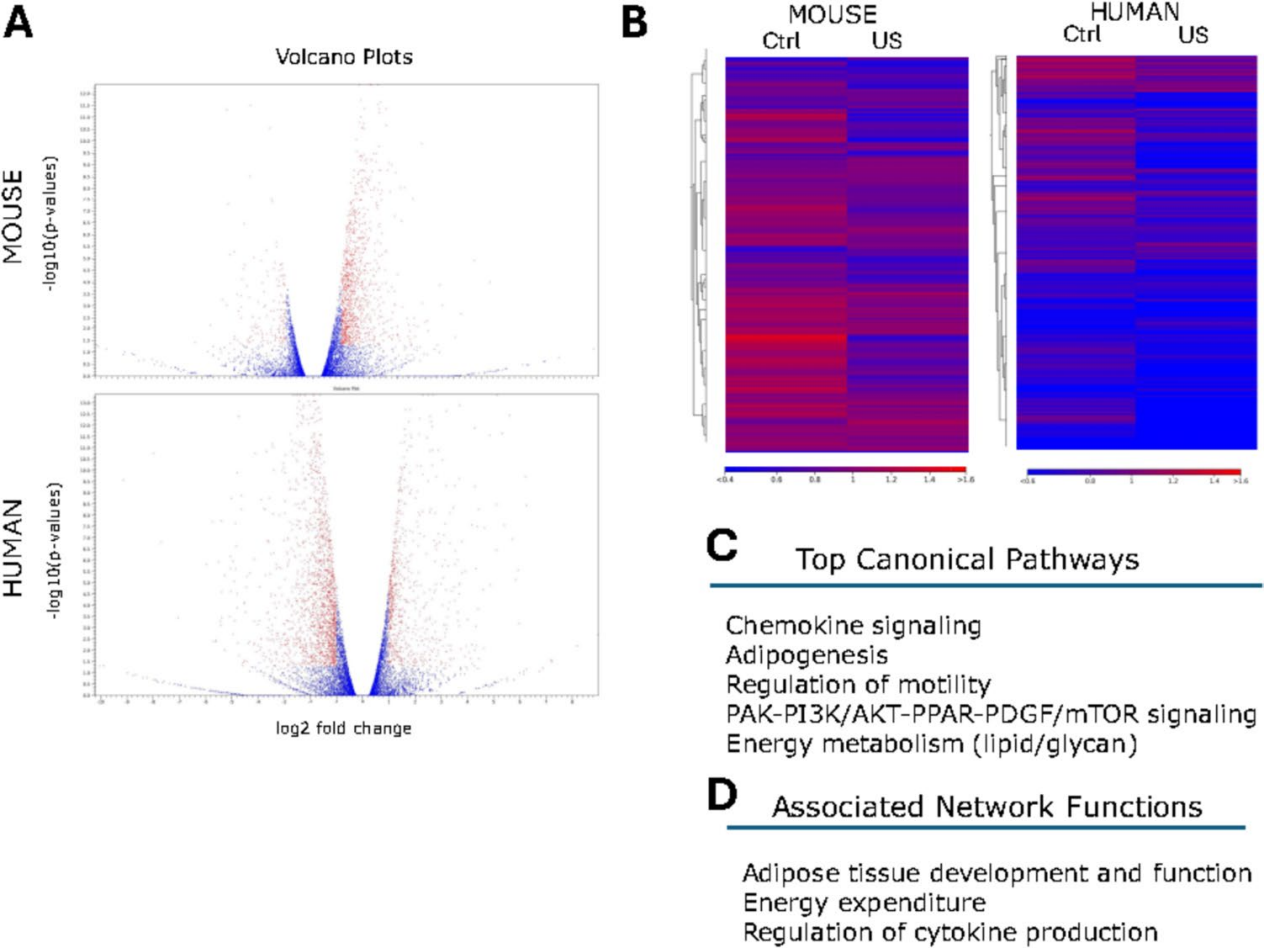
RNA-seq results. (**A**) Volcano plot showing genes differentially expressed between control and ultrasound-stimulated (US) mouse and human ASCs. (**B**) Heat map graphs, where the red zones correlate with upregulated genes and the blue zones with the absence of changes in expression. (**C**) Top canonical pathways and (**D**) associated network functions where major differentially expressed genes, that fulfil the conditions to present a *p*-value under 0.05 and fold change over 3 or under −3, were clustered

Given the above results in proliferation with respect to ultrasound application on mouse and human ASCs, we performed a depth analysis of changes in gene expression by RNA-seq in LIPUS-stimulated mouse and human ASCs compared with unstimulated mouse and human ASCs (Supp. Dataset 1 and 2). Surprisingly, but at the same time consistent with previous results [67, 74–78], only 270 genes were differentially expressed in human ASCs by the LIPUS stimulation protocol used and around 130 genes were differentially expressed in LIPUS-stimulated mouse ASCs, as shown by Volcano plots (Fig. 3A).

Heat map analysis revealed relevant changes in expression patterns after LIPUS stimulation, in both mouse and human samples (Fig. 3B). Of the 270 differentially expressed genes in human ASCs, 120 were upregulated by ultrasounds and 150 were significantly downregulated. In mouse ASCs, LIPUS stimulation produced an upregulation of the expression of 83 genes, while causing a downregulation in the expression of 47 genes. Interestingly, among the genes differentially expressed after LIPUS stimulation, both in human and mouse ASCs, those encoding for differentiation, metabolism and cell motility were highlighted and at least 15 genes encoded for proteins belonging to the chemokine signalling complex (Fig. 3C). Finally, the RNA-seq analysis showed that the ultrasound protocol used in mouse and human ASCs triggered relevant changes in gene expression, highlighting networks such as energy metabolism, adipose tissue development and function and regulation of cytokine production (Fig. 3D). Therefore, we continue with a more in-depth characterization of the LIPUS stimulated ASCs to confirm these results.

### Low-Intensity Ultrasound Stimulation Slightly Increases their Migration Capacity

**Fig. 4:**
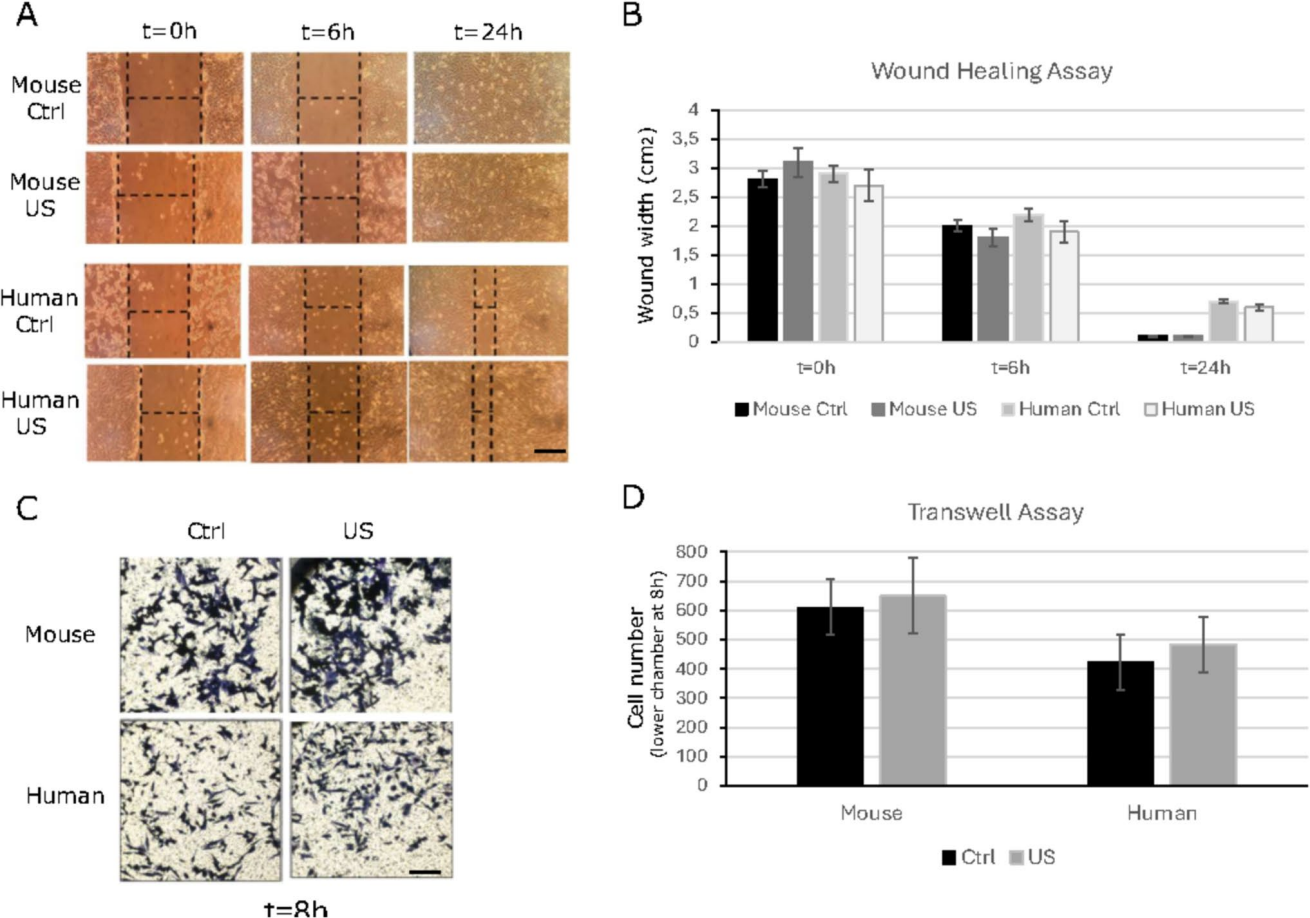
Migration assays. (**A**) Representative images of the wound area at time 0 h, 6 h and 24 h after scratching. One representative experiment out of four are shown of mouse and human ASCs cultures under control conditions (Ctrl) or treated with ultrasound (US). Bar, 100 mm (**B**) Data are shown from a representative experiment out of four performed and denote mean ± SD. Statistical analysis was performed using Student’s t test comparing US vs. Ctrl into mouse or human condition at each time. No major significant differences were observed. (**C**) Representative images of the migrated cells in the Transwell migration assay. One independent experiment out of five for mouse and human ASCs under control conditions (Ctrl) or treated with ultrasound (US) are shown. Bar 30 mm (**D**) Data are shown from a representative experiment out of five performed and denote mean ± SD. Statistical analysis was performed using Student’s t test. Not major significant differences were quantified

Next, to evaluate whether the LIPUS stimulation could effectively affect the ability of mouse and human ASCs to migrate, we performed two different migration-based assays. Firstly, an in vitro scratch-healing assay was performed to analyse the two-dimensional cell migration. No major significant differences in scratch closure were observed in mouse or human ASCs between LIPUS-stimulated or non-stimulated ASCs, although there was a consistent tendency for the scratch to close earlier after LIPUS application (Fig. 4A and B).

To assess the individual migration of mouse and human ASCs and the impact of LIPUS stimulation, a migration assay using Transwell chambers was performed. Similar to the results of the scratch assay, no major changes in migration were observed between mouse or human ASCs comparing LIPUS-stimulated or non-stimulated ASCs, but again the LIPUS stimulation seemed to slightly accelerate the individual migration of ASCs (Fig. 4C and D), although no significant differences could be quantified.

Overall, the results of the migration assays suggest that, although the expression of several genes related to motility of ASCs may be modified after LIPUS stimulation, at the parameters used, LIPUS stimulation could only slightly accelerate the migration capacity of ASCs while maintaining their stemness properties.

### Differentiation to Mature Adipocytes was Diminished by Low-intensity Ultrasound Stimulation

We next analyzed the differentiation capacity of mouse and human ASCs to mature in full functional adipocytes after the action of LIPUS. The expression status of several molecules involved in the adipogenesis process was also confirmed.

**Fig. 5:**
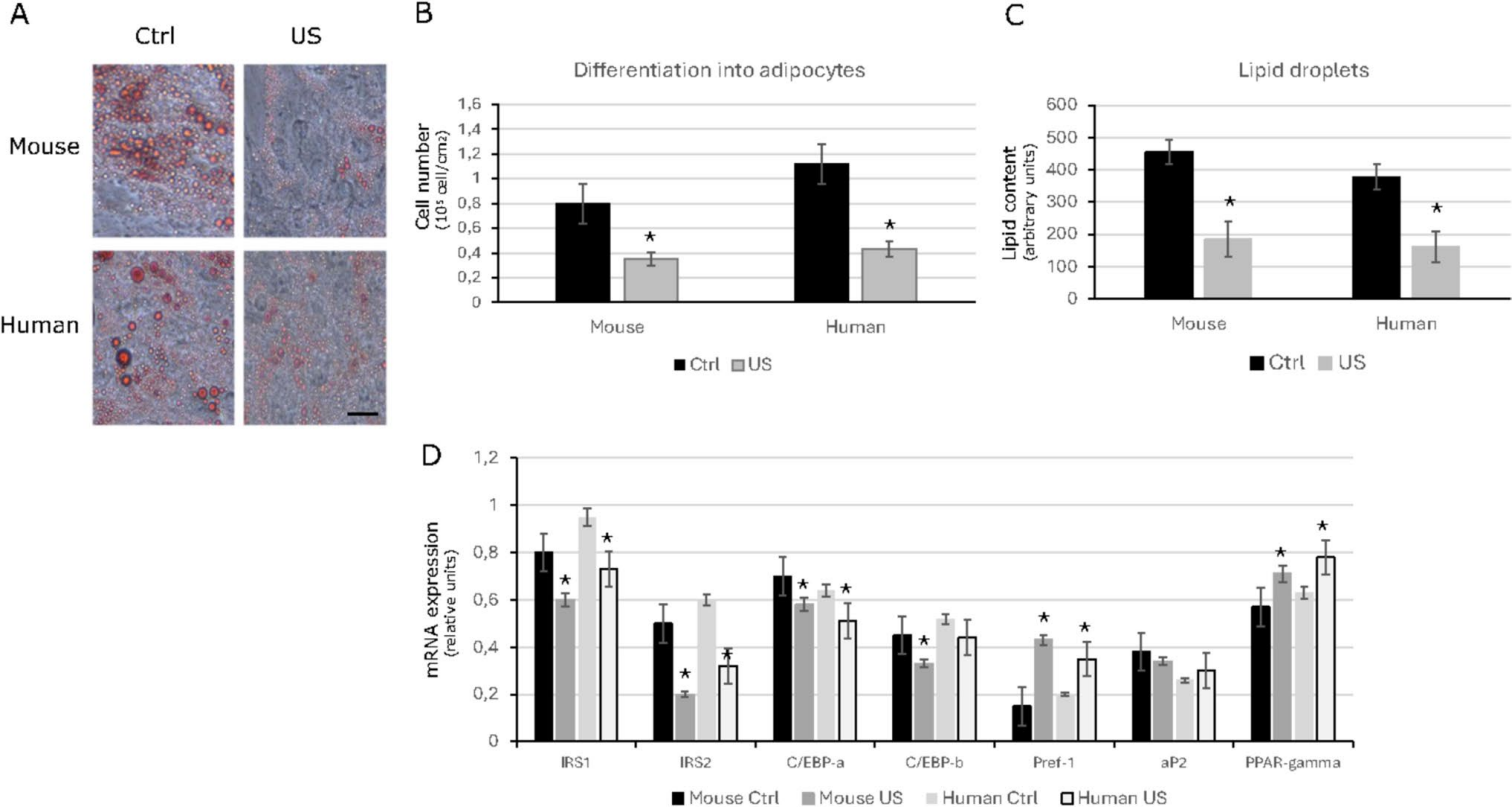
Differentiation of mouse and human ASCs under control conditions or treated with ultrasound (US). (**A**) Representative image of Oil Red O staining in mouse and human ASCs under control conditions or treated with ultrasound (US) differentiated into adipocytes after 7 days. Bar, 20 mm. (**B**) Cell number of mature adipocytes quantified in mouse and human ASCs under control conditions or treated with ultrasound (US) (*n* = 3). *p < 0.05. (**C**) Intracellular lipid accumulation in differentiating mouse and human ASCs under control conditions or treated with ultrasound (US). *p < 0.05. (**D**) Gene expression profile of differentiated mouse and human ASCs under control conditions or treated with ultrasound (US). (*n* = 3). *p < 0.05

Interestingly, although LIPUS-stimulated mouse and human ASCs were able to differentiate into mature adipocytes, as defined by Oil Red O staining of lipid droplets, the number of adipocytes was significantly lower compared with non-stimulated ASCs (Fig. 5A). Analysis of Oil Red O staining after 7 days of differentiation revealed that ASCs rapidly differentiated into well-defined mature adipocytes, while LIPUS-stimulated ASCs did not reach complete maturity, and the derived adipocytes presented smaller lipid droplets (Fig. 5B). Moreover, quantification of lipids of differentiated ASCs revealed a 60%- 75% reduction in the triglyceride composition of the droplets of mouse and human LIPUS-stimulated ASCs respectively compared with un-stimulated ASCs (Fig. 5C).

To corroborate the differences in maturity between the mouse and human LIPUS-stimulated and unstimulated ASCs, we next analysed the expression of several adipogenic and stemness markers that were candidates to be modified by LIPUS stimulation according to RNAseq results. Major decreases were observed in the expression of insulin receptor substrate-1 and 2 (*IRS1* and *IRS)2* and CCAAT/enhancer-binding protein alpha (*C/EBP-a)* in both LIPUS-stimulated mouse and human ASCs. A significant decrease in the expression of CCAAT/enhancer-binding protein beta (*C/EBP-b)* was also observed after LIPUS stimulation in mouse ASCs. All of them are relevant genes for adipogenesis (Fig. 5D). On the other hand, for both LIPUS-stimulated mouse and human ASCs, we detected an increased expression of preadipocyte factor 1 (*Pref-1*) and peroxisome proliferator-activated receptor g (*PPAR-g*) expression, in agreement with the blockade of adipogenesis and the maintenance of stemness properties. No major changes were observed with adipocyte fatty acid binding protein (*aP2*) (Fig. 5D).

Taken together, these results demonstrate that mouse and human LIPUS-stimulated ASCs have an impaired ability to differentiate correctly compared to unstimulated ASCs.

### Adipokine Release Pattern was Modified by Low-intensity Ultrasound Stimulation

**Fig. 6:**
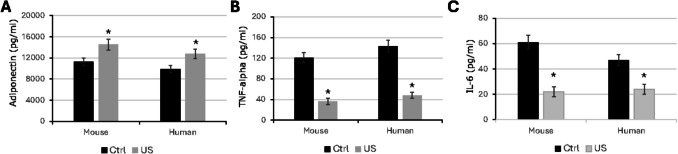
Cytokine expression pattern. (**A**) Mouse and human ASCs under control conditions or treated with ultrasound (US) were differentiated for up to 7 days and cultured overnight in serum-free medium. Adiponectin, IL-6, and TNF-alpha content were measured in conditioned medium (pg/mL) (*n* = 4). *p < 0.05

Adipocytes are not only storage cells but can also release many different factors, such as hormones, classical cytokines and growth factors [79]. In fact, in the RNAseq results major differences in cytokines expression after LIPUS stimulation were detected. An in-depth examination of adipokine secretion patterns during ASCs differentiation revealed that whereas adiponectin content in conditioned medium of mouse and human LIPUS-stimulated ASC was significantly increased in comparison with unstimulated ASC (Fig. 6A), other cytokines such as TNF-alpha or IL-6 were strongly decreased in the conditioned medium after LIPUS stimulation (Fig. 6B and C).

Interestingly, inflammatory adipokines seemed to be downregulated after LIPUS treatment, in both mouse and human ASCs, which could be beneficial in the context of an inflammatory adipose tissue.

### Low-intensity Ultrasound Stimulation of Mouse Adipose Tissue Explants Modified their Physiological Functions

**Fig. 7:**
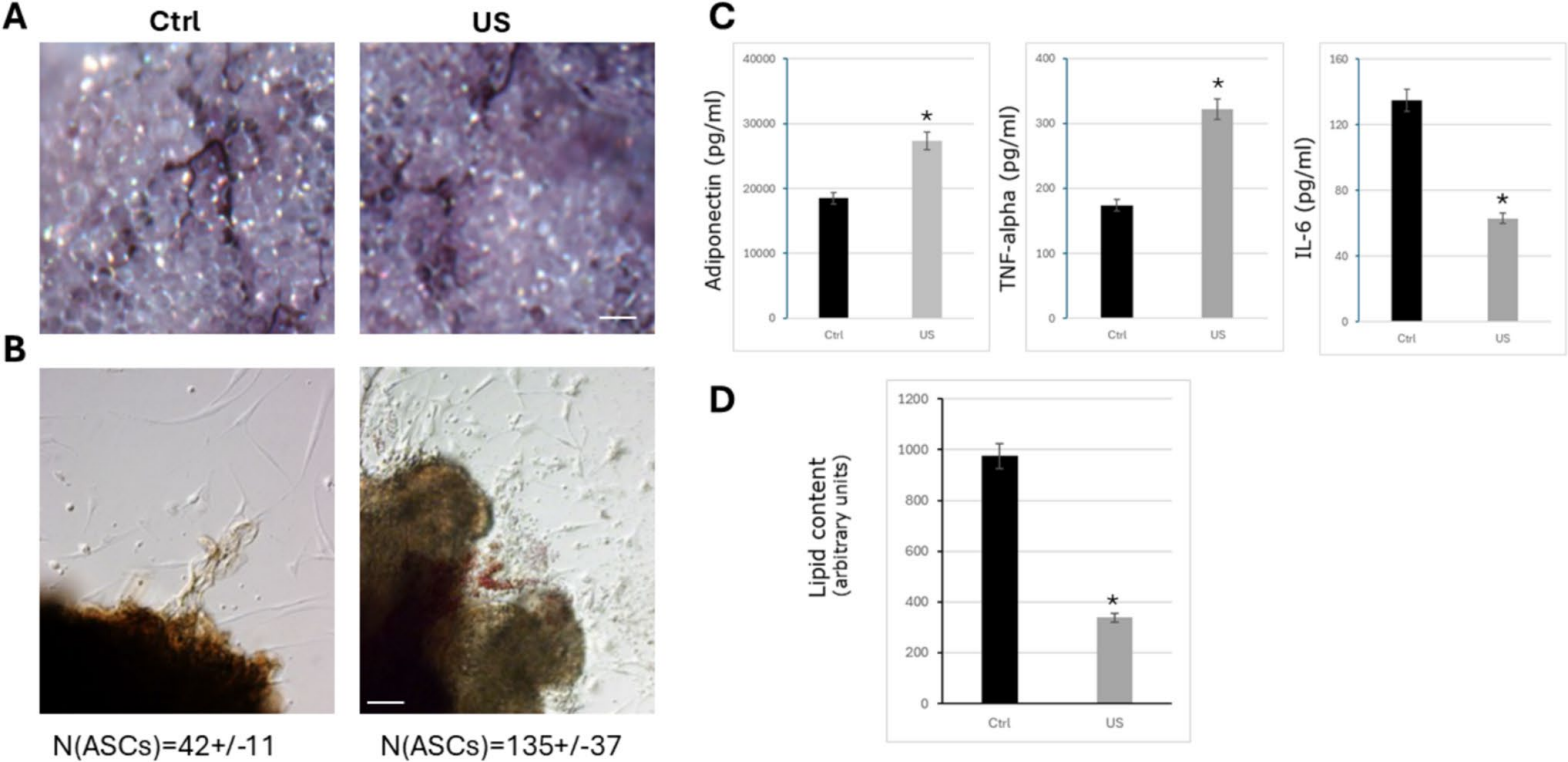
Mouse adipose tissue explants are influenced by LIPUS stimulation. (**A**) Representative image of mouse adipose tissue explants stained with haematoxylin under control conditions or treated with ultrasound (US) after 24 h from plated. Bar, 100 mm. (**B**) Representative image of the border of mouse adipose tissue explants under control conditions or treated with ultrasound (US) after 24 h from plated. Note ASCs coming out from the explants. ASCs number (N) under control conditions or treated with ultrasound (US) (*n* = 3). *p < 0.05. (**C**) Cytokine expression pattern. Mouse adipose tissue explants under control conditions or treated with ultrasound (US) were cultured for up to 24 h and supernatants were collected. Adiponectin, IL-6, and TNF-alpha content were measured in conditioned medium (pg/mL) (*n* = 3). *p < 0.05. (**D**) Intracellular lipid accumulation in mouse adipose tissue explants under control conditions or treated with ultrasound (US). *p < 0.05

The culture of adipose tissue explants is a physiological method to mimic the adipose tissue’s natural environment [74]. This system maintains cellular function of adipose tissue together with a good structure and architecture of the tissue that allow to study the adipose tissue biology. Adipose tissue explants were isolated from mice and cultured onto p24 wells plates. As shown in Fig. 7A, no differences were observed microscopically on the adipose explants after LIPUS stimulation in comparison with unstimulated explants. Nevertheless, 24 h after LIPUS stimulation the quantity of ASCs that sprouted out of the adipose explants were three times higher than in unstimulated plates (Fig. 7B), indicating that ASCs after LIPUS stimulation maintained their stemness and migration properties, instead of differentiated into mature adipocytes and stayed inside the explants.

In fact, LIPUS-stimulated adipose tissue explants seemed not to reach complete maturity as the derived lipid droplets were apparently smaller and the quantification of lipids of adipose tissue explants revealed an important reduction in the triglyceride composition of the droplets of mouse LIPUS-stimulated adipose tissue explants compared with un-stimulated adipose tissue explants (Fig. 7D). Finally, inflammatory properties of the mouse adipose tissue explants were analysed. Adipokine secretion patterns analysed in the media of mouse adipose tissue explants revealed similar results to the ones observed on ASCs previously. In fact, adiponectin concentration was significantly increased after LIPUS stimulation in comparison with unstimulated adipose tissue explants (Fig. 7C, left panel). TNF-alpha and IL-6 were strongly decreased in the conditioned medium of adipose tissue explants after LIPUS stimulation (Fig. 7C, central and right panels).

## Discussion

Obesity is a multifactorial disorder characterized by chronic inflammation throughout the body, where the adipose tissue is the main regulatory organ [5]. In this study, we used two adipose stem cell lines derived from obese mouse and human adipose tissue to investigate the effect of applying low-intensity pulsed ultrasounds on the main biological properties of these cells and their contribution to the niche of the pathological adipose tissue of obese subjects.

While it is confirmed that LIPUS is completely safe for use in mouse and human ASCs, our results show no major differences in proliferation or cell death, which is encouraging to support LIPUS as a therapeutic tool as previously described in other models**,** [64, 73]. On the other hand, the parameters used in the present study were optimal to interfere mainly with the adipogenic and inflammatory process. Ultrasounds used at higher doses (1–150 W/cm^2^), as for example during liposuctions, reduced ASCs proliferation and survival as well as adipocyte’s structure, decreasing the possibilities of fat regrowth [77, 78].

After applying LIPUS treatment, the RNAseq analysis revealed that around 270 genes were differentially expressed in human adipose stem cells (ASCs), 120 were upregulated and 150 were downregulated due to ultrasound treatment. However, in mouse ASCs, LIPUS stimulation resulted in the only upregulation of 83 genes and the downregulation of 47 genes. LIPUS stimulation highlighted the impact of ultrasounds in three major associated networks such as adipose tissue development and function, energy metabolism and cytokine production. Compared to previous studies conducted with LIPUS, these results share common pathways such as those implicated in metabolic activity and anti-inflammatory effects, through different signaling pathways including nuclear factor-κB (NF-κB), mitogen-activated protein kinase (MAPK), and phosphatidylinositol-3-kinase/serine/threonine kinase (PI3 K/Akt) [67, 74–78]. Overall, there is a change in expression trends in both human and mouse samples; although slight differences in expression patterns are evident, showing mouse samples with a higher level of activation in unstimulated mice cells (controls) compared to human unstimulated cells (Fig. 2A). Human ASCs samples are always more diverse, which may explain some of the more differences observed in murine ASCs compared to human samples. However, it is crucial to carefully consider the experimental design and interpretation when comparing fat depots between rodents and humans, as there are significant differences across various depots in both species [9, 10, 45, 80].

Notably, the genes that showed differential expression following LIPUS stimulation in both mouse and human ASCs, were related to differentiation, metabolism, and cell motility. Previous genomics studies on adipose tissue, specifically in WAT, have demonstrated the relevance of these pathways in obesity patients [67]. In fact, in vitro studies showed that the behavior and quality of ASC from obese patients had lower proliferative ability, increased senescence, and increased proinflammatory cytokine expression [81]. Recent studies have explored the effects of high-fat diets on gene expression in adipose tissue, revealing specific gene alterations that contribute to obesity and insulin resistance such as ADIPOQ, CD36, PPARG, and IL6. They also found genetic variations and differential gene expression in adipose tissue for ECM genes such as SIRT1 [82]. Additionally, at least 15 of the LIPUS regulated genes were associated with the chemokine signaling complex. Expression of those chemokines like CCL2 and CCL5 was elevated in numerous obese adipose tissues as well as other chemokines such as CCL19 [37, 83].

In contrast to our RNAseq results where modification of genes implicated in cell motility was clearly detected on ASCs after LIPUS-stimulation, we did not observe significative differences in the two models tested for quantification of migration. At the parameters used, LIPUS stimulation could only slightly accelerate the migration capacity of ASCs while maintaining their stemness properties. Increasing LIPUS parameters as well as increasing the number of replicates could help to visualize in vitro the effect of gene expression modification.

Due to the LIPUS induced significant changes in ASCs gene expression, particularly in networks related to energy metabolism, adipose tissue development and function, as well as cytokine production regulation, we proceeded with a more detailed characterization of the LIPUS-stimulated ASCs to validate these findings. Accordingly, we found remarkable differences in the differentiation capacity of ASCs after LIPUS stimulation, both in mouse and human cells, which supports the possibility of diminishing the differentiation of ASCs into mature adipocyte, thus avoiding their contribution to the increase of adipose mass and the pathological changes occurring during obesity. The observed effects were exclusively due to changes in the differentiation potential of ASCs, and not due to decrease of overall ASCs or senescence effects, as our results showed that neither proliferation, nor cell death, were affected by LIPUS stimulation on ASCs at the parameter used. Under normal conditions, white adipose tissue (WAT) presents adipocytes containing fewer but larger lipid droplets with an important lipid accumulation during obesity [38]. Previous studies described greater differentiation of ASCs in obese individuals, as a compensatory response to the inflammatory environment [18, 30], preventing the onset of lipotoxicity and glucotoxicity [29]. Mouse and human ASCs stimulated with LIPUS presented small lipid droplets with a very reduced lipid content, resembling structures typical of non-adipogenic lineages or even in some cases of brown adipose tissue (BAT) structures [84]. Interestingly, a recent study about the role of ASC in aging, observed that obesity prematurely induces a decrease in adipose tissue precursors proportions and there was an altered negative regulation of fat cell differentiation [85]. In this sense, the use of the LIPUS technique could allow for a ‘healthier ASC phenotype’, and a compensatory mechanism to contrast the obesogenic environment. Further analysis could be desirable to study and clarify the complexity of the obesogenic context and the crosstalk with the different cell types.

According to our current knowledge, the main contributors to obesity-induced systemic inflammation are the cytokines and other hormone-like molecules released by the WAT, [58, 86]. Indeed, the gene expression level of leptin was increased in parallel with body weight in different animal models [54]. Therefore, we examined the cytokine secretion profile of mouse and human ASCs after LIPUS application. More interestingly, the levels of inflammatory cytokines were modified by LIPUS application, reducing the inflammatory phenotype of mouse and human ASCs. In our study, adiponectin was clearly increased in beneficial of the surrounding tissue and favouring an anti-inflammatory state [42, 58]. Adiponectin, released by either adipocyte or ASCs [19], normally is decreased in obesity [43] In contrast, in our study the proinflammatory cytokines such as TNF-alpha or IL-6 were strongly reduced in the presence of LIPUS, reducing their secretion by ASCs as they maintained a stemness phenotype, with a non-inflammatory profile [19, 86, 87]. Chronic unresolved systemic and adipose tissue inflammation drives obesity-related cardiometabolic disease. Drugs targeting pro-inflammatory cytokines, or inflammasome. activation, are approved for clinical use but can elicit serious adverse effects (such as weight gain and increased susceptibility to infections), hampering their clinical implementation. Our results, both inflammation and cytokines gene expression modification as well as adipokines in vitro measurements, strongly suggest that the use of LIPUS has great potential for the control of the adipose tissue inflammation. Future in vivo studies will confirm the potential of LIPUS as an anti-inflammatory tool that may reduce chronic inflammation in obese patients.

Finally, and more interestingly, all the changes observed in ASCs in this study in presence of LIPUS were not reversible and could be maintained over several passages, helping to preserve a safety and favourable environment that would be ideal for the treatment of adipose tissue in obese subjects [54, 57, 58].

Although our results provide new insights about the mechanisms involved in the use of LIPUS on ASCs, the conclusions laid only in in vitro experiments with controlled environment and with a low number of replicates. For this reason, a standardized ex vivo model of mouse adipose tissue was also used to confirm the results observed in the ASCs in vitro model. As previously described, adipose explants reproduce the physiological conditions of the tissue, and the principal metabolic and cellular functions can be measured [74]. In agreement with previous authors, adipose tissue explants kept their morphology and survival for more than 24 h and interestingly, LIPUS stimulated produced a reduction of the inflammatory environment by decreasing the quantity of adipokines and at the same time, reduced the quantity of lipid accumulation in the adipose tissue. We hypothesize that it is due to the lower differentiation of ASCs, that increase in number during isolation from adipose tissue after LIPUSs stimulation and therefore the number of mature adipocytes is reduced. Future i*n vivo* experiments with animal models where ASCs will be in the complex adipose tissue context, may confirm these results. Thus, although LIPUS is a promising field and may impact on the function of different cell types, still much more research is needed to advance in its translation into the clinical practice.

## Conclusions

The final aim of the study was to evaluate the possibilities of using LIPUS stimulation to influence the physiology of adipose stem cells and thus their contribution to the development of adipose tissue and obesity. Ultrasound stimulation of mouse and human ASCs with this LIPUS device had no detrimental effects on cell viability; however, the functional properties of the cells studied were modified in response to the LIPUS signal due to the inhibition and induction of several genes and proteins that directly affect the adipogenesis and inflammatory capacity of the ASCs. In conclusion, the use of LIPUS equipment with the fixed parameters may reduce the differentiation into mature adipocytes of adipose stem cells as well as their inflammatory phenotype, diminishing their contribution to the adipose mass developed in obese subjects and maintaining their stemness properties. LIPUS impact similarly on mouse adipose tissue explants limiting the differentiation capacity of the tissue and reducing the inflammatory environment. We present sufficient evidence to highlight the importance of standardizing ultrasound application parameters and methods to be used as a potential therapeutic tool. Future in vivo experiments will provide new insights into this promising field.

## Supplementary Information

Below is the link to the electronic supplementary material.

Supplementary file1 (PDF 1012 KB)

Supplementary file2 (PDF 11446 KB)

## Data Availability

All datasets are included in the article as supplementary figures.
