# Supplementary material for: Low Intensity Pulsed Ultrasounds Modulate Adipose Stem Cells Differentiation
Source: Stem Cell Rev Rep. 2025 May 23;21(6):1760–75. doi: 10.1007/s12015-025-10896-7 (PMC12356745; doi:10.1007/s12015-025-10896-7)
Supplement: Supplementary file 1 — Supplementary file1 (PDF 1012 KB) [file 12015_2025_10896_MOESM1_ESM.pdf]

Name	Chromosome	Region	Max group mean	Log_2 fold change	Fold change	P-value	FDR p-value	ENSEMBL
Gm7357	1	4522905..4	0,596892	-2,21429	-4,64052	0,000498	0,008591	ENSMUSG(
Trpa1	1	compleme	6,185995	3,697684	12,97519	0	0	ENSMUSG(
Sbspon	1	compleme	0,517674	5,846	57,52033	1,38E-07	7,14E-06	ENSMUSG(
Gdap1	1	17145362.	2,546542	-1,02687	-2,03759	0,000563	0,009534	ENSMUSG(
Crispld1	1	17727045.	2,864157	-2,41538	-5,33459	7,47E-07	3,1E-05	ENSMUSG(
Mcm3	1	compleme	441,5566	1,546306	2,920684	6,76E-10	6,24E-08	ENSMUSG(
Efhc1	1	20951626.	17,3586	1,441043	2,71517	1,48E-08	9,93E-07	ENSMUSG(
Tmem14a	1	21218575.	128,6907	2,628911	6,185588	0	0	ENSMUSG(
Rims1	1	compleme	0,638029	3,136195	8,792023	1,39E-08	9,42E-07	ENSMUSG(
Bend6	1	compleme	0,792261	1,505035	2,838316	0,002226	0,030386	ENSMUSG(
Cnnm4	1	36471620.	1,278226	-1,15746	-2,23064	0,001268	0,018828	ENSMUSG(
Txndc9	1	compleme	1,475242	1,089509	2,128017	0,000249	0,004794	ENSMUSG(
Eif5b	1	37998010.	6,356673	-1,48551	-2,80017	3,06E-06	0,000106	ENSMUSG(
Pdcl3	1	38985608.	6,885999	-1,36304	-2,57227	1,57E-07	7,97E-06	ENSMUSG(
Npas2	1	39193731.	2,128299	-2,50428	-5,67366	2,59E-10	2,66E-08	ENSMUSG(
Cnot11	1	39534992.	16,54012	-1,12493	-2,1809	1,33E-07	6,91E-06	ENSMUSG(
Map4k4	1	39900913.	68,59066	-2,05918	-4,16748	0	0	ENSMUSG(
Tex30	1	compleme	35,19311	1,806018	3,496757	5,55E-16	1,95E-13	ENSMUSG(
Kdelc1	1	compleme	11,7412	1,599299	3,02996	2,28E-11	2,93E-09	ENSMUSG(
Bivm	1	44118957.	15,7674	1,589996	3,010485	3,9E-13	7,39E-11	ENSMUSG(
Ercc5	1	44147744.	10,6729	1,240686	2,363109	3,04E-05	0,000775	ENSMUSG(
Gulp1	1	44551511.	6,340439	1,048467	2,068331	4,16E-05	0,001019	ENSMUSG(
Col3a1	1	45311538.	245,5452	5,797962	55,6366	0	0	ENSMUSG(
Sdpr	1	51289126.	10,78628	1,08182	2,116705	6,19E-06	0,000197	ENSMUSG(
Gm28177	1	52077261.	1,185861	-2,13058	-4,37894	0,001031	0,015869	ENSMUSG(
Mfsd6	1	compleme	0,600143	-1,65107	-3,14067	0,00169	0,024083	ENSMUSG(
Osgepl1	1	53313624.	2,684575	1,114723	2,165534	0,000345	0,006276	ENSMUSG(
Dnah7a	1	compleme	0,066272	1,999749	3,999304	0,002484	0,033315	ENSMUSG(
Hecw2	1	compleme	1,306704	-1,43178	-2,69779	6,39E-05	0,001487	ENSMUSG(
Hspe1	1	55088132.	87,2937	-1,00357	-2,00495	4,2E-07	1,87E-05	ENSMUSG(
4930558J1	1	compleme	0,137339	2,754516	6,748264	0,002031	0,028055	ENSMUSG(
Fam117b	1	59913006.	0,488615	-1,47283	-2,77566	0,003034	0,039492	ENSMUSG(
Nrp2	1	62703285.	6,58602	-2,67635	-6,39237	0	0	ENSMUSG(
Gpr1	1	compleme	2,42524	-1,80243	-3,48806	0,000391	0,006961	ENSMUSG(
Adam23	1	63445891.	1,160398	-3,44512	-10,8914	2,54E-14	6,53E-12	ENSMUSG(
Fzd5	1	compleme	0,792061	-1,06328	-2,08967	0,00368	0,04663	ENSMUSG(
Atic	1	71557150.	7,003069	-1,44582	-2,72417	1,82E-05	0,000501	ENSMUSG(
March4	1	compleme	0,248207	-3,48132	-11,1681	0,000987	0,015284	ENSMUSG(
Smarcal1	1	72583251.	2,337714	-1,83478	-3,56718	9,23E-09	6,5E-07	ENSMUSG(
Tns1	1	compleme	1,7595	-1,49753	-2,82358	5,02E-07	2,19E-05	ENSMUSG(
Zfp142	1	compleme	1,427284	-2,23237	-4,69907	1,8E-11	2,37E-09	ENSMUSG(
Stk36	1	74601445.	2,974522	-1,18121	-2,26766	3,57E-06	0,000121	ENSMUSG(
Ttll4	1	74661745.	1,469211	-2,47157	-5,54649	3,55E-15	1,06E-12	ENSMUSG(
Abcb6	1	compleme	16,37063	1,136608	2,198635	3,18E-07	1,46E-05	ENSMUSG(
Atg9a	1	compleme	9,578705	-1,7479	-3,35868	2E-15	6,24E-13	ENSMUSG(

Tuba4a	1	compleme	14,15611	-1,04475	-2,063	1,79E-06	6,66E-05	ENSMUSG(
Speg	1	75375297.	1,128389	-1,91963	-3,78326	4,8E-10	4,57E-08	ENSMUSG(
Epha4	1	compleme	2,782372	-1,02591	-2,03624	3,12E-05	0,000793	ENSMUSG(
Kcne4	1	78816758.	0,727238	2,571833	5,945645	1,11E-05	0,000327	ENSMUSG(
Irs1	1	compleme	5,605857	-1,20802	-2,3102	1,39E-07	7,16E-06	ENSMUSG(
Ncl	1	compleme	69,41792	-2,3032	-4,93551	2,3E-11	2,96E-09	ENSMUSG(
Cops7b	1	86582904.	4,086783	-1,44456	-2,72181	4,86E-08	2,85E-06	ENSMUSG(
Ngef	1	compleme	12,24566	1,836737	3,572012	8,77E-15	2,49E-12	ENSMUSG(
Dgkd	1	87853287.	6,967181	-1,47963	-2,78877	1,93E-07	9,6E-06	ENSMUSG(
Trpm8	1	88277661.	0,664615	3,278779	9,705341	2,77E-08	1,72E-06	ENSMUSG(
Sh3bp4	1	89070415.	12,88871	-1,88185	-3,68546	1,33E-15	4,38E-13	ENSMUSG(
Agap1	1	89454806.	2,818876	-1,33693	-2,52613	1,41E-08	9,54E-07	ENSMUSG(
Gbx2	1	compleme	0,524089	-1,91644	-3,77491	0,003886	0,048741	ENSMUSG(
Ramp1	1	91179822.	4,346899	1,376712	2,596759	1,11E-05	0,000326	ENSMUSG(
Traf3ip1	1	91494647.	2,42002	-1,04343	-2,06113	0,002204	0,030136	ENSMUSG(
Hdac4	1	compleme	1,615189	-1,26896	-2,40987	4,03E-05	0,000992	ENSMUSG(
St8sia4	1	compleme	0,269873	-1,85526	-3,61818	0,000877	0,013813	ENSMUSG(
Gin1	1	97770172.	6,170424	1,170335	2,25064	6,56E-06	0,000206	ENSMUSG(
Pam	1	compleme	44,69697	1,031088	2,043564	1,22E-06	4,77E-05	ENSMUSG(
Gm5260	1	104513001	4,4082	-1,59408	-3,01901	0,000429	0,007538	ENSMUSG(
Tnfrsf11a	1	105780718	0,386196	-1,85368	-3,61421	0,001593	0,022943	ENSMUSG(
Zcchc2	1	105990406	1,50866	-1,77733	-3,42791	7,02E-06	0,000219	ENSMUSG(
Phlpp1	1	106171752	2,665305	-2,18997	-4,56294	1,28E-12	2,17E-10	ENSMUSG(
Bcl2	1	compleme	0,692407	-1,71979	-3,29389	7,86E-06	0,000242	ENSMUSG(
Clasp1	1	118389058	5,49201	-1,12775	-2,18518	8,2E-08	4,5E-06	ENSMUSG(
Tfcp2l1	1	118627945	0,123107	-2,77307	-6,8356	0,00083	0,013187	ENSMUSG(
Gli2	1	compleme	0,811966	-2,99775	-7,98754	1,48E-09	1,26E-07	ENSMUSG(
En1	1	120602418	2,306243	-1,2646	-2,4026	0,00055	0,009353	ENSMUSG(
Gpr39	1	125676995	1,735804	-1,60436	-3,04062	3,63E-05	0,000907	ENSMUSG(
Lypd1	1	compleme	0,337618	-3,01743	-8,09724	8,45E-05	0,001895	ENSMUSG(
Cxcr4	1	compleme	7,273437	4,855536	28,95089	9,99E-15	2,78E-12	ENSMUSG(
Il24	1	compleme	8,435556	-4,87212	-29,2856	1,73E-05	0,000479	ENSMUSG(
Mapkapk2	1	compleme	12,88235	-1,13215	-2,19185	1,67E-06	6,25E-05	ENSMUSG(
Srgap2	1	compleme	8,132847	-1,03741	-2,05254	2,42E-07	1,16E-05	ENSMUSG(
Slc41a1	1	131827493	3,492258	-1,32034	-2,49725	3,03E-07	1,4E-05	ENSMUSG(
Rab29	1	131867224	8,429217	-1,38612	-2,61375	0,000195	0,003866	ENSMUSG(
Elk4	1	132007607	1,770168	-2,55261	-5,86693	1,11E-09	9,78E-08	ENSMUSG(
Nfasc	1	compleme	0,363361	-1,93486	-3,82341	1,99E-05	0,000539	ENSMUSG(
Mdm4	1	compleme	0,875764	-1,10704	-2,15403	0,000792	0,012698	ENSMUSG(
Pik3c2b	1	133045667	0,598601	-1,66153	-3,16352	0,000277	0,005235	ENSMUSG(
Sox13	1	compleme	2,251939	-1,59679	-3,02469	5,14E-06	0,000167	ENSMUSG(
Chil1	1	134182176	2,096396	-5,43371	-43,2225	1,83E-05	0,000502	ENSMUSG(
Adora1	1	compleme	0,132379	-2,61507	-6,12654	0,003189	0,041245	ENSMUSG(
Tmem183a	1	compleme	48,94401	1,023425	2,032738	1,58E-06	5,98E-05	ENSMUSG(
Elf3	1	compleme	3,131491	-2,52912	-5,77219	0,003054	0,039744	ENSMUSG(
Ipo9	1	compleme	16,15404	-1,00691	-2,00961	5,45E-07	2,36E-05	ENSMUSG(
Nav1	1	compleme	1,302781	-1,16159	-2,23704	0,00217	0,029762	ENSMUSG(
Igfn1	1	compleme	0,394756	-2,49909	-5,65329	0,000246	0,004747	ENSMUSG(

Kif21b	1	136131389	1,151048	-3,71822	-13,1612	4,34E-11	5,3E-09	ENSMUSG(
Atp6v1g3	1	138273738	0,758648	5,927978	60,88344	4,58E-08	2,72E-06	ENSMUSG(
Nek7	1	compleme	13,19593	1,164512	2,241574	7,94E-09	5,69E-07	ENSMUSG(
Gm16332	1	compleme	0,239732	2,745768	6,707469	0,000166	0,003392	ENSMUSG(
Cfh	1	compleme	1,317698	2,504365	5,673997	1,88E-12	3,04E-10	ENSMUSG(
Cdc73	1	compleme	3,314811	-1,09212	-2,13187	9,58E-06	0,000287	ENSMUSG(
Pla2g4a	1	compleme	61,41597	1,213825	2,319519	0,000153	0,00318	ENSMUSG(
Hmcn1	1	compleme	0,117789	-3,29208	-9,79526	4,01E-06	0,000135	ENSMUSG(
1700025G(	1	compleme	7,47859	1,220927	2,330964	3,88E-08	2,33E-06	ENSMUSG(
Nmnat2	1	152954993	1,254119	-1,90766	-3,75199	4,71E-06	0,000155	ENSMUSG(
Pappa2	1	compleme	2,038209	-5,72351	-52,8383	1,79E-10	1,92E-08	ENSMUSG(
Rc3h1	1	160906418	3,772811	-1,00318	-2,00442	3,89E-05	0,000963	ENSMUSG(
Prrc2c	1	compleme	8,000927	-1,39336	-2,6269	1,02E-07	5,41E-06	ENSMUSG(
Pou2f1	1	compleme	0,751308	-1,21298	-2,31816	5,76E-05	0,001354	ENSMUSG(
Rgs4	1	compleme	1,350797	-2,64597	-6,25918	0,000375	0,006714	ENSMUSG(
Sdhc	1	compleme	3,847637	-1,51251	-2,85307	3,54E-06	0,000121	ENSMUSG(
Adamts4	1	171250421	2,408711	-1,04861	-2,06853	0,002407	0,032522	ENSMUSG(
Usp21	1	compleme	2,230816	-1,26812	-2,40847	0,000118	0,002544	ENSMUSG(
Ufc1	1	compleme	16,21954	-1,05752	-2,08134	7,45E-06	0,000231	ENSMUSG(
Dedd	1	171329145	3,746306	-1,61277	-3,05838	1,08E-09	9,53E-08	ENSMUSG(
Pfdn2	1	171345670	27,9087	-2,27164	-4,82872	1,55E-15	4,95E-13	ENSMUSG(
Ncstn	1	compleme	8,81136	-1,00674	-2,00937	2,71E-05	0,000704	ENSMUSG(
Pea15a	1	compleme	25,26675	-1,63979	-3,11621	2,67E-12	4,14E-10	ENSMUSG(
Cfap45	1	172520801	0,562522	-1,94608	-3,85325	0,002267	0,030892	ENSMUSG(
Chml	1	compleme	2,794565	-1,24257	-2,36619	1,78E-06	6,65E-05	ENSMUSG(
Cep170	1	compleme	25,50176	-1,26888	-2,40975	5,38E-10	5,05E-08	ENSMUSG(
Akt3	1	compleme	15,4697	-1,36259	-2,57147	4,87E-08	2,85E-06	ENSMUSG(
Ahctf1	1	compleme	14,08551	-1,3307	-2,51524	1,39E-11	1,87E-09	ENSMUSG(
Itpkb	1	180330485	1,251388	-2,39075	-5,24429	8,06E-09	5,76E-07	ENSMUSG(
Parp1	1	180568924	85,9903	-1,31273	-2,48411	5,85E-10	5,45E-08	ENSMUSG(
Tmem63a	1	180942344	3,37148	-1,14161	-2,20627	6,63E-05	0,001534	ENSMUSG(
Ephx1	1	compleme	7,918233	-1,2932	-2,45071	6,15E-05	0,001438	ENSMUSG(
Trp53bp2	1	182409172	13,98818	-1,87665	-3,6722	0	0	ENSMUSG(
Capn2	1	compleme	52,57374	-1,30745	-2,47504	8,03E-10	7,33E-08	ENSMUSG(
Capn8	1	182565007	0,499319	-2,36424	-5,14883	0,000259	0,00495	ENSMUSG(
Disp1	1	compleme	1,529413	-1,39572	-2,63121	0,000516	0,008846	ENSMUSG(
Aida	1	183296590	6,14088	-1,17288	-2,25461	2,74E-06	9,65E-05	ENSMUSG(
Mia3	1	compleme	13,49858	-1,14841	-2,21669	9,55E-08	5,12E-06	ENSMUSG(
Dusp10	1	184013302	9,332654	-1,45462	-2,74085	1,04E-07	5,48E-06	ENSMUSG(
Nsl1	1	191063012	4,134014	1,233909	2,352034	2,39E-06	8,55E-05	ENSMUSG(
Nenf	1	compleme	55,81121	1,015831	2,022067	1,9E-05	0,000518	ENSMUSG(
Lamb3	1	193207699	10,3945	-2,93723	-7,65942	3,12E-06	0,000108	ENSMUSG(
Plxna2	1	194618218	1,953694	-1,60637	-3,04485	4,72E-08	2,78E-06	ENSMUSG(
Cr1l	1	compleme	0,047469	2,583663	5,994597	0,00394	0,049269	ENSMUSG(
Fam171a1	2	3114224..3	4,478458	-1,01502	-2,02093	0,002013	0,027874	ENSMUSG(
Frmd4a	2	4017717..4	0,660206	-1,63293	-3,10142	1,04E-07	5,5E-06	ENSMUSG(
Celf2	2	compleme	0,166325	-1,77738	-3,42804	7,38E-05	0,001686	ENSMUSG(
Taf3	2	compleme	0,53679	-2,20366	-4,60648	1,73E-05	0,00048	ENSMUSG(

Pfkfb3	2	compleme	3,794668	-1,11164	-2,16091	1,87E-05	0,000511	ENSMUSG(
Rbm17	2	compleme	17,47328	-1,27954	-2,42761	5,49E-07	2,37E-05	ENSMUSG(
Pip4k2a	2	compleme	6,066118	-1,13118	-2,19038	2,19E-05	0,000583	ENSMUSG(
Msrb2	2	19371440.	7,680148	1,071013	2,100909	0,00355	0,04523	ENSMUSG(
Etl4	2	19909780.	0,391427	-2,02936	-4,08223	1,52E-05	0,00043	ENSMUSG(
Psd4	2	24367580.	0,14472	1,716802	3,28707	0,002435	0,032797	ENSMUSG(
Nsmf	2	25054355.	5,669702	-1,53414	-2,89617	3,72E-10	3,64E-08	ENSMUSG(
Anapc2	2	25272478.	2,073527	-1,61202	-3,05679	0,000379	0,006772	ENSMUSG(
Abca2	2	25428703.	2,965362	-1,25595	-2,38824	2,07E-05	0,000557	ENSMUSG(
Tmem141	2	compleme	37,25071	1,407852	2,653419	6,08E-09	4,47E-07	ENSMUSG(
Snapc4	2	compleme	0,955366	-1,54635	-2,92077	4,06E-05	0,000998	ENSMUSG(
Sec16a	2	compleme	1,162426	-1,86472	-3,64197	2,59E-06	9,19E-05	ENSMUSG(
Notch1	2	compleme	1,642824	-1,77482	-3,42196	1,29E-08	8,85E-07	ENSMUSG(
Surf1	2	compleme	2,686905	-1,4751	-2,78004	0,001133	0,01717	ENSMUSG(
Adamtsl2	2	27079379.	0,09164	2,077229	4,219959	0,002471	0,033177	ENSMUSG(
Vav2	2	compleme	3,441598	-1,0175	-2,02441	9,18E-05	0,002038	ENSMUSG(
Brd3	2	compleme	1,530167	-1,34836	-2,54623	7,2E-05	0,00165	ENSMUSG(
Wdr5	2	27515157.	6,491119	-1,0934	-2,13376	1,07E-05	0,000317	ENSMUSG(
Ppp1r26	2	28446800.	1,31801	-1,20245	-2,3013	0,001091	0,016686	ENSMUSG(
Mrps2	2	28468066.	8,736338	-1,84032	-3,58089	1,19E-13	2,51E-11	ENSMUSG(
Gtf3c4	2	compleme	5,4369	-1,46834	-2,76703	8,08E-10	7,37E-08	ENSMUSG(
Ntng2	2	compleme	3,315906	-1,81961	-3,52986	1,73E-05	0,000479	ENSMUSG(
6530402F1	2	compleme	1,300792	-2,16281	-4,47786	4,44E-06	0,000147	ENSMUSG(
Rapgef1	2	29619720.	4,058693	-1,67304	-3,18886	6,61E-10	6,13E-08	ENSMUSG(
Slc27a4	2	29802634.	5,153193	-1,09042	-2,12936	2,94E-05	0,000754	ENSMUSG(
Sptan1	2	29965560.	18,3312	-1,63405	-3,10383	1,44E-12	2,39E-10	ENSMUSG(
Pkn3	2	30077684.	1,206126	-1,49229	-2,81336	0,000266	0,005068	ENSMUSG(
Tbc1d13	2	30133746.	1,855267	-1,16486	-2,24212	0,000543	0,00925	ENSMUSG(
Lrrc8a	2	30237715.	13,7402	-1,1858	-2,2749	2,32E-07	1,12E-05	ENSMUSG(
Nup188	2	30286397.	14,3805	-1,63868	-3,11381	8,33E-14	1,82E-11	ENSMUSG(
Ptges	2	compleme	3,292583	-4,12624	-17,4631	1,3E-08	8,91E-07	ENSMUSG(
Usp20	2	30982279.	1,121412	-1,07552	-2,10748	0,001711	0,024324	ENSMUSG(
Ass1	2	31470207.	485,1025	1,677743	3,19927	2,77E-13	5,51E-11	ENSMUSG(
Abl1	2	31688376.	3,849704	-1,63327	-3,10215	1,22E-11	1,69E-09	ENSMUSG(
Aif1l	2	31950139.	3,931647	2,617171	6,135459	2,22E-15	6,85E-13	ENSMUSG(
Prrc2b	2	32151082.	12,47993	-2,05867	-4,16601	2,78E-15	8,36E-13	ENSMUSG(
Dnm1	2	compleme	1,131401	-1,14596	-2,21293	0,001525	0,02206	ENSMUSG(
Ciz1	2	32352327.	4,663233	-2,07966	-4,22708	2,39E-13	4,81E-11	ENSMUSG(
Fam102a	2	32535332.	0,632626	-1,71723	-3,28805	0,000453	0,007911	ENSMUSG(
Eng	2	32646595.	1,276546	-1,98505	-3,95875	6,46E-06	0,000204	ENSMUSG(
Ttc16	2	compleme	0,184075	-2,00613	-4,01703	0,002919	0,038178	ENSMUSG(
Ptrh1	2	32775786.	5,35568	1,351723	2,552168	0,0007	0,01145	ENSMUSG(
Stxbp1	2	compleme	2,339634	-1,04703	-2,06628	0,001462	0,021262	ENSMUSG(
Angptl2	2	33216069.	3,30206	-1,55063	-2,92945	1,9E-06	7,03E-05	ENSMUSG(
Lmx1b	2	compleme	0,373928	-2,55183	-5,86377	0,000156	0,00322	ENSMUSG(
Hspa5	2	34771970.	80,93777	-1,92927	-3,80862	4,38E-13	8,21E-11	ENSMUSG(
Fbxw2	2	compleme	7,113355	-1,29161	-2,44801	1,3E-08	8,94E-07	ENSMUSG(
Hc	2	compleme	0,752	1,369463	2,583744	0,003288	0,042332	ENSMUSG(

Dab2ip	2	35558266.	1,297545	-1,41379	-2,66436	0,000122	0,00261	ENSMUSG(
Gm13437	2	compleme	63,78287	1,01023	2,014232	7,16E-07	2,98E-05	ENSMUSG(
Gm13461	2	compleme	400,6663	1,17901	2,264214	9,32E-09	6,54E-07	ENSMUSG(
Zeb2	2	compleme	2,539325	-2,2551	-4,77366	0	0	ENSMUSG(
Mbd5	2	48949508.	0,512678	-1,28043	-2,42911	0,000242	0,00469	ENSMUSG(
Kif5c	2	49619298.	1,733903	-1,17955	-2,26507	0,000375	0,006714	ENSMUSG(
Lypd6	2	50066429.	0,997056	-4,84183	-28,6771	4,11E-08	2,46E-06	ENSMUSG(
Galnt5	2	57997884.	7,726885	2,155359	4,454794	8,77E-15	2,49E-12	ENSMUSG(
Pkp4	2	59160850.	13,53508	-1,08474	-2,12099	1,86E-07	9,31E-06	ENSMUSG(
Tanc1	2	59612042.	4,215779	-1,54257	-2,91314	8,76E-11	9,94E-09	ENSMUSG(
Gm13581	2	61224792.	1162,186	1,440026	2,713257	1,95E-12	3,14E-10	ENSMUSG(
Dpp4	2	compleme	1,457731	2,847921	7,199621	2,32E-09	1,85E-07	ENSMUSG(
Fign	2	compleme	0,561347	-2,17588	-4,51862	1,64E-06	6,17E-05	ENSMUSG(
Scn3a	2	compleme	4,837672	4,055997	16,63324	0	0	ENSMUSG(
Scn2a	2	65620771.	0,53258	2,343118	5,07398	3,46E-06	0,000118	ENSMUSG(
Galnt3	2	compleme	5,55685	2,687148	6,440389	1,11E-16	4,39E-14	ENSMUSG(
Scn1a	2	compleme	0,565123	3,154059	8,901565	2,26E-10	2,34E-08	ENSMUSG(
B3galt1	2	67565871.	0,333331	-2,17689	-4,52178	0,001306	0,019298	ENSMUSG(
Gorasp2	2	70661576.	22,78692	-1,41163	-2,66038	5,56E-12	8,02E-10	ENSMUSG(
Cybrd1	2	71117923.	5,528326	1,027429	2,038388	4,84E-05	0,001163	ENSMUSG(
Dlx1	2	71528113.	1,266096	-1,90175	-3,73667	2,28E-05	0,000605	ENSMUSG(
Itga6	2	71745616.	83,79507	1,521421	2,870737	7,57E-11	8,69E-09	ENSMUSG(
Cdca7	2	72476159.	3,662165	-1,01563	-2,02179	0,00132	0,019468	ENSMUSG(
Wipf1	2	compleme	0,999796	-1,45429	-2,74022	0,000337	0,006151	ENSMUSG(
Chn1	2	compleme	2,516341	-1,34098	-2,53323	1,77E-05	0,000489	ENSMUSG(
Hoxd11	2	74679557.	0,456162	2,022828	4,063796	0,002863	0,037567	ENSMUSG(
Gm28309	2	74683446.	1,832083	2,137029	4,398554	0,000331	0,006072	ENSMUSG(
Hoxd10	2	74691924.	0,795374	2,38705	5,230867	0,000143	0,003	ENSMUSG(
Hoxd9	2	74697727.	1,521335	-1,99394	-3,98324	5,04E-05	0,001203	ENSMUSG(
Rbm45	2	76369984.	10,64569	1,072438	2,102984	1,63E-05	0,000457	ENSMUSG(
Osbpl6	2	76406508.	0,834075	-1,87566	-3,66969	2,1E-05	0,000563	ENSMUSG(
Fkbp7	2	compleme	12,34493	1,086928	2,124212	1,19E-05	0,000345	ENSMUSG(
Pde1a	2	compleme	0,07325	2,540884	5,819453	0,001051	0,016142	ENSMUSG(
Zfp804a	2	82053222.	1,199776	-1,9226	-3,79105	1,13E-05	0,00033	ENSMUSG(
Calcrl	2	compleme	6,114316	5,827189	56,77519	0	0	ENSMUSG(
Selenoh	2	compleme	26,2884	1,417343	2,670932	1,86E-09	1,53E-07	ENSMUSG(
Clp1	2	compleme	8,618811	1,18868	2,279441	1,11E-06	4,38E-05	ENSMUSG(
Timm10	2	84826997.	143,6492	1,510722	2,849526	5,38E-13	9,9E-11	ENSMUSG(
Rtn4rl2	2	compleme	0,757118	-2,28699	-4,88038	0,00203	0,028044	ENSMUSG(
Olfr1032	2	86005540.	0,271529	2,103814	4,298444	0,003995	0,049843	ENSMUSG(
Ptprj	2	compleme	2,497442	-1,28078	-2,42971	3,42E-05	0,000861	ENSMUSG(
Kbtbd4	2	90904740.	12,81104	1,329991	2,514011	1,53E-08	1,02E-06	ENSMUSG(
Ptpmt1	2	compleme	11,47661	1,052623	2,074298	1,32E-05	0,00038	ENSMUSG(
Nr1h3	2	compleme	7,188114	1,306916	2,474121	4,34E-07	1,92E-05	ENSMUSG(
Ddb2	2	compleme	6,601383	1,512791	2,853615	9,65E-08	5,15E-06	ENSMUSG(
Pacsin3	2	91255954.	22,14576	1,202194	2,300893	3,58E-08	2,18E-06	ENSMUSG(
Arfgap2	2	91264974.	15,51497	1,18118	2,267621	4,9E-08	2,86E-06	ENSMUSG(
Pex16	2	92374676.	9,037628	1,289834	2,445	3,41E-06	0,000117	ENSMUSG(

Alkbh3	2	compleme	6,448793	1,242859	2,366671	6,34E-07	2,68E-05	ENSMUSG(
Prr5l	2	compleme	0,260494	-3,28615	-9,75503	0,000651	0,010807	ENSMUSG(
Commd9	2	101886247	16,4845	1,471226	2,772574	2,59E-08	1,62E-06	ENSMUSG(
Abtb2	2	10356631(	2,188822	-1,45531	-2,74216	2,46E-06	8,79E-05	ENSMUSG(
Hipk3	2	compleme	14,03692	1,190844	2,282862	2,03E-09	1,64E-07	ENSMUSG(
Cstf3	2	10459052:	8,686946	-1,0462	-2,06508	8,27E-07	3,39E-05	ENSMUSG(
Rcn1	2	compleme	36,75225	-1,00944	-2,01313	2,04E-06	7,48E-05	ENSMUSG(
Gm13925	2	10947700:	4,265784	-2,1735	-4,51117	0,003373	0,043337	ENSMUSG(
Gm13935	2	compleme	6,334288	1,415337	2,66722	0,000517	0,00887	ENSMUSG(
Lpcat4	2	11223946{	3,184216	-3,01989	-8,11109	2,35E-13	4,76E-11	ENSMUSG(
Slc12a6	2	11226582!	1,046099	-2,0691	-4,19625	2,12E-08	1,35E-06	ENSMUSG(
Aven	2	11249296	1,1395	1,314529	2,487211	0,000167	0,003417	ENSMUSG(
Grem1	2	compleme	9,241158	-1,83272	-3,56209	1,09E-08	7,62E-07	ENSMUSG(
Meis2	2	compleme	0,624666	-2,19521	-4,57957	1,71E-06	6,4E-05	ENSMUSG(
Thbs1	2	11811187(	13,62657	-2,18647	-4,5519	5,32E-08	3,07E-06	ENSMUSG(
Chst14	2	11892649(	4,787531	-1,14294	-2,20831	0,000321	0,005924	ENSMUSG(
Rmdn3	2	compleme	2,941414	-1,30241	-2,46641	0,000253	0,004854	ENSMUSG(
Chp1	2	11954769	9,110926	-1,3153	-2,48854	5,76E-08	3,3E-06	ENSMUSG(
Rtf1	2	11967506{	8,207685	-1,67535	-3,19397	2,23E-12	3,53E-10	ENSMUSG(
Rpap1	2	compleme	2,898494	-1,65438	-3,14787	8,92E-10	8,05E-08	ENSMUSG(
Zfp106	2	compleme	3,580317	-1,49957	-2,82759	1,02E-09	9,07E-08	ENSMUSG(
Ppip5k1	2	compleme	1,111026	-1,54627	-2,92061	7,76E-07	3,2E-05	ENSMUSG(
Sord	2	12223474!	5,97123	-1,55126	-2,93072	2,41E-07	1,15E-05	ENSMUSG(
Myef2	2	compleme	4,127509	-3,44813	-10,9142	0	0	ENSMUSG(
Fgf7	2	12603465{	15,71459	5,4139	42,63303	0	0	ENSMUSG(
Snrnp200	2	12720838(	33,3966	-1,779	-3,43188	2,1E-10	2,21E-08	ENSMUSG(
Tmem127	2	12724790{	3,328405	-1,28893	-2,44346	3,38E-06	0,000116	ENSMUSG(
1500011K1	2	compleme	9,216921	1,156583	2,229288	0,001127	0,017092	ENSMUSG(
Ckap2l	2	compleme	14,3639	-1,12539	-2,18161	4,41E-06	0,000146	ENSMUSG(
Il1a	2	compleme	15,26669	-3,3632	-10,2902	2,16E-06	7,85E-05	ENSMUSG(
Il1b	2	compleme	51,81787	-2,95601	-7,75977	0	0	ENSMUSG(
Slc4a11	2	compleme	0,270997	-2,84178	-7,16902	0,000141	0,002956	ENSMUSG(
Cdc25b	2	13118694!	4,230924	-1,16688	-2,24525	1,22E-05	0,000355	ENSMUSG(
Mavs	2	13123406:	1,601686	-1,6903	-3,22724	1,86E-05	0,000511	ENSMUSG(
Rnf24	2	compleme	0,618371	-2,32395	-5,007	3,76E-06	0,000127	ENSMUSG(
Crls1	2	13284666(	16,43213	1,118622	2,171395	1,48E-05	0,000419	ENSMUSG(
Tmx4	2	compleme	3,246166	-1,4661	-2,76273	2,33E-08	1,47E-06	ENSMUSG(
Ankef1	2	13650191(	2,887846	-1,49618	-2,82095	6,7E-06	0,00021	ENSMUSG(
Jag1	2	compleme	26,66373	-1,25018	-2,37871	1,58E-09	1,33E-07	ENSMUSG(
Btbd3	2	13825656!	4,989005	-1,36811	-2,58133	1,41E-06	5,43E-05	ENSMUSG(
Ism1	2	13967817{	1,36845	-2,08508	-4,24297	5,49E-05	0,001296	ENSMUSG(
Esf1	2	compleme	14,00199	-1,95742	-3,88367	0	0	ENSMUSG(
Flrt3	2	compleme	0,582312	2,329913	5,02775	3,76E-06	0,000127	ENSMUSG(
Foxa2	2	compleme	4,225352	-1,31846	-2,49399	0,000712	0,011607	ENSMUSG(
Syndig1	2	14982921:	0,243434	4,173443	18,04394	0,000293	0,005484	ENSMUSG(
C530025M	2	compleme	0,738699	4,359049	20,52128	0,000324	0,005963	ENSMUSG(
Nsfl1c	2	15149418;	28,039	-1,18988	-2,28134	4,39E-09	3,3E-07	ENSMUSG(
Psmf1	2	compleme	3,601143	-1,97974	-3,94423	1,18E-11	1,64E-09	ENSMUSG(

H13	2	15266946:	4,393717	-1,75621	-3,3781	1,51E-08	1,01E-06	ENSMUSG(
Id1	2	15273625:	127,9146	1,0826	2,11785	2,27E-08	1,44E-06	ENSMUSG(
Bcl2l1	2	compleme	9,31549	-1,40071	-2,64031	6,35E-10	5,9E-08	ENSMUSG(
Tpx2	2	15284796	47,22769	-1,07388	-2,10508	4,47E-07	1,98E-05	ENSMUSG(
Plagl2	2	compleme	1,182792	-1,55046	-2,9291	0,000111	0,002409	ENSMUSG(
Kif3b	2	15329141:	6,648395	-1,00015	-2,0002	2,21E-05	0,000587	ENSMUSG(
Asxl1	2	15334584!	4,729962	-1,19371	-2,2874	8,34E-07	3,41E-05	ENSMUSG(
Dnmt3b	2	15364945(	0,981058	-1,29095	-2,4469	0,001334	0,019645	ENSMUSG(
Dynlrb1	2	15523653:	47,17413	-1,52797	-2,88381	1,3E-11	1,77E-09	ENSMUSG(
Map1lc3a	2	15527629	1,683634	-1,93491	-3,82354	0,002406	0,032517	ENSMUSG(
Cep250	2	15595645{	6,845351	-1,09873	-2,14167	3,07E-06	0,000107	ENSMUSG(
Romo1	2	15614403{	133,4413	1,201428	2,299671	2,86E-08	1,77E-06	ENSMUSG(
Epb41l1	2	15642090{	0,769266	-1,53891	-2,90575	9,08E-05	0,002018	ENSMUSG(
Myl9	2	15677542(	24,25506	1,108005	2,155474	1,45E-05	0,000411	ENSMUSG(
Dsn1	2	compleme	12,99757	1,247765	2,374732	7,11E-08	3,95E-06	ENSMUSG(
Manbal	2	15736759	6,503305	-1,67857	-3,20111	0,000113	0,002435	ENSMUSG(
Src	2	15741844	3,733553	-1,35369	-2,55564	5,52E-07	2,38E-05	ENSMUSG(
Ctnnbl1	2	15773740:	31,20794	-1,15851	-2,23227	8,23E-08	4,51E-06	ENSMUSG(
Ralgapb	2	15840984{	5,190469	-1,18613	-2,27541	6,39E-08	3,62E-06	ENSMUSG(
Plcg1	2	16073130(	10,47726	-1,37996	-2,60261	5,03E-10	4,76E-08	ENSMUSG(
Zhx3	2	compleme	2,34962	-1,13288	-2,19297	7,93E-06	0,000244	ENSMUSG(
L3mbtl1	2	16294347:	0,21281	5,965944	62,5069	2,33E-05	0,000617	ENSMUSG(
Mybl2	2	16305468	23,5164	-1,67663	-3,1968	4,92E-12	7,23E-10	ENSMUSG(
Tox2	2	16320312!	1,521589	-3,77233	-13,6642	4,04E-11	5,01E-09	ENSMUSG(
Ada	2	compleme	3,136458	-1,60491	-3,04178	7,84E-05	0,001776	ENSMUSG(
Slpi	2	compleme	3,897944	2,199311	4,5926	5,97E-06	0,00019	ENSMUSG(
Pigt	2	16449752(	29,3127	-1,04121	-2,05795	2,49E-07	1,19E-05	ENSMUSG(
Dnttip1	2	16474598:	15,46999	-1,23911	-2,36053	3,94E-05	0,000973	ENSMUSG(
Zswim1	2	16482268	2,203021	-1,16576	-2,24352	0,001458	0,021208	ENSMUSG(
Ctsa	2	16483287:	18,10855	-1,04334	-2,06099	9,58E-07	3,85E-05	ENSMUSG(
Pcif1	2	16487930	1,365411	-1,16744	-2,24613	0,001312	0,019368	ENSMUSG(
Slc12a5	2	16496080:	0,631747	-1,47219	-2,77443	0,00041	0,007244	ENSMUSG(
Ncoa5	2	compleme	6,729182	-1,80663	-3,49823	1,08E-09	9,53E-08	ENSMUSG(
Elmo2	2	compleme	3,607096	-1,03513	-2,0493	3,62E-05	0,000905	ENSMUSG(
Eya2	2	16559503:	0,40472	-3,94161	-15,3654	0,00269	0,035596	ENSMUSG(
Prex1	2	compleme	11,25086	-1,06636	-2,09414	4,09E-07	1,82E-05	ENSMUSG(
Stau1	2	compleme	15,02882	-1,23244	-2,34964	2,45E-09	1,94E-07	ENSMUSG(
B4galt5	2	compleme	10,27008	-1,48995	-2,80879	9,19E-11	1,04E-08	ENSMUSG(
Spata2	2	compleme	0,840331	-2,5561	-5,88117	7,16E-06	0,000223	ENSMUSG(
Snai1	2	16753819!	3,19395	-1,86772	-3,64954	6,81E-06	0,000213	ENSMUSG(
Ube2v1	2	compleme	5,334743	-1,71325	-3,27899	1,95E-08	1,25E-06	ENSMUSG(
Cebpb	2	16768891!	19,51071	-1,21477	-2,32103	3,1E-05	0,000788	ENSMUSG(
Fam65c	2	compleme	0,687348	-2,34955	-5,09664	0,000839	0,013307	ENSMUSG(
Adnp	2	compleme	1,10541	-1,34391	-2,53838	0,001063	0,016304	ENSMUSG(
Dpm1	2	compleme	31,34879	1,141155	2,205576	5,17E-08	3E-06	ENSMUSG(
Zfp217	2	compleme	2,05404	-1,48812	-2,80523	3,7E-06	0,000125	ENSMUSG(
Dok5	2	17073180	0,555176	3,120171	8,69491	2,95E-05	0,000754	ENSMUSG(
Tfap2c	2	17254959:	4,799418	-1,26072	-2,39616	3E-06	0,000104	ENSMUSG(

Gnas	2	17428432(	44,54723	-1,16291	-2,23909	1,11E-08	7,72E-07	ENSMUSG(
Phactr3	2	17811897!	0,372424	5,414184	42,64143	2,62E-10	2,67E-08	ENSMUSG(
Sycp2	2	compleme	0,636035	1,542391	2,912768	0,000743	0,011998	ENSMUSG(
Cdh4	2	17944243:	0,229825	-3,07371	-8,41933	0,000139	0,002925	ENSMUSG(
Lama5	2	compleme	2,372077	-1,24955	-2,37768	2,49E-05	0,000654	ENSMUSG(
Ythdf1	2	compleme	19,02468	-1,51046	-2,84901	2,03E-10	2,15E-08	ENSMUSG(
Eef1a2	2	compleme	139,5988	-3,67461	-12,7693	6,18E-14	1,42E-11	ENSMUSG(
Zbtb46	2	compleme	1,034546	1,314201	2,486645	0,00078	0,012523	ENSMUSG(
Tpd52	3	compleme	1,491038	-1,78643	-3,4496	0,000506	0,008707	ENSMUSG(
Zfp704	3	compleme	0,356098	-2,43589	-5,41096	1,57E-05	0,000439	ENSMUSG(
Gm6236	3	9899430..!	0,738659	2,880498	7,364044	0,000587	0,009876	ENSMUSG(
Fabp5	3	10012548.	0,480882	3,932584	15,26953	5,28E-08	3,05E-06	ENSMUSG(
E2f5	3	14578641.	5,32766	-1,97176	-3,92247	1,12E-05	0,000328	ENSMUSG(
Dnajc5b	3	19508595.	3,154204	6,22787	74,95068	1,16E-12	1,98E-10	ENSMUSG(
Nlgn1	3	compleme	2,709791	-2,21563	-4,64486	0	0	ENSMUSG(
Pld1	3	27938695.	1,232034	-2,35462	-5,1146	0,00019	0,003798	ENSMUSG(
Tnik	3	28263214.	1,014119	-1,73256	-3,32317	1,15E-05	0,000337	ENSMUSG(
Mecom	3	compleme	4,450166	-1,60053	-3,03255	5,94E-09	4,38E-07	ENSMUSG(
Kcnmb2	3	31902507.	5,524137	7,105117	137,6745	0	0	ENSMUSG(
Ndufb5	3	32736990.	10,02756	1,178748	2,263803	1,67E-06	6,25E-05	ENSMUSG(
Trpc3	3	compleme	0,312674	-2,92414	-7,5902	0,000292	0,005467	ENSMUSG(
Fgf2	3	37348346.	16,41286	-1,11137	-2,1605	1,31E-05	0,000378	ENSMUSG(
Spry1	3	37639947.	1,264575	-2,83023	-7,11189	0,001181	0,017754	ENSMUSG(
Ankrd50	3	compleme	1,287649	-1,08985	-2,12852	0,000613	0,010274	ENSMUSG(
Fat4	3	38886940.	0,432465	-1,69031	-3,22726	2,46E-05	0,000647	ENSMUSG(
Pcdh18	3	compleme	3,62321	1,613206	3,059309	3,15E-08	1,94E-06	ENSMUSG(
Maml3	3	compleme	1,069095	-2,79337	-6,93246	2,04E-12	3,27E-10	ENSMUSG(
Lhfp	3	53041528.	11,61547	1,65195	3,142582	2,75E-05	0,000713	ENSMUSG(
Ufm1	3	compleme	6,491194	1,352112	2,552856	3,09E-09	2,4E-07	ENSMUSG(
Dclk1	3	55242364.	0,829554	2,139043	4,404697	5,76E-08	3,3E-06	ENSMUSG(
Mab21l1	3	55782510.	1,264072	2,34566	5,08293	0,000309	0,005734	ENSMUSG(
Commd2	3	compleme	17,3814	1,000476	2,00066	6,67E-06	0,000209	ENSMUSG(
Rnf13	3	57736062.	17,14043	1,096004	2,137618	2,77E-08	1,72E-06	ENSMUSG(
Tsc22d2	3	58414715.	1,258601	-1,17816	-2,26288	0,00082	0,013057	ENSMUSG(
P2ry1	3	61002795.	0,652667	-1,79517	-3,47056	0,001159	0,017485	ENSMUSG(
Arhgef26	3	62338344.	0,443445	-3,53233	-11,5701	6,87E-08	3,85E-06	ENSMUSG(
Mme	3	63241537.	1,274742	-3,48477	-11,1949	6,83E-07	2,86E-05	ENSMUSG(
Veph1	3	compleme	4,527917	-4,71904	-26,3374	1,22E-15	4,04E-13	ENSMUSG(
Ptx3	3	66219910.	342,2749	-1,04621	-2,0651	0,000686	0,011263	ENSMUSG(
Shox2	3	compleme	3,24422	-1,52872	-2,88529	0,000297	0,005555	ENSMUSG(
Mfsd1	3	67582741.	27,11655	1,045314	2,063816	4,06E-07	1,81E-05	ENSMUSG(
Serpini1	3	75557547.	9,903768	1,637434	3,11112	1,45E-06	5,53E-05	ENSMUSG(
Fstl5	3	76074270.	0,229162	2,294165	4,904699	0,000186	0,003736	ENSMUSG(
Ppid	3	79591342.	165,1486	4,189642	18,24769	0	0	ENSMUSG(
Etfdh	3	compleme	68,2861	4,341055	20,26692	0	0	ENSMUSG(
4930579G:	3	79629079.	25,88476	3,059708	8,338041	1,66E-13	3,43E-11	ENSMUSG(
Fgb	3	compleme	0,681717	4,171816	18,02361	2,56E-10	2,64E-08	ENSMUSG(
Fam160a1	3	compleme	0,615352	-1,43188	-2,69798	0,001727	0,024505	ENSMUSG(

Sh3d19	3	85971109.	3,311065	-1,04821	-2,06796	8,99E-05	0,002002	ENSMUSG(
Rps3a1	3	compleme	400,2903	1,204813	2,305073	2,5E-08	1,57E-06	ENSMUSG(
Etv3	3	87525407.	2,043634	-1,61635	-3,06599	1,86E-08	1,2E-06	ENSMUSG(
Pear1	3	compleme	0,56896	-3,3011	-9,85665	2,25E-08	1,43E-06	ENSMUSG(
Prcc	3	compleme	20,37204	-1,97602	-3,93405	4,36E-13	8,19E-11	ENSMUSG(
Rrnad1	3	compleme	1,157814	-1,43637	-2,70639	0,000115	0,002475	ENSMUSG(
Isg20l2	3	87930314.	5,173928	-1,82763	-3,54953	5,66E-11	6,77E-09	ENSMUSG(
Crabp2	3	87948666.	66,60699	-1,01135	-2,0158	3,51E-06	0,00012	ENSMUSG(
Gpatch4	3	88043108.	6,021433	-1,07762	-2,11056	0,000497	0,00859	ENSMUSG(
Iqgap3	3	88081971.	6,804216	-1,15168	-2,22172	7,67E-08	4,23E-06	ENSMUSG(
Mef2d	3	88142372.	1,461125	-1,53911	-2,90616	6,65E-05	0,001537	ENSMUSG(
Slc25a44	3	compleme	2,57766	-1,35784	-2,56301	9,82E-05	0,002158	ENSMUSG(
Lmna	3	compleme	197,8854	1,167989	2,246983	1,23E-08	8,48E-07	ENSMUSG(
Mex3a	3	88532395.	1,737948	-1,25706	-2,39008	0,000129	0,00273	ENSMUSG(
Ubqln4	3	88553758.	13,47567	-1,68242	-3,20966	6,69E-14	1,53E-11	ENSMUSG(
Arhgef2	3	88605966.	8,849679	-1,42556	-2,68618	2,07E-12	3,29E-10	ENSMUSG(
Rit1	3	88716838.	7,117958	1,092788	2,132858	7,13E-06	0,000222	ENSMUSG(
5830417I1	3	88775216.	1,221086	-1,61301	-3,0589	4,22E-05	0,001031	ENSMUSG(
Rusc1	3	compleme	1,460873	-1,15178	-2,22189	0,001702	0,024228	ENSMUSG(
Scamp3	3	89177473.	25,49162	-1,03652	-2,05127	2,9E-06	0,000101	ENSMUSG(
Fam189b	3	89183143.	1,879944	-1,33123	-2,51617	0,000502	0,008654	ENSMUSG(
Gba	3	89202928.	19,5477	-1,11694	-2,16887	3,93E-07	1,76E-05	ENSMUSG(
Pygo2	3	89430214.	3,58629	-1,1957	-2,29055	4,6E-05	0,001111	ENSMUSG(
Adar	3	89715022.	12,34921	-1,38371	-2,60939	4,09E-10	3,96E-08	ENSMUSG(
Ubap2l	3	compleme	19,41527	-1,78369	-3,44306	3,64E-13	7,04E-11	ENSMUSG(
Rab13	3	90213695.	25,28656	-1,63134	-3,09802	2,48E-11	3,17E-09	ENSMUSG(
Crtc2	3	90254163.	2,560204	-2,41422	-5,33032	1,75E-10	1,88E-08	ENSMUSG(
Dennd4b	3	90265185.	2,740952	-1,61107	-3,05479	9,89E-08	5,25E-06	ENSMUSG(
Gatad2b	3	90293178.	1,704838	-1,67926	-3,20264	2,18E-08	1,39E-06	ENSMUSG(
Ints3	3	compleme	8,22826	-1,06366	-2,09022	1,87E-05	0,000512	ENSMUSG(
Chtop	3	compleme	10,98744	-2,22106	-4,66237	1,57E-12	2,56E-10	ENSMUSG(
S100a13	3	90514435.	49,85785	1,178106	2,262795	9,83E-07	3,93E-05	ENSMUSG(
S100a3	3	90560578.	6,649123	1,509185	2,846491	3,5E-10	3,46E-08	ENSMUSG(
Gm42674	3	90601904.	70,49118	2,519675	5,734528	1,11E-16	4,39E-14	ENSMUSG(
S100a4	3	90603771.	237,0664	2,096727	4,27738	6,14E-12	8,79E-10	ENSMUSG(
S100a6	3	90612882.	835,8347	1,871088	3,658083	0	0	ENSMUSG(
S100a11	3	93520488.	20,89556	-2,54081	-5,81914	1,57E-13	3,26E-11	ENSMUSG(
Tdrkh	3	94413273.	1,126812	-1,23915	-2,3606	0,000809	0,012917	ENSMUSG(
Snx27	3	compleme	5,305762	-1,09991	-2,14342	3,46E-06	0,000118	ENSMUSG(
Tuft1	3	compleme	4,966386	-1,30701	-2,47428	9,16E-06	0,000276	ENSMUSG(
Cgn	3	compleme	0,2188	-3,39658	-10,5311	0,000198	0,003918	ENSMUSG(
Pogz	3	94837567.	1,576399	-1,53772	-2,90336	2,54E-06	9,04E-05	ENSMUSG(
Pip5k1a	3	compleme	30,07128	-1,00575	-2,00798	3,67E-07	1,66E-05	ENSMUSG(
Mllt11	3	compleme	27,60225	-2,77586	-6,84885	1,77E-10	1,9E-08	ENSMUSG(
Prune1	3	compleme	3,233202	-1,20017	-2,29767	6,6E-05	0,001529	ENSMUSG(
Arnt	3	95434388.	6,190929	-1,13634	-2,19823	1,94E-07	9,65E-06	ENSMUSG(
Ctss	3	95526786.	14,17056	-1,11922	-2,1723	0,000379	0,006769	ENSMUSG(
Golph3l	3	95588934.	3,904531	-1,33249	-2,51837	3,69E-05	0,00092	ENSMUSG(

Ecm1	3	compleme	3,120864	-1,93831	-3,83258	1,05E-06	4,18E-05	ENSMUSG(
Anp32e	3	95929246.	36,60283	-1,64308	-3,12331	7,77E-16	2,69E-13	ENSMUSG(
Mtmr11	3	96162004.	1,177368	-2,38148	-5,21072	5,48E-05	0,001296	ENSMUSG(
Sf3b4	3	96172332.	9,009502	-2,44876	-5,45948	2,33E-11	2,98E-09	ENSMUSG(
Hist2h2ab	3	96219865.	1199,118	1,374094	2,592052	1,54E-11	2,06E-09	ENSMUSG(
Hist2h2ac	3	compleme	2401,181	1,434511	2,702905	1,22E-12	2,07E-10	ENSMUSG(
Hist2h3c1	3	96246685.	3,078576	2,218603	4,654424	2,72E-07	1,28E-05	ENSMUSG(
Gm42743	3	96269721.	35,6432	-1,57818	-2,98594	6,84E-12	9,7E-10	ENSMUSG(
Pafah1b1-p	3	compleme	8,829218	-1,303	-2,46742	0,000244	0,004729	ENSMUSG(
Txnip	3	96557957.	4,918319	1,219432	2,32855	0,000621	0,010388	ENSMUSG(
Pias3	3	96696384.	2,419724	-1,58486	-2,99978	9,29E-06	0,000279	ENSMUSG(
Bcl9	3	compleme	0,829891	-1,41055	-2,65839	0,000653	0,010821	ENSMUSG(
Pde4dip	3	compleme	3,044493	-1,06639	-2,09419	8,04E-07	3,31E-05	ENSMUSG(
Notch2	3	98013527.	23,99849	-1,14348	-2,20913	5,18E-08	3E-06	ENSMUSG(
Phgdh	3	compleme	59,61796	-1,20903	-2,31182	1,7E-08	1,11E-06	ENSMUSG(
Zfp697	3	98382461.	0,997099	-2,0683	-4,19391	1,42E-07	7,28E-06	ENSMUSG(
Tbx15	3	99240381.	13,57006	-1,18458	-2,27297	9,98E-08	5,3E-06	ENSMUSG(
Fam46c	3	compleme	1,253165	1,998994	3,997211	1,84E-05	0,000505	ENSMUSG(
Igsf3	3	101377083	6,342347	-1,60376	-3,03935	1,23E-10	1,37E-08	ENSMUSG(
Atp1a1	3	compleme	88,64473	-1,60647	-3,04505	1,61E-08	1,06E-06	ENSMUSG(
Slc22a15	3	compleme	0,438616	-1,55405	-2,93641	0,002423	0,032711	ENSMUSG(
Hipk1	3	compleme	14,37967	-1,29455	-2,453	1,76E-09	1,46E-07	ENSMUSG(
Gm43064	3	103809573	0,201527	7,171004	144,1078	0,002059	0,028397	ENSMUSG(
Ptpn22	3	103859795	2,619105	-5,03439	-32,7719	9,61E-09	6,73E-07	ENSMUSG(
Mov10	3	compleme	7,845857	-1,86009	-3,63031	2,1E-14	5,53E-12	ENSMUSG(
Cttnbp2nl	3	compleme	3,646416	-1,91419	-3,76903	1,39E-12	2,31E-10	ENSMUSG(
Dennd2d	3	106482405	0,261037	2,769476	6,818603	1,95E-05	0,000532	ENSMUSG(
Lamtor5	3	107278858	27,08453	-1,2673	-2,40711	1,06E-06	4,21E-05	ENSMUSG(
Kcnc4	3	compleme	1,495463	-1,06763	-2,09599	0,002842	0,037388	ENSMUSG(
Slc6a17	3	compleme	1,5031	-2,08161	-4,2328	5,39E-10	5,05E-08	ENSMUSG(
Alx3	3	107595031	5,261917	-1,33684	-2,52597	1,7E-05	0,000473	ENSMUSG(
Ahcyl1	3	compleme	38,74404	-1,34626	-2,54252	5,51E-07	2,38E-05	ENSMUSG(
Gm9515	3	compleme	8,373742	-1,5104	-2,84889	4,87E-05	0,00117	ENSMUSG(
Gstm6	3	compleme	6,661052	-1,19155	-2,28398	3,47E-05	0,000872	ENSMUSG(
Gstm3	3	compleme	9,784826	-1,58788	-3,00607	8,54E-08	4,63E-06	ENSMUSG(
Atxn7l2	3	compleme	3,349998	-1,08024	-2,11439	2,54E-05	0,000665	ENSMUSG(
Sort1	3	108284082	0,402276	-2,25566	-4,77552	0,00031	0,005753	ENSMUSG(
Psrc1	3	108383839	3,362602	-1,56309	-2,95486	7,48E-06	0,000232	ENSMUSG(
Celsr2	3	compleme	1,274267	-1,89737	-3,72533	3,26E-06	0,000112	ENSMUSG(
5330417C2	3	compleme	0,275468	-1,9651	-3,9044	0,001474	0,021397	ENSMUSG(
Prpf38b	3	compleme	14,46375	-1,95882	-3,88744	6,86E-14	1,55E-11	ENSMUSG(
Ntng1	3	compleme	0,791096	-2,21431	-4,64059	2,88E-07	1,34E-05	ENSMUSG(
S1pr1	3	compleme	1,403104	-5,94523	-61,616	2,33E-08	1,47E-06	ENSMUSG(
Slc35a3	3	compleme	2,457175	1,010688	2,014872	1,72E-05	0,000477	ENSMUSG(
Palmd	3	compleme	1,814061	-1,58481	-2,99967	0,000868	0,013699	ENSMUSG(
Plppr4	3	compleme	2,348907	1,890983	3,708877	1,55E-06	5,89E-05	ENSMUSG(
Plppr5	3	117574836	0,161414	2,34409	5,0774	0,00208	0,028618	ENSMUSG(
Dpyd	3	118562129	16,98258	1,363156	2,572474	2,1E-12	3,33E-10	ENSMUSG(

Rwdd3	3	compleme	1,201272	1,011304	2,015733	0,002334	0,03165	ENSMUSG0
Tmem56	3	compleme	0,249484	-2,42439	-5,36803	0,002898	0,037952	ENSMUSG0
Pde5a	3	122728947	2,711862	2,660537	6,322684	0	0	ENSMUSG0
Ndst3	3	compleme	0,24933	3,681049	12,82644	7,34E-07	3,05E-05	ENSMUSG0
Ank2	3	compleme	1,301273	-2,39001	-5,24162	1,64E-10	1,78E-08	ENSMUSG0
Zgrf1	3	127553489	2,369016	1,212391	2,317213	9,09E-07	3,67E-05	ENSMUSG0
Gar1	3	compleme	2,735808	-1,82734	-3,54883	8,28E-05	0,00186	ENSMUSG0
Cfi	3	129835884	1,607485	2,194002	4,575731	9,52E-06	0,000285	ENSMUSG0
Col25a1	3	130131501	0,038542	2,559741	5,89602	0,001771	0,025027	ENSMUSG0
Ostc	3	compleme	85,19289	1,051121	2,072139	1,37E-07	7,08E-06	ENSMUSG0
Rpl34	3	compleme	21,45787	1,187529	2,277623	2,86E-07	1,33E-05	ENSMUSG0
Lef1	3	131110471	0,432494	-2,33448	-5,04369	6,87E-06	0,000215	ENSMUSG0
Ints12	3	133091840	3,008977	-1,01011	-2,01407	0,000229	0,004474	ENSMUSG0
Tet2	3	compleme	0,512786	-1,28702	-2,44023	0,00037	0,006641	ENSMUSG0
Pdlim5	3	compleme	9,853125	-1,53132	-2,8905	1,39E-12	2,31E-10	ENSMUSG0
Cyr61	3	compleme	14,5252	-1,50387	-2,83602	2,25E-06	8,11E-05	ENSMUSG0
Wdr63	3	compleme	0,855617	2,484928	5,598065	0,000388	0,006924	ENSMUSG0
Mcoln3	3	146117450	3,043317	1,191824	2,284415	0,000856	0,01355	ENSMUSG0
Mcoln2	3	146149833	2,126563	1,1079	2,155317	0,001206	0,018061	ENSMUSG0
Lpar3	3	146220963	9,328394	1,736813	3,332981	3,66E-08	2,21E-06	ENSMUSG0
Ssx2ip	3	146404642	17,33752	1,13974	2,203412	1,28E-06	4,98E-05	ENSMUSG0
Ttll7	3	146852367	1,161144	-2,2082	-4,62099	5,83E-06	0,000187	ENSMUSG0
Adgrl2	3	compleme	6,816896	-7,96915	-250,584	7,78E-14	1,73E-11	ENSMUSG0
Gm43573	3	148989152	0,355894	3,172109	9,013633	0,002717	0,035922	ENSMUSG0
Ifi44	3	compleme	8,493191	1,036698	2,051527	0,000316	0,005851	ENSMUSG0
Ifi44l	3	compleme	0,386288	2,722046	6,598079	0,00052	0,00891	ENSMUSG0
Ptgfr	3	compleme	2,276957	2,450715	5,46687	1,2E-14	3,3E-12	ENSMUSG0
Nexn	3	compleme	3,528951	-1,3731	-2,59027	0,001181	0,017748	ENSMUSG0
Ak5	3	compleme	32,292	1,148614	2,217007	2,37E-06	8,51E-05	ENSMUSG0
Pigk	3	152714100	9,86093	1,16043	2,23524	5,17E-08	3E-06	ENSMUSG0
Cryz	3	154596711	11,22294	1,023125	2,032316	4,75E-06	0,000156	ENSMUSG0
Fpgt	3	compleme	2,716244	1,156136	2,228598	9,41E-05	0,002078	ENSMUSG0
Lrriq3	3	155093434	0,159549	2,353548	5,110797	0,000923	0,014408	ENSMUSG0
Lyn	4	3678115..3	2,688705	-1,25905	-2,39338	0,000361	0,006519	ENSMUSG0
Car8	4	compleme	0,959326	-5,71641	-52,579	0,000196	0,003894	ENSMUSG0
Chd7	4	8690406..8	0,884899	-2,95948	-7,77846	7,05E-13	1,26E-10	ENSMUSG0
Gdf6	4	9844372..9	2,464036	2,758174	6,765394	1,72E-08	1,12E-06	ENSMUSG0
Plekhf2	4	compleme	9,562575	1,798601	3,478827	6,89E-13	1,24E-10	ENSMUSG0
Ccne2	4	11191351.	33,06806	1,240083	2,362122	3,37E-09	2,59E-07	ENSMUSG0
Esrp1	4	compleme	0,134441	-4,57018	-23,7553	0,002652	0,03519	ENSMUSG0
Rbm12b2	4	12089439.	4,386787	-1,4376	-2,70869	3,06E-06	0,000106	ENSMUSG0
Ripk2	4	compleme	4,486375	-1,62703	-3,08876	8,78E-07	3,56E-05	ENSMUSG0
Ccnc	4	21727701.	34,79946	1,095961	2,137554	4,03E-07	1,81E-05	ENSMUSG0
Usp45	4	21767156.	3,939371	1,145359	2,212011	5,11E-06	0,000166	ENSMUSG0
Ankrd6	4	compleme	0,22079	-3,56137	-11,8054	0,000598	0,010063	ENSMUSG0
Rragd	4	32983037.	2,417244	-5,25485	-38,1829	8,91E-08	4,82E-06	ENSMUSG0
Ube2j1	4	33031416.	8,611124	-1,03758	-2,05278	1,29E-05	0,000373	ENSMUSG0
Chmp5	4	40948407.	111,7445	1,091255	2,130592	2,07E-07	1,02E-05	ENSMUSG0

Aqp3	4	compleme	3,292928	-3,11546	-8,66659	0,000313	0,005795	ENSMUSG(
Dcaf12	4	compleme	5,894748	-1,24683	-2,37319	1,54E-06	5,83E-05	ENSMUSG(
Ubap1	4	41348996.	7,229492	-1,64417	-3,12568	3,08E-10	3,08E-08	ENSMUSG(
Fam219a	4	compleme	2,829395	-1,76625	-3,40169	1,71E-07	8,6E-06	ENSMUSG(
Il11ra1	4	41699989.	0,696517	1,473557	2,777057	0,001492	0,021635	ENSMUSG(
Dnajb5	4	42949814.	0,510971	-1,92232	-3,79032	0,000307	0,005714	ENSMUSG(
Fam214b	4	compleme	2,78154	-2,13313	-4,38669	1,61E-10	1,75E-08	ENSMUSG(
Rusc2	4	43381979.	18,63153	-1,73329	-3,32486	0	0	ENSMUSG(
Tesk1	4	43441939.	9,086072	-1,51382	-2,85565	5,61E-09	4,16E-07	ENSMUSG(
Tln1	4	compleme	98,06476	-1,21268	-2,31768	5,96E-09	4,39E-07	ENSMUSG(
Rgp1	4	43578715.	13,87562	-1,59943	-3,03024	9,81E-14	2,11E-11	ENSMUSG(
Gm12472	4	43588072.	1,566582	2,066722	4,189338	0,000185	0,00371	ENSMUSG(
Glipr2	4	43957401.	5,081009	-1,36484	-2,57547	7,57E-05	0,001726	ENSMUSG(
Rnf38	4	compleme	2,254471	-1,27153	-2,41417	5,19E-07	2,26E-05	ENSMUSG(
Gm12677	4	compleme	0,476196	3,171868	9,012127	0,003695	0,046785	ENSMUSG(
Tmod1	4	46038935.	0,408244	-2,26312	-4,80028	0,001086	0,016612	ENSMUSG(
Anp32b	4	46450902.	12,67822	-2,01676	-4,04673	1,29E-08	8,85E-07	ENSMUSG(
Col15a1	4	47208161.	0,790087	-2,17659	-4,52084	0,000697	0,011409	ENSMUSG(
Nipsnap3a	4	52989272.	8,947332	1,47417	2,778238	8,99E-07	3,64E-05	ENSMUSG(
Nipsnap3b	4	53011880.	9,783047	1,573716	2,976705	3,48E-08	2,13E-06	ENSMUSG(
Abca1	4	compleme	8,859912	-2,69292	-6,46622	2,82E-06	9,92E-05	ENSMUSG(
Zfp462	4	54945048.	3,103555	-1,72399	-3,30349	6,89E-13	1,24E-10	ENSMUSG(
Klf4	4	compleme	2,963086	-1,34853	-2,54652	3,54E-05	0,000888	ENSMUSG(
Fam206a	4	56802345.	0,350217	-2,48089	-5,58243	0,000135	0,00286	ENSMUSG(
Ctnnal1	4	compleme	39,06398	1,012451	2,017335	7,11E-07	2,97E-05	ENSMUSG(
Epb41l4b	4	compleme	1,442914	-1,32577	-2,50667	8,58E-05	0,001917	ENSMUSG(
Palm2	4	57434247.	0,716293	-3,29495	-9,81476	0,00016	0,003289	ENSMUSG(
Pakap	4	57568179.	13,75738	-2,17578	-4,51829	3,92E-12	5,9E-10	ENSMUSG(
Akap2	4	57717657.	8,418773	-1,5142	-2,8564	3,83E-07	1,72E-05	ENSMUSG(
Inip	4	compleme	1,999845	1,001863	2,002584	0,0012	0,017993	ENSMUSG(
Zfp618	4	62965573.	1,079484	-1,09441	-2,13526	0,002425	0,032732	ENSMUSG(
Tmem268	4	63558781.	0,868247	-1,81556	-3,51996	9,97E-05	0,002184	ENSMUSG(
Pappa	4	65124174.	1,222744	-4,63034	-24,7669	8,96E-12	1,25E-09	ENSMUSG(
Tle1	4	compleme	1,01067	-1,37307	-2,5902	0,000187	0,003746	ENSMUSG(
Frmd3	4	74013442.	0,889448	-3,70178	-13,0121	8,35E-08	4,54E-06	ENSMUSG(
Tmem261	4	compleme	8,646062	1,10834	2,155974	0,00077	0,012379	ENSMUSG(
Gm11765	4	compleme	18,10415	-1,66186	-3,16424	3,72E-08	2,25E-06	ENSMUSG(
Adamtsl1	4	85514172.	1,971152	-1,18538	-2,27423	9,19E-07	3,71E-05	ENSMUSG(
Rraga	4	86575668.	16,60658	-1,14902	-2,21763	4,41E-05	0,001071	ENSMUSG(
Dennd4c	4	86748555.	5,603313	-1,02413	-2,03374	5,41E-05	0,001279	ENSMUSG(
Klhl9	4	compleme	50,25442	1,400089	2,639178	5,36E-08	3,09E-06	ENSMUSG(
Mtap	4	89137122.	11,68214	2,122561	4,354662	2,9E-09	2,27E-07	ENSMUSG(
Caap1	4	compleme	3,257142	1,472089	2,774234	1,07E-05	0,000316	ENSMUSG(
Ift74	4	94614491.	9,279784	1,166694	2,244967	1,11E-05	0,000326	ENSMUSG(
Nfia	4	97772734.	0,668675	-2,97199	-7,84617	6,71E-11	7,81E-09	ENSMUSG(
Gm12846	4	98664366.	144,8592	1,037723	2,052985	4,56E-06	0,000151	ENSMUSG(
Foxd3	4	99656299.	0,810509	-1,76591	-3,40089	0,003492	0,044613	ENSMUSG(
Itgb3bp	4	compleme	5,824722	1,036517	2,051269	0,000236	0,004586	ENSMUSG(

Ror1	4	100095791	2,80355	-1,47395	-2,77782	2,19E-05	0,000583	ENSMUSG0
Dnajc6	4	101496648	0,931564	-3,58157	-11,9718	1,93E-09	1,58E-07	ENSMUSG0
Oma1	4	103313812	13,07353	1,148289	2,216509	4,28E-06	0,000143	ENSMUSG0
Prkaa2	4	compleme	1,354288	-1,73081	-3,31915	0,000103	0,002247	ENSMUSG0
Plpp3	4	105157347	11,45074	1,313711	2,485802	0,000109	0,00237	ENSMUSG0
Ssbp3	4	106910701	1,57452	-2,27941	-4,85478	6,97E-08	3,9E-06	ENSMUSG0
Glis1	4	107434591	0,64642	-1,5464	-2,92087	0,003842	0,048276	ENSMUSG0
Gpx7	4	compleme	8,541659	1,176919	2,260934	9,05E-05	0,002014	ENSMUSG0
3110021N2	4	108719649	0,149506	2,989178	7,940215	0,002245	0,030608	ENSMUSG0
Foxd2	4	compleme	3,900908	-1,03702	-2,05198	0,001688	0,024063	ENSMUSG0
Gm12848	4	115652389	309,3498	1,16788	2,246813	1,74E-08	1,13E-06	ENSMUSG0
Mknk1	4	115839198	1,982296	-1,18134	-2,26788	0,000669	0,011037	ENSMUSG0
Dmbx1	4	compleme	0,130221	-3,53283	-11,5741	0,001761	0,024922	ENSMUSG0
Mast2	4	compleme	3,364555	-2,48634	-5,60355	4,44E-16	1,61E-13	ENSMUSG0
Mmachc	4	compleme	1,60151	-1,42046	-2,67671	0,002013	0,027873	ENSMUSG0
Gm12993	4	compleme	10,92667	1,906758	3,749655	6,71E-05	0,00155	ENSMUSG0
Hectd3	4	116995317	2,692122	-1,21491	-2,32126	9,92E-05	0,002175	ENSMUSG0
Dmap1	4	compleme	5,173893	-1,28824	-2,4423	0,000185	0,003707	ENSMUSG0
Slc6a9	4	117834506	2,697464	-2,01171	-4,0326	2,19E-10	2,28E-08	ENSMUSG0
St3gal3	4	compleme	3,866875	-1,18374	-2,27165	0,00012	0,002563	ENSMUSG0
Ptprf	4	compleme	10,94066	-3,61408	-12,2446	0	0	ENSMUSG0
Szt2	4	compleme	1,977016	-1,2358	-2,35512	8,08E-06	0,000248	ENSMUSG0
Cdc20	4	compleme	99,60599	-1,01285	-2,01789	2,72E-06	9,6E-05	ENSMUSG0
Foxj3	4	119537004	6,253528	-1,02755	-2,03857	2,11E-06	7,68E-05	ENSMUSG0
Mfsd2a	4	compleme	13,10776	-1,18438	-2,27266	1,48E-05	0,000418	ENSMUSG0
Pabpc4	4	123262351	10,72764	-1,04159	-2,05849	1,13E-05	0,00033	ENSMUSG0
Macf1	4	compleme	10,53199	-1,15529	-2,22729	4,23E-07	1,88E-05	ENSMUSG0
Mtf1	4	124802104	0,903954	-2,03489	-4,09791	2,03E-07	1,01E-05	ENSMUSG0
9930104L0	4	124937048	1,944572	1,002104	2,002919	0,000964	0,014989	ENSMUSG0
Zc3h12a	4	compleme	10,50059	-1,79058	-3,45954	1,75E-08	1,14E-06	ENSMUSG0
Lsm10	4	126096623	6,093678	-1,85595	-3,6199	3,01E-05	0,000768	ENSMUSG0
Map7d1	4	compleme	37,54796	-1,22831	-2,34293	4,86E-10	4,62E-08	ENSMUSG0
Adprhl2	4	compleme	14,07217	1,112474	2,162161	9,41E-07	3,79E-05	ENSMUSG0
Clspn	4	126556935	8,925721	-1,06325	-2,08963	8,48E-06	0,000258	ENSMUSG0
Psmb2	4	126677630	136,054	-1,22876	-2,34365	5,05E-10	4,76E-08	ENSMUSG0
AU040320	4	126753544	4,964692	-1,19677	-2,29226	1,65E-06	6,2E-05	ENSMUSG0
Tmem35b	4	127123691	2,68915	1,447519	2,727385	0,000395	0,007015	ENSMUSG0
Tmem54	4	129105548	5,753417	-1,57501	-2,97937	0,000887	0,013942	ENSMUSG0
Sync	4	129287617	9,591168	1,663865	3,168643	5,25E-10	4,94E-08	ENSMUSG0
Gm26722	4	129309081	5,484777	2,095237	4,272965	2,85E-07	1,33E-05	ENSMUSG0
Marcksl1	4	129513581	12,08521	-2,27966	-4,85563	3,69E-13	7,1E-11	ENSMUSG0
Tmem39b	4	compleme	5,790169	-1,44274	-2,71837	1,38E-05	0,000394	ENSMUSG0
Spocd1	4	129929249	2,494929	-1,47916	-2,78786	2,05E-05	0,000553	ENSMUSG0
Serinc2	4	compleme	37,44594	-1,50454	-2,83733	4,11E-12	6,17E-10	ENSMUSG0
Fabp3	4	130308595	2,63334	8,984487	506,5241	9,27E-05	0,002053	ENSMUSG0
Pum1	4	130663321	20,24323	-1,08482	-2,12111	3,73E-07	1,69E-05	ENSMUSG0
Srsf4	4	131873617	16,16586	-1,75751	-3,38114	7,36E-14	1,65E-11	ENSMUSG0
Epb41	4	compleme	0,87234	-1,68146	-3,20752	2,56E-05	0,00067	ENSMUSG0

Taf12	4	13227437[illegible]	22,00722	1,030208	2,042319	4,6E-07	2,03E-05	ENSMUSG[illegible]
Snhg3	4	compleme	12,43047	-1,48588	-2,80087	3E-06	0,000104	ENSMUSG[illegible]
Sesn2	4	compleme	1,838135	-1,63664	-3,1094	3,48E-05	0,000874	ENSMUSG[illegible]
Gm13033	4	13288476[illegible]	7,995138	-1,44884	-2,72988	0,001096	0,016737	ENSMUSG[illegible]
Ahdc1	4	13301126[illegible]	0,736417	-2,18724	-4,55432	2,38E-07	1,14E-05	ENSMUSG[illegible]
Wasf2	4	13313050[illegible]	4,740145	-1,30923	-2,47809	5,39E-06	0,000174	ENSMUSG[illegible]
Gpr3	4	compleme	1,243022	-1,6995	-3,24789	0,000185	0,00371	ENSMUSG[illegible]
Wdtc1	4	compleme	5,805672	-1,27134	-2,41386	8,71E-07	3,54E-05	ENSMUSG[illegible]
Slc9a1	4	13336970[illegible]	1,119673	-1,74196	-3,34489	1,48E-05	0,000418	ENSMUSG[illegible]
Nudc	4	compleme	140,6908	-1,84776	-3,59941	1,87E-14	5,02E-12	ENSMUSG[illegible]
Sfn	4	compleme	5,344777	-3,10168	-8,58418	3,6E-08	2,19E-06	ENSMUSG[illegible]
Zdhhc18	4	compleme	2,915248	-1,3992	-2,63755	1,69E-07	8,52E-06	ENSMUSG[illegible]
Arid1a	4	compleme	1,924067	-1,95221	-3,86968	9,78E-07	3,91E-05	ENSMUSG[illegible]
Sh3bgrl3	4	compleme	60,7006	-1,77292	-3,41745	3,56E-12	5,42E-10	ENSMUSG[illegible]
Selenon	4	compleme	4,096097	-1,95537	-3,87816	6,28E-09	4,6E-07	ENSMUSG[illegible]
Runx3	4	13512065[illegible]	0,924871	-2,28331	-4,86794	1,51E-06	5,76E-05	ENSMUSG[illegible]
Myom3	4	13575971[illegible]	3,032239	-3,51148	-11,4041	7,07E-09	5,13E-07	ENSMUSG[illegible]
Eloa	4	compleme	12,45374	-1,36564	-2,57691	1,55E-09	1,31E-07	ENSMUSG[illegible]
E2f2	4	13617239[illegible]	2,588367	-1,1155	-2,16671	0,000183	0,003681	ENSMUSG[illegible]
Htr1d	4	13642352[illegible]	0,48076	-2,97182	-7,84526	0,000191	0,003815	ENSMUSG[illegible]
Luzp1	4	13646976[illegible]	7,231356	-1,26263	-2,39932	2,07E-07	1,02E-05	ENSMUSG[illegible]
Hspg2	4	13746876[illegible]	7,980392	-1,60413	-3,04011	3,68E-09	2,81E-07	ENSMUSG[illegible]
Rap1gap	4	13766472[illegible]	1,391198	-2,10385	-4,29856	7,99E-09	5,71E-07	ENSMUSG[illegible]
Alpl	4	compleme	0,533432	-2,91881	-7,56221	1,71E-05	0,000476	ENSMUSG[illegible]
Ece1	4	13786223[illegible]	19,83031	-1,45682	-2,74503	1,59E-12	2,58E-10	ENSMUSG[illegible]
Camk2n1	4	13845431[illegible]	5,940681	2,133504	4,387819	0	0	ENSMUSG[illegible]
Capzb	4	13919289[illegible]	31,71691	-1,38722	-2,61574	1,5E-09	1,27E-07	ENSMUSG[illegible]
Ubr4	4	13935260[illegible]	8,63132	-1,55508	-2,93851	1,33E-10	1,48E-08	ENSMUSG[illegible]
Atp13a2	4	14098687[illegible]	3,367763	-1,28268	-2,43291	6,37E-07	2,69E-05	ENSMUSG[illegible]
Szrd1	4	compleme	28,56592	-1,75637	-3,37848	5,82E-14	1,35E-11	ENSMUSG[illegible]
Fam131c	4	14136822[illegible]	0,682348	-3,90346	-14,9643	0,000548	0,009325	ENSMUSG[illegible]
Plekhm2	4	compleme	9,211205	-1,54387	-2,91575	1,67E-11	2,22E-09	ENSMUSG[illegible]
Ddi2	4	compleme	1,136105	-1,07846	-2,11178	0,000935	0,014567	ENSMUSG[illegible]
Agmat	4	14174667[illegible]	1,516558	-4,76473	-27,1849	0,000252	0,004852	ENSMUSG[illegible]
Tmem51	4	compleme	4,335648	-1,274	-2,41831	0,000361	0,006515	ENSMUSG[illegible]
Kazn	4	compleme	0,483844	-1,29823	-2,45926	0,001204	0,018043	ENSMUSG[illegible]
Plod1	4	compleme	20,21972	-1,46271	-2,75626	2,43E-12	3,83E-10	ENSMUSG[illegible]
Agtrap	4	compleme	5,250748	-1,00872	-2,01213	0,000984	0,015263	ENSMUSG[illegible]
Ubiad1	4	compleme	4,885375	-1,25955	-2,39421	9,78E-05	0,00215	ENSMUSG[illegible]
Mtor	4	14844861[illegible]	12,6606	-1,15762	-2,2309	3,72E-09	2,84E-07	ENSMUSG[illegible]
Srm	4	14859150[illegible]	8,020447	-1,58756	-3,0054	9,4E-06	0,000282	ENSMUSG[illegible]
Pex14	4	compleme	3,589003	-1,25884	-2,39303	6,89E-07	2,88E-05	ENSMUSG[illegible]
Kif1b	4	compleme	4,804286	-1,11407	-2,16455	6,79E-08	3,81E-06	ENSMUSG[illegible]
Ube4b	4	compleme	6,286694	-1,44064	-2,71442	8,24E-09	5,87E-07	ENSMUSG[illegible]
Clstn1	4	14958646[illegible]	11,52199	-2,16866	-4,49605	0	0	ENSMUSG[illegible]
Pik3cd	4	compleme	5,350521	-3,07968	-8,45425	0	0	ENSMUSG[illegible]
Spsb1	4	compleme	6,116821	-1,30103	-2,46405	2,5E-08	1,57E-06	ENSMUSG[illegible]
Tnfrsf9	4	15091456[illegible]	0,716178	-2,7703	-6,82248	0,000627	0,010472	ENSMUSG[illegible]

Nol9	4	15203932	1,79877	-1,17206	-2,25333	0,001242	0,018529	ENSMUSG(
Tnfrsf25	4	15211593	0,524063	-2,08368	-4,23889	0,002856	0,037489	ENSMUSG(
Acot7	4	15217813	29,08053	-1,6309	-3,09707	1,48E-13	3,09E-11	ENSMUSG(
Gpr153	4	15227423	1,054648	1,874906	3,667776	0,000635	0,010589	ENSMUSG(
Chd5	4	15233865	1,244076	-1,7473	-3,35729	2,49E-07	1,19E-05	ENSMUSG(
Kcnab2	4	compleme	1,542565	-2,68503	-6,43096	4,57E-10	4,39E-08	ENSMUSG(
Nphp4	4	15247670	1,067254	-1,06331	-2,08972	0,003921	0,049116	ENSMUSG(
Cep104	4	15397519	2,482393	-1,38733	-2,61595	8,75E-06	0,000265	ENSMUSG(
Tprgl	4	compleme	2,467351	-1,67296	-3,18867	5,91E-05	0,001388	ENSMUSG(
Megf6	4	15417073	5,592918	1,179277	2,264632	5,46E-06	0,000176	ENSMUSG(
Arhgef16	4	compleme	3,587989	-3,59229	-12,0611	1,48E-09	1,26E-07	ENSMUSG(
Gnb1	4	15549136	44,70414	-1,23128	-2,34776	2,29E-07	1,11E-05	ENSMUSG(
Cdk11b	4	15562485	12,11975	-1,70848	-3,26816	6,11E-09	4,48E-07	ENSMUSG(
Isg15	4	compleme	18,21842	1,734976	3,328738	3,98E-05	0,000984	ENSMUSG(
Adam22	5	compleme	0,239104	-1,59404	-3,01893	0,002809	0,037015	ENSMUSG(
Abcb1a	5	8660077..8	1,318699	2,803031	6,979053	2,4E-07	1,15E-05	ENSMUSG(
Abcb1b	5	8798147..8	1,13796	3,331508	10,06662	6,6E-11	7,73E-09	ENSMUSG(
Crot	5	compleme	1,948223	1,302945	2,46732	0,000359	0,006501	ENSMUSG(
Sema3d	5	12383385.	4,179008	3,710003	13,08646	3,33E-16	1,24E-13	ENSMUSG(
Sema3a	5	13125414.	13,26352	1,158877	2,232836	5,01E-09	3,75E-07	ENSMUSG(
Pclo	5	14514918.	0,085515	-2,21546	-4,64429	0,002438	0,032826	ENSMUSG(
Cacna2d1	5	15934691.	1,747983	-1,74066	-3,34189	4,99E-10	4,73E-08	ENSMUSG(
Hgf	5	16553495.	21,36571	1,546256	2,920582	1,01E-05	0,0003	ENSMUSG(
Cd36	5	compleme	0,351736	2,67923	6,405141	2,58E-05	0,000672	ENSMUSG(
Napepld	5	compleme	1,51418	1,316984	2,491447	0,00073	0,011833	ENSMUSG(
Tmub1	5	compleme	7,992332	-1,01422	-2,01982	0,000682	0,01121	ENSMUSG(
Prkag2	5	compleme	1,347169	-1,61249	-3,0578	1,33E-05	0,000382	ENSMUSG(
Insig1	5	28071363.	11,68363	1,371478	2,587354	6,37E-07	2,69E-05	ENSMUSG(
Mapre3	5	30814641.	1,850045	-1,66876	-3,17941	1,57E-05	0,000439	ENSMUSG(
Tmem214	5	30868012.	2,056067	-2,05413	-4,15293	8,57E-13	1,49E-10	ENSMUSG(
Cad	5	31054780.	12,55044	-1,14283	-2,20814	3,35E-05	0,000845	ENSMUSG(
Zfp513	5	compleme	2,171873	-1,27253	-2,41585	0,0014	0,02049	ENSMUSG(
Supt7l	5	compleme	4,337536	-1,3083	-2,47649	6,89E-07	2,88E-05	ENSMUSG(
Bre	5	31697684.	5,807811	-1,61202	-3,0568	2,69E-09	2,13E-07	ENSMUSG(
Fosl2	5	32135801.	5,825633	-1,7436	-3,34869	6,46E-13	1,17E-10	ENSMUSG(
Prr14l	5	compleme	1,017648	-1,28551	-2,43768	0,000393	0,006977	ENSMUSG(
Spon2	5	compleme	1,034921	1,889689	3,705555	0,00015	0,003113	ENSMUSG(
Tacc3	5	33658128.	2,302662	-1,58015	-2,99001	6,35E-05	0,00148	ENSMUSG(
Nelfa	5	compleme	2,476843	-1,01484	-2,02068	0,000835	0,013264	ENSMUSG(
Nat8l	5	33995984.	0,802462	-1,44816	-2,7286	0,000243	0,004711	ENSMUSG(
Htt	5	34761740.	1,353368	-1,05168	-2,07294	4,34E-05	0,001056	ENSMUSG(
Mrfap1	5	compleme	47,92672	-1,30447	-2,46993	1,22E-06	4,77E-05	ENSMUSG(
Man2b2	5	compleme	1,652742	-1,20638	-2,30758	0,003457	0,044257	ENSMUSG(
Ppp2r2c	5	36868513.	2,934264	-1,84601	-3,59504	5,99E-08	3,41E-06	ENSMUSG(
Wfs1	5	compleme	3,145828	-1,02843	-2,03981	0,001226	0,018335	ENSMUSG(
Wdr1	5	compleme	45,83464	-1,47631	-2,78235	7,87E-12	1,11E-09	ENSMUSG(
Bod1l	5	compleme	2,51318	-1,48482	-2,79883	3,79E-07	1,71E-05	ENSMUSG(
Bst1	5	43818885.	0,731754	2,103917	4,298749	5,16E-05	0,001227	ENSMUSG(

Fgfbp1	5	compleme	2,53973	4,658318	25,25186	3,53E-10	3,48E-08	ENSMUSG(
Slit2	5	47983138.	2,015063	-1,07457	-2,10609	0,000175	0,003551	ENSMUSG(
Ppargc1a	5	compleme	0,327161	-2,4094	-5,31255	0,000135	0,002857	ENSMUSG(
Ccdc149	5	compleme	3,35242	-1,28774	-2,44146	0,000345	0,006278	ENSMUSG(
Lgi2	5	compleme	1,390497	-1,73382	-3,32607	5,31E-05	0,001258	ENSMUSG(
Smim20	5	53267083.	9,610092	1,093855	2,134436	0,000344	0,006259	ENSMUSG(
Cckar	5	compleme	0,101296	4,629141	24,7463	0,000468	0,008159	ENSMUSG(
Tbc1d19	5	53809606.	4,388014	1,43108	2,696485	1,07E-06	4,23E-05	ENSMUSG(
Pcdh7	5	57717967.	1,107797	1,046942	2,066146	0,002321	0,031514	ENSMUSG(
Rpl9	5	compleme	225,7063	1,086718	2,123903	7,29E-06	0,000226	ENSMUSG(
Ube2k	5	65537233.	5,3745	-1,00526	-2,00731	4,28E-05	0,001042	ENSMUSG(
Chrna9	5	65934921.	0,271286	-4,51379	-22,8447	0,002306	0,031373	ENSMUSG(
Gabra2	5	compleme	3,059369	2,765944	6,801928	6,65E-11	7,75E-09	ENSMUSG(
Slain2	5	72914304.	7,698407	-1,08413	-2,12009	1,6E-05	0,000447	ENSMUSG(
Dcun1d4	5	73481000.	18,12954	2,179602	4,530285	6,85E-10	6,32E-08	ENSMUSG(
Sgcb	5	compleme	5,28757	1,722329	3,299687	6,31E-05	0,001471	ENSMUSG(
Usp46	5	compleme	3,135759	1,690022	3,226615	1,97E-06	7,24E-05	ENSMUSG(
Scfd2	5	compleme	17,01458	3,306257	9,891967	0	0	ENSMUSG(
Fip1l1	5	74535449.	83,61896	3,054985	8,310786	0	0	ENSMUSG(
Lnx1	5	compleme	7,696051	3,54453	11,66836	0	0	ENSMUSG(
Chic2	5	compleme	14,17206	3,227715	9,36783	0	0	ENSMUSG(
Pdgfra	5	75152292.	74,14981	4,386815	20,92006	0	0	ENSMUSG(
C530008M	5	76656512.	0,194935	-2,6084	-6,09826	0,000164	0,003356	ENSMUSG(
Srp72	5	76974683.	23,12878	-1,4555	-2,74252	1,13E-12	1,95E-10	ENSMUSG(
Igfbp7	5	compleme	565,7071	2,473555	5,554108	0	0	ENSMUSG(
Adgrl3	5	81020138.	0,486137	1,976283	3,934781	1,55E-06	5,89E-05	ENSMUSG(
Utp3	5	88554462.	10,34968	-1,14373	-2,20951	8,26E-05	0,001857	ENSMUSG(
Adamts3	5	compleme	5,222891	1,168638	2,247993	5,19E-06	0,000168	ENSMUSG(
Ankrd17	5	compleme	16,03866	-1,06108	-2,0865	2,17E-06	7,86E-05	ENSMUSG(
Cxcl3	5	90786103.	4,586891	-7,05508	-132,981	1,3E-05	0,000375	ENSMUSG(
Cxcl1	5	90891241.	71,25137	-2,42712	-5,37817	0	0	ENSMUSG(
Cxcl2	5	90903871.	7,121322	-1,59682	-3,02475	5,37E-07	2,33E-05	ENSMUSG(
Ccni	5	compleme	19,01733	-1,17408	-2,2565	1,57E-07	7,97E-06	ENSMUSG(
Prdm8	5	98167198.	4,407322	-1,05527	-2,07811	5,63E-05	0,001326	ENSMUSG(
Aff1	5	10369237‹	0,413472	-1,5575	-2,94343	0,000789	0,012656	ENSMUSG(
Nudt9	5	10404630€	1,586425	-2,26393	-4,80297	8,91E-06	0,000269	ENSMUSG(
Lrrc8b	5	10541577£	0,605486	-2,33484	-5,04495	4,32E-06	0,000144	ENSMUSG(
Zfp326	5	10587656£	1,72382	-1,77368	-3,41924	1,97E-06	7,25E-05	ENSMUSG(
Gm28050	5	compleme	3,235214	1,571887	2,972932	0,002586	0,034511	ENSMUSG(
Tgfbr3	5	compleme	3,805474	-1,37654	-2,59645	8,13E-08	4,46E-06	ENSMUSG(
Ephx4	5	10740273€	1,613068	5,116295	34,68633	2,61E-11	3,31E-09	ENSMUSG(
Chfr	5	11013584′	1,152537	5,607612	48,75954	1,11E-15	3,75E-13	ENSMUSG(
Pxmp2	5	compleme	8,426731	1,450149	2,732362	2,07E-05	0,000558	ENSMUSG(
Fbrsl1	5	compleme	0,781783	-2,20985	-4,62627	9,69E-06	0,000289	ENSMUSG(
Ulk1	5	compleme	0,430774	-2,45623	-5,4878	1,97E-05	0,000535	ENSMUSG(
Hscb	5	compleme	16,92914	1,326708	2,508297	1,16E-06	4,55E-05	ENSMUSG(
Asphd2	5	compleme	0,629135	-2,38698	-5,23062	0,000125	0,00266	ENSMUSG(
Acacb	5	11414653£	0,547264	-1,65062	-3,1397	6,63E-05	0,001534	ENSMUSG(

Kctd10	5	compleme	3,650821	-2,06197	-4,17557	1,23E-06	4,82E-05	ENSMUSG(
Tchp	5	11470776(	5,172412	-1,29713	-2,45739	5,46E-07	2,36E-05	ENSMUSG(
Rnf10	5	compleme	28,86765	-1,21686	-2,3244	2,45E-09	1,94E-07	ENSMUSG(
Dynll1	5	compleme	33,461	-1,69072	-3,22818	1,88E-14	5,02E-12	ENSMUSG(
Pxn	5	11550667(	4,218598	-2,61418	-6,12273	0	0	ENSMUSG(
Cit	5	11584527{	4,619889	-1,42309	-2,68159	1,26E-09	1,1E-07	ENSMUSG(
Prkab1	5	compleme	3,398904	-1,02603	-2,03641	0,000798	0,012772	ENSMUSG(
Hspb8	5	compleme	19,07842	-1,42651	-2,68796	1,09E-09	9,61E-08	ENSMUSG(
Rnft2	5	compleme	0,574311	-2,12664	-4,36698	0,000756	0,012176	ENSMUSG(
Med13l	5	11856067{	5,103517	-1,24958	-2,37772	9,76E-09	6,83E-07	ENSMUSG(
Tpcn1	5	compleme	5,441746	-2,60029	-6,0641	0	0	ENSMUSG(
Dtx1	5	compleme	0,705141	1,760849	3,388975	0,000304	0,005666	ENSMUSG(
Oas2	5	compleme	0,31932	2,561305	5,902414	1,4E-05	0,0004	ENSMUSG(
Oas1e	5	compleme	0,419536	2,647547	6,266009	0,000137	0,002877	ENSMUSG(
Oas1b	5	12081263{	0,539147	3,274169	9,674376	4,35E-06	0,000145	ENSMUSG(
Oas1h	5	12086142:	0,205441	3,361473	10,2779	0,000327	0,006014	ENSMUSG(
Oas1g	5	compleme	2,205956	3,462387	11,02256	3,59E-11	4,48E-09	ENSMUSG(
Oas1a	5	compleme	2,000783	3,778071	13,7187	1,67E-09	1,4E-07	ENSMUSG(
Ptpn11	5	compleme	21,06741	-1,28263	-2,43283	8,44E-09	5,99E-07	ENSMUSG(
Camkk2	5	compleme	1,372583	-1,15003	-2,21919	0,000474	0,008246	ENSMUSG(
Orai1	5	12301507-	2,533606	-1,14231	-2,20735	0,002426	0,032732	ENSMUSG(
Rhof	5	compleme	0,354871	-2,56055	-5,89931	0,000379	0,006769	ENSMUSG(
Setd1b	5	12314219:	0,256977	-2,14135	-4,41175	0,000391	0,006961	ENSMUSG(
Bcl7a	5	12334383-	0,462285	-2,88289	-7,37624	5,37E-09	3,99E-07	ENSMUSG(
Mlxip	5	12339479{	0,603232	-1,5937	-3,01822	0,000145	0,003024	ENSMUSG(
Vps37b	5	compleme	4,100316	-1,45497	-2,74151	8,81E-06	0,000266	ENSMUSG(
Arl6ip4	5	12411608{	101,6248	1,048288	2,068074	1,04E-07	5,48E-06	ENSMUSG(
Pitpnm2	5	compleme	0,539786	-1,15615	-2,22863	0,001271	0,018867	ENSMUSG(
Ccdc92	5	compleme	0,80339	-2,36857	-5,1643	0,000176	0,003563	ENSMUSG(
Scarb1	5	compleme	3,139728	-1,96335	-3,89966	7,99E-14	1,76E-11	ENSMUSG(
Dhx37	5	compleme	6,590536	-1,17359	-2,25572	2,98E-06	0,000104	ENSMUSG(
Stx2	5	compleme	2,933034	1,043244	2,060857	0,000244	0,004723	ENSMUSG(
Gtf2ird1	5	compleme	2,95645	-1,29877	-2,46018	2,55E-07	1,21E-05	ENSMUSG(
Limk1	5	compleme	5,01384	-1,57702	-2,98353	1,36E-11	1,84E-09	ENSMUSG(
Stx1a	5	13502348:	5,719379	-1,00868	-2,01207	0,000278	0,00525	ENSMUSG(
Pom121	5	compleme	0,865592	-1,60085	-3,03323	0,000886	0,013926	ENSMUSG(
Rhbdd2	5	13563261{	2,56636	-1,35027	-2,54959	8,27E-07	3,39E-05	ENSMUSG(
Tmem120a	5	compleme	3,472593	-1,10608	-2,15261	0,002571	0,03433	ENSMUSG(
Hspb1	5	13588791{	12,58689	-3,63457	-12,4198	0	0	ENSMUSG(
Ywhag	5	compleme	21,12719	-1,62507	-3,08457	2,64E-11	3,35E-09	ENSMUSG(
Dtx2	5	13599480(	0,760873	-1,66044	-3,16112	4,26E-05	0,00104	ENSMUSG(
Serpine1	5	compleme	11,78986	-1,15591	-2,22826	5,92E-08	3,38E-06	ENSMUSG(
Srrt	5	compleme	24,39602	-1,16307	-2,23933	6,57E-07	2,77E-05	ENSMUSG(
Gigyf1	5	13751854{	3,670843	-1,00405	-2,00562	0,000365	0,006573	ENSMUSG(
Pcolce	5	compleme	16,78246	-1,28721	-2,44056	1,68E-08	1,1E-06	ENSMUSG(
Lrch4	5	13762912:	1,061948	-2,24095	-4,7271	1,83E-06	6,8E-05	ENSMUSG(
Taf6	5	compleme	9,020021	-1,48879	-2,80653	7,21E-10	6,62E-08	ENSMUSG(
Cnpy4	5	13818748{	6,810778	1,424569	2,684343	1,49E-06	5,69E-05	ENSMUSG(

Lamtor4	5	138255482	34,75533	1,300811	2,463674	2,02E-08	1,3E-06	ENSMUSG(
Dnaaf5	5	139150223	1,763884	-1,436	-2,7057	0,000179	0,003614	ENSMUSG(
Ints1	5	compleme	9,522205	-1,09722	-2,13943	1,19E-07	6,22E-06	ENSMUSG(
Tmem184a	5	compleme	0,133853	-2,85443	-7,2322	0,003068	0,039898	ENSMUSG(
Chst12	5	140505550	3,962853	-1,23934	-2,36091	9,25E-05	0,002052	ENSMUSG(
Gna12	5	compleme	3,896732	-1,65069	-3,13985	3,23E-06	0,000112	ENSMUSG(
Foxk1	5	142401497	3,064397	-1,86115	-3,63296	3,67E-10	3,6E-08	ENSMUSG(
Tnrc18	5	compleme	2,2953	-1,50107	-2,83053	1,9E-08	1,23E-06	ENSMUSG(
Fbxl18	5	compleme	0,606829	-1,79849	-3,47856	4,1E-06	0,000137	ENSMUSG(
Actb	5	compleme	406,9482	-1,66276	-3,16622	5,84E-11	6,97E-09	ENSMUSG(
Daglb	5	143464584	1,147979	-1,47923	-2,788	6,97E-05	0,001604	ENSMUSG(
Rac1	5	compleme	23,52544	-1,85454	-3,61637	1,17E-14	3,22E-12	ENSMUSG(
Eif2ak1	5	143817788	7,552953	-1,23288	-2,35036	1,45E-07	7,42E-06	ENSMUSG(
Bri3	5	144244437	4,539755	-1,71692	-3,28734	1,83E-05	0,000504	ENSMUSG(
Baiap2l1	5	compleme	4,055526	-1,78597	-3,4485	1,28E-05	0,00037	ENSMUSG(
Trrap	5	144767732	1,915233	-1,35029	-2,54963	6,53E-06	0,000205	ENSMUSG(
Cpsf4	5	145167213	2,738056	-1,20497	-2,30532	0,000107	0,002333	ENSMUSG(
Rpl21	5	146832890	1,444772	1,30177	2,465311	0,000957	0,014892	ENSMUSG(
Gtf3a	5	146948657	42,28224	1,14951	2,218385	4,01E-08	2,41E-06	ENSMUSG(
Slc7a1	5	compleme	3,385346	-1,31938	-2,49559	5,35E-05	0,001266	ENSMUSG(
Hsph1	5	compleme	14,87837	-1,08342	-2,11905	2,24E-06	8,08E-05	ENSMUSG(
N4bp2l2	5	compleme	2,866801	1,111891	2,161288	4,85E-06	0,000159	ENSMUSG(
Gng11	6	4003904..4	94,61452	2,257987	4,783235	0	0	ENSMUSG(
Col1a2	6	4504814..4	30,5424	4,607046	24,3702	4,47E-07	1,98E-05	ENSMUSG(
Casd1	6	4600839..4	5,850415	1,002658	2,003688	6,5E-05	0,001508	ENSMUSG(
2610001J0	6	compleme	5,820356	1,194966	2,289394	4,63E-06	0,000153	ENSMUSG(
Tes	6	17065149.	14,80224	1,113346	2,163468	1,08E-06	4,29E-05	ENSMUSG(
Cav2	6	17281185.	24,05322	1,860215	3,630619	1,21E-14	3,31E-12	ENSMUSG(
Cav1	6	17306335.	187,659	1,129042	2,187134	0,002006	0,027789	ENSMUSG(
Capza2	6	17636234.	44,68506	1,090528	2,12952	1,59E-07	8,07E-06	ENSMUSG(
Cped1	6	21985916.	2,400335	1,462313	2,755497	3,09E-07	1,43E-05	ENSMUSG(
Aass	6	compleme	1,988999	-3,05763	-8,32603	0,000317	0,005866	ENSMUSG(
Rbm28	6	compleme	4,556073	-1,10779	-2,15515	1,41E-05	0,000402	ENSMUSG(
Flnc	6	29433256.	34,77968	-1,05136	-2,07248	4,72E-06	0,000155	ENSMUSG(
Cpa2	6	30541582.	0,253723	-3,5006	-11,3184	0,002472	0,033181	ENSMUSG(
Cpa4	6	30568369.	9,626145	-2,47742	-5,56901	6,93E-08	3,88E-06	ENSMUSG(
Copg2	6	compleme	5,93886	-1,25693	-2,38986	1,71E-07	8,6E-06	ENSMUSG(
Podxl	6	compleme	0,715162	-1,66884	-3,17959	0,000286	0,005364	ENSMUSG(
Akr1b3	6	compleme	6,528968	-2,55795	-5,88871	2,53E-11	3,22E-09	ENSMUSG(
Akr1b7	6	34412334.	4,421992	-1,63013	-3,0954	0,000869	0,013708	ENSMUSG(
Cald1	6	34598500.	11,00937	-1,34772	-2,5451	8,29E-11	9,47E-09	ENSMUSG(
Trim24	6	37870811.	2,791772	-1,15071	-2,22023	2,36E-05	0,000623	ENSMUSG(
Fmc1	6	38533502.	16,79988	1,524268	2,876406	4,03E-06	0,000135	ENSMUSG(
Hipk2	6	compleme	2,119352	-1,74006	-3,34049	6,82E-08	3,82E-06	ENSMUSG(
Kdm7a	6	compleme	0,553412	-1,77888	-3,43159	0,000592	0,009966	ENSMUSG(
Dennd2a	6	compleme	3,82812	-1,29309	-2,45052	2,83E-07	1,32E-05	ENSMUSG(
Casp2	6	42264985.	0,693628	-1,2283	-2,3429	0,002688	0,035583	ENSMUSG(
Zyx	6	42349630.	2,901394	-1,77117	-3,41331	2,37E-07	1,14E-05	ENSMUSG(

Zfp398	6	47835661.	0,714064	-1,06511	-2,09233	0,001398	0,020475	ENSMUSG(
Zfp777	6	compleme	2,436052	-1,27493	-2,41988	0,000183	0,003678	ENSMUSG(
Tra2a	6	compleme	5,167985	-1,0696	-2,09885	0,000169	0,003453	ENSMUSG(
Mpp6	6	50110241.	15,83575	1,026203	2,036657	5,31E-06	0,000172	ENSMUSG(
Dfna5	6	compleme	0,80219	-3,20512	-9,22223	3,27E-08	2E-06	ENSMUSG(
Hnrnpa2b1	6	compleme	91,59406	-2,22714	-4,68205	3,99E-11	4,97E-09	ENSMUSG(
Hoxa1	6	compleme	1,891768	-3,2781	-9,70076	3,62E-08	2,19E-06	ENSMUSG(
Hoxa5	6	compleme	1,692484	-1,41017	-2,65768	0,001767	0,024984	ENSMUSG(
Cpvl	6	compleme	3,371476	-3,87893	-14,7121	0,000143	0,00299	ENSMUSG(
Inmt	6	compleme	1,072065	1,602545	3,036785	0,001645	0,023547	ENSMUSG(
Abcg2	6	58584523.	2,490569	1,586801	3,003825	1,78E-09	1,47E-07	ENSMUSG(
C130060K:	6	65381105.	1,54287	-1,98606	-3,96153	0,000177	0,003585	ENSMUSG(
Ndnf	6	65671590.	0,204927	-3,87024	-14,6238	0,000156	0,00322	ENSMUSG(
Prdm5	6	65778988.	4,299047	1,713239	3,278962	1,66E-08	1,09E-06	ENSMUSG(
Thnsl2	6	compleme	0,948696	3,500538	11,31793	3,46E-07	1,58E-05	ENSMUSG(
Krcc1	6	71271677.	24,97313	1,104155	2,149729	6,17E-07	2,62E-05	ENSMUSG(
Polr1a	6	71909053.	4,34001	-1,54904	-2,92623	4,38E-10	4,23E-08	ENSMUSG(
St3gal5	6	72097592.	7,268192	2,219163	4,656233	3,39E-13	6,59E-11	ENSMUSG(
Tmem150a	6	72355447.	2,383902	-1,64477	-3,12698	0,000256	0,004905	ENSMUSG(
Mat2a	6	compleme	68,53618	-1,35466	-2,55736	8,92E-07	3,62E-05	ENSMUSG(
Tmsb10	6	compleme	3298,535	1,242899	2,366737	3,37E-09	2,59E-07	ENSMUSG(
Hk2	6	compleme	3,781596	-1,20937	-2,31237	0,000321	0,005925	ENSMUSG(
Loxl3	6	83034173.	1,059233	1,768325	3,406583	4,29E-05	0,001044	ENSMUSG(
Dctn1	6	83165920.	38,25083	-1,04475	-2,06301	1,21E-06	4,75E-05	ENSMUSG(
Tet3	6	compleme	1,452811	-1,16753	-2,24626	0,000177	0,003585	ENSMUSG(
Tex261	6	compleme	5,14677	-1,0631	-2,08942	0,000501	0,008645	ENSMUSG(
Cyp26b1	6	compleme	2,353301	-1,38993	-2,62065	0,000118	0,002539	ENSMUSG(
Add2	6	86028681.	3,259156	-3,92894	-15,231	0	0	ENSMUSG(
Tgfa	6	86195223.	0,395855	-1,90072	-3,73399	0,001667	0,023811	ENSMUSG(
Pcbp1	6	compleme	19,55137	-1,31254	-2,48379	4,85E-05	0,001164	ENSMUSG(
Mxd1	6	compleme	0,578722	-1,67716	-3,19797	0,000299	0,005582	ENSMUSG(
Aak1	6	86849517.	0,553741	-1,44615	-2,7248	1,4E-05	0,0004	ENSMUSG(
Isy1	6	compleme	17,15991	1,264724	2,402812	2,11E-10	2,22E-08	ENSMUSG(
Cnbp	6	compleme	477,8935	1,021532	2,030074	2,63E-07	1,24E-05	ENSMUSG(
H1fx	6	compleme	8,27757	-1,0475	-2,06694	0,002598	0,034633	ENSMUSG(
Rab7	6	compleme	11,09011	-1,21348	-2,31897	0,000392	0,006968	ENSMUSG(
Rpn1	6	88084482.	237,7761	1,252366	2,382319	2,87E-10	2,89E-08	ENSMUSG(
Gata2	6	88193891.	1,41897	-1,77076	-3,41235	2,84E-05	0,000731	ENSMUSG(
Eefsec	6	compleme	1,033463	-1,77396	-3,41992	0,001091	0,016682	ENSMUSG(
Ruvbl1	6	88465409.	198,3866	1,034506	2,048412	5,69E-07	2,44E-05	ENSMUSG(
Mcm2	6	compleme	108,8194	1,10351	2,148768	2,77E-07	1,3E-05	ENSMUSG(
Tpra1	6	88902251.	19,71438	1,178642	2,263636	7,1E-08	3,95E-06	ENSMUSG(
Txnrd3	6	89643988.	5,796146	1,396007	2,631721	4,58E-07	2,02E-05	ENSMUSG(
Nup210	6	compleme	6,432546	-1,95648	-3,88114	1,44E-15	4,68E-13	ENSMUSG(
Lsm3	6	91515928.	91,78999	1,061811	2,087551	1,65E-07	8,35E-06	ENSMUSG(
Nr2c2	6	92091390.	1,477917	-1,12442	-2,18014	2,91E-05	0,000747	ENSMUSG(
Prickle2	6	compleme	2,600016	1,509744	2,847595	2,1E-08	1,34E-06	ENSMUSG(
Frmd4b	6	compleme	1,615329	-1,44736	-2,72709	4,34E-08	2,59E-06	ENSMUSG(

Gxylt2	6	10070473	0,692745	-2,2914	-4,8953	2,4E-07	1,15E-05	ENSMUSG(
Cntn3	6	compleme	0,494129	6,259494	76,61175	2,75E-11	3,48E-09	ENSMUSG(
Sumf1	6	compleme	6,292846	1,107539	2,154778	4,07E-05	0,001001	ENSMUSG(
Bhlhe40	6	10866062	5,890065	-1,15648	-2,22913	1,8E-05	0,000495	ENSMUSG(
Edem1	6	10882864	2,769258	-1,38975	-2,62034	3,26E-06	0,000112	ENSMUSG(
Setd5	6	11307736	3,927965	-1,28733	-2,44076	1,46E-08	9,83E-07	ENSMUSG(
Lhfpl4	6	compleme	0,375841	-2,91468	-7,54059	0,000166	0,003402	ENSMUSG(
Brpf1	6	11330713	4,636197	-1,07294	-2,10372	7,75E-06	0,000239	ENSMUSG(
Pparg	6	11536095	4,204625	-1,36234	-2,57102	0,000488	0,008455	ENSMUSG(
Wnt5b	6	compleme	2,866302	1,766516	3,402312	1,23E-05	0,000357	ENSMUSG(
Wnk1	6	compleme	5,181231	-1,0698	-2,09914	1E-04	0,002188	ENSMUSG(
Cecr2	6	12066636	0,121548	-2,93971	-7,67256	0,000292	0,00547	ENSMUSG(
Bid	6	compleme	8,533118	1,227282	2,341256	2,78E-07	1,3E-05	ENSMUSG(
Phc1	6	compleme	2,558501	-1,19072	-2,28266	7,86E-05	0,001779	ENSMUSG(
Foxj2	6	12281991	1,81813	-2,28733	-4,88152	3,74E-08	2,25E-06	ENSMUSG(
Tpi1	6	compleme	648,9304	1,004511	2,006263	1,33E-06	5,13E-05	ENSMUSG(
Ptms	6	compleme	41,73291	-1,59489	-3,02072	5,09E-11	6,14E-09	ENSMUSG(
Mlf2	6	12493138	21,72115	-1,19232	-2,28521	4,24E-09	3,2E-07	ENSMUSG(
Pianp	6	12499669	0,364098	3,633859	12,41368	6,52E-06	0,000205	ENSMUSG(
Zfp384	6	12500914	3,50107	-1,47405	-2,77801	2,17E-08	1,38E-06	ENSMUSG(
Chd4	6	12509598	51,166	-1,01631	-2,02273	1,93E-06	7,11E-05	ENSMUSG(
Nop2	6	12513190	15,03501	-1,29707	-2,4573	2,55E-07	1,21E-05	ENSMUSG(
Ltbr	6	compleme	15,48387	-1,11447	-2,16515	4,38E-06	0,000146	ENSMUSG(
Kcna1	6	compleme	0,38221	-2,84152	-7,16773	0,000266	0,00506	ENSMUSG(
Prmt8	6	compleme	6,301014	1,112697	2,162495	8,03E-06	0,000247	ENSMUSG(
Foxm1	6	12836296	7,190614	-1,48998	-2,80884	1,12E-06	4,41E-05	ENSMUSG(
Gm8837	6	13113094	10,6505	1,018769	2,026189	0,000554	0,009415	ENSMUSG(
Ybx3	6	compleme	50,85786	-1,51242	-2,85288	8,47E-14	1,84E-11	ENSMUSG(
Gm6375	6	compleme	29,83811	-1,30108	-2,46413	4,38E-06	0,000146	ENSMUSG(
Etv6	6	13403570	1,436815	-2,04945	-4,13947	7,24E-08	4,02E-06	ENSMUSG(
Dusp16	6	compleme	2,047689	-1,37525	-2,59413	2,1E-05	0,000564	ENSMUSG(
Gprc5a	6	13506565	4,349825	-1,61136	-3,0554	1,93E-05	0,000526	ENSMUSG(
Pbp2	6	compleme	33,90924	1,048847	2,068876	4,78E-06	0,000157	ENSMUSG(
Atf7ip	6	13650616	2,146809	-1,9906	-3,97402	1,07E-13	2,27E-11	ENSMUSG(
H2afj	6	13680824	547,6904	1,059986	2,084911	2,2E-07	1,08E-05	ENSMUSG(
Wbp11	6	compleme	12,25934	-2,32884	-5,024	0	0	ENSMUSG(
Arhgdib	6	compleme	6,423481	-2,39301	-5,25252	3,85E-05	0,000956	ENSMUSG(
Rerg	6	compleme	1,447507	5,338986	40,47576	7,77E-15	2,27E-12	ENSMUSG(
Eps8	6	compleme	34,45084	1,326648	2,508192	1,69E-08	1,11E-06	ENSMUSG(
Mgst1	6	13814031	30,3179	-1,48009	-2,78967	2,75E-10	2,78E-08	ENSMUSG(
Lmo3	6	compleme	0,294856	-5,25499	-38,1864	0,000556	0,009446	ENSMUSG(
Kcnj8	6	compleme	3,394565	-1,39371	-2,62753	0,002535	0,033919	ENSMUSG(
Abcc9	6	compleme	1,292783	-1,71642	-3,2862	1,97E-05	0,000535	ENSMUSG(
C2cd5	6	compleme	2,499768	-1,08628	-2,12326	0,001164	0,017541	ENSMUSG(
Etfrf1	6	14521113	4,572182	1,506033	2,840279	2,18E-05	0,000582	ENSMUSG(
Bhlhe41	6	compleme	0,244447	-3,57928	-11,9529	0,000164	0,003357	ENSMUSG(
Ppfibp1	6	14688848	6,743009	-1,22801	-2,34243	4,9E-08	2,86E-06	ENSMUSG(
Ergic2	6	compleme	55,50345	1,177078	2,261183	1,33E-08	9,07E-07	ENSMUSG(

Dennd5b	6	compleme	3,806729	-1,06581	-2,09334	1,29E-05	0,000373	ENSMUSG(
Amn1	6	compleme	2,838183	1,018691	2,02608	0,002103	0,028912	ENSMUSG(
Myadm	7	3289080..3	0,928667	-2,85269	-7,22344	2,71E-08	1,69E-06	ENSMUSG(
Cnot3	7	3645268..3	2,487548	-1,45101	-2,73398	0,002597	0,03463	ENSMUSG(
Mboat7	7	compleme	2,767364	-1,18396	-2,272	1,81E-06	6,72E-05	ENSMUSG(
Leng8	7	4137039..4	1,273751	-1,10282	-2,14774	0,001744	0,024715	ENSMUSG(
Eps8l1	7	4460674..4	0,11231	3,147396	8,860549	0,000116	0,002506	ENSMUSG(
Tnni3	7	compleme	0,229339	2,294982	4,907477	0,002446	0,032899	ENSMUSG(
Ppp6r1	7	compleme	7,235202	-1,29372	-2,45159	1,94E-05	0,000527	ENSMUSG(
Brsk1	7	4690604..4	0,828682	-4,78588	-27,5863	6,98E-07	2,92E-05	ENSMUSG(
Il11	7	compleme	5,078545	-2,35254	-5,10722	1,24E-06	4,83E-05	ENSMUSG(
Rpl28	7	4792874..4	29,62215	-2,66665	-6,34954	2,39E-10	2,47E-08	ENSMUSG(
Isoc2b	7	compleme	13,91728	1,567503	2,963914	4,58E-10	4,4E-08	ENSMUSG(
Isoc2a	7	4877153..4	16,54122	1,457366	2,746066	7,51E-11	8,63E-09	ENSMUSG(
Ssc5d	7	4925785..4	0,095804	3,079051	8,450584	0,00036	0,006513	ENSMUSG(
Epn1	7	5080235..5	10,96723	-1,48956	-2,80804	1,05E-06	4,16E-05	ENSMUSG(
Rasl2-9	7	compleme	243,3074	1,067977	2,096492	2,34E-07	1,13E-05	ENSMUSG(
Zfp551	7	compleme	4,870419	1,239293	2,360828	4,55E-05	0,0011	ENSMUSG(
Zfp606	7	12478293.	1,396916	1,175871	2,259292	0,000348	0,006317	ENSMUSG(
Zfp110	7	12834761.	0,730067	-2,36313	-5,14486	4,6E-05	0,00111	ENSMUSG(
Rps5	7	12922290.	654,7297	1,213202	2,318517	1,28E-09	1,11E-07	ENSMUSG(
Zfp324	7	12965838.	1,371676	1,151999	2,222216	0,001709	0,024308	ENSMUSG(
Gltscr2	7	compleme	20,66083	-1,01034	-2,01439	5,68E-06	0,000183	ENSMUSG(
Zc3h4	7	16400910.	4,132398	-2,18327	-4,54182	4,2E-11	5,16E-09	ENSMUSG(
Arhgap35	7	compleme	10,67038	-1,37324	-2,59053	5,08E-08	2,95E-06	ENSMUSG(
Prkd2	7	16842902.	3,959542	-1,26235	-2,39886	1,92E-06	7,09E-05	ENSMUSG(
Calm3	7	compleme	104,2887	-1,0837	-2,11947	7,73E-08	4,26E-06	ENSMUSG(
Gm26821	7	compleme	0,098035	3,339227	10,12063	0,001762	0,024933	ENSMUSG(
Mypop	7	18991245.	0,926909	-1,89153	-3,71027	0,002396	0,032395	ENSMUSG(
Sympk	7	19024377.	5,392876	-1,44958	-2,73128	8,43E-11	9,6E-09	ENSMUSG(
Dmwd	7	19076227.	4,94741	-1,29975	-2,46186	2,19E-05	0,000583	ENSMUSG(
Fbxo46	7	19119859.	0,912319	-2,07606	-4,21655	4,83E-06	0,000158	ENSMUSG(
Qpctl	7	compleme	3,181635	-1,5425	-2,91299	2,66E-06	9,39E-05	ENSMUSG(
Fosb	7	compleme	0,527614	-2,31029	-4,95984	0,002877	0,037701	ENSMUSG(
Ercc2	7	19382010.	5,712307	-1,46783	-2,76605	7,72E-09	5,54E-07	ENSMUSG(
Clasrp	7	compleme	3,921972	-1,3554	-2,55867	1,59E-08	1,06E-06	ENSMUSG(
Relb	7	compleme	2,560268	-1,16167	-2,23716	0,000163	0,003346	ENSMUSG(
Clptm1	7	compleme	8,389416	-1,70821	-3,26756	2,13E-11	2,76E-09	ENSMUSG(
Apoe	7	compleme	0,614161	-2,97743	-7,87584	0,000846	0,013409	ENSMUSG(
Bcl3	7	compleme	0,855977	-2,18536	-4,54839	0,000322	0,005933	ENSMUSG(
Smg9	7	24399619.	11,05211	-1,11519	-2,16623	1,64E-06	6,17E-05	ENSMUSG(
Cadm4	7	24482023.	1,41362	-2,99038	-7,94683	9,04E-05	0,002013	ENSMUSG(
Xrcc1	7	24546289.	9,318427	-1,49327	-2,81525	1,12E-11	1,55E-09	ENSMUSG(
Lypd3	7	24636550.	0,578554	-4,71523	-26,2678	0,001094	0,016719	ENSMUSG(
Gm4881	7	compleme	5,805869	-1,53984	-2,90763	7,99E-05	0,001804	ENSMUSG(
Atp1a3	7	compleme	0,714622	-1,37464	-2,59303	0,003823	0,048102	ENSMUSG(
Zfp574	7	25072567.	6,022124	-1,65939	-3,15883	2,7E-10	2,75E-08	ENSMUSG(
Zfp526	7	25221425.	4,127812	-1,35642	-2,56049	1,37E-06	5,28E-05	ENSMUSG(

Gsk3a	7	compleme	7,025969	-2,70282	-6,51071	0	0	ENSMUSG(
Erf	7	compleme	26,78718	-1,25568	-2,38779	1,98E-09	1,62E-07	ENSMUSG(
Cic	7	25267704.	3,225653	-2,17794	-4,52507	1,11E-16	4,39E-14	ENSMUSG(
Megf8	7	25317164.	7,006773	-1,69819	-3,24493	2,42E-14	6,25E-12	ENSMUSG(
Atp5sl	7	25619414.	2,722462	-1,65606	-3,15155	9,01E-06	0,000272	ENSMUSG(
Tgfb1	7	25687002.	3,723618	-2,28526	-4,87453	2,07E-09	1,68E-07	ENSMUSG(
Hnrnpul1	7	compleme	68,83358	-2,00255	-4,00709	0	0	ENSMUSG(
Axl	7	compleme	77,07376	-1,60271	-3,03713	2,62E-10	2,67E-08	ENSMUSG(
Rab4b	7	compleme	4,070391	-1,48455	-2,7983	3,32E-05	0,000839	ENSMUSG(
Snrpa	7	compleme	17,34285	-1,10997	-2,15842	2,15E-06	7,83E-05	ENSMUSG(
Itpkc	7	compleme	1,747099	-1,08496	-2,12131	0,003467	0,044346	ENSMUSG(
Gm15567	7	compleme	0,153707	2,339829	5,062426	0,003128	0,040565	ENSMUSG(
Numbl	7	27258433.	0,903716	-1,62572	-3,08596	4,03E-05	0,000992	ENSMUSG(
Ltbp4	7	compleme	2,458635	-1,44271	-2,7183	3,42E-06	0,000117	ENSMUSG(
Shkbp1	7	compleme	13,84108	-1,66665	-3,17477	7,15E-13	1,28E-10	ENSMUSG(
Pld3	7	compleme	17,82594	-1,20483	-2,30511	2E-06	7,34E-05	ENSMUSG(
2310022A1	7	27553233.	3,286552	-1,37699	-2,59725	1,69E-06	6,32E-05	ENSMUSG(
Akt2	7	27591552.	16,71935	-1,0594	-2,08407	1,49E-07	7,59E-06	ENSMUSG(
Map3k10	7	compleme	4,402073	-2,41342	-5,32735	3,03E-14	7,63E-12	ENSMUSG(
Fbl	7	28169710.	67,83918	-1,39839	-2,63608	2,75E-09	2,16E-07	ENSMUSG(
Supt5	7	compleme	26,81696	-1,96097	-3,89324	0	0	ENSMUSG(
Rps16	7	28350652.	313,8265	-2,86114	-7,26587	0	0	ENSMUSG(
Plekhg2	7	compleme	0,541811	-2,1386	-4,40333	4,28E-06	0,000143	ENSMUSG(
Zfp36	7	compleme	0,627778	-2,53272	-5,78662	8,66E-05	0,001933	ENSMUSG(
Paf1	7	28392951.	39,05762	-1,957	-3,88254	0	0	ENSMUSG(
Samd4b	7	compleme	14,3488	-2,1067	-4,30705	0	0	ENSMUSG(
Lrfn1	7	28451980.	0,382216	-3,54298	-11,6559	5,99E-05	0,001404	ENSMUSG(
Pak4	7	compleme	5,643977	-2,76671	-6,80553	0	0	ENSMUSG(
Rinl	7	28788969.	0,319393	3,649868	12,55219	1,72E-05	0,000477	ENSMUSG(
Ech1	7	28825217.	8,812726	-2,65287	-6,28919	1,11E-16	4,39E-14	ENSMUSG(
Actn4	7	compleme	112,1482	-1,62892	-3,0928	2,9E-14	7,33E-12	ENSMUSG(
Sipa1l3	7	compleme	2,764997	-2,04145	-4,1166	7,83E-14	1,74E-11	ENSMUSG(
Zfp568	7	29983955.	0,162715	1,863553	3,639027	0,001162	0,017527	ENSMUSG(
Capns1	7	compleme	91,69084	-1,43492	-2,70368	2,52E-12	3,95E-10	ENSMUSG(
Wdr62	7	compleme	4,721472	-1,79176	-3,46237	4,18E-11	5,15E-09	ENSMUSG(
Lrfn3	7	compleme	0,968798	-2,3353	-5,04656	3,69E-05	0,00092	ENSMUSG(
Aplp1	7	compleme	3,596944	-2,64189	-6,24149	5E-09	3,74E-07	ENSMUSG(
Lin37	7	compleme	5,745478	-1,20417	-2,30405	0,000607	0,010187	ENSMUSG(
Psenen	7	compleme	12,87437	-1,33758	-2,52726	1,68E-05	0,000469	ENSMUSG(
Kmt2b	7	compleme	1,110269	-2,39407	-5,2564	2,97E-09	2,32E-07	ENSMUSG(
Rbm42	7	compleme	31,54862	-1,37939	-2,60159	3,7E-10	3,62E-08	ENSMUSG(
Sbsn	7	30751471.	0,430822	-3,49244	-11,2545	0,000162	0,00333	ENSMUSG(
Usf2-ps1	7	compleme	7,318544	-1,86995	-3,65521	0,000991	0,015334	ENSMUSG(
Usf2	7	compleme	26,93227	-1,28895	-2,44351	1,33E-09	1,14E-07	ENSMUSG(
Fxyd5	7	compleme	0,849879	-1,74493	-3,35179	0,000172	0,003496	ENSMUSG(
Gramd1a	7	compleme	4,839169	-1,97533	-3,93218	2,64E-13	5,29E-11	ENSMUSG(
Gm12760	7	compleme	261,2973	1,298631	2,459953	2,83E-09	2,22E-07	ENSMUSG(
Gpi1	7	compleme	48,50449	-1,21971	-2,329	4,29E-09	3,23E-07	ENSMUSG(

Lsm14a	7	compleme	24,95448	-1,77729	-3,42782	2,22E-16	8,45E-14	ENSMUSG(
Pepd	7	34912379.	10,5497	-1,32325	-2,50229	1,47E-08	9,89E-07	ENSMUSG(
Gpatch1	7	compleme	6,170404	-1,93119	-3,8137	7,23E-13	1,29E-10	ENSMUSG(
Rhpn2	7	35334170.	12,837	-2,84982	-7,20911	1,16E-09	1,02E-07	ENSMUSG(
Cep89	7	35397035.	1,816648	-1,34925	-2,5478	0,000688	0,011287	ENSMUSG(
Nudt19	7	compleme	2,065771	-2,12575	-4,36431	5,81E-07	2,49E-05	ENSMUSG(
Siglecf	7	43351341.	0,133911	3,911106	15,04389	0,001268	0,018828	ENSMUSG(
Clec11a	7	compleme	16,55461	1,607651	3,047553	3,89E-12	5,87E-10	ENSMUSG(
Myh14	7	compleme	0,73925	-2,54988	-5,85587	1,64E-06	6,17E-05	ENSMUSG(
Nup62	7	44816088.	8,318994	-1,0067	-2,00931	0,000102	0,002225	ENSMUSG(
Akt1s1	7	44848991.	3,019766	-1,47077	-2,7717	0,000233	0,004547	ENSMUSG(
Prmt1	7	compleme	180,6739	1,106046	2,152549	5,88E-08	3,36E-06	ENSMUSG(
Scaf1	7	compleme	3,470092	-1,72585	-3,30774	5,05E-09	3,76E-07	ENSMUSG(
Prr12	7	compleme	0,512977	-1,42251	-2,68052	0,001998	0,027716	ENSMUSG(
Nosip	7	45062429.	31,77316	1,343311	2,53733	4,05E-11	5,01E-09	ENSMUSG(
Rcn3	7	compleme	76,95383	1,114651	2,165426	8,24E-08	4,51E-06	ENSMUSG(
Fcgrt	7	compleme	1,406682	2,736723	6,665544	6,08E-07	2,59E-05	ENSMUSG(
Rps11	7	compleme	3755,016	1,040861	2,057455	1,47E-07	7,54E-06	ENSMUSG(
Rpl13a	7	compleme	1480,466	1,366421	2,578301	5,96E-12	8,56E-10	ENSMUSG(
Pih1d1	7	45154303.	30,80587	1,078733	2,11218	3,24E-06	0,000112	ENSMUSG(
Ruvbl2	7	compleme	180,1959	1,257712	2,391161	1,32E-09	1,14E-07	ENSMUSG(
Car11	7	45699843.	2,538979	-2,28109	-4,86043	0,002992	0,038989	ENSMUSG(
Cyth2	7	compleme	5,753809	-1,37999	-2,60266	9,63E-05	0,002124	ENSMUSG(
E2f8	7	compleme	1,067929	-1,22412	-2,33612	0,001608	0,023131	ENSMUSG(
Htatip2	7	49759115.	6,117284	6,945386	123,2451	0	0	ENSMUSG(
Gm6290	7	compleme	9,800377	-1,52211	-2,8721	9,23E-06	0,000278	ENSMUSG(
Gm17838	7	compleme	0,77686	-3,3221	-10,0012	0,003986	0,049752	ENSMUSG(
Nipa1	7	compleme	1,12118	-1,68179	-3,20825	0,000225	0,004409	ENSMUSG(
Mcee	7	64392607.	7,289784	1,086818	2,12405	0,002197	0,030066	ENSMUSG(
Lrrk1	7	compleme	2,516492	-1,57792	-2,98539	4,45E-07	1,97E-05	ENSMUSG(
Synm	7	compleme	1,751371	-1,62598	-3,08651	1,33E-06	5,15E-05	ENSMUSG(
Gm18805	7	compleme	9,535201	1,169529	2,249383	0,000537	0,009167	ENSMUSG(
Slco3a1	7	compleme	0,676868	-2,8104	-7,01479	1,38E-08	9,39E-07	ENSMUSG(
Akap13	7	75455534.	4,147237	-1,25934	-2,39386	3,32E-09	2,56E-07	ENSMUSG(
Anpep	7	compleme	28,30078	-1,42865	-2,69195	2,02E-09	1,64E-07	ENSMUSG(
Zfp710	7	80024814.	2,751318	-1,19013	-2,28173	4,73E-06	0,000155	ENSMUSG(
Sema4b	7	80186841.	1,184679	-1,18608	-2,27533	0,00342	0,043876	ENSMUSG(
Man2a2	7	compleme	2,790983	-1,5338	-2,89548	2,85E-09	2,23E-07	ENSMUSG(
Furin	7	compleme	5,973485	-1,85484	-3,61711	8,88E-16	3,04E-13	ENSMUSG(
Crtc3	7	compleme	1,118872	-1,08247	-2,11766	0,000677	0,011151	ENSMUSG(
Iqgap1	7	compleme	26,88363	-1,06438	-2,09127	2,76E-07	1,3E-05	ENSMUSG(
Nmb	7	compleme	1,024055	2,814016	7,032396	0,000247	0,004767	ENSMUSG(
Zfp592	7	80993681.	2,703187	-1,99875	-3,99653	1,27E-10	1,42E-08	ENSMUSG(
Cpeb1	7	compleme	4,094454	-2,23425	-4,70518	1,97E-11	2,57E-09	ENSMUSG(
Whamm	7	81571266.	2,639981	1,034909	2,048985	0,002232	0,03046	ENSMUSG(
Sh3gl3	7	82173840.	0,611719	-2,86005	-7,26042	0,000192	0,00383	ENSMUSG(
Mex3b	7	82867333.	0,80843	-2,85135	-7,21674	1,66E-05	0,000464	ENSMUSG(
Mesdc1	7	compleme	1,515041	-1,83865	-3,57676	2,7E-06	9,52E-05	ENSMUSG(

Cemip	7	compleme	0,518895	-2,79715	-6,95066	0,001617	0,023226	ENSMUSG(
Tmem135	7	compleme	4,12413	1,311976	2,482813	2,42E-07	1,16E-05	ENSMUSG(
Prss23	7	compleme	98,66199	1,171729	2,252816	8,81E-09	6,24E-07	ENSMUSG(
Hikeshi	7	compleme	18,63924	1,510229	2,848553	5,4E-12	7,82E-10	ENSMUSG(
Eed	7	compleme	8,837105	1,035787	2,050232	7,67E-06	0,000237	ENSMUSG(
Sytl2	7	90302252.	18,75723	4,27609	19,37454	0	0	ENSMUSG(
Tmem126a	7	compleme	9,486199	1,440923	2,714944	2,05E-05	0,000554	ENSMUSG(
Rab30	7	92741603.	2,194043	1,443876	2,720507	7,28E-08	4,03E-06	ENSMUSG(
Nars2	7	96951505.	8,011484	1,035018	2,049139	5,65E-06	0,000182	ENSMUSG(
Clns1a	7	97696634.	119,7021	1,439704	2,712652	3,74E-13	7,16E-11	ENSMUSG(
Dgat2	7	compleme	6,048399	-1,14719	-2,21483	0,002688	0,035583	ENSMUSG(
Serpinh1	7	compleme	266,9274	1,313544	2,485514	2,15E-10	2,24E-08	ENSMUSG(
Gdpd5	7	99381414.	0,491713	-1,93879	-3,83385	0,000617	0,010338	ENSMUSG(
Arrb1	7	99535466.	0,851086	-2,05539	-4,15657	4,39E-08	2,61E-06	ENSMUSG(
Spcs2	7	compleme	4,345067	-1,11817	-2,17071	0,001999	0,027716	ENSMUSG(
Coa4	7	10053707:	20,95836	1,147358	2,215078	5,57E-07	2,39E-05	ENSMUSG(
Mrpl48	7	compleme	2,279919	1,276004	2,421673	8,41E-06	0,000256	ENSMUSG(
Clpb	7	10166363:	9,698279	1,19999	2,297381	3,21E-09	2,48E-07	ENSMUSG(
Lrrc51	7	compleme	4,355217	1,303279	2,467891	0,001116	0,016965	ENSMUSG(
Xndc1	7	10206551:	0,451241	1,462006	2,754912	0,003459	0,04426	ENSMUSG(
Rhog	7	compleme	5,581062	-3,15853	-8,92919	9,23E-11	1,04E-08	ENSMUSG(
Stim1	7	10226780(	2,228524	-1,39628	-2,63221	3,86E-07	1,74E-05	ENSMUSG(
Prkcdbp	7	compleme	16,30381	1,806045	3,496823	4,89E-10	4,64E-08	ENSMUSG(
Hpx	7	compleme	0,211128	4,440253	21,70947	0,001624	0,023322	ENSMUSG(
Gm8982	7	10612317:	11,73117	1,329521	2,513192	0,001362	0,019987	ENSMUSG(
Cyb5r2	7	compleme	8,84961	-1,02478	-2,03465	0,000284	0,005342	ENSMUSG(
Gm26599	7	compleme	0,077904	4,106991	17,23167	0,001611	0,023166	ENSMUSG(
St5	7	compleme	4,348728	-1,99366	-3,98246	5,28E-14	1,24E-11	ENSMUSG(
Rnf141	7	compleme	6,495981	1,116096	2,167596	1,39E-06	5,35E-05	ENSMUSG(
Ctr9	7	11102895:	7,984613	-1,21106	-2,31507	4,32E-05	0,001052	ENSMUSG(
1700012D:	7	compleme	4,837548	-1,55041	-2,929	3,76E-05	0,000934	ENSMUSG(
Galnt18	7	compleme	16,8207	1,623896	3,082063	2,74E-09	2,16E-07	ENSMUSG(
Mical2	7	11222585(	3,574857	-1,20053	-2,29823	3,16E-05	0,000801	ENSMUSG(
Rras2	7	compleme	30,22695	1,1855	2,274423	6,92E-09	5,03E-07	ENSMUSG(
Gm4353	7	compleme	19,71652	1,040549	2,05701	2,81E-05	0,000726	ENSMUSG(
Rps15a	7	compleme	104,9868	1,590527	3,011594	1,98E-14	5,26E-12	ENSMUSG(
Coq7	7	compleme	5,986695	1,072157	2,102575	1,26E-05	0,000363	ENSMUSG(
Gprc5b	7	compleme	0,951944	-1,89219	-3,71198	7,43E-05	0,001697	ENSMUSG(
Mettl9	7	12103444!	132,3627	1,020247	2,028266	2,25E-06	8,11E-05	ENSMUSG(
Cog7	7	compleme	2,670208	-1,0749	-2,10657	0,000888	0,013944	ENSMUSG(
Gga2	7	compleme	3,907964	-1,29527	-2,45423	4,57E-06	0,000151	ENSMUSG(
Ndufab1	7	compleme	12,73923	1,12296	2,177934	5,38E-08	3,09E-06	ENSMUSG(
Plk1	7	12215943!	49,38338	-1,75638	-3,37848	7,94E-14	1,75E-11	ENSMUSG(
Tnrc6a	7	12312388!	7,957133	-1,05279	-2,07453	2,64E-05	0,000689	ENSMUSG(
Atxn2l	7	compleme	4,7215	-1,16472	-2,2419	0,000688	0,011287	ENSMUSG(
Eif3c	7	compleme	155,9602	-1,39865	-2,63654	3,26E-07	1,49E-05	ENSMUSG(
Bola2	7	12669540:	138,506	1,306372	2,473188	2,14E-10	2,23E-08	ENSMUSG(
Gm42742	7	compleme	15,11439	1,099106	2,142219	2,17E-06	7,86E-05	ENSMUSG(

Dctpp1	7	compleme	13,95081	-1,0641	-2,09086	0,003993	0,049824	ENSMUSG(
Sephs2	7	compleme	3,557963	-1,55179	-2,93181	0,000668	0,011026	ENSMUSG(
E430018J2	7	compleme	0,721283	1,464037	2,758792	0,003061	0,039825	ENSMUSG(
Zfp688	7	compleme	1,977287	1,292041	2,448743	0,000723	0,011756	ENSMUSG(
Fbrs	7	12747919‹	2,137129	-1,69458	-3,23684	1,36E-06	5,23E-05	ENSMUSG(
Bcl7c	7	compleme	12,58314	1,298963	2,460519	5,09E-10	4,8E-08	ENSMUSG(
Setd1a	7	12777667(	1,076702	-1,91977	-3,78364	9,08E-06	0,000274	ENSMUSG(
Zfp646	7	12787622:	2,243227	-1,10113	-2,14523	0,000127	0,002703	ENSMUSG(
Bag3	7	12852361€	10,47336	-1,68387	-3,21288	1,35E-10	1,5E-08	ENSMUSG(
Plpp4	7	12925703:	1,20485	-5,60515	-48,6763	0,0002	0,003957	ENSMUSG(
Tacc2	7	13057743ε	0,636965	-1,7557	-3,37689	1,8E-06	6,7E-05	ENSMUSG(
Htra1	7	13093611:	27,34312	1,665659	3,172585	1,81E-06	6,74E-05	ENSMUSG(
Dmbt1	7	13103205:	1,104535	-2,19888	-4,59122	2,54E-06	9,04E-05	ENSMUSG(
Ikzf5	7	compleme	0,849445	-1,66046	-3,16117	0,00044	0,007705	ENSMUSG(
Oat	7	compleme	101,7767	1,107167	2,154222	7,64E-07	3,16E-05	ENSMUSG(
Mgmt	7	13689461‹	2,280785	1,30959	2,47871	9,95E-05	0,002181	ENSMUSG(
Mapk1ip1	7	compleme	0,769391	-2,83604	-7,14055	0,000448	0,007843	ENSMUSG(
Bnip3	7	compleme	28,04642	1,050347	2,071027	0,000166	0,003404	ENSMUSG(
Fuom	7	compleme	1,949015	1,273213	2,416992	0,000167	0,00341	ENSMUSG(
Rnh1	7	compleme	38,67821	1,349919	2,548979	1,69E-10	1,83E-08	ENSMUSG(
Irf7	7	compleme	0,254442	4,406825	21,21224	4,37E-06	0,000145	ENSMUSG(
Cd151	7	14146739:	50,15402	1,004372	2,00607	9,53E-07	3,83E-05	ENSMUSG(
Tspan4	7	14147524(	64,39108	1,74431	3,350345	0	0	ENSMUSG(
Mob2	7	compleme	3,973691	1,095418	2,136749	2,16E-05	0,000578	ENSMUSG(
Mrpl23	7	14253268€	39,63695	1,150112	2,219312	2,21E-08	1,41E-06	ENSMUSG(
H19	7	compleme	0,128284	2,583614	5,994395	0,001878	0,026295	ENSMUSG(
Tssc4	7	14306924‹	2,501433	1,360422	2,567603	0,000702	0,011476	ENSMUSG(
Phlda2	7	compleme	9,658752	-2,05144	-4,14521	5,18E-06	0,000168	ENSMUSG(
Oraov1	7	14491510(	0,788662	1,489713	2,808331	0,002062	0,02843	ENSMUSG(
Mrgprf	7	14530082ε	0,474361	3,672283	12,74874	3,2E-05	0,000811	ENSMUSG(
Insr	8	compleme	4,786764	-1,13767	-2,20025	2,67E-06	9,42E-05	ENSMUSG(
Zfp358	8	3493138..:	5,058135	-1,64932	-3,13686	7,08E-08	3,94E-06	ENSMUSG(
Mcoln1	8	3500457..:	0,938325	-2,47365	-5,55445	1,63E-06	6,14E-05	ENSMUSG(
Stxbp2	8	3630955..:	0,673964	-3,32281	-10,0061	1,21E-05	0,000353	ENSMUSG(
Trappc5	8	3676299..:	22,39526	1,023493	2,032835	3,44E-07	1,57E-05	ENSMUSG(
Evi5l	8	4166567..4	2,191594	-1,69956	-3,24801	8,31E-08	4,53E-06	ENSMUSG(
Map2k7_1	8	4238740..4	7,760705	-1,27193	-2,41484	5,55E-07	2,39E-05	ENSMUSG(
Cers4	8	4493026..4	0,569927	1,446997	2,7264	3,84E-05	0,000952	ENSMUSG(
Irs2	8	compleme	0,727195	-1,51179	-2,85163	0,001063	0,016304	ENSMUSG(
Ankrd10	8	compleme	4,209029	1,0084	2,011679	9,82E-06	0,000293	ENSMUSG(
Pcid2	8	compleme	30,20043	1,578002	2,985561	1,21E-13	2,55E-11	ENSMUSG(
Ank1	8	22974844.	1,635938	-2,16584	-4,48729	1,55E-12	2,55E-10	ENSMUSG(
Sfrp1	8	23411502.	17,858	-1,1552	-2,22715	8,94E-08	4,83E-06	ENSMUSG(
1810011O	8	compleme	1,273832	-3,27512	-9,68076	0,000887	0,013936	ENSMUSG(
Letm2	8	compleme	0,594466	-1,60202	-3,03569	0,00076	0,012244	ENSMUSG(
Ash2l	8	compleme	2,421067	-1,0884	-2,12637	0,001778	0,025102	ENSMUSG(
Adgra2	8	27085583.	2,17503	-1,28909	-2,44374	0,002944	0,038463	ENSMUSG(
Nrg1	8	compleme	1,845258	-2,52483	-5,75507	0	0	ENSMUSG(

Ubxn8	8	compleme	11,47141	1,272271	2,415415	2,19E-07	1,07E-05	ENSMUSG(
Gtf2e2	8	33731833.	45,24077	1,006759	2,009391	2,91E-06	0,000102	ENSMUSG(
Prag1	8	36094828.	0,259648	-2,21442	-4,64095	0,000175	0,003558	ENSMUSG(
Dlc1	8	compleme	4,838402	-2,09787	-4,28077	0	0	ENSMUSG(
Pdgfrl	8	40926212.	0,942454	-1,96802	-3,9123	0,0011	0,016774	ENSMUSG(
Gm6284	8	43158754.	0,572418	-1,81422	-3,5167	0,001673	0,023873	ENSMUSG(
Ankrd37	8	compleme	0,880962	1,946334	3,853941	0,000988	0,0153	ENSMUSG(
Slc25a4	8	compleme	74,84756	-1,50218	-2,8327	1,31E-09	1,13E-07	ENSMUSG(
Stox2	8	compleme	0,37769	-2,50326	-5,66964	2,38E-06	8,54E-05	ENSMUSG(
Tenm3	8	compleme	2,818691	-1,57563	-2,98065	9,91E-10	8,86E-08	ENSMUSG(
Neil3	8	compleme	23,10133	1,866677	3,646917	3,18E-14	7,95E-12	ENSMUSG(
Wdr17	8	compleme	0,780533	-2,36518	-5,15216	3,32E-06	0,000114	ENSMUSG(
Mfap3l	8	60632827.	0,220283	-3,22398	-9,34361	7,77E-05	0,001764	ENSMUSG(
Sh3rf1	8	61223872.	1,88532	-1,1008	-2,14473	0,000135	0,002859	ENSMUSG(
Palld	8	compleme	9,286475	1,302192	2,466032	1,71E-09	1,42E-07	ENSMUSG(
Msmo1	8	compleme	4,233095	1,145531	2,212275	4,12E-06	0,000138	ENSMUSG(
Csgalnact1	8	compleme	1,257306	-4,81228	-28,0958	2,98E-09	2,32E-07	ENSMUSG(
Atp6v1b2	8	69088646.	12,74747	-1,23905	-2,36044	5,05E-08	2,94E-06	ENSMUSG(
Atp13a1	8	69791163.	17,13653	-1,20045	-2,29811	1,81E-07	9,07E-06	ENSMUSG(
Gmip	8	69808679.	0,950327	-2,05376	-4,15186	1,53E-06	5,81E-05	ENSMUSG(
Gatad2a	8	compleme	19,27679	-1,668	-3,17773	1,16E-13	2,45E-11	ENSMUSG(
Crtc1	8	compleme	0,33725	-1,81624	-3,52163	5,84E-05	0,001372	ENSMUSG(
Jund	8	70698949.	8,149358	-1,6469	-3,1316	4,22E-06	0,000141	ENSMUSG(
Pik3r2	8	compleme	3,579197	-1,76071	-3,38865	1,02E-09	9,07E-08	ENSMUSG(
Mast3	8	compleme	0,338715	-1,9316	-3,81477	0,001159	0,017485	ENSMUSG(
Ccdc124	8	compleme	19,75006	-2,937	-7,6582	9,76E-14	2,11E-11	ENSMUSG(
Map1s	8	70905932.	0,993075	-2,2623	-4,79756	4,08E-07	1,82E-05	ENSMUSG(
Colgalt1	8	71610998.	27,60769	-1,42804	-2,69081	3,25E-11	4,07E-09	ENSMUSG(
Unc13a	8	compleme	0,734145	-2,56116	-5,90182	1E-08	7,01E-07	ENSMUSG(
Fcho1	8	compleme	0,292831	-4,31002	-19,8356	0,000196	0,003884	ENSMUSG(
Rab8a	8	72161200.	23,43452	-1,22607	-2,33929	6,65E-10	6,15E-08	ENSMUSG(
Ap1m1	8	72240018.	17,03944	-1,01	-2,01391	5,55E-06	0,000179	ENSMUSG(
Eps15l1	8	compleme	1,789369	-2,34233	-5,07123	1,66E-08	1,09E-06	ENSMUSG(
Cherp	8	compleme	0,960279	-2,8366	-7,14334	1,44E-06	5,49E-05	ENSMUSG(
Slc35e1	8	compleme	4,865633	-1,34435	-2,53916	1,2E-07	6,26E-06	ENSMUSG(
Med26	8	compleme	1,742341	-2,45618	-5,48762	3,59E-07	1,64E-05	ENSMUSG(
Sin3b	8	72723285.	2,914518	-1,38155	-2,60548	2,12E-07	1,04E-05	ENSMUSG(
Nr3c2	8	76899442.	0,176421	-3,14815	-8,86517	0,000451	0,007877	ENSMUSG(
Zfp827	8	79028437.	0,430109	-1,67884	-3,20171	0,000203	0,004005	ENSMUSG(
Otud4	8	79639618.	4,681669	-1,62597	-3,08649	4,45E-08	2,65E-06	ENSMUSG(
Hhip	8	compleme	0,493966	-3,91855	-15,1217	3,24E-07	1,49E-05	ENSMUSG(
Gab1	8	compleme	0,584944	-1,98572	-3,96061	0,000131	0,00278	ENSMUSG(
Il15	8	compleme	0,443778	1,972304	3,923943	0,002833	0,037298	ENSMUSG(
Tecr	8	compleme	35,83544	-1,95727	-3,88326	0	0	ENSMUSG(
Dnajb1	8	83608193.	39,37014	-1,65349	-3,14594	0	0	ENSMUSG(
Pkn1	8	compleme	17,7949	-1,28508	-2,43696	2,38E-09	1,89E-07	ENSMUSG(
Adgre5	8	compleme	1,891975	-1,13735	-2,19977	0,001896	0,026503	ENSMUSG(
Adgrl1	8	83900105.	3,071666	-1,88738	-3,69964	2,52E-09	1,99E-07	ENSMUSG(

Rfx1	8	84066834.	0,450752	-1,63372	-3,10313	0,00263	0,034934	ENSMUSG(
Dcaf15	8	compleme	10,6323	-1,20463	-2,30478	1,43E-06	5,49E-05	ENSMUSG(
Cc2d1a	8	compleme	3,495875	-1,49183	-2,81246	1,31E-06	5,06E-05	ENSMUSG(
Zswim4	8	compleme	1,751346	-1,12969	-2,18812	0,001889	0,02643	ENSMUSG(
Nacc1	8	compleme	13,33065	-1,38102	-2,60453	1,06E-09	9,39E-08	ENSMUSG(
Gadd45gip	8	84831522.	41,23085	-1,22588	-2,33899	6,57E-08	3,71E-06	ENSMUSG(
Rad23a	8	compleme	140,2355	-1,42172	-2,67904	7,85E-12	1,11E-09	ENSMUSG(
Mast1	8	compleme	0,306077	-1,96985	-3,91727	0,00134	0,019715	ENSMUSG(
Tnpo2	8	85036915.	16,58084	-1,50645	-2,8411	4,61E-10	4,42E-08	ENSMUSG(
Orc6	8	85299632.	16,34004	1,00237	2,003289	6,28E-05	0,001465	ENSMUSG(
4921524J1	8	compleme	13,10713	1,272173	2,41525	5,23E-07	2,28E-05	ENSMUSG(
Dnaja2	8	compleme	161,103	1,36669	2,578782	5,44E-11	6,54E-09	ENSMUSG(
Nkd1	8	88521354.	0,540545	2,45063	5,466548	1,41E-05	0,000402	ENSMUSG(
Sall1	8	compleme	1,681084	-3,41532	-10,6688	3,64E-14	8,86E-12	ENSMUSG(
Ces1d	8	compleme	1,600241	-5,37861	-41,6027	1,23E-05	0,000357	ENSMUSG(
Gnao1	8	93809966.	0,292816	-1,60629	-3,04467	0,001542	0,022267	ENSMUSG(
Bbs2	8	compleme	81,6393	1,107378	2,154537	2,56E-06	9,09E-05	ENSMUSG(
Mt1	8	94179082.	213,4825	1,610182	3,052903	5E-15	1,47E-12	ENSMUSG(
Nlrc5	8	94434356.	1,067956	-1,36653	-2,57849	0,001412	0,020633	ENSMUSG(
Cpne2	8	94532990.	28,84707	-1,07217	-2,10259	1,72E-05	0,000478	ENSMUSG(
Adgrg1	8	94974751.	3,571198	-5,1877	-36,4462	0	0	ENSMUSG(
Zfp319	8	compleme	1,404218	-1,83633	-3,571	1,89E-07	9,41E-06	ENSMUSG(
Mmp15	8	95352268.	5,333245	-1,33747	-2,52707	0,000141	0,002967	ENSMUSG(
Csnk2a2	8	compleme	7,147951	-1,20555	-2,30625	7,2E-06	0,000224	ENSMUSG(
Cnot1	8	compleme	32,30845	-1,05045	-2,07118	1,27E-05	0,000367	ENSMUSG(
Cmtm4	8	compleme	0,213481	-2,03818	-4,10728	0,001354	0,01989	ENSMUSG(
Ces2d-ps	8	104867488	0,231431	3,195473	9,160797	0,000698	0,011416	ENSMUSG(
Gm8798	8	104886862	0,74881	-1,47594	-2,78165	0,003121	0,04049	ENSMUSG(
D230025D	8	105225145	2,566467	-1,15781	-2,23119	0,000397	0,00704	ENSMUSG(
Lrrc36	8	105413571	1,247016	-2,02196	-4,06136	0,002593	0,034588	ENSMUSG(
Carmil2	8	105686274	1,424375	-3,56202	-11,8107	1,24E-11	1,71E-09	ENSMUSG(
Acd	8	compleme	1,313044	-1,32082	-2,49809	0,001988	0,027641	ENSMUSG(
Ranbp10	8	compleme	0,80678	-1,72551	-3,30697	0,000109	0,00237	ENSMUSG(
Edc4	8	105880881	11,02003	-1,12738	-2,18461	1,53E-05	0,000431	ENSMUSG(
Psmb10	8	compleme	6,110429	1,010644	2,01481	0,000839	0,013307	ENSMUSG(
Nfatc3	8	106058840	4,051592	-1,79225	-3,46354	1,89E-11	2,48E-09	ENSMUSG(
Slc7a6	8	106168857	5,129019	-1,41128	-2,65974	1,46E-05	0,000414	ENSMUSG(
Gm10073	8	compleme	345,6943	-2,51837	-5,72935	0	0	ENSMUSG(
Chtf8	8	compleme	8,951143	-1,30897	-2,47764	1,04E-06	4,13E-05	ENSMUSG(
Utp4	8	106893636	10,4485	-1,08903	-2,12731	4,38E-05	0,001063	ENSMUSG(
Terf2	8	compleme	3,177972	-1,55848	-2,94543	7,96E-07	3,28E-05	ENSMUSG(
Nfat5	8	107293470	1,396266	-1,0777	-2,11066	0,000177	0,003577	ENSMUSG(
Nob1	8	compleme	7,635316	-2,31627	-4,98041	2,66E-15	8,07E-13	ENSMUSG(
Wwp2	8	107436365	3,032105	-1,72045	-3,29538	2,17E-09	1,75E-07	ENSMUSG(
Dhx38	8	compleme	20,89433	-1,21082	-2,31469	2,14E-07	1,05E-05	ENSMUSG(
Atxn1l	8	compleme	3,219036	-1,84546	-3,59368	1,06E-10	1,2E-08	ENSMUSG(
Ap1g1	8	109778554	11,47659	-1,31898	-2,4949	9,02E-09	6,37E-07	ENSMUSG(
Phlpp2	8	109868542	1,326749	-1,00038	-2,00053	0,002913	0,038118	ENSMUSG(

Vac14	8	110618585	1,823713	-1,80245	-3,48812	3,73E-08	2,25E-06	ENSMUSG(
Mtss1l	8	110721476	3,112662	-2,52347	-5,74963	2,01E-12	3,23E-10	ENSMUSG(
Aars	8	111033144	28,09173	-1,40651	-2,65096	5,08E-11	6,14E-09	ENSMUSG(
Pdpr	8	111094630	5,200785	-1,08984	-2,1285	2,55E-05	0,000668	ENSMUSG(
Wdr59	8	compleme	2,432749	-1,66882	-3,17955	8,32E-08	4,53E-06	ENSMUSG(
Tmem231	8	compleme	1,182481	-1,7677	-3,40511	0,002386	0,032293	ENSMUSG(
Gm6793	8	compleme	1,282607	-2,10316	-4,2965	0,000878	0,013826	ENSMUSG(
Mon1b	8	113635587	1,037483	-1,69211	-3,2313	9,26E-05	0,002053	ENSMUSG(
Hsd17b2	8	117701904	6,967089	5,129563	35,0068	1,11E-16	4,39E-14	ENSMUSG(
Cotl1	8	compleme	29,38549	-1,08802	-2,12582	3,99E-05	0,000984	ENSMUSG(
Usp10	8	119910360	10,86552	-1,35797	-2,56324	4,73E-09	3,54E-07	ENSMUSG(
Crispld2	8	119992438	0,148525	-3,33885	-10,118	0,00375	0,047367	ENSMUSG(
Mthfsd	8	compleme	1,215686	2,051941	4,146634	1,7E-07	8,55E-06	ENSMUSG(
Map1lc3b	8	121590361	0,336222	-2,4462	-5,44977	6,19E-05	0,001448	ENSMUSG(
Zcchc14	8	compleme	0,807702	-1,51423	-2,85647	0,000244	0,00472	ENSMUSG(
Jph3	8	121729623	1,170918	-1,9493	-3,86186	0,001749	0,024772	ENSMUSG(
Slc7a5	8	compleme	48,74567	-1,01156	-2,01609	0,000111	0,002409	ENSMUSG(
Zc3h18	8	122376609	3,418003	-2,2605	-4,79156	4,44E-16	1,61E-13	ENSMUSG(
Cdt1	8	122568015	3,575314	-1,10938	-2,15753	0,000739	0,011953	ENSMUSG(
Galns	8	compleme	3,859123	-1,51365	-2,85531	2,34E-06	8,4E-05	ENSMUSG(
Ankrd11	8	compleme	1,713691	-1,97879	-3,94162	2,59E-10	2,66E-08	ENSMUSG(
Chmp1a	8	compleme	5,364016	-1,23768	-2,35818	1,5E-05	0,000424	ENSMUSG(
Vps9d1	8	compleme	0,945948	-1,51761	-2,86316	0,000765	0,012303	ENSMUSG(
Fanca	8	compleme	0,984713	-1,0911	-2,13037	0,002215	0,030264	ENSMUSG(
Mc1r	8	123407107	5,992335	2,085143	4,243172	2,97E-13	5,87E-11	ENSMUSG(
Tubb3	8	123411424	309,6352	1,09872	2,141646	4,95E-05	0,001185	ENSMUSG(
Def8	8	123423527	39,67322	2,762695	6,786628	0	0	ENSMUSG(
Afg3l1	8	123477903	0,965296	2,033735	4,094635	3,07E-05	0,000782	ENSMUSG(
Gas8	8	123518834	12,73656	2,174371	4,51389	8,46E-11	9,62E-09	ENSMUSG(
Acta1	8	compleme	88,48704	-1,09258	-2,13255	3,3E-06	0,000114	ENSMUSG(
Galnt2	8	124231391	22,47585	-1,39933	-2,63779	7,62E-11	8,72E-09	ENSMUSG(
Pgbd5	8	compleme	0,485941	-3,19059	-9,12986	0,000154	0,003186	ENSMUSG(
Exoc8	8	compleme	3,471491	-1,17188	-2,25305	1,39E-05	0,000396	ENSMUSG(
Nrp1	8	128358604	8,651989	-1,17621	-2,25982	2,84E-08	1,76E-06	ENSMUSG(
Gucy1a2	9	3532778..3	2,159752	-1,29096	-2,44691	0,000507	0,008716	ENSMUSG(
Aasdhppt	9	compleme	22,23635	1,232647	2,349977	3,87E-08	2,32E-06	ENSMUSG(
Gria4	9	compleme	0,281217	3,069591	8,395351	1,12E-07	5,89E-06	ENSMUSG(
Casp4	9	5308828..5	8,809305	1,208586	2,31111	9,73E-08	5,19E-06	ENSMUSG(
Pdgfd	9	6168584..6	1,356773	1,939119	3,834715	4,5E-05	0,001089	ENSMUSG(
Mmp1b	9	compleme	1,684842	3,618942	12,28599	1,41E-08	9,55E-07	ENSMUSG(
Mmp3	9	7445822..7	27,72028	3,686948	12,87899	2,52E-07	1,2E-05	ENSMUSG(
Mmp1a	9	7464141..7	5,312314	3,685225	12,86362	1,3E-12	2,19E-10	ENSMUSG(
Mmp10	9	7502352..7	21,06333	3,717037	13,15042	2,12E-07	1,04E-05	ENSMUSG(
Mmp8	9	7558456..7	2,954585	3,51554	11,43623	3,13E-12	4,83E-10	ENSMUSG(
Birc2	9	compleme	24,70525	1,086997	2,124314	2,56E-06	9,09E-05	ENSMUSG(
Arhgap42	9	compleme	0,464643	-1,37677	-2,59687	0,003923	0,049121	ENSMUSG(
Maml2	9	13297957.	0,646357	-1,30888	-2,4775	0,001754	0,024835	ENSMUSG(
Cwc15	9	14500617.	91,70542	1,471602	2,773298	2,31E-14	6,02E-12	ENSMUSG(

Ankrd49	9	compleme	5,642358	1,284453	2,435896	2,05E-07	1,02E-05	ENSMUSG(
Dnmt1	9	compleme	22,53575	-1,19721	-2,29296	2,16E-07	1,06E-05	ENSMUSG(
Mrpl4	9	21002738.	12,5292	-1,17722	-2,2614	1E-05	0,000298	ENSMUSG(
Icam1	9	21015985.	0,781249	-1,9286	-3,80687	0,001355	0,019898	ENSMUSG(
RP23-186(	9	compleme	3,110547	-2,00573	-4,01593	2,18E-09	1,75E-07	ENSMUSG(
Raver1	9	compleme	0,313112	-3,97749	-15,7523	0,000331	0,006074	ENSMUSG(
Kri1	9	compleme	9,410397	-1,21486	-2,32117	9,04E-07	3,66E-05	ENSMUSG(
Slc44a2	9	21320698.	0,393689	-2,28998	-4,89049	0,000441	0,007716	ENSMUSG(
Ilf3	9	21367871.	29,17484	-1,91802	-3,77904	2,55E-10	2,63E-08	ENSMUSG(
Tmed1	9	compleme	29,89983	1,085241	2,12173	9,94E-06	0,000296	ENSMUSG(
Carm1	9	21546894.	10,2861	-1,77656	-3,42609	1,92E-13	3,93E-11	ENSMUSG(
Smarca4	9	21616169.	19,86007	-1,4332	-2,70045	1,94E-11	2,53E-09	ENSMUSG(
Dock6	9	compleme	1,63897	-1,38699	-2,61533	1,87E-05	0,000511	ENSMUSG(
Plppr2	9	21937033.	6,15816	-1,58656	-3,00333	2,66E-08	1,66E-06	ENSMUSG(
Prkcsh	9	22002806.	72,47054	-1,64951	-3,13727	3,82E-13	7,27E-11	ENSMUSG(
Cnn1	9	22099216.	0,397634	-2,72021	-6,58971	0,002129	0,029256	ENSMUSG(
Bmper	9	23223076.	0,284915	2,259634	4,788701	0,000194	0,003854	ENSMUSG(
Tbx20	9	compleme	0,288022	-2,15966	-4,46808	2,84E-05	0,000732	ENSMUSG(
RP23-118I:	9	25349498.	6,202786	4,281082	19,44169	4,22E-15	1,25E-12	ENSMUSG(
Vps26b	9	compleme	5,621359	1,039769	2,055899	9,54E-05	0,002105	ENSMUSG(
Jam3	9	compleme	2,3999	3,400894	10,5626	5,16E-07	2,25E-05	ENSMUSG(
Igsf9b	9	27299204.	0,181168	2,220628	4,660964	0,000115	0,002474	ENSMUSG(
Opcml	9	27790775.	0,252771	3,334796	10,08959	2,12E-09	1,71E-07	ENSMUSG(
Ntm	9	compleme	2,607948	3,673294	12,75768	0	0	ENSMUSG(
Adamts8	9	30942562.	0,775274	4,377126	20,78003	3,1E-10	3,1E-08	ENSMUSG(
Zbtb44	9	31030644.	5,206015	2,034073	4,095595	1,26E-11	1,73E-09	ENSMUSG(
Tmem45b	9	compleme	0,583894	1,793447	3,46642	0,003262	0,0421	ENSMUSG(
Fli1	9	compleme	9,779131	-1,48998	-2,80885	1,74E-07	8,74E-06	ENSMUSG(
St3gal4	9	compleme	9,686215	1,670294	3,182794	2,14E-08	1,37E-06	ENSMUSG(
Foxred1	9	compleme	5,3023	1,471488	2,773077	2,33E-07	1,13E-05	ENSMUSG(
Fam118b	9	compleme	2,636815	1,548373	2,924871	3,81E-07	1,72E-05	ENSMUSG(
Cdon	9	35421128.	2,254538	1,975004	3,931294	2,78E-07	1,3E-05	ENSMUSG(
Ddx25	9	compleme	0,12054	2,592313	6,030649	0,000551	0,009364	ENSMUSG(
Pus3	9	35558595.	3,171948	1,213779	2,319444	0,001994	0,027705	ENSMUSG(
Hyls1	9	compleme	4,143227	1,405395	2,648903	8,36E-06	0,000255	ENSMUSG(
Chek1	9	compleme	13,85099	1,728584	3,314024	3,25E-12	4,98E-10	ENSMUSG(
Tmem218	9	37208223.	3,84413	1,618999	3,071618	5,49E-06	0,000177	ENSMUSG(
RP23-80D2	9	39074102.	16,87719	-1,24614	-2,37207	5,63E-05	0,001326	ENSMUSG(
AW551984	9	compleme	0,944582	-1,36506	-2,57587	0,00097	0,015078	ENSMUSG(
Gramd1b	9	compleme	0,624008	-2,41394	-5,32929	0,000198	0,00393	ENSMUSG(
2610203C:	9	41327260.	2,032539	2,205477	4,612271	1,55E-15	4,95E-13	ENSMUSG(
Mcam	9	44134469.	0,829832	-2,56455	-5,91571	0,000272	0,005155	ENSMUSG(
Dpagt1	9	44326019.	6,463805	-1,02305	-2,03222	0,000158	0,003265	ENSMUSG(
Trappc4	9	compleme	32,78432	1,470761	2,77168	1,63E-11	2,18E-09	ENSMUSG(
Rps25	9	44407139.	38,45809	1,046622	2,065687	9,63E-08	5,15E-06	ENSMUSG(
Bcl9l	9	44482825.	1,134969	-1,30576	-2,47214	0,001485	0,021554	ENSMUSG(
Tmem25	9	compleme	0,077147	2,988682	7,937485	0,003145	0,040758	ENSMUSG(
Kmt2a	9	compleme	2,526193	-1,02675	-2,03744	0,00128	0,018961	ENSMUSG(

Mpzl3	9	45055186.	0,635246	1,49083	2,810506	0,000177	0,003583	ENSMUSG(
Tagln	9	compleme	2,094823	-2,1581	-4,46328	0,002067	0,028489	ENSMUSG(
Pafah1b2	9	compleme	36,1849	1,165304	2,242805	1,61E-08	1,06E-06	ENSMUSG(
Rexo2	9	compleme	44,36129	1,188023	2,278403	5,31E-09	3,95E-07	ENSMUSG(
Nnmt	9	compleme	52,63372	1,421678	2,678968	4,81E-06	0,000158	ENSMUSG(
Ttc12	9	compleme	2,157367	1,281307	2,430591	0,000259	0,004947	ENSMUSG(
Ncam1	9	compleme	4,226268	-2,01133	-4,03153	3,33E-16	1,24E-13	ENSMUSG(
Pts	9	compleme	11,78037	1,777545	3,428423	3,31E-14	8,25E-12	ENSMUSG(
Sdhd	9	compleme	74,33542	1,481866	2,793097	1,27E-12	2,15E-10	ENSMUSG(
Timm8b	9	50603910.	117,4991	1,74989	3,36333	2,22E-15	6,85E-13	ENSMUSG(
Fdx1	9	compleme	5,761864	1,272386	2,415608	7,2E-07	3E-05	ENSMUSG(
Atm	9	compleme	7,321933	1,111619	2,160879	9,86E-07	3,94E-05	ENSMUSG(
Acat1	9	compleme	60,52863	1,41736	2,670963	5,39E-12	7,82E-10	ENSMUSG(
Rab39	9	compleme	1,073512	-4,51245	-22,8235	1,97E-06	7,25E-05	ENSMUSG(
Slc35f2	9	53771538.	17,37248	1,034584	2,048524	5,93E-06	0,000189	ENSMUSG(
Wdr61	9	compleme	20,3813	1,043949	2,061864	2,4E-07	1,15E-05	ENSMUSG(
Etfa	9	compleme	156,684	1,279297	2,427207	1,4E-09	1,2E-07	ENSMUSG(
Peak1	9	compleme	1,291087	-2,07406	-4,21071	1,19E-09	1,04E-07	ENSMUSG(
Cspg4	9	56865033.	10,8313	-1,07469	-2,10627	1,39E-07	7,16E-06	ENSMUSG(
Snx33	9	compleme	4,304358	-1,18163	-2,26833	8,83E-06	0,000267	ENSMUSG(
Imp3	9	56937475.	37,96012	1,091976	2,131658	1,12E-05	0,000328	ENSMUSG(
Sin3a	9	57072040.	10,26996	-1,08255	-2,11778	8,08E-06	0,000248	ENSMUSG(
1700017B(	9	compleme	0,591211	-2,61385	-6,12133	5,13E-06	0,000167	ENSMUSG(
Scamp5	9	compleme	0,485396	-2,2754	-4,84131	0,00056	0,0095	ENSMUSG(
RP23-371E	9	57483810.	21,57757	1,258808	2,392979	3,3E-06	0,000114	ENSMUSG(
Cox5a	9	57521274.	22,08431	-1,26682	-2,40631	3,07E-08	1,9E-06	ENSMUSG(
Scamp2	9	57560943.	11,50049	-1,5474	-2,9229	1,64E-08	1,08E-06	ENSMUSG(
Csk	9	compleme	9,764261	-1,23444	-2,3529	1,44E-06	5,49E-05	ENSMUSG(
Edc3	9	57708540.	3,053912	-1,16879	-2,24823	2,55E-05	0,000668	ENSMUSG(
Sema7a	9	57940112.	7,756566	-2,95661	-7,76296	0	0	ENSMUSG(
Ccdc33	9	compleme	0,253546	-3,7833	-13,7685	0,000828	0,013157	ENSMUSG(
Stra6	9	58063788.	2,292977	-7,70784	-209,069	1,97E-09	1,61E-07	ENSMUSG(
Stoml1	9	58253164.	3,989557	1,015417	2,021487	0,000334	0,006113	ENSMUSG(
6030419C	9	58488603.	3,41743	1,060319	2,085393	0,002219	0,030319	ENSMUSG(
Hcn4	9	58823412.	0,126881	-4,40949	-21,2514	0,002353	0,031901	ENSMUSG(
Thsd4	9	compleme	1,248889	-1,11027	-2,15886	7,89E-05	0,001785	ENSMUSG(
Tle3	9	61372366.	3,983705	-1,5932	-3,01717	4,73E-11	5,74E-09	ENSMUSG(
Rplp1	9	compleme	38,58226	-1,22039	-2,33009	2,16E-05	0,000578	ENSMUSG(
Anp32a	9	62341293.	63,13221	-1,40896	-2,65545	5,93E-11	7,05E-09	ENSMUSG(
Coro2b	9	compleme	1,376961	-1,42863	-2,6919	0,000177	0,003577	ENSMUSG(
Cln6	9	62838785.	5,922118	-1,46844	-2,76722	2,44E-06	8,73E-05	ENSMUSG(
Gm10653	9	compleme	58,42343	-1,34237	-2,53568	8,75E-07	3,55E-05	ENSMUSG(
Map2k5	9	compleme	2,602011	-1,3923	-2,62498	8,38E-05	0,001881	ENSMUSG(
Smad3	9	compleme	8,181816	-1,50281	-2,83393	2,44E-09	1,94E-07	ENSMUSG(
Oaz2	9	65668001.	12,49927	-1,47409	-2,77809	9,29E-09	6,53E-07	ENSMUSG(
Snx1	9	compleme	8,296663	-1,20156	-2,29987	0,000301	0,005624	ENSMUSG(
Gcnt3	9	compleme	0,540237	-5,0751	-33,7099	1,06E-05	0,000314	ENSMUSG(
Fam81a	9	compleme	0,269887	-4,13733	-17,5978	0,000293	0,005479	ENSMUSG(

Myo1e	9	70207350.	8,161445	-2,34253	-5,07192	3,61E-14	8,82E-12	ENSMUSG(
Tmod2	9	compleme	0,257455	-4,48487	-22,3914	6,35E-06	0,000201	ENSMUSG(
Fam83b	9	compleme	0,197767	1,9303	3,811345	0,00245	0,032949	ENSMUSG(
Eef1a1	9	compleme	9546,025	1,072132	2,102538	8,79E-08	4,76E-06	ENSMUSG(
Filip1	9	compleme	0,266507	-4,37347	-20,7274	0,000254	0,004879	ENSMUSG(
Myo6	9	80165031.	1,851681	-1,09065	-2,1297	9,28E-05	0,002055	ENSMUSG(
Sh3bgrl2	9	83548327.	0,26344	-2,12045	-4,3483	0,001247	0,018577	ENSMUSG(
Elovl4	9	compleme	0,790917	-3,61913	-12,2876	6,47E-06	0,000204	ENSMUSG(
Me1	9	compleme	7,242743	-1,16216	-2,23793	2,8E-06	9,86E-05	ENSMUSG(
Tbx18	9	compleme	4,340996	-1,10603	-2,15253	0,000126	0,002689	ENSMUSG(
Nt5e	9	88327197.	51,70447	1,702531	3,254714	5,4E-14	1,26E-11	ENSMUSG(
Gm10634	9	88719629.	0,192534	2,836098	7,140862	0,001167	0,017571	ENSMUSG(
Bcl2a1a	9	88956900.	2,663312	-2,2383	-4,71841	0,002876	0,037698	ENSMUSG(
Ctsh	9	90054152.	0,347958	2,376214	5,191726	7,04E-05	0,00162	ENSMUSG(
Tbc1d2b	9	compleme	2,093796	-1,15569	-2,22791	3,98E-06	0,000134	ENSMUSG(
Plscr1	9	92249750.	6,760845	1,609666	3,051811	1,42E-07	7,28E-06	ENSMUSG(
Plscr2	9	92275602.	1,279258	1,430409	2,695231	0,001292	0,019115	ENSMUSG(
1700057G(	9	92309377.	6,260817	1,406335	2,650629	1,05E-05	0,000309	ENSMUSG(
Plscr4	9	92457373.	14,86899	2,062974	4,178467	1,81E-10	1,94E-08	ENSMUSG(
Chst2	9	compleme	0,523328	-2,18708	-4,55384	4,87E-06	0,000159	ENSMUSG(
Atp1b3	9	compleme	12,16971	-1,20701	-2,30859	2,62E-06	9,26E-05	ENSMUSG(
Faim	9	98986373.	18,66008	1,05403	2,076322	6,6E-06	0,000207	ENSMUSG(
Faiml	9	compleme	13,37889	1,046076	2,064905	0,000466	0,008121	ENSMUSG(
Nck1	9	compleme	28,82876	1,308067	2,476096	1,16E-10	1,3E-08	ENSMUSG(
Slc35g2	9	compleme	34,24288	1,050988	2,071949	8,69E-07	3,54E-05	ENSMUSG(
Stag1	9	10059779ε	17,27031	1,138545	2,201588	1,15E-06	4,54E-05	ENSMUSG(
Pccb	9	compleme	28,11647	1,049551	2,069886	1,32E-06	5,12E-05	ENSMUSG(
Ryk	9	10283491	74,62164	1,168388	2,247603	2,52E-08	1,58E-06	ENSMUSG(
Srprb	9	compleme	30,55255	1,334308	2,521544	6,86E-11	7,96E-09	ENSMUSG(
Mrpl3	9	10505323	41,79281	1,006836	2,009499	1,14E-06	4,49E-05	ENSMUSG(
Nek11	9	compleme	1,859348	1,263626	2,400985	0,000287	0,00539	ENSMUSG(
Alas1	9	compleme	13,102	-1,05153	-2,07272	3,49E-06	0,000119	ENSMUSG(
Dusp7	9	10636863	3,382689	-1,06047	-2,08562	0,000714	0,01163	ENSMUSG(
Rad54l2	9	compleme	3,075617	-1,1403	-2,20427	3,11E-06	0,000108	ENSMUSG(
Vprbp	9	10682187	6,823411	-1,08272	-2,11803	9,31E-06	0,00028	ENSMUSG(
Dock3	9	compleme	0,386842	-3,0959	-8,54986	8,23E-07	3,38E-05	ENSMUSG(
Uba7	9	10797550	1,957515	2,300678	4,926891	5,6E-11	6,72E-09	ENSMUSG(
Ip6k1	9	10800250	3,13983	-1,02975	-2,04168	4,84E-05	0,001163	ENSMUSG(
Dag1	9	compleme	18,37659	-1,63594	-3,1079	1,11E-16	4,39E-14	ENSMUSG(
Qrich1	9	10851680	12,08751	-1,31214	-2,4831	1,15E-10	1,29E-08	ENSMUSG(
Wdr6	9	compleme	14,12185	-1,21096	-2,31491	9,64E-08	5,15E-06	ENSMUSG(
Slc25a20	9	10866208	5,63971	1,07527	2,107117	0,000431	0,007575	ENSMUSG(
Nckipsd	9	10880836	1,934378	-1,06163	-2,08728	0,001819	0,025591	ENSMUSG(
Celsr3	9	10882632	0,460395	-1,1389	-2,20214	0,001113	0,016932	ENSMUSG(
Map4	9	10993146	3,831146	-1,75687	-3,37965	4,63E-14	1,11E-11	ENSMUSG(
Smarcc1	9	11011770	17,22264	-1,33293	-2,51913	1,46E-10	1,6E-08	ENSMUSG(
Nbeal2	9	compleme	1,029451	-1,84599	-3,595	3,35E-05	0,000844	ENSMUSG(
Cmtm7	9	compleme	1,180943	-1,82242	-3,53673	3,5E-05	0,000879	ENSMUSG(

Osbpl10	9	114978569	1,435305	-1,11582	-2,16719	0,001817	0,02558	ENSMUSG(
Tgfbr2	9	compleme	8,412403	-1,49906	-2,82658	5,46E-12	7,89E-10	ENSMUSG(
Acvr2b	9	119402118	1,085295	-1,51129	-2,85065	0,001194	0,017912	ENSMUSG(
Myrip	9	120301513	0,276066	-3,26425	-9,60809	8,11E-05	0,001827	ENSMUSG(
Trak1	9	121297502	1,394812	-1,6712	-3,1848	3,61E-09	2,76E-07	ENSMUSG(
Clec3b	9	123150946	0,911157	2,28784	4,883245	0,001342	0,019729	ENSMUSG(
Akap12	10	4266380..4	5,597271	-3,36709	-10,318	0	0	ENSMUSG(
Lats1	10	7681214..7	2,964406	-1,50384	-2,83597	2,65E-07	1,24E-05	ENSMUSG(
Sash1	10	compleme	4,166795	-1,40154	-2,64183	4,76E-07	2,09E-05	ENSMUSG(
Samd5	10	compleme	0,583297	2,093449	4,267672	0,000875	0,013784	ENSMUSG(
Rab32	10	compleme	19,85712	1,222973	2,334272	2,23E-07	1,09E-05	ENSMUSG(
B230208H	10	compleme	1,25423	-3,43556	-10,8195	5,56E-05	0,001313	ENSMUSG(
Hivep2	10	13966075.	2,711118	-1,10596	-2,15241	4,29E-06	0,000143	ENSMUSG(
Nhsl1	10	18318985.	1,61652	-1,1071	-2,15412	8,42E-05	0,00189	ENSMUSG(
Hebp2	10	compleme	15,38027	1,129399	2,187675	1,9E-06	7,03E-05	ENSMUSG(
Arfgef3	10	compleme	0,818059	-2,26258	-4,79849	2,17E-09	1,75E-07	ENSMUSG(
Perp	10	18845020.	104,9356	1,369048	2,583001	1,78E-11	2,36E-09	ENSMUSG(
Tnfaip3	10	compleme	2,513016	-1,82549	-3,54428	1,22E-11	1,68E-09	ENSMUSG(
Map3k5	10	19934472.	14,07911	1,148462	2,216775	2,3E-08	1,46E-06	ENSMUSG(
Map7	10	20148471.	0,550779	-2,24226	-4,73137	0,000421	0,007415	ENSMUSG(
Myb	10	compleme	0,224026	-2,93751	-7,66088	0,000857	0,013566	ENSMUSG(
Tcf21	10	compleme	30,99745	1,001973	2,002737	0,001493	0,021635	ENSMUSG(
Epb41l2	10	25359798.	7,557622	-1,8767	-3,67234	2,24E-07	1,09E-05	ENSMUSG(
RP23-138N	10	26626547.	2,360588	3,362555	10,28561	0,000561	0,009521	ENSMUSG(
Arhgap18	10	26753421.	29,39814	2,019862	4,055449	0	0	ENSMUSG(
Rspo3	10	compleme	0,599364	5,712571	52,4391	1,28E-09	1,11E-07	ENSMUSG(
Trmt11	10	compleme	8,975611	1,116417	2,168079	3,18E-06	0,00011	ENSMUSG(
Gm5422	10	31248023.	56,97704	-1,20122	-2,29934	8,83E-08	4,78E-06	ENSMUSG(
Col10a1	10	34389981.	0,298829	2,28185	4,86301	0,003766	0,047548	ENSMUSG(
Frk	10	34483399.	0,315604	1,60915	3,050721	0,001709	0,024308	ENSMUSG(
Fyn	10	39368855.	12,6133	-2,06897	-4,19588	2,22E-16	8,45E-14	ENSMUSG(
Traf3ip2	10	39612934.	7,491337	-1,27157	-2,41424	2,24E-05	0,000596	ENSMUSG(
Cdk19	10	40339564.	1,588553	-1,51097	-2,85001	1,8E-05	0,000496	ENSMUSG(
Wasf1	10	40883475.	3,947965	-1,74929	-3,36193	2,27E-07	1,1E-05	ENSMUSG(
Sobp	10	compleme	1,323036	2,012297	4,034242	2,2E-06	7,97E-05	ENSMUSG(
Bend3	10	43478831.	0,415581	-1,44935	-2,73084	0,002848	0,037411	ENSMUSG(
Cd24a	10	43578284.	5,405778	-5,0342	-32,7676	3,62E-08	2,19E-06	ENSMUSG(
Prdm1	10	compleme	0,914902	-2,22138	-4,66339	1,88E-06	6,98E-05	ENSMUSG(
Popdc3	10	45178098.	5,363814	-2,37875	-5,20087	5,44E-07	2,36E-05	ENSMUSG(
Asf1a	10	53596757.	12,36185	1,012813	2,017842	1,67E-05	0,000466	ENSMUSG(
Fam184a	10	compleme	0,786804	-2,04837	-4,13639	0,001463	0,021262	ENSMUSG(
Sh3rf3	10	58813359.	1,035666	-1,86133	-3,63342	6,11E-07	2,6E-05	ENSMUSG(
P4ha1	10	59323296.	28,03313	1,026909	2,037654	4,92E-05	0,00118	ENSMUSG(
Sgpl1	10	compleme	2,805295	-1,55619	-2,94077	5,12E-08	2,98E-06	ENSMUSG(
Hnrnph3	10	compleme	9,310677	-1,37	-2,5847	7,38E-06	0,000229	ENSMUSG(
Mypn	10	compleme	5,762262	-1,33447	-2,52182	3,56E-06	0,000121	ENSMUSG(
Herc4	10	63243810.	23,64789	1,606347	3,044798	1,56E-12	2,55E-10	ENSMUSG(
Prmt2	10	compleme	11,47906	1,041181	2,057912	9,62E-06	0,000288	ENSMUSG(

Ybey	10	compleme	2,365667	1,478529	2,786646	5,04E-06	0,000164	ENSMUSG(
Pcbp3	10	compleme	1,795813	-3,39398	-10,5121	6,37E-09	4,66E-07	ENSMUSG(
Sumo3_1	10	77606097.	11,16651	1,374761	2,59325	1,33E-07	6,9E-06	ENSMUSG(
1810043G(	10	77978524.	0,550508	1,46448	2,759639	0,001849	0,025958	ENSMUSG(
Cstb	10	78425669.	9,814443	-2,83653	-7,14299	1,34E-09	1,15E-07	ENSMUSG(
Hcn2	10	79716634.	0,90823	-2,95923	-7,77708	7,93E-05	0,001791	ENSMUSG(
Polrmt	10	compleme	2,239598	-2,1663	-4,48872	1,71E-09	1,42E-07	ENSMUSG(
Rnf126	10	compleme	10,38879	-1,05159	-2,07281	6,21E-05	0,00145	ENSMUSG(
Ptbp1	10	79854427.	32,48438	-1,768	-3,40582	1,03E-13	2,21E-11	ENSMUSG(
Med16	10	compleme	2,608575	-1,34069	-2,53273	6,84E-05	0,001578	ENSMUSG(
R3hdm4	10	compleme	1,471873	-1,33975	-2,53107	0,001655	0,02368	ENSMUSG(
Cnn2	10	79988584.	16,21688	-1,97561	-3,93295	1,44E-15	4,68E-13	ENSMUSG(
Polr2e	10	compleme	13,02866	-1,20686	-2,30834	2,5E-06	8,93E-05	ENSMUSG(
Sbno2	10	compleme	2,927918	-1,1985	-2,29502	2,57E-07	1,22E-05	ENSMUSG(
Gamt	10	compleme	14,65793	-1,26181	-2,39797	0,000579	0,009753	ENSMUSG(
Rps15	10	80292453.	129,0517	-1,13493	-2,19607	4,88E-08	2,86E-06	ENSMUSG(
Uqcr11	10	compleme	46,47646	1,170282	2,250557	2,43E-05	0,000641	ENSMUSG(
Rexo1	10	compleme	0,408806	-1,597	-3,02513	0,000711	0,0116	ENSMUSG(
Abhd17a	10	compleme	20,8272	1,083157	2,118667	1,9E-06	7,02E-05	ENSMUSG(
Adat3	10	80602880.	0,615412	-1,9419	-3,8421	0,000233	0,004545	ENSMUSG(
Ap3d1	10	compleme	11,40809	-1,15066	-2,22015	7,65E-09	5,5E-07	ENSMUSG(
Dot1l	10	80755206.	0,951108	-1,19335	-2,28683	0,001359	0,019952	ENSMUSG(
Sf3a2	10	80798198.	4,187766	-1,93738	-3,8301	4,12E-07	1,83E-05	ENSMUSG(
Tle2	10	81574561.	2,04495	-1,05241	-2,074	0,000402	0,007122	ENSMUSG(
Hcfc2	10	82696160.	6,103754	1,074231	2,105599	6,27E-06	0,000199	ENSMUSG(
Eid3	10	82866626.	0,969568	3,064303	8,364639	2,94E-06	0,000103	ENSMUSG(
Chst11	10	82985498.	1,195632	-2,95799	-7,77039	1,33E-07	6,9E-06	ENSMUSG(
Aldh1l2	10	compleme	1,622513	-1,13834	-2,20128	0,000729	0,011819	ENSMUSG(
Nuak1	10	compleme	2,360835	-1,74619	-3,35472	2,7E-07	1,27E-05	ENSMUSG(
Btbd11	10	85386814.	2,805158	-1,72916	-3,31535	8,34E-10	7,59E-08	ENSMUSG(
Prdm4	10	compleme	6,888661	-1,09389	-2,13449	3,93E-06	0,000133	ENSMUSG(
Timp3	10	86300372.	1,514902	-3,18612	-9,10158	2,03E-06	7,45E-05	ENSMUSG(
Gnptab	10	88379132.	3,226435	-1,07112	-2,10106	2,28E-05	0,000604	ENSMUSG(
Elk3	10	compleme	7,437345	-1,45929	-2,74972	1,33E-06	5,15E-05	ENSMUSG(
Lta4h	10	93453411.	17,1433	-1,24406	-2,36864	0,000348	0,006322	ENSMUSG(
Snrpf	10	compleme	143,3821	-1,30322	-2,46779	3,38E-08	2,07E-06	ENSMUSG(
Ntn4	10	93640681.	13,87374	-1,72294	-3,30109	1,3E-12	2,19E-10	ENSMUSG(
Fgd6	10	94036001.	1,156769	-2,06136	-4,1738	5,95E-08	3,39E-06	ENSMUSG(
Tmcc3	10	94311949.	0,368749	-2,72763	-6,62366	0,000115	0,002481	ENSMUSG(
Socs2	10	compleme	3,649526	1,336996	2,526247	3,82E-09	2,9E-07	ENSMUSG(
Dcn	10	97479609.	4,812886	5,998166	63,91868	0	0	ENSMUSG(
Lum	10	97565128.	0,950603	4,414912	21,33147	4,34E-11	5,3E-09	ENSMUSG(
Poc1b	10	99107036.	1,676204	-1,1466	-2,21391	0,00163	0,023388	ENSMUSG(
Dusp6	10	99263231.	47,80439	1,110758	2,15959	9,27E-08	4,99E-06	ENSMUSG(
Slc6a15	10	10336778	37,82204	1,327508	2,509688	6,03E-08	3,42E-06	ENSMUSG(
RP24-137J	10	10644439	11,37584	1,426566	2,688061	0,000741	0,011969	ENSMUSG(
Ppfia2	10	10647033	0,219901	-1,8821	-3,6861	0,000989	0,015319	ENSMUSG(
Acss3	10	compleme	2,026937	1,194697	2,288968	0,000259	0,004947	ENSMUSG(

Lin7a	10	107271686	1,822126	2,366969	5,158564	6,61E-07	2,78E-05	ENSMUSG(
Ptprq	10	compleme	6,159484	2,421193	5,356136	2,66E-06	9,39E-05	ENSMUSG(
Phlda1	10	111506286	57,98719	-2,15926	-4,46685	0	0	ENSMUSG(
Trhde	10	compleme	0,558217	-1,85906	-3,62772	0,001287	0,019044	ENSMUSG(
RP23-43M1	10	compleme	308,0255	1,370183	2,585033	1,56E-10	1,71E-08	ENSMUSG(
Ptprr	10	116018213	0,297933	-3,88634	-14,7878	0,000409	0,007228	ENSMUSG(
Ptprb	10	116275523	0,107362	-2,07716	-4,21976	0,002759	0,036427	ENSMUSG(
Kcnmb4	10	compleme	1,104943	-2,26806	-4,81675	0,00061	0,010226	ENSMUSG(
Rab3ip	10	compleme	3,447912	-1,7165	-3,28638	0,000484	0,008392	ENSMUSG(
RP23-468J	10	118258688	44,03868	2,582977	5,991748	5,9E-14	1,36E-11	ENSMUSG(
RP24-211K	10	compleme	17,19843	2,681887	6,416945	5,14E-14	1,22E-11	ENSMUSG(
RP24-211K	10	compleme	17,29461	2,657827	6,310817	2,92E-13	5,8E-11	ENSMUSG(
Hmga2	10	compleme	11,97676	-1,62015	-3,07406	9,44E-15	2,65E-12	ENSMUSG(
Msrb3	10	compleme	3,005802	-1,2278	-2,34209	0,0001	0,002192	ENSMUSG(
Tbc1d30	10	compleme	0,522914	-1,74576	-3,35372	0,000701	0,011453	ENSMUSG(
Ppm1h	10	122678762	0,26407	-2,05408	-4,1528	0,003176	0,041118	ENSMUSG(
Ctdsp2	10	126978717	3,640853	-1,50311	-2,83453	5,18E-07	2,26E-05	ENSMUSG(
March9	10	compleme	3,361775	-1,24995	-2,37834	0,002432	0,032783	ENSMUSG(
B4galnt1	10	127165225	4,75659	-2,15342	-4,44881	2,75E-10	2,78E-08	ENSMUSG(
Dtx3	10	compleme	2,479233	-1,21324	-2,31857	0,001877	0,026294	ENSMUSG(
Pip4k2c	10	compleme	4,600718	-1,01645	-2,02294	2,53E-05	0,000663	ENSMUSG(
Inhbe	10	compleme	0,343866	-2,64753	-6,26592	0,00254	0,033967	ENSMUSG(
R3hdm2	10	127380327	4,65688	-2,00375	-4,01041	7,01E-14	1,58E-11	ENSMUSG(
Nxph4	10	compleme	0,496025	-2,36806	-5,16246	0,003469	0,044358	ENSMUSG(
Baz2a	10	128091577	3,741946	-2,26349	-4,80153	1,22E-15	4,04E-13	ENSMUSG(
Timeless	10	128232065	10,70738	-1,65483	-3,14887	1,57E-08	1,04E-06	ENSMUSG(
Cs	10	128337734	22,34299	-1,13743	-2,19988	1,96E-06	7,21E-05	ENSMUSG(
Smarcc2	10	128459248	13,32195	-1,29968	-2,46174	6,24E-08	3,54E-06	ENSMUSG(
Rab5b	10	compleme	6,12749	-1,07578	-2,10786	4,49E-05	0,001086	ENSMUSG(
Rdh5	10	compleme	1,151369	2,044345	4,124861	8,59E-05	0,001919	ENSMUSG(
Eif4enif1	11	3202392..3	2,206461	-1,25611	-2,38852	0,000327	0,006011	ENSMUSG(
Tug1	11	compleme	2,261801	-1,07248	-2,10305	0,001233	0,018415	ENSMUSG(
Morc2a	11	3649494..3	4,174946	-1,7559	-3,37736	1,05E-08	7,33E-07	ENSMUSG(
Slc35e4	11	compleme	4,775991	1,897848	3,726569	5,79E-10	5,41E-08	ENSMUSG(
Sf3a1	11	4160350..4	6,025121	-1,79556	-3,47149	8,25E-07	3,39E-05	ENSMUSG(
Lif	11	4257557..4	7,493255	-1,38837	-2,61783	4,51E-08	2,68E-06	ENSMUSG(
Ascc2	11	4637747..4	4,928144	-1,25963	-2,39434	3,32E-05	0,000839	ENSMUSG(
Gas2l1	11	compleme	2,631521	-1,04413	-2,06213	0,000957	0,014894	ENSMUSG(
Ube2d-ps	11	5762129..5	1,340475	-1,75649	-3,37874	9,65E-05	0,002126	ENSMUSG(
Aebp1	11	5861947..5	6,912846	-1,84746	-3,59866	1,33E-12	2,23E-10	ENSMUSG(
Zmiz2	11	6389074..6	1,865882	-2,21039	-4,62801	2,16E-08	1,38E-06	ENSMUSG(
Tns3	11	compleme	6,025793	-1,52332	-2,87452	1,3E-11	1,77E-09	ENSMUSG(
Upp1	11	9118103..9	6,358451	-2,95863	-7,77386	1,79E-11	2,36E-09	ENSMUSG(
Sec61g	11	compleme	23,7169	1,142563	2,207728	3,69E-07	1,67E-05	ENSMUSG(
Ppp3r1	11	17159263.	13,11581	-1,21082	-2,31469	8,27E-06	0,000253	ENSMUSG(
Gm12021	11	compleme	2,349102	-1,25371	-2,38454	0,003485	0,044525	ENSMUSG(
Spred2	11	19924375.	2,426501	-1,17232	-2,25373	0,00051	0,008752	ENSMUSG(
Actr2	11	compleme	20,81328	-1,53027	-2,88839	2,3E-07	1,11E-05	ENSMUSG(

Zrsr1	11	22973215.	2,102944	-2,13413	-4,38971	2,51E-07	1,19E-05	ENSMUSG(
Fancl	11	26386135.	11,35946	1,165245	2,242713	3,8E-07	1,72E-05	ENSMUSG(
Efemp1	11	28853204.	7,126234	-3,86396	-14,5602	1,44E-11	1,94E-09	ENSMUSG(
Eml6	11	compleme	1,242205	-2,02077	-4,05801	0,000234	0,004552	ENSMUSG(
Sptbn1	11	compleme	50,47353	-1,2248	-2,33723	1,63E-09	1,37E-07	ENSMUSG(
Gpr75	11	30885358.	0,772198	-1,79374	-3,46713	0,002896	0,037939	ENSMUSG(
Nprl3	11	compleme	1,193735	-1,2352	-2,35414	0,000664	0,010976	ENSMUSG(
Mpg	11	32226505.	29,89509	1,768102	3,406055	0	0	ENSMUSG(
Stk10	11	32533305.	7,330203	-1,6433	-3,12379	4,49E-11	5,46E-09	ENSMUSG(
Fgf18	11	compleme	1,122203	1,995251	3,986855	0,001659	0,023711	ENSMUSG(
Fam196b	11	34314822.	2,208917	-1,07785	-2,11088	0,002313	0,031434	ENSMUSG(
Rars	11	compleme	130,5311	1,051246	2,072318	1,15E-06	4,54E-05	ENSMUSG(
Tenm2	11	compleme	4,276444	-2,70647	-6,52722	1,81E-14	4,9E-12	ENSMUSG(
Ccnjl	11	43528784.	1,034838	-3,15797	-8,92571	3,6E-07	1,64E-05	ENSMUSG(
Ebf1	11	44617317.	0,491476	-2,68221	-6,4184	5,75E-07	2,46E-05	ENSMUSG(
Adam19	11	46055992.	3,45002	-1,55662	-2,94163	2,36E-10	2,45E-08	ENSMUSG(
Cyfip2	11	compleme	2,346555	-2,34634	-5,08531	9,32E-08	5,01E-06	ENSMUSG(
Sgcd	11	compleme	0,121751	2,824981	7,086046	0,000152	0,003152	ENSMUSG(
Psme2b	11	compleme	39,05873	1,13213	2,191821	1E-05	0,000298	ENSMUSG(
Olfr1381	11	49543449.	0,057145	4,185174	18,19127	0,003017	0,039299	ENSMUSG(
Gfpt2	11	49794178.	17,6261	-1,55213	-2,93249	5,4E-06	0,000174	ENSMUSG(
Maml1	11	compleme	4,15322	-1,57539	-2,98016	1,31E-08	8,97E-07	ENSMUSG(
Col23a1	11	51289920.	10,61272	4,695747	25,91557	0	0	ENSMUSG(
Hnrnpab	11	compleme	87,70911	-1,36519	-2,5761	1,3E-07	6,75E-06	ENSMUSG(
0610009B2	11	compleme	7,121984	1,019463	2,027164	0,002664	0,035321	ENSMUSG(
Jade2	11	compleme	6,697606	-1,6255	-3,08549	3,55E-14	8,73E-12	ENSMUSG(
Cdkl3	11	52004221.	1,181507	1,24191	2,365114	0,001805	0,025419	ENSMUSG(
Tcf7	11	compleme	1,752312	-1,61772	-3,0689	0,001028	0,01585	ENSMUSG(
Aff4	11	53350833.	8,607743	-1,34421	-2,53891	1,58E-10	1,72E-08	ENSMUSG(
Kif3a	11	53567379.	7,680304	-1,3275	-2,50968	4,73E-08	2,78E-06	ENSMUSG(
Slc22a5	11	compleme	2,034094	-1,15105	-2,22076	0,000935	0,014567	ENSMUSG(
Slc22a4	11	compleme	2,392634	-1,70758	-3,26613	8,15E-05	0,001835	ENSMUSG(
Acsl6	11	54303798.	0,135174	-3,39239	-10,5006	0,003616	0,045965	ENSMUSG(
Tnip1	11	compleme	30,09921	-2,42339	-5,3643	0	0	ENSMUSG(
Larp1	11	58009064.	17,75602	-2,47993	-5,57872	1,89E-13	3,89E-11	ENSMUSG(
Rnf187	11	compleme	9,222323	-1,00727	-2,0101	0,000429	0,007543	ENSMUSG(
Hist3h2a	11	58954685.	292,7424	1,274742	2,419556	6,4E-09	4,67E-07	ENSMUSG(
Guk1	11	compleme	47,42328	1,100608	2,14445	3,04E-07	1,41E-05	ENSMUSG(
Arf1	11	compleme	222,2316	-1,38422	-2,61031	2,9E-11	3,65E-09	ENSMUSG(
Zkscan17	11	compleme	3,469247	-1,22677	-2,34042	6,29E-06	0,000199	ENSMUSG(
Nlrp3	11	59541568.	0,702604	-2,87659	-7,3441	0,000254	0,004875	ENSMUSG(
Rai1	11	60105013.	0,744847	-2,09586	-4,27481	1,58E-06	5,96E-05	ENSMUSG(
Gid4	11	60417145.	2,029855	-1,28507	-2,43693	0,000279	0,005264	ENSMUSG(
Smcr8	11	60777524.	2,73923	-1,59543	-3,02185	2,8E-08	1,74E-06	ENSMUSG(
Gm12611	11	60816375.	2,293113	-1,75453	-3,37417	0,002427	0,032733	ENSMUSG(
Tmem11	11	compleme	16,18493	-1,58823	-3,0068	3,35E-09	2,58E-07	ENSMUSG(
Map2k3	11	60932033.	1,481512	-1,32803	-2,5106	0,001739	0,024653	ENSMUSG(
Aldh3a1	11	61207537.	65,91993	2,593528	6,035728	4,11E-11	5,08E-09	ENSMUSG(

Aldh3a2	11	compleme	18,46165	1,581121	2,992023	3,61E-13	6,99E-11	ENSMUSG(
Mfap4	11	61485431.	0,680702	2,770674	6,824267	6,45E-05	0,001497	ENSMUSG(
Mapk7	11	compleme	0,71848	-1,42166	-2,67894	0,002659	0,035258	ENSMUSG(
Epn2	11	compleme	4,365428	-1,13277	-2,19279	2,88E-05	0,000741	ENSMUSG(
Fam83g	11	61684091.	2,897001	-1,88722	-3,69921	6,43E-11	7,54E-09	ENSMUSG(
Specc1	11	61956763.	2,324796	-1,75998	-3,38694	8,25E-08	4,51E-06	ENSMUSG(
Ncor1	11	compleme	6,898231	-1,28606	-2,43861	3,49E-09	2,68E-07	ENSMUSG(
Pmp22	11	63128982.	7,314263	-1,092	-2,13169	1,19E-05	0,000347	ENSMUSG(
Arhgap44	11	compleme	1,138828	-1,57594	-2,9813	1,3E-05	0,000375	ENSMUSG(
Ntn1	11	compleme	0,585095	-3,33826	-10,1139	8,62E-07	3,52E-05	ENSMUSG(
Myh10	11	68691559.	12,96262	-1,33167	-2,51693	8,09E-12	1,14E-09	ENSMUSG(
Pfas	11	compleme	8,066193	-1,1646	-2,24172	1,32E-06	5,11E-05	ENSMUSG(
Ctc1	11	69015911.	0,571387	-2,67503	-6,38653	3,51E-07	1,6E-05	ENSMUSG(
Per1	11	69095217.	0,484669	-1,34172	-2,53453	0,003921	0,049116	ENSMUSG(
Trappc1	11	69323980.	7,250382	-2,34418	-5,07773	3,54E-07	1,62E-05	ENSMUSG(
Chd3	11	compleme	51,05613	-2,2722	-4,8306	0	0	ENSMUSG(
Cd68	11	compleme	17,81298	-2,56544	-5,91935	2,33E-14	6,05E-12	ENSMUSG(
Zbtb4	11	69765912.	10,62621	-2,19419	-4,57633	4,77E-14	1,14E-11	ENSMUSG(
Nlgn2	11	compleme	2,950292	-2,50234	-5,66604	2,75E-10	2,78E-08	ENSMUSG(
Plscr3	11	69846376.	1,183524	-1,77474	-3,42176	0,000288	0,005402	ENSMUSG(
Neurl4	11	69901072.	8,096607	-1,35385	-2,55593	4,53E-08	2,68E-06	ENSMUSG(
Gps2	11	69913888.	62,11039	-1,03328	-2,04667	2,51E-05	0,000659	ENSMUSG(
Eif5a	11	compleme	354,5916	-1,61116	-3,05498	1,16E-12	1,98E-10	ENSMUSG(
Elp5	11	compleme	12,7541	-1,4554	-2,74232	1,68E-08	1,1E-06	ENSMUSG(
Gabarap	11	69991143.	86,05795	-1,08184	-2,11674	2,55E-05	0,000668	ENSMUSG(
Dlg4	11	70017085.	4,921394	-1,61341	-3,05975	1,84E-07	9,21E-06	ENSMUSG(
0610010K1	11	compleme	11,81246	-1,1348	-2,19589	0,000606	0,010169	ENSMUSG(
Gm21988	11	compleme	6,686949	-1,91386	-3,76815	7,41E-08	4,1E-06	ENSMUSG(
Rnasek	11	compleme	47,1351	-3,77703	-13,7088	0	0	ENSMUSG(
Pelp1	11	compleme	21,65667	-2,38459	-5,22194	0	0	ENSMUSG(
Arrb2	11	70432635.	6,1779	-2,70746	-6,5317	5,9E-14	1,36E-11	ENSMUSG(
Pld2	11	70540064.	2,141086	-1,54018	-2,90831	2,05E-05	0,000554	ENSMUSG(
Mink1	11	70562881.	12,78309	-2,47035	-5,54179	0	0	ENSMUSG(
Rnf167	11	70647235.	18,74151	-2,45067	-5,4667	0	0	ENSMUSG(
Pfn1	11	compleme	1271,259	-1,68145	-3,2075	4,44E-16	1,61E-13	ENSMUSG(
Camta2	11	compleme	2,845714	-3,37487	-10,3738	0	0	ENSMUSG(
Dhx33	11	compleme	4,871398	-1,07353	-2,10458	0,000209	0,004132	ENSMUSG(
Nlrp1b	11	compleme	0,275474	-2,81368	-7,03073	0,000177	0,003577	ENSMUSG(
Wscd1	11	71749920.	1,491141	-1,94948	-3,86235	3,34E-05	0,000843	ENSMUSG(
4933427D1	11	compleme	3,869252	-1,28911	-2,44376	1,15E-06	4,54E-05	ENSMUSG(
Mybbp1a	11	72441355.	9,141343	-1,6223	-3,07866	7,4E-13	1,31E-10	ENSMUSG(
Gsg2	11	compleme	8,13076	-1,26844	-2,40901	1,56E-06	5,9E-05	ENSMUSG(
Emc6	11	compleme	20,96372	-2,26589	-4,80952	2,74E-14	6,97E-12	ENSMUSG(
Trpv1	11	73234292.	1,26139	-1,40438	-2,64704	0,001262	0,01876	ENSMUSG(
Rap1gap2	11	compleme	9,208295	-1,26405	-2,40169	3,23E-07	1,49E-05	ENSMUSG(
Cluh	11	74649495.	30,09965	-1,70681	-3,26439	4,44E-16	1,61E-13	ENSMUSG(
Mettl16	11	74770830.	2,261345	-1,09839	-2,14115	4,19E-05	0,001026	ENSMUSG(
Sgsm2	11	compleme	6,541805	-1,09414	-2,13486	0,00016	0,003291	ENSMUSG(

Smg6	11	74925823.	5,295822	-1,77383	-3,4196	3,34E-13	6,55E-11	ENSMUSG(
Hic1	11	compleme	0,774997	-2,38722	-5,23147	1,04E-05	0,000308	ENSMUSG(
Rpa1	11	compleme	11,71676	-1,45783	-2,74694	5,91E-07	2,52E-05	ENSMUSG(
Wdr81	11	compleme	4,056577	-1,37304	-2,59015	3,12E-07	1,44E-05	ENSMUSG(
Prpf8	11	75486816.	61,30438	-2,0723	-4,20558	8,24E-13	1,44E-10	ENSMUSG(
Slc43a2	11	75531694.	0,33227	-2,23022	-4,69205	9,98E-06	0,000297	ENSMUSG(
Pitpna	11	75588097.	14,9906	-1,99879	-3,99664	3,16E-13	6,22E-11	ENSMUSG(
Myo1c	11	75650504.	28,87576	-1,96427	-3,90214	0	0	ENSMUSG(
Ywhae	11	75732869.	341,7851	-2,31399	-4,97256	1,11E-15	3,75E-13	ENSMUSG(
Vps53	11	compleme	5,48774	-2,37576	-5,19008	2,09E-14	5,53E-12	ENSMUSG(
Fam57a	11	76179671.	1,397819	-1,07651	-2,10893	0,003248	0,041943	ENSMUSG(
Gemin4	11	compleme	6,008373	-1,49901	-2,82649	2,83E-06	9,95E-05	ENSMUSG(
Abr	11	compleme	13,18073	-1,31961	-2,49599	1,93E-09	1,58E-07	ENSMUSG(
Gosr1	11	compleme	7,074092	-1,20144	-2,29969	3,85E-07	1,73E-05	ENSMUSG(
Blmh	11	76924809.	6,906639	-2,05751	-4,16268	0	0	ENSMUSG(
Nsrp1	11	compleme	3,075991	-3,93519	-15,2971	0	0	ENSMUSG(
Ssh2	11	77216287.	0,980902	-1,87138	-3,65882	5,83E-09	4,3E-07	ENSMUSG(
Gm12346	11	77293444.	310,8401	-1,90688	-3,74998	1,11E-16	4,39E-14	ENSMUSG(
Ankrd13b	11	compleme	2,203031	-2,4927	-5,62831	2,2E-09	1,76E-07	ENSMUSG(
Git1	11	77493562.	5,736859	-2,51703	-5,72403	0	0	ENSMUSG(
Taok1	11	compleme	12,05148	-1,19333	-2,2868	1,57E-09	1,33E-07	ENSMUSG(
Nufip2	11	77686155.	49,5766	-1,29823	-2,45928	2,73E-10	2,77E-08	ENSMUSG(
Myo18a	11	77763246.	4,268409	-1,76856	-3,40714	6,27E-13	1,14E-10	ENSMUSG(
Phf12	11	77982754.	4,869183	-2,48627	-5,60329	1,11E-16	4,39E-14	ENSMUSG(
Flot2	11	78037931.	25,77856	-1,90801	-3,7529	1,69E-13	3,49E-11	ENSMUSG(
Eral1	11	compleme	14,84165	-2,15333	-4,44854	0	0	ENSMUSG(
Fam222b	11	78094660.	0,641868	-4,50202	-22,6592	2,29E-06	8,25E-05	ENSMUSG(
Tlcd1	11	78176711.	1,893617	-1,97602	-3,93408	3,01E-05	0,000769	ENSMUSG(
Supt6	11	compleme	17,6533	-2,56016	-5,89773	0	0	ENSMUSG(
2610507B1	11	78261752.	27,97614	-1,25606	-2,38843	8,69E-10	7,86E-08	ENSMUSG(
Spag5	11	78301529.	11,07555	-1,11478	-2,16562	3,02E-07	1,4E-05	ENSMUSG(
Aldoc	11	78322968.	1,408177	-2,60597	-6,08799	2,75E-05	0,000713	ENSMUSG(
Pigs	11	78328415.	11,61354	-1,30489	-2,47065	2,84E-06	9,96E-05	ENSMUSG(
Sarm1	11	compleme	1,717201	-1,28882	-2,44328	0,000177	0,003583	ENSMUSG(
Fam58b	11	compleme	15,97547	1,029116	2,040773	2,82E-05	0,000728	ENSMUSG(
Ksr1	11	compleme	0,83135	-3,41746	-10,6846	7,32E-11	8,44E-09	ENSMUSG(
Nf1	11	79339693.	4,752585	-1,00496	-2,00689	6,13E-06	0,000195	ENSMUSG(
Psmd11	11	80428615.	36,96908	-1,58613	-3,00243	9,1E-15	2,57E-12	ENSMUSG(
Lig3	11	82781108.	7,023259	-1,09462	-2,13558	4,8E-07	2,11E-05	ENSMUSG(
Rffl	11	compleme	2,62976	-1,78281	-3,44096	1,01E-09	8,96E-08	ENSMUSG(
Ap2b1	11	83299024.	12,24528	-2,03793	-4,10655	0	0	ENSMUSG(
Rasl10b	11	83409137.	0,354869	-3,46213	-11,0206	8,46E-05	0,001896	ENSMUSG(
Taf15	11	83473086.	9,355583	-2,4742	-5,55661	8,88E-16	3,04E-13	ENSMUSG(
Rpl9-ps1	11	compleme	161,0586	1,041949	2,059008	4,26E-05	0,00104	ENSMUSG(
Synrg	11	83964428.	1,174627	-2,00198	-4,0055	1,48E-07	7,58E-06	ENSMUSG(
Acaca	11	84129672.	4,373071	-1,11066	-2,15944	3,35E-06	0,000115	ENSMUSG(
Aatf	11	compleme	46,5578	-1,25367	-2,38448	1,23E-10	1,38E-08	ENSMUSG(
Lhx1	11	compleme	0,90961	-1,59141	-3,01344	0,001157	0,017474	ENSMUSG(

Pigw	11	compleme	2,051619	-1,12611	-2,18269	0,002761	0,036441	ENSMUSG(
Gdpd1	11	compleme	0,431071	-3,04068	-8,22881	0,002186	0,029938	ENSMUSG(
Prr11	11	compleme	15,08978	-1,5354	-2,8987	4,22E-09	3,19E-07	ENSMUSG(
Sept4	11	87568903.	2,641316	1,504266	2,836802	0,000409	0,007222	ENSMUSG(
Rnf43	11	87662722.	0,200203	-3,74175	-13,3776	0,002058	0,028395	ENSMUSG(
Tspoap1	11	87760541.	0,162736	3,515096	11,43271	1,61E-06	6,06E-05	ENSMUSG(
Trim25	11	88999376.	2,466649	-1,44846	-2,72917	6,57E-05	0,001521	ENSMUSG(
Nme2	11	compleme	338,613	1,02708	2,037895	4,93E-07	2,16E-05	ENSMUSG(
Nme1	11	compleme	77,14801	1,080241	2,114389	1,17E-07	6,12E-06	ENSMUSG(
Acsf2	11	compleme	17,2955	1,2792	2,427044	1,48E-08	9,92E-07	ENSMUSG(
Col1a1	11	94936224.	21,90188	-1,8948	-3,71871	6,54E-06	0,000205	ENSMUSG(
Ppp1r9b	11	94991035.	11,09405	-1,55405	-2,9364	1,82E-10	1,94E-08	ENSMUSG(
Igf2bp1	11	compleme	3,922734	-3,48202	-11,1735	0	0	ENSMUSG(
Hoxb9	11	96271457.	1,478697	-2,54811	-5,84869	2,16E-07	1,06E-05	ENSMUSG(
Hoxb3	11	96323326.	0,816294	-1,38301	-2,60811	0,003461	0,044285	ENSMUSG(
Nfe2l1	11	compleme	66,96457	-1,89623	-3,7224	0	0	ENSMUSG(
Sp2	11	compleme	0,448161	-2,39375	-5,25521	0,000404	0,007148	ENSMUSG(
Scrn2	11	97029938.	9,530367	1,141562	2,206198	1,78E-05	0,000492	ENSMUSG(
Tbkbp1	11	compleme	1,252408	-1,70973	-3,27101	0,000191	0,003815	ENSMUSG(
Mrpl45	11	97315716.	10,01314	-1,08302	-2,11847	1,42E-05	0,000404	ENSMUSG(
Arhgap23	11	97415533.	3,272574	-2,60732	-6,09372	4,61E-12	6,84E-10	ENSMUSG(
Mllt6	11	97663414.	6,256975	-2,13376	-4,38861	0	0	ENSMUSG(
Cisd3	11	97685826.	16,89171	-1,22553	-2,33842	5,4E-05	0,001278	ENSMUSG(
Pcgf2	11	compleme	1,435151	-2,4684	-5,53429	5,37E-08	3,09E-06	ENSMUSG(
Cwc25	11	compleme	4,613554	-1,61474	-3,06256	4,43E-10	4,27E-08	ENSMUSG(
Lasp1	11	97799000.	13,30237	-1,51527	-2,85852	3,81E-12	5,77E-10	ENSMUSG(
Cacnb1	11	compleme	1,715771	-1,72933	-3,31573	5,3E-06	0,000171	ENSMUSG(
Fbxl20	11	compleme	1,058364	-1,05065	-2,07147	0,001114	0,016944	ENSMUSG(
Med1	11	compleme	13,38487	-1,9167	-3,77558	0	0	ENSMUSG(
Cdk12	11	98203059.	10,62344	-2,03768	-4,10585	0	0	ENSMUSG(
Stard3	11	98358368.	6,38952	-1,38965	-2,62015	4,66E-08	2,75E-06	ENSMUSG(
Mien1	11	compleme	12,06172	-1,09027	-2,12914	0,001186	0,017807	ENSMUSG(
Csf3	11	98701263.	35,54258	-9,12566	-558,595	1,73E-12	2,8E-10	ENSMUSG(
Med24	11	compleme	9,693295	-1,35725	-2,56196	3,89E-10	3,8E-08	ENSMUSG(
Nr1d1	11	compleme	1,618987	-2,86735	-7,29721	7,21E-09	5,21E-07	ENSMUSG(
Casc3	11	98804905.	20,43901	-1,84319	-3,58803	2,22E-16	8,45E-14	ENSMUSG(
Wipf2	11	98863638.	2,226159	-1,80009	-3,48242	1,87E-09	1,54E-07	ENSMUSG(
Rara	11	98927818.	3,42779	-1,25631	-2,38884	1,17E-05	0,000341	ENSMUSG(
Krt15	11	compleme	7,904347	1,228279	2,342873	0,000925	0,014443	ENSMUSG(
Krt19	11	compleme	2,12106	1,7476	3,357996	0,000977	0,015169	ENSMUSG(
Krt17	11	compleme	1,208165	1,498411	2,825313	0,003151	0,040834	ENSMUSG(
Krt42	11	compleme	0,69036	1,829465	3,554051	0,001661	0,023733	ENSMUSG(
Jup	11	compleme	2,692065	-4,62471	-24,6705	4,26E-11	5,22E-09	ENSMUSG(
Cnp	11	10057490(	11,225	-1,05657	-2,07998	6,9E-06	0,000215	ENSMUSG(
Kat2a	11	compleme	7,729285	-1,44247	-2,71785	3,16E-07	1,46E-05	ENSMUSG(
Rab5c	11	compleme	41,3219	-1,23243	-2,34963	8,25E-09	5,87E-07	ENSMUSG(
Kcnh4	11	compleme	0,190932	1,909304	3,756278	0,003676	0,046593	ENSMUSG(
Coasy	11	10108256!	7,573101	-1,68717	-3,22025	1,4E-07	7,21E-06	ENSMUSG(

Mlx	11	101087277	19,95958	-1,04405	-2,06201	8,16E-05	0,001836	ENSMUSG(
Tubg1	11	101119938	31,74188	-1,34611	-2,54226	4,62E-10	4,42E-08	ENSMUSG(
Tubg2	11	101155907	42,69013	-1,31232	-2,4834	2,94E-10	2,95E-08	ENSMUSG(
Cntnap1	11	101170523	4,869527	-1,95819	-3,88574	1,24E-14	3,38E-12	ENSMUSG(
Aoc2	11	101325063	0,582211	-2,49955	-5,65509	0,000269	0,005117	ENSMUSG(
Vat1	11	compleme	56,48279	-1,57845	-2,9865	1,55E-15	4,95E-13	ENSMUSG(
Etv4	11	compleme	20,07422	-1,52701	-2,88188	6,63E-11	7,75E-09	ENSMUSG(
Tmem101	11	compleme	19,24476	1,103995	2,149491	4,57E-06	0,000151	ENSMUSG(
G6pc3	11	102189620	11,23864	-1,40402	-2,64638	4,6E-08	2,72E-06	ENSMUSG(
Grn	11	102430315	12,09556	-1,83643	-3,57125	2,15E-14	5,65E-12	ENSMUSG(
Gpatch8	11	compleme	4,2491	-1,83321	-3,56328	5,31E-13	9,8E-11	ENSMUSG(
Fzd2	11	102604396	5,576482	-1,30384	-2,46886	5,88E-07	2,51E-05	ENSMUSG(
Adam11	11	102761439	0,341687	-2,15162	-4,44326	0,002147	0,029472	ENSMUSG(
Eftud2	11	compleme	21,59566	-1,17822	-2,26298	3,59E-08	2,18E-06	ENSMUSG(
Kif18b	11	compleme	5,415579	-2,4028	-5,28828	3,97E-10	3,86E-08	ENSMUSG(
C1ql1	11	compleme	7,25711	-1,27166	-2,41439	0,000173	0,003512	ENSMUSG(
Plcd3	11	compleme	3,869931	-1,49791	-2,82434	2,38E-06	8,55E-05	ENSMUSG(
Hexim1	11	103116231	7,791307	-1,86042	-3,63112	2,78E-08	1,73E-06	ENSMUSG(
Fmnl1	11	103171107	1,043434	-1,92544	-3,79852	8,29E-06	0,000253	ENSMUSG(
Map3k14	11	compleme	1,171586	-1,62087	-3,0756	4,48E-05	0,001086	ENSMUSG(
Mapt	11	104231390	0,165256	-2,97027	-7,83682	0,000313	0,005796	ENSMUSG(
Itgb3	11	104608000	1,903017	-2,27047	-4,82479	1,75E-09	1,45E-07	ENSMUSG(
Tanc2	11	105589986	1,190618	-1,40425	-2,64681	2,76E-06	9,69E-05	ENSMUSG(
Limd2	11	compleme	0,68815	-1,55098	-2,93015	0,00207	0,028512	ENSMUSG(
Strada	11	compleme	1,869828	-1,19188	-2,28451	0,00014	0,002952	ENSMUSG(
Ddx42	11	106216926	22,65442	-1,22968	-2,34516	1,4E-07	7,2E-06	ENSMUSG(
Psmc5	11	106256154	42,43066	-1,27047	-2,4124	8,58E-07	3,5E-05	ENSMUSG(
Ern1	11	compleme	0,633677	-1,3838	-2,60954	0,001108	0,016879	ENSMUSG(
Polg2	11	compleme	0,72224	-1,59035	-3,01123	0,000504	0,008672	ENSMUSG(
Pitpnc1	11	compleme	4,331501	-1,40452	-2,64729	2,44E-05	0,000642	ENSMUSG(
Psmd12	11	107479484	34,76541	-1,06992	-2,09932	1,53E-05	0,000432	ENSMUSG(
Prkca	11	compleme	2,091438	-1,47998	-2,78944	8,78E-08	4,76E-06	ENSMUSG(
Slc16a6	11	compleme	16,90733	-2,58142	-5,98529	9,31E-09	6,54E-07	ENSMUSG(
Abca8b	11	compleme	1,347386	1,489081	2,8071	1,9E-05	0,000519	ENSMUSG(
Cdr2l	11	115381916	1,572639	-1,91408	-3,76873	2,6E-06	9,2E-05	ENSMUSG(
Tmem94	11	115765433	4,7787	-1,65891	-3,15778	3,15E-11	3,95E-09	ENSMUSG(
Itgb4	11	115974709	1,633096	-1,98557	-3,9602	0,001046	0,016071	ENSMUSG(
Unk	11	116030322	4,825219	-1,35921	-2,56544	4,35E-06	0,000145	ENSMUSG(
Wbp2	11	compleme	12,26662	-2,44769	-5,45541	0	0	ENSMUSG(
Mrpl38	11	compleme	12,79275	-1,32758	-2,50982	9,06E-07	3,66E-05	ENSMUSG(
Srp68	11	compleme	9,363746	-1,02955	-2,04138	0,000337	0,00615	ENSMUSG(
Ubald2	11	116434094	5,345043	-1,79685	-3,4746	6,57E-07	2,77E-05	ENSMUSG(
Ube2o	11	compleme	5,362022	-1,61669	-3,0667	5,58E-09	4,14E-07	ENSMUSG(
Cygb	11	compleme	4,737401	1,207142	2,308799	0,000199	0,003942	ENSMUSG(
Tnrc6c	11	117654289	0,987542	-1,63397	-3,10367	1,19E-06	4,69E-05	ENSMUSG(
Syngr2	11	117809668	9,399555	-1,73852	-3,33693	9,81E-08	5,22E-06	ENSMUSG(
Cyth1	11	compleme	2,702188	-1,13215	-2,19185	0,000509	0,008748	ENSMUSG(
Usp36	11	compleme	1,500578	-1,26881	-2,40962	0,000234	0,004566	ENSMUSG(

Timp2	11	compleme	6,133278	-1,5781	-2,98577	1,35E-06	5,19E-05	ENSMUSG(
Cant1	11	compleme	3,983263	-1,05495	-2,07764	5,95E-05	0,001396	ENSMUSG(
Gaa	11	119267887	2,241374	-1,10339	-2,14859	0,001019	0,015725	ENSMUSG(
Rnf213	11	119393100	1,495886	-1,23533	-2,35436	1,49E-06	5,68E-05	ENSMUSG(
Endov	11	119491347	1,584184	1,785152	3,446547	1,79E-07	8,97E-06	ENSMUSG(
Rptor	11	119602905	2,973129	-1,26349	-2,40076	4,42E-06	0,000146	ENSMUSG(
Baiap2	11	119942763	3,970838	-1,69259	-3,23237	1,41E-10	1,55E-08	ENSMUSG(
Actg1	11	compleme	437,5722	-1,06278	-2,08896	6,52E-06	0,000205	ENSMUSG(
Mrpl12	11	120484613	12,19132	-2,50952	-5,69432	1,5E-10	1,64E-08	ENSMUSG(
Arhgdia	11	compleme	13,32017	-1,62864	-3,09221	2,14E-07	1,05E-05	ENSMUSG(
Pycr1	11	compleme	12,31589	1,119952	2,173398	7,58E-07	3,14E-05	ENSMUSG(
Cenpx	11	compleme	3,666956	1,67915	3,202391	3,81E-05	0,000946	ENSMUSG(
Hmga1-rs1	11	120762794	33,0476	-2,6998	-6,49713	0	0	ENSMUSG(
Fasn	11	compleme	7,776784	-1,70255	-3,25475	4,37E-07	1,94E-05	ENSMUSG(
Csnk1d	11	compleme	5,626307	-2,08665	-4,24759	1,72E-08	1,12E-06	ENSMUSG(
Tbcd	11	121451949	1,304351	-1,49523	-2,81908	3,23E-05	0,000819	ENSMUSG(
Kif3c	12	3365132..3	1,91031	-2,50133	-5,66207	6,85E-14	1,55E-11	ENSMUSG(
Asxl2	12	3426857..3	1,050871	-1,20123	-2,29936	5,37E-05	0,001271	ENSMUSG(
Efr3b	12	compleme	0,326972	-1,71969	-3,29366	0,000279	0,005262	ENSMUSG(
Itsn2	12	4592638..4	3,727392	-1,0024	-2,00333	2,16E-06	7,84E-05	ENSMUSG(
Klhl29	12	compleme	1,355551	-1,46184	-2,75459	1,16E-05	0,000338	ENSMUSG(
Vsnl1	12	compleme	68,40146	2,432656	5,398863	0	0	ENSMUSG(
Fam84a	12	compleme	0,340767	-4,0537	-16,6068	0,000728	0,011819	ENSMUSG(
Trib2	12	compleme	13,67886	2,142112	4,414077	6,29E-11	7,4E-09	ENSMUSG(
Lpin1	12	compleme	16,01958	1,770083	3,410736	2,09E-06	7,62E-05	ENSMUSG(
Kcnf1	12	compleme	1,676798	1,217989	2,326223	0,00062	0,010378	ENSMUSG(
Odc1	12	17544873.	113,2401	1,050233	2,070865	3,53E-06	0,00012	ENSMUSG(
Iah1	12	21316392.	19,43373	1,779779	3,433736	1,73E-08	1,13E-06	ENSMUSG(
Klf11	12	24651371.	2,229351	-1,16985	-2,24989	0,000323	0,005944	ENSMUSG(
Rrm2	12	24708241.	62,25402	-1,26571	-2,40445	2,1E-06	7,65E-05	ENSMUSG(
Id2	12	compleme	14,42728	1,146019	2,213023	0,000331	0,006068	ENSMUSG(
Rsad2	12	compleme	0,407153	4,322369	20,00612	3,84E-08	2,31E-06	ENSMUSG(
Cmpk2	12	26469204.	0,742317	1,803694	3,49113	0,000723	0,011758	ENSMUSG(
Sox11	12	compleme	0,236128	2,454422	5,480934	4,95E-05	0,001185	ENSMUSG(
Pxdn	12	29937608.	30,26733	-1,05734	-2,08109	5E-06	0,000163	ENSMUSG(
Sh3yl1	12	30911668.	2,289423	1,222926	2,334197	0,000734	0,011891	ENSMUSG(
Lamb1	12	31265234.	29,41351	-1,02027	-2,0283	5,24E-06	0,00017	ENSMUSG(
Cbll1	12	compleme	5,122224	-1,32121	-2,49875	6,51E-07	2,75E-05	ENSMUSG(
Prkar2b	12	compleme	2,343112	-2,56403	-5,91359	1,29E-06	5,02E-05	ENSMUSG(
Hdac9	12	compleme	2,191725	-1,76137	-3,39019	7,02E-07	2,93E-05	ENSMUSG(
Scin	12	compleme	0,285767	2,247994	4,750218	0,001845	0,025924	ENSMUSG(
Dock4	12	40446053.	6,103224	-1,21127	-2,31541	2,32E-08	1,47E-06	ENSMUSG(
Nrcam	12	44328885.	0,530537	-2,82138	-7,06837	2,17E-06	7,86E-05	ENSMUSG(
Stxbp6	12	compleme	0,212157	-3,04991	-8,2816	0,001148	0,017353	ENSMUSG(
Coch	12	51593341.	2,556043	5,944042	61,56516	0	0	ENSMUSG(
Akap6	12	52699383.	0,433388	2,039783	4,111837	4,11E-06	0,000138	ENSMUSG(
Npas3	12	53248677.	0,889387	1,630846	3,096944	5,24E-05	0,001245	ENSMUSG(
Egln3	12	compleme	1,119921	3,553471	11,7409	5,03E-09	3,75E-07	ENSMUSG(

Foxa1	12	compleme	0,558032	-2,38739	-5,23209	0,0004	0,00708	ENSMUSG(
Pnn	12	59066884.	21,29954	-1,76132	-3,39009	3,69E-14	8,92E-12	ENSMUSG(
Klhdc2	12	69296681.	9,010725	1,218795	2,327523	1,49E-06	5,69E-05	ENSMUSG(
L3hypdh	12	compleme	5,70882	1,278398	2,425695	4,59E-06	0,000151	ENSMUSG(
Mnat1	12	73123717.	13,34169	1,069765	2,099091	1,36E-05	0,00039	ENSMUSG(
Zbtb1	12	76370266.	5,292986	1,050084	2,070651	1,73E-06	6,46E-05	ENSMUSG(
Churc1	12	76765538.	6,728854	1,161275	2,23655	6,16E-07	2,62E-05	ENSMUSG(
Plek2	12	compleme	8,108193	-3,94508	-15,4024	1,79E-05	0,000494	ENSMUSG(
Actn1	12	compleme	50,35687	-1,73851	-3,3369	2,69E-14	6,87E-12	ENSMUSG(
Dcaf5	12	compleme	2,626703	-1,3773	-2,59781	1E-06	4E-05	ENSMUSG(
Galnt16	12	80518277.	1,262892	-1,82718	-3,54844	1,25E-05	0,000362	ENSMUSG(
Ccdc177	12	compleme	0,801802	2,112295	4,323786	1,11E-06	4,38E-05	ENSMUSG(
Susd6	12	80790510.	0,76703	-2,04829	-4,13614	3,92E-05	0,00097	ENSMUSG(
Smoc1	12	81026808.	4,64901	-1,83962	-3,57917	6,29E-07	2,67E-05	ENSMUSG(
Sipa1l1	12	82170016.	2,772043	-1,00812	-2,01129	8,17E-05	0,001837	ENSMUSG(
Zfyve1	12	compleme	1,21106	-2,49447	-5,63523	2,47E-07	1,18E-05	ENSMUSG(
Rbm25	12	83631236.	14,15032	-1,4861	-2,8013	6,99E-08	3,9E-06	ENSMUSG(
Numb	12	compleme	7,732876	-2,20258	-4,60303	1,11E-16	4,39E-14	ENSMUSG(
Pnma1	12	compleme	2,810293	-1,15209	-2,22236	0,00239	0,032332	ENSMUSG(
Elmsan1	12	compleme	1,673692	-2,45001	-5,4642	2,44E-12	3,84E-10	ENSMUSG(
Ltbp2	12	compleme	1,2316	-2,01534	-4,04276	1,02E-06	4,07E-05	ENSMUSG(
Vash1	12	86678700.	0,663294	-2,33151	-5,03333	0,000332	0,006082	ENSMUSG(
Cep128	12	compleme	0,880572	-1,20726	-2,30898	0,000309	0,005742	ENSMUSG(
Ston2	12	compleme	0,316243	-3,57262	-11,8977	1,54E-05	0,000433	ENSMUSG(
Foxn3	12	compleme	2,366356	-1,93385	-3,82072	9,04E-09	6,38E-07	ENSMUSG(
Ttc7b	12	compleme	3,718126	-1,5468	-2,92169	1,96E-07	9,72E-06	ENSMUSG(
Gpr68	12	compleme	0,742878	1,430208	2,694856	0,002259	0,03079	ENSMUSG(
Fbln5	12	compleme	0,612322	-2,32206	-5,00046	1,16E-05	0,000338	ENSMUSG(
Asb2	12	compleme	0,226872	-4,48873	-22,4513	0,002021	0,027947	ENSMUSG(
Otub2	12	103388682	3,586738	-1,39862	-2,63649	0,000732	0,011867	ENSMUSG(
B430119L(	12	103895897	0,559862	2,494467	5,635201	0,000151	0,003127	ENSMUSG(
Dicer1	12	compleme	6,034146	-1,06503	-2,09222	9,33E-07	3,76E-05	ENSMUSG(
Clmn	12	compleme	0,303836	-1,63577	-3,10753	0,000617	0,010338	ENSMUSG(
Gskip	12	105685352	7,326058	1,020213	2,028219	0,000324	0,005972	ENSMUSG(
Papola	12	105784694	24,32709	-1,07627	-2,10858	5,32E-07	2,31E-05	ENSMUSG(
Evl	12	108554720	1,130857	-1,39464	-2,62924	0,002443	0,032876	ENSMUSG(
Dync1h1	12	110601452	33,1174	-1,83544	-3,5688	7,88E-15	2,29E-12	ENSMUSG(
Hsp90aa1	12	compleme	182,6797	-1,86341	-3,63866	2,22E-16	8,45E-14	ENSMUSG(
Rcor1	12	111039351	2,312684	-1,20735	-2,30913	8,43E-05	0,00189	ENSMUSG(
Cdc42bpb	12	compleme	6,863232	-1,43927	-2,71184	3,36E-10	3,33E-08	ENSMUSG(
Ckb	12	compleme	32,7826	-1,79229	-3,46364	5,84E-07	2,49E-05	ENSMUSG(
Siva1	12	112644828	24,01429	1,50882	2,845772	7,95E-09	5,69E-07	ENSMUSG(
Cep170b	12	112722174	3,263639	-1,07972	-2,11362	3,04E-05	0,000775	ENSMUSG(
Ahnak2	12	compleme	0,785504	-1,54665	-2,92139	3,3E-06	0,000114	ENSMUSG(
Mta1	12	113098278	9,338847	-1,83747	-3,57382	4,18E-12	6,24E-10	ENSMUSG(
Crip1	12	113146316	0,296169	3,281175	9,721473	0,000332	0,006082	ENSMUSG(
Klf6	13	5861489..5	4,265093	-1,53877	-2,90547	6,09E-06	0,000194	ENSMUSG(
Pitrm1	13	6548157..6	27,44117	-1,11745	-2,16963	5,13E-08	2,98E-06	ENSMUSG(

Idi1	13	8885501..8	4,788943	1,031203	2,043728	0,000709	0,011574	ENSMUSG(
Larp4b	13	9093881..9	4,756666	-1,66144	-3,16332	1,46E-09	1,25E-07	ENSMUSG(
Actn2	13	compleme	13,51893	-1,45077	-2,73354	1,21E-10	1,36E-08	ENSMUSG(
Heatr1	13	12395398.	19,42336	-1,07356	-2,10462	1,14E-07	5,95E-06	ENSMUSG(
Lgals8	13	compleme	4,046028	-1,01904	-2,02657	0,003967	0,049523	ENSMUSG(
Gpr137b	13	compleme	2,822485	-1,85034	-3,60585	1,57E-06	5,95E-05	ENSMUSG(
Nid1	13	13437602.	9,946712	-2,02277	-4,06363	0	0	ENSMUSG(
Lyst	13	13590409.	1,956967	-1,06703	-2,09511	7,83E-05	0,001774	ENSMUSG(
Hecw1	13	compleme	1,628237	-1,64259	-3,12227	2,27E-09	1,81E-07	ENSMUSG(
Hist1h2ai	13	21716422.	965,8818	1,39756	2,634556	2,92E-11	3,68E-09	ENSMUSG(
Hist1h2bq	13	compleme	10,59471	1,126043	2,182593	9,67E-06	0,000289	ENSMUSG(
Hist1h2ao	13	21810465.	284,6091	1,884204	3,691491	0	0	ENSMUSG(
Zfp184	13	21945094.	19,72798	2,303308	4,935881	1,11E-16	4,39E-14	ENSMUSG(
Hist1h2ah	13	compleme	1711,267	1,625529	3,085553	9,88E-15	2,76E-12	ENSMUSG(
Hist1h4h	13	23531050.	9966,791	2,363932	5,147714	0	0	ENSMUSG(
Hist1h2bh	13	compleme	174,0652	-1,08613	-2,12303	6,13E-07	2,61E-05	ENSMUSG(
Hist1h3f	13	23544465.	479,8971	1,0111	2,015447	1,58E-06	5,96E-05	ENSMUSG(
Hist1h3e	13	compleme	1218,141	1,214462	2,320543	2,03E-09	1,64E-07	ENSMUSG(
Hist1h2ae	13	compleme	3168,67	1,634643	3,105106	5,55E-16	1,95E-13	ENSMUSG(
Hist1h3d	13	23575763.	958,2653	1,065729	2,093227	2,21E-07	1,08E-05	ENSMUSG(
Hist1h4d	13	23581598.	10689,34	2,35312	5,109281	0	0	ENSMUSG(
Hist1h2ac	13	compleme	1401,872	1,37219	2,588633	1,95E-11	2,55E-09	ENSMUSG(
Hist1h3c	13	compleme	505,0742	1,441681	2,716372	3,6E-12	5,47E-10	ENSMUSG(
4930558J2	13	compleme	0,462625	2,918127	7,558643	0,000178	0,003589	ENSMUSG(
Hist1h4a	13	compleme	63,56323	1,348167	2,545884	6,46E-09	4,71E-07	ENSMUSG(
Hist1h2aa	13	23934462.	77,43478	1,072499	2,103073	1,02E-05	0,000302	ENSMUSG(
Fam65b	13	24582189.	0,401717	-1,99498	-3,98611	1,09E-05	0,00032	ENSMUSG(
Sox4	13	compleme	6,164555	-1,12027	-2,17388	0,001941	0,027058	ENSMUSG(
E2f3	13	compleme	4,199999	-1,09444	-2,1353	4,19E-05	0,001026	ENSMUSG(
Foxc1	13	31806691.	2,285875	-1,52192	-2,87172	3,76E-05	0,000934	ENSMUSG(
Gmds	13	compleme	4,90463	-1,54513	-2,91831	3,77E-07	1,71E-05	ENSMUSG(
Fars2	13	36117411.	20,57305	1,028481	2,039876	4,48E-06	0,000148	ENSMUSG(
Dsp	13	38151328.	0,647303	-4,3185	-19,9526	6,67E-08	3,75E-06	ENSMUSG(
Tfap2a	13	compleme	1,372501	-2,52195	-5,7436	2,83E-07	1,32E-05	ENSMUSG(
Pak1ip1	13	41001023.	39,97113	1,304995	2,470828	6,15E-11	7,29E-09	ENSMUSG(
Tmem14c	13	41016250.	53,43713	1,107174	2,154232	1,66E-07	8,41E-06	ENSMUSG(
Elovl2	13	compleme	10,06178	1,332985	2,519233	8,15E-07	3,35E-05	ENSMUSG(
Hivep1	13	42052021.	1,508359	-2,00683	-4,01899	1,48E-08	9,93E-07	ENSMUSG(
Gfod1	13	compleme	0,402995	-1,69614	-3,24033	0,002279	0,031037	ENSMUSG(
Rnf182	13	43615710.	2,906935	-2,08195	-4,23378	9,5E-08	5,1E-06	ENSMUSG(
Jarid2	13	44729474.	1,456047	-1,78267	-3,44063	7,15E-07	2,98E-05	ENSMUSG(
Hsp25-ps1	13	compleme	45,74227	-3,08678	-8,49596	0	0	ENSMUSG(
Atxn1	13	compleme	1,794999	-1,25996	-2,39488	2,34E-05	0,000619	ENSMUSG(
Rbm24	13	46418300.	1,567288	-1,80352	-3,49071	0,000396	0,007022	ENSMUSG(
Cap2	13	46501848.	5,092408	-2,21794	-4,65228	1,4E-08	9,49E-07	ENSMUSG(
Nup153	13	compleme	16,03431	-1,0002	-2,00027	7,78E-06	0,00024	ENSMUSG(
Rnf144b	13	47122720.	0,920894	-1,82243	-3,53676	9,27E-05	0,002053	ENSMUSG(
Id4	13	48261427.	0,841126	2,570339	5,939491	0,001878	0,026295	ENSMUSG(

Fam120a	13	compleme	30,09314	-1,16386	-2,24057	4,05E-07	1,81E-05	ENSMUSG(
Cenpp	13	compleme	8,366976	1,395353	2,630528	7,72E-05	0,001757	ENSMUSG(
Aspn	13	49544443.	0,326158	3,158822	8,931001	7,15E-05	0,001641	ENSMUSG(
Hist1h2al	13	compleme	4256,118	1,511108	2,850289	7,95E-13	1,4E-10	ENSMUSG(
S1pr3	13	51408618.	1,719113	-2,28788	-4,8834	1,04E-07	5,48E-06	ENSMUSG(
Shc3	13	compleme	1,046314	-3,62239	-12,3154	3,9E-05	0,000964	ENSMUSG(
Sema4d	13	compleme	0,385799	-2,5224	-5,74538	0,000118	0,002535	ENSMUSG(
Syk	13	52583173.	0,260566	3,178544	9,053927	4,66E-07	2,05E-05	ENSMUSG(
Simc1	13	54503779.	2,354342	-1,30342	-2,46813	2,53E-05	0,000664	ENSMUSG(
4833439L1	13	compleme	13,05462	-1,06639	-2,09419	8,43E-06	0,000257	ENSMUSG(
Nop16	13	compleme	14,3825	-1,61823	-3,06999	2,99E-07	1,39E-05	ENSMUSG(
Rnf44	13	compleme	1,089389	-1,99973	-3,99925	7,53E-06	0,000233	ENSMUSG(
Mxd3	13	compleme	7,559096	1,392436	2,625215	9,66E-07	3,87E-05	ENSMUSG(
Pdlim7	13	compleme	6,044991	-1,34284	-2,5365	1,22E-08	8,39E-07	ENSMUSG(
Ddx46	13	55635027.	25,2037	-1,40179	-2,64229	1,7E-10	1,84E-08	ENSMUSG(
Hnrnpa0	13	compleme	9,216507	-1,94878	-3,86047	6,65E-08	3,74E-06	ENSMUSG(
2210016F1	13	compleme	3,391307	1,184782	2,27329	0,000347	0,006297	ENSMUSG(
Golm1	13	compleme	7,586912	-1,2635	-2,40078	3,59E-07	1,64E-05	ENSMUSG(
Dapk1	13	60601947.	0,942508	-2,31561	-4,97816	1,83E-06	6,78E-05	ENSMUSG(
Cntnap3	13	compleme	9,501262	1,106252	2,152857	9,07E-07	3,67E-05	ENSMUSG(
Gm26965	13	compleme	0,415583	7,21955	149,0394	0,002331	0,031625	ENSMUSG(
BC048507	13	67863326.	9,847619	-1,62115	-3,0762	2,67E-05	0,000693	ENSMUSG(
Mtrr	13	compleme	12,51554	-1,01834	-2,02559	3,22E-06	0,000111	ENSMUSG(
Papd7	13	compleme	8,342226	-1,2686	-2,40928	1,64E-07	8,32E-06	ENSMUSG(
Med10	13	69809882.	25,79958	-1,15744	-2,23061	1,44E-05	0,000408	ENSMUSG(
Adamts16	13	compleme	0,492121	-5,02253	-32,5037	5,67E-07	2,43E-05	ENSMUSG(
Ndufs6	13	compleme	95,48703	-1,15461	-2,22624	3,25E-07	1,49E-05	ENSMUSG(
Lpcat1	13	73467197.	11,03045	-1,26824	-2,40868	1,37E-08	9,33E-07	ENSMUSG(
Slc12a7	13	73763697.	3,165127	-2,41555	-5,33524	3,82E-11	4,77E-09	ENSMUSG(
Hnrnpa1l2-	13	74864305.	128,2188	-2,37321	-5,18092	5,55E-16	1,95E-13	ENSMUSG(
Pcsk1	13	75089826.	1,745203	-3,6309	-12,3882	0,000146	0,003058	ENSMUSG(
Rhobtb3	13	compleme	94,60545	1,827065	3,548144	8,23E-12	1,15E-09	ENSMUSG(
Gm6311	13	75954352.	302,1101	-1,09543	-2,13676	0,000194	0,003854	ENSMUSG(
Mctp1	13	76384535.	5,421618	-2,1405	-4,40916	3,3E-10	3,28E-08	ENSMUSG(
Nr2f1	13	compleme	4,0399	1,078973	2,112532	4,46E-05	0,001082	ENSMUSG(
Arrdc3	13	80883384.	37,12797	1,841058	3,582726	3,33E-07	1,53E-05	ENSMUSG(
Gm8399	13	81064684.	23,26431	-1,67375	-3,19043	1,26E-07	6,56E-06	ENSMUSG(
Mef2c	13	83504034.	3,995354	1,396704	2,632993	2,14E-05	0,000572	ENSMUSG(
Gm4076	13	compleme	1,460593	-2,98867	-7,93742	0,00218	0,02989	ENSMUSG(
Vcan	13	compleme	2,155856	-1,34738	-2,5445	4,41E-05	0,001071	ENSMUSG(
Ssbp2	13	91460283.	1,016	-2,01609	-4,04486	0,000336	0,00614	ENSMUSG(
Jmy	13	compleme	1,282796	-1,36723	-2,57974	0,000115	0,002487	ENSMUSG(
Arsb	13	93771679.	1,51881	-1,12169	-2,17601	0,001904	0,026589	ENSMUSG(
Wdr41	13	94976344.	5,605639	1,058395	2,082613	2,75E-05	0,000713	ENSMUSG(
F2rl1	13	compleme	0,633431	-5,58121	-47,8755	0,000172	0,003512	ENSMUSG(
Iqgap2	13	compleme	2,067912	1,893085	3,714286	7,55E-09	5,43E-07	ENSMUSG(
Hmgcr	13	compleme	3,253106	-1,13981	-2,20353	0,000935	0,014567	ENSMUSG(
Gm6169	13	compleme	11,96608	-1,78026	-3,43488	1,86E-08	1,2E-06	ENSMUSG(

Gm10260	13	compleme	245,0106	1,111815	2,161174	5,28E-07	2,29E-05	ENSMUSG(
Tmem171	13	compleme	3,016658	-1,54348	-2,91497	8E-05	0,001805	ENSMUSG(
Map1b	13	compleme	12,48874	-2,78571	-6,89576	0	0	ENSMUSG(
Ocln	13	compleme	0,39015	-4,00666	-16,074	2,72E-05	0,000706	ENSMUSG(
Pik3r1	13	compleme	1,230231	-1,17579	-2,25916	0,00028	0,005285	ENSMUSG(
Mast4	13	compleme	0,295646	-1,52069	-2,86929	0,00018	0,003621	ENSMUSG(
Cenpk	13	104228955	22,28069	1,162382	2,238267	9,65E-07	3,87E-05	ENSMUSG(
Adamts6	13	104287873	5,077602	-1,28858	-2,44288	0,000985	0,015264	ENSMUSG(
Depdc1b	13	108316332	7,057106	-1,24863	-2,37615	5,33E-06	0,000172	ENSMUSG(
Actbl2	13	111255013	1,000761	-2,14803	-4,43221	0,000375	0,006714	ENSMUSG(
Plpp1	13	112800894	6,098359	1,310755	2,480713	0,00023	0,004504	ENSMUSG(
Esm1	13	113209659	18,08891	-2,76923	-6,81744	8,44E-15	2,44E-12	ENSMUSG(
Fst	13	compleme	102,3292	1,442064	2,717094	1,22E-09	1,07E-07	ENSMUSG(
Parp8	13	compleme	8,435732	-1,0323	-2,04528	2,03E-05	0,000549	ENSMUSG(
Ccl28	13	119623819	0,29339	3,327739	10,04036	2E-06	7,34E-05	ENSMUSG(
Flnb	14	7817957..7	49,72527	-1,15834	-2,23201	1,93E-08	1,24E-06	ENSMUSG(
Dnase1l3	14	compleme	0,041502	3,337853	10,111	0,003605	0,045838	ENSMUSG(
Acox2	14	compleme	5,890955	-1,23792	-2,35859	0,000227	0,004443	ENSMUSG(
Oit1	14	compleme	0,198079	3,487426	11,21553	0,001634	0,023428	ENSMUSG(
Cadps	14	compleme	2,419458	6,100581	68,62111	0	0	ENSMUSG(
Atxn7	14	14012491.	1,789026	-1,24637	-2,37244	4,24E-05	0,001036	ENSMUSG(
Rarb	14	compleme	2,082854	-2,96254	-7,79494	2,68E-05	0,000696	ENSMUSG(
Ube2e2	14	compleme	4,336475	-1,47796	-2,78554	3,31E-07	1,52E-05	ENSMUSG(
Nid2	14	19751257.	1,460341	-2,56062	-5,8996	3,66E-07	1,66E-05	ENSMUSG(
Saysd1	14	compleme	5,075206	3,180374	9,065418	3,18E-12	4,88E-10	ENSMUSG(
Usp54	14	compleme	1,358386	-1,21794	-2,32614	0,000116	0,002506	ENSMUSG(
Zswim8	14	20707552.	5,820282	-1,02335	-2,03264	1,17E-05	0,000339	ENSMUSG(
Ndst2	14	compleme	2,864833	-1,39966	-2,63839	5,97E-05	0,0014	ENSMUSG(
Dlg5	14	compleme	5,558248	-1,30249	-2,46654	6,35E-07	2,68E-05	ENSMUSG(
Polr3a	14	compleme	6,856471	-1,10835	-2,15599	1,34E-05	0,000384	ENSMUSG(
Ppif	14	25694154.	8,473292	-1,1178	-2,17016	8,64E-06	0,000262	ENSMUSG(
Gm9780	14	compleme	0,555415	2,004175	4,011592	0,003808	0,047928	ENSMUSG(
Gm2178	14	26514554.	482,8864	1,270577	2,41258	6,06E-10	5,63E-08	ENSMUSG(
Dcp1a	14	30479565.	2,523798	-1,13322	-2,19347	0,000224	0,0044	ENSMUSG(
Spcs1	14	compleme	25,04254	-1,08472	-2,12096	2,84E-06	9,97E-05	ENSMUSG(
Pbrm1	14	31019138.	6,121553	-1,08167	-2,11648	2,59E-05	0,000676	ENSMUSG(
Nt5dc2	14	31134853.	23,64969	-1,77711	-3,4274	1,64E-11	2,18E-09	ENSMUSG(
Dnah1	14	compleme	0,472628	-1,31337	-2,48521	0,000206	0,004078	ENSMUSG(
Ogdhl	14	32322019.	2,463586	-1,27885	-2,42646	0,000388	0,006924	ENSMUSG(
Sncg	14	compleme	1,082548	-3,68296	-12,8435	0,00134	0,019715	ENSMUSG(
Tspan14	14	compleme	5,354834	-1,89415	-3,71702	6,02E-08	3,42E-06	ENSMUSG(
Fam213a	14	compleme	5,548052	-1,93755	-3,83056	0,000406	0,007181	ENSMUSG(
Ptger2	14	44988195.	2,018207	2,940304	7,675733	1,92E-09	1,57E-07	ENSMUSG(
Mapk1ip1l	14	47298314.	2,910976	-2,65046	-6,27867	1,02E-13	2,19E-11	ENSMUSG(
Peli2	14	48120869.	0,64248	-2,52156	-5,74202	7,19E-05	0,001648	ENSMUSG(
Parp2	14	50807946.	8,886494	-1,13447	-2,19538	0,000126	0,002674	ENSMUSG(
Mettl3	14	compleme	23,05495	1,045485	2,06406	1,65E-07	8,35E-06	ENSMUSG(
Trav3d-2	14	52887587.	1,78941	2,204593	4,609445	0,001426	0,020806	ENSMUSG(

Abhd4	14	54254188.	1,787621	-1,58863	-3,00764	6,54E-05	0,001516	ENSMUSG(
Oxa1l	14	54360841.	4,697986	-1,07614	-2,10839	7E-05	0,001611	ENSMUSG(
Lrp10	14	54464164.	7,508717	-1,93874	-3,83372	2,54E-12	3,96E-10	ENSMUSG(
Cdh24	14	compleme	2,96522	-1,85984	-3,62967	1,49E-06	5,69E-05	ENSMUSG(
Acin1	14	compleme	15,22357	-1,7705	-3,41173	5,55E-16	1,95E-13	ENSMUSG(
Gm20521	14	54883441.	2,9429	-4,88662	-29,5814	1,73E-07	8,68E-06	ENSMUSG(
Ap1g2	14	compleme	3,028448	-1,2028	-2,30187	0,000156	0,00323	ENSMUSG(
Irf9	14	55603571.	2,787602	1,935032	3,823866	1,64E-09	1,38E-07	ENSMUSG(
Rabggta	14	compleme	5,917928	-1,06127	-2,08676	6,63E-05	0,001534	ENSMUSG(
Lats2	14	compleme	1,576601	-1,07972	-2,11363	0,000813	0,01296	ENSMUSG(
Micu2	14	compleme	27,35836	1,113483	2,163673	4,96E-07	2,17E-05	ENSMUSG(
Kcnrg	14	61607482.	4,010705	1,650037	3,138416	0,000235	0,004569	ENSMUSG(
Rnaseh2b	14	62292589.	4,953745	1,151196	2,220979	1,69E-06	6,32E-05	ENSMUSG(
Wdfy2	14	62837690.	3,90204	-1,65847	-3,15682	4E-05	0,000985	ENSMUSG(
Fam167a	14	63436394.	0,471952	-2,60589	-6,08768	0,001045	0,016065	ENSMUSG(
Sox7	14	63943674.	0,794418	-1,6443	-3,12596	0,003063	0,039843	ENSMUSG(
Kif13b	14	64652531.	2,808301	-2,22771	-4,68389	1,6E-09	1,35E-07	ENSMUSG(
Ints9	14	64950045.	10,01868	-1,20459	-2,30472	4,68E-07	2,06E-05	ENSMUSG(
Fzd3	14	compleme	0,600375	-3,36892	-10,3311	0,00078	0,012525	ENSMUSG(
Clu	14	65968483.	14,38409	-1,13847	-2,20147	7,27E-05	0,001665	ENSMUSG(
Ephx2	14	compleme	1,31545	1,741513	3,343857	0,00123	0,018387	ENSMUSG(
Nkx3-1	14	69190638.	0,710771	-1,80431	-3,49263	0,001978	0,027524	ENSMUSG(
Gm21451	14	69390854.	0,355553	4,272682	19,32882	0,000566	0,009571	ENSMUSG(
Loxl2	14	69609068.	7,557463	-1,62919	-3,0934	1,53E-08	1,02E-06	ENSMUSG(
Rhobtb2	14	compleme	6,212699	-1,06474	-2,0918	1,48E-05	0,000419	ENSMUSG(
Ccar2	14	compleme	29,26839	-1,33088	-2,51556	1,98E-10	2,1E-08	ENSMUSG(
Sorbs3	14	compleme	3,908439	-1,70117	-3,25165	7,94E-08	4,37E-06	ENSMUSG(
Reep4	14	70545251.	3,39006	-2,72884	-6,62921	3,8E-09	2,88E-07	ENSMUSG(
Hr	14	70552212.	3,056738	-1,47129	-2,7727	2,57E-05	0,000671	ENSMUSG(
Fam160b2	14	compleme	2,69998	-2,55227	-5,86555	5,02E-12	7,35E-10	ENSMUSG(
Dmtn	14	compleme	0,550568	-3,02445	-8,13671	2,54E-05	0,000665	ENSMUSG(
Lpar6	14	73237895.	0,171544	3,229884	9,381922	4,71E-05	0,001135	ENSMUSG(
Lcp1	14	75131101.	1,090202	-5,50062	-45,2742	1,85E-08	1,2E-06	ENSMUSG(
Zc3h13	14	75284373.	8,664363	-1,03644	-2,05116	0,000565	0,009565	ENSMUSG(
Gtf2f2	14	compleme	90,47308	1,239501	2,361169	1,9E-09	1,56E-07	ENSMUSG(
Rps2-ps6	14	compleme	38,28233	-1,06282	-2,089	0,000332	0,006078	ENSMUSG(
Enox1	14	77156763.	0,57462	-1,85516	-3,61793	0,002272	0,030946	ENSMUSG(
Tnfsf11	14	compleme	0,151588	3,747093	13,42726	0,0005	0,008629	ENSMUSG(
Rgcc	14	compleme	7,094369	1,940687	3,838884	5,25E-07	2,29E-05	ENSMUSG(
Pibf1	14	99099433.	12,80079	1,020215	2,028221	1,41E-05	0,000403	ENSMUSG(
Klf5	14	99298691.	6,490912	-1,35763	-2,56263	6,2E-05	0,001448	ENSMUSG(
Klf12	14	compleme	1,621667	-2,83505	-7,13565	1,77E-06	6,61E-05	ENSMUSG(
Commd6	14	compleme	26,37843	1,359481	2,565928	1,9E-08	1,22E-06	ENSMUSG(
Cln5	14	10307021€	7,391587	1,447601	2,727541	2,14E-07	1,05E-05	ENSMUSG(
Slitrk6	14	compleme	0,189466	3,181563	9,072898	0,000664	0,010976	ENSMUSG(
Slitrk5	14	11167511£	2,321824	-3,07047	-8,40044	5,7E-13	1,04E-10	ENSMUSG(
Gpc5	14	11509221£	1,256222	2,430467	5,390678	1,94E-06	7,15E-05	ENSMUSG(
Tgds	14	compleme	17,22819	1,269305	2,410455	2,4E-07	1,15E-05	ENSMUSG(

Cldn10	14	118787908	0,366439	3,236671	9,426165	1,16E-05	0,000338	ENSMUSG(
Dnajc3	14	118937932	14,89245	1,652837	3,144513	1,14E-08	7,93E-07	ENSMUSG(
Uggt2	14	compleme	3,811234	1,298005	2,458886	3,36E-08	2,06E-06	ENSMUSG(
Farp1	14	121035200	3,554727	-1,04683	-2,06599	0,003869	0,048564	ENSMUSG(
Zic5	14	compleme	2,359168	-1,36794	-2,58102	7,18E-05	0,001646	ENSMUSG(
Itgbl1	14	123659971	11,09099	1,606564	3,045257	6,31E-06	0,0002	ENSMUSG(
Ccdc152	15	compleme	39,23486	2,572232	5,947286	5,89E-11	7,01E-09	ENSMUSG(
Ghr	15	compleme	0,590079	-1,19328	-2,28673	0,002682	0,03553	ENSMUSG(
Fbxo4	15	compleme	9,348316	1,254796	2,386335	7,62E-07	3,15E-05	ENSMUSG(
Dab2	15	6299788..6	4,091079	-1,56719	-2,96328	8,02E-10	7,33E-08	ENSMUSG(
Osmr	15	compleme	0,492201	-1,3081	-2,47616	0,000389	0,006935	ENSMUSG(
Slc1a3	15	compleme	2,138429	-1,10805	-2,15554	0,000524	0,008983	ENSMUSG(
Rai14	15	compleme	35,06736	-1,8182	-3,5264	3,52E-14	8,69E-12	ENSMUSG(
Adamts12	15	11064790.	5,920352	-2,38623	-5,22788	1,11E-16	4,39E-14	ENSMUSG(
Npr3	15	compleme	1,53897	-2,62664	-6,17584	0,00015	0,003122	ENSMUSG(
Pdzd2	15	compleme	0,199523	-1,96571	-3,90604	5,58E-05	0,001316	ENSMUSG(
6030458C1	15	compleme	6,219956	-1,73728	-3,33406	1,37E-10	1,51E-08	ENSMUSG(
Basp1	15	compleme	80,49279	-2,44034	-5,42771	0	0	ENSMUSG(
Myo10	15	25622525.	24,84792	-1,40643	-2,6508	2,12E-10	2,22E-08	ENSMUSG(
Fam134b	15	25843264.	1,465893	-5,3596	-41,0584	5,83E-07	2,49E-05	ENSMUSG(
Gm6576	15	27025386.	18,39591	-1,53596	-2,89981	1,09E-05	0,00032	ENSMUSG(
Dap	15	31224314.	1,489489	-2,27507	-4,84021	6,42E-05	0,001492	ENSMUSG(
Sdc2	15	32920723.	4,865657	1,14017	2,20407	0,000338	0,006155	ENSMUSG(
Rida	15	compleme	8,834711	1,118742	2,171575	3,58E-06	0,000122	ENSMUSG(
Lrp12	15	compleme	7,409266	-2,22612	-4,67873	0	0	ENSMUSG(
Angpt1	15	compleme	7,2072	2,353145	5,109369	9,63E-10	8,65E-08	ENSMUSG(
Eif3e	15	compleme	712,0648	1,089364	2,127802	3,32E-07	1,52E-05	ENSMUSG(
Sybu	15	compleme	1,280344	-2,7058	-6,52419	4,2E-05	0,001028	ENSMUSG(
Csmd3	15	compleme	0,296944	1,445865	2,724262	0,000645	0,010729	ENSMUSG(
Trps1	15	compleme	8,310874	-1,06564	-2,09309	1,26E-06	4,9E-05	ENSMUSG(
Tnfrsf11b	15	compleme	1570,038	5,215903	37,16577	0	0	ENSMUSG(
Nov	15	54745702.	31,06076	1,487979	2,804958	1,79E-05	0,000495	ENSMUSG(
Deptor	15	55112317.	1,923252	2,28261	4,865574	8,47E-13	1,48E-10	ENSMUSG(
Col14a1	15	55307750.	0,46474	2,648315	6,269348	1,34E-06	5,17E-05	ENSMUSG(
Zhx2	15	57694665.	0,660212	-2,86168	-7,26859	1,74E-05	0,000483	ENSMUSG(
Fam83a	15	57985419.	0,345103	-3,18694	-9,1068	0,002331	0,031625	ENSMUSG(
Mtss1	15	compleme	0,508371	-2,09715	-4,27863	9,72E-05	0,002141	ENSMUSG(
Washc5	15	compleme	23,13231	-1,25741	-2,39067	1,4E-09	1,2E-07	ENSMUSG(
Nsmce2	15	59374198.	19,17249	1,056032	2,079206	3,57E-05	0,000895	ENSMUSG(
Trib1	15	59648350.	3,72591	-1,88046	-3,68193	7,12E-09	5,15E-07	ENSMUSG(
Fam84b	15	compleme	1,13008	1,159972	2,234531	0,003638	0,046145	ENSMUSG(
Efr3a	15	65787034.	26,12071	1,299577	2,461567	2,49E-06	8,88E-05	ENSMUSG(
Kcnq3	15	compleme	1,037361	-3,38074	-10,4161	4,84E-13	9,04E-11	ENSMUSG(
Ndrg1	15	compleme	35,50197	1,250027	2,378459	2,91E-05	0,000747	ENSMUSG(
Khdrbs3	15	68928420.	16,63158	2,580958	5,98337	1,22E-15	4,04E-13	ENSMUSG(
Chrac1	15	73090392.	3,15139	-1,23092	-2,34716	0,00193	0,026921	ENSMUSG(
Gsdmd	15	75862339.	12,98875	1,188724	2,279511	1,42E-06	5,44E-05	ENSMUSG(
Eef1d	15	compleme	113,0468	1,184082	2,272187	1,39E-08	9,42E-07	ENSMUSG(

Fam83h	15	compleme	2,936919	-1,58694	-3,00412	7,67E-08	4,23E-06	ENSMUSG(
Scrib	15	compleme	12,23603	-1,10743	-2,15461	1,7E-07	8,55E-06	ENSMUSG(
Grina	15	76246807.	28,51366	-1,84765	-3,59915	1,11E-16	4,39E-14	ENSMUSG(
Exosc4	15	76327397.	33,08203	1,106105	2,152637	4,12E-07	1,83E-05	ENSMUSG(
Cpsf1	15	compleme	4,28513	-1,56651	-2,96188	2,4E-08	1,51E-06	ENSMUSG(
Arhgap39	15	compleme	0,342892	-1,64548	-3,12853	0,002183	0,029907	ENSMUSG(
Myh9	15	compleme	59,61266	-1,86858	-3,65174	2,29E-07	1,11E-05	ENSMUSG(
Mpst	15	78406712.	15,39245	1,405017	2,648208	1,12E-08	7,8E-07	ENSMUSG(
Elfn2	15	compleme	0,448113	-1,9313	-3,81399	0,001129	0,017123	ENSMUSG(
Cdc42ep1	15	78842647.	5,683743	-1,83775	-3,57452	1,1E-07	5,78E-06	ENSMUSG(
Lgals1	15	78926725.	2682,089	1,12984	2,188345	3,81E-07	1,72E-05	ENSMUSG(
Triobp	15	78947724.	9,850614	1,208352	2,310735	2,65E-07	1,24E-05	ENSMUSG(
Tmem184b	15	compleme	14,00554	-1,09568	-2,13714	0,000111	0,002404	ENSMUSG(
Dmc1	15	compleme	1,900383	2,985799	7,921641	2,29E-07	1,11E-05	ENSMUSG(
Cbx6	15	compleme	6,741002	-1,7961	-3,47279	3,4E-10	3,37E-08	ENSMUSG(
Rpl3	15	compleme	119,0026	-1,10579	-2,15217	1,13E-05	0,00033	ENSMUSG(
Syngr1	15	80091334.	0,765916	-1,51468	-2,85735	0,003657	0,046363	ENSMUSG(
Rps19bp1	15	compleme	25,9961	1,365675	2,576968	9,18E-08	4,95E-06	ENSMUSG(
Tnrc6b	15	80711319.	3,100034	-1,65679	-3,15315	1,52E-07	7,77E-06	ENSMUSG(
Mkl1	15	compleme	1,549445	-1,37014	-2,58496	0,000257	0,004918	ENSMUSG(
Rbx1	15	81466316.	91,89581	1,512915	2,853861	4,12E-13	7,78E-11	ENSMUSG(
Tob2	15	compleme	3,064694	-1,8923	-3,71226	3,98E-06	0,000134	ENSMUSG(
Phf5a	15	compleme	106,3804	1,21465	2,320845	3,62E-08	2,19E-06	ENSMUSG(
Srebf2	15	82147266.	9,752088	-1,07437	-2,1058	0,000708	0,011566	ENSMUSG(
Ndufa6	15	compleme	240,601	1,16511	2,242504	4,03E-08	2,41E-06	ENSMUSG(
Cyb5r3	15	compleme	6,783771	-1,48337	-2,79601	7,77E-05	0,001764	ENSMUSG(
Tspo	15	83563592.	183,401	1,907684	3,752063	0	0	ENSMUSG(
Ttll12	15	compleme	13,77453	-1,07353	-2,10458	1,65E-05	0,000461	ENSMUSG(
Fbln1	15	85205949.	9,860895	-1,21057	-2,31429	6,7E-07	2,81E-05	ENSMUSG(
Atxn10	15	85336381.	25,86355	1,121359	2,175519	3,1E-06	0,000108	ENSMUSG(
Gtse1	15	85859707.	2,011711	-1,69613	-3,24031	0,000474	0,008243	ENSMUSG(
Celsr1	15	compleme	1,157099	-1,84066	-3,58173	8,98E-08	4,85E-06	ENSMUSG(
Fam19a5	15	87625230.	0,500347	8,188508	291,7336	0,000349	0,006328	ENSMUSG(
Zbed4	15	88751660.	1,919973	-1,38944	-2,61977	0,000121	0,002597	ENSMUSG(
Creld2	15	88819646.	12,44236	1,594406	3,019702	1,18E-07	6,18E-06	ENSMUSG(
Plxnb2	15	compleme	4,902825	-1,43004	-2,69455	7,83E-06	0,000241	ENSMUSG(
Dennd6b	15	compleme	0,954983	-2,65405	-6,29431	4,26E-05	0,00104	ENSMUSG(
Sco2	15	compleme	13,22666	1,067458	2,095738	3,87E-05	0,000959	ENSMUSG(
Tymp	15	compleme	1,406193	1,800712	3,483921	0,000366	0,006591	ENSMUSG(
Kif21a	15	compleme	3,006016	-1,52471	-2,87729	5,48E-07	2,37E-05	ENSMUSG(
Hdac7	15	compleme	7,10537	-1,51273	-2,8535	1E-09	8,92E-08	ENSMUSG(
Vdr	15	compleme	1,510847	-2,77693	-6,85395	4,77E-12	7,04E-10	ENSMUSG(
Tmem106c	15	97964229.	53,31241	1,039257	2,055169	1,91E-07	9,5E-06	ENSMUSG(
Col2a1	15	compleme	0,165596	1,983635	3,954884	0,001996	0,027705	ENSMUSG(
Kmt2d	15	compleme	1,163068	-1,14005	-2,20388	0,00029	0,005436	ENSMUSG(
Nckap5l	15	compleme	4,308143	-1,65362	-3,14622	1,73E-09	1,44E-07	ENSMUSG(
Asic1	15	99670718.	0,652856	-1,90868	-3,75466	0,002521	0,033752	ENSMUSG(
Gm17349	15	compleme	1,889441	3,342792	10,14567	0,001791	0,025245	ENSMUSG(

Smarcd1	15	99702287.	18,11282	-1,4214	-2,67845	2,08E-10	2,19E-08	ENSMUSG(
Lima1	15	compleme	14,32376	-1,24172	-2,36481	1,6E-09	1,35E-07	ENSMUSG(
Slc4a8	15	100761747	0,251818	-2,47792	-5,57093	6,28E-06	0,000199	ENSMUSG(
Acvr1b	15	101174067	2,989286	-1,25131	-2,38057	9,74E-05	0,002145	ENSMUSG(
Krt87	15	compleme	3,436303	-1,68835	-3,22288	0,000393	0,006977	ENSMUSG(
Krt81	15	compleme	1,424306	-1,90388	-3,74219	0,001862	0,026121	ENSMUSG(
Krt83	15	compleme	3,431134	-3,63308	-12,407	1,68E-09	1,4E-07	ENSMUSG(
Krt75	15	compleme	2,078189	-4,85705	-28,9814	2,22E-10	2,31E-08	ENSMUSG(
Krt5	15	compleme	2,094478	-2,67483	-6,38564	2,03E-05	0,00055	ENSMUSG(
Spryd3	15	compleme	4,657905	-1,81521	-3,51911	2,24E-07	1,09E-05	ENSMUSG(
Espl1	15	102296293	8,549344	-1,81811	-3,52619	2,44E-15	7,45E-13	ENSMUSG(
Prr13	15	102459028	16,86369	-1,61884	-3,07127	6,2E-11	7,31E-09	ENSMUSG(
Atf7	15	compleme	0,694175	-2,04659	-4,1313	6,13E-07	2,61E-05	ENSMUSG(
Hoxc13	15	102921103	1,676776	-1,91963	-3,78326	3,36E-05	0,000846	ENSMUSG(
Hoxc11	15	102954427	3,543142	-1,4975	-2,82353	0,000318	0,005883	ENSMUSG(
Hoxc10	15	102966796	5,061705	-1,55725	-2,94292	8,45E-06	0,000257	ENSMUSG(
Hoxc8	15	102990607	0,785612	-3,41437	-10,6617	0,000156	0,003226	ENSMUSG(
Hoxc6	15	102998257	2,478477	-1,30912	-2,4779	0,000386	0,00689	ENSMUSG(
Hoxc4	15	103018934	1,985666	-1,60336	-3,0385	7,76E-05	0,001764	ENSMUSG(
Cbx5	15	compleme	8,448717	-1,49538	-2,81938	1,22E-07	6,33E-06	ENSMUSG(
Hnrnpa1	15	103240432	242,5911	-2,01173	-4,03267	1,3E-11	1,77E-09	ENSMUSG(
Zfp385a	15	compleme	0,917215	-2,18732	-4,55458	0,000745	0,012022	ENSMUSG(
Naa60	16	3872375..3	0,991244	-1,82872	-3,55222	0,000425	0,00748	ENSMUSG(
Tfap4	16	compleme	14,24086	-1,37346	-2,5909	7,42E-05	0,001695	ENSMUSG(
Ubald1	16	compleme	0,655074	-2,68647	-6,43735	3,48E-06	0,000119	ENSMUSG(
Anks3	16	compleme	3,565797	1,027779	2,038883	0,002849	0,037411	ENSMUSG(
Litaf	16	compleme	3,526431	-1,39822	-2,63575	2,47E-05	0,000648	ENSMUSG(
Gspt1	16	compleme	55,61331	-1,07984	-2,1138	5,82E-06	0,000186	ENSMUSG(
Cpped1	16	compleme	1,054776	-1,64965	-3,13757	7,12E-05	0,001635	ENSMUSG(
Myh11	16	compleme	1,512285	-1,84961	-3,60403	3,68E-05	0,000917	ENSMUSG(
Smpd4	16	17619354.	8,245351	-1,71631	-3,28596	5,26E-14	1,24E-11	ENSMUSG(
Med15	16	compleme	4,143856	-1,14838	-2,21664	0,001468	0,021326	ENSMUSG(
Dgcr6	16	18052860.	5,17049	1,257365	2,390587	1,45E-05	0,000412	ENSMUSG(
Prodh	16	compleme	0,084766	2,460733	5,504964	0,00251	0,033614	ENSMUSG(
Zdhhc8	16	compleme	1,436844	-1,67592	-3,19522	0,000134	0,002839	ENSMUSG(
Gnb1l	16	18498768.	1,754733	1,146701	2,21407	0,000651	0,010806	ENSMUSG(
2510002D2	16	18836578.	4,571879	2,575554	5,960997	4,31E-07	1,91E-05	ENSMUSG(
Mrpl40	16	compleme	46,85053	1,211707	2,316115	8,32E-08	4,53E-06	ENSMUSG(
Lamp3	16	compleme	2,293213	3,118451	8,684549	8,59E-10	7,81E-08	ENSMUSG(
B3gnt5	16	19760208.	1,774779	-2,22685	-4,68109	3,19E-09	2,47E-07	ENSMUSG(
Klhl24	16	20097554.	2,483712	-1,2532	-2,38369	0,003118	0,040459	ENSMUSG(
Abcc5	16	compleme	2,426758	-1,94443	-3,84887	3,36E-13	6,56E-11	ENSMUSG(
Alg3	16	compleme	34,68404	-1,3127	-2,48405	5,77E-09	4,27E-07	ENSMUSG(
Eif4g1	16	20668313.	145,3469	-1,35384	-2,55591	9,92E-10	8,86E-08	ENSMUSG(
Liph	16	compleme	0,707106	-3,63111	-12,39	7,29E-06	0,000226	ENSMUSG(
Igf2bp2	16	compleme	14,92457	-1,12486	-2,1808	4,11E-08	2,46E-06	ENSMUSG(
Etv5	16	compleme	26,29656	-1,63787	-3,11206	1,78E-15	5,58E-13	ENSMUSG(
Eif4a2	16	23107444.	213,9794	1,429872	2,694228	1,49E-12	2,46E-10	ENSMUSG(

St6gal1	16	23224740.	0,486482	-2,64224	-6,24302	0,000255	0,004894	ENSMUSG(
Bcl6	16	compleme	3,908157	-2,55553	-5,87885	1,11E-16	4,39E-14	ENSMUSG(
Trp63	16	25683763.	1,478264	-2,48786	-5,60944	1,31E-07	6,82E-06	ENSMUSG(
Il1rap	16	26581704.	4,142966	1,190981	2,283079	2,58E-06	9,14E-05	ENSMUSG(
Fgf12	16	compleme	0,704887	-2,77716	-6,85501	1,91E-05	0,00052	ENSMUSG(
Hrasls	16	29209695.	1,005011	-2,15516	-4,45419	0,000267	0,005083	ENSMUSG(
Bdh1	16	31422280.	0,881529	-4,12905	-17,4972	1,87E-05	0,000511	ENSMUSG(
Rnf168	16	32277459.	4,303185	-1,01684	-2,02348	0,000503	0,008659	ENSMUSG(
Ubxn7	16	32332252.	3,144567	-1,60457	-3,04106	3,94E-10	3,83E-08	ENSMUSG(
Tm4sf19	16	32400506.	4,193159	-3,4869	-11,2114	9,83E-07	3,93E-05	ENSMUSG(
Tnk2	16	32643874.	4,534993	-1,49899	-2,82645	1,46E-08	9,87E-07	ENSMUSG(
Rubcn	16	compleme	1,308036	-2,73906	-6,67637	4,14E-12	6,2E-10	ENSMUSG(
Slc12a8	16	33517328.	0,785682	1,680354	3,205066	0,000393	0,006977	ENSMUSG(
Sec22a	16	compleme	8,907453	1,460672	2,752366	9,5E-10	8,55E-08	ENSMUSG(
Dirc2	16	compleme	5,550912	1,020983	2,029302	4,65E-05	0,00112	ENSMUSG(
Parp9	16	35938470.	7,713321	1,801039	3,484712	6,68E-12	9,49E-10	ENSMUSG(
Ccdc58	16	36071660.	152,0352	1,20009	2,29754	1,78E-09	1,47E-07	ENSMUSG(
Rabl3	16	37539885.	6,43566	1,089886	2,128571	2,55E-06	9,06E-05	ENSMUSG(
Lrrc58	16	37868389.	62,38767	-1,30026	-2,46273	7,23E-08	4,02E-06	ENSMUSG(
Slc35a5	16	compleme	10,82537	1,1101	2,158606	4,17E-07	1,85E-05	ENSMUSG(
Abhd10	16	compleme	6,022426	1,213153	2,318437	9,5E-07	3,82E-05	ENSMUSG(
Phldb2	16	compleme	10,08567	-1,08533	-2,12186	8,12E-06	0,000249	ENSMUSG(
Ift57	16	49699233.	50,06797	1,011282	2,015701	6,63E-07	2,79E-05	ENSMUSG(
Cd47	16	49855618.	10,47273	1,27696	2,423279	2,02E-09	1,64E-07	ENSMUSG(
Nfkbiz	16	compleme	7,648799	-1,59591	-3,02284	2,1E-10	2,21E-08	ENSMUSG(
Nxpe3	16	compleme	1,940482	-1,84673	-3,59684	7,96E-08	4,38E-06	ENSMUSG(
Rpl24	16	55966275.	2540,615	1,082793	2,118133	9,86E-08	5,25E-06	ENSMUSG(
Zbtb11	16	55973883.	14,46471	1,05751	2,081336	7,77E-07	3,21E-05	ENSMUSG(
Trmt10c	16	compleme	24,47125	1,19684	2,29237	6,19E-08	3,51E-06	ENSMUSG(
Senp7	16	56048338.	1,335578	1,055337	2,078204	3,68E-05	0,000917	ENSMUSG(
Adgrg7	16	compleme	19,40447	1,977811	3,938949	0	0	ENSMUSG(
Tmem45a	16	compleme	1,536197	1,376182	2,595804	0,001313	0,019377	ENSMUSG(
Tbc1d23	16	compleme	36,55517	1,1109	2,159803	1,85E-08	1,2E-06	ENSMUSG(
Epha3	16	compleme	0,442981	1,952397	3,87017	0,000889	0,013962	ENSMUSG(
Vgll3	16	65815633.	1,032746	-1,68587	-3,21735	0,000812	0,012952	ENSMUSG(
Ncam2	16	81200697.	0,179709	2,97182	7,845253	0,000162	0,00333	ENSMUSG(
Mir155hg	16	84703167.	2,583944	3,026503	8,14832	2,83E-08	1,75E-06	ENSMUSG(
Mrpl39	16	compleme	23,88459	1,135329	2,196687	2,07E-07	1,02E-05	ENSMUSG(
Usp16	16	87454703.	10,66448	1,251164	2,380333	5,71E-10	5,34E-08	ENSMUSG(
Bach1	16	87698945.	12,98921	1,44007	2,71334	5,42E-11	6,53E-09	ENSMUSG(
Il10rb	16	91406164.	2,280735	1,133333	2,19365	0,00092	0,014366	ENSMUSG(
Tmem50b	16	compleme	4,194751	1,385847	2,613253	7,27E-05	0,001664	ENSMUSG(
Slc5a3	16	92058322.	6,166312	1,382083	2,606443	0,000131	0,002783	ENSMUSG(
Smim11	16	92301286.	14,72007	1,189861	2,281307	2,88E-05	0,000741	ENSMUSG(
Runx1	16	compleme	0,376595	-1,35997	-2,5668	0,002135	0,029312	ENSMUSG(
Setd4	16	compleme	1,343848	1,502744	2,833813	2,46E-05	0,000648	ENSMUSG(
Erg	16	compleme	1,323867	-2,30447	-4,93987	7,15E-05	0,001641	ENSMUSG(
Hmgn1	16	compleme	36,18359	1,389611	2,620081	4,76E-12	7,04E-10	ENSMUSG(

Mx1	16	compleme	1,605055	2,903495	7,482367	7,18E-10	6,6E-08	ENSMUSG(
Mx2	16	97535308.	3,519014	2,818435	7,053968	1,1E-12	1,9E-10	ENSMUSG(
Zbtb21	16	compleme	2,728399	-1,34894	-2,54725	1,05E-06	4,16E-05	ENSMUSG(
Arid1b	17	4994332..5	1,992999	-1,37802	-2,59911	1,87E-05	0,000511	ENSMUSG(
Ezr	17	compleme	54,86141	-1,22101	-2,33109	3,18E-08	1,96E-06	ENSMUSG(
Igf2r	17	compleme	11,55891	-1,54385	-2,91571	9,64E-10	8,65E-08	ENSMUSG(
Acat3	17	compleme	7,177024	1,080188	2,114312	0,000137	0,002877	ENSMUSG(
Afdn	17	13760539.	11,55697	-1,11513	-2,16614	1,68E-08	1,1E-06	ENSMUSG(
Thbs2	17	compleme	20,77817	-1,08581	-2,12256	1,06E-07	5,6E-06	ENSMUSG(
Psmb1	17	compleme	578,7732	1,04991	2,070401	7,44E-07	3,09E-05	ENSMUSG(
Rgmb	17	compleme	6,635087	-2,42157	-5,35754	2,22E-16	8,45E-14	ENSMUSG(
Vmn2r99	17	19362135.	0,19326	2,219765	4,658175	0,001874	0,026274	ENSMUSG(
Gm5145	17	20570362.	7,119794	-1,20142	-2,29966	0,001034	0,01592	ENSMUSG(
Zfp160	17	21008941.	12,30292	1,269122	2,410149	1,13E-07	5,94E-06	ENSMUSG(
Hcfc1r1	17	23673596.	19,46594	-1,15157	-2,22155	0,000252	0,004847	ENSMUSG(
Tnfrsf12a	17	compleme	100,4284	1,457692	2,746687	4,58E-12	6,81E-10	ENSMUSG(
Paqr4	17	compleme	10,77291	1,16551	2,243126	1,26E-06	4,9E-05	ENSMUSG(
Flywch2	17	compleme	46,53537	1,096623	2,138536	2,06E-06	7,54E-05	ENSMUSG(
Srrm2	17	23790662.	3,341918	-1,84445	-3,59116	2,5E-07	1,19E-05	ENSMUSG(
Elob	17	compleme	855,3374	1,225597	2,338521	6,29E-09	4,6E-07	ENSMUSG(
Prss27	17	24038144.	0,782165	-3,2652	-9,61442	0,000983	0,015253	ENSMUSG(
Amdhd2	17	compleme	4,161883	1,226573	2,340105	8,71E-05	0,001944	ENSMUSG(
Atp6v0c	17	compleme	1,606665	-9,89745	-953,74	0,003887	0,048742	ENSMUSG(
Rnps1	17	24414675.	57,63903	-1,11751	-2,16972	1E-07	5,31E-06	ENSMUSG(
Dnase1l2	17	compleme	0,218396	1,966143	3,907221	0,003781	0,047703	ENSMUSG(
Tsc2	17	compleme	5,703975	-1,23815	-2,35896	6,3E-07	2,67E-05	ENSMUSG(
Slc9a3r2	17	compleme	1,987081	-1,98959	-3,97123	0,000145	0,003026	ENSMUSG(
Zfp598	17	24669752.	6,656554	-1,22033	-2,33001	7,08E-06	0,000221	ENSMUSG(
Rps2	17	24718116.	182,9283	-1,17572	-2,25906	6,49E-05	0,001506	ENSMUSG(
Msrb1	17	24736642.	6,503928	-1,20584	-2,30672	0,002533	0,033904	ENSMUSG(
Fahd1	17	compleme	9,220994	1,029244	2,040954	0,000402	0,007119	ENSMUSG(
Nubp2	17	compleme	34,2589	1,252847	2,383112	3,57E-08	2,18E-06	ENSMUSG(
BC003965	17	25184561.	1,139035	1,391002	2,622608	0,001205	0,018052	ENSMUSG(
Gnptg	17	compleme	6,702405	1,085643	2,122322	3,3E-05	0,000836	ENSMUSG(
Ube2i	17	compleme	17,24116	-1,07436	-2,10579	7,6E-06	0,000235	ENSMUSG(
Lmf1	17	25579174.	0,420362	1,584718	2,999492	0,000114	0,002473	ENSMUSG(
Narfl	17	25773776.	5,155778	1,139376	2,202857	2,69E-06	9,48E-05	ENSMUSG(
Haghl	17	compleme	1,434019	1,668235	3,178256	0,000269	0,005117	ENSMUSG(
Fam173a	17	compleme	4,02264	1,670799	3,183909	0,000477	0,008295	ENSMUSG(
Metrn	17	compleme	6,577588	1,199258	2,296215	0,001541	0,022264	ENSMUSG(
Fam195a	17	compleme	30,95466	1,129353	2,187607	2,55E-06	9,06E-05	ENSMUSG(
Arhgdig	17	compleme	0,097123	2,878239	7,352519	0,000804	0,012864	ENSMUSG(
Kifc5b	17	26917091.	1,854485	-1,88172	-3,68514	3,22E-05	0,000817	ENSMUSG(
Syngap1	17	26941253.	0,511001	-1,91867	-3,78074	0,000294	0,005493	ENSMUSG(
Zbtb9	17	26973167.	1,058997	-1,83975	-3,57948	0,000284	0,005341	ENSMUSG(
Itpr3	17	27057304.	23,68868	-1,64826	-3,13456	3,38E-14	8,37E-12	ENSMUSG(
Hmga1	17	27556620.	56,27439	-1,71936	-3,29291	0	0	ENSMUSG(
Rps10	17	compleme	810,6641	1,087367	2,124858	2,09E-07	1,03E-05	ENSMUSG(

Snrpc	17	27839974.	75,67	2,105953	4,304821	3,23E-10	3,22E-08	ENSMUSG(
Taf11	17	compleme	38,61232	3,033455	8,187683	0	0	ENSMUSG(
Ppard	17	28232754.	2,882643	-1,48659	-2,80227	2,17E-05	0,000579	ENSMUSG(
Brpf3	17	28801090.	0,982808	-1,63891	-3,11431	0,000234	0,004564	ENSMUSG(
Tbc1d22b	17	29549788.	1,867034	-1,52225	-2,8724	3,6E-05	0,000901	ENSMUSG(
Zfand3	17	30005087.	4,997095	-1,87143	-3,65895	7,94E-07	3,27E-05	ENSMUSG(
Glo1	17	compleme	229,5743	2,887981	7,402336	0	0	ENSMUSG(
Tmprss3	17	compleme	0,513776	-3,80117	-13,9401	0,001137	0,017223	ENSMUSG(
U2af1	17	compleme	15,60289	-1,26468	-2,40273	0,001121	0,017025	ENSMUSG(
Sik1	17	compleme	0,909096	-2,43554	-5,40965	2,38E-07	1,14E-05	ENSMUSG(
Pdxk-ps	17	compleme	3,348516	1,18609	2,275353	0,000413	0,007288	ENSMUSG(
Brd4	17	compleme	6,929016	-1,62759	-3,08996	1,99E-10	2,11E-08	ENSMUSG(
Wiz	17	compleme	4,748525	-1,69993	-3,24885	6,88E-09	5E-07	ENSMUSG(
Zfp414	17	33629090.	0,501859	-2,88108	-7,36704	0,000951	0,014809	ENSMUSG(
Hnrnpm	17	compleme	39,71757	-1,16321	-2,23955	0,000978	0,015179	ENSMUSG(
Angptl4	17	compleme	2,758489	1,516514	2,860988	6,11E-05	0,001431	ENSMUSG(
Kifc1	17	compleme	2,327322	-1,63659	-3,10931	3,73E-05	0,000929	ENSMUSG(
Tapbp	17	33915899.	27,3238	-1,11508	-2,16608	3,56E-08	2,17E-06	ENSMUSG(
Brd2	17	compleme	69,111	-1,22647	-2,33994	7,51E-09	5,41E-07	ENSMUSG(
Gpsm3	17	34589806.	1,206141	-2,2943	-4,90517	0,000754	0,012164	ENSMUSG(
Pbx2	17	34591266.	4,990582	-1,39113	-2,62284	4,21E-08	2,51E-06	ENSMUSG(
Agpat1	17	34604262.	5,752299	-1,77933	-3,43267	6,18E-11	7,3E-09	ENSMUSG(
Atf6b	17	34647146.	4,600024	-2,54516	-5,83674	1,51E-12	2,49E-10	ENSMUSG(
Tnxa	17	34771395.	6,961694	-1,02894	-2,04052	6,41E-05	0,001492	ENSMUSG(
Hspa1b	17	compleme	85,9538	-1,04175	-2,05873	9,77E-05	0,00215	ENSMUSG(
Hspa1a	17	compleme	10,00523	-1,3633	-2,57273	8,05E-06	0,000247	ENSMUSG(
Vars	17	35000987.	9,032209	-1,65827	-3,15637	7,26E-08	4,02E-06	ENSMUSG(
Apom	17	compleme	1,984792	2,373091	5,180498	1,83E-05	0,000504	ENSMUSG(
Bag6	17	35135178.	26,38133	-1,46576	-2,76208	2,08E-13	4,24E-11	ENSMUSG(
Prrc2a	17	compleme	13,84866	-2,53607	-5,80007	0	0	ENSMUSG(
Gtf2h4	17	compleme	2,965693	-1,2269	-2,34063	0,000249	0,004802	ENSMUSG(
Ier3	17	35821684.	30,13588	-1,41059	-2,65846	3,62E-05	0,000906	ENSMUSG(
Flot1	17	35823230.	26,92134	-1,1399	-2,20365	1,02E-07	5,39E-06	ENSMUSG(
2310061I0	17	compleme	0,881082	-1,84023	-3,58068	0,001297	0,019185	ENSMUSG(
Ppp1r10	17	35916434.	4,925939	-1,84923	-3,60307	6,27E-12	8,93E-10	ENSMUSG(
Abcf1	17	compleme	21,11692	-1,29428	-2,45255	1,23E-09	1,07E-07	ENSMUSG(
Prr3	17	compleme	1,719385	-2,0568	-4,16061	3,19E-08	1,96E-06	ENSMUSG(
Gm10499	17	compleme	0,617155	-3,29987	-9,84825	0,001725	0,024491	ENSMUSG(
Gm5682	17	compleme	17,07416	-1,78073	-3,43599	0,002077	0,028592	ENSMUSG(
Ppp1r11	17	compleme	8,989288	-1,46864	-2,76761	1,53E-08	1,02E-06	ENSMUSG(
Znrd1	17	compleme	7,690911	1,19895	2,295726	0,000475	0,008256	ENSMUSG(
Rn18s-rs5	17	compleme	5,19403	3,528918	11,54277	2,46E-08	1,55E-06	ENSMUSG(
Cdc5l	17	compleme	130,3985	2,649635	6,275084	3,66E-15	1,09E-12	ENSMUSG(
Aars2	17	45506841.	40,2104	2,982392	7,902956	0	0	ENSMUSG(
Nfkbie	17	45555716.	4,025845	2,714167	6,562145	1,54E-09	1,31E-07	ENSMUSG(
Slc35b2	17	45563964.	148,1234	3,190473	9,129102	6,66E-16	2,32E-13	ENSMUSG(
Slc29a1	17	compleme	11,57058	1,467591	2,765598	7,55E-05	0,001721	ENSMUSG(
Tmem63b	17	compleme	9,42858	1,800885	3,484339	3,87E-07	1,74E-05	ENSMUSG(

Mrpl14	17	45686322.	315,9777	3,557333	11,77237	0	0	ENSMUSG(
Tjap1	17	compleme	1,84359	-1,37822	-2,59948	0,00103	0,015864	ENSMUSG(
Zfp318	17	46383731.	1,431301	-1,6124	-3,05759	1,31E-06	5,06E-05	ENSMUSG(
Ttbk1	17	compleme	0,458717	-1,90435	-3,74339	0,00024	0,004656	ENSMUSG(
Dnph1	17	46496789.	9,158047	1,281571	2,431035	0,000622	0,01041	ENSMUSG(
Mrpl2	17	46646229.	23,11337	-1,41276	-2,66247	1,62E-07	8,22E-06	ENSMUSG(
Tbcc	17	46890684.	11,83428	1,109636	2,157912	0,000194	0,003854	ENSMUSG(
Trerf1	17	47140875.	1,431592	-2,04863	-4,13712	1,78E-10	1,91E-08	ENSMUSG(
Gm5814	17	47410363.	8,176335	-2,14433	-4,42086	0,000503	0,008665	ENSMUSG(
Bysl	17	compleme	2,163425	-2,07141	-4,20297	1,21E-06	4,74E-05	ENSMUSG(
Foxp4	17	compleme	0,493616	-1,95009	-3,86398	0,001076	0,016476	ENSMUSG(
St6gal2	17	55445382.	0,098012	4,005791	16,06436	1,53E-05	0,000431	ENSMUSG(
Chaf1a	17	56040416.	7,877644	-1,05865	-2,08299	3,52E-05	0,000884	ENSMUSG(
Sema6b	17	compleme	3,516784	-1,66154	-3,16355	2,97E-05	0,00076	ENSMUSG(
Dpp9	17	compleme	13,11456	-1,40825	-2,65415	2,28E-09	1,82E-07	ENSMUSG(
Kdm4b	17	56326062.	1,202564	-1,09122	-2,13055	0,002849	0,037411	ENSMUSG(
Ptprs	17	compleme	5,435316	-1,33813	-2,52823	2,51E-07	1,19E-05	ENSMUSG(
Safb2	17	compleme	1,050089	-2,83399	-7,13043	2,01E-08	1,29E-06	ENSMUSG(
Safb	17	56584825.	4,776269	-2,45303	-5,47565	2,71E-09	2,13E-07	ENSMUSG(
Ranbp3	17	56673225.	5,793668	-1,2468	-2,37314	3,34E-06	0,000115	ENSMUSG(
Rfx2	17	compleme	0,555129	-2,16259	-4,47718	0,00065	0,0108	ENSMUSG(
Mllt1	17	compleme	2,842566	-1,50105	-2,83049	7,9E-06	0,000243	ENSMUSG(
Gtf2f1	17	compleme	31,0001	-1,07662	-2,1091	2,16E-06	7,85E-05	ENSMUSG(
Khsrp	17	compleme	17,20436	-1,26645	-2,4057	2,49E-07	1,19E-05	ENSMUSG(
C3	17	compleme	16,30042	-2,95868	-7,7741	0	0	ENSMUSG(
Trip10	17	57249451.	17,36572	-1,31699	-2,49147	5,68E-08	3,26E-06	ENSMUSG(
Nudt12	17	compleme	4,219742	1,288632	2,442963	4,58E-07	2,02E-05	ENSMUSG(
Man2a1	17	64600736.	6,662214	-1,05691	-2,08048	4,43E-06	0,000147	ENSMUSG(
Ptprm	17	compleme	4,348961	-1,27721	-2,4237	0,000219	0,004313	ENSMUSG(
Lama1	17	67697265.	0,400935	-1,51098	-2,85004	0,002203	0,030131	ENSMUSG(
Arhgap28	17	compleme	0,308858	2,019217	4,053637	0,001809	0,025462	ENSMUSG(
Myl12b	17	compleme	254,1601	1,069382	2,098534	5,63E-07	2,42E-05	ENSMUSG(
Emilin2	17	compleme	3,824543	-2,70988	-6,54269	9,33E-06	0,00028	ENSMUSG(
Lbh	17	72918305.	4,325537	2,89479	7,437356	5,62E-14	1,31E-11	ENSMUSG(
Galnt14	17	compleme	1,930946	-1,90202	-3,73735	2,48E-06	8,85E-05	ENSMUSG(
Fam98a	17	compleme	14,15776	-1,78034	-3,43507	5,64E-08	3,23E-06	ENSMUSG(
Vit	17	78508063.	4,221318	1,427095	2,689047	1,07E-05	0,000316	ENSMUSG(
Cdc42ep3	17	compleme	35,74999	-1,07775	-2,11074	7,88E-07	3,25E-05	ENSMUSG(
Rmdn2	17	79614900.	2,734202	1,271503	2,414129	0,001275	0,018906	ENSMUSG(
Pkdcc	17	83215292.	4,719504	1,373549	2,591071	4,7E-06	0,000155	ENSMUSG(
Zfp36l2	17	compleme	2,996399	-1,30147	-2,46481	9,34E-05	0,002065	ENSMUSG(
Epas1	17	86753907.	15,29648	-1,16777	-2,24663	1,69E-06	6,32E-05	ENSMUSG(
Kcnk12	17	compleme	0,255989	2,676362	6,392419	0,000676	0,011124	ENSMUSG(
Epc1	18	compleme	3,430013	-1,16184	-2,23743	3,54E-05	0,000888	ENSMUSG(
Greb1l	18	10325177.	0,620723	-2,07986	-4,22765	3,67E-06	0,000125	ENSMUSG(
Gata6	18	11052510.	3,261212	-1,91613	-3,77408	2,25E-07	1,09E-05	ENSMUSG(
Npc1	18	compleme	3,242013	-1,5084	-2,84494	1,74E-10	1,88E-08	ENSMUSG(
Ankrd29	18	compleme	4,473142	-1,64163	-3,12019	1,23E-05	0,000356	ENSMUSG(

Lama3	18	12333819.	2,337246	-1,86651	-3,64649	8,28E-09	5,89E-07	ENSMUSG(
Kctd1	18	compleme	4,941906	-1,06596	-2,09357	6,23E-05	0,001454	ENSMUSG(
Cdh2	18	compleme	13,755	-2,37571	-5,1899	1,11E-16	4,39E-14	ENSMUSG(
Dsg2	18	20558074.	2,048976	-5,00314	-32,0698	1,29E-11	1,76E-09	ENSMUSG(
B4galt6	18	compleme	1,615128	-1,73604	-3,33119	1,19E-06	4,66E-05	ENSMUSG(
Rnf125	18	20944625.	1,009871	-2,24501	-4,7404	0,001864	0,026142	ENSMUSG(
Dtna	18	23415415.	1,068347	-4,43939	-21,6965	3,52E-07	1,61E-05	ENSMUSG(
Mapre2	18	23752333.	2,799248	-1,63537	-3,10667	4,77E-10	4,56E-08	ENSMUSG(
Mocos	18	24653691.	4,882561	-1,19057	-2,28244	0,000602	0,010124	ENSMUSG(
Fhod3	18	24709445.	2,625146	-4,77938	-27,4623	7,33E-13	1,3E-10	ENSMUSG(
Tpgs2	18	compleme	6,758784	-1,28978	-2,44491	0,000339	0,006176	ENSMUSG(
Gm7729	18	27598644.	108,617	-2,88738	-7,39926	0	0	ENSMUSG(
Sap130	18	31634371.	2,646326	-1,72577	-3,30757	2,89E-06	0,000101	ENSMUSG(
Ercc3	18	32240300.	18,65317	-1,30564	-2,47194	3,62E-09	2,77E-07	ENSMUSG(
Camk4	18	32939041.	0,4485	-1,99863	-3,99619	3,74E-05	0,000931	ENSMUSG(
Stard4	18	compleme	0,721709	1,365139	2,576011	0,001271	0,018867	ENSMUSG(
Epb41l4a	18	compleme	0,401367	5,109168	34,51538	4,64E-09	3,49E-07	ENSMUSG(
Kdm3b	18	34777008.	16,00602	-1,00986	-2,01372	4,04E-07	1,81E-05	ENSMUSG(
Reep2	18	34840589.	1,690356	-2,94593	-7,70571	0,000332	0,006078	ENSMUSG(
Tmem173	18	compleme	5,134214	-1,66449	-3,17003	4,93E-08	2,87E-06	ENSMUSG(
Cxxc5	18	35829818.	18,61181	1,636995	3,110173	2,26E-11	2,92E-09	ENSMUSG(
Slc35a4	18	36679215.	3,326299	-2,1035	-4,29751	3,01E-10	3,02E-08	ENSMUSG(
Diaph1	18	compleme	9,641782	-1,07676	-2,10929	2,1E-05	0,000563	ENSMUSG(
Rell2	18	37955079.	2,463406	-1,58404	-2,99809	0,000246	0,004747	ENSMUSG(
Arap3	18	compleme	4,046293	-1,36869	-2,58236	3,86E-07	1,74E-05	ENSMUSG(
Spry4	18	compleme	1,796789	-2,22159	-4,66408	7,55E-07	3,13E-05	ENSMUSG(
Arhgap26	18	38601534.	0,387195	-1,74127	-3,34329	4,22E-05	0,001031	ENSMUSG(
Yipf5	18	compleme	45,04927	1,069236	2,098322	8,32E-08	4,53E-06	ENSMUSG(
Prelid2	18	compleme	26,58885	1,43125	2,696802	2,21E-07	1,08E-05	ENSMUSG(
Sh3rf2	18	42053667.	2,092027	-1,86917	-3,65322	9,07E-06	0,000273	ENSMUSG(
Mcc	18	compleme	0,782115	-2,24862	-4,7523	1,31E-06	5,09E-05	ENSMUSG(
Trim36	18	compleme	1,458421	-2,51163	-5,70264	1,73E-06	6,46E-05	ENSMUSG(
Aldh7a1	18	compleme	5,819009	-1,36796	-2,58105	1,28E-06	4,98E-05	ENSMUSG(
Fbn2	18	compleme	7,95793	-1,28163	-2,43114	1,48E-09	1,26E-07	ENSMUSG(
Slc27a6	18	58556257.	0,577369	4,878318	29,41169	2,56E-07	1,21E-05	ENSMUSG(
Adamts19	18	58836764.	6,537871	6,518312	91,66579	0	0	ENSMUSG(
Chsy3	18	59175401.	2,235104	-2,20051	-4,59643	0,000148	0,00308	ENSMUSG(
Smim3	18	compleme	19,43437	1,403704	2,6458	7,72E-06	0,000238	ENSMUSG(
Synpo	18	compleme	1,349324	-2,99849	-7,9916	3,81E-13	7,27E-11	ENSMUSG(
Rps14	18	60774510.	892,1659	1,056408	2,079748	6,76E-07	2,84E-05	ENSMUSG(
Tcof1	18	compleme	4,522313	-1,84623	-3,59559	3,61E-10	3,55E-08	ENSMUSG(
Arsi	18	60911780.	0,299508	2,558379	5,890455	0,000196	0,003884	ENSMUSG(
Ppargc1b	18	compleme	0,704268	-1,93123	-3,81381	0,000389	0,006937	ENSMUSG(
Arhgef37	18	compleme	1,910571	1,572877	2,974974	0,000163	0,003346	ENSMUSG(
Ablim3	18	compleme	11,23662	-1,46554	-2,76167	7,27E-11	8,39E-09	ENSMUSG(
Apcdd1	18	62922327.	0,911898	1,742488	3,346117	0,00251	0,033614	ENSMUSG(
Wdr7	18	63708695.	2,8125	-1,12782	-2,18528	1,71E-05	0,000476	ENSMUSG(
Nedd4l	18	64887756.	4,708681	-3,01606	-8,08957	0	0	ENSMUSG(

Zfp532	18	65580230.	4,418646	-1,28694	-2,44009	4,73E-08	2,78E-06	ENSMUSG(
Ccbe1	18	compleme	0,53542	-1,7714	-3,41385	3,81E-06	0,000129	ENSMUSG(
Gnal	18	67088336.	0,84647	-2,17137	-4,50451	0,000273	0,005181	ENSMUSG(
Rab27b	18	compleme	2,690541	-1,51304	-2,85411	0,000726	0,011804	ENSMUSG(
Ctif	18	compleme	2,767875	-2,37874	-5,20084	2,44E-15	7,45E-13	ENSMUSG(
Hdhd2	18	76930126.	4,302577	-1,13433	-2,19517	2,66E-05	0,000693	ENSMUSG(
8030462N	18	compleme	6,693712	-1,42354	-2,68243	5,53E-07	2,38E-05	ENSMUSG(
Adnp2	18	compleme	6,207528	-1,3665	-2,57844	1,16E-07	6,05E-06	ENSMUSG(
Ctdp1	18	compleme	3,435673	-1,12692	-2,18391	0,000238	0,00462	ENSMUSG(
Zfp516	18	82910879.	1,418452	-1,57849	-2,98658	4,11E-05	0,001009	ENSMUSG(
Tshz1	18	compleme	1,956418	-1,92038	-3,78523	9,47E-08	5,09E-06	ENSMUSG(
Cndp2	18	compleme	11,41711	-1,29408	-2,4522	8,69E-06	0,000263	ENSMUSG(
Neto1	18	86394952.	0,80405	-4,77398	-27,3596	3,96E-06	0,000134	ENSMUSG(
Mrpl21	19	3282901..3	38,12586	1,226794	2,340462	1,09E-08	7,62E-07	ENSMUSG(
Ndufv1	19	compleme	88,68321	1,074503	2,105997	2,62E-07	1,24E-05	ENSMUSG(
Gstp1	19	compleme	629,6832	1,121538	2,175787	2,9E-08	1,79E-06	ENSMUSG(
Gstp2	19	compleme	108,5879	1,211493	2,315772	6,42E-09	4,68E-07	ENSMUSG(
Gstp3	19	compleme	198,308	1,102071	2,146627	1,6E-08	1,06E-06	ENSMUSG(
Ctsf	19	4855129..4	7,729768	7,918779	241,9859	0	0	ENSMUSG(
Actn3	19	compleme	7,833395	-1,94475	-3,84971	1,18E-12	2,02E-10	ENSMUSG(
Zdhhc24	19	4878668..4	3,194358	1,683749	3,212617	1,56E-06	5,9E-05	ENSMUSG(
Peli3	19	compleme	3,707255	1,479979	2,789446	9,37E-06	0,000281	ENSMUSG(
Mrpl11	19	4962306..4	17,34328	1,376667	2,596678	1,67E-09	1,4E-07	ENSMUSG(
B4gat1	19	5038826..5	22,21855	1,181124	2,267533	4,76E-06	0,000156	ENSMUSG(
Brms1	19	5041404..5	61,09482	1,476874	2,78345	6,15E-12	8,79E-10	ENSMUSG(
Yif1a	19	5088538..5	43,10514	1,251795	2,381375	4,11E-09	3,11E-07	ENSMUSG(
Klc2	19	compleme	7,270588	-1,47789	-2,7854	3,21E-08	1,97E-06	ENSMUSG(
Banf1	19	compleme	41,57889	-2,35602	-5,11955	3,61E-10	3,55E-08	ENSMUSG(
Eif1ad	19	5366813..5	7,398743	1,139035	2,202337	3,02E-06	0,000105	ENSMUSG(
Drap1	19	compleme	138,0379	1,04659	2,065641	5,89E-06	0,000188	ENSMUSG(
Rela	19	5637483..5	8,320041	-2,04999	-4,14103	4,09E-10	3,96E-08	ENSMUSG(
Sipa1	19	compleme	8,623339	-1,75499	-3,37524	5,94E-13	1,09E-10	ENSMUSG(
Pcnx3	19	compleme	5,436674	-1,24934	-2,37732	5,7E-07	2,44E-05	ENSMUSG(
Map3k11	19	5689131..5	6,179288	-1,06761	-2,09597	3,24E-05	0,000821	ENSMUSG(
Ltbp3	19	5740904..5	9,273294	-1,13095	-2,19002	4,56E-06	0,000151	ENSMUSG(
Dpf2	19	compleme	8,039344	-1,02325	-2,0325	3,97E-06	0,000134	ENSMUSG(
Cdc42ep2	19	compleme	8,059338	-1,34084	-2,53298	6,75E-06	0,000211	ENSMUSG(
Vps51	19	compleme	20,09409	1,421467	2,678578	2,57E-12	4,01E-10	ENSMUSG(
Sf1	19	6363690..6	11,2261	-1,86391	-3,63992	2,06E-12	3,28E-10	ENSMUSG(
Ccdc88b	19	compleme	0,256964	1,529782	2,887421	0,003926	0,049142	ENSMUSG(
Trmt112	19	6909698..6	50,70826	1,191046	2,283182	5,28E-08	3,05E-06	ENSMUSG(
Esrra	19	compleme	24,99732	1,07974	2,113655	1,17E-06	4,6E-05	ENSMUSG(
Bad	19	6941861..6	11,52514	1,339619	2,530845	6,62E-08	3,73E-06	ENSMUSG(
Ppp1r14b	19	6975048..6	371,0382	1,234399	2,352833	2,55E-08	1,6E-06	ENSMUSG(
Nudt22	19	compleme	8,733824	1,576437	2,982323	1,45E-06	5,53E-05	ENSMUSG(
Macrod1	19	7056768..7	26,9229	1,864093	3,640389	4,98E-12	7,3E-10	ENSMUSG(
Cox8a	19	compleme	276,8851	1,045347	2,063862	1,68E-07	8,48E-06	ENSMUSG(
Mark2	19	compleme	2,041561	-1,43739	-2,7083	5,06E-06	0,000165	ENSMUSG(

Atl3	19	7494040..7	25,45678	1,205789	2,306634	4,83E-08	2,84E-06	ENSMUSG(
Hrasls5	19	7612541..7	5,097345	2,427828	5,380827	7,28E-09	5,26E-07	ENSMUSG(
Wdr74	19	8735827..8	91,36461	1,213213	2,318534	7,46E-10	6,83E-08	ENSMUSG(
Bscl2	19	8837467..8	3,764857	1,002835	2,003934	0,000432	0,007587	ENSMUSG(
1810009A1	19	8888853..8	29,27232	-1,03558	-2,04993	2,57E-05	0,000671	ENSMUSG(
Rom1	19	compleme	4,833611	1,9722	3,92366	1,93E-07	9,63E-06	ENSMUSG(
Incenp	19	compleme	10,32808	-1,30994	-2,47931	3,68E-05	0,000919	ENSMUSG(
Fth1	19	9982703..9	870,0416	-2,2785	-4,85174	7,74E-13	1,36E-10	ENSMUSG(
Fads2	19	compleme	36,6211	1,021474	2,029992	2,08E-05	0,00056	ENSMUSG(
Myrf	19	compleme	0,133185	-2,53445	-5,79354	0,000779	0,01252	ENSMUSG(
Cpsf7	19	10525244.	12,16291	-1,08667	-2,12384	7,76E-05	0,001764	ENSMUSG(
Cyb561a3	19	10577454.	13,10955	1,011017	2,015331	0,000108	0,002358	ENSMUSG(
Tmem132a	19	compleme	21,08496	-1,08792	-2,12568	1,24E-06	4,83E-05	ENSMUSG(
Patl1	19	11912399.	6,615416	-1,64432	-3,12601	3,75E-09	2,86E-07	ENSMUSG(
Fam111a	19	12545740.	4,938912	1,629283	3,093593	8,69E-10	7,86E-08	ENSMUSG(
Tle4	19	compleme	3,128018	-1,44665	-2,72574	9,34E-07	3,76E-05	ENSMUSG(
Psat1	19	compleme	6,487552	-1,78003	-3,43432	1,82E-11	2,39E-09	ENSMUSG(
Pcsk5	19	compleme	1,111977	-1,52747	-2,8828	0,000176	0,003563	ENSMUSG(
Rorb	19	compleme	0,173297	2,773571	6,837982	4,86E-07	2,13E-05	ENSMUSG(
Dock8	19	24999529.	1,246409	-2,08074	-4,23024	1,6E-07	8,15E-06	ENSMUSG(
Kank1	19	25236975.	14,5914	-1,54453	-2,91709	8,16E-12	1,14E-09	ENSMUSG(
Vldlr	19	27216484.	0,416121	-4,40324	-21,1596	9,56E-08	5,12E-06	ENSMUSG(
Ermp1	19	compleme	1,402604	-1,58133	-2,99246	0,000124	0,00265	ENSMUSG(
Dkk1	19	compleme	147,4533	1,35873	2,564593	4,59E-08	2,72E-06	ENSMUSG(
Prkg1	19	compleme	0,79209	4,303939	19,75216	3,26E-12	4,98E-10	ENSMUSG(
Acta2	19	compleme	143,493	-1,00893	-2,01242	1,59E-05	0,000447	ENSMUSG(
Ch25h	19	compleme	8,645936	4,529814	23,09989	0	0	ENSMUSG(
Ifit1	19	34640871.	24,71687	1,724201	3,303971	1,11E-07	5,84E-06	ENSMUSG(
Pcgf5	19	36379067.	5,615168	-1,49885	-2,82617	0,001145	0,017312	ENSMUSG(
Ppp1r3c	19	compleme	3,455544	1,88916	3,704196	6,71E-08	3,77E-06	ENSMUSG(
Hhex	19	37434810.	4,394917	-1,57613	-2,98168	0,000653	0,010821	ENSMUSG(
Fra10ac1	19	compleme	6,008177	-1,22177	-2,33233	0,00066	0,01092	ENSMUSG(
Pdlim1	19	compleme	2,689802	-1,36994	-2,5846	2,31E-05	0,000611	ENSMUSG(
Sorbs1	19	compleme	0,460661	-3,14464	-8,84364	0,000196	0,003884	ENSMUSG(
Lcor	19	41482645.	1,700152	-1,10946	-2,15765	0,000246	0,004754	ENSMUSG(
AI606181	19	41593363.	0,277959	-3,43069	-10,783	0,002042	0,02818	ENSMUSG(
Hif1an	19	44562850.	1,947121	-1,13125	-2,19048	0,00039	0,006942	ENSMUSG(
Twnk	19	45006558.	3,156602	-1,29239	-2,44933	0,000149	0,003101	ENSMUSG(
Kazald1	19	45075241.	2,956949	1,45019	2,732441	0,000397	0,00704	ENSMUSG(
Npm3	19	compleme	11,64665	1,40933	2,656137	5,45E-06	0,000176	ENSMUSG(
9130011E1	19	compleme	3,410122	-1,23954	-2,36123	0,000496	0,008572	ENSMUSG(
Ldb1	19	compleme	6,606441	-1,60686	-3,04589	1,48E-08	9,92E-07	ENSMUSG(
Pprc1	19	46044886.	2,759336	-1,09853	-2,14136	0,000323	0,005944	ENSMUSG(
Nolc1	19	46075863.	27,67354	-1,57429	-2,97788	2,61E-13	5,24E-11	ENSMUSG(
Gbf1	19	46152509.	7,028864	-1,86256	-3,63652	8,55E-15	2,46E-12	ENSMUSG(
Nfkb2	19	46304737.	8,628191	-1,68532	-3,21611	1,47E-11	1,97E-09	ENSMUSG(
Sufu	19	46396896.	0,427141	-1,73874	-3,33743	0,001244	0,018554	ENSMUSG(
Trim8	19	46501648.	7,481763	-1,54481	-2,91765	1,1E-09	9,7E-08	ENSMUSG(

Wbp1l	19	46599084.	1,662103	-1,06825	-2,09689	0,003555	0,045282	ENSMUSG(
Cnnm2	19	46761596.	0,981676	-1,44054	-2,71422	0,00139	0,020351	ENSMUSG(
Stn1	19	compleme	5,640136	1,059705	2,084506	0,000307	0,005714	ENSMUSG(
Cfap43	19	compleme	0,669129	-1,90828	-3,75362	2,09E-07	1,03E-05	ENSMUSG(
Rpl13a-ps1	19	compleme	471,1265	1,092869	2,132977	1,43E-07	7,35E-06	ENSMUSG(
Dusp5	19	53529109.	3,149698	-1,55393	-2,93617	8,64E-05	0,001928	ENSMUSG(
Pdcd4	19	53892231.	13,7129	-1,14712	-2,21472	9,61E-07	3,86E-05	ENSMUSG(
Nhlrc2	19	56548261.	3,759703	-1,21625	-2,32342	2,36E-05	0,000625	ENSMUSG(
Atrnl1	19	57611034.	0,413209	-5,40386	-42,3374	3E-05	0,000766	ENSMUSG(
Hspa12a	19	compleme	1,309719	-1,83695	-3,57254	2,49E-06	8,88E-05	ENSMUSG(
Emx2	19	59458372.	0,569818	-2,44839	-5,45807	0,000154	0,003189	ENSMUSG(
Eif3a	19	compleme	77,39065	-1,24878	-2,3764	2,97E-07	1,38E-05	ENSMUSG(
Nudt11	X	6047453..6	0,218851	2,40805	5,307565	0,001791	0,025245	ENSMUSG(
Gm36995	X	compleme	0,114557	7,240216	151,1897	0,001943	0,027064	ENSMUSG(
Prickle3	X	7657260..7	0,357208	-2,35136	-5,10304	0,000413	0,007293	ENSMUSG(
Plp2	X	compleme	20,88816	-1,48192	-2,7932	1,34E-09	1,15E-07	ENSMUSG(
Tfe3	X	7762560..7	1,25524	-1,43887	-2,71109	1E-05	0,000298	ENSMUSG(
Gripap1	X	7789765..7	5,965906	-1,06246	-2,08849	4,56E-06	0,000151	ENSMUSG(
Otud5	X	7841364..7	4,805987	-1,14369	-2,20945	0,000125	0,002664	ENSMUSG(
Tbc1d25	X	compleme	2,075002	-1,53351	-2,8949	4,04E-05	0,000995	ENSMUSG(
Dynlt3	X	compleme	13,6171	1,282089	2,431909	2,86E-07	1,33E-05	ENSMUSG(
Sytl5	X	9885622..9	2,771204	4,582045	23,95152	0	0	ENSMUSG(
Bcor	X	compleme	3,944235	-1,36386	-2,57372	2,6E-07	1,23E-05	ENSMUSG(
Med14	X	compleme	2,686508	-2,12039	-4,34812	1,67E-15	5,27E-13	ENSMUSG(
Maoa	X	16619698.	0,219224	2,383712	5,218778	0,000713	0,01162	ENSMUSG(
Ndp	X	compleme	0,576401	3,18511	9,095227	2,84E-05	0,000731	ENSMUSG(
Jade3	X	20425688.	1,108075	-1,60367	-3,03915	2,93E-05	0,000752	ENSMUSG(
Uba1	X	20658326.	29,53255	-1,23546	-2,35456	3,16E-09	2,45E-07	ENSMUSG(
Cdk16	X	20687954.	16,85426	-1,19239	-2,2853	1,17E-08	8,11E-07	ENSMUSG(
Usp11	X	20703906.	5,749749	-1,87233	-3,66123	2,9E-12	4,48E-10	ENSMUSG(
Timp1	X	20870166.	34,26911	-1,0173	-2,02413	2,07E-05	0,000558	ENSMUSG(
Gm5124	X	21360865.	18,51522	-1,50792	-2,844	8,84E-10	7,99E-08	ENSMUSG(
Klhl13	X	compleme	4,37565	1,301691	2,465177	2,78E-05	0,000719	ENSMUSG(
Gm4907	X	23882553.	4,636423	1,032534	2,045614	0,002547	0,034039	ENSMUSG(
Lonrf3	X	36328353.	1,505559	-1,4624	-2,75567	0,002818	0,037121	ENSMUSG(
Slc25a43	X	36743659.	9,830401	-1,25167	-2,38117	0,000248	0,004789	ENSMUSG(
Slc25a5	X	36795651.	151,245	-2,2823	-4,86454	0	0	ENSMUSG(
Ube2a	X	36873900.	20,9056	-1,89798	-3,72692	2,34E-13	4,76E-11	ENSMUSG(
Nkrf	X	compleme	8,041527	-1,87332	-3,66374	5,24E-13	9,7E-11	ENSMUSG(
Sept6	X	compleme	2,586219	-1,29885	-2,46034	4,92E-06	0,000161	ENSMUSG(
Upf3b	X	compleme	3,161054	-3,50136	-11,3244	0	0	ENSMUSG(
Nkap	X	37126795.	4,482139	-1,98904	-3,96972	7,21E-11	8,35E-09	ENSMUSG(
Rnf113a1	X	37191245.	8,909831	-1,19189	-2,28453	0,002657	0,035243	ENSMUSG(
Zbtb33	X	38189793.	7,00929	-1,75132	-3,36665	5,15E-12	7,51E-10	ENSMUSG(
Lamp2	X	compleme	12,57896	-1,69081	-3,22837	4,89E-13	9,1E-11	ENSMUSG(
Bcorl1	X	48341358.	0,098267	-2,71737	-6,57671	0,002688	0,035583	ENSMUSG(
Elf4	X	compleme	1,566688	-1,53346	-2,89479	2,11E-06	7,67E-05	ENSMUSG(
Aifm1	X	compleme	9,514309	-1,66938	-3,18077	1,36E-08	9,3E-07	ENSMUSG(

Stk26	X	50841047.	2,054348	1,767525	3,404693	5,07E-08	2,95E-06	ENSMUSG(
Hs6st2	X	compleme	2,751556	-5,39337	-42,0305	1,56E-10	1,71E-08	ENSMUSG(
Fam122b	X	compleme	2,507257	1,438464	2,710321	5,33E-05	0,001262	ENSMUSG(
Mospd1	X	compleme	9,164101	1,193956	2,287792	3,6E-06	0,000122	ENSMUSG(
Fgf13	X	compleme	0,980103	1,458769	2,748738	0,001658	0,023703	ENSMUSG(
Fmr1	X	68678541.	4,833985	-1,11899	-2,17195	6,66E-06	0,000209	ENSMUSG(
Gabrq	X	72825178.	0,322191	-4,12802	-17,4846	0,000252	0,004847	ENSMUSG(
Dusp9	X	73639419.	4,379298	1,809621	3,505501	8,83E-09	6,25E-07	ENSMUSG(
Slc6a8	X	73673150.	4,678281	-1,4738	-2,77752	1,22E-08	8,39E-07	ENSMUSG(
Hcfc1	X	compleme	3,937253	-1,5861	-3,00236	3,4E-06	0,000117	ENSMUSG(
Atp6ap1	X	74297097.	8,962697	-1,92468	-3,79654	4,34E-14	1,05E-11	ENSMUSG(
Gdi1	X	74304998.	13,04442	-1,85435	-3,61589	0	0	ENSMUSG(
Slc10a3	X	compleme	2,778164	-1,20114	-2,29921	0,000734	0,011891	ENSMUSG(
G6pdx	X	compleme	4,707236	-1,27998	-2,42835	9,61E-05	0,00212	ENSMUSG(
Cmc4	X	compleme	6,969333	1,03445	2,048333	0,002729	0,03608	ENSMUSG(
Vbp1	X	75514299.	25,53123	1,029134	2,040799	4,28E-06	0,000143	ENSMUSG(
Rab39b	X	compleme	0,305217	2,225327	4,676169	0,000964	0,014989	ENSMUSG(
Tmem47	X	81070698.	2,643091	2,829514	7,108345	4,37E-09	3,29E-07	ENSMUSG(
Dmd	X	82948870.	0,11221	-1,46591	-2,76238	0,00327	0,042185	ENSMUSG(
Tab3	X	85574022.	2,085471	-1,41402	-2,6648	1,2E-06	4,72E-05	ENSMUSG(
Pcyt1b	X	93654863.	1,07331	-3,42729	-10,7577	3,02E-05	0,00077	ENSMUSG(
Maged1	X	compleme	10,10083	-1,44303	-2,71892	2,84E-09	2,23E-07	ENSMUSG(
Msn	X	96096042.	62,11094	-1,17629	-2,25995	1,62E-08	1,07E-06	ENSMUSG(
Ar	X	98148769.	0,505173	-1,3042	-2,46946	0,001206	0,018061	ENSMUSG(
Med12	X	101274030	1,784918	-1,14808	-2,21619	0,00015	0,003112	ENSMUSG(
Nhsl2	X	101849385	0,094552	-3,25734	-9,56219	0,000107	0,002336	ENSMUSG(
Slc16a2	X	compleme	3,25277	-1,50158	-2,83152	5,14E-07	2,25E-05	ENSMUSG(
Cox7b	X	106015700	78,54963	1,045506	2,06409	4,04E-07	1,81E-05	ENSMUSG(
Pou3f4	X	110814280	0,143165	4,061058	16,69169	0,000754	0,012164	ENSMUSG(
Hdx	X	compleme	0,225094	3,783652	13,77187	6,61E-08	3,73E-06	ENSMUSG(
Klhl4	X	114474333	0,422356	2,651833	6,284651	0,00012	0,002567	ENSMUSG(
Tceal8	X	compleme	7,893055	1,102919	2,147889	8,66E-06	0,000262	ENSMUSG(
D330045A1	X	139480367	0,084109	3,029162	8,163356	0,002823	0,037181	ENSMUSG(
Chrdl1	X	compleme	0,125655	-4,58516	-24,0033	0,003628	0,046058	ENSMUSG(
Pak3	X	143518591	0,114861	-2,78575	-6,89595	0,000545	0,009286	ENSMUSG(
Tro	X	compleme	0,385121	2,273445	4,834762	4,11E-05	0,001009	ENSMUSG(
Fgd1	X	151046150	1,832784	-2,01724	-4,04807	7,85E-08	4,32E-06	ENSMUSG(
Phf8	X	151520672	1,260071	-1,07987	-2,11384	0,000361	0,006518	ENSMUSG(
Huwe1	X	151800807	7,385431	-1,6718	-3,18611	2,23E-11	2,88E-09	ENSMUSG(
Smc1a	X	152016428	14,37948	-2,24852	-4,75196	3,33E-16	1,24E-13	ENSMUSG(
Gm15266	X	compleme	73,35397	1,11411	2,164614	1,01E-05	0,000299	ENSMUSG(
Tspyl2	X	compleme	4,37301	-1,07131	-2,10134	0,000275	0,00522	ENSMUSG(
Shroom2	X	compleme	0,106559	-2,79961	-6,96252	0,003511	0,044816	ENSMUSG(
Mageh1	X	compleme	3,772044	2,776975	6,854138	3,09E-09	2,4E-07	ENSMUSG(
Ubqln2	X	153498227	3,332227	-1,07343	-2,10444	0,001435	0,020922	ENSMUSG(
2210013O2	X	153723590	5,290516	-1,46724	-2,76493	3,47E-06	0,000119	ENSMUSG(
Prdx4	X	compleme	226,2428	1,349468	2,548181	3,29E-10	3,28E-08	ENSMUSG(
Yy2	X	compleme	0,641285	4,331744	20,13654	3,32E-05	0,000839	ENSMUSG(

Sh3kbp1	X	159627272	16,8974	-3,199	-9,18324	0	0	ENSMUSG(
Map3k15	X	159988433	1,467152	-4,93654	-30,6228	1,62E-08	1,07E-06	ENSMUSG(
Ppef1	X	compleme	1,61321	1,513729	2,855472	0,001709	0,024308	ENSMUSG(
Nhs	X	compleme	1,222918	-1,31565	-2,48915	9,08E-05	0,002018	ENSMUSG(
Zrsr2	X	compleme	1,023527	-1,71291	-3,27822	9,51E-06	0,000285	ENSMUSG(
Asb9	X	164506327	1,911859	-4,95905	-31,1044	8,58E-05	0,001917	ENSMUSG(
Gpm6b	X	166238911	0,333054	-2,71676	-6,57397	4,63E-05	0,001117	ENSMUSG(
Arhgap6	X	168795099	0,246351	-2,26742	-4,8146	0,000651	0,010805	ENSMUSG(
Mid1	X	169685199	4,042149	-1,06564	-2,0931	1,33E-05	0,000382	ENSMUSG(
Gm2098	Y	compleme	6,836526	-1,05391	-2,07615	0,002007	0,027794	ENSMUSG(
Uba1y	Y	818649..84	4,170944	-1,69688	-3,242	2,21E-09	1,77E-07	ENSMUSG(
Uty	Y	compleme	0,357012	1,383931	2,609786	0,002107	0,02895	ENSMUSG(
Gm18798	Y	2678944..2	0,239409	3,966248	15,63002	0,000368	0,006626	ENSMUSG(
Gm28649	Y	compleme	2,660937	-1,54367	-2,91535	0,00037	0,006648	ENSMUSG(
Mid1-ps1	Y	90753057.	6,046064	-1,52449	-2,87685	5,21E-05	0,001238	ENSMUSG(
mt-Nd1	MT	2751..3707	1,536609	-2,22709	-4,68188	0,001151	0,01739	ENSMUSG(
mt-Co1	MT	5328..6872	49,0513	-1,2694	-2,41062	6,41E-08	3,62E-06	ENSMUSG(
mt-Co3	MT	8607..9390	11,08988	-4,07831	-16,8925	8,13E-14	1,78E-11	ENSMUSG(
mt-Nd4	MT	10167..115	1,887298	-2,81265	-7,02575	4,66E-05	0,001123	ENSMUSG(
mt-Cytb	MT	14145..152	5,959345	-1,69781	-3,24408	8,14E-06	0,000249	ENSMUSG(

00000102269
00000032769
00000032719
00000025777
00000025776
00000041859
00000041809
00000025933
00000041670
00000042182
00000037408
00000058407
00000026083
00000026078
00000026077
00000003135
00000026074
00000026049
00000026047
00000041684
00000026048
00000056870
00000026043
00000045954
00000101628
00000041439
00000026096
00000096141
00000042807
00000073676
00000097519
00000041040
00000025969
00000046856
00000025964
00000045005
00000026192
00000039372
00000039354
00000055322
00000026135
00000033276
00000033257
00000026198
00000033124

00000026202
00000026207
00000026235
00000047330
00000055980
00000026234
00000026240
00000026259
00000070738
00000036251
00000036206
00000055013
00000034486
00000034353
00000034292
00000026313
00000040710
00000026333
00000026335
00000099464
00000026321
00000038866
00000044340
00000057329
00000064302
00000026380
00000048402
00000058665
00000026343
00000026344
00000045382
00000026420
00000016528
00000026425
00000013275
00000026433
00000026436
00000026442
00000054387
00000026447
00000070643
00000064246
00000042429
00000042305
00000003051
00000041879
00000009418
00000051985

00000041642

00000026394

00000026393

00000089991

00000026365

00000026361

00000056220

00000066842

00000032666

00000042751

00000073530

00000040423

00000040225

00000026565

00000038530

00000058076

00000006403

00000053483

00000062963

00000013973

00000006412

00000003458

00000013698

00000026546

00000078185

00000057335

00000019699

00000026491

00000038855

00000026496

00000026519

00000038776

00000026510

00000026509

00000038599

00000030768

00000042901

00000056050

00000039384

00000062510

00000037499

00000026639

00000026640

00000016481

00000050530

00000026657

00000002107

00000025782

00000026773
00000037197
00000026737
00000023094
00000036617
00000026979
00000006476
00000026965
00000026944
00000026939
00000036281
00000026924
00000026923
00000015790
00000036040
00000009621
00000026918
00000026917
00000035829
00000035772
00000035666
00000035513
00000079499
00000039844
00000059316
00000057738
00000026785
00000039678
00000007476
00000052533
00000050737
00000026854
00000076441
00000026842
00000001864
00000039262
00000026825
00000039205
00000039157
00000026814
00000039021
00000053746
00000026797
00000004105
00000038765
00000026864
00000035949
00000026874

00000026883
00000083431
00000081999
00000026872
00000036792
00000026764
00000050447
00000026828
00000026991
00000035168
00000083197
00000035000
00000075324
00000057182
00000075318
00000026994
00000064329
00000034780
00000014959
00000027015
00000041911
00000027111
00000055612
00000075284
00000056486
00000042499
00000099521
00000050368
00000043342
00000042369
00000042359
00000002732
00000059173
00000070866
00000059588
00000076437
00000027079
00000027076
00000050896
00000042796
00000025314
00000005505
00000063235
00000002108
00000002109
00000027257
00000027255
00000027222

00000040174
00000032841
00000027163
00000032724
00000027177
00000027176
00000005973
00000081746
00000083320
00000027134
00000027130
00000003604
00000074934
00000027210
00000040152
00000074916
00000070730
00000014077
00000027304
00000034032
00000027288
00000033526
00000027227
00000027201
00000027208
00000003660
00000034850
00000051319
00000048327
00000027399
00000027398
00000074796
00000027330
00000037523
00000048911
00000027357
00000034723
00000074771
00000027276
00000062098
00000074766
00000045624
00000051379
00000037025
00000074736
00000074737
00000027455
00000032869

00000019188
00000042745
00000007659
00000027469
00000051413
00000027475
00000042548
00000027478
00000047459
00000027602
00000038241
00000067847
00000027624
00000067818
00000027635
00000063019
00000027646
00000027649
00000027652
00000016933
00000035877
00000035576
00000017861
00000074607
00000017697
00000017002
00000017721
00000017299
00000017764
00000017760
00000039849
00000017740
00000039804
00000017670
00000017897
00000039621
00000039536
00000017929
00000047030
00000042821
00000078923
00000056501
00000074577
00000051149
00000078919
00000052056
00000027560
00000028640

00000027523
00000027525
00000060445
00000000305
00000015647
00000038848
00000016349
00000027583
00000027506
00000040209
00000102795
00000027533
00000027552
00000027606
00000063887
00000027695
00000027692
00000027684
00000037610
00000027673
00000027716
00000037225
00000037211
00000044864
00000046743
00000037892
00000061143
00000048332
00000027746
00000027797
00000056947
00000036513
00000036503
00000027806
00000027765
00000036885
00000027820
00000027831
00000027832
00000027833
00000027775
00000027834
00000034098
00000027804
00000027809
00000027811
00000033831
00000051000

00000028082
00000028081
00000003382
00000028073
00000004895
00000004896
00000048039
00000004885
00000028069
00000028068
00000001419
00000050144
00000028063
00000074480
00000008604
00000028059
00000028057
00000078684
00000041263
00000028049
00000032657
00000028048
00000047824
00000027951
00000042520
00000027935
00000027936
00000042404
00000042390
00000027933
00000001017
00000042312
00000001021
00000105518
00000001020
00000001025
00000027907
00000041912
00000028136
00000005968
00000068876
00000038902
00000028126
00000053192
00000015711
00000015522
00000038642
00000046519

00000028108
00000015749
00000045934
00000068856
00000063689
00000068855
00000093769
00000050936
00000097041
00000038393
00000028101
00000038256
00000038170
00000027878
00000053398
00000050064
00000027868
00000044468
00000042035
00000033161
00000033147
00000008730
00000105053
00000027843
00000002227
00000062127
00000027901
00000087260
00000027895
00000027894
00000014603
00000027893
00000105872
00000068762
00000004038
00000048997
00000068747
00000068744
00000068740
00000040412
00000027881
00000059857
00000045092
00000027957
00000033377
00000044667
00000033342
00000033308

00000028133
00000028132
00000053965
00000027977
00000032826
00000051278
00000028010
00000058952
00000058897
00000041084
00000062006
00000027985
00000028016
00000040943
00000028273
00000028195
00000043020
00000036853
00000011008
00000036832
00000036825
00000036745
00000028184
00000104786
00000028037
00000039146
00000028036
00000039103
00000039058
00000039047
00000028199
00000053870
00000028182
00000042228
00000041261
00000041235
00000051279
00000049969
00000028212
00000040728
00000052137
00000041135
00000028252
00000040455
00000040183
00000028278
00000028277
00000028419

00000028435
00000028436
00000028437
00000028439
00000073889
00000036052
00000036002
00000035969
00000028458
00000028465
00000028468
00000085351
00000028480
00000035696
00000081127
00000028328
00000028333
00000028339
00000015242
00000015247
00000015243
00000060206
00000003032
00000038827
00000038816
00000028434
00000090053
00000089945
00000038729
00000038544
00000028358
00000045917
00000028370
00000008305
00000049122
00000028398
00000081391
00000066113
00000070934
00000038024
00000070923
00000062937
00000028578
00000028576
00000028565
00000081046
00000067261
00000028549

00000035305
00000028528
00000035069
00000028518
00000028517
00000061887
00000034762
00000028597
00000094958
00000055210
00000082043
00000028708
00000028707
00000003810
00000028690
00000082063
00000046861
00000009640
00000028542
00000028538
00000033295
00000033253
00000006398
00000032998
00000028655
00000011257
00000028649
00000028890
00000044730
00000042677
00000050188
00000028849
00000042558
00000042489
00000028837
00000028830
00000070737
00000028786
00000001333
00000096944
00000047945
00000053730
00000028784
00000023232
00000028773
00000028580
00000028911
00000028906

00000028899

00000085241

00000028893

00000083816

00000037692

00000028868

00000049649

00000037622

00000028854

00000028851

00000047281

00000037553

00000007880

00000028843

00000050989

00000070691

00000037139

00000028668

00000018983

00000070687

00000001089

00000028763

00000041351

00000028766

00000057530

00000046447

00000028745

00000066036

00000036622

00000040842

00000006218

00000028917

00000078515

00000040706

00000040616

00000040606

00000019055

00000029007

00000047719

00000028991

00000006442

00000028975

00000063077

00000028960

00000039953

00000039936

00000039911

00000028965

00000028948
00000024793
00000028937
00000042804
00000005045
00000028931
00000039577
00000039523
00000029030
00000057751
00000029032
00000029064
00000029062
00000035692
00000040537
00000040584
00000028970
00000003623
00000040254
00000028883
00000061601
00000040118
00000028864
00000002944
00000044968
00000028958
00000028944
00000045294
00000029166
00000038828
00000013629
00000043059
00000053134
00000052139
00000029135
00000054280
00000037379
00000037313
00000029111
00000048142
00000029104
00000055302
00000029119
00000029120
00000039474
00000005103
00000061755
00000029082

00000048373
00000031558
00000029167
00000045790
00000039252
00000061461
00000029193
00000039178
00000029108
00000047215
00000029203
00000029205
00000000560
00000036087
00000051674
00000029156
00000054814
00000062110
00000029227
00000029228
00000029229
00000029231
00000036377
00000036323
00000036256
00000037605
00000070697
00000043635
00000055204
00000029379
00000029380
00000058427
00000063015
00000035456
00000029313
00000029310
00000070639
00000029290
00000099061
00000029287
00000033805
00000014668
00000029499
00000043323
00000029512
00000043510
00000029348
00000042010

00000001098
00000002486
00000041740
00000009013
00000029528
00000029516
00000029513
00000041548
00000032850
00000018076
00000032741
00000029603
00000032690
00000066867
00000029605
00000001168
00000066861
00000052776
00000043733
00000029471
00000049686
00000029449
00000038384
00000029438
00000038342
00000066278
00000029404
00000029406
00000037979
00000037936
00000029480
00000029428
00000023079
00000029674
00000007207
00000053293
00000039917
00000039886
00000004951
00000051391
00000004947
00000037411
00000037364
00000029714
00000029718
00000093445
00000036980
00000036968

00000050552
00000025857
00000029547
00000036687
00000036599
00000000149
00000056493
00000039477
00000066640
00000029580
00000039206
00000001847
00000029613
00000047843
00000038859
00000045482
00000029625
00000041453
00000016503
00000041313
00000029657
00000029655
00000032766
00000029661
00000015189
00000052419
00000029552
00000000058
00000007655
00000015733
00000062980
00000029695
00000029701
00000068699
00000071553
00000039070
00000025607
00000025608
00000001642
00000052131
00000029761
00000029833
00000019689
00000061436
00000042599
00000038456
00000029863
00000029860

00000062519
00000071477
00000029817
00000038388
00000029821
00000004980
00000029844
00000038253
00000052955
00000003477
00000029802
00000029917
00000049001
00000029913
00000054474
00000053012
00000049553
00000056091
00000055912
00000053907
00000079523
00000000628
00000000693
00000031865
00000034832
00000014748
00000063415
00000030000
00000029999
00000051695
00000001156
00000057230
00000030056
00000030057
00000044927
00000079477
00000030062
00000015053
00000033216
00000030079
00000002870
00000002871
00000000811
00000030091
00000034192
00000005893
00000030020
00000030064

00000030074
00000030075
00000030101
00000030103
00000030104
00000034269
00000042873
00000001632
00000000440
00000030170
00000045962
00000071226
00000004446
00000040669
00000003154
00000023456
00000030122
00000030120
00000030329
00000038346
00000063870
00000038279
00000030339
00000047976
00000030350
00000001517
00000090099
00000030189
00000107950
00000030199
00000030203
00000046733
00000047104
00000030213
00000060032
00000030216
00000030220
00000030222
00000015766
00000008540
00000030226
00000030247
00000030249
00000030279
00000040370
00000030256
00000016487
00000030304

00000030313
00000068250
00000068566
00000035632
00000035596
00000035545
00000006154
00000035458
00000052296
00000035390
00000004371
00000030432
00000052605
00000086784
00000035279
00000035203
00000083649
00000034071
00000030386
00000058638
00000012848
00000004500
00000041560
00000059273
00000058230
00000041187
00000019370
00000097038
00000048481
00000023118
00000030410
00000050428
00000030407
00000003545
00000030400
00000061028
00000002983
00000002981
00000002985
00000053175
00000002210
00000054793
00000051768
00000057454
00000108367
00000040907
00000045252
00000046541

00000057177
00000040857
00000005442
00000045039
00000057229
00000002603
00000040725
00000002602
00000053291
00000061479
00000003752
00000084974
00000063160
00000040488
00000089832
00000003363
00000049643
00000004056
00000040390
00000046865
00000003435
00000037563
00000037552
00000044786
00000003437
00000109336
00000030600
00000030602
00000051735
00000053898
00000054808
00000030583
00000074221
00000001794
00000037020
00000036957
00000006651
00000036845
00000036835
00000006307
00000036733
00000046056
00000108352
00000058239
00000009687
00000001248
00000083434
00000036427

00000066568
00000063931
00000063808
00000030494
00000023072
00000034875
00000039013
00000004473
00000030739
00000109511
00000011096
00000109324
00000038406
00000046574
00000003421
00000019539
00000003420
00000003429
00000074129
00000003423
00000003868
00000003273
00000003269
00000046179
00000039745
00000109050
00000108326
00000047037
00000033429
00000015133
00000030554
00000108485
00000025790
00000066406
00000039062
00000048897
00000030539
00000038886
00000030530
00000030527
00000030536
00000025723
00000005621
00000025586
00000045795
00000030638
00000057706
00000070462

00000052353
00000039428
00000039405
00000062797
00000030619
00000030616
00000030615
00000030643
00000018995
00000025439
00000030747
00000070436
00000035314
00000018909
00000035227
00000044881
00000030706
00000001829
00000064307
00000099481
00000073982
00000030987
00000037060
00000030895
00000098987
00000048065
00000097220
00000031024
00000030788
00000005609
00000110424
00000038296
00000038244
00000055723
00000091900
00000008683
00000030652
00000008734
00000030876
00000034951
00000030872
00000030869
00000030867
00000052707
00000032637
00000030738
00000047721
00000107068

00000042462
00000049091
00000078580
00000045251
00000042423
00000030814
00000042308
00000049739
00000030847
00000070366
00000030852
00000006205
00000047517
00000040167
00000030934
00000054612
00000041775
00000078566
00000025466
00000038650
00000025498
00000025510
00000025511
00000025147
00000037772
00000000031
00000045752
00000010760
00000031072
00000031070
00000005534
00000047264
00000004567
00000004626
00000040236
00000011832
00000002948
00000008206
00000038894
00000031508
00000038542
00000031543
00000031548
00000056313
00000037363
00000031575
00000031486
00000062991

00000052906
00000031585
00000050271
00000031523
00000031595
00000109991
00000050914
00000031633
00000038143
00000031561
00000039396
00000039375
00000031647
00000031642
00000058056
00000031604
00000036356
00000006273
00000031862
00000036246
00000036180
00000003575
00000071076
00000031834
00000031833
00000007721
00000019261
00000034807
00000034799
00000070000
00000003037
00000003033
00000006276
00000052488
00000019731
00000045248
00000031622
00000031618
00000071064
00000036990
00000064325
00000031714
00000031712
00000031708
00000005483
00000057672
00000002885
00000013033

00000031706
00000037103
00000036686
00000035671
00000001910
00000033751
00000003813
00000053693
00000031691
00000031697
00000036934
00000031701
00000031661
00000031665
00000056973
00000031748
00000031755
00000031765
00000074151
00000034361
00000031785
00000046556
00000031790
00000046707
00000036550
00000096188
00000031884
00000110597
00000031889
00000054320
00000050357
00000038000
00000037415
00000036270
00000031897
00000031902
00000031904
00000060019
00000046691
00000041438
00000031921
00000003847
00000003848
00000031930
00000037993
00000069895
00000031731
00000031732

00000010936

00000033763

00000031960

00000033624

00000031959

00000031951

00000092086

00000078908

00000031844

00000031827

00000031826

00000031825

00000031816

00000031812

00000061410

00000025318

00000040010

00000017478

00000006585

00000015027

00000035569

00000000743

00000001062

00000032815

00000074037

00000062380

00000001482

00000031967

00000040220

00000031972

00000089704

00000050751

00000074030

00000025810

00000041624

00000025894

00000025892

00000033538

00000032006

00000041620

00000043613

00000043089

00000047562

00000005800

00000057367

00000050730

00000031925

00000004096

00000031931
00000004099
00000003299
00000037405
00000111497
00000010205
00000035047
00000057193
00000032178
00000032180
00000032185
00000032187
00000032198
00000040563
00000003402
00000001349
00000031963
00000031965
00000111558
00000031988
00000031990
00000034275
00000062257
00000059974
00000031994
00000047412
00000041737
00000016087
00000032038
00000039048
00000050471
00000038119
00000032101
00000032103
00000050555
00000032113
00000032121
00000111684
00000038112
00000040111
00000074415
00000032135
00000032123
00000032112
00000009927
00000063382
00000002032
00000002028

00000070305
00000032085
00000003131
00000032026
00000032271
00000040219
00000039542
00000032067
00000000171
00000039016
00000032051
00000034218
00000032047
00000055069
00000042195
00000061559
00000032314
00000074305
00000032911
00000032733
00000032288
00000042557
00000032300
00000040722
00000111414
00000000088
00000040188
00000032312
00000038957
00000038264
00000037716
00000032327
00000032333
00000066607
00000032338
00000032289
00000032280
00000007892
00000032249
00000041729
00000032245
00000074250
00000058444
00000032402
00000040652
00000032382
00000032226
00000032224

00000032220
00000032186
00000032358
00000037742
00000034898
00000033577
00000032261
00000032262
00000032418
00000032419
00000032420
00000074149
00000102037
00000032359
00000037410
00000032369
00000032372
00000074139
00000032377
00000033350
00000032412
00000032463
00000096316
00000032475
00000070287
00000037286
00000032527
00000032547
00000032553
00000032563
00000035032
00000032786
00000053716
00000040661
00000040325
00000039716
00000032596
00000032594
00000039952
00000006673
00000066357
00000032602
00000032598
00000023473
00000032479
00000032481
00000056724
00000032436

00000040875
00000032440
00000061393
00000041794
00000032536
00000025784
00000038587
00000040021
00000015305
00000060487
00000019832
00000097378
00000015501
00000039835
00000019853
00000019852
00000019851
00000019850
00000071369
00000019996
00000019982
00000045680
00000019978
00000111882
00000039031
00000019880
00000019792
00000039684
00000039462
00000019779
00000019843
00000019842
00000038481
00000019831
00000038248
00000038214
00000047139
00000038151
00000019848
00000019857
00000019856
00000037990
00000019916
00000020097
00000020069
00000020067
00000020064
00000020230

00000033126
00000001120
00000020265
00000020284
00000005054
00000020331
00000020329
00000035890
00000006498
00000013833
00000035781
00000004665
00000004667
00000035673
00000020150
00000063457
00000020163
00000047417
00000003346
00000035370
00000020198
00000061589
00000020211
00000034771
00000020246
00000109864
00000034612
00000020256
00000020032
00000020042
00000035529
00000020044
00000035311
00000008398
00000015889
00000020018
00000020019
00000020021
00000020023
00000020027
00000019929
00000036446
00000019952
00000019960
00000019894
00000112066
00000053825
00000035948

00000019906
00000035916
00000020205
00000050663
00000112099
00000020151
00000020154
00000054934
00000064181
00000112252
00000112814
00000112419
00000056758
00000051236
00000052302
00000034613
00000078429
00000040502
00000006731
00000040415
00000025417
00000047492
00000025404
00000040258
00000040054
00000039994
00000005683
00000025369
00000000711
00000025350
00000020454
00000056579
00000034543
00000048807
00000002129
00000034394
00000020412
00000034201
00000083844
00000020473
00000041164
00000020422
00000020407
00000078974
00000033953
00000082852
00000045671
00000020152

00000044068
00000004018
00000020467
00000044072
00000020315
00000043999
00000020289
00000020287
00000020272
00000057967
00000069911
00000018848
00000049336
00000044707
00000057098
00000011256
00000020340
00000020354
00000078153
00000095187
00000020363
00000050567
00000063564
00000020358
00000007777
00000020387
00000020389
00000000782
00000049470
00000018395
00000018900
00000020334
00000020333
00000020400
00000037331
00000020496
00000078851
00000020444
00000048076
00000020472
00000032691
00000062115
00000018415
00000049323
00000081578
00000043284
00000018932
00000019102

00000010025
00000042436
00000001034
00000001036
00000042377
00000042331
00000018501
00000018217
00000033389
00000020902
00000020900
00000020899
00000020898
00000020893
00000049299
00000018474
00000018774
00000018750
00000051790
00000019461
00000047284
00000023170
00000078812
00000018565
00000018567
00000020886
00000020831
00000040904
00000093989
00000018921
00000060216
00000020828
00000020827
00000040746
00000018293
00000040712
00000040620
00000070390
00000020811
00000020807
00000040463
00000050107
00000047260
00000005952
00000038807
00000020741
00000010554
00000038351

00000038290
00000043099
00000000751
00000045374
00000020850
00000038178
00000017781
00000017774
00000020849
00000017288
00000069808
00000049396
00000017631
00000010392
00000020840
00000037958
00000037926
00000083899
00000037907
00000011877
00000017291
00000037857
00000000631
00000037791
00000061981
00000020832
00000037750
00000019437
00000002052
00000010277
00000002055
00000017390
00000041958
00000050132
00000049489
00000018334
00000020716
00000017428
00000020697
00000020696
00000035152
00000020684
00000020680
00000081906
00000034940
00000020532
00000018697
00000018698

00000045140
00000061666
00000020493
00000020486
00000034177
00000034156
00000000275
00000020857
00000037601
00000076435
00000001506
00000038976
00000013415
00000020875
00000048763
00000038615
00000018678
00000020877
00000038517
00000018882
00000049807
00000038437
00000078695
00000018537
00000018541
00000038366
00000020882
00000020883
00000018160
00000003119
00000018167
00000002580
00000038067
00000017210
00000020889
00000078676
00000038013
00000037992
00000054146
00000020911
00000035557
00000053654
00000001552
00000006782
00000020918
00000019173
00000035355
00000001755

00000017801
00000035198
00000045007
00000017167
00000078651
00000034993
00000017724
00000020921
00000034793
00000034708
00000034621
00000050288
00000020926
00000020929
00000051378
00000045532
00000020937
00000048878
00000055805
00000020941
00000018411
00000020689
00000053580
00000040699
00000069631
00000020705
00000020708
00000020715
00000020718
00000040430
00000020720
00000050965
00000041920
00000020620
00000050910
00000020747
00000020758
00000020770
00000034341
00000020775
00000020780
00000050628
00000020802
00000020810
00000025571
00000048277
00000017132
00000033909

00000017466
00000025575
00000025579
00000070327
00000039850
00000025583
00000025372
00000062825
00000039640
00000025132
00000025140
00000025144
00000078249
00000025153
00000025162
00000039230
00000020668
00000037486
00000020658
00000020640
00000020627
00000054459
00000020607
00000020601
00000020593
00000051726
00000011179
00000062054
00000020653
00000020649
00000020644
00000020641
00000020638
00000063632
00000020674
00000020669
00000002900
00000020659
00000002997
00000004698
00000002565
00000035954
00000020598
00000046314
00000020953
00000061603
00000021010
00000035105

00000035451
00000020994
00000020978
00000019718
00000021103
00000033454
00000090258
00000021118
00000015143
00000049106
00000021130
00000062961
00000021133
00000021136
00000042700
00000042628
00000010608
00000021224
00000054383
00000042507
00000002020
00000021256
00000061533
00000020961
00000033713
00000033530
00000047415
00000021186
00000021200
00000021203
00000086432
00000041415
00000021097
00000044715
00000021111
00000021262
00000018707
00000021270
00000037896
00000021279
00000001270
00000064326
00000072825
00000072812
00000021144
00000006360
00000000078
00000021193

00000058258
00000033499
00000052374
00000050244
00000057554
00000021306
00000005397
00000019726
00000021301
00000071516
00000069307
00000094248
00000006720
00000069302
00000060981
00000064168
00000100210
00000069273
00000069272
00000099583
00000061482
00000069270
00000069310
00000075029
00000060093
00000060081
00000036006
00000076431
00000016477
00000050295
00000038372
00000021420
00000054889
00000021359
00000038683
00000021361
00000021364
00000021366
00000051335
00000044164
00000038518
00000078915
00000046876
00000038132
00000021373
00000021374
00000038068
00000021379

00000038014
00000021391
00000021388
00000091383
00000067586
00000021448
00000021451
00000021457
00000043183
00000025871
00000025869
00000034928
00000021485
00000021493
00000021500
00000007836
00000021550
00000021556
00000021559
00000033063
00000097565
00000064063
00000034617
00000034575
00000021598
00000049538
00000021606
00000021608
00000017756
00000086743
00000021587
00000021589
00000061833
00000021596
00000069171
00000074794
00000043889
00000005583
00000096449
00000021614
00000003992
00000021690
00000042082
00000042015
00000021678
00000021676
00000021670
00000057762

0000069117
0000052485
0000052727
0000021638
0000041417
0000034751
0000021714
0000046169
0000021697
0000055194
0000021759
0000042379
0000021765
0000021725
0000074715
0000025278
0000025279
0000021751
0000021749
0000054423
0000021738
0000017491
0000058317
0000021806
0000045107
0000034235
0000021819
0000039308
0000021782
0000025280
0000021868
0000094800
0000078128
0000021962
0000021917
0000042323
0000071547
0000019027
0000021913
0000023064
0000037824
0000021792
0000037759
0000021840
0000021846
0000036023
0000022160
0000106533

00000040997
00000000959
00000022175
00000059674
00000022185
00000092232
00000040701
00000002325
00000040472
00000021959
00000021973
00000046168
00000021932
00000014547
00000035095
00000063060
00000060012
00000021975
00000007989
00000022037
00000022040
00000022061
00000094526
00000034205
00000022075
00000033712
00000022091
00000033589
00000022096
00000022095
00000022099
00000033446
00000021998
00000022000
00000067995
00000095427
00000022012
00000022015
00000022018
00000022064
00000005148
00000072294
00000075486
00000022125
00000045871
00000033214
00000022112
00000022130

00000022132
00000022136
00000042104
00000025555
00000041703
00000032925
00000091119
00000055737
00000022184
00000022150
00000022146
00000005360
00000022246
00000047497
00000022206
00000022197
00000022195
00000045763
00000022272
00000022270
00000090544
00000039168
00000022261
00000022323
00000022305
00000022309
00000022336
00000022340
00000022311
00000038679
00000063727
00000037362
00000022419
00000022371
00000071757
00000051225
00000022353
00000022350
00000059586
00000032501
00000072568
00000015002
00000056258
00000005125
00000022332
00000068391
00000022575
00000055762

00000046761
00000022568
00000022564
00000034259
00000034022
00000033697
00000022443
00000071711
00000043460
00000049521
00000068220
00000033088
00000009035
00000022429
00000089715
00000060036
00000022415
00000051518
00000047888
00000042292
00000022400
00000048546
00000061360
00000022463
00000022450
00000018042
00000041736
00000016757
00000006369
00000016541
00000022385
00000016028
00000054863
00000034333
00000023272
00000036606
00000015377
00000091780
00000022615
00000022629
00000022475
00000022479
00000052369
00000022483
00000048154
00000023009
00000023017
00000091604

00000023018
00000023022
00000023032
00000000532
00000047641
00000067615
00000067613
00000022986
00000061527
00000036966
00000058290
00000023048
00000099083
00000001655
00000001656
00000022484
00000001657
00000001661
00000075394
00000009575
00000046434
00000000552
00000005982
00000005718
00000039568
00000022515
00000022500
00000062203
00000065979
00000018830
00000005899
00000012114
00000003531
00000003526
00000060166
00000000884
00000071632
00000022706
00000041247
00000022686
00000062901
00000022822
00000033809
00000045983
00000044626
00000033581
00000013089
00000022884

00000022885
00000022508
00000022510
00000022514
00000022523
00000022525
00000046598
00000014074
00000053774
00000079625
00000022791
00000035629
00000035506
00000034473
00000022848
00000022906
00000075229
00000022827
00000034158
00000022664
00000033157
00000033149
00000032965
00000055447
00000035356
00000075033
00000098274
00000022601
00000044763
00000052917
00000022755
00000022754
00000022749
00000052504
00000091243
00000022762
00000097418
00000022889
00000025616
00000025612
00000022969
00000022964
00000089774
00000051989
00000022952
00000022948
00000040732
00000040681

00000000386
00000023341
00000046962
00000069729
00000052397
00000023830
00000062480
00000068036
00000023885
00000014769
00000048027
00000090304
00000071273
00000067942
00000023904
00000023905
00000023909
00000023911
00000039218
00000055839
00000050762
00000036820
00000024121
00000034681
00000024136
00000002496
00000002504
00000041130
00000044533
00000075705
00000045316
00000039183
00000067722
00000035521
00000015120
00000002279
00000002280
00000061046
00000057411
00000002274
00000025732
00000073433
00000024301
00000067629
00000079605
00000042644
00000046711
00000052146

00000024217
00000024218
00000002250
00000063952
00000042203
00000044477
00000024026
00000024034
00000061613
00000024042
00000002791
00000024002
00000024050
00000073423
00000059208
00000002289
00000079553
00000024308
00000024335
00000034786
00000034673
00000034254
00000015461
00000092200
00000090877
00000091971
00000007029
00000024391
00000024392
00000024393
00000001524
00000003541
00000059714
00000050705
00000039220
00000038762
00000038500
00000073403
00000092265
00000036398
00000036315
00000106106
00000023932
00000023938
00000023947
00000037089
00000023942
00000036026

00000023939
00000012296
00000015597
00000015599
00000040658
00000002767
00000036430
00000064043
00000096361
00000023988
00000023991
00000024172
00000002835
00000001227
00000001229
00000024201
00000013236
00000042625
00000071054
00000002372
00000024206
00000024212
00000002658
00000007670
00000024164
00000019487
00000024228
00000024085
00000033278
00000032796
00000024043
00000034868
00000024053
00000024063
00000024064
00000002017
00000024076
00000036533
00000036368
00000024247
00000045817
00000024140
00000050138
00000024240
00000042942
00000005836
00000024413
00000057766

)0000024421
)0000036225
)0000024304
)0000044393
)0000056124
)0000033107
)0000024302
)0000024277
)0000039616
)0000034295
)0000024269
)0000046636
)0000024260
)0000024382
)0000038128
)0000024378
)0000024376
)0000038773
)0000038555
)0000024349
)0000046668
)0000033272
)0000024456
)0000044024
)0000024451
)0000024427
)0000036452
)0000024487
)0000056671
)0000057719
)0000071856
)0000033949
)0000053644
)0000024598
)0000024600
)0000053441
)0000058152
)0000038059
)0000043079
)0000024608
)0000024613
)0000036412
)0000033871
)0000045094
)0000032735
)0000071847
)0000040560
)0000024589

00000042439
00000046318
00000024524
00000024511
00000052928
00000025421
00000047466
00000053950
00000033323
00000058881
00000046982
00000024644
00000050321
00000024829
00000037916
00000060803
00000038155
00000058216
00000083282
00000006457
00000006463
00000024901
00000024902
00000047379
00000080268
00000024875
00000024862
00000024844
00000024841
00000024914
00000024927
00000056917
00000054874
00000004054
00000024940
00000024826
00000045664
00000024797
00000024949
00000047810
00000038812
00000024955
00000024959
00000056612
00000037349
00000036278
00000035885
00000024969

00000024759
00000024973
00000042729
00000071657
00000071653
00000071648
00000024660
00000024661
00000024665
00000036098
00000034820
00000034445
00000024736
00000046139
00000024691
00000024642
00000024640
00000024713
00000036192
00000052085
00000032702
00000024924
00000046324
00000024868
00000052920
00000035783
00000050370
00000034459
00000024805
00000067279
00000024986
00000054237
00000055044
00000025006
00000025019
00000074873
00000036450
00000025209
00000025213
00000056209
00000039901
00000025223
00000055491
00000015176
00000025224
00000025225
00000025231
00000025034

00000047731
00000064105
00000042694
00000044948
00000062083
00000034765
00000024975
00000025078
00000054843
00000025092
00000043969
00000024991
00000073295
00000103436
00000031145
00000031146
00000000134
00000031153
00000031154
00000039201
00000031176
00000054453
00000040363
00000064127
00000025037
00000040138
00000037315
00000001924
00000031065
00000031066
00000001131
00000051537
00000036782
00000068113
00000016239
00000037636
00000016319
00000016308
00000044149
00000050379
00000036572
00000016409
00000036537
00000048047
00000016534
00000036959
00000031103
00000036932

00000031112
00000062184
00000036022
00000023074
00000031137
00000000838
00000031344
00000031383
00000019558
00000031386
00000019087
00000015291
00000032806
00000031400
00000090110
00000031197
00000031202
00000025666
00000045103
00000035476
00000035246
00000025151
00000031207
00000046532
00000079487
00000079481
00000033965
00000031231
00000056854
00000034551
00000025597
00000051579
00000042498
00000031283
00000031284
00000025272
00000025265
00000041229
00000025261
00000041133
00000081306
00000041096
00000045180
00000047238
00000050148
00000086316
00000025289
00000091736

00000040990
00000031303
00000062168
00000059493
00000031370
00000031384
00000031342
00000031355
00000035299
00000100968
00000069053
00000068457
00000101064
00000100360
00000095134
00000064341
00000064351
00000064358
00000064363
00000064370
