# Supplementary material for: Low Intensity Pulsed Ultrasounds Modulate Adipose Stem Cells Differentiation
Source: Stem Cell Rev Rep. 2025 May 23;21(6):1760–75. doi: 10.1007/s12015-025-10896-7 (PMC12356745; doi:10.1007/s12015-025-10896-7)
Supplement: Supplementary file 2 — Supplementary file2 (PDF 11446 KB) [file 12015_2025_10896_MOESM2_ESM.pdf]

Name	Chromosome	Region	hoASCs	Lipus hoASCs	Log_2 fold change
OR4F5	1	65419..71585	-	-	#N/D
OR4F29	1	complement(450740..451678)	-	0,03	-3,804623889
OR4F16	1	complement(685716..686654)	-	0,01	-2,89252005
SAMD11	1	923923..944575	7,14	5,84	0,51280541
NOC2L	1	complement(944203..959309)	65,77	66,31	0,211297107
KLHL17	1	960584..965719	11,79	10,64	0,370487794
PLEKHN1	1	966482..975865	1,71	2,04	-0,032601568
PERM1	1	complement(975198..982117)	0,21	0,31	-0,34470872
HES4	1	complement(998962..1000172)	3,61	5,18	-0,298192833
ISG15	1	1001138..1014540	22,62	19,79	0,416248876
AGRN	1	1020120..1056118	55,23	46,92	0,458460996
RNF223	1	complement(1070967..1074306)	0,12	1,66	-3,563420387
C1orf159	1	complement(1081818..1116361)	3,23	3,63	0,053611548
TTLL10	1	1173880..1197936	0,01	-	3,967268276
TNFRSF18	1	complement(1203508..1206592)	0,09	0,03	1,790975958
TNFRSF4	1	complement(1211326..1214153)	0,15	0,14	0,3726069
SDF4	1	complement(1216931..1232031)	55,23	60,85	0,083272955
B3GALT6	1	1232237..1235041	6,16	7,80	-0,117104816
C1QTNF12	1	complement(1242446..1246722)	0,91	0,30	1,811664804
UBE2J2	1	complement(1253909..1273864)	11,03	14,16	-0,136548627
SCNN1D	1	1280436..1292029	1,26	1,91	-0,372404447
ACAP3	1	complement(1292390..1309609)	13,55	11,00	0,524319219
PUSL1	1	1308597..1311677	14,94	10,62	0,715686135
INTS11	1	complement(1311585..1324687)	19,66	17,73	0,372051595
CPTP	1	1324756..1328896	9,46	12,06	-0,1273232
TAS1R3	1	1331280..1335314	3,08	2,63	0,449743538
DVL1	1	complement(1335276..1349418)	62,64	58,27	0,327456032
MXRA8	1	complement(1352689..1361777)	35,86	26,55	0,656443297
AURKAIP1	1	complement(1373730..1375207)	36,58	39,97	0,094957402
CCNL2	1	complement(1385711..1399335)	21,89	20,72	0,302321573
MRPL20	1	complement(1401909..1407293)	7,89	9,82	-0,092996132
ANKRD65	1	complement(1418420..1421769)	0,02	0,02	-0,032860829
TMEM88B	1	1425871..1430255	0,01	0,04	-1,326441896
VWA1	1	1434861..1442882	1,17	0,76	0,840374804
ATAD3C	1	1449689..1470163	0,15	0,14	0,35593437
ATAD3B	1	1471765..1497848	8,70	5,67	0,841857109
ATAD3A	1	1512162..1534685	19,33	17,01	0,407613208
TMEM240	1	complement(1534778..1540624)	0,81	1,04	-0,151597341
SSU72	1	complement(1541673..1574863)	14,68	13,99	0,293347997
FNDC10	1	complement(1598012..1600135)	5,00	10,18	-0,802421134
MIB2	1	1615454..1630605	9,53	7,15	0,636577887
MMP23B	1	1632163..1635263	0,27	0,38	-0,24259662
CDK11B	1	complement(1635225..1659012)	9,37	10,13	0,110489764
SLC35E2B	1	complement(1659529..1692795)	16,90	21,25	-0,107414732
CDK11A	1	complement(1702379..1724357)	6,26	5,72	0,352835036

NADK	1	complement(1751232..1780457)	12,58	16,64	-0,180498521
GNB1	1	complement(1785285..1892292)	91,30	85,37	0,320009462
CALML6	1	1915108..1917296	0,20	0,08	1,520939857
TMEM52	1	complement(1917590..1919279)	0,29	0,04	2,926600518
CFAP74	1	complement(1921951..2003837)	0,00	0,00	-0,923955849
GABRD	1	2019329..2030758	0,03	0,01	2,399873901
PRKCZ	1	2050411..2185395	0,97	0,65	0,799468365
FAAP20	1	complement(2184461..2212720)	6,31	6,44	0,193038818
SKI	1	2227388..2310213	14,07	14,10	0,219607989
MORN1	1	complement(2321253..2391707)	1,20	1,17	0,263727762
RER1	1	2391775..2405442	38,41	20,77	1,110235314
PEX10	1	complement(2403964..2413797)	5,71	6,72	-0,011382514
PLCH2	1	2425980..2505532	0,26	0,19	0,628864151
PANK4	1	complement(2508537..2526597)	11,20	8,90	0,554526479
HES5	1	complement(2528745..2530263)	-	-	0,188027316
TNFRSF14	1	2555639..2565382	0,19	0,01	4,424919836
gene:ENSG000	1	2566410..2569888	0,01	0,01	-0,429837071
PRXL2B	1	2586491..2591469	4,23	4,45	0,152135849
MMEL1	1	complement(2590639..2633016)	-	-	0,188027316
TTC34	1	complement(2636986..2801693)	0,36	0,52	-0,283343244
ACTRT2	1	3021467..3022903	-	-	#N/D
PRDM16	1	3069168..3438621	1,14	0,38	1,805109072
gene:ENSG000	1	3205988..3208664	0,08	0,02	1,932233931
ARHGEF16	1	3454665..3481113	0,64	5,18	-2,789460568
MEGF6	1	complement(3487951..3611508)	15,25	15,56	0,194861392
TPRG1L	1	3625015..3630127	25,96	15,92	0,92822848
WRAP73	1	complement(3630767..3652761)	11,69	12,43	0,135417967
TP73	1	3652516..3736201	1,46	1,84	-0,111042298
CCDC27	1	3746460..3771645	0,02	0,02	-0,032860829
SMIM1	1	3772749..3775982	0,07	0,05	0,533811434
LRRC47	1	complement(3778559..3796498)	16,07	16,40	0,193428444
CEP104	1	complement(3812086..3857396)	4,11	5,42	-0,176140932
DFFB	1	3857267..3885429	2,65	2,65	0,222805552
C1orf174	1	complement(3889125..3900293)	15,17	11,02	0,685090376
AJAP1	1	4654609..4792534	0,07	-	9,235935336
NPHP4	1	complement(5862811..5992473)	6,73	5,30	0,566675156
KCNAB2	1	5990927..6101193	3,27	4,91	-0,364054893
CHD5	1	complement(6101787..6180321)	0,91	0,22	2,290375291
gene:ENSG000	1	complement(6159430..6197757)	0,18	0,06	1,790975958
RPL22	1	complement(6185020..6209389)	29,66	37,39	-0,11133285
RNF207	1	6205475..6221299	2,27	2,85	-0,104739364
ICMT	1	complement(6221193..6235972)	21,60	24,88	0,019022132
HES3	1	6244179..6245578	-	0,02	-2,89252005
GPR153	1	complement(6247353..6261098)	2,87	1,34	1,324027446
ACOT7	1	complement(6264269..6393767)	16,53	42,07	-1,124553511
HES2	1	complement(6412418..6424670)	0,10	0,45	-1,899356265
ESPN	1	6424776..6461367	0,03	0,04	-0,120778226
TNFRSF25	1	complement(6460786..6466175)	8,93	5,12	1,024174615

PLEKHG5	1	complement(6467122..6520074)	13,63	10,36	0,618500622
NOL9	1	complement(6521347..6554513)	5,45	10,02	-0,656231002
TAS1R1	1	6555307..6579755	0,04	0,06	-0,411705719
ZBTB48	1	6579994..6589280	3,86	3,75	0,266079802
KLHL21	1	complement(6590724..6614607)	7,12	8,46	-0,025734505
PHF13	1	6613731..6624030	14,86	13,85	0,325227623
THAP3	1	6624868..6635586	3,82	4,28	0,059105861
DNAJC11	1	complement(6634168..6701924)	9,99	17,02	-0,5459323
CAMTA1	1	6785454..7769706	1,22	1,23	0,215865579
VAMP3	1	7771296..7781432	23,69	21,31	0,375953875
PER3	1	7784291..7845177	2,08	1,74	0,480058467
UTS2	1	complement(7843083..7853512)	0,01	0,06	-2,854330803
TNFRSF9	1	complement(7915871..7943165)	0,86	12,47	-3,639406394
PARK7	1	7954291..7985505	57,11	62,41	0,095085428
ERRFI1	1	complement(8004404..8026309)	37,67	29,24	0,588374525
SLC45A1	1	8318114..8344167	0,46	0,79	-0,560025111
RERE	1	complement(8352397..8848921)	7,80	13,50	-0,568633305
ENO1	1	complement(8861000..8879190)	#######	#####	0,601625505
CA6	1	8945867..8975092	-	0,01	-3,804623889
SLC2A7	1	complement(9002973..9026423)	-	-	0,188027316
SLC2A5	1	complement(9035106..9088478)	0,08	0,03	1,536756392
GPR157	1	complement(9100305..9129102)	6,03	6,55	0,103041333
H6PD	1	9234774..9271337	14,97	21,72	-0,313484975
SPSB1	1	9292894..9369532	7,12	12,79	-0,620853526
SLC25A33	1	9539465..9585173	1,84	2,73	-0,347297924
TMEM201	1	9588911..9614877	4,53	7,23	-0,452480614
PIK3CD	1	9629889..9729114	6,47	12,85	-0,765508479
CLSTN1	1	complement(9728926..9823984)	32,07	48,44	-0,371820022
gene:ENSG000	1	complement(9826289..9828271)	0,01	0,03	-0,979341556
CTNNBIP1	1	complement(9848276..9910336)	4,26	3,34	0,573329629
LZIC	1	complement(9922113..9943407)	5,66	6,41	0,042665631
NMNAT1	1	9943428..9985501	1,02	1,61	-0,438745401
RBP7	1	9997206..10016021	0,44	0,44	0,230226832
UBE4B	1	10032832..10181239	10,99	11,68	0,135013304
KIF1B	1	10210570..10381603	5,26	7,87	-0,356992815
PGD	1	10398592..10420511	33,59	52,05	-0,408587194
CENPS-CORT	1	10430102..10452153	0,18	0,39	-0,915620774
CENPS	1	10430433..10442808	1,34	3,31	-1,077661012
CORT	1	10450031..10451998	0,24	0,30	-0,109163979
DFFA	1	complement(10456522..10472529)	7,00	12,66	-0,631961966
PEX14	1	10472288..10630758	11,14	13,88	-0,094717098
CASZ1	1	complement(10636604..10796650)	0,34	0,41	-0,07726937
C1orf127	1	complement(10946471..10982076)	-	0,00	-2,89252005
TARDBP	1	11012344..11030528	18,42	20,70	0,054537455
MASP2	1	complement(11022009..11047239)	0,37	0,60	-0,478541483
SRM	1	complement(11054584..11060020)	47,10	57,55	-0,06604967
EXOSC10	1	complement(11066618..11099869)	17,44	24,80	-0,285248719
MTOR	1	complement(11106535..11262556)	14,59	14,94	0,189508215

ANGPTL7	1	11189355..11195981	0,40	0,09	2,39970345
UBIAD1	1	11273198..11296049	4,10	4,77	0,006064275
DISP3	1	11479155..11537551	0,02	0,02	0,728122296
FBXO2	1	complement(11637018..11655785)	1,01	0,75	0,653899425
FBXO44	1	11654375..11663327	7,74	9,49	-0,071882954
MAD2L2	1	complement(11658918..11691811)	17,11	22,36	-0,162411461
FBXO6	1	11664200..11674354	3,86	5,07	-0,168873456
DRAXIN	1	11691710..11725857	0,27	0,86	-1,43941041
AGTRAP	1	11736084..11754802	23,54	39,81	-0,534814153
C1orf167	1	11761787..11789585	0,01	-	4,516772761
MTHFR	1	complement(11785723..11806455)	1,35	1,57	0,009394981
CLCN6	1	11806096..11848079	8,05	3,52	1,416399782
NPPA	1	complement(11845709..11848345)	0,23	0,25	0,085296906
NPPB	1	complement(11857464..11858945)	-	-	0,188027316
KIAA2013	1	complement(11919591..11926428)	26,22	21,69	0,496574548
PLOD1	1	11934205..11975538	97,18	78,14	0,537553782
MFN2	1	11980181..12015211	27,53	31,61	0,02395151
MIIP	1	12019466..12032045	9,21	13,97	-0,378455699
TNFRSF8	1	12063303..12144207	0,01	0,07	-2,850473978
TNFRSF1B	1	12166991..12209228	0,66	1,60	-1,06256822
VPS13D	1	12230030..12512047	4,65	4,76	0,190089331
DHRS3	1	complement(12567909..12620133)	15,43	38,19	-1,084059965
AADACL4	1	12644085..12667076	-	0,01	-2,89252005
AADACL3	1	12716110..12728760	-	-	#N/D
CFAP107	1	12746200..12763699	-	-	0,188027316
PRAMEF12	1	12773738..12777906	0,01	-	4,516772761
PRAMEF1	1	12791397..12796628	-	-	#N/D
PRAMEF11	1	complement(12824605..12831410)	-	-	#N/D
HNRNPCL1	1	complement(12847377..12848720)	-	-	0,188027316
PRAMEF2	1	12857086..12861909	-	-	#N/D
PRAMEF4	1	complement(12879212..12886201)	-	-	#N/D
PRAMEF10	1	complement(12892896..12898270)	-	-	#N/D
PRAMEF7	1	12916610..12920751	-	-	#N/D
PRAMEF6	1	complement(12938472..12947580)	-	-	#N/D
PRAMEF27	1	complement(13049476..13056575)	-	-	#N/D
HNRNPCL3	1	13060769..13062878	-	-	#N/D
PRAMEF25	1	13068677..13077884	-	-	0,188027316
HNRNPCL2	1	complement(13115488..13116854)	-	-	#N/D
PRAMEF26	1	complement(13148905..13158116)	-	-	#N/D
HNRNPCL4	1	complement(13163914..13165631)	-	-	#N/D
PRAMEF9	1	13171848..13179459	-	-	#N/D
PRAMEF13	1	complement(13196188..13201409)	-	-	#N/D
PRAMEF18	1	complement(13222695..13226154)	-	-	#N/D
PRAMEF5	1	13254198..13263435	-	-	#N/D
PRAMEF8	1	complement(13281304..13285137)	-	-	#N/D
PRAMEF33	1	13303539..13308907	-	-	#N/D
PRAMEF15	1	13315581..13322598	-	-	#N/D
PRAMEF14	1	complement(13341892..13347134)	-	-	#N/D

PRAMEF19	1	complement(13368431..13371947)	-	-	#N/D
PRAMEF17	1	13389632..13392629	-	-	#N/D
PRAMEF20	1	13410450..13421328	-	-	0,188027316
LRRC38	1	complement(13474973..13514003)	-	0,18	-7,624346382
PDPN	1	13583465..13617957	21,69	46,93	-0,890109951
PRDM2	1	13700188..13825079	5,55	6,02	0,10597057
KAZN	1	13892792..15118043	0,28	1,07	-1,730884421
TMEM51	1	15152532..15220478	1,65	4,88	-1,343696154
FHAD1	1	15236521..15400283	0,05	0,10	-0,683584987
EFHD2	1	15409888..15430339	61,41	87,88	-0,293931931
CTRC	1	15438442..15449242	0,05	0,03	0,857064368
CELA2A	1	15456728..15472091	0,06	-	5,224730805
CELA2B	1	15465909..15491395	0,01	-	3,068695355
CASP9	1	complement(15490832..15526534)	1,74	2,26	-0,153775479
DNAJC16	1	15526813..15592379	2,63	2,90	0,082318649
AGMAT	1	complement(15571699..15585051)	0,20	0,81	-1,779908685
DDI2	1	15617458..15669044	3,41	3,72	0,098960915
RSC1A1	1	15659713..15662033	0,71	1,28	-0,628345113
PLEKHM2	1	15684320..15734769	21,37	26,42	-0,082869417
SLC25A34	1	15736258..15741392	0,13	0,60	-1,920947998
TMEM82	1	15742499..15747982	0,01	0,13	-2,851509423
FBLIM1	1	15756607..15786594	3,61	6,40	-0,601964914
UQCRHL	1	complement(15807169..15809348)	0,21	0,48	-0,967522504
SPEN	1	15836095..15940456	6,12	8,53	-0,255791978
ZBTB17	1	complement(15941869..15976132)	10,93	11,78	0,114920143
SRARP	1	16004236..16008807	0,00	0,00	-0,01185201
HSPB7	1	complement(16014028..16019594)	0,00	-	3,068695355
CLCNKA	1	16018875..16034050	-	0,01	-2,89252005
CLCNKB	1	16040252..16057311	0,03	0,02	0,943435191
FAM131C	1	complement(16057769..16073651)	1,84	3,77	-0,80949263
EPHA2	1	complement(16124337..16156069)	56,72	70,56	-0,091883293
ARHGEF19	1	complement(16197854..16212652)	10,36	12,09	0,000504537
CPLANE2	1	complement(16231692..16237183)	1,27	2,81	-0,926694723
FBXO42	1	complement(16246840..16352480)	3,38	4,24	-0,104197247
SZRD1	1	16352575..16398145	57,25	43,26	0,627172939
SPATA21	1	complement(16387117..16437424)	0,04	0,04	0,247752455
NECAP2	1	16440721..16460078	21,40	17,03	0,552589776
NBPF1	1	complement(16562319..16613562)	2,22	3,99	-0,624258181
CROCC	1	16740273..16972964	1,61	2,70	-0,520623581
MFAP2	1	complement(16974502..16980632)	56,99	62,05	0,100337035
ATP13A2	1	complement(16985958..17011928)	11,16	14,47	-0,151295454
gene:ENSG000	1	complement(17005068..17011757)	-	-	#N/D
SDHB	1	complement(17018664..17054151)	36,81	43,85	-0,029426829
PADI2	1	complement(17066761..17119451)	0,06	0,04	0,847144352
PADI1	1	17205128..17246007	0,03	0,06	-0,695225492
PADI3	1	17249098..17284233	11,76	10,84	0,340942702
PADI4	1	17308195..17364004	-	-	0,188027316
PADI6	1	17372196..17401699	0,00	-	3,068695355

RCC2	1	complement(17406760..17439677)	48,62	65,70	-0,211292969
ARHGEF10L	1	17539698..17697874	3,94	7,62	-0,728518761
ACTL8	1	17755333..17827063	0,01	0,01	-0,025382928
IGSF21	1	18107798..18378483	-	-	#N/D
gene:ENSG000	1	18109389..18115861	-	-	#N/D
KLHDC7A	1	18480930..18485974	0,04	0,01	2,314628527
PAX7	1	18630846..18748866	-	-	#N/D
TAS1R2	1	complement(18839599..18859660)	0,01	-	3,967268276
ALDH4A1	1	complement(18871430..18902724)	1,34	0,91	0,785271469
IFFO2	1	complement(18904280..18956676)	5,29	7,09	-0,199701813
UBR4	1	complement(19074510..19210266)	40,43	30,19	0,644303468
EMC1	1	complement(19215660..19251552)	18,06	25,15	-0,254880762
MRTO4	1	19251805..19260128	16,68	21,53	-0,144887323
AKR7L	1	complement(19265982..19274194)	0,11	0,20	-0,661178051
AKR7A3	1	complement(19282573..19288770)	0,26	0,28	0,117033586
AKR7A2	1	complement(19303965..19312144)	27,77	25,62	0,339558481
SLC66A1	1	19312326..19329300	3,91	6,08	-0,414859949
CAPZB	1	complement(19338775..19485539)	15,19	14,97	0,244865347
MICOS10	1	19484403..19629821	5,96	7,30	-0,069448119
NBL1	1	19596979..19658456	25,08	35,18	-0,26532214
MICOS10-NBL1	1	19597067..19656927	0,65	0,60	0,342926853
HTR6	1	19664875..19680966	0,10	0,33	-1,548674239
TMCO4	1	complement(19682240..19799945)	2,19	4,38	-0,775397047
RNF186	1	complement(19814029..19815283)	-	-	0,188027316
OTUD3	1	19882395..19912945	3,54	2,93	0,498618984
PLA2G2E	1	complement(19920009..19923617)	-	-	0,188027316
PLA2G2A	1	complement(19975431..19980416)	0,02	0,03	-0,025382928
PLA2G5	1	20028179..20091911	0,09	0,44	-2,052125812
PLA2G2D	1	complement(20111939..20119566)	0,03	-	5,697545031
PLA2G2F	1	20139323..20150381	0,01	-	3,967268276
PLA2G2C	1	complement(20161253..20186518)	0,01	-	4,913749003
UBXN10	1	20186096..20196050	0,03	0,01	2,493547104
VWA5B1	1	20290875..20359518	0,00	-	3,068695355
CAMK2N1	1	complement(20482391..20486210)	24,66	13,72	1,069505154
MUL1	1	complement(20499448..20508151)	14,30	16,86	-0,01374614
FAM43B	1	20552573..20555020	1,01	0,34	1,796291465
CDA	1	20589086..20618903	1,96	9,30	-2,0223967
PINK1	1	20633458..20651511	36,64	51,98	-0,281687379
DDOST	1	complement(20651767..20661544)	104,20	#####	0,083646802
KIF17	1	complement(20664014..20718017)	0,13	0,15	0,019747239
SH2D5	1	complement(20719731..20732837)	1,41	2,44	-0,570662453
HP1BP3	1	complement(20740266..20787323)	23,78	20,93	0,406897325
EIF4G3	1	complement(20806292..21177285)	9,54	12,66	-0,185546443
ECE1	1	complement(21217247..21345572)	29,21	46,88	-0,459303833
NBPF3	1	21440128..21485005	4,42	5,28	-0,033351886
ALPL	1	21509397..21578410	0,10	0,89	-2,97893343
gene:ENSG000	1	21547404..21554002	-	-	#N/D
RAP1GAP	1	complement(21596221..21669357)	1,50	2,97	-0,758730808

USP48	1	complement(21678298..21783606)	5,80	5,95	0,186490296
LDLRAD2	1	21812265..21825225	0,80	0,81	0,207641439
HSPG2	1	complement(21822244..21937310)	52,45	53,73	0,188192614
CELA3B	1	21977022..21998642	0,01	-	3,068695355
CELA3A	1	22001657..22012542	-	0,01	-2,89252005
gene:ENSG000	1	22025142..22092943	13,24	16,28	-0,074843426
CDC42	1	22052627..22101360	2,57	3,44	-0,197978699
WNT4	1	complement(22117313..22143969)	0,26	0,48	-0,650060631
gene:ENSG000	1	complement(22364630..22366482)	-	-	#N/D
ZBTB40	1	22428838..22531157	4,85	5,35	0,081635936
EPHA8	1	22563489..22603595	0,00	0,01	-0,579815015
C1QA	1	22635077..22639678	-	-	#N/D
gene:ENSG000	1	22636506..22644067	-	-	#N/D
C1QC	1	22643014..22648110	-	-	#N/D
C1QB	1	22652762..22661637	-	-	#N/D
EPHB2	1	22710839..22921500	10,43	13,63	-0,163099087
LACTBL1	1	complement(22953043..22972866)	-	-	#N/D
TEX46	1	complement(23010834..23019100)	0,07	0,07	0,278120973
KDM1A	1	23019443..23083689	11,57	19,71	-0,545978537
LUZP1	1	complement(23084030..23178152)	9,55	10,43	0,096157727
gene:ENSG000	1	complement(23094326..23094376)	-	-	#N/D
HTR1D	1	complement(23191895..23217502)	0,23	1,57	-2,563481375
HNRNPR	1	complement(23303771..23344336)	13,48	21,95	-0,480120281
ZNF436	1	complement(23359448..23369836)	3,54	4,86	-0,23486906
TCEA3	1	complement(23380909..23424748)	0,31	0,50	-0,470153583
ASAP3	1	complement(23428563..23484637)	8,03	10,82	-0,207188005
E2F2	1	complement(23506438..23531233)	1,27	6,39	-2,110864282
ID3	1	complement(23557926..23559501)	173,15	89,89	1,168816031
RPL11	1	23691742..23696835	112,58	#####	0,04450251
ELOA	1	23743448..23762059	9,63	11,83	-0,073731435
PITHD1	1	23778418..23788232	10,11	11,85	-0,005758196
LYPLA2	1	23791110..23795539	30,87	22,94	0,651438866
GALE	1	complement(23795599..23800781)	33,32	43,02	-0,145626853
HMGCL	1	complement(23801885..23838620)	12,68	10,35	0,516582166
FUCA1	1	complement(23845077..23868290)	2,22	2,30	0,172973324
CNR2	1	complement(23870515..23913362)	0,01	0,02	-1,009277154
PNRC2	1	23956839..23963462	7,75	7,80	0,215051332
SRSF10	1	complement(23964347..23980927)	2,88	5,11	-0,604365895
MYOM3	1	complement(24056035..24112135)	0,07	2,41	-4,970804568
IL22RA1	1	complement(24119771..24143140)	0,56	1,13	-0,796838649
IFNLR1	1	complement(24154168..24187959)	0,57	1,77	-1,41880913
GRHL3	1	24199558..24364482	0,02	0,07	-1,733452083
STPG1	1	complement(24356999..24416934)	3,64	5,87	-0,465451296
NIPAL3	1	24415802..24472976	14,31	14,37	0,216656415
RCAN3	1	24502351..24541040	2,29	2,71	-0,024560612
NCMAP	1	24556087..24609328	0,00	0,00	-0,01185201
SRRM1	1	24631716..24673281	26,68	25,70	0,276859817
CLIC4	1	24745382..24844321	19,95	33,14	-0,508781599

RUNX3	1	complement(24899511..24965121)	7,58	10,10	-0,191749227
SYF2	1	complement(25222276..25232502)	3,05	4,79	-0,427918344
RSRP1	1	complement(25242249..25338213)	7,79	6,30	0,529018595
RHD	1	25272393..25330445	0,20	0,28	-0,251343412
TMEM50A	1	25338317..25362361	14,07	26,37	-0,683044611
RHCE	1	complement(25362249..25430192)	0,26	0,09	1,687772256
MACO1	1	25430858..25500209	3,98	4,39	0,083053678
LDLRAP1	1	25543606..25568886	5,91	8,97	-0,379113414
MAN1C1	1	25616791..25784450	0,07	0,08	0,062859924
SELENON	1	25800193..25818221	28,78	45,72	-0,444914365
gene:ENSG000	1	25811470..25823744	-	-	0,188027316
MTFR1L	1	25818640..25832942	6,15	5,76	0,318095085
AUNIP	1	complement(25831913..25859458)	1,84	3,73	-0,79784072
PAQR7	1	complement(25861484..25875708)	6,17	11,27	-0,647660906
STMN1	1	complement(25884181..25906991)	27,78	38,67	-0,25420727
PAFAH2	1	complement(25959767..25998117)	1,38	2,04	-0,342563618
EXTL1	1	26019884..26036464	0,02	0,04	-0,802495947
SLC30A2	1	complement(26037252..26046118)	0,03	0,02	1,106450337
TRIM63	1	complement(26051301..26068436)	-	-	0,188027316
PDIK1L	1	26111165..26125555	0,70	1,38	-0,763718706
FAM110D	1	26159079..26163962	0,07	0,04	1,035876212
C1orf232	1	complement(26164101..26168963)	0,01	0,01	-0,01185201
ZNF593OS	1	complement(26169516..26171832)	0,60	0,79	-0,174454712
ZNF593	1	26169908..26170873	10,59	10,54	0,229787231
CNKSR1	1	26177484..26189884	0,38	0,94	-1,060280938
CATSPER4	1	26190561..26202968	-	0,01	-3,804623889
CEP85	1	26234200..26278808	17,56	16,22	0,337939787
SH3BGRL3	1	26280086..26281522	132,05	#####	0,467283349
UBXN11	1	complement(26281328..26318363)	1,67	2,21	-0,180362768
CD52	1	26317958..26320523	0,02	-	3,068695355
CRYBG2	1	complement(26321698..26360080)	0,00	0,02	-2,854330803
ZNF683	1	complement(26361634..26374522)	-	-	#N/D
LIN28A	1	26410817..26429728	0,04	0,04	-0,038353104
DHDDS	1	26432282..26471306	11,28	6,12	1,105045123
HMGN2	1	26472440..26476642	56,14	61,51	0,091247477
RPS6KA1	1	26529761..26575030	14,76	14,77	0,22189108
ARID1A	1	26693236..26782104	8,27	11,81	-0,291804844
PIGV	1	26787054..26800659	1,58	2,90	-0,656110891
ZDHHC18	1	26826688..26857604	5,70	7,79	-0,22829365
SFN	1	26863149..26864456	0,66	3,35	-2,11757587
GPN2	1	complement(26876132..26890283)	5,73	5,51	0,279600118
GPATCH3	1	complement(26890488..26900467)	2,88	4,18	-0,314291669
NUDC	1	26900238..26946871	26,92	38,55	-0,294860428
NR0B2	1	complement(26911489..26913975)	-	-	0,188027316
KDF1	1	complement(26949562..26960468)	0,03	0,06	-0,698295353
TRNP1	1	26993692..27000886	3,67	13,16	-1,617735449
TENT5B	1	complement(27005020..27012850)	1,17	2,53	-0,893378543
SLC9A1	1	complement(27098809..27166981)	5,96	6,48	0,101919247

WDTC1	1	27234632..27308636	15,27	15,43	0,207937124
TMEM222	1	27322145..27336400	9,68	7,86	0,524188476
SYTL1	1	27342020..27353937	0,11	0,18	-0,496749983
MAP3K6	1	complement(27355184..27366961)	7,15	6,10	0,452686157
FCN3	1	complement(27369110..27374824)	0,04	0,12	-1,239733301
CD164L2	1	complement(27379176..27383333)	-	-	0,188027316
GPR3	1	27392622..27395814	6,23	4,90	0,569976571
WASF2	1	complement(27404230..27490167)	57,46	47,61	0,494286781
AHDC1	1	complement(27534035..27604431)	7,41	5,20	0,734797204
FGR	1	complement(27612064..27635185)	0,01	0,02	-0,345401666
IFI6	1	complement(27666064..27672212)	25,40	11,29	1,393120441
FAM76A	1	27725961..27763116	2,12	2,25	0,136320077
STX12	1	27773219..27824443	5,58	4,72	0,464960147
PPP1R8	1	27830782..27851676	5,61	11,38	-0,797334242
THEMIS2	1	27872543..27886685	4,01	3,09	0,597741156
RPA2	1	complement(27891524..27914746)	9,10	13,62	-0,358620625
SMPDL3B	1	27935000..27959157	0,01	0,17	-3,93781363
XKR8	1	27959588..27968093	4,49	8,32	-0,667749165
EYA3	1	complement(27970344..28088637)	7,87	8,44	0,122746663
PTAFR	1	complement(28147166..28193936)	0,02	0,04	-0,916746549
DNAJC8	1	complement(28199456..28233029)	24,54	28,16	0,02477417
ATP5IF1	1	28236109..28246906	2,92	4,99	-0,550263861
SESN2	1	28259518..28282491	11,49	11,48	0,224967627
MED18	1	28329002..28335965	5,05	7,96	-0,432065476
PHACTR4	1	28369582..28500364	5,89	10,80	-0,653340723
RCC1	1	28505943..28539300	12,27	25,39	-0,826785997
gene:ENSG000	1	28544460..28546542	0,24	0,51	-0,841388213
TRNAU1AP	1	28553085..28578545	2,66	2,91	0,095353195
TAF12	1	complement(28587829..28648291)	1,66	1,88	0,040288885
RAB42	1	28592200..28595449	1,11	4,51	-1,80317965
GMEB1	1	28668778..28719353	2,35	2,43	0,176181168
YTHDF2	1	28736621..28769775	15,70	21,67	-0,241768453
OPRD1	1	28812170..28871267	0,01	0,00	0,677647514
EPB41	1	28887091..29120046	1,28	2,63	-0,817591991
TMEM200B	1	complement(29119429..29123903)	14,50	17,47	-0,045804165
SRSF4	1	complement(29147743..29181900)	23,89	27,74	0,007087539
MECR	1	complement(29192657..29230942)	3,55	4,81	-0,215910138
PTPRU	1	29236516..29326813	6,96	4,51	0,848548513
gene:ENSG000	1	complement(30576655..30576825)	-	-	#N/D
MATN1	1	complement(30711277..30723585)	0,21	0,27	-0,115272106
LAPTM5	1	complement(30732469..30757774)	1,70	0,87	1,190693832
SDC3	1	complement(30869466..30908758)	13,24	8,26	0,904417906
PUM1	1	complement(30931506..31065991)	13,30	16,52	-0,090153505
NKAIN1	1	complement(31179745..31239887)	0,30	0,83	-1,246779113
SNRNP40	1	complement(31259568..31296788)	22,64	30,49	-0,206075107
ZCCHC17	1	31296982..31364953	16,84	14,51	0,437716955
FABP3	1	complement(31365253..31376850)	1,75	1,23	0,724190077
SERINC2	1	31409565..31434680	35,14	71,86	-0,80880061

TINAGL1	1	31576485..31587686	0,01	0,13	-2,914224352
HCRTR1	1	31617686..31632518	0,06	0,20	-1,470436154
PEF1	1	complement(31629866..31644896)	37,37	40,43	0,109575715
COL16A1	1	complement(31652263..31704319)	20,92	47,65	-0,964677353
ADGRB2	1	complement(31727117..31764893)	6,50	11,54	-0,606007923
SPOCD1	1	complement(31790422..31816022)	6,92	14,58	-0,851117045
PTP4A2	1	complement(31906421..31944856)	22,06	33,43	-0,377129646
gene:ENSG000	1	complement(31919563..31938010)	-	-	#N/D
KHDRBS1	1	32013868..32060850	37,61	47,83	-0,123670706
TMEM39B	1	32072031..32102866	10,63	13,93	-0,167205654
KPNA6	1	32108056..32176563	32,78	34,13	0,164779231
TXLNA	1	32179675..32198285	32,12	30,97	0,275325324
CCDC28B	1	32200595..32205453	1,22	2,19	-0,61648817
IQCC	1	32205671..32208682	2,45	3,02	-0,082546605
DCDC2B	1	32209089..32216192	0,45	0,75	-0,517365801
TMEM234	1	complement(32214472..32222359)	2,55	3,54	-0,251010961
EIF3I	1	32221077..32245397	36,25	36,30	0,221062176
FAM167B	1	32247222..32248856	2,20	2,53	0,020321506
LCK	1	32251244..32286165	-	0,01	-2,89252005
HDAC1	1	32292083..32333635	34,94	49,69	-0,285112249
MARCKSL1	1	complement(32333839..32336233)	27,71	23,78	0,443951921
TSSK3	1	32351521..32364312	7,77	4,96	0,870110338
FAM229A	1	complement(32361270..32364278)	1,59	0,97	0,928101031
BSDC1	1	complement(32364633..32394731)	20,82	16,81	0,532053961
gene:ENSG000	1	32465057..32600792	-	0,01	-3,804623889
ZBTB8B	1	32465072..32496686	0,01	0,00	2,805678585
ZBTB8A	1	32539427..32605941	1,17	1,16	0,229308284
ZBTB8OS	1	complement(32600172..32650903)	3,67	4,06	0,077339399
RBBP4	1	32651142..32686211	14,58	14,25	0,255810148
SYNC	1	complement(32679906..32703596)	1,28	2,36	-0,656761074
NHSL3	1	32741830..32774970	8,25	14,07	-0,547834636
YARS1	1	complement(32775237..32818031)	30,12	51,16	-0,541227628
S100PBP	1	32816767..32858875	6,82	9,12	-0,19475444
FNDC5	1	complement(32862268..32872482)	0,17	0,29	-0,520756762
HPCA	1	32885994..32898441	0,15	0,20	-0,249271007
TMEM54	1	complement(32894594..32901438)	14,20	22,07	-0,413761607
RNF19B	1	complement(32936445..32964809)	7,92	10,16	-0,136797213
gene:ENSG000	1	32973553..32974463	0,02	0,13	-2,123038518
AK2	1	complement(33007986..33080996)	15,21	15,17	0,226224507
AZIN2	1	33081104..33123492	0,69	0,48	0,749910281
gene:ENSG000	1	complement(33141871..33143230)	0,27	0,30	0,047199759
TRIM62	1	complement(33145399..33182059)	0,83	0,96	0,007298837
gene:ENSG000	1	33162851..33166298	0,14	0,24	-0,547020484
ZNF362	1	33256492..33300719	2,01	4,06	-0,79215997
A3GALT2	1	complement(33306766..33321098)	-	-	0,188027316
PHC2	1	complement(33323623..33431095)	22,39	20,57	0,345338663
ZSCAN20	1	33472645..33501643	1,64	1,62	0,242349529
CSMD2	1	complement(33513998..34165842)	0,17	0,01	4,926309697

HMGB4	1	33860475..33864791	0,00	-	3,068695355
C1orf94	1	34166883..34219131	0,00	-	3,068695355
SMIM12	1	complement(34712737..34859755)	1,43	1,92	-0,204793188
GJB5	1	34755047..34758512	0,12	0,07	1,046316654
GJB4	1	34759740..34762327	0,06	0,01	2,805678585
GJB3	1	34781214..34786369	0,21	0,04	2,621275023
GJA4	1	34792999..34795747	0,16	0,11	0,847144352
DLGAP3	1	complement(34865436..34929650)	0,01	0,02	-0,820024868
gene:ENSG000	1	complement(34974356..34985313)	0,59	0,57	0,283030801
TMEM35B	1	complement(34981380..34985347)	3,23	4,24	-0,169625651
gene:ENSG000	1	complement(34981533..35031741)	3,19	3,51	0,085810763
ZMYM6	1	complement(34986165..35031945)	0,64	0,89	-0,258012617
ZMYM1	1	35032172..35115859	1,46	3,15	-0,891291495
SFPQ	1	complement(35176378..35193446)	16,98	33,13	-0,740827733
ZMYM4	1	35268709..35422058	5,77	9,01	-0,418984385
KIAA0319L	1	complement(35393883..35557950)	6,11	8,30	-0,218667165
NCDN	1	35557473..35567274	14,95	8,10	1,107220643
TFAP2E	1	35573314..35595328	0,41	0,27	0,860254673
PSMB2	1	complement(35599541..35641526)	30,46	43,99	-0,307254171
C1orf216	1	complement(35713877..35718894)	4,37	6,16	-0,271699281
CLSPN	1	complement(35720218..35769978)	2,90	3,96	-0,226555386
AGO4	1	35808016..35857890	1,32	2,02	-0,393235374
AGO1	1	35869808..35930532	6,12	7,03	0,02312709
AGO3	1	35930718..36072500	1,42	1,63	0,024861241
TEKT2	1	36084094..36088275	0,01	0,07	-3,171495545
ADPRS	1	36088892..36093932	11,93	11,07	0,330840686
COL8A2	1	complement(36095239..36125222)	0,47	2,17	-1,976773296
TRAPPC3	1	complement(36136570..36156053)	22,02	23,02	0,159266663
MAP7D1	1	36155579..36180849	51,95	56,96	0,090238603
THRAP3	1	36224432..36305357	27,54	32,96	-0,036329273
SH3D21	1	36306368..36329340	0,94	1,37	-0,329711321
EVA1B	1	complement(36322030..36324154)	0,90	0,38	1,486447129
STK40	1	complement(36339624..36385924)	7,08	7,07	0,225058839
LSM10	1	complement(36391238..36397908)	21,60	22,37	0,172607034
OSCP1	1	complement(36415827..36450451)	1,09	0,81	0,650093208
MRPS15	1	complement(36455718..36464384)	34,71	44,11	-0,122677537
CSF3R	1	complement(36466043..36483278)	0,00	0,01	-0,923955849
GRIK3	1	complement(36795527..37034515)	-	-	0,188027316
ZC3H12A	1	37474580..37484377	8,81	25,07	-1,284927251
MEAF6	1	complement(37489993..37514766)	5,68	6,83	-0,041860999
SNIP1	1	complement(37534449..37554293)	4,14	3,09	0,645757335
DNALI1	1	37556919..37566857	0,29	0,18	0,897381942
GNL2	1	complement(37566816..37595937)	28,79	20,43	0,718094194
RSPO1	1	complement(37611350..37634892)	0,01	0,00	1,833201638
AIRIM	1	complement(37681570..37692249)	7,77	7,59	0,256576225
CDCA8	1	37692481..37709719	23,91	31,54	-0,176261642
EPHA10	1	complement(37713880..37765133)	0,00	0,01	-0,429837071
MANEAL	1	37793847..37801137	3,13	4,65	-0,348280592

YRDC	1	complement(37802945..37808208)	19,77	18,66	0,306492771
C1orf122	1	37806979..37809454	6,32	6,74	0,130015054
MTF1	1	complement(37809574..37859592)	5,22	4,48	0,44280709
INPP5B	1	complement(37860697..37947053)	1,72	3,11	-0,632697025
SF3A3	1	complement(37956975..37990075)	25,77	28,74	0,065884412
FHL3	1	complement(37996770..38005606)	10,14	10,50	0,172428255
UTP11	1	38009258..38024820	10,08	12,44	-0,07997877
POU3F1	1	complement(38043829..38046793)	0,01	0,02	-0,742377908
RRAGC	1	complement(38838198..38859772)	3,84	5,44	-0,278222468
MYCBP	1	complement(38862493..38873368)	3,35	4,43	-0,179479462
gene:ENSG000	1	complement(38864501..38881617)	0,02	0,04	-0,509043067
GJA9	1	complement(38874069..38881587)	0,06	0,25	-1,912098279
RHBDL2	1	complement(38885807..38941830)	0,57	0,54	0,317429213
AKIRIN1	1	38991276..39006059	16,21	20,18	-0,093467956
NDUFS5	1	39026318..39034636	30,34	39,17	-0,145493852
MACF1	1	39081316..39487177	7,79	9,82	-0,109930152
BMP8A	1	39491636..39529869	0,44	1,08	-1,064978039
PABPC4	1	complement(39560709..39576790)	37,06	40,36	0,099804899
HEYL	1	complement(39623435..39639643)	0,33	0,24	0,706799617
NT5C1A	1	complement(39651229..39672107)	0,02	0,01	1,675491804
HPCAL4	1	complement(39678648..39691485)	0,02	0,03	-0,35396495
PPIE	1	39692182..39763914	5,87	5,53	0,309788097
BMP8B	1	complement(39757182..39788865)	0,86	1,00	0,012623706
OXCT2	1	complement(39769523..39771348)	0,18	0,20	0,10636078
TRIT1	1	complement(39838110..39883511)	1,00	1,58	-0,430427212
MYCL	1	complement(39895428..39902256)	0,01	0,09	-2,849607196
MFSD2A	1	39955112..39969968	15,54	8,77	1,048615125
CAP1	1	40040233..40072649	80,86	78,59	0,264134506
PPT1	1	complement(40072710..40097260)	28,35	39,04	-0,238914378
RLF	1	40161387..40240921	3,28	3,47	0,141265692
TMCO2	1	40245947..40251684	0,02	-	3,068695355
ZMPSTE24	1	40258041..40294180	3,65	4,93	-0,208937923
COL9A2	1	complement(40300489..40317813)	0,01	0,01	0,366665713
SMAP2	1	40344850..40423326	72,00	41,27	1,026109081
ZFP69B	1	40450102..40463718	0,44	0,85	-0,729396448
gene:ENSG000	1	40473055..40474059	0,04	0,08	-0,602143793
ZFP69	1	40477290..40496343	0,15	0,46	-1,395469483
EXO5	1	40508741..40516038	0,48	1,26	-1,178319922
ZNF684	1	40531573..40548167	0,45	0,42	0,349679232
RIMS3	1	complement(40620680..40665682)	1,89	1,71	0,366959971
NFYC	1	40691648..40771603	5,44	8,07	-0,345865995
KCNQ4	1	40783787..40840452	0,04	0,03	0,496306595
CITED4	1	complement(40861054..40862363)	6,72	15,23	-0,957956509
CTPS1	1	40979300..41012565	7,29	9,82	-0,206163313
SLFNL1	1	complement(41015589..41023237)	1,46	1,95	-0,190332776
SCMH1	1	complement(41027202..41242306)	7,36	13,04	-0,603338448
FOXO6	1	41361922..41383590	0,56	0,50	0,385526129
EDN2	1	complement(41478775..41484683)	0,04	0,06	-0,291161992

HIVEP3	1	complement(41506365..42035925)	0,20	0,48	-1,061652664
gene:ENSG000	1	complement(41585306..41628816)	-	-	#N/D
GUCA2B	1	42153410..42155820	-	-	#N/D
GUCA2A	1	complement(42162690..42164745)	-	-	#N/D
FOXJ3	1	complement(42176539..42335877)	8,02	10,93	-0,223923505
RIMKLA	1	42380792..42424232	-	0,01	-5,734998842
ZMYND12	1	complement(42430329..42456253)	0,02	0,03	-0,032860829
PPCS	1	42456117..42473385	2,03	4,63	-0,965066804
CCDC30	1	42463221..42657190	0,03	0,08	-1,211223407
PPIH	1	42658335..42676758	5,01	6,42	-0,133615524
YBX1	1	42682418..42703805	64,44	86,28	-0,198064558
CLDN19	1	complement(42733093..42740254)	0,02	0,02	-0,03547153
P3H1	1	complement(42746335..42767084)	39,26	29,01	0,659456546
C1orf50	1	42767245..42779491	0,87	0,70	0,536617506
gene:ENSG000	1	42767292..42794493	0,48	0,67	-0,262262901
TMEM269	1	42784991..42816619	0,09	0,03	1,861974871
SVBP	1	complement(42807052..42817397)	1,58	2,31	-0,320456619
ERMAP	1	42817122..42844991	2,13	3,56	-0,519555248
ZNF691	1	42846573..42852477	1,83	3,50	-0,709239189
SLC2A1	1	complement(42925353..42958893)	31,95	30,92	0,270356523
CFAP144	1	43145153..43156396	-	-	0,188027316
EBNA1BP2	1	complement(43164175..43270936)	19,73	25,68	-0,157119959
CFAP57	1	43172330..43254358	0,04	0,07	-0,435819897
TMEM125	1	43269983..43274002	0,02	-	4,913749003
C1orf210	1	complement(43281877..43285617)	0,01	0,01	0,886720911
TIE1	1	43300982..43323108	0,10	0,13	-0,115669387
MPL	1	43337818..43354466	0,02	0,06	-1,024862207
CDC20	1	43358981..43363203	92,89	99,56	0,123017746
ELOVL1	1	complement(43363398..43368074)	45,15	45,30	0,218295399
MED8	1	complement(43383917..43389808)	40,10	28,85	0,698166201
SZT2	1	43389882..43454247	6,38	7,06	0,077148764
HYI	1	complement(43450989..43453989)	0,67	1,87	-1,243691824
PTPRF	1	43525187..43623666	13,27	49,88	-1,686722163
KDM4A	1	43650149..43705518	7,39	9,43	-0,12916385
gene:ENSG000	1	43650149..43931014	-	-	0,188027316
ST3GAL3	1	43705824..43931165	2,31	3,24	-0,268075824
ARTN	1	43933320..43937240	0,32	0,75	-1,020619595
IPO13	1	43946950..43968022	10,91	11,78	0,113122083
DPH2	1	43970000..43973369	22,46	14,59	0,845087403
ATP6V0B	1	43974487..43978295	25,41	20,53	0,530347338
B4GALT2	1	43978943..43991170	20,92	19,95	0,291987704
CCDC24	1	43991359..43996528	2,21	2,37	0,120536862
SLC6A9	1	complement(43991500..44031467)	3,14	7,16	-0,967135545
KLF17	1	44118821..44135140	0,49	0,62	-0,128626792
KLF18	1	complement(44137821..44141631)	0,02	0,02	-0,034419864
DMAP1	1	44213455..44220681	4,46	6,18	-0,248517812
ERI3	1	complement(44221070..44355260)	23,50	28,88	-0,07423927
RNF220	1	44405194..44651723	8,42	14,74	-0,584684266

TMEM53	1	complement(44635238..44674481)	1,19	1,54	-0,143226965
ARMH1	1	44674692..44725591	0,04	0,02	1,106450337
KIF2C	1	44739818..44767767	44,11	50,19	0,036839827
RPS8	1	44775251..44778779	608,03	#####	-0,053585128
BEST4	1	complement(44783585..44788170)	0,04	0,03	0,686693064
PLK3	1	44800377..44805990	4,81	4,26	0,398274349
DYNLT4	1	complement(44805893..44807351)	0,74	0,70	0,308239503
BTBD19	1	44808523..44815585	2,46	3,20	-0,156131922
PTCH2	1	complement(44819844..44843253)	0,14	0,27	-0,733207555
EIF2B3	1	complement(44850522..44986722)	9,29	10,64	0,026303966
HECTD3	1	complement(45002540..45011324)	10,01	7,64	0,613847126
UROD	1	45010950..45015575	9,01	10,74	-0,031462221
ZSWIM5	1	complement(45016399..45306209)	0,40	0,12	1,956634751
HPDL	1	45326895..45328710	0,61	1,65	-1,218823184
MUTYH	1	complement(45329163..45340893)	1,03	3,05	-1,341264525
gene:ENSG000	1	complement(45329262..45491155)	-	-	0,188027316
TOE1	1	45340052..45343973	3,17	6,59	-0,833823749
TESK2	1	complement(45343883..45491163)	1,13	0,94	0,484485545
CCDC163	1	complement(45493866..45500079)	1,12	1,20	0,123653723
MMACHC	1	45500300..45513382	1,88	2,65	-0,272077443
PRDX1	1	complement(45510914..45542732)	237,37	#####	0,472926561
AKR1A1	1	45550543..45570049	22,04	18,13	0,504880626
NASP	1	45583846..45618904	7,91	11,29	-0,289517892
CCDC17	1	complement(45620044..45624057)	0,10	0,28	-1,211653859
GPBP1L1	1	complement(45627304..45688113)	22,05	26,63	-0,049180253
TMEM69	1	45688181..45694436	6,86	10,09	-0,333969908
IPP	1	complement(45694324..45750653)	1,85	2,56	-0,244179355
MAST2	1	45786987..46036122	15,21	19,63	-0,145134299
PIK3R3	1	complement(46040140..46133036)	0,31	0,40	-0,163275632
P3R3URF-PIK3I	1	complement(46043661..46176488)	-	-	0,188027316
TSPAN1	1	46175073..46185962	0,25	0,37	-0,314763249
P3R3URF	1	complement(46175486..46176488)	-	-	#N/D
POMGNT1	1	complement(46188683..46220305)	19,95	19,90	0,226819224
LURAP1	1	46203334..46221256	0,05	0,02	1,505976878
RAD54L	1	46246461..46278480	11,84	10,53	0,392281113
LRRC41	1	complement(46261196..46303616)	18,27	23,37	-0,132283725
UQCRH	1	46303698..46316776	89,75	97,82	0,09894891
NSUN4	1	46340789..46365152	3,50	4,06	0,005967004
FAAH	1	46394317..46413848	0,35	0,51	-0,313650454
DMBX1	1	46489836..46516216	3,13	1,84	0,987847332
TMEM275	1	complement(46532166..46543969)	0,01	0,01	0,886720911
KNCN	1	complement(46545641..46551647)	-	0,00	-2,89252005
MKNK1	1	complement(46557407..46616843)	1,75	2,51	-0,296163526
MOB3C	1	complement(46607719..46616811)	2,07	2,12	0,190330367
ATPAF1	1	complement(46632737..46673867)	5,06	7,34	-0,314747948
TEX38	1	46668855..46673594	0,13	0,12	0,320475827
EFCAB14	1	complement(46674659..46719146)	14,36	11,42	0,553972104
CYP4B1	1	46757838..46819413	-	-	#N/D

CYP4A11	1	complement(46929177..46941484)	-	-	#N/D
CYP4X1	1	47023669..47050751	0,01	0,01	0,886720911
CYP4Z1	1	47067231..47118318	-	-	0,188027316
CYP4A22	1	47137435..47149727	-	0,02	-5,071123353
PDZK1IP1	1	complement(47183582..47191044)	0,81	5,92	-2,642447219
TAL1	1	complement(47216290..47232225)	0,00	-	3,068695355
STIL	1	complement(47250139..47314892)	7,12	7,58	0,132904637
CMPK1	1	47333790..47394866	6,70	9,64	-0,302758391
FOXE3	1	47416285..47418052	0,03	0,01	2,144183439
FOXD2	1	47438044..47440691	0,40	0,67	-0,508681738
TRABD2B	1	complement(47760528..47997385)	1,09	0,47	1,432851591
gene:ENSG000	1	48102068..48104736	-	-	0,188027316
SLC5A9	1	48222685..48248644	0,03	-	6,053060169
gene:ENSG000	1	complement(48262230..48263179)	-	-	#N/D
SPATA6	1	complement(48295373..48472208)	0,42	0,09	2,425031254
AGBL4	1	complement(48532854..50023954)	-	-	0,188027316
BEND5	1	complement(48727519..48776969)	-	-	#N/D
gene:ENSG000	1	49994318..49996218	-	-	#N/D
ELAVL4	1	50024029..50203772	-	-	0,188027316
DMRTA2	1	complement(50417550..50423443)	-	0,02	-5,071123353
FAF1	1	complement(50437028..50960267)	6,14	8,57	-0,25736889
CDKN2C	1	50960745..50974634	6,57	6,17	0,313963611
C1orf185	1	51102221..51148086	-	-	0,188027316
RNF11	1	51236273..51273447	9,06	9,25	0,19272771
TTC39A	1	complement(51287258..51345116)	0,01	0,07	-2,084873543
EPS15	1	complement(51354263..51519328)	5,44	9,35	-0,559402299
OSBPL9	1	51577179..51798427	2,95	6,00	-0,798572959
NRDC	1	complement(51789191..51878805)	24,60	36,44	-0,343481204
RAB3B	1	complement(51907956..51990700)	0,96	1,04	0,112537101
TXNDC12	1	complement(52020131..52055772)	12,88	22,48	-0,580425077
gene:ENSG000	1	complement(52020153..52033350)	-	0,12	-6,89165328
KTI12	1	complement(52032103..52033810)	4,55	6,44	-0,280261628
BTF3L4	1	52056199..52090716	2,43	5,79	-1,031075836
ZFYVE9	1	52142089..52348671	3,43	5,19	-0,371852782
CC2D1B	1	complement(52345723..52366205)	7,29	8,59	-0,014584748
ORC1	1	complement(52372829..52404423)	7,08	7,13	0,214008914
PRPF38A	1	52404602..52420836	6,92	5,50	0,553762157
TUT4	1	complement(52408282..52553487)	3,27	5,73	-0,585762531
GPX7	1	52602371..52609051	6,91	6,55	0,300374804
SHISAL2A	1	52633168..52669683	-	-	0,188027316
COA7	1	complement(52684449..52698347)	2,64	4,43	-0,524740313
ZYG11B	1	52726453..52827336	4,77	5,49	0,018779818
ZYG11A	1	52842511..52894998	3,12	5,08	-0,480889884
ECHDC2	1	complement(52895910..52927212)	2,69	3,86	-0,29799993
SCP2	1	52927276..53051698	3,34	4,22	-0,115835215
PODN	1	53062052..53085501	0,01	0,01	-0,025382928
SLC1A7	1	complement(53087179..53142638)	0,01	0,04	-1,700542576
CPT2	1	53196792..53214197	3,08	6,48	-0,848103463

CZIB	1	complement(53214099..53220634)	12,58	14,97	-0,027333144
MAGOH	1	complement(53226900..53238518)	4,67	7,24	-0,410501092
LRP8	1	complement(53242364..53328469)	9,04	6,65	0,66622943
gene:ENSG000	1	complement(53413149..53440018)	-	-	#N/D
DMRTB1	1	53459399..53467488	-	-	0,188027316
GLIS1	1	complement(53506237..53739164)	2,08	2,42	0,004585342
NDC1	1	complement(53765478..53838463)	9,63	14,92	-0,408551486
YIPF1	1	complement(53851719..53889798)	4,70	6,15	-0,165183408
DIO1	1	53891239..53911086	0,44	0,35	0,52541098
IFT25	1	complement(53916574..53945699)	1,91	3,04	-0,448094599
LRRC42	1	53946077..53968168	15,49	16,11	0,166536215
LDLRAD1	1	complement(54007298..54018186)	-	0,01	-2,89252005
TMEM59	1	complement(54026681..54053504)	5,77	5,86	0,200070675
gene:ENSG000	1	complement(54033126..54034880)	0,37	0,28	0,61677221
TCEANC2	1	54053584..54112519	1,05	1,05	0,219716385
gene:ENSG000	1	complement(54132686..54200073)	0,02	0,03	-0,392761763
CDCP2	1	complement(54132687..54153770)	0,03	0,00	2,972512804
CYB5RL	1	complement(54169651..54200036)	1,52	1,55	0,198229834
MRPL37	1	54184041..54225464	49,37	49,93	0,206796188
SSBP3	1	complement(54225432..54413479)	6,85	4,96	0,687373911
ACOT11	1	54542257..54639192	0,18	0,46	-1,131890617
FAM151A	1	complement(54609181..54623556)	0,01	0,03	-0,979341556
MROH7	1	54641754..54710266	0,02	0,00	2,972512804
MROH7-TTC4	1	54641786..54742308	0,03	0,01	1,520939857
TTC4	1	54715861..54742657	6,88	14,00	-0,802328322
PARS2	1	complement(54756898..54764523)	2,39	4,13	-0,562709095
TTC22	1	complement(54779712..54801323)	-	-	0,188027316
CIMAP2	1	54806063..54842252	0,08	0,01	2,583287272
DHCR24	1	complement(54849627..54887195)	16,12	14,42	0,382957358
TMEM61	1	54886800..54992293	-	0,02	-4,359055975
BSND	1	54998933..55017172	0,00	-	3,068695355
PCSK9	1	55039445..55064852	0,00	0,00	0,886720911
USP24	1	complement(55066359..55215378)	7,04	11,23	-0,449429285
gene:ENSG000	1	complement(56173433..56524495)	0,04	0,08	-0,858027988
PLPP3	1	complement(56494761..56645301)	2,51	5,10	-0,798005366
PRKAA2	1	56645314..56715335	0,57	1,56	-1,236723366
FYB2	1	complement(56718789..56819696)	-	-	0,188027316
C8A	1	56854768..56918223	-	-	#N/D
C8B	1	complement(56929207..56974383)	-	-	#N/D
DAB1	1	complement(56994778..58546734)	-	-	#N/D
OMA1	1	complement(58415384..58546802)	1,29	2,43	-0,697911586
TACSTD2	1	complement(58575433..58577252)	0,04	0,08	-0,895537122
MYSM1	1	complement(58643440..58700090)	2,87	2,89	0,213704415
JUN	1	complement(58776845..58784048)	66,03	68,67	0,166548558
FGGY	1	59296638..59810647	0,50	0,63	-0,103937769
HOOK1	1	59814786..59876322	0,01	0,00	2,805678585
CYP2J2	1	complement(59893308..59926773)	0,38	0,01	5,038586073
C1orf87	1	complement(59987269..60073770)	-	-	0,188027316

NFIA	1	60865259..61462788	0,44	1,59	-1,6297186
TM2D1	1	complement(61681046..61725423)	1,47	1,58	0,12508369
PATJ	1	61742477..62178675	1,72	2,12	-0,076694796
L1TD1	1	62194849..62212328	0,01	-	4,516772761
KANK4	1	complement(62236165..62319434)	0,00	-	3,068695355
USP1	1	62436297..62451804	3,11	6,09	-0,747179961
DOCK7	1	complement(62454298..62688386)	2,25	4,46	-0,764546869
ANGPTL3	1	62597520..62606313	-	0,02	-4,758582517
gene:ENSG000	1	62607766..62609584	0,01	0,06	-2,123038518
ATG4C	1	62784132..62865516	0,95	1,26	-0,185334727
FOXD3	1	63322567..63325128	0,09	0,45	-2,049089959
ALG6	1	63367575..63438553	0,33	0,97	-1,338764754
ITGB3BP	1	complement(63440770..63593721)	2,49	2,96	-0,02292166
EFCAB7	1	63523372..63572693	0,45	1,18	-1,160932741
PGM1	1	63593411..63660245	14,71	21,17	-0,301816043
ROR1	1	63774017..64181498	0,21	2,78	-3,483495637
UBE2U	1	64203623..64267368	-	-	#N/D
CACHD1	1	64470129..64693058	1,02	0,73	0,714718624
RAVER2	1	64745075..64833232	1,60	2,52	-0,433720424
JAK1	1	complement(64833223..65067754)	10,90	15,74	-0,306911399
AK4	1	65147549..65232145	2,82	4,97	-0,591104229
DNAJC6	1	65248219..65415871	0,19	0,89	-1,976368803
LEPROT	1	65420587..65436007	4,27	5,25	-0,076097095
LEPR	1	65420652..65641559	1,02	1,23	-0,049189047
PDE4B	1	65792514..66374579	2,09	2,78	-0,190511586
SGIP1	1	66533267..66751139	0,00	0,00	-0,03031053
DYNLT5	1	66752459..66779047	-	-	0,188027316
INSL5	1	complement(66797740..66801276)	-	-	#N/D
DNAI4	1	complement(66812885..66924856)	0,03	0,04	-0,116491265
MIER1	1	66924895..66988619	1,26	1,78	-0,279112256
SLC35D1	1	complement(66999350..67054148)	4,23	4,25	0,218474715
C1orf141	1	complement(67092165..67231853)	-	-	#N/D
IL23R	1	67138907..67259979	-	-	0,188027316
IL12RB2	1	67307364..67398724	0,09	0,12	-0,312517757
SERBP1	1	complement(67407810..67430415)	14,20	24,68	-0,574272165
GADD45A	1	67685201..67688334	5,33	14,66	-1,23722427
GNG12	1	complement(67701475..67833467)	36,73	44,09	-0,040398823
DIRAS3	1	complement(68045886..68051717)	0,04	0,07	-0,480718451
WLS	1	complement(68098473..68233120)	18,40	18,01	0,253892339
RPE65	1	complement(68428822..68451103)	0,02	0,00	2,972512804
DEPDC1	1	complement(68474152..68497221)	2,97	5,95	-0,78064598
LRRC7	1	69567922..70151945	0,00	0,01	-0,659786428
LRRC40	1	complement(70144805..70205579)	1,32	3,18	-1,050863346
SRSF11	1	70205682..70253052	10,96	14,18	-0,148922055
ANKRD13C	1	complement(70258999..70354734)	2,03	2,70	-0,189723912
CTH	1	70411218..70439851	0,41	0,36	0,391652203
PTGER3	1	complement(70852353..71047816)	0,05	-	8,477495833
ZRANB2	1	complement(71063291..71081289)	13,21	11,50	0,423647614

NEGR1	1	complement(71395943..72282539)	0,07	0,07	0,21219198
gene:ENSG000	1	complement(72979014..72979314)	-	-	#N/D
LRRIQ3	1	complement(74026015..74198187)	0,07	0,06	0,457552023
FPGT-TNNI3K	1	74198235..74544393	0,14	0,04	2,140592446
FPGT	1	74198238..74234086	0,51	0,71	-0,236360587
TNNI3K	1	74235387..74544428	0,04	-	6,68162262
LRRC53	1	complement(74469376..74512611)	0,00	-	3,068695355
ERICH3	1	complement(74568117..74673792)	0,00	-	3,967268276
CRYZ	1	complement(74705482..74733408)	6,73	7,03	0,159919916
TYW3	1	74733152..74766678	3,55	3,98	0,058018119
LHX8	1	75128434..75161533	0,46	0,01	5,278680762
SLC44A5	1	complement(75202129..75611114)	0,05	0,00	4,486751555
ACADM	1	75724431..75787575	2,19	3,04	-0,252120176
RABGGTB	1	75786197..75795086	10,40	18,33	-0,594470689
MSH4	1	75796882..75913242	0,01	0,07	-2,165931592
ASB17	1	complement(75918873..75932404)	-	-	#N/D
ST6GALNAC3	1	76074746..76634603	0,12	0,04	1,893972143
ST6GALNAC5	1	76867480..77067546	-	-	0,188027316
PIGK	1	complement(77088989..77219430)	3,10	6,11	-0,755952402
AK5	1	77282019..77559966	5,08	4,39	0,434946945
ZZZ3	1	complement(77562416..77683419)	4,50	7,49	-0,512870846
USP33	1	complement(77695987..77759852)	5,61	9,32	-0,509887819
MIGA1	1	77779624..77879540	3,58	3,45	0,278339902
NEXN	1	77888513..77943895	0,37	0,64	-0,574417477
FUBP1	1	complement(77944055..77979110)	15,57	16,01	0,183599439
DNAJB4	1	77979175..78017964	2,26	2,55	0,050297337
GIPC2	1	77979542..78138444	-	0,01	-4,758582517
PTGFR	1	78303884..78540701	3,49	1,09	1,90118513
IFI44L	1	78619902..78646145	1,78	0,53	1,972145746
IFI44	1	78649796..78664078	19,78	15,60	0,565867113
ADGRL4	1	complement(78889764..79282124)	0,68	0,44	0,854555827
ADGRL2	1	81306147..81993932	1,32	4,68	-1,605738476
TTLL7	1	complement(83865024..83999150)	0,34	0,77	-0,966420081
PRKACB	1	84078062..84238498	1,84	2,35	-0,131398639
SAMD13	1	84298366..84389957	0,08	0,32	-1,826065601
DNASE2B	1	84398484..84415018	-	0,03	-4,758582517
RPF1	1	84479259..84498352	4,30	8,08	-0,687089198
GNG5	1	complement(84498323..84506581)	7,07	8,46	-0,035642117
SPATA1	1	84506300..84567379	0,06	0,15	-1,034083043
CTBS	1	complement(84549611..84574480)	2,13	2,01	0,310684095
SSX2IP	1	complement(84643706..84690803)	3,19	2,86	0,380168608
LPAR3	1	complement(84811602..84893206)	9,57	3,78	1,562944463
MCOLN2	1	complement(84925583..84997113)	1,67	1,51	0,365963631
DNAI3	1	84999147..85133138	0,10	0,05	1,254956342
MCOLN3	1	complement(85018082..85048500)	0,59	0,91	-0,396392012
gene:ENSG000	1	complement(85152487..85154203)	0,55	0,68	-0,072878372
SYDE2	1	complement(85156889..85201724)	0,64	0,98	-0,40015857
C1orf52	1	complement(85249953..85259672)	2,23	2,28	0,193581018

BCL10	1	complement(85265776..85276632)	6,33	5,70	0,374729101
DDAH1	1	complement(85318481..85578363)	2,62	5,29	-0,79038442
CCN1	1	85580761..85584589	312,48	#####	0,40999061
ZNHIT6	1	complement(85649417..85708433)	2,62	3,34	-0,129048005
COL24A1	1	complement(85729233..86156943)	0,02	0,04	-0,533146901
ODF2L	1	complement(86346824..86396342)	0,59	0,63	0,109587967
CLCA2	1	86424171..86456553	0,01	0,02	-0,602143793
CLCA1	1	86468368..86500259	-	-	#N/D
CLCA4	1	86547078..86580754	-	-	#N/D
SH3GLB1	1	86704570..86748184	9,33	8,74	0,318311589
SELENOF	1	complement(86862445..86914424)	17,91	22,92	-0,132158505
HS2ST1	1	86914635..87109982	2,53	3,52	-0,251169131
gene:ENSG000	1	86993009..87169204	0,05	0,06	-0,187130731
LMO4	1	87328880..87348923	8,18	8,94	0,095145647
gene:ENSG000	1	complement(87805286..87808372)	0,72	0,78	0,099504141
PKN2	1	88684222..88836255	4,71	6,91	-0,330010286
GTF2B	1	complement(88852633..88891944)	6,77	6,45	0,294197029
KYAT3	1	complement(88935773..88992953)	1,68	2,82	-0,522138795
RBMXL1	1	complement(88979456..88992960)	1,85	1,89	0,196979268
GBP3	1	complement(89006679..89022894)	4,16	2,92	0,73455649
GBP1	1	complement(89051882..89065360)	3,86	4,66	-0,049095468
GBP2	1	complement(89106132..89150456)	7,46	3,46	1,331887568
GBP7	1	complement(89131742..89176009)	-	-	0,188027316
GBP4	1	complement(89181144..89198942)	0,90	0,22	2,288053589
GBP5	1	complement(89256189..89272860)	0,40	0,14	1,684089349
GBP6	1	89364059..89388160	0,00	-	3,068695355
LRRC8B	1	89524829..89597861	0,25	1,01	-1,791243573
gene:ENSG000	1	89579592..89579654	-	-	#N/D
LRRC8C	1	89633072..89769903	2,17	2,37	0,092456093
gene:ENSG000	1	89633140..89933250	-	0,06	-4,359055975
LRRC8D	1	89821014..89936611	2,58	8,80	-1,54945687
ZNF326	1	89995110..90035533	0,75	1,78	-1,027416998
BARHL2	1	complement(90711539..90717302)	0,01	0,04	-1,766505595
ZNF644	1	complement(90915298..91022272)	2,56	4,24	-0,503754424
HFM1	1	complement(91260766..91404856)	0,00	-	3,068695355
CDC7	1	91500851..91525764	2,08	3,56	-0,552900067
TGFBR3	1	complement(91680343..91906335)	1,46	3,26	-0,935320884
BRDT	1	91949343..92014426	-	-	0,188027316
EPHX4	1	92029985..92063538	0,11	-	6,68162262
SETSIP	1	complement(92074533..92075411)	-	-	#N/D
BTBD8	1	92080305..92184725	0,30	0,58	-0,715888502
C1orf146	1	92217915..92245813	-	-	0,188027316
GLMN	1	complement(92246402..92298987)	0,24	0,77	-1,456487983
RPAP2	1	92299059..92402056	0,22	0,55	-1,106453702
GFI1	1	complement(92473043..92486925)	0,01	0,27	-4,073522345
EVI5	1	complement(92508696..92792404)	2,30	3,45	-0,364657567
RPL5	1	92832013..92841924	222,47	#####	-0,013073709
DIPK1A	1	complement(92832737..92961522)	2,18	3,28	-0,366389634

MTF2	1	93079235..93139079	2,27	3,81	-0,521462861
TMED5	1	complement(93149742..93180516)	2,66	4,37	-0,493918139
CCDC18	1	93179919..93278730	1,21	1,29	0,123104561
DR1	1	93345907..93369493	3,99	5,68	-0,287555467
FNBP1L	1	93448118..93554661	3,69	4,09	0,072570447
BCAR3	1	complement(93561741..93847150)	18,37	20,46	0,067816974
DNTTIP2	1	complement(93866284..93879918)	2,14	3,37	-0,431085235
GCLM	1	complement(93885199..93909456)	6,28	12,14	-0,727718394
ABCA4	1	complement(93992834..94121148)	0,04	0,33	-2,900658071
ARHGAP29	1	complement(94148988..94275068)	27,62	22,71	0,504979665
ABCD3	1	94418389..94518666	4,72	7,52	-0,449474916
F3	1	complement(94529173..94541759)	2,34	2,28	0,263777085
SLC44A3	1	94820342..94895246	0,03	0,12	-1,79450424
CNN3	1	complement(94896949..94927223)	23,24	24,52	0,145924935
ALG14	1	complement(94974405..95072951)	0,42	0,57	-0,223402815
TLCD4	1	95117355..95197607	0,02	0,63	-4,791263866
TLCD4-RWDD3	1	95117923..95247225	0,09	0,39	-1,912702987
RWDD3	1	95234210..95247225	0,85	1,42	-0,518783434
PTBP2	1	96721665..96823738	0,39	0,65	-0,538586735
DPYD	1	complement(97077743..97995000)	15,23	9,28	0,938843755
SNX7-DT	1	complement(98660388..98661059)	-	0,06	-4,359055975
SNX7	1	98661701..98760500	6,92	8,59	-0,089725334
PLPPR5	1	complement(98890245..99227929)	0,00	0,05	-3,929150322
PLPPR4	1	99264292..99309590	0,11	0,53	-2,115975705
PALMD	1	99646113..99694541	0,01	0,52	-5,606102519
FRRS1	1	complement(99703970..99766635)	1,31	1,90	-0,314157322
AGL	1	99850361..99924023	1,04	1,86	-0,608085835
SLC35A3	1	99969351..100035634	0,60	0,59	0,246779963
gene:ENSG000	1	99970011..100083321	1,40	1,69	-0,052362695
MFSD14A	1	100038095..100083377	7,64	8,12	0,134278913
SASS6	1	complement(100083563..10013295	0,89	1,72	-0,732206769
TRMT13	1	100133150..100150496	0,43	1,01	-1,025924733
LRRC39	1	complement(100148448..10017826	0,04	0,07	-0,35396495
DBT	1	complement(100186919..10024983	1,04	1,64	-0,428591982
RTCA	1	100266216..100292769	6,39	10,15	-0,445225836
CDC14A	1	100345001..100520277	0,10	0,37	-1,605322167
GPR88	1	100538139..100542021	0,00	0,01	-0,923955849
VCAM1	1	100719742..100739045	-	-	0,188027316
EXTL2	1	complement(100872372..10089517	2,89	5,09	-0,591372998
SLC30A7	1	100896076..100981757	3,50	3,87	0,079776658
DPH5	1	complement(100989623..10102608	3,55	7,38	-0,833832819
S1PR1	1	101236865..101243713	0,03	0,81	-4,445556825
OLFM3	1	complement(101802560..10199692	-	-	#N/D
COL11A1	1	complement(102876467..10310887	0,02	0,00	3,601075255
RNPC3	1	103525691..103555239	2,81	1,78	0,879192444
AMY2B	1	103553815..103579534	0,10	0,25	-1,097609863
AMY2A	1	103617427..103625780	-	-	#N/D
AMY1A	1	103655760..103664554	-	-	#N/D

AMY1B	1	complement(103687415..10369645	-	-	0,188027316
AMY1C	1	103745323..103758726	-	-	#N/D
PRMT6	1	107056674..107067636	0,54	2,40	-1,937536105
NTNG1	1	107140007..107484923	0,07	0,78	-3,220648684
VAV3	1	complement(107571161..10796518	0,02	0,03	-0,297455117
SLC25A24	1	complement(108134043..10820084	9,26	13,70	-0,34132027
NBPF4	1	complement(108222464..10824408	0,56	3,29	-2,330988883
NBPF6	1	108450282..108471920	0,43	1,82	-1,86321968
EEIG2	1	108560089..108644900	10,64	13,64	-0,134535565
HENMT1	1	complement(108648290..10866152	0,57	1,87	-1,502372274
PRPF38B	1	108692310..108702928	5,75	8,17	-0,284925904
FNDC7	1	108712908..108742749	0,02	0,07	-1,773114872
STXBP3	1	108746674..108809523	3,93	7,92	-0,788501538
AKNAD1	1	complement(108815898..10896348	0,06	0,11	-0,67834146
GPSM2	1	108875350..108934545	14,46	16,18	0,06134432
CLCC1	1	complement(108881885..10896352	6,35	6,72	0,141695536
WDR47	1	complement(108970214..10904211	7,02	7,39	0,149116092
TAF13	1	complement(109062496..10907601	18,26	12,63	0,754863163
TMEM167B	1	109090764..109096934	34,17	27,43	0,540297448
CFAP276	1	complement(109105951..10911385	-	0,01	-2,89252005
ELAPOR1	1	109113679..109206781	0,07	0,22	-1,476323427
SARS1	1	109213918..109238182	84,35	#####	-0,991370904
CELSR2	1	109249539..109275751	2,19	5,83	-1,191822249
PSRC1	1	complement(109279556..10928318	37,11	46,46	-0,101025956
MYBPHL	1	complement(109292365..10930701	-	-	0,188027316
SORT1	1	complement(109309568..10939791	0,04	0,56	-3,618606214
PSMA5	1	complement(109399042..10942644	20,52	26,77	-0,160613757
SYPL2	1	109466546..109482134	0,01	0,04	-1,275310173
ATXN7L2	1	109483479..109492804	3,97	7,30	-0,657055794
CYB561D1	1	109494052..109502932	10,25	5,97	1,003212755
AMIGO1	1	complement(109504178..10950973	0,08	0,07	0,578841284
GPR61	1	109539872..109548406	0,13	0,48	-1,623233658
GNAI3	1	109548615..109600195	21,34	20,48	0,282919184
GNAT2	1	complement(109603091..10961992	0,30	0,61	-0,793441774
AMPD2	1	109616104..109632051	148,58	65,83	1,397578486
GSTM4	1	109656099..109665496	10,06	9,63	0,286546568
GSTM2	1	109668022..109709551	6,54	2,89	1,398015315
GSTM1	1	109687814..109709039	148,33	#####	0,454588736
GSTM5	1	109711780..109775428	2,65	0,72	2,105931571
GSTM3	1	complement(109733932..10974103	18,91	8,14	1,439412651
EPS8L3	1	complement(109750080..10976402	0,02	0,10	-2,316867748
CSF1	1	109910242..109930992	340,58	#####	0,406850207
AHCYL1	1	109984765..110023742	131,11	#####	0,216836822
STRIP1	1	110031577..110074641	14,17	20,95	-0,341026951
ALX3	1	complement(110059870..11007067	8,32	11,34	-0,222808913
UBL4B	1	110112443..110113947	0,45	0,98	-0,894595241
SLC6A17	1	110150494..110202202	2,80	6,70	-1,03473807
KCNC4	1	110210314..110283100	2,03	1,89	0,328189647

RBM15	1	110338506..110346681	30,84	20,39	0,820082805
SLC16A4	1	complement(110362851..11039108	1,52	7,95	-2,160026051
LAMTOR5	1	complement(110401249..11040794	40,40	44,51	0,083414036
PROK1	1	110451149..110457358	0,03	0,01	1,833201638
KCNA10	1	complement(110517217..11051917	-	-	#N/D
KCNA2	1	complement(110519837..11063147	-	-	0,188027316
KCNA3	1	complement(110653560..11067494	-	0,00	-3,804623889
CD53	1	110871188..110899922	0,02	-	4,516772761
LRIF1	1	complement(110947190..11096396	1,01	2,41	-1,025061188
DRAM2	1	complement(111117163..11114020	11,37	13,01	0,02899046
CEPT1	1	111139479..111185104	11,04	15,64	-0,27870992
DENND2D	1	complement(111185969..11120453	0,61	0,20	1,806167066
CHI3L2	1	111200771..111243440	0,53	0,94	-0,594593634
CHIA	1	111290851..111320566	-	-	#N/D
CIMAP3	1	111346600..111353013	0,01	0,01	0,886720911
OVGP1	1	complement(111414319..11142773	1,60	0,48	1,948631618
WDR77	1	complement(111439890..11144925	21,76	34,95	-0,460558335
ATP5PB	1	111448864..111462773	41,05	50,21	-0,06745373
C1orf162	1	111473792..111478512	0,16	0,75	-2,029804394
TMIGD3	1	complement(111483348..11156396	-	-	0,188027316
ADORA3	1	complement(111499429..11150363	-	-	0,188027316
RAP1A	1	111542218..111716691	4,82	4,66	0,270048214
INKA2	1	complement(111680630..11175582	2,22	2,59	0,001924182
DDX20	1	111754832..111775602	1,90	3,35	-0,595517891
KCND3	1	complement(111770662..11198966	0,03	0,02	0,713573923
CTTNBP2NL	1	112396214..112463456	5,79	7,32	-0,114122975
WNT2B	1	112466541..112530165	0,35	0,86	-1,076617241
ST7L	1	complement(112523514..11262082	0,78	1,63	-0,839426761
CAPZA1	1	112619805..112671616	62,26	72,49	0,003665486
MOV10	1	112673141..112700756	18,53	22,03	-0,026753466
RHOC	1	complement(112701127..11270743	83,67	63,15	0,629001888
gene:ENSG000	1	complement(112702614..11271143	0,09	0,05	1,15046174
PPM1J	1	complement(112709994..11271533	1,11	0,76	0,767354284
TAFA3	1	112718905..112727235	0,12	0,28	-1,033707606
SLC16A1	1	complement(112911847..11295759	22,06	24,63	0,063918043
LRIG2	1	113073198..113132260	3,54	3,04	0,444988142
MAGI3	1	113390515..113685923	9,53	5,64	0,979730661
PHTF1	1	complement(113696831..11375948	1,43	3,81	-1,186997705
RSBN1	1	complement(113761832..11381247	2,62	2,61	0,227802446
PTPN22	1	complement(113813811..11387175	0,09	0,79	-2,836989326
BCL2L15	1	complement(113876816..11388758	0,04	0,01	1,699751701
AP4B1	1	complement(113894194..11390571	5,98	3,90	0,838890992
DCLRE1B	1	113904619..113914086	9,00	8,90	0,239413978
HIPK1	1	113929324..113977869	15,42	16,36	0,13777991
OLFML3	1	113979391..114035572	0,04	0,23	-2,227495745
SYT6	1	complement(114089291..11415388	-	-	0,188027316
TRIM33	1	complement(114392790..11451120	2,75	5,38	-0,744721935
BCAS2	1	complement(114567557..11458162	5,18	8,30	-0,457553767

DENND2C	1	complement(114582848..11467042	0,33	0,06	2,708312405
AMPD1	1	complement(114673090..11469561	-	-	#N/D
NRAS	1	complement(114704469..11471677	17,49	17,49	0,223055714
CSDE1	1	complement(114716913..11475867	82,44	#####	-0,185986935
SIKE1	1	complement(114769479..11478068	3,82	4,35	0,038075232
SYCP1	1	114854863..114995370	-	-	#N/D
TSHB	1	115029826..115034302	-	-	0,188027316
TSPAN2	1	complement(115048011..11508950	0,02	0,01	1,704893826
NGF	1	complement(115285904..11533877	0,18	1,18	-2,451915753
VANGL1	1	115641970..115698224	3,63	7,22	-0,768377989
CASQ2	1	complement(115698038..11579319	-	0,00	-3,804623889
NHLH2	1	complement(115836377..11584391	-	-	0,188027316
SLC22A15	1	115976513..116070054	0,51	2,61	-2,131514688
MAB21L3	1	116111399..116138149	0,03	0,23	-2,923227684
ATP1A1	1	116372668..116410261	133,03	#####	0,325640929
CD58	1	complement(116514534..11657103	0,83	2,11	-1,116256757
IGSF3	1	complement(116574399..11666775	10,27	18,25	-0,606870416
CD2	1	116754430..116769229	0,01	-	3,068695355
PTGFRN	1	116909916..116990353	18,30	33,63	-0,65480292
CD101	1	117001750..117036552	0,09	0,25	-1,258499948
TTF2	1	117060326..117107453	5,95	8,41	-0,2770115
TRIM45	1	complement(117111060..11712258	1,59	1,86	-0,004485621
VTCN1	1	complement(117143587..11721096	-	0,01	-3,804623889
MAN1A2	1	117367449..117528872	4,31	5,09	-0,017906004
gene:ENSG000	1	117493515..117495006	0,02	0,05	-0,999120035
TENT5C	1	117606048..117628389	19,86	1,71	3,764296345
GDAP2	1	complement(117863485..11792962	4,46	4,17	0,320176926
WDR3	1	117929720..117966543	4,92	6,59	-0,200629143
SPAG17	1	complement(117953590..11818522	0,01	0,01	0,822166453
TBX15	1	complement(118883046..11898955	13,22	16,65	-0,109612958
WARS2	1	complement(119031216..11914065	4,37	5,55	-0,123236217
HAO2	1	119368779..119394130	0,01	-	3,068695355
HSD3B2	1	119414931..119423035	-	-	#N/D
HSD3B1	1	119507198..119515054	-	-	0,188027316
ZNF697	1	complement(119619377..11964826	5,59	6,48	0,00985905
PHGDH	1	119648411..119744218	36,31	#####	-1,274412317
HMGCS2	1	complement(119748002..11976890	-	-	0,188027316
REG4	1	complement(119794017..11981158	-	0,01	-3,804623889
ADAM30	1	complement(119893533..11989651	-	-	#N/D
NOTCH2	1	complement(119911553..12010077	24,61	34,49	-0,263718741
SEC22B	1	complement(120150898..12017652	9,34	9,34	0,222371878
NBPF8	1	120415035..120469676	4,35	5,08	-0,001372453
NOTCH2NLR	1	120723912..120794851	0,60	0,41	0,773832143
NBPF26	1	120723945..120842229	1,72	2,48	-0,305325581
PPIAL4A	1	120889771..120890530	-	0,07	-4,758582517
gene:ENSG000	1	complement(120913151..12091423	1,67	2,52	-0,367495397
FAM72B	1	complement(121167646..12118553	12,07	16,56	-0,233693053
SRGAP2C	1	121184811..121392874	9,45	9,32	0,243110561

H3-7	1	complement(143894544..14390597	0,06	0,12	-0,682787006
FAM72C	1	complement(143955287..14397198	13,01	12,29	0,305082939
PPIAL4E	1	complement(144372875..14437363	-	-	#N/D
NBPF15	1	complement(144421390..14446167	10,64	9,91	0,325271259
PPIAL4F	1	144592893..144593652	-	-	0,188027316
SRGAP2B	1	complement(144887265..14509552	8,71	7,34	0,469318443
FAM72D	1	145095974..145112696	8,94	8,61	0,27763089
gene:ENSG000	1	145214851..145215938	4,62	3,53	0,608819338
PPIAL4D	1	complement(145241415..14524217	-	-	#N/D
NBPF20	1	complement(145289900..14542560	0,57	0,53	0,325657152
gene:ENSG000	1	145459798..145462007	0,01	0,15	-3,583354092
GPR89A	1	145607988..145670650	4,52	4,69	0,16902693
PDZK1	1	complement(145670852..14570814	0,31	0,28	0,387480186
CD160	1	145719471..145739288	0,26	0,18	0,757650522
RNF115	1	complement(145738868..14582409	4,70	3,07	0,837427433
POLR3C	1	145824053..145844402	2,87	3,62	-0,112356879
NUDT17	1	145845630..145848954	1,32	0,97	0,669111947
PIAS3	1	complement(145848522..14585983	23,15	13,06	1,048775964
ANKRD35	1	complement(145866560..14588586	0,01	-	4,913749004
ITGA10	1	complement(145891208..14591011	0,86	0,24	2,07077298
PEX11B	1	complement(145911350..14591871	12,14	8,58	0,722884498
gene:ENSG000	1	complement(145917714..14592674	0,33	0,02	3,878355004
RBM8A	1	complement(145921556..14592767	11,81	11,76	0,22930762
gene:ENSG000	1	145927258..145977811	-	-	#N/D
LIX1L	1	complement(145933423..14595801	25,55	20,49	0,541100867
ANKRD34A	1	complement(145959441..14596457	0,30	0,48	-0,441067735
POLR3GL	1	145964690..145978848	3,85	3,95	0,188953094
TXNIP	1	complement(145992435..14599657	123,99	2,15	6,072677392
HJV	1	complement(146017468..14603674	0,01	-	4,516772761
NBPF10	1	complement(146064711..14622900	1,65	1,90	0,016133085
NOTCH2NLA	1	complement(146146203..14622904	2,33	2,36	0,203493971
PPIAL4H	1	complement(146344006..14634476	-	-	0,188027316
NBPF12	1	146938324..146996202	1,91	2,03	0,135031879
PRKAB2	1	complement(147155106..14717255	4,80	3,82	0,552879685
FMO5	1	complement(147175351..14724305	0,10	0,04	1,503963791
CHD1L	1	147242654..147295765	11,13	9,96	0,383383172
BCL9	1	147541501..147626216	6,88	4,67	0,781872774
gene:ENSG000	1	147611590..147611796	-	-	#N/D
ACP6	1	complement(147629652..14767052	1,58	1,42	0,376971111
GJA5	1	complement(147756199..14777336	-	0,00	-2,89252005
GJA8	1	147902795..147909269	-	-	#N/D
GPR89B	1	147928393..147993592	2,97	3,14	0,143345671
NBPF11	1	complement(148102047..14815232	2,33	2,43	0,165138953
gene:ENSG000	1	148458814..148459871	4,31	4,25	0,244817537
PPIAL4G	1	complement(148482548..14848330	-	0,02	-2,89252005
NBPF14	1	complement(148531385..14867974	4,13	4,95	-0,038047289
NOTCH2NLB	1	complement(148600285..14867974	2,77	2,69	0,268563173
NUDT4B	1	148748774..148752415	0,67	1,78	-1,1951475

PDE4DIP	1	148808140..149048286	6,65	4,60	0,75447775
NBPF9	1	complement(149052186..14910356	6,54	8,04	-0,07424063
NOTCH2NLC	1	149390621..149471833	2,25	2,25	0,225935889
gene:ENSG000	1	149390623..149556361	0,10	0,07	0,814832154
NBPF19	1	149475045..149556361	2,16	2,25	0,164425194
PPIAL4C	1	149583848..149584607	0,04	-	4,516772761
FCGR1A	1	149782671..149791675	-	-	0,188027316
H2BC18	1	complement(149782689..14981237	23,98	95,63	-1,772478577
H3C13	1	complement(149813225..14981369	13,54	66,36	-2,06967598
H4C14	1	149832657..149839767	11,01	42,26	-1,717833004
H3C14	1	complement(149840687..14984120	0,04	3,47	-6,062446108
H2AC18	1	complement(149842218..14984275	105,62	#####	-1,291238372
H2AC19	1	149851061..149851594	30,33	44,78	-0,339135814
H3C15	1	149852608..149853125	119,88	#####	-0,909969311
H4C15	1	complement(149854045..14986115	8,76	39,46	-1,948036921
H2BC21	1	complement(149884459..14988668	14,43	29,46	-0,806408281
H2AC20	1	149886918..149887411	33,15	64,32	-0,732825929
H2AC21	1	complement(149887469..14988796	15,23	34,57	-0,959331279
BOLA1	1	149887890..149900798	2,54	3,68	-0,312111557
SV2A	1	complement(149903318..14991784	0,02	0,06	-0,96248933
SF3B4	1	complement(149923317..14992780	28,05	25,11	0,382742752
MTMR11	1	complement(149928651..14993687	1,52	2,23	-0,330539581
OTUD7B	1	complement(149937812..15001072	10,16	8,13	0,543986205
VPS45	1	150067279..150145329	3,80	2,88	0,62114849
PLEKHO1	1	150149183..150164720	1,30	3,77	-1,310418436
ANP32E	1	complement(150218417..15023615	8,35	12,27	-0,331867419
CA14	1	150257251..150265078	0,43	0,22	1,21552103
APH1A	1	complement(150265399..15026958	44,58	36,66	0,505053095
C1orf54	1	150268200..150280916	1,10	0,76	0,740705165
CIART	1	150282543..150287093	0,01	-	3,967268276
MRPS21	1	150293861..150308979	31,25	24,91	0,550050973
PRPF3	1	150321479..150353233	26,22	17,27	0,825395415
RPRD2	1	150363091..150476566	4,01	5,18	-0,144692229
TARS2	1	150487414..150507602	18,81	17,52	0,325623747
ECM1	1	150508062..150513789	15,90	11,91	0,64023759
ADAMTSL4	1	150549369..150560937	3,61	5,28	-0,324123161
MCL1	1	complement(150560895..15057973	30,82	27,01	0,413316484
ENSA	1	complement(150600851..15062961	19,35	14,40	0,648937189
GOLPH3L	1	complement(150646230..15069715	17,06	7,95	1,324822635
HORMAD1	1	complement(150698060..15072089	-	-	0,188027316
CTSS	1	complement(150730079..15076595	2,17	4,59	-0,856325918
CTSK	1	complement(150794880..15080957	0,79	1,00	-0,116686013
ARNT	1	complement(150809713..15087670	16,40	13,98	0,453254847
CTXND2	1	150887136..150913292	-	-	0,188027316
SETDB1	1	150926263..150964744	9,84	10,01	0,197960011
CERS2	1	complement(150960583..15097500	71,09	57,63	0,526061471
ANXA9	1	150982249..150995634	0,35	0,69	-0,769868354
MINDY1	1	complement(150996549..15100837	0,87	1,58	-0,633692773

PRUNE1	1	151008420..151035713	9,07	7,96	0,410919956
BNIPL	1	151036321..151047720	0,62	0,41	0,823572319
C1orf56	1	151047751..151051986	1,21	1,63	-0,203710916
CDC42SE1	1	complement(151050971..15107032	28,78	24,12	0,477727681
MLLT11	1	151060397..151069544	10,14	28,20	-1,253112193
GABPB2	1	151070578..151125542	0,47	0,96	-0,801954421
SEMA6C	1	complement(151131685..15114663	0,86	1,13	-0,175514678
TNFAIP8L2	1	151156649..151159749	0,74	0,87	-0,00400401
SCNM1	1	151156664..151170296	7,17	6,90	0,277917543
LYSMD1	1	complement(151159748..15116594	9,42	6,27	0,810223689
TMOD4	1	complement(151169986..15117628	0,03	0,03	0,366665713
VPS72	1	complement(151176304..15119532	25,55	19,59	0,606111021
PIP5K1A	1	151197949..151249536	46,92	38,96	0,491227032
PSMD4	1	151254709..151267479	45,42	47,90	0,146423635
ZNF687	1	151281618..151292176	12,93	8,78	0,781583761
PI4KB	1	complement(151291797..15132771	25,31	17,93	0,719966534
RFX5	1	complement(151340640..15134732	8,82	12,79	-0,312462687
SELENBP1	1	complement(151364304..15137270	0,03	-	5,480421266
PSMB4	1	151399560..151401937	177,70	#####	0,310449993
POGZ	1	complement(151402724..15145949	9,82	9,78	0,228702692
CGN	1	151510510..151538692	0,01	0,19	-4,688504655
TUFT1	1	151540305..151583583	3,95	5,96	-0,370823882
SNX27	1	151612006..151699091	13,38	14,39	0,118306587
CELF3	1	complement(151700058..15171680	0,10	0,12	0,041983265
RIIAD1	1	151710433..151729805	0,01	0,01	-0,01185201
MRPL9	1	complement(151759647..15176349	17,65	22,22	-0,108912539
OAZ3	1	151762899..151771334	0,19	0,17	0,367532518
TDRKH	1	complement(151770107..15179141	2,23	2,00	0,38125471
LINGO4	1	complement(151800264..15180541	0,01	0,01	-0,025382928
RORC	1	complement(151806071..15183184	0,24	0,31	-0,16577378
C2CD4D	1	complement(151837818..15184055	-	0,01	-2,89252005
THEM5	1	complement(151847101..15185371	-	0,02	-3,804623889
THEM4	1	complement(151870866..15190963	0,65	1,78	-1,235611073
S100A10	1	complement(151982915..15199385	130,98	62,39	1,293073332
S100A11	1	complement(152032506..15204790	153,88	#####	-0,039801119
TCHHL1	1	complement(152084141..15208906	-	0,00	-2,89252005
TCHH	1	complement(152106317..15211544	0,00	-	4,516772761
RPTN	1	complement(152153595..15215922	-	-	0,188027316
HRNR	1	complement(152212076..15222419	0,00	0,02	-3,545596224
FLG	1	complement(152302165..15232523	-	0,01	-5,327865481
FLG2	1	complement(152348735..15236000	-	-	0,188027316
CRNN	1	complement(152409243..15241426	-	-	0,188027316
LCE5A	1	152510803..152512177	-	-	0,188027316
CRCT1	1	152514482..152516008	-	-	0,188027316
LCE3E	1	complement(152565654..15256678	-	-	#N/D
LCE3D	1	complement(152579381..15258051	-	0,02	-2,89252005
LCE3C	1	152600234..152601086	-	-	#N/D
LCE3B	1	152613811..152614098	-	-	#N/D

LCE3A	1	complement(152622834..15262310	-	-	#N/D
LCE2D	1	152663380..152664659	-	-	#N/D
LCE2C	1	152675279..152676569	-	-	#N/D
LCE2B	1	152686123..152687397	-	-	#N/D
LCE2A	1	152698345..152699442	-	0,02	-2,89252005
LCE4A	1	152708160..152709434	-	-	#N/D
KPLCE	1	152719522..152720470	-	0,04	-4,359055975
KPRP	1	152758025..152762056	-	0,04	-5,734998842
LCE1F	1	152775140..152777024	-	-	0,188027316
LCE1E	1	152786214..152788426	-	-	#N/D
LCE1D	1	152796721..152798181	-	-	#N/D
LCE1C	1	complement(152804832..15280665	-	-	0,188027316
LCE1B	1	152811971..152813108	-	-	#N/D
LCE1A	1	152827473..152828097	-	-	#N/D
LCE6A	1	152842856..152843983	-	-	#N/D
LCE7A	1	152859996..152861252	-	-	#N/D
SMCP	1	152878322..152885047	-	-	#N/D
IVL	1	152908546..152911886	-	0,01	-3,804623889
SPRR5	1	152947206..152949255	-	-	#N/D
SPRR4	1	152970648..152972574	-	-	#N/D
SPRR1A	1	152984081..152985814	-	-	#N/D
SPRR3	1	153001747..153003856	-	-	#N/D
SPRR1B	1	153031203..153032900	-	-	#N/D
SPRR2D	1	complement(153039732..15304193	-	-	0,188027316
SPRR2A	1	complement(153056120..15305751	-	-	#N/D
SPRR2B	1	complement(153070226..15307161	-	-	#N/D
SPRR2E	1	complement(153093135..15310618	-	0,02	-2,89252005
SPRR2F	1	complement(153112121..15311351	-	-	0,188027316
SPRR2G	1	complement(153149582..15315089	-	-	#N/D
LELP1	1	153203430..153205120	-	-	#N/D
PRR9	1	153217584..153219310	-	-	#N/D
LORICRIN	1	153259687..153262124	-	-	#N/D
PGLYRP3	1	complement(153297116..15331295	-	-	0,188027316
PGLYRP4	1	complement(153330120..15334884	-	-	#N/D
S100A9	1	153357854..153361023	-	-	0,188027316
S100A12	1	complement(153373711..15337562	-	-	#N/D
S100A8	1	complement(153390032..15339107	-	-	0,188027316
S100A7A	1	153416520..153423222	-	-	0,188027316
S100A7	1	complement(153457744..15346065	-	0,03	-2,89252005
S100A6	1	complement(153534599..15353624	742,19	#####	1,659677652
S100A5	1	complement(153537147..15354176	0,09	0,10	0,130034011
S100A4	1	complement(153543613..15355013	45,18	55,27	-0,067778572
S100A3	1	complement(153547329..15354925	6,18	6,80	0,0841365
S100A2	1	complement(153561108..15356789	5,38	9,95	-0,664483856
S100A16	1	complement(153606886..15361314	81,78	89,48	0,093305184
S100A14	1	complement(153614255..15361698	0,15	0,09	0,950323601
S100A13	1	complement(153618787..15363136	23,20	20,39	0,409354775
S100A1	1	153627926..153632039	0,99	0,61	0,928418774

CHTOP	1	153633982..153646306	11,09	15,30	-0,240350781
SNAPIN	1	153658703..153661852	11,89	12,82	0,114612981
ILF2	1	complement(153661788..15367102	117,68	#####	0,450581747
NPR1	1	153678688..153693992	0,04	0,12	-1,193156405
INTS3	1	153728050..153774808	21,65	16,02	0,65785716
SLC27A3	1	153774354..153780157	1,60	1,75	0,089700651
GATAD2B	1	complement(153789030..15392336	17,55	14,61	0,487678655
DENND4B	1	complement(153929501..15394671	18,25	17,32	0,298408889
CRTC2	1	complement(153947669..15395861	17,05	14,17	0,489954745
SLC39A1	1	complement(153959099..15396818	53,36	37,87	0,71783821
gene:ENSG000	1	complement(153959151..15397588	0,09	-	7,117975732
CREB3L4	1	153967534..153974361	2,73	2,82	0,178266762
JTB	1	complement(153974269..15397767	43,66	34,17	0,576599224
gene:ENSG000	1	complement(153975850..15398624	3,29	1,89	1,023913142
RAB13	1	complement(153981617..15398635	40,06	29,70	0,654879844
RPS27	1	153990762..153992155	148,29	#####	0,112724568
NUP210L	1	complement(153992690..15415511	0,05	0,02	1,476992126
TPM3	1	complement(154155308..15419464	27,22	21,22	0,582813015
CFAP141	1	complement(154199085..15420633	-	-	0,188027316
C1orf43	1	complement(154206696..15422063	77,71	68,07	0,414285288
UBAP2L	1	154220179..154271510	47,57	43,41	0,355199369
HAX1	1	154272355..154275875	41,02	38,99	0,296349483
AQP10	1	154321090..154325325	0,06	0,06	0,335747576
ATP8B2	1	154325525..154351304	36,20	23,98	0,817579787
IL6R	1	154405193..154469450	9,14	4,16	1,35789666
SHE	1	complement(154469772..15450241	0,02	0,00	2,493547104
TDRD10	1	154502219..154548147	-	0,02	-4,758582517
UBE2Q1	1	complement(154548577..15455902	13,09	11,62	0,395207265
CHRNB2	1	154567778..154580013	0,37	0,33	0,381188483
ADAR	1	complement(154581695..15462801	47,09	36,55	0,588466584
KCNN3	1	complement(154697455..15487028	0,04	0,01	3,019137699
PMVK	1	complement(154924740..15493671	11,02	7,27	0,822003783
PBXIP1	1	complement(154944076..15495612	7,89	3,63	1,341814545
PYGO2	1	complement(154957026..15496385	15,75	11,83	0,636223148
SHC1	1	complement(154962298..15497439	95,60	42,00	1,409798097
CKS1B	1	154974653..154979251	87,86	78,97	0,377135537
FLAD1	1	154983338..154993111	8,54	7,10	0,489869638
LENEP	1	154993586..154994315	0,53	0,32	0,954694521
ZBTB7B	1	155002630..155018523	22,09	17,04	0,597650189
DCST2	1	complement(155018520..15503378	0,45	0,29	0,890647722
DCST1	1	155033824..155050930	0,19	0,25	-0,216556223
ADAM15	1	155050566..155062775	49,31	40,87	0,493922393
EFNA4	1	155063737..155069553	7,21	4,43	0,924989816
EFNA4-EFNA3	1	155063748..155086807	1,70	0,37	2,404727338
EFNA3	1	155078837..155087538	3,48	2,44	0,738301908
EFNA1	1	155127876..155134899	11,15	8,33	0,644526061
SLC50A1	1	155135344..155138857	28,06	20,58	0,670676549
DPM3	1	complement(155139891..15514059	15,29	6,66	1,421476494

KRTCAP2	1	complement(155169408..15517347	52,36	46,23	0,402873152
gene:ENSG000	1	complement(155169409..15518727	2,21	1,82	0,497630782
TRIM46	1	155173787..155184971	0,23	0,29	-0,071178992
MUC1	1	complement(155185824..15519291	1,81	1,62	0,383041446
THBS3	1	complement(155195588..15520905	6,20	4,92	0,5550009
MTX1	1	155208695..155213824	25,41	20,86	0,507585464
GBA1	1	complement(155234452..15524469	20,60	18,29	0,39423622
ENTREP3	1	complement(155247205..15525548	32,16	33,86	0,14890911
SCAMP3	1	complement(155255979..15526243	45,55	28,51	0,898970469
CLK2	1	complement(155262868..15527849	13,61	11,80	0,428587201
HCN3	1	155277463..155289848	1,74	1,42	0,51979571
PKLR	1	complement(155289293..15530143	0,05	0,04	0,78195819
FDPS	1	155308748..155320666	50,12	29,19	1,002833783
RUSC1	1	155320894..155331114	9,10	6,92	0,618533378
ASH1L	1	complement(155335268..15556316	7,19	6,58	0,351136425
MSTO1	1	155563235..155614951	22,63	11,15	1,243961357
gene:ENSG000	1	complement(155626755..15564864	1,13	1,37	-0,055294757
YY1AP1	1	complement(155659446..15568933	8,56	7,03	0,508271246
DAP3	1	155687960..155739010	24,38	23,26	0,290886256
GON4L	1	complement(155749659..15585940	11,81	7,56	0,867815332
SYT11	1	155859567..155885199	11,51	11,44	0,232405849
RIT1	1	complement(155897808..15591140	4,74	3,07	0,850148695
KHDC4	1	complement(155913045..15593441	27,13	17,72	0,837795297
RXFP4	1	155941638..155943087	0,39	0,45	0,04358869
ARHGEF2	1	complement(155946851..15600707	25,74	27,41	0,132266554
SSR2	1	complement(156009048..15602095	134,90	70,34	1,162567766
UBQLN4	1	complement(156035299..15605379	27,28	31,68	0,007036188
LAMTOR2	1	156054782..156058506	19,58	15,27	0,58193956
RAB25	1	156061160..156070504	0,02	0,06	-1,291882393
MEX3A	1	complement(156072013..15608246	7,59	5,46	0,699796182
LMNA	1	156082572..156140081	157,83	65,67	1,488078606
SEMA4A	1	156147366..156177752	0,32	0,14	1,422135691
SLC25A44	1	156193932..156212796	17,58	7,40	1,471946004
PMF1-BGLAP	1	156212982..156243332	3,02	2,98	0,240657544
PMF1	1	156212993..156240042	24,34	19,96	0,509579471
BGLAP	1	156242184..156243317	0,38	-	7,040630395
PAQR6	1	complement(156243320..15624811	3,33	2,80	0,472046696
SMG5	1	complement(156249224..15628282	44,36	35,29	0,553180711
TMEM79	1	156282935..156293185	4,84	3,58	0,657882527
GLMP	1	complement(156290089..15629568	1,73	0,15	3,703800249
VHLL	1	complement(156298624..15629930	0,06	0,21	-1,462209385
CCT3	1	complement(156308968..15636787	120,94	#####	-0,028772123
TSACC	1	156337314..156346995	0,18	0,35	-0,694397966
RHBG	1	156369211..156385219	-	0,01	-2,89252005
MEF2D	1	complement(156463727..15650077	28,73	12,74	1,396184334
IQGAP3	1	complement(156525405..15657260	26,94	36,41	-0,211196464
TTC24	1	156579723..156587719	0,01	0,01	0,921097799
NAXE	1	156591756..156609507	6,95	7,40	0,133439539

GPATCH4	1	complement(156594301..15660149	3,14	4,46	-0,281009531
HAPLN2	1	156619331..156625725	0,10	0,02	2,233007235
BCAN	1	156641390..156659532	0,42	1,03	-1,063118818
NES	1	complement(156668763..15667740	0,02	0,03	-0,728065629
CRABP2	1	complement(156699606..15670581	94,87	54,01	1,035653169
ISG20L2	1	complement(156721891..15672876	16,34	12,73	0,582831069
METTL25B	1	156728442..156736960	10,04	11,70	0,001729924
MRPL24	1	complement(156737303..15674159	18,54	18,56	0,221416586
HDGF	1	complement(156742109..15676692	263,40	#####	0,286933892
PRCC	1	156750610..156800815	32,70	29,97	0,348945796
SH2D2A	1	complement(156806243..15681684	0,17	2,63	-3,741711296
NTRK1	1	156815636..156881850	0,08	0,22	-1,286431802
INSRR	1	complement(156840063..15685911	0,01	0,01	-0,291161992
PEAR1	1	156893698..156916429	1,11	2,68	-1,046160424
LRRC71	1	156920632..156933094	0,03	0,02	0,677647514
ARHGEF11	1	complement(156934840..15704690	6,87	8,73	-0,121952094
ETV3L	1	complement(157092043..15711241	-	-	0,188027316
ETV3	1	complement(157121191..15713847	11,08	5,92	1,127457212
FCRL5	1	complement(157513377..15755251	-	-	0,188027316
FCRL4	1	complement(157573747..15759808	-	-	0,188027316
FCRL3	1	complement(157674321..15770076	-	-	0,188027316
FCRL2	1	complement(157745733..15777713	-	-	#N/D
FCRL1	1	complement(157794403..15782012	-	-	#N/D
CD5L	1	complement(157830911..15789825	-	-	#N/D
KIRREL1	1	157993273..158100262	27,67	23,11	0,482868408
SMIM42	1	complement(158127287..15812830	-	-	0,188027316
CD1D	1	158178030..158186427	0,51	0,04	3,867166128
CD1A	1	158254424..158258269	-	-	#N/D
CD1C	1	158289923..158294774	-	-	#N/D
CD1B	1	complement(158327951..15833153	-	-	#N/D
CD1E	1	158353696..158357553	-	-	#N/D
OR10T2	1	complement(158398522..15839946	-	-	#N/D
OR10K2	1	complement(158418210..15842623	-	-	#N/D
OR10K1	1	158461574..158470857	-	-	0,188027316
OR10R2	1	158472220..158480936	-	-	#N/D
OR6Y1	1	complement(158544550..15855440	-	-	#N/D
OR6P1	1	complement(158560606..15857058	-	-	#N/D
OR10X1	1	complement(158578919..15857989	-	-	#N/D
OR10Z1	1	158605268..158612514	-	-	#N/D
SPTA1	1	complement(158610704..15868671	0,00	-	3,068695355
OR6K2	1	complement(158699678..15870065	-	-	#N/D
OR6K3	1	complement(158716327..15872072	-	-	0,188027316
OR6N1	1	complement(158747814..15877219	-	-	0,188027316
OR6K6	1	158754720..158755891	-	-	#N/D
OR6N2	1	complement(158774222..15878120	-	-	#N/D
MNDA	1	158831351..158849506	-	-	0,188027316
PYHIN1	1	158930796..158977059	0,00	-	3,068695355
IFI16	1	158999968..159055155	5,10	9,85	-0,726522507

PYDC5	1	complement(158999971..15900275	-	0,02	-4,758582517
AIM2	1	complement(159061599..15918784	-	-	0,188027316
CADM3	1	159171609..159203313	-	-	0,188027316
ACKR1	1	159204875..159206500	-	-	0,188027316
FCER1A	1	159289714..159308224	-	-	#N/D
OR10J3	1	complement(159313720..15931465	-	-	0,188027316
OR10J4	1	159432204..159433138	-	-	#N/D
OR10J1	1	159437845..159443078	-	-	#N/D
OR10J5	1	complement(159535078..15953600	-	-	#N/D
APCS	1	159587826..159588865	-	-	0,188027316
CRP	1	complement(159712289..15971458	-	-	#N/D
DUSP23	1	159780932..159782543	5,03	3,01	0,9661917
FCRL6	1	159800511..159816257	-	-	#N/D
SLAMF8	1	159826811..159837492	0,00	0,07	-3,842392802
gene:ENSG000	1	complement(159834474..15987305	4,65	2,48	1,132962668
VSIG8	1	complement(159854316..15986265	0,09	0,04	1,356372188
CFAP45	1	complement(159872364..15990016	0,91	0,53	0,991324028
gene:ENSG000	1	complement(159910094..15991055	0,35	0,37	0,165798947
TAGLN2	1	complement(159918107..15992550	372,96	#####	0,654340416
IGSF9	1	complement(159927039..15994561	0,02	0,13	-2,780890846
SLAMF9	1	complement(159951492..15995423	0,08	0,03	1,339142659
KCNJ10	1	complement(159998651..16007048	0,00	0,01	-0,999120035
PIGM	1	complement(160024953..16003199	0,86	0,80	0,317599095
KCNJ9	1	160081538..160090563	0,17	0,05	1,994903778
IGSF8	1	complement(160091340..16009894	17,18	9,40	1,093395021
ATP1A2	1	160115759..160143591	-	0,05	-7,042589436
ATP1A4	1	160151586..160186980	-	0,00	-2,89252005
CASQ1	1	160190575..160201886	-	-	0,188027316
PEA15	1	160205380..160215376	65,12	57,86	0,393760915
DCAF8	1	complement(160215715..16026254	15,96	18,57	0,004292994
gene:ENSG000	1	complement(160216800..16028513	0,21	0,19	0,327978397
PEX19	1	complement(160276807..16028634	14,33	14,70	0,186016951
COPA	1	complement(160288594..16034356	95,01	61,28	0,855724159
NCSTN	1	160343294..160358952	26,14	27,40	0,154910247
NHLH1	1	160367071..160372846	0,43	0,23	1,151671868
VANGL2	1	160400564..160428670	0,07	0,00	4,913364866
SLAMF6	1	complement(160485030..16052326	-	-	#N/D
CD84	1	complement(160541095..16057951	0,00	-	3,967268276
SLAMF1	1	complement(160608106..16064704	-	-	#N/D
CD48	1	complement(160678746..16071183	-	-	#N/D
SLAMF7	1	160739057..160754821	-	0,00	-2,89252005
LY9	1	160796074..160828261	-	-	0,188027316
CD244	1	complement(160830160..16086288	-	-	#N/D
ITLN1	1	complement(160876540..16088518	-	-	#N/D
ITLN2	1	complement(160945025..16095480	-	0,04	-4,758582517
F11R	1	complement(160995211..16102117	1,04	1,60	-0,398973764
gene:ENSG000	1	complement(160997957..16103896	0,73	0,75	0,18652918
TSTD1	1	complement(161037631..16103897	0,06	0,07	-0,032860829

USF1	1	complement(161039251..16104597	20,12	14,46	0,699538657
ARHGAP30	1	complement(161046946..16106997	0,51	0,85	-0,52975795
NECTIN4	1	complement(161070998..16108955	0,02	0,11	-1,926147748
KLHDC9	1	161098361..161100346	0,15	0,14	0,299198032
PFDN2	1	complement(161100556..16111805	47,13	52,09	0,078745874
NIT1	1	161118086..161125445	8,11	6,40	0,565126081
DEDD	1	complement(161120974..16113268	10,28	13,33	-0,152470371
UFC1	1	161152776..161158856	20,52	19,83	0,272525555
USP21	1	161159450..161165723	15,23	15,16	0,229781514
PPOX	1	161166056..161178013	5,44	3,87	0,716822397
B4GALT3	1	complement(161171310..16117796	22,12	16,74	0,625053353
ADAMTS4	1	complement(161184302..16119905	6,80	3,88	1,032501177
NDUFS2	1	161197104..161214723	35,90	28,84	0,538886548
FCER1G	1	161215234..161220699	0,11	0,13	-0,037246625
APOA2	1	complement(161222292..16122363	-	-	0,188027316
TOMM40L	1	161225939..161230746	5,68	4,78	0,470093845
NR1I3	1	complement(161229666..16123824	0,44	0,22	1,222723871
PCP4L1	1	161258745..161285450	-	-	0,188027316
MPZ	1	complement(161304735..16130996	0,17	0,29	-0,497637598
SDHC	1	161314381..161363206	36,15	31,94	0,401708596
CFAP126	1	complement(161364733..16136787	2,55	0,85	1,799842286
FCGR2A	1	161505430..161524013	0,60	0,11	2,726379252
HSPA6	1	161524540..161526894	0,22	0,20	0,363774137
FCGR3A	1	complement(161541759..16155096	-	0,01	-4,758582517
gene:ENSG000	1	complement(161544807..16163145	-	0,02	-2,89252005
FCGR2C	1	161581339..161605662	-	-	0,188027316
FCGR3B	1	complement(161623196..16163196	0,00	-	3,068695355
FCGR2B	1	161663143..161678654	-	0,01	-3,804623889
FCRLA	1	161706972..161714352	0,02	0,19	-3,120099933
FCRLB	1	161721544..161728143	0,17	0,66	-1,708137286
DUSP12	1	161749758..161757238	4,66	7,99	-0,55449986
ATF6	1	161766298..161977574	5,74	7,32	-0,127339113
OLFML2B	1	complement(161983192..16202386	0,22	0,18	0,512224708
NOS1AP	1	162069691..162370475	0,23	0,26	0,066806316
gene:ENSG000	1	162365407..162383531	0,03	0,28	-3,033849607
SPATA46	1	complement(162373203..16237685	0,06	0,07	0,097868349
C1orf226	1	162378841..162386812	0,87	1,03	-0,019916795
SH2D1B	1	complement(162395268..16241213	-	0,04	-5,734998842
UHMK1	1	162497251..162529631	10,66	8,32	0,581525751
UAP1	1	162561722..162601240	42,42	26,92	0,879440836
DDR2	1	162631373..162787405	16,65	20,24	-0,058220812
HSD17B7	1	162790702..162812823	4,62	1,61	1,740831591
CCDC190	1	complement(162824458..16286876	0,01	0,03	-1,015460095
RGS4	1	163068775..163076802	0,15	0,34	-0,933737555
RGS5	1	complement(163111121..16332179	0,25	0,24	0,242847311
NUF2	1	163266576..163355764	3,77	6,03	-0,456248278
PBX1	1	164555584..164899296	0,51	1,07	-0,863097885
LMX1A	1	complement(165201867..16535671	-	-	#N/D

RXRG	1	complement(165400922..16544535	-	-	0,188027316
LRRC52	1	165544000..165563957	-	-	0,188027316
MGST3	1	165631213..165661796	18,73	15,42	0,50420291
ALDH9A1	1	complement(165662216..16569856	10,33	9,16	0,396533203
TMCO1	1	complement(165724293..16582775	8,78	6,51	0,65505094
UCK2	1	165827614..165911618	10,70	18,57	-0,572783953
FAM78B	1	complement(166057426..16616700	0,02	0,06	-1,348036508
POGK	1	166839447..166856359	9,07	9,16	0,208070708
TADA1	1	complement(166856510..16687626	6,20	5,60	0,368950608
ILDR2	1	complement(166895711..16697554	0,00	0,00	-0,923955849
MAEL	1	166975582..167022214	-	-	0,188027316
GPA33	1	complement(167052836..16716647	0,01	0,01	-0,025382928
STYXL2	1	167094075..167129165	-	-	0,188027316
POU2F1	1	167220876..167427345	3,01	3,04	0,21151389
CD247	1	complement(167425027..16751864	0,02	0,03	-0,161981728
CREG1	1	complement(167529117..16755380	1,90	3,25	-0,549067837
RCSD1	1	167630093..167708696	0,01	0,02	-0,442604891
MPZL1	1	167721192..167791919	26,06	22,57	0,430283434
ADCY10	1	complement(167809386..16791421	0,02	0,03	0,181581643
MPC2	1	complement(167916675..16793707	5,64	5,44	0,273470113
DCAF6	1	167935783..168075843	7,96	8,63	0,10655097
GPR161	1	complement(168079542..16813766	5,53	6,18	0,062708195
TIPRL	1	168178962..168202109	7,48	8,18	0,093758876
SFT2D2	1	168225938..168253021	9,06	7,84	0,431530678
TBX19	1	168280877..168314426	0,91	0,64	0,718148832
XCL2	1	complement(168540768..16854399	-	-	0,188027316
XCL1	1	168576605..168582069	0,01	-	3,068695355
DPT	1	complement(168695468..16872920	0,31	0,17	1,139303216
ATP1B1	1	169105697..169310992	2,26	1,93	0,448471175
gene:ENSG000	1	complement(169112164..16911277	0,16	0,15	0,319286298
NME7	1	complement(169132531..16936794	4,62	4,36	0,307535661
BLZF1	1	169367970..169396540	4,31	2,09	1,269593731
CCDC181	1	complement(169394870..16946066	0,06	0,03	1,422471951
SLC19A2	1	complement(169463909..16948594	1,66	2,43	-0,324831945
F5	1	complement(169511951..16958658	-	0,00	-4,359055975
SELP	1	complement(169588849..16963019	0,01	-	4,516772761
FIRRM	1	169662007..169854080	3,04	3,77	-0,087547376
SELL	1	complement(169690665..16971170	0,02	0,29	-3,432784426
SELE	1	complement(169722640..16976470	-	0,00	-2,89252005
METTL18	1	complement(169792529..16979496	0,95	1,92	-0,791503134
SCYL3	1	complement(169849631..16989426	0,94	1,13	-0,045707711
KIFAP3	1	complement(169921326..17008520	2,75	2,44	0,395327405
NTMT2	1	170145959..170168866	-	0,01	-2,89252005
GORAB	1	170531819..170553834	1,75	1,64	0,323306399
PRRX1	1	170662728..170739421	0,03	0,01	1,781479726
MROH9	1	170935526..171064765	-	-	#N/D
FMO3	1	171090901..171117819	0,00	-	3,068695355
FMO2	1	171185249..171212686	-	-	#N/D

FMO1	1	171248471..171285978	-	-	0,188027316
FMO4	1	171314183..171342084	0,20	0,19	0,306578081
PRRC2C	1	171485530..171593511	26,34	26,33	0,223444527
MYOCOS	1	171600621..171638799	-	0,01	-4,359055975
MYOC	1	complement(171635417..17165268	-	-	0,188027316
VAMP4	1	complement(171700160..17174207	1,20	1,00	0,48780222
METTL13	1	171781660..171814023	10,64	11,38	0,126389751
DNM3	1	171817887..172418466	0,11	0,09	0,531171047
PIGC	1	complement(172370189..17244408	6,08	5,67	0,324452277
C1orf105	1	172420685..172468831	0,01	0,03	-1,877914477
SUCO	1	172532349..172611833	7,27	3,41	1,315574285
FASLG	1	172659103..172666876	-	-	0,188027316
gene:ENSG000	1	172752586..172752924	-	-	#N/D
TNFSF18	1	complement(173039202..17305094	0,02	0,04	-0,865575989
TNFSF4	1	complement(173162645..17347758	0,16	0,17	0,154704305
PRDX6	1	173477330..173488815	91,50	94,12	0,182348046
SLC9C2	1	complement(173500460..17360307	-	-	0,188027316
gene:ENSG000	1	complement(173596060..17366983	-	-	0,188027316
ANKRD45	1	complement(173608336..17366985	0,01	0,03	-0,999120035
TEX50	1	173635338..173637130	-	-	#N/D
KLHL20	1	173714941..173786692	2,60	2,32	0,387042276
CENPL	1	complement(173799550..17382488	5,25	4,70	0,384260992
DARS2	1	173824653..173858808	10,11	11,49	0,037947451
ZBTB37	1	173868082..173903547	2,14	1,88	0,407895864
SERPINC1	1	complement(173903800..17391732	0,01	0,01	-0,01185201
RC3H1	1	complement(173931084..17402235	3,37	3,54	0,153031443
gene:ENSG000	1	complement(174022122..17402229	-	-	#N/D
RABGAP1L	1	174159410..174995308	0,71	0,73	0,178220904
GPR52	1	174447964..174449545	0,12	0,26	-0,946179537
CACYBP	1	174999163..175012027	6,43	9,94	-0,405763056
MRPS14	1	complement(175010789..17502342	6,92	5,57	0,53743298
TNN	1	175067833..175148075	0,02	0,01	1,505976878
KIAA0040	1	complement(175156986..17519299	1,00	0,97	0,253476232
TNR	1	complement(175315194..17574359	0,00	-	3,068695355
COP1	1	complement(175944831..17620728	6,69	6,03	0,371179423
PAPPA2	1	176463171..176845601	0,19	8,95	-5,3354972
ASTN1	1	complement(176857302..17716497	-	0,00	-3,804623889
BRINP2	1	177170958..177282422	0,03	0,02	0,946003371
SEC16B	1	complement(177923956..17798430	0,68	0,18	2,133305217
RASAL2	1	178094104..178484147	3,54	2,12	0,962712128
CLEC20A	1	complement(178479240..17849963	-	0,02	-4,359055975
TEX35	1	178513109..178548602	0,10	0,05	1,391893083
RALGPS2	1	178725165..178921842	7,57	6,47	0,449416078
ANGPTL1	1	complement(178849535..17887107	0,30	0,19	0,873673054
FAM20B	1	179025804..179076567	20,72	15,11	0,678833215
TOR3A	1	179082070..179098023	12,49	11,00	0,405977896
ABL2	1	complement(179099330..17922968	16,66	13,62	0,513589626
SOAT1	1	179293714..179358680	6,98	7,56	0,107485083

AXDND1	1	179365720..179554735	0,03	0,04	-0,322898219
NPHS2	1	complement(179550539..17957595	-	-	0,188027316
TDRD5	1	179591613..179691272	-	-	0,188027316
FAM163A	1	179743291..179816198	-	-	0,188027316
TOR1AIP2	1	complement(179839967..17987780	10,89	8,59	0,566638154
TOR1AIP1	1	179882042..179920077	8,99	9,83	0,09527418
gene:ENSG000	1	179882316..179882393	-	-	#N/D
CEP350	1	179954674..180114880	3,50	3,69	0,149360541
QSOX1	1	180154869..180204030	29,05	31,74	0,094988215
LHX4	1	180230264..180278984	0,30	0,69	-0,977186075
ACBD6	1	complement(180269653..18050295	5,00	5,52	0,082097618
XPR1	1	180632022..180890279	4,60	3,48	0,625816588
KIAA1614	1	180912897..180951614	1,24	1,31	0,136749756
STX6	1	complement(180972712..18102312	6,28	6,52	0,169928958
MR1	1	181033374..181061938	2,07	2,59	-0,096673477
IER5	1	181088700..181092900	5,02	6,66	-0,183244227
CACNA1E	1	181317690..181813262	0,02	0,02	0,648961805
ZNF648	1	complement(182054570..18206171	-	-	#N/D
GLUL	1	complement(182378098..18239220	11,83	7,40	0,900697347
TEDDM1	1	complement(182398117..18240066	0,01	0,03	-0,999120035
RGSL1	1	182409192..182560599	-	-	0,188027316
RNASEL	1	complement(182573634..18258925	0,86	0,86	0,233345616
RGS16	1	complement(182598623..18260438	0,04	0,15	-1,660236228
RGS8	1	complement(182641816..18268458	-	0,00	-3,804623889
NPL	1	182789293..182830384	0,24	0,99	-1,793421469
DHX9	1	182839347..182887982	21,80	35,22	-0,468995376
SHCBP1L	1	complement(182899865..18295352	-	-	#N/D
LAMC1	1	183023420..183145592	75,38	74,86	0,232992776
LAMC2	1	183186238..183245127	0,28	0,83	-1,323168854
NMNAT2	1	complement(183248237..18341838	2,18	5,38	-1,083201667
SMG7	1	183472216..183598246	20,63	21,03	0,195084913
NCF2	1	complement(183554461..18359090	0,14	3,05	-4,223972735
ARPC5	1	complement(183620846..18363578	8,79	9,59	0,097133902
RGL1	1	183636085..183928532	3,14	3,65	0,006533345
APOBEC4	1	complement(183646275..18365331	0,05	0,03	1,165729824
COLGALT2	1	complement(183929854..18403772	0,07	0,16	-1,05421397
TSEN15	1	184051651..184123978	2,47	3,73	-0,373819367
C1orf21	1	184387029..184629019	1,48	1,66	0,052307436
EDEM3	1	complement(184690237..18475490	6,81	6,00	0,405797088
NIBAN1	1	complement(184790724..18497450	4,85	18,88	-1,737595738
RNF2	1	185045497..185102603	2,26	2,82	-0,09416771
TRMT1L	1	complement(185118101..18515707	2,34	3,62	-0,406186415
SWT1	1	185157080..185291781	0,36	0,51	-0,285525724
gene:ENSG000	1	complement(185292384..18529437	0,30	0,29	0,248447397
IVNS1ABP	1	complement(185296388..18531727	17,01	9,07	1,130420693
gene:ENSG000	1	185518651..185520986	-	-	0,188027316
HMCN1	1	185734391..186190949	0,53	0,38	0,690098833
PRG4	1	186296279..186314567	0,01	0,01	0,921097799

TPR	1	complement(186311652..18637569	8,88	10,64	-0,038031942
ODR4	1	186375838..186421378	3,71	3,20	0,434235685
PDC	1	complement(186443566..18646111	0,02	0,02	0,524121557
PTGS2	1	complement(186671791..18668092	51,45	54,98	0,127317097
PLA2G4A	1	186828949..186988981	6,18	9,45	-0,388573762
BRINP3	1	complement(190097658..19047840	-	0,01	-4,359055975
RGS18	1	192158462..192185815	0,01	0,01	-0,025382928
RGS21	1	192316992..192367285	-	-	0,188027316
RGS1	1	192575763..192580024	0,01	0,01	-0,01185201
RGS13	1	192636138..192660311	-	0,01	-2,89252005
RGS2	1	192809039..192812275	0,77	1,78	-0,985987826
UCHL5	1	complement(193012250..19306008	2,68	2,71	0,208168712
RO60	1	193059454..193091777	5,70	4,57	0,542553248
GLRX2	1	complement(193090866..19310611	0,53	0,84	-0,425990634
CDC73	1	193121983..193254815	4,43	4,42	0,225792668
B3GALT2	1	complement(193178730..19318661	0,15	0,09	0,926873464
KCNT2	1	complement(196225779..19660922	0,05	0,29	-2,151316909
CFH	1	196651754..196752476	1,14	0,51	1,387325809
gene:ENSG000	1	196651852..196795393	0,09	0,03	1,563962
CFHR3	1	196774813..196795407	-	-	0,188027316
CFHR1	1	196819731..196837159	-	-	0,188027316
CFHR4	1	196888014..196918795	-	-	#N/D
CFHR2	1	196943738..196959622	-	-	0,188027316
CFHR5	1	196975010..197009678	-	-	#N/D
F13B	1	complement(197038741..19706726	-	-	0,188027316
ASPM	1	complement(197084121..19714669	5,43	8,68	-0,453657224
ZBTB41	1	complement(197153682..19720129	2,51	1,39	1,073546177
CRB1	1	197268204..197478455	0,00	0,02	-2,075817774
DENND1B	1	complement(197504748..19777569	0,76	0,98	-0,147263316
C1orf53	1	197902630..197907367	0,23	0,56	-1,030971428
LHX9	1	197911902..197935478	0,01	0,02	-1,024862207
NEK7	1	198156994..198322420	3,65	4,83	-0,180143216
ATP6V1G3	1	complement(198523222..19854094	-	-	0,188027316
PTPRC	1	198638457..198757476	0,00	-	4,516772761
NR5A2	1	200027614..200177420	0,01	0,03	-0,877094735
ZNF281	1	complement(200404940..20041005	5,38	6,55	-0,05963535
KIF14	1	complement(200551497..20062075	4,77	6,95	-0,318340321
DDX59	1	complement(200623896..20066990	3,24	3,78	0,001150329
CAMSAP2	1	200738893..200860704	6,69	7,04	0,147589273
GPR25	1	200872981..200874178	-	0,02	-3,804623889
INAVA	1	200891048..200915742	0,22	0,62	-1,299892006
KIF21B	1	complement(200969390..20102371	2,54	4,30	-0,536343598
CACNA1S	1	complement(201039512..20111245	0,00	0,00	-0,01185201
ASCL5	1	complement(201113943..20112718	0,11	0,27	-1,138421553
TMEM9	1	complement(201134772..20117157	19,83	12,12	0,932956798
IGFN1	1	201190824..201228952	0,14	5,33	-4,978601321
PKP1	1	201283452..201332993	0,01	0,02	-1,406253396
TNNT2	1	complement(201359008..20137776	-	-	#N/D

LAD1	1	complement(201380833..20139991	-	0,04	-6,052163584
TNNI1	1	complement(201403784..20142986	0,00	-	3,967268276
PHLDA3	1	complement(201464278..20146923	4,17	6,90	-0,503017589
CSRP1	1	complement(201483530..20150945	35,04	44,16	-0,110809771
NAV1	1	201539127..201826969	16,18	8,82	1,098588539
IPO9	1	201829149..201884291	14,37	14,67	0,193504177
SHISA4	1	201888680..201892587	10,54	5,84	1,074800339
LMOD1	1	complement(201896456..20194654	0,12	0,16	-0,162653199
TIMM17A	1	201955503..201970664	28,01	15,84	1,045401637
RNPEP	1	201982372..202006147	54,03	47,36	0,413136595
ELF3	1	202007945..202017183	0,18	4,59	-4,475254952
GPR37L1	1	202122886..202133592	0,14	0,20	-0,276257463
ARL8A	1	complement(202133404..20214474	20,86	21,53	0,177569001
PTPN7	1	complement(202147013..20216158	0,00	0,03	-2,272922624
LGR6	1	202193799..202319781	0,01	0,01	-0,03031053
UBE2T	1	complement(202331544..20234198	20,84	18,63	0,385366036
PPP1R12B	1	202348699..202592706	1,77	1,33	0,630278066
SYT2	1	complement(202590596..20271045	0,13	0,10	0,608088594
KDM5B	1	complement(202724495..20280848	9,49	6,97	0,667079364
RABIF	1	complement(202878282..20288914	1,21	1,48	-0,069427012
KLHL12	1	complement(202891116..20292863	8,98	10,29	0,025345762
ADIPOR1	1	complement(202940826..20295857	88,61	58,38	0,824928501
CYB5R1	1	complement(202961873..20296727	6,99	6,50	0,326543866
TMEM183A	1	203007374..203024848	20,51	15,89	0,591122363
PPFIA4	1	203026491..203078740	2,97	0,59	2,545685196
MYOG	1	complement(203083129..20308601	0,02	-	4,516772761
ADORA1	1	203090654..203167405	1,20	0,24	2,518715368
MYBPH	1	complement(203167811..20317582	0,59	0,26	1,37609024
CHI3L1	1	complement(203178931..20318670	20,82	19,50	0,317531809
CHIT1	1	complement(203212827..20327364	0,00	0,01	-0,01185201
BTG2	1	203305491..203309602	1,10	1,22	0,076360455
FMOD	1	complement(203340628..20335175	-	0,03	-5,327865481
PRELP	1	203475806..203491352	0,01	0,03	-1,239733301
OPTC	1	203494121..203508949	-	0,03	-4,758582517
ATP2B4	1	203626832..203744081	20,60	13,68	0,81279176
LAX1	1	203765177..203776372	0,01	0,03	-1,21700111
ZBED6	1	203795623..203854124	16,16	9,00	1,067934084
ZC3H11A	1	203795623..203854999	8,26	9,51	0,020133018
gene:ENSG000	1	203802094..203802192	-	-	#N/D
SNRPE	1	203861599..203871152	5,86	5,93	0,206626194
SOX13	1	204073115..204127743	8,71	7,09	0,518819427
ETNK2	1	complement(204131062..20415204	6,27	2,83	1,368831694
REN	1	complement(204154819..20419032	0,01	0,01	-0,01185201
KISS1	1	complement(204190341..20419649	0,02	-	3,068695355
GOLT1A	1	complement(204198163..20421398	-	-	0,188027316
PLEKHA6	1	complement(204218853..20437821	0,03	0,05	-0,231893514
PPP1R15B	1	complement(204396492..20441188	28,84	17,45	0,947357501
PIK3C2B	1	complement(204422628..20449480	2,17	2,32	0,121697613

MDM4	1	204516379..204558120	7,15	7,74	0,109703588
LRRN2	1	complement(204617170..20468573	0,33	0,37	0,056332742
NFASC	1	204828651..205022822	0,57	0,76	-0,180888607
CNTN2	1	205042937..205078289	0,16	0,04	2,073656517
TMEM81	1	complement(205083129..20508446	5,38	2,35	1,417006167
RBBP5	1	complement(205086142..20512201	12,14	9,46	0,582539324
DSTYK	1	complement(205142505..20521170	5,80	4,89	0,470193112
TMCC2	1	205227946..205285632	3,59	2,92	0,525012018
NUAK2	1	complement(205302063..20532174	8,06	3,37	1,48105681
KLHDC8A	1	complement(205336061..20535709	-	-	0,188027316
LEMD1	1	complement(205381378..20545595	0,34	0,09	2,175640146
BLACAT1	1	complement(205434885..20545709	1,55	0,91	0,990883954
CDK18	1	205504596..205532793	2,45	2,16	0,406525598
MFSD4A	1	205568885..205602918	0,19	0,16	0,470379593
ELK4	1	complement(205597556..20563201	8,80	8,20	0,326244209
SLC45A3	1	complement(205657851..20568050	4,58	5,90	-0,143322157
NUCKS1	1	complement(205712822..20575018	72,61	71,60	0,243432463
RAB29	1	complement(205767986..20577548	8,09	9,72	-0,042521169
SLC41A1	1	complement(205789094..20581374	15,10	15,20	0,213512821
PM20D1	1	complement(205828025..20585013	0,22	0,16	0,715831587
SLC26A9	1	complement(205913048..20594346	0,03	0,27	-2,848736836
RAB7B	1	complement(205976740..20600346	0,25	1,23	-2,067863108
CTSE	1	complement(206008535..20602390	0,04	0,01	2,469244963
RHEX	1	206053173..206102449	0,01	-	3,068695355
AVPR1B	1	complement(206106936..20611769	0,02	0,01	0,939616118
FAM72A	1	complement(206186179..20620441	5,37	5,66	0,148654919
SRGAP2	1	206203346..206464436	13,34	12,01	0,374896785
IKBKE	1	206470476..206496889	1,78	2,98	-0,521856716
RASSF5	1	206507531..206589448	2,13	1,80	0,469341756
gene:ENSG000	1	206541758..206544892	0,08	0,09	0,085296906
EIF2D	1	complement(206571292..20661246	27,93	19,67	0,728686301
DYRK3	1	206635536..206684419	6,85	7,93	0,010736714
MAPKAPK2	1	206684905..206734281	41,84	34,15	0,516217734
IL10	1	complement(206767602..20677454	-	0,02	-4,758582517
IL19	1	206770764..206842981	-	-	0,188027316
IL20	1	206865623..206869223	-	0,17	-6,809818361
IL24	1	206897443..206904139	1,71	21,09	-3,40023654
FCMR	1	complement(206903317..20692324	0,07	0,24	-1,593583371
PIGR	1	complement(206928522..20694646	0,00	0,02	-2,854330803
FCAMR	1	complement(206957965..20697062	0,01	0,01	0,366665713
C1orf116	1	complement(207018522..20703275	0,07	0,15	-0,812343145
PFKFB2	1	207034366..207081024	0,21	0,43	-0,793540375
YOD1	1	complement(207043849..20705298	3,75	3,79	0,207628091
C4BPB	1	207088860..207099993	0,03	0,01	1,436225396
C4BPA	1	207104233..207144972	0,12	0,02	3,074342434
CD55	1	207321519..207386804	19,64	23,70	-0,048280615
CR2	1	207453024..207489895	0,05	0,06	-0,039232151
CR1	1	207496147..207641765	-	0,00	-2,89252005

CR1L	1	207645113..207738416	0,03	-	5,224730805
CD46	1	207752037..207795513	17,92	19,23	0,121708275
CD34	1	complement(207880972..20791140	0,02	0,03	-0,239612513
PLXNA2	1	complement(208022242..20824438	0,82	1,54	-0,69133989
CAMK1G	1	209583714..209613939	0,03	0,06	-1,024862207
LAMB3	1	complement(209614870..20965242	29,67	10,29	1,750001165
G0S2	1	209675412..209676390	2,92	0,01	7,667319551
HSD11B1	1	209686178..209734949	0,01	0,09	-3,307263034
TRAF3IP3	1	209756032..209782320	0,02	0,00	2,399873901
C1orf74	1	complement(209779208..20978455	2,94	2,51	0,451535326
gene:ENSG000	1	complement(209779629..20980617	0,01	0,04	-1,024862207
IRF6	1	complement(209785617..20980617	0,02	0,07	-1,553849734
UTP25	1	209827972..209857565	2,21	3,45	-0,415666486
SYT14	1	209900923..210171389	1,09	1,21	0,069564636
SERTAD4	1	210232796..210246631	1,06	1,28	-0,051999101
HHAT	1	210328252..210676296	0,54	0,68	-0,125389054
KCNH1	1	complement(210676823..21113416	0,01	0,02	-0,96248933
gene:ENSG000	1	210678315..210681932	0,02	0,06	-1,599259329
gene:ENSG000	1	complement(211082872..21118853	-	-	0,188027316
RCOR3	1	211258377..211316385	2,27	2,44	0,118695148
TRAF5	1	211326615..211374946	2,14	3,37	-0,432087224
RD3	1	complement(211476522..21149216	-	-	0,188027316
SLC30A1	1	complement(211571568..21157916	4,43	4,32	0,258993782
NEK2	1	complement(211658657..21167563	10,66	11,01	0,176189791
LPGAT1	1	complement(211743457..21183076	9,34	8,66	0,33253685
INTS7	1	complement(211940399..21203555	4,94	6,15	-0,09181548
DTL	1	212035553..212107400	11,46	10,30	0,376509286
PPP2R5A	1	212285410..212361853	3,27	2,76	0,470571645
PACC1	1	complement(212363928..21241490	7,56	7,70	0,196623756
NENF	1	212432920..212446379	33,15	24,04	0,686348034
ATF3	1	212565334..212620777	3,43	1,33	1,593542027
GARIN4	1	212624474..212626775	-	0,08	-6,630750384
BATF3	1	complement(212686417..21269984	1,92	4,62	-1,04001139
NSL1	1	complement(212726153..21279178	1,35	1,48	0,092448641
TATDN3	1	212791828..212816830	1,41	2,33	-0,494350766
SPATA45	1	complement(212830141..21284764	0,02	-	3,068695355
FLVCR1	1	212858275..212899363	1,38	1,80	-0,164918791
VASH2	1	212950520..212992037	0,23	0,19	0,499306338
ANGEL2	1	complement(212992182..21301586	2,33	3,86	-0,502682626
RPS6KC1	1	213051233..213274774	9,05	7,05	0,583605536
PROX1	1	213983181..214041510	0,01	0,01	-0,034419864
SMYD2	1	214281102..214337131	10,20	8,06	0,562451028
PTPN14	1	complement(214348700..21455244	27,01	15,47	1,027166401
CENPF	1	214603185..214664574	13,80	20,14	-0,322705329
KCNK2	1	215005775..215237090	-	0,03	-6,187931073
KCTD3	1	215567304..215621807	9,43	10,90	0,013542156
USH2A	1	complement(215622891..21642344	0,02	0,02	0,010647093
ESRRG	1	complement(216503246..21713775	0,01	-	5,224730805

GPATCH2	1	complement(217426992..21763109	1,39	1,53	0,08246439
SPATA17	1	217631324..217871696	0,04	0,09	-0,83425475
RRP15	1	218285293..218337983	1,11	2,16	-0,742577666
TGFB2	1	218345336..218444619	0,28	0,50	-0,591149913
LYPLAL1	1	219173869..219212865	1,17	2,64	-0,951914291
ZC3H11B	1	complement(219608012..21961310	0,01	0,00	1,436225396
SLC30A10	1	complement(219685427..21995901	0,00	0,00	-0,01185201
EPRS1	1	complement(219968600..22004653	40,74	70,72	-0,572487289
BPNT1	1	complement(220057482..22009046	7,15	7,38	0,178178138
IARS2	1	220094132..220148041	33,51	46,84	-0,260053898
RAB3GAP2	1	complement(220148293..22027252	6,31	7,35	0,003841475
MARK1	1	220528136..220664461	-	0,02	-5,902279478
C1orf115	1	220690363..220699153	0,01	-	3,967268276
MTARC2	1	220748225..220784815	0,15	0,52	-1,56152657
MTARC1	1	220786352..220819659	-	0,00	-2,89252005
gene:ENSG000	1	220786990..220884503	-	-	0,188027316
HLX	1	220879431..220885059	2,06	3,29	-0,452397097
DUSP10	1	complement(221701424..22174208	5,64	8,02	-0,286196493
HHIPL2	1	complement(222522258..22254810	3,53	2,64	0,638806571
TAF1A	1	complement(222557902..22258993	1,25	2,48	-0,77303436
MIA3	1	222618097..222668007	7,43	7,76	0,160540017
AIDA	1	complement(222668013..22271321	29,44	29,79	0,206078802
BROX	1	222712553..222735196	8,27	11,48	-0,250402282
FAM177B	1	222737202..222751004	-	0,01	-2,89252005
DISP1	1	222815022..223005995	1,32	2,58	-0,74383191
TLR5	1	complement(223109404..22314329	0,15	0,17	0,03104718
SUSD4	1	complement(223220819..22336423	0,12	0,15	-0,096654898
CCDC185	1	223393415..223395465	-	-	0,188027316
CAPN8	1	complement(223538007..22366572	0,01	0,01	0,524121557
CAPN2	1	223701593..223776018	76,98	84,42	0,089988523
TP53BP2	1	complement(223779893..22384595	7,75	15,97	-0,820598726
FBXO28	1	224114111..224162047	2,97	5,52	-0,67264683
DEGS1	1	224175756..224193441	28,24	37,39	-0,182111084
NVL	1	complement(224227334..22433018	4,87	6,74	-0,246434345
CNIH4	1	224356858..224379459	8,99	15,61	-0,574020434
WDR26	1	complement(224385146..22443703	18,86	18,02	0,288653716
CNIH3	1	224434660..224740554	0,18	3,44	-4,042209031
DNAH14	1	224896262..225399292	0,08	0,27	-1,470089433
LBR	1	complement(225401502..22542892	9,27	14,55	-0,426759196
ENAH	1	complement(225486765..22565314	20,04	12,43	0,912599166
SRP9	1	225777813..225790468	34,58	34,30	0,234995751
EPHX1	1	225810124..225845563	44,65	24,60	1,082955309
TMEM63A	1	complement(225845536..22588238	8,18	8,45	0,174981076
LEFTY1	1	complement(225886282..22591138	0,08	0,07	0,371589373
gene:ENSG000	1	complement(225886696..22592427	0,06	-	6,053060169
PYCR2	1	complement(225919877..22592434	40,72	49,93	-0,070945163
LEFTY2	1	complement(225936598..22594138	-	-	#N/D
SDE2	1	complement(225982702..22599934	14,80	11,16	0,630967568

H3-3A	1	226061851..226072019	28,41	32,18	0,04347938
ACBD3	1	complement(226144679..22618674	36,48	23,36	0,866145167
MIXL1	1	226223618..226227060	0,03	0,04	-0,509043067
LIN9	1	complement(226231149..22630986	1,05	1,94	-0,660801537
PARP1	1	complement(226360210..22640815	44,49	78,97	-0,604617276
STUM	1	226548764..226609230	0,01	0,01	-0,345401666
ITPKB	1	complement(226631690..22673932	5,45	6,58	-0,049327821
PSEN2	1	226870184..226927726	0,64	1,94	-1,377136919
gene:ENSG000	1	226870184..226987545	0,25	0,66	-1,199147384
COQ8A	1	226940286..226987544	5,85	10,15	-0,573615559
CDC42BPA	1	complement(226989865..22731849	5,64	11,31	-0,780815294
ZNF678	1	227563543..227677443	1,35	0,83	0,924524006
SNAP47	1	227728200..227781826	4,79	4,98	0,165540427
JMJD4	1	complement(227730425..22773541	7,29	9,92	-0,221713812
PRSS38	1	227815675..227846470	-	-	#N/D
WNT9A	1	complement(227918656..22794793	0,93	1,03	0,067694082
WNT3A	1	228006998..228061271	0,04	0,02	1,202745498
ARF1	1	228082708..228099212	184,31	#####	0,248156609
C1orf35	1	complement(228100726..22810541	10,82	9,36	0,430973554
MRPL55	1	complement(228106679..22810931	19,66	14,73	0,63975121
gene:ENSG000	1	228121523..228123771	0,35	0,25	0,668366169
GUK1	1	228139962..228148984	42,28	24,53	1,008328568
GJC2	1	228149930..228159826	1,84	0,65	1,717250575
IBA57	1	228165804..228182257	1,41	1,44	0,193083385
OBSCN	1	228208044..228378876	1,11	0,94	0,454767968
TRIM11	1	complement(228393673..22840683	9,56	6,33	0,817757255
TRIM17	1	complement(228407935..22841686	1,16	0,89	0,611530917
H3-4	1	complement(228424845..22842536	0,19	0,25	-0,187130731
H2AC25	1	complement(228434777..22845787	1,59	3,85	-1,050935193
H2BC26	1	228458103..228463104	0,80	2,62	-1,483276241
gene:ENSG000	1	complement(228486188..22848712	0,22	0,40	-0,645709667
RNF187	1	228487382..228499899	51,80	81,70	-0,434469612
RHOU	1	228735479..228746664	0,68	0,92	-0,219533795
RAB4A	1	229271062..229305894	5,63	5,23	0,328450022
CCSAP	1	complement(229321011..22934329	5,30	4,50	0,4605252
ACTA1	1	complement(229430365..22943410	0,01	0,01	0,886720911
NUP133	1	complement(229440259..22950834	9,62	10,72	0,066394462
ABCB10	1	complement(229516582..22955870	4,62	6,54	-0,278569774
TAF5L	1	complement(229593134..22962612	5,04	5,95	-0,015909615
URB2	1	229626247..229660200	4,09	5,84	-0,292088502
GALNT2	1	230057990..230282122	62,80	65,70	0,157911733
PGBD5	1	complement(230314490..23042633	0,07	0,18	-1,148050522
COG2	1	230642481..230693982	4,86	6,27	-0,142710817
AGT	1	complement(230690776..23074557	0,10	0,08	0,504324092
CAPN9	1	230747384..230802003	0,00	-	3,068695355
C1orf198	1	complement(230837119..23086958	7,26	6,13	0,466745149
TTC13	1	complement(230906243..23097887	6,41	10,97	-0,551133359
ARV1	1	230978981..231000733	6,71	6,03	0,376388572

FAM89A	1	complement(231018958..23104025	2,63	5,72	-0,899894623
TRIM67	1	231162058..231221565	0,00	0,01	-0,979341556
C1orf131	1	complement(231223763..23124118	1,03	1,93	-0,684791762
GNPAT	1	231241207..231277973	9,28	14,28	-0,397894493
EXOC8	1	complement(231332753..23133785	6,18	5,21	0,470506183
SPRTN	1	231337104..231355023	1,44	2,03	-0,273624865
EGLN1	1	complement(231363751..23142228	3,37	5,18	-0,398039704
gene:ENSG000	1	complement(231363797..23152860	0,46	0,81	-0,612137455
TSNAX	1	231528541..231566524	6,27	6,71	0,125154706
TSNAX-DISC1	1	231528653..231819244	0,71	0,30	1,44756432
DISC1	1	231626790..232041272	0,54	0,60	0,074418411
SIPA1L2	1	complement(232397965..23263057	18,66	14,43	0,593975711
MAP10	1	232805416..232809929	0,20	0,32	-0,454950411
NTPCR	1	232950605..232983882	2,70	4,39	-0,479306847
PCNX2	1	complement(232983435..23329572	2,81	6,97	-1,086510941
MAP3K21	1	233327724..233385148	1,96	2,89	-0,337770276
KCNK1	1	233614106..233672514	5,24	5,80	0,077347696
SLC35F3	1	233904676..234324511	1,37	0,85	0,916675911
COA6	1	234373456..234385080	1,07	1,72	-0,455562721
TARBP1	1	complement(234391313..23447917	3,17	5,12	-0,470884529
IRF2BP2	1	complement(234604269..23461017	36,67	32,51	0,396726586
TOMM20	1	complement(235109341..23512883	53,50	62,74	-0,006647057
RBM34	1	complement(235131183..23516128	2,54	4,10	-0,468284827
ARID4B	1	complement(235131634..23532821	2,80	2,89	0,179699077
GGPS1	1	235327350..235344532	2,60	4,09	-0,432495681
gene:ENSG000	1	235328570..235448952	2,16	2,62	-0,053436758
TBCE	1	235367360..235452443	1,93	3,43	-0,604393539
B3GALNT2	1	complement(235447190..23550445	1,97	4,61	-1,00397816
GNG4	1	complement(235547685..23565075	4,03	8,69	-0,884108447
LYST	1	complement(235661041..23588372	0,87	2,02	-0,987782582
NID1	1	complement(235975830..23606510	24,21	37,18	-0,395994242
GPR137B	1	236142505..236221865	2,76	3,22	-0,002208824
ERO1B	1	complement(236214681..23628201	0,77	0,84	0,097057426
EDARADD	1	236348257..236502915	0,28	0,21	0,658715752
LGALS8	1	236518000..236552981	2,92	4,33	-0,342475324
HEATR1	1	complement(236549005..23660451	9,99	17,95	-0,621578812
ACTN2	1	236664141..236764631	0,06	0,42	-2,523447975
MTR	1	236795260..236921278	6,76	9,56	-0,277439253
MT1HL1	1	complement(237004103..23700444	0,13	0,04	1,833201638
RYR2	1	237042184..237833988	0,04	0,13	-1,508457995
ZP4	1	complement(237877864..23789092	-	-	0,188027316
CHRM3	1	239386565..239915452	0,01	0,03	-1,42562615
FMN2	1	240014348..240475187	2,76	4,21	-0,386913318
GREM2	1	complement(240489573..24061215	0,06	0,04	0,713573923
RGS7	1	complement(240767636..24135737	0,01	0,01	-0,032860829
FH	1	complement(241497511..24151979	15,44	18,32	-0,023917141
KMO	1	241532134..241595642	0,01	0,02	-1,015460095
OPN3	1	complement(241590102..24167737	2,44	3,56	-0,317975367

CHML	1	complement(241628851..24164036	1,54	2,05	-0,185225972
WDR64	1	241652278..241802777	0,00	0,00	-0,01185201
EXO1	1	241847967..241895148	6,92	8,76	-0,11690739
BECN2	1	241957767..241959062	-	0,01	-2,89252005
MAP1LC3C	1	complement(241995490..24199909	0,12	0,03	2,028855185
PLD5	1	complement(242082986..24252469	-	-	0,188027316
CEP170	1	complement(243124428..24325579	11,43	17,84	-0,419759523
SDCCAG8	1	243256034..243500091	2,37	4,34	-0,648409147
AKT3	1	complement(243488233..24385107	7,31	10,41	-0,285512669
ZBTB18	1	244048547..244057476	6,11	4,09	0,801754697
gene:ENSG000	1	244064330..244067909	-	-	0,188027316
SPMIP3	1	244352635..244389663	-	0,06	-5,071123353
ADSS2	1	complement(244408494..24445190	5,07	9,29	-0,650770378
CATSPERE	1	244454377..244641177	0,01	-	3,967268276
DESI2	1	244653103..244709033	13,38	16,02	-0,036559013
C1orf202	1	complement(244729701..24473101	-	0,01	-2,89252005
COX20	1	244835616..244845057	6,24	4,80	0,600818559
HNRNPU	1	complement(244840638..24486456	11,02	18,61	-0,532701181
EFCAB2	1	244969682..245127164	0,36	1,18	-1,510424175
KIF26B	1	245154985..245709432	0,05	0,08	-0,442944103
SMYD3	1	complement(245749342..24650731	6,94	8,53	-0,075857383
TFB2M	1	complement(246540561..24656626	10,65	8,83	0,494157336
CNST	1	246566444..246668595	7,10	8,71	-0,072642483
SCCPDH	1	246724409..246768137	2,48	7,21	-1,318448257
AHCTF1	1	complement(246839098..24693194	11,54	18,80	-0,481192027
ZNF695	1	complement(246945547..24700809	2,01	2,36	-0,011694743
ZNF670-ZNF69	1	complement(246945547..24707881	0,21	0,23	0,078678574
ZNF670	1	complement(247034637..24707881	0,44	1,04	-1,02733789
ZNF669	1	complement(247099962..24710437	2,09	2,69	-0,142555763
ZNF124	1	complement(247121975..24717202	1,29	1,47	0,036270595
VN1R5	1	247255972..247257210	-	0,01	-2,89252005
ZNF496	1	complement(247297412..24733186	18,10	19,02	0,15196282
NLRP3	1	247332331..247449108	0,08	0,45	-2,222748653
OR2B11	1	complement(247449118..24745810	0,00	0,01	-0,579815015
GCSAML	1	247507058..247577690	-	0,00	-2,89252005
OR2C3	1	complement(247524677..24753644	-	-	#N/D
OR2G2	1	247588360..247589313	-	-	#N/D
OR2G3	1	247605586..247606515	-	-	#N/D
OR14L1	1	247617143..247622372	-	-	#N/D
OR13G1	1	complement(247670812..24767973	-	-	#N/D
OR6F1	1	complement(247711436..24771664	-	-	#N/D
gene:ENSG000	1	complement(247711813..24772376	-	-	#N/D
OR14A2	1	complement(247722957..24774814	-	-	#N/D
OR14K1	1	247738615..247739559	-	-	#N/D
OR1C1	1	complement(247754846..24776055	0,24	0,14	0,99036649
OR9H1P	1	247770169..247779524	-	-	0,188027316
OR14A16	1	complement(247814660..24782411	-	-	#N/D
OR11L1	1	complement(247840928..24784189	-	-	#N/D

TRIM58	1	247857187..247880138	1,76	2,70	-0,391759583
OR2W3	1	247895587..247896531	1,30	2,33	-0,616534205
OR2T8	1	247920252..247923152	0,07	0,13	-0,711958745
OR2AJ1	1	247924889..247935339	-	-	#N/D
OR2L8	1	247948858..247949796	0,07	0,04	0,933337975
OR2AK2	1	247965233..247966386	0,05	0,03	0,677647514
OR2L5	1	248013660..248024276	0,03	0,01	2,616997665
OR2L2	1	248030070..248042305	0,01	0,01	0,524121557
OR2L3	1	248046836..248063407	0,01	-	4,913749003
OR2L13	1	248095184..248101163	0,19	0,03	2,775224447
OR2M5	1	248145148..248146086	-	0,08	-5,327865481
OR2M2	1	248174821..248181067	-	0,01	-2,89252005
OR2M3	1	248197265..248212925	0,01	0,02	-0,613815027
OR2M4	1	248231417..248244679	-	0,05	-7,112521263
OR2T33	1	complement(248269917..24827797	0,01	0,02	-0,999120035
OR2T12	1	complement(248290139..24830342	-	-	0,188027316
OR2M7	1	complement(248323630..24832456	-	-	#N/D
OR14C36	1	248348775..248349713	-	-	#N/D
OR2T4	1	248361665..248362627	0,03	-	4,516772761
OR2T6	1	248375746..248391811	0,00	0,00	0,886720911
OR2T1	1	248403048..248408020	-	-	#N/D
OR2T7	1	248436359..248444316	-	-	#N/D
OR2T2	1	248445512..248455725	-	-	#N/D
OR2T3	1	248473351..248474307	-	-	#N/D
OR2T5	1	248488589..248491113	-	-	#N/D
OR2G6	1	248508073..248527337	0,00	-	3,068695355
OR2T29	1	complement(248556912..24856277	-	-	0,188027316
OR2T34	1	complement(248573801..24857475	-	-	#N/D
OR2T10	1	complement(248590487..24859770	-	-	#N/D
OR2T11	1	complement(248623557..24863509	-	-	#N/D
OR2T35	1	complement(248636356..24864527	-	-	#N/D
OR2T27	1	complement(248649838..24865552	-	-	0,188027316
OR14I1	1	complement(248678138..24870276	-	-	0,188027316
gene:ENSG000	1	complement(248681249..24874834	-	-	0,188027316
LYPD8	1	complement(248739415..24875575	0,02	-	3,967268276
SH3BP5L	1	complement(248810446..24882591	18,37	16,74	0,356854227
ZNF672	1	248838210..248849517	5,10	5,65	0,073188275
ZNF692	1	complement(248850006..24885914	13,37	15,61	-0,000962752
PGBD2	1	248906196..248919946	0,38	0,61	-0,446667185
FAM110C	2	complement(38814..46870)	-	-	0,188027316
SH3YL1	2	complement(217730..266398)	0,71	0,62	0,427621122
ACP1	2	264140..278283	17,11	17,47	0,192359347
ALKAL2	2	complement(279558..288851)	0,02	0,04	-0,742377908
TMEM18	2	complement(663877..677406)	1,49	2,09	-0,269348857
gene:ENSG000	2	687799..688348	0,06	-	4,516772761
SNTG2	2	950849..1367613	0,01	-	3,967268276
TPO	2	1374066..1543711	-	0,01	-4,359055975
PXDN	2	complement(1631887..1744852)	89,10	#####	-0,035055993

MYT1L	2	complement(1789113..2331664)	0,00	0,00	1,487770062
EIPR1	2	complement(3188925..3377830)	7,70	10,96	-0,286465347
TRAPPC12	2	3379675..3485094	6,29	8,27	-0,173471342
ADI1	2	complement(3497366..3519531)	6,26	6,99	0,065038771
gene:ENSG000	2	complement(3532008..3558333)	0,58	2,80	-2,059039885
RNASEH1	2	complement(3541430..3558333)	12,26	12,47	0,198424965
RPS7	2	3575260..3580920	356,81	#####	0,01948309
COLEC11	2	3594832..3644644	0,09	0,06	0,838252066
ALLC	2	3658200..3702671	-	-	0,188027316
DCDC2C	2	3703575..3848008	0,01	-	3,967268276
SOX11	2	5692384..5701385	0,88	-	12,37811262
CMPK2	2	complement(6840570..6866635)	0,28	0,16	1,000097356
RSAD2	2	6865557..6898239	0,16	0,01	5,078029536
RNF144A	2	6917412..7068286	2,47	1,73	0,738974511
ID2	2	8678845..8684461	2,04	0,99	1,261064935
KIDINS220	2	complement(8721081..8837630)	2,87	2,97	0,172515838
MBOAT2	2	complement(8852690..9003709)	5,93	8,79	-0,34453205
ASAP2	2	9206765..9405683	6,99	11,97	-0,553681082
ITGB1BP1	2	complement(9403475..9423528)	5,10	6,16	-0,048428304
CPSF3	2	9423651..9473101	8,60	13,02	-0,375293309
IAH1	2	9473658..9496543	4,04	2,61	0,853993469
ADAM17	2	complement(9488486..9556732)	9,62	9,48	0,245318775
YWHAQ	2	complement(9583967..9630997)	122,61	#####	-0,086195721
TAF1B	2	9843443..9934416	1,68	3,07	-0,64153208
GRHL1	2	9951693..10002277	0,17	0,37	-0,926376786
KLF11	2	10042849..10054836	9,03	6,24	0,755890739
CYS1	2	complement(10056473..10080944)	1,06	0,88	0,482507336
RRM2	2	10120698..10211725	22,08	20,77	0,311542264
HPCAL1	2	10302889..10427617	45,75	41,84	0,351954859
ODC1	2	complement(10439968..10448327)	16,94	32,53	-0,717858411
NOL10	2	complement(10562347..10689987)	2,07	2,97	-0,294252771
ATP6V1C2	2	10721100..10785110	1,11	0,44	1,543073063
PDIA6	2	complement(10783391..10837977)	82,32	67,56	0,508218682
KCNF1	2	10911934..10914225	1,45	1,53	0,143776779
C2orf50	2	11133128..11151317	0,00	0,01	-0,999120035
SLC66A3	2	11155198..11178870	2,21	1,68	0,622469641
ROCK2	2	complement(11179759..11348330)	6,98	7,47	0,125034383
E2F6	2	complement(11444375..11466177)	2,67	5,07	-0,703547783
GREB1	2	11482341..11642788	0,63	0,35	1,056288763
NTSR2	2	complement(11658178..11670195)	-	-	#N/D
LPIN1	2	11677595..11827409	24,32	8,67	1,711269053
TRIB2	2	12716910..12742734	40,97	7,74	2,626852466
LRATD1	2	14632700..14650814	0,12	0,36	-1,352982508
NBAS	2	complement(15166916..15561340)	7,57	7,52	0,232529259
DDX1	2	15591178..15634346	9,19	13,64	-0,346240747
MYCN	2	15940550..15947007	0,00	0,03	-2,447197442
gene:ENSG000	2	complement(16541690..16545695)	-	-	0,188027316
CYRIA	2	complement(16549459..16666331)	2,07	0,47	2,361663624

RAD51AP2	2	complement(17510579..17518440)	0,02	0,05	-1,133870313
VSNL1	2	17539126..17657018	6,51	2,08	1,866632018
SMC6	2	complement(17663812..17800242)	2,06	3,90	-0,698311621
GEN1	2	17753858..17788946	0,39	0,96	-1,063673948
MSGN1	2	17816460..17817798	-	0,01	-2,89252005
KCNS3	2	17877847..18361616	3,23	3,61	0,060123826
RDH14	2	complement(18554723..18560679)	3,64	6,06	-0,513550676
NT5C1B-RDH14	2	complement(18555545..18589564)	-	-	#N/D
NT5C1B	2	complement(18562871..18589572)	0,02	0,07	-1,43066689
OSR1	2	complement(19351485..19358623)	0,13	0,69	-2,181819801
TTC32	2	complement(19896631..19901983)	1,64	1,97	-0,04074212
WDR35	2	complement(19910263..19990105)	0,74	2,23	-1,375646562
MATN3	2	complement(19992052..20012668)	0,09	0,01	4,247318137
LAPTM4A	2	complement(20032650..20051628)	69,38	83,18	-0,038577725
SDC1	2	complement(20200797..20225433)	46,90	76,56	-0,483740376
PUM2	2	complement(20248691..20352234)	9,76	14,61	-0,359049966
RHOB	2	20447074..20449440	4,20	3,63	0,436392693
HS1BP3	2	complement(20560448..20651130)	2,19	2,33	0,137559042
GDF7	2	20667144..20679243	0,11	0,17	-0,431610725
LDAH	2	complement(20684014..20823130)	1,50	2,13	-0,286112123
APOB	2	complement(21001429..21044073)	-	-	0,188027316
gene:ENSG000	2	21023496..21024170	-	-	#N/D
TDRD15	2	21123968..21144368	-	-	#N/D
KLHL29	2	23385179..23708611	0,91	2,16	-1,024081002
gene:ENSG000	2	complement(23616499..23618000)	0,01	0,07	-1,955757882
ATAD2B	2	complement(23748664..23927123)	1,14	1,15	0,214832297
UBXN2A	2	23927285..24004909	1,24	2,35	-0,703399532
MFSD2B	2	24010081..24063321	0,10	0,10	0,258323606
WDCP	2	complement(24029347..24049575)	2,91	3,75	-0,140834528
FKBP1B	2	24049701..24063681	0,25	0,52	-0,836322869
SF3B6	2	complement(24067586..24076373)	17,85	29,59	-0,505820023
FAM228B	2	24076526..24169640	0,33	0,50	-0,385798402
TP53I3	2	complement(24077433..24085861)	0,72	0,66	0,34359785
PFN4	2	complement(24114809..24123464)	0,11	0,23	-0,891420415
gene:ENSG000	2	24124366..24190436	0,06	0,06	0,335747576
FAM228A	2	24175053..24200849	0,04	0,02	1,655502569
ITSN2	2	complement(24202864..24360536)	2,15	3,82	-0,602509633
NCOA1	2	24491254..24770702	1,41	2,32	-0,495517122
PTRHD1	2	complement(24789728..24793391)	6,30	7,92	-0,107632082
CENPO	2	24793136..24822376	15,45	15,21	0,245475542
ADCY3	2	complement(24819169..24920237)	11,64	19,75	-0,539648912
DNAJC27	2	complement(24943636..24972094)	0,86	0,79	0,335806287
EFR3B	2	25042076..25159135	0,17	0,32	-0,678317122
POMC	2	complement(25160853..25168903)	-	-	#N/D
DNMT3A	2	complement(25227855..25342590)	2,94	2,44	0,491012849
DTNB	2	complement(25377198..25673647)	4,00	4,86	-0,056791737
ASXL2	2	complement(25733753..25878487)	3,41	4,83	-0,280256769
KIF3C	2	complement(25926598..25982749)	5,70	8,32	-0,32281323

RAB10	2	26034084..26137454	13,96	22,16	-0,443488269
GAREM2	2	26173088..26189663	0,98	2,07	-0,856000163
HADHA	2	complement(26190635..26244672)	48,98	44,08	0,375094918
HADHB	2	26243170..26290465	43,34	36,24	0,481094959
ADGRF3	2	complement(26308173..26346817)	0,16	0,16	0,181522713
SELENOI	2	26308547..26395885	3,54	4,53	-0,131487418
DRC1	2	26401920..26456711	0,01	0,01	0,524121557
OTOF	2	complement(26457203..26558756)	0,00	0,01	-2,190455314
CIMIP2C	2	26562585..26579532	0,01	-	3,068695355
CIB4	2	complement(26581205..26641366)	0,03	-	3,967268276
KCNK3	2	26692722..26733420	0,31	0,14	1,410783062
SLC35F6	2	26764284..26781231	23,63	20,71	0,413597206
CENPA	2	26764289..26801067	16,05	17,94	0,061824989
DPYSL5	2	26847747..26950351	0,00	-	3,068695355
MAPRE3	2	26970637..27027219	10,23	21,95	-0,87834249
TMEM214	2	27032910..27041694	61,91	34,51	1,066208846
AGBL5	2	27042364..27070622	17,49	16,76	0,284803361
OST4	2	complement(27070472..27071654)	88,22	75,59	0,44594619
EMILIN1	2	27078615..27086403	27,71	38,13	-0,237488741
KHK	2	27086772..27100762	0,64	0,90	-0,271084065
CGREF1	2	complement(27098889..27119128)	1,93	1,69	0,416124542
ABHD1	2	27123789..27130812	0,56	0,17	1,970773164
PREB	2	complement(27130756..27134666)	41,83	26,96	0,856609213
PRR30	2	complement(27136848..27139410)	-	0,02	-4,359055975
TCF23	2	27149004..27156974	0,02	0,01	1,704893826
SLC5A6	2	complement(27199587..27212958)	3,10	8,44	-1,221442914
ATRAID	2	27212041..27217178	40,60	55,74	-0,234336338
CAD	2	27217369..27243943	21,61	27,51	-0,125374237
SLC30A3	2	complement(27253684..27275817)	0,17	0,20	-0,015715007
DNAJC5G	2	27275433..27281499	-	0,01	-2,89252005
TRIM54	2	27282429..27307439	0,03	0,05	-0,698295353
UCN	2	complement(27307400..27308445)	1,11	1,09	0,250457617
MPV17	2	complement(27309492..27325680)	11,76	10,29	0,416127914
GTF3C2	2	complement(27325849..27356999)	15,70	16,03	0,192708839
EIF2B4	2	complement(27364352..27370338)	11,51	14,13	-0,072150083
SNX17	2	27370496..27377535	43,83	49,74	0,040419179
ZNF513	2	complement(27377235..27380790)	5,59	3,04	1,100689962
PPM1G	2	complement(27381195..27409591)	30,12	42,59	-0,276657953
NRBP1	2	27427790..27442259	29,77	34,34	0,016807095
KRTCAP3	2	27442366..27446481	0,52	0,45	0,43489398
IFT172	2	complement(27444377..27489805)	3,23	3,07	0,296492063
FNDC4	2	complement(27491883..27495200)	2,93	1,38	1,307032735
GCKR	2	27496839..27523684	0,09	0,12	-0,230810911
SPATA31H1	2	27537386..27582722	0,14	0,33	-1,002724481
ZNF512	2	27582969..27623217	14,17	12,78	0,372173808
CCDC121	2	complement(27625638..27629012)	0,17	0,41	-1,006402385
GPN1	2	27628247..27651511	6,97	10,66	-0,389919189
SUPT7L	2	complement(27650809..27663840)	4,74	7,32	-0,40541063

SLC4A1AP	2	27663426..27695366	2,75	3,67	-0,192937772
MRPL33	2	27771717..27988087	3,48	5,51	-0,441585871
RBKS	2	complement(27781379..27890681)	0,35	0,63	-0,648470902
BABAM2	2	27889941..28338901	7,32	15,14	-0,824578637
FOSL2	2	28392448..28417317	20,06	20,79	0,171228101
PLB1	2	28457145..28644142	0,02	0,09	-1,643292087
PPP1CB	2	28751640..28802940	9,46	13,86	-0,328012399
SPDYA	2	28782517..28850611	0,01	0,05	-2,854330803
TRMT61B	2	complement(28849821..28870309)	3,25	5,08	-0,422249962
WDR43	2	28894667..28948219	9,11	13,34	-0,326602933
TOGARAM2	2	28956611..29052230	1,60	2,07	-0,147552901
PCARE	2	complement(29060976..29074523)	0,00	0,01	-1,955757882
CLIP4	2	29097705..29199777	1,46	2,57	-0,591239223
ALK	2	complement(29192774..29921586)	0,07	0,03	1,569539429
YPEL5	2	30146941..30160533	10,88	7,08	0,843458342
LBH	2	30231534..30323730	7,58	3,69	1,261620392
gene:ENSG000	2	30408170..30408346	-	-	#N/D
LCLAT1	2	30447226..30644225	2,44	4,01	-0,493146342
gene:ENSG000	2	30677762..30679895	-	0,05	-5,902279478
CAPN13	2	complement(30722771..30820542)	-	-	#N/D
GALNT14	2	complement(30910467..31155202)	1,95	1,27	0,841451004
CAPN14	2	complement(31173056..31233858)	0,08	-	7,72394349
EHD3	2	31234152..31269451	1,26	0,45	1,720306639
XDH	2	complement(31334321..31414742)	0,45	0,55	-0,052661606
SRD5A2	2	complement(31522480..31580938)	-	-	0,188027316
MEMO1	2	complement(31865060..32011230)	1,89	3,31	-0,582446554
DPY30	2	complement(31867809..32039805)	8,22	11,20	-0,22311346
SPAST	2	32063556..32157637	1,23	2,07	-0,523768196
SLC30A6	2	32165841..32224379	2,28	4,35	-0,704948833
NLRC4	2	complement(32224453..32265732)	0,04	0,13	-1,33363117
YIPF4	2	32277904..32316594	1,65	2,65	-0,463566525
BIRC6	2	32357023..32619571	10,20	9,47	0,330378379
gene:ENSG000	2	32563328..32566436	0,18	0,11	0,956049759
TTC27	2	32628032..32821051	5,00	6,76	-0,210171739
LTBP1	2	32946953..33399509	11,25	46,54	-1,825556462
RASGRP3	2	33436324..33564750	4,39	1,01	2,336495374
FAM98A	2	complement(33532744..33599347)	8,25	5,83	0,722847971
CRIM1	2	36355778..36551135	41,11	38,53	0,316636009
FEZ2	2	complement(36531805..36646087)	8,98	11,93	-0,186145014
VIT	2	36696690..36814794	0,68	0,24	1,704554862
STRN	2	complement(36837698..36966536)	1,96	2,41	-0,074086109
gene:ENSG000	2	complement(36839922..36842539)	-	-	#N/D
HEATR5B	2	complement(36968383..37084372)	3,13	2,88	0,339774556
GPATCH11	2	37084518..37099244	2,55	2,81	0,085242985
EIF2AK2	2	complement(37099210..37157522)	8,21	9,47	0,017385131
SULT6B1	2	complement(37167820..37196598)	-	0,01	-2,89252005
CEBPZOS	2	37196488..37216193	7,13	8,56	-0,040306327
CEBPZ	2	complement(37201612..37231596)	6,23	8,48	-0,221853418

NDUFAF7	2	37231631..37253403	2,99	3,81	-0,123392415
PRKD3	2	complement(37250502..37324833)	5,68	8,16	-0,298559143
QPCT	2	37342827..37373322	0,09	0,04	1,38420186
CDC42EP3	2	complement(37641882..37738468)	10,35	19,21	-0,669064935
RMDN2	2	37923187..38067142	0,04	0,09	-1,148773034
CYP1B1	2	complement(38066973..38109902)	1,64	2,96	-0,630333373
gene:ENSG000	2	complement(38121935..38123881)	0,01	0,06	-2,123038518
ATL2	2	complement(38293954..38377285)	3,84	5,45	-0,284037979
HNRNPLL	2	complement(38561969..38603586)	4,31	4,44	0,182174577
GALM	2	38666081..38741237	2,33	2,02	0,42790613
SRSF7	2	complement(38743599..38751494)	13,91	15,49	0,067873771
GEMIN6	2	38751534..38785002	1,28	1,47	0,013292371
DHX57	2	complement(38797729..38875934)	2,68	4,38	-0,487553681
MORN2	2	38875976..38929072	0,68	0,62	0,345831909
ARHGEF33	2	38889875..38975454	0,02	0,03	-0,517568707
SOS1	2	complement(38962206..39124345)	1,58	2,69	-0,541937686
CDKL4	2	complement(39168045..39243983)	0,01	0,03	-1,766505595
MAP4K3	2	complement(39249266..39437301)	1,48	3,18	-0,882480178
TMEM178A	2	39664982..39717963	0,36	0,60	-0,524704094
THUMPD2	2	complement(39736060..39779267)	0,79	1,82	-0,992690832
SLC8A1	2	complement(40097270..40611053)	0,06	0,06	0,200108858
PKDCC	2	42048021..42058517	1,99	2,93	-0,334794469
EML4	2	42169353..42332548	4,37	7,13	-0,482424992
COX7A2L	2	complement(42333546..42425088)	9,66	7,84	0,523545361
KCNG3	2	complement(42442017..42493982)	0,06	0,13	-0,852483336
MTA3	2	42494569..42756947	1,95	3,84	-0,755886673
OXER1	2	complement(42762499..42764135)	0,01	0,19	-3,524780994
HAAO	2	complement(42767089..42792593)	-	0,03	-4,359055975
ZFP36L2	2	complement(43222402..43226606)	21,41	24,07	0,054066857
THADA	2	complement(43230851..43596038)	2,43	3,83	-0,431207935
PLEKHH2	2	43637260..43767987	1,48	0,84	1,053214611
C1GALT1C1L	2	complement(43675151..43676429)	0,09	0,69	-2,651295567
DYNC2LI1	2	43774039..43810010	0,62	1,24	-0,782116263
ABCG5	2	complement(43812472..43838865)	0,00	0,02	-1,877914477
ABCG8	2	43831942..43882988	0,01	0,03	-1,026620426
LRPPRC	2	complement(43886224..43996226)	7,81	13,38	-0,554297898
PPM1B	2	44167969..44244384	1,27	1,76	-0,251085573
gene:ENSG000	2	44168851..44320310	0,02	0,02	0,750935198
SLC3A1	2	44275458..44321494	0,11	0,04	1,648411306
PREPL	2	complement(44316281..44361862)	6,83	7,30	0,125480562
CAMKMT	2	44361947..44772592	0,64	0,98	-0,390458435
gene:ENSG000	2	complement(44927914..44928396)	0,02	-	3,068695355
SIX3	2	44941702..44946071	0,02	0,01	0,366665713
SIX2	2	complement(45005182..45009452)	2,60	5,25	-0,792235445
SRBD1	2	complement(45388680..45612165)	2,00	3,86	-0,726732524
PRKCE	2	45651345..46187990	2,34	3,94	-0,525806317
EPAS1	2	46293667..46386697	84,02	50,38	0,960834713
TMEM247	2	46479565..46484425	-	0,06	-4,359055975

ATP6V1E2	2	complement(46490750..46542577)	0,96	2,42	-1,113058896
RHOQ	2	46541806..46584688	4,31	6,27	-0,318857174
PIGF	2	complement(46580937..46617055)	3,90	3,66	0,315729633
CRIPT	2	46616416..46630176	2,92	1,88	0,863827154
gene:ENSG000	2	46668870..46670778	0,28	0,18	0,870585929
SOCS5	2	46698952..46780245	5,37	4,39	0,514615303
MCFD2	2	complement(46901870..46941855)	46,91	30,83	0,828708837
TTC7A	2	46915869..47076137	11,13	9,36	0,473546432
STPG4	2	complement(47045538..47155308)	0,01	0,08	-2,408690112
gene:ENSG000	2	complement(47065941..47176511)	0,04	0,01	1,833201638
CALM2	2	complement(47160084..47176921)	37,18	72,53	-0,740749312
EPCAM	2	47345158..47387601	0,02	0,03	-0,034419864
MSH2	2	47403067..47663146	1,98	1,98	0,217492577
KCNK12	2	complement(47509290..47570985)	0,12	0,18	-0,294226751
MSH6	2	47695530..47810063	11,11	13,69	-0,078466942
FBXO11	2	complement(47789316..47906498)	4,19	5,64	-0,205285815
FOXN2	2	48314637..48379295	1,90	2,52	-0,185788665
PPP1R21	2	48440598..48515391	1,10	2,20	-0,779767613
STON1	2	48529383..48598513	2,35	2,22	0,305258084
STON1-GTF2A1	2	48529925..48776517	0,02	0,04	-0,613815027
GTF2A1L	2	48617798..48733148	-	-	0,188027316
gene:ENSG000	2	complement(48632291..48755724)	-	0,01	-3,804623889
LHCGR	2	complement(48686774..48755730)	0,00	-	3,068695355
FSHR	2	complement(48962157..49154527)	-	-	#N/D
NRXN1	2	complement(49918503..51225575)	0,00	-	3,068695355
ASB3	2	complement(53532672..53860160)	2,57	3,01	-0,00095136
CHAC2	2	53767804..53775196	0,16	0,47	-1,370061022
ERLEC1	2	53787009..53833038	5,35	5,13	0,281723918
GPR75	2	complement(53852912..53859967)	0,55	0,68	-0,093942634
PSME4	2	complement(53864069..53970993)	16,87	15,70	0,326399603
ACYP2	2	53970838..54305300	0,36	0,58	-0,477265688
TSPYL6	2	complement(54253178..54256229)	0,01	0,01	0,366665713
C2orf73	2	54330034..54383742	-	-	0,188027316
SPTBN1	2	54456327..54671446	23,25	41,21	-0,60234866
EML6	2	54723499..54972025	0,36	0,85	-1,027946447
RTN4	2	complement(54972187..55112621)	33,99	44,24	-0,157419785
CLHC1	2	complement(55172547..55232563)	0,34	0,66	-0,752503294
RPS27A	2	55231903..55235853	106,43	#####	-0,343343251
MTIF2	2	complement(55236595..55269347)	4,22	7,24	-0,557463464
CCDC88A	2	complement(55287842..55419895)	3,18	4,73	-0,349832977
CFAP36	2	55519604..55545079	2,78	3,35	-0,04568335
PPP4R3B	2	complement(55547292..55618880)	5,39	8,64	-0,457985388
PNPT1	2	complement(55634061..55693863)	2,89	6,79	-1,008123458
EFEMP1	2	complement(55865967..55924139)	0,30	4,96	-3,816413171
CCDC85A	2	56183990..56386172	0,00	-	3,068695355
VRK2	2	57907629..58159920	1,94	4,85	-1,095720356
FANCL	2	complement(58159243..58241410)	0,55	1,08	-0,7378463
BCL11A	2	complement(60450520..60554467)	0,00	0,00	0,537167373

PAPOLG	2	60756253..60802086	1,19	1,89	-0,441161964
REL	2	60881491..60931612	1,04	2,14	-0,823772118
PUS10	2	complement(60940222..61018259)	0,37	0,52	-0,275402671
PEX13	2	61017225..61051990	2,71	2,47	0,35893355
SANBR	2	61065871..61138034	0,38	1,28	-1,532913534
C2orf74	2	61145068..61164828	0,93	1,52	-0,482550308
USP34	2	complement(61187463..61471087)	7,80	11,04	-0,277515637
XPO1	2	complement(61476032..61538741)	10,59	18,41	-0,575036064
FAM161A	2	complement(61824848..61854143)	0,47	0,85	-0,637862646
CCT4	2	complement(61868085..61888671)	82,87	#####	-0,223648614
COMMD1	2	61888724..62147247	2,78	3,44	-0,080919639
B3GNT2	2	62196115..62224731	2,84	3,56	-0,106458273
TMEM17	2	complement(62500218..62511894)	0,51	0,55	0,115378953
EHBP1	2	62673851..63046487	3,66	6,03	-0,49930582
OTX1	2	63050057..63057836	0,75	0,92	-0,074335959
WDPCP	2	complement(63119559..63827843)	0,32	0,39	-0,092291962
gene:ENSG000	2	complement(63517892..63519268)	0,13	0,03	2,411819079
MDH1	2	63588609..63607197	32,09	37,84	-0,014796082
UGP2	2	63840952..63891562	7,99	10,42	-0,160535649
VPS54	2	complement(63892146..64019428)	1,91	3,82	-0,7753453
PELI1	2	complement(64092652..64144420)	0,82	1,83	-0,939442275
LGALSL	2	64453969..64461381	1,95	2,62	-0,2057561
AFTPH	2	64524299..64593005	4,03	4,44	0,083244857
SERTAD2	2	complement(64631621..64751005)	10,37	13,63	-0,17157727
SLC1A4	2	64988477..65023865	2,48	7,71	-1,412043021
CEP68	2	65056354..65087004	1,79	3,13	-0,582361158
RAB1A	2	complement(65070696..65130331)	19,43	17,43	0,379717076
ACTR2	2	65227788..65271253	38,88	46,65	-0,039791066
SPRED2	2	complement(65310851..65432637)	7,43	9,42	-0,119033561
gene:ENSG000	2	65790039..65793533	-	0,01	-4,359055975
MEIS1	2	66433452..66573869	0,27	0,73	-1,212373679
ETAA1	2	67397322..67412089	0,28	0,76	-1,207619287
C1D	2	complement(68041130..68110948)	1,52	2,26	-0,351021176
DNAAF10	2	complement(68122936..68157549)	2,00	2,78	-0,254136746
gene:ENSG000	2	complement(68131238..68261230)	0,45	0,57	-0,127881547
PNO1	2	68157888..68176238	7,47	10,89	-0,321052663
PPP3R1	2	complement(68178857..68256237)	11,84	14,74	-0,092642133
CNRIP1	2	complement(68284171..68320051)	11,70	19,14	-0,48672355
PLEK	2	68365282..68397453	0,01	0,03	-1,766505595
FBXO48	2	complement(68459422..68467294)	0,37	0,86	-0,99873585
APLF	2	68467572..68655862	0,23	0,68	-1,364484473
PROKR1	2	68643579..68658251	-	0,00	-2,89252005
ARHGAP25	2	68679601..68826833	0,02	0,20	-2,936294647
BMP10	2	complement(68860909..68871397)	-	0,01	-4,758582517
GKN2	2	complement(68945232..68952893)	-	0,01	-2,89252005
GKN1	2	68974573..68980980	-	-	0,188027316
ANTXR1	2	69013176..69249327	7,77	10,41	-0,19879704
GFPT1	2	complement(69319780..69387250)	21,54	15,03	0,74220856

NFU1	2	complement(69396113..69437628)	2,85	5,02	-0,592552486
AAK1	2	complement(69457997..69674349)	3,53	4,87	-0,239407456
ANXA4	2	69644425..69827112	2,20	4,39	-0,771721343
GMCL1	2	69829660..69881384	1,21	1,34	0,077973282
SNRNP27	2	69893956..69905575	4,65	5,62	-0,048521332
MXD1	2	69897688..69942945	3,11	3,30	0,135289222
ASPRV1	2	complement(69960089..69961631)	0,36	0,57	-0,45432432
PCBP1	2	70087477..70089203	89,95	#####	0,049086972
C2orf42	2	complement(70149885..70248615)	1,68	2,69	-0,452969968
TIA1	2	complement(70209444..70248660)	3,53	4,83	-0,227946058
PCYOX1	2	70257386..70281185	4,39	7,20	-0,490347322
SNRPG	2	complement(70281362..70293740)	5,18	10,18	-0,751891957
FAM136A	2	complement(70295976..70302067)	7,46	11,11	-0,351675225
TGFA	2	complement(70447284..70554193)	2,63	5,52	-0,846004259
ADD2	2	complement(70607618..70768225)	3,85	10,96	-1,286054198
FIGLA	2	complement(70777310..70790643)	-	-	#N/D
CLEC4F	2	complement(70808643..70820599)	0,02	0,01	2,144183439
CD207	2	complement(70830211..70835816)	0,05	0,02	1,339142659
VAX2	2	70900576..70965373	0,61	0,84	-0,248126412
ATP6V1B1	2	70935900..70965431	0,60	0,43	0,682057798
gene:ENSG000	2	complement(70939318..70995336)	0,47	0,92	-0,732212606
TEX261	2	complement(70968325..70994873)	26,17	27,02	0,176959428
ANKRD53	2	70978380..70985499	0,16	0,05	1,774926715
NAGK	2	71064344..71079808	8,49	4,80	1,043706441
MCEE	2	complement(71109684..71130239)	6,67	3,82	1,024984556
MPHOSPH10	2	71130310..71150101	2,68	2,67	0,227324217
PAIP2B	2	complement(71182738..71227103)	0,25	0,07	2,080372167
ZNF638	2	71276561..71435069	3,69	5,80	-0,430788891
DYSF	2	71453561..71686763	0,20	0,34	-0,52156326
CYP26B1	2	complement(72129238..72147862)	7,93	14,32	-0,628920647
EXOC6B	2	complement(72175984..72826041)	4,86	5,37	0,079458473
SPR	2	72887382..72892158	6,47	12,04	-0,672768809
EMX1	2	72916260..72936071	0,01	0,01	-0,025382928
SFXN5	2	complement(72942036..73075619)	0,81	1,16	-0,3023046
RAB11FIP5	2	complement(73073382..73156721)	23,38	17,98	0,601705172
NOTO	2	73202574..73212513	0,03	0,03	-0,03547153
SMYD5	2	73214222..73227221	19,71	21,97	0,066821524
PRADC1	2	complement(73228010..73233239)	12,44	6,63	1,131745545
CCT7	2	73233420..73253021	107,48	#####	-0,336720691
FBXO41	2	complement(73254682..73284478)	1,76	2,12	-0,049204095
EGR4	2	complement(73290929..73293701)	-	-	0,188027316
ALMS1	2	73385758..73625166	3,84	4,08	0,134372353
NAT8	2	complement(73640723..73642422)	0,01	-	3,068695355
NAT8B	2	complement(73700576..73701340)	-	0,02	-2,89252005
TPRKB	2	complement(73729104..73737400)	1,93	2,48	-0,133690344
gene:ENSG000	2	complement(73761269..73761781)	2,02	0,86	1,45329859
DUSP11	2	complement(73761782..73780132)	1,94	1,85	0,291950687
C2orf78	2	73784183..73817148	0,02	0,01	1,232079601

STAMBP	2	73828916..73873659	4,32	5,03	0,004572638
ACTG2	2	73892314..73919865	0,01	0,03	-1,21700111
DGUOK	2	73926826..73958961	34,39	43,57	-0,118228364
TET3	2	73984910..74108176	4,03	6,03	-0,359102753
gene:ENSG000	2	complement(73998337..73998548)	0,05	-	3,068695355
BOLA3	2	complement(74135400..74147912)	17,99	28,67	-0,449196372
MOB1A	2	complement(74152528..74178898)	15,16	21,83	-0,302685019
MTHFD2	2	74186172..74217565	14,24	34,98	-1,07351133
gene:ENSG000	2	74191388..74192245	-	-	#N/D
gene:ENSG000	2	complement(74211604..74363377)	0,01	0,01	-0,034419864
SLC4A5	2	complement(74216242..74343416)	0,15	0,14	0,340291281
DCTN1	2	complement(74361154..74392087)	23,61	27,16	0,020758678
gene:ENSG000	2	complement(74393836..74421662)	0,03	0,01	1,781479726
C2orf81	2	complement(74414176..74421619)	0,73	1,15	-0,427108277
WDR54	2	74421678..74425755	15,15	20,83	-0,236284572
RTKN	2	complement(74425835..74442422)	3,45	5,42	-0,428980411
INO80B	2	74455087..74457944	6,30	5,78	0,347830304
INO80B-WBP1	2	74455088..74460884	4,95	3,28	0,815329394
WBP1	2	74458400..74460891	18,12	13,13	0,688103114
MOGS	2	complement(74461057..74465410)	38,18	39,79	0,163329075
MRPL53	2	complement(74471982..74472687)	20,88	24,45	-0,004859568
CCDC142	2	complement(74471986..74483408)	2,09	2,38	0,036189213
TTC31	2	74483073..74494559	6,09	7,87	-0,147775942
LBX2	2	complement(74497517..74503316)	1,26	1,56	-0,082919068
PCGF1	2	complement(74505043..74507695)	14,49	15,92	0,087783947
TLX2	2	74513463..74517148	0,04	0,02	0,7939095
DQX1	2	complement(74518131..74526281)	0,15	0,07	1,353579474
AUP1	2	complement(74526645..74529760)	52,69	54,28	0,180265282
HTRA2	2	74529596..74533350	9,43	10,54	0,062817873
LOXL3	2	complement(74532258..74555690)	0,50	0,72	-0,311858036
DOK1	2	74549026..74557551	3,46	5,74	-0,505515346
M1AP	2	complement(74557883..74648338)	0,18	0,14	0,526008598
SEMA4F	2	74654228..74683853	3,52	4,14	-0,011475983
HK2	2	74834127..74893359	16,31	9,99	0,931072601
POLE4	2	74958643..74970128	16,46	14,21	0,43541744
TACR1	2	complement(75046463..75199520)	0,16	0,10	0,995352755
EVA1A	2	complement(75469302..75569722)	13,65	7,47	1,093038023
MRPL19	2	75646783..75690851	5,34	6,84	-0,132714084
GCFC2	2	complement(75652000..75710985)	1,14	2,10	-0,660468237
LRRTM4	2	complement(76747685..77593319)	0,00	-	3,967268276
REG3G	2	79025686..79028505	-	-	#N/D
REG1B	2	complement(79085023..79088019)	-	-	#N/D
REG1A	2	79120362..79123409	-	-	#N/D
REG3A	2	complement(79157003..79159753)	-	-	#N/D
CTNNA2	2	79185231..80648861	0,00	0,02	-1,766505595
LRRTM1	2	complement(80288351..80304752)	0,00	-	3,068695355
SUCLG1	2	complement(84423528..84460045)	32,95	39,03	-0,021445102
DNAH6	2	84516455..84819589	0,01	0,00	2,616997665

TRABD2A	2	complement(84821650..84907008)	2,00	9,95	-2,092099765
TMSB10	2	84905656..84906671	#######	#####	0,75597131
KCMF1	2	84971093..85059472	8,07	8,49	0,149381051
TCF7L1	2	85133392..85310387	1,83	1,27	0,743617806
TGOLN2	2	complement(85318027..85328251)	49,19	52,13	0,139167114
RETSAT	2	complement(85341955..85354531)	10,25	9,05	0,40278046
ELMOD3	2	85354394..85391752	3,01	3,29	0,09385509
CAPG	2	complement(85394753..85418432)	67,72	93,59	-0,243633241
SH2D6	2	85418714..85437029	0,16	0,30	-0,680831957
MAT2A	2	85539168..85545281	117,00	75,81	0,849208303
GGCX	2	complement(85544720..85561532)	8,40	5,13	0,933002526
VAMP8	2	85561562..85582031	0,39	0,52	-0,209104491
VAMP5	2	85584431..85593406	11,60	17,15	-0,340599269
RNF181	2	85595725..85597708	22,34	20,73	0,330705934
TMEM150A	2	complement(85598547..85603196)	1,28	1,88	-0,325312456
USP39	2	85602856..85649283	21,34	28,85	-0,212317174
C2orf68	2	complement(85605254..85612066)	5,75	7,25	-0,109906608
SFTPB	2	complement(85657314..85668741)	0,11	0,98	-2,887919746
GNLY	2	85685175..85698852	0,01	0,05	-1,877914477
ATOH8	2	85751344..85791383	2,27	5,26	-0,990237184
ST3GAL5	2	complement(85837120..85905199)	0,67	0,57	0,45074404
POLR1A	2	complement(86020216..86106155)	5,57	8,63	-0,407743045
PTCD3	2	86106223..86142157	3,54	6,64	-0,682481493
IMMT	2	complement(86143932..86195472)	34,06	44,24	-0,154229993
MRPL35	2	86199355..86213790	4,31	6,08	-0,273844341
REEP1	2	complement(86213993..86338083)	0,05	0,05	0,21051339
KDM3A	2	86440647..86492716	3,95	4,02	0,196323703
CHMP3	2	complement(86503430..86563479)	13,19	15,53	-0,01236566
RNF103-CHMP	2	complement(86505668..86721122)	0,37	0,94	-1,133977666
RNF103	2	complement(86603398..86623866)	2,73	4,05	-0,345268078
RMND5A	2	86720291..86778041	5,61	7,72	-0,239275928
CD8A	2	complement(86784610..86808396)	-	-	0,188027316
CD8B	2	complement(86815339..86861924)	-	-	0,188027316
RGPD1	2	86913783..87013976	0,01	0,00	0,921097799
PLGLB1	2	complement(87002559..87021852)	0,05	0,06	-0,038943307
IGKV3OR2-268	2	87338511..87339035	-	-	#N/D
PLGLB2	2	87748087..87759476	0,08	0,12	-0,321745402
RGPD2	2	complement(87755960..87825796)	0,01	0,07	-2,767459854
KRCC1	2	complement(88027205..88064252)	0,39	1,24	-1,45627855
SMYD1	2	88067825..88113384	-	-	0,188027316
FABP1	2	complement(88122982..88128062)	-	-	#N/D
THNSL2	2	88170295..88186636	1,09	0,70	0,872136265
FOXI3	2	complement(88446787..88452693)	0,00	0,00	-0,01185201
SPMIP9	2	88524649..88529585	-	-	#N/D
EIF2AK3	2	complement(88556741..88691518)	4,83	2,90	0,957600094
RPIA	2	88691673..88750929	5,51	9,04	-0,491984262
IGKC	2	complement(88857161..88857683)	-	-	0,188027316
IGKJ5	2	complement(88860568..88860605)	-	-	#N/D

IGKJ4	2	complement(88860886..88860922)	-	-	#N/D
IGKJ3	2	complement(88861221..88861258)	-	-	#N/D
IGKJ2	2	complement(88861525..88861563)	-	-	#N/D
IGKJ1	2	complement(88861886..88861923)	-	-	#N/D
IGKV4-1	2	88885397..88886153	-	-	#N/D
IGKV5-2	2	88897232..88897784	-	-	#N/D
IGKV1-5	2	complement(88947301..88947957)	-	-	#N/D
IGKV1-6	2	complement(88966262..88966767)	-	-	#N/D
IGKV3-7	2	complement(88978468..88979081)	-	-	#N/D
IGKV1-8	2	complement(88992409..88992931)	-	-	#N/D
IGKV1-9	2	complement(89009982..89010515)	-	-	#N/D
IGKV3-11	2	complement(89027171..89027731)	-	-	#N/D
IGKV1-12	2	complement(89040224..89040745)	-	-	#N/D
IGKV3-15	2	complement(89085177..89085787)	-	-	#N/D
IGKV1-16	2	complement(89099859..89100361)	-	-	#N/D
IGKV1-17	2	complement(89117342..89117844)	-	-	#N/D
IGKV3-20	2	complement(89142574..89143160)	-	-	#N/D
IGKV6-21	2	complement(89159751..89160366)	-	-	#N/D
IGKV2-24	2	complement(89176328..89177160)	-	-	#N/D
IGKV1-27	2	complement(89213423..89213928)	-	-	#N/D
IGKV2-28	2	complement(89221698..89222461)	-	-	#N/D
IGKV2-30	2	complement(89244781..89245596)	0,01	-	3,068695355
IGKV1-33	2	complement(89268001..89268506)	-	-	#N/D
IGKV1-37	2	complement(89297264..89297785)	-	-	#N/D
IGKV1-39	2	complement(89319625..89320146)	-	-	#N/D
IGKV2-40	2	complement(89330116..89330429)	-	-	#N/D
IGKV2D-40	2	89851758..89852493	-	-	#N/D
IGKV1D-39	2	89862482..89862981	-	-	0,188027316
IGKV1D-37	2	89884740..89885216	-	-	#N/D
IGKV1D-33	2	89913982..89914545	-	-	#N/D
IGKV2D-30	2	89936859..89937679	-	-	0,188027316
IGKV2D-29	2	89947512..89948269	-	-	#N/D
IGKV2D-28	2	89959979..89960748	-	-	#N/D
IGKV2D-26	2	89985922..89986702	-	-	#N/D
IGKV2D-24	2	90004797..90005629	-	-	#N/D
IGKV6D-21	2	90021567..90022185	-	-	#N/D
IGKV3D-20	2	90038848..90039479	-	-	#N/D
IGKV6D-41	2	90069662..90070238	-	-	#N/D
IGKV1D-17	2	90082635..90083291	-	-	#N/D
IGKV1D-16	2	90100236..90100738	-	-	#N/D
IGKV3D-15	2	90114838..90115402	-	-	#N/D
IGKV1D-13	2	90154073..90154574	-	-	#N/D
IGKV1D-12	2	90159840..90160335	-	-	#N/D
IGKV3D-11	2	90172802..90173414	-	-	#N/D
IGKV1D-42	2	90190193..90190681	-	-	#N/D
IGKV1D-43	2	90209873..90210529	-	-	#N/D
IGKV1D-8	2	90220727..90221384	-	-	0,188027316
IGKV3D-7	2	90234812..90235370	-	-	#N/D

AQP7B	2	94587101..94612744	0,00	0,00	-0,01185201
TEKT4	2	94871430..94876823	0,35	0,20	1,04649872
MAL	2	95025677..95053992	0,07	-	5,697545031
MRPS5	2	complement(95085369..95122006)	7,94	8,80	0,074411964
gene:ENSG000	2	complement(95085391..95165391)	-	0,14	-8,779996217
ZNF514	2	complement(95122087..95165668)	1,52	1,68	0,081871519
ZNF2	2	95165432..95184317	1,05	1,28	-0,059169963
ZNF892	2	95207521..95259774	0,08	0,22	-1,269575343
PROM2	2	95274449..95291308	0,21	0,11	1,15385364
KCNIP3	2	95297327..95386077	1,44	1,90	-0,173945682
FAHD2A	2	95402708..95416616	5,41	5,61	0,169627905
TRIM43B	2	complement(95477008..95484754)	-	-	0,188027316
TRIM43	2	95592001..95599778	0,01	-	3,068695355
ANKRD36C	2	complement(95836919..95991831)	1,22	1,73	-0,2821963
GPAT2	2	complement(96021946..96039451)	0,02	0,01	1,487770062
ADRA2B	2	complement(96112876..96116571)	0,03	0,00	3,257511884
ASTL	2	complement(96122818..96138502)	0,14	0,11	0,589040426
DUSP2	2	complement(96143169..96145440)	1,04	1,98	-0,702741592
STARD7	2	complement(96184859..96208827)	19,85	32,76	-0,49987897
TMEM127	2	complement(96248514..96266047)	7,43	6,99	0,310971497
CIAO1	2	96266159..96274173	12,55	16,48	-0,169575018
SNRNP200	2	complement(96274338..96321271)	50,71	58,80	0,009385446
ITPRIPL1	2	96325317..96330517	2,05	2,94	-0,299762705
NCAPH	2	96335766..96377091	6,88	9,40	-0,227292836
NEURL3	2	complement(96497646..96508109)	0,04	0,09	-0,833928617
ARID5A	2	96536743..96552634	2,91	2,57	0,399163993
KANSL3	2	complement(96593170..96642787)	13,94	18,21	-0,162136384
FER1L5	2	96642737..96704887	0,04	0,02	1,087674602
LMAN2L	2	complement(96705929..96740064)	8,59	12,52	-0,320490624
CNNM4	2	96760902..96811874	4,88	3,96	0,524846693
CNNM3	2	96816245..96835382	6,01	5,05	0,475024639
ANKRD23	2	complement(96824526..96857934)	0,88	0,85	0,277578098
ANKRD39	2	complement(96836611..96858016)	1,70	1,93	0,041811156
SEMA4C	2	complement(96859718..96870757)	5,57	7,75	-0,252781843
FAM178B	2	complement(96875882..96986580)	-	0,00	-2,89252005
FAHD2B	2	complement(97083583..97094882)	2,28	5,28	-0,987116793
gene:ENSG000	2	97094935..97097433	0,01	0,03	-1,291882393
ANKRD36	2	97113153..97264521	1,37	2,09	-0,382676282
ANKRD36B	2	complement(97492663..97589877)	1,18	1,36	0,009937674
COX5B	2	97646062..97648383	55,87	80,74	-0,308264094
ACTR1B	2	complement(97655939..97664044)	23,95	21,12	0,40402541
C2orf92	2	97664217..97703066	0,06	0,05	0,415033091
ZAP70	2	97713576..97739862	-	-	0,188027316
TMEM131	2	complement(97756333..97995948)	4,54	6,63	-0,321321017
VWA3B	2	98087116..98313299	-	0,02	-6,187931073
CNGA3	2	98346188..98398601	-	0,00	-2,89252005
INPP4A	2	98444854..98594392	3,18	4,13	-0,154095645
COA5	2	complement(98599314..98608515)	2,82	3,95	-0,263935387

UNC50	2	98608579..98618515	7,91	9,79	-0,084981231
MGAT4A	2	complement(98619106..98731132)	0,17	0,05	2,134557132
CRACDL	2	complement(98793846..98936259)	-	-	0,188027316
TSGA10	2	complement(98997261..99154964)	0,04	0,06	-0,259918184
gene:ENSG000	2	99141485..99322741	0,20	0,33	-0,473035668
C2orf15	2	99141707..99151669	0,03	0,08	-1,015460095
LIPT1	2	99154955..99163157	0,38	0,71	-0,673712886
gene:ENSG000	2	99154998..99195298	0,02	0,14	-2,665078517
MITD1	2	complement(99161427..99181058)	2,08	2,42	0,005235028
MRPL30	2	99181152..99199561	5,68	8,05	-0,280316138
LYG2	2	complement(99242246..99255666)	-	0,01	-2,89252005
LYG1	2	complement(99284238..99304742)	0,21	0,66	-1,407464189
TXNDC9	2	complement(99318982..99340702)	3,37	3,39	0,214765885
EIF5B	2	99337389..99401326	6,58	8,14	-0,084679238
REV1	2	complement(99400475..99490035)	4,84	5,89	-0,060659939
AFF3	2	complement(99545419..100192428	0,48	1,35	-1,268436134
LONRF2	2	complement(100271875..10032250	0,12	0,65	-2,268446847
CHST10	2	complement(100391860..10041766	1,06	2,56	-1,05171374
NMS	2	100470482..100483280	-	-	#N/D
PDCL3	2	100562993..100576739	4,65	8,18	-0,591096643
NPAS2	2	100820139..100996829	2,09	12,31	-2,333615654
RPL31	2	101002229..101024032	21,85	27,77	-0,122966139
TBC1D8	2	complement(101007228..10125286	22,17	17,92	0,530324117
CNOT11	2	101252886..101270316	8,86	14,98	-0,535234398
RNF149	2	complement(101271219..10130870	3,11	4,67	-0,361118771
CREG2	2	complement(101345550..10138750	0,01	0,10	-3,60395309
RFX8	2	complement(101397359..10147511	0,08	0,07	0,397821164
MAP4K4	2	101696850..101894690	14,25	33,00	-0,988591073
IL1R2	2	101991960..102028544	0,02	0,01	1,436225396
IL1R1	2	102064544..102179874	2,19	2,17	0,24177978
IL1RL2	2	102187006..102240002	-	-	0,188027316
IL1RL1	2	102311502..102352037	-	-	0,188027316
IL18R1	2	102311529..102398777	0,04	0,01	1,735012788
IL18RAP	2	102418689..102452565	-	-	0,188027316
SLC9A4	2	102473226..102533972	-	-	0,188027316
SLC9A2	2	102619553..102711355	0,02	-	6,053060169
MFSD9	2	complement(102714630..10273688	0,54	0,86	-0,428231892
TMEM182	2	102736905..103019900	0,29	0,39	-0,187687902
gene:ENSG000	2	104706612..104707506	-	-	#N/D
POU3F3	2	104853287..104858574	0,03	0,30	-3,026508668
MRPS9	2	105038069..105099960	5,86	10,16	-0,569011365
GPR45	2	105241743..105243467	-	-	0,188027316
TGFBRAP1	2	complement(105264391..10532973	2,95	6,09	-0,822874701
C2orf49	2	105337532..105349211	2,64	2,56	0,267654702
FHL2	2	complement(105357712..10543851	17,04	18,55	0,100291815
NCK2	2	105744912..105894274	5,94	8,95	-0,368743553
ECRG4	2	106063246..106078155	-	-	#N/D
UXS1	2	complement(106093308..10619430	5,90	7,25	-0,073950315

RGPD3	2	complement(106391290..10646841	0,03	0,06	-0,852483336
CD8B2	2	106487364..106544297	0,00	-	3,967268276
ST6GAL2	2	complement(106801600..10688710	0,12	0,00	6,120265843
RGPD4	2	107826892..107892544	0,22	0,22	0,210154486
SLC5A7	2	107986523..108013994	-	-	0,188027316
SULT1C3	2	108239968..108265351	0,01	0,02	-0,923955849
SULT1C2	2	108288639..108309915	-	0,12	-7,525269081
SULT1C4	2	108377911..108388989	0,01	0,00	1,436225396
GCC2	2	108449107..108509415	2,17	2,32	0,130868932
LIMS1	2	108533671..108687246	5,81	8,45	-0,317476896
RANBP2	2	108719482..108785810	5,80	8,75	-0,368926569
CCDC138	2	108786757..108885477	0,80	1,07	-0,199745438
EDAR	2	complement(108894471..10898937	0,03	0,04	-0,038531947
SH3RF3	2	109129205..109504634	2,66	6,31	-1,022996178
SEPTIN10	2	complement(109542799..10961414	8,31	10,28	-0,083603625
SOWAHC	2	109614364..109618990	2,98	3,29	0,082612581
RGPD5	2	109792758..109857705	1,54	4,48	-1,313058192
LIMS3	2	109898428..109925625	0,31	0,20	0,82168119
gene:ENSG000	2	109898432..109968557	0,01	0,00	2,399873901
MALL	2	complement(110083870..11011602	0,44	0,11	2,234504644
NPHP1	2	complement(110122311..11020506	0,37	0,45	-0,073903958
MTLN	2	complement(110211529..11024542	4,10	6,48	-0,437670848
gene:ENSG000	2	complement(110402934..11047307	-	0,00	-2,89252005
LIMS4	2	complement(110445883..11047307	0,01	0,35	-4,793942311
RGPD6	2	complement(110513802..11061084	3,00	1,85	0,923203836
BUB1	2	complement(110637528..11067806	19,02	26,76	-0,269900477
ACOXL	2	110732539..111118548	0,01	0,01	0,221270597
BCL2L11	2	111119378..111168444	0,49	0,66	-0,204536885
gene:ENSG000	2	111121153..111123765	-	-	#N/D
ANAPC1	2	complement(111611639..11188469	8,65	13,57	-0,425652557
MERTK	2	111898607..112029561	0,47	0,41	0,397027826
TMEM87B	2	112055269..112119318	3,02	3,32	0,086702133
FBLN7	2	112138385..112188218	0,01	0,04	-1,955757882
gene:ENSG000	2	112188364..112190635	-	-	0,188027316
ZC3H8	2	complement(112211529..11225513	0,43	0,84	-0,721548116
ZC3H6	2	112275597..112340059	1,05	1,18	0,055570313
RGPD8	2	complement(112368369..11243448	1,21	2,53	-0,845358462
TTL	2	112482156..112541739	3,01	5,79	-0,719425653
POLR1B	2	112541915..112579818	4,69	6,22	-0,182494664
CHCHD5	2	112584240..112589275	2,83	3,68	-0,159100057
SLC20A1	2	112645939..112663825	44,15	35,50	0,537677953
NT5DC4	2	112721020..112742879	0,57	0,38	0,821660554
CKAP2L	2	complement(112736349..11276466	9,43	16,45	-0,578943111
IL1A	2	complement(112773925..11278449	0,82	38,26	-5,323385508
IL1B	2	complement(112829751..11283681	35,18	#####	-2,23517388
gene:ENSG000	2	112840328..112844195	0,17	0,11	0,84332233
IL37	2	112911165..112918882	0,13	0,04	1,790975958
IL36G	2	112973203..112985658	0,06	0,06	0,370333154

IL36A	2	113005459..113008044	-	-	0,188027316
IL36B	2	complement(113022089..11305286	-	-	#N/D
IL36RN	2	113058638..113065382	-	-	0,188027316
IL1F10	2	113067970..113075843	-	-	#N/D
IL1RN	2	113099315..113134016	0,07	0,08	0,047814213
PSD4	2	113157325..113209396	0,32	0,32	0,212051509
PAX8	2	complement(113215997..11327892	1,33	1,88	-0,275230079
IGKV1OR2-108	2	113406396..113406872	0,11	0,22	-0,698295353
ZNG1B	2	113437691..113496204	2,17	2,80	-0,144462105
FOXD4L1	2	113498665..113501150	0,03	0,04	0,152452079
gene:ENSG000	2	complement(113605867..11360790	0,23	0,17	0,628610431
RABL2A	2	113627229..113643396	0,97	0,38	1,569599485
SLC35F5	2	complement(113705011..11375669	4,17	4,19	0,215924563
ACTR3	2	113890063..113962596	14,61	20,32	-0,252394775
gene:ENSG000	2	113904086..113905258	0,02	0,03	-0,579815015
DPP10	2	114442299..115845780	-	-	#N/D
DDX18	2	117814691..117832377	16,55	18,08	0,095346201
CCDC93	2	complement(117915478..11801413	3,82	5,18	-0,216815756
gene:ENSG000	2	118014174..118015679	0,42	0,79	-0,704661923
INSIG2	2	118088452..118110997	3,42	2,82	0,499637326
EN1	2	complement(118842171..11884764	8,42	5,25	0,903803589
MARCO	2	118942194..118994660	-	-	0,188027316
C1QL2	2	complement(119156243..11915875	0,01	-	3,967268276
STEAP3	2	119223831..119265652	32,55	19,03	0,996998492
C2orf76	2	complement(119302225..11936683	1,70	1,62	0,291609679
DBI	2	119366924..119372550	5,66	4,84	0,446850044
TMEM37	2	119429901..119438504	0,18	0,15	0,483752748
SCTR	2	complement(119439843..11952530	0,00	-	3,068695355
CFAP221	2	119544432..119662251	-	-	0,188027316
TMEM177	2	119679167..119686507	2,70	6,69	-1,084466017
PTPN4	2	119759922..119984899	0,62	1,25	-0,792557422
EPB41L5	2	120013077..120179119	1,45	2,23	-0,392608337
TMEM185B	2	complement(120217479..12022340	7,49	9,37	-0,101015418
RALB	2	120240064..120294710	15,59	12,60	0,530547648
INHBB	2	120346136..120351803	0,05	0,05	0,181581643
LINC01101	2	complement(120464335..12046621	-	-	0,188027316
GLI2	2	120735623..120992653	1,94	4,12	-0,866792932
TFCP2L1	2	complement(121216587..12128520	0,61	0,77	-0,097074194
CLASP1	2	complement(121337776..12164947	11,06	13,11	-0,022732134
gene:ENSG000	2	121688203..121689714	0,07	0,04	0,943435191
NIFK	2	complement(121726945..12173691	6,26	8,40	-0,200780607
TSN	2	121737103..121767853	9,50	11,66	-0,071569713
gene:ENSG000	2	complement(123850522..12385069	-	-	#N/D
CNTNAP5	2	124025287..124921219	-	-	#N/D
GYPC	2	126656133..126696667	10,15	10,47	0,178592842
TEX51	2	126898864..126902097	-	-	#N/D
BIN1	2	complement(127048027..12710728	15,12	26,67	-0,595200605
CYP27C1	2	complement(127183832..12722031	0,83	1,18	-0,282565423

ERCC3	2	complement(127257290..12729416	10,07	12,04	-0,034934591
MAP3K2	2	complement(127298668..12738846	3,78	3,83	0,204189545
PROC	2	127418427..127429242	0,20	0,18	0,336996335
IWS1	2	complement(127436207..12752688	7,87	9,81	-0,096264704
MYO7B	2	127535683..127637729	0,98	0,19	2,601463421
LIMS2	2	complement(127638381..12768178	0,08	0,15	-0,636703361
GPR17	2	127645864..127652639	0,00	0,01	-0,923955849
WDR33	2	complement(127701027..12781118	2,95	5,86	-0,770190472
SFT2D3	2	127701497..127705242	-	-	0,188027316
POLR2D	2	complement(127843553..12785815	6,69	10,21	-0,386809976
AMMECR1L	2	complement(127861630..12788596	6,84	7,17	0,155489477
SAP130	2	complement(127941217..12802812	11,58	11,27	0,261585113
UGGT1	2	128091200..128195677	8,94	12,35	-0,242530879
HS6ST1	2	complement(128236716..12831886	12,98	8,26	0,874484836
RAB6C	2	129979666..129982738	-	-	0,188027316
POTEF	2	complement(130073535..13012922	1,63	1,23	0,623324903
CCDC74B	2	complement(130139287..13014513	0,72	0,63	0,398179933
SMPD4	2	complement(130151392..13018275	28,39	24,54	0,433308733
MZT2B	2	130181737..130190729	57,94	65,33	0,049840906
TUBA3E	2	complement(130191745..13019843	0,10	0,11	0,067331041
CCDC115	2	complement(130337933..13034269	3,48	3,64	0,155855272
IMP4	2	130342877..130347967	25,07	25,41	0,203670202
PTPN18	2	130356045..130375405	6,20	5,38	0,42853049
POTEI	2	complement(130459455..13050970	0,60	0,31	1,185938863
CFC1B	2	130521197..130528604	-	-	#N/D
CFC1	2	complement(130592165..13059957	-	-	#N/D
POTEJ	2	130611440..130658037	0,03	0,11	-1,660236228
GPR148	2	130729070..130730336	-	0,02	-3,804623889
AMER3	2	130755540..130768134	-	-	0,188027316
ARHGEF4	2	130836914..131047263	1,59	5,04	-1,436708726
SMIM39	2	131034928..131047253	0,68	0,44	0,869744788
FAM168B	2	complement(131047876..13109346	28,98	29,73	0,186339716
PLEKHB2	2	131104847..131353709	26,19	23,74	0,36471998
POTEE	2	131209536..131265278	1,12	0,59	1,140434864
RAB6D	2	complement(131360492..13136416	-	-	0,188027316
MZT2A	2	complement(131464900..13149274	27,69	31,42	0,040596718
TUBA3D	2	131476119..131482934	0,28	0,51	-0,659790972
CCDC74A	2	131527675..131533666	0,61	0,70	0,027472172
ANKRD30BL	2	complement(132147591..13225796	-	-	#N/D
GPR39	2	132416805..132646582	0,93	3,98	-1,868567779
LYPD1	2	complement(132643286..13267157	0,34	0,28	0,526530246
NCKAP5	2	complement(132671788..13356846	0,13	0,30	-0,950020521
gene:ENSG000	2	133117004..133119052	0,01	-	3,068695355
MGAT5	2	134119983..134454621	15,86	16,10	0,201667548
TMEM163	2	complement(134455759..13471900	0,03	0,06	-0,609885528
ACMSD	2	134838616..134902034	0,02	-	4,516772761
CCNT2	2	134918235..134959342	2,12	2,50	-0,015622736
MAP3K19	2	complement(134964485..13504746	0,01	0,01	1,15046174

RAB3GAP1	2	135052289..135176667	6,51	9,21	-0,276894998
ZRANB3	2	complement(135136916..13553121	0,94	1,61	-0,550048676
R3HDM1	2	135531455..135725270	5,11	8,07	-0,436733656
UBXN4	2	135741734..135785056	11,93	16,37	-0,233055261
LCT	2	complement(135787850..13583718	-	0,02	-5,902279478
MCM6	2	complement(135839626..13587644	14,82	23,20	-0,423869979
DARS1	2	complement(135905881..13598610	15,01	15,14	0,210772041
CXCR4	2	complement(136114349..13611917	0,07	0,01	2,493547104
gene:ENSG000	2	136230753..136233245	-	-	#N/D
THSD7B	2	136765545..137677718	-	-	0,188027316
HNMT	2	137964020..138016364	0,01	0,02	-1,406253396
SPOPL	2	138501770..138573547	1,50	1,94	-0,14629979
NXPH2	2	complement(138669157..13878039	0,00	0,07	-3,750082345
LRP1B	2	complement(140231423..14213101	0,01	0,08	-2,701105812
KYNU	2	142877657..143055833	0,75	2,04	-1,215384971
ARHGAP15	2	143091362..143768352	-	-	0,188027316
GTDC1	2	complement(143938068..14433256	0,43	0,61	-0,281260679
ZEB2	2	complement(144364364..14452105	0,43	2,07	-2,055357423
gene:ENSG000	2	complement(144494265..14449687	0,02	0,13	-2,180662299
gene:ENSG000	2	complement(145874520..14587484	-	-	#N/D
ACVR2A	2	147844517..147930826	0,43	0,89	-0,838035806
ORC4	2	complement(147930396..14802160	0,76	1,59	-0,83317533
MBD5	2	148021011..148516971	2,30	2,00	0,424353137
gene:ENSG000	2	148044380..148044894	-	-	0,188027316
gene:ENSG000	2	148062154..148062577	0,03	-	3,068695355
EPC2	2	148644440..148787569	0,88	1,79	-0,795403604
KIF5C	2	148875227..149026759	0,31	0,55	-0,629661068
LYPD6B	2	149038107..149215262	0,02	-	4,913749003
LYPD6	2	149329985..149474138	0,16	0,62	-1,757372353
MMADHC	2	complement(149569637..14958777	29,08	30,00	0,178495863
RND3	2	complement(150468195..15053901	25,60	45,85	-0,617786979
RBM43	2	complement(151247940..15126186	0,62	0,69	0,064864076
NMI	2	complement(151270470..15128989	0,39	1,63	-1,848824238
TNFAIP6	2	151357592..151380046	1,52	5,89	-1,726624819
RIF1	2	151409883..151508013	4,23	6,02	-0,285621079
NEB	2	complement(151485336..15173448	0,00	0,01	-1,459576629
ARL5A	2	complement(151788984..15182849	1,94	2,31	-0,026043117
gene:ENSG000	2	complement(151802651..15197394	0,01	-	3,967268276
CACNB4	2	complement(151832771..15209916	0,00	0,00	0,677647514
STAM2	2	complement(152116801..15217576	3,70	3,63	0,249301805
FMNL2	2	152335174..152649826	7,12	13,49	-0,699074627
PRPF40A	2	complement(152651593..15271801	4,12	6,67	-0,470635569
ARL6IP6	2	152717647..152762396	3,56	5,13	-0,303354088
RPRM	2	complement(153477338..15347876	-	-	#N/D
GALNT13	2	153871922..154453979	0,20	-	9,705627292
KCNJ3	2	154697855..154858354	0,00	-	3,068695355
NR4A2	2	complement(156324437..15634234	0,83	0,33	1,542968708
GPD2	2	156435290..156613735	3,04	4,63	-0,384172775

GALNT5	2	157257705..157318491	1,85	1,47	0,554255676
ERMN	2	complement(157318631..15732771	-	-	0,188027316
CYTIP	2	complement(157414619..15748896	-	-	0,188027316
ACVR1C	2	complement(157526767..15762886	0,02	0,01	1,602082935
ACVR1	2	complement(157736251..15787633	5,09	4,67	0,345830835
UPP2	2	157876679..158136154	-	-	0,188027316
CCDC148	2	complement(158171073..15845675	0,01	0,02	-0,509043067
PKP4	2	158456952..158682879	4,23	7,43	-0,588176768
DAPL1	2	158795317..158862781	0,11	0,13	0,067331041
TANC1	2	158968640..159232659	9,76	13,77	-0,27367018
WDSUB1	2	complement(159235798..15928670	1,54	3,51	-0,969067262
BAZ2B	2	complement(159318979..15961656	1,41	2,13	-0,371588094
MARCHF7	2	159712457..159771027	7,52	10,27	-0,226852667
CD302	2	complement(159768628..15979825	1,27	1,56	-0,077327459
LY75-CD302	2	complement(159771851..15990471	-	-	0,188027316
LY75	2	complement(159803355..15990475	0,01	-	5,697545031
PLA2R1	2	complement(159932006..16006261	0,61	0,61	0,225457358
ITGB6	2	complement(160099667..16027188	0,00	0,02	-2,272922624
RBMS1	2	complement(160272151..16049380	11,39	11,83	0,168434176
gene:ENSG000	2	complement(160849313..16085067	-	-	#N/D
TANK	2	161136908..161236230	3,30	5,73	-0,575540295
PSMD14	2	161308425..161411717	25,07	44,28	-0,597281282
TBR1	2	161416297..161425870	0,01	0,03	-1,009277154
SLC4A10	2	161424332..161985282	0,00	0,01	-0,979341556
gene:ENSG000	2	161433874..161437458	-	-	0,188027316
DPP4	2	complement(161992245..16207439	0,08	0,13	-0,447879498
GCG	2	complement(162142882..16215240	-	-	0,188027316
FAP	2	complement(162170684..16224515	1,06	3,20	-1,368517098
IFIH1	2	complement(162267074..16231868	1,16	0,90	0,58783605
GCA	2	162318840..162371595	0,17	0,04	2,232316103
KCNH7	2	complement(162371407..16283876	-	-	#N/D
FIGN	2	complement(163593396..16373601	0,60	1,03	-0,548257242
GRB14	2	complement(164492417..16462148	0,24	1,04	-1,865273967
COBLL1	2	complement(164653624..16484367	1,84	0,73	1,563587284
SLC38A11	2	complement(164894354..16495552	-	0,00	-2,89252005
SCN3A	2	complement(165087526..16520405	1,88	0,26	3,094103692
SCN2A	2	165194993..165392310	0,01	0,03	-1,521413109
CSRNP3	2	165469647..165689407	0,21	0,43	-0,791567809
GALNT3	2	complement(165747588..16584620	1,40	1,12	0,53808671
TTC21B	2	complement(165857475..16595385	0,83	1,31	-0,435111413
SCN1A	2	complement(165984641..16618280	-	0,00	-2,89252005
SCN9A	2	complement(166195185..16637600	0,01	0,26	-3,959008136
SCN7A	2	complement(166403573..16661148	-	-	0,188027316
XIRP2	2	166888480..167259753	-	-	0,188027316
B3GALT1	2	167293001..167874045	0,02	0,27	-3,243572833
STK39	2	complement(167954020..16824759	4,63	8,84	-0,709221364
CERS6	2	168456249..168775134	4,41	6,13	-0,252108422
NOSTRIN	2	168786539..168865514	0,21	0,19	0,318344292

SPC25	2	complement(168834132..16891337	1,74	3,63	-0,833980976
G6PC2	2	168901291..168910000	-	-	#N/D
ABCB11	2	complement(168915498..16903132	0,00	0,01	-1,291882393
DHRS9	2	169064789..169096167	0,02	0,02	-0,03547153
LRP2	2	complement(169127109..16936253	0,00	-	3,068695355
BBS5	2	169479480..169506655	1,66	2,49	-0,359711303
gene:ENSG000	2	169479480..169525922	-	-	0,188027316
KLHL41	2	169509702..169526258	0,05	0,08	-0,356881532
FASTKD1	2	complement(169528508..16957387	1,37	2,25	-0,491899902
PPIG	2	169584342..169641406	1,96	2,40	-0,069207889
CFAP210	2	complement(169645425..16969440	0,03	0,05	-0,225481127
PHOSPHO2	2	169694454..169701708	0,10	0,24	-0,974683893
KLHL23	2	169694488..169776989	0,77	2,45	-1,451528766
SSB	2	169791933..169812064	2,91	6,25	-0,881434421
METTL5	2	complement(169810081..16982493	5,56	8,38	-0,370056702
UBR3	2	169827454..170084131	3,98	4,83	-0,056488356
MYO3B	2	170178145..170655171	-	0,01	-5,071123353
SP5	2	170715337..170718078	0,19	0,48	-1,115145538
ERICH2	2	170766878..170798977	0,19	0,32	-0,569683738
GAD1	2	170813213..170861151	0,06	0,11	-0,607153722
GORASP2	2	170928464..170967130	41,58	39,45	0,298767677
TLK1	2	complement(170990823..17123131	1,91	3,96	-0,829144216
METTL8	2	complement(171315746..17143480	0,99	1,98	-0,776521001
DCAF17	2	171434217..171485052	1,63	2,68	-0,493907591
CYBRD1	2	171522247..171558129	15,21	9,75	0,86440135
DYNC1I2	2	171687409..171750158	12,44	13,81	0,071868315
SLC25A12	2	complement(171783405..17199985	2,82	6,09	-0,885556102
HAT1	2	171922448..171983686	6,22	8,93	-0,298551472
METAP1D	2	171999943..172082430	1,70	3,49	-0,812507038
DLX1	2	172084740..172089677	2,81	3,80	-0,210730202
DLX2	2	complement(172099438..17210290	0,85	1,22	-0,293375392
ITGA6	2	172234216..172506459	31,66	20,62	0,841401862
gene:ENSG000	2	complement(172423807..17242417	-	-	0,188027316
PDK1	2	172555373..172608669	4,89	2,42	1,237460078
RAPGEF4	2	172735274..173052893	0,30	0,33	0,103594607
MAP3K20	2	173075435..173268015	14,16	13,47	0,295334522
CDCA7	2	173354820..173368997	4,53	9,41	-0,832622091
SP3	2	complement(173880850..17396537	4,98	5,16	0,171983643
OLA1	2	complement(174072447..17424859	10,11	13,07	-0,147607252
gene:ENSG000	2	complement(174334438..17433756	0,23	0,13	1,000910029
SP9	2	174334954..174338500	0,22	0,24	0,102321646
CIR1	2	complement(174348022..17439571	3,61	3,55	0,248401478
SCRN3	2	174395730..174429575	1,17	1,93	-0,504703521
GPR155	2	complement(174431571..17448709	0,78	0,29	1,649068961
gene:ENSG000	2	complement(174545385..17454633	-	-	0,188027316
WIPF1	2	complement(174559572..17468291	4,32	4,90	0,041362097
CHRNA1	2	complement(174747592..17478793	-	-	0,188027316
CHN1	2	complement(174798809..17500538	0,26	0,64	-1,044923531

ATF2	2	complement(175072250..17516838	8,35	8,39	0,216364377
ATP5MC3	2	complement(175176258..17518171	22,90	29,29	-0,131673597
LNPK	2	complement(175923882..17600283	3,36	3,06	0,354760035
EVX2	2	complement(176077472..17608396	0,02	0,00	2,399873901
HOXD13	2	176092721..176095944	0,41	0,40	0,253745487
HOXD12	2	176099795..176102489	0,09	0,04	1,338212201
HOXD11	2	176104216..176109754	7,04	0,36	4,520860516
HOXD10	2	176108790..176119937	7,93	0,44	4,406731348
HOXD9	2	176122719..176124937	3,83	2,83	0,658067968
HOXD8	2	176129694..176132695	1,86	2,11	0,04088011
HOXD3	2	176136612..176173102	0,12	0,09	0,644262894
HOXD4	2	176151550..176153226	0,30	0,82	-1,206425756
HOXD1	2	176188668..176190907	0,27	0,07	2,207653649
gene:ENSG000	2	complement(176189919..17619253	-	0,04	-5,902279478
MTX2	2	176269395..176338025	4,60	11,00	-1,033801091
gene:ENSG000	2	176822030..176823653	0,01	0,05	-1,54862452
HNRNPA3	2	177212694..177223959	19,27	27,64	-0,297765408
NFE2L2	2	complement(177218667..17739275	5,54	5,52	0,228465404
AGPS	2	177392746..177567024	1,21	3,23	-1,198348232
IFT70B	2	complement(177548998..17755279	0,44	0,90	-0,80295113
IFT70A	2	complement(177612999..17761874	0,38	0,49	-0,136418181
PDE11A	2	complement(177623244..17810833	0,03	0,01	2,777333658
RBM45	2	178112424..178139011	0,46	0,80	-0,572687201
OSBPL6	2	178194481..178402893	0,46	1,71	-1,681108651
PRKRA	2	complement(178431292..17845151	5,15	7,20	-0,259269247
PJVK	2	178451346..178462102	0,13	0,32	-1,070309526
FKBP7	2	complement(178463664..17847860	1,94	1,95	0,215099035
PLEKHA3	2	178480457..178516463	1,27	1,47	0,009541756
TTN	2	complement(178525989..17883080	0,08	0,14	-0,686976958
gene:ENSG000	2	178554561..178558966	0,07	0,09	-0,185877329
CCDC141	2	complement(178829757..17905013	0,00	0,00	0,221270597
SESTD1	2	complement(179101678..17926483	2,55	3,10	-0,057839253
ZNF385B	2	complement(179441982..17986161	0,01	0,12	-3,779192815
CWC22	2	complement(179944876..18000729	2,19	4,36	-0,768801999
gene:ENSG000	2	180801227..180801533	-	-	#N/D
UBE2E3	2	180967248..181076585	19,33	13,94	0,694674109
ITGA4	2	181457202..181538940	3,95	6,66	-0,531547755
CERKL	2	complement(181535041..18168066	0,01	-	5,224730805
NEUROD1	2	complement(181668295..18168082	-	-	0,188027316
ITPRID2	2	181891730..181930738	28,82	17,46	0,946695841
PPP1R1C	2	181954241..182131398	0,04	0,04	0,199980087
PDE1A	2	complement(182139968..18252335	0,01	0,00	1,833201638
DNAJC10	2	182716255..182794464	2,62	4,83	-0,659786359
FRZB	2	complement(182833275..18286663	0,04	0,08	-0,573028908
NCKAP1	2	complement(182909115..18303885	5,07	8,46	-0,515970566
DUSP19	2	183078559..183100008	0,30	0,45	-0,331663259
NUP35	2	183117513..183161680	2,08	4,42	-0,861305719
ZNF804A	2	184598529..184939492	0,79	1,25	-0,44038952

FSIP2	2	185738804..185833290	0,01	0,02	-0,372134404
ZC3H15	2	186486253..186509361	6,79	11,52	-0,54002414
ITGAV	2	186590010..186680901	15,90	18,23	0,026447733
FAM171B	2	186694060..186765959	1,83	3,14	-0,553437824
ZSWIM2	2	complement(186827475..18684920	-	0,00	-2,89252005
CALCRL	2	complement(187341964..18744846	0,01	0,02	-0,35396495
TFPI	2	complement(187464230..18756576	4,64	8,92	-0,719790797
GULP1	2	188291669..188595931	0,47	0,48	0,195245043
COL3A1	2	188974373..189012746	4,24	7,67	-0,632688412
COL5A2	2	complement(189031898..18922531	87,77	71,10	0,526812094
WDR75	2	189441446..189475552	4,19	7,45	-0,605551325
SLC40A1	2	complement(189560590..18958375	0,15	0,13	0,400954972
ASNSD1	2	189661385..189670831	2,97	5,32	-0,617960627
ASDURF	2	189661452..189666437	-	-	0,188027316
gene:ENSG000	2	189661519..189667881	-	-	0,188027316
ANKAR	2	189674290..189761193	0,16	0,19	-0,040434423
OSGEPL1	2	complement(189746660..18976322	1,45	2,93	-0,790375088
ORMDL1	2	complement(189770267..18978436	3,85	4,89	-0,121922898
PMS1	2	189784085..189877629	0,93	1,63	-0,585178143
C2orf88	2	189879609..190203484	0,13	0,20	-0,384353332
MSTN	2	complement(190055700..19006272	0,03	0,01	1,15046174
HIBCH	2	complement(190189735..19034419	1,70	2,23	-0,170989204
INPP1	2	190343570..190371665	3,50	5,65	-0,469168595
MFSD6	2	190408355..190509205	0,77	1,51	-0,746259648
NEMP2	2	complement(190504338..19053472	0,74	1,77	-1,027685406
NAB1	2	190646746..190692766	5,41	8,22	-0,381734713
GLS	2	190880821..190965552	9,35	8,41	0,376659305
STAT1	2	complement(190908460..19102096	16,43	14,34	0,41932359
STAT4	2	complement(191029576..19117843	0,14	0,24	-0,60923571
gene:ENSG000	2	complement(191154118..19115607	0,01	0,01	-0,025382928
MYO1B	2	191245185..191425389	14,42	19,68	-0,225659028
NABP1	2	191678068..191741097	2,44	3,34	-0,233018151
CAVIN2	2	complement(191834310..19184708	1,26	3,04	-1,046566942
TMEFF2	2	complement(191949043..19219493	0,00	0,11	-5,419341399
SLC39A10	2	195575977..195737702	5,32	4,97	0,319111119
DNAH7	2	complement(195737703..19606883	0,02	0,04	-1,003644204
STK17B	2	complement(196133583..19617650	4,20	5,09	-0,053989481
HECW2	2	complement(196189099..19659368	0,33	0,89	-1,201659954
CCDC150	2	196639554..196763490	0,56	0,86	-0,395468512
GTF3C3	2	complement(196763035..19679972	3,87	6,10	-0,434478098
C2orf66	2	complement(196804417..19680935	0,02	0,03	-0,579815015
gene:ENSG000	2	complement(196831975..19683219	0,05	-	3,068695355
PGAP1	2	complement(196833004..19692779	0,44	0,73	-0,517139567
ANKRD44	2	complement(196967017..19731117	0,42	0,55	-0,157369033
SF3B1	2	complement(197388515..19743507	28,87	27,27	0,305158922
COQ10B	2	197453493..197475310	11,31	9,75	0,437289275
HSPD1	2	complement(197486584..19751673	73,90	#####	-0,461279544
HSPE1	2	197500140..197503449	17,08	28,55	-0,517907287

HSPE1-MOB4	2	197500413..197550726	4,70	5,21	0,075258038
MOB4	2	197515571..197553699	1,78	3,01	-0,53717552
RFTN2	2	complement(197568224..19767604	0,96	0,41	1,457162612
MARS2	2	197705369..197708395	2,74	5,00	-0,646206551
BOLL	2	complement(197726879..19778676	-	0,01	-3,804623889
PLCL1	2	197804593..198572581	0,00	0,00	1,436225396
SATB2	2	complement(199269505..19947126	1,87	2,80	-0,354681587
FTCDNL1	2	complement(199760544..19985119	0,12	0,08	0,835238189
C2orf69	2	199911293..199955935	1,93	2,05	0,139991111
TYW5	2	complement(199928913..19995573	0,88	1,24	-0,265008963
MAIP1	2	199955317..200008540	3,02	5,44	-0,625629303
SPATS2L	2	200305881..200482264	9,20	9,79	0,133289188
KCTD18	2	complement(200488958..20051978	2,44	4,53	-0,672717409
SGO2	2	200510008..200584096	1,66	2,36	-0,287361657
AOX1	2	200586014..200677064	2,60	7,43	-1,293225156
BZW1	2	200810594..200827338	21,86	18,48	0,465944326
CLK1	2	complement(200853009..20086469	3,48	2,98	0,44898347
PPIL3	2	complement(200870907..20088930	3,18	3,84	-0,04814209
NIF3L1	2	200889327..200903938	9,58	12,97	-0,214936715
ORC2	2	complement(200908977..20096368	4,41	6,63	-0,364298753
HYCC2	2	complement(200973718..20107167	1,91	0,97	1,194323104
NDUFB3	2	201071433..201085750	4,34	4,56	0,151493119
CFLAR	2	201116154..201176687	1,14	1,61	-0,274149345
CASP10	2	201182872..201229428	0,51	0,93	-0,625547619
CASP8	2	201233443..201361836	1,68	4,56	-1,218325655
FLACC1	2	complement(201288271..20135739	0,02	0,12	-2,453497736
TRAK2	2	complement(201377207..20145150	3,29	6,03	-0,65164077
STRADB	2	201387858..201480846	3,46	7,55	-0,903227567
C2CD6	2	complement(201487421..20161917	-	0,00	-3,804623889
TMEM237	2	complement(201620184..20164357	3,79	6,49	-0,555546558
MPP4	2	complement(201644870..20169869	1,80	3,65	-0,799094299
ALS2	2	complement(201700267..20178211	1,98	2,43	-0,074237158
CDK15	2	201790461..201895550	0,08	0,17	-0,877307165
FZD7	2	202033855..202038441	3,81	7,20	-0,69556315
KIAA2012	2	202073255..202205188	-	0,00	-2,89252005
SUMO1	2	complement(202206182..20223859	10,55	15,48	-0,330077598
NOP58	2	202265736..202303661	8,87	13,61	-0,394548396
BMPR2	2	202376327..202567751	4,48	4,57	0,19571455
gene:ENSG000	2	202377112..202377168	-	-	#N/D
FAM117B	2	202634969..202769757	1,41	2,73	-0,725245531
ICA1L	2	complement(202773150..20287176	0,08	0,28	-1,517329466
WDR12	2	complement(202874261..20301479	2,85	5,25	-0,660234696
CARF	2	202912214..202988263	0,22	0,46	-0,858786815
NBEAL1	2	203014608..203226378	1,25	1,24	0,233135644
CYP20A1	2	203238977..203306026	1,31	2,82	-0,875732408
ABI2	2	203328280..203447728	4,53	4,69	0,175078955
RAPH1	2	complement(203394345..20353533	6,70	6,39	0,291273475
CD28	2	203706475..203738912	0,01	0,01	-0,032860829

CTLA4	2	203853888..203873965	-	-	0,188027316
ICOS	2	203936763..203961577	-	-	0,188027316
PARD3B	2	204545475..205620162	0,20	0,97	-2,029716628
NRP2	2	205681990..205798133	1,48	13,45	-2,961962445
INO80D	2	complement(205993721..20608630	1,38	2,09	-0,378107242
NDUFS1	2	complement(206114817..20615950	9,26	11,21	-0,052092636
EEF1B2	2	206159585..206162928	62,40	84,65	-0,216762016
CMKLR2	2	complement(206175316..20621804	0,04	0,96	-4,411630898
ZDBF2	2	206274663..206314427	1,51	2,45	-0,474872027
ADAM23	2	206443532..206621127	0,72	1,38	-0,703606105
FAM237A	2	206642487..206649365	-	-	#N/D
DYTN	2	complement(206651621..20672157	-	-	#N/D
MDH1B	2	complement(206737763..20676532	0,05	0,01	2,469244963
FASTKD2	2	206765357..206796189	2,45	4,65	-0,700810861
CPO	2	206939518..206969474	0,02	0,01	0,886720911
KLF7	2	complement(207074137..20717385	13,06	10,20	0,579232458
CREB1	2	207529737..207605988	2,12	2,52	-0,027334546
METTL21A	2	complement(207580631..20762592	2,02	2,53	-0,097171834
CCNYL1	2	207711540..207761839	4,64	4,08	0,406093258
FZD5	2	complement(207762598..20776990	1,35	1,42	0,145932901
PLEKHM3	2	complement(207821288..20802552	1,34	1,90	-0,28860812
CRYGD	2	complement(208121607..20812452	-	-	0,188027316
CRYGC	2	complement(208128137..20812982	-	0,02	-2,89252005
CRYGB	2	complement(208142573..20814615	-	-	#N/D
CRYGA	2	complement(208160740..20816358	-	-	#N/D
C2orf80	2	complement(208165343..20819003	-	-	#N/D
IDH1	2	complement(208236229..20826607	9,66	15,34	-0,443976474
PIKFYVE	2	208266255..208358746	5,74	4,93	0,441642721
PTH2R	2	208359714..208854503	-	0,01	-4,359055975
MAP2	2	209424047..209734147	0,11	0,19	-0,523703834
UNC80	2	209771569..209999300	0,00	0,01	-0,895537122
RPE	2	210002565..210022260	6,32	9,24	-0,32411904
KANSL1L	2	complement(210021421..21017140	0,58	1,08	-0,661595496
ACADL	2	complement(210187126..21022544	0,02	0,07	-1,806405564
MYL1	2	complement(210290150..21031517	-	-	0,188027316
LANCL1	2	complement(210427131..21047765	6,49	7,85	-0,0505628
CPS1	2	210477682..210679107	1,67	3,49	-0,838957684
ERBB4	2	complement(211375717..21253884	0,02	0,11	-2,167624691
IKZF2	2	complement(212999691..21315242	0,63	0,26	1,507473769
SPAG16	2	213284389..214410501	0,30	0,40	-0,156149184
VWC2L	2	214411054..214578976	0,01	0,01	0,921097799
BARD1	2	complement(214725646..21480968	1,73	2,62	-0,381787103
ABCA12	2	complement(214931542..21513862	0,01	0,03	-2,325891564
ATIC	2	215311956..215349773	44,69	59,65	-0,193535503
FN1	2	complement(215360440..21543607	120,04	99,30	0,496681293
MREG	2	complement(215942584..21603409	0,40	0,89	-0,921324423
PECR	2	complement(215996329..21608295	2,90	2,91	0,216266577
TMEM169	2	216081866..216102783	0,09	0,17	-0,715442817

XRCC5	2	216107464..216206303	66,81	75,10	0,054312068
gene:ENSG000	2	216211404..216213519	2,56	2,09	0,512694132
MARCHF4	2	complement(216257865..21637248	0,04	1,20	-4,53781369
SMARCAL1	2	216412383..216483053	8,82	12,55	-0,285855511
RPL37A	2	216498825..216579180	85,27	#####	-0,090195651
IGFBP2	2	216632828..216664436	4,80	17,38	-1,633952709
IGFBP5	2	complement(216672105..21669554	75,60	1,27	6,116458775
TNP1	2	complement(216859458..21686006	0,03	-	3,068695355
TNS1	2	complement(217799588..21803398	8,91	12,74	-0,293076554
RUFY4	2	218034960..218090584	-	-	#N/D
CXCR2	2	218125289..218137251	-	-	0,188027316
CXCR1	2	complement(218162841..21816696	0,00	-	3,068695355
ARPC2	2	218217141..218254356	57,12	62,51	0,093126369
GPBAR1	2	218259496..218263861	0,22	0,13	1,008480854
AAMP	2	complement(218264129..21827017	32,73	35,78	0,094478959
PNKD	2	218269651..218346793	8,27	9,20	0,069941964
TMBIM1	2	complement(218274197..21829258	24,28	22,25	0,349170421
CATIP	2	218356857..218368099	-	-	0,188027316
SLC11A1	2	218382029..218396894	0,27	0,27	0,190439085
CTDSP1	2	218397136..218405941	14,40	14,10	0,253152082
VIL1	2	218419121..218453295	0,05	0,04	0,388660064
USP37	2	complement(218450251..21856835	3,12	2,65	0,45850932
CNOT9	2	218568580..218597080	23,30	23,32	0,221462607
PLCD4	2	218607855..218637184	0,10	0,12	-0,039908994
ZNF142	2	complement(218633329..21865965	4,56	5,16	0,045110169
BCS1L	2	218658764..218663443	8,86	12,00	-0,215032978
RNF25	2	complement(218663892..21867200	6,84	7,17	0,155070718
STK36	2	218672069..218702716	4,32	4,69	0,10190356
TTLL4	2	218710835..218755416	6,46	10,98	-0,543519952
CYP27A1	2	218781749..218815293	2,58	3,40	-0,175814617
PRKAG3	2	complement(218822308..21883208	-	-	#N/D
WNT6	2	218859805..218874233	0,68	0,09	3,128388931
WNT10A	2	218880852..218899581	0,01	0,03	-0,979341556
CDK5R2	2	218959666..218962155	0,04	0,36	-2,957315122
FEV	2	complement(218981087..21898518	-	0,02	-3,804623889
CRYBA2	2	complement(218990189..21899342	0,01	-	3,068695355
CFAP65	2	complement(219002846..21904152	0,00	0,00	1,436225396
IHH	2	complement(219054424..21906092	0,00	0,02	-1,877914477
NHEJ1	2	complement(219069355..21916086	1,57	2,08	-0,179216737
gene:ENSG000	2	complement(219075329..21917082	0,12	0,08	0,692913342
SLC23A3	2	complement(219161465..21917009	0,15	0,19	-0,122056335
CNPPD1	2	complement(219171897..21917810	7,57	10,11	-0,194142587
RETREG2	2	219176225..219185475	13,44	12,29	0,351906655
ZFAND2B	2	219195237..219209651	6,77	5,50	0,522241853
ABCB6	2	complement(219209772..21921899	10,93	8,09	0,657133042
gene:ENSG000	2	complement(219209772..21922273	1,49	1,31	0,410322726
ATG9A	2	complement(219219380..21922971	22,96	24,63	0,121802242
ANKZF1	2	219229783..219236679	15,06	10,40	0,756648121

GLB1L	2	complement(219236598..21924547	1,52	1,88	-0,083085814
STK16	2	219245455..219250337	7,19	7,78	0,109148008
TUBA4A	2	complement(219249710..21927790	8,96	9,85	0,085303039
TUBA4B	2	219253243..219272197	0,02	0,04	-0,742377908
DNAJB2	2	219279342..219286898	11,08	7,98	0,697560218
PTPRN	2	complement(219289623..21930964	0,01	0,00	1,436225396
RESP18	2	complement(219327407..21933317	-	-	#N/D
DNPEP	2	complement(219372043..21940002	7,04	10,74	-0,385512336
DES	2	219418377..219426735	0,41	0,48	-0,0234459
gene:ENSG000	2	219427477..219429319	-	-	0,188027316
SPEG	2	219434843..219493629	4,93	7,27	-0,336260726
gene:ENSG000	2	219491867..219498244	0,05	0,08	-0,509043067
SPEGNB	2	219496131..219498287	0,60	0,55	0,354491181
GMPPA	2	219498865..219513898	23,52	12,15	1,175824843
ASIC4	2	219514170..219538772	0,66	0,19	2,045168398
CHPF	2	complement(219538954..21954380	35,23	25,04	0,715732876
TMEM198	2	219543663..219550595	0,98	0,85	0,419921914
OBSL1	2	complement(219550728..21957185	5,00	5,27	0,148762343
INHA	2	219569162..219575711	1,86	2,34	-0,107254101
STK11IP	2	219597857..219616451	6,51	6,20	0,295204124
SLC4A3	2	219627394..219641980	3,33	3,62	0,102534078
EPHA4	2	complement(221418027..22157420	0,33	0,71	-0,868721661
PAX3	2	complement(222199887..22229899	2,25	3,12	-0,249723165
gene:ENSG000	2	complement(222314148..22232015	-	0,03	-6,532127252
SGPP2	2	222424543..222562621	0,02	0,10	-2,416176665
FARSB	2	complement(222566899..22265609	7,14	9,27	-0,153693525
MOGAT1	2	222671658..222709930	0,03	-	4,516772761
ACSL3	2	222860942..222944639	8,65	5,60	0,851791307
KCNE4	2	223052190..223198399	0,27	0,04	2,810902903
SCG2	2	complement(223596940..22360236	0,02	0,19	-2,888990891
AP1S3	2	complement(223667680..22383802	0,48	1,11	-0,975663943
gene:ENSG000	2	complement(223757258..22394529	0,34	0,82	-1,039680863
WDFY1	2	complement(223855716..22394535	9,50	12,35	-0,155368863
MRPL44	2	223957463..223967714	10,42	12,97	-0,092063235
SERPINE2	2	complement(223975045..22403931	35,42	43,67	-0,078877845
FAM124B	2	complement(224378698..22440210	0,01	0,00	1,436225396
CUL3	2	complement(224470150..22458539	3,18	5,16	-0,47503221
CCDC195	2	complement(224703764..22471636	-	-	#N/D
DOCK10	2	complement(224765090..22504246	1,48	3,15	-0,866250242
NYAP2	2	225399710..225729887	0,00	0,01	-1,955757882
gene:ENSG000	2	225761559..225762112	-	-	0,188027316
IRS1	2	complement(226731312..22679982	7,32	8,99	-0,072931333
RHBDD1	2	226835581..226999215	5,86	8,99	-0,393168311
COL4A4	2	complement(227002714..22716445	0,04	0,25	-2,309467187
COL4A3	2	227164624..227314792	0,03	0,09	-1,380411375
MFF	2	227325151..227357833	13,53	15,91	-0,010629735
TM4SF20	2	complement(227362038..22738199	0,03	0,04	-0,392761763
SCYGR1	2	complement(227387683..22738794	-	-	#N/D

AGFG1	2	227472152..227561217	4,70	7,67	-0,483652086
SCYGR2	2	227598893..227599255	-	-	#N/D
SCYGR10	2	227608784..227609101	-	-	#N/D
SCYGR3	2	complement(227614538..22761484	-	-	#N/D
SCYGR4	2	227617498..227617815	-	-	#N/D
SCYGR5	2	227666804..227667061	-	-	#N/D
SLC19A3	2	complement(227683763..22771802	0,01	0,19	-4,092949529
SCYGR6	2	complement(227724440..22772475	-	-	#N/D
SCYGR7	2	227728335..227728625	-	-	#N/D
SCYGR8	2	227745894..227746220	-	-	#N/D
CCL20	2	227805739..227817564	0,04	0,17	-1,806405564
DAW1	2	227871054..227924344	-	0,04	-5,902279478
SPHKAP	2	complement(227979955..22818168	0,01	0,01	-1,009277154
PID1	2	complement(228850526..22927128	1,92	4,74	-1,082762656
DNER	2	complement(229357629..22971455	100,46	49,62	1,240603152
TRIP12	2	complement(229763837..22992323	22,38	24,13	0,114867022
FBXO36	2	229922302..230013119	2,87	2,23	0,588792132
SLC16A14	2	complement(230034982..23006899	0,05	0,03	1,212352745
SP110	2	complement(230165186..23022572	1,56	0,87	1,062376951
SP140	2	230203110..230313215	0,04	0,06	-0,450526226
SP140L	2	230327184..230403732	2,78	3,08	0,073071041
gene:ENSG000	2	complement(230410700..23041252	0,11	0,07	0,877784832
SP100	2	230415942..230545606	5,59	5,56	0,231155225
CAB39	2	230712842..230821075	36,61	27,96	0,611832085
ITM2C	2	230864639..230879248	27,53	25,36	0,341234158
GPR55	2	complement(230907318..23096106	0,48	0,32	0,797195761
SPATA3	2	230990324..231025055	-	-	#N/D
C2orf72	2	231037523..231049719	0,75	1,06	-0,276811142
PSMD1	2	231056845..231173116	14,76	22,27	-0,370457627
HTR2B	2	complement(231108230..23112504	0,14	0,21	-0,309905447
ARMC9	2	231198546..231376848	1,91	1,49	0,583266217
B3GNT7	2	231395671..231408799	0,18	0,13	0,64712878
NCL	2	complement(231453531..23148364	27,76	54,37	-0,746587598
NMUR1	2	complement(231523187..23153044	0,05	0,03	0,949238826
TEX44	2	231592864..231594276	0,01	0,02	-0,923955849
PTMA	2	231706895..231713551	207,91	#####	0,930742679
PDE6D	2	complement(231732433..23178627	6,20	9,13	-0,336256224
COPS7B	2	231781671..231809254	15,23	17,35	0,034541256
NPPC	2	complement(231921809..23192639	0,04	0,04	0,366665713
DIS3L2	2	231961245..232344350	3,01	3,91	-0,157058833
ALPP	2	232378724..232382889	0,01	0,01	-0,025382928
ALPG	2	232406844..232410714	0,00	0,01	-0,923955849
ALPI	2	232456125..232460753	-	-	0,188027316
ECEL1	2	complement(232479827..23248783	-	-	0,188027316
PRSS56	2	232520388..232525716	-	-	0,188027316
CHRND	2	232525993..232536667	0,09	0,16	-0,67834146
CHRNG	2	232539692..232548115	0,06	0,04	0,891510018
TIGD1	2	complement(232543883..23255055	2,76	2,61	0,304652107

EIF4E2	2	232550659..232583644	10,59	9,64	0,358548518
EFHD1	2	232606057..232682776	1,70	0,31	2,674312935
GIGYF2	2	232697299..232860605	6,94	6,15	0,397751352
KCNJ13	2	complement(232765802..23277656	0,34	0,24	0,771729157
SNORC	2	232857270..232878708	0,76	0,71	0,33134849
NGEF	2	complement(232878701..23301325	2,89	3,32	0,026515305
NEU2	2	233032672..233035057	-	0,05	-4,758582517
INPP5D	2	233059967..233207903	0,01	0,02	-0,253352605
ATG16L1	2	233210051..233295674	7,55	7,53	0,227553445
SAG	2	233307816..233347055	-	-	#N/D
DGKD	2	233354494..233472104	18,28	25,66	-0,266481112
USP40	2	complement(233475526..23356678	5,08	8,59	-0,535663905
UGT1A8	2	233617633..233773300	-	-	0,188027316
UGT1A10	2	233636448..233773300	-	-	0,188027316
UGT1A9	2	233671898..233773300	-	-	#N/D
UGT1A7	2	233681901..233773300	0,14	0,03	2,293606031
UGT1A6	2	233691607..233773300	0,07	0,05	0,503684153
UGT1A5	2	233712907..233773300	-	0,03	-5,327865481
UGT1A4	2	233718736..233773300	-	0,03	-5,071123353
UGT1A3	2	233729042..233773300	0,02	0,02	0,677647514
UGT1A1	2	233760270..233773300	0,05	0,17	-1,593583371
MROH2A	2	233775679..233833423	0,01	0,00	0,524121557
HJURP	2	complement(233833416..23385456	43,58	36,71	0,470490459
TRPM8	2	233917373..234019522	0,19	0,05	2,175495614
SPP2	2	234050679..234077134	-	-	#N/D
gene:ENSG000	2	234082259..234084490	-	-	#N/D
ARL4C	2	complement(234493041..23449708	16,57	14,92	0,374536699
SH3BP4	2	234952017..235055714	26,98	31,30	0,009026972
AGAP1	2	235494043..236131793	4,93	6,82	-0,244349844
GBX2	2	complement(236165236..23616838	0,34	0,55	-0,454739926
ASB18	2	complement(236193459..23626440	0,01	0,04	-1,573534033
IQCA1	2	complement(236324147..23650753	0,02	0,00	2,144183439
ACKR3	2	236567787..236582354	1,85	3,97	-0,879486715
COPS8	2	237085882..237100474	17,93	14,41	0,538072435
COL6A3	2	complement(237324003..23741432	471,46	#####	0,657287548
MLPH	2	237485428..237555322	0,03	0,02	0,946003371
PRLH	2	237566574..237567175	0,08	-	3,967268276
RAB17	2	complement(237574322..23760161	0,04	0,11	-1,193454645
LRRFIP1	2	237627587..237813682	7,66	10,24	-0,194745094
RBM44	2	237798389..237842808	0,03	0,06	-0,892557502
RAMP1	2	237858893..237912106	9,82	4,26	1,429687524
UBE2F	2	237966827..238042782	7,08	8,82	-0,09272739
UBE2F-SCLY	2	237967014..238099412	0,11	0,13	0,04994166
SCLY	2	238060924..238099413	1,66	4,78	-1,298152308
ESPNL	2	238100340..238133287	0,01	0,03	-1,700542576
KLHL30	2	238138668..238152947	0,02	0,23	-3,487602266
ERFE	2	238158970..238168900	3,08	5,12	-0,509505365
ILKAP	2	complement(238170402..23820370	6,56	8,56	-0,160200922

HES6	2	complement(238238267..23824066	0,57	0,60	0,15381605
PER2	2	complement(238244044..23829010	2,13	2,60	-0,06431482
TRAF3IP1	2	238320441..238400897	1,19	2,17	-0,649780906
ASB1	2	238426742..238452250	19,03	21,99	0,014497982
TWIST2	2	238848032..238910534	11,51	13,79	-0,037694272
HDAC4	2	complement(239048168..23940165	3,85	4,37	0,042564887
NDUFA10	2	complement(239892450..24002574	4,47	4,58	0,188950331
OR6B2	2	complement(240029491..24003042	-	-	0,188027316
OR6B3	2	complement(240044571..24005352	-	-	#N/D
COPS9	2	complement(240126563..24013680	6,58	5,27	0,5431912
OTOS	2	complement(240139026..24014456	0,03	-	3,967268276
GPC1	2	240435663..240468076	15,54	14,49	0,323572077
ANKMY1	2	complement(240479422..24056920	1,73	2,17	-0,102245281
DUSP28	2	240560054..240565256	0,83	0,93	0,0535491
RNPEPL1	2	240565804..240581372	11,04	9,00	0,518622784
CAPN10	2	240586734..240617705	5,07	4,72	0,324705285
GPR35	2	240605430..240633159	0,20	0,15	0,579129509
AQP12B	2	complement(240676418..24068290	-	0,00	-2,89252005
AQP12A	2	240691866..240698483	-	-	#N/D
KIF1A	2	complement(240713761..24082429	0,00	0,01	-2,665078517
AGXT	2	240868824..240880502	0,03	0,00	2,972512804
MAB21L4	2	complement(240886048..24089688	0,05	0,04	0,652912318
CROCC2	2	240906330..240993311	0,04	0,27	-2,516746689
SNED1	2	240998618..241095568	6,45	4,05	0,895081733
MTERF4	2	complement(241072169..24110233	3,09	3,31	0,122380723
PASK	2	complement(241106099..24115026	2,33	2,44	0,155126034
PPP1R7	2	241149576..241183652	7,51	8,18	0,099812314
ANO7	2	241188509..241225976	0,33	0,26	0,557338541
HDLBP	2	complement(241227264..24131706	143,54	#####	0,5969896
SEPTIN2	2	241315100..241354027	65,00	55,50	0,451105136
FARP2	2	241356285..241494841	4,49	4,48	0,229126728
STK25	2	complement(241492670..24150973	12,20	12,25	0,217420146
BOK	2	241551424..241574131	1,14	2,23	-0,746929527
THAP4	2	complement(241584405..24163715	30,08	40,39	-0,202283643
ATG4B	2	241637213..241673857	14,12	11,22	0,55456917
DTYMK	2	complement(241675747..24168694	25,56	22,18	0,427761488
ING5	2	241687085..241729478	4,84	3,28	0,783834658
D2HGDH	2	241734602..241768816	7,48	7,66	0,189933898
GAL3ST2	2	241776822..241804287	0,62	0,37	0,939969759
NEU4	2	241809065..241817413	0,09	0,09	0,258323606
PDCD1	2	complement(241849884..24185889	0,01	0,01	-0,025382928
RTP5	2	241869600..241873823	-	0,01	-4,359055975
FAM240C	2	complement(241893988..24190255	0,08	-	5,224730805
gene:ENSG000	2	242001209..242006013	0,11	0,02	2,444723701
CHL1	3	196763..409417	0,79	-	12,15314923
CNTN6	3	1092658..1404217	0,01	-	4,913749003
CNTN4	3	2098813..3057959	0,01	0,00	2,616997665
IL5RA	3	complement(3066324..3126613)	0,00	-	3,967268276

TRNT1	3	3126933..3153435	0,78	0,82	0,146369226
CRBN	3	complement(3144628..3179727)	1,50	1,97	-0,175983394
SUMF1	3	complement(3700814..4467273)	2,59	2,46	0,296949798
LRRN1	3	3799431..3849834	0,00	-	3,967268276
SETMAR	3	4303332..4317265	0,64	0,73	0,042175144
ITPR1	3	4493345..4847506	1,47	0,82	1,069793676
BHLHE40	3	4979437..4985323	61,68	20,39	1,819915036
ARL8B	3	5122249..5180911	3,53	4,82	-0,22724022
EDEM1	3	5187646..5219958	16,72	13,74	0,506698044
GRM7	3	6770001..7741533	0,00	-	3,068695355
LMCD1	3	8501807..8574668	0,49	0,29	0,998421453
SSUH2	3	complement(8619386..8745040)	0,03	0,06	-0,472094744
CAV3	3	8733802..8841808	0,04	0,04	0,278120973
OXTR	3	complement(8750381..8769628)	0,12	0,17	-0,282866473
RAD18	3	complement(8775402..8963773)	3,04	3,37	0,074088422
SRGAP3	3	complement(8980591..9363053)	0,18	0,24	-0,205055954
THUMPD3	3	9362971..9386791	3,46	4,80	-0,251863572
SETD5	3	9397615..9479240	11,78	10,48	0,391144042
LHFPL4	3	complement(9498361..9553822)	-	0,10	-7,964031459
MTMR14	3	9649433..9702393	4,70	5,17	0,087624273
CPNE9	3	9703826..9729908	0,02	0,01	0,524121557
BRPF1	3	9731729..9748019	4,14	3,51	0,461268591
OGG1	3	9749944..9788219	2,41	2,30	0,288451187
CAMK1	3	complement(9757347..9769992)	1,48	1,93	-0,165683115
TADA3	3	complement(9779967..9793011)	13,59	17,12	-0,11076123
ARPC4	3	9792495..9807101	31,26	32,96	0,146979727
ARPC4-TTLL3	3	9793082..9835401	0,90	0,50	1,068573585
TTLL3	3	9808086..9855138	2,36	2,22	0,310914414
RPUSD3	3	complement(9837849..9844602)	5,15	8,83	-0,554382218
CIDEC	3	complement(9866711..9880255)	0,05	0,01	2,399873901
JAGN1	3	9890574..9894349	8,47	11,78	-0,252578906
IL17RE	3	9902612..9916402	0,26	0,20	0,595489841
IL17RC	3	9917074..9933630	4,53	3,38	0,644085268
gene:ENSG000	3	9917116..9945385	0,66	0,08	3,332808981
CRELD1	3	9933793..9945413	8,03	3,65	1,360280668
PRRT3	3	complement(9939450..9952408)	0,40	0,81	-0,804482703
EMC3	3	complement(9962682..10011202)	6,32	6,36	0,212409843
FANCD2	3	10026370..10101932	5,04	8,07	-0,455610623
FANCD2OS	3	complement(10081317..10108255)	0,01	0,09	-3,171495545
BRK1	3	10115675..10127190	30,56	22,82	0,644174449
VHL	3	10141778..10153667	7,24	10,60	-0,326977422
IRAK2	3	10164919..10243745	5,52	11,68	-0,857286893
TATDN2	3	10248023..10281218	8,66	8,45	0,258826148
gene:ENSG000	3	10249042..10249366	-	-	#N/D
gene:ENSG000	3	10249372..10285796	5,01	4,00	0,549777553
GHRL	3	complement(10285666..10292947)	0,28	0,29	0,168652135
SEC13	3	complement(10293131..10321112)	56,95	43,43	0,614231778
ATP2B2	3	complement(10324023..10708007)	0,00	0,02	-3,929150322

SLC6A11	3	10816201..10940714	0,01	0,13	-4,339590204
SLC6A1	3	10992186..11039247	-	0,00	-3,804623889
HRH1	3	11137093..11263557	1,26	2,60	-0,824405521
ATG7	3	11272309..11557665	3,36	5,02	-0,354972402
VGLL4	3	complement(11556069..11771350)	5,34	3,22	0,949465708
TAMM41	3	complement(11790442..11846919)	1,17	1,53	-0,163909736
SYN2	3	12004388..12192032	0,00	0,02	-2,854330803
TIMP4	3	complement(12153068..12158912)	0,80	2,46	-1,390005231
PPARG	3	12287368..12434356	1,56	2,65	-0,545616123
TSEN2	3	12484421..12541549	0,41	1,02	-1,085892735
MKRN2OS	3	complement(12514934..12561059)	0,07	0,09	-0,225481127
MKRN2	3	12557057..12586208	4,01	3,54	0,402112798
RAF1	3	complement(12582101..12664201)	7,00	7,27	0,169230707
TMEM40	3	complement(12733528..12769457)	0,22	0,06	2,010690243
CAND2	3	12796472..12834804	0,50	0,32	0,856091781
gene:ENSG000	3	12825768..12871919	-	-	0,188027316
RPL32	3	complement(12834485..12841582)	90,43	98,80	0,095377769
IQSEC1	3	complement(12897043..13283281)	2,17	2,55	-0,012167805
NUP210	3	complement(13316235..13420322)	8,65	30,12	-1,576321723
HDAC11	3	13479724..13506424	1,77	1,73	0,253873209
FBLN2	3	13549125..13638422	10,19	18,29	-0,621513903
WNT7A	3	complement(13816258..13880071)	-	-	#N/D
CHCHD4	3	complement(14112077..14124870)	4,79	7,97	-0,511933422
TMEM43	3	14125015..14143680	17,31	27,68	-0,453914038
gene:ENSG000	3	14135217..14148252	-	-	0,188027316
XPC	3	complement(14145147..14178621)	4,11	6,88	-0,519898282
LSM3	3	14178817..14201122	4,30	3,54	0,502587834
SLC6A6	3	14402576..14489349	20,56	8,55	1,488418118
GRIP2	3	complement(14489107..14556075)	0,04	0,04	0,221971569
CCDC174	3	14651762..14672659	2,10	2,26	0,115614686
C3orf20	3	14675141..14773036	-	0,02	-4,758582517
FGD5	3	14810853..14934571	0,00	-	3,068695355
NR2C2	3	14947583..15053600	3,43	4,14	-0,047935586
MRPS25	3	complement(15009611..15065339)	1,82	2,11	0,008919525
RBSN	3	complement(15070073..15099163)	4,77	4,46	0,319338916
CAPN7	3	15206152..15252916	2,86	3,81	-0,192493317
SH3BP5	3	complement(15254353..15341368)	1,59	2,44	-0,397049609
METTL6	3	complement(15381275..15440566)	0,98	1,20	-0,066317643
EAF1	3	15427598..15450635	5,85	6,21	0,136544559
gene:ENSG000	3	complement(15439199..15455940)	-	-	0,188027316
COLQ	3	complement(15450133..15521751)	0,06	0,14	-1,073091301
HACL1	3	complement(15560699..15601852)	2,92	3,91	-0,20065217
BTD	3	15601341..15722311	0,83	0,95	0,027026053
ANKRD28	3	complement(15667236..15859771)	2,70	5,65	-0,841912113
gene:ENSG000	3	15731877..15732536	0,21	0,35	-0,50374212
GALNT15	3	16174680..16231992	0,11	3,37	-4,662330716
DPH3	3	complement(16257061..16264969)	1,89	2,05	0,10438076
OXNAD1	3	16265160..16350299	1,70	1,90	0,058438721

RFTN1	3	complement(16313574..16514026)	17,13	10,10	0,986181484
DAZL	3	complement(16586792..16670306)	-	0,00	-2,89252005
PLCL2	3	16802651..17090604	0,47	1,02	-0,896832778
TBC1D5	3	complement(17157162..18444817)	7,78	8,04	0,17678774
SATB1	3	complement(18345377..18445621)	0,70	-	12,13930273
KCNH8	3	19148510..19535642	-	-	0,188027316
EFHB	3	complement(19879472..19947025)	0,02	0,02	-0,291161992
RAB5A	3	19947097..19985175	6,45	6,74	0,159485605
PP2D1	3	complement(19979961..20012267)	0,03	0,05	-0,609885528
KAT2B	3	20040446..20154404	1,22	1,50	-0,072141505
SGO1	3	complement(20160593..20186206)	2,12	3,32	-0,426281881
ZNF385D	3	complement(21412218..22373321)	0,02	0,08	-1,601968487
UBE2E2	3	23203020..23591794	1,86	4,26	-0,972872275
UBE2E1	3	23805955..23891640	7,41	13,02	-0,590206817
NKIRAS1	3	complement(23889951..23946591)	0,55	0,97	-0,590043813
RPL15	3	23916591..23924374	86,52	98,94	0,029655422
NR1D2	3	23945286..23980617	3,67	5,97	-0,479109106
THRB	3	complement(24117153..24495756)	0,55	0,59	0,116781085
RARB	3	24687887..25597932	0,42	0,41	0,259258901
TOP2B	3	complement(25597978..25664909)	5,59	7,99	-0,290782674
NGLY1	3	complement(25718944..25790039)	2,31	4,04	-0,583399691
OXSM	3	25782917..25794534	0,77	1,90	-1,074041794
LRRC3B	3	26622772..26717537	-	-	0,188027316
NEK10	3	complement(27106484..27369460)	0,13	0,11	0,537280603
SLC4A7	3	complement(27372723..27484420)	27,47	8,74	1,875574955
EOMES	3	complement(27715949..27722711)	0,17	0,19	0,038948827
CMC1	3	28241584..28325142	0,26	0,41	-0,398635075
AZI2	3	complement(28315003..28349050)	1,51	2,78	-0,660133637
ZCWPW2	3	28348721..28538122	0,05	0,05	0,263392682
gene:ENSG000	3	28349178..29767537	0,06	0,07	0,111704005
RBMS3	3	28574791..30010391	1,28	1,16	0,370243698
TGFBR2	3	30606478..30694249	9,47	17,64	-0,675153881
GADL1	3	complement(30726197..30894661)	0,00	-	3,068695355
STT3B	3	31532638..31637616	11,91	17,59	-0,339771637
OSBPL10	3	complement(31657912..32077580)	0,51	0,94	-0,662644144
ZNF860	3	31981750..31991723	0,00	-	3,068695355
GPD1L	3	32105689..32168709	2,15	4,67	-0,892593374
CMTM8	3	32238679..32370321	0,85	1,37	-0,470614564
CMTM7	3	32391698..32483067	1,83	5,47	-1,357995214
CMTM6	3	complement(32481312..32502852)	7,16	8,98	-0,103222382
DYNC1LI1	3	complement(32525974..32570858)	5,76	8,03	-0,256266873
CNOT10	3	32685145..32773875	2,36	3,98	-0,533743063
TRIM71	3	32817997..32897824	0,00	-	3,967268276
CCR4	3	32951644..32956349	0,01	-	3,967268276
GLB1	3	complement(32996609..33097202)	10,96	14,19	-0,149484748
TMPPE	3	complement(33090421..33097146)	1,40	1,85	-0,183213163
CRTAP	3	33114014..33147773	34,05	35,75	0,15276221
SUSD5	3	complement(33150043..33218810)	3,71	8,46	-0,965987864

FBXL2	3	33277025..33403662	2,41	1,97	0,510284258
UBP1	3	complement(33388336..33441371)	7,79	9,59	-0,077752808
CLASP2	3	complement(33496245..33718356)	1,55	3,59	-0,987702238
PDCD6IP	3	33798571..33869707	8,51	11,14	-0,165378413
ARPP21	3	35638945..35794496	0,01	-	5,224730805
STAC	3	36380503..36548007	0,96	2,96	-1,395570374
DCLK3	3	complement(36712421..36764553)	-	-	0,188027316
gene:ENSG000	3	complement(36823151..36825158)	-	0,01	-2,89252005
TRANK1	3	complement(36826819..36945057)	0,48	0,88	-0,660041545
EPM2AIP1	3	complement(36985043..36993131)	2,35	1,92	0,517737779
MLH1	3	36993226..37050896	4,07	6,35	-0,418682854
LRRFIP2	3	complement(37052626..37183689)	2,38	4,13	-0,574603118
GOLGA4	3	37243191..37366879	6,27	7,55	-0,045816419
ITGA9	3	37452115..37823507	0,04	0,05	0,101548111
CTDSPL	3	37861880..37984469	6,42	6,17	0,279267577
VILL	3	37988059..38007188	0,37	0,36	0,264630959
PLCD1	3	complement(38007496..38029642)	0,40	0,23	0,983189474
DLEC1	3	38039205..38124025	0,10	0,10	0,231089287
ACAA1	3	complement(38103129..38137242)	5,14	5,00	0,26263885
MYD88	3	38138552..38143024	1,06	2,56	-1,043022851
OXSR1	3	38165089..38255484	9,75	11,16	0,027857799
SLC22A13	3	38265812..38278757	0,11	0,18	-0,523371639
SLC22A14	3	38282294..38318575	0,03	0,05	-0,225481127
XYLB	3	38346760..38421348	0,52	1,00	-0,715537481
ACVR2B	3	38453890..38493142	0,86	1,52	-0,600922493
EXOG	3	38496127..38542161	0,67	1,16	-0,577368757
SCN5A	3	complement(38548057..38649743)	0,04	0,01	1,833543142
SCN10A	3	complement(38696802..38816286)	-	-	#N/D
SCN11A	3	complement(38845764..39052157)	0,00	-	3,967268276
WDR48	3	39052013..39096671	3,16	4,71	-0,353594918
GORASP1	3	complement(39095222..39108369)	5,36	4,81	0,379783662
TTC21A	3	39107680..39138903	0,60	0,53	0,378134219
CSRNP1	3	complement(39141855..39154562)	6,69	3,20	1,286689632
XIRP1	3	complement(39183210..39192596)	-	0,02	-5,902279478
CX3CR1	3	complement(39263495..39281735)	-	-	#N/D
CCR8	3	39329709..39333680	-	-	0,188027316
SLC25A38	3	39383370..39397351	6,37	7,63	-0,037037039
RPSA	3	39406716..39426255	169,29	#####	0,459051723
MOBP	3	39467198..39529479	0,00	0,01	-0,429837071
MYRIP	3	39808914..40260321	0,01	0,03	-0,895537122
EIF1B	3	40309707..40312424	8,88	10,84	-0,063724738
gene:ENSG000	3	40312086..40312214	-	-	#N/D
ENTPD3	3	40387184..40428744	0,01	0,02	-1,291882393
RPL14	3	40457292..40468587	20,92	26,31	-0,107631517
ZNF619	3	40477113..40491053	0,98	1,63	-0,505632991
ZNF620	3	40477131..40518736	0,16	0,17	0,151862326
ZNF621	3	40524878..40574685	2,64	3,71	-0,268177535
CTNNB1	3	41194741..41260096	25,87	30,33	-0,00665048

ULK4	3	complement(41246599..41962130)	0,53	0,70	-0,169256483
TRAK1	3	42013802..42225890	4,03	5,36	-0,189612621
CCK	3	complement(42257825..42266185)	0,05	0,18	-1,733452083
LYZL4	3	complement(42397083..42410610)	-	-	0,188027316
VIPR1	3	42489299..42537573	0,01	0,00	2,144183439
SEC22C	3	complement(42547969..42601080)	4,22	4,96	-0,011459343
SS18L2	3	42581840..42596934	1,40	1,69	-0,048526456
NKTR	3	42600655..42648735	5,76	5,07	0,407197027
ZBTB47	3	42653697..42667580	3,45	4,31	-0,099182656
KLHL40	3	42685537..42692544	0,02	-	4,913749003
HHATL	3	complement(42692663..42702824)	-	-	0,188027316
CCDC13	3	complement(42705756..42773253)	-	-	0,188027316
gene:ENSG000	3	complement(42705756..42804518)	0,45	0,55	-0,070597843
HIGD1A	3	complement(42782908..42804490)	3,51	5,24	-0,356862023
ACKR2	3	42804752..42887974	0,03	0,01	2,060408965
gene:ENSG000	3	42809446..43055871	-	-	0,188027316
gene:ENSG000	3	42809483..42942792	-	-	#N/D
CYP8B1	3	complement(42856005..42875898)	-	0,00	-2,89252005
ZNF662	3	42906142..42919334	-	-	0,188027316
KRBOX1	3	42936386..42942792	-	-	0,188027316
GASK1A	3	42979087..43067898	0,02	0,01	1,225128498
POMGNT2	3	complement(43079229..43106085)	5,66	6,47	0,029121702
SNRK	3	43286512..43424764	2,77	2,31	0,486638772
ANO10	3	complement(43354859..43691594)	6,11	6,31	0,176955208
ABHD5	3	43690108..43734371	0,59	0,91	-0,405329959
gene:ENSG000	3	complement(44115818..44117487)	0,25	0,73	-1,343647809
TOPAZ1	3	44241886..44332098	-	-	0,188027316
TCAIM	3	44338119..44409451	0,70	1,10	-0,43116429
ZNF445	3	complement(44431705..44477670)	2,26	2,27	0,212178304
ZNF852	3	complement(44491766..44510636)	0,62	0,79	-0,14436205
ZKSCAN7	3	44555193..44594173	0,02	0,02	0,221270597
ZNF660	3	44578223..44599694	0,00	0,03	-2,752886291
ZNF197	3	44584888..44648471	1,20	1,71	-0,28839846
ZNF35	3	44648732..44660791	1,65	2,16	-0,166292563
ZNF502	3	44712643..44723831	-	-	0,188027316
ZNF501	3	44729596..44737083	-	-	0,188027316
KIAA1143	3	complement(44737661..44761619)	2,35	3,23	-0,23412648
KIF15	3	44761721..44873376	2,40	3,33	-0,248697508
TMEM42	3	44861904..44865670	2,37	4,32	-0,640957542
TGM4	3	44874608..44914990	0,02	0,03	-0,698295353
ZDHHC3	3	complement(44915257..44976185)	2,12	2,73	-0,144628711
EXOSC7	3	44975241..45036066	7,24	10,65	-0,332725125
CLEC3B	3	45001548..45036071	0,42	0,10	2,300601245
CDCP1	3	complement(45082277..45146422)	0,40	0,59	-0,330097335
TMEM158	3	complement(45224466..45226287)	52,89	55,75	0,146951051
LARS2	3	45388561..45554726	3,42	4,80	-0,266740381
LIMD1	3	45555394..45686341	7,81	6,38	0,514826075
SACM1L	3	45689056..45745412	1,79	2,06	0,020738276

gene:ENSG000	3	45749627..45750651	0,07	0,01	2,616997665
SLC6A20	3	complement(45755449..45796574)	-	-	0,188027316
LZTFL1	3	complement(45823316..45916042)	0,53	0,85	-0,473934688
CCR9	3	45884425..45903174	0,01	0,01	1,232079601
FYCO1	3	complement(45917903..45995824)	8,14	8,74	0,12030593
CXCR6	3	45940933..45948351	0,61	0,41	0,805200703
XCR1	3	complement(46016990..46085825)	-	-	0,188027316
CCR3	3	46130890..46266706	-	-	0,188027316
CCR1	3	complement(46201711..46208313)	-	-	0,188027316
CCR2	3	46353864..46360940	-	-	0,188027316
CCR5	3	46370946..46376206	-	-	0,188027316
CCRL2	3	46407166..46412997	0,02	0,13	-2,783775817
LTF	3	complement(46435645..46485234)	-	0,34	-9,252260195
RTP3	3	46494611..46500950	0,01	0,07	-3,021611439
LRRC2	3	complement(46515385..46606948)	-	0,00	-2,89252005
CRIPTO	3	46574534..46582457	-	-	0,188027316
gene:ENSG000	3	46581150..46617226	-	-	#N/D
FAM240A	3	46612525..46626543	-	-	0,188027316
ALS2CL	3	complement(46668995..46693704)	1,66	1,49	0,37844427
TMIE	3	46694528..46710886	-	-	0,188027316
gene:ENSG000	3	complement(46712115..46812574)	0,02	0,00	2,144183439
PRSS50	3	complement(46712117..46717869)	0,06	0,07	-0,036228841
MYL3	3	complement(46835110..46882178)	0,01	0,04	-1,326441896
PTH1R	3	46877721..46903799	0,04	0,15	-1,806412189
CCDC12	3	complement(46921726..46982010)	9,05	8,64	0,290626592
NBEAL2	3	46979666..47009704	2,81	5,74	-0,807863606
SETD2	3	complement(47016428..47164113)	2,89	4,66	-0,462357959
KIF9	3	complement(47227998..47283451)	0,46	0,52	0,035182746
KLHL18	3	47282917..47346816	3,24	4,65	-0,298653684
gene:ENSG000	3	47346950..47349073	0,39	0,65	-0,533249498
PTPN23	3	47381011..47413435	11,87	12,45	0,15487016
SCAP	3	complement(47413681..47477126)	15,40	17,53	0,036312578
ELP6	3	complement(47495640..47513712)	4,98	7,19	-0,306967153
CSPG5	3	complement(47562238..47580792)	0,17	0,29	-0,560386154
SMARCC1	3	complement(47585269..47782106)	9,34	16,28	-0,577957757
DHX30	3	47802909..47850195	13,11	18,52	-0,275295617
MAP4	3	complement(47850690..48089272)	29,43	30,51	0,171373292
CDC25A	3	complement(48157146..48188417)	3,99	5,23	-0,169754364
CAMP	3	48223347..48225491	-	-	#N/D
ZNF589	3	48241100..48299253	1,47	1,53	0,171448003
NME6	3	complement(48290722..48301685)	1,23	1,91	-0,419088516
SPINK8	3	complement(48306842..48333661)	-	-	#N/D
FBXW12	3	48372219..48401259	0,01	0,01	0,886720911
PLXNB1	3	complement(48403854..48430086)	1,29	1,51	0,00206882
CCDC51	3	complement(48432164..48440456)	3,04	3,39	0,067038605
TMA7	3	48440257..48444208	8,61	10,75	-0,097623293
ATRIP	3	48446710..48467645	1,27	1,91	-0,359872043
TREX1	3	48465811..48467645	5,54	3,05	1,08438549

SHISA5	3	complement(48467798..48504826)	26,21	31,17	-0,02682677
PFKFB4	3	complement(48517684..48562015)	5,74	2,90	1,206303489
UCN2	3	complement(48561718..48563781)	0,53	0,76	-0,289997566
COL7A1	3	complement(48564073..48595329)	2,18	2,69	-0,082403359
UQCRC1	3	complement(48599002..48610976)	88,96	73,62	0,496231698
TMEM89	3	complement(48620759..48621769)	0,04	0,02	0,886720911
SLC26A6	3	complement(48625723..48635493)	13,68	9,44	0,758450101
CELSR3	3	complement(48636463..48662886)	1,69	2,26	-0,192704011
NCKIPSD	3	complement(48673844..48686364)	8,77	6,48	0,66078426
IP6K2	3	complement(48688003..48740353)	6,47	7,51	0,007885293
PRKAR2A	3	complement(48744597..48847874)	7,21	9,29	-0,141777668
SLC25A20	3	complement(48856926..48898904)	5,97	3,77	0,886659661
ARIH2	3	48918821..48986382	7,46	10,59	-0,281410439
P4HTM	3	48989889..49007153	0,01	0,07	-2,394315395
WDR6	3	49007062..49015953	22,46	19,93	0,395236457
DALRD3	3	complement(49015488..49022293)	5,09	5,35	0,150735108
NDUFAF3	3	49020459..49023495	14,17	16,05	0,04333322
IMPDH2	3	complement(49024325..49029447)	48,98	61,66	-0,108950937
gene:ENSG000	3	complement(49024345..49094071)	0,45	0,34	0,643033354
QRICH1	3	complement(49029707..49094363)	10,34	13,48	-0,159648864
QARS1	3	complement(49095932..49105130)	33,83	25,85	0,611083736
USP19	3	complement(49108046..49120938)	9,82	11,72	-0,031731143
LAMB2	3	complement(49121114..49133118)	36,06	23,63	0,832918188
CCDC71	3	complement(49162535..49166331)	7,83	10,34	-0,179355802
KLHDC8B	3	49171598..49176486	1,47	1,06	0,693934086
C3orf84	3	complement(49177634..49191858)	0,02	0,02	-0,01185201
IHO1	3	49198428..49258106	0,01	-	3,967268276
C3orf62	3	complement(49268596..49277232)	0,26	0,46	-0,592372147
USP4	3	complement(49277144..49340712)	8,25	8,76	0,136243579
GPX1	3	complement(49357174..49358605)	50,32	71,98	-0,293327116
gene:ENSG000	3	complement(49357176..49412051)	20,84	21,28	0,193409129
RHOA	3	complement(49359139..49412998)	64,92	53,67	0,49768313
TCTA	3	49412212..49416476	11,18	4,64	1,491548832
gene:ENSG000	3	complement(49416777..49429314)	1,64	1,61	0,244083419
AMT	3	complement(49416778..49422685)	1,71	1,40	0,516893999
NICN1	3	complement(49422333..49429326)	3,10	3,22	0,167845261
DAG1	3	49468713..49535618	28,05	34,22	-0,063797062
BSN	3	49554477..49671549	0,10	0,20	-0,822727922
APEH	3	49674014..49683971	10,34	11,21	0,106554926
MST1	3	complement(49683947..49689501)	1,11	2,37	-0,879222306
RNF123	3	49689538..49721529	5,22	7,03	-0,205635632
AMIGO3	3	complement(49716829..49719684)	0,23	0,33	-0,279043308
GMPPB	3	complement(49716844..49723973)	6,89	4,04	0,992822562
IP6K1	3	complement(49724294..49786542)	7,08	7,74	0,094603033
CDHR4	3	complement(49790732..49799873)	0,11	0,06	1,18925945
INKA1	3	49803261..49805030	0,75	0,88	0,000143391
UBA7	3	complement(49805209..49813953)	4,54	2,01	1,40184567
TRAIP	3	complement(49828601..49856574)	2,09	3,10	-0,34109713

CAMKV	3	complement(49857988..49869935)	-	0,01	-3,804623889
MST1R	3	complement(49886902..49903873)	0,30	0,12	1,500104618
MON1A	3	complement(49907160..49930173)	1,34	1,67	-0,100007152
RBM6	3	49940007..50100045	3,63	4,89	-0,207134768
RBM5	3	50088919..50119021	3,74	4,19	0,058075928
SEMA3F	3	50155045..50189075	24,68	14,94	0,946705855
GNAT1	3	50191610..50197696	0,00	0,00	-0,01185201
SLC38A3	3	50205246..50221486	0,03	0,03	0,279559702
GNAI2	3	50226292..50259362	65,31	43,88	0,796835528
SEMA3B	3	50267558..50277546	2,72	4,67	-0,556629892
LSMEM2	3	50279087..50288114	0,78	0,65	0,489125864
IFRD2	3	complement(50287732..50292918)	15,89	22,19	-0,258828572
HYAL3	3	complement(50292831..50299405)	1,21	1,83	-0,37812993
NAA80	3	complement(50296402..50299416)	4,49	3,49	0,587501636
HYAL1	3	complement(50299890..50312381)	0,30	0,26	0,447351109
HYAL2	3	complement(50317790..50322782)	7,79	5,59	0,702137191
TUSC2	3	complement(50320027..50328251)	6,48	5,21	0,537700075
RASSF1	3	complement(50329782..50340980)	10,68	7,15	0,801117662
ZMYND10	3	complement(50341110..50345732)	1,41	0,61	1,423234266
NPRL2	3	complement(50347330..50350826)	14,46	5,26	1,681859443
CYB561D2	3	50350845..50358460	10,46	4,91	1,314146646
gene:ENSG000	3	50350892..50367923	1,61	0,25	2,8695898
TMEM115	3	complement(50354750..50359521)	12,60	14,50	0,021368299
CACNA2D2	3	complement(50362613..50504244)	2,62	0,23	3,754714054
C3orf18	3	complement(50558025..50571027)	2,84	2,87	0,205396752
HEMK1	3	50569152..50596168	2,11	1,35	0,867067197
CISH	3	complement(50606489..50611774)	2,04	0,55	2,120504946
MAPKAPK3	3	50611520..50649291	6,76	11,55	-0,549408384
DOCK3	3	50674927..51384198	0,12	0,29	-1,060852257
MANF	3	51385291..51389397	63,26	29,81	1,308385463
RBM15B	3	51391285..51397908	5,52	6,38	0,014653112
DCAF1	3	complement(51395867..51500015)	6,43	7,04	0,092601791
RAD54L2	3	51538719..51668667	3,72	5,19	-0,259236894
TEX264	3	51662693..51704323	6,95	9,19	-0,18104627
GRM2	3	51707068..51718613	0,01	-	3,967268276
IQCF6	3	complement(51778561..51779241)	-	-	#N/D
gene:ENSG000	3	51798803..51830856	-	-	#N/D
IQCF3	3	51826883..51833389	-	-	#N/D
IQCF2	3	51861615..51863424	-	-	#N/D
IQCF5	3	complement(51873721..51875601)	-	-	0,188027316
IQCF1	3	complement(51894876..51903353)	0,01	-	3,068695355
RRP9	3	complement(51933429..51941904)	13,41	15,77	-0,010462103
PARP3	3	51942345..51948867	2,27	4,67	-0,81482981
gene:ENSG000	3	complement(51951849..51953902)	0,16	0,13	0,589040426
GPR62	3	51955381..51957499	0,13	0,10	0,599973812
PCBP4	3	complement(51957454..51974016)	8,14	7,35	0,37054244
ABHD14B	3	complement(51968510..51983409)	14,82	12,07	0,520258392
ABHD14A	3	51971426..51981196	1,03	1,76	-0,557587017

ABHD14A-ACY1	3	51974706..51989183	0,48	0,79	-0,504753595
ACY1	3	51983340..51989197	3,78	5,68	-0,362351858
RPL29	3	complement(51993522..51995895)	144,47	#####	-0,105918147
DUSP7	3	complement(52048919..52056571)	5,51	5,22	0,300711925
POC1A	3	complement(52075226..52154690)	5,30	8,19	-0,404961026
ALAS1	3	52198086..52214327	23,64	26,39	0,064579527
gene:ENSG000	3	complement(52217016..52219012)	0,09	0,03	1,613114315
TLR9	3	complement(52221080..52225645)	0,27	0,18	0,778186742
gene:ENSG000	3	complement(52221081..52231190)	-	-	0,188027316
TWF2	3	complement(52228612..52246788)	26,52	26,17	0,242000302
PPM1M	3	52245759..52250599	7,45	7,07	0,299939848
WDR82	3	complement(52254434..52288020)	13,42	16,28	-0,055668656
GLYCTK	3	52287089..52295257	1,06	1,27	-0,044537703
DNAH1	3	52316319..52400492	1,06	1,25	-0,010391722
BAP1	3	complement(52401008..52410008)	21,00	20,43	0,26313635
PHF7	3	52410660..52423641	0,78	1,35	-0,570791846
SEMA3G	3	complement(52433035..52445103)	0,03	0,03	-0,038353104
TNNC1	3	complement(52451100..52454041)	0,04	0,17	-1,700542576
NISCH	3	52455118..52493068	7,28	5,94	0,515405263
STAB1	3	52495338..52524495	0,04	0,01	2,208050693
NT5DC2	3	complement(52524385..52535054)	29,56	25,19	0,454020428
UQCC5	3	52534013..52579237	1,42	0,80	1,054811554
PBRM1	3	complement(52545352..52685917)	2,07	3,45	-0,515683861
GNL3	3	52681156..52694497	26,27	26,28	0,222938812
GLT8D1	3	complement(52694486..52706032)	7,96	8,76	0,08471788
SPCS1	3	52704955..52711148	4,50	5,71	-0,122280863
NEK4	3	complement(52708444..52770946)	1,54	2,40	-0,415467779
ITIH1	3	52777595..52792068	-	0,00	-2,89252005
ITIH3	3	52794768..52808799	0,00	-	3,068695355
ITIH4	3	complement(52812962..52830688)	0,00	0,01	-0,923955849
gene:ENSG000	3	complement(52813282..52835729)	0,16	0,15	0,31154454
MUSTN1	3	complement(52833114..52835019)	0,38	0,22	1,011020992
STIMATE-MUST	3	complement(52833121..52897562)	0,08	-	6,338059249
STIMATE	3	complement(52836219..52897548)	3,46	3,25	0,313690417
gene:ENSG000	3	complement(52848085..52848553)	0,41	0,17	1,524737598
SFMBT1	3	complement(52903572..53046750)	0,56	1,10	-0,752176122
gene:ENSG000	3	complement(52969119..53099453)	0,13	0,11	0,509087802
gene:ENSG000	3	53046166..53048122	0,02	0,05	-1,21700111
RFT1	3	complement(53088483..53130453)	4,13	5,24	-0,119833845
PRKCD	3	53156009..53192717	4,70	5,87	-0,097994791
TKT	3	complement(53224712..53256052)	152,89	#####	0,693994429
DCP1A	3	complement(53283429..53347586)	5,04	4,96	0,245718088
CACNA1D	3	53328963..53813733	0,02	0,00	2,505933813
CHDH	3	complement(53812335..53846419)	0,88	1,42	-0,480120657
IL17RB	3	53846568..53865794	1,31	1,46	0,0692796
ACTR8	3	complement(53867066..53882152)	2,68	2,32	0,435044658
SELENOK	3	complement(53884417..53891885)	9,92	6,51	0,830368143
CACNA2D3	3	54122552..55074557	0,11	0,23	-0,925363975

LRTM1	3	complement(54918231..54967088)	-	-	0,188027316
WNT5A	3	complement(55465715..55490539)	5,32	6,51	-0,068991272
ERC2	3	complement(55508311..56469095)	0,00	0,01	-0,742377908
CCDC66	3	56557161..56621837	0,53	0,77	-0,326910579
TASOR	3	complement(56620132..56683265)	2,43	4,41	-0,634565453
ARHGEF3	3	complement(56727418..57079329)	0,81	1,13	-0,258012617
SPATA12	3	57060664..57075432	0,17	0,15	0,387002327
IL17RD	3	complement(57089982..57170306)	0,59	0,28	1,304349308
HESX1	3	complement(57197838..57227606)	0,14	0,27	-0,741496626
APPL1	3	57227726..57278105	3,18	5,44	-0,54999016
ASB14	3	complement(57268342..57292685)	0,07	0,08	0,170743842
DNAH12	3	complement(57293699..57544344)	0,00	0,02	-2,851509423
PDE12	3	57556274..57566848	2,71	2,78	0,186652871
ARF4	3	complement(57571363..57598220)	81,13	39,62	1,257212312
DENND6A	3	complement(57625454..57693077)	2,72	3,32	-0,063705124
SLMAP	3	57755450..57930003	3,53	4,02	0,034771432
FLNB	3	58008398..58172251	39,15	41,16	0,150502615
DNASE1L3	3	complement(58192257..58214697)	-	-	#N/D
ABHD6	3	58237532..58295693	1,57	2,85	-0,642848377
RPP14	3	58306245..58324695	1,38	2,08	-0,368058514
HTD2	3	58306262..58320193	0,66	0,44	0,792289667
PXK	3	58332880..58426127	4,52	7,84	-0,570367853
PDHB	3	complement(58427630..58433857)	15,68	17,82	0,039216974
KCTD6	3	58492096..58502360	0,97	1,95	-0,787825511
ACOX2	3	complement(58505136..58537283)	0,87	2,75	-1,431182608
FAM107A	3	complement(58564117..58627610)	5,22	0,82	2,895381082
FAM3D	3	complement(58633946..58666834)	0,03	0,02	1,232079601
CFAP20DC	3	complement(58717365..59050084)	0,21	0,32	-0,381509203
FHIT	3	complement(59747277..61251459)	0,18	0,03	2,96099218
PTPRG	3	61561569..62297609	2,40	5,54	-0,980834023
CEP15	3	62319015..62336213	-	-	0,188027316
FEZF2	3	complement(62369681..62374324)	-	-	#N/D
CADPS	3	complement(62398346..62875416)	0,02	0,03	-0,125580693
SYNPR	3	63228315..63616924	-	-	0,188027316
SNTN	3	63652675..63679020	-	0,00	-2,89252005
C3orf49	3	63819299..63848636	0,02	0,10	-2,097518818
THOC7	3	complement(63833870..63863868)	7,64	14,87	-0,738686402
ATXN7	3	63863155..64003462	3,01	3,83	-0,124670504
PSMD6	3	complement(64010550..64024010)	9,11	15,82	-0,572777535
PRICKLE2	3	complement(64092236..64445476)	1,53	1,23	0,535267551
ADAMTS9	3	complement(64515654..64688000)	0,09	0,00	4,504010512
MAGI1	3	complement(65353525..66038918)	0,61	0,89	-0,336202363
SLC25A26	3	66133610..66388116	0,74	0,86	0,008622951
LRIG1	3	complement(66378797..66501263)	1,72	9,40	-2,228256633
KBTBD8	3	66998307..67011210	0,07	0,20	-1,271864916
SUCLG2	3	complement(67360460..67654612)	9,70	12,98	-0,198207985
TAFA1	3	68004247..68545621	0,01	0,01	-0,025382928
TAFA4	3	complement(68731766..68953297)	-	-	#N/D

EOGT	3	complement(68975217..69013684)	1,29	2,29	-0,597522013
TMF1	3	complement(69019827..69052339)	5,52	4,00	0,68600154
UBA3	3	complement(69054730..69080408)	6,38	8,57	-0,202317543
ARL6IP5	3	69084937..69106092	11,58	11,33	0,254902165
LMOD3	3	complement(69106065..69123032)	0,01	0,01	0,366665713
FRMD4B	3	complement(69168782..69542583)	0,47	1,90	-1,774973995
MITF	3	69739456..69968336	1,36	0,66	1,260794901
MDFIC2	3	complement(70194479..70312638)	-	-	#N/D
FOXP1	3	complement(70954693..71583978)	2,58	3,79	-0,329611556
gene:ENSG000	3	complement(70959226..71754229)	0,09	0,07	0,593914433
EIF4E3	3	complement(71675414..71754773)	1,03	1,09	0,142593754
GPR27	3	71753855..71756496	2,28	3,20	-0,269798013
PROK2	3	complement(71771655..71785206)	0,03	-	5,224730805
RYBP	3	complement(72371825..72446621)	1,45	2,37	-0,484097548
SHQ1	3	complement(72749277..72861914)	2,23	2,70	-0,054290452
GXYLT2	3	72888046..72998138	1,60	3,17	-0,761233651
PPP4R2	3	72996803..73069198	2,42	3,49	-0,306563708
EBLN2	3	73061659..73063337	0,78	0,86	0,08263035
PDZRN3	3	complement(73382431..73624941)	2,88	2,74	0,294397295
gene:ENSG000	3	73624410..73627626	0,16	0,23	-0,312517757
CNTN3	3	complement(74262568..74614659)	0,41	-	10,51034653
FRG2C	3	75664328..75667220	0,01	-	3,068695355
ZNF717	3	complement(75678660..75785583)	0,01	0,01	0,933337975
ROBO2	3	75906695..77649964	0,07	0,17	-1,000193896
ROBO1	3	complement(78597239..79767998)	6,98	8,10	0,00852035
GBE1	3	complement(81489703..81761645)	13,20	11,70	0,396610578
CADM2	3	84958989..86074429	0,00	0,06	-5,261110799
VGLL3	3	complement(86876388..86991149)	0,79	1,16	-0,329334278
CHMP2B	3	87227271..87255556	3,62	5,42	-0,359305909
POU1F1	3	complement(87259404..87276584)	-	-	0,188027316
HTR1F	3	87792706..87993839	-	-	#N/D
CGGBP1	3	complement(88051944..88149885)	4,02	5,64	-0,264681851
gene:ENSG000	3	complement(88058139..88058216)	-	-	#N/D
ZNF654	3	88059255..88144660	0,60	0,80	-0,185893966
C3orf38	3	88149959..88168729	1,74	2,28	-0,168135121
CSNKA2IP	3	88338416..88467594	-	-	0,188027316
EPHA3	3	89107621..89482134	0,95	0,11	3,273123071
PROS1	3	complement(93873051..93980003)	0,52	0,73	-0,276106712
ARL13B	3	93980139..94055678	1,05	1,63	-0,401285029
STX19	3	complement(94014365..94028597)	-	0,03	-4,359055975
DHFR2	3	complement(94047836..94063389)	0,51	0,85	-0,503995877
NSUN3	3	94062980..94131832	1,42	1,62	0,040224022
EPHA6	3	96814581..97761532	0,06	0,12	-0,83662739
ARL6	3	97764521..97801229	0,65	0,65	0,233012783
CRYBG3	3	97822011..97944984	1,60	2,07	-0,14833844
RIOX2	3	complement(97941818..97972457)	3,64	4,59	-0,109335088
GABRR3	3	complement(97985102..98035315)	-	-	0,188027316
OR5AC1	3	98064472..98065396	-	-	#N/D

OR5AC2	3	98087173..98088102	-	-	#N/D
OR5H1	3	98130721..98138548	-	-	#N/D
OR5H14	3	98147479..98156614	-	-	0,188027316
OR5H15	3	98166696..98169774	-	-	#N/D
OR5H6	3	98263252..98265356	-	-	#N/D
OR5H2	3	98282505..98284070	-	-	#N/D
OR5H8	3	98306752..98312843	-	-	0,188027316
OR5K4	3	98353854..98354819	-	-	#N/D
OR5K3	3	98390666..98391631	-	-	#N/D
OR5K1	3	98463201..98472924	-	-	0,188027316
OR5K2	3	98497681..98498631	0,02	-	3,967268276
CLDND1	3	complement(98497912..98523066)	10,10	10,68	0,141857503
gene:ENSG000	3	complement(98521133..98585498)	-	-	0,188027316
GPR15	3	98531978..98534681	0,05	-	6,575938476
CPOX	3	complement(98579446..98593648)	7,07	7,62	0,114313339
ST3GAL6	3	98732236..98821201	0,79	0,76	0,291642747
DCBLD2	3	complement(98795941..98901695)	38,08	34,73	0,356005946
COL8A1	3	99638475..99799226	1,33	1,05	0,567085155
CMSS1	3	99817837..100181732	2,12	3,80	-0,620503103
FILIP1L	3	complement(99828811..100114513	0,64	0,70	0,109636948
TBC1D23	3	100260992..100325251	9,15	5,81	0,878882703
NIT2	3	100334739..100361635	2,56	3,33	-0,15806236
TOMM70	3	complement(100363431..10040108	15,99	14,20	0,395015081
LNP1	3	100401532..100456319	1,03	1,48	-0,293016286
TMEM45A	3	100492619..100577444	4,75	3,62	0,61182288
ADGRG7	3	100609601..100695479	8,48	5,19	0,931284212
TFG	3	100709295..100748964	33,45	23,72	0,718994826
ABI3BP	3	complement(100749156..10099351	0,06	4,16	-5,806175244
IMPG2	3	complement(101222546..10132057	0,02	0,03	-0,182572847
SENP7	3	complement(101324205..10151324	1,19	1,19	0,215387298
TRMT10C	3	101561868..101566446	3,67	6,41	-0,582994385
PCNP	3	101574180..101594465	8,00	11,94	-0,354459985
ZBTB11	3	complement(101648889..10167713	7,34	3,64	1,232980644
RPL24	3	complement(101681091..10168673	118,00	#####	0,353507167
gene:ENSG000	3	101686827..101713301	0,44	0,35	0,548943472
CEP97	3	101724534..101770562	3,62	3,17	0,413409885
NXPE3	3	101779177..101843743	2,07	4,74	-0,971971648
NFKBIZ	3	101827991..101861022	6,60	12,54	-0,702916595
ZPLD1	3	102099244..102479841	0,02	0,01	0,627075281
ALCAM	3	105366909..105576900	14,02	10,22	0,679393467
CBLB	3	complement(105655461..10586955	2,40	1,64	0,76911603
CCDC54	3	107377439..107378635	-	-	0,188027316
BBX	3	107522936..107811339	9,54	10,71	0,056829768
CD47	3	complement(108043091..10809186	9,25	7,97	0,436858394
IFT57	3	complement(108160812..10822243	4,13	5,11	-0,083044868
HHLA2	3	108296529..108378285	0,01	-	3,967268276
MYH15	3	complement(108380368..10852932	1,15	0,35	1,951082728
CIP2A	3	complement(108549864..10858964	4,97	6,98	-0,269010361

DZIP3	3	108589705..108694840	1,39	1,88	-0,217014913
RETNLB	3	complement(108743424..10875741	-	-	0,188027316
TRAT1	3	108822770..108855005	-	-	#N/D
GUCA1C	3	complement(108907792..10895387	-	-	#N/D
MORC1	3	complement(108958248..10911813	-	-	#N/D
C3orf85	3	109118252..109151401	-	-	#N/D
DPPA2	3	complement(109293788..10931651	-	-	0,188027316
DPPA4	3	complement(109326144..10933754	-	0,01	-3,804623889
NECTIN3	3	111070071..111275563	2,26	2,25	0,226818335
CD96	3	111292719..111665750	0,00	0,01	-1,877914477
ZBED2	3	complement(111592900..11159534	0,04	0,01	1,893574746
PLCXD2	3	111674676..111846447	0,84	1,12	-0,196977614
PHLDB2	3	111732497..111976517	1,10	7,30	-2,510064758
ABHD10	3	111979010..111993368	9,58	10,13	0,142246905
TAGLN3	3	111998739..112013887	0,01	0,01	-0,01185201
TMPRSS7	3	112034736..112081269	0,01	-	3,967268276
C3orf52	3	112086335..112131004	1,13	1,93	-0,549471352
GCSAM	3	complement(112120839..11213327	0,29	0,35	-0,040557372
SLC9C1	3	complement(112140898..11229422	-	0,02	-5,327865481
CD200	3	112332235..112363181	0,00	-	3,068695355
BTLA	3	complement(112463966..11249947	0,01	-	4,913749004
gene:ENSG000	3	complement(112525548..11252814	0,05	0,05	0,221705268
ATG3	3	complement(112532510..11256204	6,09	5,90	0,268084276
SLC35A5	3	112561709..112585579	3,60	4,11	0,032266455
CCDC80	3	complement(112596797..11264953	3,99	4,87	-0,065025083
CD200R1L	3	complement(112815709..11284686	-	-	0,188027316
CD200R1	3	complement(112921205..11297510	0,04	0,01	1,735012788
GTPBP8	3	112990984..113015060	0,80	1,35	-0,530446186
NEPRO	3	complement(113002444..11301986	4,69	4,15	0,398909608
BOC	3	113211003..113287459	1,55	1,06	0,767868951
CFAP44	3	complement(113286930..11344161	0,04	0,29	-2,51055936
gene:ENSG000	3	complement(113361901..11351515	0,56	0,81	-0,307800806
SPICE1	3	complement(113442718..11351518	3,43	2,35	0,769086627
SIDT1	3	113532555..113629575	0,10	0,04	1,59879334
USF3	3	complement(113648385..11369664	1,71	1,98	0,006412963
NAA50	3	complement(113716458..11374630	10,74	12,81	-0,030176933
ATP6V1A	3	113746770..113812056	17,41	10,85	0,905512652
GRAMD1C	3	113828182..113947174	0,59	0,53	0,352784409
ZDHHC23	3	113947901..113965401	1,89	2,13	0,051844367
CCDC191	3	complement(113964137..11405659	1,01	0,90	0,383442927
QTRT2	3	114005833..114088422	4,44	5,35	-0,044142638
DRD3	3	complement(114127580..11419940	0,01	0,00	1,436225396
ZNF80	3	complement(114234631..11423757	0,01	-	4,913749003
TIGIT	3	114276913..114310288	0,01	0,01	-0,429837071
ZBTB20	3	complement(114314500..11514729	1,53	1,33	0,422759299
GAP43	3	115623510..115721490	0,07	0,46	-2,527458649
LSAMP	3	complement(115802374..11713938	0,09	0,09	0,198563439
IGSF11	3	complement(118900557..11914606	0,01	-	4,913749003

TEX55	3	119146151..119160042	0,01	-	3,068695355
gene:ENSG000	3	119147375..119187807	-	-	#N/D
UPK1B	3	119173517..119205143	0,01	0,02	-0,579815015
B4GALT4	3	complement(119211732..11924094	4,25	3,57	0,471523456
ARHGAP31	3	119294383..119420714	4,18	3,99	0,291291148
TMEM39A	3	complement(119428949..11946883	20,82	11,41	1,090934293
POGLUT1	3	119468963..119494708	1,34	3,28	-1,064750239
TIMMDC1	3	119498547..119525090	17,50	21,27	-0,058222518
CD80	3	complement(119524293..11955961	0,03	0,02	0,750935198
ADPRH	3	119579268..119589945	-	0,03	-6,312013662
PLA1A	3	119597875..119629811	-	0,03	-5,071123353
POPDC2	3	complement(119636457..11966532	0,01	0,15	-3,524780994
COX17	3	complement(119654513..11967745	3,65	3,40	0,325142013
CFAP91	3	119703022..119767102	0,13	0,04	1,923488028
gene:ENSG000	3	119703076..119807570	-	-	0,188027316
NR1I2	3	119780484..119818487	0,00	-	3,068695355
GSK3B	3	complement(119821321..12009499	6,27	8,75	-0,258354336
GPR156	3	complement(120164645..12028522	0,74	1,51	-0,806799421
LRRC58	3	complement(120324509..12034935	4,25	4,88	0,021531736
FSTL1	3	complement(120392293..12045101	89,15	#####	-0,238670328
NDUFB4	3	120596328..120602507	27,53	23,99	0,421795147
HGD	3	complement(120628172..12068226	0,04	0,03	0,438394362
RABL3	3	complement(120684938..12074299	3,40	3,22	0,298086854
GTF2E1	3	120742637..120783069	3,79	4,83	-0,12619366
STXBP5L	3	120908072..121424761	0,01	0,05	-2,024811859
POLQ	3	complement(121431431..12154598	2,47	2,99	-0,047945116
ARGFX	3	121567949..121590622	0,00	0,01	-0,579815015
FBXO40	3	121593379..121630295	0,00	0,00	0,886720911
HCLS1	3	complement(121631399..12166092	0,04	0,23	-2,227495745
GOLGB1	3	complement(121663199..12174996	6,87	6,44	0,316998625
IQCB1	3	complement(121769761..12183507	2,68	3,57	-0,18771636
EAF2	3	121835183..121886526	0,45	0,06	3,153671623
SLC15A2	3	121894401..121944188	0,08	0,01	2,633477237
ILDR1	3	complement(121987323..12202224	-	-	#N/D
CD86	3	122055362..122121139	-	-	#N/D
CASR	3	122183668..122291629	-	-	0,188027316
CSTA	3	122325248..122341969	-	0,20	-7,112521263
MIX23	3	complement(122359591..12238323	7,86	10,41	-0,182182842
FAM162A	3	122384161..122412334	4,66	3,00	0,859642823
WDR5B	3	complement(122411846..12241606	3,04	2,46	0,528353807
KPNA1	3	complement(122421902..12251494	12,18	11,62	0,290798611
PARP9	3	complement(122527924..12256457	1,85	1,44	0,580965278
DTX3L	3	122564338..122575203	6,37	6,29	0,240736053
PARP15	3	122577628..122639047	0,01	0,01	-0,345401666
PARP14	3	122680839..122730840	5,18	5,27	0,199546133
HSPBAP1	3	complement(122739999..12279383	0,97	1,49	-0,401198537
SLC49A4	3	122795069..122881139	2,68	3,22	-0,039097479
SEMA5B	3	complement(122909082..12302860	-	0,00	-2,89252005

PDIA5	3	123067025..123225227	9,35	9,32	0,228783421
SEC22A	3	123201927..123274136	4,63	2,72	0,988809191
ADCY5	3	complement(123282296..12344909	0,18	0,05	2,010071359
HACD2	3	complement(123490820..12358505	8,21	11,23	-0,229462296
MYLK	3	complement(123610049..12388433	2,08	2,61	-0,106068192
CCDC14	3	complement(123897305..12396140	2,29	3,54	-0,404190463
ROPN1	3	complement(123968521..12399217	0,01	0,00	0,886720911
KALRN	3	124033369..124726325	0,03	0,01	1,91355908
UMPS	3	124730433..124749273	3,34	3,74	0,06071259
ITGB5	3	complement(124761948..12490141	59,61	44,43	0,647086235
gene:ENSG000	3	complement(124791119..12479310	0,43	0,46	0,151832325
MUC13	3	complement(124905442..12495381	0,02	0,10	-1,950575847
HEG1	3	complement(124965710..12505599	34,39	29,55	0,441511859
SLC12A8	3	complement(125082636..12521286	2,14	2,51	-0,009900705
ZNF148	3	complement(125225669..12537532	3,36	3,36	0,223016009
SNX4	3	complement(125446650..12552020	1,27	3,15	-1,086009109
OSBPL11	3	complement(125528858..12559549	5,11	5,43	0,134271667
ROPN1B	3	125969160..125983454	0,01	0,00	1,833201638
SLC41A3	3	complement(126006357..12610156	7,13	6,14	0,438534927
ALDH1L1	3	complement(126103562..12619799	0,10	0,02	2,714070677
KLF15	3	complement(126342635..12635740	0,06	0,21	-1,685373637
CFAP100	3	126394909..126436556	0,01	0,03	-0,742377908
gene:ENSG000	3	complement(126432796..12643456	0,33	0,39	-0,040268748
ZXDC	3	complement(126437601..12647589	9,92	6,85	0,757149236
UROC1	3	complement(126481166..12651777	0,01	0,00	1,833201638
CHST13	3	126524155..126543291	-	-	#N/D
C3orf22	3	complement(126526999..12655896	-	0,01	-2,89252005
TXNRD3	3	complement(126571779..12665512	3,47	3,62	0,16297984
CHCHD6	3	126704240..126960420	12,85	11,36	0,400899947
gene:ENSG000	3	126973065..126976426	0,08	0,07	0,513216292
PLXNA1	3	126982693..127037389	27,19	36,06	-0,184242461
PRR23E	3	127193131..127198185	-	-	#N/D
PRR20G	3	complement(127283783..12728804	0,04	-	4,913749003
TPRA1	3	complement(127571232..12759826	17,03	13,03	0,609291445
MCM2	3	127598223..127622436	35,29	35,79	0,202678626
PODXL2	3	127629185..127672802	23,06	30,31	-0,171253531
ABTB1	3	127672935..127680926	10,19	8,54	0,47795442
MGLL	3	complement(127689062..12805219	26,42	19,34	0,672577235
KBTBD12	3	127915232..127987671	0,01	0,00	1,232079601
SEC61A1	3	128051641..128071705	125,13	61,90	1,238613445
RUVBL1	3	complement(128064778..12815391	22,84	26,05	0,03359983
EEFSEC	3	128153481..128408646	3,74	5,32	-0,284851449
DNAJB8	3	complement(128462437..12846696	0,02	0,02	0,438394362
GATA2	3	complement(128479427..12849320	2,28	3,21	-0,272570331
RPN1	3	complement(128619969..12868107	172,22	#####	0,627872669
RAB7A	3	128693669..128825942	25,53	28,43	0,067473441
ACAD9	3	128879596..128924003	4,83	4,47	0,335910787
CFAP92	3	complement(128909866..12900269	0,48	0,30	0,902256919

gene:ENSG000	3	complement(128914833..12891506	-	-	#N/D
EFCC1	3	129001304..129040742	0,02	0,02	-0,034419864
GP9	3	129060779..129062406	-	-	0,188027316
RAB43	3	complement(129087569..12912280	30,39	11,26	1,655636337
ISY1-RAB43	3	complement(129087575..12916103	5,73	3,40	0,97868959
ISY1	3	complement(129127415..12916106	5,76	4,96	0,43899538
CNBP	3	complement(129167827..12918392	63,47	59,96	0,304980772
COPG1	3	129249606..129277773	126,95	71,75	1,046334631
HMCES	3	129278828..129306186	13,95	14,05	0,212487389
H1-10	3	complement(129314771..12931628	22,66	43,72	-0,724965329
EFCAB12	3	complement(129401321..12942863	0,10	0,09	0,299198032
IFT122	3	129429607..129520510	4,18	3,30	0,561534388
MBD4	3	complement(129430947..12944017	8,76	6,27	0,70584073
RHO	3	129528639..129535344	-	-	0,188027316
H1-8	3	129543175..129551467	-	-	#N/D
PLXND1	3	complement(129555214..12960667	16,64	16,89	0,20155817
TMCC1	3	complement(129647792..12989371	2,91	3,37	0,012122324
TRH	3	129974688..129977935	0,02	0,01	1,436225396
ALG1L2	3	130081831..130113227	0,05	0,05	0,278120973
COL6A5	3	130345516..130484846	-	-	#N/D
COL6A6	3	130517177..130677044	0,05	0,05	0,214261684
PIK3R4	3	complement(130678934..13074682	9,01	8,15	0,367402608
ATP2C1	3	130850595..131016712	6,76	9,12	-0,209051765
gene:ENSG000	3	complement(130868004..13086935	0,13	0,13	0,152110417
ASTE1	3	complement(131013875..13102764	1,68	1,57	0,325502247
NEK11	3	131026850..131350465	0,41	0,50	-0,081249298
NUDT16	3	131381671..131388830	3,58	2,39	0,807680593
MRPL3	3	complement(131462212..13150298	24,02	35,35	-0,334322538
CPNE4	3	complement(131533555..13228541	0,02	0,02	0,728122296
ACP3	3	132317369..132368302	0,60	0,14	2,284375045
DNAJC13	3	132417502..132539032	11,08	8,78	0,55811588
ACAD11	3	complement(132558138..13266008	7,10	7,26	0,191101352
NPHP3-ACAD1	3	complement(132558142..13272245	0,48	0,56	0,010116986
ACKR4	3	132597270..132618967	0,26	0,15	1,07164464
UBA5	3	132654446..132679794	1,99	1,90	0,290263239
NPHP3	3	complement(132680609..13272243	2,14	1,58	0,663118388
TMEM108	3	133038391..133397775	0,28	0,10	1,760985954
BFSP2	3	133400056..133475222	0,01	-	3,967268276
CDV3	3	133573686..133590261	30,85	29,84	0,27107576
TOPBP1	3	complement(133598175..13366238	12,52	10,80	0,435828003
TF	3	133746040..133796641	0,04	0,03	0,760732555
SRPRB	3	133784023..133825772	22,20	12,82	1,014854346
RAB6B	3	complement(133824235..13389588	5,42	3,02	1,065240975
SLCO2A1	3	complement(133932701..13405218	0,08	0,14	-0,605862664
RYK	3	complement(134065303..13425085	15,67	15,40	0,248224032
AMOTL2	3	complement(134355347..13437547	28,87	26,06	0,370655477
ANAPC13	3	complement(134477706..13448671	19,61	15,79	0,536031685
CEP63	3	134485699..134587789	0,88	1,09	-0,098072933

KY	3	complement(134599923..13465163	0,02	0,02	-0,35396495
EPHB1	3	134795260..135260467	0,06	0,04	0,87260107
PPP2R3A	3	135965728..136147894	4,39	2,85	0,847273864
MSL2	3	complement(136148917..13619724	5,97	6,57	0,085718133
PCCB	3	136250340..136337896	5,44	6,78	-0,095997209
STAG1	3	complement(136336236..13675240	2,56	2,34	0,355529539
SLC35G2	3	136818647..136855888	3,24	4,94	-0,384713161
NCK1	3	136862208..136951606	3,63	2,92	0,534873832
IL20RB	3	136946230..137011085	3,94	5,24	-0,186605589
SOX14	3	137764315..137766334	-	-	#N/D
CLDN18	3	137998735..138033655	0,28	0,30	0,130973079
DZIP1L	3	complement(138061990..13811581	3,02	3,27	0,11047039
A4GNT	3	complement(138123713..13813239	0,06	0,04	0,686693064
DBR1	3	complement(138160988..13817494	2,93	4,81	-0,49137977
ARMC8	3	138187248..138298384	2,07	3,35	-0,472220773
NME9	3	complement(138261437..13832988	0,08	0,11	-0,168615427
MRAS	3	138347648..138405534	3,21	3,49	0,101506996
ESYT3	3	138434586..138481686	0,30	0,49	-0,478316965
gene:ENSG000	3	138482065..138485755	0,08	0,16	-0,890449674
CEP70	3	complement(138494344..13859453	2,12	3,09	-0,322967173
FAIM	3	138608606..138633376	0,28	0,92	-1,475060794
PIK3CB	3	complement(138652698..13883492	7,74	7,96	0,184079838
FOXL2	3	complement(138944224..13894713	0,00	0,01	-1,478387936
FOXL2NB	3	138947217..138953990	0,01	0,07	-2,386799575
PRR23A	3	complement(139003962..13900626	-	0,01	-3,804623889
MRPS22	3	139005806..139360497	5,14	5,48	0,13090973
PRR23B	3	complement(139019031..13902092	-	-	0,188027316
PRR23C	3	complement(139042102..13904489	-	-	0,188027316
COPB2	3	complement(139353946..13938973	46,31	37,09	0,543491195
RBP2	3	complement(139452884..13948074	0,05	0,02	1,833201638
RBP1	3	complement(139517434..13953982	0,03	0,02	0,686693064
NMNAT3	3	complement(139560180..13967801	0,10	0,02	2,324847179
CLSTN2	3	139935185..140577397	0,02	0,00	3,398899791
TRIM42	3	140678064..140701150	-	-	#N/D
SLC25A36	3	140941830..140980995	2,43	4,06	-0,517779478
SPSB4	3	141051347..141148611	0,13	0,05	1,420083537
PXYLP1	3	141228726..141367753	0,31	0,58	-0,649810963
ZBTB38	3	141324213..141449792	11,41	21,89	-0,716798028
RASA2	3	141487027..141615344	2,39	4,08	-0,545207836
RNF7	3	141738249..141747560	10,95	12,80	-0,001892673
gene:ENSG000	3	141738474..141807919	-	-	0,188027316
GRK7	3	141763408..141819352	0,05	0,05	0,341596824
ATP1B3	3	141876124..141926549	22,16	29,85	-0,206784509
gene:ENSG000	3	complement(141936707..14193854	0,02	0,03	-0,032860829
TFDP2	3	complement(141944428..14214954	1,61	3,71	-0,978606085
GK5	3	complement(142157527..14222559	1,35	2,49	-0,661263565
XRN1	3	complement(142306607..14244806	3,17	4,86	-0,39588867
ATR	3	complement(142449007..14257873	2,79	4,81	-0,561977474

PLS1	3	142596393..142713664	0,48	1,39	-1,301027264
TRPC1	3	142724034..142807888	1,81	3,25	-0,619426882
PCOLCE2	3	complement(142815922..14288920	13,89	16,11	0,009212532
PAQR9	3	complement(142949164..14296367	0,18	0,15	0,429629179
U2SURP	3	142964497..143060725	5,49	13,18	-1,040347013
CHST2	3	143119771..143124014	0,13	1,25	-3,008359679
SLC9A9	3	complement(143265222..14384848	0,38	0,44	0,017263614
DIPK2A	3	143971823..144048719	1,43	1,93	-0,20358885
gene:ENSG000	3	complement(144442211..14444584	0,01	-	4,516772761
PLOD2	3	complement(146035139..14616372	13,84	13,09	0,303737548
PLSCR4	3	complement(146192335..14625117	15,78	6,16	1,579652971
PLSCR2	3	complement(146391421..14649599	0,03	0,01	1,487770062
gene:ENSG000	3	complement(146504570..14650656	0,09	0,05	1,138491112
PLSCR1	3	complement(146515180..14654485	9,22	10,29	0,064057725
PLSCR5	3	complement(146576555..14660534	-	-	0,188027316
ZIC4	3	complement(147386046..14740680	0,73	0,80	0,088317343
ZIC1	3	147393422..147510293	3,24	4,58	-0,277252418
AGTR1	3	148697784..148743008	0,07	0,20	-1,218904208
CPB1	3	148791102..148860187	-	-	#N/D
CPA3	3	148865296..148897203	-	0,03	-4,758582517
GYG1	3	148991408..149031775	5,57	8,47	-0,380547471
HLTF	3	complement(149030127..14908655	13,05	19,62	-0,364535182
HPS3	3	149129638..149173732	10,88	10,18	0,319043829
CP	3	complement(149162410..14922182	9,77	4,46	1,354969038
TM4SF18	3	complement(149318498..14933441	0,40	0,00	6,93907576
TM4SF1	3	complement(149369022..14937769	22,07	17,99	0,517793545
TM4SF4	3	149474697..149503394	0,01	0,04	-2,190455314
WWTR1	3	complement(149517235..14973671	19,13	14,34	0,638874639
COMMD2	3	complement(149738472..14975249	5,44	9,18	-0,531175805
ANKUB1	3	complement(149761100..14996838	-	0,01	-3,804623889
RNF13	3	149812770..149962139	5,35	7,85	-0,328903816
PFN2	3	complement(149964904..15005078	40,01	60,70	-0,378021388
TSC22D2	3	150408298..150466422	5,89	4,29	0,679939495
SERP1	3	complement(150541998..15060322	18,38	17,02	0,33405645
EIF2A	3	150546678..150586016	22,47	24,46	0,100667542
SELENOT	3	150602875..150630436	7,35	11,37	-0,407356546
ERICH6	3	complement(150659885..15070397	0,04	0,01	1,893574746
SIAH2	3	complement(150741125..15076347	19,98	14,75	0,660433594
MINDY4B	3	complement(150870376..15090543	0,01	0,02	-0,579815015
gene:ENSG000	3	complement(150882997..15094174	-	-	0,188027316
CLRN1	3	complement(150926163..15097272	-	0,00	-2,89252005
MED12L	3	151085286..151437072	0,24	0,04	2,932911641
GPR171	3	complement(151197832..15120321	0,02	-	4,913749004
P2RY14	3	complement(151212117..15127854	0,00	-	3,068695355
GPR87	3	complement(151294086..15131682	0,19	-	7,621425725
P2RY13	3	complement(151326312..15132954	0,01	-	4,516772761
P2RY12	3	complement(151336843..15138475	0,00	-	3,068695355
IGSF10	3	complement(151425384..15146106	0,35	0,08	2,344956929

AADACL2	3	151733916..151761339	0,00	0,01	-0,923955849
AADAC	3	151814073..151828488	0,07	-	6,20258586
SUCNR1	3	151873643..151884619	0,10	0,01	4,193099983
MBNL1	3	152243828..152465780	32,09	21,74	0,785286556
P2RY1	3	152835131..152841439	0,21	1,34	-2,447762159
RAP2B	3	153162226..153170627	4,18	4,71	0,050637566
ARHGEF26	3	154121003..154257827	0,36	1,08	-1,357197721
DHX36	3	complement(154272546..15432448	2,87	5,01	-0,582623037
gene:ENSG000	3	complement(154334809..15433704	-	-	#N/D
GPR149	3	complement(154334943..15443019	-	-	0,188027316
MME	3	155024124..155183704	0,46	1,15	-1,083818926
STRIT1	3	complement(155290227..15529368	0,01	-	3,068695355
PLCH1	3	complement(155375580..15574507	0,04	0,23	-2,187832817
C3orf33	3	complement(155762617..15580627	0,62	1,24	-0,779353548
gene:ENSG000	3	complement(155763043..15585444	0,37	0,13	1,659232506
SLC33A1	3	complement(155821024..15585445	4,80	3,86	0,538419916
GMPS	3	155870650..155944020	11,96	16,59	-0,248622853
KCNAB1	3	156037701..156538756	0,52	0,87	-0,516786006
SSR3	3	complement(156539553..15655514	54,23	74,46	-0,234326831
TIPARP	3	156673235..156706770	20,77	15,37	0,657384221
LEKR1	3	156825481..157046129	0,11	0,29	-1,122244492
CCNL1	3	complement(157146508..15716076	9,11	9,13	0,219998835
VEPH1	3	complement(157259742..15753361	0,52	4,37	-2,856479229
PTX3	3	157436850..157443633	28,94	#####	-1,827557205
SHOX2	3	complement(158095905..15810642	1,92	4,17	-0,896959687
RSRC1	3	158105855..158545730	4,11	7,89	-0,717654153
MLF1	3	158571163..158607252	4,11	6,52	-0,441310498
GFM1	3	158644527..158695581	3,80	6,48	-0,548608608
LXN	3	complement(158645822..15867264	0,04	0,02	1,232079601
RARRES1	3	complement(158696892..15873248	0,10	0,11	0,129542238
MFSD1	3	158732198..158829719	3,29	3,39	0,18064429
gene:ENSG000	3	158869898..158871821	0,21	0,05	2,150707884
IQCJ-SCHIP1	3	158962235..159897358	1,51	1,54	0,195897487
IQCJ	3	158962928..159266307	0,03	0,03	0,370333154
SCHIP1	3	159273244..159897629	1,93	1,91	0,237006493
IL12A	3	159988835..159996019	0,84	1,56	-0,675560267
C3orf80	3	160225422..160228213	0,19	0,25	-0,150965276
TRIM59-IFT80	3	complement(160227454..16044982	-	0,04	-6,052163584
IFT80	3	complement(160256986..16039988	1,59	3,00	-0,698223892
SMC4	3	160399274..160434954	13,97	19,22	-0,237395585
TRIM59	3	complement(160432445..16048577	0,82	0,61	0,643386476
KPNA4	3	complement(160495007..16056557	8,10	13,78	-0,544351278
ARL14	3	160677160..160678448	-	0,02	-3,804623889
PPM1L	3	160755602..161078902	0,07	0,44	-2,478405345
B3GALNT1	3	complement(161083883..16110541	1,06	1,63	-0,392727095
NMD3	3	161104696..161253532	7,95	11,92	-0,361040557
SPTSSB	3	complement(161344798..16137288	0,01	0,03	-1,955757882
OTOL1	3	161496808..161503942	-	-	#N/D

SI	3	complement(164978898..16507849	-	-	#N/D
SLITRK3	3	complement(165186720..16519710	0,01	0,17	-3,550672619
BCHE	3	complement(165772904..16583746	0,01	0,60	-6,093005473
ZBBX	3	complement(167239843..16740787	0,00	0,00	-0,01185201
SERPINI2	3	complement(167441914..16747897	-	0,01	-3,804623889
WDR49	3	complement(167478684..16765398	0,05	0,06	0,129542238
PDCD10	3	complement(167683298..16773493	2,58	3,92	-0,380285621
SERPINI1	3	167735243..167825569	16,20	3,23	2,550018036
GOLIM4	3	complement(168008689..16809592	20,57	12,19	0,978173015
MECOM	3	complement(169083499..16966377	0,33	1,83	-2,257191814
ACTRT3	3	complement(169766921..16976956	0,45	0,60	-0,216956919
MYNN	3	169773396..169789716	3,02	2,87	0,300074959
LRRC34	3	complement(169793003..16981298	0,58	1,00	-0,573213779
LRRIQ4	3	169812870..169837773	0,01	0,06	-1,997339153
LRRC31	3	complement(169839172..16986993	0,01	-	3,967268276
SAMD7	3	169911572..169939175	-	-	#N/D
SEC62	3	169966635..169998373	5,94	7,46	-0,104305474
GPR160	3	170037995..170085392	0,11	0,43	-1,791789098
PHC3	3	complement(170086732..17018174	5,74	6,04	0,147318607
PRKCI	3	170222424..170305977	6,22	10,01	-0,462269831
SKIL	3	170357342..170396835	5,18	7,93	-0,391661797
CLDN11	3	170418868..170454733	2,29	4,55	-0,768755165
gene:ENSG000	3	170418868..170860380	0,11	0,15	-0,152395412
SLC7A14	3	complement(170459548..17058607	-	-	0,188027316
RPL22L1	3	complement(170864875..17087020	3,22	7,58	-1,010951438
EIF5A2	3	complement(170888418..17090864	1,97	3,22	-0,487592008
SLC2A2	3	complement(170996347..17102674	-	0,00	-2,89252005
TNIK	3	complement(171058414..17146040	0,15	0,69	-2,013816183
gene:ENSG000	3	171460516..171462256	0,02	0,41	-3,770173478
PLD1	3	complement(171600404..17181095	1,29	3,00	-0,993568691
TMEM212	3	171843349..171938715	-	0,01	-3,804623889
FNDC3B	3	172039578..172401669	22,93	29,09	-0,120365974
gene:ENSG000	3	complement(172301654..17230305	0,47	0,63	-0,196738859
GHSR	3	complement(172443291..17244845	-	-	0,188027316
TNFSF10	3	complement(172505508..17252347	0,17	0,27	-0,464572644
NCEH1	3	complement(172630249..17271121	19,08	15,75	0,500355476
ECT2	3	172750682..172821474	11,97	20,61	-0,560804741
SPATA16	3	complement(172889357..17314123	-	-	0,188027316
NLGN1	3	173396284..174294372	0,14	1,01	-2,58401435
NAALADL2	3	174438573..175810548	0,38	0,27	0,721550307
TBL1XR1	3	complement(177019340..17722800	11,09	14,05	-0,117887129
KCNMB2	3	178272932..178844429	0,04	-	6,575938476
gene:ENSG000	3	178419123..178843300	-	0,04	-4,758582517
ZMAT3	3	complement(178960121..17907221	2,24	2,24	0,218585506
PIK3CA	3	179148114..179240093	2,12	3,35	-0,440400291
KCNMB3	3	complement(179236691..17926700	0,10	0,12	-0,094144596
ZNF639	3	179323031..179338583	3,35	4,70	-0,262839668
MFN1	3	179347709..179394936	5,70	6,11	0,124224687

GNB4	3	complement(179396088..17945147	7,62	7,91	0,169019054
ACTL6A	3	179562886..179588407	19,76	28,38	-0,299290832
MRPL47	3	complement(179588285..17960464	16,33	24,99	-0,391111266
NDUFB5	3	179604690..179627647	5,81	8,41	-0,309017801
gene:ENSG000	3	179604807..179784179	2,87	5,53	-0,723959837
USP13	3	179653032..179804366	3,94	7,25	-0,657155125
PEX5L	3	complement(179794958..18003705	0,02	0,23	-3,46253132
TTC14	3	180602163..180617828	3,26	3,10	0,294238224
CCDC39	3	complement(180602858..18068494	0,01	0,01	-0,034419864
FXR1	3	180868141..180982753	9,95	12,63	-0,120960764
DNAJC19	3	complement(180983697..18098977	3,23	4,03	-0,097158952
SOX2	3	181711925..181714436	0,01	-	3,967268276
ATP11B	3	182793503..182921629	3,98	5,74	-0,306617002
DCUN1D1	3	complement(182938074..18298595	1,48	2,73	-0,654063215
MCCC1	3	complement(183015218..18311607	2,50	4,05	-0,474259331
LAMP3	3	complement(183122215..18316383	0,05	0,75	-3,802455491
MCF2L2	3	complement(183178041..18342877	0,13	0,85	-2,457895415
B3GNT5	3	183253253..183298504	1,84	2,92	-0,438529402
KLHL6	3	complement(183487551..18355570	-	-	0,188027316
KLHL24	3	183635610..183684519	2,52	3,11	-0,084934697
YEATS2	3	183697797..183812624	14,65	22,95	-0,423854341
gene:ENSG000	3	complement(183815568..18388488	0,26	1,24	-2,004462034
MAP6D1	3	complement(183815922..18382559	0,24	0,19	0,605197342
PARL	3	complement(183829271..18388493	15,14	20,61	-0,222025933
ABCC5	3	complement(183919934..18401793	4,00	7,02	-0,589575374
HTR3D	3	184031544..184039369	-	-	#N/D
HTR3C	3	184053047..184060673	-	-	#N/D
HTR3E	3	184097064..184106995	0,01	0,01	-0,025382928
EIF2B5	3	184135038..184146127	13,38	19,06	-0,286915212
DVL3	3	184155377..184173614	20,43	26,86	-0,172176255
AP2M1	3	184174689..184184214	124,46	#####	-0,146790381
ABCF3	3	184186095..184194012	16,39	19,92	-0,058405584
VWA5B2	3	184229585..184242329	0,32	0,21	0,81963172
ALG3	3	complement(184242301..18424954	29,80	46,44	-0,416811402
EEF1AKMT4	3	184249672..184258759	17,89	14,90	0,487223697
EEF1AKMT4-EC	3	184249695..184293031	0,02	0,01	1,487770062
CAMK2N2	3	complement(184259213..18426155	3,48	6,38	-0,654119861
ECE2	3	184276011..184293031	0,02	0,00	2,399873901
PSMD2	3	184299198..184309050	136,13	#####	-0,634322074
EIF4G1	3	184314495..184335358	96,43	#####	-0,279885136
FAM131A	3	184335926..184348421	3,41	4,26	-0,097601195
CLCN2	3	complement(184346185..18436165	0,97	1,90	-0,745826695
POLR2H	3	184361710..184368596	13,64	18,86	-0,244754163
THPO	3	complement(184371935..18438196	0,00	0,04	-3,021611439
CHRD	3	184380054..184390736	0,03	0,28	-2,965760829
EPHB3	3	184561785..184582408	1,85	0,81	1,414662145
MAGEF1	3	complement(184710364..18471206	16,51	30,42	-0,658117552
VPS8	3	184812143..185052614	2,57	4,35	-0,535945326

C3orf70	3	complement(185076838..18515306	-	-	0,188027316
EHHADH	3	complement(185190624..18528199	1,02	1,46	-0,29392706
MAP3K13	3	185282941..185489094	1,28	1,16	0,36608288
TMEM41A	3	complement(185476496..18549905	3,46	6,35	-0,65398649
LIPH	3	complement(185506262..18555258	0,02	0,27	-3,646670376
SENP2	3	185582496..185633551	4,88	5,29	0,105810977
IGF2BP2	3	complement(185643130..18582504	31,78	53,19	-0,519993488
TRA2B	3	complement(185914558..18593810	11,94	15,50	-0,153656473
ETV5	3	complement(186046314..18611031	32,98	42,22	-0,13332382
DGKG	3	complement(186105668..18636223	0,85	1,62	-0,714754254
CRYGS	3	complement(186538441..18654670	0,19	0,39	-0,842464336
TBCCD1	3	complement(186546067..18657054	3,04	3,88	-0,129446691
DNAJB11	3	186567403..186585800	10,93	9,02	0,501293389
gene:ENSG000	3	186581995..186597103	-	-	#N/D
AHSG	3	186613060..186621318	-	-	0,188027316
FETUB	3	186635969..186653141	-	-	#N/D
HRG	3	186660216..186678234	-	-	#N/D
KNG1	3	186717348..186744410	-	-	#N/D
EIF4A2	3	186783205..186789897	81,64	87,37	0,125214745
RFC4	3	complement(186789880..18680705	12,14	19,69	-0,474910086
ADIPOQ	3	186842704..186858463	-	0,00	-2,89252005
ST6GAL1	3	186930325..187078553	1,56	3,88	-1,090938964
RPL39L	3	complement(187120948..18718090	4,32	6,50	-0,366811934
RTP1	3	187197486..187201462	-	0,01	-2,89252005
MASP1	3	complement(187217282..18729198	0,05	0,06	-0,283147548
RTP4	3	187368385..187372076	0,06	0,04	0,627075281
gene:ENSG000	3	187383101..187384849	-	0,01	-3,804623889
SST	3	complement(187668912..18767039	-	-	#N/D
RTP2	3	complement(187698259..18770255	0,02	-	4,516772761
BCL6	3	complement(187721377..18774572	4,51	7,07	-0,426679265
LPP	3	188153284..188890671	5,31	7,67	-0,306661224
FLJ42393	3	188178543..188180812	0,54	0,61	0,038027092
TPRG1	3	188947214..189325304	0,02	0,15	-2,404153325
TP63	3	189631389..189897276	0,10	2,66	-4,504128045
P3H2	3	complement(189956728..19012243	69,99	47,11	0,794295094
CLDN1	3	complement(190305707..19032244	0,44	2,62	-2,357342101
CLDN16	3	190322541..190412138	0,07	0,10	-0,313021381
TMEM207	3	complement(190428655..19044990	-	-	#N/D
IL1RAP	3	190514051..190659750	1,17	1,56	-0,200579894
GMNC	3	complement(190852737..19089242	0,01	0,00	2,144183439
OSTN	3	191199241..191265615	0,01	0,01	0,524121557
UTS2B	3	complement(191267168..19133052	0,07	0,14	-0,741496626
CCDC50	3	191329085..191398659	7,96	10,39	-0,160750184
PYDC2	3	191461163..191461456	-	-	0,188027316
FGF12	3	complement(192139390..19276776	0,36	1,49	-1,810421434
MB21D2	3	complement(192796815..19291785	2,21	5,96	-1,208540654
PLAAT1	3	193241128..193277738	0,20	2,03	-3,087941368
ATP13A5	3	complement(193274789..19337882	-	0,01	-3,804623889

ATP13A4	3	complement(193398967..19359311	0,01	0,01	-1,176557791
OPA1	3	193593144..193697811	5,69	12,55	-0,916887359
HES1	3	194136148..194138732	3,69	7,76	-0,849168938
CPN2	3	complement(194339768..19435132	0,01	0,01	-0,025382928
LRRC15	3	complement(194355249..19436974	0,02	0,03	-0,446980808
GP5	3	complement(194394821..19439926	0,00	0,00	-0,01185201
ATP13A3	3	complement(194402672..19449836	17,60	20,60	-0,003743277
TMEM44	3	complement(194587673..19463368	6,34	6,69	0,14448645
LSG1	3	complement(194640791..19467246	10,85	14,69	-0,214506405
FAM43A	3	194685883..194689037	5,33	13,60	-1,126463796
XXYLT1	3	complement(195068284..19527115	7,41	11,43	-0,401919722
ACAP2	3	complement(195274745..19544304	7,56	7,82	0,173548051
PPP1R2	3	complement(195514428..19554338	5,59	7,48	-0,197073617
APOD	3	complement(195568705..19558403	0,05	0,02	1,487770062
MUC20	3	195720884..195741123	0,04	0,07	-0,513530912
MUC4	3	complement(195746765..19581197	0,00	0,00	0,366665713
TNK2	3	complement(195863364..19591194	10,32	10,39	0,214054519
gene:ENSG000	3	complement(195908695..19590956	-	0,10	-5,545746556
TFRC	3	complement(196012511..19608215	18,04	53,69	-1,35067347
ZDHHC19	3	complement(196197452..19621143	0,01	0,01	-0,01185201
SLC51A	3	196211487..196243178	0,22	0,13	0,956365906
PCYT1A	3	complement(196214222..19628795	8,57	9,84	0,023049665
gene:ENSG000	3	complement(196248230..19631822	1,90	0,64	1,791915116
DYNLT2B	3	complement(196291219..19631829	2,35	3,16	-0,203197293
TM4SF19-DYNL	3	complement(196316082..19633837	0,30	-	7,621425725
TM4SF19	3	complement(196319342..19633850	3,65	9,61	-1,175717056
UBXN7	3	complement(196347662..19643243	8,65	9,86	0,034712534
RNF168	3	complement(196468783..19650376	13,70	10,54	0,601238131
SMCO1	3	complement(196506879..19651534	0,01	0,06	-1,955757882
WDR53	3	complement(196554177..19656867	4,42	5,23	-0,019146739
FBXO45	3	196568611..196589059	3,25	4,59	-0,276114412
NRROS	3	196639694..196662004	0,03	0,03	0,438394362
PIGX	3	196639775..196736007	2,88	4,23	-0,327807865
CEP19	3	complement(196706277..19671225	0,84	1,18	-0,262913248
PAK2	3	196739857..196832647	16,20	22,61	-0,257538704
SENP5	3	196867856..196934714	12,07	11,75	0,262624837
NCBP2	3	complement(196935402..19694259	10,85	12,06	0,07048964
NCBP2AS2	3	196942674..196943543	12,68	15,60	-0,075678508
PIGZ	3	complement(196946356..19696906	2,03	2,23	0,091326076
MELTF	3	complement(196980590..19702983	2,09	2,54	-0,059352951
DLG1	3	complement(197042560..19729933	2,38	4,46	-0,681953616
BDH1	3	complement(197509783..19757332	0,04	0,27	-2,683840696
RUBCN	3	complement(197668867..19774972	3,29	4,90	-0,351545334
FYTTD1	3	197737179..197787596	6,79	9,76	-0,300127961
LRCH3	3	197791226..197888436	2,02	5,14	-1,124074367
IQCG	3	complement(197889077..19796014	0,61	0,79	-0,143689071
RPL35A	3	197950190..197956610	180,61	#####	0,110783833
LMLN	3	197960200..198043720	1,03	1,90	-0,664653672

ZNF595	4	53286..88208	0,59	2,28	-1,737025869
ZNF718	4	124501..202303	0,82	0,86	0,162999456
ZNF732	4	complement(270675..305474)	0,01	0,01	-0,025382928
ZNF141	4	337814..384868	0,25	0,17	0,79485688
ZNF721	4	complement(425815..499156)	0,65	1,30	-0,764868284
PIGG	4	499210..540200	2,76	3,05	0,074282248
TMEM271	4	complement(573880..576295)	0,04	-	6,053060169
PDE6B	4	625573..670782	0,02	0,02	0,152452079
ATP5ME	4	complement(672436..674330)	26,90	30,08	0,061952944
MYL5	4	673580..682028	0,92	2,04	-0,91870908
SLC49A3	4	complement(681829..689271)	0,06	0,16	-1,251400229
PCGF3	4	705748..770089	7,47	6,64	0,392977955
CPLX1	4	complement(784957..826129)	0,13	0,04	1,887651853
GAK	4	complement(849276..932373)	12,19	10,30	0,465461358
TMEM175	4	932387..958656	3,37	3,00	0,390062541
DGKQ	4	complement(958887..986895)	4,56	3,74	0,507408522
SLC26A1	4	complement(979073..993440)	0,41	0,82	-0,790180158
IDUA	4	986997..1004564	2,85	2,53	0,397178023
FGFRL1	4	1009936..1026898	53,26	29,70	1,065960603
RNF212	4	complement(1056250..1113564)	0,01	-	4,516772761
SPON2	4	complement(1166932..1208962)	4,39	3,06	0,743040137
CTBP1	4	complement(1211445..1250333)	18,13	15,92	0,410647152
MAEA	4	1289887..1340147	5,51	7,38	-0,197628667
UVSSA	4	1345691..1395989	2,61	2,35	0,370499035
NKX1-1	4	complement(1402932..1406442)	0,01	-	3,068695355
FAM53A	4	complement(1617915..1684313)	0,28	0,59	-0,829765364
SLBP	4	complement(1692731..1712344)	18,62	17,45	0,316620981
TACC3	4	1712858..1745171	23,08	23,53	0,195214817
TMEM129	4	complement(1715952..1721358)	13,68	10,92	0,548285391
FGFR3	4	1793293..1808872	0,29	0,22	0,660315937
LETM1	4	complement(1811479..1856156)	12,14	18,54	-0,387345158
NSD2	4	1871393..1982207	11,15	14,71	-0,17690556
NELFA	4	complement(1982717..2041903)	7,49	8,85	-0,017619567
NICOL1	4	2041995..2043964	4,48	8,32	-0,67037305
NAT8L	4	2059327..2069089	2,74	7,94	-1,312544994
POLN	4	complement(2071918..2242121)	0,02	0,12	-2,538954594
gene:ENSG000	4	complement(2078998..2242276)	0,09	0,07	0,45565389
HAUS3	4	complement(2227464..2242133)	0,53	0,60	0,036719254
MXD4	4	complement(2247432..2262109)	11,88	8,89	0,642263897
ZFYVE28	4	complement(2269582..2418651)	1,38	0,98	0,718540329
CFAP99	4	2418955..2463002	-	0,01	-2,89252005
RNF4	4	2462220..2515857	8,72	10,14	0,005215115
gene:ENSG000	4	2512477..2625320	-	-	0,188027316
FAM193A	4	2536647..2732573	4,16	4,71	0,044787266
TNIP2	4	complement(2741648..2756342)	6,17	8,54	-0,246699197
SH3BP2	4	2793023..2841291	8,01	7,81	0,258537722
ADD1	4	2843844..2930076	13,51	17,48	-0,148757061
MFSD10	4	complement(2930561..2934834)	19,55	18,98	0,265819943

NOP14	4	complement(2937933..2963406)	7,23	11,18	-0,406310538
GRK4	4	2963571..3040760	0,42	0,43	0,158455527
HTT	4	3041363..3243957	7,91	6,93	0,412337112
MSANTD1	4	3244369..3271738	0,03	0,06	-0,723055004
RGS12	4	3293021..3439913	2,39	2,18	0,354892043
HGFAC	4	3441968..3449486	0,24	0,08	1,803279461
DOK7	4	3463306..3501473	0,13	0,08	0,92552177
LRPAP1	4	complement(3503612..3532446)	3,10	3,17	0,19018737
ADRA2C	4	3766348..3768526	1,63	0,85	1,164902758
OTOP1	4	complement(4188726..4226929)	0,02	0,01	1,436225396
TMEM128	4	complement(4235542..4248223)	2,95	4,56	-0,401285029
LYAR	4	complement(4267701..4290154)	1,99	4,61	-0,992442643
ZBTB49	4	4290251..4321786	1,40	1,06	0,624749521
NSG1	4	4348140..4419058	0,09	0,04	1,232127098
STX18	4	complement(4415742..4542346)	1,68	2,54	-0,367976696
gene:ENSG000	4	4820405..4821452	0,23	0,21	0,330019825
MSX1	4	4859665..4863936	3,16	3,52	0,067237068
CYTL1	4	complement(5014586..5019458)	-	-	#N/D
STK32B	4	5051480..5500994	0,28	0,04	3,004247668
EVC2	4	complement(5542772..5709548)	-	-	0,188027316
EVC	4	5711201..5814305	2,42	5,99	-1,085480708
CRMP1	4	complement(5748084..5893086)	0,38	1,06	-1,237568963
C4orf50	4	complement(5897373..6200555)	-	-	0,188027316
JAKMIP1	4	complement(6026199..6200591)	0,00	0,00	-0,01185201
gene:ENSG000	4	complement(6064977..6070162)	-	-	#N/D
WFS1	4	6269849..6303265	5,22	4,50	0,437418131
PPP2R2C	4	complement(6320578..6563600)	3,79	9,40	-1,086725274
MAN2B2	4	6575189..6623362	2,37	2,54	0,123405092
MRFAP1	4	6640091..6642729	23,63	30,96	-0,166707062
MRFAP1L2	4	6663396..6676755	2,81	2,86	0,199735017
gene:ENSG000	4	complement(6691995..6692970)	0,01	0,01	-0,01185201
S100P	4	6693878..6697170	0,14	0,25	-0,609885528
gene:ENSG000	4	complement(6697861..6698809)	-	-	0,188027316
MRFAP1L1	4	complement(6707701..6709865)	1,67	3,33	-0,775029173
BLOC1S4	4	6716174..6717664	2,00	3,64	-0,642805434
KIAA0232	4	6781375..6884170	2,25	3,48	-0,403021077
TBC1D14	4	6909242..7033118	4,66	7,27	-0,419619991
CCDC96	4	complement(7040849..7043001)	0,06	0,07	-0,037899139
TADA2B	4	7041899..7057952	1,97	2,49	-0,115320502
GRPEL1	4	complement(7058671..7068085)	7,43	9,35	-0,109383557
SORCS2	4	7192538..7742827	0,14	0,24	-0,538013855
PSAPL1	4	complement(7430285..7434930)	-	0,02	-5,734998842
AFAP1	4	complement(7758714..7939926)	11,52	18,79	-0,482638749
ABLIM2	4	complement(7965310..8158832)	0,10	0,15	-0,297924525
SH3TC1	4	8182072..8241803	0,93	1,09	-0,012817077
HTRA3	4	8269754..8307098	-	0,00	-2,89252005
ACOX3	4	complement(8366282..8440723)	2,41	3,18	-0,177920515
TRMT44	4	8436140..8493531	2,42	3,10	-0,138117414

GPR78	4	8558725..8619761	0,02	0,22	-2,996884072
CPZ	4	8592660..8619759	0,15	0,22	-0,295431037
HMX1	4	complement(8846076..8871839)	2,67	1,86	0,746924834
FAM90A26	4	9170409..9176730	-	0,01	-2,89252005
USP17L10	4	9210657..9212249	-	-	0,188027316
USP17L11	4	9215405..9216997	-	0,06	-5,545746556
USP17L12	4	9220152..9221744	-	-	#N/D
USP17L13	4	9224896..9226488	-	-	0,188027316
USP17L15	4	9234385..9236046	-	-	#N/D
USP17L17	4	9243879..9245471	-	-	#N/D
USP17L18	4	9248630..9250222	-	-	0,188027316
USP17L19	4	9253378..9254970	-	-	#N/D
USP17L20	4	9258124..9259716	-	-	#N/D
USP17L21	4	9262872..9264464	-	-	#N/D
USP17L22	4	9267619..9269211	-	-	#N/D
USP17L23	4	9272364..9272914	-	-	#N/D
USP17L24	4	9325165..9326757	-	-	#N/D
USP17L25	4	9329911..9331503	-	-	#N/D
USP17L26	4	9334658..9336250	-	-	#N/D
USP17L5	4	9339403..9340995	-	-	#N/D
USP17L27	4	9344148..9345740	-	-	#N/D
USP17L28	4	9348893..9350485	-	-	#N/D
USP17L29	4	9353638..9355230	-	-	#N/D
USP17L30	4	9363129..9364721	-	-	#N/D
DEFB131A	4	9444414..9450666	-	-	#N/D
SLC2A9	4	complement(9771153..10054936)	-	0,01	-3,804623889
DRD5	4	9781634..9784009	-	-	#N/D
WDR1	4	complement(10068089..10116972)	33,75	41,88	-0,088212658
ZNF518B	4	complement(10439880..10457426)	2,56	4,29	-0,522504219
CLNK	4	complement(10486395..10684768)	0,00	-	3,967268276
HS3ST1	4	complement(11393150..11429564)	0,24	0,08	1,750537062
RAB28	4	complement(13361354..13484365)	2,10	4,24	-0,788213922
NKX3-2	4	complement(13540830..13544508)	0,39	0,43	0,088035447
BOD1L1	4	complement(13568738..13627725)	2,55	3,03	-0,025573181
CPEB2	4	15002481..15070153	0,93	0,78	0,469303608
C1QTNF7	4	15339818..15446167	0,02	0,03	-0,27623233
CC2D2A	4	15469865..15601552	0,12	0,04	1,911711628
FBXL5	4	complement(15604381..15681679)	2,26	2,98	-0,173051231
FAM200B	4	15681662..15690447	0,69	0,89	-0,142944
BST1	4	15703065..15738313	0,07	0,19	-1,14333638
CD38	4	15778275..15853232	0,00	0,01	-1,478387936
FGFBP1	4	complement(15935577..15938740)	0,65	0,20	1,917932571
FGFBP2	4	complement(15960245..15969309)	-	-	#N/D
PROM1	4	complement(15963076..16084378)	-	0,00	-2,89252005
TAPT1	4	complement(16160505..16227410)	0,89	1,88	-0,85770975
LDB2	4	complement(16501541..16898678)	3,93	2,40	0,93783576
QDPR	4	complement(17460261..17512206)	3,22	4,61	-0,292549082
CLRN2	4	17515165..17527104	-	-	#N/D

LAP3	4	17577198..17607972	6,71	7,87	-0,005815278
MED28	4	17614641..17634105	3,82	2,69	0,725902426
FAM184B	4	complement(17629306..17781621)	0,03	0,03	0,64694678
DCAF16	4	complement(17800655..17810758)	6,73	5,91	0,4091549
NCAPG	4	17810979..17844865	4,55	7,14	-0,427865413
LCORL	4	complement(17841187..18021876)	0,49	0,64	-0,168541905
gene:ENSG000	4	19098199..19098740	-	-	#N/D
gene:ENSG000	4	complement(19257951..19258337)	-	-	#N/D
SLIT2	4	20251905..20620561	6,91	6,72	0,263520626
PACRGL	4	20696282..20752907	0,77	1,34	-0,572826494
KCNIP4	4	complement(20728606..21948772)	-	-	0,188027316
ADGRA3	4	complement(22345071..22516066)	6,60	6,92	0,155154935
GBA3	4	22692914..22819575	-	-	0,188027316
PPARGC1A	4	complement(23755041..23904089)	0,32	0,16	1,214029926
DHX15	4	complement(24517441..24584554)	35,46	36,78	0,170213304
SOD3	4	24789912..24800842	0,09	0,13	-0,356881532
CCDC149	4	complement(24803514..24980204)	3,69	3,53	0,287033841
LGI2	4	complement(24998847..25030946)	0,15	2,17	-3,667843731
SEPSECS	4	complement(25120014..25160550)	0,68	1,03	-0,383569734
PI4K2B	4	25160663..25279204	2,94	3,53	-0,043720484
ZCCHC4	4	25312774..25370383	2,07	3,51	-0,541602299
ANAPC4	4	25377263..25418498	1,18	2,05	-0,575948632
SLC34A2	4	25648011..25678748	0,00	-	3,068695355
SEL1L3	4	complement(25747433..25863760)	2,68	9,88	-1,660256426
SMIM20	4	25861830..25929813	4,73	6,81	-0,301373312
RBPJ	4	26163455..26435131	7,92	5,62	0,718696788
CCKAR	4	complement(26481396..26490484)	0,01	-	3,967268276
TBC1D19	4	26576437..26756223	0,53	0,76	-0,295330255
STIM2	4	26857601..27025381	0,60	1,00	-0,520356039
PCDH7	4	30720369..31146805	0,12	0,10	0,468187801
ARAP2	4	complement(35948221..36244514)	0,20	0,74	-1,670113526
DTHD1	4	36281616..36347511	-	-	0,188027316
NWD2	4	37244743..37449463	0,02	0,01	1,391449417
C4orf19	4	37453925..37623495	0,06	0,08	-0,311141754
RELL1	4	complement(37590800..37686376)	6,23	5,52	0,396248497
PGM2	4	37826660..37862937	5,14	7,35	-0,293965144
gene:ENSG000	4	complement(37866561..37867091)	0,24	0,44	-0,617784428
TBC1D1	4	37891084..38139175	7,36	8,90	-0,050430086
PTTG2	4	37960398..37961128	0,04	0,04	0,524121557
gene:ENSG000	4	38390754..38391675	-	-	#N/D
KLF3	4	38664197..38701517	3,72	3,48	0,320662444
TLR10	4	complement(38772238..38782990)	0,00	-	3,068695355
TLR1	4	complement(38790677..38856817)	0,02	0,21	-3,062817276
TLR6	4	complement(38822897..38856817)	0,19	0,29	-0,369347044
FAM114A1	4	38867677..38945739	38,40	26,35	0,76639195
TMEM156	4	complement(38966744..39032922)	3,22	11,43	-1,603181024
KLHL5	4	39045039..39126857	4,28	7,29	-0,543218728
WDR19	4	39182504..39285810	0,66	1,61	-1,068458636

RFC1	4	complement(39287456..39366375)	9,71	12,77	-0,171726974
KLB	4	39406930..39451533	0,06	0,13	-0,839042789
RPL9	4	complement(39452587..39458931)	41,32	64,29	-0,414601215
LIAS	4	39459004..39485109	0,34	0,59	-0,588552701
UGDH	4	complement(39498755..39528311)	20,28	11,07	1,096390981
SMIM14	4	complement(39546336..39638902)	4,34	2,33	1,12327065
UBE2K	4	39698109..39782792	3,77	5,80	-0,398801099
PDS5A	4	complement(39822863..39977956)	7,28	12,26	-0,529329626
N4BP2	4	40056797..40158252	0,41	0,78	-0,710565877
RHOH	4	40191011..40246967	0,01	0,01	-0,03547153
CHRNA9	4	40335333..40355217	-	0,05	-5,902279478
RBM47	4	complement(40423267..40630875)	0,15	0,22	-0,352489165
NSUN7	4	40749925..40811184	0,01	0,01	0,921097799
APBB2	4	complement(40810027..41216714)	1,77	5,43	-1,392521882
gene:ENSG000	4	complement(41143022..41143099)	-	-	#N/D
UCHL1	4	41256413..41268455	32,27	#####	-1,549108153
LIMCH1	4	41359607..41700044	10,16	6,32	0,908223405
PHOX2B	4	complement(41744082..41748725)	-	-	#N/D
TMEM33	4	41935129..41960803	4,99	8,48	-0,541927035
DCAF4L1	4	41981756..41986465	0,05	0,09	-0,452538262
SLC30A9	4	41990502..42090461	2,78	3,84	-0,244216679
BEND4	4	complement(42110853..42152878)	0,08	0,25	-1,343558861
SHISA3	4	42397488..42402487	0,81	1,03	-0,129063904
ATP8A1	4	complement(42408373..42657121)	0,39	0,19	1,268440284
GRXCR1	4	42892713..43030658	0,13	0,14	0,042784718
KCTD8	4	complement(44173903..44448809)	0,02	0,00	2,399873901
YIPF7	4	complement(44622088..44678556)	-	-	0,188027316
GUF1	4	44678420..44700928	1,32	5,42	-1,816964495
GNPDA2	4	complement(44682200..44726588)	0,49	1,12	-0,979481474
GABRG1	4	complement(46035769..46124054)	-	0,02	-6,312013662
GABRA2	4	complement(46243548..46475230)	0,65	0,49	0,644801687
COX7B2	4	complement(46734827..46909245)	-	-	0,188027316
GABRA4	4	complement(46918900..46993581)	-	-	0,188027316
GABRB1	4	46993723..47426447	1,91	0,08	4,814327986
COMMD8	4	complement(47450787..47463702)	2,10	2,61	-0,090667983
ATP10D	4	47485275..47593486	2,44	2,23	0,353256028
CORIN	4	complement(47593999..47838106)	0,03	0,05	-0,791308264
NFXL1	4	complement(47847233..47914667)	3,80	4,10	0,111018493
NIPAL1	4	47914142..48040173	0,08	0,36	-1,898279099
CNGA1	4	complement(47935977..48016681)	0,02	0,02	0,278120973
TXK	4	complement(48066393..48134250)	0,00	0,04	-3,171495545
TEC	4	complement(48135783..48269838)	0,29	0,86	-1,355391835
SLAIN2	4	48341529..48426201	5,37	5,70	0,137396327
SLC10A4	4	48483343..48489526	0,06	0,08	-0,152395412
ZAR1	4	48490252..48494389	-	0,06	-5,545746556
FRYL	4	complement(48497357..48780322)	2,21	2,93	-0,18155122
OCIAD1	4	48805212..48861817	6,32	11,58	-0,650782711
OCIAD2	4	complement(48885019..48906937)	-	0,01	-2,89252005

CWH43	4	48986275..49062081	-	-	0,188027316
gene:ENSG000	4	49229573..49231284	-	-	0,188027316
DCUN1D4	4	51843000..51916837	11,07	2,71	2,251698742
LRRC66	4	complement(51993652..52020363)	0,05	0,04	0,539899626
SGCB	4	complement(52020706..52038299)	14,08	5,68	1,531647566
SPATA18	4	52051304..52097299	0,01	0,01	1,232079601
USP46	4	complement(52590960..52659301)	3,33	1,25	1,640488274
ERVMER34-1	4	complement(52722618..52751586)	0,04	0,00	3,69954316
RASL11B	4	52862317..52866835	0,39	0,06	2,920796843
SCFD2	4	complement(52872982..53366066)	49,75	5,93	3,291132239
FIP1L1	4	53377569..53464382	18,01	4,18	2,331629232
gene:ENSG000	4	53377839..54295272	8,74	1,37	2,901065951
LNX1	4	complement(53459301..53701405)	0,99	0,43	1,430459993
CHIC2	4	complement(54009789..54064605)	16,73	4,87	2,003912825
GSX2	4	54099523..54102498	-	-	0,188027316
PDGFRA	4	54229280..54298245	31,46	1,88	4,290945617
KIT	4	54657267..54740783	0,08	0,03	1,535717141
KDR	4	complement(55078481..55125595)	0,06	0,01	2,560506508
SRD5A3	4	55346213..55373100	2,24	2,39	0,131724542
gene:ENSG000	4	55346228..55388368	0,10	0,11	-0,040054557
TMEM165	4	55395957..55453397	3,73	5,26	-0,272384496
CLOCK	4	complement(55427903..55546909)	2,26	2,95	-0,165518323
PDCL2	4	complement(55556519..55592245)	-	0,03	-3,804623889
NMU	4	complement(55595229..55636698)	1,17	0,62	1,147042138
EXOC1L	4	55819790..55837489	-	-	0,188027316
EXOC1	4	55853648..55905086	3,56	3,67	0,182118086
CEP135	4	55948871..56033361	0,48	0,81	-0,532759149
CRACD	4	56049098..56330609	0,06	0,29	-1,966375368
AASDH	4	complement(56338287..56387508)	1,24	1,89	-0,387276174
PPAT	4	complement(56393362..56435615)	5,09	5,76	0,044042156
PAICS	4	56435741..56464578	28,87	33,79	-0,004169195
SRP72	4	56467617..56503681	14,00	19,58	-0,260743629
ARL9	4	56505209..56524959	-	0,03	-5,327865481
SPMAP2L	4	56530606..56603507	-	-	0,188027316
HOPX	4	complement(56647989..56681877)	0,06	0,05	0,485631682
gene:ENSG000	4	complement(56662469..56665694)	-	0,03	-5,545746556
SPINK2	4	complement(56809860..56821742)	-	-	0,188027316
REST	4	56907876..56966808	3,95	4,14	0,153022073
NOA1	4	complement(56963350..56977606)	8,57	8,51	0,23315472
POLR2B	4	56977722..57031158	12,20	14,46	-0,022520774
IGFBP7	4	complement(57030773..57110385)	33,46	22,32	0,807041161
ADGRL3	4	61200326..62078335	0,01	-	6,20258586
TECRL	4	complement(64275257..64409468)	-	-	#N/D
EPHA5	4	complement(65319563..65670495)	0,55	0,12	2,408319512
CENPC	4	complement(67468762..67545503)	1,05	1,43	-0,224754359
STAP1	4	67558727..67607337	-	-	0,188027316
gene:ENSG000	4	complement(67607856..67610313)	0,08	0,11	-0,189986357
UBA6	4	complement(67612652..67701155)	1,43	2,86	-0,773021283

GNRHR	4	complement(67737118..67754388)	0,02	0,04	-0,833928617
TMPRSS11D	4	complement(67820876..67884002)	0,03	0,05	-0,542645869
TMPRSS11A	4	complement(67909395..67964140)	0,01	0,02	-0,999120035
TMPRSS11F	4	complement(68053198..68129869)	-	-	#N/D
TMPRSS11B	4	complement(68226653..68245694)	-	-	0,188027316
YTHDC1	4	complement(68310387..68350090)	3,97	5,19	-0,162104486
TMPRSS11E	4	68447463..68497604	-	-	0,188027316
UGT2B17	4	complement(68537173..68576413)	-	-	#N/D
UGT2B15	4	complement(68646597..68670652)	-	-	#N/D
UGT2B10	4	68815994..68832023	-	-	0,188027316
UGT2A3	4	complement(68928463..68951804)	-	-	#N/D
UGT2B7	4	69051363..69112987	-	-	0,188027316
UGT2B11	4	complement(69199951..69214748)	-	-	#N/D
UGT2B28	4	69280475..69295050	-	-	#N/D
UGT2B4	4	complement(69480165..69526014)	0,01	-	4,516772761
UGT2A2	4	complement(69588417..69639642)	0,01	-	3,967268276
UGT2A1	4	complement(69588417..69653249)	-	-	0,188027316
SULT1B1	4	complement(69721167..69787961)	0,36	0,07	2,476031563
gene:ENSG000	4	complement(69810780..69847786)	-	-	#N/D
SULT1E1	4	complement(69841212..69860145)	0,01	-	3,068695355
CSN1S1	4	69931068..69946574	-	-	#N/D
CSN2	4	complement(69955256..69965728)	-	-	#N/D
STATH	4	69995966..70002570	0,02	-	3,068695355
HTN3	4	70028455..70036538	-	-	#N/D
HTN1	4	70050438..70058848	-	-	#N/D
PRR27	4	70133616..70176799	-	-	#N/D
ODAM	4	70195725..70204576	-	-	#N/D
FDCSP	4	70226124..70235252	0,13	0,05	1,487770062
CSN3	4	70238382..70251474	-	-	#N/D
CABS1	4	70334981..70337116	-	-	0,188027316
SMR3A	4	70360760..70367158	-	-	#N/D
SMR3B	4	70370093..70390244	-	-	#N/D
OPRPN	4	70397931..70410195	-	-	0,188027316
MUC7	4	70430492..70482997	-	-	0,188027316
AMTN	4	70518569..70532743	0,81	4,81	-2,341238543
AMBN	4	70592256..70607288	-	0,01	-3,804623889
ENAM	4	70628744..70646824	-	-	0,188027316
JCHAIN	4	complement(70655541..70681817)	0,01	-	3,068695355
UTP3	4	70688532..70690551	2,99	4,44	-0,346102924
RUFY3	4	70704204..70808619	0,93	1,19	-0,129115988
GRSF1	4	complement(70815783..70839897)	5,10	6,83	-0,197152825
MOB1B	4	70902326..71022449	1,73	1,76	0,193729633
DCK	4	70992538..71030914	2,91	3,87	-0,188449885
SLC4A4	4	71062667..71572087	0,01	0,04	-1,301789231
GC	4	complement(71741696..71804041)	-	-	#N/D
NPFFR2	4	72031902..72148305	-	-	0,188027316
ADAMTS3	4	complement(72280969..72569221)	3,18	2,71	0,455671508
COX18	4	complement(73052362..73069755)	1,33	1,77	-0,184968544

ANKRD17	4	complement(73073376..73258798)	11,45	14,79	-0,146018451
ALB	4	73397114..73421482	-	0,03	-5,071123353
AFP	4	73431138..73456174	0,06	0,29	-2,107984006
AFM	4	73481745..73504001	-	-	#N/D
RASSF6	4	complement(73571550..73620631)	-	0,00	-3,804623889
CXCL8	4	73740519..73743716	100,53	#####	-1,497359844
CXCL6	4	73836640..73849064	-	-	0,188027316
PF4V1	4	73853296..73854483	2,08	0,52	2,207249427
CXCL1	4	73869393..73871308	17,68	#####	-3,069805818
PF4	4	complement(73980811..73982027)	0,02	-	3,967268276
gene:ENSG000	4	complement(73981074..73988190)	0,03	-	4,516772761
PPBP	4	complement(73986439..73988190)	-	-	#N/D
CXCL5	4	complement(73995642..73998677)	-	0,01	-2,89252005
CXCL3	4	complement(74036589..74038807)	1,52	14,65	-3,039551554
CXCL2	4	complement(74097040..74099196)	0,20	10,24	-5,422666299
MTHFD2L	4	74114174..74303099	0,22	0,70	-1,458402019
EPGN	4	74308470..74316789	0,02	0,03	-0,698295353
EREG	4	74365145..74388749	1,67	0,00	9,261752543
AREG	4	74445136..74455005	0,03	0,12	-1,462209385
BTC	4	complement(74744759..74794523)	-	0,10	-7,112521263
PARM1	4	74933095..75050115	0,00	0,04	-2,752886291
RCHY1	4	complement(75479033..75514764)	0,25	0,63	-1,134422051
THAP6	4	75513946..75550473	0,47	0,79	-0,532646131
ODAPH	4	75556048..75565871	0,02	0,01	1,833201638
CDKL2	4	complement(75576496..75630716)	0,00	-	3,068695355
G3BP2	4	complement(75641849..75724589)	6,15	7,12	0,013176102
USO1	4	75724577..75814286	9,19	11,80	-0,13812293
PPEF2	4	complement(75859864..75902571)	0,00	-	3,068695355
NAAA	4	complement(75913660..75941013)	1,16	1,46	-0,106734978
SDAD1	4	complement(75940950..75990962)	3,33	7,16	-0,879160916
CXCL9	4	complement(76001275..76007509)	-	-	0,188027316
ART3	4	76011184..76114048	0,00	-	3,068695355
CXCL10	4	complement(76021118..76023497)	1,06	0,41	1,591905597
CXCL11	4	complement(76033682..76041415)	0,08	0,05	0,947848749
NUP54	4	complement(76107562..76148485)	0,75	1,48	-0,747690873
SCARB2	4	complement(76158737..76234536)	9,76	14,02	-0,298948661
FAM47E	4	76214040..76283783	0,00	0,01	-2,190455314
FAM47E-STBD1	4	76251721..76311129	0,02	0,05	-0,802495947
STBD1	4	76306733..76311130	1,78	1,92	0,119381384
CCDC158	4	complement(76312997..76421868)	-	-	0,188027316
SHROOM3	4	76435229..76783253	6,91	4,10	0,974738565
SOWAHB	4	complement(76894152..76898144)	0,01	0,01	0,524121557
SEPTIN11	4	76949751..77040384	7,84	9,04	0,018034489
CCNI	4	complement(77047155..77076309)	55,84	58,60	0,153513336
CCNG2	4	77157207..77433388	2,28	1,47	0,860863436
CXCL13	4	77511753..77611834	-	-	0,188027316
CNOT6L	4	complement(77713387..77819615)	3,90	3,21	0,501288561
MRPL1	4	77862830..77952785	1,75	3,93	-0,946623809

FRAS1	4	78057323..78544269	0,68	2,46	-1,626957381
ANXA3	4	78551747..78610451	0,01	0,01	-0,923955849
BMP2K	4	78776342..78916365	0,68	1,77	-1,146140298
PAQR3	4	complement(78887127..78939438)	1,01	2,13	-0,848132679
NAA11	4	complement(79225694..79326061)	-	0,01	-3,804623889
GK2	4	complement(79406361..79408228)	-	-	0,188027316
ANTXR2	4	complement(79901146..80125454)	12,11	8,58	0,721107
PRDM8	4	80183879..80204329	6,48	6,32	0,257423702
FGF5	4	80266639..80336680	7,86	5,51	0,734250806
CFAP299	4	80335730..80963750	0,03	0,02	0,842164604
BMP3	4	81030708..81057627	0,01	-	4,516772761
PRKG2	4	complement(81087370..81215222)	0,02	0,01	1,339142659
RASGEF1B	4	complement(81426393..82044244)	0,00	-	4,913749003
HNRNPD	4	complement(82352498..82374503)	8,71	15,79	-0,63564215
HNRNPDL	4	complement(82422565..82430462)	10,12	14,38	-0,283139913
ENOPH1	4	82430590..82461177	9,31	11,83	-0,123498925
TMEM150C	4	complement(82483170..82562357)	0,15	0,33	-0,883182473
SCD5	4	complement(82629539..82798796)	3,55	3,58	0,212034648
SEC31A	4	complement(82818509..82901166)	29,35	18,83	0,86315924
THAP9	4	82900684..82919969	0,16	0,38	-0,994568817
LIN54	4	complement(82909973..83012926)	2,12	2,71	-0,135203026
COPS4	4	83034447..83075818	4,60	5,57	-0,050976706
PLAC8	4	complement(83090048..83137075)	0,01	0,00	1,436225396
COQ2	4	complement(83261536..83284914)	1,75	3,46	-0,763193636
HPSE	4	complement(83292461..83335153)	0,23	1,43	-2,394823532
HELQ	4	complement(83407343..83455855)	0,49	1,10	-0,929097704
MRPS18C	4	83455932..83469735	1,67	1,63	0,25796212
ABRAXAS1	4	complement(83459517..83523348)	0,23	0,36	-0,418110575
GPAT3	4	83535914..83605875	3,00	2,24	0,642232763
NKX6-1	4	complement(84491985..84499292)	2,42	1,61	0,812875251
CDS1	4	84583127..84651334	0,36	0,45	-0,079877481
WDFY3	4	complement(84668765..84966690)	3,14	3,65	0,007087583
ARHGAP24	4	85475150..86002668	3,65	2,49	0,777100717
MAPK10	4	complement(85990007..86594625)	0,05	0,02	1,433126423
PTPN13	4	86594315..86815171	1,36	2,64	-0,735000598
SLC10A6	4	complement(86823468..86849384)	-	-	0,188027316
C4orf36_1	4	complement(86876205..86892422)	0,01	0,14	-4,298551384
C4orf36_2	4	complement(86886472..86936178)	0,03	0,12	-1,820127312
AFF1	4	86935002..87141039	1,42	3,57	-1,105853418
KLHL8	4	complement(87160103..87240314)	1,00	1,76	-0,59168207
HSD17B13	4	complement(87303789..87322886)	0,01	0,02	-0,579815015
HSD17B11	4	complement(87336515..87391188)	4,31	6,24	-0,309546275
NUDT9	4	87422573..87459455	1,45	2,16	-0,347359374
SCPPPQ1	4	complement(87460802..87471050)	-	-	#N/D
SPARCL1	4	complement(87473335..87531061)	-	0,02	-5,071123353
DSPP	4	87608529..87616873	-	-	0,188027316
DMP1	4	87650280..87664361	-	-	0,188027316
IBSP	4	87799554..87812435	-	-	#N/D

MEPE	4	87821398..87846814	-	-	0,188027316
SPP1	4	87975667..87983532	-	0,01	-2,89252005
PKD2	4	88007635..88077777	1,33	1,99	-0,358110394
ABCG2	4	complement(88090150..88231628)	0,31	0,67	-0,91211496
PPM1K	4	complement(88257620..88284769)	1,08	0,92	0,45637718
HERC6	4	88378739..88443097	0,81	2,39	-1,344531961
HERC5	4	88457119..88506163	1,28	2,97	-0,990624958
PIGY	4	complement(88520998..88523776)	5,78	7,35	-0,122598427
PYURF	4	complement(88520998..88523776)	5,69	7,28	-0,131710421
HERC3_1	4	88523810..88708450	0,20	0,20	0,184190295
HERC3_2	4	88592434..88708541	3,89	3,56	0,353623079
NAP1L5	4	complement(88695913..88697829)	0,33	0,72	-0,922272643
FAM13A	4	complement(88725955..89111398)	0,76	0,77	0,203943534
TIGD2	4	89111533..89114901	0,69	1,30	-0,687329474
GPRIN3	4	complement(89236383..89307937)	0,06	0,22	-1,680670052
SNCA	4	complement(89700345..89838315)	0,03	0,18	-2,146299826
MMRN1	4	89879532..89954629	0,01	-	4,516772761
CCSER1	4	90127394..91605295	0,04	0,01	2,806848694
gene:ENSG000	4	91603275..91605292	-	-	#N/D
gene:ENSG000	4	complement(92260367..92260751)	-	-	#N/D
GRID2	4	92303966..93810157	0,00	0,02	-2,94381988
ATOH1	4	93828753..93830964	-	0,06	-6,052163584
SMARCAD1	4	94207611..94291292	1,01	2,54	-1,108813598
HPGDS	4	complement(94298535..94342876)	-	0,02	-3,804623889
PDLIM5	4	94451857..94668227	4,73	14,36	-1,378735372
BMPR1B	4	94757955..95158448	0,04	0,46	-3,142640803
UNC5C	4	complement(95162504..95549206)	0,01	0,03	-1,963538535
PDHA2	4	95840093..95841464	-	-	#N/D
STPG2	4	complement(97184093..98143476)	0,01	0,00	0,524121557
RAP1GDS1	4	98261384..98443858	5,27	6,53	-0,085776523
TSPAN5	4	complement(98470367..98658611)	11,73	8,58	0,674549059
EIF4E	4	complement(98879276..98929133)	6,04	7,90	-0,164236136
METAP1	4	98995659..99062809	9,50	12,64	-0,189189971
ADH5	4	complement(99070978..99088801)	14,24	16,55	0,006275332
ADH4	4	complement(99123657..99157792)	-	-	0,188027316
ADH6	4	complement(99202638..99219537)	-	-	0,188027316
ADH1A	4	complement(99276369..99291003)	-	-	#N/D
ADH1B	4	complement(99304971..99321401)	-	-	0,188027316
ADH1C	4	complement(99336497..99352746)	-	-	0,188027316
ADH7	4	complement(99412261..99435510)	-	-	#N/D
C4orf17	4	99511012..99542303	-	-	0,188027316
TRMT10A	4	complement(99546709..99564039)	0,32	0,32	0,22951241
MTTP	4	99564081..99623997	0,00	0,03	-3,431345623
C4orf54	4	complement(99636529..99657828)	-	-	0,188027316
DAPP1	4	99816827..99870190	0,01	0,00	1,436225396
gene:ENSG000	4	99844138..99844696	-	-	#N/D
LAMTOR3	4	complement(99878336..99894428)	1,64	1,33	0,521337597
DNAJB14	4	complement(99896248..99946618)	0,73	1,11	-0,378582182

gene:ENSG000	4	complement(99942081..99942522)	-	-	0,188027316
H2AZ1	4	complement(99948086..99950355)	135,76	#####	0,118198232
DDIT4L	4	complement(100185870..10019078	0,11	0,06	1,05243937
EMCN	4	complement(100395341..10088012	-	-	#N/D
PPP3CA	4	complement(101023409..10134827	7,84	8,55	0,098506771
BANK1	4	101411286..102074812	0,01	0,02	-0,291161992
SLC39A8	4	complement(102251080..10243125	0,66	1,58	-1,042114079
NFKB1	4	102501330..102617302	4,63	12,00	-1,150438755
MANBA	4	complement(102630770..10276099	1,12	1,69	-0,365081776
UBE2D3	4	complement(102794383..10286889	8,57	12,23	-0,289405711
CISD2	4	102868974..102892807	1,23	1,98	-0,455312087
SLC9B1	4	complement(102885048..10301971	0,01	0,02	-0,032860829
SLC9B2	4	complement(103019868..10308582	0,85	1,27	-0,356263013
BDH2	4	complement(103077592..10309987	0,75	1,18	-0,42000448
CENPE	4	complement(103105349..10319844	0,90	2,04	-0,962306006
TACR3	4	complement(103586031..10371998	0,00	-	3,068695355
CXXC4	4	complement(104468308..10449490	0,13	0,21	-0,523143746
TET2	4	105145875..105279816	0,77	0,83	0,110430682
PPA2	4	complement(105369077..10547406	3,92	6,29	-0,460599103
ARHGEF38	4	105552620..105708093	0,00	0,00	-0,01185201
INTS12	4	complement(105682627..10589598	2,07	2,27	0,090089603
GSTCD	4	105708778..105847725	1,86	3,24	-0,575928791
NPNT	4	105894775..106004027	-	0,00	-2,89252005
TBCK	4	complement(106041599..10631668	0,87	1,31	-0,366945756
AIMP1	4	106315544..106349456	1,96	2,29	-0,00426763
GIMD1	4	complement(106357392..10636877	-	-	0,188027316
DKK2	4	complement(106921802..10728380	0,47	0,44	0,333375268
PAPSS1	4	complement(107590276..10772023	10,89	10,82	0,231848486
SGMS2	4	107824563..107915047	1,83	2,70	-0,336636432
CYP2U1	4	107931549..107953461	0,35	0,45	-0,127542018
HADH	4	107989714..108035241	4,23	5,61	-0,184018131
LEF1	4	complement(108047545..10816895	0,34	0,94	-1,260703444
RPL34	4	108620569..108630412	85,89	86,56	0,211817847
OSTC	4	108650585..108667820	35,29	28,15	0,54943844
ETNPPL	4	complement(108742048..10876305	0,00	-	3,068695355
COL25A1	4	complement(108808725..10930275	0,00	-	3,068695355
SEC24B	4	109433772..109540896	5,36	7,10	-0,181596505
MCUB	4	109560209..109688719	2,46	4,56	-0,667547407
CASP6	4	complement(109688622..10970358	1,25	2,41	-0,729802357
PLA2G12A	4	complement(109709989..10973007	1,40	1,70	-0,055671406
gene:ENSG000	4	complement(109713916..10980199	0,05	0,09	-0,535949509
CFI	4	complement(109731008..10980215	0,05	0,05	0,112279066
GAR1	4	109815510..109824740	2,19	3,53	-0,463937839
RRH	4	109827972..109849123	0,03	0,06	-0,734644919
LRIT3	4	109848107..109872315	0,04	0,03	0,539899626
EGF	4	109912883..110013766	0,19	0,27	-0,260955063
ELOVL6	4	complement(110045846..11019919	1,12	2,49	-0,925904837
ENPEP	4	110365733..110565285	0,00	-	3,068695355

PITX2	4	complement(110617423..11064212	1,00	0,61	0,941168742
FAM241A	4	112145454..112195256	0,25	0,59	-1,008238256
AP1AR	4	112231740..112273110	0,57	1,33	-0,987686814
TIFA	4	complement(112272968..11228595	1,53	1,77	0,010269587
ALPK1	4	112285509..112442621	1,19	2,31	-0,73698741
NEUROG2	4	complement(112513516..11251618	0,01	0,87	-6,199824157
ZGRF1	4	complement(112539333..11263699	0,78	0,99	-0,127219237
LARP7	4	112637077..112657696	0,95	1,44	-0,371818859
ANK2	4	112818032..113384221	0,17	1,50	-2,906404009
CAMK2D	4	complement(113418054..11376192	1,41	1,61	0,026280866
ARSJ	4	complement(113900284..11397972	4,69	6,68	-0,286804244
UGT8	4	114598770..114678225	0,64	-	10,88140693
NDST4	4	complement(114827763..11511387	-	-	#N/D
TRAM1L1	4	complement(117083554..11708557	0,35	0,45	-0,167500809
NDST3	4	118033618..118258634	0,02	0,00	3,381348802
PRSS12	4	complement(118280038..11835300	22,66	9,32	1,504058661
METTL14	4	118685392..118715433	0,77	1,33	-0,559106523
SEC24D	4	complement(118722823..11883868	25,84	19,77	0,609217894
SYNPO2	4	118850688..119061247	0,01	0,03	-1,529134385
MYOZ2	4	119135832..119187789	-	0,01	-3,804623889
USP53	4	119212587..119295518	0,86	2,15	-1,104523507
C4orf3	4	complement(119296419..11930444	4,25	1,70	1,546252275
FABP2	4	complement(119317250..11932213	0,00	-	3,068695355
PDE5A	4	complement(119494397..11962880	5,37	0,68	3,193651934
MAD2L1	4	complement(120055623..12006685	6,83	8,59	-0,108024016
PRDM5	4	complement(120684919..12092287	0,35	0,71	-0,798351906
NDNF	4	complement(121035613..12107302	0,07	0,91	-3,535066198
TNIP3	4	complement(121131408..12122746	0,12	0,63	-2,157434172
QRFPR	4	complement(121328642..12138105	1,00	3,17	-1,433132656
ANXA5	4	complement(121667946..12169699	218,31	#####	0,334773806
SMIM43	4	complement(121758881..12176542	0,12	0,15	-0,09009037
EXOSC9	4	121801318..121817021	4,44	5,47	-0,07924561
CCNA2	4	complement(121816444..12182388	22,65	35,73	-0,434756847
BBS7	4	complement(121824329..12187048	1,69	2,84	-0,524940765
TRPC3	4	complement(121874481..12195206	0,05	0,10	-0,62372666
BLTP1	4	122152331..122364167	3,34	3,70	0,076793348
ADAD1	4	122378966..122429802	-	-	#N/D
IL2	4	complement(122451470..12245672	-	-	#N/D
IL21	4	complement(122610108..12262106	0,01	-	4,516772761
BBS12	4	122732702..122744942	0,72	1,15	-0,447707157
FGF2	4	122826682..122898236	27,93	32,79	-0,008333269
NUDT6	4	complement(122888697..12292296	5,66	5,01	0,398613129
AFG2A	4	122923070..123319433	4,13	5,12	-0,086085541
SPRY1	4	123396795..123403760	0,21	0,98	-2,007103284
ANKRD50	4	complement(124664048..12471273	2,59	4,19	-0,471169415
FAT4	4	125314918..125492932	1,08	0,69	0,874362246
INTU	4	127623271..127726737	0,31	0,50	-0,445516201
SLC25A31	4	127730400..127774292	-	-	#N/D

HSPA4L	4	127781821..127840733	0,07	0,58	-2,892998218
PLK4	4	127880893..127899224	1,88	3,79	-0,784729175
MFSD8	4	complement(127917799..12796603	0,46	0,57	-0,092451835
ABHD18	4	127965315..128039953	0,74	1,05	-0,278164096
LARP1B	4	128061286..128222931	1,19	1,28	0,122125735
PGRMC2	4	complement(128269237..12828882	5,33	4,84	0,362419335
JADE1	4	128809700..128875224	2,60	4,53	-0,578350165
SCLT1	4	complement(128864921..12909360	0,88	1,35	-0,383325929
C4orf33	4	129093317..129116640	0,26	0,26	0,202556837
gene:ENSG000	4	130808507..130811933	-	-	#N/D
PCDH10	4	133149294..133208606	0,14	0,33	-0,970642998
PABPC4L	4	complement(134196333..13420178	0,69	0,50	0,683781233
gene:ENSG000	4	136553236..136553739	-	-	#N/D
PCDH18	4	complement(137518918..13753249	1,37	0,47	1,766831949
gene:ENSG000	4	complement(137601899..13760243	-	-	#N/D
SLC7A11	4	complement(138164097..13824234	4,65	3,71	0,548752384
NOCT	4	139015781..139045939	8,97	7,78	0,427891577
ELF2	4	complement(139028112..13917721	2,57	2,67	0,16292293
MGARP	4	complement(139266165..13928022	0,65	0,26	1,520058371
NDUFC1	4	complement(139266880..13930255	2,05	2,94	-0,293219818
NAA15	4	139301446..139420033	2,77	4,97	-0,620001681
RAB33B	4	139453232..139476609	1,51	1,22	0,524711265
SETD7	4	complement(139495941..13960669	4,86	6,57	-0,21195857
MGST2	4	139665768..139740745	1,18	2,30	-0,738617061
MAML3	4	complement(139716753..14015418	1,38	2,92	-0,862433178
SCOC	4	140257286..140385728	1,56	2,16	-0,242727746
CLGN	4	complement(140388453..14042766	0,59	3,50	-2,346526297
MGAT4D	4	complement(140442262..14049837	-	-	0,188027316
gene:ENSG000	4	140498402..140501330	-	-	0,188027316
ELMOD2	4	140524168..140553770	0,73	0,96	-0,176096669
UCP1	4	complement(140559431..14056896	-	-	0,188027316
TBC1D9	4	complement(140620782..14075638	4,87	5,26	0,110101802
RNF150	4	complement(140859807..14121287	0,54	1,03	-0,719430178
ZNF330	4	141220887..141234697	5,60	7,34	-0,167342532
IL15	4	141636583..141733987	0,16	0,23	-0,324995935
INPP4B	4	complement(142023160..14284743	0,05	0,32	-2,316305563
USP38	4	143184917..143223874	1,72	2,78	-0,466196338
GAB1	4	143336876..143474565	0,25	0,32	-0,103870231
SMARCA5	4	143513702..143557486	4,35	7,93	-0,642153613
FREM3	4	complement(143577302..14370067	-	-	#N/D
GYPE	4	complement(143870864..14390556	0,09	0,10	-0,038823173
GYPB	4	complement(143996104..14401938	-	-	#N/D
GYPA	4	complement(144109303..14414075	-	-	0,188027316
gene:ENSG000	4	complement(144210701..14509817	0,14	0,14	0,21051339
HHIP	4	144646156..144745271	0,03	0,96	-4,92445917
ANAPC10	4	complement(144831908..14509854	0,94	1,36	-0,307637701
ABCE1	4	145098288..145129524	7,44	13,92	-0,679724985
OTUD4	4	complement(145110838..14518058	4,60	6,64	-0,30654833

gene:ENSG000	4	145376909..145377957	-	-	0,188027316
SMAD1	4	145481194..145559176	1,02	2,97	-1,317698309
MMAA	4	145599042..145660033	0,29	0,41	-0,248559523
C4orf51	4	145680146..145771032	-	-	0,188027316
ZNF827	4	complement(145757627..14593882	1,00	1,72	-0,563211054
gene:ENSG000	4	complement(145830456..14583298	0,04	0,09	-0,795259817
LSM6	4	146175703..146200000	0,52	0,92	-0,608009178
gene:ENSG000	4	146180851..146181316	-	-	0,188027316
REELD1	4	146214515..146232267	-	0,01	-4,359055975
SLC10A7	4	complement(146253975..14652237	0,60	0,66	0,090983797
POU4F2	4	146638893..146642474	-	-	#N/D
TTC29	4	complement(146706617..14694588	0,02	0,02	-0,034419864
gene:ENSG000	4	147025914..147030072	-	-	#N/D
EDNRA	4	147480917..147544954	0,22	0,17	0,578420105
gene:ENSG000	4	147487971..147496062	0,03	0,01	1,718769773
TMEM184C	4	147617386..147672044	2,37	3,00	-0,117250849
PRMT9	4	complement(147637785..14768416	0,58	1,19	-0,817225819
ARHGAP10	4	147732063..148072776	3,79	5,17	-0,226714007
NR3C2	4	complement(148078762..14844469	0,13	0,30	-0,98959301
gene:ENSG000	4	complement(148819351..14881995	-	0,02	-2,89252005
IQCM	4	complement(149351709..14989623	-	-	0,188027316
DCLK2	4	150078445..150257438	2,40	1,99	0,492654348
LRBA	4	complement(150264435..15101572	2,78	4,21	-0,375998087
MAB21L2	4	150582151..150584693	0,16	0,52	-1,513693554
RPS3A	4	151099624..151104642	298,62	#####	0,174517716
SH3D19	4	complement(151102751..15132560	5,40	7,08	-0,167543014
PRSS48	4	151277161..151291862	0,14	0,06	1,524737598
FHIP1A	4	151409176..151670503	1,64	1,26	0,595882208
GATB	4	complement(151670504..15176100	4,62	5,20	0,051562764
FBXW7	4	complement(152320544..15253609	0,66	0,79	-0,021860417
TMEM154	4	complement(152618628..15268001	0,48	0,80	-0,51200864
TIGD4	4	complement(152769354..15277973	0,00	0,05	-3,171495545
ARFIP1	4	152779937..152918463	4,12	3,29	0,550938589
FHDC1	4	152936323..152979671	0,09	0,31	-1,502135627
TRIM2	4	153152163..153339319	0,48	0,92	-0,716964382
gene:ENSG000	4	153152435..153415081	0,23	0,82	-1,601483858
MND1	4	153344649..153415118	0,19	0,53	-1,249300337
TMEM131L	4	153466346..153636711	1,16	3,10	-1,196502487
TLR2	4	153684050..153706260	0,30	0,32	0,110137839
RNF175	4	complement(153710160..15376002	0,08	0,23	-1,294428504
SFRP2	4	complement(153780591..15378908	-	0,01	-2,89252005
DCHS2	4	complement(154231742..15449179	0,10	0,06	1,015680915
PLRG1	4	complement(154535005..15455040	3,50	6,02	-0,558911861
FGB	4	154563011..154572807	-	0,00	-2,89252005
FGA	4	complement(154583126..15459074	-	-	0,188027316
FGG	4	complement(154604134..15461296	-	-	0,188027316
LRAT	4	154626945..154753120	0,03	0,06	-0,711958745
RBM46	4	154781213..154828813	0,01	0,02	-1,54862452

NPY2R	4	155208636..155217078	0,00	-	3,068695355
MAP9	4	complement(155342658..15537697	0,60	0,96	-0,46321315
GUCY1A1	4	155666726..155737059	0,00	0,04	-4,644601042
GUCY1B1	4	155758992..155807811	1,92	2,60	-0,216653811
ASIC5	4	complement(155829729..15586627	-	-	0,188027316
TDO2	4	155854738..155920406	0,02	0,78	-4,819887118
CTSO	4	complement(155921580..15595391	0,17	0,23	-0,215523701
PDGFC	4	complement(156760454..15697179	5,82	6,67	0,026822796
gene:ENSG000	4	156876435..156876820	-	-	0,188027316
gene:ENSG000	4	156886645..156886862	0,05	-	3,068695355
GLRB	4	157076125..157172090	0,25	1,22	-2,085793402
GRIA2	4	157204182..157387146	-	-	0,188027316
gene:ENSG000	4	complement(157818101..15781857	-	-	#N/D
gene:ENSG000	4	complement(157945817..15794664	-	-	#N/D
gene:ENSG000	4	complement(158109500..15811015	-	-	#N/D
gene:ENSG000	4	complement(158111361..15811193	-	-	#N/D
GASK1B	4	complement(158124474..15817331	0,05	0,15	-1,38304323
TMEM144	4	158201604..158255416	0,27	0,54	-0,756857929
RXFP1	4	158315311..158653372	0,07	-	7,865233509
C4orf46	4	complement(158666675..15867225	18,18	5,45	1,962069561
ETFDH	4	158671968..158710742	5,12	1,38	2,115495011
PPID	4	complement(158709127..15872339	60,54	7,64	3,208821026
FNIP2	4	158769026..158908050	1,55	1,78	0,022293682
SPMIP2	4	complement(158893134..15903876	-	-	0,188027316
RAPGEF2	4	159103013..159360174	3,83	5,19	-0,214976041
FSTL5	4	complement(161383897..16216400	0,08	0,01	4,081082838
NAF1	4	complement(163110073..16316689	9,47	14,29	-0,37050585
NPY1R	4	complement(163323962..16334483	0,05	0,06	-0,038943307
NPY5R	4	163343892..163351934	-	-	0,188027316
TKTL2	4	complement(163471095..16347375	0,01	-	3,967268276
TMA16	4	163494442..163520539	3,40	7,11	-0,840743916
MARCHF1	4	complement(163524298..16438405	0,32	0,39	-0,066032107
SMIM31	4	164754064..164803795	-	-	0,188027316
APELA	4	164877178..164898965	-	-	#N/D
TRIM61	4	complement(164954446..16497766	0,00	0,04	-2,854330803
TRIM60	4	165016458..165041749	-	-	#N/D
TRIM75	4	165053866..165060554	0,02	0,07	-1,326441896
TMEM192	4	complement(165070608..16520854	1,70	2,07	-0,064957746
KLHL2	4	165207561..165323156	2,03	3,98	-0,748026799
GK3	4	complement(165277807..16527970	0,10	0,11	0,129542238
MSMO1	4	165327667..165343164	27,73	6,28	2,364961236
CPE	4	165361194..165498547	2,26	1,31	1,011648597
TLL1	4	165873237..166104457	0,04	0,05	-0,238046633
SPOCK3	4	complement(166733384..16723479	0,17	0,04	2,17190734
ANXA10	4	168092537..168187736	0,01	0,01	0,886720911
DDX60	4	complement(168216291..16831888	2,71	3,42	-0,11006391
DDX60L	4	complement(168356735..16853778	0,42	0,99	-1,007738464
PALLD	4	168497052..168928457	11,10	10,33	0,326656117

gene:ENSG000	4	168843988..168844735	0,25	0,35	-0,271714382
CBR4	4	complement(168863770..16901027	2,32	2,72	-0,005003697
SH3RF1	4	complement(169094259..16927095	3,54	5,93	-0,520857211
NEK1	4	complement(169369704..16961263	0,51	0,98	-0,72146066
CLCN3	4	169612633..169723673	2,16	4,77	-0,9203906
HPF1	4	complement(169729470..16975794	2,05	3,95	-0,72394961
MFAP3L	4	complement(169986597..17003303	0,05	0,13	-1,090667122
AADAT	4	complement(170060222..17009169	0,71	1,20	-0,543355098
GALNTL6	4	171812254..173041559	0,05	0,12	-0,985911557
GALNT7	4	173168811..173323967	4,99	3,88	0,587055663
HMGB2	4	complement(173331376..17333443	39,73	28,94	0,680386348
SAP30	4	173369969..173377532	0,65	0,99	-0,387676669
SCRG1	4	complement(173384701..17340638	0,05	0,04	0,671396427
HAND2	4	complement(173524969..17353022	0,46	1,46	-1,437427094
FBXO8	4	complement(174236658..17428366	5,61	5,13	0,354230967
CEP44	4	174283730..174333380	3,36	2,48	0,665633134
HPGD	4	complement(174490175..17452315	0,01	0,00	1,833201638
GLRA3	4	complement(174636920..17482924	0,00	-	3,967268276
ADAM29	4	174829668..174978180	-	-	0,188027316
GPM6A	4	complement(175632934..17600266	0,01	0,21	-4,860182699
WDR17	4	176065841..176182818	0,24	0,71	-1,361901259
SPATA4	4	complement(176184579..17619558	0,01	0,05	-1,573534033
ASB5	4	complement(176213673..17627757	0,00	-	3,068695355
SPCS3	4	176319966..176332245	10,14	10,64	0,152915249
VEGFC	4	complement(176683538..17679292	22,47	8,84	1,569044871
NEIL3	4	177309874..177362936	15,18	9,07	0,966480506
AGA	4	complement(177430774..17744243	1,20	1,37	0,030881081
TENM3	4	182143987..182803024	2,08	6,01	-1,305813999
DCTD	4	complement(182890060..18291793	13,47	20,63	-0,391633671
WWC2	4	183099257..183320777	5,41	6,17	0,034781457
CLDN22	4	complement(183318100..18332027	-	-	#N/D
CLDN24	4	complement(183321764..18332242	0,15	0,14	0,319286298
CDKN2AIP	4	183444635..183449064	2,34	2,37	0,203847685
ING2	4	183505058..183512429	0,79	0,64	0,532882088
RWDD4	4	complement(183639635..18365920	3,02	5,08	-0,528331655
TRAPPC11	4	183659267..183713594	6,43	8,43	-0,167472589
STOX2	4	183797692..184023526	0,36	0,31	0,435781864
gene:ENSG000	4	184014035..184016382	0,03	0,06	-0,678413358
ENPP6	4	complement(184088706..18422123	0,00	0,01	-0,923955849
IRF2	4	complement(184385702..18447455	2,21	3,01	-0,22526545
CASP3	4	complement(184627696..18465006	2,73	3,84	-0,266228035
PRIMPOL	4	184649667..184694963	0,69	1,75	-1,117948334
CENPU	4	complement(184694085..18473413	3,33	5,68	-0,547732108
ACSL1	4	complement(184755595..18482681	3,22	6,07	-0,690682055
gene:ENSG000	4	complement(184984277..18498812	-	-	#N/D
HELT	4	185018490..185020953	-	-	#N/D
SLC25A4	4	185143266..185150382	2,08	2,54	-0,068558532
CFAP97	4	complement(185159665..18520950	2,08	3,95	-0,704171624

SNX25	4	185204237..185370185	1,57	2,16	-0,23120094
LRP2BP	4	complement(185363872..18539592	0,08	0,14	-0,521235018
ANKRD37	4	185396021..185400628	1,03	0,55	1,120775174
UFSP2	4	complement(185399537..18542597	3,25	2,74	0,468570323
CFAP96	4	185426249..185449826	0,06	0,01	2,616997665
CCDC110	4	complement(185445182..18547175	0,01	-	4,516772761
PDLIM3	4	complement(185500660..18553550	0,24	0,68	-1,288891203
SORBS2	4	complement(185585444..18595665	0,00	0,03	-2,476645158
TLR3	4	186068911..186088073	0,16	0,18	0,087268902
FAM149A	4	186104419..186175337	0,20	0,15	0,691759053
FLJ38576	4	complement(186189032..18619149	0,05	0,15	-1,42562615
CYP4V2	4	186191567..186213463	0,43	1,52	-1,583244145
gene:ENSG000	4	186208979..186258469	-	0,01	-2,89252005
KLKB1	4	186226438..186258471	-	-	0,188027316
F11	4	186266189..186289681	-	-	0,188027316
gene:ENSG000	4	complement(186426546..18655532	0,05	0,17	-1,573534033
MTNR1A	4	complement(186526792..18655556	0,00	0,00	0,886720911
FAT1	4	complement(186587794..18672672	9,24	13,98	-0,375236236
ZFP42	4	187995771..188005046	0,01	0,00	0,886720911
TRIML2	4	complement(188091272..18810960	-	0,06	-6,187931073
TRIML1	4	188139441..188147743	-	-	0,188027316
gene:ENSG000	4	complement(188740507..18874128	2,48	3,74	-0,372094779
FRG1	4	189940855..189963202	4,91	5,86	-0,031523766
FRG2	4	complement(190024367..19002725	-	-	0,188027316
DUX4	4	190173774..190185942	-	0,04	-5,071123353
PLEKHG4B	5	92168..189972	0,66	0,09	3,021797743
LRRC14B	5	191495..196334	0,08	0,10	-0,112641019
CCDC127	5	complement(196868..218153)	2,12	2,81	-0,183254678
gene:ENSG000	5	218298..314955	0,69	0,58	0,456781768
SDHA	5	218303..257082	69,51	69,66	0,220012408
PDCD6	5	271621..314974	14,75	17,56	-0,028814129
PDCD6-AHRR	5	271670..438291	0,82	0,92	0,052920537
AHRR	5	321714..438291	12,19	15,28	-0,101918816
EXOC3	5	443175..471937	6,63	12,43	-0,683444208
SLC9A3	5	complement(470456..524449)	3,86	8,09	-0,845168407
CEP72	5	612340..667168	3,68	6,85	-0,671411638
TPPP	5	complement(659862..693352)	0,89	2,06	-0,984044233
ZDHHC11B	5	complement(710355..784729)	0,20	0,37	-0,672302987
ZDHHC11	5	complement(795605..858973)	0,11	0,65	-2,280369121
BRD9	5	complement(850291..892801)	6,68	11,88	-0,607863542
TRIP13	5	892884..919357	26,77	51,94	-0,733250569
NKD2	5	1008802..1038943	15,07	20,90	-0,249384322
SLC12A7	5	complement(1050384..1112063)	22,56	31,67	-0,265941494
SLC6A19	5	1201595..1225111	0,00	-	3,967268276
SLC6A18	5	1225381..1246189	0,01	-	3,967268276
TERT	5	complement(1253147..1295068)	0,29	0,44	-0,382467671
CLPTM1L	5	complement(1317752..1345099)	38,34	66,95	-0,581247405
gene:ENSG000	5	complement(1383159..1386409)	-	0,01	-4,359055975

SLC6A3	5	complement(1392794..1445440)	0,00	0,01	-1,291882393
LPCAT1	5	complement(1456480..1523962)	28,16	32,60	0,011891879
MRPL36	5	complement(1798385..1801366)	13,55	25,22	-0,673013407
NDUFS6	5	1801407..1816048	19,50	42,30	-0,894293248
gene:ENSG000	5	complement(1858409..1859890)	-	-	#N/D
IRX4	5	complement(1877413..1887236)	0,02	-	5,480421266
IRX2	5	complement(2745845..2751677)	1,77	4,39	-1,090287084
IRX1	5	3595832..3601403	3,28	8,27	-1,109648288
ADAMTS16	5	5140330..5320304	0,03	1,98	-5,916838842
ICE1	5	5420664..5490220	6,48	13,15	-0,798973258
gene:ENSG000	5	6134303..6137171	-	-	#N/D
MED10	5	complement(6371874..6378571)	14,45	20,64	-0,291595563
UBE2QL1	5	6448859..6496723	0,01	-	4,516772761
NSUN2	5	complement(6599239..6633291)	16,22	27,40	-0,532911617
SRD5A1	5	6633290..6676539	1,53	2,16	-0,278247466
TENT4A	5	6713432..6757044	10,48	13,88	-0,181368545
ADCY2	5	7396138..7830081	0,00	-	4,516772761
CFAP90	5	complement(7830378..7851151)	-	-	0,188027316
MTRR	5	7851186..7906025	8,23	17,74	-0,885811459
FASTKD3	5	complement(7859159..7869037)	1,78	3,69	-0,827850919
gene:ENSG000	5	complement(8080186..8080761)	-	-	#N/D
gene:ENSG000	5	8143618..8143784	-	-	#N/D
gene:ENSG000	5	8458842..8459183	0,32	0,19	0,943435191
gene:ENSG000	5	8572047..8572206	-	-	#N/D
gene:ENSG000	5	8614356..8614696	-	-	#N/D
gene:ENSG000	5	complement(8659764..8660059)	-	-	#N/D
gene:ENSG000	5	complement(8669018..8669250)	-	-	#N/D
gene:ENSG000	5	complement(8685581..8685816)	-	-	#N/D
gene:ENSG000	5	complement(8717684..8717883)	-	-	#N/D
gene:ENSG000	5	8754869..8755159	-	-	#N/D
gene:ENSG000	5	8793875..8794197	-	-	#N/D
gene:ENSG000	5	complement(8980404..8980659)	-	-	#N/D
SEMA5A	5	complement(9035033..9546075)	1,90	1,28	0,794350758
gene:ENSG000	5	complement(9498401..9498775)	0,09	0,07	0,524121557
TAS2R1	5	complement(9627347..9712378)	0,01	0,03	-0,820024868
gene:ENSG000	5	10057502..10057807	-	-	#N/D
gene:ENSG000	5	complement(10064243..10064476)	-	0,17	-4,359055975
gene:ENSG000	5	10070531..10070841	-	-	0,188027316
gene:ENSG000	5	10082571..10082772	-	-	0,188027316
gene:ENSG000	5	10168783..10169006	0,05	0,12	-0,923955849
gene:ENSG000	5	complement(10171030..10171214)	-	0,14	-3,804623889
ATPSCKMT	5	complement(10225507..10249897)	0,95	2,42	-1,132074568
CCT5	5	10249929..10266389	47,65	#####	-1,203347345
CMBL	5	complement(10275875..10307902)	9,98	15,04	-0,367505514
gene:ENSG000	5	10342813..10343131	0,03	0,24	-2,447197442
MARCHF6	5	10353695..10440388	14,17	22,97	-0,474591892
ROPN1L	5	10441524..10472029	0,14	0,09	0,800439495
gene:ENSG000	5	10541454..10543192	-	-	#N/D

gene:ENSG000	5	10556582..10556815	-	-	0,188027316
ANKRD33B	5	10564070..10657816	1,62	6,90	-1,867523727
gene:ENSG000	5	complement(10675317..10675575)	0,04	-	3,068695355
gene:ENSG000	5	complement(10676096..10676423)	0,10	0,16	-0,429837071
DAP	5	complement(10679230..10761234)	87,61	90,92	0,169586087
CTNND2	5	complement(10971836..11904446)	0,05	0,02	1,409373041
DNAH5	5	complement(13690328..14011818)	0,93	1,63	-0,585315005
TRIO	5	14143342..14532128	19,57	32,82	-0,523080256
OTULINL	5	14581792..14616180	0,10	0,34	-1,525485139
OTULIN	5	14664664..14699850	5,01	8,33	-0,509529516
ANKH	5	complement(14704800..14871778)	4,92	8,36	-0,541849465
FBXL7	5	15500180..15939795	2,82	1,85	0,836505835
MARCHF11	5	complement(16067139..16180762)	-	-	#N/D
ZNF622	5	complement(16451519..16465800)	12,48	16,83	-0,208912359
RETREG1	5	complement(16473038..16617101)	0,02	0,49	-4,497357634
MYO10	5	complement(16661907..16936288)	22,60	32,44	-0,298458607
BASP1	5	17065598..17276843	25,74	70,89	-1,238736757
LINC02218	5	17444010..17487371	-	-	#N/D
H3Y2	5	complement(17490967..17492076)	-	-	#N/D
TAF11L2	5	17498231..17498827	-	-	#N/D
TAF11L3	5	17518367..17518963	-	-	#N/D
TAF11L4	5	17521801..17522397	-	-	#N/D
TAF11L5	5	17525235..17525831	-	-	#N/D
TAF11L6	5	17528669..17529265	-	-	#N/D
TAF11L7	5	complement(17585162..17585758)	-	-	#N/D
TAF11L8	5	complement(17590364..17590960)	-	-	#N/D
TAF11L9	5	complement(17593798..17594394)	-	-	#N/D
TAF11L10	5	complement(17597232..17597828)	-	-	#N/D
TAF11L11	5	17604177..17605910	0,01	0,01	-0,923955849
TAF11L12	5	17610392..17612111	-	0,04	-5,071123353
TAF11L13	5	17632088..17632684	-	-	0,188027316
TAF11L14	5	17634460..17635053	-	-	#N/D
H3Y1	5	complement(17654868..17655849)	-	-	0,188027316
CDH18	5	complement(19471296..20575873)	0,02	0,09	-1,991746904
CDH12	5	complement(21750673..22853344)	0,01	0,03	-1,723418138
PRDM9	5	23443586..23528093	-	-	#N/D
CDH10	5	complement(24487100..24644978)	0,02	0,12	-2,379051091
gene:ENSG000	5	25349841..25350073	-	-	#N/D
gene:ENSG000	5	complement(25425412..25425599)	-	-	#N/D
gene:ENSG000	5	25716271..25716715	-	-	#N/D
CDH9	5	complement(26880597..27121150)	-	0,00	-2,89252005
gene:ENSG000	5	29979669..29983114	-	-	0,188027316
CDH6	5	31193686..31329146	84,02	30,23	1,697675156
DROSHA	5	complement(31400494..31532196)	9,83	21,91	-0,933634214
C5orf22	5	31532287..31555053	3,08	9,57	-1,413112666
gene:ENSG000	5	31558573..31559875	0,16	0,35	-0,915873205
PDZD2	5	31639131..32110932	0,72	1,49	-0,82277962
GOLPH3	5	complement(32124716..32174319)	14,70	24,84	-0,533543671

MTMR12	5	complement(32226994..32312987)	4,10	12,36	-1,370606513
ZFR	5	complement(32354350..32444740)	10,02	25,34	-1,115074851
SUB1	5	32531633..32604079	3,00	10,45	-1,577549595
NPR3	5	32689070..32791724	0,06	1,22	-4,158653702
TARS1	5	33440696..33468091	26,11	77,46	-1,345889811
ADAMTS12	5	complement(33523535..33892019)	3,07	15,49	-2,111090766
RXFP3	5	33936386..33938918	-	-	0,188027316
SLC45A2	5	complement(33944623..33984693)	0,00	0,04	-3,171495545
AMACR	5	complement(33986165..34008104)	0,39	1,51	-1,74667269
C1QTNF3-AMA	5	complement(33987174..34124528)	0,12	0,34	-1,29692133
gene:ENSG000	5	complement(33997007..34008084)	0,09	0,16	-0,573028908
C1QTNF3	5	complement(34017858..34043213)	0,09	0,10	0,196117621
gene:ENSG000	5	complement(34105750..34106336)	0,04	-	3,967268276
gene:ENSG000	5	complement(34124162..34124385)	-	-	0,188027316
RAI14	5	34656328..34832612	21,98	46,81	-0,867594367
TTC23L	5	34838833..34900511	0,11	0,16	-0,35077705
RAD1	5	complement(34905260..34918989)	9,49	10,03	0,143779734
BRIX1	5	34915677..34925996	16,15	28,61	-0,601998962
DNAJC21	5	34929559..34958964	1,75	3,35	-0,714307876
AGXT2	5	complement(34998101..35048135)	-	0,01	-2,89252005
PRLR	5	complement(35048756..35230487)	0,00	0,03	-2,488565592
SPEF2	5	35617844..35814611	0,06	0,09	-0,374940916
IL7R	5	35852695..35879603	2,25	3,76	-0,519389806
gene:ENSG000	5	complement(35895689..35896020)	-	-	#N/D
CAPSL	5	complement(35904288..35938779)	-	0,05	-4,758582517
UGT3A1	5	complement(35951006..36001028)	-	-	0,188027316
UGT3A2	5	complement(36035021..36071358)	0,05	0,04	0,53887001
LMBRD2	5	complement(36098407..36151887)	1,04	2,04	-0,745707472
SKP2	5	36151989..36196849	2,63	6,67	-1,118144273
NADK2	5	complement(36192589..36242279)	4,48	7,74	-0,565724376
RANBP3L	5	complement(36246913..36302114)	0,04	0,06	-0,182572847
SLC1A3	5	36596588..36688334	0,36	3,73	-3,139063293
NIPBL	5	36876769..37066413	5,29	11,51	-0,898964949
CPLANE1	5	complement(37106228..37249376)	0,95	2,74	-1,311422342
NUP155	5	complement(37288137..37371106)	11,83	26,08	-0,917648429
WDR70	5	37379285..37753435	5,18	11,30	-0,900355094
GDNF	5	complement(37812677..37840041)	0,03	0,16	-1,929764521
EGFLAM	5	38258409..38465480	0,00	0,02	-1,766505595
LIFR	5	complement(38474668..38608354)	0,52	1,64	-1,446432168
OSMR	5	38845858..38945596	8,16	23,78	-1,320869951
RICTOR	5	complement(38937920..39074402)	4,33	10,22	-1,016078735
FYB1	5	complement(39105252..39274528)	-	0,03	-6,312013662
C9	5	complement(39284140..39371324)	-	0,00	-2,89252005
DAB2	5	complement(39371675..39462300)	9,12	12,55	-0,237626852
TTC33	5	complement(40512333..40755963)	1,52	2,45	-0,466352493
PTGER4	5	40679915..40693735	0,79	1,30	-0,49944968
PRKAA1	5	complement(40759389..40798374)	6,24	12,41	-0,7684976
RPL37	5	complement(40825262..40835222)	22,17	39,28	-0,602301686

CARD6	5	40841308..40860175	1,09	2,53	-0,991451224
C7	5	40909492..41020216	0,01	-	4,516772761
MROH2B	5	complement(40998017..41071342)	-	-	0,188027316
C6	5	complement(41142116..41261438)	-	-	0,188027316
PLCXD3	5	complement(41306952..41510601)	0,27	0,02	3,973992448
OXCT1	5	complement(41730065..41870425)	4,10	16,51	-1,787912234
RIMOC1	5	41904188..41921636	2,62	5,93	-0,953393664
FBXO4	5	41925254..41941743	0,36	1,61	-1,953478014
GHR	5	42423439..42721878	0,37	0,76	-0,815723659
CCDC152	5	42756818..42802439	1,10	0,62	1,06332802
SELENOP	5	complement(42799880..42887392)	0,13	0,14	0,145728619
ANXA2R	5	complement(43039233..43043170)	0,57	1,39	-1,045837523
ZNF131	5	43065176..43192021	2,62	4,75	-0,634000158
NIM1K	5	43192071..43280850	0,06	0,75	-3,527699604
HMGCS1	5	complement(43287470..43313512)	14,88	4,69	1,890089429
CCL28	5	complement(43376645..43412391)	0,37	0,03	3,643664736
TMEM267	5	complement(43444252..43483893)	0,90	3,03	-1,525322757
C5orf34	5	complement(43486709..43515148)	1,70	5,14	-1,374100622
PAIP1	5	complement(43526267..43557758)	11,15	26,52	-1,026587528
gene:ENSG000	5	complement(43586367..43587543)	0,42	0,36	0,437355004
NNT	5	43602692..43707405	3,24	8,20	-1,114693213
FGF10	5	complement(44300247..44389706)	-	0,00	-2,89252005
MRPS30	5	44808947..44820428	8,88	24,84	-1,261193738
HCN1	5	complement(45254948..45696498)	0,01	0,01	0,279559702
EMB	5	complement(50396192..50443248)	0,78	0,63	0,532870396
PARP8	5	50665899..50846519	0,72	1,41	-0,753320698
ISL1	5	51383448..51394730	1,03	0,47	1,370798369
gene:ENSG000	5	51963992..51964445	-	-	#N/D
PELO	5	52787916..52804044	8,75	8,50	0,265261047
ITGA1	5	52787916..52959209	1,03	1,13	0,092986867
ITGA2	5	52989340..53094779	38,54	18,71	1,265653773
MOCS2	5	complement(53095679..53110063)	2,91	3,13	0,113755661
FST	5	53480626..53487134	3,25	6,03	-0,670428527
NDUFS4	5	53560633..53683338	17,45	14,97	0,443414362
ARL15	5	complement(53883942..54310582)	0,67	0,66	0,235790403
gene:ENSG000	5	complement(54286194..54286331)	-	-	#N/D
HSPB3	5	54455699..54456377	0,08	-	5,224730805
SNX18	5	54517759..54546586	4,21	3,08	0,674932421
gene:ENSG000	5	54643557..54645987	0,01	-	3,967268276
ESM1	5	complement(54977867..55022671)	0,45	7,16	-3,775116496
GZMK	5	55024256..55034570	-	-	#N/D
GZMA	5	55102646..55110252	0,01	-	3,068695355
CDC20B	5	complement(55112971..55173177)	0,01	0,00	2,399873901
GPX8	5	55160167..55167297	13,18	10,91	0,494794546
MCIDAS	5	complement(55219564..55227315)	0,11	0,54	-2,065185514
CCNO	5	complement(55231152..55233608)	1,72	3,26	-0,698254717
DHX29	5	complement(55256055..55307694)	6,08	4,75	0,577372904
MTREX	5	55307989..55425579	7,03	7,10	0,209398638

PLPP1	5	complement(55424854..55534969)	5,00	4,00	0,543999154
gene:ENSG000	5	55484403..55485086	0,06	0,11	-0,602143793
SLC38A9	5	complement(55625845..55773194)	0,99	1,27	-0,134327154
DDX4	5	55738017..55817157	-	-	0,188027316
IL31RA	5	55851357..55922854	1,01	1,84	-0,640784765
IL6ST	5	complement(55935095..55995022)	11,15	14,87	-0,191711448
ANKRD55	5	complement(56099680..56233330)	0,02	0,15	-2,331952431
MAP3K1	5	56815549..56896152	1,09	1,43	-0,166681736
SETD9	5	56909260..56925532	0,50	1,01	-0,785442678
MIER3	5	complement(56919602..56971675)	1,35	1,30	0,27798418
GPBP1	5	57173948..57264679	7,40	7,16	0,270794857
ACTBL2	5	complement(57480018..57482811)	0,02	0,14	-2,587642893
PLK2	5	complement(58453982..58460139)	44,87	84,91	-0,697074738
GAPT	5	58491435..58497090	-	-	#N/D
RAB3C	5	58582221..58859394	-	-	#N/D
PDE4D	5	complement(58969038..60522120)	0,73	0,69	0,29552718
DEPDC1B	5	complement(60596912..60700190)	4,16	5,25	-0,112150779
ELOVL7	5	complement(60751791..60844274)	0,07	0,26	-1,739252581
ERCC8	5	complement(60866454..60945073)	0,72	0,88	-0,068683348
NDUFAF2	5	60945177..61154531	0,50	0,48	0,261171565
gene:ENSG000	5	complement(61114432..61116753)	0,14	0,12	0,405347199
SMIM15	5	complement(61157704..61162468)	2,95	2,77	0,31333683
ZSWIM6	5	61332258..61546172	4,51	3,16	0,739358058
KIF2A	5	62306162..62391025	3,65	4,26	-0,000370753
gene:ENSG000	5	62306206..62537249	-	-	0,188027316
DIMT1	5	complement(62347284..62403943)	2,19	2,46	0,053947323
IPO11	5	62403972..62628582	2,93	3,50	-0,033330619
LRRC70	5	62578819..62581446	0,05	0,09	-0,480718451
HTR1A	5	complement(63957874..63962507)	-	-	0,188027316
RNF180	5	64165843..64372869	0,00	-	3,967268276
RGS7BP	5	64506015..64612319	-	-	#N/D
SHISAL2B	5	64690442..64718190	0,03	-	4,516772761
SREK1IP1	5	complement(64718148..64768691)	1,39	1,41	0,202783507
CWC27	5	64766368..65102412	0,31	0,30	0,296473249
ADAMTS6	5	complement(65148738..65481920)	0,63	3,59	-2,295552597
CENPK	5	complement(65517766..65563168)	1,72	1,45	0,466953158
PPWD1	5	65563236..65587549	2,82	2,82	0,220270725
TRIM23	5	complement(65589690..65625975)	2,37	1,40	0,981452936
TRAPPC13	5	65625004..65666233	1,38	1,47	0,127613443
SHLD3	5	65625027..65630928	0,17	0,31	-0,587717084
SGTB	5	complement(65665928..65723035)	3,51	2,98	0,458503059
NLN	5	65722205..65871725	7,29	5,59	0,60629448
ERBIN	5	65883128..66082546	10,29	9,26	0,374407717
SREK1	5	66139971..66183615	2,15	2,29	0,127859244
MAST4	5	66596380..67169593	0,73	1,03	-0,280697733
CD180	5	complement(67179613..67196799)	0,00	-	3,068695355
PIK3R1	5	68215740..68301821	1,12	0,83	0,654806145
SLC30A5	5	69093991..69131069	7,96	5,14	0,855427317

CCNB1	5	69167135..69178245	65,83	58,12	0,402939029
gene:ENSG000	5	complement(69186359..69189511)	0,30	0,38	-0,103841944
CENPH	5	69189574..69210357	8,88	6,27	0,724560788
MRPS36	5	69217760..69230158	0,90	1,06	-0,018282827
CDK7	5	69234795..69277430	8,98	9,25	0,180589835
CCDC125	5	complement(69280175..69332809)	0,42	0,35	0,505612156
AK6	5	complement(69350984..69370013)	0,55	0,85	-0,388127965
TAF9	5	complement(69362026..69370013)	5,23	3,68	0,727878304
RAD17	5	69369293..69414801	2,30	2,78	-0,049196822
MARVELD2	5	69415065..69444330	0,02	0,04	-0,61619184
OCLN	5	69492292..69558104	0,06	0,48	-2,883673516
GTF2H2C	5	69560191..69595221	2,43	2,71	0,064880797
SERF1B	5	70025251..70043113	2,18	2,43	0,071080968
SMN2	5	70049638..70078522	2,83	0,82	2,007124979
SERF1A	5	70900669..70918530	0,29	0,39	-0,179400748
SMN1	5	70925030..70953942	9,44	7,24	0,604318781
NAIP	5	complement(70968166..71025339)	1,23	0,95	0,59169181
GTF2H2	5	complement(71035016..71067689)	1,25	1,37	0,087021161
BDP1	5	71455651..71567820	1,39	1,75	-0,11430102
MCCC2	5	71579531..71658706	7,87	10,83	-0,237353794
CARTPT	5	71719275..71721048	-	-	#N/D
MAP1B	5	72107234..72209565	35,85	27,82	0,588861888
MRPS27	5	complement(72214953..72321717)	8,24	7,57	0,345306252
PTCD2	5	72320367..72368395	1,23	1,46	-0,019327311
ZNF366	5	complement(72439903..72507410)	0,03	0,03	0,042784718
TNPO1	5	72816312..72916733	10,79	11,48	0,133115101
FCHO2	5	72956041..73090522	1,13	1,35	-0,032991179
TMEM171	5	73120569..73131809	2,41	11,55	-2,038950896
TMEM174	5	73173193..73175143	-	-	#N/D
FOXD1	5	complement(73444827..73448777)	5,63	8,38	-0,350601114
BTF3	5	73498408..73505667	47,99	55,23	0,020394171
ANKRA2	5	complement(73552190..73565667)	3,11	4,07	-0,164792864
UTP15	5	73565443..73583380	2,09	2,54	-0,057831367
ARHGEF28	5	73626158..73941993	5,89	7,63	-0,150277068
ENC1	5	complement(74627406..74641424)	2,21	5,15	-0,999596632
HEXB	5	74640023..74722647	17,19	16,48	0,283964972
GFM2	5	complement(74721206..74767147)	7,60	7,52	0,238281714
NSA2	5	74766991..74780113	3,97	3,09	0,587725413
FAM169A	5	complement(74777574..74866966)	0,63	1,16	-0,668236163
GCNT4	5	complement(75025346..75052558)	0,02	0,00	3,122038495
ANKRD31	5	complement(75068275..75236911)	-	0,00	-2,89252005
HMGCR	5	75336329..75364001	9,14	1,97	2,437612072
CERT1	5	complement(75356345..75512138)	0,90	1,04	0,009605424
POLK	5	75511845..75609991	1,43	1,23	0,440953515
ANKDD1B	5	75611182..75681773	-	0,01	-4,359055975
POC5	5	complement(75674124..75717448)	2,77	3,45	-0,095085301
SV2C	5	76083383..76353939	-	0,00	-2,89252005
IQGAP2	5	76403285..76708132	1,49	0,57	1,619772656

F2RL2	5	complement(76615482..76623413)	0,14	0,87	-2,443094593
F2R	5	76716126..76735770	19,83	5,36	2,11158216
F2RL1	5	76818933..76835315	1,05	2,50	-1,035337851
S100Z	5	76850001..76921650	-	-	0,188027316
CRHBP	5	76953045..76981158	-	-	0,188027316
AGGF1	5	77029251..77065234	2,77	2,49	0,37490877
gene:ENSG000	5	77030902..77152155	-	0,67	-9,631483993
ZBED3	5	complement(77072072..77087285)	0,72	1,97	-1,237381712
PDE8B_1	5	77086732..77427124	0,13	0,41	-1,421086527
PDE8B_2	5	77210449..77428256	0,14	0,29	-0,855277625
WDR41	5	complement(77425970..77620611)	6,92	5,29	0,611698214
OTP	5	complement(77628712..77638713)	0,02	0,00	1,833201638
TBCA	5	complement(77691166..77868780)	6,24	6,79	0,10141904
AP3B1	5	complement(78000522..78294762)	4,16	3,28	0,565826565
SCAMP1	5	78360611..78480739	2,14	2,60	-0,055102366
LHFPL2	5	complement(78485215..78770021)	4,92	5,48	0,068083368
ARSB	5	complement(78777209..78986087)	2,11	2,00	0,296364609
DMGDH	5	complement(78997564..79236038)	0,01	0,12	-3,709032983
BHMT2	5	79069767..79090069	0,00	0,01	-0,923955849
BHMT	5	79111809..79132288	0,00	0,06	-3,431345623
JMY	5	79236131..79327211	2,43	2,74	0,047735318
HOMER1	5	complement(79372636..79514134)	1,31	1,90	-0,310280134
TENT2	5	79612120..79688246	4,53	3,83	0,463896017
CMYA5	5	79689836..79800240	0,08	0,10	0,017665628
MTX3	5	complement(79976716..79991262)	4,89	4,54	0,330654306
THBS4	5	79991311..80083287	1,70	1,91	0,048828356
SERINC5	5	complement(80111651..80256048)	6,33	2,89	1,354317339
SPZ1	5	80319625..80321842	-	-	#N/D
ZFYVE16	5	80408013..80483379	1,29	1,32	0,189043025
FAM151B	5	80487969..80542563	0,10	0,09	0,34461079
ANKRD34B	5	complement(80556755..80570280)	-	0,01	-4,359055975
DHFR	5	complement(80626226..80654983)	13,13	10,20	0,587792741
MSH3	5	80654652..80876815	2,18	2,98	-0,225131431
RASGRF2	5	80960363..81230162	2,90	2,82	0,262195635
CKMT2	5	81233320..81266398	0,09	0,08	0,441431721
ZCCHC9	5	81301587..81313297	2,09	2,59	-0,085282209
ACOT12	5	complement(81329996..81394179)	-	-	0,188027316
SSBP2	5	complement(81412804..81751797)	0,23	0,73	-1,441194847
ATG10	5	81972023..82276857	1,03	1,77	-0,565931431
RPS23	5	complement(82273320..82278396)	107,83	#####	0,232111443
TMEM167A	5	complement(83052846..83077863)	13,81	7,76	1,055248695
XRCC4	5	83077498..83353787	1,75	2,54	-0,311362096
VCAN	5	83471618..83582303	4,16	8,46	-0,801137851
HAPLN1	5	complement(83637805..83720855)	-	-	0,188027316
EDIL3	5	complement(83940554..84384880)	2,72	0,68	2,231353839
gene:ENSG000	5	complement(86305233..86306149)	-	-	#N/D
COX7C	5	86617928..86620962	46,26	52,04	0,053101208
RASA1	5	87267883..87391931	10,89	8,57	0,568712176

CCNH	5	complement(87318416..87413029)	4,21	4,35	0,176184186
TMEM161B	5	complement(88189633..88269476)	1,27	1,17	0,340280805
MEF2C	5	complement(88717117..88904257)	1,87	0,99	1,138441248
CETN3	5	complement(90392257..90409766)	0,29	0,57	-0,780012524
MBLAC2	5	complement(90458209..90474771)	0,80	1,39	-0,570913816
POLR3G	5	90471748..90514557	1,02	1,72	-0,529310863
LYSMD3	5	complement(90515611..90529584)	2,47	1,24	1,215147556
ADGRV1	5	90529344..91164437	0,01	0,00	0,604285378
ARRDC3	5	complement(91368631..91383317)	3,39	2,66	0,570831248
NR2F1	5	93583222..93594611	50,64	5,25	3,492857645
gene:ENSG000	5	93600357..93603598	0,01	-	4,913749003
ARB2A	5	complement(93617725..94111699)	3,16	2,88	0,356309005
POU5F2	5	complement(93733220..93741600)	0,07	0,10	-0,334032395
KIAA0825	5	complement(94150851..94618604)	0,06	0,11	-0,728777657
SLF1	5	94618669..94739436	1,11	1,16	0,161987213
MCTP1	5	complement(94703690..95285094)	0,04	1,46	-5,020928004
FAM81B	5	95391366..95450454	-	-	0,188027316
SKIC3	5	complement(95461755..95555050)	9,42	9,96	0,141458404
ARSK	5	95555101..95605102	3,95	2,75	0,745395683
GPR150	5	95620087..95622142	0,01	0,01	-0,01185201
RFESD	5	95646777..95684773	0,17	0,33	-0,750889283
SPATA9	5	complement(95652181..95698711)	0,03	0,05	-0,678413358
RHOBTB3	5	95713522..95824383	35,88	16,89	1,310252936
GLRX	5	complement(95751319..95822726)	15,15	4,88	1,858592015
ELL2	5	complement(95885098..95961851)	18,16	11,17	0,924067761
PCSK1	5	complement(96390333..96434143)	0,07	0,27	-1,634633046
CAST	5	96525267..96779595	16,91	20,93	-0,084616606
ERAP1	5	complement(96760810..96808100)	2,32	4,14	-0,610305149
ERAP2	5	96875986..96919703	0,48	0,48	0,218966179
LNPEP	5	96935394..97037513	4,14	4,70	0,040076468
LIX1	5	complement(97091867..97142753)	-	0,00	-2,89252005
RIOK2	5	complement(97160867..97183247)	1,55	1,57	0,204730841
RGMB	5	98768650..98798643	29,36	22,69	0,594777762
CHD1	5	complement(98853985..98929007)	2,85	4,55	-0,452642685
FAM174A	5	100535338..100586741	3,87	3,34	0,437182866
ST8SIA4	5	complement(100806933..10090328	0,45	0,13	2,028551842
SLCO4C1	5	complement(102233986..10229628	0,01	0,08	-3,432520866
SLCO6A1	5	complement(102371774..10249901	-	-	#N/D
PAM	5	102753981..103029730	30,30	23,10	0,614641634
GIN1	5	complement(103086000..10312013	0,57	0,76	-0,201875293
PPIP5K2	5	103120149..103212799	1,35	1,29	0,297156015
MACIR	5	103258763..103278660	3,32	3,90	-0,009771006
NUDT12	5	complement(103548855..10356279	1,88	1,58	0,468872184
gene:ENSG000	5	104434772..104435499	-	-	#N/D
EFNA5	5	complement(107376894..10767093	24,07	6,21	2,178488794
FBXL17	5	complement(107859035..10838209	2,64	2,21	0,482100913
FER	5	108747841..109196841	2,26	2,22	0,251670916
PJA2	5	complement(109334713..10940997	34,37	14,77	1,441642807

MAN2A1	5	109689927..109869625	3,95	5,56	-0,272071327
TMEM232	5	complement(110289233..11073895	0,01	0,01	0,524121557
SLC25A46	5	110738136..110765161	2,65	2,94	0,074433986
TSLP	5	111070062..111078026	0,15	0,39	-1,178258044
WDR36	5	111092321..111130502	7,31	6,89	0,307819965
CAMK4	5	111223653..111494886	0,27	0,49	-0,619184457
STARD4	5	complement(111496033..11151259	1,40	0,40	2,050738662
NREP	5	complement(111662621..11199746	2,90	1,44	1,23039988
EPB41L4A	5	complement(112142441..11241931	0,48	0,47	0,236189289
APC	5	112707518..112846239	4,01	3,32	0,495027396
gene:ENSG000	5	112827213..112867582	4,61	1,12	2,264088688
SRP19	5	112861188..112898371	1,29	1,23	0,291331219
REEP5	5	complement(112876385..11292228	14,66	13,30	0,363434328
DCP2	5	112976702..113022195	3,52	2,48	0,727934086
MCC	5	complement(113022106..11348882	0,65	1,17	-0,61565921
TSSK1B	5	complement(113432553..11343498	0,02	0,02	0,677647514
YTHDC2	5	113513694..113595285	2,43	3,37	-0,249596237
KCNN2	5	114055926..114496500	0,01	0,07	-2,267318466
TRIM36	5	complement(115124762..11518054	0,16	0,49	-1,38148702
PGGT1B	5	complement(115204012..11526287	1,00	1,06	0,133740542
CCDC112	5	complement(115267190..11529669	0,44	0,72	-0,476279372
FEM1C	5	complement(115520908..11554477	3,99	3,24	0,520609247
TICAM2	5	complement(115578496..11560247	0,44	0,22	1,198355443
TMED7-TICAM2	5	complement(115578642..11562616	12,26	6,60	1,117176364
TMED7	5	complement(115613210..11563299	12,60	8,00	0,878555277
CDO1	5	complement(115804733..11581665	-	-	0,188027316
ATG12	5	complement(115828200..11584183	5,65	4,65	0,504678784
AP3S1	5	115841592..115914081	8,86	9,72	0,088292924
LVRN	5	115962454..116027619	0,12	0,07	1,10774742
ARL14EPL	5	116032324..116060118	-	0,06	-5,902279478
COMMD10	5	116085016..116412762	5,00	6,25	-0,096880265
SEMA6A	5	complement(116443555..11657482	0,17	0,09	1,15731167
DTWD2	5	complement(118836074..11898854	1,17	0,93	0,55991646
DMXL1	5	119037772..119249138	2,29	3,15	-0,237331546
TNFAIP8	5	119268692..119399688	0,95	0,76	0,542078508
HSD17B4	5	119452465..119637199	9,77	9,33	0,290164206
FAM170A	5	119629558..119635822	0,02	-	4,516772761
PRR16	5	120464300..120687332	4,47	0,67	2,965445903
gene:ENSG000	5	121363002..121365314	-	-	#N/D
FTMT	5	121851882..121852833	-	-	#N/D
SRFBP1	5	121961975..122075570	3,24	3,60	0,070976277
LOX	5	complement(122063195..12207841	25,26	11,80	1,32053718
ZNF474	5	122129546..122182658	0,18	0,14	0,542675196
ZNF475	5	122129622..122182658	-	-	0,188027316
SNCAIP	5	122311354..122464219	0,16	0,10	0,868396042
SNX2	5	122775079..122834543	4,82	5,57	0,015745404
SNX24	5	122843439..123029354	3,45	4,97	-0,3029312
PPIC	5	complement(123023250..12303672	17,56	13,00	0,656760812

PRDM6	5	123089241..123194266	0,63	0,28	1,415304379
CEP120	5	complement(123344890..12342359	3,41	6,01	-0,593761697
gene:ENSG000	5	complement(123508736..12350918	0,02	0,03	-0,01185201
CSNK1G3	5	123512177..123617049	5,66	6,44	0,035886721
ZNF608	5	complement(124636913..12474880	2,66	1,20	1,365589924
GRAMD2B	5	126360132..126496494	2,48	2,68	0,110439217
gene:ENSG000	5	126496279..126498604	0,07	0,15	-0,88099304
ALDH7A1	5	complement(126531200..12659536	9,27	7,97	0,442089045
PHAX	5	126600925..126627252	6,94	6,91	0,229495656
SPMIP10	5	126631705..126636284	-	-	#N/D
LMNB1	5	126776623..126837020	18,85	17,24	0,351920943
MARCHF3	5	complement(126867714..12703055	0,45	0,51	0,051798552
C5orf63	5	complement(127045235..12707350	0,82	0,88	0,120176359
gene:ENSG000	5	127073587..127075604	-	-	0,188027316
MEGF10	5	127290796..127465737	-	0,00	-3,804623889
PRRC1	5	127517617..127555085	33,82	13,59	1,537998438
CTXN3	5	127649082..127658630	-	-	#N/D
CCDC192	5	127703361..127941507	-	0,01	-2,89252005
SLC12A2	5	128083766..128189677	1,84	1,86	0,207576039
FBN2	5	complement(128257909..12865918	6,52	5,60	0,443270712
SLC27A6	5	128538013..129033642	0,97	-	11,00362655
ISOC1	5	129094749..129114028	4,57	4,69	0,186485086
ADAMTS19	5	129460281..129738683	0,45	-	10,62739129
MINAR2	5	129748094..129766732	0,03	0,01	2,616997665
CHSY3	5	129904465..130186634	1,84	1,40	0,619228309
HINT1	5	complement(131155383..13122446	16,06	16,49	0,18478603
LYRM7	5	131170944..131205428	1,67	2,18	-0,159752273
CDC42SE2	5	131245493..131398447	3,37	4,22	-0,103152899
gene:ENSG000	5	complement(131261321..13126160	-	-	#N/D
RAPGEF6	5	complement(131423921..13163523	1,84	2,01	0,101262082
gene:ENSG000	5	complement(131425891..13179698	-	0,11	-8,469487915
FNIP1	5	complement(131641714..13179701	5,71	4,65	0,519795041
gene:ENSG000	5	131797415..131797929	0,04	0,10	-0,979341556
MEIKIN	5	complement(131806990..13194569	-	-	#N/D
gene:ENSG000	5	complement(131807143..13201163	-	-	#N/D
ACSL6	5	complement(131949973..13201224	-	-	0,188027316
IL3	5	132060655..132063204	-	-	#N/D
CSF2	5	132073789..132076170	2,19	49,87	-4,285083427
P4HA2	5	complement(132190147..13229531	21,95	20,17	0,344904401
PDLIM4	5	132257696..132273454	13,57	16,69	-0,075112571
SLC22A4	5	132294394..132344190	0,63	1,60	-1,12599766
SLC22A5	5	132369710..132395613	3,58	4,11	0,024253137
gene:ENSG000	5	132410832..132646079	1,92	1,70	0,397214771
IRF1	5	complement(132440440..13250871	10,18	6,62	0,845143658
IL5	5	complement(132541445..13255683	-	-	0,188027316
RAD50	5	132556019..132646349	2,80	5,74	-0,813429088
IL13	5	132656263..132661110	-	-	0,188027316
IL4	5	132673986..132682678	0,03	-	3,967268276

KIF3A	5	complement(132692628..13273763	1,51	1,58	0,15401472
CCNI2	5	132747426..132754403	0,24	0,27	0,010196258
SEPTIN8	5	complement(132750819..13280724	13,38	9,47	0,720840353
SOWAHA	5	132813302..132816786	0,03	0,08	-1,24748841
SHROOM1	5	complement(132822141..13283065	1,96	4,01	-0,809792229
GDF9	5	complement(132861181..13286688	0,33	0,29	0,432713037
UQCRQ	5	132866630..132868847	22,47	20,13	0,381874319
LEAP2	5	132873444..132875046	0,30	0,27	0,37286305
AFF4	5	complement(132875395..13296363	14,18	13,63	0,28013697
ZCCHC10	5	complement(132996985..13302660	1,85	2,03	0,088758872
HSPA4	5	133052013..133106449	25,71	29,31	0,034167309
gene:ENSG000	5	133052769..133052971	0,69	0,64	0,335747576
FSTL4	5	complement(133196455..13361254	0,05	0,07	-0,138778669
gene:ENSG000	5	133457805..133458331	-	-	#N/D
gene:ENSG000	5	complement(133574829..13357529	-	-	#N/D
C5orf15	5	complement(133955510..13396867	16,47	14,02	0,455359543
VDAC1	5	complement(133971871..13400497	129,74	#####	0,418904047
TCF7	5	134114681..134151865	5,76	7,87	-0,22648293
SKP1	5	complement(134148935..13417696	5,64	5,35	0,299878163
gene:ENSG000	5	complement(134157088..13422586	23,73	35,51	-0,358227903
PPP2CA	5	complement(134194035..13422607	16,77	19,66	-0,006310886
gene:ENSG000	5	complement(134196905..13437104	0,18	0,30	-0,488617625
CDKL3	5	complement(134286350..13437104	0,24	0,30	-0,103458017
UBE2B	5	134371184..134392108	3,28	4,01	-0,063449406
gene:ENSG000	5	complement(134394360..13439500	0,25	0,20	0,539899626
CDKN2AIPNL	5	complement(134402065..13441188	15,53	14,51	0,320379332
gene:ENSG000	5	complement(134522613..13452309	0,09	0,08	0,366665713
JADE2	5	134524312..134583230	7,27	10,81	-0,349251922
SAR1B	5	complement(134601149..13464927	7,83	4,18	1,127154235
SEC24A	5	134648785..134727909	18,94	8,87	1,317359362
CAMLG	5	134738495..134752157	10,45	8,24	0,566640626
DDX46	5	134758771..134855133	7,44	6,94	0,323254455
C5orf24	5	134845680..134859735	6,19	5,07	0,511059135
TXNDC15	5	134874371..134901635	6,81	5,88	0,434951147
PCBD2	5	134905120..135007959	0,67	1,14	-0,537025239
gene:ENSG000	5	134905235..134905691	1,06	1,22	0,025242741
CATSPER3	5	134967907..135011696	0,12	0,32	-1,212100133
PITX1	5	complement(135027734..13503481	71,10	26,96	1,62229401
MACROH2A1	5	complement(135334381..13539991	42,16	39,34	0,323152037
DCANP1	5	complement(135444214..13544734	-	-	#N/D
TIFAB	5	complement(135444226..13545235	-	-	#N/D
SLC25A48	5	135476913..135889770	-	0,00	-2,89252005
NEUROG1	5	complement(135534282..13553596	-	-	#N/D
CXCL14	5	complement(135570679..13557927	0,01	-	3,967268276
IL9	5	complement(135892246..13589584	-	-	#N/D
LECT2	5	complement(135922279..13595498	0,03	-	4,913749004
TGFBI	5	136028988..136063818	#######	#####	1,471444572
SMAD5	5	136132845..136188747	8,70	6,58	0,626396008

SMIM32	5	complement(136191468..13619316	0,11	0,21	-0,672666002
TRPC7	5	complement(136212745..13636554	-	0,03	-5,734998842
SPOCK1	5	complement(136975298..13759837	27,10	30,21	0,066715195
KLHL3	5	complement(137617500..13773608	0,15	0,79	-2,198570533
HNRNPA0	5	complement(137745651..13775436	9,19	11,22	-0,065734421
MYOT	5	137867858..137887851	0,02	0,10	-2,084096673
PKD2L2	5	137887968..137942747	0,07	0,11	-0,488718175
FAM13B	5	complement(137937960..13805196	1,75	1,62	0,33448048
WNT8A	5	138083990..138092365	0,01	0,01	0,886720911
NME5	5	complement(138115175..13813944	-	-	0,188027316
BRD8	5	complement(138139770..13817895	11,36	11,94	0,151122977
KIF20A	5	138178719..138187723	64,92	40,19	0,914745985
CDC23	5	complement(138187650..13821334	15,82	13,00	0,506555371
GFRA3	5	complement(138252380..13827462	0,02	0,02	-0,032860829
CDC25C	5	complement(138285269..13833835	6,85	7,26	0,138270498
FAM53C	5	138331935..138349729	15,17	13,80	0,358982784
KDM3B	5	138352685..138437028	16,62	18,09	0,100287898
REEP2	5	138439057..138446969	0,66	3,40	-2,131187459
EGR1	5	138465479..138469303	2,43	2,32	0,292272291
ETF1	5	complement(138506095..13854323	30,32	28,42	0,31617847
HSPA9	5	complement(138553756..13857567	45,53	76,13	-0,518710636
CTNNA1	5	138610967..138935034	67,75	72,42	0,126783169
LRRTM2	5	complement(138868921..13887536	0,20	0,19	0,332366215
SIL1	5	complement(138946724..13929355	13,20	9,84	0,646326173
MATR3_1	5	139273752..139331671	6,05	9,53	-0,43132859
MATR3_2	5	139293674..139331677	15,49	20,60	-0,18853096
PAIP2	5	139341587..139369720	8,05	9,96	-0,085431744
SLC23A1	5	complement(139367196..13938455	0,06	0,07	0,059935958
MZB1	5	complement(139387467..13939008	0,14	0,04	2,012292537
PROB1	5	complement(139390592..13939510	1,93	1,22	0,878870715
SPATA24	5	complement(139396563..13940408	3,10	2,51	0,523209216
DNAJC18	5	complement(139408588..13944449	0,07	0,01	3,309261876
gene:ENSG000	5	complement(139442316..13946270	0,04	-	4,913749003
ECSCR	5	complement(139448560..13946274	6,55	1,27	2,589372795
SMIM33	5	139471004..139473700	0,02	0,03	-0,579815015
STING1	5	complement(139475533..13948293	24,09	17,56	0,678800887
UBE2D2	5	139526431..139628434	14,42	15,19	0,14860146
CXXC5	5	139647299..139683882	16,59	5,52	1,811312981
PSD2	5	139795808..139844466	0,25	0,09	1,775851108
NRG2	5	complement(139846779..14004329	1,16	1,19	0,194897692
PURA	5	140107777..140125619	2,72	1,96	0,691098151
IGIP	5	140125937..140129392	3,29	1,51	1,348089286
CYSTM1	5	140175156..140282052	12,78	13,08	0,189846116
PFDN1	5	complement(140245035..14030311	50,59	39,86	0,566940201
HBEGF	5	complement(140332843..14034660	4,32	7,11	-0,49577117
SLC4A9	5	140360194..140375141	0,01	0,02	-0,999120035
ANKHD1	5	140401814..140539856	14,34	8,34	1,004455199
ANKHD1-EIF4E	5	140401908..140549569	3,57	6,65	-0,676772663

SRA1	5	complement(140537340..14055767	33,60	16,92	1,212763269
EIF4EBP3	5	140547662..140549576	1,21	4,17	-1,554344645
APBB3	5	complement(140558268..14056478	9,64	7,91	0,507808097
gene:ENSG000	5	140564446..140569100	14,10	10,46	0,653844336
SLC35A4	5	140564819..140569100	11,52	9,18	0,551095986
CD14	5	complement(140631728..14063370	0,56	0,21	1,609139139
NDUFA2	5	complement(140638740..14064777	28,72	20,98	0,676364455
TMCO6	5	140639435..140645408	4,49	6,87	-0,389632996
IK	5	140647058..140662480	18,81	15,86	0,469000681
WDR55	5	140664868..140674124	14,28	7,47	1,158265469
DND1	5	complement(140670794..14067357	0,07	0,16	-0,892557502
HARS1	5	complement(140673035..14069153	16,26	13,54	0,487472397
HARS2	5	140691430..140699305	10,81	10,14	0,315308031
ZMAT2	5	140698680..140706686	21,91	15,10	0,759910529
PCDHA1	5	140786136..141012347	0,00	0,00	-0,01185201
PCDHA2	5	140794852..141012347	0,00	0,01	-0,979341556
PCDHA3	5	140801028..141012347	-	0,02	-6,426264264
PCDHA4	5	140807037..141012347	0,00	0,00	0,886720911
PCDHA5	5	140821604..141012347	0,00	0,01	-0,03031053
PCDHA6	5	140827958..141012347	0,00	0,00	-0,01185201
PCDHA7	5	140834248..141012347	-	0,01	-4,359055975
PCDHA8	5	140841187..141012347	-	-	0,188027316
PCDHA9	5	140847772..141012347	-	-	0,188027316
PCDHA10	5	140855883..141012347	0,00	-	4,913749004
PCDHA11	5	140868183..141012347	-	-	0,188027316
PCDHA12	5	140875302..141012347	-	-	0,188027316
PCDHA13	5	140882124..141012347	-	-	0,188027316
PCDHAC1	5	140926299..141012347	-	-	0,188027316
PCDHAC2	5	140966470..141012347	0,05	0,01	2,2519113
PCDHB1	5	141051374..141059346	-	0,00	-4,359055975
PCDHB2	5	141094606..141098703	0,11	0,01	3,708788417
PCDHB3	5	141100473..141103827	0,02	0,00	2,144183439
PCDHB4	5	141121818..141125623	-	-	0,188027316
PCDHB5	5	141135206..141138615	-	0,00	-2,89252005
PCDHB6	5	141150057..141153287	0,00	0,01	-0,923955849
PCDHB7	5	141172644..141176383	-	0,01	-4,359055975
PCDHB8	5	141177790..141182369	0,04	0,01	1,655502569
PCDHB16	5	141182387..141186226	0,09	0,11	-0,039848397
PCDHB9	5	141187127..141191541	0,02	0,04	-0,61619184
PCDHB10	5	141192353..141195647	0,01	0,05	-1,586291974
PCDHB11	5	141199610..141203779	0,01	0,05	-2,394315395
PCDHB12	5	141208697..141212571	0,01	0,00	0,886720911
PCDHB13	5	141213919..141218979	0,01	0,17	-4,054274529
PCDHB14	5	141223343..141227759	0,10	0,28	-1,279718482
gene:ENSG000	5	141223372..141245861	-	-	0,188027316
PCDHB15	5	141245395..141249365	0,00	0,08	-4,538214549
TAF7	5	complement(141259884..14132078	19,44	16,55	0,455170681
SLC25A2	5	complement(141302635..14130404	-	-	0,188027316

PCDHGA1	5	141330514..141512975	0,03	0,06	-0,469698965
PCDHGA2	5	141338760..141512975	0,01	0,03	-1,599259329
PCDHGA3	5	141343829..141512975	-	0,02	-5,327865481
PCDHGB1	5	141350099..141512975	0,11	0,04	1,687772256
PCDHGA4	5	141355021..141512975	0,14	0,20	-0,333505503
PCDHGB2	5	141359994..141512975	0,90	1,19	-0,174024656
PCDHGA5	5	141364162..141512975	0,26	0,16	0,937239725
PCDHGB3	5	141370242..141512975	0,22	0,17	0,63344897
PCDHGA6	5	141373891..141512975	0,13	0,10	0,574044204
PCDHGA7	5	141382739..141512975	0,13	0,08	0,956769225
PCDHGB4	5	141387698..141512975	0,08	0,03	1,479650156
PCDHGA8	5	141390157..141512975	0,06	0,03	0,986573927
PCDHGB5	5	141397947..141512975	0,03	0,03	0,280049548
PCDHGA9	5	141402778..141512975	0,03	0,34	-3,324622276
PCDHGB6	5	141408021..141512975	0,03	0,04	-0,259918184
PCDHGA10	5	141412987..141512975	0,14	0,46	-1,489324697
PCDHGB7	5	141417645..141512975	0,16	0,65	-1,840127166
PCDHGA11	5	141421047..141512975	0,08	0,10	-0,108568732
PCDHGA12	5	141430507..141512975	0,02	0,01	1,07890858
PCDHGC3	5	141475947..141512977	42,60	23,65	1,071972752
PCDHGC4	5	141484997..141512975	0,57	0,19	1,797756065
PCDHGC5	5	141489081..141512975	2,44	0,98	1,54123657
DIAPH1	5	complement(141515016..14161905	46,32	34,25	0,658780056
HDAC3	5	complement(141620876..14163684	34,00	26,30	0,593882763
RELL2	5	141636950..141641064	4,60	4,92	0,125455074
FCHSD1	5	complement(141639302..14165141	4,46	2,76	0,917241243
ARAP3	5	complement(141653401..14168223	9,15	13,45	-0,332327672
PCDH1	5	complement(141853090..14187924	0,21	3,95	-4,026586278
DELE1	5	141923855..141942047	16,54	11,68	0,72559361
PCDH12	5	complement(141943581..14196974	0,69	0,27	1,558670352
RNF14	5	141958328..141990292	5,07	6,52	-0,14072714
GNPDA1	5	complement(141991749..14201304	6,31	4,88	0,593590511
NDFIP1	5	142108779..142154440	10,68	11,74	0,086839209
gene:ENSG000	5	142165767..142168387	0,40	0,30	0,627460269
SPRY4	5	complement(142310427..14232645	15,68	8,32	1,136672581
FGF1	5	complement(142592178..14269807	0,16	0,15	0,290731497
ARHGAP26	5	142770377..143229011	0,93	1,95	-0,850001739
gene:ENSG000	5	complement(142946569..14294710	0,16	0,10	0,939616118
NR3C1	5	complement(143277931..14343551	15,54	8,02	1,176974681
gene:ENSG000	5	complement(143375628..14337588	-	-	0,188027316
gene:ENSG000	5	143406959..143407420	-	0,03	-2,89252005
HMHB1	5	143812161..143820716	-	-	#N/D
YIPF5	5	complement(144158162..14417071	20,19	10,16	1,21369663
KCTD16	5	144170873..144485686	0,05	0,03	0,93472471
PRELID2	5	complement(145471799..14583536	1,03	1,31	-0,129935585
GRXCR2	5	complement(145858521..14593712	-	0,01	-2,89252005
SH3RF2	5	145936578..146081791	1,01	4,77	-2,009387662
PLAC8L1	5	complement(146084313..14610557	0,14	0,53	-1,637978496

LARS1	5	complement(146110566..14618269	17,44	21,73	-0,094005913
gene:ENSG000	5	146203550..146339251	14,82	13,21	0,389550991
RBM27	5	146203605..146289223	5,09	5,37	0,147096773
POU4F3	5	146338839..146341728	0,05	0,04	0,415033091
TCERG1	5	146447311..146511961	18,31	17,97	0,249763501
GPR151	5	complement(146513144..14651619	0,02	0,05	-1,151227584
PPP2R2B	5	complement(146580742..14708478	0,00	-	3,967268276
STK32A	5	147234963..147387855	0,21	0,12	1,066323203
DPYSL3	5	complement(147390808..14751006	104,39	55,69	1,129641032
JAKMIP2	5	complement(147585438..14778277	-	0,00	-2,89252005
SPINK1	5	complement(147824572..14783167	-	0,04	-4,758582517
SCGB3A2	5	147870682..147882191	-	-	0,188027316
C5orf46	5	complement(147880726..14790653	0,85	1,46	-0,558390435
SPINK5	5	148025683..148137382	-	-	0,188027316
SPINK14	5	148168546..148175619	-	-	#N/D
SPINK6	5	148202794..148215137	0,09	-	5,224730805
MARCOL	5	148221650..148243580	-	-	0,188027316
SPINK13	5	148268180..148286255	0,06	0,02	1,704893826
SPINK7	5	148312419..148315922	-	-	#N/D
SPINK9	5	148321203..148339852	-	-	#N/D
FBXO38	5	148383935..148442836	8,35	7,82	0,31855789
HTR4	5	complement(148446042..14867723	0,01	-	5,88622595
ADRB2	5	148826611..148828623	10,56	9,81	0,328278123
SH3TC2	5	complement(148923639..14906316	0,24	0,33	-0,265164028
ABLIM3	5	149141483..149260542	18,00	24,86	-0,242605071
AFAP1L1	5	149271859..149343637	2,57	3,71	-0,305041727
GRPEL2	5	149345430..149354583	8,52	7,67	0,374723258
PCYOX1L	5	149358037..149369653	6,49	5,50	0,463133012
IL17B	5	complement(149371324..14940420	0,02	-	3,068695355
CSNK1A1	5	complement(149492982..14955147	27,52	25,63	0,325732151
ARHGEF37	5	149551947..149634968	1,24	0,63	1,211538876
PPARGC1B	5	149730298..149855022	0,86	1,71	-0,776850162
PDE6A	5	complement(149857953..14994479	0,06	0,08	-0,341127758
SLC26A2	5	149960758..149993455	3,20	2,34	0,677109284
TIGD6	5	complement(149993118..15000065	0,75	1,11	-0,340837323
HMGXB3	5	150000046..150053142	22,74	17,39	0,610073255
CSF1R	5	complement(150053291..15011337	0,25	0,21	0,420031878
PDGFRB	5	complement(150113839..15015587	4,77	4,99	0,157738539
CDX1	5	150166778..150184558	-	-	0,188027316
SLC6A7	5	150190062..150222788	0,01	0,01	1,232079601
CAMK2A	5	complement(150219491..15029009	0,04	0,01	2,193392976
ARSI	5	complement(150296343..15033930	1,27	0,10	3,8741972
TCOF1	5	150357629..150400308	21,14	33,72	-0,450437993
CD74	5	complement(150401637..15041296	0,10	0,18	-0,551135338
RPS14	5	complement(150442635..15044973	154,75	#####	0,157014908
NDST1	5	150485818..150558211	19,44	16,98	0,418180552
SYNPO	5	150601080..150659207	4,92	7,59	-0,403784308
MYOZ3	5	150660882..150679368	0,03	0,12	-1,707946229

RBM22	5	complement(150690792..15070107	17,10	15,81	0,336516457
DCTN4	5	complement(150708440..15075909	14,37	13,74	0,288314484
SMIM3	5	150778757..150796734	34,66	18,17	1,154503041
IRGM	5	150846521..150900736	0,04	0,03	0,627075281
ZNF300	5	complement(150894392..15090498	0,01	0,00	1,436225396
GPX3	5	151020591..151028988	0,67	3,90	-2,31248843
TNIP1	5	complement(151029945..15109357	39,35	57,22	-0,317200527
ANXA6	5	complement(151100706..15115781	103,10	43,21	1,477566744
CCDC69	5	complement(151181052..15122409	0,15	0,43	-1,273095516
GM2A	5	151212150..151270440	12,22	7,40	0,947269689
SLC36A3	5	complement(151276358..15130376	0,00	-	3,068695355
SLC36A2	5	complement(151314972..15134759	0,01	0,00	2,144183439
SLC36A1	5	151437046..151492379	1,53	1,43	0,326777194
FAT2	5	complement(151504092..15159133	0,03	0,02	1,028629528
SPARC	5	complement(151661096..15168697	333,54	#####	1,609503233
ATOX1	5	complement(151742316..15177253	5,58	5,71	0,188786592
G3BP1	5	151771045..151812911	18,70	19,41	0,169229826
GLRA1	5	complement(151822513..15192485	0,09	0,18	-0,678357384
NMUR2	5	complement(152391546..15243336	0,01	-	3,967268276
GRIA1	5	153489615..153813869	-	-	0,188027316
FAM114A2	5	complement(153990148..15403893	2,14	2,19	0,192315634
MFAP3	5	154038959..154220478	6,40	5,02	0,573696355
GALNT10	5	154190730..154420984	5,74	13,54	-1,013481164
SAP30L	5	154445997..154461053	2,82	3,11	0,081170143
HAND1	5	complement(154474972..15447822	3,30	0,50	2,934599125
LARP1	5	154682986..154817605	67,13	62,95	0,31579615
FAXDC2	5	complement(154818492..15485925	0,65	0,49	0,628350396
CNOT8	5	154857553..154876792	13,08	9,09	0,749055034
GEMIN5	5	complement(154887411..15493821	11,51	12,06	0,15519732
MRPL22	5	154941073..154969411	6,95	5,95	0,448085865
KIF4B	5	155013755..155018141	0,02	0,02	0,181652234
SGCD	5	155870344..156767788	0,03	-	7,819657177
PPP1R2B	5	156850295..156852528	-	-	#N/D
TIMD4	5	complement(156919292..15696322	-	-	#N/D
HAVCR1	5	complement(157026742..15706939	-	-	0,188027316
HAVCR2	5	complement(157085422..15714286	0,02	0,01	1,106450337
MED7	5	complement(157137424..15715901	1,80	1,54	0,447566726
ITK	5	157142933..157255185	-	-	0,188027316
GARIN3	5	complement(157161846..15716626	-	-	#N/D
CYFIP2	5	157266079..157395595	0,31	2,22	-2,630374837
gene:ENSG000	5	complement(157341596..15746010	0,10	0,13	-0,110214389
FNDC9	5	complement(157341598..15734567	-	0,01	-3,804623889
ADAM19	5	complement(157395534..15757577	45,68	28,53	0,902276066
NIPAL4	5	157460213..157474722	0,36	0,22	0,97136535
SOX30	5	complement(157625679..15767148	0,10	0,16	-0,464572644
C5orf52	5	157671533..157680158	-	-	0,188027316
THG1L	5	157731420..157741449	2,83	3,93	-0,251894674
LSM11	5	157743712..157760709	3,57	2,14	0,963030082

CLINT1	5	complement(157785743..15785914	26,29	17,41	0,817908917
EBF1	5	complement(158695920..15909991	0,13	0,18	-0,310479774
gene:ENSG000	5	159106380..159108617	-	0,02	-4,359055975
RNF145	5	complement(159157409..15921005	18,74	14,68	0,57571465
UBLCP1	5	159263290..159286036	2,34	3,92	-0,524374987
IL12B	5	complement(159314780..15933086	-	-	0,188027316
ADRA1B	5	159865080..159973012	0,39	0,81	-0,832103457
TTC1	5	160009113..160065543	21,29	23,56	0,076949232
PWWP2A	5	complement(160061801..16011945	2,07	1,73	0,482492039
FABP6	5	160187367..160238735	0,96	2,12	-0,924067845
CCNJL	5	complement(160249083..16034539	0,59	0,82	-0,248052328
C1QTNF2	5	complement(160347754..16037064	0,07	0,21	-1,445715219
FAM200C	5	complement(160393148..16040005	2,69	1,96	0,678644885
SLU7	5	complement(160401641..16042171	6,48	5,80	0,384178868
PTTG1	5	160421855..160428739	142,45	#####	0,728502124
ATP10B	5	complement(160563120..16085221	0,00	0,03	-4,231853224
GABRB2	5	complement(161288429..16154904	-	-	#N/D
GABRA6	5	161547063..161702593	-	-	#N/D
GABRA1	5	161847063..161899981	-	-	0,188027316
GABRG2	5	162000057..162162977	-	-	#N/D
CCNG1	5	163437569..163446151	19,19	14,45	0,632713934
NUDCD2	5	complement(163446526..16346010	1,12	1,33	-0,02505883
HMMR	5	163460203..163491941	4,17	10,88	-1,16025046
MAT2B	5	163503114..163519558	7,09	7,53	0,134826835
TENM2	5	166979029..168264157	1,66	9,16	-2,245039106
gene:ENSG000	5	168128856..168130422	0,04	0,16	-1,676700393
WWC1	5	168291645..168472303	1,73	6,41	-1,663670818
RARS1	5	168486451..168519301	37,63	25,83	0,765858311
FBLL1	5	168529305..168530634	0,13	0,27	-0,841031413
PANK3	5	complement(168548495..16857936	8,12	5,85	0,695700171
SLIT3	5	complement(168661740..16930113	0,20	0,60	-1,352450233
gene:ENSG000	5	complement(169012148..16901314	-	0,01	-2,89252005
SPDL1	5	169583636..169604778	7,52	11,60	-0,402759011
DOCK2	5	169637268..170083382	1,02	0,52	1,177993005
INSYN2B	5	complement(169861303..16998049	2,82	4,57	-0,473357971
FOXI1	5	170105897..170109734	-	-	0,188027316
C5orf58	5	170229104..170252575	-	-	#N/D
LCP2	5	complement(170246233..17029781	-	-	0,188027316
KCNIP1	5	170353487..170736632	-	-	0,188027316
KCNMB1	5	complement(170374671..17038963	0,00	0,01	-0,579815015
GABRP	5	170763350..170814047	-	-	0,188027316
RANBP17	5	170861870..171300015	0,79	0,99	-0,08891014
TLX3	5	171309248..171312139	-	-	0,188027316
NPM1	5	171387116..171411810	115,81	#####	0,188140875
FGF18	5	171419647..171457626	0,07	0,15	-0,855058638
SMIM23	5	171782432..171796126	-	-	#N/D
FBXW11	5	complement(171861549..17200687	18,94	16,72	0,403239951
STK10	5	complement(172042079..17218822	8,82	9,25	0,154180956

EFCAB9	5	172194172..172203454	-	0,02	-2,89252005
UBTD2	5	complement(172209646..17228376	4,52	4,96	0,089276538
SH3PXD2B	5	complement(172325000..17245452	17,72	11,81	0,809065517
gene:ENSG000	5	complement(172609094..17260944	-	-	#N/D
NEURL1B	5	172641263..172691540	8,53	3,92	1,345823487
DUSP1	5	complement(172768096..17277119	40,40	45,56	0,049897677
ERGIC1	5	172834251..172952792	53,29	45,76	0,442763768
RPL26L1	5	172958729..172969771	9,11	11,05	-0,055449444
ATP6V0E1	5	172983771..173035445	18,44	21,03	0,033898741
CREBRF	5	173056352..173139284	1,50	1,04	0,750355635
BNIP1	5	173144442..173164387	8,98	6,40	0,712448484
NKX2-5	5	complement(173232109..17323531	1,82	2,00	0,085451207
STC2	5	complement(173314723..17332844	26,63	19,71	0,657676018
BOD1	5	complement(173607145..17361665	23,33	20,65	0,398901049
CPEB4	5	173888349..173961980	2,54	1,49	0,993196892
C5orf47	5	173973779..174006140	-	-	0,188027316
NSG2	5	174045706..174243501	0,01	-	4,516772761
MSX2	5	174724582..174730896	2,58	1,84	0,710432815
DRD1	5	complement(175440036..17544418	-	-	0,188027316
SFXN1	5	175477062..175529742	16,42	9,87	0,957241557
HRH2	5	175658030..175710756	0,00	0,02	-1,955757882
CPLX2	5	175796310..175884021	0,04	0,03	0,3118704
gene:ENSG000	5	complement(175903874..17590437	0,15	0,03	2,616997665
THOC3	5	complement(175917873..17603468	25,72	21,93	0,45309229
gene:ENSG000	5	176058717..176159271	0,00	0,00	1,436225396
SIMC1	5	176238367..176345991	8,72	9,06	0,168193039
KIAA1191	5	complement(176346062..17636180	43,24	32,90	0,617411572
ARL10	5	176365487..176401865	0,07	0,10	-0,353530367
NOP16	5	complement(176383946..17638892	4,88	5,42	0,071849369
HIGD2A	5	176388751..176389761	63,26	49,14	0,587573849
CLTB	5	complement(176392501..17641653	21,60	21,02	0,262741219
gene:ENSG000	5	176439090..176439238	-	-	#N/D
FAF2	5	176447628..176510074	30,21	24,33	0,535547097
RNF44	5	complement(176526712..17653802	18,29	19,85	0,104683209
CDHR2	5	176542511..176595974	0,14	0,18	-0,126356954
GPRIN1	5	complement(176595802..17661015	9,60	10,36	0,113633347
SNCB	5	complement(176620082..17663055	0,52	0,61	-0,02162583
EIF4E1B	5	176630618..176646644	0,01	0,01	0,524121557
TSPAN17	5	176647387..176659054	21,35	13,92	0,840227061
UNC5A	5	176810519..176880898	1,58	0,11	4,092526095
HK3	5	complement(176880869..17689934	0,01	0,02	-0,429837071
UIMC1	5	complement(176905005..17702263	9,77	7,37	0,630465197
ZNF346	5	177022696..177081189	2,49	2,58	0,174363534
FGFR4	5	177086905..177098144	9,56	5,73	0,962107296
NSD1	5	177131830..177300213	8,79	9,93	0,047515605
RAB24	5	complement(177301198..17730374	8,19	6,52	0,552402479
MXD3	5	complement(177301461..17731275	8,92	4,54	1,197473248
PRELID1	5	177303799..177306949	131,46	#####	0,60698803

LMAN2	5	complement(177315805..17735184	38,48	27,47	0,709343448
RGS14	5	177357924..177372596	1,49	0,75	1,218720674
SLC34A1	5	177379235..177398848	0,01	0,01	0,921097799
PFN3	5	complement(177400109..17740066	-	-	#N/D
F12	5	complement(177402133..17741658	0,80	0,59	0,656305912
GRK6	5	177403204..177442901	15,42	14,27	0,334407804
PRR7	5	177446445..177456286	4,70	4,07	0,43058305
DBN1	5	complement(177456608..17747440	50,92	45,11	0,397862416
gene:ENSG000	5	complement(177476631..17747965	2,28	1,82	0,547369808
PDLIM7	5	complement(177483394..17749760	30,84	29,69	0,277722053
DOK3	5	complement(177501904..17751127	0,28	0,98	-1,588981524
DDX41	5	complement(177511577..17751696	28,15	15,78	1,058157088
FAM193B	5	complement(177519789..17755458	13,42	9,27	0,756161578
TMED9	5	177592203..177597242	132,75	66,89	1,211999038
B4GALT7	5	177600132..177610330	14,65	9,47	0,852736379
FAM153A	5	complement(177707981..17778443	0,01	0,01	1,613114315
PROP1	5	complement(177992235..17799624	0,02	0,04	-0,742377908
FAM153CP	5	178006348..178086529	0,04	0,02	1,173554051
N4BP3	5	178113532..178126081	1,27	2,84	-0,939779173
RMND5B	5	178130996..178150568	3,84	4,33	0,051040459
NHP2	5	complement(178149463..17815389	72,38	49,74	0,76424118
GMCL2	5	complement(178184505..17818737	-	-	0,188027316
HNRNPAB	5	178204533..178211163	51,60	62,95	-0,063749629
PHYKPL	5	complement(178208471..17823280	4,82	3,93	0,517334179
COL23A1	5	complement(178237476..17859039	10,95	0,53	4,586837404
CLK4	5	complement(178602664..17863061	1,08	0,92	0,45565867
MSANTD5	5	complement(178694572..17869776	0,01	-	3,068695355
gene:ENSG000	5	complement(178694605..17872933	-	-	0,188027316
ZNF354A	5	complement(178711512..17873065	0,60	1,37	-0,9643006
ZNF354B	5	178859953..178888122	0,94	1,33	-0,270605806
ZFP2	5	178895898..178933212	0,04	0,11	-1,31418657
ZNF454	5	178941191..178966433	0,00	0,01	-0,01185201
GRM6	5	complement(178977587..17899620	-	0,01	-5,071123353
ZNF879	5	179023804..179035064	0,30	0,41	-0,204358996
ZNF354C	5	179060373..179083977	1,10	0,80	0,674487149
ADAMTS2	5	complement(179110853..17934546	6,68	6,06	0,365090747
gene:ENSG000	5	179522483..179525074	-	-	#N/D
RUFY1	5	179550554..179610012	7,85	7,72	0,246614374
HNRNPH1	5	complement(179614178..17963478	26,88	33,92	-0,112411957
C5orf60	5	complement(179641544..17965843	-	-	0,188027316
CBY3	5	complement(179678560..17968103	-	0,16	-6,187931073
CANX	5	179678628..179731641	73,49	62,73	0,451493534
MAML1	5	179732822..179777283	10,83	10,00	0,338274372
LTC4S	5	179793980..179796647	0,07	0,07	0,221270597
MGAT4B	5	complement(179797597..17980695	51,58	52,38	0,200863367
SQSTM1	5	179806398..179838078	174,19	#####	0,475567139
MRNIP	5	complement(179835133..17986217	6,40	4,58	0,703766024
TBC1D9B	5	complement(179862066..17990789	30,31	25,19	0,489972762

RNF130	5	complement(179911651..18007211	1,45	2,03	-0,260043332
gene:ENSG000	5	complement(180078357..18008169	0,00	-	3,068695355
RASGEF1C	5	complement(180100795..18020921	0,06	0,07	-0,130997631
MAPK9	5	complement(180233143..18029209	7,00	7,83	0,060759283
GFPT2	5	complement(180300698..18035333	11,78	28,89	-1,070678311
CNOT6	5	180494379..180578358	7,28	5,37	0,660917168
SCGB3A1	5	complement(180590105..18059149	-	-	#N/D
FLT4	5	complement(180601506..18064962	0,25	0,26	0,198293473
OR2Y1	5	complement(180739123..18074005	-	-	#N/D
MGAT1	5	complement(180784780..18081565	15,56	8,55	1,086489082
gene:ENSG000	5	180810401..180811384	0,58	1,34	-0,983012395
ZFP62	5	complement(180847611..18086128	3,14	3,24	0,17973782
BTNL8	5	180899077..180950906	-	-	#N/D
BTNL3	5	180988846..181006727	-	-	#N/D
BTNL9	5	181040225..181061521	0,08	0,06	0,598747665
OR2V1	5	complement(181123122..18113116	-	-	0,188027316
OR2V2	5	181147586..181159285	-	0,01	-4,758582517
TRIM7	5	complement(181193924..18120529	1,36	1,98	-0,317951442
TRIM41	5	181222499..181235808	20,15	16,05	0,550861284
RACK1	5	complement(181236897..18124809	292,17	#####	0,346651807
TRIM52	5	complement(181254077..18126115	5,52	3,60	0,837963645
OR4F3	5	181367268..181368262	0,04	-	4,913749003
DUSP22	6	291630..351355	1,38	2,11	-0,39205622
IRF4	6	391739..411443	0,04	0,02	1,101077806
EXOC2	6	complement(485154..693139)	6,88	7,57	0,085409984
HUS1B	6	complement(655939..657100)	0,18	0,28	-0,432646637
FOXQ1	6	1312098..1314758	0,26	1,30	-2,09876579
FOXF2	6	1389576..1395603	1,81	3,69	-0,803257683
FOXC1	6	1609915..1613897	0,52	1,45	-1,251815602
GMDS	6	complement(1623806..2245605)	3,89	5,20	-0,194421419
gene:ENSG000	6	2561947..2564143	0,03	-	5,697545031
MYLK4	6	complement(2663629..2770330)	0,01	0,04	-1,382135565
WRNIP1	6	2765393..2786952	7,75	13,95	-0,623922191
SERPINB1	6	complement(2832332..2841959)	5,57	14,58	-1,166635046
SERPINB9	6	complement(2887270..2903309)	-	0,01	-4,758582517
SERPINB6	6	complement(2948159..2972165)	3,91	6,58	-0,528272436
NQO2	6	2987987..3019755	2,05	3,18	-0,408136371
RIPK1	6	3063824..3115187	0,95	1,56	-0,48750376
BPHL	6	3118374..3153578	0,71	1,12	-0,440701235
TUBB2A	6	complement(3153497..3157544)	6,44	9,21	-0,293849269
TUBB2B	6	complement(3223324..3231730)	0,17	4,43	-4,441631045
PSMG4	6	3231403..3303373	1,31	1,14	0,423320275
SLC22A23	6	complement(3268962..3457050)	1,39	4,21	-1,379733114
PXDC1	6	complement(3722614..3751713)	10,16	13,50	-0,188046277
FAM50B	6	3849373..3851320	0,01	0,01	-0,025382928
gene:ENSG000	6	3982673..3984130	0,23	0,21	0,327607907
PRPF4B	6	4021267..4064983	3,05	4,16	-0,227740799
FAM217A	6	complement(4049434..4087344)	-	0,02	-4,359055975

ECI2	6	complement(4115693..4135597)	7,12	10,68	-0,361635665
CDYL	6	4706159..4955551	3,66	6,28	-0,556346017
RPP40	6	complement(4994717..5004063)	0,85	1,47	-0,563725926
PPP1R3G	6	5085341..5089487	0,24	0,15	0,866489095
LYRM4	6	complement(5103629..5260950)	6,32	5,75	0,357942017
FARS2	6	5261044..5829192	4,21	2,42	1,023777665
NRN1	6	complement(5997999..6007605)	17,70	3,17	2,704120537
F13A1	6	complement(6144084..6321013)	-	-	0,188027316
LY86	6	6588108..6654983	-	-	0,188027316
gene:ENSG000	6	6685148..6686984	0,03	0,16	-2,142515934
RREB1	6	7107597..7251980	14,12	5,83	1,499451181
SSR1	6	complement(7268306..7347446)	9,94	12,72	-0,133210069
CAGE1	6	complement(7326656..7389742)	0,26	0,02	3,771172776
RIOK1	6	7389793..7418037	5,59	7,11	-0,12476925
DSP	6	7541326..7586717	0,40	0,01	5,435289547
SNRNP48	6	7590198..7611967	3,24	4,24	-0,162927593
BMP6	6	7726099..7881728	1,49	2,51	-0,52496067
TXNDC5	6	complement(7881517..7910788)	153,91	#####	0,464522329
BLOC1S5-TXNI	6	complement(7881522..8064364)	0,51	1,10	-0,901517191
BLOC1S5	6	complement(8013567..8064396)	4,06	4,18	0,179571118
EEF1E1-BLOC1	6	complement(8015726..8102530)	0,17	-	6,461896167
EEF1E1	6	complement(8073360..8102559)	3,77	4,11	0,09999794
SLC35B3	6	complement(8411435..8435561)	2,25	1,90	0,462283035
TFAP2A	6	complement(10393186..10419659)	11,46	5,34	1,324522167
GCNT2	6	10492223..10629368	3,52	4,10	0,006132496
C6orf52	6	complement(10671418..10694797)	0,35	2,15	-2,385866638
PAK1IP1	6	10694972..10709782	9,49	14,04	-0,340861958
TMEM14C	6	10722915..10731129	29,54	39,75	-0,204904465
TMEM14B	6	10747759..10852753	7,40	6,68	0,370227418
gene:ENSG000	6	10747794..10930423	0,15	0,09	0,951907113
MAK	6	complement(10762723..10838553)	0,25	0,22	0,397346413
GCM2	6	complement(10873223..10882041)	0,06	0,07	-0,038353104
SYCP2L	6	10886831..10979320	0,49	1,52	-1,402423491
ELOVL2	6	complement(10980759..11044305)	6,23	9,44	-0,375485924
SMIM13	6	11093834..11138733	3,25	6,60	-0,797216073
ERVFRD-1	6	complement(11102324..11111845)	0,40	0,54	-0,236114041
NEDD9	6	complement(11183298..11382348)	1,74	0,66	1,617901248
TMEM170B	6	11537749..11583524	0,40	0,85	-0,882129993
ADTRP	6	complement(11712054..11807046)	3,13	7,54	-1,04333406
HIVEP1	6	12008762..12164999	5,94	9,99	-0,52815077
EDN1	6	12290361..12297194	3,00	14,20	-2,017486282
PHACTR1	6	12716312..13290446	0,23	0,29	-0,115422038
TBC1D7	6	complement(13266542..13328583)	2,43	3,06	-0,112424358
GFOD1	6	complement(13357830..13487662)	0,37	1,58	-1,862335304
SIRT5	6	13574227..13615158	1,67	3,60	-0,882394346
NOL7	6	13615335..13632739	6,31	11,48	-0,641165508
RANBP9	6	complement(13621498..13711835)	9,68	13,33	-0,238425844
MCUR1	6	complement(13786557..13814568)	5,29	7,09	-0,197469302

RNF182	6	13924446..13980310	3,68	5,15	-0,263379302
CD83	6	14117256..14136918	0,88	2,69	-1,386976918
JARID2	6	15246069..15522042	2,63	3,25	-0,079967357
DTNBP1	6	complement(15522807..15663058)	1,32	2,28	-0,56453902
MYLIP	6	16129086..16148248	1,70	1,47	0,433787963
GMPR	6	16238587..16295549	2,35	3,55	-0,372204443
ATXN1	6	complement(16299112..16761491)	4,71	3,76	0,54796507
gene:ENSG000	6	complement(16328342..16328431)	-	-	#N/D
STMND1	6	17102050..17131378	-	0,01	-2,89252005
RBM24	6	17281361..17293871	1,66	1,75	0,148715264
CAP2	6	17393595..17557780	3,48	6,96	-0,775538902
FAM8A1	6	17600302..17611719	3,07	2,26	0,66326501
NUP153	6	complement(17615035..17706925)	12,88	15,82	-0,072873993
KIF13A	6	complement(17759183..17987635)	2,84	3,87	-0,224881267
NHLRC1	6	complement(18120440..18122677)	0,11	0,03	2,068714833
TPMT	6	complement(18128311..18155077)	1,80	2,34	-0,150220497
KDM1B	6	18155329..18223854	1,94	3,36	-0,573287625
DEK	6	complement(18223860..18264548)	3,96	6,53	-0,499277774
RNF144B	6	18387350..18468870	1,07	1,74	-0,480072106
ID4	6	19837370..19842197	0,17	0,03	2,528640085
MBOAT1	6	complement(20099684..20212469)	2,42	1,07	1,398322029
E2F3	6	20401879..20493714	4,22	5,37	-0,125729718
CDKAL1	6	20534457..21232404	2,36	3,40	-0,301420661
SOX4	6	21593751..21598619	4,86	4,89	0,213884226
gene:ENSG000	6	21822536..21822737	-	-	#N/D
PRL	6	complement(22287244..22302826)	-	-	#N/D
HDGFL1	6	22569566..22571666	-	-	#N/D
NRSN1	6	24126186..24154900	-	0,00	-2,89252005
DCDC2	6	complement(24171755..24358059)	-	0,00	-2,89252005
MRS2	6	24402908..24426194	1,89	3,41	-0,626333389
GPLD1	6	complement(24424565..24495205)	0,04	0,04	0,038295137
ALDH5A1	6	24494867..24537207	0,00	-	3,967268276
KIAA0319	6	complement(24544104..24646191)	0,01	0,08	-3,41605715
TDP2	6	complement(24649979..24666930)	2,47	4,53	-0,652326714
ACOT13	6	24667035..24705065	1,79	2,60	-0,315651167
C6orf62	6	complement(24704861..24720825)	23,34	24,73	0,140034975
GMNN	6	24774931..24786099	3,75	5,73	-0,388119501
ARMH2	6	complement(24797335..24798917)	-	-	0,188027316
gene:ENSG000	6	complement(24797373..24809798)	-	-	0,188027316
RIPOR2	6	complement(24804282..25042170)	0,03	0,05	-0,394019938
CARMIL1	6	25279078..25620530	1,02	1,99	-0,738222659
SCGN	6	25652201..25701783	-	-	#N/D
gene:ENSG000	6	25652210..25738044	-	-	#N/D
H2AC1	6	complement(25723397..25726562)	-	-	0,188027316
H2BC1	6	25726777..25727345	-	-	0,188027316
SLC17A4	6	25754699..25781199	-	-	#N/D
SLC17A1	6	complement(25782915..25832052)	-	-	#N/D
SLC17A3	6	complement(25833066..25882286)	-	-	#N/D

SLC17A2	6	complement(25912754..25930691)	-	-	0,188027316
TRIM38	6	25962802..25991231	2,70	1,70	0,890329758
H1-1	6	complement(26017032..26017787)	0,13	0,10	0,537167373
H3C1	6	26020451..26020958	13,67	39,40	-1,304063853
H4C1	6	26021649..26022050	24,35	44,51	-0,646766696
H4C2	6	complement(26026896..26027283)	59,72	#####	-1,174104783
H3C2	6	complement(26031589..26032099)	65,26	#####	-1,140623264
H2AC4	6	complement(26033092..26033618)	6,96	35,48	-2,126814469
H2BC3	6	complement(26043227..26043713)	3,96	18,66	-2,011404153
H3C3	6	26045384..26045869	41,51	#####	-1,38431292
H1-2	6	complement(26055740..26056470)	65,17	#####	-1,570218153
H2BC4	6	complement(26086317..26123926)	18,30	34,41	-0,688101699
HFE	6	26087226..26098343	0,88	1,22	-0,253462415
H4C3	6	26103933..26104337	59,08	#####	-1,300434254
H1-6	6	complement(26107412..26108135)	-	0,50	-7,525269081
gene:ENSG000	6	26123173..26554396	0,11	0,19	-0,597061533
H2AC6	6	26124145..26139116	6,17	19,05	-1,402287047
H1-4	6	26156329..26157115	67,19	#####	-1,345258925
gene:ENSG000	6	26157122..26533457	0,04	-	13,30230985
H2BC5	6	26158122..26171349	38,55	81,04	-0,848869556
H2BC6	6	26172059..26184655	4,25	7,81	-0,653761598
H4C4	6	complement(26188710..26189112)	31,44	76,27	-1,055430702
gene:ENSG000	6	complement(26195595..26199293)	5,32	16,89	-1,443245908
H3C4	6	complement(26196784..26197286)	8,30	8,24	0,233360015
H2AC7	6	complement(26198784..26199293)	1,65	14,75	-2,93530491
H2BC7	6	26199516..26199988	31,50	#####	-1,473188814
H4C5	6	26204610..26205021	45,14	90,06	-0,773261286
H2BC8	6	complement(26216200..26216688)	25,18	90,23	-1,618103919
H2AC8	6	26216921..26217437	24,38	#####	-2,045965809
H3C6	6	26224199..26227473	6,60	17,44	-1,177422535
H1-3	6	complement(26234212..26234987)	33,21	#####	-1,977255004
H4C6	6	26240393..26240793	-	-	#N/D
H4C7	6	complement(26246611..26246996)	-	-	#N/D
H3C7	6	complement(26250142..26250635)	41,62	74,93	-0,625037519
H2BC9	6	26251614..26252075	13,23	37,30	-1,271718479
H3C8	6	complement(26270918..26271413)	67,26	#####	-1,206715721
H2BC10	6	26272931..26273412	32,57	78,06	-1,037818183
H4C8	6	complement(26277609..26285638)	14,41	41,28	-1,295023761
BTN3A2	6	26365159..26378320	0,55	1,20	-0,915396813
BTN2A2	6	26383096..26394874	0,71	0,69	0,271293592
BTN3A1	6	26402237..26415208	4,57	6,49	-0,282587913
BTN3A3	6	26440472..26453415	2,78	4,03	-0,31438068
BTN2A1	6	26457904..26476621	4,65	5,22	0,05601529
BTN1A1	6	26500303..26510425	-	0,00	-2,89252005
HMGN4	6	26538366..26546933	8,34	12,77	-0,392045738
ABT1	6	26596953..26600739	4,87	5,54	0,036051473
ZNF322	6	complement(26634383..26659752)	3,20	2,75	0,443938795
H2BC11	6	complement(27125897..27132795)	11,77	37,07	-1,431742412

H2AC11	6	27133043..27133535	40,64	#####	-1,10942402
H4C9	6	27139282..27139678	8,64	53,35	-2,403362639
H2BC12	6	complement(27146361..27146855)	120,87	#####	-0,852259661
H2AC12	6	27147106..27147562	24,25	74,00	-1,386051153
PRSS16	6	27247701..27256624	0,67	1,02	-0,389539493
POM121L2	6	complement(27285903..27312273)	0,00	-	3,068695355
ZNF391	6	27374615..27403908	0,65	1,18	-0,640876104
ZNF184	6	complement(27450743..27473118)	4,75	1,36	2,030472991
H2BC13	6	complement(27807479..27807929)	75,43	64,02	0,459698459
H2AC13	6	27808173..27808667	78,64	79,67	0,204199237
H3C10	6	27810051..27811300	38,51	44,59	0,011446689
H2AC14	6	complement(27814302..27814777)	63,43	73,06	0,019093518
H2BC14	6	27815022..27815489	13,52	46,56	-1,560214544
H4C11	6	27824092..27824480	118,61	#####	0,330425999
H4C12	6	complement(27831174..27831560)	221,30	#####	0,193272392
H2AC15	6	complement(27837880..27838375)	13,07	11,64	0,389381842
H2BC15	6	27838545..27855709	29,56	9,62	1,842829486
H2AC16	6	27865317..27865798	12,62	32,37	-1,135907694
H1-5	6	complement(27866792..27867588)	115,70	#####	0,02928985
H3C11	6	complement(27871845..27872346)	80,33	84,31	0,153307384
H4C13	6	complement(27873148..27873534)	3,34	4,42	-0,177963309
H3C12	6	complement(27890315..27893106)	25,23	59,58	-1,016487481
H2AC17	6	complement(27892699..27893185)	133,04	90,74	0,775182611
H2BC17	6	27893425..27893891	194,64	#####	1,156487189
OR2B2	6	complement(27911185..27912396)	0,04	-	4,913749003
OR2B6	6	27957241..27958182	-	-	0,188027316
OR2B8P	6	complement(28053228..28054165)	-	-	0,188027316
ZNF165	6	28080568..28089563	0,12	0,12	0,292946376
ZSCAN16	6	28107689..28130082	0,30	0,29	0,263681049
ZKSCAN8	6	28141883..28159460	4,22	4,35	0,176731721
ZKSCAN8P1	6	28161769..28169594	0,47	0,53	0,035357676
gene:ENSG000	6	28170845..28172521	0,18	0,33	-0,656211125
ZSCAN9	6	28224886..28233487	1,29	1,83	-0,283000548
ZKSCAN4	6	complement(28241697..28252269)	0,55	0,62	0,052656314
NKAPL	6	28259297..28260958	-	-	0,188027316
ZSCAN26	6	28267058..28278224	1,64	3,04	-0,66443488
gene:ENSG000	6	28267121..28281580	0,06	0,26	-1,859185627
PGBD1	6	28281572..28302549	0,59	1,07	-0,625271011
ZSCAN31	6	complement(28324693..28356271)	1,13	0,27	2,288265149
ZKSCAN3	6	28349947..28369172	0,72	1,00	-0,236695109
ZSCAN12	6	complement(28378955..28399747)	0,13	0,26	-0,729588604
ZSCAN23	6	complement(28431930..28443502)	0,43	0,26	0,957905734
GPX6	6	complement(28503296..28528215)	-	-	#N/D
GPX5	6	28525881..28534955	-	-	0,188027316
SCAND3	6	complement(28570535..28616212)	0,05	0,20	-1,725182339
TRIM27	6	complement(28903002..28923988)	12,35	12,30	0,228716565
ZNF311	6	complement(28994785..29005316)	0,32	1,03	-1,469346838
OR2W1	6	complement(29044213..29045175)	-	-	#N/D

OR2B3	6	complement(29086208..29087313)	-	-	#N/D
OR2J1	6	29099490..29102701	-	-	#N/D
OR2J3	6	29108058..29114770	-	-	#N/D
OR2J2	6	29170907..29175811	-	-	#N/D
OR14J1	6	29301701..29313017	-	-	0,188027316
OR5V1	6	complement(29353749..29431967)	-	-	#N/D
OR12D3	6	complement(29373423..29375291)	-	-	#N/D
OR12D2	6	29395631..29398008	0,01	-	3,068695355
OR12D1	6	29414928..29418905	-	-	#N/D
OR11A1	6	complement(29425504..29457071)	-	-	#N/D
OR10C1	6	29439306..29440977	-	-	#N/D
OR2H1	6	29457155..29464328	0,00	-	3,068695355
MAS1L	6	complement(29486697..29487956)	-	-	#N/D
OR2I1P	6	29550407..29557773	-	-	0,188027316
UBD	6	complement(29555515..29559732)	-	-	0,188027316
GABBR1	6	complement(29555629..29633976)	2,68	4,57	-0,546037802
OR2H2	6	29585121..29590500	0,01	-	4,913749004
MOG	6	29657002..29672372	0,02	-	4,913749003
ZFP57	6	complement(29672483..29681155)	-	0,01	-2,89252005
HLA-F	6	29722775..29738528	1,15	5,09	-1,921730015
HLA-G	6	29826967..29831125	-	0,01	-2,89252005
HLA-A	6	29941260..29949572	36,94	57,73	-0,420998436
POLR1H	6	30058899..30064909	10,58	12,26	0,011299258
PPP1R11	6	30066709..30070333	21,47	16,15	0,633475983
RNF39	6	complement(30070266..30075849)	1,31	0,36	2,064993148
TRIM31	6	complement(30102897..30113090)	0,02	0,03	-0,429837071
TRIM40	6	30136124..30148735	-	-	0,188027316
TRIM10	6	complement(30151943..30161211)	-	0,00	-2,89252005
TRIM15	6	30163206..30172693	-	-	#N/D
TRIM26	6	complement(30184455..30213427)	5,99	9,37	-0,423584256
gene:ENSG000	6	complement(30282349..30286054)	0,62	1,07	-0,569913477
TRIM39	6	30326479..30343729	1,85	2,18	-0,010680753
TRIM39-RPP21	6	30328907..30346854	-	-	0,188027316
RPP21	6	30345131..30346884	10,44	12,49	-0,036258583
HLA-E	6	30489509..30494194	15,09	22,39	-0,346430685
GNL1	6	complement(30541381..30556489)	7,07	8,49	-0,039955132
PRR3	6	30557280..30563723	5,66	8,33	-0,333805222
ABCF1	6	30571393..30597179	13,36	16,91	-0,1167058
PPP1R10	6	complement(30600413..30618612)	4,20	10,69	-1,124498533
MRPS18B	6	30617840..30626395	44,82	52,37	-0,001618715
ATAT1	6	30626842..30646823	3,41	2,47	0,686744297
C6orf136	6	30647039..30653210	4,68	6,09	-0,157412375
DHX16	6	complement(30653119..30673006)	8,90	11,45	-0,140051411
PPP1R18	6	complement(30676389..30687895)	39,92	19,29	1,272159879
NRM	6	complement(30688047..30691420)	10,54	9,74	0,336285221
MDC1	6	complement(30699807..30717447)	9,20	13,50	-0,329964787
TUBB	6	30717435..30725538	443,01	#####	0,183115811
FLOT1	6	complement(30727709..30742732)	37,26	45,12	-0,052997798

IER3	6	complement(30743199..30744548)	156,00	#####	0,07184024
DDR1	6	30876421..30900156	7,49	5,87	0,573884725
GTF2H4	6	30908207..30914106	7,10	7,92	0,066638891
VARS2	6	30914205..30926459	6,42	8,27	-0,141905262
SFTA2	6	complement(30931353..30955636)	-	-	#N/D
MUCL3	6	30934523..30954221	-	-	0,188027316
MUC21	6	30983718..30989903	0,00	-	3,068695355
MUC22	6	31010474..31035402	-	0,00	-2,89252005
C6orf15	6	complement(31111223..31112575)	-	-	0,188027316
PSORS1C1	6	31114750..31140092	0,18	0,07	1,443009573
CDSN	6	complement(31115087..31120446)	0,14	0,17	-0,084060118
PSORS1C2	6	complement(31137534..31139066)	0,11	0,04	1,505976878
CCHCR1	6	complement(31142439..31158238)	8,75	6,49	0,652737366
TCF19	6	31158331..31167159	13,99	8,60	0,924915589
POU5F1	6	complement(31164337..31180731)	0,50	0,42	0,459457806
HLA-C	6	complement(31268749..31272130)	112,50	#####	-0,806803749
HLA-B	6	complement(31353872..31367067)	20,58	#####	-2,09627416
MICA	6	31399784..31415315	11,14	8,54	0,606189548
MICB	6	31494881..31511124	9,30	14,24	-0,392321702
MCCD1	6	31528962..31530232	0,29	0,27	0,299198032
DDX39B	6	complement(31530219..31542448)	35,29	59,95	-0,541271958
ATP6V1G2-DD)	6	complement(31530219..31546608)	0,65	1,47	-0,962199585
ATP6V1G2	6	complement(31544444..31548427)	0,31	0,26	0,449237967
NFKBIL1	6	31546870..31558829	7,96	7,59	0,290885225
LTA	6	31572054..31574324	0,03	0,02	0,677647514
TNF	6	31575565..31578336	0,01	0,02	-1,478387936
LTB	6	complement(31580525..31582522)	0,14	0,01	3,381348802
LST1	6	31586124..31588909	0,08	0,05	0,7939095
NCR3	6	complement(31588895..31593006)	-	0,01	-2,89252005
AIF1	6	31615217..31617021	-	-	#N/D
PRRC2A	6	31620715..31637771	52,28	73,17	-0,261973651
gene:ENSG000	6	31622774..31622860	-	-	#N/D
gene:ENSG000	6	31623874..31625499	-	-	#N/D
BAG6	6	complement(31639028..31652705)	56,59	57,05	0,211189599
APOM	6	31652416..31658210	1,03	0,53	1,167467031
C6orf47	6	complement(31658298..31660778)	4,28	4,34	0,204389673
GPANK1	6	complement(31661228..31666283)	3,03	4,35	-0,299876984
CSNK2B	6	31665227..31670343	36,84	41,13	0,064150239
gene:ENSG000	6	31666102..31673546	1,33	0,82	0,922109187
LY6G5B	6	31669976..31673776	5,61	4,01	0,707181697
LY6G5C	6	complement(31676684..31684040)	0,03	0,16	-2,206266309
ABHD16A	6	complement(31686955..31703356)	2,72	3,87	-0,283560312
LY6G6F	6	31706866..31710679	-	-	#N/D
LY6G6F-LY6G6	6	31706904..31717918	-	-	0,188027316
LY6G6D	6	31715348..31717919	-	-	0,188027316
MPIG6B	6	31718594..31726714	0,13	0,07	1,158302359
LY6G6C	6	complement(31718648..31721746)	-	-	0,188027316
DDAH2	6	complement(31727038..31730617)	7,71	5,62	0,679769451

CLIC1	6	complement(31730581..31739763)	103,74	#####	0,261679185
MSH5	6	31739677..31762676	2,72	4,47	-0,495522945
MSH5-SAPCD1	6	31740020..31764851	1,47	1,37	0,327138251
SAPCD1	6	31762656..31764850	0,15	0,94	-2,45006661
VWA7	6	complement(31765590..31777328)	0,16	0,34	-0,826779163
VARS1	6	complement(31777518..31795752)	32,82	31,67	0,274265135
LSM2	6	complement(31797396..31806966)	29,38	30,68	0,160783578
HSPA1L	6	complement(31809619..31815283)	0,34	0,74	-0,9003408
HSPA1A	6	31815543..31817946	16,87	18,39	0,09915744
HSPA1B	6	31827738..31830254	19,31	20,36	0,146514314
NEU1	6	complement(31857659..31862905)	6,76	5,26	0,584060094
SLC44A4	6	complement(31863192..31879046)	0,04	0,03	0,468811989
EHMT2	6	complement(31879759..31897698)	11,34	16,60	-0,326849129
C2	6	31897785..31945673	0,18	0,14	0,571209622
ZBTB12	6	complement(31899613..31902086)	4,71	5,55	-0,0153732
gene:ENSG000	6	31927698..31952048	-	-	0,188027316
CFB	6	31945650..31952086	1,29	5,72	-1,929342195
NELFE	6	complement(31952087..31959038)	24,37	18,60	0,612529193
SKIC2	6	31959116..31969818	31,91	21,53	0,790873826
DXO	6	complement(31969810..31972290)	7,03	7,34	0,161870751
STK19	6	31971091..31982821	7,33	4,27	1,003745752
C4A	6	31982057..32002681	-	-	0,188027316
C4B	6	32014795..32035418	2,17	1,40	0,856814015
CYP21A2	6	32038327..32041644	0,33	0,47	-0,280938285
TNXB	6	complement(32041153..32115334)	2,43	5,32	-0,906173306
ATF6B	6	complement(32115264..32128253)	15,78	15,33	0,265540833
FKBPL	6	complement(32128707..32130288)	2,30	2,65	0,015789763
PRRT1	6	complement(32148359..32153083)	0,51	0,87	-0,5545259
gene:ENSG000	6	complement(32150495..32154373)	0,30	0,31	0,181581643
PPT2	6	32153441..32163678	8,66	16,64	-0,71927674
PPT2-EGFL8	6	32153845..32171978	1,52	1,79	-0,013278474
EGFL8	6	32164595..32168281	0,43	0,35	0,5160204
AGPAT1	6	complement(32168212..32178096)	14,58	16,31	0,061255232
RNF5	6	32178405..32180793	28,11	21,91	0,582716678
AGER	6	complement(32180968..32184322)	3,28	2,13	0,843602282
PBX2	6	complement(32184733..32190202)	12,56	16,34	-0,156553899
GPSM3	6	complement(32190766..32195523)	0,76	0,71	0,337438408
NOTCH4	6	complement(32194843..32224067)	0,05	0,07	-0,168615427
TSBP1	6	complement(32288526..32371907)	-	0,01	-2,89252005
BTNL2	6	complement(32393339..32407181)	-	-	0,188027316
HLA-DRA	6	32439878..32445046	-	-	0,188027316
HLA-DRB5	6	complement(32517353..32530287)	-	-	0,188027316
HLA-DRB1	6	complement(32577902..32589848)	-	-	0,188027316
HLA-DQA1	6	32628179..32647062	-	-	0,188027316
HLA-DQB1	6	complement(32659467..32668383)	-	-	0,188027316
HLA-DQA2	6	32741391..32747198	-	-	0,188027316
HLA-DQB2	6	complement(32756098..32763532)	-	-	0,188027316
HLA-DOB	6	complement(32812763..32820466)	4,47	3,46	0,594083222

gene:ENSG000	6	complement(32813767..32838822)	3,73	6,49	-0,572955694
TAP2	6	complement(32821833..32838739)	26,59	51,71	-0,736600635
PSMB8	6	complement(32840717..32844679)	41,52	#####	-1,1224006
PSMB9	6	32844136..32859851	12,00	38,19	-1,447478164
TAP1	6	complement(32845209..32853816)	51,57	91,83	-0,609300421
HLA-DMB	6	complement(32934629..32941028)	1,69	0,66	1,576781533
gene:ENSG000	6	complement(32937364..32953122)	0,04	0,25	-2,532772701
HLA-DMA	6	complement(32948613..32969094)	7,48	6,13	0,510493659
BRD2	6	32968594..32981505	218,49	#####	1,25542879
HLA-DOA	6	complement(33004182..33009591)	0,02	0,01	0,278120973
HLA-DPA1	6	complement(33064569..33080775)	0,40	0,22	1,100188052
HLA-DPB1	6	33075936..33089696	1,66	1,14	0,769098186
COL11A2	6	complement(33162681..33192499)	4,68	1,95	1,489572315
RXRB	6	complement(33193588..33200665)	93,31	85,76	0,344856023
SLC39A7	6	33200305..33204439	300,22	#####	0,768640982
HSD17B8	6	33204655..33206831	1,26	1,13	0,382329448
RING1	6	33208500..33212722	58,51	60,50	0,174806554
VPS52	6	complement(33250272..33272047)	45,61	60,27	-0,178986268
RPS18	6	33272075..33276511	#######	#####	-0,027469749
B3GALT4	6	33277123..33284832	1,31	3,39	-1,147634897
WDR46	6	complement(33279108..33289247)	78,44	76,91	0,251457468
PFDN6	6	33289302..33298401	23,78	24,66	0,1703443
RGL2	6	complement(33291654..33298942)	50,97	46,12	0,367209319
TAPBP	6	complement(33299694..33314284)	107,02	99,18	0,332857163
ZBTB22	6	complement(33314406..33317942)	5,73	7,27	-0,120942793
DAXX	6	complement(33318558..33323016)	12,34	17,74	-0,30106382
gene:ENSG000	6	complement(33321386..33329286)	-	-	0,188027316
SMIM40	6	complement(33323628..33329279)	0,09	-	5,224730805
KIFC1	6	33391823..33409896	31,32	24,31	0,589075958
PHF1	6	33410399..33416453	22,42	12,96	1,013317297
CUTA	6	complement(33416442..33418317)	52,84	34,21	0,850162456
SYNGAP1	6	33419661..33453689	3,39	3,83	0,045030887
ZBTB9	6	33453970..33457544	2,65	3,05	0,018857187
BAK1	6	complement(33572547..33580293)	16,35	14,50	0,395813082
ITPR3	6	33620365..33696574	20,00	35,19	-0,591956735
UQCC2	6	complement(33694293..33711727)	12,49	13,80	0,078730587
IP6K3	6	complement(33721662..33746905)	-	-	0,188027316
LEMD2	6	complement(33771202..33789130)	14,23	15,91	0,061576163
MLN	6	complement(33794673..33804003)	-	-	0,188027316
GRM4	6	complement(34018643..34155622)	0,01	0,01	1,106450337
HMGA1	6	34236873..34246231	370,02	#####	-0,069697459
SMIM29	6	complement(34246381..34249108)	9,52	16,29	-0,551782195
NUDT3	6	complement(34279679..34392669)	3,48	4,59	-0,175168268
RPS10-NUDT3	6	complement(34284887..34426071)	2,74	5,64	-0,816328813
RPS10	6	complement(34417454..34426069)	110,04	#####	0,067519657
PACSIN1	6	34466061..34535229	0,89	0,27	1,938358338
SPDEF	6	complement(34537802..34556333)	0,07	0,11	-0,335149681
ILRUN	6	complement(34587288..34696859)	17,90	16,44	0,34597513

SNRPC	6	34757505..34773857	105,46	37,14	1,728725329
BLTP3A	6	34792083..34877514	9,85	5,61	1,036218817
TAF11	6	complement(34877462..34889192)	12,35	3,55	2,0225212
ANKS1A	6	34889255..35091406	9,14	3,02	1,821074094
TCP11	6	complement(35118071..35148610)	0,14	0,19	-0,17711992
SCUBE3	6	35213956..35253079	2,19	1,52	0,753748182
ZNF76	6	35258909..35295985	5,35	5,57	0,162493844
DEF6	6	35297818..35321771	0,03	0,09	-1,51742441
PPARD	6	35342558..35428191	31,20	35,04	0,055557633
FANCE	6	35452338..35467104	6,42	9,33	-0,315892919
RPL10A	6	35468401..35470785	326,90	#####	-0,054863861
TEAD3	6	complement(35473597..35497079)	12,93	14,09	0,099507798
TULP1	6	complement(35497874..35512896)	0,05	0,19	-1,852021737
FKBP5	6	complement(35573585..35728583)	0,88	1,66	-0,693597753
ARMC12	6	35737032..35749079	0,21	0,29	-0,283656182
CLPSL2	6	35776594..35779552	0,43	0,10	2,244774768
CLPSL1	6	35781019..35793675	-	-	0,188027316
CLPS	6	complement(35794982..35797344)	-	-	#N/D
LHFPL5	6	35797206..35845397	0,78	1,02	-0,164939868
SRPK1	6	complement(35832966..35921342)	11,09	20,31	-0,649799336
SLC26A8	6	complement(35943516..36024868)	0,01	0,03	-1,326441896
MAPK14	6	36027782..36111236	8,74	10,28	-0,011994721
MAPK13	6	36127809..36144524	1,86	2,63	-0,275674015
BRPF3	6	36196744..36232790	7,90	6,28	0,553896116
PNPLA1	6	36243203..36313955	0,03	0,02	0,371589373
BNIP5	6	complement(36315761..36336888)	0,00	-	3,068695355
ETV7	6	complement(36354091..36387800)	0,05	0,10	-0,617784428
PXT1	6	complement(36390551..36442854)	0,02	-	4,913749004
KCTD20	6	36442767..36491143	7,95	10,31	-0,151735741
STK38	6	complement(36493892..36547479)	5,53	8,11	-0,330851978
SRSF3	6	36594353..36605600	8,63	15,01	-0,57497936
CDKN1A	6	36676460..36687337	21,70	8,17	1,63157299
RAB44	6	36697826..36733184	0,01	-	3,967268276
CPNE5	6	complement(36740775..36839444)	0,29	0,06	2,432561492
PPIL1	6	complement(36854827..36874803)	15,34	19,57	-0,12844312
C6orf89	6	36871870..36928964	13,80	10,80	0,576498805
PI16	6	36948263..36964837	0,01	0,02	-0,03031053
MTCH1	6	complement(36965807..36986298)	28,53	26,92	0,306578819
FGD2	6	37005646..37029069	-	0,00	-2,89252005
PIM1	6	37170152..37175428	30,92	32,89	0,133634479
TMEM217	6	complement(37212180..37258155)	0,05	0,09	-0,685624119
TMEM217B	6	complement(37212181..37258144)	0,12	0,05	1,356372188
TBC1D22B	6	37257772..37332970	4,43	4,97	0,056782328
RNF8	6	37353979..37394734	1,68	2,78	-0,49939941
CMTR1	6	37433219..37482827	11,63	12,63	0,10443356
CCDC167	6	complement(37482938..37499893)	25,20	20,51	0,520489623
gene:ENSG000	6	37567716..37571460	0,02	0,03	-0,392761763
MDGA1	6	complement(37630679..37699306)	1,18	0,91	0,598322641

ZFAND3	6	37819727..38154624	23,89	31,58	-0,179257072
BTBD9	6	complement(38168451..38640148)	2,10	2,08	0,233963218
GLO1	6	complement(38675925..38703145)	136,65	52,31	1,608382397
DNAH8	6	38715311..39030792	0,12	-	10,29410543
GLP1R	6	39048781..39091303	0,00	0,01	-0,979341556
SAYSD1	6	complement(39104063..39115193)	2,33	0,94	1,535747454
KCNK5	6	complement(39188971..39229475)	0,67	1,71	-1,123450828
KCNK17	6	complement(39299001..39314461)	-	-	0,188027316
KCNK16	6	complement(39314698..39322968)	-	-	#N/D
KIF6	6	complement(39329990..39725408)	0,01	0,01	-0,291161992
DAAM2	6	39792298..39904877	0,45	0,02	4,823315988
gene:ENSG000	6	39818751..39823227	0,01	-	4,516772761
MOCS1	6	complement(39899578..39934551)	0,52	0,51	0,247265835
LRFN2	6	complement(40391591..40587364)	-	-	0,188027316
UNC5CL	6	complement(41026895..41039221)	0,23	0,43	-0,678323122
OARD1	6	complement(41033627..41097787)	0,61	1,24	-0,811355613
TSPO2	6	41042467..41044337	0,02	0,13	-2,408690112
APOBEC2	6	41053202..41064891	0,20	0,43	-0,884921293
NFYA	6	41072974..41102403	8,07	4,67	1,011569918
TREML1	6	complement(41149337..41154347)	0,01	0,02	-0,923955849
TREM2	6	complement(41158506..41163186)	-	-	#N/D
TREML2	6	complement(41189749..41201149)	-	-	0,188027316
TREML4	6	41228339..41238882	-	0,00	-2,89252005
TREM1	6	complement(41267926..41286682)	0,01	0,03	-1,275310173
NCR2	6	41335608..41350889	-	-	#N/D
gene:ENSG000	6	complement(41381392..41381832)	-	-	#N/D
gene:ENSG000	6	41405819..41406561	-	-	0,188027316
FOXP4	6	41546381..41602384	3,88	6,07	-0,424492558
MDFI	6	41636882..41654244	-	0,01	-2,89252005
TFEB	6	complement(41683978..41736259)	0,84	0,75	0,388133193
PGC	6	complement(41736711..41754109)	-	0,01	-2,89252005
FRS3	6	complement(41770176..41786542)	0,93	1,90	-0,80160576
gene:ENSG000	6	41780349..41790141	0,42	0,54	-0,147519858
PRICKLE4	6	41780782..41787452	0,09	0,24	-1,189836591
TOMM6	6	41787662..41789898	44,65	50,87	0,034911975
USP49	6	complement(41789896..41895375)	1,19	0,88	0,654418356
gene:ENSG000	6	complement(41793314..41921139)	0,38	0,48	-0,086870163
MED20	6	complement(41905354..41921139)	6,02	5,59	0,3280516
BYSL	6	41921499..41933046	10,40	14,02	-0,207719026
CCND3	6	complement(41934934..42050357)	14,32	9,86	0,760545446
TAF8	6	42050513..42087461	3,07	4,41	-0,301615701
C6orf132	6	complement(42092233..42142620)	0,01	0,03	-1,423700593
CIMIP3	6	42155406..42163418	-	-	0,188027316
GUCA1ANB-GU	6	42155406..42180056	-	-	#N/D
GUCA1A	6	42173364..42181338	0,02	0,02	0,278120973
GUCA1B	6	complement(42183284..42194956)	0,64	0,87	-0,2223026
MRPS10	6	complement(42206807..42217861)	10,79	15,51	-0,300221243
TRERF1	6	complement(42224931..42452051)	3,25	6,00	-0,663498591

UBR2	6	42564029..42693505	3,37	3,35	0,2343658
PRPH2	6	complement(42696598..42722597)	0,02	0,08	-1,854949791
TBCC	6	complement(42744498..42746103)	4,16	5,57	-0,197214773
BICRAL	6	42782052..42868558	2,06	3,54	-0,556628589
RPL7L1	6	42879616..42889925	8,57	19,20	-0,941093052
C6orf226	6	complement(42890265..42890821)	1,84	1,95	0,136738565
PTCRA	6	42915989..42925838	-	-	#N/D
CNPY3	6	42929480..42939294	19,13	26,35	-0,238610056
GNMT	6	42960754..42963880	0,19	0,17	0,397821164
PEX6	6	complement(42963865..42979181)	9,48	11,17	-0,013146665
PPP2R5D	6	42984553..43012342	21,03	19,37	0,342039075
MEA1	6	complement(43011143..43016868)	6,33	7,23	0,030945874
KLHDC3	6	43014103..43021298	22,95	23,32	0,200033166
RRP36	6	43021623..43034156	9,22	10,13	0,087163702
CUL7	6	complement(43037617..43053943)	15,09	8,98	0,971322483
KLC4	6	43040777..43075095	5,83	4,81	0,499617449
gene:ENSG000	6	complement(43051066..43058185)	0,38	-	8,927635869
MRPL2	6	complement(43054029..43059438)	22,53	22,08	0,252123724
PTK7	6	43076307..43161719	18,97	27,10	-0,291340616
SRF	6	43171269..43181506	7,70	10,98	-0,28813595
CUL9	6	43182184..43224587	3,50	4,44	-0,120463605
DNPH1	6	complement(43225629..43229481)	4,80	6,05	-0,10935383
TTBK1	6	43243481..43288258	0,00	-	3,068695355
SLC22A7	6	43295694..43305538	-	-	#N/D
CRIP3	6	complement(43299710..43308826)	-	-	#N/D
ZNF318	6	complement(43307134..43369647)	2,70	4,40	-0,482957925
ABCC10	6	43427366..43450427	2,56	3,55	-0,251111074
DLK2	6	complement(43450352..43456632)	0,08	0,12	-0,341646209
TJAP1	6	43477523..43506556	2,64	3,80	-0,301717269
LRRC73	6	complement(43506968..43510346)	0,22	0,01	4,081082838
POLR1C	6	43509702..43562419	2,27	2,79	-0,072685299
YIPF3	6	complement(43511832..43516985)	28,09	17,20	0,930815711
XPO5	6	complement(43522334..43576038)	7,51	13,30	-0,60134665
POLH	6	43576185..43620523	4,10	3,24	0,563457402
GTPBP2	6	complement(43605316..43629264)	10,98	18,65	-0,541327703
MAD2L1BP	6	43629540..43640941	3,47	6,07	-0,585418467
RSPH9	6	43645036..43672600	0,38	0,56	-0,346459826
MRPS18A	6	complement(43671202..43687791)	10,74	14,01	-0,161225536
VEGFA	6	43770184..43786487	27,79	31,67	0,034699168
MRPL14	6	complement(44113451..44127452)	88,85	21,77	2,252154978
TMEM63B	6	44126914..44155519	52,93	9,11	2,761973808
CAPN11	6	44158811..44184401	0,11	-	7,772593938
MYMX	6	44216926..44218234	0,09	0,02	2,616997665
SLC29A1	6	44219553..44234142	42,19	12,36	1,994334414
HSP90AB1	6	44246166..44253888	#######	#####	2,260666358
SLC35B2	6	complement(44254096..44257890)	195,41	20,96	3,443631001
NFKBIE	6	complement(44258166..44265788)	10,41	3,08	1,97930754
TMEM151B	6	44270450..44307506	0,46	0,07	2,87617639

gene:ENSG000	6	44273194..44378957	0,48	0,12	2,249149436
TCTE1	6	complement(44278734..44297698)	0,07	0,00	4,037428367
AARS2	6	complement(44298731..44313347)	19,19	4,89	2,196148422
SPATS1	6	44342650..44380179	0,01	-	4,516772761
CDC5L	6	44387706..44450425	18,55	4,93	2,134433938
gene:ENSG000	6	44551577..44553281	-	-	#N/D
SUPT3H	6	complement(44809317..45377953)	0,00	0,02	-2,665078517
RUNX2	6	45328157..45664349	0,67	4,53	-2,523317226
CLIC5	6	complement(45880827..46080348)	-	0,00	-2,89252005
ENPP4	6	46129989..46146688	0,05	0,06	0,160934243
ENPP5	6	complement(46159185..46170980)	-	-	0,188027316
RCAN2	6	complement(46220736..46491972)	0,02	0,11	-2,207536876
CYP39A1	6	complement(46549580..46652830)	0,22	0,67	-1,400368801
SLC25A27	6	46652915..46678190	1,16	1,45	-0,100638299
TDRD6	6	46687875..46704319	0,01	0,02	-0,916746549
PLA2G7	6	complement(46704201..46735693)	-	0,01	-3,804623889
ANKRD66	6	46746933..46759506	-	-	0,188027316
MEP1A	6	46793389..46839778	0,00	-	3,068695355
ADGRF5	6	complement(46852522..46954943)	0,00	-	3,068695355
ADGRF1	6	complement(46997708..47042350)	0,03	0,05	-0,326616345
TNFRSF21	6	complement(47231532..47309905)	16,59	15,69	0,303579676
CD2AP	6	47477789..47627263	2,01	2,67	-0,183386094
ADGRF4	6	47685864..47722021	-	0,06	-6,630750384
OPN5	6	47781982..47832780	0,00	-	3,967268276
PTCHD4	6	complement(47856673..48111197)	-	-	0,188027316
MMUT	6	complement(49430360..49463253)	6,88	4,64	0,790067575
CENPQ	6	49463370..49493107	1,36	2,63	-0,733213062
GLYATL3	6	49499923..49528078	3,63	3,56	0,25296024
C6orf141	6	49550666..49561907	0,29	0,92	-1,431078651
RHAG	6	complement(49605175..49636839)	-	-	0,188027316
CRISP2	6	complement(49692358..49713590)	-	-	#N/D
gene:ENSG000	6	49721931..49724007	-	-	0,188027316
CRISP3	6	complement(49727376..49744437)	-	-	0,188027316
PGK2	6	complement(49785660..49787285)	-	0,01	-2,89252005
CRISP1	6	complement(49834257..49877096)	-	-	#N/D
DEFB114	6	complement(49960249..49964164)	-	-	#N/D
DEFB113	6	complement(49968677..49969625)	-	-	#N/D
DEFB110	6	complement(50009138..50021994)	-	-	#N/D
DEFB112	6	complement(50042099..50049929)	-	-	#N/D
TFAP2D	6	50713526..50773033	-	-	#N/D
TFAP2B	6	50818723..50847619	-	-	#N/D
PKHD1	6	complement(51615299..52087613)	0,00	0,00	1,487770062
IL17A	6	52186375..52190638	-	-	#N/D
IL17F	6	complement(52236681..52245689)	-	-	#N/D
MCM3	6	complement(52264014..52284881)	154,77	#####	0,796569419
PAQR8	6	52361421..52407777	6,11	5,81	0,295773454
EFHC1	6	52362123..52529886	3,68	1,82	1,243051061
TRAM2	6	complement(52497408..52577060)	80,41	38,34	1,291606101

TMEM14A	6	52671113..52686588	32,89	14,81	1,374377749
GSTA2	6	complement(52750087..52763475)	-	0,05	-5,071123353
GSTA1	6	complement(52791371..52803860)	-	0,01	-2,89252005
GSTA5	6	complement(52831655..52840843)	-	-	#N/D
GSTA3	6	complement(52896639..52909698)	-	-	#N/D
GSTA4	6	complement(52977948..52995304)	2,05	1,69	0,50601315
CILK1	6	complement(53001279..53061824)	1,32	1,45	0,084158117
gene:ENSG000	6	complement(53041266..53041385)	-	-	#N/D
FBXO9	6	53051991..53100873	1,91	2,44	-0,130049644
gene:ENSG000	6	53065602..53065673	-	-	#N/D
GCM1	6	complement(53126961..53148841)	0,02	0,01	0,677647514
ELOVL5	6	complement(53267398..53349179)	22,52	17,39	0,595920393
GCLC	6	complement(53497341..53616970)	1,47	2,11	-0,296977215
KLHL31	6	complement(53647916..53665756)	0,00	0,00	-0,025382928
LRRC1	6	53794497..53924125	0,63	1,05	-0,513000895
MLIP	6	53929982..54266280	0,00	0,01	-1,478387936
TINAG	6	54307859..54390142	-	-	0,188027316
FAM83B	6	54846771..54945099	-	0,00	-2,89252005
HCRTR2	6	55106460..55282617	-	-	0,188027316
GFRAL	6	55327469..55402493	-	-	0,188027316
HMGCLL1	6	complement(55434373..55579197)	-	-	#N/D
BMP5	6	complement(55753653..55875590)	-	-	0,188027316
COL21A1	6	complement(56056590..56394094)	0,07	0,03	1,579466277
DST	6	complement(56457987..56954830)	4,14	5,90	-0,285923832
BEND6	6	56955107..57027346	0,50	0,50	0,225608001
KIAA1586	6	57046532..57055239	0,47	0,80	-0,563725926
ZNF451	6	57086844..57170305	2,17	2,48	0,030646003
BAG2	6	57172326..57189833	1,07	2,45	-0,966808346
RAB23	6	complement(57186992..57222307)	1,53	3,16	-0,828995901
PRIM2	6	57314805..57646850	6,18	7,84	-0,119725596
KHDRBS2	6	complement(61679961..62286225)	-	-	#N/D
FKBP1C	6	63211469..63213027	0,01	0,02	-0,579815015
LGSN	6	complement(63275951..63319983)	-	-	0,188027316
PTP4A1	6	63521746..63583588	3,16	3,43	0,102157253
gene:ENSG000	6	63572480..63583588	1,08	1,02	0,301792876
PHF3	6	63635802..63779336	1,60	2,29	-0,28853663
EYS	6	complement(63719980..65707226)	0,03	0,01	2,48746355
ZC3H11C	6	65301476..65306574	0,06	0,04	0,599973812
ADGRB3	6	68635282..69390571	0,02	0,06	-1,612441833
gene:ENSG000	6	68635890..68635967	-	-	#N/D
LMBRD1	6	complement(69672757..69867236)	1,56	1,25	0,534195331
COL19A1	6	69866556..70212468	0,03	0,06	-0,552210716
COL9A1	6	complement(70216040..70303084)	-	-	#N/D
FAM135A	6	70412941..70561174	0,64	1,11	-0,572442429
SDHAF4	6	70566917..70589569	5,75	6,73	-0,003784636
SMAP1	6	70667776..70862011	3,43	1,89	1,078457793
B3GAT2	6	complement(70856679..70957060)	0,04	0,04	0,14381523
OGFRL1	6	71288811..71309059	2,07	3,44	-0,509627712

gene:ENSG000	6	complement(71386852..71390829)	0,28	0,31	0,0885439
RIMS1	6	71886550..72403150	0,13	0,00	5,158544001
KCNQ5	6	72621792..73198853	0,75	1,85	-1,077055708
gene:ENSG000	6	complement(73209746..73263196)	-	0,04	-5,071123353
KHDC1L	6	complement(73223544..73225770)	0,44	-	7,673595028
KHDC1	6	complement(73241314..73310365)	2,64	2,94	0,06802896
DPPA5	6	complement(73353063..73354276)	-	-	#N/D
KHDC3L	6	73362658..73364171	0,01	-	3,068695355
OOEP	6	complement(73368555..73395133)	0,06	0,13	-0,858027988
DDX43	6	73394828..73417566	-	0,01	-2,89252005
CGAS	6	complement(73413515..73452297)	3,38	4,82	-0,287840533
MTO1	6	73461578..73509236	1,49	1,51	0,207481534
EEF1A1	6	complement(73489308..73525587)	710,39	#####	0,369090329
SLC17A5	6	complement(73593379..73653992)	18,45	10,65	1,015761602
CD109	6	73695785..73828316	17,16	14,00	0,516381412
gene:ENSG000	6	73971346..73973115	0,01	-	3,967268276
COL12A1	6	complement(75084326..75206267)	47,57	7,20	2,948123414
COX7A2	6	complement(75237675..75250323)	42,66	43,50	0,194858547
TMEM30A	6	complement(75252924..75284948)	17,78	20,62	0,009445048
FILIP1	6	complement(75291859..75493800)	0,03	0,07	-1,122719572
SENP6	6	75601509..75718281	1,70	2,67	-0,431269404
MYO6	6	75749201..75919537	3,36	3,83	0,034080367
IMPG1	6	complement(75921114..76072678)	-	0,00	-2,89252005
HTR1B	6	complement(77460924..77463491)	-	-	0,188027316
MEI4	6	77650274..77927045	0,04	0,06	-0,415525303
IRAK1BP1	6	78867551..78946440	0,07	0,13	-0,704873462
PHIP	6	complement(78934419..79078287)	3,08	4,49	-0,317501234
HMGN3	6	complement(79201245..79234689)	2,84	2,01	0,719078521
LCA5	6	complement(79484991..79537458)	1,20	0,97	0,525666066
gene:ENSG000	6	79573877..79575530	0,10	0,06	0,857064368
SH3BGRL2	6	79631329..79703655	0,34	0,78	-0,96448386
ELOVL4	6	complement(79914814..79947553)	0,21	0,22	0,193567768
TTK	6	80003887..80042527	7,51	9,67	-0,141560545
BCKDHB	6	80106647..80346270	2,32	2,10	0,365206793
gene:ENSG000	6	80440730..80441172	0,02	0,15	-2,190455314
TENT5A	6	complement(81491439..81752774)	1,21	1,66	-0,23132078
IBTK	6	complement(82169986..82247754)	11,84	13,55	0,028989396
TPBG	6	82363206..82367420	22,46	21,09	0,313492048
UBE3D	6	complement(82892390..83065841)	0,26	0,57	-0,897439679
DOP1A	6	83067666..83171350	1,23	1,23	0,226561496
PGM3	6	complement(83147324..83193936)	5,89	4,48	0,618215372
RWDD2A	6	83193357..83198935	1,24	1,76	-0,285328336
ME1	6	complement(83210402..83431051)	13,90	20,89	-0,365340706
PRSS35	6	83512534..83525704	-	-	0,188027316
SNAP91	6	complement(83552880..83709691)	0,10	0,02	2,746538262
RIPPLY2	6	83853360..83857515	0,05	0,02	1,436225396
CYB5R4	6	83859656..83967423	0,88	1,25	-0,287547253
MRAP2	6	84033772..84090881	0,45	0,14	1,921539871

CEP162	6	complement(84124241..84227643)	0,17	0,19	0,083292484
TBX18	6	complement(84687351..84764598)	1,44	2,71	-0,690557618
NT5E	6	85449584..85495791	23,22	19,46	0,47778669
gene:ENSG000	6	complement(85498441..85499058)	0,26	0,08	1,833480694
SNX14	6	complement(85504776..85594156)	1,85	2,76	-0,35887352
gene:ENSG000	6	complement(85504776..85678733)	0,10	0,11	0,029879875
SYNCRIP	6	complement(85607779..85644063)	7,75	11,55	-0,352692806
HTR1E	6	86937528..87016679	-	-	#N/D
CGA	6	complement(87085498..87095106)	-	-	#N/D
ZNF292	6	87151803..87265943	2,38	1,81	0,614638675
GJB7	6	complement(87282980..87329278)	0,02	0,06	-1,326441896
SMIM8	6	87322583..87399749	0,20	0,38	-0,668692575
C6orf163	6	87344813..87365463	0,05	0,09	-0,695225492
gene:ENSG000	6	87356831..87358605	0,03	0,08	-1,151227584
CFAP206	6	87407972..87464465	0,03	0,01	1,704893826
gene:ENSG000	6	87408012..87511634	-	0,03	-5,071123353
SLC35A1	6	87470623..87512336	1,58	2,36	-0,355298567
RARS2	6	complement(87513459..87590028)	4,60	5,11	0,071562933
ORC3	6	87590067..87667453	7,24	10,01	-0,243996098
AKIRIN2	6	complement(87674860..87702233)	10,25	15,81	-0,401899487
SPACA1	6	88047841..88066838	-	-	#N/D
CNR1	6	complement(88139864..88166347)	0,01	0,01	-0,602143793
gene:ENSG000	6	88172261..88175762	0,01	-	4,516772761
RNGTT	6	complement(88609897..88963618)	4,35	4,56	0,157058896
gene:ENSG000	6	88964527..88966587	0,04	0,04	0,152452079
PNRC1	6	89080751..89085160	8,81	8,95	0,199426954
SRSF12	6	complement(89095959..89118071)	0,05	0,03	0,868595576
PM20D2	6	89146055..89165565	1,63	4,24	-1,151647036
GABRR1	6	complement(89177504..89231278)	-	-	0,188027316
GABRR2	6	complement(89254464..89315299)	0,32	0,32	0,205737021
UBE2J1	6	complement(89326625..89352722)	9,10	10,39	0,031979139
RRAGD	6	complement(89364616..89412273)	0,97	0,50	1,165824703
ANKRD6	6	89433152..89633834	0,13	0,08	0,940842966
LYRM2	6	complement(89568144..89638749)	1,36	2,01	-0,339723025
MDN1	6	complement(89642498..89819794)	3,69	5,36	-0,314990414
CASP8AP2	6	89829894..89874436	2,15	2,62	-0,064293079
GJA10	6	89894469..89921760	-	-	#N/D
BACH2	6	complement(89926528..90296843)	0,11	0,18	-0,414068839
MAP3K7	6	complement(90513573..90587086)	6,33	6,81	0,116661497
EPHA7	6	complement(93240020..93419687)	0,04	0,02	1,205365598
gene:ENSG000	6	93811825..93813036	-	-	#N/D
gene:ENSG000	6	94397664..94398829	-	-	#N/D
MANEA	6	95577485..95609470	0,76	0,87	0,041164566
FUT9	6	96015974..96215612	-	-	0,188027316
UFL1	6	96521595..96555276	5,10	5,28	0,171915119
FHL5	6	96562548..96618626	-	-	#N/D
GPR63	6	complement(96794125..96837477)	0,38	0,00	7,506609459
NDUFAF4	6	complement(96889315..96897891)	1,35	3,43	-1,119443592

KLHL32	6	96924620..97140754	-	0,00	-2,89252005
MMS22L	6	complement(97142161..97283217)	1,83	3,05	-0,516097209
POU3F2	6	98834574..98839458	0,06	0,01	2,2519113
FBXL4	6	complement(98868535..98948006)	2,24	2,88	-0,143357866
FAXC	6	complement(99271168..99350062)	0,14	0,24	-0,55873135
COQ3	6	complement(99369401..99394195)	3,92	4,18	0,130557764
PNISR	6	complement(99397629..99425331)	7,34	8,35	0,037104623
USP45	6	complement(99432325..99521728)	0,90	1,37	-0,381449863
TSTD3	6	99520976..99589899	0,22	0,43	-0,706992932
CCNC	6	complement(99542387..99568825)	4,22	4,66	0,07757741
PRDM13	6	99606774..99615578	0,01	0,01	-0,025382928
MCHR2	6	complement(99918519..99994247)	0,00	-	3,068695355
SIM1	6	complement(100385009..10046492	3,64	1,19	1,829867959
ASCC3	6	complement(100508194..10088137	8,19	9,38	0,027450093
GRIK2	6	100962701..102081622	0,27	0,22	0,512203632
HACE1	6	complement(104728094..10485991	3,90	3,36	0,439519233
LIN28B	6	104936616..105083332	0,00	0,03	-3,545596224
BVES	6	complement(105096822..10513715	1,19	2,46	-0,818500482
POPDC3	6	complement(105157900..10518001	1,95	4,61	-1,020275639
PREP	6	complement(105273218..10545406	4,69	4,89	0,164025993
PRDM1	6	105993463..106109939	0,40	2,08	-2,152639115
ATG5	6	complement(106045423..10632579	3,11	4,51	-0,311382011
CRYBG1	6	106360717..106572017	1,82	2,30	-0,117424839
RTN4IP1	6	complement(106570771..10662949	2,45	4,21	-0,555317428
QRSL1	6	106629578..106668417	1,96	3,56	-0,637067026
CD24	6	complement(106969831..10697562	0,08	1,34	-3,875577712
MTRES1	6	107028199..107051586	0,84	1,46	-0,578793525
BEND3	6	complement(107065182..10711551	1,16	1,58	-0,227509028
PDSS2	6	complement(107152562..10745956	3,73	3,92	0,152069802
SOBP	6	107490106..107661306	0,76	0,40	1,156142985
gene:ENSG000	6	complement(107697299..10770021	-	-	#N/D
SCML4	6	complement(107702154..10782431	-	-	#N/D
SEC63	6	complement(107867756..10795820	6,65	6,91	0,167424885
OSTM1	6	complement(108029245..10816585	1,60	2,18	-0,221794565
NR2E1	6	108166022..108188805	0,02	0,02	0,627075281
gene:ENSG000	6	108178871..108179317	-	-	0,188027316
SNX3	6	complement(108211222..10826124	20,50	28,83	-0,26882039
AFG1L	6	108294991..108526796	0,91	1,42	-0,412102299
gene:ENSG000	6	complement(108359084..10835986	0,10	0,15	-0,392761763
FOXO3	6	108559835..108684774	11,77	9,60	0,51590911
ARMC2	6	108848416..108974476	0,52	0,44	0,474460604
SESN1	6	complement(108984309..10909484	0,34	0,48	-0,254442735
CEP57L1	6	109095110..109174418	0,80	1,08	-0,200823769
CD164	6	complement(109366514..10938246	15,00	16,43	0,091123477
PPIL6	6	complement(109390215..10944117	0,08	0,17	-0,808080641
SMPD2	6	109440724..109443919	2,89	4,29	-0,347715741
MICAL1	6	complement(109444062..10946596	7,80	10,13	-0,153586889
ZBTB24	6	complement(109460632..10948323	1,85	2,61	-0,277907644

AK9	6	complement(109492855..10969121	0,06	0,09	-0,482804861
FIG4	6	109690609..109878098	0,64	0,91	-0,290300238
GPR6	6	109978256..109980720	-	-	#N/D
WASF1	6	complement(110099819..11018000	3,71	6,51	-0,586257052
CDC40	6	110180141..110254275	3,14	4,02	-0,133884899
METTL24	6	complement(110243940..11035834	0,04	0,02	1,391449417
DDO	6	complement(110391771..11041557	0,46	0,43	0,316931753
SLC22A16	6	complement(110424687..11047664	-	-	#N/D
CDK19	6	complement(110609978..11081595	2,73	3,92	-0,299356125
AMD1	6	110874770..110898879	7,82	10,35	-0,180185517
GTF3C6	6	110958706..110967890	3,11	8,36	-1,205238061
RPF2	6	110982015..111028263	2,87	4,85	-0,533910201
SLC16A10	6	111087503..111231194	0,02	0,05	-1,034421145
MFSD4B	6	111259327..111445354	1,25	1,26	0,215987432
REV3L	6	complement(111299028..11148371	1,92	3,07	-0,454961907
TRAF3IP2	6	complement(111555381..11160690	2,80	5,56	-0,768460054
FYN	6	complement(111660332..11187345	1,07	7,72	-2,631557881
CCN6	6	112054075..112069686	-	0,01	-2,89252005
TUBE1	6	complement(112070663..11208752	2,06	7,90	-1,71800131
FAM229B	6	112087591..112102790	1,51	1,63	0,11204843
LAMA4	6	complement(112107931..11225493	20,19	18,75	0,329834166
gene:ENSG000	6	112236806..112350094	-	0,01	-2,89252005
RFPL4B	6	112347330..112351294	0,01	0,05	-1,766505595
MARCKS	6	113857345..113863475	10,67	24,26	-0,961700855
HDAC2	6	complement(113933028..11401130	4,00	6,05	-0,372264466
HS3ST5	6	complement(114055596..11434302	0,05	0,01	2,570892441
FRK	6	complement(115931149..11606089	0,08	0,13	-0,514501962
NT5DC1	6	116100851..116249497	1,48	1,47	0,234204495
COL10A1	6	complement(116118909..11615874	0,10	0,08	0,603378003
TSPYL4	6	complement(116249964..11625407	6,01	7,21	-0,040238611
DSE	6	116254173..116444861	4,40	5,11	0,005853457
TSPYL1	6	complement(116267760..11627993	5,94	8,04	-0,214698262
gene:ENSG000	6	116399395..116463696	0,32	0,38	-0,039936706
CALHM6	6	116461370..116463771	0,04	0,01	1,833201638
TRAPPC3L	6	complement(116494989..11654568	-	-	0,188027316
CALHM5	6	116511639..116524788	0,08	0,26	-1,45627855
CALHM4	6	116529013..116561127	-	0,01	-4,359055975
RWDD1	6	116571409..116597675	1,78	2,45	-0,23643704
RSPH4A	6	116616479..116632985	-	0,05	-6,312013662
ZUP1	6	complement(116635618..11666879	1,95	2,97	-0,385753107
KPNA5	6	116681187..116741867	0,11	0,21	-0,727193482
FAM162B	6	complement(116752197..11676571	0,03	0,04	-0,03031053
GPRC6A	6	complement(116792085..11682908	-	-	0,188027316
RFX6	6	116877212..116932161	-	-	#N/D
VGLL2	6	117265558..117273565	0,01	-	3,967268276
ROS1	6	complement(117287353..11742594	0,05	0,01	3,030543327
gene:ENSG000	6	complement(117318211..11757357	-	-	#N/D
DCBLD1	6	117453817..117569858	5,76	5,30	0,341462153

GOPC	6	complement(117560269..11760254	3,34	4,71	-0,274700165
NUS1	6	117675469..117710727	10,98	8,23	0,640069038
SLC35F1	6	117907264..118317676	0,01	0,00	1,833201638
CEP85L	6	complement(118460772..11871007	0,72	0,99	-0,232451594
PLN	6	118548296..118561716	0,03	0,08	-1,193454645
MCM9	6	complement(118813442..11893516	0,61	1,65	-1,223305218
ASF1A	6	118894152..118909171	2,09	3,47	-0,508995095
FAM184A	6	complement(118959763..11914938	0,10	0,48	-2,044309497
MAN1A1	6	complement(119177205..11934976	1,18	4,31	-1,641174889
TBC1D32	6	complement(121079494..12133474	0,58	0,97	-0,532096879
GJA1	6	121435595..121449727	2,70	11,97	-1,927605691
HSF2	6	122399551..122433119	3,94	4,86	-0,079818621
gene:ENSG000	6	complement(122436789..12243922	2,07	1,61	0,583068919
SERINC1	6	complement(122443351..12247180	20,37	20,46	0,216834367
PKIB	6	122471917..122726373	0,04	0,02	0,943435191
gene:ENSG000	6	122471923..122484161	0,03	0,04	-0,231893514
FABP7	6	122779716..122784074	-	-	0,188027316
SMPDL3A	6	122789049..122809720	0,17	0,32	-0,652468584
CLVS2	6	122996235..123072925	0,00	-	3,068695355
TRDN	6	complement(123216339..12363718	-	-	0,188027316
NKAIN2	6	123803865..124825640	0,02	0,01	0,750935198
RNF217	6	124962437..125092633	1,24	1,25	0,212988657
TPD52L1	6	125119049..125264407	0,12	0,09	0,61999838
HDDC2	6	complement(125219962..12530207	9,82	16,61	-0,534895042
HEY2	6	125747664..125761269	0,01	-	4,516772761
NCOA7	6	125781161..125932034	2,27	6,64	-1,328684172
HINT3	6	125956770..125980244	2,88	4,32	-0,361429892
TRMT11	6	125986479..126203817	0,92	1,77	-0,725803687
CENPW	6	126340115..126348875	13,56	13,27	0,254746132
RSPO3	6	127118671..127199481	-	0,00	-2,89252005
RNF146	6	127266726..127288567	3,45	4,24	-0,074274688
ECHDC1	6	complement(127288712..12734360	2,55	3,33	-0,160132589
KIAA0408	6	complement(127438406..12745938	0,02	0,03	-0,450526226
gene:ENSG000	6	complement(127438406..12751900	0,05	0,25	-2,0442463
MTCL3	6	complement(127472806..12751933	0,10	0,33	-1,475060794
C6orf58	6	127519455..127591820	-	0,05	-5,071123353
THEMIS	6	complement(127708072..12791863	-	-	0,188027316
PTPRK	6	complement(127968785..12852061	6,63	7,99	-0,046381039
LAMA2	6	128883138..129516566	4,19	4,54	0,107453852
ARHGAP18	6	complement(129576132..12971017	2,63	2,34	0,392111488
TMEM244	6	complement(129831244..12986154	0,50	0,18	1,669223118
L3MBTL3	6	130013699..130141449	1,61	1,90	-0,014954692
SAMD3	6	complement(130144315..13036542	0,00	0,01	-0,579815015
TMEM200A	6	130366017..130443067	0,05	0,17	-1,619631301
SMLR1	6	130827406..130837135	0,01	0,02	-0,979341556
EPB41L2	6	complement(130839347..13106332	7,30	12,13	-0,50866226
AKAP7	6	131135467..131283535	0,18	0,65	-1,630804469
ARG1	6	131470832..131584332	0,03	0,02	0,366665713

MED23	6	complement(131573966..13162824	4,19	4,58	0,092347689
ENPP3	6	131628442..131747418	0,03	0,04	-0,161981728
OR2A4	6	complement(131699644..13170140	-	-	0,188027316
CTAGE9	6	complement(131708441..13171101	0,01	-	4,516772761
ENPP1	6	131808016..131895155	1,32	2,48	-0,687943622
CCN2	6	complement(131948176..13195137	190,91	#####	0,080184688
gene:ENSG000	6	complement(132130252..13213179	0,01	0,03	-1,478387936
MOXD1	6	complement(132296055..13240147	0,31	0,46	-0,342980486
STX7	6	complement(132445867..13251319	1,08	1,45	-0,197221063
TAAR9	6	132538277..132539336	-	-	#N/D
TAAR8	6	132552672..132553756	-	-	#N/D
TAAR6	6	132570322..132571359	-	-	0,188027316
TAAR5	6	complement(132588592..13258974	-	-	#N/D
TAAR2	6	complement(132617022..13262427	-	-	#N/D
TAAR1	6	complement(132643312..13265918	-	0,01	-3,804623889
VNN1	6	complement(132680849..13271405	0,12	1,27	-3,205932832
VNN2	6	complement(132743870..13276345	-	0,08	-6,630750384
SLC18B1	6	complement(132769370..13281333	0,81	2,83	-1,587412392
RPS12	6	132814569..132817564	312,11	#####	-0,320823032
gene:ENSG000	6	132891924..132893819	0,01	-	3,068695355
EYA4	6	133240514..133532128	2,33	2,41	0,177624698
TCF21	6	133889113..133895553	4,47	8,11	-0,637095984
TBPL1	6	133952170..133990432	1,34	2,31	-0,556243065
gene:ENSG000	6	133953304..133980088	-	-	#N/D
SLC2A12	6	complement(133987581..13405262	0,48	0,78	-0,482951539
SGK1	6	complement(134169248..13431811	0,89	1,58	-0,607609704
ALDH8A1	6	complement(134917393..13495011	0,27	0,16	0,977254701
HBS1L	6	complement(134960378..13510305	4,32	4,20	0,263548242
MYB	6	135181308..135219173	0,03	0,15	-2,257016545
AHI1	6	complement(135283407..13549843	0,83	1,26	-0,374348443
PDE7B	6	135851701..136195574	0,07	0,31	-1,874522562
MTFR2	6	complement(136231024..13625033	3,11	4,14	-0,190644385
BCLAF1	6	complement(136256627..13628985	8,77	10,85	-0,083729148
MAP7	6	complement(136342281..13655081	0,01	0,10	-3,138140303
MAP3K5	6	complement(136557046..13679309	3,43	3,93	0,026898349
PEX7	6	136822564..136913934	0,64	0,94	-0,330699851
SLC35D3	6	136922301..136925660	0,10	0,04	1,338212201
IL20RA	6	complement(136999971..13704518	-	0,06	-7,112521263
IL22RA2	6	complement(137143820..13717364	-	-	#N/D
IFNGR1	6	complement(137197483..13721944	4,39	8,93	-0,800836195
OLIG3	6	complement(137492199..13749439	-	-	#N/D
TNFAIP3	6	137867214..137883314	2,96	7,51	-1,120924095
PERP	6	complement(138088505..13810741	21,34	21,21	0,232047876
ARFGEF3	6	138161939..138344663	0,22	1,98	-2,935400679
PBOV1	6	complement(138215986..13821849	0,03	0,04	0,152452079
SMIM28	6	138377905..138383486	-	-	0,188027316
HEBP2	6	138403531..138422197	1,36	1,88	-0,241607331
NHSL1	6	complement(138422043..13869257	4,57	6,12	-0,197376161

CCDC28A	6	138773769..138793319	7,69	9,65	-0,104331359
ECT2L	6	138795911..138904070	0,07	0,05	0,762506872
REPS1	6	complement(138903493..13898826	1,47	3,95	-1,198168756
ABRACL	6	139028745..139043302	2,14	3,97	-0,667338695
HECA	6	139135080..139180802	5,06	5,34	0,143767192
TXLNB	6	complement(139240061..13929199	0,02	0,05	-1,459576629
CITED2	6	complement(139371807..13937464	12,57	4,99	1,557537846
NMBR	6	complement(142058330..14214712	0,02	0,05	-0,916746549
GJE1	6	142132925..142135358	-	0,01	-2,89252005
VTA1	6	142147162..142224685	3,35	4,84	-0,307044989
ADGRG6	6	142301854..142446266	0,47	1,33	-1,262831769
HIVEP2	6	complement(142751469..14295669	4,86	7,09	-0,322054129
AIG1	6	143060496..143341058	1,39	2,17	-0,4182632
ADAT2	6	complement(143422832..14345069	0,77	1,55	-0,776436199
PEX3	6	143450805..143490616	1,49	3,13	-0,851732318
FUCA2	6	complement(143494812..14351172	12,60	22,40	-0,607467702
PHACTR2	6	143536845..143831185	2,17	4,62	-0,863436858
gene:ENSG000	6	143807092..143844617	-	0,07	-4,758582517
LTV1	6	143843338..143863812	3,93	6,41	-0,482229263
gene:ENSG000	6	143857318..143938471	-	-	#N/D
ZC2HC1B	6	143864436..143938343	-	-	0,188027316
PLAGL1	6	complement(143940300..14406459	1,20	1,95	-0,474938011
SF3B5	6	complement(144094884..14409557	60,05	53,03	0,402579103
STX11	6	144150487..144191939	0,02	0,34	-3,955116077
UTRN	6	144285335..144853034	3,26	3,15	0,270679318
EPM2A	6	complement(145382535..14573602	0,26	0,61	-0,972403035
gene:ENSG000	6	complement(145789270..14579197	0,51	0,40	0,55496733
FBXO30	6	complement(145793502..14581479	1,95	2,93	-0,365941839
SHPRH	6	complement(145864245..14596442	1,39	1,82	-0,157810568
GRM1	6	146027646..146437601	0,01	0,03	-1,854949791
RAB32	6	146543833..146554953	14,38	20,19	-0,267000916
ADGB	6	146598967..146815462	-	-	0,188027316
STXBP5	6	147204180..147391010	1,43	2,20	-0,39434104
SAMD5	6	147508690..147737547	0,53	0,39	0,686887173
SASH1	6	148272304..148552044	3,84	7,44	-0,731961947
UST	6	148747030..149076990	4,50	4,09	0,361117646
TAB2	6	149218641..149411613	9,89	15,16	-0,393394445
SUMO4	6	149400262..149401278	0,29	0,17	1,007134146
ZC3H12D	6	complement(149446795..14948501	0,06	0,11	-0,649547365
PPIL4	6	complement(149504495..14954604	2,55	3,59	-0,272822577
GINM1	6	149566294..149591748	5,85	8,84	-0,373120151
KATNA1	6	complement(149594873..14964897	5,71	8,42	-0,337116352
LATS1	6	complement(149658153..14971810	5,15	6,33	-0,072879867
NUP43	6	complement(149724315..14974966	16,41	17,22	0,153747341
PCMT1	6	149749444..149811420	18,23	17,23	0,304920122
gene:ENSG000	6	complement(149817937..14989601	0,90	0,83	0,350522493
LRP11	6	complement(149818757..14986435	23,39	15,37	0,82818762
RAET1E	6	complement(149883179..14989811	0,77	1,29	-0,528250071

RAET1G	6	complement(149916878..14992312	1,84	2,03	0,07799027
ULBP2	6	149942014..149949235	8,56	11,30	-0,177100828
ULBP1	6	149963943..149973715	0,60	2,49	-1,833316798
RAET1L	6	complement(150018334..15002553	0,05	0,51	-3,150194326
ULBP3	6	complement(150061053..15006912	2,14	3,97	-0,667101374
PPP1R14C	6	150143044..150250392	0,00	0,06	-3,307263034
IYD	6	150368892..150405969	0,00	0,02	-1,806405564
PLEKHG1	6	150599883..150843665	0,28	1,28	-1,969366621
MTHFD1L	6	150865679..151101887	8,48	13,87	-0,487683651
AKAP12	6	151239967..151358559	1,42	13,36	-3,006383496
ZBTB2	6	complement(151364115..15139155	3,10	4,57	-0,33823873
RMND1	6	complement(151398898..15145215	1,72	1,99	0,014123602
ARMT1	6	151452258..151470101	2,47	3,76	-0,385739131
CCDC170	6	151494017..151621193	0,04	0,03	0,320475827
ESR1	6	151656691..152129619	0,01	0,04	-1,911135835
SYNE1	6	complement(152121687..15263780	0,87	1,76	-0,794310776
MYCT1	6	152697897..152724569	-	-	0,188027316
VIP	6	152750797..152759765	-	0,01	-2,89252005
FBXO5	6	complement(152970519..15298357	7,72	9,78	-0,119124765
MTRF1L	6	complement(152987362..15300270	2,72	4,00	-0,333166787
RGS17	6	complement(153004459..15313128	1,18	1,01	0,447490693
OPRM1	6	154010496..154246867	0,00	0,00	0,933337975
IPCEF1	6	complement(154154496..15435680	-	-	0,188027316
gene:ENSG000	6	complement(154159828..15451011	-	-	0,188027316
CNKSR3	6	complement(154387515..15451068	0,52	0,60	0,019083861
SCAF8	6	154733378..154834244	8,35	10,86	-0,155038323
TIAM2	6	154832697..155257723	1,03	0,63	0,932617894
TFB1M	6	complement(155256134..15531449	4,97	5,88	-0,019452379
CLDN20	6	155264013..155276548	0,67	1,08	-0,469170375
NOX3	6	complement(155395368..15545583	-	-	0,188027316
ARID1B	6	156776020..157210779	1,85	3,00	-0,476594475
TMEM242	6	complement(157289025..15732360	1,16	1,93	-0,514973697
ZDHHC14	6	157381133..157678157	1,26	1,65	-0,171923879
SNX9	6	157700387..157945077	15,19	13,46	0,397838477
SYNJ2	6	157981863..158099176	10,33	13,39	-0,150482076
SERAC1	6	complement(158109519..15816828	0,36	0,82	-0,971131426
GTF2H5	6	158168350..158199344	0,81	1,35	-0,510851499
TULP4	6	158232236..158511828	4,20	5,39	-0,134881205
TMEM181	6	158536436..158635433	5,34	6,72	-0,108832718
DYNLT1	6	complement(158636474..15864474	88,64	76,19	0,441318737
SYTL3	6	158650014..158764876	3,81	5,11	-0,199587254
EZR	6	complement(158765741..15881936	27,13	47,87	-0,596249352
RSPH3	6	complement(158972871..15900020	2,01	2,03	0,210205105
TAGAP	6	complement(159034468..15909582	0,02	0,00	2,805678585
FNDC1	6	159169400..159272108	0,13	0,03	2,346036958
SOD2	6	complement(159669069..15976252	13,08	18,25	-0,257617963
WTAP	6	159725585..159756319	7,12	10,54	-0,343576921
ACAT2	6	159762045..159779112	14,85	8,81	0,976374432

TCP1	6	complement(159778498..15978970	59,61	74,11	-0,090880605
MRPL18	6	159789812..159798436	43,19	37,21	0,43779006
PNLDC1	6	159800249..159820704	0,01	0,04	-2,665078517
MAS1	6	159890988..159917447	0,00	0,00	0,677647514
IGF2R	6	159969082..160113507	16,81	20,04	-0,030690503
SLC22A1	6	160121815..160158718	0,04	0,11	-1,177556582
SLC22A2	6	complement(160171061..16027763	-	-	0,188027316
SLC22A3	6	160348378..160452577	0,01	0,04	-2,272922624
LPA	6	complement(160531482..16066427	-	-	0,188027316
PLG	6	160702194..160754097	-	0,00	-2,89252005
MAP3K4	6	160991727..161117385	9,00	9,23	0,186111117
AGPAT4	6	complement(161129967..16127406	2,57	3,32	-0,149254666
PRKN	6	complement(161347417..16272777	0,63	0,75	-0,007513213
PACRG	6	162727132..163315500	0,06	0,11	-0,548994131
QKI	6	163414000..163578592	5,76	6,58	0,030319117
C6orf118	6	complement(165279664..16530960	-	0,01	-3,804623889
PDE10A	6	complement(165327287..16598811	1,65	2,37	-0,305659697
TBXT	6	complement(166157656..16616870	0,02	0,00	1,833201638
PRR18	6	complement(166305300..16630844	0,07	0,04	1,021047459
SFT2D1	6	complement(166319728..16634257	14,64	17,33	-0,02014739
MPC1	6	complement(166364919..16638301	7,23	6,47	0,384372662
gene:ENSG000	6	166388136..166389920	0,04	0,06	-0,225481127
RPS6KA2	6	complement(166409364..16690645	7,52	17,08	-0,961512219
RAMACL	6	complement(166583009..16658667	0,21	0,37	-0,583899589
gene:ENSG000	6	complement(166858094..16695612	0,05	0,09	-0,429837071
RNASET2	6	complement(166922113..16695719	0,85	0,92	0,109081959
CEP43	6	166999174..167052718	0,86	1,19	-0,238166027
gene:ENSG000	6	166999336..167139138	0,16	0,40	-1,038823755
CCR6	6	167111807..167139141	0,02	0,04	-0,895537122
GPR31	6	complement(167155247..16715798	-	-	0,188027316
UNC93A	6	167271169..167316014	-	-	0,188027316
TTLL2	6	167325071..167359503	0,02	-	5,697545031
AFDN	6	167826893..167972023	7,38	10,49	-0,284876538
KIF25	6	167996241..168045091	0,01	0,01	0,533811434
FRMD1	6	complement(168053095..16810151	-	0,00	-2,89252005
DACT2	6	complement(168292830..16831977	-	0,00	-2,89252005
SMOC2	6	168441151..168673445	0,14	-	8,506549955
gene:ENSG000	6	168820423..168820656	-	-	0,188027316
gene:ENSG000	6	169033381..169034050	-	-	0,188027316
THBS2	6	complement(169215780..16925405	26,38	31,82	-0,047293308
gene:ENSG000	6	complement(169419969..16970204	0,12	0,35	-1,341126845
WDR27	6	complement(169457212..16970206	6,60	5,85	0,395980921
C6orf120	6	169702126..169706360	1,90	3,99	-0,846423788
PHF10	6	complement(169703902..16972556	3,47	5,91	-0,54423962
DYNLT2	6	complement(169740109..16975158	0,22	0,21	0,257973405
ERMARD	6	169751622..169781600	2,41	3,75	-0,413731121
DLL1	6	complement(170282206..17030656	3,06	7,07	-0,985780572
FAM120B	6	170290703..170407067	8,44	8,55	0,203830914

PSMB1	6	complement(170535120..17055330	70,61	93,17	-0,176916038
TBP	6	170554302..170572870	6,28	7,63	-0,058923651
PDCD2	6	complement(170575295..17058469	4,51	6,76	-0,3597974
FAM20C	7	192571..260772	56,49	33,21	0,989598643
gene:ENSG000	7	complement(227554..229557)	3,16	1,82	1,018937794
FOXL3	7	290170..294422	0,03	0,01	1,436225396
PDGFA	7	complement(497258..520296)	1,76	6,48	-1,652504277
PRKAR1B	7	complement(549197..727650)	0,88	3,31	-1,679533814
DNAAF5	7	726699..786475	12,29	19,83	-0,466860165
SUN1	7	816615..896435	23,46	22,90	0,257763787
GET4	7	876554..896436	6,72	5,89	0,414193356
ADAP1	7	complement(897900..955407)	1,71	3,48	-0,801857689
COX19	7	complement(898778..975549)	2,96	3,48	-0,007374547
CYP2W1	7	983181..989640	0,19	0,13	0,804426526
C7orf50	7	complement(996986..1138260)	11,61	13,31	0,025710752
GPR146	7	1044546..1059261	0,44	0,42	0,273076026
GPER1	7	1082208..1093815	0,35	0,41	0,002888944
ZFAND2A	7	complement(1152071..1160759)	11,68	12,16	0,164345101
UNCX	7	1232872..1237326	-	0,06	-6,052163584
MICALL2	7	complement(1428465..1459470)	10,21	7,39	0,688475751
INTS1	7	complement(1470277..1504389)	29,62	33,09	0,063396633
MAFK	7	1530702..1543043	25,03	23,74	0,299058736
TMEM184A	7	complement(1542235..1560821)	0,88	2,33	-1,177387898
PSMG3	7	complement(1567332..1571005)	14,66	22,76	-0,411648869
ELFN1	7	1665745..1747946	0,04	0,17	-2,004877445
MAD1L1	7	complement(1815793..2233243)	5,14	7,95	-0,407976528
gene:ENSG000	7	complement(1815804..2242215)	0,38	0,25	0,815138756
MRM2	7	complement(2234195..2242205)	5,01	9,73	-0,735420729
NUDT1	7	2242226..2251146	6,15	7,67	-0,094186403
SNX8	7	complement(2251770..2354318)	4,24	5,62	-0,184990829
EIF3B	7	2354086..2380745	53,89	58,36	0,108102568
CHST12	7	2403588..2448484	1,69	2,00	-0,021906604
GRIFIN	7	complement(2474728..2476894)	0,02	-	3,068695355
LFNG	7	2512529..2529177	0,82	1,93	-1,022507502
BRAT1	7	complement(2537810..2555694)	11,81	16,11	-0,225194221
IQCE	7	2558972..2614733	2,63	3,26	-0,085279467
TTYH3	7	2631961..2664802	54,67	49,27	0,37331514
AMZ1	7	2679522..2775500	1,37	0,93	0,773862526
GNA12	7	complement(2721822..2870786)	32,73	29,24	0,385992706
CARD11	7	complement(2906142..3044228)	-	-	0,188027316
SDK1	7	3301252..4269000	1,16	1,06	0,352491326
FOXK1	7	4682295..4771442	35,16	28,67	0,517277959
AP5Z1	7	4775615..4794397	3,91	2,56	0,83724511
RADIL	7	complement(4797055..4883716)	0,33	0,28	0,468792596
PAPOLB	7	complement(4857738..4862030)	0,02	0,01	0,933337975
MMD2	7	complement(4905989..4959213)	-	-	#N/D
RBAK-RBAKDN	7	4983718..5073221	2,99	3,04	0,201470596
RBAK	7	5045821..5069487	1,49	1,92	-0,139219495

WIPI2	7	5190196..5233840	10,24	14,88	-0,315904264
SLC29A4	7	5274369..5306912	1,74	1,06	0,94072882
TNRC18	7	complement(5306790..5425414)	16,88	15,19	0,375901604
FBXL18	7	complement(5431335..5513809)	6,25	5,66	0,3666032
ACTB	7	complement(5526409..5563902)	#######	#####	0,148921756
FSCN1	7	5592816..5606655	130,72	83,28	0,873443135
RNF216	7	complement(5620047..5781696)	14,69	16,43	0,061274404
OCM	7	5879827..5886363	0,01	-	3,068695355
CCZ1	7	5898725..5926550	8,69	13,20	-0,379226558
RSPH10B	7	complement(5925550..5970689)	0,00	0,03	-3,171495545
PMS2	7	complement(5970925..6009130)	2,68	5,21	-0,738464149
AIMP2	7	6009255..6023834	5,20	12,70	-1,064258776
EIF2AK1	7	complement(6022247..6059175)	31,88	30,81	0,272315131
ANKRD61	7	6031376..6036552	1,16	0,76	0,840617833
USP42	7	6104949..6161564	2,07	2,73	-0,177229269
CYTH3	7	complement(6161779..6272644)	9,45	11,53	-0,063829056
SAGSIN1	7	complement(6329411..6348967)	3,81	3,27	0,446326262
FAM220A	7	complement(6329411..6348981)	3,66	3,48	0,295934227
RAC1	7	6374527..6403967	56,73	75,01	-0,179840177
DAGLB	7	complement(6409126..6484190)	10,95	17,00	-0,411643306
KDELR2	7	complement(6445953..6484190)	122,66	84,96	0,75285849
GRID2IP	7	complement(6496778..6551461)	0,05	0,03	0,951907113
ZDHHC4	7	6577434..6589374	10,21	16,81	-0,496393568
INTS15	7	6590021..6608726	5,93	7,80	-0,172887587
ZNF853	7	6615610..6624290	4,75	8,18	-0,560813823
ZNF316	7	6637318..6658279	4,26	4,65	0,097630028
ZNF12	7	complement(6688433..6706947)	3,78	3,33	0,406049333
RSPH10B2	7	6754103..6799365	0,03	0,01	2,028855185
CCZ1B	7	complement(6794134..6826770)	6,39	9,15	-0,294524399
C1GALT1	7	7156934..7248616	1,57	1,64	0,161855087
COL28A1	7	complement(7356203..7535873)	-	0,01	-4,359055975
MIOS	7	7566875..7608932	1,74	2,98	-0,555846766
RPA3	7	complement(7636518..7718607)	3,51	4,47	-0,12718574
UMAD1	7	7640711..7968020	1,07	1,26	-0,017250151
GLCCI1	7	7968796..8094272	0,54	0,98	-0,649951755
ICA1	7	complement(8113184..8262687)	0,11	0,00	5,105203822
NXPH1	7	8433609..8752961	-	-	#N/D
NDUFA4	7	complement(10931943..10940153)	27,30	31,11	0,034994664
PHF14	7	10973336..11169623	1,10	1,80	-0,485469532
THSD7A	7	complement(11370365..11832198)	0,01	0,01	0,822166453
TMEM106B	7	12211270..12243367	0,90	1,82	-0,796335105
VWDE	7	complement(12330885..12403941)	0,35	0,41	-0,033842715
gene:ENSG000	7	12469621..12542222	-	-	#N/D
SCIN	7	12570577..12660182	0,24	0,02	3,553775873
ARL4A	7	12686856..12690958	2,32	3,30	-0,284394282
ETV1	7	complement(13891229..13991425)	1,70	3,25	-0,713452165
DGKB	7	complement(14145049..14974777)	0,00	0,00	0,886720911
AGMO	7	complement(15200317..15562015)	-	-	0,188027316

MEOX2	7	complement(15611212..15686683)	0,00	-	3,068695355
CRPPA	7	complement(16087525..16502504)	0,01	-	5,224730805
SOSTDC1	7	complement(16461481..16530580)	-	0,01	-2,89252005
LRRC72	7	16526825..16581568	-	-	0,188027316
ANKMY2	7	complement(16599779..16645817)	4,66	5,46	-0,006465862
BZW2	7	16646131..16706523	26,42	41,45	-0,426708538
TSPAN13	7	16753755..16784536	6,53	3,64	1,067235204
AGR2	7	complement(16791811..16833433)	0,01	-	3,967268276
AGR3	7	complement(16859412..16881987)	-	-	#N/D
AHR	7	16916359..17346152	7,11	8,85	-0,092646517
gene:ENSG000	7	17299295..17467234	0,26	2,87	-3,2382542
SNX13	7	complement(17790761..17940501)	0,86	1,56	-0,636205391
gene:ENSG000	7	17940503..17942926	0,13	0,24	-0,671251709
gene:ENSG000	7	complement(18000290..18000879)	-	-	#N/D
PRPS1L1	7	complement(18026770..18027846)	-	-	#N/D
HDAC9	7	18086949..19002416	0,34	1,62	-2,026860213
TWIST1	7	complement(19020991..19117636)	2,41	4,16	-0,560727183
FERD3L	7	complement(19144782..19145421)	-	-	#N/D
POLR1F	7	complement(19695461..19709037)	2,43	3,47	-0,292282728
TMEM196	7	complement(19719315..19773617)	-	0,00	-2,89252005
MACC1	7	complement(20134655..20217404)	0,00	0,00	-0,923955849
ITGB8	7	20330702..20415754	0,04	0,33	-2,817959892
ABCB5	7	20615667..20777038	0,01	0,00	1,487770062
SP8	7	complement(20782279..20786886)	0,54	0,31	1,030095441
SP4	7	21428043..21514822	1,41	1,50	0,137179732
DNAH11	7	21543039..21901839	0,02	0,05	-0,899746987
CDCA7L	7	complement(21900899..21945903)	12,29	19,62	-0,452329845
RAPGEF5	7	complement(22118236..22357154)	0,42	1,37	-1,480592543
STEAP1B	7	complement(22419444..22727613)	2,76	4,79	-0,573858235
IL6	7	22725884..22732002	0,06	2,94	-5,431628243
TOMM7	7	complement(22812628..22822849)	28,47	30,38	0,12949296
HYCC1	7	complement(22889371..23014130)	2,65	4,74	-0,615349402
KLHL7	7	23105758..23177914	1,98	1,79	0,368319899
NUP42	7	23181841..23201011	1,46	2,66	-0,636858577
GPNMB	7	23235967..23275108	8,10	20,97	-1,148967463
MALSU1	7	23298739..23311729	5,62	6,39	0,038404559
IGF2BP3	7	complement(23310209..23470491)	20,09	18,80	0,318349084
TRA2A	7	complement(23504780..23532041)	5,05	6,25	-0,082902068
CCDC126	7	23597382..23644708	0,29	0,57	-0,724811928
FAM221A	7	23680130..23703249	0,05	0,01	2,644301309
STK31	7	23710203..23832513	0,00	-	3,068695355
NPY	7	24284188..24291862	-	-	0,188027316
PALS2	7	24573268..24694193	1,25	2,02	-0,467226929
GSDME	7	complement(24698355..24757940)	2,00	5,59	-1,25792634
OSBPL3	7	complement(24796540..24981634)	1,09	3,62	-1,508368093
CYCS	7	complement(25118656..25125260)	4,41	3,27	0,655793295
SPMIP4	7	complement(25134692..25180356)	0,20	0,51	-1,127631925
NPVF	7	complement(25224570..25228486)	-	-	#N/D

NFE2L3	7	26152198..26187137	5,04	12,74	-1,113801656
HNRNPA2B1	7	complement(26171151..26201529)	23,00	30,61	-0,189395689
CBX3	7	26201162..26213607	9,84	15,90	-0,468645589
SNX10	7	26291862..26374383	0,45	0,90	-0,788525066
gene:ENSG000	7	26538378..26541048	-	-	0,188027316
SKAP2	7	complement(26667068..26995239)	3,40	4,33	-0,125189279
HOXA1	7	complement(27092993..27096000)	1,87	2,38	-0,129786108
HOXA2	7	complement(27100354..27102686)	0,56	0,32	1,007748497
HOXA3	7	complement(27106184..27152583)	0,39	0,50	-0,123582665
HOXA4	7	complement(27128507..27130780)	1,44	1,34	0,326951125
HOXA5	7	complement(27141052..27143681)	0,88	1,55	-0,586020238
HOXA6	7	complement(27145396..27150603)	1,54	2,32	-0,368422782
HOXA7	7	complement(27153716..27157936)	0,90	1,94	-0,879396373
HOXA9	7	complement(27162438..27175180)	7,26	5,53	0,616931552
gene:ENSG000	7	complement(27163535..27180013)	0,04	0,05	-0,03031053
HOXA10	7	complement(27170605..27180261)	3,34	4,04	-0,054076194
HOXA11	7	complement(27181157..27185232)	0,98	1,33	-0,213875752
HOXA13	7	complement(27193503..27200091)	0,47	0,28	0,978261531
EVX1	7	27242700..27250493	0,01	-	4,516772761
gene:ENSG000	7	27380562..27381206	-	0,02	-2,89252005
gene:ENSG000	7	27412164..27412891	-	-	#N/D
HIBADH	7	complement(27525442..27662883)	16,89	20,98	-0,089779635
TAX1BP1	7	27739331..27844564	5,76	6,98	-0,052796448
JAZF1	7	complement(27830573..28180795)	2,10	3,52	-0,522428935
CREB5	7	28299321..28825894	0,55	1,18	-0,875804961
TRIL	7	complement(28953358..28958330)	0,18	0,14	0,516890726
CPVL	7	complement(28995235..29195451)	0,28	1,45	-2,142747972
CHN2	7	29146547..29514328	0,12	0,31	-1,106048499
PRR15	7	29563835..29567293	0,01	0,05	-2,665078517
WIPF3	7	29806486..29917061	0,02	0,03	-0,667094398
SCRN1	7	complement(29920104..29990289)	41,48	46,65	0,0535467
FKBP14	7	complement(30010587..30026702)	7,42	6,17	0,488284582
PLEKHA8	7	30027404..30130483	2,09	2,67	-0,127308956
MTURN	7	30134986..30162762	1,51	2,30	-0,384655257
ZNRF2	7	30284597..30367689	0,61	0,94	-0,394101868
gene:ENSG000	7	30362400..30412502	-	0,21	-6,187931073
NOD1	7	complement(30424527..30478784)	0,95	1,69	-0,597192004
GGCT	7	complement(30496621..30504841)	5,35	8,18	-0,389102559
gene:ENSG000	7	complement(30497077..30550761)	0,11	0,23	-0,802495947
GARS1	7	30580533..30634033	32,41	57,12	-0,594711667
CRHR2	7	complement(30651942..30700129)	0,01	0,01	-0,579815015
INMT	7	30697985..30757602	0,07	0,04	0,949238826
INMT-MINDY4	7	30752137..30892081	-	-	0,188027316
MINDY4	7	30771417..30892387	0,50	0,68	-0,200336428
gene:ENSG000	7	30852273..30923812	-	-	0,188027316
AQP1	7	30911853..30925517	1,71	0,62	1,681912684
GHRHR	7	30938669..30993254	-	-	0,188027316
ADCYAP1R1	7	31052308..31111474	0,00	0,03	-2,562907834

NEUROD6	7	complement(31337465..31340726)	-	-	#N/D
ITPRID1	7	31514090..31656504	-	-	0,188027316
PPP1R17	7	31687215..31708455	-	-	0,188027316
PDE1C	7	complement(31751179..32428131)	0,10	0,05	1,250500836
LSM5	7	complement(32485338..32495283)	1,18	2,23	-0,699311885
AVL9	7	32495426..32588726	5,86	7,29	-0,092769458
KBTBD2	7	complement(32868172..32894131)	5,26	6,93	-0,175535588
FKBP9	7	32957404..33006930	29,21	41,19	-0,273042544
NT5C3A	7	complement(33014130..33062796)	0,45	1,87	-1,839148623
RP9	7	complement(33094797..33109405)	1,86	2,01	0,108745319
BBS9	7	33109557..33877180	1,75	1,70	0,267432056
BMPER	7	33904308..34156427	0,00	0,02	-1,955757882
NPSR1	7	34658218..34878332	0,00	-	3,068695355
DPY19L1	7	complement(34927292..35038271)	8,62	7,56	0,412635873
TBX20	7	complement(35202430..35254100)	2,74	3,43	-0,098693291
HERPUD2	7	complement(35632659..35697459)	5,76	9,02	-0,423509564
SEPTIN7	7	35800932..35907110	3,57	6,07	-0,54359633
EEPD1	7	36153254..36301538	1,71	1,30	0,614997852
MATCAP2	7	complement(36324152..36390125)	0,43	0,51	-0,029706575
ANLN	7	36389821..36453791	57,09	54,52	0,289681533
AOAH	7	complement(36512941..36724549)	0,06	0,31	-2,197774549
ELMO1	7	complement(36852906..37449223)	0,02	0,01	1,165729824
GPR141	7	37683766..37833788	-	-	0,188027316
gene:ENSG000	7	37683843..37950412	-	-	0,188027316
NME8	7	37848597..37900397	-	-	#N/D
SFRP4	7	complement(37905932..38025695)	0,00	0,02	-1,877914477
EPDR1	7	37920640..37951936	4,69	12,42	-1,182702842
STARD3NL	7	38178245..38230670	10,29	14,10	-0,231195473
TRGC2	7	complement(38239580..38249572)	0,00	0,00	0,886720911
TRGJ2	7	complement(38253380..38253429)	-	-	#N/D
TRGJP2	7	complement(38256337..38256396)	-	-	#N/D
TRGC1	7	complement(38257879..38265678)	-	0,00	-2,89252005
TARP	7	complement(38259643..38273636)	-	-	#N/D
TRGJ1	7	complement(38269491..38269540)	-	-	#N/D
TRGJP	7	complement(38273587..38273647)	-	-	#N/D
TRGJP1	7	complement(38276259..38276318)	-	-	#N/D
TRGV11	7	complement(38291616..38292078)	-	-	#N/D
TRGV10	7	complement(38299811..38300322)	-	-	#N/D
TRGV9	7	complement(38317017..38318861)	-	-	0,188027316
TRGV8	7	complement(38330343..38330935)	-	-	#N/D
TRGV5	7	complement(38349355..38350022)	-	0,10	-5,071123353
TRGV4	7	complement(38353715..38354517)	0,03	0,02	0,886720911
TRGV3	7	complement(38358512..38359162)	0,05	0,04	0,524121557
TRGV2	7	complement(38362864..38363518)	-	0,02	-2,89252005
TRGV1	7	complement(38367586..38368169)	0,02	0,04	-0,923955849
AMPH	7	complement(38383704..38631420)	1,80	2,12	-0,007260347
VPS41	7	complement(38722974..38932394)	2,79	3,70	-0,183891286
POU6F2	7	38977909..39493095	-	-	#N/D

YAE1	7	39566385..39610320	1,02	1,36	-0,195415915
RALA	7	39623565..39708120	7,28	9,04	-0,089858458
CDK13	7	39950121..40099580	2,31	3,20	-0,246244993
MPLKIP	7	complement(40126027..40134622)	1,34	1,69	-0,104705338
SUGCT	7	40135005..40860763	0,47	0,79	-0,523305188
INHBA	7	complement(41667168..41705834)	-	0,12	-8,596940655
GLI3	7	complement(41960949..42264100)	1,66	3,43	-0,822104278
C7orf25	7	complement(42909273..42912305)	0,13	0,42	-1,491057895
gene:ENSG000	7	complement(42909273..42932214)	1,57	3,51	-0,938230806
PSMA2	7	complement(42916828..42932185)	30,63	39,01	-0,126050708
MRPL32	7	42932376..42948958	3,73	3,99	0,124655513
HECW1	7	43112629..43566001	1,17	2,64	-0,953126159
STK17A	7	43582758..43650713	8,93	9,40	0,149281447
COA1	7	complement(43608456..43729717)	5,23	6,81	-0,158280084
BLVRA	7	43758680..43807342	18,97	20,44	0,115606477
MRPS24	7	complement(43866558..43869893)	88,40	98,72	0,063645244
URGCP-MRPS2	7	complement(43866558..43906589)	-	0,24	-6,630750384
URGCP	7	complement(43875894..43926411)	6,58	8,16	-0,087818104
UBE2D4	7	43926436..43956136	1,45	4,32	-1,351098106
SPDYE1	7	43997897..44010124	0,62	0,66	0,137696299
DBNL	7	44044640..44069456	7,86	6,75	0,443329217
PGAM2	7	complement(44062727..44065567)	0,03	-	3,967268276
POLM	7	complement(44072062..44082530)	3,98	4,39	0,080763977
AEBP1	7	44104345..44114562	36,80	50,21	-0,225062112
POLD2	7	complement(44114631..44124358)	28,36	32,17	0,041605791
MYL7	7	complement(44138864..44141332)	-	-	#N/D
GCK	7	complement(44143213..44198170)	0,00	0,00	-0,01185201
YKT6	7	44200959..44214294	31,76	22,41	0,725758755
CAMK2B	7	complement(44210019..44334577)	0,84	0,38	1,375755096
gene:ENSG000	7	complement(44367140..44369292)	0,02	0,04	-0,602143793
NUDCD3	7	complement(44379119..44490658)	12,26	11,96	0,258566805
NPC1L1	7	complement(44512535..44541330)	0,16	0,04	2,111228773
DDX56	7	complement(44565804..44575051)	16,74	20,39	-0,061453019
TMED4	7	complement(44577894..44582287)	35,18	36,23	0,180927836
OGDH	7	44606572..44709066	28,11	43,27	-0,399073463
ZMIZ2	7	44748581..44769881	7,30	11,83	-0,474230882
PPIA	7	44796680..44824564	167,96	#####	0,199047179
H2AZ2	7	complement(44826791..44848087)	16,27	15,02	0,339183126
PURB	7	complement(44876299..44885530)	5,37	5,39	0,218326706
MYO1G	7	complement(44962662..44979088)	0,01	0,05	-3,030577401
CCM2	7	44999475..45076469	4,64	4,99	0,118374426
NACAD	7	complement(45080437..45088969)	0,01	0,14	-3,432784426
TBRG4	7	complement(45100100..45112047)	25,69	43,68	-0,542633401
RAMP3	7	45157791..45186302	-	-	0,188027316
ADCY1	7	45574140..45723116	0,04	0,01	1,948668083
CCDC201	7	complement(45859994..45873082)	-	0,02	-5,545746556
IGFBP1	7	45888360..45893660	-	0,04	-5,071123353
IGFBP3	7	complement(45912245..45921874)	180,36	45,87	2,198195793

TNS3	7	complement(47275154..47582558)	15,51	17,17	0,07649724
PKD1L1	7	complement(47740202..47948466)	0,03	0,04	-0,248488186
HUS1	7	complement(47963288..47979615)	5,12	6,41	-0,102482976
SUN3	7	complement(47987148..48029119)	0,01	0,03	-1,877914477
C7orf57	7	48035511..48061304	0,10	0,19	-0,741496626
UPP1	7	48088628..48108736	1,99	14,95	-2,687308347
ABCA13	7	48171458..48647497	0,02	0,01	1,942207352
gene:ENSG000	7	48847766..48851625	-	-	#N/D
CDC14C	7	48924547..48927454	-	-	#N/D
gene:ENSG000	7	complement(49760897..49762764)	-	-	0,188027316
VWC2	7	49773638..49921950	0,00	-	5,224730805
ZPBP	7	complement(49850421..50121329)	-	-	#N/D
SPMIP7	7	50095883..50159256	-	-	#N/D
IKZF1	7	50304068..50405101	-	-	0,188027316
FIGNL1	7	complement(50444128..50450390)	1,45	3,24	-0,935280584
DDC	7	complement(50458436..50565405)	-	-	0,188027316
GRB10	7	complement(50590063..50793462)	9,09	29,04	-1,452236832
COBL	7	complement(51016212..51316818)	0,01	0,01	0,939616118
POM121L12	7	53035633..53036925	-	-	#N/D
VSTM2A	7	54542325..54571080	-	-	#N/D
SEC61G	7	complement(54752250..54759974)	18,79	15,16	0,53294632
EGFR	7	55019017..55211628	18,34	18,51	0,210134962
LANCL2	7	55365337..55433737	1,08	2,16	-0,769558545
VOPP1	7	complement(55436056..55572988)	16,58	19,13	0,01662095
SEPTIN14	7	complement(55793540..55862755)	-	-	0,188027316
gene:ENSG000	7	55887277..55955239	0,11	0,15	-0,189986357
ZNF713	7	55887456..55942530	0,06	0,03	1,067257943
NIPSNAP2	7	55951793..56000181	9,17	11,93	-0,155627278
MRPS17	7	55951877..55956500	3,76	4,89	-0,154325296
PSPH	7	complement(56011051..56051604)	5,72	10,56	-0,661376168
CCT6A	7	56051685..56063989	54,09	77,57	-0,296878923
SUMF2	7	56064002..56080670	39,82	40,19	0,209992324
PHKG1	7	complement(56080283..56092996)	0,57	0,57	0,22699766
CHCHD2	7	complement(56101573..56106476)	296,50	#####	-0,09546054
NUPR2	7	complement(56114681..56116417)	-	-	#N/D
ZNF479	7	complement(57117676..57139864)	0,00	-	3,068695355
gene:ENSG000	7	complement(57402181..57404717)	-	-	#N/D
ZNF716	7	57450177..57473559	0,00	-	3,967268276
ZNF722	7	63998849..64018081	-	-	0,188027316
ZNF727	7	64045429..64114556	-	0,00	-2,89252005
ZNF735	7	64207203..64220290	-	-	#N/D
ZNF679	7	64228474..64266931	0,01	0,02	-0,923955849
ZNF736	7	64307459..64356634	1,05	1,34	-0,127452396
ZNF680	7	complement(64519878..64563075)	0,43	0,74	-0,553478168
ZNF107	7	64666099..64711577	0,40	0,67	-0,537013076
ZNF138	7	64794388..64833681	0,36	0,48	-0,183305199
ZNF273	7	64870172..64930981	0,49	0,49	0,223787394
ZNF117	7	complement(64970663..65006687)	0,36	0,45	-0,106974837

ERV3-1	7	complement(64990356..65006743)	2,23	2,33	0,159886069
ZNF92	7	65373799..65401136	0,66	0,64	0,259182603
VKORC1L1	7	65873074..65959563	4,61	5,49	-0,028625638
GUSB	7	complement(65960684..65982215)	9,96	7,21	0,689482007
ASL	7	66075800..66094697	3,00	2,82	0,315006137
gene:ENSG000	7	66087761..66152277	0,12	0,39	-1,510710537
CRCP	7	66114604..66154568	6,46	8,27	-0,133728423
TPST1	7	66205317..66420543	6,48	9,26	-0,292633422
gene:ENSG000	7	66474556..66475749	0,03	0,12	-1,859185627
KCTD7	7	66628881..66649067	4,32	2,08	1,274375598
gene:ENSG000	7	66628958..66811189	0,17	0,39	-1,004676383
RABGEF1	7	66682164..66811464	3,45	3,91	0,044510833
TMEM248	7	66921225..66958551	13,81	12,60	0,355526534
SBDS	7	complement(66987680..66995693)	8,43	11,66	-0,245374641
TYW1	7	66995173..67239514	2,31	2,95	-0,130651619
SPDYE21	7	67276941..67289005	0,18	0,17	0,287440329
AUTS2	7	69598296..70793506	0,51	0,22	1,462441748
GALNT17	7	71132144..71713600	0,01	-	4,516772761
CALN1	7	complement(71779491..72447151)	0,00	-	3,068695355
TYW1B	7	complement(72558744..72828200)	0,17	0,21	-0,040245191
POM121	7	72879349..72951440	6,95	8,23	-0,020527568
TRIM74	7	complement(72959485..72969466)	0,01	-	3,068695355
SPDYE8	7	complement(73019719..73029766)	0,00	0,07	-3,842392802
SPDYE11	7	complement(73047862..73057911)	0,01	0,08	-3,189853383
SPDYE9	7	complement(73075972..73086038)	0,01	0,09	-2,783775817
SPDYE10	7	complement(73104008..73155431)	0,32	0,42	-0,180175095
NSUN5	7	complement(73302516..73308826)	7,69	13,79	-0,619610253
TRIM50	7	complement(73312536..73328082)	-	0,01	-2,89252005
FKBP6	7	73328161..73358637	0,01	0,01	-0,034419864
FZD9	7	73433778..73436120	0,20	0,10	1,165619831
BAZ1B	7	complement(73440406..73522293)	12,85	17,51	-0,223215874
BCL7B	7	complement(73536356..73557690)	5,94	10,13	-0,546349447
TBL2	7	complement(73567537..73578791)	4,27	4,86	0,035463862
MLXIPL	7	complement(73593194..73624543)	0,04	0,10	-1,187746923
VPS37D	7	73667831..73672112	1,20	1,01	0,473414753
DNAJC30	7	complement(73680918..73683453)	2,39	2,33	0,260632539
BUD23	7	73683597..73698212	21,06	27,16	-0,143511222
STX1A	7	complement(73699206..73719672)	6,28	9,63	-0,393485843
ABHD11	7	complement(73736094..73738852)	1,40	2,58	-0,656304809
CLDN3	7	complement(73768997..73770270)	0,02	0,02	-0,025382928
CLDN4	7	73799542..73832690	0,26	0,43	-0,511092853
METTL27	7	complement(73834590..73842516)	0,17	0,47	-1,202073017
TMEM270	7	73861159..73865890	-	0,05	-4,758582517
ELN	7	74027789..74069907	0,02	0,03	-0,204080844
LIMK1	7	74082933..74122525	11,71	20,69	-0,597406227
EIF4H	7	74174231..74197122	86,64	89,65	0,173825949
LAT2	7	74199652..74229834	0,32	0,41	-0,128662146
RFC2	7	complement(74231499..74254458)	12,55	13,08	0,163586736

CLIP2	7	74289407..74405935	7,63	8,33	0,0955457
GTF2IRD1	7	74453790..74602605	8,63	10,65	-0,08137673
gene:ENSG000	7	complement(74633510..74633884)	-	-	#N/D
GTF2I	7	74650231..74760692	12,91	17,37	-0,205072069
NCF1	7	74774011..74789315	-	0,02	-4,359055975
gene:ENSG000	7	complement(74796150..74890585)	1,59	1,88	-0,015903119
GTF2IRD2	7	complement(74796151..74851605)	0,12	0,05	1,39448012
SPDYE12	7	complement(74904289..74915078)	0,23	0,21	0,358266557
CASTOR2	7	74964705..75031528	4,95	3,72	0,634606028
RCC1L	7	complement(75027122..75074228)	6,44	7,97	-0,084815414
GTF2IRD2B	7	75092573..75149817	0,42	0,70	-0,505439616
SPDYE13	7	75280790..75289501	-	-	0,188027316
SPDYE14	7	75307376..75317429	0,07	-	7,117975732
SPDYE15	7	75335343..75345396	0,07	-	7,040630395
TRIM73	7	75395063..75410996	1,07	1,37	-0,127808707
POM121C	7	complement(75416786..75486299)	8,33	9,65	0,010448728
SPDYE5	7	75492320..75504316	0,59	0,36	0,96621421
HIP1	7	complement(75533298..75738962)	8,53	10,32	-0,052863101
gene:ENSG000	7	complement(75625089..75626970)	0,08	0,12	-0,316223056
CCL26	7	complement(75769533..75789896)	5,71	6,59	0,014529123
CCL24	7	complement(75810825..75823356)	0,19	0,16	0,431381374
RHBDD2	7	75842602..75888926	10,81	14,20	-0,170533649
POR	7	75899200..75986855	4,11	6,43	-0,4228942
TMEM120A	7	complement(75986831..75994656)	9,18	5,96	0,846496419
STYXL1	7	complement(75996338..76048004)	10,32	12,06	-0,002069614
gene:ENSG000	7	complement(76043977..76045963)	0,61	0,67	0,080117289
MDH2	7	76048051..76067508	48,38	62,84	-0,154208811
SRRM3	7	76201896..76287288	1,09	1,12	0,193851997
HSPB1	7	76302673..76304295	70,44	93,75	-0,189345516
YWHAG	7	complement(76326799..76358991)	35,76	44,09	-0,079112967
SSC4D	7	complement(76389334..76409697)	0,08	0,08	0,193538923
ZP3	7	76397518..76442071	0,03	0,18	-2,511769393
DTX2	7	76461676..76505995	4,62	9,30	-0,785913253
UPK3B	7	76510525..76516522	0,13	0,55	-1,877405265
SPDYE16	7	complement(76531313..76543297)	0,17	0,22	-0,171334412
POMZP3	7	complement(76609986..76627279)	1,25	1,53	-0,07357437
SPDYE17	7	complement(77022306..77032361)	-	-	0,188027316
SPDYE18	7	complement(77050391..77062558)	0,16	0,13	0,512489134
CCDC146	7	77122434..77329533	0,10	0,21	-0,937261109
FGL2	7	complement(77193369..77199848)	0,05	0,04	0,397821164
GSAP	7	complement(77310751..77416349)	0,92	2,46	-1,200832609
PTPN12	7	77537295..77640069	8,16	10,15	-0,090738283
RSBN1L	7	77696459..77783022	1,57	1,77	0,04549907
TMEM60	7	complement(77793728..77798434)	4,02	4,52	0,054937116
PHTF2	7	77798773..77957503	1,90	2,21	0,000536653
MAGI2	7	complement(78017055..79453667)	0,09	0,12	-0,108185133
gene:ENSG000	7	78134079..78134851	-	-	0,188027316
gene:ENSG000	7	78170195..78170915	-	-	#N/D

GNAI1	7	79768028..80226181	1,01	0,80	0,557080863
CD36	7	80369575..80679277	0,02	0,02	0,758596463
GNAT3	7	complement(80458635..80512064)	-	-	#N/D
SEMA3C	7	complement(80742538..80922359)	4,73	4,79	0,203803963
gene:ENSG000	7	complement(80972516..80974724)	-	-	#N/D
HGF	7	complement(81699010..81770438)	5,50	2,23	1,525325584
CACNA2D1	7	complement(81946444..82443956)	0,32	1,37	-1,892216387
PCLO	7	complement(82754012..83162930)	0,06	0,25	-1,902831519
SEMA3E	7	complement(83363238..83649139)	0,02	0,03	-0,905350572
gene:ENSG000	7	complement(83372652..83373096)	-	-	#N/D
SEMA3A	7	complement(83955777..84492724)	3,82	2,68	0,735426158
gene:ENSG000	7	complement(84939335..84940256)	2,69	2,56	0,296735332
SEMA3D	7	complement(84995553..85187056)	0,47	0,04	3,772420266
GRM3	7	86643909..86864879	-	-	0,188027316
ELAPOR2	7	complement(86876906..87059654)	0,13	0,12	0,289563225
DMTF1	7	87152409..87196337	3,49	4,59	-0,171597013
TMEM243	7	complement(87196160..87220587)	3,22	2,57	0,549732725
CROT	7	87345664..87399794	1,06	0,80	0,627983819
ABCB4	7	complement(87401696..87480435)	0,52	0,22	1,448196538
ABCB1	7	complement(87503017..87713323)	0,12	0,33	-1,211118168
RUNDC3B	7	87627548..87832296	0,09	0,15	-0,565420428
SLC25A40	7	complement(87833568..87876360)	1,31	1,73	-0,180552232
DBF4	7	87876216..87909553	1,54	2,84	-0,661164706
ADAM22	7	87934143..88202889	0,09	0,28	-1,343911946
SRI	7	complement(88205115..88226993)	7,83	8,92	0,033846757
STEAP4	7	complement(88270892..88306894)	0,00	0,00	-0,579815015
ZNF804B	7	88759700..89338528	-	-	0,188027316
TEX47	7	complement(88794106..88795737)	-	-	#N/D
STEAP1	7	90154456..90164829	9,85	11,07	0,054105403
STEAP2	7	90167590..90238137	5,19	3,73	0,700987705
CFAP69	7	90245174..90311063	0,26	0,29	0,054158824
FAM237B	7	complement(90316503..90321304)	0,01	0,01	0,524121557
GTPBP10	7	90335223..90391453	0,47	0,93	-0,749418247
gene:ENSG000	7	complement(90345873..90346218)	-	-	0,188027316
CLDN12	7	90383721..90513402	2,84	5,20	-0,650350715
CDK14	7	90466424..91210590	2,13	2,66	-0,098317969
PTTG1IP2	7	90469639..90513397	-	-	0,188027316
FZD1	7	91264433..91271326	1,96	2,02	0,183617405
MTERF1	7	complement(91692008..91880702)	0,54	0,71	-0,185046348
AKAP9	7	91940840..92110673	0,97	1,30	-0,199478952
CYP51A1	7	complement(92084987..92134803)	7,93	6,12	0,596576713
gene:ENSG000	7	complement(92112159..92179511)	-	-	0,188027316
gene:ENSG000	7	complement(92112176..92245924)	0,05	0,17	-1,659968152
LRRD1	7	complement(92141643..92179531)	0,00	0,00	-0,01185201
gene:ENSG000	7	complement(92144723..92245943)	-	-	0,188027316
KRIT1	7	complement(92197498..92246166)	0,82	1,34	-0,481006728
ANKIB1	7	92245974..92401383	4,84	6,69	-0,244331754
GATAD1	7	92447482..92460075	3,28	4,61	-0,266692162

ERVW-1	7	complement(92468380..92477986)	0,10	0,18	-0,622803245
PEX1	7	complement(92487020..92528520)	1,32	3,11	-1,017493581
RBM48	7	92528773..92540481	0,65	0,89	-0,224192696
FAM133B	7	complement(92560758..92590393)	0,92	1,32	-0,296617084
CDK6	7	complement(92604921..92836573)	13,54	14,77	0,097848561
SAMD9	7	complement(93099513..93118023)	0,76	1,60	-0,855261158
SAMD9L	7	complement(93130056..93148385)	0,34	0,52	-0,359131936
HEPACAM2	7	complement(93188534..93226469)	0,01	-	3,967268276
VPS50	7	93232340..93361123	0,65	0,81	-0,092748365
CALCR	7	complement(93424486..93574730)	-	-	0,188027316
GNGT1	7	93591573..93911265	0,01	-	3,967268276
TFPI2	7	complement(93885396..93890753)	1,57	7,13	-1,96469203
GNG11	7	93921735..93928610	0,82	0,68	0,49430597
gene:ENSG000	7	complement(93954044..93956519)	0,07	0,06	0,582943981
BET1	7	complement(93962762..94004382)	1,70	1,56	0,350391895
COL1A2	7	94394895..94431227	101,93	0,42	8,149573959
CASD1	7	94509219..94557019	0,32	0,73	-0,98372055
SGCE	7	complement(94524204..94656572)	1,51	2,84	-0,688873199
PEG10	7	94656325..94669695	0,05	0,05	0,111510372
PPP1R9A	7	94907202..95296415	0,31	1,01	-1,485915867
PON1	7	complement(95297676..95324532)	-	0,01	-3,804623889
PON3	7	complement(95359872..95396375)	-	-	0,188027316
PON2	7	complement(95404862..95435329)	3,53	6,39	-0,633679847
ASB4	7	95478444..95540233	-	-	0,188027316
gene:ENSG000	7	95542145..95544542	-	-	0,188027316
PDK4	7	complement(95583499..95596516)	1,40	1,11	0,560955567
DYNC1I1	7	95772506..96110322	0,19	0,11	0,976755802
gene:ENSG000	7	96118647..96119995	0,06	0,06	0,370333154
SLC25A13	7	complement(96120220..96322147)	1,89	4,82	-1,124317665
SEM1	7	complement(96481626..96709880)	0,53	0,61	0,023940618
DLX6	7	97005553..97011040	0,06	0,01	3,601075255
DLX5	7	complement(97020396..97024950)	0,02	-	4,913749003
SDHAF3	7	97117698..97181763	2,02	1,68	0,485216225
TAC1	7	97732084..97740472	-	-	#N/D
ASNS	7	complement(97851677..97872542)	7,11	29,50	-1,828678364
OCM2	7	complement(97984687..97991169)	-	-	#N/D
LMTK2	7	98106862..98209638	2,54	3,29	-0,149385026
BHLHA15	7	98211439..98215457	0,30	0,06	2,533759284
TECPR1	7	complement(98214624..98252232)	1,97	2,11	0,125050968
BRI3	7	98252379..98310441	17,01	22,04	-0,150461649
BAIAP2L1	7	complement(98291650..98401090)	9,50	10,70	0,052352569
NPTX2	7	98617285..98629869	1,02	0,18	2,712615062
TMEM130	7	complement(98846488..98870771)	0,08	0,02	2,029255507
TRRAP	7	98877933..99050831	5,63	7,12	-0,114152003
SMURF1	7	complement(99027440..99144108)	6,03	6,63	0,087143303
KPNA7	7	complement(99173572..99250075)	0,03	0,01	1,232079601
gene:ENSG000	7	99325879..99394653	2,07	1,81	0,419166696
ARPC1A	7	99325898..99366262	29,15	33,01	0,043635356

ARPC1B	7	99374249..99394816	17,52	20,85	-0,027887517
PDAP1	7	complement(99392048..99408597)	20,46	23,54	0,020976082
BUD31	7	99408641..99419616	13,45	14,60	0,104532103
PTCD1	7	complement(99416739..99466163)	1,65	2,15	-0,160654455
ATP5MF-PTCD1	7	complement(99419749..99466197)	1,14	2,41	-0,860586318
CPSF4	7	99438922..99457373	4,25	5,38	-0,115983168
ATP5MF	7	complement(99448475..99466186)	31,84	41,39	-0,155602312
ZNF789	7	99472890..99503650	0,93	0,71	0,625492578
ZNF394	7	complement(99473877..99504857)	3,32	2,68	0,533664031
ZKSCAN5	7	99504662..99534700	2,34	2,52	0,116751102
FAM200A	7	complement(99546300..99558536)	0,58	0,74	-0,117548621
ZNF655	7	99558406..99576453	2,38	3,44	-0,309151009
gene:ENSG000	7	99558695..99607810	0,10	-	5,88622595
TMEM225B	7	99598267..99611045	0,31	0,84	-1,194845373
ZSCAN25	7	99616946..99632408	1,48	1,99	-0,201757475
CYP3A5	7	complement(99648194..99679996)	0,03	0,08	-0,995745964
CYP3A7-CYP3A	7	complement(99684957..99735102)	0,01	0,03	-1,877914477
CYP3A7	7	complement(99705036..99735196)	-	-	0,188027316
CYP3A4	7	complement(99756960..99784248)	-	-	0,188027316
CYP3A43	7	99828013..99866093	-	-	0,188027316
OR2AE1	7	complement(99876062..99877033)	-	-	0,188027316
TRIM4	7	complement(99876958..99919531)	3,37	2,49	0,656159171
GJC3	7	complement(99923266..99929620)	-	0,01	-2,89252005
AZGP1	7	complement(99966720..99976042)	0,01	0,04	-2,447197442
ZKSCAN1	7	100015572..100041689	7,89	7,33	0,328373085
ZSCAN21	7	100049774..100065040	1,37	1,90	-0,247117657
ZNF3	7	complement(100064033..10008254	3,58	3,39	0,303735194
COPS6	7	100088969..100092187	34,60	42,49	-0,073488219
MCM7	7	complement(100092233..10010194	29,11	43,01	-0,340019693
AP4M1	7	100100858..100110345	1,31	1,68	-0,132766272
TAF6	7	complement(100106876..10011984	10,93	14,66	-0,201060024
CNPY4	7	100119634..100125508	2,48	2,83	0,032485903
MBLAC1	7	100126785..100128495	0,22	0,53	-1,060361083
LAMTOR4	7	100148912..100155944	8,04	7,75	0,275555721
TRAPPC14	7	complement(100154420..10015872	1,59	2,37	-0,347376179
GAL3ST4	7	complement(100159244..10016861	0,29	0,09	1,812851642
GPC2	7	complement(100169606..10017738	0,85	0,38	1,372710959
STAG3	7	100177563..100214387	0,30	0,26	0,433337257
gene:ENSG000	7	100205357..100221488	0,71	0,83	-0,011737268
PVRIG	7	100218241..100221490	-	0,06	-5,545746556
SPDYE3	7	100307702..100322196	0,21	0,37	-0,608698077
PILRB	7	100352176..100367831	7,11	11,18	-0,430000619
PILRA	7	100367530..100400096	0,03	0,15	-1,997339153
gene:ENSG000	7	100397577..100434107	0,02	0,09	-1,820127312
ZCWPW1	7	complement(100400826..10042899	0,47	0,91	-0,727556434
MEPCE	7	100428322..100434126	11,74	13,25	0,049092414
PPP1R35	7	complement(100435282..10043649	10,28	11,23	0,095850288
SPACDR	7	complement(100456620..10046426	2,64	1,16	1,413742545

TSC22D4	7	complement(100463359..10047923	11,41	9,28	0,521488553
gene:ENSG000	7	complement(100478099..10047817	-	-	#N/D
NYAP1	7	100483927..100494802	0,63	1,71	-1,214709708
AGFG2	7	100539203..100568220	3,08	5,14	-0,516997527
SAP25	7	complement(100572228..10057390	1,15	0,93	0,523042964
LRCH4	7	complement(100574011..10058612	7,71	8,74	0,041476749
FBXO24	7	100583982..100601117	0,39	0,30	0,592761598
PCOLCE	7	100602363..100608175	57,03	71,40	-0,10113834
MOSPD3	7	100612102..100615384	5,47	3,81	0,744912919
TFR2	7	complement(100620416..10064277	0,05	0,24	-1,912702987
ACTL6B	7	complement(100643097..10065644	-	-	0,188027316
GNB2	7	100673567..100679174	113,84	86,45	0,620173452
GIGYF1	7	complement(100679507..10069425	14,51	15,33	0,143642579
POP7	7	100706121..100707486	30,64	28,27	0,33945905
EPO	7	100720468..100723700	0,03	0,02	0,366665713
ZAN	7	100733595..100797797	0,00	0,01	-1,54862452
EPHB4	7	complement(100802565..10082752	18,11	19,33	0,128993956
SLC12A9	7	100826820..100867010	2,80	2,83	0,208120582
TRIP6	7	100867387..100873454	37,29	34,69	0,327060815
SRRT	7	100875103..100888664	21,10	21,49	0,196895905
UFSP1	7	complement(100888721..10088971	0,53	0,83	-0,424573546
ACHE	7	complement(100889994..10089697	0,04	0,14	-1,685373637
MUC3A	7	100949534..100968347	-	0,00	-4,359055975
MUC12	7	100969565..101018939	0,00	0,00	-1,478387936
MUC17	7	101020072..101058859	-	-	0,188027316
TRIM56	7	101085481..101097967	8,10	7,11	0,412478107
SERPINE1	7	101127104..101139247	62,99	#####	-0,618255709
AP1S1	7	101154456..101161596	38,73	37,12	0,284415091
VGF	7	complement(101162509..10116556	0,31	1,07	-1,585262905
NAT16	7	complement(101170496..10118029	0,20	0,32	-0,419846786
MOGAT3	7	complement(101195007..10120103	0,00	-	3,068695355
PLOD3	7	complement(101205977..10121842	32,57	34,52	0,139227369
ZNHIT1	7	101218165..101224190	37,71	29,95	0,55543151
CLDN15	7	complement(101232092..10123882	2,37	3,30	-0,253871093
FIS1	7	complement(101239458..10125231	21,09	21,45	0,199046655
IFT22	7	complement(101310914..10132182	1,00	1,06	0,137634714
COL26A1	7	101362875..101559024	0,01	0,01	0,921097799
MYL10	7	complement(101613330..10162929	0,01	-	3,068695355
CUX1	7	101815904..102283958	4,96	5,58	0,052798311
SH2B2	7	102285091..102321711	0,37	1,17	-1,425557447
SPDYE6	7	complement(102345746..10235644	0,09	0,08	0,41017726
PRKRIP1	7	102363872..102426676	4,42	3,58	0,528737501
ORAI2	7	102433106..102456825	5,96	4,16	0,742561685
ALKBH4	7	complement(102456238..10246486	1,59	2,33	-0,32378971
LRWD1	7	102464956..102473168	3,12	4,86	-0,414067661
POLR2J	7	complement(102473128..10247892	12,36	13,04	0,145258443
RASA4B	7	complement(102479976..10251777	3,55	1,27	1,710988422
POLR2J3_1	7	complement(102537918..10257265	9,49	8,24	0,428006081

UPK3BL2	7	complement(102537919..10254369	-	-	#N/D
gene:ENSG000	7	complement(102541501..10259244	0,49	0,09	2,705344625
SPDYE2	7	102551226..102563538	1,71	2,12	-0,089442189
POLR2J3_2	7	complement(102562133..10257258	3,12	3,21	0,180397501
RASA4	7	complement(102579646..10261675	6,18	2,27	1,669166442
gene:ENSG000	7	complement(102582523..10264286	0,98	0,81	0,486990889
UPK3BL1	7	complement(102637025..10264279	25,33	23,74	0,316133249
gene:ENSG000	7	complement(102637049..10267164	8,31	9,11	0,091489179
SPDYE2B	7	102650319..102662625	0,37	0,42	0,050720573
POLR2J2	7	complement(102665368..10267166	2,99	2,60	0,426246047
FAM185A	7	102748971..102809225	0,62	0,76	-0,06279832
FBXL13	7	complement(102811189..10307483	0,05	0,08	-0,381994707
LRRC17	7	102913000..102945111	0,01	0,05	-2,272922624
ARMC10	7	103074881..103099759	3,17	4,37	-0,238923336
NAPEPLD	7	complement(103099776..10314956	1,21	1,31	0,113136888
gene:ENSG000	7	complement(103161947..10316266	0,09	0,05	0,933337975
PMPCB	7	103297407..103331818	1,92	2,88	-0,366299242
DNAJC2	7	complement(103312289..10334483	1,50	1,69	0,048866515
PSMC2	7	103328570..103370346	7,36	9,73	-0,180018544
SLC26A5	7	complement(103352730..10344620	0,05	0,03	0,947848749
RELN	7	complement(103471381..10398965	0,74	0,05	3,999922913
ORC5	7	complement(104126341..10420804	0,87	2,51	-1,312962221
LHFPL3	7	104328603..104908561	-	-	0,188027316
KMT2E	7	104940943..105115019	3,07	3,11	0,203494773
gene:ENSG000	7	105040848..105040925	-	-	#N/D
gene:ENSG000	7	105040858..105040956	-	-	#N/D
SRPK2	7	complement(105110704..10539930	3,80	5,94	-0,41993023
PUS7	7	complement(105439661..10552227	1,87	4,43	-1,025787412
RINT1	7	105532169..105567677	6,07	4,73	0,583247053
EFCAB10	7	complement(105565120..10560087	0,12	0,06	1,223902512
ATXN7L1	7	complement(105604772..10587659	0,35	0,58	-0,525962829
CDHR3	7	105876796..106036432	0,03	0,05	-0,734644919
gene:ENSG000	7	106035798..106036431	-	-	#N/D
SYPL1	7	complement(106090505..10611258	9,95	10,79	0,106031313
NAMPT	7	complement(106248298..10628596	2,23	5,82	-1,163324332
CCDC71L	7	complement(106654360..10666115	1,01	1,42	-0,259956936
PIK3CG	7	106865278..106908980	0,00	-	3,068695355
PRKAR2B	7	107044705..107161811	0,23	0,79	-1,524342426
HBP1	7	107168961..107202522	7,76	5,44	0,734388767
COG5	7	complement(107201372..10756426	3,22	5,22	-0,474505738
GPR22	7	107470018..107475684	0,04	0,05	-0,03814481
DUS4L	7	107563484..107578523	0,27	1,24	-1,950675815
DUS4L-BCAP29	7	107563971..107623317	0,80	1,08	-0,222616524
BCAP29	7	107580246..107629170	0,40	0,93	-0,98295826
SLC26A4	7	107660828..107717809	0,11	0,21	-0,752409298
CBLL1	7	107743073..107761667	2,27	2,93	-0,147752327
SLC26A3	7	complement(107765467..10780322	-	0,00	-2,89252005
DLD	7	107891162..107931730	5,10	7,28	-0,290208901

LAMB1	7	complement(107923799..10800321	47,54	42,05	0,400125743
LAMB4	7	complement(108023548..10813036	0,06	0,02	1,871856751
NRCAM	7	complement(108147623..10845671	0,03	0,47	-3,693821535
PNPLA8	7	complement(108470417..10856966	1,50	1,91	-0,124395118
THAP5	7	complement(108554543..10856975	1,50	1,94	-0,143605598
DNAJB9	7	108569867..108574850	2,22	1,25	1,051659102
gene:ENSG000	7	complement(108900105..10890522	-	0,00	-2,89252005
IMMP2L	7	complement(110662644..11156251	0,52	0,80	-0,416091576
LRRN3	7	111091006..111125454	0,00	0,04	-3,431345623
DOCK4	7	complement(111726110..11220640	1,83	3,12	-0,543153932
ZNF277	7	112206695..112343934	1,04	1,41	-0,216300073
IFRD1	7	112422887..112481017	2,57	6,07	-1,015116917
gene:ENSG000	7	112450460..112490941	0,76	0,95	-0,098032497
gene:ENSG000	7	112450487..112450645	0,54	0,65	-0,036800208
LSMEM1	7	112480853..112491062	0,02	0,07	-1,586291974
TMEM168	7	complement(112762377..11279042	0,67	1,05	-0,431071739
BMT2	7	complement(112819147..11293987	0,34	0,39	0,05238535
gene:ENSG000	7	complement(113075124..11307718	0,01	0,03	-0,979341556
GPR85	7	complement(113078331..11308777	0,09	0,25	-1,23833311
SMIM30	7	complement(113116718..11311856	12,59	12,54	0,228872273
gene:ENSG000	7	113451072..113451402	-	-	#N/D
PPP1R3A	7	complement(113876777..11407592	-	-	#N/D
FOXP2	7	114086327..114693772	0,01	0,03	-1,177989845
MDFIC	7	114922094..115019917	2,57	3,16	-0,075621987
TFEC	7	complement(115935148..11615989	-	-	0,188027316
gene:ENSG000	7	complement(116209234..11621151	0,26	0,23	0,391440738
TES	7	116210506..116258783	9,10	10,59	0,003869538
CAV2	7	116287380..116508541	20,01	7,40	1,657860805
CAV1	7	116524994..116561179	136,89	52,79	1,597848545
MET	7	116672196..116798377	9,59	17,82	-0,670793109
CAPZA2	7	116811070..116922049	2,37	3,74	-0,433954396
ST7	7	116953238..117230176	1,15	2,01	-0,581293627
gene:ENSG000	7	116954391..117119719	0,04	0,10	-0,979341556
WNT2	7	complement(117275451..11732315	0,01	-	4,516772761
CFTR	7	117287120..117715971	-	-	0,188027316
ASZ1	7	complement(117363222..11742812	-	-	#N/D
CTTNBP2	7	complement(117710651..11787413	0,12	0,97	-2,767219528
LSM8	7	118184144..118204035	0,45	0,60	-0,182644906
ANKRD7	7	118214669..118496171	-	-	0,188027316
KCND2	7	120273175..120750337	-	0,04	-6,89165328
TSPAN12	7	complement(120787320..12085840	0,59	0,83	-0,276603625
ING3	7	120950763..120977216	0,32	0,64	-0,78091093
CPED1	7	120988697..121297442	6,24	2,83	1,363590728
WNT16	7	121325367..121341104	0,12	0,03	2,33996017
FAM3C	7	complement(121348878..12139636	5,49	5,01	0,35659249
PTPRZ1	7	121873089..122062036	0,00	0,01	-1,877914477
AASS	7	complement(122064583..12214430	0,26	0,55	-0,840073477
FEZF1	7	complement(122301303..12231069	-	-	0,188027316

CADPS2	7	complement(122318411..12288675	0,05	0,01	2,362275056
RNF133	7	complement(122697735..12269911	-	-	0,188027316
RNF148	7	complement(122701668..12270292	0,02	-	3,967268276
TAS2R16	7	complement(122994704..12299570	-	-	#N/D
SLC13A1	7	complement(123113531..12319997	0,00	0,00	-0,01185201
IQUB	7	complement(123452193..12353507	0,04	0,02	1,520939857
NDUFA5	7	complement(123536997..12355790	0,34	0,46	-0,219932999
ASB15	7	123567010..123639481	0,00	-	3,068695355
LMOD2	7	123655866..123664290	-	0,00	-2,89252005
WASL	7	complement(123681943..12374900	4,35	5,07	0,003194046
HYAL4	7	123828983..123877481	-	-	0,188027316
SPAM1	7	123925237..123971414	-	-	#N/D
TMEM229A	7	complement(124030921..12403306	-	-	#N/D
SSU72L6	7	124476317..124477664	-	-	#N/D
gene:ENSG000	7	complement(124742312..12474501	-	0,01	-4,359055975
GPR37	7	complement(124743885..12476579	0,95	0,72	0,619859391
POT1	7	complement(124822386..12492998	1,66	2,37	-0,293568509
GRM8	7	complement(126438598..12725309	0,00	-	3,068695355
ZNF800	7	complement(127346790..12743192	1,42	1,97	-0,249649032
gene:ENSG000	7	complement(127349833..12735029	0,49	0,67	-0,230810911
gene:ENSG000	7	complement(127359785..12736475	1,06	1,04	0,26204734
GCC1	7	complement(127580628..12759361	5,02	5,20	0,170780757
ARF5	7	127588386..127591700	28,13	39,78	-0,276786822
FSCN3	7	127591409..127602144	0,01	0,01	0,886720911
PAX4	7	complement(127610292..12761814	-	0,01	-2,89252005
SND1	7	127652194..128092609	77,90	69,35	0,390874978
LRRC4	7	complement(128027071..12803210	0,42	0,23	1,080472146
LEP	7	128241278..128257629	0,05	0,02	1,443009573
RBM28	7	complement(128297685..12834390	2,41	3,12	-0,151946595
PRRT4	7	complement(128350325..12836169	0,03	0,02	0,7939095
IMPDH1	7	complement(128392277..12840998	34,23	30,48	0,390429928
HILPDA	7	128455849..128458418	20,40	7,37	1,691369386
METTL2B	7	128476729..128506602	3,35	4,38	-0,165005573
GARIN1A	7	128671693..128687877	0,02	0,13	-2,286400912
GARIN1B	7	128709061..128731743	-	0,01	-3,804623889
CALU	7	128739292..128773400	72,00	65,74	0,354191802
OPN1SW	7	complement(128772485..12877579	0,12	0,21	-0,617784428
CCDC136	7	128790757..128822132	0,14	0,20	-0,249410284
FLNC	7	128830406..128859274	61,66	42,14	0,772272599
KCP	7	complement(128862042..12891071	-	0,00	-3,804623889
ATP6V1F	7	128862856..128865847	37,94	38,69	0,194753583
SPMIP1	7	128866330..128872047	0,19	0,14	0,666323756
IRF5	7	128937457..128950038	0,04	0,08	-0,925287283
gene:ENSG000	7	complement(128952527..12895331	0,20	0,08	1,520939857
TNPO3	7	complement(128954180..12905517	10,97	13,54	-0,080354057
TSPAN33	7	129144707..129169699	1,68	1,13	0,798722853
SMO	7	129188633..129213545	6,23	9,88	-0,442824249
AHCYL2	7	129225030..129430211	1,89	2,15	0,03870605

STRIP2	7	129434432..129488399	0,85	1,96	-0,989802068
SMKR1	7	129502531..129512918	0,06	0,48	-2,849607196
NRF1	7	129611720..129757082	2,65	4,42	-0,515092383
UBE2H	7	complement(129830732..12995296	21,90	18,91	0,434670342
ZC3HC1	7	complement(130018287..13005140	2,99	4,42	-0,340043426
KLHDC10	7	130070534..130135705	7,78	5,48	0,728333578
TMEM209	7	complement(130164713..13020777	2,90	4,94	-0,544325261
SSMEM1	7	130206344..130216844	-	-	0,188027316
CPA2	7	130266863..130289798	-	-	0,188027316
CPA4	7	130293134..130324180	3,40	6,75	-0,766001711
CPA5	7	130344816..130368730	0,00	0,02	-2,190455314
CPA1	7	130380339..130388114	-	0,02	-3,804623889
CEP41	7	complement(130393771..13044243	0,73	1,10	-0,363732393
MEST	7	130486171..130506465	0,02	0,03	-0,253352605
COPG2	7	complement(130506238..13066874	6,02	8,28	-0,235828942
TSGA13	7	complement(130668643..13068743	-	-	0,188027316
KLF14	7	complement(130730697..13073420	-	0,03	-5,734998842
MKLN1	7	131110096..131496632	2,49	3,26	-0,162971892
PODXL	7	complement(131500262..13155821	6,84	18,51	-1,213165807
PLXNA4	7	complement(132123340..13264868	0,07	0,02	2,207323465
CHCHD3	7	complement(132784870..13308209	4,48	8,41	-0,686669424
EXOC4	7	133253073..134066589	8,30	9,20	0,074958945
LRGUK	7	134127299..134264595	0,02	0,02	0,112279066
SLC35B4	7	complement(134289332..13431693	2,22	3,48	-0,424258014
AKR1B1	7	complement(134442356..13445928	216,75	#####	-0,236010248
AKR1B10	7	134527567..134541412	2,40	0,33	3,089338065
AKR1B15	7	134549110..134579875	0,19	0,19	0,222001418
BPGM	7	134646811..134679816	1,84	2,77	-0,363974072
CALD1	7	134744252..134970729	2,86	7,41	-1,149271239
AGBL3	7	134986508..135147963	0,04	0,03	0,689783732
CYREN	7	complement(135092363..13517079	4,26	8,06	-0,697997145
TMEM140	7	135148072..135166215	4,78	7,34	-0,395922298
WDR91	7	complement(135183839..13521155	0,97	0,81	0,472521263
STRA8	7	135231979..135258663	-	-	#N/D
CNOT4	7	complement(135361795..13551014	1,47	1,84	-0,102302517
NUP205	7	135557917..135648757	13,36	19,96	-0,356273679
STMP1	7	135662496..135693418	5,62	6,18	0,086389406
SLC13A4	7	complement(135681231..13572925	0,11	0,14	-0,168615427
FAM180A	7	complement(135728348..13574881	0,73	1,67	-0,964106246
MTPN	7	complement(135926760..13597735	28,67	29,68	0,173236347
CHRM2	7	136868652..137020255	0,01	0,00	1,833201638
PTN	7	complement(137227341..13734377	0,72	0,92	-0,13106911
DGKI	7	complement(137381037..13784709	0,01	0,04	-1,220681718
CREB3L2	7	complement(137874979..13800208	21,85	20,46	0,317953739
AKR1D1	7	138002324..138118305	-	-	#N/D
TRIM24	7	138460259..138589996	1,19	1,44	-0,053163191
SVOPL	7	complement(138594285..13870136	-	-	0,188027316
ATP6V0A4	7	complement(138706294..13879956	0,00	0,02	-2,447197442

TMEM213	7	138797952..138838101	0,02	-	5,697545031
KIAA1549	7	complement(138831381..13898138	0,84	2,26	-1,205536581
ZC3HAV1L	7	complement(139025706..13903604	3,88	4,13	0,13502175
ZC3HAV1	7	complement(139043515..13913212	12,80	6,72	1,153382492
IFT56	7	139133744..139191986	0,78	1,19	-0,374097717
UBN2	7	139230356..139308236	1,31	1,24	0,299082491
FMC1	7	139339457..139346328	1,62	0,70	1,423663067
LUC7L2	7	139340359..139423457	7,06	8,82	-0,098513034
FMC1-LUC7L2	7	139341360..139422599	6,99	5,72	0,512230301
KLRG2	7	complement(139452690..13948367	-	-	0,188027316
CLEC2L	7	139523685..139544985	0,08	0,02	2,345408045
HIPK2	7	complement(139561570..13977799	11,87	8,41	0,719839642
TBXAS1	7	139777051..140020325	2,32	0,84	1,681609974
PARP12	7	complement(140023749..14006295	2,59	4,56	-0,59501179
KDM7A	7	complement(140084746..14017698	0,42	1,23	-1,316630763
SLC37A3	7	complement(140293693..14040443	2,75	2,54	0,335069089
RAB19	7	140404058..140427974	0,01	0,00	1,436225396
MKRN1	7	complement(140453033..14047953	6,70	7,25	0,108332264
DENND2A	7	complement(140518420..14067399	3,77	5,94	-0,432358851
ADCK2	7	140672945..140696261	5,42	8,61	-0,445240871
NDUFB2	7	140690777..140722790	4,28	4,27	0,224857542
BRAF	7	complement(140719327..14092492	1,58	1,68	0,134776934
MRPS33	7	complement(141002610..14101522	0,98	1,97	-0,777583346
TMEM178B	7	141074064..141480380	0,01	0,01	-0,253352605
AGK	7	141551278..141655244	2,16	3,11	-0,297465379
DENND11	7	complement(141656728..14170216	2,62	4,24	-0,470691625
WEE2	7	141708353..141731271	0,00	0,01	-0,923955849
SSBP1	7	141738334..141787922	6,45	9,85	-0,387559941
TAS2R3	7	141764097..141765197	0,19	0,29	-0,432646637
TAS2R4	7	141776674..141781691	0,08	0,10	-0,001674045
TAS2R5	7	141790217..141791367	0,15	0,34	-0,940108714
PRSS37	7	complement(141836300..14184148	0,02	0,01	1,833201638
OR9A1P	7	141887148..141888092	-	-	0,188027316
MGAM	7	141907813..142106747	-	0,01	-5,071123353
OR9A4	7	141916399..141920625	-	-	#N/D
CLEC5A	7	complement(141927357..14194700	-	0,00	-2,89252005
TAS2R38	7	complement(141972631..14197377	-	-	#N/D
MGAM2	7	142111718..142222324	-	0,00	-3,804623889
PRSS58	7	complement(142252143..14225805	-	-	#N/D
TRBV2	7	142300924..142301432	-	-	#N/D
TRBV3-1	7	142308542..142309048	-	-	#N/D
TRBV4-1	7	142313184..142313666	-	-	#N/D
TRBV5-1	7	142320677..142321544	-	-	#N/D
TRBV6-1	7	142328297..142328786	-	-	#N/D
TRBV7-1	7	142332182..142332701	-	-	#N/D
TRBV4-2	7	142345421..142345985	-	-	#N/D
TRBV6-2	7	142349152..142349664	-	-	#N/D
TRBV7-2	7	142352819..142353358	0,02	-	3,068695355

TRBV6-4	7	142380806..142381261	-	-	#N/D
TRBV7-3	7	142384329..142384841	0,04	0,03	0,886720911
TRBV5-3	7	142389202..142389668	-	-	0,188027316
TRBV9	7	142391891..142392412	-	-	#N/D
TRBV10-1	7	142399860..142400377	-	-	#N/D
TRBV11-1	7	142407672..142408136	-	-	#N/D
TRBV10-2	7	142424965..142425465	-	-	#N/D
TRBV6-5	7	142450947..142451448	-	-	0,188027316
TRBV7-4	7	142455174..142455635	-	-	0,188027316
TRBV5-4	7	142462916..142463581	-	-	0,188027316
TRBV6-6	7	142469537..142470013	-	-	0,188027316
TRBV5-5	7	142482548..142483019	-	-	0,188027316
TRBV6-7	7	142487863..142488295	-	-	#N/D
TRBV7-6	7	142492132..142492673	-	-	#N/D
TRBV5-6	7	142500028..142500534	-	-	0,188027316
TRBV6-8	7	142507382..142507810	-	-	#N/D
TRBV7-7	7	142511626..142512127	-	-	0,188027316
TRBV5-7	7	142520090..142520556	-	-	#N/D
TRBV7-9	7	142529290..142529762	-	-	0,188027316
TRBV13	7	142535809..142536292	-	-	0,188027316
TRBV10-3	7	142544212..142544685	-	-	0,188027316
TRBV11-3	7	142554836..142555318	0,02	0,11	-1,877914477
TRBV12-3	7	142560423..142560931	0,11	-	5,224730805
TRBV12-4	7	142563740..142564245	0,26	0,10	1,515286335
TRBV12-5	7	142580917..142581427	-	-	0,188027316
TRBV14	7	142587868..142588359	-	-	0,188027316
TRBV15	7	142592928..142593473	-	-	#N/D
TRBV16	7	142598016..142598469	-	-	#N/D
TRBV17	7	142601628..142602360	-	-	#N/D
TRBV18	7	142615716..142616415	-	-	#N/D
TRBV19	7	142618849..142619532	-	-	#N/D
TRBV20-1	7	142626649..142627399	-	-	#N/D
TRBV23-1	7	142645961..142646467	-	-	#N/D
TRBV24-1	7	142656701..142657213	-	-	#N/D
TRBV25-1	7	142670740..142671244	-	-	0,188027316
TRBV27	7	142715346..142715861	-	-	#N/D
TRBV28	7	142720660..142721160	-	-	#N/D
TRBV29-1	7	142740206..142740894	-	-	#N/D
PRSS1	7	142749468..142753072	-	-	0,188027316
PRSS2	7	142760398..142774564	-	-	0,188027315
TRBD1	7	142786213..142786224	-	-	#N/D
TRBJ1-1	7	142786880..142786927	-	-	#N/D
TRBJ1-2	7	142787017..142787064	-	-	#N/D
TRBJ1-3	7	142787630..142787679	-	-	#N/D
TRBJ1-4	7	142788225..142788275	-	-	#N/D
TRBJ1-5	7	142788498..142788547	-	-	#N/D
TRBJ1-6	7	142788988..142789040	-	-	#N/D
TRBC1	7	142791694..142793368	-	-	0,188027316

gene:ENSG000	7	142795705..142795720	-	-	#N/D
TRBJ2-1	7	142796365..142796414	-	-	0,188027316
TRBJ2-2	7	142796560..142796610	-	-	#N/D
TRBJ2-2P	7	142796697..142796742	-	-	#N/D
TRBJ2-3	7	142796847..142796895	-	-	0,188027316
TRBJ2-4	7	142796998..142797047	-	-	#N/D
TRBJ2-5	7	142797119..142797166	-	-	#N/D
TRBJ2-6	7	142797239..142797291	-	-	#N/D
TRBJ2-7	7	142797456..142797502	-	-	#N/D
TRBC2	7	142801041..142802748	-	-	0,188027315
TRBV30	7	complement(142812586..14281339	-	-	0,188027316
EPHB6	7	142855061..142871094	0,15	0,02	2,883472611
TRPV6	7	complement(142871208..14288574	-	-	0,188027316
TRPV5	7	complement(142908101..14293374	-	-	#N/D
LLCFC1	7	142939343..142940868	-	0,03	-3,804623889
KEL	7	complement(142941114..14296236	0,06	0,04	0,947848749
OR9A2	7	complement(143026200..14302713	0,03	-	4,516772761
OR6V1	7	143052341..143053282	-	0,01	-2,89252005
PIP	7	143132077..143139739	-	-	#N/D
TAS2R39	7	143183419..143184435	-	0,06	-5,071123353
TAS2R40	7	143222037..143223079	-	0,01	-2,89252005
gene:ENSG000	7	complement(143224042..14322423	-	-	#N/D
GSTK1	7	143244093..143270854	3,41	4,78	-0,261870726
TMEM139	7	143279957..143288048	0,14	0,12	0,478416905
CASP2	7	143288215..143307696	5,19	4,77	0,345431791
CLCN1	7	143316111..143352083	-	-	0,188027316
FAM131B	7	complement(143353400..14336277	0,55	0,48	0,433666422
ZYX	7	143381295..143391111	32,86	39,87	-0,055722523
EPHA1	7	complement(143390289..14340885	0,06	0,03	1,38420186
TAS2R60	7	143443453..143444409	0,01	-	3,068695355
TAS2R41	7	143477873..143478796	-	-	0,188027316
OR10AC1	7	complement(143509256..14351946	-	-	0,188027316
CTAGE15	7	143571801..143574387	0,10	0,06	1,015592606
TCAF2	7	143620952..143730410	0,76	0,20	2,168288058
TCAF2C	7	complement(143639230..14364764	0,34	0,12	1,769816095
CTAGE6	7	complement(143755089..14375769	-	0,01	-3,804623889
TCAF1	7	complement(143851375..14390219	9,45	8,57	0,364100396
OR2F2	7	143935233..143936186	-	-	0,188027316
OR2F1	7	143954844..143997312	-	0,00	-2,89252005
OR6B1	7	144000320..144008793	0,00	-	3,068695355
OR2A5	7	144048948..144058845	0,01	0,00	3,257511884
OR2A25	7	144069811..144075870	0,01	-	3,967268276
OR2A12	7	144086278..144098953	0,00	0,01	-0,923955849
OR2A2	7	144109583..144110539	-	-	0,188027316
OR2A14	7	144123176..144131188	0,00	0,01	-0,01185201
CTAGE4	7	144183466..144186080	0,12	0,50	-1,835232196
ARHGEF35	7	complement(144186083..14419587	1,12	1,31	0,001651839
OR2A42	7	complement(144228244..14423960	0,03	0,07	-0,979460945

OR2A7	7	complement(144257663..14426479	0,16	0,32	-0,837858054
CTAGE8	7	complement(144266674..14426928	0,35	0,05	2,894264316
OR2A1	7	144312419..144322737	0,06	0,08	-0,25262343
ARHGEF5	7	144355288..144380632	3,51	4,20	-0,034765644
NOBOX	7	complement(144397240..14441022	-	-	0,188027316
TPK1	7	complement(144451941..14483639	0,32	0,46	-0,313208636
gene:ENSG000	7	146050062..146050366	-	-	#N/D
CNTNAP2	7	146116002..148420998	0,00	0,00	1,436225396
C7orf33	7	148590766..148615860	-	-	0,188027316
CUL1	7	148697914..148801110	6,66	10,64	-0,452259104
EZH2	7	complement(148807257..14888432	1,67	2,52	-0,374795983
PDIA4	7	complement(149003062..14902866	127,12	72,41	1,034986598
ZNF786	7	complement(149069641..14909078	0,48	1,08	-0,948244511
ZNF425	7	complement(149102784..14912632	0,19	0,27	-0,272994834
ZNF398	7	149126416..149183042	2,30	2,51	0,100579681
ZNF282	7	149195546..149226238	8,01	9,50	-0,022677131
ZNF212	7	149239651..149255606	3,81	3,45	0,364729529
ZNF783	7	149262171..149297302	3,19	4,16	-0,159739315
ZNF777	7	complement(149431363..14946106	3,82	5,44	-0,285778546
ZNF746	7	complement(149472696..14949781	2,02	2,46	-0,060225217
KRBA1	7	149714781..149734575	1,51	2,12	-0,270457556
ZNF467	7	complement(149764182..14977358	0,27	1,46	-2,235397384
ZNF862	7	149838375..149867479	2,32	2,09	0,372524226
gene:ENSG000	7	149851572..149852170	0,58	0,56	0,258323606
ATP6V0E2	7	149872968..149891204	0,76	1,70	-0,937812999
gene:ENSG000	7	149881477..149882105	-	-	0,188027316
ACTR3C	7	complement(150243916..15032372	0,00	0,00	0,886720911
LRRC61	7	150323263..150338156	-	0,01	-3,804623889
RARRES2	7	complement(150338317..15034166	-	-	0,188027316
REPIN1	7	150368189..150374044	9,22	18,87	-0,809882655
ZNF775	7	150368790..150398630	1,03	1,24	-0,046667749
gene:ENSG000	7	150400702..150412470	1,39	1,48	0,130972286
GIMAP8	7	150450630..150479393	0,01	0,01	-0,579815015
GIMAP7	7	150514872..150521073	-	-	#N/D
GIMAP4	7	150567369..150573953	0,01	0,06	-2,123038518
GIMAP6	7	complement(150625375..15063264	-	-	0,188027316
GIMAP2	7	150685697..150693641	0,10	0,27	-1,161583953
GIMAP1	7	150716606..150724284	0,01	-	4,516772761
GIMAP1-GIMAP	7	150716668..150743646	-	0,02	-4,359055975
GIMAP5	7	150722253..150750033	0,01	-	3,068695355
TMEM176B	7	complement(150791285..15080136	-	0,01	-2,89252005
TMEM176A	7	150800403..150805118	-	-	#N/D
AOC1	7	150824627..150861504	-	-	#N/D
KCNH2	7	complement(150944961..15097832	0,05	0,02	1,527464488
NOS3	7	150991017..151014588	0,01	0,01	0,933337975
ATG9B	7	complement(151012209..15102449	0,01	0,02	-0,609885528
ABCB8	7	151028422..151047782	7,14	7,08	0,2337074
ASIC3	7	151048292..151052756	1,77	2,23	-0,116544731

CDK5	7	complement(151053815..15105789	8,89	8,33	0,316548747
SLC4A2	7	151057210..151076526	7,16	9,33	-0,158630761
gene:ENSG000	7	151061928..151061978	-	-	#N/D
FASTK	7	complement(151076624..15108086	23,24	21,67	0,324168633
TMUB1	7	complement(151081085..15108349	10,54	11,19	0,137339669
AGAP3	7	151085831..151144436	11,87	11,19	0,307828232
GBX1	7	complement(151148009..15117474	0,04	0,07	-0,542645869
ASB10	7	complement(151175698..15118783	-	0,01	-2,89252005
IQCA1L	7	complement(151190873..15120549	0,00	0,01	-1,478387936
H2BK1	7	complement(151207837..15121048	0,06	0,07	-0,03031053
ABCF2-H2BK1	7	complement(151207837..15122716	2,52	3,31	-0,173135562
ABCF2	7	complement(151211484..15122720	6,98	9,45	-0,212786977
CHPF2	7	151232483..151240979	18,48	14,60	0,563350777
SMARCD3	7	complement(151238764..15127789	3,04	2,96	0,263789327
NUB1	7	151341772..151378449	6,50	8,61	-0,183029367
WDR86	7	complement(151375909..15141072	0,02	0,04	-0,820024868
CRYGN	7	complement(151428832..15144081	0,02	0,00	2,144183439
RHEB	7	complement(151466012..15152012	9,58	15,57	-0,477733519
PRKAG2	7	complement(151556124..15187721	0,75	1,41	-0,693018657
GALNTL5	7	151956379..152019929	-	-	#N/D
GALNT11	7	152025674..152122340	4,42	4,21	0,291263929
KMT2C	7	complement(152134922..15243664	2,50	2,41	0,278235628
XRCC2	7	complement(152644776..15267619	1,33	2,27	-0,540244534
ACTR3B	7	152759749..152855378	1,05	1,69	-0,460541217
DPP6	7	153748133..154894285	0,00	-	3,068695355
PAXIP1	7	complement(154943687..15500312	1,95	3,13	-0,464216412
HTR5A	7	155070324..155087392	-	-	#N/D
gene:ENSG000	7	155222903..155223851	0,12	0,37	-1,323024316
INSIG1	7	155297776..155310235	32,07	5,78	2,693814506
EN2	7	155458129..155464831	0,63	0,79	-0,11175128
CNPY1	7	complement(155474206..15555545	0,00	-	3,068695355
gene:ENSG000	7	complement(155474256..15564440	0,10	0,06	0,877253457
RBM33	7	155644451..155781485	4,60	5,03	0,093212531
SHH	7	complement(155799980..15581246	-	0,01	-4,359055975
RNF32	7	156640281..156677130	0,42	0,66	-0,450527092
LMBR1	7	complement(156668946..15689321	2,48	4,15	-0,520162596
NOM1	7	156949712..156973176	2,25	2,79	-0,086655722
MNX1	7	complement(156994051..15701066	0,53	1,42	-1,194726935
UBE3C	7	157138926..157269370	16,88	21,17	-0,103517232
DNAJB6	7	157335381..157417439	4,55	6,18	-0,218867855
gene:ENSG000	7	157525783..157527325	0,05	0,02	1,704893826
PTPRN2	7	complement(157539056..15858782	0,84	1,04	-0,089687277
NCAPG2	7	complement(158631169..15870480	6,90	7,57	0,088220704
ESYT2	7	complement(158730995..15883025	14,84	12,40	0,481889628
DYNC2I1	7	158856558..158956747	1,04	1,31	-0,121142511
VIPR2	7	complement(159028175..15914486	-	-	0,188027316
OR4F21	8	complement(166086..167024)	0,03	0,01	1,436225396
ZNF596	8	232137..264703	0,27	0,26	0,277335209

FBXO25	8	406428..477967	0,80	1,19	-0,340692832
gene:ENSG000	8	complement(486117..488512)	0,10	0,02	2,38125567
TDRP	8	complement(489803..545781)	0,86	0,26	1,962399953
ERICH1	8	complement(614746..738106)	0,89	0,85	0,287193869
DLGAP2	8	737628..1708476	0,00	0,01	-1,955757882
CLN8	8	1755778..1801711	3,96	2,44	0,920619917
KBTBD11-OT1	8	1763888..1958627	2,11	0,77	1,679019064
ARHGEF10	8	1823926..1958641	10,58	9,17	0,429157298
KBTBD11	8	1973677..2006936	2,23	2,27	0,195722482
MYOM2	8	2045046..2165552	0,09	0,06	0,754754074
CSMD1	8	complement(2935353..4994972)	-	0,00	-2,89252005
MCPH1	8	6406592..6648508	0,90	1,00	0,073097757
ANGPT2	8	complement(6499632..6563409)	0,09	0,12	-0,112903715
AGPAT5	8	6708642..6761503	5,40	8,23	-0,384936004
XKR5	8	complement(6808517..6835524)	0,35	0,22	0,914073329
DEFB1	8	complement(6870592..6877936)	-	-	#N/D
DEFA6	8	complement(6924697..6926076)	-	-	#N/D
DEFA4	8	complement(6935820..6938306)	-	-	#N/D
DEFA1	8	complement(6977649..6980092)	-	-	#N/D
DEFA1B	8	complement(6996766..6999198)	-	-	#N/D
DEFA3	8	complement(7015869..7018297)	-	-	#N/D
DEFA5	8	complement(7055304..7056739)	-	-	#N/D
FAM90A15	8	7256904..7259914	0,01	-	3,068695355
FAM90A3	8	7264526..7267536	-	-	#N/D
FAM90A13	8	7279770..7282780	0,01	-	3,068695355
FAM90A5	8	7287392..7290402	0,02	-	4,516772761
FAM90A20	8	7295014..7298024	0,05	0,02	1,704893826
DEFB109B	8	7312722..7320096	0,06	-	4,516772761
USP17L1	8	7332387..7333979	0,03	0,05	-0,602143793
USP17L4	8	7337115..7338707	-	-	0,188027316
ZNF705G	8	complement(7355517..7385558)	-	-	0,188027316
DEFB108C	8	7373114..7377500	-	-	#N/D
DEFB4B	8	complement(7414855..7416863)	-	-	#N/D
DEFB103B	8	complement(7428888..7430348)	-	-	#N/D
SPAG11B	8	complement(7442684..7463674)	-	-	#N/D
DEFB104B	8	complement(7470308..7475082)	-	-	#N/D
DEFB106B	8	complement(7482504..7486400)	-	-	#N/D
DEFB105B	8	7487679..7489593	-	-	#N/D
DEFB107B	8	7495911..7509311	-	-	#N/D
PRR23D1	8	7539627..7542450	-	-	0,188027316
FAM90A7	8	complement(7556700..7559712)	-	-	#N/D
FAM90A22	8	complement(7571995..7575006)	-	-	#N/D
FAM90A23	8	complement(7579644..7582653)	0,02	-	3,967268276
FAM90A14	8	7715443..7718453	-	-	#N/D
FAM90A18	8	7723091..7726101	-	-	#N/D
FAM90A16	8	7730739..7733749	-	-	#N/D
FAM90A8	8	7738387..7741396	-	-	#N/D
FAM90A17	8	7746034..7749044	-	-	#N/D

FAM90A19	8	7753682..7756692	-	-	#N/D
FAM90A9	8	7761330..7764340	-	-	#N/D
FAM90A10	8	7768977..7771988	0,07	0,11	-0,446980808
PRR23D2	8	complement(7778591..7781413)	-	-	#N/D
DEFB107A	8	complement(7811720..7815716)	-	-	#N/D
DEFB105A	8	complement(7821004..7823880)	-	-	0,188027316
DEFB106A	8	7825139..7829053	-	-	#N/D
DEFB104A	8	7836436..7841242	-	-	#N/D
SPAG11A	8	7847876..7868867	-	-	#N/D
DEFB103A	8	7881392..7882663	-	-	#N/D
DEFB4A	8	7894677..7896716	-	-	#N/D
ZNF705B	8	7926337..7952413	-	-	#N/D
USP17L8	8	complement(7971661..7973253)	-	-	#N/D
USP17L3	8	complement(7976393..7977985)	-	-	0,188027316
DEFB109C	8	complement(7990270..7997645)	0,02	-	3,068695355
FAM90A11	8	complement(8012342..8015352)	0,05	-	5,697545031
FAM90A24	8	complement(8019988..8022998)	-	-	0,188027316
FAM90A12	8	complement(8027636..8030646)	-	-	#N/D
PRAG1	8	complement(8317736..8386439)	1,59	4,04	-1,118166577
CLDN23	8	8701937..8704096	0,44	0,53	-0,024232739
MFHAS1	8	complement(8783354..8893630)	7,72	8,62	0,063300284
ERI1	8	9002147..9116746	4,34	4,58	0,146453707
PPP1R3B	8	complement(9136255..9151574)	2,68	2,81	0,156970667
TNKS	8	9555912..9782346	6,41	8,50	-0,185092954
MSRA	8	10054292..10428891	2,08	2,10	0,210874432
PRSS51	8	complement(10482878..10547624)	0,01	0,01	0,921097799
PRSS55	8	10525532..10554166	-	-	0,188027316
RP1L1	8	complement(10606349..10712187)	0,09	0,40	-1,942208657
C8orf74	8	10672628..10700590	0,00	0,04	-2,752886291
SOX7	8	complement(10723768..10730511)	1,77	6,43	-1,640063459
PINX1_1	8	complement(10725399..10839847)	0,11	0,71	-2,445395909
PINX1_2	8	complement(10764961..10839884)	1,67	2,28	-0,227252388
gene:ENSG000	8	complement(10856085..10859436)	0,00	0,00	-0,01185201
XKR6	8	complement(10896045..11201833)	0,37	0,35	0,318409904
MTMR9	8	11284816..11328146	2,51	2,84	0,046855599
SLC35G5	8	11331012..11332360	0,14	0,06	1,524737598
FAM167A	8	complement(11421476..11475908)	0,78	4,45	-2,281600729
BLK	8	11486894..11564599	0,00	0,05	-2,854330803
GATA4	8	11676959..11760002	0,11	0,01	4,228586218
NEIL2	8	11769639..11787345	5,27	13,91	-1,177703739
FDFT1	8	11795573..11839395	36,67	18,53	1,207869037
CTSB	8	complement(11842524..11869533)	105,70	88,95	0,471943021
DEFB136	8	complement(11973937..11974599)	-	-	#N/D
DEFB135	8	11982256..11984590	-	-	#N/D
DEFB134	8	complement(11993174..12000752)	-	-	#N/D
DEFB130B	8	complement(12064389..12071747)	-	-	0,188027316
ZNF705D	8	12089338..12115516	-	-	0,188027316
USP17L7	8	complement(12132417..12134099)	0,03	0,18	-2,267318466

USP17L2	8	complement(12136435..12138849)	0,02	0,20	-3,199972692
DEFB109D	8	complement(12150888..12158033)	0,04	-	3,068695355
FAM86B1	8	complement(12182096..12194133)	1,13	1,83	-0,476593749
DEFB130A	8	complement(12310962..12318316)	-	-	#N/D
FAM86B2	8	complement(12424411..12436406)	0,31	0,65	-0,844357529
LONRF1	8	complement(12721906..12756073)	0,69	1,41	-0,804813925
TRMT9B	8	12945642..13031503	0,06	0,21	-1,667678763
DLC1	8	complement(13083361..13604610)	11,52	25,80	-0,940573969
C8orf48	8	13566869..13568288	0,17	0,20	-0,039381237
SGCZ	8	complement(14084845..15238431)	0,01	0,01	0,7939095
TUSC3	8	15417215..15766649	9,72	13,33	-0,232336389
MSR1	8	complement(16107878..16567490)	0,03	0,07	-0,688055268
FGF20	8	complement(16992181..17002345)	-	0,01	-2,89252005
MICU3	8	17027238..17122642	0,13	0,22	-0,53662877
ZDHHC2	8	17156482..17224799	1,83	3,78	-0,824821523
CNOT7	8	complement(17224966..17246878)	4,11	6,13	-0,35386709
VPS37A	8	17246931..17302427	3,06	5,13	-0,522929151
MTMR7	8	complement(17296794..17413528)	0,11	0,11	0,232014551
SLC7A2	8	17497088..17570573	0,58	3,14	-2,217163519
gene:ENSG000	8	complement(17498647..17499238)	-	0,22	-6,052163584
gene:ENSG000	8	17498737..17499157	0,03	-	3,068695355
SLC7A2-IT1	8	17500012..17500605	-	0,02	-2,89252005
PDGFRL	8	17576433..17644071	0,37	1,91	-2,138400111
MTUS1	8	complement(17643795..17801094)	0,33	1,08	-1,491199659
FGL1	8	complement(17864380..17910365)	-	-	0,188027316
PCM1	8	17922842..18029948	4,58	6,96	-0,382074565
ASAH1	8	complement(18055992..18084998)	1,87	3,72	-0,774225387
NAT1	8	18170477..18223689	0,12	0,25	-0,837858054
NAT2	8	18391282..18401218	-	0,01	-2,89252005
PSD3	8	complement(18527303..19084730)	5,62	7,74	-0,237279395
gene:ENSG000	8	complement(18864681..18865247)	0,65	0,53	0,521284552
gene:ENSG000	8	19277284..19277573	-	-	#N/D
SH2D4A	8	19313693..19396218	4,83	12,46	-1,143245827
gene:ENSG000	8	complement(19375128..19375622)	0,02	-	3,068695355
CSGALNACT1	8	complement(19404161..19758029)	0,13	1,62	-3,462863794
INTS10	8	19817391..19852083	13,83	16,82	-0,059157122
LPL	8	19901717..19967259	-	0,01	-3,804623889
SLC18A1	8	complement(20144855..20183206)	-	-	#N/D
ATP6V1B2	8	20197381..20226819	10,44	15,12	-0,310562689
LZTS1	8	complement(20246165..20303963)	1,16	0,32	2,078307643
gene:ENSG000	8	complement(21129990..21130100)	-	-	#N/D
GFRA2	8	complement(21690398..21812357)	0,13	0,01	4,336935047
DOK2	8	complement(21908873..21913690)	-	0,01	-2,89252005
XPO7	8	21919662..22006585	38,01	40,55	0,129742901
NPM2	8	22024125..22036897	0,02	-	4,516772761
FGF17	8	22042398..22048809	0,02	0,03	-0,429837071
DMTN	8	22048995..22082527	1,12	2,34	-0,838305216
FHIP2B	8	22089150..22104911	16,13	16,89	0,157050727

NUDT18	8	complement(22106874..22109419)	5,24	5,51	0,15056136
HR	8	complement(22114419..22133384)	4,81	20,26	-1,852099348
HRURF	8	complement(22130458..22131010)	-	-	#N/D
REEP4	8	complement(22138020..22141951)	27,56	34,25	-0,090643581
LGI3	8	complement(22146830..22157084)	0,04	0,05	-0,2582584
SFTPC	8	22156913..22164479	0,01	0,04	-2,190455314
BMP1	8	22165140..22212326	6,23	10,14	-0,478865473
PHYHIP	8	complement(22219703..22232101)	0,38	0,94	-1,078164554
POLR3D	8	22245133..22254601	30,10	17,08	1,040887078
PIWIL2	8	22275316..22357568	0,11	0,20	-0,667072477
SLC39A14	8	22367278..22434129	33,16	48,57	-0,327354281
PPP3CC	8	22440819..22541142	2,57	4,16	-0,468772295
SORBS3	8	22544986..22575788	24,15	32,59	-0,209013167
PDLIM2	8	22578279..22598025	3,67	3,85	0,151281014
gene:ENSG000	8	22589274..22602084	0,48	0,39	0,5160204
C8orf58	8	22599599..22604150	2,66	3,05	0,023580474
CCAR2	8	22604757..22620964	22,94	24,82	0,109525696
BIN3	8	complement(22620418..22669148)	6,46	7,00	0,106446423
EGR3	8	complement(22687659..22693480)	0,03	0,09	-1,567022657
PEBP4	8	complement(22713251..23000000)	0,02	0,01	0,886720911
RHOBTB2	8	22987417..23020509	5,09	5,76	0,044509797
TNFRSF10B	8	complement(23020133..23069031)	65,83	71,55	0,102941438
gene:ENSG000	8	23084403..23115536	1,82	2,70	-0,344322823
TNFRSF10C	8	23102921..23117445	0,66	1,22	-0,646883614
TNFRSF10D	8	complement(23135588..23164027)	0,02	0,01	0,533811434
TNFRSF10A	8	complement(23190452..23225102)	4,23	11,59	-1,230986359
CHMP7	8	23243637..23262000	12,29	16,48	-0,20042495
R3HCC1	8	23270120..23296279	4,12	7,63	-0,667210622
LOXL2	8	complement(23296897..23425328)	137,08	73,64	1,119555404
ENTPD4	8	complement(23385783..23457695)	9,59	15,84	-0,501954655
SLC25A37	8	23528956..23575463	22,20	27,08	-0,063286677
NKX3-1	8	complement(23678697..23682938)	0,52	3,31	-2,439332643
NKX2-6	8	complement(23701740..23706756)	-	-	0,188027316
STC1	8	complement(23841929..23854806)	0,07	0,28	-1,687083277
ADAM28	8	24294069..24359014	0,02	0,01	0,78195819
ADAMDEC1	8	24384285..24406013	-	-	0,188027316
ADAM7	8	24440930..24526970	-	-	0,188027316
NEFM	8	24913758..24919098	0,04	0,03	0,652912318
NEFL	8	complement(24950955..24956721)	0,04	0,10	-1,199187398
DOCK5	8	25184689..25418082	8,31	11,60	-0,259231203
GNRH1	8	complement(25419258..25424654)	1,17	0,97	0,491873527
KCTD9	8	complement(25427847..25458476)	7,15	8,58	-0,04086896
CDCA2	8	25459199..25507911	14,91	17,26	0,011922287
EBF2	8	complement(25841725..26045413)	0,02	0,01	1,505976878
PPP2R2A	8	26291508..26372680	7,45	11,97	-0,461048452
BNIP3L	8	26383054..26505636	16,51	11,15	0,788631318
PNMA2	8	complement(26504701..26514092)	0,34	1,14	-1,547491253
DPYSL2	8	26514031..26658178	38,81	33,13	0,45150763

ADRA1A	8	complement(26748150..26867278)	0,00	-	3,068695355
STMN4	8	complement(27235308..27258420)	0,00	0,00	-0,01185201
TRIM35	8	complement(27284886..27311272)	8,33	8,97	0,115099341
PTK2B	8	27311482..27459391	0,47	0,63	-0,206224897
CHRNA2	8	complement(27459756..27479883)	0,00	0,00	-0,01185201
EPHX2	8	27490781..27545564	1,35	1,43	0,13917316
CLU	8	complement(27596917..27614700)	11,24	29,55	-1,171338957
SCARA3	8	27633868..27676776	38,31	38,67	0,209528221
gene:ENSG000	8	27701194..27703789	0,29	0,22	0,615908439
CCDC25	8	complement(27733316..27772653)	4,91	9,33	-0,702117901
ESCO2	8	27771949..27812640	1,22	2,63	-0,88749953
PBK	8	complement(27809624..27838082)	7,48	16,79	-0,942197451
SCARA5	8	complement(27869883..27992673)	0,26	2,20	-2,861959026
NUGGC	8	complement(28021964..28083936)	-	0,00	-2,89252005
ELP3	8	28089673..28191156	6,52	8,91	-0,227327107
PNOC	8	28316986..28343355	0,01	-	3,068695355
ZNF395	8	complement(28345590..28402701)	22,84	16,04	0,732270429
FBXO16	8	complement(28348287..28490278)	0,17	0,38	-0,922272643
FZD3	8	28494205..28574267	0,00	0,02	-2,640348898
EXTL3	8	28600469..28756561	8,49	11,35	-0,194938177
INTS9	8	complement(28767661..28890242)	2,69	5,98	-0,930574088
HMBOX1	8	28890395..29064764	4,70	5,09	0,11028254
KIF13B	8	complement(29067278..29263124)	0,95	2,55	-1,195745915
DUSP4	8	complement(29333064..29350684)	8,20	10,81	-0,174960564
gene:ENSG000	8	29587001..29588426	0,04	0,05	-0,291161992
SARAF	8	complement(30063003..30083208)	18,89	25,97	-0,236012084
LEPROTL1	8	30095408..30177208	2,96	5,20	-0,59265242
MBOAT4	8	complement(30131671..30144665)	0,07	0,08	-0,03760504
DCTN6	8	30156319..30183639	4,12	6,12	-0,349154757
RBPMS	8	30384511..30572256	2,52	5,09	-0,791532553
gene:ENSG000	8	30552345..30553766	0,44	1,06	-1,03864658
GTF2E2	8	complement(30578318..30658236)	10,01	12,39	-0,08395356
SMIM18	8	30638580..30646064	0,08	0,06	0,750935198
GSR	8	complement(30678066..30727846)	34,48	37,73	0,093199622
UBXN8	8	30729131..30767006	1,75	1,77	0,204906147
PPP2CB	8	complement(30774457..30814314)	13,40	18,70	-0,25785246
TEX15	8	complement(30831544..30913008)	-	0,00	-4,359055975
PURG	8	complement(30995802..31033715)	0,03	0,06	-0,708502297
WRN	8	31033788..31176138	1,55	3,63	-1,000652074
NRG1	8	31639222..32855666	0,13	1,03	-2,80590735
gene:ENSG000	8	32647202..32647390	-	-	#N/D
FUT10	8	complement(33370824..33473146)	1,93	2,84	-0,339764701
TTI2	8	complement(33473386..33513185)	5,26	6,59	-0,101628462
MAK16	8	33485182..33501262	4,13	7,28	-0,596190077
RNF122	8	complement(33547754..33567128)	2,98	4,34	-0,316267218
DUSP26	8	complement(33591330..33600023)	0,02	-	4,516772761
UNC5D	8	35235475..35796550	-	-	0,188027316
gene:ENSG000	8	35254344..35256605	-	-	#N/D

KCNU1	8	36784324..36936125	-	-	0,188027316
ZNF703	8	37695782..37700019	1,75	2,29	-0,166769505
ERLIN2	8	37736601..37758422	7,86	8,28	0,148737617
PLPBP	8	37762595..37779768	6,27	8,89	-0,280702932
ADGRA2	8	37784191..37844896	3,25	8,83	-1,220553203
BRF2	8	complement(37843268..37849861)	9,00	3,96	1,408170575
RAB11FIP1	8	complement(37858618..37899497)	2,94	4,49	-0,388086799
gene:ENSG000	8	37858949..37861333	-	-	0,188027316
GOT1L1	8	complement(37934281..37940124)	-	-	#N/D
gene:ENSG000	8	complement(37934340..37965953)	-	-	0,188027316
ADRB3	8	complement(37962990..37966599)	-	-	0,188027316
EIF4EBP1	8	38030534..38060365	97,58	#####	-0,521387786
ASH2L	8	38105493..38144076	7,98	11,48	-0,302824896
STAR	8	complement(38142700..38150992)	0,08	0,13	-0,488934172
LSM1	8	complement(38163335..38176730)	8,40	8,34	0,233988465
BAG4	8	38176533..38213301	4,35	4,43	0,198279209
DDHD2	8	38225218..38275558	7,25	9,78	-0,20872963
PLPP5	8	complement(38263130..38269243)	7,83	5,01	0,867076888
NSD3	8	complement(38269704..38382272)	6,49	7,74	-0,030806488
LETM2	8	38386207..38409527	0,73	1,71	-0,997334732
FGFR1	8	complement(38400215..38468834)	15,92	26,34	-0,503204803
TACC1	8	38728186..38853028	9,48	11,80	-0,093387847
PLEKHA2	8	38901235..38973912	10,41	17,67	-0,540376583
HTRA4	8	38974228..38988663	0,01	0,02	-1,478387936
TM2D2	8	complement(38988808..38996824)	3,49	3,73	0,124149154
ADAM9	8	38996754..39105445	22,10	17,28	0,577863608
ADAM32	8	39106990..39284917	0,10	0,12	-0,088430208
ADAM18	8	39584489..39730065	-	-	#N/D
ADAM2	8	complement(39743735..39838227)	-	-	#N/D
IDO1	8	39902275..39928790	0,05	0,95	-3,946260392
IDO2	8	39934614..40016392	-	0,03	-5,327865481
TCIM	8	40153482..40155310	0,18	1,75	-3,075606654
ZMAT4	8	complement(40530590..40897833)	0,00	0,00	-0,01185201
SFRP1	8	complement(41261962..41309473)	82,39	47,69	1,01192838
GOLGA7	8	41488200..41511095	5,68	6,64	-0,003548322
GINS4	8	41529218..41545030	7,81	5,71	0,674594263
GPAT4	8	41577187..41625001	10,68	10,82	0,204885873
NKX6-3	8	complement(41645177..41650817)	-	0,01	-3,804623889
ANK1	8	complement(41653220..41896741)	0,89	4,23	-2,026770319
KAT6A	8	complement(41929479..42051994)	5,40	8,04	-0,351001815
AP3M2	8	42152946..42171673	4,04	8,51	-0,851126484
PLAT	8	complement(42174718..42207709)	6,12	31,51	-2,140040046
IKBKB	8	42271302..42332460	7,63	10,96	-0,299306003
POLB	8	42338454..42371808	3,70	6,94	-0,685709077
DKK4	8	complement(42374063..42377229)	-	0,01	-2,89252005
VDAC3	8	42391624..42405937	18,49	28,08	-0,379772936
SLC20A2	8	complement(42416475..42541926)	3,14	3,73	-0,022123854
SMIM19	8	42541155..42555195	1,20	2,48	-0,825930379

CHRNB3	8	42697366..42737407	-	-	#N/D
CHRNA6	8	complement(42752620..42796392)	-	-	0,188027316
THAP1	8	complement(42836674..42843325)	2,18	2,74	-0,108500906
RNF170	8	complement(42849637..42897290)	1,07	1,94	-0,629950337
HOOK3	8	42896946..43030535	4,26	3,94	0,335751641
gene:ENSG000	8	43018424..43077334	-	-	#N/D
FNTA	8	43034194..43085788	4,00	5,05	-0,115105301
POMK	8	43093498..43131180	7,85	8,00	0,195394115
HGSNAT	8	43140464..43202855	5,37	7,44	-0,246000684
POTEA	8	43292483..43363518	-	-	0,188027316
SPIDR	8	47260878..47736306	6,82	8,91	-0,162507919
CEBPD	8	complement(47736913..47738164)	4,61	11,57	-1,10523141
PRKDC	8	complement(47773111..47960178)	35,63	54,83	-0,398727307
MCM4	8	47960185..47978160	33,13	42,62	-0,1401892
UBE2V2	8	48008415..48064708	1,87	3,17	-0,537038547
gene:ENSG000	8	complement(48428143..48431041)	-	0,02	-4,758582517
CLXN	8	complement(48710789..48735311)	-	-	#N/D
SNAI2	8	complement(48917598..48921740)	2,20	3,47	-0,434027893
PPDPFL	8	49054311..49076093	-	-	0,188027316
SNTG1	8	49909789..50796692	0,00	-	3,068695355
PXDNL	8	complement(51319577..51809445)	0,02	0,05	-1,177556582
PCMTD1	8	complement(51817575..51899186)	1,71	1,42	0,494191326
ST18	8	complement(52110838..52460959)	0,03	0,05	-0,841031413
ALKAL1	8	complement(52534037..52565430)	0,01	-	3,068695355
RB1CC1	8	complement(52622458..52745843)	4,70	9,09	-0,727935371
NPBWR1	8	52939182..52943734	0,01	-	5,224730805
OPRK1	8	complement(53225724..53251637)	0,01	-	5,480421266
ATP6V1H	8	complement(53715543..53843558)	4,06	5,48	-0,208772963
RGS20	8	53851795..53959303	0,49	0,53	0,103629615
TCEA1	8	complement(53966552..54022448)	11,42	23,08	-0,791418896
LYPLA1	8	complement(54046367..54102017)	6,83	11,50	-0,528472765
MRPL15	8	54135241..54148514	6,82	11,06	-0,473101216
SOX17	8	54457935..54460892	0,51	1,19	-0,99351865
RP1	8	54509422..54871720	0,00	0,00	0,278120973
XKR4	8	55102028..55542054	0,00	0,00	1,15046174
TMEM68	8	complement(55696424..55773407)	1,49	2,21	-0,346266712
TGS1	8	55773446..55826445	1,80	4,32	-1,04167531
LYN	8	55879835..56014169	1,15	2,79	-1,059889598
RPS20	8	complement(56067254..56074510)	29,96	35,49	-0,021177731
MOS	8	complement(56112942..56113982)	-	-	#N/D
PLAG1	8	complement(56160909..56211324)	0,74	0,56	0,605703828
CHCHD7	8	56211686..56218809	1,75	0,96	1,089164073
SDR16C5	8	complement(56300005..56320776)	-	-	0,188027316
PENK	8	complement(56436674..56446671)	-	-	#N/D
BPNT2	8	complement(56957931..56993867)	15,50	14,23	0,346968832
FAM110B	8	57994523..58204279	1,12	1,53	-0,227865267
UBXN2B	8	58411359..58451501	1,60	4,50	-1,264947929
CYP7A1	8	complement(58490178..58500163)	-	0,00	-2,89252005

SDCBP	8	58552924..58582859	16,40	24,72	-0,368893045
NSMAF	8	complement(58583508..58659853)	5,05	10,14	-0,78435493
TOX	8	complement(58805412..59119147)	0,69	1,07	-0,410536542
CA8	8	complement(60185412..60281400)	0,00	-	3,068695355
RAB2A	8	60516936..60623644	7,75	9,31	-0,041286379
CHD7	8	60678740..60868028	0,16	0,11	0,764125105
CLVS1	8	61057158..61501645	0,08	0,08	0,248330452
ASPH	8	complement(61500556..61714640)	21,90	17,97	0,50844412
NKAIN3	8	62248591..63014000	0,07	0,03	1,297868377
GGH	8	complement(63014881..63039407)	8,31	9,87	-0,025510309
TTPA	8	complement(63048553..63086053)	0,14	0,06	1,493871253
YTHDF3	8	63168553..63212786	7,31	7,44	0,197028025
BHLHE22	8	64580365..64583627	-	-	#N/D
CYP7B1	8	complement(64587763..64798737)	-	-	0,188027316
ARMC1	8	complement(65602458..65634217)	6,30	8,46	-0,202004981
MTFR1	8	65644734..65771261	7,82	9,07	0,008724315
PDE7A	8	complement(65714334..65842322)	1,40	1,82	-0,154463129
DNAJC5B	8	66021553..66101245	0,14	0,01	3,680943824
TRIM55	8	66126896..66175485	0,06	0,05	0,372224007
CRH	8	complement(66176376..66178464)	0,03	0,01	1,833201638
RRS1	8	66429014..66430733	4,04	6,53	-0,469150261
gene:ENSG000	8	66430185..66445324	-	-	0,188027316
gene:ENSG000	8	66432486..66518524	-	0,04	-6,532127252
ADHFE1	8	66432492..66468907	0,26	0,18	0,727860646
VXN	8	66492326..66518524	0,20	0,17	0,407898783
MYBL1	8	complement(66562175..66614247)	2,55	3,09	-0,054760217
VCPIP1	8	complement(66628487..66667231)	3,48	3,41	0,251716076
C8orf44-SGK3	8	66667596..66860472	0,57	0,46	0,51622761
SGK3	8	66712734..66862022	0,35	0,58	-0,504234411
MCMDC2	8	66870749..66922048	0,19	0,29	-0,411543023
TCF24	8	complement(66946501..66962591)	-	-	0,188027316
PPP1R42	8	complement(66964099..67056604)	-	0,01	-2,89252005
COPS5	8	complement(67043079..67083783)	10,17	10,98	0,111578684
CSPP1	8	67062417..67196778	0,65	1,04	-0,453856375
ARFGEF1	8	complement(67173511..67343781)	3,76	4,69	-0,096889159
CPA6	8	complement(67422038..67746378)	0,01	0,26	-4,294043836
PREX2	8	67952046..68237032	-	-	#N/D
C8orf34	8	68330955..68819023	0,00	0,01	-0,820024868
SULF1	8	69466624..69660915	0,02	0,26	-3,274163821
SLCO5A1	8	complement(69667046..69834978)	0,01	0,09	-3,06014616
gene:ENSG000	8	69938168..69938400	-	-	#N/D
PRDM14	8	complement(70051651..70071693)	-	-	0,188027316
NCOA2	8	complement(70109782..70403808)	3,07	3,76	-0,070768043
TRAM1	8	complement(70573218..70608416)	12,43	15,00	-0,047808482
LACTB2	8	complement(70635318..70669185)	1,32	2,12	-0,461037557
XKR9	8	70669339..70790371	0,02	0,01	1,232079601
EYA1	8	complement(71197433..71592025)	0,17	0,36	-0,861184345
MSC	8	complement(71841560..71844412)	2,35	2,91	-0,084059169

TRPA1	8	complement(72019917..72075584)	0,68	0,39	1,038010068
KCNB2	8	72537225..72938349	-	-	0,188027316
TERF1	8	73008856..73048123	1,59	2,49	-0,418046958
SBSPON	8	complement(73064543..73124088)	0,03	0,05	-0,667094398
C8orf89	8	complement(73241331..73259530)	-	-	#N/D
RPL7	8	complement(73290242..73295789)	119,06	#####	-0,15438538
RDH10	8	73294602..73325281	1,38	2,17	-0,431249998
STAU2	8	complement(73420369..73747708)	1,46	3,40	-0,999474418
gene:ENSG000	8	complement(73688691..73878854)	1,97	3,37	-0,548709117
UBE2W	8	complement(73780097..73878910)	1,01	1,30	-0,147136572
ELOC	8	complement(73939169..73972307)	2,90	3,62	-0,095379017
TMEM70	8	73972437..73982783	1,63	2,59	-0,446709823
LY96	8	73991392..74029079	0,12	0,63	-2,180662299
JPH1	8	complement(74234700..74321540)	0,08	1,26	-3,831913121
GDAP1	8	74320613..74518007	0,11	0,42	-1,765630517
PI15	8	74824534..74855029	-	0,01	-5,071123353
CRISPLD1	8	74984505..75034558	0,94	1,61	-0,555953972
HNF4G	8	75407914..75566834	0,12	0,59	-2,016033648
ZFHX4	8	76681239..76867281	2,32	3,44	-0,348179196
PEX2	8	complement(76980258..77001044)	5,23	5,01	0,285123805
PKIA	8	78516340..78605267	0,20	0,64	-1,492779647
ZC2HC1A	8	78666050..78719765	0,54	1,20	-0,944949736
IL7	8	complement(78675743..78805523)	0,24	0,55	-0,965925409
STMN2	8	79611117..79666158	-	-	0,188027316
HEY1	8	complement(79762371..79767857)	0,94	0,87	0,345769486
MRPS28	8	complement(79918717..80030289)	5,22	6,50	-0,092367755
gene:ENSG000	8	complement(79918860..80080775)	0,01	0,10	-3,431345623
TPD52	8	complement(80034745..80231232)	1,68	5,69	-1,54010177
ZBTB10	8	80485619..80526265	1,09	1,54	-0,265797763
ZNF704	8	complement(80628451..80874781)	0,85	0,77	0,372537449
PAG1	8	complement(80967810..81112068)	0,34	0,34	0,228068029
FABP5	8	81280536..81284777	1,05	0,07	4,125693624
PMP2	8	complement(81440326..81447439)	-	-	0,188027316
FABP9	8	complement(81458253..81461579)	-	-	#N/D
FABP4	8	complement(81478419..81483236)	-	0,18	-6,426264264
FABP12	8	complement(81524981..81590139)	-	-	0,188027316
IMPA1	8	complement(81656914..81686331)	1,83	3,50	-0,711750369
SLC10A5	8	complement(81693631..81695058)	0,02	-	3,967268276
gene:ENSG000	8	81696368..81698694	0,11	0,25	-0,942651554
ZFAND1	8	complement(81701334..81732903)	3,29	4,72	-0,301092261
CHMP4C	8	81732448..81759515	-	-	0,188027316
SNX16	8	complement(81799581..81842866)	0,58	0,80	-0,238372146
RALYL	8	84182787..84921844	-	0,00	-2,89252005
LRRCC1	8	85107215..85146080	0,08	0,18	-1,017144031
E2F5	8	85177154..85217158	1,02	1,63	-0,456446736
RBIS	8	complement(85214048..85220421)	3,68	5,46	-0,344584998
CA13	8	85220587..85284073	0,49	0,68	-0,260587286
CA1	8	complement(85327608..85379014)	0,00	0,02	-1,877914477

CA3	8	85373436..85449040	0,03	0,02	0,677647514
CA2	8	85463968..85481493	0,82	1,36	-0,499643331
ATP6V0D2	8	85987323..86154225	0,06	0,30	-2,187933007
PSKH2	8	complement(86047109..86088621)	0,00	-	3,068695355
SLC7A13	8	complement(86214063..86321146)	-	-	#N/D
WWP1	8	86342547..86478420	3,79	5,70	-0,364771718
RMDN1	8	complement(86468257..86514357)	3,54	3,80	0,117494852
CPNE3	8	86514435..86561498	12,12	14,41	-0,02672535
CNGB3	8	complement(86553977..86743675)	0,00	0,02	-2,854330803
CNBD1	8	86866415..87615219	-	0,01	-3,804623889
DCAF4L2	8	complement(87870747..87874015)	-	-	#N/D
MMP16	8	complement(88032011..88328025)	0,65	1,28	-0,751564283
RIPK2	8	89757806..89791064	7,09	10,50	-0,342779562
OSGIN2	8	89901849..89927888	1,75	2,70	-0,401867393
NBN	8	complement(89924515..90003228)	1,26	2,75	-0,903852564
DECR1	8	90001405..90053633	2,06	2,23	0,107493176
CALB1	8	complement(90058608..90095475)	0,01	0,02	-0,979341556
TMEM64	8	complement(90621995..90791632)	1,25	2,02	-0,472624569
NECAB1	8	90791741..90959393	0,05	0,06	-0,154366259
C8orf88	8	complement(90958471..90985238)	3,28	6,72	-0,809002784
PIP4P2	8	complement(90993802..91040872)	2,14	3,14	-0,329347233
OTUD6B	8	91070196..91087095	1,01	1,46	-0,31338577
LRRC69	8	91101832..91219257	0,02	0,10	-1,854949791
SLC26A7	8	91209494..91398155	0,01	0,01	-0,999120035
RUNX1T1	8	complement(91954972..92103385)	-	0,00	-3,804623889
TRIQK	8	complement(92883532..93017673)	2,73	2,63	0,276844474
CIBAR1	8	93698561..93731527	0,94	1,51	-0,4525694
RBM12B	8	complement(93728155..93741017)	3,18	3,48	0,095202953
RBM12B-AS1	8	93740121..93740773	1,63	2,24	-0,23112813
TMEM67	8	93754844..93819234	0,43	0,85	-0,750064784
PDP1	8	93857807..93926068	5,15	9,84	-0,70955092
CDH17	8	complement(94127162..94217303)	0,02	0,03	-0,35396495
GEM	8	complement(94249253..94262350)	1,48	2,01	-0,213175465
RAD54B	8	complement(94371960..94475115)	1,49	1,91	-0,134342944
FSBP	8	complement(94378377..94436952)	0,28	0,34	-0,040712532
VIRMA	8	complement(94487689..94553529)	9,90	9,41	0,296634353
ESRP1	8	94641074..94707466	-	0,19	-8,621133689
DPY19L4	8	94719703..94793836	1,26	2,07	-0,491695378
INTS8	8	94813311..94881746	2,95	4,11	-0,25864746
CCNE2	8	complement(94879770..94896678)	0,49	0,80	-0,485513214
NDUFAF6	8	94895533..95116455	0,59	1,18	-0,791097171
TP53INP1	8	complement(94925972..94949378)	0,61	0,22	1,711526018
PLEKHF2	8	95115642..95156698	1,15	1,07	0,324280941
CFAP418	8	complement(95244913..95269201)	0,89	1,11	-0,097118144
GDF6	8	complement(96142333..96160806)	0,33	0,10	1,992330895
UQCRB	8	complement(96222947..96235546)	0,83	1,36	-0,488495935
MTERF3	8	complement(96239398..96261610)	2,30	5,08	-0,921170239
PTDSS1	8	96261902..96336995	14,64	16,63	0,039004799

SDC2	8	96493813..96611790	0,71	2,35	-1,497567927
CPQ	8	96645242..97149654	2,72	3,47	-0,129484751
TSPYL5	8	complement(97273488..97277928)	-	0,00	-2,89252005
MTDH	8	97644184..97730260	13,15	16,88	-0,137033603
LAPTM4B	8	97775057..97853013	34,36	49,40	-0,300910193
MATN2	8	97868840..98036724	0,39	0,61	-0,396859423
RPL30	8	complement(98024851..98046469)	98,96	#####	-0,236790457
ERICH5	8	98064522..98093610	-	-	0,188027316
RIDA	8	complement(98102344..98117171)	3,19	4,47	-0,265149968
POP1	8	98117293..98159835	5,03	5,87	0,001235685
NIPAL2	8	complement(98189826..98294393)	0,76	0,64	0,472266212
STK3	8	complement(98371228..98942827)	2,20	3,00	-0,229146258
gene:ENSG000	8	complement(98391401..98391514)	-	-	#N/D
KCNS2	8	98426958..98432853	0,04	0,02	1,306856523
OSR2	8	98944403..98952104	0,26	0,25	0,258500025
VPS13B	8	99013266..99877580	1,89	2,84	-0,363823394
COX6C	8	complement(99873200..99893707)	8,93	10,84	-0,055945401
RGS22	8	complement(99960936..100131268	-	-	0,188027316
FBXO43	8	complement(100133351..10014581	0,51	1,07	-0,842202212
POLR2K	8	100150623..100154003	8,66	9,14	0,144004766
SPAG1	8	100157906..100259278	0,19	0,76	-1,811767632
RNF19A	8	complement(100257060..10033621	2,88	3,02	0,15191977
ANKRD46	8	complement(100509752..10055978	1,09	1,92	-0,58932884
SNX31	8	complement(100572889..10066341	0,05	0,01	2,028855185
PABPC1	8	complement(100685816..10072280	113,32	#####	-0,230782175
YWHAZ	8	complement(100916523..10095338	54,54	70,19	-0,140772849
ZNF706	8	complement(101177878..10120619	3,48	5,14	-0,337104845
GRHL2	8	101492439..101669726	-	-	0,188027316
NCALD	8	complement(101686542..10212490	-	-	0,188027316
RRM2B	8	complement(102204502..10223896	1,48	1,84	-0,093440184
UBR5	8	complement(102252273..10241275	14,37	11,13	0,591807239
ODF1	8	102551589..102561018	-	-	0,188027316
KLF10	8	complement(102648784..10265572	8,37	4,68	1,061858705
AZIN1	8	complement(102826111..10289386	19,65	30,29	-0,401064773
gene:ENSG000	8	complement(102858046..10286386	-	-	#N/D
ATP6V1C1	8	103021063..103073051	2,27	3,62	-0,450077267
BAALC	8	103140713..103230305	0,04	0,03	0,415033091
FZD6	8	103298433..103332866	3,39	6,61	-0,738894019
CTHRC1	8	103371538..103382989	0,17	0,21	-0,039701368
SLC25A32	8	complement(103398635..10341518	7,14	10,55	-0,339172963
gene:ENSG000	8	complement(103398658..10350189	-	0,04	-5,902279478
DCAF13	8	103414714..103443453	10,07	9,11	0,367266316
RIMS2	8	103500610..104254430	0,00	-	3,068695355
DPYS	8	complement(104330324..10446705	-	0,01	-2,89252005
DCSTAMP	8	104339087..104356689	-	-	#N/D
gene:ENSG000	8	complement(104478539..10448078	-	0,03	-5,327865481
LRP12	8	complement(104489231..10458925	3,40	6,41	-0,6917455
ZFPM2	8	104590733..105804539	3,94	4,59	0,002751384

OXR1	8	106270144..106752694	1,88	2,07	0,089166853
ABRA	8	complement(106759483..10677024	0,04	0,02	1,391449417
ANGPT1	8	complement(107249482..10749805	0,18	0,17	0,333865958
RSPO2	8	complement(107899316..10808364	0,02	0,01	1,487770062
EIF3E	8	complement(108162787..10844349	9,78	9,65	0,241324673
EMC2	8	108443601..108489196	2,19	1,40	0,861974877
TMEM74	8	complement(108606850..10878759	0,68	0,05	3,948358132
TRHR	8	109086585..109121565	0,05	0,02	1,138491112
NUDCD1	8	complement(109240919..10933412	5,75	3,84	0,806152853
ENY2	8	109334324..109345954	3,11	2,34	0,633220615
PKHD1L1	8	109362461..109537207	0,01	0,03	-1,423700593
EBAG9	8	109539711..109570072	1,53	2,52	-0,494259137
gene:ENSG000	8	109539726..109539800	-	-	0,188027316
SYBU	8	complement(109573978..10969179	0,11	1,30	-3,334117684
KCNV1	8	complement(109963636..10997577	0,00	0,04	-2,640348898
CSMD3	8	complement(112222928..11343693	0,02	0,05	-0,836322869
TRPS1	8	complement(115408496..11580967	6,27	5,93	0,304468029
EIF3H	8	complement(116642130..11676692	61,87	50,70	0,510385652
UTP23	8	116766505..116849463	6,51	5,82	0,385450005
RAD21	8	complement(116845934..11687477	141,95	#####	0,032874017
AARD	8	116938207..116944487	0,07	0,02	2,233007235
SLC30A8	8	116950273..117176714	0,01	-	4,516772761
MED30	8	117520713..117540262	5,47	7,15	-0,162463735
EXT1	8	complement(117794490..11811182	59,20	38,18	0,855981317
SAMD12	8	complement(118189455..11862211	0,25	0,35	-0,266141172
TNFRSF11B	8	complement(118923557..11895188	72,06	10,50	3,001397933
COLEC10	8	118995452..119108455	5,99	0,31	4,492723746
MAL2	8	119165034..119245673	0,01	0,01	-0,025382928
CCN3	8	119416446..119424434	11,07	4,77	1,436225981
ENPP2	8	complement(119557086..11967345	2,74	7,47	-1,224920427
TAF2	8	complement(119730774..11983286	4,30	3,93	0,354848745
DSCC1	8	complement(119833976..11985589	2,22	2,54	0,027804663
gene:ENSG000	8	complement(119838736..11984038	0,22	0,47	-0,85687934
DEPTOR	8	119873717..120050918	3,28	0,81	2,229388287
COL14A1	8	120059780..120373573	0,02	0,01	0,441431721
MRPL13	8	complement(120380761..12044540	17,49	13,57	0,588431436
MTBP	8	120445400..120542133	3,19	3,89	-0,062455023
SNTB1	8	complement(120535745..12081327	1,71	1,22	0,703125942
HAS2	8	complement(121612116..12164144	3,07	6,81	-0,926399932
ZHX2	8	122781655..122974510	0,74	2,07	-1,26821507
DERL1	8	complement(123013170..12304230	24,59	20,92	0,456399068
TBC1D31	8	123041968..123152153	1,92	2,58	-0,202545082
FAM83A	8	123178960..123210079	0,01	0,10	-3,813033392
C8orf76	8	complement(123219967..12324137	6,94	7,31	0,147131925
ZHX1-C8orf76	8	complement(123226189..12327428	2,47	1,62	0,831585909
ZHX1	8	complement(123248451..12327554	3,35	2,77	0,49865163
ATAD2	8	complement(123319850..12341635	10,34	14,25	-0,239559876
NTAQ1	8	123416726..123470028	2,76	1,87	0,788971001

FBXO32	8	complement(123497889..12354120	5,01	12,76	-1,124834589
KLHL38	8	complement(123644442..12365380	0,48	0,53	0,082082968
ANXA13	8	complement(123680794..12373740	-	0,02	-4,359055975
FAM91A1	8	123768439..123815452	10,84	8,95	0,500006105
FER1L6	8	123851987..124120061	0,00	0,07	-3,93781363
TMEM65	8	complement(124306189..12437270	2,63	3,03	0,017578091
TRMT12	8	124450820..124462150	4,50	5,90	-0,16818635
RNF139	8	124474880..124488618	4,11	5,83	-0,281840362
TATDN1	8	complement(124488485..12453945	2,26	5,85	-1,148466024
NDUFB9	8	124539101..124580648	8,95	12,56	-0,265222621
MTSS1	8	complement(124550784..12472847	0,73	0,90	-0,071398309
ZNF572	8	124973295..124979389	-	-	0,188027316
SQLE	8	124998497..125022283	36,80	14,92	1,526121153
WASHC5	8	complement(125024260..12509181	6,97	10,07	-0,307483753
NSMCE2	8	125091679..125367120	5,25	4,48	0,451351051
TRIB1	8	125430358..125438403	5,23	9,06	-0,570401462
LRATD2	8	complement(126552443..12655847	3,99	2,50	0,897761982
POU5F1B	8	127322183..127420066	0,15	0,09	0,977254701
MYC	8	127735434..127742951	14,32	31,66	-0,921321821
GSDMC	8	complement(129748196..12978662	0,02	-	5,224730805
CYRIB	8	complement(129839593..13001750	3,77	7,27	-0,724846191
ASAP1	8	complement(130052104..13044367	23,15	23,97	0,172906222
ADCY8	8	complement(130780301..13104090	0,17	0,01	3,731534371
EFR3A	8	131904093..132013642	18,36	13,71	0,644254972
OC90	8	complement(132024216..13205938	-	-	#N/D
gene:ENSG000	8	complement(132024238..13208565	-	0,02	-4,359055975
HHLA1	8	complement(132061480..13211115	0,01	0,00	0,886720911
KCNQ3	8	complement(132120861..13248109	0,50	2,21	-1,919837674
DNAAF11	8	complement(132570416..13267559	0,14	0,07	1,290722889
TMEM71	8	complement(132685007..13276071	0,07	0,20	-1,266903375
PHF20L1	8	132775358..132848807	2,74	3,49	-0,128288418
TG	8	132866958..133134903	0,08	0,12	-0,42157054
SLA	8	complement(133036728..13310291	0,01	0,15	-4,490480318
CCN4	8	133191039..133231690	0,09	1,18	-3,535369658
NDRG1	8	complement(133237175..13330202	527,43	60,60	3,344620266
ST3GAL1	8	complement(133454848..13357192	63,33	21,16	1,804574082
ZFAT	8	complement(134477788..13471304	5,00	3,69	0,661459842
gene:ENSG000	8	134764809..134767276	0,00	0,02	-1,877914477
gene:ENSG000	8	complement(134783694..13478587	0,06	0,11	-0,581183184
KHDRBS3	8	135457456..135656722	3,57	0,55	2,918755553
FAM135B	8	complement(138130023..13849726	-	-	0,188027316
COL22A1	8	complement(138588235..13891404	0,42	0,38	0,374038156
KCNK9	8	complement(139600838..13970410	-	0,01	-4,359055975
TRAPPC9	8	complement(139727725..14045857	3,64	2,98	0,513520735
C8orf17	8	139931172..139933942	0,02	0,01	1,232079601
CHRAC1	8	140511311..140517154	7,93	8,17	0,179891049
AGO2	8	complement(140520156..14063563	16,30	15,20	0,32324664
gene:ENSG000	8	complement(140572142..14057281	1,19	0,85	0,707809632

gene:ENSG000	8	140591684..140593201	0,25	0,19	0,626665492
PTK2	8	complement(140657900..14100221	14,14	12,03	0,455684329
gene:ENSG000	8	complement(140820599..14082241	0,55	0,62	0,05554619
DENND3	8	141117278..141195808	6,92	5,40	0,579324056
SLC45A4	8	complement(141207166..14130830	1,24	2,01	-0,474297701
gene:ENSG000	8	complement(141224288..14122694	0,06	0,06	0,280049548
GPR20	8	complement(141356470..14136728	0,04	0,02	0,933337975
PTP4A3	8	141391995..141432454	3,41	5,32	-0,419518106
MROH5	8	complement(141433829..14150723	0,03	0,01	1,515286335
C8orf90	8	141514638..141518737	-	-	#N/D
TSNARE1	8	complement(142212080..14240318	1,89	2,48	-0,169409177
ADGRB1	8	142449430..142545009	6,12	2,40	1,575657919
ARC	8	complement(142611049..14261447	0,18	0,72	-1,801120213
JRK	8	complement(142657460..14268196	13,92	9,16	0,827627511
PSCA	8	142670308..142682725	0,89	1,36	-0,382614164
LY6K	8	142700111..142705127	12,84	17,66	-0,23758926
THEM6	8	142727223..142736927	19,18	15,43	0,536836036
SLURP1	8	complement(142740949..14274240	0,51	0,12	2,2519113
LYPD2	8	complement(142750150..14275253	0,11	0,02	2,399873901
LYNX1-SLURP2	8	complement(142764334..14277822	0,19	0,04	2,444941163
SLURP2	8	complement(142764338..14276982	-	-	0,188027316
LYNX1	8	complement(142771197..14277781	2,26	0,17	3,930649024
LY6D	8	complement(142784882..14278653	-	-	0,188027316
GML	8	142834247..142916506	-	-	#N/D
CYP11B1	8	complement(142872356..14287984	0,01	-	3,967268276
CYP11B2	8	complement(142910559..14291784	-	-	0,188027316
LY6E	8	143017982..143023832	64,10	48,64	0,621378008
LY6S	8	complement(143040839..14304831	0,02	0,03	-0,442604891
LY6L	8	143080630..143083486	-	-	#N/D
LY6H	8	complement(143157914..14316071	-	-	0,188027316
GPIHBP1	8	143213218..143217170	0,26	0,33	-0,091687096
ZFP41	8	143246821..143262705	3,59	4,66	-0,153543846
gene:ENSG000	8	143247110..143276403	0,87	0,65	0,655355882
GLI4	8	143267433..143276931	10,72	9,02	0,472494429
ZNF696	8	143289676..143299952	2,31	2,40	0,169963263
TOP1MT	8	complement(143304384..14335997	5,43	5,18	0,291437
RHPN1	8	143368876..143384221	0,91	0,45	1,248349675
MAFA	8	complement(143419182..14343073	0,10	0,59	-2,366967663
ZC3H3	8	complement(143437659..14354144	13,46	10,79	0,542048549
GSDMD	8	143553207..143563062	19,11	10,16	1,134725782
MROH6	8	complement(143566192..14357277	1,21	1,52	-0,097364777
NAPRT	8	complement(143574785..14357864	2,66	2,26	0,46073674
gene:ENSG000	8	143578358..143583304	7,62	4,01	1,146636971
EEF1D	8	complement(143579697..14359954	75,30	45,16	0,96057598
TIGD5	8	143597831..143603224	11,67	9,23	0,562465582
PYCR3	8	complement(143603210..14360977	9,77	6,47	0,816817099
GFUS	8	complement(143612618..14361804	32,88	18,03	1,089824665
ZNF623	8	143636019..143656418	7,33	7,10	0,268384246

ZNF707	8	143684452..143713898	5,03	3,05	0,94389011
CCDC166	8	complement(143706694..14370810	0,06	0,03	1,15046174
MAPK15	8	143716340..143722458	0,21	0,19	0,348362252
FAM83H	8	complement(143723933..14373823	5,28	6,49	-0,073639266
IQANK1	8	143734139..143790645	0,21	0,30	-0,311878307
SCRIB	8	complement(143790920..14381577	28,13	33,22	-0,016895337
PUF60	8	complement(143816344..14383070	45,73	41,49	0,363591751
NRBP2	8	complement(143833583..14384097	11,70	5,67	1,266447163
EPPK1	8	complement(143857324..14387846	1,14	0,04	4,901448392
PLEC	8	complement(143915153..14397673	130,67	79,06	0,948048087
PARP10	8	complement(143977153..14401277	4,09	3,19	0,582516918
GRINA	8	143990056..143993415	73,89	64,17	0,426439382
SPATC1	8	144012280..144047114	0,17	0,27	-0,401282968
OPLAH	8	complement(144051266..14406396	3,92	4,20	0,126622534
gene:ENSG000	8	144078642..144084330	1,32	0,96	0,672637532
EXOSC4	8	144078648..144080648	9,93	8,09	0,518260757
GPAA1	8	144082337..144093149	41,54	20,28	1,257527204
CYC1	8	144095039..144097525	111,25	98,96	0,391950385
SHARPIN	8	complement(144098633..14410812	13,93	10,33	0,654367589
MAF1	8	144104420..144107611	41,00	35,08	0,44809614
WDR97	8	144107726..144118328	0,47	0,29	0,908816498
HGH1	8	144137774..144140851	7,54	10,48	-0,253315738
MROH1	8	144148016..144261927	3,16	1,64	1,165312113
BOP1	8	complement(144262045..14429143	18,63	27,14	-0,31923445
SCX	8	144266453..144268481	0,37	0,22	0,954925991
HSF1	8	144291591..144314720	21,44	21,51	0,218411076
DGAT1	8	complement(144314584..14432691	6,07	5,08	0,478213698
SCRT1	8	complement(144330565..14433648	0,01	0,03	-0,865575989
SLC52A2	8	144333957..144361286	10,65	8,75	0,507501939
TMEM249	8	complement(144352219..14435495	0,06	0,11	-0,609885528
gene:ENSG000	8	complement(144353228..14435560	0,07	0,25	-1,521413109
FBXL6	8	complement(144355431..14435937	2,97	3,63	-0,064604826
ADCK5	8	144373101..144393242	0,92	0,89	0,265711686
CPSF1	8	complement(144393229..14440933	10,78	10,02	0,328826608
SLC39A4	8	complement(144409742..14441684	1,24	1,33	0,120486284
VPS28	8	complement(144423601..14442856	7,31	6,72	0,344080071
TONSL	8	complement(144428775..14444444	3,85	6,58	-0,549503108
ZFTRAF1	8	complement(144449582..14446287	2,53	2,56	0,208418304
TMEM276-ZFTR	8	complement(144449582..14446543	1,61	1,06	0,822039847
TMEM276	8	complement(144463817..14446566	2,72	2,75	0,203583112
KIFC2	8	144466043..144474202	4,74	4,16	0,408512117
FOXH1	8	complement(144473412..14447584	0,20	0,19	0,310048643
PPP1R16A	8	144477969..144502121	5,24	5,62	0,121173934
GPT	8	144502973..144507174	0,10	0,24	-0,995745964
MFSD3	8	144509070..144511213	6,34	6,57	0,171539102
RECQL4	8	complement(144511288..14451784	8,58	6,51	0,621559931
LRRC14	8	144517992..144525172	4,34	3,51	0,52985788
LRRC24	8	complement(144522388..14452703	0,02	0,17	-2,51724811

C8orf82	8	complement(144525733..14452913	2,72	2,86	0,152586399
ARHGAP39	8	complement(144529179..14468584	0,68	1,28	-0,693469658
ZNF251	8	complement(144720907..14475641	3,20	3,08	0,276066396
ZNF34	8	complement(144772224..14478734	0,34	0,54	-0,412772116
RPL8	8	complement(144789765..14479258	211,88	#####	-0,038877028
ZNF517	8	144798876..144811169	0,70	1,29	-0,654093811
ZNF7	8	144827518..144847509	2,74	2,46	0,379937316
COMMD5	8	complement(144841042..14485373	4,14	3,60	0,423517351
ZNF250	8	complement(144876497..14490216	0,90	0,81	0,370879258
ZNF16	8	complement(144930358..14495088	1,77	1,61	0,360594289
C8orf33	8	145052465..145066685	8,35	6,82	0,514739189
WASHC1	9	complement(14475..73865)	7,61	9,76	-0,135177452
FOXD4	9	complement(116231..118417)	0,03	0,23	-2,491858398
ZNG1A	9	complement(120837..179147)	0,65	0,83	-0,131564856
DOCK8	9	214854..465259	0,03	0,09	-1,463794966
KANK1	9	470291..746105	6,05	9,46	-0,420992751
DMRT1	9	841690..969090	0,06	0,20	-1,576120299
DMRT3	9	976655..991732	0,46	0,69	-0,367229229
DMRT2	9	1049858..1057552	0,01	0,01	0,524121557
SMARCA2	9	1980290..2193624	3,59	2,62	0,67865808
VLDLR	9	2621182..2660056	0,58	0,55	0,282944308
KCNV2	9	2717510..2730037	0,14	0,18	-0,138778669
PUM3	9	complement(2720469..2844095)	8,98	14,94	-0,512328246
RFX3	9	complement(3218297..3526004)	0,32	0,38	-0,027489946
GLIS3	9	complement(3824127..4348392)	4,56	5,51	-0,048875094
SLC1A1	9	4490468..4587469	2,75	3,18	0,015299392
SPATA6L	9	complement(4553386..4666674)	0,02	0,02	0,335747576
PLPP6	9	4662294..4665258	0,67	1,76	-1,167622375
CDC37L1	9	4679559..4708399	1,23	1,22	0,237473004
AK3	9	complement(4709556..4742043)	4,16	6,10	-0,329342399
RCL1	9	4792944..4885917	2,53	4,10	-0,472702886
JAK2	9	4984390..5129948	0,47	0,73	-0,406626925
INSL6	9	complement(5123880..5185647)	-	-	0,188027316
INSL4	9	5231419..5235304	0,01	-	3,068695355
RLN2	9	complement(5299864..5304716)	-	-	0,188027316
RLN1	9	complement(5334930..5339876)	-	-	0,188027316
PLGRKT	9	complement(5357971..5437925)	3,64	5,77	-0,441442902
CD274	9	5450503..5470566	0,13	0,19	-0,328873587
PDCD1LG2	9	5510531..5571282	0,11	0,56	-2,117732478
RIC1	9	5629025..5776557	3,11	3,05	0,251472153
ERMP1	9	complement(5749832..5879537)	1,71	3,79	-0,925981887
BRD10	9	complement(5881596..6008482)	1,16	2,18	-0,691498448
MLANA	9	5890889..5910606	0,03	0,09	-1,270895709
RANBP6	9	complement(6011025..6015625)	2,61	3,22	-0,077892073
IL33	9	6215786..6257983	0,00	0,01	-1,478387936
TPD52L3	9	6328375..6331891	-	-	0,188027316
UHRF2	9	6413151..6507054	6,95	7,70	0,07483016
GLDC	9	complement(6532467..6645729)	0,06	0,12	-0,685154657

KDM4C	9	6720863..7175648	1,35	1,48	0,085027812
DMAC1	9	complement(7796500..7888380)	5,99	7,03	-0,007719597
PTPRD	9	complement(8314246..10613002)	-	-	0,188027316
TYRP1	9	12685439..12710285	1,58	0,11	4,115406007
LURAP1L	9	12775020..12823060	8,40	8,53	0,202007017
MPDZ	9	complement(13105706..13279692)	19,22	4,82	2,217695232
NFIB	9	complement(14081843..14398983)	33,33	36,49	0,092317414
ZDHHC21	9	complement(14611071..14693432)	7,32	6,42	0,412089315
CER1	9	complement(14719724..14722733)	-	-	#N/D
FREM1	9	complement(14737152..14910995)	0,57	0,37	0,848527467
TTC39B	9	complement(15163622..15307360)	6,02	3,79	0,890418467
SNAPC3	9	15422734..15465953	49,81	32,81	0,825542182
PSIP1	9	complement(15464066..15510995)	30,07	46,48	-0,40522247
CCDC171	9	15553043..16061663	0,26	0,53	-0,783700775
BNC2	9	complement(16409503..16870843)	11,85	4,21	1,716003638
CNTLN	9	17134982..17503923	0,38	0,23	0,932336258
SH3GL2	9	17579066..17797124	-	-	0,188027316
ADAMTSL1	9	17906563..18910950	2,33	1,67	0,699026692
SAXO1	9	complement(18858906..19049358)	0,04	0,02	1,090636318
RRAGA	9	19049427..19051025	44,59	21,80	1,255705973
HAUS6	9	complement(19053141..19102904)	4,16	4,48	0,115004723
PLIN2	9	complement(19108375..19149290)	260,97	58,57	2,378667846
DENND4C	9	19230435..19374281	2,74	2,86	0,161257481
RPS6	9	complement(19375715..19380236)	179,25	#####	0,267372512
ACER2	9	19409009..19452505	0,24	0,20	0,462809804
SLC24A2	9	complement(19507452..19789037)	-	-	0,188027316
MLLT3	9	complement(20341669..20622499)	5,03	4,30	0,447341205
FOCAD	9	20658309..20995955	15,43	11,22	0,682700819
HACD4	9	complement(20999509..21031640)	1,76	2,01	0,024822938
IFNB1	9	complement(21077104..21077942)	-	0,03	-3,804623889
IFNW1	9	complement(21140632..21141832)	0,01	-	3,068695355
IFNA21	9	complement(21165637..21166660)	-	-	0,188027316
IFNA4	9	complement(21186618..21187587)	-	-	#N/D
IFNA7	9	complement(21201469..21202205)	-	-	#N/D
IFNA10	9	complement(21206181..21207143)	-	-	#N/D
IFNA16	9	complement(21216373..21217311)	0,02	-	3,967268276
IFNA17	9	complement(21227243..21228222)	-	-	#N/D
IFNA14	9	complement(21239002..21240005)	0,01	-	3,068695355
IFNA5	9	complement(21304326..21305313)	0,04	-	4,913749003
KLHL9	9	complement(21329665..21335404)	33,29	16,96	1,195678159
IFNA6	9	complement(21350253..21350956)	-	-	#N/D
IFNA13	9	complement(21367372..21368057)	-	0,06	-4,359055975
IFNA2	9	complement(21384255..21385398)	-	-	#N/D
IFNA8	9	21409117..21410185	0,01	0,07	-2,447197442
IFNA1	9	21440439..21441316	1,38	0,46	1,808072527
IFNE	9	complement(21480839..21482313)	2,77	1,33	1,286084006
MTAP	9	21802636..21937651	2,71	0,63	2,317519202
gene:ENSG000	9	21892188..21893857	-	-	#N/D

CDKN2A	9	complement(21967752..21995301)	-	-	#N/D
CDKN2B	9	complement(22002903..22009305)	-	-	#N/D
DMRTA1	9	22446824..22455740	-	-	#N/D
ELAVL2	9	complement(23690104..23826337)	0,00	0,01	-0,923955849
IZUMO3	9	complement(24542952..24545946)	-	-	#N/D
TUSC1	9	complement(25676969..25678444)	0,80	1,00	-0,105206212
gene:ENSG000	9	complement(26642697..26644595)	-	-	#N/D
CAAP1	9	complement(26840685..26892803)	0,63	0,64	0,205081722
PLAA	9	complement(26903372..26947242)	4,57	2,77	0,947404778
IFT74	9	26947039..27066134	0,26	0,26	0,25450695
LRRC19	9	complement(26993136..27005672)	0,01	-	4,913749004
TEK	9	27109141..27230174	0,00	0,01	-1,877914477
EQTN	9	complement(27284654..27297150)	-	-	#N/D
MOB3B	9	complement(27325209..27529814)	0,08	0,02	2,010690243
IFNK	9	27524290..27526498	0,01	-	3,068695355
C9orf72	9	complement(27535640..27573866)	0,58	0,50	0,441433416
LINGO2	9	complement(27937617..29213601)	-	0,00	-2,89252005
ACO1	9	32384603..32454769	7,54	11,78	-0,420051491
RIGI	9	complement(32455302..32526348)	2,99	2,99	0,218853232
gene:ENSG000	9	complement(32455323..32552603)	0,11	0,09	0,514359296
TOPORS	9	complement(32540544..32552586)	1,98	3,04	-0,392769443
SMIM27	9	32551144..32568621	0,62	0,61	0,242870808
NDUFB6	9	complement(32553001..32573184)	5,77	5,79	0,218261552
TAF1L	9	complement(32629454..32635669)	-	-	0,188027316
TMEM215	9	32783540..32789201	-	0,02	-5,734998842
APTX	9	complement(32886601..33025130)	1,62	2,02	-0,102515879
DNAJA1	9	33025273..33039907	17,46	18,37	0,15022809
SMU1	9	complement(33041765..33076674)	6,54	7,50	0,023920163
B4GALT1	9	complement(33104082..33167356)	34,97	33,70	0,276395687
SPINK4	9	33218365..33248567	-	-	0,188027316
BAG1	9	complement(33247820..33264720)	0,84	1,55	-0,658909788
CHMP5	9	33264879..33282070	11,16	10,92	0,25453736
NFX1	9	33290512..33371157	7,98	7,73	0,270239072
AQP7	9	complement(33383179..33402682)	0,01	0,01	0,533811434
AQP3	9	complement(33441156..33447596)	0,58	1,05	-0,624941617
NOL6	9	complement(33461353..33473930)	21,42	14,42	0,793494995
ANKRD18B	9	33524251..33608848	0,04	0,22	-2,101348846
TRBV20OR9-2	9	33617845..33618508	0,05	0,04	0,524121557
PRSS3	9	33750679..33799231	-	-	0,188027316
UBE2R2	9	33817160..33920399	23,01	15,14	0,82699274
UBAP2	9	complement(33921693..34049388)	3,73	3,73	0,221867911
DCAF12	9	complement(34086387..34127399)	8,16	9,21	0,04720462
UBAP1	9	34179005..34252523	9,68	12,55	-0,151873663
KIF24	9	complement(34252380..34329268)	1,27	1,41	0,073044232
NUDT2	9	34329506..34343711	4,78	5,01	0,156977426
MYORG	9	complement(34366666..34376898)	0,30	0,25	0,468792596
SPMIP6	9	complement(34379019..34397828)	0,06	0,04	0,750935198
FAM219A	9	complement(34398184..34458570)	13,21	12,26	0,330696128

DNAI1	9	34457414..34520988	0,02	0,06	-1,389560774
ENHO	9	complement(34521043..34522990)	0,12	0,35	-1,375237148
CNTFR	9	complement(34551432..34590140)	0,09	0,01	3,878355004
RPP25L	9	complement(34610486..34612104)	1,82	4,38	-1,044090701
DCTN3	9	complement(34613545..34620523)	12,38	9,42	0,616415664
ARID3C	9	complement(34621049..34629066)	0,05	0,05	0,112279066
SIGMAR1	9	complement(34634722..34637844)	16,81	25,17	-0,359110655
GALT	9	34638133..34651035	2,32	2,57	0,073524015
gene:ENSG000	9	34646631..34661886	1,17	0,54	1,334701539
IL11RA	9	34652162..34661902	1,46	0,53	1,685301922
CCL27	9	complement(34661880..34664048)	0,05	0,09	-0,579815015
gene:ENSG000	9	complement(34664163..34666112)	0,81	0,81	0,217613373
CCL19	9	complement(34689570..34691276)	0,06	0,02	1,833201638
CCL21	9	complement(34709005..34710136)	0,06	0,03	1,232079601
SPATA31F1	9	complement(34723053..34729488)	0,08	0,02	1,896631019
SPATA31F3	9	complement(34889066..34895764)	0,02	-	3,967268276
PHF24	9	34957608..34982544	0,10	0,05	1,070910392
DNAJB5	9	34989641..34998900	2,41	1,41	0,998240664
gene:ENSG000	9	35001913..35003663	0,14	0,16	0,027316923
gene:ENSG000	9	complement(35038625..35044326)	0,16	0,20	-0,12474297
SPATA31G1	9	35041095..35045986	0,16	0,35	-0,892398897
VCP	9	complement(35053928..35072668)	46,89	38,48	0,508104694
gene:ENSG000	9	complement(35065339..35074175)	-	0,02	-2,89252005
FANCG	9	complement(35073835..35080004)	7,83	9,00	0,0227276
PIGO	9	complement(35087237..35096619)	5,58	6,15	0,083459717
STOML2	9	complement(35099776..35103195)	29,74	42,02	-0,275222519
ATOSB	9	complement(35104112..35116341)	8,51	3,36	1,563519275
UNC13B	9	35161992..35405338	1,13	1,15	0,207718114
gene:ENSG000	9	complement(35360540..35362054)	0,01	0,02	-0,025382928
RUSC2	9	35490111..35561898	67,15	69,27	0,17826084
CIMIP2B	9	complement(35561831..35563899)	0,75	0,65	0,432490079
TESK1	9	35605262..35610041	36,50	31,87	0,418796761
CD72	9	complement(35609982..35646810)	2,25	0,68	1,957897087
SIT1	9	complement(35649295..35650931)	0,11	0,12	0,199980087
CCDC107	9	35658290..35661511	13,45	7,31	1,102229094
ARHGEF39	9	complement(35658875..35675866)	5,75	9,69	-0,53092077
CA9	9	35673928..35681159	58,65	8,08	3,082448546
TPM2	9	complement(35681992..35690056)	119,44	#####	0,411715108
TLN1	9	complement(35696948..35732195)	219,99	#####	0,594122023
CREB3	9	35732598..35736999	95,85	46,38	1,270380051
GBA2	9	complement(35736866..35749228)	26,83	27,25	0,200684628
RGP1	9	35749287..35758585	45,26	41,35	0,353388069
MSMP	9	complement(35752990..35756613)	-	-	0,188027316
NPR2	9	35791003..35809732	21,35	28,40	-0,18847027
SPAG8	9	complement(35808045..35812272)	1,40	0,85	0,938270311
HINT2	9	complement(35812960..35815354)	24,26	40,36	-0,511351261
gene:ENSG000	9	complement(35812960..35828732)	0,04	-	5,697545031
TMEM8B	9	35814451..35865518	7,90	6,93	0,411632515

FAM221B	9	complement(35816391..35828747)	0,04	0,16	-1,61577177
OR13J1	9	complement(35869463..35870401)	0,18	0,01	3,791717619
HRCT1	9	35906202..35907136	64,27	46,42	0,692532028
SPAAR	9	35909490..35937153	3,67	1,77	1,271561941
OR2S2	9	complement(35957108..35958154)	0,04	-	4,913749003
OR13C7	9	complement(36002909..36003867)	-	-	#N/D
RECK	9	36036913..36124455	9,78	13,42	-0,233603795
GLIPR2	9	36136536..36163913	3,58	12,80	-1,615638294
CCIN	9	36169388..36171334	0,03	0,14	-2,075817774
CLTA	9	36190856..36304781	68,64	89,63	-0,16187354
GNE	9	complement(36214441..36277042)	17,59	24,12	-0,232622532
RNF38	9	complement(36336396..36487548)	8,34	9,61	0,018949585
MELK	9	36572862..36677683	27,35	44,06	-0,464781421
PAX5	9	complement(36833269..37034268)	0,10	0,03	2,096059218
ZCCHC7	9	37120574..37358149	3,69	4,03	0,098308305
GRHPR	9	37422666..37436990	8,27	10,13	-0,068871957
ZBTB5	9	complement(37438102..37465450)	10,91	5,05	1,333623574
POLR1E	9	37485948..37503697	13,90	12,57	0,36848462
FBXO10	9	complement(37510892..37576380)	0,78	1,30	-0,503242401
gene:ENSG000	9	complement(37512547..37592469)	0,12	0,45	-1,664396418
TOMM5	9	complement(37582646..37592604)	4,04	4,32	0,125316469
gene:ENSG000	9	complement(37588413..38068687)	0,20	0,16	0,498550921
FRMPD1	9	37650954..37746904	0,00	0,01	-1,54862452
TRMT10B	9	37753803..37778972	1,13	0,60	1,136637005
EXOSC3	9	complement(37759234..37832117)	0,68	0,98	-0,305727193
DCAF10	9	37800554..37867666	3,62	4,51	-0,093584672
SLC25A51	9	complement(37879400..37904353)	4,21	4,38	0,165739528
SHB	9	complement(37915898..38069227)	7,50	6,58	0,412325592
ALDH1B1	9	38392702..38398661	8,91	15,52	-0,577208591
IGFBPL1	9	complement(38406528..38424454)	0,32	0,12	1,638931178
ANKRD18A	9	complement(38540567..38620596)	0,15	0,39	-1,104940165
FAM240B	9	complement(38694266..38720133)	-	-	#N/D
CNTNAP3	9	complement(39064710..39288315)	1,77	1,14	0,867396532
SPATA31A1	9	39355669..39361962	-	-	#N/D
gene:ENSG000	9	41012231..41014133	0,42	0,73	-0,552318638
gene:ENSG000	9	41061290..41062917	0,32	0,39	-0,069857544
FOXD4L6	9	complement(41126435..41128681)	0,02	0,03	-0,602143793
ZNG1F	9	complement(41131306..41199261)	0,42	0,83	-0,758232969
gene:ENSG000	9	complement(41269916..41273997)	0,32	0,53	-0,501757106
CNTNAP3B	9	complement(41890314..42129510)	2,17	2,14	0,241608598
SPATA31A6	9	42183659..42189887	-	-	0,188027316
SPATA31A5	9	60914407..60920653	-	-	0,188027316
SPATA31A7	9	61190036..61196280	-	-	#N/D
CNTNAP3C	9	61330717..61453030	5,71	2,40	1,474460315
gene:ENSG000	9	61982796..61986934	0,20	0,12	1,014173129
FOXD4L5	9	complement(65282101..65285209)	0,05	0,06	0,059935958
ZNG1E	9	65668805..65734041	1,05	1,31	-0,097607554
FOXD4L4	9	65737146..65738396	-	0,08	-5,734998842

ZNF658	9	66856426..66932141	0,23	0,49	-0,841236666
SPATA31A3	9	complement(66986304..66992550)	-	-	#N/D
ANKRD20A1	9	67832765..67920552	0,05	0,05	0,41017726
ZNG1C	9	68232003..68300035	0,64	0,62	0,263413787
gene:ENSG000	9	complement(68250469..68251656)	0,54	0,56	0,193567768
FOXD4L3	9	68302867..68305084	-	0,04	-5,327865481
PGM5	9	68328308..68531061	0,25	0,64	-1,128707697
TMEM252	9	complement(68536580..68540883)	-	-	#N/D
PIP5K1B	9	68705240..69009176	-	-	0,188027316
PABIR1	9	68780065..68785566	0,26	0,68	-1,15864305
PRKACG	9	complement(69012504..69014113)	-	-	#N/D
gene:ENSG000	9	69035747..69255187	0,38	0,34	0,358431027
FXN	9	69035751..69079076	0,39	0,78	-0,77742972
TJP2	9	69121264..69274615	1,87	3,74	-0,779559625
ENTREP1	9	69324567..69392558	0,08	-	7,511061969
APBA1	9	complement(69427532..69672371)	0,15	0,02	2,945448271
PTAR1	9	complement(69709522..69760011)	2,81	2,36	0,47407714
CFAP95	9	69820817..69906227	-	0,01	-2,89252005
MAMDC2	9	70043848..70226972	0,07	0,03	1,404839344
SMC5	9	70258978..70354873	1,44	2,02	-0,257239899
KLF9	9	complement(70384604..70414657)	4,91	5,29	0,114109067
TRPM3	9	complement(70529060..71446977)	0,00	0,00	2,144183439
CEMIP2	9	complement(71683366..71816690)	1,62	1,86	0,019375398
ABHD17B	9	complement(71862452..71911193)	1,58	1,53	0,271875754
C9orf85	9	71911510..71986054	0,63	0,62	0,260158562
C9orf57	9	complement(72051376..72072721)	-	-	0,188027316
GDA	9	72114595..72257193	-	0,01	-4,359055975
ZFAND5	9	complement(72351413..72365235)	4,78	7,12	-0,353634912
TMC1	9	72521608..72838297	0,01	0,00	2,144183439
ALDH1A1	9	complement(72900671..73080442)	0,00	0,05	-3,171495545
ANXA1	9	73151865..73170393	55,51	71,11	-0,134148311
RORB	9	74497335..74693177	0,04	0,01	2,918623677
TRPM6	9	complement(74722495..74888094)	0,01	0,00	2,144183439
C9orf40	9	complement(74946583..74952912)	1,86	2,38	-0,133880432
CARNMT1	9	complement(74980790..75028423)	1,58	1,59	0,209971835
NMRK1	9	complement(75060573..75088217)	1,50	1,77	-0,018321083
OSTF1	9	75088514..75147265	6,30	4,12	0,834340256
PCSK5	9	75890644..76362975	0,14	0,56	-1,78455157
RFK	9	complement(76385526..76394517)	1,16	2,63	-0,95624349
GCNT1	9	76419850..76651203	0,40	1,46	-1,635430646
PRUNE2	9	complement(76611376..76906114)	0,07	0,01	3,063523174
FOXB2	9	77019655..77020953	-	-	0,188027316
VPS13A	9	77177445..77421537	0,86	1,45	-0,527504269
GNA14	9	complement(77423079..77648322)	0,01	0,02	-0,429837071
GNAQ	9	complement(77716097..78031811)	3,30	4,02	-0,058313058
CEP78	9	78236062..78279690	0,70	1,30	-0,668780442
PSAT1	9	78297125..78330093	12,93	75,72	-2,32695552
TLE4	9	79571773..79726882	1,71	1,96	0,028718699

gene:ENSG000	9	79913269..79915736	-	-	#N/D
TLE1	9	complement(81583683..81689547)	1,17	2,79	-1,035565917
SPATA31D4	9	81928428..81934998	-	-	0,188027316
SPATA31D3	9	81943500..81950093	-	-	0,188027316
SPATA31D1	9	81988772..81995253	-	-	0,188027316
gene:ENSG000	9	82543718..82544196	-	-	0,188027316
RASEF	9	complement(82979590..83063177)	0,03	0,12	-1,792324626
FRMD3	9	complement(83242990..83538546)	0,23	0,94	-1,816355547
IDNK	9	83623049..83644130	0,21	0,37	-0,609292623
UBQLN1	9	complement(83659968..83707958)	20,94	32,05	-0,390756365
GKAP1	9	complement(83739425..83829516)	0,37	0,84	-0,944682804
KIF27	9	complement(83834099..83921465)	0,18	0,48	-1,2173018
QNG1	9	complement(83938311..83956986)	1,20	2,40	-0,772782592
HNRNPK	9	complement(83968083..83980631)	45,83	72,67	-0,442084707
RMI1	9	83980798..84004074	1,42	2,31	-0,480422549
SLC28A3	9	complement(84275457..84340758)	0,00	0,04	-3,651459213
NTRK2	9	84668375..85095751	0,00	0,00	1,436225396
AGTPBP1	9	complement(85546539..85742029)	1,75	4,83	-1,240282502
NAA35	9	85941146..86025462	3,09	3,83	-0,088747924
GOLM1	9	complement(86026146..86100173)	6,45	16,93	-1,169757685
C9orf153	9	complement(86220265..86259657)	0,13	0,13	0,188596267
ISCA1	9	complement(86264546..86283102)	3,64	5,90	-0,475130584
TUT7	9	complement(86287733..86354454)	3,23	4,41	-0,223057072
GAS1	9	complement(86944362..86947506)	0,17	0,35	-0,758638109
DAPK1	9	87497228..87708634	0,75	1,69	-0,945294429
CTSL	9	87724051..87731469	7,91	17,09	-0,888334737
SPATA31E1	9	87882877..87888903	-	-	0,188027316
SPATA31C1	9	87914488..87923657	0,01	-	3,967268276
gene:ENSG000	9	87956214..87956883	-	-	#N/D
CDK20	9	complement(87966441..87974753)	2,70	2,58	0,293611449
SPATA31C2	9	complement(88129305..88138475)	-	-	0,188027316
gene:ENSG000	9	88177695..88178578	-	-	0,188027316
SPIN1	9	88388430..88478694	13,07	14,34	0,08892854
NXNL2	9	88534033..88584274	0,27	0,15	1,09830913
S1PR3	9	88990863..89005155	7,87	6,23	0,559973672
SHC3	9	complement(89005771..89178818)	0,04	0,23	-2,181997193
CKS2	9	89311195..89316703	34,69	44,96	-0,15083853
SECISBP2	9	89318500..89359663	2,05	3,83	-0,677639385
SEMA4D	9	complement(89360787..89498130)	0,87	2,15	-1,078674197
GADD45G	9	89605012..89606555	0,10	-	6,461896167
gene:ENSG000	9	90158028..90158646	-	-	#N/D
DIRAS2	9	complement(90609832..90642862)	0,00	0,01	-0,923955849
SYK	9	90801787..90898549	0,28	-	9,936661209
AUH	9	complement(91213815..91361918)	2,30	3,25	-0,27708802
NFIL3	9	complement(91409045..91423832)	14,53	11,82	0,521321932
ROR2	9	complement(91563091..91950228)	0,23	0,00	6,507677441
SPTLC1	9	complement(92000087..92115413)	3,92	4,31	0,08491496
IARS1	9	complement(92210207..92293854)	35,64	65,00	-0,644061726

NOL8	9	complement(92297358..92325636)	1,55	2,46	-0,439767721
CENPP	9	92325953..92620529	1,41	1,39	0,247172482
OGN	9	complement(92383268..92404696)	0,04	0,03	0,857064368
OMD	9	complement(92412380..92424471)	0,01	0,04	-1,275310173
ASPN	9	complement(92456205..92482506)	0,00	0,02	-1,877914477
ECM2	9	complement(92493554..92536655)	0,06	0,26	-1,879530179
IPPK	9	complement(92613183..92670131)	5,08	4,21	0,49256053
BICD2	9	complement(92711363..92764833)	10,70	10,85	0,202852481
ZNF484	9	complement(92844182..92878038)	1,65	1,95	-0,021345675
FGD3	9	92947523..93036236	0,03	0,02	0,842164604
SUSD3	9	93058688..93085133	-	0,01	-2,89252005
CARD19	9	93096217..93113283	22,40	13,56	0,947319232
NINJ1	9	complement(93121496..93134251)	44,31	38,96	0,408711855
WNK2	9	93184122..93320572	0,71	1,27	-0,609811448
FAM120AOS	9	complement(93431441..93453581)	1,24	2,08	-0,521917878
FAM120A	9	93451685..93566112	34,69	40,66	-0,00599253
PHF2	9	93576584..93679587	9,35	12,34	-0,177029268
BARX1	9	complement(93951627..93955355)	17,76	24,04	-0,214223726
PTPDC1	9	94030794..94109856	1,64	3,24	-0,756606348
ZNF169	9	94259298..94301829	1,18	1,15	0,263810895
NUTM2F	9	complement(94318198..94328644)	0,07	0,03	1,443009573
MFSD14B	9	94374569..94461042	18,84	23,34	-0,086277916
FBP2	9	complement(94558720..94593824)	0,01	0,03	-1,478387936
FBP1	9	complement(94603133..94640249)	0,01	0,01	-0,025382928
AOPEP	9	94726604..95151793	3,65	5,72	-0,424149632
FANCC	9	complement(95099054..95426796)	1,86	2,22	-0,031482069
PTCH1	9	complement(95442980..95517057)	0,37	0,39	0,152607907
ERCC6L2	9	95871264..96121154	0,78	1,18	-0,371572321
HSD17B3	9	complement(96235306..96302176)	0,03	0,02	0,933337975
gene:ENSG000	9	complement(96235331..96352058)	-	-	0,188027316
SLC35D2	9	complement(96313444..96383711)	1,54	2,35	-0,380451825
ZNF367	9	complement(96385941..96418370)	2,85	2,64	0,3342011
HABP4	9	96450169..96491336	3,60	3,21	0,388862879
CDC14B	9	complement(96490241..96619843)	2,13	3,40	-0,450445934
PRXL2C	9	complement(96639577..96655317)	3,51	4,89	-0,257737184
ZNF510	9	complement(96754553..96778129)	1,00	1,89	-0,700730592
ZNF782	9	complement(96816269..96875623)	0,30	0,61	-0,817635324
NUTM2G	9	96928310..96940253	0,70	0,59	0,471874257
CTSV	9	complement(97029677..97156556)	0,42	0,10	2,220284521
CCDC180	9	97307304..97378751	0,69	0,81	-0,008614843
TDRD7	9	97412096..97496125	3,41	4,44	-0,157174442
TMOD1	9	97501180..97601743	0,49	1,56	-1,442068371
TSTD2	9	complement(97600080..97633368)	2,11	4,05	-0,715627133
NCBP1	9	97633668..97673748	8,97	13,02	-0,313941185
XPA	9	complement(97674909..97697340)	0,65	0,90	-0,243824186
FOXE1	9	97853226..97856717	0,01	0,01	-0,979341556
TRMO	9	complement(97904489..97922500)	0,44	0,94	-0,867788586
HEMGN	9	complement(97926791..97944856)	-	-	#N/D

ANP32B	9	97983341..98015943	33,26	62,54	-0,687853998
NANS	9	98056732..98083077	16,95	16,56	0,25694108
TRIM14	9	complement(98069275..98119222)	7,32	11,52	-0,430051445
CORO2A	9	complement(98120975..98192637)	0,33	0,33	0,240355318
TBC1D2	9	complement(98199011..98255649)	2,40	2,58	0,113343021
GABBR2	9	complement(98288109..98708935)	0,02	0,01	1,060045679
ANKS6	9	complement(98731329..98796965)	4,68	8,72	-0,675134027
GALNT12	9	98807670..98850081	0,65	2,72	-1,838549229
COL15A1	9	98943179..99070787	0,15	0,86	-2,278969788
TGFBR1	9	99104038..99154192	3,57	7,54	-0,856294354
ALG2	9	complement(99216425..99221942)	12,58	7,39	0,990638443
SEC61B	9	99222064..99230615	50,54	38,63	0,610842138
NR4A3	9	99821855..99866891	0,08	0,10	-0,101388123
STX17	9	99906654..99974534	1,86	3,52	-0,695261803
ERP44	9	complement(99979185..100099052	4,31	4,73	0,090130846
INVS	9	100099243..100302175	3,15	5,26	-0,518053383
TEX10	9	complement(100302077..10035294	6,48	8,35	-0,142519533
MSANTD3	9	100427143..100451734	16,15	18,17	0,053404109
MSANTD3-TME	9	100442271..100577636	0,66	1,41	-0,882839722
TMEFF1	9	100473149..100577636	0,20	0,46	-0,99139794
CAVIN4	9	100578079..100588389	0,18	0,39	-0,892735797
PLPPR1	9	101028727..101325135	-	0,01	-3,804623889
BAAT	9	complement(101354182..10138540	0,04	0,07	-0,451418876
MRPL50	9	complement(101387633..10139861	1,80	2,62	-0,320108067
ZNF189	9	101398851..101410660	1,60	2,67	-0,51644536
ALDOB	9	complement(101420560..10144966	0,02	0,01	0,677647514
PGAP4	9	complement(101473170..10153353	6,78	10,97	-0,471354285
RNF20	9	101533853..101563344	14,33	12,58	0,41160939
GRIN3A	9	complement(101569352..10173864	0,37	0,24	0,880879044
PPP3R2	9	complement(101591604..10159502	0,01	0,01	-0,025382928
CYLC2	9	102995311..103018488	-	-	#N/D
SMC2	9	104094260..104141419	4,78	7,78	-0,479704945
OR13F1	9	104504263..104505222	-	-	#N/D
OR13C4	9	complement(104526253..10452720	-	-	#N/D
OR13C3	9	complement(104535749..10453685	-	-	0,188027316
OR13C8	9	104569168..104570130	-	-	#N/D
OR13C5	9	complement(104598457..10459941	-	-	#N/D
OR13C2	9	complement(104604671..10460562	-	-	#N/D
OR13C9	9	complement(104617248..10461820	-	-	#N/D
OR13D1	9	104694518..104695462	-	-	0,188027316
NIPSNAP3A	9	104747683..104760120	5,53	5,61	0,201999604
NIPSNAP3B	9	104764129..104777764	0,10	0,13	-0,146895384
ABCA1	9	complement(104781006..10492815	1,36	6,97	-2,133166603
SLC44A1	9	105244622..105439171	3,76	5,93	-0,434165233
FSD1L	9	105447796..105552433	0,21	0,24	0,013119411
FKTN	9	105558122..105653820	0,99	1,46	-0,344644713
gene:ENSG000	9	105656369..105657989	0,01	-	3,068695355
TAL2	9	105662457..105663124	-	0,04	-3,804623889

TMEM38B	9	105694541..105776629	1,01	3,18	-1,424756343
ZNF462	9	106863166..107013634	2,96	6,51	-0,916548228
RAD23B	9	107283137..107332192	20,36	32,22	-0,439251792
KLF4	9	complement(107484852..10749048	1,52	2,48	-0,488780736
ACTL7B	9	complement(108854588..10885598	0,04	-	5,224730805
ACTL7A	9	108862266..108863756	0,01	0,02	-0,025382928
ELP1	9	complement(108866898..10893432	9,04	9,00	0,229324885
ABITRAM	9	108934400..108950744	2,49	3,47	-0,260223257
CTNNAL1	9	complement(108942569..10901352	18,71	20,03	0,124782729
TMEM245	9	complement(109015135..10911994	14,02	11,11	0,55913692
FRRS1L	9	complement(109130293..10916724	0,00	0,00	0,886720911
EPB41L4B	9	complement(109171974..10932105	0,51	1,37	-1,211288669
PTPN3	9	complement(109375466..10949831	2,01	3,32	-0,496135381
PALM2AKAP2	9	109498325..110172512	6,60	12,25	-0,669788295
C9orf152	9	complement(110190048..11020815	0,06	0,05	0,539899626
TXN	9	complement(110243810..11025650	175,91	#####	0,171505738
TXNDC8	9	complement(110303476..11033789	-	-	0,188027316
SVEP1	9	complement(110365248..11057988	7,24	11,80	-0,481561753
MUSK	9	110668779..110806558	0,74	0,40	1,097273818
LPAR1	9	complement(110873263..11103847	41,62	31,62	0,619564427
OR2K2	9	complement(111327229..11133022	-	0,02	-3,804623889
ECPAS	9	complement(111360685..11148474	21,70	29,99	-0,243715743
ZNF483	9	111525159..111577844	0,05	0,07	-0,092855397
PTGR1	9	complement(111549722..11159989	4,80	6,73	-0,265882807
DNAJC25	9	111631334..111654351	3,11	1,97	0,880321082
DNAJC25-GNG	9	111631386..111670229	2,19	2,16	0,239897934
GNG10	9	111661605..111670226	7,18	9,79	-0,223504934
SHOC1	9	complement(111686171..11179500	0,02	0,01	0,686693064
UGCG	9	111896814..111935369	4,56	12,45	-1,226636405
SUSD1	9	complement(112040783..11217529	0,51	1,17	-0,969467581
PTBP3	9	complement(112217716..11233366	7,96	10,79	-0,215696039
HSDL2	9	112380080..112472405	4,93	8,20	-0,510254081
KIAA1958	9	112486827..112669397	0,95	1,59	-0,518918312
INIP	9	complement(112683926..11271814	5,24	5,12	0,257955945
SNX30	9	112750760..112881671	3,59	4,56	-0,12086997
SLC46A2	9	complement(112878920..11289087	0,09	0,13	-0,230810911
ZNF883	9	complement(112988129..11301219	0,06	0,01	2,178324378
ZFP37	9	complement(113038377..11305675	0,26	0,28	0,078690271
SLC31A2	9	113150976..113164140	0,32	0,20	0,877062004
FKBP15	9	complement(113161006..11322145	3,82	3,86	0,209338052
SLC31A1	9	113221544..113264492	22,43	15,25	0,779698549
CDC26	9	complement(113255835..11327557	9,31	10,54	0,044092704
PRPF4	9	113275642..113294009	12,80	14,98	-0,003211434
RNF183	9	complement(113297093..11330337	0,00	0,00	-0,01185201
WDR31	9	complement(113313222..11334029	0,44	0,79	-0,627768142
BSPRY	9	113349541..113371233	-	0,01	-3,804623889
HDHD3	9	complement(113373419..11337698	0,35	2,37	-2,531796136
ALAD	9	complement(113386312..11340129	4,84	4,46	0,340715994

POLE3	9	complement(113407235..11341067	40,23	27,66	0,763289581
C9orf43	9	113410054..113429690	0,14	0,19	-0,185877329
RGS3	9	113444731..113597743	5,96	4,77	0,543714662
gene:ENSG000	9	complement(113614589..11361737	0,03	0,02	0,627075281
ZNF618	9	113876282..114056593	3,81	5,49	-0,303105902
AMBP	9	complement(114060127..11407832	0,01	0,06	-2,665078517
KIF12	9	complement(114086126..11409929	0,01	0,01	0,524121557
COL27A1	9	114155537..114312511	4,27	4,51	0,146172058
ORM1	9	114323098..114326479	-	-	0,188027316
ORM2	9	114329869..114333251	-	0,03	-3,804623889
AKNA	9	complement(114334156..11439440	0,78	2,58	-1,507851184
WHRN	9	complement(114402080..11450547	6,81	4,70	0,758851047
ATP6V1G1	9	114587706..114598915	5,07	6,99	-0,24047191
TMEM268	9	114611206..114646422	5,06	8,36	-0,501311646
TEX53	9	complement(114656304..11466237	0,09	-	4,913749004
TEX48	9	complement(114666434..11468219	0,01	0,05	-1,478387936
TNFSF15	9	complement(114784652..11480603	0,05	0,12	-1,162245248
TNFSF8	9	complement(114893343..11493059	0,02	0,03	-0,187130731
TNC	9	complement(115019575..11511820	36,75	27,14	0,660227471
PAPPA	9	116153791..116402321	0,03	1,37	-5,210157339
ASTN2	9	complement(116423112..11741507	0,46	0,83	-0,622396411
TRIM32	9	116687305..116701300	7,36	11,07	-0,366696501
gene:ENSG000	9	117704157..118083691	0,02	0,03	-0,411705719
TLR4	9	117704175..117724735	0,44	0,76	-0,558773912
BRINP1	9	complement(119153458..11936943	-	-	0,188027316
CDK5RAP2	9	complement(120388875..12058017	5,77	8,84	-0,393527905
MEGF9	9	complement(120600811..12071447	0,90	1,14	-0,110150842
FBXW2	9	complement(120751978..12079341	6,78	7,80	0,02157679
B3GALT9	9	120792403..120801787	0,24	1,09	-1,931691461
PSMD5	9	complement(120815496..12084295	3,47	4,48	-0,143520374
PHF19	9	complement(120855651..12089489	11,18	17,49	-0,422119814
TRAF1	9	complement(120902393..12092917	0,20	1,74	-2,882204567
C5	9	complement(120932987..12107519	0,15	0,27	-0,568613722
CNTRL	9	121074660..121177729	0,75	1,69	-0,948088917
RAB14	9	complement(121178133..12122301	13,20	12,93	0,252700665
GSN	9	121207794..121332843	26,57	8,66	1,840260838
STOM	9	complement(121338987..12137030	14,56	24,97	-0,554533261
GGTA1	9	complement(121444991..12149984	0,02	0,00	2,805678585
DAB2IP	9	121567057..121785530	5,73	4,55	0,557061751
TTLL11	9	complement(121815674..12209360	2,68	4,06	-0,37740248
NDUFA8	9	complement(122144058..12215977	25,83	28,73	0,069618411
MORN5	9	122159908..122200088	0,01	-	3,068695355
LHX6	9	complement(122202577..12222962	0,21	0,18	0,446962266
RBM18	9	complement(122237622..12226484	2,89	3,67	-0,119705757
MRRF	9	122264603..122331337	2,42	3,04	-0,10343275
PTGS1	9	122370530..122395703	0,01	0,02	-0,036228841
OR1J1	9	complement(122476958..12247792	-	0,03	-3,804623889
OR1J2	9	122510802..122511743	-	-	#N/D

OR1J4	9	122519141..122520082	-	-	#N/D
OR1N1	9	complement(122526358..12252729	-	-	#N/D
OR1N2	9	122553112..122554214	-	-	#N/D
OR1L8	9	complement(122567117..12258338	0,01	0,02	-0,579815015
OR1Q1	9	122614738..122615682	-	-	0,188027316
OR1B1	9	complement(122628125..12265762	0,01	-	3,068695355
OR1L1	9	122661566..122662648	-	-	#N/D
OR1L3	9	122675130..122676104	-	-	#N/D
OR1L4	9	122723990..122724925	0,02	-	3,967268276
OR1L6	9	122742303..122750783	-	-	0,188027316
OR5C1	9	122788933..122789895	0,03	0,03	0,524121557
PDCL	9	complement(122798389..12282858	5,59	4,32	0,595582371
OR1K1	9	122800123..122801073	0,06	0,03	1,232079601
RC3H2	9	complement(122844556..12290535	6,10	6,71	0,085746744
ZBTB6	9	complement(122908056..12291332	1,14	2,08	-0,641047221
ZBTB26	9	complement(122915566..12293151	1,63	1,66	0,192856067
RABGAP1	9	122940833..123104866	7,84	9,86	-0,108559539
GPR21	9	123033667..123035665	0,37	0,26	0,757774125
STRBP	9	complement(123109500..12326858	0,58	0,64	0,082536894
CRB2	9	123356170..123380324	0,06	0,10	-0,665849199
DENND1A	9	complement(123379654..12393015	4,52	6,63	-0,327964904
LHX2	9	124001670..124033301	1,41	1,39	0,2407757
NEK6	9	124257606..124353307	19,24	15,28	0,555148182
PSMB7	9	complement(124353465..12441544	87,37	#####	-0,321191658
ADGRD2	9	124450451..124478589	-	-	#N/D
NR5A1	9	complement(124481236..12450742	0,00	0,00	-0,01185201
NR6A1	9	complement(124517275..12477131	0,46	1,65	-1,617409804
OLFML2A	9	124777133..124814885	2,60	3,38	-0,156398693
WDR38	9	124853417..124857890	0,23	0,31	-0,225350138
RPL35	9	complement(124857880..12486198	293,46	#####	-0,030995769
ARPC5L	9	124862130..124877733	5,84	7,90	-0,211947464
GOLGA1	9	complement(124878275..12494849	2,99	4,26	-0,290743537
SCAI	9	complement(124942608..12514352	0,90	0,94	0,168960109
PPP6C	9	complement(125146573..12518993	3,92	5,87	-0,359907068
RABEPK	9	125200542..125234161	4,62	7,36	-0,449467568
HSPA5	9	complement(125234853..12524138	501,20	#####	2,014917647
GAPVD1	9	125261794..125367207	8,87	8,82	0,231464709
MAPKAP1	9	complement(125437393..12570723	16,71	20,51	-0,072808266
PBX3	9	125747345..125967377	1,88	3,61	-0,720326074
MVB12B	9	126326829..126507041	0,83	2,27	-1,234949449
gene:ENSG000	9	126518840..126520773	-	-	0,188027316
LMX1B	9	126613928..126701032	2,18	2,59	-0,029542848
ZBTB43	9	126805006..126838210	4,81	4,44	0,336703394
ZBTB34	9	126860639..126885878	2,46	2,12	0,437943258
RALGPS1	9	126914774..127223166	0,54	0,70	-0,155231601
ANGPTL2	9	complement(127087348..12712263	7,97	20,58	-1,145495429
GARNL3	9	127224265..127393660	0,81	1,11	-0,243456517
SLC2A8	9	127397138..127408424	2,50	2,75	0,084920286

ZNF79	9	127424374..127445372	1,32	1,33	0,209912867
RPL12	9	complement(127447674..12745140	351,74	#####	-0,442091152
LRSAM1	9	127451489..127503499	6,77	6,17	0,355271717
NIBAN2	9	complement(127505339..12757898	124,19	97,31	0,574957055
STXBP1	9	127579370..127696027	6,39	7,75	-0,055018049
PTRH1	9	complement(127690348..12772487	1,57	1,78	0,041014393
CFAP157	9	127706988..127716002	0,89	0,69	0,604446282
TTC16	9	127716079..127731590	0,02	0,04	-0,698295353
TOR2A	9	complement(127731524..12773531	2,42	4,10	-0,536400385
SH2D3C	9	complement(127738317..12777871	0,13	0,11	0,476636705
CDK9	9	127785679..127790792	17,27	16,47	0,291712182
FPGS	9	127794597..127814327	16,27	20,63	-0,119978621
ENG	9	complement(127811130..12785477	23,19	24,02	0,17242468
AK1	9	complement(127866486..12787767	6,13	6,40	0,162564217
gene:ENSG000	9	complement(127867850..12790602	3,87	5,22	-0,208660515
ST6GALNAC6	9	complement(127885321..12790540	12,64	8,26	0,838015583
ST6GALNAC4	9	complement(127907886..12791704	20,70	25,15	-0,057950797
PIP5KL1	9	complement(127920881..12793078	1,67	1,64	0,25436233
DPM2	9	complement(127935099..12793785	22,49	22,29	0,235843167
EEIG1	9	complement(127940582..12798098	7,45	10,51	-0,272173735
NAIF1	9	complement(128061233..12806820	2,21	2,77	-0,103691389
SLC25A25	9	128068201..128109245	3,31	3,08	0,329424882
PTGES2	9	complement(128120693..12812846	13,33	22,29	-0,519009405
LCN2	9	128149071..128153453	0,03	9,45	-7,950488657
BBLN	9	128160265..128163924	10,97	21,04	-0,716791027
CIZ1	9	complement(128161251..12820438	15,88	22,87	-0,303157483
DNM1	9	128191655..128255248	0,52	0,28	1,131870305
GOLGA2	9	complement(128255829..12827602	43,09	25,04	1,006094872
SWI5	9	128275379..128316123	8,49	5,06	0,970321191
TRUB2	9	complement(128305159..12832244	5,13	6,81	-0,18748773
COQ4	9	128322544..128334072	6,54	6,56	0,218341691
SLC27A4	9	128340527..128361470	3,86	7,17	-0,670806982
URM1	9	128371361..128392016	14,60	13,41	0,345573005
CERCAM	9	128411751..128437351	10,71	11,24	0,152713568
ODF2	9	128455186..128501292	15,14	13,95	0,34137934
GLE1	9	128504655..128543874	8,18	8,54	0,162271557
SPTAN1	9	128552558..128633662	14,70	26,76	-0,641463115
DYNC2I2	9	complement(128633653..12865684	20,14	26,39	-0,166964133
SET	9	128683424..128696400	50,93	79,32	-0,41594175
PKN3	9	128702503..128720916	2,06	5,43	-1,172300828
ZDHHC12	9	complement(128720870..12872412	20,26	15,78	0,583141234
ZER1	9	complement(128729786..12877241	9,81	11,42	0,002984928
TBC1D13	9	128787253..128810430	6,36	7,74	-0,060393158
ENDOG	9	128818500..128822676	3,31	5,39	-0,479058409
SPOUT1	9	complement(128819651..12882979	4,46	5,78	-0,148823122
KYAT1_1	9	complement(128819663..12888193	0,50	0,39	0,595825986
KYAT1_2	9	complement(128832942..12888249	7,87	6,21	0,564476253
LRRC8A	9	128882133..128918039	30,16	18,36	0,939120887

PHYHD1	9	128920966..128942041	0,08	0,09	0,1041167
gene:ENSG000	9	128941478..128957021	-	0,03	-2,89252005
DOLK	9	complement(128945530..12894760	3,58	5,61	-0,425075774
NUP188	9	128947699..129007096	13,95	27,06	-0,7327928
SH3GLB2	9	complement(129007036..12902833	12,16	17,53	-0,305243539
MIGA2	9	129036621..129072082	5,84	4,36	0,644221994
DOLPP1	9	129081111..129090438	10,53	11,42	0,106669629
CRAT	9	complement(129094142..12911118	8,76	8,64	0,243617361
PTPA	9	129110950..129148946	27,66	39,53	-0,292081232
gene:ENSG000	9	129139413..129260575	0,51	0,15	1,984933898
IER5L	9	complement(129175552..12917826	1,07	1,79	-0,521359746
NTMT1	9	129608884..129636135	9,18	10,49	0,03034059
C9orf50	9	complement(129612225..12962110	-	0,01	-2,89252005
ASB6	9	complement(129634604..12964216	8,70	8,97	0,17977035
PRRX2	9	129665647..129722674	15,19	25,97	-0,550377193
PTGES	9	complement(129738331..12975304	0,60	14,46	-4,357651592
TOR1B	9	129803157..129811281	7,62	9,52	-0,097130172
TOR1A	9	complement(129812942..12982424	6,98	13,08	-0,683201816
C9orf78	9	complement(129827290..12983586	10,12	13,94	-0,239797909
USP20	9	129834698..129881828	6,88	8,04	-0,001221245
FNBP1	9	complement(129887187..13004318	8,75	15,72	-0,621828134
GPR107	9	130053426..130140169	18,13	16,97	0,3182486
NCS1	9	130172404..130237303	23,22	32,34	-0,254989577
HMCN2	9	130265760..130434123	0,11	0,15	-0,218090152
ASS1	9	130444961..130501274	77,87	81,35	0,160015663
FUBP3	9	130578965..130638352	8,49	10,52	-0,086753761
PRDM12	9	130664594..130682986	0,20	0,42	-0,805823172
EXOSC2	9	130693721..130707288	4,87	7,36	-0,372078012
ABL1	9	130713043..130887675	23,74	33,39	-0,268656443
gene:ENSG000	9	130713626..130713724	-	-	#N/D
QRFP	9	complement(130892707..13089681	0,46	0,92	-0,759963738
FIBCD1	9	complement(130902438..13093928	2,73	2,16	0,559777899
LAMC3	9	131009174..131094473	0,33	1,37	-1,842983438
AIF1L	9	131096476..131123152	2,43	0,68	2,058433349
NUP214	9	131125573..131234663	11,18	10,10	0,368852475
FAM78A	9	complement(131258076..13127736	0,81	1,71	-0,854767567
PLPP7	9	131289459..131359022	1,19	2,31	-0,728820745
PRRC2B	9	131373636..131500197	37,02	48,14	-0,155932762
gene:ENSG000	9	131430129..131430218	-	-	#N/D
gene:ENSG000	9	131432759..131446473	-	-	#N/D
POMT1	9	131502789..131523806	1,78	3,14	-0,592892095
UCK1	9	complement(131523801..13153126	7,15	6,53	0,352498653
PRRT1B	9	131545523..131559815	0,02	0,02	0,677647514
RAPGEF1	9	complement(131576770..13174007	10,13	13,53	-0,194602829
MED27	9	complement(131852928..13207986	8,25	12,22	-0,343897827
NTNG2	9	132162058..132244526	0,63	2,24	-1,614603879
SETX	9	complement(132261356..13235498	6,87	6,06	0,403666242
TTF1	9	complement(132375548..13240685	3,48	3,34	0,281738969

CFAP77	9	132410043..132573319	0,02	-	4,516772761
BARHL1	9	132582606..132590252	0,02	0,03	-0,742377908
DDX31	9	complement(132592997..13267040	3,27	5,30	-0,473297756
GTF3C4	9	132670035..132694953	6,93	8,62	-0,09114249
AK8	9	complement(132725578..13287877	0,20	0,09	1,347985101
SPACA9	9	132878027..132890201	3,24	2,00	0,913670811
TSC1	9	complement(132891348..13294687	5,51	5,04	0,352296008
GFI1B	9	132944000..132991687	0,01	-	4,516772761
GTF3C5	9	133030675..133058503	17,50	19,31	0,080933873
CEL	9	133061981..133071861	0,41	0,69	-0,520806424
RALGDS	9	complement(133097720..13314933	8,00	8,48	0,138104294
gene:ENSG000	9	complement(133098121..13316391	0,23	0,59	-1,163105886
GBGT1	9	complement(133152948..13316393	1,82	3,05	-0,517325294
OBP2B	9	complement(133205277..13320925	-	-	#N/D
ABO	9	complement(133233278..13327602	-	-	#N/D
SURF6	9	complement(133328776..13333618	4,38	3,81	0,423359164
MED22	9	complement(133338312..13334813	7,43	8,36	0,052328941
RPL7A	9	133348218..133351426	559,14	#####	0,215484403
SURF1	9	complement(133351758..13335667	9,10	11,65	-0,133258536
SURF2	9	133356550..133361158	1,16	3,09	-1,184109753
SURF4	9	complement(133361450..13337616	149,82	#####	0,60903608
STKLD1	9	133376366..133406096	0,47	0,46	0,231585954
REXO4	9	complement(133406059..13341809	6,59	8,94	-0,21728169
ADAMTS13	9	133414358..133459402	0,87	1,06	-0,063830454
CACFD1	9	133459965..133470848	2,44	1,16	1,297782107
SLC2A6	9	complement(133471094..13347912	0,53	4,63	-2,890744764
MYMK	9	complement(133514586..13352861	-	-	#N/D
ADAMTSL2	9	133532164..133575519	0,05	0,02	1,657777123
FAM163B	9	complement(133577081..13360938	-	-	#N/D
DBH	9	133636363..133659329	-	-	0,188027316
SARDH	9	complement(133663560..13373995	-	-	0,188027316
VAV2	9	complement(133761894..13399260	9,05	10,30	0,035859376
BRD3OS	9	134025481..134034666	2,98	4,42	-0,344836471
BRD3	9	complement(134030305..13406853	9,38	12,35	-0,173273458
WDR5	9	134135365..134159968	10,01	16,75	-0,520284974
gene:ENSG000	9	134298463..134300413	0,02	-	4,516772761
RXRA	9	134317098..134440585	15,88	14,86	0,319590453
COL5A1	9	134641803..134844843	68,57	64,72	0,306334999
FCN2	9	134880810..134887523	-	-	0,188027316
FCN1	9	complement(134903232..13491791	0,00	0,00	-0,01185201
gene:ENSG000	9	134992958..134995854	-	-	#N/D
OLFM1	9	135075422..135121180	6,71	4,82	0,69929014
PPP1R26	9	135479079..135488893	10,27	17,36	-0,53477109
PIERCE1	9	complement(135495181..13550173	1,20	0,89	0,655800924
MRPS2	9	135499984..135504673	14,89	26,66	-0,61741705
LCN1	9	135521438..135526540	0,01	-	3,068695355
OBP2A	9	135546126..135549969	-	-	#N/D
PAEP	9	135561756..135566955	-	0,04	-5,071123353

gene:ENSG000	9	135604345..135619893	-	-	0,188027316
GLT6D1	9	complement(135623648..13563954	-	-	#N/D
LCN9	9	135663309..135666961	-	-	0,188027316
SOHLH1	9	complement(135693407..13570449	-	-	#N/D
KCNT1	9	135702185..135795508	0,07	0,03	1,508704279
CAMSAP1	9	complement(135808487..13590754	7,63	7,68	0,213818005
UBAC1	9	complement(135932969..13596137	11,97	15,27	-0,128283084
NACC2	9	complement(136006537..13609528	7,42	10,01	-0,207791772
TMEM250	9	complement(136114581..13611887	14,47	14,64	0,206216215
LHX3	9	complement(136196250..13620512	0,00	-	3,068695355
QSOX2	9	complement(136206333..13624581	24,69	33,79	-0,229528152
CCDC187	9	complement(136249973..13630690	0,00	0,01	-0,602143793
gene:ENSG000	9	136249978..136251205	-	-	#N/D
GPSM1	9	136327476..136359605	9,80	7,72	0,566019993
DNLZ	9	complement(136359480..13636374	1,40	0,94	0,796091947
gene:ENSG000	9	complement(136361904..13637364	0,35	0,61	-0,576603911
CARD9	9	complement(136363956..13637368	0,03	0,01	1,487770062
SNAPC4	9	complement(136375567..13640017	4,65	5,29	0,038244205
ENTR1	9	complement(136401922..13641061	12,03	19,62	-0,482381854
PMPCA	9	136410630..136427992	14,39	13,99	0,263360162
INPP5E	9	complement(136428619..13643984	4,03	7,04	-0,580472845
SEC16A	9	complement(136440096..13648375	35,16	20,37	1,01078167
NOTCH1	9	complement(136494433..13654604	4,13	5,78	-0,262184578
EGFL7	9	136658856..136672678	7,72	9,03	-0,001798148
AGPAT2	9	complement(136673143..13668745	34,40	29,20	0,459586457
DIPK1B	9	136712572..136724742	4,43	8,71	-0,751873341
LCN10	9	complement(136738167..13674335	0,02	-	5,224730805
gene:ENSG000	9	complement(136738167..13674845	-	-	#N/D
LCN6	9	complement(136744017..13674852	-	-	0,188027316
LCN8	9	complement(136754386..13675854	-	-	#N/D
LCN15	9	complement(136759634..13676625	-	-	#N/D
TMEM141	9	136791344..136793317	22,67	17,44	0,60160218
gene:ENSG000	9	136791379..136800595	0,22	0,67	-1,371968249
CCDC183	9	136796338..136807741	0,47	0,38	0,534199066
RABL6	9	136807943..136841187	22,48	26,21	0,001787474
AJM1	9	136842478..136848801	3,65	2,14	0,994311827
PHPT1	9	136848724..136851027	31,46	27,11	0,437632607
MAMDC4	9	136850943..136860799	3,86	1,74	1,373156838
EDF1	9	complement(136862119..13686630	70,65	57,63	0,517030179
TRAF2	9	136881912..136926607	12,64	14,61	0,014233418
FBXW5	9	complement(136940435..13694473	15,45	11,43	0,658095393
C8G	9	136945185..136946975	0,28	0,26	0,37286305
LCN12	9	136949551..136955497	0,04	0,03	0,524121557
PTGDS	9	136975092..136981742	0,01	0,01	-0,01185201
gene:ENSG000	9	136977518..136985435	-	-	#N/D
LCNL1	9	136981904..136986410	0,01	-	3,967268276
PAXX	9	136992422..136993984	15,07	19,47	-0,146752201
CLIC3	9	complement(136994608..13699656	0,21	0,43	-0,788818581

ABCA2	9	complement(137007234..13702891	14,18	14,70	0,170965548
FUT7	9	complement(137030174..13703208	0,03	0,11	-1,495423763
gene:ENSG000	9	complement(137031241..13704043	0,10	-	5,88622595
NPDC1	9	complement(137039463..13704617	31,67	34,02	0,120002064
ENTPD2	9	complement(137048107..13705406	0,03	0,04	-0,291161992
SAPCD2	9	complement(137062127..13707055	9,30	13,77	-0,342831809
UAP1L1	9	137077517..137084539	7,57	9,43	-0,093579211
MAN1B1	9	137086857..137109189	13,35	12,65	0,300450023
DPP7	9	complement(137110542..13711830	24,80	26,80	0,110908727
GRIN1	9	137138346..137168756	0,01	0,01	0,370333154
LRRC26	9	complement(137168758..13717005	-	-	0,188027316
gene:ENSG000	9	complement(137168854..13717198	-	-	0,188027316
TMEM210	9	complement(137170858..13717205	-	-	#N/D
ANAPC2	9	complement(137174784..13718856	10,40	9,77	0,313496978
SSNA1	9	137188660..137190370	25,49	33,81	-0,18410786
TPRN	9	complement(137191617..13720419	6,43	6,63	0,178720559
TMEM203	9	complement(137204082..13720564	14,01	18,88	-0,20731287
NDOR1	9	137205685..137219361	9,56	11,71	-0,070107908
gene:ENSG000	9	137217494..137219361	-	-	0,188027316
RNF208	9	complement(137220247..13722224	0,68	1,52	-0,937887369
CYSRT1	9	137225174..137226315	0,24	1,44	-2,351812703
RNF224	9	137227502..137229640	0,19	0,50	-1,135565977
SLC34A3	9	137230757..137236555	0,39	0,71	-0,653321988
TUBB4B	9	137241287..137243707	184,52	#####	-0,015416089
CIMIP2A	9	complement(137243584..13724777	0,13	0,32	-1,082062778
STPG3	9	137251261..137253483	0,65	0,80	-0,079351899
NELFB	9	137255327..137273542	26,97	33,33	-0,082061147
TOR4A	9	137277726..137282641	0,96	6,01	-2,420078091
NRARP	9	complement(137299631..13730227	0,24	1,00	-1,79650791
EXD3	9	complement(137306896..13742321	1,48	1,26	0,460381166
NOXA1	9	137423350..137434406	0,12	0,10	0,426338815
ENTPD8	9	complement(137434364..13744181	0,19	0,16	0,524667622
NSMF	9	complement(137447570..13745933	15,98	18,83	-0,013703415
PNPLA7	9	complement(137459952..13755040	0,30	0,26	0,388545257
MRPL41	9	137551879..137552555	23,45	26,12	0,067976342
DPH7	9	complement(137554444..13757892	14,61	8,01	1,089638088
ZMYND19	9	complement(137582081..13759051	14,39	18,29	-0,122934051
ARRDC1	9	137605685..137615360	5,90	6,64	0,052926704
EHMT1	9	137618992..137870016	2,64	3,55	-0,203718138
CACNA1B	9	137877782..138124624	0,00	0,01	-0,345401666
TUBB8	10	complement(46888..74163)	0,03	0,00	2,805678585
ZMYND11	10	130088..254637	1,85	2,72	-0,332020852
DIP2C	10	complement(274201..689668)	5,20	4,59	0,404399846
LARP4B	10	complement(806933..931821)	5,60	7,37	-0,172367771
GTPBP4	10	988434..1019932	7,74	9,50	-0,071305802
IDI2	10	complement(1018910..1025859)	-	-	0,188027316
IDI1	10	complement(1039152..1049119)	5,71	1,16	2,5186989
WDR37	10	1049538..1160991	1,84	1,38	0,639769022

ADARB2	10	complement(1177313..1737525)	0,00	-	4,516772761
PFKP	10	3066333..3137718	45,76	39,89	0,420978692
PITRM1	10	complement(3137728..3172841)	11,60	16,59	-0,29297004
KLF6	10	complement(3775996..3785281)	10,87	7,55	0,749141185
AKR1E2	10	4786629..4848062	0,03	0,08	-1,019619711
AKR1C1	10	4963253..4983283	7,92	3,38	1,453767461
AKR1C2	10	complement(4987775..5018031)	12,29	3,90	1,878276624
AKR1C3	10	5035354..5107686	10,14	6,29	0,912806182
AKR1C8	10	complement(5153920..5185187)	0,03	0,07	-0,820024868
AKR1C4	10	5195462..5218949	0,03	0,04	-0,032860829
gene:ENSG000	10	5346651..5347277	-	-	#N/D
UCN3	10	5364966..5374692	-	-	#N/D
TUBAL3	10	complement(5393101..5404828)	0,02	0,14	-2,640348898
NET1	10	5412557..5459056	6,58	9,45	-0,29914239
CALML5	10	complement(5498697..5499570)	-	-	0,188027316
CALML3	10	5524961..5526771	-	-	0,188027316
ASB13	10	complement(5638867..5666595)	1,52	6,00	-1,759367832
TASOR2	10	5684731..5763779	7,05	7,72	0,092057297
GDI2	10	complement(5765223..5842132)	35,21	39,61	0,053411531
ANKRD16	10	complement(5861616..5889906)	1,22	1,26	0,173805779
FBH1	10	5890203..5937594	8,00	9,76	-0,064128188
IL15RA	10	complement(5943639..5978187)	2,42	5,42	-0,937344864
IL2RA	10	complement(6010689..6062370)	0,00	-	3,068695355
RBM17	10	6089034..6117457	3,82	5,79	-0,377009585
PFKFB3	10	6144934..6254644	7,64	11,25	-0,334861515
PRKCQ	10	complement(6427143..6580301)	-	0,03	-5,734998842
SFMBT2	10	complement(7158624..7411488)	0,13	0,17	-0,175820716
gene:ENSG000	10	7411599..7413467	-	0,01	-2,89252005
ITIH5	10	complement(7559270..7666998)	0,00	0,01	-1,877914477
ITIH2	10	7703316..7749520	0,00	-	3,068695355
KIN	10	complement(7750962..7787993)	0,64	0,59	0,350174264
ATP5F1C	10	7788147..7807815	52,77	68,29	-0,148827748
TAF3	10	7818497..8016631	0,78	1,13	-0,317772683
GATA3	10	8045378..8075198	0,10	0,00	4,828866653
gene:ENSG000	10	10394670..10395737	-	-	#N/D
CELF2	10	10798397..11336675	0,02	0,11	-2,081103478
gene:ENSG000	10	11395808..11398792	-	-	#N/D
USP6NL	10	complement(11460510..11611666)	0,78	2,36	-1,367338176
ECHDC3	10	11742366..11764070	5,56	7,61	-0,230278374
PROSER2	10	11823339..11872277	0,19	2,38	-3,397930467
UPF2	10	complement(11920022..12043170)	2,99	2,45	0,509563477
DHTKD1	10	12068954..12123221	7,88	10,72	-0,221083393
SEC61A2	10	12129637..12169961	2,18	3,08	-0,277836229
NUDT5	10	complement(12165330..12195891)	5,60	7,15	-0,129041443
CDC123	10	12195965..12250589	18,87	33,05	-0,58588674
CAMK1D	10	12349547..12835545	2,16	2,75	-0,124254305
CCDC3	10	complement(12896625..13099652)	0,85	1,56	-0,651615838
OPTN	10	13099449..13138308	4,23	6,56	-0,409670236

MCM10	10	13161558..13211110	2,80	4,13	-0,335750818
UCMA	10	complement(13221766..13234374)	0,01	0,04	-1,478387936
PHYH	10	complement(13277796..13302412)	1,18	2,64	-0,946144973
SEPHS1	10	complement(13317428..13348298)	8,65	13,13	-0,378325328
BEND7	10	complement(13438484..13529014)	0,27	0,42	-0,40381199
PRPF18	10	13586939..13668445	1,77	1,85	0,153714138
FRMD4A	10	complement(13643706..14462142)	0,83	1,84	-0,927586557
FAM107B	10	complement(14518557..14774897)	4,87	8,42	-0,568372862
CDNF	10	complement(14819245..14838575)	0,08	0,11	-0,242839224
MSANTD7	10	14838302..14846999	3,05	3,84	-0,111036226
HSPA14	10	14838306..14871741	1,98	2,47	-0,094783946
SUV39H2	10	14878820..14904315	1,66	2,56	-0,404191064
DCLRE1C	10	complement(14897359..14954432)	0,84	0,94	0,048408985
MEIG1	10	14959388..14988050	0,02	-	3,068695355
OLAH	10	15032227..15073853	0,02	0,11	-2,243372656
ACBD7	10	complement(15075475..15088776)	0,38	0,61	-0,470419141
RPP38	10	15097180..15139818	2,56	3,31	-0,146704649
NMT2	10	complement(15102584..15168693)	5,92	5,24	0,399193019
FAM171A1	10	complement(15211643..15371289)	7,66	9,09	-0,023290411
ITGA8	10	complement(15513954..15719922)	0,35	0,18	1,196536601
MINDY3	10	complement(15778170..15860507)	1,13	2,02	-0,610543938
PTER	10	16436943..16513745	1,38	3,17	-0,971413509
C1QL3	10	complement(16513734..16521879)	0,17	0,45	-1,185225212
RSU1	10	complement(16590611..16817463)	10,15	9,39	0,334858569
CUBN	10	complement(16823966..17129811)	0,09	0,24	-1,180761504
TRDMT1	10	complement(17137336..17202054)	0,15	0,38	-1,147892761
VIM	10	17228241..17237593	499,16	#####	0,96764847
ST8SIA6	10	complement(17315421..17454595)	0,00	0,01	-2,190455314
HACD1	10	complement(17589032..17617374)	0,90	1,78	-0,767907096
STAM	10	17644151..17716824	3,72	4,46	-0,03874654
TMEM236	10	17752201..17800868	0,01	0,04	-1,905028696
MRC1	10	17809348..17911164	-	-	0,188027316
SLC39A12	10	17951839..18043292	-	-	0,188027316
CACNB2	10	18140424..18543557	0,11	0,19	-0,525269964
NSUN6	10	complement(18545561..18659285)	0,87	1,41	-0,476603504
ARL5B	10	18659431..18681639	1,28	1,72	-0,209043429
MALRD1	10	19048801..19790401	-	0,00	-2,89252005
PLXDC2	10	19816239..20289856	0,13	0,00	6,152370853
NEBL	10	complement(20779973..21293011)	0,00	0,01	-1,009277154
SKIDA1	10	complement(21513475..21526368)	0,08	0,17	-0,896370857
MLLT10	10	21524646..21743630	2,90	4,34	-0,355733595
DNAJC1	10	complement(21756548..22003769)	1,83	3,00	-0,48567832
EBLN1	10	complement(22208475..22218015)	-	-	0,188027316
gene:ENSG000	10	22252072..22254589	0,02	0,07	-1,495423763
COMMD3	10	22316386..22320306	4,09	7,45	-0,643255797
COMMD3-BMI1	10	22316388..22329542	2,39	4,86	-0,802602206
BMI1	10	22321099..22331484	0,79	2,71	-1,564786128
SPAG6	10	22345445..22454224	-	0,01	-3,804623889

PIP4K2A	10	complement(22534854..22714578)	3,65	5,74	-0,429771538
gene:ENSG000	10	complement(22693953..22694639)	0,17	0,24	-0,27623233
ARMC3	10	22928024..23038523	-	-	#N/D
MSRB2	10	23095579..23122013	3,91	4,14	0,140868413
PTF1A	10	23192312..23194245	-	-	0,188027316
C10orf67	10	complement(23202696..23344845)	0,00	-	4,516772761
OTUD1	10	23439075..23442377	1,27	1,17	0,339796566
KIAA1217	10	23694746..24547848	1,40	1,61	0,024589918
gene:ENSG000	10	complement(24481180..24483034)	0,14	0,11	0,541455488
ARHGAP21	10	complement(24583609..24723887)	7,62	7,30	0,2849562
PRTFDC1	10	complement(24848614..24952606)	0,08	0,04	1,306856523
ENKUR	10	complement(24981979..25062279)	0,01	0,02	-0,509043067
THNSL1	10	25016612..25026664	0,59	1,30	-0,925397715
GPR158	10	25174802..25602229	0,00	-	3,068695355
MYO3A	10	25934229..26212532	-	-	#N/D
GAD2	10	26216665..26304558	-	-	0,188027316
APBB1IP	10	26438341..26567803	0,01	-	4,516772761
gene:ENSG000	10	complement(26695091..26697625)	0,01	0,03	-0,742377908
PDSS1	10	26697701..26746798	1,58	3,03	-0,712242505
ABI1	10	complement(26746593..26861087)	4,48	5,37	-0,036263208
ANKRD26	10	complement(26973793..27100494)	0,28	0,49	-0,597540461
YME1L1	10	complement(27110111..27155266)	12,31	14,34	0,003394094
MASTL	10	27154824..27187953	4,70	5,20	0,077445248
ACBD5	10	complement(27168135..27243046)	2,04	2,60	-0,13234142
PTCHD3	10	complement(27397121..27414368)	-	-	0,188027316
RAB18	10	27504174..27543207	2,46	1,78	0,689060094
MKX	10	complement(27672874..27746060)	0,12	1,65	-3,540678377
ODAD2	10	complement(27775186..27999079)	0,21	0,75	-1,648978095
MPP7	10	complement(28050993..28334486)	0,05	0,25	-2,140832369
WAC	10	28532493..28623112	5,42	8,43	-0,412302615
BAMBI	10	28677510..28682932	5,56	2,14	1,597587694
LYZL1	10	29289061..29318328	-	-	#N/D
SVIL	10	complement(29457338..29736959)	5,94	5,25	0,399182944
JCAD	10	complement(30012803..30115494)	6,49	3,62	1,06627519
MTPAP	10	complement(30309801..30374448)	1,96	3,33	-0,540077876
MAP3K8	10	30434021..30461833	0,15	0,67	-1,972932022
LYZL2	10	complement(30611779..30629762)	-	-	#N/D
ZNF438	10	complement(30820207..31031937)	1,18	1,22	0,181511649
ZEB1	10	31318495..31529814	7,93	8,38	0,144041054
ARHGAP12	10	complement(31805398..31928876)	2,81	3,35	-0,027793282
KIF5B	10	complement(32009015..32056425)	10,89	17,47	-0,458156436
EPC1	10	complement(32267751..32378798)	1,17	1,94	-0,508746477
CCDC7	10	32446140..32882874	0,05	0,12	-1,229481981
ITGB1	10	complement(32887273..33005792)	34,17	35,91	0,151118371
NRP1	10	complement(33177492..33336262)	3,86	5,38	-0,25754949
PARD3	10	complement(34109560..34815325)	4,52	7,50	-0,50651808
CUL2	10	complement(35008504..35127006)	2,04	3,13	-0,400108099
CREM	10	35126791..35212958	0,37	0,96	-1,158741683

CCNY	10	35247025..35572669	4,54	8,08	-0,609968675
GJD4	10	35605341..35608935	0,01	0,01	-0,01185201
FZD8	10	complement(35638247..35642296)	2,58	7,38	-1,290694479
ANKRD30A	10	37125598..37232567	-	-	0,188027316
ZNF248	10	complement(37776526..37858106)	1,08	1,16	0,11924883
ZNF25	10	complement(37949573..37976647)	1,04	1,56	-0,358320313
ZNF33A	10	38010650..38065088	0,86	1,18	-0,240009304
ZNF37A	10	38094334..38150293	0,58	1,22	-0,851678948
PLD5P1	10	38094368..38360098	0,58	0,12	2,448634596
ZNF33B	10	complement(42574185..42638570)	0,19	0,63	-1,514000782
BMS1	10	42782795..42834937	5,05	6,74	-0,194142403
RET	10	43077064..43130351	0,22	0,30	-0,223367083
CSGALNACT2	10	43138445..43185302	3,69	4,99	-0,2126295
RASGEF1A	10	complement(43194535..43267065)	0,11	0,07	0,9331436
FXYD4	10	43371636..43376335	-	-	#N/D
HNRNPF	10	complement(43385617..43409186)	25,57	32,67	-0,130590512
ZNF487	10	43436841..43483181	0,12	0,20	-0,546422776
ZNF239	10	complement(43556344..43574618)	0,61	2,04	-1,519385429
ZNF485	10	43606419..43617904	0,37	0,67	-0,651638909
ZNF32	10	complement(43643860..43648881)	4,75	6,50	-0,229905453
CXCL12	10	complement(44370165..44386493)	0,03	0,21	-2,351812703
TMEM72	10	44911316..44937010	0,01	-	4,516772761
RASSF4	10	44959407..44995891	0,99	1,01	0,198423015
DEPP1	10	complement(44970981..44978809)	15,98	7,35	1,343012252
ZNF22	10	45000923..45005326	3,81	7,55	-0,764436667
OR13A1	10	complement(45302298..45315608)	0,01	-	3,967268276
ALOX5	10	45374176..45446119	0,00	-	3,068695355
MARCHF8	10	complement(45454585..45594906)	1,60	1,67	0,164636548
ZFAND4	10	complement(45615500..45672780)	0,30	0,53	-0,570605638
WASHC2C	10	45727200..45792964	2,51	3,98	-0,438997054
AGAP4	10	complement(45825594..45853875)	1,15	1,35	-0,003024291
TIMM23	10	45972489..46003742	18,57	25,09	-0,210624859
NCOA4	10	complement(46005088..46030623)	17,38	18,92	0,100240659
MSMB	10	complement(46033307..46048180)	0,01	-	3,068695355
ANTXRL	10	46286345..46330207	0,01	0,00	1,436225396
ANXA8L1	10	46375627..46391784	0,00	0,22	-5,045026341
NPY4R	10	complement(46461099..46465958)	0,01	0,04	-2,447197442
GPRIN2	10	complement(46541736..46556658)	0,01	0,00	3,122038495
SYT15	10	46578217..46594173	0,12	0,10	0,480892176
PTPN20	10	46911396..47002488	-	-	0,188027316
GDF10	10	47300197..47313577	0,00	0,00	-0,01185201
GDF2	10	47322454..47327588	-	-	0,188027316
RBP3	10	47348363..47357881	0,01	-	4,516772761
ZNF488	10	complement(47365496..47384293)	0,34	0,34	0,227257374
ANXA8	10	complement(47460162..47484158)	0,01	0,06	-3,112412319
FAM25G	10	complement(47487219..47491700)	-	-	#N/D
AGAP9	10	complement(47501854..47523638)	1,76	2,50	-0,285716458
SYT15B	10	complement(47744717..47763592)	0,42	0,31	0,664862733

NPY4R2	10	47918662..47923524	-	-	0,188027316
FAM25C	10	complement(47995322..47999791)	-	-	#N/D
FRMPD2	10	complement(48153088..48274696)	0,03	0,03	-0,038687174
MAPK8	10	48306639..48439360	1,53	2,27	-0,347966844
gene:ENSG000	10	complement(48443836..48445820)	0,16	0,18	0,062859924
ARHGAP22	10	complement(48446036..48656265)	1,74	1,71	0,242344032
WDFY4	10	48684873..48982956	0,01	0,00	1,704893826
LRRC18	10	complement(48909480..48939840)	-	-	#N/D
VSTM4	10	complement(49014236..49115522)	1,30	1,13	0,419616189
FAM170B	10	complement(49131154..49134021)	-	-	#N/D
TMEM273	10	complement(49154725..49188585)	-	-	0,188027316
C10orf71	10	49299170..49327492	-	-	#N/D
DRGX	10	complement(49364066..49396089)	-	0,08	-6,89165328
ERCC6	10	complement(49454168..49539538)	1,28	2,12	-0,504820461
CHAT	10	49609095..49667942	-	-	#N/D
SLC18A3	10	49610310..49612720	-	-	#N/D
C10orf53	10	49679651..49710261	-	-	0,188027316
OGDHL	10	complement(49734641..49762379)	0,00	0,01	-1,478387936
PARG	10	complement(49818279..49970203)	2,62	4,03	-0,397426229
TIMM23B	10	49942049..49974850	1,89	1,82	0,277098714
TIMM23B-AGAF	10	49942056..50011654	1,15	1,72	-0,354125527
AGAP6	10	49982190..50011654	1,56	1,87	-0,043916314
WASHC2A	10	50067888..50133509	2,40	3,45	-0,300017618
ASAH2	10	complement(50182778..50279720)	0,07	0,10	-0,278418015
SGMS1	10	complement(50305586..50625163)	0,81	1,70	-0,843527805
gene:ENSG000	10	50334538..50336123	0,20	0,20	0,231894219
ASAH2B	10	50739318..50816495	0,46	0,72	-0,430968154
A1CF	10	complement(50799409..50885675)	0,01	0,00	1,704893826
PRKG1	10	50990888..52298423	0,14	0,01	4,885871388
CSTF2T	10	complement(51695486..51699595)	4,07	4,39	0,112162195
DKK1	10	52314281..52318042	41,95	17,38	1,494157101
MBL2	10	complement(52765380..52772784)	-	0,00	-2,89252005
PCDH15	10	complement(53802771..55627942)	-	-	0,188027316
ZWINT	10	complement(56357227..56361273)	9,11	11,76	-0,145015195
IPMK	10	complement(58191517..58267894)	0,61	0,81	-0,176337809
CISD1	10	58269162..58289586	2,84	4,64	-0,48374464
UBE2D1	10	58334979..58370751	0,52	1,16	-0,939973695
TFAM	10	58385345..58399220	0,72	2,16	-1,359018676
BICC1	10	58512872..58831435	2,83	2,31	0,51846017
PHYHIPL	10	59176643..59247774	-	0,00	-2,89252005
FAM13C	10	complement(59246130..59363181)	0,00	0,04	-3,929150322
SLC16A9	10	complement(59650764..59736002)	0,35	1,30	-1,646305426
MRLN	10	complement(59736692..59756041)	-	-	0,188027316
CCDC6	10	complement(59788747..59906556)	5,54	8,46	-0,386399596
ANK3	10	complement(60026298..60733490)	0,02	0,11	-2,335649993
CDK1	10	60778331..60794852	18,99	24,46	-0,142218914
RHOBTB1	10	complement(60869438..61001440)	0,73	1,82	-1,100280237
TMEM26	10	complement(61406642..61453381)	0,00	-	3,967268276

CABCOCO1	10	61662929..61766766	0,01	0,01	-0,923955849
ARID5B	10	61901684..62096944	4,89	2,63	1,119064042
RTKN2	10	complement(62183035..62268844)	-	0,13	-9,282913273
ZNF365	10	62374192..62480288	0,05	0,70	-3,69923231
gene:ENSG000	10	62374192..62672011	-	0,02	-4,758582517
ADO	10	62804720..62808479	3,01	4,61	-0,390205027
EGR2	10	complement(62811996..62819167)	0,02	0,06	-1,382135565
NRBF2	10	63133247..63155031	4,82	5,27	0,094263105
JMJD1C	10	complement(63167221..63521850)	1,91	3,41	-0,609563152
REEP3	10	63521401..63625128	4,29	5,20	-0,054009887
CTNNA3	10	complement(65912457..67763637)	0,00	0,00	0,921097799
LRRTM3	10	66926036..67101551	-	-	#N/D
gene:ENSG000	10	66926308..66926346	-	-	#N/D
DNAJC12	10	complement(67796669..67838188)	0,34	0,48	-0,277641547
SIRT1	10	67884656..67918390	1,70	2,18	-0,139685568
HERC4	10	complement(67921899..68075301)	3,03	2,88	0,298417916
MYPN	10	68087897..68212017	1,73	4,36	-1,106727501
ATOH7	10	complement(68230595..68232113)	-	0,02	-3,804623889
PBLD	10	complement(68282660..68333049)	0,60	0,58	0,291290979
HNRNPH3	10	68331174..68343193	11,03	17,43	-0,437353338
RUFY2	10	complement(68341107..68407294)	0,97	1,15	-0,028139022
DNA2	10	complement(68414064..68472121)	1,02	1,74	-0,551222892
SLC25A16	10	complement(68477998..68527523)	0,52	0,57	0,094511369
TET1	10	68560337..68694487	0,46	0,55	-0,05141869
CCAR1	10	68721012..68792377	4,00	5,80	-0,313868012
STOX1	10	68827531..68895432	0,02	0,08	-1,733452083
DDX50	10	68901286..68946847	10,83	10,94	0,208616723
DDX21	10	68956135..68985068	15,90	24,86	-0,421379792
KIFBP	10	68988803..69043544	2,43	2,81	0,010602512
SRGN	10	69088103..69104805	0,85	1,43	-0,521093106
VPS26A	10	69123512..69174412	0,61	1,46	-1,035071468
SUPV3L1	10	69180234..69209099	4,49	8,14	-0,636438152
HKDC1	10	69220332..69267552	0,15	0,32	-0,886501061
HK1	10	69269984..69401884	33,41	33,00	0,240651535
TACR2	10	complement(69403903..69416918)	0,10	0,11	0,07524965
TSPAN15	10	69451465..69507666	1,49	5,79	-1,731180761
NEUROG3	10	complement(69571698..69573422)	-	-	#N/D
FAM241B	10	69630247..69633596	1,69	3,93	-0,996896713
COL13A1	10	69801880..69964275	6,24	4,16	0,808454235
MACROH2A2	10	70052544..70112282	1,22	1,56	-0,134423503
AIFM2	10	complement(70098223..70132934)	2,01	4,47	-0,931643612
TYSND1	10	complement(70137981..70146700)	4,95	8,83	-0,611228106
SAR1A	10	complement(70147289..70170523)	9,88	8,64	0,417615532
PPA1	10	complement(70202835..70233911)	18,96	25,82	-0,221941383
NPFFR1	10	complement(70247329..70284004)	0,00	0,01	-0,979341556
LRRC20	10	complement(70298970..70382650)	2,78	4,12	-0,342871185
EIF4EBP2	10	70404145..70428618	7,08	5,36	0,624610899
NODAL	10	complement(70431936..70447951)	0,59	0,43	0,661950635

gene:ENSG000	10	70434829..70436556	0,61	0,41	0,776492807
PALD1	10	70478767..70668754	2,08	4,47	-0,876760549
PRF1	10	complement(70597348..70602759)	0,01	0,02	-0,579815015
ADAMTS14	10	70672506..70762441	0,98	1,20	-0,060556265
TBATA	10	complement(70771238..70785401)	-	-	#N/D
SGPL1	10	70815905..70895816	2,45	5,83	-1,029245185
PCBD1	10	complement(70882280..70888565)	11,93	25,22	-0,85636197
UNC5B	10	71212570..71302864	1,56	8,53	-2,230601023
SLC29A3	10	71319259..71381423	0,29	0,47	-0,472539984
gene:ENSG000	10	71364243..71366374	0,07	0,07	0,199980087
CDH23	10	71396920..71815947	0,02	0,02	0,271943794
C10orf105	10	complement(71711701..71737824)	0,01	-	5,224730805
VSIR	10	complement(71747556..71773520)	1,95	2,88	-0,338883099
PSAP	10	complement(71816298..71851251)	134,99	#####	-0,094141675
CHST3	10	71964395..72013558	5,80	6,20	0,12590375
SPOCK2	10	complement(72059034..72089032)	0,04	0,16	-1,658571518
ASCC1	10	complement(72096032..72217134)	2,53	3,35	-0,181555492
ANAPC16	10	72216000..72235860	5,36	7,69	-0,297904668
DDIT4	10	72273919..72276036	46,00	69,99	-0,382388128
DNAJB12	10	complement(72332830..72355149)	3,83	5,36	-0,263673776
MICU1	10	complement(72367340..72626131)	6,89	9,60	-0,255147217
MCU	10	72692143..72887694	4,11	6,10	-0,347658382
gene:ENSG000	10	72730557..72731175	-	0,02	-2,89252005
gene:ENSG000	10	72756398..72757016	0,05	0,23	-1,859185627
OIT3	10	72893584..72933036	-	-	0,188027316
PLA2G12B	10	complement(72934762..72954806)	-	0,02	-4,359055975
P4HA1	10	complement(73007217..73096974)	19,66	12,38	0,890968794
NUDT13	10	73110375..73131828	0,19	0,27	-0,302285567
ECD	10	complement(73130155..73169055)	4,33	4,58	0,142986205
FAM149B1	10	73168119..73244504	0,94	0,96	0,182497033
DNAJC9	10	complement(73183362..73247255)	8,05	10,01	-0,091311586
MRPS16	10	complement(73248843..73252693)	16,01	24,72	-0,403762097
CFAP70	10	complement(73253762..73358864)	0,02	0,25	-3,10137138
ANXA7	10	complement(73375101..73414076)	19,74	27,94	-0,278233168
MSS51	10	complement(73423579..73433561)	0,29	0,49	-0,502977045
PPP3CB	10	complement(73436433..73496024)	1,86	2,94	-0,43380617
USP54	10	complement(73497538..73626117)	0,97	1,98	-0,804478553
MYOZ1	10	complement(73631612..73641474)	-	-	0,188027316
SYNPO2L	10	complement(73644881..73663803)	0,01	0,06	-2,51724811
AGAP5	10	complement(73674287..73698109)	0,89	1,54	-0,555664605
gene:ENSG000	10	complement(73742962..73744316)	8,51	5,25	0,920360619
SEC24C	10	73744372..73772161	42,89	27,09	0,885806554
gene:ENSG000	10	complement(73769264..73772862)	1,06	0,47	1,392464111
FUT11	10	73772276..73780251	4,86	5,11	0,15239303
CHCHD1	10	73782047..73783652	1,80	2,43	-0,205409577
ZSWIM8	10	73785606..73801797	13,85	7,91	1,03189482
gene:ENSG000	10	73791218..73791490	0,47	0,09	2,469244963
gene:ENSG000	10	complement(73796514..73811651)	1,03	0,68	0,813144657

NDST2	10	complement(73801911..73811798)	2,14	3,82	-0,614313802
CAMK2G	10	complement(73812501..73874591)	10,85	12,76	-0,011289981
PLAU	10	73909177..73917496	95,88	#####	-0,370954989
VCL	10	73995193..74121363	39,70	48,68	-0,071061171
AP3M1	10	complement(74120255..74150842)	5,72	7,84	-0,230530617
ADK	10	74151202..74709963	4,09	5,27	-0,141056795
KAT6B	10	74824927..75032624	1,26	1,45	0,014104441
DUSP29	10	complement(75037472..75073642)	-	-	0,188027316
DUSP13B_1	10	complement(75094432..75106499)	-	0,01	-3,804623889
DUSP13B_2	10	complement(75094432..75109206)	0,01	-	3,967268276
DUSP13A	10	complement(75094434..75109221)	-	-	0,188027316
SAMD8	10	75099586..75182123	3,39	3,13	0,336298473
VDAC2	10	75210154..75231448	50,09	46,66	0,325586298
COMTD1	10	complement(75233641..75236030)	1,27	1,25	0,23986974
ZNF503	10	complement(75397830..75401764)	3,01	1,83	0,939707463
LRMDA	10	75431624..76560168	0,13	0,31	-0,964805136
gene:ENSG000	10	75450081..75451725	0,05	0,18	-1,733452083
gene:ENSG000	10	76270206..76270800	0,02	-	3,068695355
KCNMA1	10	complement(76869601..77638369)	3,46	3,69	0,132521692
DLG5	10	complement(77790791..77926755)	9,56	10,14	0,137770507
POLR3A	10	complement(77953148..78029522)	8,02	7,88	0,247830581
RPS24	10	78033760..78056813	34,00	41,60	-0,068054292
ZMIZ1	10	79068966..79316519	26,59	22,62	0,456451571
PPIF	10	79347469..79355334	20,75	29,95	-0,306358794
ZCCHC24	10	complement(79382325..79445624)	9,60	9,77	0,197762292
EIF5AL1	10	79512533..79516440	0,09	0,07	0,519108598
SFTPA2	10	complement(79555852..79560407)	0,04	0,00	3,122038495
SFTPA1	10	79610939..79615455	0,01	-	4,516772761
gene:ENSG000	10	complement(79681973..79684094)	1,54	2,08	-0,20640502
NUTM2B	10	79703227..79714681	0,22	0,32	-0,294020623
NUTM2E	10	79826739..79850878	0,32	0,70	-0,894737938
SFTPD	10	complement(79937467..79982614)	0,01	-	3,068695355
TMEM254	10	80078646..80092557	1,70	2,17	-0,133393856
gene:ENSG000	10	complement(80091411..80091867)	0,40	0,31	0,582943981
PLAC9	10	80131682..80145359	0,40	0,60	-0,331950906
ANXA11	10	complement(80150889..80205572)	15,86	16,63	0,15471469
gene:ENSG000	10	80249710..80254121	-	-	0,188027316
MAT1A	10	complement(80271820..80289658)	-	0,00	-2,89252005
DYDC1	10	complement(80336105..80356755)	-	-	0,188027316
DYDC2	10	80344745..80368073	-	-	0,188027316
PRXL2A	10	80407829..80437115	0,27	1,96	-2,623209925
TSPAN14	10	80454265..80533124	3,22	4,49	-0,257656354
SH2D4B	10	80537902..80646560	0,03	0,01	1,914812876
NRG3	10	81875194..82987179	-	-	#N/D
GHITM	10	84139482..84154841	11,49	15,45	-0,204272634
GPR15LG	10	84173801..84185294	-	-	#N/D
CDHR1	10	84194537..84219621	-	-	0,188027316
LRIT2	10	complement(84220571..84225544)	-	-	#N/D

RGR	10	84230666..84259960	-	-	#N/D
LRIT1	10	complement(84231520..84241546)	-	-	#N/D
CCSER2	10	84328586..84518521	3,38	4,47	-0,182048681
gene:ENSG000	10	complement(85526880..85531115)	-	-	#N/D
gene:ENSG000	10	complement(85555484..85557436)	-	-	0,188027316
GRID1	10	complement(85599552..86366795)	0,10	0,01	3,773441473
WAPL	10	complement(86435256..86521792)	5,35	6,36	-0,027908912
OPN4	10	86654518..86666460	-	-	0,188027316
gene:ENSG000	10	86654518..86700020	-	-	0,188027316
LDB3	10	86666785..86736072	0,05	0,05	0,269034809
BMPR1A	10	86755786..86932825	1,17	1,94	-0,50500085
MMRN2	10	complement(86935540..86969481)	1,26	1,08	0,445474064
gene:ENSG000	10	86948677..86949875	0,04	0,05	-0,034419864
SNCG	10	86958599..86963258	3,40	4,98	-0,327033434
ADIRF	10	86968432..86983934	23,21	15,17	0,837047621
FAM25A	10	87020294..87024730	0,48	0,47	0,257973405
GLUD1	10	complement(87050202..87094843)	23,50	27,78	-0,018154569
SHLD2	10	87094161..87191465	2,76	4,42	-0,456177592
NUTM2A	10	87225448..87236908	0,44	0,67	-0,380462423
NUTM2D	10	87357720..87370695	0,21	0,63	-1,381075858
MINPP1	10	87504875..87553461	1,66	3,01	-0,63531736
PAPSS2	10	87659613..87747705	13,61	11,71	0,440217068
ATAD1	10	complement(87751512..87841361)	2,54	4,68	-0,656322433
KLLN	10	complement(87859158..87863533)	0,03	0,05	-0,421907201
PTEN	10	87862638..87971930	1,89	2,26	-0,036806358
gene:ENSG000	10	87863559..87863654	-	-	#N/D
RNLS	10	complement(88273864..88584530)	0,14	0,01	4,799964753
LIPJ	10	88586753..88606976	-	-	0,188027316
LIPF	10	88664441..88678814	-	-	#N/D
LIPK	10	88706249..88752776	-	-	0,188027316
LIPN	10	88759982..88779626	-	0,01	-4,359055975
LIPM	10	88802730..88820546	-	-	#N/D
ANKRD22	10	complement(88819896..88851844)	0,03	0,06	-1,026620426
STAMBPL1	10	88879734..88975153	1,47	2,18	-0,347620123
ACTA2	10	complement(88934822..88991339)	2,88	3,44	-0,036708357
FAS	10	88953813..89029605	0,49	0,64	-0,162239662
CH25H	10	complement(89205629..89207317)	0,14	0,16	0,027316923
LIPA	10	complement(89213569..89414557)	5,73	7,00	-0,066798935
IFIT2	10	89283694..89309271	2,03	2,26	0,073862788
IFIT3	10	89327307..89377473	1,48	2,97	-0,778103729
IFIT1B	10	89378056..89385205	-	0,01	-2,89252005
IFIT1	10	89392546..89406487	1,56	1,33	0,451848563
IFIT5	10	89414568..89421605	0,89	1,99	-0,933311994
SLC16A12	10	complement(89430299..89556641)	-	0,02	-5,734998842
PANK1	10	complement(89579497..89645242)	0,11	0,31	-1,308821499
KIF20B	10	89701590..89774939	1,21	2,25	-0,669240458
HTR7	10	complement(90740823..90858039)	1,68	2,00	-0,027429235
RPP30	10	90871952..90908553	3,93	5,18	-0,174859268

ANKRD1	10	complement(90912096..90921087)	-	0,09	-6,312013662
PCGF5	10	91163012..91284337	0,73	1,94	-1,177070078
HECTD2	10	91409280..91514829	0,11	0,32	-1,339605424
PPP1R3C	10	complement(91628442..91633071)	0,45	0,94	-0,843252269
TNKS2	10	91798426..91865475	4,39	5,48	-0,096576406
FGFBP3	10	complement(91906584..91909486)	0,11	0,20	-0,714743776
BTAF1	10	91923770..92031437	3,51	4,13	-0,010340719
CPEB3	10	complement(92046692..92291078)	0,15	0,17	0,054158824
MARCHF5	10	92291167..92353964	2,70	3,55	-0,170031284
IDE	10	complement(92451684..92574096)	1,85	2,97	-0,462288362
KIF11	10	92574105..92655395	5,65	7,55	-0,196226647
HHEX	10	92689955..92695647	1,93	2,12	0,085332514
EXOC6	10	92826831..93059493	0,36	0,75	-0,838873077
CYP26C1	10	93060798..93069540	0,02	0,02	0,468811989
CYP26A1	10	93073475..93077885	0,03	-	5,480421266
MYOF	10	complement(93306429..93482334)	20,33	25,34	-0,094726611
CEP55	10	93496612..93529092	8,45	15,76	-0,675607606
FFAR4	10	93566665..93604480	-	-	0,188027316
RBP4	10	complement(93591687..93601744)	0,33	0,37	0,059083318
PDE6C	10	93612537..93666010	0,05	0,02	1,833480694
FRA10AC1	10	complement(93667883..93702592)	0,35	0,42	-0,06908215
LGI1	10	93757840..93806272	0,00	-	3,068695355
SLC35G1	10	93893973..93956062	0,30	0,49	-0,493532193
PLCE1	10	93993931..94332823	1,03	1,46	-0,28636885
NOC3L	10	complement(94333226..94362959)	2,46	4,80	-0,740622701
TBC1D12	10	94402541..94536332	0,30	0,48	-0,469194701
HELLS	10	94545329..94613905	1,93	2,98	-0,403321334
CYP2C18	10	94683729..94736190	0,00	-	3,068695355
gene:ENSG000	10	94688154..94853073	-	-	0,188027316
CYP2C19	10	94762681..94855547	-	0,01	-3,804623889
CYP2C9	10	94938658..94990091	-	-	0,188027316
CYP2C8	10	complement(95036772..95069497)	-	-	0,188027316
ACSM6	10	95194200..95228929	-	-	0,188027316
PDLIM1	10	complement(95237572..95291012)	104,83	56,23	1,121709461
SORBS1	10	complement(95311771..95561414)	0,04	0,13	-1,424000763
ALDH18A1	10	complement(95605941..95656711)	49,75	32,04	0,857894621
TCTN3	10	complement(95659823..95694143)	3,93	4,04	0,182693807
ENTPD1	10	95711779..95877266	0,30	0,77	-1,122743343
gene:ENSG000	10	95860548..95924844	-	-	#N/D
CC2D2B	10	95907603..96033745	0,01	0,02	-0,204080844
CCNJ	10	96043394..96060870	0,87	1,34	-0,402742874
ZNF518A	10	96129715..96205288	0,24	0,68	-1,290788487
BLNK	10	complement(96189171..96271587)	-	-	0,188027316
DNTT	10	96304409..96338564	-	-	#N/D
OPALIN	10	complement(96343221..96359365)	-	-	#N/D
TLL2	10	complement(96364608..96513926)	0,01	0,02	-0,759769633
TM9SF3	10	complement(96518110..96587452)	7,81	8,37	0,123250313
PIK3AP1	10	complement(96593315..96720514)	0,05	0,11	-1,003644204

LCOR	10	96832254..96995956	0,86	1,43	-0,506320199
SLIT1	10	complement(96998038..97185959)	0,05	0,06	0,111413187
ARHGAP19-SLI	10	complement(97153045..97292637)	0,04	0,50	-3,393977063
ARHGAP19	10	complement(97222173..97292673)	3,49	2,84	0,520096769
FRAT1	10	97319271..97321915	0,38	0,83	-0,8859082
FRAT2	10	complement(97332497..97334729)	3,19	3,06	0,285940279
RRP12	10	complement(97356358..97426076)	10,27	8,77	0,451149438
PGAM1	10	97426191..97433444	260,96	#####	0,8964512
EXOSC1	10	complement(97435909..97446017)	6,36	6,52	0,18816508
ZDHHC16	10	97446170..97457370	13,06	8,96	0,766835921
MMS19	10	complement(97458324..97498794)	17,65	19,13	0,106969279
UBTD1	10	97498924..97571206	7,61	5,39	0,72060064
ANKRD2	10	97572499..97583884	0,01	0,03	-0,579815015
HOGA1	10	97584323..97612802	0,30	0,71	-1,049055588
gene:ENSG000	10	97584374..97673910	0,50	0,73	-0,33112605
C10orf62	10	97589721..97590934	0,06	0,22	-1,603003548
MORN4	10	complement(97614553..97633500)	3,42	4,07	-0,02811631
PI4K2A	10	97640671..97676434	15,95	13,18	0,498285242
AVPI1	10	complement(97677424..97687241)	5,72	8,59	-0,363899123
MARVELD1	10	97713173..97718150	20,17	24,94	-0,083450899
ZFYVE27	10	97737121..97760907	7,72	7,01	0,36322323
SFRP5	10	complement(97766751..97771999)	0,01	0,01	-0,025382928
GOLGA7B	10	97849843..97871580	0,12	0,34	-1,229397288
CRTAC1	10	complement(97865000..98030828)	-	0,07	-6,969094344
R3HCC1L	10	98134624..98244897	1,14	1,71	-0,364913885
LOXL4	10	complement(98247690..98268194)	0,06	0,08	-0,109163979
PYROXD2	10	complement(98383565..98415182)	0,12	1,30	-3,23260079
HPS1	10	complement(98410939..98446963)	1,84	2,48	-0,20724371
gene:ENSG000	10	complement(98414002..98446920)	-	-	0,188027316
HPSE2	10	complement(98457077..99235862)	-	-	0,188027316
CNNM1	10	99329356..99394330	0,01	-	4,913749003
GOT1	10	complement(99396870..99430624)	13,19	16,92	-0,13661758
NKX2-3	10	99532942..99536524	0,02	0,02	0,366665713
SLC25A28	10	complement(99610519..99622793)	6,72	6,71	0,223490374
gene:ENSG000	10	complement(99651595..99732112)	0,18	0,23	-0,124434919
ENTPD7	10	99659509..99711241	4,04	3,11	0,601422592
CUTC	10	99702558..99756134	6,33	7,00	0,076549588
COX15	10	complement(99710868..99732127)	7,92	7,35	0,329793544
ABCC2	10	99782640..99852594	0,57	0,96	-0,529476416
DNMBP	10	complement(99875577..100009947	5,82	5,32	0,352016609
CPN1	10	complement(100042193..10008186	0,01	-	3,068695355
ERLIN1	10	complement(100150094..10018603	11,41	14,89	-0,160695402
CHUK	10	complement(100188300..10022959	2,01	2,48	-0,076869122
CWF19L1	10	complement(100232298..10026768	6,51	7,14	0,090111716
BLOC1S2	10	complement(100273280..10028668	1,28	2,45	-0,713412967
PKD2L1	10	complement(100288149..10033026	-	-	0,188027316
SCD	10	100347233..100364826	66,53	34,77	1,159309379
WNT8B	10	100463009..100483744	0,05	0,04	0,686693064

SEC31B	10	complement(100486646..10051986	2,17	2,04	0,314973083
gene:ENSG000	10	complement(100505628..10052988	0,69	0,76	0,09147195
NDUFB8	10	complement(100523740..10053000	18,61	25,86	-0,251648056
HIF1AN	10	100529072..100559998	3,33	3,70	0,070504173
PAX2	10	100735396..100829944	0,02	0,03	-0,037899139
SLF2	10	100912963..100965134	2,56	3,01	-0,013235106
MRPL43	10	complement(100969458..10098751	6,41	6,87	0,124290152
SEMA4G	10	100969504..100985871	0,45	0,67	-0,348107513
TWNK	10	100987367..100994403	2,48	5,04	-0,798814337
LZTS2	10	100996618..101007836	9,39	10,69	0,035346606
PDZD7	10	complement(101007679..10103229	0,35	0,23	0,819113012
SFXN3	10	101031234..101041241	15,48	12,73	0,50483536
KAZALD1	10	101061989..101068131	3,44	4,20	-0,065841822
TLX1	10	101131300..101137789	-	0,01	-2,89252005
LBX1	10	complement(101226994..10122946	0,02	-	4,913749003
BTRC	10	101354033..101557321	3,59	3,71	0,176192305
DPCD	10	101570560..101609662	6,70	5,90	0,406976744
POLL	10	complement(101578882..10158827	3,83	5,39	-0,272368699
FBXW4	10	complement(101610664..10169529	5,45	9,35	-0,554349385
FGF8	10	complement(101770109..10178037	0,02	0,04	-0,429837071
NPM3	10	complement(101781325..10178344	10,01	14,84	-0,344516067
OGA	10	complement(101784443..10181846	8,07	9,00	0,065045551
KCNIP2	10	complement(101825974..10184392	0,47	0,55	-0,005891296
ARMH3	10	complement(101845599..10205619	3,39	5,17	-0,386620283
HPS6	10	102065349..102068036	3,88	6,28	-0,47066204
LDB1	10	complement(102106489..10212036	7,91	8,50	0,120157856
PPRC1	10	102132994..102150333	14,03	16,98	-0,051975691
NOLC1	10	102152176..102163871	19,65	25,70	-0,163895687
ELOVL3	10	102226299..102229589	0,13	0,08	0,868595576
PITX3	10	complement(102230189..10224151	0,07	0,04	1,106450337
GBF1	10	102245371..102382899	27,62	17,36	0,892846038
NFKB2	10	102394110..102402524	10,37	16,45	-0,442059268
PSD	10	complement(102402617..10242153	0,12	0,15	-0,04020626
FBXL15	10	102419189..102423136	1,05	1,14	0,09935726
CUEDC2	10	complement(102423245..10243258	25,23	32,71	-0,151418269
C10orf95	10	complement(102449837..10245154	0,17	0,43	-1,126151098
MFSD13A	10	102461395..102477045	0,86	1,29	-0,355809297
ACTR1A	10	complement(102461881..10250271	30,96	32,71	0,143918554
SUFU	10	102503972..102633535	3,83	4,20	0,089553068
TRIM8	10	102642503..102658318	7,98	12,49	-0,423264117
ARL3	10	complement(102673731..10271439	3,66	3,09	0,469073071
SFXN2	10	102714538..102743492	0,19	0,27	-0,265744555
WBP1L	10	102743948..102834516	2,13	2,44	0,023973455
CYP17A1	10	complement(102830531..10283747	-	0,01	-2,89252005
BORCS7	10	102854259..102864961	0,57	1,13	-0,770089458
BORCS7-ASMT	10	102854272..102901899	0,03	0,04	-0,392761763
AS3MT	10	102869470..102901899	-	-	0,188027316
CNNM2	10	102918294..103090222	0,50	0,65	-0,148700328

NT5C2	10	complement(103087185..10327760	2,48	3,70	-0,352506829
RPEL1	10	103245887..103248016	-	0,01	-2,89252005
INA	10	103277138..103290346	-	-	0,188027316
PCGF6	10	complement(103302796..10335114	0,47	1,31	-1,249432603
TAF5	10	103367966..103389065	2,04	2,28	0,064991927
ATP5MK	10	complement(103389041..10339649	18,25	21,80	-0,033463872
PDCD11	10	103396626..103446294	12,53	13,67	0,097591053
CALHM2	10	complement(103446786..10345240	1,03	0,68	0,82517372
CALHM1	10	complement(103453240..10345890	0,04	0,08	-0,695225492
CALHM3	10	complement(103472804..10347924	0,16	0,20	-0,095847935
NEURL1	10	103493705..103592546	0,02	0,06	-1,438989101
SH3PXD2A	10	complement(103594027..10385558	6,49	7,49	0,015212554
STN1	10	complement(103856806..10391833	1,33	1,58	-0,026226886
SLK	10	103967140..104029233	2,68	4,11	-0,394801465
COL17A1	10	complement(104031286..10408588	0,38	0,63	-0,49524119
SFR1	10	104122058..104126385	1,09	1,66	-0,388321596
CFAP43	10	complement(104129888..10423236	0,01	0,01	-0,442604891
GSTO1	10	104235356..104267459	21,04	24,04	0,030712229
GSTO2	10	104268873..104304950	0,12	0,17	-0,278240389
ITPRIP	10	complement(104309698..10433846	5,33	7,45	-0,260912665
CFAP58	10	104353833..104455102	0,08	0,18	-0,896276369
SORCS3	10	104641290..105265242	-	-	0,188027316
SORCS1	10	complement(106573663..10716470	0,00	-	3,967268276
XPNPEP1	10	complement(109864766..10992355	14,77	16,67	0,047958637
ADD3	10	109996368..110135565	4,84	6,57	-0,216519691
MXI1	10	110207605..110287365	3,29	3,54	0,114071999
SMNDC1	10	complement(110290730..11030493	2,41	2,73	0,046932136
DUSP5	10	110497907..110511533	1,14	4,01	-1,589929985
SMC3	10	110567684..110606048	3,47	7,82	-0,949435862
RBM20	10	110644336..110839468	0,12	0,52	-1,906611097
PDCD4	10	110871795..110900006	2,46	3,69	-0,358280585
BBIP1	10	complement(110898730..11091920	0,60	1,06	-0,601905861
SHOC2	10	110919367..111017307	1,58	1,96	-0,090509867
ADRA2A	10	111077029..111080907	0,00	-	3,068695355
GPAM	10	complement(112149865..11221537	0,75	1,23	-0,505356219
TECTB	10	112283400..112305038	-	0,01	-4,758582517
ACSL5	10	112374116..112428379	0,86	2,33	-1,219307769
ZDHHC6	10	complement(112424428..11244757	2,00	3,12	-0,417446552
VTI1A	10	112446998..112818744	1,33	1,94	-0,319121809
gene:ENSG000	10	112831169..112832454	0,04	0,11	-1,151227584
TCF7L2	10	112950247..113167678	1,82	2,37	-0,159029955
HABP2	10	113550837..113589602	-	-	0,188027316
NRAP	10	complement(113588714..11366407	-	0,00	-2,89252005
CASP7	10	113679162..113730907	3,24	3,53	0,098753548
PLEKHS1	10	113751262..113783429	-	-	0,188027316
DCLRE1A	10	complement(113834725..11385438	0,99	2,45	-1,08718551
NHLRC2	10	113854661..113917194	2,73	3,54	-0,149547374
ADRB1	10	114043866..114046904	0,26	0,43	-0,508081883

CCDC186	10	complement(114120862..11417423	1,20	0,84	0,743872514
TDRD1	10	114179270..114232666	0,00	0,00	-0,579815015
VWA2	10	114239254..114294489	0,01	0,01	1,15046174
AFAP1L2	10	complement(114294824..11440475	0,62	0,34	1,086043018
gene:ENSG000	10	complement(114424445..11442739	0,01	0,00	1,436225396
ABLIM1	10	complement(114431112..11476806	5,85	4,73	0,53007736
FHIP2A	10	114821744..114899832	3,47	3,68	0,138635521
TRUB1	10	114938195..114977676	2,31	3,46	-0,357169069
ATRNL1	10	115093365..115948999	0,06	0,01	2,416243161
GFRA1	10	complement(116056925..11627680	0,00	0,16	-5,94450803
CCDC172	10	116324448..116380029	-	-	0,188027316
PNLIPRP3	10	116427847..116477957	-	0,01	-2,89252005
PNLIP	10	116545931..116567855	-	-	#N/D
PNLIPRP1	10	116590385..116609175	-	-	#N/D
PNLIPRP2	10	116620953..116645143	-	-	0,188027316
SPMIP5	10	complement(116663696..11667026	-	-	0,188027316
HSPA12A	10	complement(116671192..11685025	0,90	2,37	-1,177128482
ENO4	10	116849499..116911788	0,12	0,07	1,082824074
SHTN1	10	complement(116881477..11712658	1,24	2,25	-0,635841558
VAX1	10	complement(117128521..11713830	0,05	0,11	-0,925343986
KCNK18	10	117197489..117210299	-	-	0,188027316
SLC18A2	10	117241093..117279430	0,01	-	4,516772761
PDZD8	10	complement(117277274..11737544	3,08	3,72	-0,048162176
EMX2	10	117542445..117549546	0,30	0,21	0,725177616
RAB11FIP2	10	complement(118004916..11804694	0,95	2,02	-0,862041239
FAM204A	10	complement(118297925..11834232	0,86	0,94	0,101060544
PRLHR	10	complement(118589997..11859564	0,00	-	3,068695355
CACUL1	10	complement(118674167..11875524	4,00	3,64	0,357799835
NANOS1	10	119029714..119033730	0,27	0,44	-0,487486146
EIF3A	10	complement(119033670..11908081	11,62	18,07	-0,413681442
DENND10	10	119104086..119137984	2,32	4,83	-0,835318682
SFXN4	10	complement(119140767..11916571	2,01	4,68	-0,99894279
PRDX3	10	complement(119167720..11917881	24,52	37,49	-0,389339326
GRK5	10	119207571..119459745	4,08	2,91	0,710885484
RGS10	10	complement(119499817..11954271	9,54	15,32	-0,46128019
TIAL1	10	complement(119571802..11959702	7,10	9,01	-0,120007996
BAG3	10	119651380..119677819	18,40	17,80	0,270962334
INPP5F	10	119726042..119829147	1,12	1,74	-0,420733778
MCMBP	10	complement(119829404..11989255	11,21	15,71	-0,264518587
SEC23IP	10	119892707..119944657	7,51	6,10	0,52338803
PLPP4	10	120457227..120592065	0,23	0,69	-1,360796335
WDR11	10	120851305..120909524	4,09	5,99	-0,326216329
FGFR2	10	complement(121478332..12159845	0,04	0,00	3,602286742
ATE1	10	complement(121709393..12192880	1,18	2,38	-0,782358655
NSMCE4A	10	complement(121957091..12197521	3,86	7,44	-0,724850277
TACC2	10	121989163..122254545	0,92	2,05	-0,936187001
BTBD16	10	122271296..122338159	-	-	0,188027316
PLEKHA1	10	122374696..122432354	1,92	2,88	-0,36128517

ARMS2	10	122454653..122457352	0,03	0,06	-0,979341556
HTRA1	10	122458551..122514907	16,37	7,84	1,285622678
DMBT1	10	122560665..122643736	0,37	0,82	-0,926270099
SPADH	10	122672850..122679494	-	-	#N/D
C10orf120	10	complement(122697709..12269984	-	-	#N/D
CUZD1	10	complement(122832158..12284617	0,03	0,02	0,533811434
gene:ENSG000	10	complement(122832167..12287958	0,08	0,17	-0,757909027
FAM24B	10	complement(122849078..12287964	3,47	3,43	0,24132042
FAM24A	10	122910610..122913111	-	-	0,188027316
C10orf88	10	complement(122930901..12295431	1,81	1,59	0,411861573
PSTK	10	122954381..122997513	0,43	0,65	-0,37860259
IKZF5	10	complement(122990807..12300881	1,68	1,47	0,412521982
ACADSB	10	123008979..123058290	0,59	1,04	-0,590675911
HMX3	10	123135970..123139423	1,69	2,88	-0,544888997
HMX2	10	123148136..123150672	0,03	0,01	1,833201638
BUB3	10	123154402..123170467	11,14	14,26	-0,133581699
GPR26	10	123666355..123697399	-	0,02	-6,630750384
CPXM2	10	complement(123706207..12394026	0,62	3,30	-2,198382835
CHST15	10	complement(124007668..12409359	1,09	1,96	-0,626424883
OAT	10	complement(124397303..12441897	26,47	28,72	0,105639897
NKX1-2	10	complement(124445243..12445003	0,03	0,06	-0,517568707
LHPP	10	124461823..124617888	4,29	3,73	0,42472488
gene:ENSG000	10	complement(124617080..12479172	0,25	0,47	-0,708332913
FAM53B	10	complement(124619292..12474437	3,51	5,07	-0,308290467
EEF1AKMT2	10	complement(124748149..12479188	1,00	1,20	-0,046451947
ABRAXAS2	10	124801819..124836667	3,25	4,30	-0,178035431
ZRANB1	10	124942123..124988189	7,29	7,02	0,278140897
CTBP2	10	complement(124984317..12516117	5,55	5,67	0,19089402
TEX36	10	complement(125576522..12568316	-	0,09	-5,902279478
EDRF1	10	125719515..125764143	2,22	2,86	-0,142779645
MMP21	10	complement(125753580..12577582	0,02	0,06	-1,51742441
UROS	10	complement(125784980..12582328	1,95	3,08	-0,434570411
BCCIP	10	125823546..125853695	4,49	6,27	-0,258059409
DHX32	10	complement(125836337..12589643	3,65	5,75	-0,431226949
FANK1	10	125896564..126009592	0,17	0,15	0,423394911
ADAM12	10	complement(126012381..12638847	1,77	3,49	-0,758529046
C10orf90	10	complement(126424997..12679870	0,22	0,27	-0,070860551
DOCK1	10	126905409..127452517	7,37	10,48	-0,286299354
INSYN2A	10	complement(127135426..12719659	0,14	0,16	0,051063317
NPS	10	127549309..127553540	-	-	#N/D
gene:ENSG000	10	complement(127734562..12773699	-	-	#N/D
FOXI2	10	127737185..127741183	-	-	0,188027316
CLRN3	10	complement(127877841..12789294	-	-	0,188027316
PTPRE	10	127907103..128085855	0,18	1,24	-2,557179697
MKI67	10	complement(128096659..12812642	19,10	22,02	0,017486154
MGMT	10	129467190..129770983	1,13	0,77	0,780886006
EBF3	10	complement(129835233..12997305	0,62	1,36	-0,903220573
C10orf143	10	complement(130020025..13011083	0,05	0,04	0,341596824

GLRX3	10	130136391..130184521	5,93	8,33	-0,266805007
TCERG1L	10	complement(131092391..13131172	-	-	#N/D
gene:ENSG000	10	complement(131831910..13183350	-	-	#N/D
PPP2R2D	10	131901008..131959834	3,07	3,23	0,149770045
BNIP3	10	complement(131967684..13198196	23,16	11,29	1,260031607
gene:ENSG000	10	131996738..131999846	0,06	0,02	1,524737598
JAKMIP3	10	132036336..132184858	0,09	0,12	-0,167226514
DPYSL4	10	132186948..132205759	1,78	1,64	0,336258871
STK32C	10	complement(132207492..13233184	5,77	2,30	1,551823777
LRRC27	10	132332154..132381508	0,39	0,35	0,380004242
PWWP2B	10	132397168..132417859	4,32	4,60	0,132283175
INPP5A	10	132537787..132783480	4,39	3,57	0,52073099
NKX6-2	10	complement(132783179..13278614	0,04	0,02	1,255976028
CFAP46	10	complement(132808392..13294282	0,01	-	5,88622595
ADGRA1	10	133087924..133131675	-	-	0,188027316
KNDC1	10	133160219..133226412	0,34	0,21	0,96323863
UTF1	10	133230217..133231558	-	-	#N/D
VENTX	10	133237855..133241928	0,01	-	4,516772761
ADAM8	10	complement(133262420..13327686	1,00	0,93	0,320974835
TUBGCP2	10	complement(133278635..13331882	10,94	8,98	0,508045472
ZNF511	10	133308914..133313162	3,83	5,25	-0,231449244
ZNF511-PRAP1	10	133309410..133352529	-	-	#N/D
CALY	10	complement(133324072..13333693	0,06	0,01	2,787439322
PRAP1	10	133347368..133352683	0,63	0,45	0,715831587
FUOM	10	complement(133355158..13335802	5,64	5,85	0,169597291
ECHS1	10	complement(133362485..13337335	43,24	42,77	0,238981923
PAOX	10	133379261..133391694	-	0,01	-3,804623889
gene:ENSG000	10	133380017..133420271	0,08	-	6,95890237
MTG1	10	133394094..133422520	2,47	4,17	-0,533668428
SPRN	10	complement(133420666..13342462	0,30	0,46	-0,400767422
SCART1	10	133453928..133523558	0,79	0,48	0,958674903
CYP2E1	10	133520406..133561220	0,91	0,40	1,40402465
SYCE1	10	complement(133553901..13356983	0,01	0,03	-0,820024868
FRG2B	10	complement(133623895..13362679	0,01	-	3,068695355
BET1L	11	complement(167784..207399)	43,19	19,15	1,396733684
SCGB1C1	11	193078..194575	-	-	0,188027316
CIMAP1A	11	196761..200261	0,69	0,17	2,220635473
RIC8A	11	207708..215113	24,61	17,47	0,717094546
SIRT3	11	complement(215030..236931)	1,99	1,93	0,267593025
PSMD13	11	236966..252984	56,19	45,79	0,518330177
NLRP6	11	278407..285388	0,01	0,01	0,366665713
PGGHG	11	289126..296107	12,90	11,38	0,40306094
IFITM5	11	complement(298200..299526)	0,12	0,12	0,152452079
IFITM2	11	303655..309397	16,22	9,23	1,036145534
gene:ENSG000	11	308408..315272	-	-	#N/D
IFITM1	11	310041..315272	16,95	5,09	1,957211874
IFITM3	11	complement(319676..329475)	63,75	36,48	1,028323196
B4GALNT4	11	369499..382117	18,57	7,73	1,487217716

PKP3	11	392614..404908	3,28	4,21	-0,136863579
SIGIRR	11	complement(405715..417455)	3,54	2,40	0,786197308
ANO9	11	complement(417933..442011)	0,03	0,07	-0,765717531
PTDSS2	11	448268..491399	9,53	7,34	0,601333379
RNH1	11	complement(494512..507300)	42,04	29,69	0,724680079
HRAS	11	complement(532242..537321)	13,09	8,97	0,768218913
LRRC56	11	537527..554912	0,31	0,34	0,073292564
LMNTD2	11	complement(554850..560738)	1,10	1,10	0,229540649
RASSF7	11	560404..564025	5,61	2,50	1,389094228
PHRF1	11	576470..612222	14,07	9,57	0,778634626
IRF7	11	complement(612553..615983)	4,11	1,16	2,045015437
CDHR5	11	complement(616577..626078)	3,20	1,53	1,283379036
SCT	11	complement(626309..627181)	0,76	0,22	1,983427248
DRD4	11	637269..640706	1,24	0,72	1,003561417
DEAF1	11	complement(644233..707118)	6,31	5,39	0,449809305
EPS8L2	11	694438..727727	2,35	2,07	0,408776501
TMEM80	11	695591..705028	5,98	3,39	1,042665211
TALDO1	11	747415..765012	63,64	49,98	0,571642486
GATD1	11	complement(767220..777488)	18,93	11,70	0,917168454
gene:ENSG000	11	779617..780755	3,17	3,93	-0,087376315
CEND1	11	complement(787115..790113)	0,52	0,81	-0,431280861
SLC25A22	11	complement(790475..798281)	17,48	11,10	0,878875832
PANO1	11	797511..799190	0,82	0,47	1,026982301
PIDD1	11	complement(799179..809753)	8,60	5,71	0,813127923
RPLP2	11	809965..812880	272,78	#####	0,577048124
PNPLA2	11	818914..825573	28,84	19,85	0,761773293
CRACR2B	11	826144..831991	0,93	1,21	-0,162486335
CD151	11	832887..839831	117,10	74,20	0,881273782
POLR2L	11	complement(837356..842529)	34,83	22,13	0,876969382
TSPAN4	11	842812..867116	54,50	29,19	1,123806561
CHID1	11	complement(867859..915058)	16,18	10,18	0,891943681
AP2A2	11	924881..1012245	9,52	8,40	0,403669337
MUC6	11	complement(1012823..1036718)	0,08	0,12	-0,339375874
MUC2	11	1074875..1110511	0,00	0,02	-1,573534033
MUC5AC	11	1157953..1201138	0,01	0,34	-4,273481334
MUC5B	11	1223066..1262172	0,04	0,02	1,170721941
TOLLIP	11	complement(1274371..1309654)	6,62	5,35	0,530588316
BRSK2	11	1389899..1462689	3,30	5,25	-0,447009253
MOB2	11	complement(1469457..1501247)	5,80	5,15	0,395269255
DUSP8	11	complement(1554051..1572271)	2,67	1,29	1,274218712
KRTAP5-1	11	complement(1584342..1585283)	0,17	0,12	0,689783732
KRTAP5-2	11	complement(1597177..1598294)	-	-	0,188027316
KRTAP5-3	11	complement(1607565..1608463)	-	-	#N/D
KRTAP5-4	11	complement(1620958..1622138)	-	-	0,188027316
KRTAP5-5	11	1629775..1630930	-	-	#N/D
KRTAP5-6	11	1697195..1697755	-	-	#N/D
IFITM10	11	complement(1732406..1750595)	0,38	0,04	3,56070282
gene:ENSG000	11	complement(1734821..1763954)	1,09	0,21	2,578168995

CTSD	11	complement(1752752..1764573)	64,84	49,97	0,598922192
SYT8	11	1828307..1837521	-	-	0,188027316
TNNI2	11	1838981..1841680	-	-	#N/D
LSP1	11	1850904..1892267	0,54	0,64	-0,019397706
PRR33	11	complement(1888078..1891895)	0,03	0,10	-1,301789231
TNNT3	11	1919703..1938706	0,08	0,01	3,257511884
MRPL23	11	1947278..1984522	14,98	11,76	0,572166968
IGF2	11	complement(2129112..2158391)	47,52	52,59	0,076824882
gene:ENSG000	11	complement(2131395..2149603)	-	-	0,188027316
INS-IGF2	11	complement(2132538..2161209)	-	-	#N/D
INS	11	complement(2159779..2161221)	-	-	#N/D
TH	11	complement(2163929..2171815)	-	-	0,188027316
ASCL2	11	complement(2268498..2270588)	-	-	0,188027316
C11orf21	11	complement(2295628..2303049)	0,35	0,44	-0,129673764
TSPAN32	11	2301997..2318204	0,88	0,75	0,446252329
CD81	11	2376177..2397802	81,30	48,85	0,958030772
TSSC4	11	2400488..2403878	14,75	8,30	1,052439726
TRPM5	11	complement(2404515..2444514)	0,07	0,03	1,314220549
KCNQ1	11	2444654..2849105	0,05	0,00	4,247318137
CDKN1C	11	complement(2883213..2885775)	0,83	0,81	0,247484228
SLC22A18	11	2899721..2925246	2,52	4,18	-0,507307931
PHLDA2	11	complement(2928273..2929420)	14,66	12,06	0,504787243
NAP1L4	11	complement(2944431..2992377)	26,90	26,92	0,221713769
CARS1	11	complement(3000922..3057613)	25,82	29,98	0,007721599
OSBPL5	11	complement(3087107..3166739)	4,06	3,20	0,56869384
MRGPRG	11	complement(3217944..3218813)	-	-	#N/D
MRGPRE	11	complement(3225030..3232417)	-	-	#N/D
ZNF195	11	complement(3339261..3379222)	0,51	0,94	-0,64617134
ART5	11	complement(3638512..3642316)	0,01	0,04	-2,190455314
ART1	11	3645128..3664416	-	-	0,188027316
CHRNA10	11	complement(3665587..3671384)	0,02	0,08	-1,586291974
NUP98	11	complement(3671083..3797792)	13,63	14,95	0,089868291
PGAP2	11	3797724..3826371	3,45	2,80	0,526765759
RHOG	11	complement(3826978..3840959)	16,16	14,73	0,356909329
STIM1	11	3854527..4093210	3,65	4,58	-0,10320883
RRM1	11	4094707..4138932	26,03	28,21	0,106896667
SSU72L5	11	4233217..4234287	-	-	#N/D
SSU72L2	11	complement(4241641..4242706)	-	-	#N/D
SSU72L4	11	complement(4287084..4288185)	-	-	#N/D
SSU72L3	11	4329790..4330864	-	-	#N/D
SSU72L1	11	complement(4338245..4339345)	-	-	#N/D
OR52B4	11	complement(4367263..4368386)	-	-	0,188027316
TRIM21	11	complement(4384897..4393702)	1,83	2,23	-0,062533634
OR52K2	11	4449295..4450361	-	-	0,188027316
OR52K1	11	4482646..4493497	-	-	0,188027316
OR52M1	11	4545191..4546144	-	-	#N/D
OR52I2	11	4581743..4593340	0,00	0,01	-0,429837071
OR52I1	11	4594039..4595013	0,04	0,08	-0,602143793

TRIM68	11	complement(4598672..4608231)	2,04	2,59	-0,120860474
OR51D1	11	4637477..4643060	-	-	#N/D
OR51E1	11	4643420..4655488	-	-	0,188027316
OR51E2	11	complement(4680171..4697854)	0,01	-	4,913749004
OR51C1P	11	complement(4690423..4697853)	-	-	0,188027316
MMP26	11	4704784..4992431	0,05	0,10	-0,833928617
OR51F1	11	complement(4768979..4769938)	-	-	#N/D
OR52R1	11	complement(4803433..4804380)	-	-	#N/D
OR51F2	11	4821321..4822456	-	-	#N/D
OR51S1	11	complement(4848237..4849208)	-	-	#N/D
OR51T1	11	4881819..4882883	-	-	#N/D
OR51A7	11	4903783..4909462	0,01	0,04	-1,21700111
OR51G2	11	complement(4912588..4919350)	0,03	0,03	0,152452079
OR51G1	11	complement(4923374..4924339)	0,02	0,04	-0,579815015
OR51A4	11	complement(4942831..4947605)	0,00	0,01	-0,979341556
OR51A2	11	complement(4954772..4955713)	0,02	0,03	-0,025382928
OR51L1	11	4994851..5005536	0,01	0,03	-0,765717531
OR52J3	11	5046526..5047461	-	0,01	-2,89252005
OR52E2	11	complement(5058650..5059627)	-	-	#N/D
OR52E1	11	5069572..5070497	-	0,03	-3,804623889
OR52A5	11	complement(5128776..5138356)	-	-	0,188027316
OR52A1	11	complement(5146824..5154757)	-	-	0,188027316
OR51V1	11	complement(5199735..5200682)	-	-	0,188027316
gene:ENSG000	11	complement(5224309..5225461)	-	-	#N/D
HBB	11	complement(5225464..5229395)	0,01	-	3,068695355
HBD	11	complement(5232678..5243657)	-	-	#N/D
HBG1	11	complement(5248269..5249857)	0,06	-	4,516772761
gene:ENSG000	11	complement(5248270..5254783)	-	-	0,188027316
HBG2	11	complement(5253188..5505605)	0,05	0,02	1,833201638
gene:ENSG000	11	complement(5254392..5678434)	0,04	0,03	0,533811434
HBE1	11	complement(5268345..5505652)	-	0,03	-3,804623889
OR51B4	11	complement(5301014..5301946)	-	0,03	-3,804623889
OR51B5	11	complement(5303444..5505652)	-	0,03	-4,359055975
OR51B2	11	complement(5323359..5324297)	-	-	#N/D
OR51B6	11	5351508..5352446	-	-	#N/D
OR51M1	11	5383812..5393263	0,01	0,02	-0,742377908
OR51Q1	11	5422111..5423206	-	0,04	-4,359055975
OR51I1	11	complement(5440570..5441514)	0,03	0,01	1,436225396
OR51I2	11	5449323..5456518	0,01	0,00	1,436225396
OR52D1	11	5488685..5489749	0,02	0,02	-0,025382928
UBQLN3	11	complement(5507300..5509958)	0,01	0,02	-0,03031053
UBQLNL	11	complement(5514393..5518431)	0,06	0,09	-0,316223056
OR52H1	11	complement(5544489..5548540)	0,01	0,01	-0,01185201
OR52B6	11	5580877..5581884	0,05	0,01	2,144183439
TRIM6	11	5596109..5612958	1,11	2,15	-0,724846943
TRIM6-TRIM34	11	5596725..5644398	0,15	0,23	-0,422512374
TRIM34	11	5619764..5644398	1,12	1,53	-0,224441402
TRIM5	11	complement(5663195..5938619)	6,55	4,03	0,923429457

TRIM22	11	5689610..5737089	0,03	0,03	0,372224007
OR52P1	11	5726502..5727464	-	-	#N/D
OR56B1	11	5736448..5738523	-	-	#N/D
OR52N4	11	5754243..5755958	0,01	-	3,068695355
OR56B2P	11	5761153..5769080	-	-	#N/D
OR52N5	11	complement(5776165..5783355)	-	-	#N/D
OR52N1	11	complement(5786471..5791265)	-	-	0,188027316
OR52N2	11	5808924..5821483	-	-	#N/D
OR52E6	11	complement(5840928..5841952)	-	-	#N/D
OR52E8	11	complement(5856749..5857690)	-	-	#N/D
OR52E4	11	5880629..5887079	-	-	#N/D
OR52E5	11	5893208..5902730	-	-	#N/D
OR56A3	11	5938751..5951347	-	-	#N/D
OR56A5	11	complement(5967553..5968494)	-	-	#N/D
OR52L1	11	complement(5985892..5986985)	0,01	-	3,068695355
OR56A4	11	complement(5999445..6006946)	-	-	#N/D
OR56A1	11	complement(6019336..6034338)	-	-	0,188027316
OR56B4	11	6107684..6108835	-	-	0,188027316
OR52B2	11	complement(6169355..6170326)	-	-	0,188027316
OR52W1	11	6199224..6200186	0,07	0,04	0,933337975
C11orf42	11	6205557..6211135	0,89	0,36	1,525325262
FHIP1B	11	complement(6211345..6234711)	13,09	6,56	1,218668479
CNGA4	11	6234765..6244479	0,01	0,01	1,436225396
CCKBR	11	6259806..6272127	0,00	-	3,068695355
CAVIN3	11	complement(6318946..6320532)	11,03	13,68	-0,086500834
SMPD1	11	6390440..6394998	18,82	17,43	0,333825494
APBB1	11	complement(6395125..6419414)	1,05	0,85	0,52160757
HPX	11	complement(6431049..6442617)	0,07	0,05	0,686693064
TRIM3	11	complement(6448613..6474459)	3,85	3,63	0,307996016
ARFIP2	11	complement(6474683..6481479)	26,64	14,56	1,095200672
gene:ENSG000	11	6481485..6508978	0,32	0,25	0,569453243
TIMM10B	11	6481501..6484681	13,84	7,56	1,094228459
DNHD1	11	6497260..6593758	2,91	1,62	1,07113677
RRP8	11	complement(6595072..6603616)	4,54	4,27	0,308430947
ILK	11	6603708..6610874	41,36	36,10	0,41896102
TAF10	11	complement(6606294..6612539)	6,81	7,22	0,138263829
TPP1	11	complement(6612768..6619448)	7,40	4,96	0,800364804
DCHS1	11	complement(6621330..6655809)	0,72	0,30	1,46318625
MRPL17	11	complement(6680385..6683340)	19,97	15,96	0,546431595
OR2AG2	11	complement(6765626..6771976)	-	0,00	-2,89252005
OR2AG1	11	6783020..6791558	0,01	0,01	-0,345401666
OR6A2	11	complement(6791736..6799689)	0,01	0,00	1,833201638
OR10A5	11	6845683..6846636	0,01	-	3,068695355
OR10A2	11	6863057..6874717	0,03	0,01	1,165729824
OR10A4	11	6876625..6877619	0,01	0,04	-1,478387936
OR2D2	11	complement(6891574..6892500)	0,05	0,07	-0,345401666
OR2D3	11	6921002..6921994	-	-	0,188027316
ZNF215	11	6926404..7001004	0,80	0,65	0,511528578

ZNF214	11	complement(6997085..7020346)	0,07	0,05	0,752292358
NLRP14	11	7020479..7071526	0,14	0,12	0,45565389
RBMXL2	11	7088998..7091148	0,01	0,01	-0,025382928
SYT9	11	7238778..7469043	0,02	0,00	2,805678585
OLFML1	11	7485388..7511377	0,04	0,04	0,371589373
PPFIBP2	11	7513298..7657127	0,40	0,56	-0,26389805
CYB5R2	11	complement(7665100..7677222)	2,83	8,92	-1,434525344
OVCH2	11	complement(7689437..7706477)	-	0,01	-2,89252005
OR5P2	11	complement(7795974..7796942)	-	-	0,188027316
OR5P3	11	complement(7824818..7830840)	-	-	0,188027316
OR10A6	11	complement(7924592..7931268)	-	-	0,188027316
OR10A3	11	complement(7937171..7941708)	-	0,01	-3,804623889
NLRP10	11	complement(7957537..7965447)	-	0,02	-5,734998842
EIF3F	11	7970251..8001862	17,07	17,46	0,189789476
TUB	11	8019244..8106243	17,06	10,02	0,990564737
gene:ENSG000	11	complement(8075573..8077781)	0,00	-	3,068695355
RIC3	11	complement(8106056..8169055)	0,06	0,08	-0,004568323
LMO1	11	complement(8224309..8268716)	0,12	0,17	-0,341646209
STK33	11	complement(8391868..8594289)	-	0,00	-2,89252005
TRIM66	11	complement(8612037..8682694)	1,91	1,87	0,258243459
RPL27A	11	8682788..8714759	25,96	29,83	0,022501823
DENND2B	11	complement(8693351..8910951)	2,52	5,05	-0,782144948
AKIP1	11	8911139..8920084	7,65	9,84	-0,140803028
C11orf16	11	complement(8920076..8933006)	0,07	0,13	-0,724610103
ASCL3	11	complement(8937578..8943016)	0,02	-	3,068695355
TMEM9B	11	complement(8947202..8965011)	4,21	4,87	0,011123413
NRIP3	11	complement(8965682..9005288)	12,83	15,77	-0,075333416
SCUBE2	11	complement(9019476..9138114)	0,03	0,06	-0,855058638
DENND5A	11	complement(9138825..9265959)	17,55	21,68	-0,081829352
TMEM41B	11	complement(9280654..9314636)	6,57	3,87	0,986461118
IPO7	11	9384652..9448127	21,43	32,77	-0,389740449
ZNF143	11	9460319..9528524	4,73	3,61	0,609977376
WEE1	11	9573670..9593457	4,66	4,95	0,135971002
SWAP70	11	9664077..9752993	6,66	7,12	0,127213538
SBF2	11	complement(9776776..10304877)	5,96	6,56	0,084438974
ADM	11	10305073..10307397	40,12	14,25	1,716187662
AMPD3	11	10308313..10507579	3,13	4,56	-0,321669044
RNF141	11	complement(10511673..10541230)	2,97	3,14	0,14234386
LYVE1	11	complement(10556966..10611689)	0,04	0,02	1,165729824
IRAG1	11	complement(10573091..10693988)	0,06	0,03	1,470916297
CTR9	11	10751018..10801625	4,26	4,95	0,005708986
EIF4G2	11	complement(10797050..10808940)	88,99	#####	-0,387873898
ZBED5	11	complement(10812074..10858796)	2,66	3,83	-0,303851874
GALNT18	11	complement(11270877..11622005)	6,51	6,82	0,156273606
CSNK2A3	11	complement(11351942..11353250)	0,14	0,20	-0,271714382
gene:ENSG000	11	complement(11725332..11726702)	-	-	0,188027316
USP47	11	11841423..11961887	3,29	5,49	-0,518032978
DKK3	11	complement(11956207..12009769)	19,70	20,95	0,134623304

MICAL2	11	12094008..12359144	9,90	11,83	-0,034304108
PARVA	11	12377563..12535356	6,63	10,74	-0,47348616
TEAD1	11	12674421..12944737	15,78	20,01	-0,119658468
RASSF10	11	13009316..13012119	0,25	1,25	-2,075921157
BMAL1	11	13276652..13387266	3,30	3,60	0,096756343
BTBD10	11	complement(13388008..13463297)	2,91	5,49	-0,691104749
PTH	11	complement(13492054..13496181)	-	-	#N/D
FAR1	11	13668659..13732346	2,33	4,07	-0,584295103
SPON1	11	13962723..14268133	0,07	0,16	-0,9040813
RRAS2	11	complement(14277922..14364506)	6,10	5,80	0,296608891
COPB1	11	complement(14443440..14500027)	37,40	34,43	0,34212412
gene:ENSG000	11	complement(14493783..14520344)	0,06	0,13	-0,802495947
PSMA1	11	complement(14504874..14643635)	30,03	46,24	-0,399885627
PDE3B	11	14643804..14872044	0,01	0,01	-0,03031053
CYP2R1	11	complement(14877440..14892231)	0,47	1,08	-0,975598807
CALCB	11	14904997..15082342	-	0,02	-5,327865481
CALCA	11	complement(14966622..14972354)	-	-	0,188027316
INSC	11	15112424..15247208	0,64	0,56	0,41459577
SOX6	11	complement(15966449..16739591)	0,01	0,00	3,49539111
C11orf58	11	16613132..16758340	4,46	7,78	-0,580860535
PLEKHA7	11	complement(16777297..17014415)	0,01	0,19	-4,832472419
RPS13	11	complement(17074388..17077715)	207,73	#####	-0,108128329
PIK3C2A	11	complement(17077730..17207986)	2,30	3,17	-0,242650729
NUCB2	11	17208153..17349980	2,36	2,98	-0,112660978
NCR3LG1	11	17351800..17377341	0,17	1,87	-3,227696343
KCNJ11	11	complement(17365172..17389331)	0,01	0,02	-1,21700111
ABCC8	11	complement(17392498..17476894)	0,00	-	4,516772761
USH1C	11	complement(17493895..17544416)	-	-	0,188027316
OTOG	11	17547259..17647150	0,00	-	4,516772761
MYOD1	11	17719571..17722136	-	-	#N/D
KCNC1	11	17734774..17856804	0,01	0,02	-1,669701709
SERGEF	11	complement(17788048..18013047)	2,28	2,22	0,260265501
TPH1	11	complement(18017555..18046269)	0,00	0,01	-2,447197442
SAAL1	11	complement(18069935..18106087)	5,99	7,31	-0,063561901
MRGPRX3	11	18120955..18138488	-	0,01	-2,89252005
MRGPRX4	11	18172837..18174280	-	-	0,188027316
SAA4	11	complement(18231355..18236802)	-	0,12	-5,327865481
SAA2-SAA4	11	complement(18231423..18248635)	-	-	0,188027316
SAA2	11	complement(18239223..18248668)	0,01	0,29	-4,029996949
SAA1	11	18266260..18269977	0,02	1,82	-6,152248244
HPS5	11	complement(18278668..18322198)	3,29	3,82	0,005714185
GTF2H1	11	18322295..18367045	2,35	3,67	-0,419423773
LDHA	11	18394560..18408425	220,40	97,07	1,406105363
LDHC	11	18412318..18452063	-	-	0,188027316
LDHAL6A	11	18455824..18479601	0,04	0,03	0,537167373
TSG101	11	complement(18468336..18526951)	6,48	7,94	-0,069508677
UEVLD	11	complement(18529609..18588747)	1,53	2,11	-0,235612267
MISFA	11	18588781..18610255	0,09	0,19	-0,841031413

gene:ENSG000	11	18601882..18602649	0,04	0,19	-1,859185627
SPTY2D1	11	complement(18606403..18634791)	14,82	5,49	1,655201327
TMEM86A	11	18693122..18704785	0,19	0,27	-0,310479774
IGSF22	11	complement(18704312..18726230)	0,00	-	3,068695355
PTPN5	11	complement(18727928..18792721)	-	-	0,188027316
MRGPRX1	11	complement(18933499..18939414)	-	-	#N/D
MRGPRX2	11	complement(19054455..19060717)	-	-	#N/D
ZDHHC13	11	19117099..19176422	2,35	4,17	-0,603722281
CSRP3	11	complement(19182030..19210571)	-	-	0,188027316
E2F8	11	complement(19224063..19241620)	1,02	2,54	-1,088206227
NAV2	11	19350724..20121601	10,79	13,56	-0,106521192
gene:ENSG000	11	20131730..20135465	0,01	0,02	-1,291882393
DBX1	11	complement(20156155..20160475)	-	-	#N/D
HTATIP2	11	20363685..20383782	0,02	-	5,224730805
PRMT3	11	20387558..20509338	1,81	4,85	-1,194030348
SLC6A5	11	20599594..20659285	-	-	#N/D
NELL1	11	20669551..21575686	-	-	#N/D
ANO5	11	21782659..22283567	-	-	0,188027316
SLC17A6	11	22338381..22379503	-	-	#N/D
FANCF	11	complement(22622533..22625823)	1,43	1,91	-0,197730047
GAS2	11	22625509..22813001	0,01	0,01	0,921097799
SVIP	11	complement(22813799..22830299)	0,41	0,52	-0,123086496
CCDC179	11	complement(22846922..22860474)	-	-	0,188027316
LUZP2	11	24496970..25082638	-	0,01	-4,359055975
ANO3	11	26188842..26663289	-	-	0,188027316
MUC15	11	complement(26559032..26572263)	-	-	0,188027316
SLC5A12	11	complement(26667020..26723427)	-	-	0,188027316
FIBIN	11	26994112..26997087	0,62	0,47	0,635021074
BBOX1	11	27040725..27127809	0,00	0,01	-0,01185201
CCDC34	11	complement(27330827..27363234)	1,53	2,25	-0,340927269
LGR4	11	complement(27365961..27472790)	2,06	2,80	-0,220636938
LIN7C	11	complement(27494418..27506769)	3,92	4,01	0,190549233
BDNF	11	complement(27654893..27722058)	1,60	1,95	-0,06305571
KIF18A	11	complement(28020619..28108156)	4,36	7,05	-0,469367932
METTL15	11	28108248..28527041	1,77	2,74	-0,409035042
KCNA4	11	complement(30009730..30017030)	-	-	#N/D
FSHB	11	30231014..30235261	-	-	0,188027316
ARL14EP	11	30323104..30338223	0,91	1,15	-0,117405945
MPPED2	11	complement(30384493..30586872)	0,00	-	3,068695355
DCDC1	11	complement(30830369..31369810)	0,01	-	5,697545031
DNAJC24	11	31369840..31432835	1,17	0,93	0,55757122
IMMP1L	11	complement(31432401..31509645)	0,60	0,82	-0,238659261
ELP4	11	31509755..31790324	0,27	0,32	-0,05591561
PAX6	11	complement(31784779..31817961)	0,94	0,82	0,427212977
gene:ENSG000	11	31812391..32104665	3,80	7,86	-0,823481587
RCN1	11	32091074..32105722	11,79	14,88	-0,112390541
WT1	11	complement(32387775..32435564)	-	-	0,188027316
EIF3M	11	32583798..32606264	6,42	7,19	0,060229937

CCDC73	11	complement(32602721..32794662)	0,00	0,01	-2,447197442
PRRG4	11	32829927..32858120	0,12	0,11	0,373668125
QSER1	11	32892811..32993316	5,34	7,83	-0,328414167
DEPDC7	11	33015876..33033582	0,49	0,73	-0,341560386
TCP11L1	11	33039417..33105943	1,67	3,35	-0,782010686
CSTF3	11	complement(33077188..33162371)	2,45	4,38	-0,615500083
HIPK3	11	33256672..33357023	5,15	3,20	0,911144241
KIAA1549L	11	33376108..33674102	1,31	1,86	-0,278813927
C11orf91	11	complement(33698261..33700826)	0,21	0,34	-0,451418876
gene:ENSG000	11	complement(33698261..33722467)	5,06	1,58	1,904107721
CD59	11	complement(33703010..33736479)	48,39	17,87	1,659928093
FBXO3	11	complement(33740939..33774543)	1,53	1,13	0,662516517
LMO2	11	complement(33858576..33892076)	0,15	0,07	1,409373041
CAPRIN1	11	34051731..34102610	33,04	26,33	0,550671306
NAT10	11	34105617..34147670	13,39	13,47	0,214684297
ABTB2	11	complement(34150987..34358010)	3,07	6,90	-0,946236494
CAT	11	34438934..34472060	30,23	22,76	0,632529233
ELF5	11	complement(34478791..34525193)	-	0,00	-2,89252005
EHF	11	34621093..34663288	0,01	0,66	-6,709309662
APIP	11	complement(34853094..34916379)	2,65	2,53	0,287517823
PDHX	11	34915829..35020591	16,02	12,09	0,629407317
CD44	11	35138882..35232402	178,44	98,51	1,080083702
SLC1A2	11	complement(35251205..35420063)	0,01	0,02	-0,868999748
PAMR1	11	complement(35431823..35530300)	0,04	0,09	-0,770531843
FJX1	11	35618460..35620865	3,45	10,08	-1,323112417
TRIM44	11	35662775..35818007	7,19	6,37	0,397271699
LDLRAD3	11	35943981..36232136	2,57	5,15	-0,779241028
gene:ENSG000	11	36196248..36197901	0,05	0,20	-1,720683953
COMMD9	11	complement(36269284..36289449)	4,01	2,31	1,01882906
PRR5L	11	36296288..36465204	0,41	1,12	-1,222995488
TRAF6	11	complement(36483769..36510272)	1,66	0,92	1,071308878
gene:ENSG000	11	36510361..36510537	0,12	-	3,967268276
RAG1	11	36510372..36593156	0,06	0,02	2,130930628
RAG2	11	complement(36575574..36598279)	0,01	-	4,516772761
IFTAP	11	36594369..36675695	0,11	0,04	1,769323604
gene:ENSG000	11	complement(38013510..38014141)	-	-	#N/D
gene:ENSG000	11	complement(40107244..40112599)	0,00	-	3,068695355
LRRC4C	11	complement(40114203..41459773)	-	-	0,188027316
API5	11	43311963..43344529	13,48	13,50	0,220218292
TTC17	11	43358920..43494933	7,29	8,88	-0,06113271
HSD17B12	11	43680680..43856617	8,26	8,39	0,20027471
ALKBH3	11	43880811..43920274	4,76	4,72	0,234312231
C11orf96	11	43942637..43943878	0,03	-	4,516772761
ACCSL	11	44047981..44059977	-	-	0,188027316
ACCS	11	44065925..44084237	0,67	1,04	-0,40880502
EXT2	11	44095648..44251962	12,50	9,81	0,572446708
ALX4	11	complement(44260440..44310139)	0,94	0,76	0,531057618
CD82	11	44564427..44620358	18,03	8,05	1,387138003

TSPAN18	11	44726465..44932423	0,87	0,89	0,191867363
TP53I11	11	complement(44885903..44951306)	11,77	5,42	1,342103888
PRDM11	11	45095806..45235110	0,36	0,34	0,290287383
SYT13	11	complement(45240302..45286341)	0,05	0,00	4,37199628
CHST1	11	complement(45647689..45665622)	0,88	0,01	6,225153323
SLC35C1	11	45804072..45813016	6,27	5,41	0,435591927
CRY2	11	45847118..45883248	7,99	3,06	1,608659666
MAPK8IP1	11	45885651..45906465	2,18	2,89	-0,182634213
FREY1	11	complement(45906513..45907272)	0,03	0,04	-0,01185201
PEX16	11	complement(45909663..45918812)	4,23	2,96	0,738848024
LARGE2	11	45921621..45929096	0,02	0,01	0,921097799
PHF21A	11	complement(45929319..46121454)	6,89	4,97	0,69433744
CREB3L1	11	46277662..46321409	22,18	21,89	0,242023251
DGKZ	11	46332905..46380554	7,75	6,50	0,475975063
MDK	11	46380756..46383837	1,07	1,27	-0,020658039
CHRM4	11	complement(46383789..46391776)	0,10	0,23	-0,933571741
AMBRA1	11	complement(46396414..46594125)	7,31	5,67	0,590468214
HARBI1	11	complement(46602861..46617909)	0,97	0,84	0,433543152
ATG13	11	46617527..46674818	17,33	10,41	0,95809513
ARHGAP1	11	complement(46677080..46700619)	36,99	15,62	1,46675307
ZNF408	11	46701030..46705912	6,43	3,40	1,140326118
F2	11	46719196..46739506	0,02	0,02	-0,429837071
CKAP5	11	complement(46743048..46846308)	44,44	41,61	0,317751645
LRP4	11	complement(46856717..46918642)	1,12	0,74	0,820854983
CSTPP1	11	46936689..47164385	4,92	3,19	0,850573873
ARFGAP2	11	complement(47164299..47177125)	10,18	8,48	0,487210685
PACSIN3	11	complement(47177522..47186443)	11,97	12,10	0,208164062
DDB2	11	47214465..47239217	8,55	5,91	0,755262849
ACP2	11	complement(47239302..47248906)	2,69	3,21	-0,030251671
NR1H3	11	47248300..47269033	1,41	1,05	0,654888812
MADD	11	47269161..47330031	10,29	7,30	0,717548795
MYBPC3	11	complement(47331406..47352702)	-	0,00	-2,89252005
SPI1	11	complement(47354860..47409369)	0,12	0,10	0,574274934
SLC39A13	11	47407132..47416496	17,80	9,00	1,207276164
PSMC3	11	complement(47418769..47426473)	53,08	40,62	0,609027372
RAPSN	11	complement(47437764..47449143)	0,01	0,01	-0,01185201
CELF1	11	complement(47465933..47565569)	16,09	14,55	0,368167095
NDUFS3	11	47565336..47584562	18,51	13,21	0,709942015
PTPMT1	11	47565430..47573461	5,62	5,81	0,175466026
KBTBD4	11	complement(47572197..47578976)	4,57	2,44	1,126808563
FAM180B	11	47586678..47589194	0,02	0,01	0,886720911
C1QTNF4	11	complement(47589667..47594411)	0,04	-	5,224730805
MTCH2	11	complement(47617315..47642607)	20,67	17,99	0,423490232
AGBL2	11	complement(47659591..47715389)	0,05	0,06	0,030621608
FNBP4	11	complement(47716494..47767443)	15,11	11,67	0,595013103
NUP160	11	complement(47778087..47848555)	11,90	10,34	0,426584899
PTPRJ	11	47980425..48170839	17,37	21,56	-0,088619809
OR4B1	11	48216810..48217739	-	-	0,188027316

OR4X2	11	48245104..48246015	-	-	#N/D
OR4X1	11	48263861..48264778	-	-	#N/D
OR4S1	11	48306223..48307152	-	-	#N/D
OR4C3	11	48324920..48325955	-	-	0,188027316
OR4C5	11	complement(48365485..48366465)	-	-	#N/D
OR4A47	11	48488793..48489722	-	-	#N/D
TRIM51G	11	complement(48975498..48983885)	-	-	#N/D
TRIM49B	11	49027501..49038451	-	-	#N/D
TRIM64C	11	complement(49053714..49059112)	-	-	0,188027316
FOLH1	11	complement(49145092..49208638)	-	-	#N/D
OR4C13	11	49952391..49953419	-	-	#N/D
OR4C12	11	complement(49981473..49982535)	-	-	#N/D
OR4C45	11	50032874..50033794	-	-	#N/D
OR4C46	11	complement(54603069..54603998)	-	-	#N/D
OR4A8	11	54682877..54683821	-	-	#N/D
OR4A5	11	54706832..54707902	-	-	#N/D
TRIM48	11	55262155..55271114	0,01	-	3,068695355
OR4A16	11	55343201..55344187	-	-	#N/D
OR4A15	11	55367974..55368918	-	-	#N/D
OR4C15	11	55554307..55555419	-	-	#N/D
OR4C16	11	55572128..55573060	-	-	#N/D
OR4C11	11	complement(55602360..55607645)	-	-	#N/D
OR4P4	11	55635113..55640309	-	-	#N/D
OR4S2	11	55648327..55652854	-	-	#N/D
OR4C6	11	55662201..55666195	-	-	#N/D
OR5D3P	11	55723776..55729621	-	-	#N/D
OR5D13	11	55773438..55774382	-	-	#N/D
OR5D14	11	55795556..55796500	-	-	#N/D
OR5L1	11	55811367..55812476	-	-	#N/D
OR5D18	11	55819630..55820571	-	-	#N/D
OR5L2	11	55827219..55828154	-	-	#N/D
OR5D16	11	55838752..55839738	-	-	#N/D
TRIM51	11	55883297..55891810	-	-	0,188027316
OR5W2	11	complement(55913650..55914582)	-	-	#N/D
OR5I1	11	complement(55935456..55936400)	-	-	#N/D
OR10AG1	11	complement(55965755..55969945)	-	-	#N/D
OR5F1	11	complement(55993681..55994625)	-	-	#N/D
OR5AS1	11	56027654..56038191	-	-	0,188027316
OR8I2	11	56093308..56094240	-	-	#N/D
OR8H2	11	56103687..56107658	-	-	#N/D
OR8H3	11	56122373..56123311	-	-	#N/D
OR8J3	11	complement(56134721..56140201)	0,00	-	3,068695355
OR8K5	11	complement(56159394..56160317)	-	-	#N/D
OR5J2	11	56176618..56177556	-	-	#N/D
OR8J2	11	complement(56208985..56215222)	-	-	#N/D
OR5T2	11	complement(56231282..56234255)	-	-	#N/D
OR5T3	11	56252254..56253222	-	-	#N/D
OR5T1	11	56274154..56276819	-	-	#N/D

OR8H1	11	complement(56288462..56292254)	0,00	-	3,068695355
OR8K3	11	56315144..56320639	-	-	#N/D
OR8K1	11	56346039..56346998	-	-	#N/D
OR8J1	11	56354291..56361511	-	-	#N/D
OR8U1	11	56375624..56376553	-	-	#N/D
OR5AL1	11	56412696..56413680	-	-	#N/D
OR8U3	11	complement(56417258..56418232)	-	-	#N/D
gene:ENSG000	11	complement(56459221..56473352)	-	-	0,188027316
OR5M9	11	complement(56462469..56463401)	-	-	#N/D
OR5M3	11	complement(56469274..56473352)	-	-	#N/D
OR5M8	11	complement(56490435..56491370)	-	-	#N/D
OR5M11	11	complement(56542340..56543257)	-	-	#N/D
OR5M10	11	complement(56576774..56577721)	-	-	#N/D
OR5M1	11	complement(56609236..56614874)	-	-	#N/D
OR5AP2	11	complement(56641489..56642439)	-	-	#N/D
OR5AR1	11	56663686..56664618	-	-	#N/D
OR9G1	11	56699095..56703884	-	-	0,188027316
OR9G4	11	complement(56741223..56748697)	-	-	#N/D
OR5G3	11	complement(56819573..56820516)	-	-	#N/D
OR5AK3P	11	56971050..56971979	-	-	#N/D
OR5AK2	11	56988914..56989843	-	-	#N/D
LRRC55	11	57181747..57191717	-	-	0,188027316
APLNR	11	complement(57233577..57237250)	0,01	-	4,516772761
TNKS1BP1	11	complement(57299638..57324952)	27,69	20,09	0,685926837
SSRP1	11	complement(57325986..57335892)	70,25	62,70	0,38728469
P2RX3	11	57338352..57372396	0,01	-	3,967268276
PRG3	11	complement(57376769..57381150)	-	-	0,188027316
PRG2	11	complement(57386780..57390650)	0,02	0,04	-0,742377908
gene:ENSG000	11	complement(57387365..57424059)	0,02	-	3,967268276
SLC43A3	11	complement(57406954..57427580)	12,38	24,63	-0,769390597
RTN4RL2	11	57460528..57477534	2,66	2,81	0,140350633
SLC43A1	11	complement(57484534..57515780)	2,22	1,61	0,686029082
TIMM10	11	complement(57528464..57530803)	18,33	14,62	0,549509907
SMTNL1	11	57537595..57550272	0,11	0,02	3,04797919
UBE2L6	11	complement(57551656..57568284)	12,26	11,19	0,354392067
SERPING1	11	57597387..57619171	0,11	-	8,073734042
YPEL4	11	complement(57645087..57649944)	0,86	0,84	0,251536748
CLP1	11	57648188..57661865	4,32	2,90	0,800419721
ZDHHC5	11	57667747..57701182	17,36	13,68	0,566036893
MED19	11	complement(57703710..57712221)	6,16	3,98	0,854169012
TMX2	11	57712580..57742987	12,69	8,95	0,726822423
gene:ENSG000	11	57712582..57818431	6,24	3,77	0,95190159
SELENOH	11	57741491..57743554	12,89	13,65	0,140295966
gene:ENSG000	11	57741779..57804771	0,22	0,04	2,469244963
BTBD18	11	complement(57743514..57752675)	1,10	0,44	1,555846241
CTNND1	11	57753243..57819546	18,26	10,57	1,011670249
OR9Q1	11	58023881..58181616	-	-	#N/D
OR6Q1	11	58030953..58031906	-	-	#N/D

OR9I1	11	complement(58116742..58125530)	-	-	0,188027316
OR9Q2	11	58189070..58194053	0,00	-	3,068695355
OR1S2	11	complement(58203204..58204142)	-	-	#N/D
OR1S1	11	58212720..58216084	-	-	0,188027316
OR10Q1	11	complement(58227882..58228918)	-	-	#N/D
OR10W1	11	complement(58266792..58268260)	-	-	#N/D
OR5B17	11	complement(58358124..58359069)	-	-	0,188027316
OR5B3	11	complement(58402464..58406874)	-	-	#N/D
OR5B2	11	complement(58421238..58428121)	-	-	0,188027316
OR5B12	11	complement(58438994..58442236)	0,01	0,01	-0,01185201
OR5B21	11	complement(58506807..58508105)	0,02	0,02	0,524121557
LPXN	11	complement(58526871..58578220)	13,38	10,29	0,602237871
ZFP91	11	58579063..58621550	25,21	18,58	0,663575478
ZFP91-CNTF	11	58579172..58624639	0,30	0,39	-0,144023397
CNTF	11	58622665..58625733	0,39	0,44	0,046890506
gene:ENSG000	11	58627435..58628528	0,27	0,22	0,464304065
GLYAT	11	complement(58640426..58731974)	-	0,01	-3,804623889
GLYATL2	11	complement(58834065..58904215)	0,05	0,14	-1,193454645
GLYATL1	11	58905398..59043527	0,10	0,24	-1,119553623
GLYATL1B	11	59086307..59094786	-	0,01	-2,89252005
FAM111B	11	59107185..59127412	0,50	0,66	-0,181235043
FAM111A	11	59142748..59155039	7,60	3,23	1,458444932
DTX4	11	59171430..59208588	54,20	7,82	3,015316235
MPEG1	11	complement(59208510..59212927)	0,45	0,09	2,470023597
OR5AN1	11	59358895..59371714	0,01	0,01	0,181652234
OR5A2	11	complement(59416969..59426413)	-	0,00	-2,89252005
OR5A1	11	59436469..59451380	0,00	-	3,068695355
OR4D6	11	59456961..59457905	-	-	#N/D
OR4D10	11	59473315..59479361	0,01	-	3,967268276
OR4D11	11	59503576..59504511	-	-	#N/D
OR4D9	11	59511368..59520703	0,00	-	3,967268276
OSBP	11	complement(59574398..59615774)	43,20	18,28	1,463601006
PATL1	11	complement(59636716..59669037)	16,10	14,08	0,417051519
OR10V1	11	complement(59712916..59713845)	-	-	0,188027316
STX3	11	59713456..59805882	1,10	1,51	-0,241211986
MRPL16	11	complement(59806140..59810778)	11,07	10,42	0,309259472
CBLIF	11	complement(59829273..59845499)	-	-	0,188027316
TCN1	11	complement(59852800..59866489)	-	-	0,188027316
OOSP3	11	59878782..59896483	-	-	0,188027316
OOSP1	11	59938432..59957480	-	-	#N/D
OOSP4A	11	59964033..59970343	-	-	#N/D
OOSP4B	11	59978020..60031095	-	-	#N/D
OOSP2	11	60040409..60048044	-	-	0,188027316
MS4A3	11	60056587..60071115	-	-	#N/D
MS4A2	11	60088261..60098467	-	0,00	-2,89252005
MS4A6A	11	complement(60172015..60184666)	-	-	#N/D
MS4A4A	11	60185657..60318080	-	-	0,188027316
MS4A4E	11	complement(60200270..60243137)	0,01	0,05	-1,859185627

MS4A6E	11	60327255..60396596	-	-	0,188027316
MS4A7	11	60378485..60395951	-	-	0,188027316
MS4A14	11	60378530..60417756	-	-	#N/D
MS4A5	11	60429572..60455214	-	-	#N/D
MS4A1	11	60455846..60470752	-	-	#N/D
MS4A12	11	60492778..60507430	-	-	#N/D
MS4A13	11	60515392..60542721	-	-	#N/D
MS4A8	11	60699585..60715807	-	-	#N/D
MS4A18	11	60724720..60745028	-	-	#N/D
MS4A15	11	60756867..60776733	0,02	0,05	-0,865575989
MS4A10	11	60785333..60801305	-	0,01	-2,89252005
CCDC86	11	60842113..60851081	18,39	19,76	0,119410812
PTGDR2	11	complement(60850933..60855950)	0,31	0,17	1,101122103
ZP1	11	60867542..60875693	0,30	0,32	0,155605841
PRPF19	11	complement(60890547..60906585)	29,32	34,68	-0,019276095
TMEM109	11	60914158..60923443	29,31	37,86	-0,146210391
TMEM132A	11	60924460..60937159	19,38	36,67	-0,697331117
SLC15A3	11	complement(60937060..60952653)	0,03	0,06	-0,728065629
CD6	11	60971680..61020377	0,00	-	3,068695355
CD5	11	61102489..61127852	0,17	0,06	1,658718133
VPS37C	11	complement(61130257..61161615)	9,99	9,07	0,36277419
gene:ENSG000	11	complement(61143380..61145464)	0,30	0,18	0,956365906
PGA3	11	61203307..61213098	-	0,00	-2,89252005
PGA4	11	61222347..61231694	-	-	0,188027316
PGA5	11	61241175..61251444	0,02	0,02	0,366665713
VWCE	11	complement(61258286..61295316)	0,38	0,28	0,664184608
DDB1	11	complement(61299451..61342596)	61,52	36,13	0,990989979
TKFC	11	61333220..61353295	3,78	4,10	0,106202674
CYB561A3	11	complement(61348754..61362283)	5,05	3,70	0,671408125
gene:ENSG000	11	61352244..61354477	0,14	0,39	-1,294428504
TMEM138	11	61361964..61377890	2,73	2,46	0,368819349
TMEM216	11	61392393..61398866	3,29	3,66	0,066627026
CPSF7	11	complement(61402641..61430031)	23,29	17,22	0,658823602
gene:ENSG000	11	complement(61426448..61427325)	0,42	0,28	0,795160006
gene:ENSG000	11	61429220..61485822	0,64	0,16	2,216806667
SDHAF2	11	61430042..61446839	10,94	6,35	1,007330054
SAXO4	11	61481120..61490931	2,26	0,43	2,624885185
LRRC10B	11	61508749..61511018	0,11	0,10	0,3118704
SYT7	11	complement(61513714..61581148)	2,68	1,95	0,678683585
gene:ENSG000	11	complement(61525708..61526602)	0,78	0,38	1,277108434
DAGLA	11	61680391..61747001	0,77	1,30	-0,53105313
MYRF	11	61752636..61788518	8,20	4,03	1,249014143
TMEM258	11	complement(61768501..61792802)	24,55	13,74	1,059804517
FEN1	11	61792911..61797238	31,94	16,45	1,180441199
FADS2	11	61792980..61867354	13,57	3,98	1,992813003
FADS1	11	complement(61799627..61829318)	9,06	7,44	0,508171209
FADS3	11	complement(61873519..61892051)	18,97	35,59	-0,684893151
RAB3IL1	11	complement(61897301..61920269)	2,42	5,03	-0,835135404

BEST1	11	61950063..61965515	0,52	2,26	-1,900413633
FTH1	11	complement(61959718..61967634)	181,98	#####	-1,85798688
gene:ENSG000	11	61967729..61969490	0,23	0,25	0,081543383
INCENP	11	62123998..62153169	32,08	23,83	0,65199131
SCGB1D1	11	62190216..62193539	-	-	#N/D
SCGB2A1	11	62208673..62213943	-	-	#N/D
SCGB1D2	11	62242239..62244812	-	0,03	-2,89252005
SCGB2A2	11	62270158..62273160	0,01	-	3,068695355
SCGB1D4	11	complement(62296281..62299075)	-	-	#N/D
ASRGL1	11	62337448..62393412	0,12	0,13	0,176743802
SCGB1A1	11	62405103..62423195	0,04	-	3,967268276
AHNAK	11	complement(62433542..62556235)	55,46	25,49	1,344617357
EEF1G	11	complement(62559596..62574086)	759,84	#####	0,648570589
gene:ENSG000	11	complement(62559603..62591531)	4,39	2,17	1,239207056
TUT1	11	complement(62575045..62591637)	16,05	8,99	1,059126648
MTA2	11	complement(62593214..62601865)	24,83	22,05	0,394042954
EML3	11	complement(62602218..62612775)	12,92	11,28	0,418758486
ROM1	11	62611722..62615116	1,32	0,81	0,924612873
B3GAT3	11	complement(62615296..62622154)	27,74	13,09	1,306087007
GANAB	11	complement(62624826..62646726)	151,54	#####	0,713177606
INTS5	11	complement(62646848..62653302)	6,13	5,27	0,439737719
gene:ENSG000	11	complement(62649694..62665178)	0,09	-	5,224730805
C11orf98	11	complement(62662817..62665210)	10,68	15,08	-0,274958486
LBHD1	11	complement(62662817..62672267)	1,41	1,55	0,080573327
CSKMT	11	62665309..62668496	1,98	2,75	-0,2487177
UQCC3	11	62670273..62673686	2,21	2,78	-0,106711941
UBXN1	11	complement(62676498..62679117)	25,57	18,31	0,7053735
LRRN4CL	11	complement(62686406..62689530)	0,03	0,03	0,438394362
BSCL2	11	complement(62689289..62709845)	5,02	1,96	1,581374521
HNRNPUL2-BS	11	complement(62690275..62727384)	7,96	4,89	0,925277556
GNG3	11	62707676..62709201	1,61	1,11	0,760035704
HNRNPUL2	11	complement(62712630..62727457)	12,26	7,63	0,907476674
TTC9C	11	62728069..62740293	6,40	4,66	0,681570014
ZBTB3	11	complement(62748319..62754184)	3,94	2,24	1,037889902
POLR2G	11	62761565..62766710	13,47	11,54	0,445569624
TAF6L	11	62771357..62787342	2,41	2,42	0,214734388
TMEM223	11	complement(62771629..62792006)	4,09	3,03	0,657326331
TMEM179B	11	62787402..62790400	14,21	11,49	0,529813384
NXF1	11	complement(62792123..62806302)	10,09	7,17	0,715749441
STX5	11	complement(62806860..62832051)	22,66	12,34	1,09950089
TEX54	11	complement(62832321..62832841)	0,83	0,37	1,366973488
WDR74	11	complement(62832342..62841809)	11,27	15,28	-0,215825224
SLC3A2	11	62856004..62888880	61,57	59,49	0,27263608
CHRM1	11	complement(62908679..62921807)	0,08	0,02	1,995169172
SLC22A6	11	complement(62936385..62984967)	-	-	#N/D
SLC22A8	11	complement(62989154..63015841)	-	-	#N/D
SLC22A24	11	complement(63079940..63144221)	-	-	#N/D
SLC22A25	11	complement(63158437..63243599)	-	-	0,188027316

SLC22A10	11	63268022..63311783	-	-	#N/D
SLC22A9	11	63369785..63410294	0,00	0,01	-0,01185201
PLAAT5	11	complement(63461404..63491194)	-	-	0,188027316
LGALS12	11	63506052..63516774	0,02	0,04	-1,009277154
PLAAT4	11	63536808..63546462	4,02	3,01	0,64127124
PLAAT2	11	complement(63552770..63563379)	0,03	0,04	-0,025382928
PLAAT3	11	complement(63573195..63616883)	3,83	2,68	0,739133062
ATL3	11	complement(63624087..63671921)	23,86	13,92	1,000788396
RTN3	11	63681446..63759891	19,28	19,82	0,183690814
ZFTA	11	complement(63759892..63768775)	1,21	1,13	0,321961862
SPINDOC	11	63813456..63827716	9,67	11,37	-0,011172685
MARK2	11	63838928..63911020	5,32	4,98	0,318923495
RCOR2	11	complement(63911230..63917164)	0,22	0,39	-0,575384421
NAA40	11	63938959..63957319	5,03	3,96	0,56761293
COX8A	11	63974620..63976543	265,74	#####	0,951422279
OTUB1	11	63985853..64001811	23,66	19,84	0,477476634
MACROD1	11	complement(63998558..64166113)	7,88	9,57	-0,056458238
FLRT1	11	64035931..64119173	0,39	1,72	-1,898706806
STIP1	11	64185272..64204543	67,38	62,30	0,336258303
FERMT3	11	64205926..64223896	0,33	0,18	1,087697349
TRPT1	11	complement(64223799..64226254)	9,48	6,40	0,790304165
NUDT22	11	64225941..64230686	6,47	3,50	1,111697561
DNAJC4	11	64230278..64234286	3,57	1,96	1,089274832
VEGFB	11	64234584..64239264	17,69	10,76	0,940310172
FKBP2	11	64241003..64244132	8,29	5,67	0,772036594
gene:ENSG000	11	64241095..64244132	2,31	0,81	1,743053564
PPP1R14B	11	complement(64244479..64246943)	69,69	44,50	0,870090054
PLCB3	11	64251530..64269150	13,40	9,97	0,649652758
BAD	11	complement(64269830..64284704)	14,44	10,75	0,649053968
GPR137	11	64270062..64289494	9,15	7,72	0,467528371
KCNK4	11	64291302..64300031	0,06	0,06	0,335747576
CATSPERZ	11	64300358..64304770	0,15	0,08	1,165729824
ESRRA	11	64305497..64316743	15,55	11,27	0,687068784
TRMT112	11	complement(64316460..64317559)	57,64	50,72	0,407655515
PRDX5	11	64318121..64321811	80,88	53,44	0,820801498
CCDC88B	11	64340204..64357534	0,32	0,24	0,646095554
RPS6KA4	11	64359148..64372215	10,98	10,15	0,335728148
SLC22A11	11	64555690..64572875	0,01	0,01	0,921097799
SLC22A12	11	64590641..64602353	0,00	-	3,068695355
NRXN2	11	complement(64606174..64723197)	0,75	0,02	5,700543876
RASGRP2	11	complement(64726911..64745456)	0,00	0,01	-1,478387936
PYGM	11	complement(64746389..64759974)	0,07	0,01	3,04797919
SF1	11	complement(64764606..64778786)	35,25	44,60	-0,116250387
MAP4K2	11	complement(64784918..64803241)	6,00	3,61	0,958354752
MEN1	11	complement(64803510..64811294)	9,27	9,47	0,191303718
CDC42BPG	11	complement(64823052..64844653)	0,34	0,33	0,253510432
EHD1	11	complement(64851642..64888296)	13,74	9,04	0,826966384
ATG2A	11	complement(64894546..64917209)	6,36	5,55	0,419552941

PPP2R5B	11	64917553..64934475	9,99	3,88	1,587872398
GPHA2	11	complement(64934471..64935893)	0,30	0,07	2,308148251
MAJIN	11	complement(64937517..64972108)	0,01	-	4,516772761
BATF2	11	complement(64987945..64997018)	0,16	0,21	-0,119809465
ARL2	11	65014160..65022184	87,95	70,97	0,5324471
ARL2-SNX15	11	65014182..65040570	-	0,47	-9,139535159
SNX15	11	65027439..65040572	3,96	6,21	-0,42768041
SAC3D1	11	65040901..65044828	8,12	5,27	0,847787179
NAALADL1	11	complement(65044818..65058553)	0,29	0,08	2,07824251
CDCA5	11	complement(65066300..65084164)	27,28	15,16	1,070132851
ZFPL1	11	65084210..65088398	31,28	12,86	1,505281907
TMEM262	11	complement(65084979..65089375)	0,65	0,11	2,734761562
VPS51	11	65089324..65111862	12,63	8,41	0,809020642
TM7SF2	11	65111845..65116384	2,72	1,56	1,025697464
ZNHIT2	11	complement(65116403..65117701)	3,01	2,58	0,446012229
gene:ENSG000	11	65118310..65119111	0,44	0,31	0,752292358
FAU	11	complement(65120630..65122177)	95,58	68,97	0,693726597
SYVN1	11	complement(65121780..65134532)	33,92	10,88	1,86349141
MRPL49	11	65122183..65127371	24,66	16,56	0,797324719
SPDYC	11	65170233..65173374	0,01	-	3,068695355
CAPN1	11	65180566..65212006	34,17	22,55	0,822585894
POLA2	11	65261913..65305959	8,59	7,98	0,329050783
gene:ENSG000	11	65261928..65326543	2,50	3,83	-0,390816985
CDC42EP2	11	65314866..65322417	4,16	9,61	-0,986084224
DPF2	11	65333843..65354262	11,47	14,49	-0,114123352
TIGD3	11	65354751..65357613	0,08	0,20	-1,124991381
SLC25A45	11	complement(65375192..65383701)	0,91	1,59	-0,579990333
FRMD8	11	65386621..65413525	20,04	7,76	1,591766764
SCYL1	11	65525077..65538704	38,78	30,89	0,551165505
LTBP3	11	complement(65538559..65558930)	57,51	43,56	0,623860602
gene:ENSG000	11	65561484..65561607	-	-	#N/D
ZNRD2	11	65570460..65573942	16,67	11,09	0,81147763
FAM89B	11	65572349..65574198	12,95	8,78	0,783187605
EHBP1L1	11	65576046..65592650	27,21	19,23	0,724019018
KCNK7	11	complement(65592836..65595996)	0,77	0,96	-0,090716335
MAP3K11	11	complement(65597756..65615382)	14,94	10,93	0,674198163
PCNX3	11	65615776..65637439	26,16	20,80	0,553778182
SIPA1	11	65638101..65650918	7,61	13,87	-0,643632289
RELA	11	complement(65653599..65663090)	22,08	18,41	0,485327453
KAT5	11	65711996..65719604	8,75	6,60	0,629261107
RNASEH2C	11	complement(65714005..65720818)	9,04	4,93	1,098396912
AP5B1	11	complement(65773898..65780976)	5,21	5,26	0,210654755
OVOL1	11	65787063..65797214	0,01	0,01	0,921097799
CFL1	11	complement(65823022..65862026)	210,78	#####	0,750208798
SNX32	11	65833834..65856896	0,25	0,22	0,385684634
MUS81	11	65857126..65867653	7,41	7,08	0,288742666
EFEMP2	11	complement(65866441..65873592)	28,02	12,18	1,424701961
CTSW	11	65879809..65883741	0,49	0,26	1,115262171

FIBP	11	complement(65883740..65888531)	22,26	21,23	0,291502448
CCDC85B	11	65890673..65891635	25,90	19,93	0,601304362
FOSL1	11	complement(65892049..65900573)	130,09	60,34	1,331230364
C11orf68	11	complement(65916810..65919062)	59,95	38,99	0,843836928
DRAP1	11	65919274..65921563	58,18	32,67	1,055606472
TSGA10IP	11	65945480..65959966	0,01	0,02	-1,478387936
SART1	11	65961728..65980137	15,57	10,17	0,83786648
EIF1AD	11	complement(65996545..66002176)	12,78	5,65	1,401968997
BANF1	11	66002228..66004149	71,59	59,10	0,499666725
CST6	11	66012008..66013505	0,02	0,07	-1,478387936
CATSPER1	11	complement(66016752..66026479)	1,83	0,97	1,13351207
GAL3ST3	11	complement(66040765..66049161)	1,27	0,49	1,606585524
SF3B2	11	66050729..66069308	63,70	39,32	0,919229964
PACS1	11	66070272..66244744	13,35	10,59	0,557535513
KLC2	11	66257294..66267860	19,84	19,93	0,216255241
RAB1B	11	66268590..66277492	132,06	65,11	1,243354219
CNIH2	11	66278175..66285301	2,05	0,69	1,784888996
YIF1A	11	complement(66284572..66289184)	47,25	21,84	1,336516718
TMEM151A	11	66291894..66296664	0,11	0,05	1,531118931
CD248	11	complement(66314494..66317044)	0,03	0,05	-0,35396495
RIN1	11	complement(66330241..66336840)	7,03	5,28	0,636006172
BRMS1	11	complement(66337333..66345125)	11,42	9,57	0,47821771
B4GAT1	11	complement(66345374..66347629)	12,48	10,01	0,540950604
SLC29A2	11	complement(66362521..66372214)	0,05	0,08	-0,335149681
NPAS4	11	66421035..66426707	0,00	-	3,068695355
MRPL11	11	complement(66435075..66466738)	15,53	14,41	0,330560381
PELI3	11	66466327..66477337	3,16	2,05	0,847971411
DPP3	11	66480013..66509657	17,13	14,24	0,49028765
gene:ENSG000	11	66509079..66533613	0,52	0,14	2,116832459
BBS1	11	66510606..66533613	2,11	1,63	0,591644471
ZDHHC24	11	complement(66520637..66546235)	5,61	2,57	1,348605852
ACTN3	11	66546395..66563334	0,03	-	6,20258586
CTSF	11	complement(66563464..66568879)	4,44	-	13,11809573
CCDC87	11	complement(66590176..66593063)	0,37	0,13	1,677438238
CCS	11	66593153..66606019	4,53	3,13	0,75647423
RBM14	11	66616626..66629934	13,66	12,79	0,317079926
RBM14-RBM4	11	66616626..66646469	2,08	1,19	1,020409857
RBM4	11	66638667..66668374	10,78	8,26	0,606953145
RBM4B	11	complement(66664998..66677887)	3,37	1,75	1,1665865
SPTBN2	11	complement(66682496..66744670)	1,34	4,24	-1,437886457
TOP6BL	11	66744451..66843516	1,39	1,49	0,124453262
RCE1	11	66842835..66846552	16,40	9,71	0,978119819
PC	11	complement(66848417..66958386)	5,13	3,83	0,645254833
LRFN4	11	66856647..66860475	15,66	13,52	0,434732119
C11orf86	11	66975277..66977009	0,34	1,02	-1,343647809
SYT12	11	67006778..67050863	0,08	0,11	-0,250823457
RHOD	11	67056847..67072017	14,51	12,82	0,402233349
KDM2A	11	67119263..67258082	22,56	9,54	1,464741762

GRK2	11	67266473..67286556	18,29	14,79	0,528899694
ANKRD13D	11	67289300..67302485	8,26	5,21	0,889472453
SSH3	11	67303478..67312607	5,31	3,30	0,910890645
RAD9A	11	67317871..67398410	6,49	2,80	1,437394899
POLD4	11	complement(67350772..67356972)	11,36	8,01	0,726762362
gene:ENSG000	11	complement(67351572..67373584)	1,15	-	9,10907445
CLCF1	11	complement(67364168..67374177)	4,83	5,33	0,079889122
PPP1CA	11	complement(67398181..67421183)	41,61	31,89	0,607123051
TBC1D10C	11	67403915..67410089	0,19	0,08	1,409373041
CARNS1	11	67414968..67425607	0,15	0,12	0,542969924
RPS6KB2	11	67428460..67435401	13,09	9,82	0,637032362
PTPRCAP	11	complement(67435510..67437682)	-	-	0,188027316
CORO1B	11	complement(67435510..67443821)	10,32	7,04	0,774089604
GPR152	11	complement(67451301..67452729)	0,08	-	6,20258586
CABP4	11	67452406..67461752	0,58	0,01	6,157559515
TMEM134	11	complement(67461710..67469272)	2,84	2,66	0,315623939
AIP	11	67468174..67491154	15,85	11,71	0,65911912
PITPNM1	11	complement(67491768..67506263)	16,73	10,97	0,831410724
CDK2AP2	11	complement(67506497..67508649)	34,18	10,60	1,912315626
CABP2	11	complement(67518912..67524517)	-	-	#N/D
GSTP1	11	67583742..67586656	148,08	#####	0,377744364
NDUFV1	11	67605653..67612554	35,59	27,64	0,587487201
NUDT8	11	complement(67627938..67629937)	2,33	1,67	0,703749638
TBX10	11	complement(67631303..67639763)	0,05	0,01	2,805678585
ACY3	11	complement(67642555..67650730)	0,01	0,03	-1,478387936
ALDH3B2	11	complement(67662155..67681224)	-	-	#N/D
UNC93B1	11	complement(67991100..68004982)	7,72	4,41	1,030598309
ALDH3B1	11	68008578..68029282	5,07	6,60	-0,156995675
NDUFS8	11	68030617..68036644	32,78	27,04	0,500904804
TCIRG1	11	68039025..68050895	14,70	8,86	0,953566782
CHKA	11	complement(68052859..68121444)	2,02	1,68	0,487642485
KMT5B	11	complement(68154863..68213852)	1,99	1,43	0,697664718
C11orf24	11	complement(68261338..68272001)	17,86	10,21	1,029684503
LRP5	11	68312591..68449275	42,69	25,91	0,943319447
PPP6R3	11	68460731..68615334	7,10	8,01	0,049508537
GAL	11	68683779..68691175	-	-	0,188027316
TESMIN	11	complement(68707440..68751520)	0,00	-	3,068695355
CPT1A	11	complement(68754620..68844410)	21,02	22,22	0,14279324
MRPL21	11	complement(68891276..68903835)	6,19	6,39	0,178656832
IGHMBP2	11	68903863..68940602	3,19	2,89	0,366527428
MRGPRD	11	complement(68980021..68980986)	-	0,01	-2,89252005
MRGPRF	11	complement(69004398..69013246)	0,15	0,08	1,215597416
TPCN2	11	69048932..69136316	1,26	1,13	0,38420438
gene:ENSG000	11	69072915..69162440	0,16	0,13	0,563874141
SMIM38	11	69155478..69162440	0,56	0,19	1,799651447
MYEOV	11	69294151..69341682	0,79	0,94	-0,024770624
CCND1	11	69641156..69654474	54,00	35,06	0,846257676
LTO1	11	complement(69653076..69675416)	2,71	2,41	0,391467603

FGF19	11	complement(69698238..69704022)	0,08	0,26	-1,39271538
FGF4	11	complement(69771022..69775341)	-	-	#N/D
FGF3	11	complement(69809968..69819416)	-	-	0,188027316
ANO1	11	69985907..70189530	0,24	0,09	1,609838754
FADD	11	70203296..70207390	14,77	16,65	0,050756317
PPFIA1	11	70270690..70385312	5,34	6,50	-0,059030401
CTTN	11	70398404..70436584	78,02	56,64	0,685131991
SHANK2	11	complement(70467854..71252577)	0,07	0,14	-0,876577045
gene:ENSG000	11	70603304..70604859	-	-	0,188027316
gene:ENSG000	11	complement(70705605..70706068)	-	-	#N/D
DHCR7	11	complement(71428193..71452868)	15,39	5,68	1,660820413
NADSYN1	11	71453109..71524107	3,12	1,91	0,935488491
KRTAP5-7	11	71527267..71528669	-	-	0,188027316
KRTAP5-8	11	71538025..71539209	-	0,02	-3,804623889
KRTAP5-9	11	71548420..71549608	-	-	0,188027316
KRTAP5-10	11	71565563..71566735	-	0,01	-2,89252005
KRTAP5-11	11	complement(71581855..71603353)	-	-	#N/D
XNDC1N-ZNF7	11	complement(71804997..71928656)	0,59	0,36	0,941070941
DEFB108B	11	71833200..71837710	-	-	#N/D
XNDC1N	11	complement(71865504..71928654)	0,38	0,23	0,939662958
DEFB131B	11	71878453..71884561	-	-	#N/D
RNF121	11	71929018..71997597	11,08	6,41	1,011678866
IL18BP	11	71998613..72007315	4,71	2,54	1,113930319
NUMA1	11	complement(72002864..72080693)	22,15	18,54	0,479981276
LRTOMT	11	72080331..72110782	0,32	0,23	0,699521352
LRRC51	11	72080337..72096895	1,41	1,12	0,560528709
LAMTOR1	11	complement(72085895..72103297)	10,82	10,68	0,24144716
TOMT	11	72105924..72109596	-	-	0,188027316
ANAPC15	11	complement(72106378..72112780)	5,56	5,04	0,365429076
FOLR3	11	72114869..72139892	0,01	0,07	-2,665078517
FOLR1	11	72189558..72196323	-	-	0,188027316
FOLR2	11	72216601..72221950	0,01	-	3,068695355
INPPL1	11	72223701..72239147	41,88	27,67	0,821120703
PHOX2A	11	complement(72239077..72245664)	0,18	0,18	0,181545758
CLPB	11	complement(72285495..72434680)	5,44	5,32	0,256348119
PDE2A	11	complement(72576141..72674591)	0,34	1,53	-1,960817956
ARAP1	11	complement(72685069..72793599)	8,12	7,80	0,281815093
STARD10	11	complement(72754729..72794047)	1,89	2,16	0,034400478
ATG16L2	11	72814406..72843674	3,08	2,46	0,543218248
FCHSD2	11	complement(72836745..73142318)	4,66	4,11	0,406096957
P2RY2	11	73218281..73242427	0,01	0,25	-3,872900136
P2RY6	11	73264498..73305103	0,11	0,46	-1,856705623
ARHGEF17	11	73308276..73369388	9,84	6,42	0,837520675
RELT	11	73376399..73397474	7,01	3,76	1,122466842
FAM168A	11	complement(73400487..73598189)	19,17	9,53	1,23101437
PLEKHB1	11	73646178..73662819	0,02	0,03	-0,698295353
RAB6A	11	complement(73675638..73761137)	18,74	13,32	0,715805398
MRPL48	11	73787872..73865133	3,34	2,90	0,42885896

COA4	11	complement(73872667..73876901)	14,59	13,27	0,36018113
PAAF1	11	73876699..73931114	2,83	2,21	0,577265678
DNAJB13	11	73951026..73970366	0,09	0,12	-0,164183768
UCP2	11	complement(73974672..73982843)	11,18	3,02	2,109221089
UCP3	11	complement(74000277..74009085)	0,08	0,10	-0,039381237
C2CD3	11	complement(74012718..74171210)	3,16	2,43	0,601048365
PPME1	11	74171267..74254703	22,63	18,03	0,5508783
gene:ENSG000	11	74204869..74205746	0,36	0,04	3,176860199
P4HA3	11	complement(74235801..74311640)	0,10	0,07	0,692913342
PGM2L1	11	complement(74330316..74398433)	0,74	0,38	1,18855122
KCNE3	11	complement(74454841..74467729)	-	0,01	-4,758582517
LIPT2	11	complement(74490519..74493724)	0,16	0,46	-1,299756374
POLD3	11	74493851..74669117	6,44	4,48	0,746562545
CHRDL2	11	complement(74696429..74731426)	0,01	-	3,967268276
gene:ENSG000	11	complement(74698231..74699658)	-	0,08	-5,902279478
RNF169	11	74748849..74842413	9,98	7,67	0,603596285
XRRA1	11	complement(74807739..74949200)	1,67	1,46	0,419218737
gene:ENSG000	11	complement(74830574..74832510)	0,15	0,09	0,901271157
SPCS2	11	74949261..74979033	7,77	6,26	0,534805438
NEU3	11	74988279..75018893	2,99	2,47	0,497887885
OR2AT4	11	complement(75081753..75096876)	0,00	0,00	-0,01185201
SLCO2B1	11	75100563..75206549	0,05	0,87	-3,766003495
TPBGL	11	75240774..75243704	0,08	-	7,391550995
ARRB1	11	complement(75260122..75351705)	3,81	3,01	0,565341664
gene:ENSG000	11	complement(75260129..75262466)	-	-	0,188027316
RPS3	11	75399515..75422280	160,10	#####	0,646181581
KLHL35	11	complement(75422394..75433203)	0,75	0,87	0,019408117
GDPD5	11	complement(75434640..75525941)	2,53	1,99	0,566568638
SERPINH1	11	75562056..75572783	80,61	60,52	0,636634285
MAP6	11	complement(75586918..75669038)	0,03	0,02	1,117604237
MOGAT2	11	75717838..75732958	-	-	0,188027316
DGAT2	11	75759512..75801535	2,52	7,17	-1,28438949
UVRAG	11	75815210..76144232	2,93	2,25	0,608184634
WNT11	11	complement(76186325..76210736)	0,01	0,03	-0,742377908
THAP12	11	complement(76349898..76381132)	3,81	5,36	-0,271126802
GVQW3	11	76381303..76414619	0,44	0,40	0,357791861
EMSY	11	76444923..76553031	3,59	2,17	0,947539136
LRRC32	11	complement(76657524..76670747)	0,01	0,01	0,933337975
TSKU	11	76782251..76798153	15,01	10,75	0,70426847
ACER3	11	76860859..77026797	1,55	2,15	-0,250381543
B3GNT6	11	77034398..77041973	0,03	0,03	-0,036228841
CAPN5	11	77066961..77126155	13,61	5,88	1,43445009
OMP	11	77102840..77103331	0,26	0,13	1,202745498
MYO7A	11	77128246..77215241	0,05	0,02	1,46559662
GDPD4	11	complement(77216558..77301687)	-	-	#N/D
PAK1	11	complement(77322017..77474635)	9,24	8,24	0,387375118
CLNS1A	11	complement(77514936..77637794)	5,83	7,17	-0,074879237
AQP11	11	77589391..77610356	0,26	0,49	-0,655326397

RSF1	11	complement(77660009..77820869)	1,86	2,11	0,037584776
AAMDC	11	77821109..77918432	1,45	0,72	1,233702218
INTS4	11	complement(77874418..77994715)	3,87	2,41	0,905491194
KCTD14	11	complement(78015715..78046191)	1,23	3,39	-1,242437599
NDUFC2-KCTD	11	complement(78016971..78079865)	1,01	1,07	0,138540008
THRSP	11	78063861..78068351	0,09	0,08	0,468811989
NDUFC2	11	complement(78068297..78079862)	8,45	7,54	0,386988572
ALG8	11	complement(78095244..78139660)	3,08	3,30	0,12274176
KCTD21	11	complement(78171249..78188626)	2,85	2,27	0,548839768
USP35	11	78188812..78215232	2,96	1,71	1,011967382
GAB2	11	complement(78215293..78418348)	0,63	1,30	-0,835164381
NARS2	11	complement(78435620..78575194)	1,70	1,60	0,317302523
TENM4	11	complement(78652829..79441030)	0,09	0,29	-1,456136688
gene:ENSG000	11	79966805..79968708	-	-	#N/D
FAM181B	11	complement(82729940..82733864)	0,00	-	3,068695355
PRCP	11	complement(82822936..82970584)	6,16	4,71	0,608491245
DDIAS	11	82899975..82958277	1,28	1,41	0,089288512
RAB30	11	complement(82973133..83071923)	1,03	0,70	0,784000751
gene:ENSG000	11	83083687..83084138	0,19	0,26	-0,204080844
PCF11	11	83156988..83187451	2,52	3,32	-0,174480952
ANKRD42	11	83193712..83260694	1,67	0,94	1,042193921
CCDC90B	11	complement(83259081..83286390)	1,01	1,42	-0,269233099
DLG2	11	complement(83455012..85628335)	0,11	0,09	0,494861198
TMEM126B	11	85628573..85636539	1,79	2,61	-0,32463526
TMEM126A	11	85647967..85656547	7,20	5,37	0,646401032
CREBZF	11	complement(85657742..85682908)	8,83	5,59	0,884068389
CCDC89	11	complement(85683848..85686195)	0,02	0,01	1,232079601
SYTL2	11	complement(85694224..85811159)	1,16	0,28	2,261289757
gene:ENSG000	11	complement(85852557..85854943)	0,25	0,15	0,930346199
CCDC83	11	85855101..85920021	-	-	0,188027316
PICALM	11	complement(85957175..86069882)	21,37	16,41	0,604183512
EED	11	86201212..86278813	2,17	2,34	0,114005016
HIKESHI	11	86302211..86345943	8,38	7,23	0,436648956
CCDC81	11	86374736..86423109	0,13	0,07	1,036529439
gene:ENSG000	11	complement(86434924..86437282)	0,02	0,01	1,833201638
ME3	11	complement(86441108..86672636)	0,69	0,91	-0,172191007
PRSS23	11	86791059..86952910	71,59	40,78	1,034964064
gene:ENSG000	11	complement(86908990..86912238)	0,06	0,12	-0,804783029
FZD4	11	complement(86945679..86955395)	1,04	1,18	0,034111367
TMEM135	11	87037844..87328824	1,17	0,66	1,033464124
gene:ENSG000	11	87121058..87121596	-	-	0,188027316
gene:ENSG000	11	87490144..87491110	-	-	#N/D
gene:ENSG000	11	87565994..87566421	-	-	#N/D
gene:ENSG000	11	complement(87718667..87721233)	-	-	#N/D
gene:ENSG000	11	87816999..87817567	-	-	#N/D
gene:ENSG000	11	complement(87879473..87880095)	-	-	0,188027316
RAB38	11	complement(88113251..88175443)	0,06	0,26	-1,877459449
gene:ENSG000	11	complement(88165575..88167691)	-	0,01	-3,804623889

CTSC	11	complement(88265069..88359684)	2,34	7,55	-1,464706991
GRM5	11	complement(88504576..89065982)	0,00	0,00	0,886720911
TYR	11	89177875..89295759	-	-	#N/D
gene:ENSG000	11	89297717..89298199	-	-	#N/D
gene:ENSG000	11	complement(89300709..89301477)	-	-	#N/D
NOX4	11	complement(89324353..89498187)	0,01	-	4,516772761
gene:ENSG000	11	complement(89533645..89534043)	-	-	#N/D
gene:ENSG000	11	89545128..89545744	-	-	#N/D
gene:ENSG000	11	complement(89548130..89548795)	-	-	0,188027316
gene:ENSG000	11	89556860..89557205	-	-	#N/D
gene:ENSG000	11	89576913..89577652	-	-	#N/D
gene:ENSG000	11	89704164..89704658	-	-	#N/D
TRIM77	11	89710299..89717872	-	-	0,188027316
TRIM49	11	complement(89797655..89808575)	-	-	0,188027316
TRIM64B	11	complement(89870438..89878487)	-	-	#N/D
TRIM49D1	11	complement(89911111..89922245)	0,01	-	3,967268276
TRIM49D2	11	89924064..89933063	-	-	0,188027316
TRIM64	11	89966037..89974072	-	-	#N/D
TRIM49C	11	90031106..90042025	-	-	0,188027316
UBTFL1	11	90085950..90087131	-	-	#N/D
gene:ENSG000	11	90125658..90126061	-	-	#N/D
NAALAD2	11	90131515..90192894	0,06	0,03	1,121384747
gene:ENSG000	11	90193614..90198120	0,78	0,55	0,737667141
CHORDC1	11	complement(90200429..90223077)	1,54	1,47	0,290518276
gene:ENSG000	11	90223153..90226538	0,15	0,04	2,146457731
gene:ENSG000	11	90731110..90731723	-	-	#N/D
gene:ENSG000	11	complement(90972316..90972521)	-	-	#N/D
gene:ENSG000	11	91013787..91015299	-	-	0,188027316
gene:ENSG000	11	complement(91093875..91094260)	-	-	#N/D
gene:ENSG000	11	complement(91184934..91185543)	-	-	#N/D
gene:ENSG000	11	complement(91208838..91209590)	-	-	#N/D
gene:ENSG000	11	91382909..91383289	-	-	#N/D
gene:ENSG000	11	91992833..91993255	-	-	#N/D
FAT3	11	92224818..92896473	-	-	0,188027316
MTNR1B	11	92969651..92985066	-	-	0,188027316
gene:ENSG000	11	93071529..93072031	-	-	#N/D
gene:ENSG000	11	93133855..93134164	-	-	0,188027316
SLC36A4	11	complement(93144174..93197991)	1,55	1,24	0,543223189
gene:ENSG000	11	93171134..93171906	-	0,03	-3,804623889
gene:ENSG000	11	complement(93206990..93207583)	-	-	0,188027316
gene:ENSG000	11	complement(93286629..93288903)	0,01	-	3,967268276
DEUP1	11	93329971..93438487	-	-	0,188027316
SMCO4	11	complement(93478472..93543391)	5,02	2,72	1,109171321
gene:ENSG000	11	93587703..93588559	-	-	#N/D
gene:ENSG000	11	93592187..93592669	-	-	0,188027316
CEP295	11	93661682..93730358	1,83	2,08	0,036783679
gene:ENSG000	11	complement(93726654..93729805)	0,49	0,49	0,201958086
TAF1D	11	complement(93729948..93784391)	5,00	3,81	0,613365127

C11orf54	11	93741591..93764749	1,68	1,45	0,431766332
gene:ENSG000	11	93741664..93812378	0,25	0,67	-1,215718723
MED17	11	93784227..93814963	0,96	1,24	-0,143100756
VSTM5	11	complement(93818232..93850618)	0,02	0,02	0,221270597
HEPHL1	11	94021354..94114208	0,01	0,02	-0,895537122
PANX1	11	94128841..94181968	8,08	6,75	0,482275313
IZUMO1R	11	94304580..94308146	-	-	#N/D
GPR83	11	complement(94377316..94401419)	0,03	0,03	0,335747576
MRE11	11	complement(94415570..94493885)	3,27	2,74	0,477187108
ANKRD49	11	94493979..94499578	1,38	1,23	0,390749061
C11orf97	11	94512461..94532123	-	-	#N/D
PIWIL4	11	94543840..94621421	0,31	0,45	-0,314541158
FUT4	11	94543921..94549895	2,61	2,51	0,280536345
gene:ENSG000	11	94559018..94559374	0,09	0,04	1,436225396
AMOTL1	11	94706431..94876748	10,64	6,30	0,979420185
CWC15	11	complement(94962620..94973586)	9,10	6,84	0,634430828
KDM4D	11	94973709..94999519	0,50	1,03	-0,818537057
KDM4E	11	95025258..95027596	-	-	#N/D
KDM4F	11	95048142..95051338	-	-	0,188027316
SRSF8	11	95066919..95071225	1,34	1,11	0,492236333
ENDOD1	11	95089846..95132645	0,90	0,89	0,237081592
SESN3	11	complement(95165513..95232541)	0,05	-	8,355048894
FAM76B	11	complement(95768953..95790409)	1,10	0,81	0,666274642
CEP57	11	95789965..95832693	3,88	4,44	0,027781722
MTMR2	11	complement(95821766..95925315)	3,06	2,83	0,337662307
MAML2	11	complement(95976598..96343195)	1,15	2,06	-0,619376106
CCDC82	11	complement(96349241..96389956)	0,40	0,41	0,173656139
JRKL	11	96389989..96507574	2,83	2,32	0,509370635
CNTN5	11	99020949..100358885	0,00	0,03	-3,112412319
ARHGAP42	11	100687288..100993941	0,05	0,30	-2,410036146
PGR	11	complement(101029624..10112981	0,04	0,01	2,357537755
TRPC6	11	complement(101451564..10187256	0,00	0,02	-2,447197442
ANGPTL5	11	complement(101890674..10191652	0,02	0,02	0,677647514
CEP126	11	101915010..102001062	0,60	0,83	-0,24321045
CFAP300	11	102047437..102084554	0,12	0,21	-0,622104509
YAP1	11	102110447..102233424	19,07	15,28	0,542917444
BIRC3	11	102317484..102339403	3,12	6,49	-0,834430082
BIRC2	11	102347211..102378670	11,73	5,79	1,241590849
TMEM123	11	complement(102396332..10247038	16,84	19,32	0,02478333
MMP7	11	complement(102520508..10253075	0,08	0,02	1,893574746
MMP20	11	complement(102576832..10262533	-	-	#N/D
MMP27	11	complement(102691487..10270576	-	-	0,188027316
MMP8	11	complement(102711796..10272705	-	-	0,188027316
MMP10	11	complement(102770502..10278062	0,05	0,02	1,339142659
MMP1	11	complement(102789401..10279816	5,45	1,74	1,870215868
MMP3	11	complement(102835801..10284360	0,20	0,38	-0,731640816
MMP12	11	complement(102862736..10287498	0,01	0,01	-0,01185201
MMP13	11	complement(102942995..10295573	0,02	-	4,913749004

DCUN1D5	11	complement(103050686..10309219	1,24	1,45	-0,003969567
DYNC2H1	11	103109410..103479863	1,08	1,76	-0,490752022
PDGFD	11	complement(103907189..10416437	0,73	0,37	1,197507089
DDI1	11	104036640..104039196	0,00	-	3,068695355
CASP12	11	complement(104885718..10489867	-	0,01	-3,804623889
CASP4	11	complement(104942866..10496936	11,20	15,53	-0,248186918
CASP5	11	complement(104994235..10502316	-	-	0,188027316
CASP1	11	complement(105025397..10503525	0,53	0,60	0,050008602
CARD16	11	complement(105041326..10510143	0,32	0,27	0,487618325
CARD18	11	complement(105137714..10553169	-	-	0,188027316
GRIA4	11	105609535..105982090	0,03	0,03	-0,159071374
MSANTD4	11	complement(105995623..10602240	3,70	3,71	0,218410864
KBTBD3	11	complement(106051098..10607745	0,22	0,15	0,769146004
AASDHPPT	11	106075501..106098699	3,29	5,39	-0,489334574
GUCY1A2	11	complement(106674019..10701847	0,04	0,22	-2,349363348
CWF19L2	11	complement(107326360..10745782	1,40	1,23	0,411365035
ALKBH8	11	complement(107502727..10756574	0,88	1,04	-0,021063634
ELMOD1	11	107591091..107666779	0,04	0,02	1,391449417
SLN	11	complement(107707378..10771969	-	0,03	-3,804623889
SLC35F2	11	complement(107790991..10792829	17,89	12,50	0,73999056
RAB39A	11	107928448..107963482	0,37	0,99	-1,186299693
CUL5	11	108008898..108107761	1,77	1,96	0,076153111
ACAT1	11	108116695..108147603	5,09	6,33	-0,091612848
NPAT	11	complement(108157215..10822263	2,98	2,21	0,656303093
ATM	11	108222804..108369102	2,35	1,76	0,640399177
C11orf65	11	complement(108308519..10846753	0,12	0,05	1,501082854
POGLUT3	11	complement(108472112..10849840	11,03	9,94	0,372956327
EXPH5	11	complement(108505435..10859376	0,12	0,42	-1,648904152
DDX10	11	108665058..108940999	2,95	3,35	0,040492229
C11orf87	11	109422190..109429167	0,00	0,00	-0,01185201
RDX	11	complement(109864295..11029671	6,31	5,83	0,336826913
ZC3H12C	11	110093392..110171841	3,31	2,74	0,498962156
FDX1	11	110429948..110464884	2,00	3,13	-0,427624973
ARHGAP20	11	complement(110577042..11071318	0,03	0,12	-1,987469741
POU2AF2	11	111245725..111286401	-	-	#N/D
POU2AF3	11	111298546..111308735	0,01	-	3,967268276
POU2AF1	11	complement(111352255..11145563	0,00	0,02	-1,877914477
BTG4	11	complement(111467526..11151436	0,02	-	4,913749003
HOATZ	11	111514778..111537043	0,06	0,01	2,570892441
LAYN	11	111540280..111561745	9,43	9,08	0,277406586
SIK2	11	111602449..111730855	6,55	4,15	0,882182399
PPP2R1B	11	complement(111726908..11176638	3,71	2,98	0,541043759
ALG9	11	complement(111782195..11187158	2,20	2,48	0,052806721
gene:ENSG000	11	complement(111786286..11187942	0,24	0,12	1,146011896
FDXACB1	11	complement(111874056..11188124	0,77	0,81	0,141239313
CFAP68	11	111878935..111885975	0,38	0,49	-0,140818302
CRYAB	11	complement(111908564..11192372	0,11	0,01	3,12712421
HSPB2	11	111912734..111914093	0,01	0,03	-0,923955849

HSPB2-C11orf5	11	111912736..111926871	-	-	#N/D
C11orf52	11	111918032..111926871	0,01	0,04	-1,291882393
DIXDC1	11	111927144..112022653	1,33	1,56	-0,010414812
DLAT	11	112025033..112064404	8,39	8,38	0,223931059
PIH1D2	11	complement(112063218..11207427	0,11	0,11	0,248330452
NKAPD1	11	112074086..112085150	3,16	4,32	-0,229406322
TIMM8B	11	complement(112084800..11208679	4,85	5,47	0,048010834
SDHD	11	112086824..112120016	14,06	9,83	0,73999958
gene:ENSG000	11	112086903..112193805	0,15	0,20	-0,187130731
IL18	11	complement(112143253..11216409	0,43	2,70	-2,408032086
TEX12	11	112167372..112172556	0,01	-	3,068695355
BCO2	11	112175510..112224699	0,05	0,13	-1,277219466
PTS	11	112226367..112269955	4,16	4,94	-0,024967935
PLET1	11	complement(112248153..11226086	0,06	0,09	-0,322898219
NCAM1	11	112961275..113278436	0,82	2,45	-1,354372115
TTC12	11	113314579..113383544	0,50	0,44	0,41459577
ANKK1	11	113387779..113400416	0,02	0,00	2,144183439
DRD2	11	complement(113409605..11347569	0,03	0,01	2,209934656
TMPRSS5	11	complement(113687547..11370637	0,27	0,11	1,492067379
ZW10	11	complement(113733187..11377373	5,28	4,56	0,432232824
CLDN25	11	113779796..113780485	-	-	#N/D
USP28	11	complement(113797874..11387557	3,08	4,23	-0,235461397
HTR3B	11	113904796..113949079	-	-	0,188027316
HTR3A	11	113975075..113990313	-	-	#N/D
ZBTB16	11	114059041..114256765	0,00	-	4,516772761
NNMT	11	114257787..114313536	6,26	13,13	-0,84575453
C11orf71	11	complement(114391443..11440051	0,66	0,59	0,366771632
RBM7	11	114400030..114414203	1,62	1,49	0,339770897
gene:ENSG000	11	114400682..114443932	1,64	1,30	0,563101942
REXO2	11	114439435..114450279	19,07	17,40	0,355135117
NXPE1	11	complement(114518934..11455988	-	0,00	-3,804623889
NXPE4	11	complement(114570591..11459578	-	0,01	-2,89252005
NXPE2	11	114678527..114707069	0,05	0,01	2,060408965
CADM1	11	complement(115169218..11550495	1,94	8,38	-1,884833095
gene:ENSG000	11	115659168..115661417	-	-	#N/D
gene:ENSG000	11	complement(115731942..11573474	0,00	-	3,068695355
gene:ENSG000	11	complement(116609995..11661009	-	-	#N/D
BUD13	11	complement(116748170..11677298	7,65	6,87	0,379700554
ZPR1	11	complement(116773799..11678803	8,10	5,67	0,738366454
APOA5	11	complement(116789367..11679242	0,02	0,03	-0,742377908
APOA4	11	complement(116820700..11682330	-	-	0,188027316
APOC3	11	116829706..116833072	-	-	#N/D
APOA1	11	complement(116835751..11683762	0,08	0,12	-0,35396495
SIK3	11	complement(116843402..11709843	1,34	2,95	-0,91326031
PAFAH1B2	11	117144284..117176894	26,58	18,39	0,754547708
SIDT2	11	117178736..117197445	5,47	2,95	1,111286329
TAGLN	11	117199370..117207464	0,39	0,78	-0,772987259
PCSK7	11	complement(117204337..11723252	4,25	3,26	0,603697683

gene:ENSG000	11	117204967..117210292	0,98	0,48	1,246799674
RNF214	11	117232625..117286454	5,59	4,91	0,407833882
BACE1	11	complement(117285232..11731625	7,38	5,02	0,780162643
CEP164	11	117314557..117413266	3,51	3,21	0,351316775
DSCAML1	11	complement(117427772..11781752	-	-	0,188027316
gene:ENSG000	11	complement(117611101..11761201	-	-	#N/D
FXYD2	11	complement(117800844..11782869	-	-	0,188027316
FXYD6-FXYD2	11	complement(117820163..11787666	-	-	0,188027316
FXYD6	11	complement(117836976..11787748	0,46	0,01	5,809872517
TMPRSS13	11	complement(117900641..11792945	0,04	0,00	4,247318137
IL10RA	11	117986370..118003037	0,15	0,03	2,526939688
SMIM35	11	complement(118003634..11808829	0,00	-	3,068695355
TMPRSS4	11	118077012..118125510	0,03	0,00	3,04797919
SCN4B	11	complement(118133377..11815288	4,67	0,07	6,19535266
SCN2B	11	complement(118162806..11817663	0,01	0,01	0,677647514
JAML	11	complement(118193725..11822509	-	-	0,188027316
MPZL3	11	complement(118226690..11825236	1,37	0,60	1,415092696
MPZL2	11	complement(118253416..11826453	0,08	0,00	4,31100363
gene:ENSG000	11	118264593..118266817	0,01	-	4,516772761
CD3E	11	118304730..118316175	-	-	#N/D
CD3D	11	complement(118339075..11834270	-	-	#N/D
CD3G	11	118344344..118355161	0,01	0,00	0,886720911
UBE4A	11	118359600..118399211	11,98	8,41	0,734168126
ATP5MG	11	118401346..118433278	5,72	4,70	0,508694741
gene:ENSG000	11	118401602..118482737	0,08	0,11	-0,153119759
KMT2A	11	118436456..118526832	9,85	7,54	0,607801679
TTC36	11	118527472..118531197	0,06	0,05	0,533811434
TMEM25	11	118531041..118547280	0,13	0,57	-1,896446579
IFT46	11	complement(118544528..11857297	5,23	4,62	0,40282344
ARCN1	11	118572390..118603033	81,93	46,14	1,051605576
PHLDB1	11	118606440..118658031	16,79	10,32	0,925161585
TREH	11	complement(118657316..11867969	0,42	0,17	1,527864725
DDX6	11	complement(118747763..11879116	27,58	19,45	0,72716575
CXCR5	11	118883892..118897787	0,15	0,09	0,9331436
BCL9L	11	complement(118893875..11892592	24,96	12,10	1,268016459
UPK2	11	118925164..118958559	0,05	0,01	1,833201638
FOXR1	11	118971712..118981287	-	-	0,188027316
CENATAC	11	118998138..119015793	2,64	3,61	-0,226554177
RPS25	11	complement(119015712..11901869	212,81	#####	0,607084064
TRAPPC4	11	119018763..119025454	11,17	7,24	0,848316791
SLC37A4	11	complement(119023751..11903090	9,36	6,66	0,714337753
HYOU1	11	complement(119044188..11905722	72,59	47,79	0,825913588
VPS11	11	119067818..119081972	7,34	3,75	1,192840375
HMBS	11	119084866..119093834	8,46	7,20	0,456502585
H2AX	11	complement(119093874..11909546	42,43	33,57	0,560747625
DPAGT1	11	complement(119096025..11910833	15,47	10,20	0,823810377
C2CD2L	11	119102198..119118544	8,92	3,19	1,707457919
HINFP	11	119121580..119136059	3,78	2,26	0,962107577

ABCG4	11	119149052..119162653	0,07	0,13	-0,552210716
NLRX1	11	119166568..119184016	5,71	6,71	-0,007576368
NHERF4	11	119185457..119190223	0,03	0,02	0,939616118
CCDC153	11	complement(119190250..11919676	0,01	0,08	-2,665078517
CBL	11	119206298..119313926	10,43	9,60	0,342753434
MCAM	11	complement(119308529..11932152	0,53	2,65	-2,109309797
RNF26	11	119334527..119337309	59,37	41,54	0,738076886
C1QTNF5	11	complement(119338942..11934094	0,04	0,16	-1,676700393
MFRP	11	complement(119338942..11934670	0,07	0,04	0,838252066
USP2	11	complement(119355215..11938171	1,53	1,77	0,004736513
THY1	11	complement(119415476..11942498	0,52	14,21	-4,546274707
NECTIN1	11	complement(119623408..11972920	8,16	4,54	1,067916931
TRIM29	11	complement(120111286..12018552	0,11	0,04	1,818169474
OAF	11	120211032..120230334	50,85	32,45	0,871227736
POU2F3	11	120236640..120319945	0,01	0,08	-2,243372656
TLCD5	11	120325296..120333686	3,18	3,21	0,206971034
ARHGEF12	11	120336413..120489937	16,27	10,75	0,820862069
GRIK4	11	120511746..120988906	0,01	-	5,88622595
TBCEL	11	121024072..121090776	1,18	1,21	0,189648458
TBCEL-TECTA	11	121024125..121113108	0,20	0,05	2,105532928
TECTA	11	121101243..121191490	0,02	0,03	-0,449191989
SC5D	11	121292681..121313410	1,71	0,86	1,208438734
SORL1	11	121452314..121633763	0,13	0,17	-0,177096604
BLID	11	complement(122115340..12211621	3,13	0,86	2,087382078
UBASH3B	11	122655722..122814473	3,27	10,02	-1,391253536
CRTAM	11	122838500..122872643	-	-	0,188027316
JHY	11	122882683..122963862	0,25	0,41	-0,4515773
BSX	11	complement(122977570..12298183	-	-	#N/D
HSPA8	11	complement(123057489..12306323	228,49	#####	1,227450616
CLMP	11	complement(123069872..12319524	12,07	11,87	0,246488532
GRAMD1B	11	123358428..123627774	0,06	0,35	-2,234721448
SCN3B	11	complement(123629187..12365524	0,00	0,00	-0,01185201
ZNF202	11	complement(123723914..12374167	3,68	2,36	0,865120739
OR6X1	11	complement(123753580..12375451	-	-	#N/D
OR6M1	11	complement(123805408..12380634	-	-	#N/D
TMEM225	11	complement(123882920..12388567	-	-	#N/D
OR8D4	11	123902167..123909229	-	-	#N/D
OR4D5	11	123939617..123940573	-	-	#N/D
OR6T1	11	complement(123942867..12394383	-	-	0,188027316
OR10S1	11	complement(123976661..12397778	-	-	#N/D
OR10G6	11	complement(123994163..12399516	-	-	#N/D
OR10G4	11	124012997..124018732	-	-	0,188027316
OR10G9	11	124023013..124023948	-	-	#N/D
OR10G8	11	124026798..124030634	-	-	#N/D
OR10G7	11	complement(124036013..12404132	-	-	#N/D
VWA5A	11	124115404..124147721	0,99	1,35	-0,220140251
OR10D3	11	124183345..124188780	-	-	#N/D
OR8G3P	11	124211707..124215828	-	-	#N/D

OR8G2P	11	124224732..124225666	-	-	#N/D
OR8G1	11	124241095..124254364	-	-	#N/D
OR8G5	11	124256376..124266224	-	-	#N/D
OR8D1	11	complement(124302831..12431509	0,00	0,01	-2,447197442
OR8D2	11	complement(124319262..12432019	-	-	0,188027316
OR8B2	11	complement(124382394..12438449	-	-	0,188027316
OR8B3	11	complement(124395534..12439902	-	-	#N/D
OR8B4	11	complement(124423942..12442487	-	-	#N/D
OR8B8	11	complement(124437445..12444569	-	-	#N/D
OR8B12	11	complement(124539635..12454533	-	-	#N/D
OR8A1	11	124566660..124582942	0,00	-	3,068695355
PANX3	11	124611428..124620356	-	-	0,188027316
TBRG1	11	124622836..124635926	3,09	3,29	0,132454425
SIAE	11	complement(124633113..12469570	1,23	1,52	-0,085878105
SPA17	11	124673844..124697518	0,85	0,69	0,516450231
NRGN	11	124739942..124747210	10,78	7,36	0,77293491
VSIG2	11	complement(124747474..12475225	0,20	0,19	0,320877146
ESAM	11	complement(124752583..12476229	0,47	0,41	0,396817912
MSANTD2	11	complement(124766498..12480070	2,76	2,00	0,685563434
ROBO3	11	124865432..124881471	4,19	3,94	0,313527773
ROBO4	11	complement(124883691..12489850	2,31	3,74	-0,472184691
HEPACAM	11	complement(124919205..12493641	0,02	0,01	0,677647514
HEPN1	11	124919250..124920677	-	-	#N/D
CCDC15	11	124954121..125041489	1,38	1,05	0,610217415
SLC37A2	11	125063302..125090516	2,32	1,05	1,37047722
TMEM218	11	complement(125094389..12511176	1,38	1,05	0,618075405
PKNOX2	11	125164687..125433389	1,14	1,82	-0,448357607
FEZ1	11	complement(125442881..12559256	2,48	2,70	0,099304337
EI24	11	125569280..125584684	31,23	28,10	0,375240908
STT3A	11	125591712..125625215	57,20	31,41	1,087560676
CHEK1	11	125625163..125676255	5,40	2,42	1,378467626
ACRV1	11	complement(125671522..12568087	0,10	0,04	1,524737598
PATE1	11	125746279..125749867	0,04	-	5,480421266
PATE2	11	complement(125776113..12577882	0,02	0,01	1,436225396
PATE3	11	125788127..125791600	0,02	-	3,967268276
PATE4	11	125833316..125840072	0,07	0,03	1,202745498
HYLS1	11	125883614..125900646	1,93	1,91	0,24113256
PUS3	11	complement(125893485..12590322	2,39	0,66	2,082967594
DDX25	11	125903348..125943702	0,00	0,00	-0,01185201
VSIG10L2	11	125946044..125958647	0,03	-	6,780090526
CDON	11	complement(125955796..12606333	5,26	0,92	2,739376144
RPUSD4	11	complement(126202096..12621169	8,44	2,35	2,069398249
FAM118B	11	126211724..126262984	2,26	0,94	1,486917014
SRPRA	11	complement(126262938..12626914	119,81	29,43	2,248522814
FOXRED1	11	126269024..126278131	9,43	3,68	1,581191901
TIRAP	11	126282497..126298845	0,67	0,53	0,560038487
DCPS	11	126304060..126350005	4,89	2,22	1,358766901
ST3GAL4	11	126355640..126440344	6,43	2,13	1,81906328

KIRREL3	11	complement(126423358..12700346	0,20	0,02	3,702588353
gene:ENSG000	11	complement(126934886..12693715	-	-	0,188027316
ETS1	11	complement(128458761..12858755	33,03	12,70	1,601685405
FLI1	11	128686535..128813267	4,76	3,17	0,811238485
KCNJ1	11	complement(128836315..12886737	0,01	0,01	0,524121557
KCNJ5	11	128891356..128921163	0,01	0,00	2,399873901
TP53AIP1	11	complement(128934731..12894339	0,00	-	3,068695355
ARHGAP32	11	complement(128965060..12927932	2,34	0,93	1,550203456
BARX2	11	129375848..129452279	-	-	#N/D
TMEM45B	11	129815848..129860003	0,05	0,03	0,822166453
NFRKB	11	complement(129863636..12989559	6,20	2,25	1,683833368
PRDM10	11	complement(129899706..13000283	3,81	1,77	1,327926028
APLP2	11	130068147..130144811	118,75	42,95	1,690368275
ST14	11	130159782..130210362	0,04	0,01	1,790975958
ZBTB44	11	complement(130226677..13031491	3,27	1,18	1,698353242
ADAMTS8	11	complement(130404923..13042860	0,03	-	6,338059249
ADAMTS15	11	130448645..130476645	3,90	1,96	1,213430207
SNX19	11	complement(130866250..13091647	5,99	1,92	1,863238485
NTM	11	131370478..132336822	0,73	0,19	2,136335055
gene:ENSG000	11	complement(132403360..13240406	-	-	#N/D
OPCML	11	complement(132414977..13353250	-	-	0,188027316
gene:ENSG000	11	complement(132771623..13277337	-	-	#N/D
gene:ENSG000	11	133064697..133068129	-	-	0,188027316
gene:ENSG000	11	complement(133297189..13330263	-	-	#N/D
SPATA19	11	complement(133840631..13384553	0,01	-	3,068695355
IGSF9B	11	complement(133896438..13395696	2,21	0,20	3,679094996
JAM3	11	134069071..134152001	2,57	0,06	5,630481329
NCAPD3	11	complement(134150113..13422550	11,81	4,02	1,778259662
VPS26B	11	134224671..134247788	10,86	4,81	1,399200824
THYN1	11	complement(134248279..13425337	5,21	4,27	0,509467282
ACAD8	11	134253548..134265855	2,67	1,28	1,279473725
GLB1L3	11	134274245..134319564	0,02	0,76	-4,968553493
GLB1L2	11	134331988..134378341	0,22	0,84	-1,715074132
B3GAT1	11	complement(134378504..13441224	0,01	0,00	1,339142659
gene:ENSG000	11	134985683..134986799	-	-	#N/D
IQSEC3	12	66767..178455	0,01	0,05	-1,660236228
SLC6A12	12	complement(190077..214570)	0,33	0,89	-1,192328844
SLC6A13	12	complement(220621..262873)	0,01	0,03	-1,406253396
KDM5A	12	complement(280057..389320)	7,64	8,14	0,13172312
CCDC77	12	389273..442642	3,17	5,12	-0,469996983
B4GALNT3	12	459939..563509	1,01	0,46	1,354944252
NINJ2	12	complement(564296..663589)	0,07	0,15	-0,838652411
WNK1	12	752579..911452	18,23	25,06	-0,236082617
RAD52	12	complement(911736..990053)	1,90	2,74	-0,306844679
ERC1	12	990509..1495933	8,10	10,99	-0,216343923
WNT5B	12	1529891..1647212	13,82	6,02	1,423074085
FBXL14	12	complement(1565993..1594581)	1,67	4,25	-1,124740141
ADIPOR2	12	1688574..1788674	19,89	22,70	0,032447159

CACNA2D4	12	complement(1791963..1918666)	0,03	0,02	0,857064368
LRTM2	12	1820267..1836753	0,02	0,00	2,399873901
DCP1B	12	complement(1946053..2004535)	2,14	3,13	-0,326843986
CACNA1C	12	1970772..2697950	0,32	0,61	-0,713624132
FKBP4	12	2794970..2805423	29,32	44,58	-0,381685885
ITFG2	12	2812622..2859791	2,90	3,03	0,16049554
NRIP2	12	complement(2825348..2835544)	1,08	0,89	0,511117202
TEX52	12	complement(2849046..2857039)	0,15	0,23	-0,358351767
FOXM1	12	complement(2857680..2877174)	50,07	59,11	-0,016455652
RHNO1	12	2876258..2889524	12,60	19,56	-0,411624
TULP3	12	2877223..2941138	14,04	13,70	0,258567369
TEAD4	12	2959330..3040676	6,35	12,88	-0,79744019
TSPAN9	12	3077355..3286564	8,26	6,29	0,617582152
PRMT8	12	3381349..3593973	-	0,01	-2,89252005
CRACR2A	12	complement(3606633..3764819)	1,75	3,15	-0,628638294
gene:ENSG000	12	complement(3610307..3612003)	0,91	1,77	-0,72819916
PARP11	12	complement(3791047..3873448)	0,72	0,60	0,482555002
CCND2	12	4269771..4305353	0,00	-	3,967268276
gene:ENSG000	12	4273762..4405490	0,02	0,02	-0,291161992
TIGAR	12	4307763..4360028	0,19	0,43	-0,948198048
FGF23	12	complement(4368227..4379712)	-	0,00	-2,89252005
FGF6	12	complement(4428155..4445815)	-	-	#N/D
C12orf4	12	complement(4487735..4538508)	1,24	2,58	-0,838140391
RAD51AP1	12	4538798..4560047	3,66	4,83	-0,177200269
DYRK4	12	4562208..4615302	1,56	2,27	-0,312586263
gene:ENSG000	12	4604944..4662643	6,64	12,31	-0,667028286
AKAP3	12	complement(4615508..4649051)	0,02	0,03	-0,253352605
NDUFA9	12	4649095..4694317	3,47	3,74	0,116271354
gene:ENSG000	12	4649101..4774184	3,96	5,52	-0,257675437
GALNT8	12	4720400..4851927	-	0,01	-3,804623889
KCNA6	12	4809334..4813318	-	-	0,188027316
KCNA1	12	4909905..4918256	0,08	0,03	1,585398399
KCNA5	12	5043879..5046788	-	-	0,188027316
NTF3	12	5432108..5521536	10,33	8,54	0,498084482
ANO2	12	complement(5531869..5946232)	0,00	0,03	-3,021611439
VWF	12	complement(5948877..6124770)	0,05	0,34	-2,501917256
CD9	12	6199715..6238271	18,75	23,41	-0,097195216
PLEKHG6	12	6310436..6328506	0,88	0,56	0,878173205
TNFRSF1A	12	complement(6328757..6342114)	101,37	51,86	1,190032861
SCNN1A	12	complement(6346843..6377730)	0,91	1,79	-0,751753197
LTBR	12	6375045..6391571	46,50	48,56	0,16053661
CD27	12	6444955..6451718	0,40	0,42	0,160842886
TAPBPL	12	6451690..6466517	1,06	1,41	-0,189286641
VAMP1	12	complement(6462237..6470677)	13,00	6,48	1,227127084
MRPL51	12	complement(6491886..6493841)	26,88	35,96	-0,196826434
NCAPD2	12	6493356..6531955	113,67	#####	0,134862111
GAPDH	12	6534512..6538374	#######	#####	0,762228173
IFFO1	12	complement(6538961..6556073)	6,05	6,34	0,15634291

NOP2	12	complement(6556863..6568691)	15,39	16,16	0,15244889
gene:ENSG000	12	complement(6556886..6607367)	0,29	0,58	-0,776113215
CHD4	12	complement(6570082..6614524)	35,33	38,56	0,096808009
LPAR5	12	complement(6618835..6635960)	0,08	0,22	-1,196510235
ACRBP	12	complement(6638075..6647433)	0,09	0,11	-0,038943307
ING4	12	complement(6650301..6663142)	11,82	6,91	0,997549139
ZNF384	12	complement(6666477..6689572)	14,37	11,40	0,557271502
PIANP	12	complement(6693791..6700815)	0,52	0,89	-0,556702828
COPS7A	12	6724014..6731875	26,86	27,36	0,196282032
MLF2	12	complement(6747996..6767475)	98,73	93,66	0,29908165
PTMS	12	6765516..6770952	141,84	#####	0,226083115
LAG3	12	6772512..6778455	0,35	0,34	0,275773946
CD4	12	6786858..6820799	0,24	0,49	-0,783447034
GPR162	12	6821624..6829972	0,66	1,90	-1,287946554
P3H3	12	6828407..6839847	19,99	22,17	0,073883237
GNB3	12	6839954..6847393	3,39	3,35	0,239521204
CDCA3	12	complement(6844793..6851292)	10,57	12,66	-0,037908727
USP5	12	6852148..6866632	43,72	49,83	0,034137879
TPI1	12	6867119..6870948	234,90	#####	0,553794137
SPSB2	12	complement(6870935..6889358)	1,95	3,09	-0,444127252
LRRC23	12	6873569..6914241	3,47	2,34	0,789426929
ENO2	12	6913745..6923698	20,60	13,41	0,842438609
ATN1	12	6924463..6942321	47,37	42,67	0,373832288
C12orf57	12	6942978..6946003	18,62	18,05	0,26801976
PTPN6	12	6946468..6961316	0,02	0,06	-1,389560774
PHB2	12	complement(6965327..6970780)	56,17	68,83	-0,070206828
EMG1	12	6970913..6979936	9,04	9,00	0,230222634
gene:ENSG000	12	6970921..6997428	8,78	8,47	0,275215085
LPCAT3	12	complement(6976185..7018477)	8,06	7,06	0,41373793
C1S	12	6988259..7071032	5,27	13,93	-1,179561903
C1R	12	complement(7080214..7092540)	15,28	36,22	-1,022301068
C1RL	12	complement(7089587..7109238)	1,63	2,42	-0,347660929
RBP5	12	complement(7115736..7128889)	-	-	0,188027316
CLSTN3	12	7129698..7158945	0,74	0,22	1,965380471
PEX5	12	7188685..7218574	8,28	11,16	-0,208008503
ACSM4	12	7304072..7328719	-	0,04	-5,734998842
CD163L1	12	complement(7346685..7479897)	1,02	2,31	-0,962301468
CD163	12	complement(7470811..7503893)	-	-	0,188027316
APOBEC1	12	complement(7649400..7665908)	-	-	#N/D
GDF3	12	complement(7689784..7695775)	-	-	#N/D
DPPA3	12	7711433..7717559	-	-	#N/D
CLEC4C	12	complement(7729383..7751605)	-	-	#N/D
NANOGNB	12	7765216..7774121	-	-	#N/D
NANOG	12	7787794..7799146	0,00	0,01	-1,291882393
SLC2A14	12	complement(7812512..7891148)	0,00	-	3,068695355
SLC2A3	12	complement(7919230..8019007)	0,01	0,02	-0,602143793
FOXJ2	12	8032716..8055517	9,61	9,44	0,247602388
C3AR1	12	complement(8056844..8066359)	0,04	0,14	-1,507780808

NECAP1	12	8076939..8097881	4,38	4,08	0,327661569
gene:ENSG000	12	complement(8080381..8082163)	0,09	0,15	-0,520458053
CLEC4A	12	8123617..8138607	0,22	0,16	0,710337568
gene:ENSG000	12	8138140..8175320	-	-	#N/D
ZNF705A	12	8157015..8188537	0,00	0,01	-0,923955849
FAM90A1	12	complement(8221260..8227618)	3,19	3,19	0,223679513
CLEC6A	12	8455962..8478330	-	-	#N/D
CLEC4D	12	8509475..8522366	-	-	0,188027316
CLEC4E	12	complement(8533305..8540905)	-	-	#N/D
AICDA	12	complement(8602155..8613242)	0,00	-	3,068695355
gene:ENSG000	12	complement(8631545..8632259)	0,03	-	3,967268276
MFAP5	12	complement(8637346..8662888)	0,00	0,00	-0,01185201
RIMKLB	12	8681600..8783095	1,65	1,29	0,575996826
A2ML1	12	8822621..8887001	-	-	0,188027316
PHC1	12	8913896..8941467	5,69	6,85	-0,045104757
M6PR	12	complement(8940361..8949761)	19,27	26,18	-0,218806173
KLRG1	12	8950044..9010760	0,20	0,35	-0,529096263
A2M	12	complement(9067664..9116229)	0,01	-	5,480421266
PZP	12	complement(9148840..9208395)	0,00	-	3,068695355
KLRB1	12	complement(9594551..9607916)	0,01	0,04	-0,979341556
CLEC2D	12	9664969..9699553	0,24	0,43	-0,612499492
CD69	12	complement(9752486..9760901)	0,04	0,01	1,893574746
KLRF1	12	9827481..9845007	0,01	0,05	-2,190455314
CLEC2B	12	complement(9852369..9869386)	5,26	2,66	1,206404045
KLRF2	12	9881489..9895833	-	-	#N/D
CLEC2A	12	complement(9898673..9932370)	0,01	-	3,068695355
CLEC12A	12	9951316..9995694	-	-	0,188027316
CLEC1B	12	complement(9985642..10013424)	-	-	#N/D
CLEC12B	12	10010627..10018796	0,01	0,02	-1,54862452
CLEC9A	12	10030678..10066031	-	-	0,188027316
CLEC1A	12	complement(10069554..10111627)	0,03	0,07	-1,026620426
CLEC7A	12	complement(10116777..10130258)	0,02	0,02	0,278120973
OLR1	12	complement(10158301..10172138)	1,62	3,29	-0,798883278
TMEM52B	12	10170542..10191804	0,05	0,10	-0,868999748
GABARAPL1	12	10212458..10223128	10,93	11,71	0,122604178
KLRD1	12	10226058..10329608	0,01	0,01	1,021047459
KLRK1	12	complement(10372353..10391874)	0,17	0,13	0,623580048
KLRC4-KLRK1	12	complement(10372353..10410146)	0,04	0,01	2,060408965
KLRC4	12	complement(10407384..10409757)	0,14	0,27	-0,695225492
gene:ENSG000	12	complement(10412312..10436001)	0,36	-	7,909414018
KLRC3	12	complement(10412315..10420595)	0,33	0,27	0,50186296
KLRC2	12	complement(10426854..10442300)	0,26	0,47	-0,61018844
KLRC1	12	complement(10442264..10454685)	-	-	0,188027316
EIF2S3B	12	10505602..10523135	0,01	0,02	-1,54862452
MAGOHB	12	complement(10604193..10613609)	1,70	2,08	-0,074491846
STYK1	12	complement(10618923..10674318)	0,05	0,09	-0,619784286
YBX3	12	complement(10699089..10723323)	57,01	73,34	-0,140453333
TAS2R7	12	complement(10801532..10802627)	-	-	0,188027316

TAS2R8	12	complement(10806051..10807286)	-	-	0,188027316
TAS2R9	12	complement(10809094..10810168)	-	-	0,188027316
PRH1	12	complement(10824960..11171608)	0,25	0,51	-0,785856194
TAS2R10	12	complement(10825219..10826358)	0,02	0,02	-0,025382928
PRR4	12	complement(10845849..10849475)	-	0,02	-2,89252005
gene:ENSG000	12	complement(10845849..11171600)	1,92	3,28	-0,548638262
TAS2R13	12	complement(10907926..10909562)	0,03	0,13	-1,686357352
PRH2	12	10929236..10934845	0,02	0,12	-2,066140368
TAS2R14	12	complement(10937408..11171573)	0,13	0,27	-0,824193273
TAS2R50	12	complement(10985913..10986912)	0,09	0,23	-1,193454645
TAS2R20	12	complement(10995961..10998304)	0,53	0,61	0,023459852
TAS2R19	12	complement(11021619..11022620)	0,37	0,71	-0,731640816
TAS2R31	12	complement(11030387..11031407)	0,29	0,53	-0,674539852
TAS2R46	12	complement(11061365..11062294)	0,22	0,14	0,877784832
TAS2R43	12	complement(11091287..11092313)	0,03	0,04	-0,03031053
TAS2R30	12	complement(11132958..11134644)	0,19	0,32	-0,523371639
SMIM10L1	12	11171194..11176016	0,69	1,69	-1,063615258
TAS2R42	12	complement(11185993..11186937)	0,01	-	3,068695355
PRB3	12	complement(11265914..11269707)	0,01	0,01	-0,01185201
PRB4	12	complement(11307077..11310436)	0,04	0,01	1,436225396
PRB1	12	complement(11351823..11355591)	-	-	#N/D
PRB2	12	complement(11391540..11501041)	-	0,03	-4,359055975
ETV6	12	11649674..11895377	3,18	6,24	-0,747047772
BCL2L14	12	12049844..12211084	0,01	0,04	-2,272922624
LRP6	12	complement(12116025..12267044)	5,44	6,77	-0,092116756
MANSC1	12	complement(12326056..12350242)	0,20	0,55	-1,26979519
BORCS5	12	12357078..12471233	1,63	1,75	0,113120544
DUSP16	12	complement(12473282..12562863)	3,73	5,49	-0,334966805
CREBL2	12	12611827..12645108	2,25	4,34	-0,724308896
GPR19	12	complement(12660890..12696207)	-	-	0,188027316
CDKN1B	12	12685498..12722369	5,88	5,29	0,376694551
APOLD1	12	12725917..12829975	3,20	4,44	-0,250138
DDX47	12	12813316..12829981	31,96	30,77	0,277752418
GPRC5A	12	12890782..12917937	2,34	13,60	-2,317611799
GPRC5D	12	complement(12940575..12952170)	0,07	0,18	-1,104061491
HEBP1	12	complement(12974870..13000265)	6,56	9,63	-0,331304
HTR7P1	12	13000420..13004830	0,60	0,82	-0,226712782
FAM234B	12	13044381..13142521	1,73	3,35	-0,730708484
GSG1	12	complement(13083532..13103683)	0,06	0,20	-1,61577177
gene:ENSG000	12	complement(13088784..13103484)	-	-	#N/D
EMP1	12	13196723..13219941	9,58	17,24	-0,624356918
GRIN2B	12	complement(13437942..13982002)	0,01	0,01	-0,039657841
gene:ENSG000	12	complement(13979928..13980895)	-	-	#N/D
ATF7IP	12	14365676..14502931	3,34	5,39	-0,468672185
PLBD1	12	complement(14503661..14567883)	0,01	0,01	-0,01185201
GUCY2C	12	complement(14612632..14696599)	-	-	0,188027316
H4C16	12	complement(14767999..14771131)	1,13	3,71	-1,492343434
H2AJ	12	14774405..14778002	0,92	1,18	-0,138901521

WBP11	12	complement(14784582..14803486)	10,19	15,28	-0,361635766
C12orf60	12	14803666..14906586	0,25	0,35	-0,256072112
SMCO3	12	complement(14804650..14814182)	0,09	0,11	-0,039050201
ART4	12	complement(14825569..14843526)	0,05	0,04	0,393705318
MGP	12	complement(14880864..14885857)	2,80	0,89	1,870535708
ERP27	12	complement(14914039..14938537)	-	0,01	-2,89252005
ARHGDIB	12	complement(14942031..14961728)	1,54	4,44	-1,301428762
PDE6H	12	14973042..14981865	-	-	0,188027316
RERG	12	complement(15107783..15348675)	0,28	0,05	2,800874173
PTPRO	12	15322257..15602175	0,03	0,04	-0,004568323
EPS8	12	complement(15620134..15882329)	9,08	11,24	-0,083767404
STRAP	12	15882387..15903478	53,35	94,54	-0,602171488
DERA	12	15911302..16037381	6,06	11,26	-0,669077678
SLC15A5	12	complement(16188485..16277685)	0,00	-	3,068695355
MGST1	12	16347142..16609259	19,37	38,69	-0,774911111
LMO3	12	complement(16548372..16610594)	0,01	0,19	-4,092949529
RERGL	12	complement(18080869..18320107)	-	0,08	-5,545746556
PIK3C2G	12	18242961..18648416	0,00	0,02	-3,171495545
PLCZ1	12	complement(18683169..18738100)	-	0,00	-2,89252005
CAPZA3	12	18738119..18739188	-	-	0,188027316
PLEKHA5	12	19129669..19376400	1,45	4,14	-1,292631288
AEBP2	12	19404045..19720801	2,11	2,83	-0,198271631
PDE3A	12	20368537..20688583	0,26	0,11	1,436812364
SLCO1C1	12	20695332..20753386	0,01	0,00	0,886720911
SLCO1B3	12	20810702..20916911	-	-	0,188027316
SLCO1B3-SLC(	12	20815674..21090245	-	-	0,188027316
gene:ENSG000	12	21047179..21176895	-	-	0,188027316
SLCO1B1	12	21131194..21239796	-	-	0,188027316
SLCO1A2	12	complement(21264600..21419594)	-	0,00	-2,89252005
IAPP	12	21354959..21379980	0,02	0,01	1,436225396
PYROXD1	12	21437615..21471250	0,86	1,90	-0,912136014
RECQL	12	complement(21468910..21501669)	15,31	14,59	0,293053799
GOLT1B	12	21501781..21518408	11,82	9,79	0,494740967
SPX	12	21526296..21541249	0,12	2,28	-3,981992128
GYS2	12	complement(21536107..21604847)	-	-	#N/D
gene:ENSG000	12	complement(21536203..21657798)	-	0,03	-6,052163584
LDHB	12	complement(21635342..21757857)	171,54	#####	-0,385356648
KCNJ8	12	complement(21764955..21775600)	0,46	3,53	-2,711422163
ABCC9	12	complement(21797389..21942543)	0,35	1,08	-1,426380073
CMAS	12	22046218..22065674	13,41	22,85	-0,545826314
ST8SIA1	12	complement(22063773..22437041)	0,46	0,95	-0,83632377
C2CD5	12	complement(22448583..22544546)	2,35	6,44	-1,229333111
ETNK1	12	22625075..22690665	2,57	3,66	-0,283091703
SOX5	12	complement(23529504..24562544)	0,14	0,27	-0,751822462
BCAT1	12	complement(24810024..24949101)	6,35	17,51	-1,240910202
gene:ENSG000	12	24902309..24904681	0,04	0,11	-1,24748841
IRAG2	12	25004342..25108335	0,00	0,00	-0,01185201
DNAI7	12	complement(25108289..25195162)	0,10	0,03	2,157702232

ETFRF1	12	25195216..25209645	0,73	0,49	0,797775468
KRAS	12	complement(25205246..25250936)	2,64	3,23	-0,067164595
LMNTD1	12	complement(25409307..25648579)	0,01	0,04	-1,573534033
gene:ENSG000	12	25423600..25424942	-	-	0,188027316
RASSF8	12	25958232..26079892	3,18	4,99	-0,430109737
BHLHE41	12	complement(26120030..26125037)	0,16	1,75	-3,190245209
SSPN	12	26121991..26299290	0,09	0,11	-0,075560925
ITPR2	12	complement(26335352..26833194)	0,82	1,37	-0,512999762
INTS13	12	complement(26905181..26938326)	7,28	12,68	-0,576965103
FGFR1OP2	12	26938470..26966650	2,71	3,94	-0,317945567
TM7SF3	12	complement(26971579..27014434)	7,48	11,78	-0,433039532
MED21	12	27022546..27066343	3,39	5,14	-0,375704511
C12orf71	12	complement(27081057..27082578)	0,01	-	3,068695355
STK38L	12	27243968..27325959	0,64	1,89	-1,342663479
BMAL2	12	27332836..27425289	3,19	6,58	-0,821713755
SMCO2	12	27446736..27502185	0,02	0,10	-1,723418138
PPFIBP1	12	27523431..27695564	5,58	10,19	-0,644591189
REP15	12	27696447..27697596	0,08	0,32	-1,660236228
MRPS35	12	27710822..27756295	16,77	26,71	-0,448062924
MANSC4	12	complement(27762427..27780236)	0,05	0,11	-1,022609537
KLHL42	12	27780048..27803040	3,58	5,14	-0,300374922
PTHLH	12	complement(27958084..27972733)	0,01	0,16	-3,744539351
CCDC91	12	28133249..28581511	1,63	3,35	-0,820448704
FAR2	12	29149016..29341121	5,17	11,00	-0,865104362
ERGIC2	12	complement(29337352..29381189)	4,58	5,52	-0,04615049
OVCH1	12	complement(29412474..29497686)	-	0,03	-6,187931073
TMTC1	12	complement(29500840..29784759)	0,86	5,89	-2,558511652
IPO8	12	complement(30628988..30695869)	8,95	13,17	-0,333715039
CAPRIN2	12	complement(30709552..30823302)	3,38	5,73	-0,536317905
TSPAN11	12	30926428..30996602	0,87	3,55	-1,799922077
DDX11	12	31073860..31104799	28,66	32,11	0,059345165
SINHCAF	12	complement(31280584..31327058)	12,52	27,58	-0,916468312
DENND5B	12	complement(31382226..31591136)	0,92	2,83	-1,404231981
AK4P3	12	complement(31615023..31652095)	1,10	1,56	-0,277603182
ETFBKMT	12	31647160..31673114	0,18	0,22	-0,0773914
AMN1	12	complement(31671142..31729121)	3,15	2,48	0,569755847
H3-5	12	complement(31791185..31792298)	0,01	0,02	-0,923955849
RESF1	12	31959370..31993107	2,04	3,25	-0,451302664
BICD1	12	32106835..32383633	2,94	3,15	0,123722292
FGD4	12	32399558..32646050	0,14	0,35	-1,116256757
DNM1L	12	32679200..32745650	7,81	11,74	-0,365188028
YARS2	12	complement(32727490..32755897)	7,68	10,21	-0,189017045
PKP2	12	complement(32790745..32896798)	0,29	0,17	1,001768153
SYT10	12	complement(33374238..33439819)	-	-	#N/D
ALG10	12	34022468..34029694	1,54	2,40	-0,419213491
ALG10B	12	38316762..38329721	0,85	0,92	0,105256292
CPNE8	12	complement(38646822..38907430)	2,18	4,15	-0,708060546
KIF21A	12	complement(39293228..39443390)	0,83	2,18	-1,176494012

ABCD2	12	complement(39550033..39619803)	0,02	0,01	0,319286298
REDIC1	12	39626167..39908300	-	0,00	-2,89252005
SLC2A13	12	complement(39755025..40106089)	0,60	0,69	0,024083222
LRRK2	12	40196744..40369285	0,10	0,30	-1,324176809
MUC19	12	40393395..40570832	-	-	0,188027316
CNTN1	12	40692439..41072415	0,01	-	4,913749003
PDZRN4	12	41188320..41574745	-	-	0,188027316
GXYLT1	12	complement(42081845..42144874)	0,34	0,71	-0,849501655
YAF2	12	complement(42157104..42238349)	1,01	1,18	0,000394449
PPHLN1	12	42238447..42459715	3,01	5,03	-0,519608204
ZCRB1	12	complement(42312086..42326257)	4,02	5,26	-0,162581986
PRICKLE1	12	complement(42456757..42590355)	0,06	0,03	1,028629528
ADAMTS20	12	complement(43353866..43552203)	0,05	-	7,993912231
PUS7L	12	complement(43718992..43758793)	0,65	0,96	-0,330463355
IRAK4	12	43758944..43798307	0,79	1,39	-0,586223097
TWF1	12	complement(43793723..43806328)	8,70	13,15	-0,373711583
TMEM117	12	43835967..44389762	0,19	0,38	-0,811114705
NELL2	12	complement(44508275..44921848)	-	0,00	-2,89252005
DBX2	12	complement(45014672..45051099)	-	-	0,188027316
ANO6	12	45215987..45482280	9,57	10,67	0,0666814
ARID2	12	45729706..45908040	2,97	4,88	-0,494142403
SCAF11	12	complement(45919131..45992120)	5,98	6,40	0,125443621
SLC38A1	12	complement(46183063..46270017)	12,80	24,39	-0,706630187
SLC38A2	12	complement(46358188..46372773)	109,76	53,38	1,263023496
gene:ENSG000	12	46404644..46407166	0,51	0,45	0,406267516
SLC38A4	12	complement(46764761..46832408)	0,51	0,35	0,773238246
AMIGO2	12	complement(47075707..47079959)	6,78	7,11	0,153998098
PCED1B	12	47079603..47236662	4,02	2,88	0,703880003
RPAP3	12	complement(47661249..47706030)	3,45	5,50	-0,449873098
ENDOU	12	complement(47709734..47725567)	0,10	0,08	0,480462838
gene:ENSG000	12	complement(47728151..47730598)	0,19	0,32	-0,543261024
RAPGEF3	12	complement(47734363..47771040)	2,95	2,86	0,267536638
SLC48A1	12	47753916..47782751	7,28	12,72	-0,583252817
HDAC7	12	complement(47782722..47833132)	23,54	24,45	0,168697669
VDR	12	complement(47841537..47943048)	3,74	6,14	-0,491322521
gene:ENSG000	12	complement(47960497..47963469)	-	-	#N/D
TMEM106C	12	47963569..47968878	32,84	29,58	0,374004785
COL2A1	12	complement(47972967..48004554)	0,00	0,01	-1,478387936
SENP1	12	complement(48039784..48106079)	2,76	5,21	-0,69199976
PFKM	12	48105139..48146404	14,74	21,37	-0,312780656
ASB8	12	complement(48147789..48181213)	4,07	4,12	0,208294499
CCDC184	12	48183644..48185926	-	-	0,188027316
gene:ENSG000	12	48189349..48191207	-	-	#N/D
OR10AD1	12	complement(48202339..48203292)	-	-	0,188027316
H1-7	12	48328980..48330279	-	-	0,188027316
ZNF641	12	complement(48337180..48351414)	0,15	0,08	1,148287281
ANP32D	12	48472559..48473622	-	-	#N/D
C12orf54	12	48482498..48496524	-	-	#N/D

OR8S1	12	48525632..48526570	-	-	#N/D
OR5BS1P	12	48559882..48562956	-	-	#N/D
LALBA	12	complement(48567684..48570066)	-	-	#N/D
KANSL2	12	complement(48653211..48682238)	6,09	8,70	-0,291198887
CCNT1	12	complement(48688458..48716998)	12,33	8,13	0,824159234
SPMIP11	12	48727435..48765790	-	-	0,188027316
ADCY6	12	complement(48766194..48789089)	13,03	9,65	0,656757512
CACNB3	12	48813794..48828941	5,07	4,86	0,283914675
DDX23	12	complement(48829756..48852842)	15,35	16,38	0,129155951
RND1	12	complement(48857145..48865870)	0,22	0,21	0,289897675
gene:ENSG000	12	complement(48903503..48957365)	5,02	0,89	2,714589504
CCDC65	12	48904110..48931840	1,50	0,70	1,312923798
FKBP11	12	complement(48921518..48926474)	3,46	2,18	0,891606873
ARF3	12	complement(48935723..48957487)	36,24	35,04	0,271503556
WNT10B	12	complement(48965340..48971735)	0,42	0,81	-0,716151339
WNT1	12	48978322..48982620	-	-	0,188027316
DDN	12	complement(48995149..48999375)	0,06	0,04	0,577093021
PRKAG1	12	complement(49002274..49018807)	12,21	12,17	0,228103087
gene:ENSG000	12	complement(49002274..49024075)	0,32	0,27	0,424316905
KMT2D	12	complement(49018975..49060794)	11,80	13,39	0,040422671
RHEBL1	12	complement(49064676..49070025)	1,33	2,71	-0,805712624
DHH	12	complement(49086656..49094801)	0,05	0,06	0,097248751
LMBR1L	12	complement(49097136..49110900)	4,36	4,76	0,094652324
TUBA1B	12	complement(49127782..49131397)	802,17	#####	0,162032642
TUBA1A	12	complement(49184686..49189324)	29,40	46,00	-0,422674218
TUBA1C	12	49188736..49274600	156,76	#####	-0,03520146
PRPH	12	49295147..49298686	4,57	3,22	0,725840206
TROAP	12	49323236..49331731	15,12	21,65	-0,294789207
C1QL4	12	complement(49332409..49337188)	1,06	2,00	-0,689879305
DNAJC22	12	49346888..49357546	3,07	3,65	-0,024076839
SPATS2	12	49366584..49527425	4,69	6,34	-0,212782661
KCNH3	12	49539030..49558337	0,21	0,48	-0,973920904
MCRS1	12	complement(49556544..49568145)	22,77	28,06	-0,078265259
PRPF40B	12	49568218..49644666	4,64	4,37	0,307737361
FAM186B	12	complement(49582885..49605639)	0,42	0,36	0,438051765
FMNL3	12	complement(49636499..49708165)	5,20	5,14	0,238525389
TMBIM6	12	49707725..49764934	107,73	#####	0,150186226
NCKAP5L	12	complement(49791146..49828750)	8,43	10,01	-0,024708634
BCDIN3D	12	complement(49836043..49843106)	0,53	1,06	-0,780794871
FAIM2	12	complement(49866896..49904217)	0,00	-	3,068695355
AQP2	12	49950737..49958878	-	0,01	-3,804623889
AQP5	12	49961872..49965682	-	-	0,188027316
AQP6	12	49967194..49977139	0,02	0,02	0,221270597
RACGAP1	12	complement(49976923..50033136)	37,98	32,24	0,459230032
ASIC1	12	50057548..50083611	2,14	2,99	-0,261059128
SMARCD1	12	50085200..50100707	19,71	33,05	-0,522574184
GPD1	12	50103982..50111313	0,09	0,08	0,353940309
COX14	12	50112082..50120457	11,83	8,70	0,665587739

CERS5	12	complement(50129289..50167533)	7,37	6,17	0,479066731
LIMA1	12	complement(50175788..50283520)	22,74	25,24	0,072908233
FAM186A	12	complement(50326230..50396622)	0,03	0,04	-0,289384009
LARP4	12	50392383..50480004	3,70	7,35	-0,766756205
DIP2B	12	50504985..50748657	6,22	9,05	-0,317527483
ATF1	12	50763710..50821162	4,07	7,48	-0,655636702
TMPRSS12	12	50842920..50887884	-	0,00	-2,89252005
TMT1A	12	50923472..50932510	0,35	0,66	-0,714014957
gene:ENSG000	12	50924969..50970700	-	0,04	-5,071123353
HIGD1C	12	50952538..50970607	-	-	0,188027316
SLC11A2	12	complement(50979401..51028566)	7,28	9,20	-0,113289164
LETMD1	12	51047962..51060424	16,00	14,08	0,406940587
CSRNP2	12	complement(51061205..51083664)	3,03	5,37	-0,602793421
TFCP2	12	complement(51093656..51173135)	3,78	4,13	0,093553596
POU6F1	12	complement(51186936..51218062)	0,92	1,15	-0,094019119
DAZAP2	12	51238724..51271362	28,90	25,30	0,414809964
SMAGP	12	complement(51244558..51270415)	2,84	5,10	-0,62216769
BIN2	12	complement(51281038..51324668)	0,00	-	3,068695355
CELA1	12	complement(51328442..51346679)	-	-	0,188027316
GALNT6	12	complement(51351247..51392867)	0,04	0,05	-0,100498275
SLC4A8	12	51391317..51515763	0,30	0,35	-0,000617537
SCN8A	12	51590266..51812864	0,23	0,29	-0,098977305
TMDD1	12	complement(51813921..51814925)	-	-	0,188027316
FIGNL2	12	complement(51817899..51848718)	0,45	0,30	0,80650109
ANKRD33	12	51888009..51891727	-	-	#N/D
ACVRL1	12	51906908..51923361	2,08	0,50	2,293109178
ACVR1B	12	51951699..51997078	2,73	4,28	-0,426842584
TAMALIN	12	52006946..52015889	0,10	0,11	0,038295137
NR4A1	12	52022832..52059507	3,40	4,78	-0,267948223
ATG101	12	52069246..52077495	33,47	21,92	0,833813887
SMIM41	12	52079704..52108255	0,02	0,03	-0,579815015
KRT80	12	complement(52168996..52192014)	9,17	48,17	-2,170830531
KRT7	12	52232520..52252186	4,03	36,14	-2,939830245
KRT86	12	52249300..52309163	0,65	3,42	-2,171736996
KRT81	12	complement(52285913..52291534)	11,26	58,00	-2,141927391
KRT83	12	complement(52314301..52321398)	0,02	0,04	-0,999120035
KRT85	12	complement(52360006..52367481)	0,00	0,04	-2,854330803
KRT84	12	complement(52377812..52385652)	-	-	#N/D
KRT82	12	complement(52393931..52406335)	-	-	#N/D
KRT75	12	complement(52424070..52434371)	-	0,89	-9,931489205
KRT6B	12	complement(52446651..52452146)	-	0,01	-2,89252005
KRT6C	12	complement(52468516..52473805)	-	-	#N/D
KRT6A	12	complement(52487176..52493257)	-	-	0,188027316
KRT5	12	complement(52514575..52520530)	-	-	0,188027316
KRT71	12	complement(52543909..52553145)	-	-	0,188027316
KRT74	12	complement(52565782..52573843)	-	-	#N/D
KRT72	12	complement(52585589..52601538)	-	-	#N/D
KRT73	12	complement(52607570..52618559)	-	-	0,188027316

KRT2	12	complement(52644558..52652211)	-	-	0,188027316
KRT1	12	complement(52674736..52680407)	-	-	#N/D
KRT77	12	complement(52689626..52703524)	-	-	#N/D
KRT76	12	complement(52768155..52777345)	-	-	#N/D
KRT3	12	complement(52789685..52796117)	0,01	-	3,967268276
KRT4	12	complement(52806549..52814116)	0,01	-	3,068695355
KRT79	12	complement(52821408..52834311)	0,04	0,04	-0,036800208
KRT78	12	complement(52837804..52849092)	0,01	0,02	-0,429837071
KRT8	12	complement(52897187..52949954)	10,55	11,27	0,127864995
KRT18	12	52948871..52952906	17,64	32,57	-0,661555694
EIF4B	12	53006282..53042215	147,98	#####	0,428670639
TNS2	12	53046969..53064379	12,70	11,08	0,42019931
SPRYD3	12	complement(53064316..53079404)	23,47	11,96	1,195261576
IGFBP6	12	53097436..53102345	16,93	32,69	-0,725543126
SOAT2	12	53103486..53124535	-	0,01	-2,89252005
CSAD	12	complement(53157663..53180925)	2,74	2,03	0,65238777
ZNF740	12	53180704..53195142	4,62	6,50	-0,271505852
ITGB7	12	complement(53191323..53207282)	0,02	0,06	-1,022609537
RARG	12	complement(53210567..53232980)	26,22	20,12	0,605134779
gene:ENSG000	12	53241900..53242722	-	-	0,188027316
MFSD5	12	53251251..53254406	7,15	7,75	0,107091228
ESPL1	12	53268299..53293638	10,25	22,13	-0,88805862
PFDN5	12	53295291..53299452	74,95	68,13	0,36067482
MYG1	12	53299695..53307177	28,29	29,20	0,177511669
AAAS	12	complement(53307456..53324864)	31,89	32,26	0,206630855
SP7	12	complement(53326575..53345315)	0,05	0,11	-0,891420415
SP1	12	53380176..53416446	17,09	17,73	0,170010923
AMHR2	12	53423855..53431672	-	0,09	-6,532127252
PRR13	12	53441678..53446645	39,74	45,94	0,014146209
PCBP2	12	53452102..53481162	68,96	84,00	-0,061574632
MAP3K12	12	complement(53479669..53500063)	5,63	5,71	0,203368685
TARBP2	12	53500921..53506431	12,91	14,83	0,023561044
NPFF	12	complement(53506688..53507484)	0,27	0,32	-0,037899139
ATF7-NPFF	12	complement(53506691..53625979)	0,15	0,48	-1,41761001
ATF7	12	complement(53507856..53626410)	13,51	12,87	0,293268753
ATP5MC2	12	complement(53632726..53677408)	83,87	82,89	0,239973987
CALCOCO1	12	complement(53708517..53727745)	7,22	4,05	1,057445783
HOXC13	12	53938831..53946544	6,25	6,61	0,14349869
HOXC12	12	53954903..53958956	1,69	3,92	-0,98900329
HOXC11	12	53973126..53977643	2,50	4,39	-0,589651268
HOXC10	12	53985065..53990279	13,50	12,86	0,293279883
gene:ENSG000	12	53985845..54034888	0,21	-	6,780090526
HOXC6	12	53990624..54030823	2,41	4,26	-0,597451782
HOXC9	12	53994895..54003337	1,73	2,23	-0,14178231
HOXC8	12	54008985..54012769	1,98	3,07	-0,408685494
HOXC4	12	54016931..54056030	1,97	2,66	-0,211437563
HOXC5	12	54033050..54035361	1,06	1,21	0,033739844
SMUG1	12	complement(54121277..54189008)	2,65	4,26	-0,46311915

CBX5	12	complement(54230942..54280133)	31,61	30,23	0,287790817
HNRNPA1	12	54280193..54287088	49,52	64,11	-0,149590406
NFE2	12	complement(54292111..54301015)	0,04	0,25	-2,37375073
COPZ1	12	54301202..54351846	62,10	60,22	0,267439246
GPR84	12	complement(54362445..54364487)	0,11	0,08	0,577093021
ZNF385A	12	complement(54369133..54391298)	3,08	11,18	-1,635431261
ITGA5	12	complement(54395261..54419266)	191,51	#####	0,239265815
GTSF1	12	complement(54455950..54473602)	-	-	0,188027316
NCKAP1L	12	54497752..54548243	0,04	0,04	0,230226832
PDE1B	12	54549601..54579239	0,01	0,01	0,524121557
PPP1R1A	12	complement(54575387..54588659)	-	-	#N/D
LACRT	12	complement(54630811..54634895)	-	-	#N/D
DCD	12	complement(54644589..54648493)	-	-	0,188027316
MUCL1	12	54830518..54896008	-	-	#N/D
TESPA1	12	complement(54948015..54984762)	-	-	0,188027316
NEUROD4	12	55019974..55030017	-	0,00	-2,89252005
OR9K2	12	55126406..55132750	-	-	#N/D
OR10A7	12	55221025..55221975	-	-	#N/D
OR6C74	12	55244609..55256625	-	-	0,188027316
OR6C6	12	complement(55293988..55296569)	-	-	#N/D
OR6C1	12	55314343..55322364	-	-	#N/D
OR6C3	12	55330043..55332687	-	-	#N/D
OR6C75	12	55362975..55369279	-	-	#N/D
OR6C65	12	55400529..55401467	-	-	#N/D
OR6C76	12	55426254..55427192	-	-	0,188027316
OR6C2	12	55444069..55453347	-	-	#N/D
OR6C70	12	complement(55469200..55470138)	-	-	#N/D
OR6C68	12	55492378..55493316	-	-	#N/D
OR6C4	12	55549602..55555832	-	-	#N/D
OR2AP1	12	55572468..55575612	-	-	#N/D
OR10P1	12	55636892..55637833	-	-	#N/D
TMT1B	12	55681736..55684611	0,37	0,57	-0,405313412
ITGA7	12	complement(55684568..55716404)	0,35	0,88	-1,102027444
gene:ENSG000	12	55716036..55724703	0,95	0,83	0,410278937
BLOC1S1	12	55716038..55720087	10,06	11,09	0,081942677
RDH5	12	55720367..55724705	0,15	0,41	-1,220681718
CD63	12	complement(55725323..55729707)	94,93	65,79	0,752113375
GDF11	12	55743122..55757264	1,69	1,78	0,144308149
SARNP	12	complement(55752463..55817724)	8,66	10,27	-0,022821681
gene:ENSG000	12	complement(55757275..55827546)	0,06	0,23	-1,819256723
ORMDL2	12	55818041..55821879	7,44	6,38	0,444910187
DNAJC14	12	complement(55820960..55830824)	10,91	11,97	0,088680243
MMP19	12	complement(55835433..55842966)	0,37	1,18	-1,431212208
PYM1	12	complement(55901413..55932618)	2,60	4,64	-0,612752634
DGKA	12	55927319..55954027	2,88	3,14	0,098478262
PMEL	12	complement(55954105..55973317)	0,27	0,25	0,341238756
CDK2	12	55966781..55972789	19,68	21,51	0,094388502
RAB5B	12	55973913..55996683	23,88	16,47	0,759157019

SUOX	12	55997180..56006641	1,66	1,89	0,03774291
IKZF4	12	56007659..56038435	1,00	1,04	0,171751211
RPS26	12	56041351..56044697	71,67	85,58	-0,03279969
ERBB3	12	56076799..56103505	0,05	0,19	-1,781691577
gene:ENSG000	12	56101331..56109289	-	0,08	-4,359055975
PA2G4	12	56104537..56113910	44,81	59,77	-0,192605376
RPL41	12	56116590..56117967	596,30	#####	0,036745261
ESYT1	12	56118250..56144674	27,16	26,20	0,27515402
ZC3H10	12	56118260..56127514	0,87	1,33	-0,377051565
MYL6B	12	56152256..56159647	23,23	27,19	-0,003994075
MYL6	12	56158346..56163496	50,00	43,48	0,424651085
SMARCC2	12	complement(56162359..56189567)	20,52	24,33	-0,022741749
RNF41	12	complement(56202179..56221933)	9,42	11,46	-0,06021222
NABP2	12	56222015..56229854	24,25	23,50	0,26876019
SLC39A5	12	56230049..56237846	0,08	0,10	-0,125580693
ANKRD52	12	complement(56237807..56258384)	23,46	31,08	-0,182481487
COQ10A	12	56266890..56270966	1,76	2,32	-0,174264943
CS	12	complement(56271699..56300391)	57,75	63,70	0,081623962
gene:ENSG000	12	complement(56285916..56316059)	4,83	5,59	0,012092495
CNPY2	12	complement(56309842..56316119)	6,21	8,53	-0,233463939
PAN2	12	complement(56316223..56334053)	15,44	14,47	0,316621114
IL23A	12	56334174..56340410	0,22	0,15	0,761197382
STAT2	12	complement(56341597..56360203)	18,01	8,06	1,382055494
APOF	12	complement(56360568..56362857)	0,08	0,04	1,316787807
TIMELESS	12	complement(56416363..56449426)	10,23	20,14	-0,753693689
MIP	12	complement(56449502..56469166)	-	0,00	-2,89252005
gene:ENSG000	12	complement(56449700..56488170)	0,01	-	4,516772761
SPRYD4	12	56468578..56479708	1,15	1,49	-0,144800046
GLS2	12	complement(56470944..56488414)	0,01	0,03	-1,955757882
RBMS2	12	56521820..56596193	9,97	9,71	0,261282254
BAZ2A	12	complement(56595596..56636816)	37,98	32,05	0,467874537
ATP5F1B	12	complement(56638175..56645984)	379,00	#####	-0,012844764
PTGES3	12	complement(56663341..56688408)	33,07	52,77	-0,451030869
NACA	12	complement(56712305..56731628)	18,40	24,17	-0,170237804
gene:ENSG000	12	complement(56714612..56741535)	0,04	0,40	-3,112412319
PRIM1	12	complement(56730438..56752374)	2,64	5,32	-0,787174005
HSD17B6	12	56752161..56787790	0,67	1,00	-0,352570699
SDR9C7	12	complement(56923133..56934408)	0,05	0,07	-0,037246625
RDH16	12	complement(56951431..56959374)	0,39	0,50	-0,153756153
GPR182	12	56994492..56998447	0,73	0,84	0,016560178
ZBTB39	12	complement(56998836..57006546)	3,50	4,76	-0,222011903
TAC3	12	complement(57010000..57028883)	0,01	0,02	-0,923955849
MYO1A	12	complement(57028517..57051198)	0,01	0,02	-1,291882393
NEMP1	12	complement(57055643..57088063)	8,41	10,14	-0,046461307
NAB2	12	57089043..57095476	11,06	7,50	0,783185558
STAT6	12	complement(57095408..57132139)	8,87	11,84	-0,193108903
LRP1	12	57128483..57213361	31,26	18,12	1,009850803
NXPH4	12	57216794..57226449	3,53	3,99	0,045936961

SHMT2	12	57229573..57234935	50,93	83,41	-0,488585075
NDUFA4L2	12	complement(57234903..57240715)	1,01	0,80	0,561237398
STAC3	12	complement(57243453..57251188)	0,20	0,50	-1,102790023
gene:ENSG000	12	complement(57249609..57296484)	0,25	0,99	-1,753672381
R3HDM2	12	complement(57253762..57431043)	11,50	19,10	-0,509236545
INHBC	12	57434784..57452062	0,01	0,11	-3,196523635
INHBE	12	57452323..57459280	0,13	5,89	-5,255912301
GLI1	12	57459785..57472268	0,24	0,50	-0,833155041
ARHGAP9	12	complement(57472264..57488814)	0,08	0,07	0,493326738
MARS1	12	57475445..57517569	36,79	80,00	-0,897816508
DDIT3	12	complement(57516588..57521737)	65,29	18,14	2,070815339
gene:ENSG000	12	complement(57517712..57520480)	-	-	#N/D
MBD6	12	57520710..57530148	10,16	11,88	-0,003050141
DCTN2	12	complement(57529633..57547224)	33,89	38,67	0,032541992
KIF5A	12	57546026..57586633	0,02	0,04	-0,451418876
PIP4K2C	12	57591174..57603418	10,68	10,18	0,29333897
DTX3	12	57604622..57609804	3,92	5,23	-0,192850628
ARHGEF25	12	57610180..57617245	7,26	11,21	-0,404222687
gene:ENSG000	12	57615280..57626125	0,56	0,28	1,254671068
B4GALNT1	12	complement(57623409..57633239)	5,96	7,78	-0,162114448
OS9	12	57693841..57728342	28,71	24,82	0,433277393
AGAP2	12	complement(57723761..57742157)	0,15	0,15	0,270448758
TSPAN31	12	57738013..57750219	4,63	4,81	0,167552915
CDK4	12	complement(57747727..57756013)	37,79	50,65	-0,199386398
MARCHF9	12	57755103..57760411	2,72	5,03	-0,664961311
CYP27B1	12	complement(57762334..57768986)	0,49	0,63	-0,124337438
METTL1	12	complement(57768471..57772119)	8,62	11,19	-0,152614394
EEF1AKMT3	12	57771492..57782541	7,08	4,11	1,007870986
gene:ENSG000	12	57772660..57797554	0,39	0,72	-0,650931469
TSFM	12	57782761..57808071	4,39	5,23	-0,029777987
AVIL	12	complement(57797376..57818734)	0,11	0,19	-0,529470812
CTDSP2	12	complement(57819927..57846729)	43,82	39,99	0,355317535
ATP23	12	57906039..57959148	0,84	0,96	0,031968631
gene:ENSG000	12	complement(58094914..58098013)	0,01	-	3,967268276
gene:ENSG000	12	58103907..58105935	-	-	#N/D
LRIG3	12	complement(58872149..58920504)	2,15	2,46	0,029889479
SLC16A7	12	59596029..59789855	1,54	2,46	-0,454945547
TAFA2	12	complement(61708273..62279150)	0,01	0,05	-2,075817774
USP15	12	62260338..62417431	0,97	1,46	-0,368002243
MON2	12	62466817..62600476	4,24	3,54	0,485818986
PPM1H	12	complement(62643994..62935150)	0,48	0,19	1,53790217
AVPR1A	12	complement(63142759..63151201)	0,00	-	3,068695355
gene:ENSG000	12	63151065..63154006	-	-	#N/D
DPY19L2	12	complement(63558913..63668939)	0,17	0,24	-0,240034333
RXYLT1	12	63779833..63809792	1,18	2,39	-0,796730863
SRGAP1	12	63843761..64162217	2,45	5,20	-0,858980001
KICS2	12	complement(64186316..64222296)	2,04	2,82	-0,243848249
C12orf56	12	complement(64264762..64390758)	0,02	0,03	-0,037246625

XPOT	12	64404392..64451125	21,72	46,69	-0,880899554
TBK1	12	64452090..64502114	1,29	2,00	-0,410681725
RASSF3	12	64507001..64697564	10,61	14,10	-0,187546769
gene:ENSG000	12	complement(64696191..64696550)	-	-	#N/D
gene:ENSG000	12	64709458..64710513	0,03	0,09	-1,21700111
GNS	12	complement(64713445..64759431)	26,43	25,58	0,270468559
TBC1D30	12	64759484..64881033	0,07	0,28	-1,683042916
gene:ENSG000	12	64759496..64977509	-	-	0,188027316
WIF1	12	complement(65050626..65121305)	-	-	0,188027316
LEMD3	12	65169583..65248355	3,49	4,39	-0,105553504
MSRB3	12	65278643..65491430	5,41	9,19	-0,54299426
HMGA2	12	65824460..65966291	2,62	6,04	-0,981122393
LLPH	12	complement(66116555..66130750)	0,92	1,03	0,066710094
gene:ENSG000	12	complement(66123917..66169985)	-	-	0,188027316
TMBIM4	12	complement(66135846..66170027)	2,81	4,08	-0,312283915
IRAK3	12	66189195..66254622	2,01	2,72	-0,216281992
HELB	12	66302493..66343643	1,07	1,25	-0,005473399
GRIP1	12	complement(66347431..67069162)	0,04	0,01	2,362275056
gene:ENSG000	12	complement(67065018..67065278)	-	-	0,188027316
CAND1	12	67269358..67319953	10,46	10,55	0,211464039
DYRK2	12	67648338..67665406	2,89	2,75	0,291685586
gene:ENSG000	12	67753701..67753817	-	-	#N/D
IFNG	12	complement(68154768..68159740)	-	-	#N/D
IL26	12	complement(68201349..68225810)	-	-	#N/D
IL22	12	complement(68248242..68253604)	-	-	#N/D
MDM1	12	complement(68272443..68332381)	0,67	1,00	-0,362594451
RAP1B	12	68610855..68671901	1,36	2,30	-0,532550638
NUP107	12	68686951..68745809	4,93	7,85	-0,448733043
SLC35E3	12	68746125..68793964	0,34	0,50	-0,321018826
MDM2	12	68808177..68845544	6,42	8,85	-0,241293833
CPM	12	complement(68842197..68971570)	5,05	3,39	0,798149091
CPSF6	12	69239569..69274358	5,80	7,91	-0,225702818
LYZ	12	69348381..69354234	0,03	0,01	2,144183439
YEATS4	12	69359710..69390870	0,90	2,53	-1,269371177
FRS2	12	69470349..69579793	2,00	2,47	-0,08218275
CCT2	12	69585426..69601570	37,41	62,28	-0,51219565
LRRC10	12	complement(69608564..69610907)	-	-	0,188027316
BEST3	12	complement(69643360..69699476)	-	0,01	-5,327865481
gene:ENSG000	12	complement(69660034..69661429)	-	-	0,188027316
RAB3IP	12	69738860..69823204	0,12	0,80	-2,477195344
gene:ENSG000	12	69801669..69855363	-	-	#N/D
MYRFL	12	69825227..69959097	-	0,01	-3,804623889
CNOT2	12	70243002..70354993	2,71	3,83	-0,273770979
gene:ENSG000	12	complement(70321542..70324274)	0,11	0,27	-0,985911557
KCNMB4	12	70366290..70434292	0,33	0,98	-1,355897675
PTPRB	12	complement(70515870..70637440)	0,04	0,23	-2,280369121
PTPRR	12	complement(70638073..70920738)	0,01	0,25	-4,135947501
TSPAN8	12	complement(71125085..71441898)	0,01	-	3,967268276

LGR5	12	71439798..71586310	-	0,01	-3,804623889
ZFC3H1	12	complement(71609599..71667725)	11,57	8,56	0,656746523
THAP2	12	71664301..71680644	0,73	0,75	0,1835506
gene:ENSG000	12	71674204..71698968	0,11	-	5,88622595
TMEM19	12	71686082..71705047	1,89	2,42	-0,134031702
RAB21	12	71754863..71800286	1,52	1,78	-0,004276811
TBC1D15	12	71839707..71927248	1,94	2,82	-0,315661025
TPH2	12	71938845..72186618	-	0,01	-3,804623889
TRHDE	12	72087266..72670758	1,60	0,86	1,123438544
ATXN7L3B	12	74537835..74545430	12,94	14,76	0,032845232
KCNC2	12	complement(75040077..75209839)	-	-	0,188027316
CAPS2	12	complement(75275979..75390928)	0,12	0,26	-0,919657005
GLIPR1L1	12	75334670..75370560	0,01	0,03	-1,478387936
GLIPR1L2	12	75391089..75432688	0,03	0,01	1,790975958
GLIPR1	12	75480753..75503863	4,57	5,90	-0,146055946
KRR1	12	complement(75490863..75511636)	1,71	2,93	-0,552701982
PHLDA1	12	complement(76025447..76031776)	79,96	90,24	0,048461174
NAP1L1	12	complement(76036585..76084735)	6,84	13,46	-0,753583876
BBS10	12	complement(76344474..76348415)	1,08	1,90	-0,590226225
OSBPL8	12	complement(76351797..76559809)	3,97	5,92	-0,35155437
ZDHHC17	12	76764103..76853696	1,42	2,48	-0,579171938
CSRP2	12	complement(76858709..76879023)	15,10	9,29	0,924214118
E2F7	12	complement(77021248..77065569)	14,40	6,12	1,456987055
NAV3	12	77324641..78213010	13,29	10,76	0,527468851
SYT1	12	78863993..79452008	0,42	1,15	-1,241600711
PAWR	12	complement(79574979..79690964)	2,20	3,58	-0,482219412
PPP1R12A	12	complement(79773563..79935460)	5,81	7,93	-0,224694055
OTOGL	12	80099537..80380880	0,01	0,03	-1,660236228
PTPRQ	12	80402178..80680273	1,76	0,17	3,601297751
MYF6	12	80707634..80709474	-	-	0,188027316
MYF5	12	80716912..80719671	-	-	#N/D
LIN7A	12	complement(80792520..80937934)	0,15	0,18	-0,101835115
ACSS3	12	80936414..81261210	0,53	0,60	0,056071047
PPFIA2	12	complement(81257975..81759553)	0,01	0,26	-4,835542945
CCDC59	12	complement(82223681..82358805)	2,86	4,37	-0,388406001
METTL25	12	82358528..82479239	0,25	0,37	-0,306871848
TMTC2	12	82686880..83134870	1,26	1,54	-0,073899702
SLC6A15	12	complement(84859491..84913629)	4,28	7,25	-0,537085004
TSPAN19	12	complement(85014311..85036277)	0,02	0,04	-0,979341556
LRRIQ1	12	85036314..85264457	0,01	0,06	-2,634741553
ALX1	12	85280220..85301784	1,44	2,84	-0,75436345
RASSF9	12	complement(85800703..85836409)	0,18	0,27	-0,352489165
NTS	12	85874295..85882992	-	-	#N/D
MGAT4C	12	complement(85955666..86838904)	-	0,00	-2,89252005
gene:ENSG000	12	87041916..87042423	-	-	#N/D
C12orf50	12	complement(87980035..88034037)	-	-	0,188027316
RLIG1	12	88033846..88050160	0,94	2,28	-1,051726366
CEP290	12	complement(88049016..88142099)	0,42	0,89	-0,844363526

TMTC3	12	88142296..88199887	2,11	3,54	-0,524665191
KITLG	12	complement(88492793..88580851)	0,46	1,03	-0,935188496
DUSP6	12	complement(89347235..89352501)	5,37	6,08	0,042133185
POC1B	12	complement(89419718..89526047)	1,79	3,01	-0,52819945
POC1B-GALNT4	12	complement(89519408..89526262)	0,28	0,30	0,087549292
GALNT4	12	complement(89519412..89524796)	0,55	0,46	0,497589335
gene:ENSG000	12	complement(89544203..89544488)	0,08	0,32	-1,766505595
ATP2B1	12	complement(89588049..89709366)	3,11	5,24	-0,528733929
CCER1	12	complement(90905622..90955176)	-	-	#N/D
EPYC	12	complement(90963682..91005026)	-	-	#N/D
KERA	12	complement(91050491..91058024)	-	-	#N/D
LUM	12	complement(91102629..91111494)	0,01	0,01	-0,03031053
DCN	12	complement(91140484..91183217)	0,03	0,06	-0,949787713
BTG1	12	complement(92140278..92145846)	9,51	8,82	0,331308791
PLEKHG7	12	92702843..92772455	-	0,00	-2,89252005
EEA1	12	complement(92770637..92929331)	1,42	2,62	-0,654161035
NUDT4	12	93377883..93408146	1,54	2,63	-0,54596473
UBE2N	12	complement(93405673..93441947)	6,89	9,49	-0,23901161
MRPL42	12	93467514..93516214	2,40	3,55	-0,341933763
SOCS2	12	93569814..93583487	7,65	3,19	1,486144286
CRADD	12	93677375..93894840	2,01	1,76	0,418136052
PLXNC1	12	94148577..94307675	0,04	0,16	-1,6628552
CEP83	12	complement(94306449..94459988)	0,60	0,93	-0,405745198
TMCC3	12	complement(94567122..94650557)	0,18	0,60	-1,482157073
NDUFA12	12	complement(94895297..95003748)	2,43	3,45	-0,282336857
NR2C1	12	complement(95020229..95073628)	1,21	2,07	-0,549803015
FGD6	12	complement(95076749..95217482)	0,93	1,95	-0,836013284
VEZT	12	95217746..95302799	3,51	7,76	-0,919342664
METAP2	12	95473520..95515839	3,75	6,94	-0,66535793
USP44	12	complement(95516560..95551476)	0,02	0,01	1,505976878
NTN4	12	complement(95657807..95791189)	1,89	15,41	-2,805127686
SNRPF	12	95858952..95903828	3,33	6,34	-0,707520982
CCDC38	12	complement(95867048..95942635)	0,01	0,01	1,232079601
AMDHD1	12	95943331..95968720	0,02	0,04	-0,865575989
HAL	12	complement(95972662..95996365)	-	0,01	-5,071123353
LTA4H	12	complement(96000753..96043520)	12,16	19,72	-0,474288762
ELK3	12	96194375..96269824	14,72	18,01	-0,068016032
CDK17	12	complement(96278261..96400480)	2,14	3,20	-0,358925273
CFAP54	12	96489571..96875555	0,01	0,02	-0,618925958
NEDD1	12	96907224..96953780	5,29	7,04	-0,188443813
gene:ENSG000	12	complement(97150081..97154627)	-	-	0,188027316
gene:ENSG000	12	complement(97563257..97563971)	-	-	#N/D
TMPO	12	98515579..98550351	10,06	14,49	-0,303180303
SLC25A3	12	98593591..98606379	31,30	36,43	0,004088398
IKBIP	12	complement(98613405..98645113)	4,13	5,73	-0,251937939
APAF1	12	98645149..98735433	3,31	2,40	0,684126647
ANKS1B	12	complement(98726457..99984936)	0,69	0,14	2,519487947
GARIN6	12	99647753..99650114	0,05	-	5,88622595

gene:ENSG000	12	complement(100026248..10002754	0,30	0,28	0,321078719
BLTP3B	12	complement(100028455..10014287	4,18	4,34	0,17049176
gene:ENSG000	12	complement(100032325..10003407	1,26	1,53	-0,054718588
ACTR6	12	100199122..100241865	0,75	0,94	-0,097532472
DEPDC4	12	complement(100203669..10026707	0,15	0,24	-0,494699922
SCYL2	12	100267140..100341715	3,34	4,76	-0,288497297
SLC17A8	12	100357074..100422055	0,00	-	3,068695355
NR1H4	12	100473708..100564414	-	0,00	-2,89252005
GAS2L3	12	100573683..100628288	2,73	3,57	-0,163624258
ANO4	12	100717526..101128641	0,03	0,03	0,591315826
SLC5A8	12	complement(101155493..10121023	-	-	0,188027316
UTP20	12	101280105..101386618	3,57	5,06	-0,279902232
ARL1	12	complement(101393116..10140777	14,15	10,39	0,669593575
SPIC	12	101475336..101486997	-	-	#N/D
MYBPC1	12	101568353..101686028	-	-	0,188027316
CHPT1	12	101696947..101744140	3,15	4,98	-0,438269802
SYCP3	12	complement(101728648..10173947	-	-	0,188027316
GNPTAB	12	complement(101745499..10183095	1,49	2,83	-0,703632534
DRAM1	12	101877580..102012130	7,31	9,93	-0,218243075
WASHC3	12	complement(102012840..10206214	3,15	3,91	-0,086596116
NUP37	12	complement(102073103..10212012	5,92	9,25	-0,420304052
PARPBP	12	102120183..102197520	0,79	1,45	-0,654845989
PMCH	12	complement(102196459..10219783	0,01	-	3,068695355
IGF1	12	complement(102395874..10248174	-	0,02	-6,312013662
PAH	12	complement(102836889..10295841	-	-	0,188027316
ASCL1	12	102957674..102960513	-	0,01	-3,804623889
C12orf42	12	complement(103237591..10349597	-	-	0,188027316
STAB2	12	103587273..103766719	0,02	0,04	-0,804783029
NT5DC3	12	complement(103770453..10384123	6,45	6,64	0,179959275
HSP90B1	12	103930107..103953931	164,21	97,71	0,972123187
UQCC6	12	complement(103940763..10396570	2,56	2,11	0,499611188
TDG	12	103965822..103988874	4,88	5,44	0,068263702
GLT8D2	12	complement(103988987..10406418	0,72	1,22	-0,552591874
HCFC2	12	104064531..104106524	1,75	1,44	0,500696373
NFYB	12	complement(104117086..10413824	0,74	1,53	-0,828977992
TXNRD1	12	104215779..104350307	79,31	76,01	0,28436149
EID3	12	104303739..104305205	1,10	1,41	-0,143330383
CHST11	12	104455295..104762014	1,02	3,61	-1,601277482
gene:ENSG000	12	104552073..104553447	0,02	0,07	-1,21700111
SLC41A2	12	complement(104801801..10495874	0,45	0,98	-0,891660216
NOPCHAP1	12	104986316..105074197	0,42	0,49	0,014413757
ALDH1L2	12	complement(105019784..10510764	3,12	6,98	-0,9409632
WASHC4	12	105107324..105169130	2,79	5,36	-0,71555679
APPL2	12	complement(105173297..10523620	6,20	9,66	-0,415868012
C12orf75	12	105235290..105396097	20,73	39,42	-0,704206293
NUAK1	12	complement(106063345..10613895	2,60	4,30	-0,500071
CKAP4	12	complement(106237881..10630427	193,23	#####	0,247986816
TCP11L2	12	106301929..106347003	2,26	3,20	-0,278884553

POLR3B	12	106357748..106510198	0,84	3,11	-1,666945086
RFX4	12	106583004..106762803	-	0,01	-4,359055975
RIC8B	12	106774621..106889316	1,52	2,49	-0,488638475
TMEM263	12	106955719..106978778	9,89	8,76	0,39733953
MTERF2	12	complement(106973120..10698716	1,40	1,29	0,341178993
CRY1	12	complement(106991364..10709354	6,41	7,96	-0,088763424
ABTB3	12	107318421..107659642	2,18	3,12	-0,288960024
PWP1	12	107685799..107713162	8,65	10,48	-0,052922714
PRDM4	12	complement(107732871..10776127	7,23	12,73	-0,593544812
ASCL4	12	107774704..107776644	-	-	0,188027316
WSCD2	12	108129288..108250537	-	-	0,188027316
CMKLR1	12	complement(108288044..10833931	0,03	0,03	0,280049548
FICD	12	108515277..108525837	2,86	1,76	0,922379408
SART3	12	complement(108522214..10856140	10,38	12,34	-0,026735672
ISCU	12	108562582..108569368	7,15	10,65	-0,351653862
TMEM119	12	complement(108589851..10859832	0,01	0,00	1,436225396
SELPLG	12	complement(108621895..10863389	0,40	0,46	0,030425674
CORO1C	12	complement(108645109..10873152	56,85	59,85	0,148877815
SSH1	12	complement(108778191..10885759	11,24	12,41	0,080296816
gene:ENSG000	12	108778912..108779166	-	-	#N/D
DAO	12	108858932..108901043	-	-	0,188027316
SVOP	12	complement(108907741..10902106	0,01	-	4,913749004
gene:ENSG000	12	complement(109020833..10902124	-	-	#N/D
USP30	12	109023089..109088023	2,06	2,82	-0,229626537
ALKBH2	12	complement(109088188..10909363	6,59	7,69	0,000851118
UNG	12	109097597..109126725	6,13	8,83	-0,305194754
ACACB	12	109116587..109268226	0,23	0,72	-1,412607258
FOXN4	12	complement(109277978..10930928	-	-	0,188027316
MYO1H	12	109347900..109455523	0,08	0,08	0,345371762
KCTD10	12	complement(109448655..10947735	10,20	8,39	0,504886574
UBE3B	12	109477402..109536705	4,93	6,35	-0,142617641
MMAB	12	complement(109553715..10957358	1,77	2,28	-0,146515917
MVK	12	109573255..109598125	5,26	2,65	1,211609767
FAM222A	12	109713825..109770495	0,05	0,13	-1,148773034
TRPV4	12	complement(109783087..10983340	0,01	0,02	-0,345401666
GLTP	12	complement(109850945..10988054	9,47	12,14	-0,134608643
TCHP	12	109900264..109983841	3,72	5,41	-0,315554756
GIT2	12	complement(109929804..10999638	3,38	5,00	-0,340700125
gene:ENSG000	12	complement(109997419..10999912	0,01	0,06	-2,854330803
ANKRD13A	12	109999186..110039763	12,96	16,82	-0,153276743
C12orf76	12	complement(110027028..11007363	4,24	2,87	0,786426613
gene:ENSG000	12	complement(110056916..11005727	-	0,14	-4,758582517
IFT81	12	110124335..110218793	1,92	2,54	-0,183548249
ATP2A2	12	110280756..110351093	112,63	50,42	1,382545993
ANAPC7	12	complement(110372900..11040373	8,75	17,29	-0,759007875
ARPC3	12	complement(110434823..11045042	66,67	57,26	0,442589684
GPN3	12	complement(110452486..11046926	5,04	6,21	-0,078612177
FAM216A	12	110468415..110490385	3,93	8,90	-0,956486264

VPS29	12	complement(110491083..11050211	7,10	9,63	-0,216686115
gene:ENSG000	12	110501614..110503441	0,58	0,77	-0,193819677
RAD9B	12	110501655..110533556	0,18	0,20	0,058911425
PPTC7	12	complement(110533245..11058331	4,63	5,80	-0,102768782
TCTN1	12	110614027..110663431	1,78	2,84	-0,449867322
HVCN1	12	complement(110627841..11070495	0,09	0,19	-0,795572188
PPP1CC	12	complement(110719680..11074293	16,19	22,77	-0,269068809
gene:ENSG000	12	110744549..110746206	0,02	0,05	-1,21700111
CCDC63	12	110846769..110907535	-	-	0,188027316
MYL2	12	complement(110909996..11105243	-	-	0,188027316
CUX2	12	111034165..111350554	0,02	0,01	2,028855185
PHETA1	12	complement(111360651..11136912	3,37	2,99	0,39211239
SH2B3	12	111405923..111451623	24,23	19,33	0,549539813
ATXN2	12	complement(111443485..11159967	6,79	7,36	0,106999862
BRAP	12	complement(111642146..11168595	4,32	4,63	0,122831714
ACAD10	12	111686053..111757107	4,72	3,95	0,481362181
gene:ENSG000	12	111753890..111791418	1,14	3,42	-1,357536201
ALDH2	12	111766887..111817532	6,04	11,43	-0,697749936
MAPKAPK5	12	111842013..111902222	1,99	3,36	-0,534450067
gene:ENSG000	12	111842237..111842368	-	-	#N/D
TMEM116	12	complement(111931282..11201318	1,86	2,43	-0,162066849
ERP29	12	112013348..112023449	17,58	18,83	0,123842836
NAA25	12	complement(112026689..11210879	5,62	8,33	-0,345197774
TRAFD1	12	112125538..112153604	21,01	17,29	0,503774412
HECTD4	12	complement(112160188..11238243	5,64	5,74	0,197427225
RPL6	12	complement(112405189..11241883	155,22	#####	-0,198489872
PTPN11	12	112418351..112509918	21,04	33,52	-0,449127127
RPH3A	12	112570380..112898881	0,00	0,02	-2,665078517
OAS1	12	112905856..112933219	1,26	0,15	3,316734344
OAS3	12	112938051..112976460	8,27	4,15	1,217989715
OAS2	12	112978395..113011723	1,80	0,33	2,668626328
DTX1	12	113056730..113098028	0,31	0,07	2,313104301
RASAL1	12	complement(113098819..11313623	-	0,01	-3,804623889
CFAP73	12	113149724..113159276	0,07	0,13	-0,659786428
DDX54	12	complement(113157173..11318547	18,91	25,40	-0,202601999
RITA1	12	113185526..113192368	6,73	13,56	-0,786548836
IQCD	12	complement(113195441..11322109	0,42	0,65	-0,397647366
TPCN1	12	113221050..113298585	13,44	22,38	-0,511894823
SLC8B1	12	complement(113298759..11335949	2,67	4,34	-0,47724084
PLBD2	12	113358566..113391629	14,81	13,57	0,34918001
SDS	12	complement(113392445..11342630	0,14	0,10	0,633765945
SDSL	12	113422380..113438276	0,72	1,08	-0,366462693
LHX5	12	complement(113462033..11347228	-	-	#N/D
RBM19	12	complement(113816738..11396632	4,46	5,25	-0,014467135
TBX5	12	complement(114353911..11440844	-	-	#N/D
TBX3	12	complement(114670255..11468417	8,57	12,24	-0,291343694
MED13L	12	complement(115957905..11627769	9,11	12,35	-0,216168041
MAP1LC3B2	12	116548105..116576606	1,13	0,87	0,603143091

SPRING1	12	complement(116710171..11673807	2,44	3,43	-0,268063206
RNFT2	12	116738178..116853631	0,71	3,02	-1,87383897
HRK	12	complement(116856144..11688144	0,00	0,01	-2,190455314
FBXW8	12	116910950..117031148	6,68	6,66	0,227203627
TESC	12	complement(117038923..11709947	0,03	0,06	-0,999120035
FBXO21	12	complement(117141991..11719047	4,97	5,68	0,03051635
NOS1	12	complement(117208142..11745217	0,01	-	6,053060169
KSR2	12	complement(117453012..11796899	0,03	0,02	0,613666035
RFC5	12	118013588..118033130	9,73	11,51	-0,018805015
WSB2	12	complement(118032687..11806243	35,84	30,80	0,441673996
VSIG10	12	complement(118063593..11813602	1,90	3,44	-0,637151044
PEBP1	12	118136124..118145584	95,02	93,13	0,252135382
TAOK3	12	complement(118149801..11837290	3,04	3,86	-0,118117241
SUDS3	12	118376555..118418033	9,56	11,13	0,003560738
SRRM4	12	118981541..119163051	0,01	-	4,913749003
HSPB8	12	119171555..119224855	6,70	19,54	-1,320849134
CCDC60	12	119334712..119541040	-	-	#N/D
TMEM233	12	119593774..119643075	-	-	0,188027316
PRKAB1	12	119667864..119681624	5,81	4,32	0,65015732
CIT	12	complement(119685791..11987732	9,55	12,96	-0,217134447
BICDL1	12	119989236..120094494	0,12	0,18	-0,387113557
RAB35	12	complement(120095099..12011750	12,77	16,15	-0,115627693
GCN1	12	complement(120127202..12019471	49,80	51,06	0,186868997
RPLP0	12	complement(120196699..12020123	642,33	#####	0,104879233
PXN	12	complement(120210439..12026577	38,38	38,62	0,213781879
SIRT4	12	120302316..120313249	1,01	0,75	0,654108145
PLA2G1B	12	complement(120322115..12032777	-	-	0,188027316
MSI1	12	complement(120341330..12036916	0,08	0,74	-2,981713224
COX6A1	12	120438090..120440737	201,96	#####	0,161425637
gene:ENSG000	12	120438198..120460006	0,06	0,05	0,524121557
TRIAP1	12	complement(120443964..12044638	6,89	8,07	-0,003112089
GATC	12	120446444..120463749	6,68	7,67	0,023304284
SRSF9	12	complement(120461672..12049594	49,93	52,95	0,138149514
DYNLL1	12	120469850..120498493	28,75	36,38	-0,116520575
COQ5	12	complement(120503279..12053443	11,24	16,89	-0,36397043
RNF10	12	120533480..120577588	24,22	30,79	-0,123382402
gene:ENSG000	12	120534697..120534957	-	-	0,188027316
POP5	12	complement(120578764..12058140	4,80	5,22	0,100237074
CABP1	12	120640626..120667324	0,04	0,11	-1,145013736
MLEC	12	120687149..120701859	71,50	49,02	0,767458204
UNC119B	12	120710458..120723640	10,18	11,81	0,008803055
ACADS	12	120725774..120740008	3,84	4,53	-0,01628375
SPPL3	12	complement(120762510..12090435	5,86	8,00	-0,225641127
HNF1A	12	120978543..121002512	0,04	0,02	1,264197729
C12orf43	12	complement(121000486..12101650	2,51	3,64	-0,312667665
OASL	12	complement(121017763..12103924	16,57	16,61	0,219213101
gene:ENSG000	12	121096027..121096429	-	0,03	-2,89252005
P2RX7	12	121132819..121188032	1,37	8,19	-2,360490099

P2RX4	12	121210065..121234106	4,40	7,69	-0,581896495
CAMKK2	12	complement(121237675..12129830	8,19	9,39	0,027253766
ANAPC5	12	complement(121308245..12139989	22,67	28,38	-0,101299142
RNF34	12	121400083..121430623	4,36	6,51	-0,355103725
KDM2B	12	complement(121429096..12158102	4,01	6,04	-0,368971301
ORAI1	12	121626509..121643218	14,51	12,39	0,45041027
MORN3	12	complement(121648742..12167263	0,02	0,02	0,152452079
TMEM120B	12	121712752..121783001	13,43	11,59	0,435322783
RHOF	12	complement(121777754..12180340	0,48	1,80	-1,686360471
SETD1B	12	121804009..121832656	5,14	6,70	-0,156919032
HPD	12	complement(121839527..12186359	2,01	1,11	1,078432826
PSMD9	12	121888732..121918297	6,54	6,28	0,282754042
gene:ENSG000	12	121888809..121921470	-	-	0,188027316
CFAP251	12	121918592..122003927	0,22	0,15	0,802470551
BCL7A	12	122019422..122062044	1,16	2,60	-0,935266958
MLXIP	12	122078756..122147344	10,75	8,94	0,488345329
LRRC43	12	122167738..122203471	-	0,03	-5,071123353
IL31	12	complement(122172029..12217422	-	0,01	-2,89252005
B3GNT4	12	122203681..122208952	0,76	0,62	0,515067753
gene:ENSG000	12	complement(122207662..12222753	0,01	0,05	-1,573534033
DIABLO	12	complement(122207668..12222606	16,95	17,19	0,202224453
gene:ENSG000	12	complement(122207779..12226642	-	-	0,188027316
VPS33A	12	complement(122229564..12226650	1,85	2,49	-0,208128128
CLIP1	12	complement(122271432..12242266	5,08	10,29	-0,795706355
ZCCHC8	12	complement(122471600..12250093	1,46	2,47	-0,533488272
RSRC2	12	complement(122503454..12252700	5,12	6,29	-0,073250749
KNTC1	12	122527246..122626396	8,67	8,31	0,28351501
HCAR2	12	complement(122701293..12270335	0,01	0,06	-3,021611439
HCAR3	12	complement(122714756..12271681	-	-	0,188027316
HCAR1	12	complement(122726076..12273084	0,00	0,01	-0,579815015
DENR	12	122752824..122771064	8,97	17,04	-0,703432294
CCDC62	12	122774526..122827528	0,03	0,14	-1,819256723
HIP1R	12	122834453..122862961	6,49	10,93	-0,527642395
VPS37B	12	complement(122865330..12289612	9,18	9,77	0,132799149
gene:ENSG000	12	complement(122870059..12288872	0,61	0,49	0,522184479
ABCB9	12	complement(122920951..12298164	1,24	1,38	0,070809796
OGFOD2	12	122974580..122980043	6,81	5,15	0,625316998
ARL6IP4	12	122980060..122982913	17,80	16,89	0,298311676
PITPNM2	12	complement(122983480..12315109	5,16	4,42	0,443541894
gene:ENSG000	12	complement(123087256..12309282	0,11	0,17	-0,356490448
MPHOSPH9	12	complement(123152320..12324401	4,11	4,97	-0,050827966
gene:ENSG000	12	complement(123152324..12315337	2,76	2,96	0,123080265
MTRFR	12	123233385..123258079	1,73	1,89	0,095983765
CDK2AP1	12	complement(123250112..12327233	10,18	10,25	0,213404123
SBNO1	12	complement(123289109..12336484	9,38	9,32	0,232258994
KMT5A	12	123384132..123409353	22,88	31,06	-0,218295434
RILPL2	12	complement(123415039..12343668	5,72	5,08	0,395999874
SNRNP35	12	123458088..123473154	2,98	2,77	0,330096094

RILPL1	12	complement(123470054..12353371	2,99	4,59	-0,392427793
TMED2	12	123584533..123598582	48,38	48,87	0,208548763
DDX55	12	123602077..123620943	4,27	5,57	-0,16204808
EIF2B1	12	complement(123620406..12363376	12,08	10,53	0,421220159
GTF2H3	12	123633739..123662604	7,42	13,31	-0,619421623
gene:ENSG000	12	123649068..123650485	0,46	0,58	-0,086781211
gene:ENSG000	12	123655528..123656128	0,32	0,22	0,800439495
TCTN2	12	123671110..123708399	8,41	4,70	1,061862453
ATP6V0A2	12	123712353..123761755	1,33	1,63	-0,068434583
gene:ENSG000	12	complement(123754246..12375479	0,14	0,17	-0,036228841
DNAH10	12	123762188..123935720	0,23	0,13	1,037053112
CCDC92	12	complement(123918660..12397283	2,97	5,52	-0,67080199
ZNF664	12	123971845..124015439	9,01	14,79	-0,491664136
RFLNA	12	123973241..124316024	0,09	0,06	0,802648025
NCOR2	12	complement(124324415..12456761	27,93	36,98	-0,181683703
gene:ENSG000	12	complement(124529722..12453022	0,28	0,15	1,060045679
gene:ENSG000	12	124537844..124539677	0,61	0,49	0,529691244
gene:ENSG000	12	124542503..124542873	0,26	0,17	0,7939095
gene:ENSG000	12	124555905..124556432	1,02	1,03	0,210584379
gene:ENSG000	12	124561869..124562283	1,20	0,84	0,725565066
gene:ENSG000	12	complement(124593181..12459369	0,02	0,20	-2,854330803
gene:ENSG000	12	complement(124622290..12462306	-	-	0,188027316
gene:ENSG000	12	124623391..124623987	-	0,07	-4,359055975
gene:ENSG000	12	complement(124637999..12463844	-	0,06	-3,804623889
gene:ENSG000	12	124725524..124725934	-	-	#N/D
SCARB1	12	complement(124776856..12488266	15,62	26,81	-0,555870026
UBC	12	complement(124911604..12491736	246,16	#####	0,764935444
DHX37	12	complement(124946825..12498913	7,77	11,19	-0,303477091
BRI3BP	12	124993645..125031231	5,26	7,93	-0,367629433
AACS	12	125065434..125143333	4,73	4,10	0,430079004
gene:ENSG000	12	125138245..125141711	0,61	0,43	0,735148113
gene:ENSG000	12	125160525..125164918	0,01	0,01	0,524121557
TMEM132B	12	125186386..125662377	0,08	0,08	0,264786301
gene:ENSG000	12	125300936..125301917	-	-	0,188027316
gene:ENSG000	12	125808668..125809808	-	-	#N/D
gene:ENSG000	12	125858862..125861119	-	-	#N/D
gene:ENSG000	12	complement(126106459..12610704	-	-	#N/D
gene:ENSG000	12	complement(126130166..12613086	-	-	0,188027316
gene:ENSG000	12	126141952..126142377	-	-	#N/D
gene:ENSG000	12	complement(126675895..12667624	-	-	0,188027316
gene:ENSG000	12	126677262..126677806	-	0,02	-2,89252005
gene:ENSG000	12	127372243..127372669	-	-	#N/D
gene:ENSG000	12	127462507..127463197	-	-	#N/D
gene:ENSG000	12	complement(127825938..12782628	-	-	#N/D
TMEM132C	12	128267170..128707911	-	-	0,188027316
gene:ENSG000	12	128288159..128289056	-	-	0,188027316
SLC15A4	12	complement(128793194..12882395	9,93	8,26	0,488220039
gene:ENSG000	12	complement(128813186..12881475	1,45	1,14	0,571364795

GLT1D1	12	128853427..128984968	1,47	0,01	6,803818576
TMEM132D	12	complement(129071725..12990402	0,00	0,03	-3,112412319
gene:ENSG000	12	129262345..129262824	-	-	#N/D
gene:ENSG000	12	complement(129839928..12984052	-	-	#N/D
gene:ENSG000	12	complement(130121925..13012297	-	-	0,188027316
gene:ENSG000	12	130144954..130145691	-	-	#N/D
FZD10	12	130162459..130165740	0,00	-	3,068695355
PIWIL1	12	130337887..130372637	-	-	0,188027316
gene:ENSG000	12	complement(130350996..13035170	-	-	#N/D
gene:ENSG000	12	130383364..130383890	-	-	#N/D
RIMBP2	12	complement(130396133..13076822	0,00	-	3,068695355
gene:ENSG000	12	130425004..130430661	-	0,00	-3,804623889
gene:ENSG000	12	complement(130465008..13046631	-	-	#N/D
gene:ENSG000	12	complement(130775694..13077654	0,03	0,05	-0,579815015
STX2	12	complement(130789600..13083925	5,28	4,26	0,533354417
RAN	12	130872037..130877678	95,20	94,45	0,2344601
ADGRD1	12	130953907..131141469	0,01	0,01	0,686693064
gene:ENSG000	12	complement(130956384..13095803	-	-	#N/D
gene:ENSG000	12	130962175..130963028	-	-	#N/D
gene:ENSG000	12	130976786..130978812	-	-	0,188027316
gene:ENSG000	12	complement(130980667..13098136	-	-	#N/D
gene:ENSG000	12	complement(130988312..13099002	-	-	0,188027316
gene:ENSG000	12	complement(131022769..13102474	-	-	0,188027316
gene:ENSG000	12	complement(131025561..13102806	-	-	0,188027316
gene:ENSG000	12	131235511..131237523	0,01	-	3,068695355
gene:ENSG000	12	131295522..131298218	-	-	#N/D
gene:ENSG000	12	131436447..131436960	-	-	#N/D
gene:ENSG000	12	complement(131478844..13148303	-	-	#N/D
gene:ENSG000	12	complement(131620178..13162076	-	-	#N/D
SFSWAP	12	131711081..131799738	17,17	14,08	0,509294235
MMP17	12	131828393..131851783	2,81	1,75	0,907776958
ULK1	12	131894622..131923150	18,66	16,66	0,386945122
gene:ENSG000	12	131896500..131898239	6,57	3,36	1,193127206
PUS1	12	131929200..131945896	6,19	4,76	0,602225073
EP400	12	131949942..132080460	13,63	13,54	0,233131768
gene:ENSG000	12	complement(132100013..13210082	2,36	1,19	1,215938002
DDX51	12	complement(132136594..13214431	4,72	3,33	0,723410466
NOC4L	12	132144457..132152473	16,00	11,28	0,72767772
GALNT9	12	complement(132196372..13232958	21,37	13,54	0,881246133
gene:ENSG000	12	132200436..132201287	0,62	0,43	0,764199436
gene:ENSG000	12	complement(132279256..13228090	1,44	1,16	0,524179151
gene:ENSG000	12	132332005..132332432	0,28	0,12	1,391449417
gene:ENSG000	12	132351855..132352322	0,02	-	3,068695355
gene:ENSG000	12	132360734..132361198	0,05	-	3,967268276
gene:ENSG000	12	132362810..132363348	0,06	0,02	1,436225396
FBRSL1	12	132489551..132585188	5,85	6,03	0,177526349
gene:ENSG000	12	132550729..132554947	3,51	1,26	1,697881929
gene:ENSG000	12	132565071..132566425	2,03	1,13	1,070077925

LRCOL1	12	complement(132603150..13261058	0,01	0,02	-0,923955849
P2RX2	12	132618776..132622388	0,16	0,10	0,90516579
gene:ENSG000	12	complement(132621999..13262297	0,45	0,39	0,457552023
POLE	12	complement(132623753..13268737	13,76	13,41	0,261254778
PXMP2	12	132687587..132704985	7,52	5,59	0,649677194
PGAM5	12	132710819..132722734	28,32	22,03	0,585739508
ANKLE2	12	complement(132725503..13276183	14,67	11,50	0,575051009
GOLGA3	12	complement(132768902..13282914	46,58	15,95	1,769575616
CHFR	12	complement(132822187..13295630	0,93	0,07	4,050383267
gene:ENSG000	12	complement(132865855..13286795	0,41	0,01	5,190275007
ZNF605	12	complement(132918306..13295630	1,19	1,21	0,201003683
ZNF26	12	132986365..133032952	0,33	0,31	0,319764832
ZNF84	12	133037292..133063304	0,71	0,98	-0,240348549
gene:ENSG000	12	complement(133073516..13307398	-	-	#N/D
gene:ENSG000	12	complement(133077762..13307839	-	-	0,188027316
ZNF140	12	133080474..133107526	-	-	0,188027316
ZNF891	12	complement(133104779..13313027	0,24	0,12	1,19231565
ZNF10	12	133130575..133159465	0,18	0,11	0,878696973
gene:ENSG000	12	133130984..133202369	0,01	-	3,068695355
ZNF268	12	133181409..133214832	0,33	0,41	-0,078178218
ANHX	12	complement(133218312..13323587	-	0,01	-2,89252005
TUBA3C	13	complement(19173772..19181824)	-	-	#N/D
TPTE2	13	complement(19422876..19561625)	0,00	-	3,068695355
MPHOSPH8	13	19633659..19673441	3,11	5,37	-0,563555668
PSPC1	13	complement(19674752..19783019)	4,57	7,62	-0,513265549
ZMYM5	13	complement(19823482..19863649)	1,36	1,26	0,334306111
ZMYM2	13	19958677..20091829	2,57	3,81	-0,342598426
gene:ENSG000	13	20092632..20094144	0,02	0,07	-1,406253396
GJA3	13	complement(20138255..20161052)	0,48	0,79	-0,51242652
GJB2	13	complement(20187463..20192938)	0,32	0,84	-1,160487474
gene:ENSG000	13	20194412..20196005	-	-	#N/D
GJB6	13	complement(20221962..20232365)	-	-	#N/D
CRYL1	13	complement(20403666..20525873)	3,74	4,46	-0,031557004
IFT88	13	20567138..20691444	0,47	1,20	-1,1222944
IL17D	13	20702127..20723098	0,42	1,30	-1,412007388
gene:ENSG000	13	20712431..20714146	0,02	0,03	-0,429837071
EEF1AKMT1	13	complement(20728731..20773961)	1,73	2,61	-0,370441974
XPO4	13	complement(20777329..20903048)	3,20	4,18	-0,163269914
LATS2	13	complement(20973036..21061586)	6,95	6,27	0,370248102
SAP18	13	21140119..21149097	13,04	15,52	-0,028211054
SKA3	13	complement(21153595..21176552)	3,99	7,56	-0,698357649
MRPL57	13	21176658..21179084	6,31	8,49	-0,205483074
ZDHHC20	13	complement(21348784..21459370)	6,32	7,01	0,072316733
MICU2	13	complement(21492691..21604181)	2,15	4,38	-0,802141095
FGF9	13	21671073..21704498	0,00	0,00	0,886720911
SGCG	13	23180979..23325162	0,16	0,10	0,952502668
SACS	13	complement(23288689..23433763)	3,52	5,76	-0,48513916
TNFRSF19	13	23570370..23676104	3,93	2,95	0,635340947

MIPEP	13	complement(23730189..23889400)	7,48	8,42	0,05293753
C1QTNF9B	13	complement(23891099..23897502)	0,22	0,14	0,861505781
gene:ENSG000	13	23979700..24321031	1,25	1,03	0,504194035
SPATA13	13	23979805..24307074	7,17	6,61	0,341598677
C1QTNF9	13	24307166..24322535	-	-	0,188027316
PARP4	13	complement(24420931..24512778)	21,23	28,70	-0,211850546
ATP12A	13	24680408..24712472	-	-	0,188027316
RNF17	13	24764169..24879921	-	-	0,188027316
CENPJ	13	complement(24882279..24922889)	8,72	5,77	0,820206382
PABPC3	13	25096136..25099254	0,00	-	3,068695355
gene:ENSG000	13	25141536..25141809	-	-	#N/D
AMER2	13	complement(25161679..25172288)	0,00	-	3,068695355
gene:ENSG000	13	complement(25190063..25190317)	-	-	#N/D
MTMR6	13	complement(25246222..25287510)	3,15	4,84	-0,396390041
NUP58	13	25301524..25365390	7,64	7,83	0,188423062
ATP8A2	13	25371974..26025851	0,02	0,05	-1,211830422
SHISA2	13	complement(26044597..26052016)	1,61	2,26	-0,270701304
RNF6	13	complement(26132115..26222314)	6,78	10,40	-0,39409272
CDK8	13	26254104..26420982	3,91	5,33	-0,22427883
WASF3	13	26557683..26688948	8,16	7,91	0,268482925
GPR12	13	complement(26755200..26760786)	-	-	#N/D
USP12	13	complement(27066156..27171811)	5,03	6,86	-0,226425246
RPL21	13	27251362..27256691	275,62	#####	0,286551402
RASL11A	13	27270830..27275192	1,71	3,87	-0,959377598
GTF3A	13	27424619..27435823	23,10	35,31	-0,388842975
MTIF3	13	complement(27435643..27450591)	5,75	5,92	0,182688067
LNX2	13	complement(27545913..27620529)	3,99	3,04	0,6133517
POLR1D	13	27620742..27744237	3,92	4,20	0,126476715
GSX1	13	27792483..27794768	-	-	0,188027316
PDX1	13	27920000..27926313	0,22	0,04	2,694220325
CDX2	13	complement(27960918..27969315)	0,40	0,07	2,791470271
URAD	13	complement(27977717..27988693)	-	-	#N/D
FLT3	13	complement(28003274..28100592)	-	0,00	-2,89252005
PAN3	13	28138506..28295335	3,97	4,64	-0,003911461
FLT1	13	complement(28300346..28495145)	0,26	0,10	1,604166182
gene:ENSG000	13	28495468..28497267	-	0,01	-2,89252005
POMP	13	28659065..28678959	6,18	11,68	-0,695006776
SLC46A3	13	complement(28700064..28718970)	1,08	1,85	-0,55997925
MTUS2	13	28820339..29505947	0,03	0,07	-0,851809139
SLC7A1	13	complement(29509414..29595688)	19,63	43,94	-0,939343611
UBL3	13	complement(29764371..29850617)	2,49	3,69	-0,342359678
gene:ENSG000	13	complement(30196443..30196881)	-	-	0,188027316
KATNAL1	13	complement(30202630..30307551)	5,01	5,99	-0,032655977
gene:ENSG000	13	30344672..30347167	0,05	0,06	-0,037899139
UBE2L5	13	30422488..30429758	0,02	0,02	0,181652234
HMGB1	13	complement(30456704..30617597)	12,70	19,90	-0,425277938
USPL1	13	30617693..30660770	4,45	4,90	0,082061103
ALOX5AP	13	30713478..30764426	0,17	2,88	-3,806403723

MEDAG	13	30906271..30925572	0,04	0,06	-0,35396495
TEX26	13	30932656..30975500	-	-	#N/D
HSPH1	13	complement(31134973..31162388)	8,60	15,44	-0,620503904
B3GLCT	13	31199975..31332276	2,27	3,82	-0,524354569
RXFP2	13	31739526..31803389	0,03	0,02	1,106450337
FRY	13	31846713..32299125	0,15	0,11	0,691198744
gene:ENSG000	13	complement(31960325..31961946)	-	-	0,188027316
ZAR1L	13	complement(32303699..32315363)	-	0,02	-4,359055975
BRCA2	13	32315086..32400268	0,78	2,19	-1,2587066
N4BP2L1	13	complement(32400723..32428311)	0,28	0,14	1,191120704
N4BP2L2	13	complement(32432417..32538885)	1,16	1,71	-0,335295593
PDS5B	13	32586452..32778019	4,48	5,93	-0,179367352
KL	13	33016423..33066143	0,00	0,00	0,886720911
STARD13	13	complement(33103137..33350630)	4,87	5,53	0,040628058
RFC3	13	33818069..33966558	6,07	9,57	-0,433279166
gene:ENSG000	13	34079411..34080134	0,07	0,07	0,278120973
NBEA	13	34942270..35673022	0,35	0,88	-1,106207677
MAB21L1	13	complement(35473419..35478764)	0,26	0,35	-0,19531246
DCLK1	13	complement(35768652..36131382)	0,07	0,15	-0,789251037
SOHLH2	13	complement(36168217..36214588)	0,01	0,07	-3,112412319
CCDC169-SOH	13	complement(36168794..36297842)	0,04	0,30	-2,790491724
CCDC169	13	complement(36222008..36297840)	0,06	0,54	-2,934541543
SPART	13	complement(36301638..36370180)	6,96	9,57	-0,235289162
CCNA1	13	36431520..36442870	0,22	2,15	-3,059813415
SERTM1	13	36674020..36697839	0,01	0,03	-0,820024868
RFXAP	13	36819222..36829104	1,13	1,57	-0,245897752
SMAD9	13	complement(36844831..36920887)	4,18	4,67	0,063039087
ALG5	13	complement(36949738..37000763)	1,04	1,57	-0,378674672
EXOSC8	13	36998816..37025881	1,62	2,50	-0,402531102
SUPT20H	13	complement(37009312..37059713)	6,45	9,54	-0,340947637
CSNK1A1L	13	complement(37103259..37105664)	-	-	#N/D
gene:ENSG000	13	complement(37142967..37146718)	-	0,00	-2,89252005
POSTN	13	complement(37562583..37598844)	0,04	0,01	2,688971416
TRPC4	13	complement(37632063..37869802)	0,02	-	6,95890237
UFM1	13	38349849..38363619	10,92	6,81	0,90414425
gene:ENSG000	13	38372856..38373935	-	-	0,188027316
FREM2	13	38687077..38887131	0,02	-	7,452543645
STOML3	13	complement(38965925..38990859)	-	-	0,188027316
PROSER1	13	complement(39009865..39038089)	5,56	9,14	-0,494131628
NHLRC3	13	39038306..39050109	1,61	2,75	-0,543851717
LHFPL6	13	complement(39209116..39603528)	4,51	3,35	0,65436594
COG6	13	39655627..39791665	3,96	3,80	0,284333023
FOXO1	13	complement(40555667..40666641)	1,46	1,81	-0,087900876
MRPS31	13	complement(40729128..40771190)	3,34	5,79	-0,572401575
SLC25A15	13	40789412..40812460	6,78	8,75	-0,144675923
ELF1	13	complement(40931924..41061440)	9,76	7,58	0,587978976
WBP4	13	41061509..41084006	1,11	1,82	-0,486041847
KBTBD6	13	complement(41127569..41132802)	6,15	4,49	0,677331017

KBTBD7	13	complement(41189834..41194569)	0,92	0,73	0,54750473
MTRF1	13	complement(41216369..41263577)	1,18	1,57	-0,188840763
NAA16	13	41311267..41377030	0,92	1,61	-0,578831345
RGCC	13	41457550..41470871	0,86	2,08	-1,058017984
VWA8	13	complement(41566835..41961120)	3,17	6,21	-0,745677273
DGKH	13	42040036..42256578	0,84	1,48	-0,596283445
AKAP11	13	42272152..42323261	6,17	8,36	-0,216497748
TNFSF11	13	42562736..42608013	0,11	0,00	4,593047663
FAM216B	13	42781550..42791549	-	0,06	-6,630750384
EPSTI1	13	complement(42886388..42992271)	0,24	0,54	-0,908488363
DNAJC15	13	43023203..43114213	1,02	2,15	-0,857409772
ENOX1	13	complement(43213130..43786972)	0,08	0,35	-1,872988591
CCDC122	13	complement(43823909..43879740)	0,08	0,24	-1,326317493
LACC1	13	43879284..43893932	0,11	0,38	-1,62216764
gene:ENSG000	13	43883800..44028526	0,01	0,03	-1,877914477
gene:ENSG000	13	complement(43894725..43895182)	-	-	0,188027316
SMIM2	13	complement(44143150..44161257)	0,02	0,04	-0,979341556
SERP2	13	44373665..44397714	-	-	#N/D
TSC22D1	13	complement(44432143..44577147)	12,26	25,06	-0,808300992
gene:ENSG000	13	44432144..44432678	-	-	#N/D
NUFIP1	13	complement(44939249..44989471)	1,71	2,85	-0,512672364
GPALPP1	13	44989529..45037669	1,82	2,06	0,040394183
GTF2F2	13	45120510..45284893	5,43	7,58	-0,258169436
KCTD4	13	complement(45192853..45201045)	0,11	0,12	0,030621608
TPT1	13	complement(45333471..45341284)	89,12	83,50	0,317062136
SLC25A30	13	complement(45393316..45418455)	6,26	4,45	0,715845635
COG3	13	45464898..45536701	9,64	5,42	1,054030727
ERICH6B	13	complement(45534522..45615739)	-	-	#N/D
CBY2	13	45702320..45714559	-	-	0,188027316
SIAH3	13	complement(45777242..45851753)	-	-	#N/D
ZC3H13	13	complement(45954465..46052759)	3,13	5,82	-0,668937872
CPB2	13	complement(46053186..46105033)	0,03	0,02	0,761197382
LCP1	13	complement(46125920..46211871)	0,37	6,09	-3,833410409
LRRC63	13	46211928..46277115	0,03	0,09	-1,510710537
RUBCNL	13	complement(46334681..46438190)	0,01	0,11	-2,959107337
LRCH1	13	46553168..46753040	3,18	3,40	0,124037224
ESD	13	complement(46771256..46797420)	16,16	16,79	0,167970486
HTR2A	13	complement(46831546..46897076)	-	-	0,188027316
SUCLA2	13	complement(47745736..48037968)	3,09	4,37	-0,27590972
NUDT15	13	48037726..48047221	6,09	9,72	-0,452215112
MED4	13	complement(48053323..48095131)	3,48	4,46	-0,135985645
ITM2B	13	48232612..48270357	5,81	6,94	-0,034208742
RB1	13	48303744..48599436	4,54	7,77	-0,552809212
LPAR6	13	complement(48389567..48444704)	0,05	0,05	0,404132137
RCBTB2	13	complement(48488963..48533256)	0,32	0,76	-1,009508441
CYSLTR2	13	48653711..48711226	-	-	0,188027316
FNDC3A	13	48975912..49209779	7,46	6,98	0,318629856
MLNR	13	49220338..49222377	0,04	0,01	2,144183439

CDADC1	13	49247925..49293485	0,45	0,71	-0,425638978
CAB39L	13	complement(49308650..49444064)	1,76	1,26	0,71386781
SETDB2	13	49444274..49495003	0,90	1,20	-0,196115815
PHF11	13	49495610..49528981	0,94	1,63	-0,575867808
RCBTB1	13	complement(49531946..49585558)	2,92	3,78	-0,146996328
ARL11	13	49628507..49633872	0,01	-	4,516772761
EBPL	13	complement(49660674..49691486)	16,48	17,29	0,153358766
KPNA3	13	complement(49699320..49792682)	12,74	15,87	-0,093683084
SPRYD7	13	complement(49912702..49936490)	2,05	3,29	-0,459334956
TRIM13	13	49995888..50020481	1,18	1,68	-0,289712297
KCNRG	13	50015254..50020922	0,23	0,67	-1,341386939
DLEU7	13	complement(50519364..50843939)	0,01	0,03	-1,022609537
RNASEH2B	13	50909747..51024120	0,69	1,38	-0,774273859
C13orf42	13	complement(51082119..51200252)	-	-	0,188027316
FAM124A	13	51222334..51284239	0,05	0,06	0,059297541
SERPINE3	13	51335773..51364735	0,02	0,06	-1,275310173
INTS6	13	complement(51354077..51454264)	2,02	2,00	0,23964339
WDFY2	13	51584455..51767709	4,72	4,23	0,38133712
gene:ENSG000	13	complement(51763951..51845159)	-	-	0,188027316
DHRS12	13	complement(51767993..51804163)	0,86	1,52	-0,597359998
TMEM272	13	complement(51813347..51845177)	0,01	0,01	1,232079601
CCDC70	13	51861969..51866232	-	-	#N/D
gene:ENSG000	13	51921251..51921587	0,13	-	4,913749003
ATP7B	13	complement(51930436..52012125)	1,84	0,58	1,888530719
ALG11	13	52012391..52079232	3,25	2,60	0,545357827
UTP14C	13	52024691..52033600	2,47	1,14	1,340837449
NEK5	13	complement(52033611..52129092)	0,16	0,09	1,058830312
NEK3	13	complement(52132639..52159597)	0,53	0,98	-0,669758913
THSD1	13	complement(52377167..52416373)	0,42	1,11	-1,197446201
VPS36	13	complement(52412602..52450634)	2,37	3,63	-0,39164702
CKAP2	13	52455429..52476628	10,14	14,09	-0,251208014
gene:ENSG000	13	complement(52497097..52500195)	0,01	0,00	0,886720911
HNRNPA1L2	13	52642431..52643773	1,03	1,24	-0,051838985
SUGT1	13	52652709..52700909	1,87	2,01	0,124903735
CNMD	13	complement(52703264..52739820)	0,05	-	5,697545031
PCDH8	13	complement(52842889..52848641)	-	-	#N/D
OLFM4	13	53028813..53052057	-	-	0,188027316
PRR20A	13	57140918..57143939	-	-	#N/D
PRR20C	13	57140918..57157082	-	-	#N/D
PRR20B	13	57147488..57150509	-	-	#N/D
PRR20D	13	57160632..57163653	-	-	#N/D
PRR20E	13	57167197..57170218	-	-	#N/D
PCDH17	13	57631744..57729311	0,11	-	9,253180878
DIAPH3	13	complement(59665583..60163928)	7,96	10,39	-0,162565358
TDRD3	13	60396457..60573878	1,42	2,24	-0,441582206
gene:ENSG000	13	60730176..60733600	-	-	#N/D
PCDH20	13	complement(61409685..61415849)	0,34	0,00	7,109944273
gene:ENSG000	13	complement(61409858..61427849)	-	-	#N/D

gene:ENSG000	13	63746741..63751080	-	0,01	-2,89252005
gene:ENSG000	13	complement(63832361..63833053)	0,02	0,02	-0,01185201
PCDH9	13	complement(66302834..67230445)	0,12	0,09	0,624756698
KLHL1	13	complement(69700594..70108493)	-	0,01	-3,804623889
gene:ENSG000	13	complement(70137831..70139431)	-	-	0,188027316
DACH1	13	complement(71437966..71867204)	0,02	-	6,053060169
MZT1	13	complement(72708367..72727629)	6,00	5,58	0,327993221
BORA	13	72727749..72756198	6,11	4,06	0,810405268
DIS3	13	complement(72752169..72782096)	3,78	3,37	0,388024577
PIBF1	13	72782133..73016461	1,55	2,15	-0,242286863
KLF5	13	73054976..73077541	0,81	2,34	-1,306978909
gene:ENSG000	13	73399031..73399205	-	-	#N/D
KLF12	13	complement(73686089..74134404)	1,00	0,32	1,85688843
gene:ENSG000	13	73800074..73802203	-	-	0,188027316
TBC1D4	13	complement(75283503..75482169)	2,92	3,58	-0,070135366
COMMD6	13	complement(75525214..75549439)	8,48	7,21	0,456640044
UCHL3	13	75549483..75606020	6,79	12,51	-0,658653553
LMO7	13	75620434..75859870	7,90	10,61	-0,203453623
KCTD12	13	complement(76880175..76886405)	6,85	7,48	0,096587666
ACOD1	13	76948511..76958638	0,10	0,24	-1,034421145
CLN5	13	76990660..77019143	0,69	0,72	0,148052303
gene:ENSG000	13	76992078..77129589	0,05	0,08	-0,53063305
FBXL3	13	complement(76992598..77027195)	2,53	3,22	-0,121750303
MYCBP2	13	complement(77042474..77327094)	4,19	5,15	-0,074898009
gene:ENSG000	13	complement(77326905..77326988)	-	-	#N/D
SCEL	13	77535674..77645263	-	-	0,188027316
SLAIN1	13	77697687..77764242	-	-	0,188027316
EDNRB	13	complement(77895481..77975529)	0,06	-	7,865233509
POU4F1	13	complement(78598362..78603552)	-	-	0,188027316
OBI1	13	complement(78614289..78659155)	2,77	4,34	-0,423484856
RBM26	13	complement(79311824..79406477)	4,66	6,85	-0,332346241
NDFIP2	13	79481155..79556077	1,16	2,56	-0,917641819
SPRY2	13	complement(80335976..80341126)	6,53	5,23	0,545158755
SLITRK1	13	complement(83877205..83882474)	-	-	0,188027316
SLITRK6	13	complement(85792790..85806683)	0,09	0,01	4,041723912
SLITRK5	13	87671371..87696272	1,00	1,51	-0,372982828
GPC5	13	91398621..92873682	0,08	0,26	-1,422055896
gene:ENSG000	13	91485793..91486870	0,01	0,06	-2,190455314
gene:ENSG000	13	complement(92239835..92241951)	-	0,01	-3,804623889
gene:ENSG000	13	92446470..92449056	-	-	#N/D
gene:ENSG000	13	complement(92658994..92659227)	-	-	#N/D
GPC6	13	93226807..94408020	0,33	0,96	-1,304347904
DCT	13	complement(94436811..94479682)	0,00	0,01	-1,478387936
TGDS	13	complement(94574054..94596242)	2,35	2,75	-0,007213608
GPR180	13	94601857..94634661	3,04	2,66	0,415868779
SOX21	13	complement(94709622..94712545)	-	-	0,188027316
ABCC4	13	complement(95019835..95301475)	7,95	6,19	0,584423165
CLDN10	13	95433604..95579759	0,01	0,01	-0,579815015

DZIP1	13	complement(95578202..95644706)	3,72	6,37	-0,551261785
DNAJC3	13	95677139..95794988	15,90	8,28	1,163966624
UGGT2	13	complement(95801580..96053482)	1,21	1,64	-0,21073593
HS6ST3	13	96090107..96839562	0,13	0,02	3,113853547
OXGR1	13	complement(96985713..96994730)	-	-	#N/D
MBNL2	13	97221434..97394120	14,85	13,75	0,333561438
RAP2A	13	97434169..97469128	2,42	2,71	0,057280803
IPO5	13	97953658..98024296	23,40	35,37	-0,373011205
FARP1	13	98142562..98455176	4,30	4,72	0,089491081
RNF113B	13	complement(98175785..98177269)	0,14	0,14	0,221852695
STK24	13	complement(98445185..98577940)	6,19	8,82	-0,287822152
SLC15A1	13	complement(98683801..98752672)	-	-	0,188027316
DOCK9	13	complement(98793429..99086625)	0,53	1,12	-0,854583099
UBAC2	13	99200774..99386504	13,07	14,95	0,029318653
GPR18	13	complement(99254732..99261744)	0,14	0,23	-0,438231658
GPR183	13	complement(99294539..99307399)	0,05	0,10	-0,728065629
TM9SF2	13	99446311..99564048	14,76	15,84	0,120717
CLYBL	13	99606669..99909459	0,41	0,50	-0,070783741
ZIC5	13	complement(99962964..99971767)	1,63	2,67	-0,486235955
ZIC2	13	99981784..99986765	7,17	7,19	0,218490121
PCCA	13	100089015..100530437	2,91	3,64	-0,096037123
GGACT	13	complement(100530164..10058952	0,37	0,50	-0,219590799
TMTC4	13	complement(100603625..10067509	5,57	8,01	-0,301114666
NALCN	13	complement(101053776..10141650	0,10	0,59	-2,269762571
ITGBL1	13	101452593..101720856	1,74	1,74	0,221241712
FGF14	13	complement(101710804..10240245	0,02	0,05	-0,953837493
gene:ENSG000	13	complement(102299790..10230027	-	-	#N/D
TPP2	13	102596958..102679958	4,59	4,84	0,147880198
METTL21C	13	complement(102685747..10269504	0,01	-	3,068695355
CCDC168	13	complement(102729367..10275907	0,01	0,01	0,097868349
TEX30	13	complement(102765888..10277381	2,27	3,03	-0,194329217
POGLUT2	13	complement(102784281..10279904	3,74	3,32	0,394262562
BIVM-ERCC5	13	102799110..102875994	0,14	0,05	1,859388559
BIVM	13	102799119..102841533	4,33	5,77	-0,191466361
ERCC5	13	102845831..102875995	12,00	7,13	0,974202367
METTL21EP	13	102880040..102896033	-	0,01	-2,89252005
SLC10A2	13	complement(103043998..10306641	-	-	#N/D
DAOA	13	105465867..105491034	-	-	#N/D
EFNB2	13	complement(106489745..10653566	0,82	4,99	-2,378133371
ARGLU1	13	complement(106541673..10656813	5,18	6,33	-0,066536381
gene:ENSG000	13	complement(106941950..10694256	-	-	#N/D
NALF1	13	complement(107163510..10786749	1,76	2,43	-0,2399074
LIG4	13	complement(108207439..10821836	1,01	1,13	0,06871173
ABHD13	13	108218392..108234243	0,57	1,02	-0,625496585
TNFSF13B	13	108251240..108308484	0,08	0,04	1,170931565
MYO16	13	108596152..109208005	0,00	0,01	-0,429837071
IRS2	13	complement(109752695..10978658	0,93	1,88	-0,793180762
LINC03032	13	110123208..110125234	0,20	0,23	-0,000603959

COL4A1	13	complement(110148963..11030720	9,62	21,23	-0,918508675
gene:ENSG000	13	complement(110265251..11026611	0,05	0,06	-0,032860829
COL4A2	13	110305812..110513209	14,34	23,82	-0,509043881
RAB20	13	complement(110523066..11056172	1,07	0,60	1,057402703
NAXD	13	110615545..110639996	9,97	9,90	0,233948319
CARS2	13	complement(110641412..11071360	9,74	18,16	-0,676261072
ING1	13	110712736..110723339	2,16	1,65	0,612272557
ANKRD10	13	complement(110878540..11091506	6,86	5,94	0,431331559
ARHGEF7	13	111114559..111305737	3,10	3,87	-0,09578401
TEX29	13	111316184..111344249	0,07	0,09	-0,253352605
SOX1	13	112067149..112071706	-	-	0,188027316
SPACA7	13	112376355..112434689	0,02	-	3,967268276
TUBGCP3	13	complement(112485011..11258820	4,84	7,69	-0,446523184
ATP11A	13	112690038..112887168	2,30	3,28	-0,284316497
MCF2L	13	112894378..113099742	0,13	0,00	7,205202197
F7	13	113105788..113120685	-	0,00	-2,89252005
F10	13	113122799..113149529	0,09	0,05	0,949238826
PROZ	13	113158648..113172386	0,22	0,51	-0,967721511
PCID2	13	complement(113177536..11320871	11,59	14,50	-0,10072384
CUL4A	13	113208193..113267108	8,36	11,89	-0,284105794
LAMP1	13	113297239..113323672	62,75	59,74	0,294089768
GRTP1	13	complement(113324163..11336414	0,45	0,20	1,422975093
ADPRHL1	13	complement(113399611..11345348	0,39	0,27	0,745351496
DCUN1D2	13	complement(113455819..11349095	4,21	4,10	0,259179967
TMCO3	13	113491021..113554590	7,85	9,17	-0,000378378
TFDP1	13	113584721..113641473	35,85	44,26	-0,080845054
ATP4B	13	complement(113648804..11365819	-	0,04	-5,071123353
GRK1	13	113667219..113737736	0,01	0,01	0,921097799
LINC00552	13	complement(113748511..11375108	0,01	0,07	-2,647023303
TMEM255B	13	113759226..113816995	0,39	1,09	-1,240279829
GAS6	13	complement(113820549..11386407	30,68	45,71	-0,35224972
C13orf46	13	complement(113953705..11397407	1,19	2,22	-0,676584361
RASA3	13	complement(113977783..11413262	20,98	17,62	0,475096105
CFAP97D2	13	114154691..114223085	-	-	0,188027316
CDC16	13	114234887..114272723	9,53	16,36	-0,555882099
UPF3A	13	114281601..114305817	2,62	4,00	-0,385645513
CHAMP1	13	114314482..114337626	2,97	4,50	-0,379795963
OR11H12	14	18601117..18602097	-	-	#N/D
POTEM	14	18967434..19003752	0,02	0,01	0,415033091
POTEG	14	complement(19402486..19434341)	-	0,02	-4,758582517
OR11H2	14	complement(19712936..19713883)	0,02	-	3,967268276
OR4N2	14	19719015..19830253	-	-	0,188027316
OR4Q3	14	19743571..19749469	-	-	#N/D
OR4M1	14	19773504..19783696	-	-	#N/D
OR4K3	14	complement(19868197..19869143)	-	-	#N/D
OR4K2	14	19875142..19883932	-	-	0,188027316
OR4K5	14	19920607..19921578	-	-	#N/D
OR4K1	14	19930917..19936757	-	-	#N/D

OR4K15	14	19975591..19976565	-	-	#N/D
OR4Q2	14	19999913..20004760	-	-	#N/D
OR4K14	14	complement(20014143..20019307)	-	-	#N/D
OR4K13	14	complement(20029399..20036038)	-	-	0,188027316
OR4L1	14	20060045..20060983	-	-	#N/D
OR4K17	14	20110739..20122199	-	-	0,188027316
OR4N5	14	20138820..20145471	-	-	#N/D
OR11G2	14	20190896..20201075	0,00	0,01	-0,923955849
OR11H6	14	20223710..20224702	-	-	0,188027316
OR11H7	14	20229402..20230346	-	0,01	-2,89252005
OR11H4	14	20239286..20244349	-	0,01	-2,89252005
TTC5	14	complement(20256558..20305960)	1,50	2,74	-0,649168101
CCNB1IP1	14	complement(20311368..20333312)	6,84	8,67	-0,118121244
PARP2	14	20343615..20357904	11,08	10,19	0,344079916
TEP1	14	complement(20365667..20413501)	2,46	2,30	0,321845422
KLHL33	14	complement(20425852..20436166)	0,03	0,01	1,060045679
OSGEP	14	complement(20446401..20455089)	2,81	3,93	-0,262372526
APEX1	14	20455191..20457772	21,52	26,69	-0,087183309
PIP4P1	14	complement(20457681..20461465)	13,93	8,32	0,966631024
PNP	14	20461992..20477094	6,12	9,85	-0,462534983
RNASE10	14	20505537..20513884	0,11	0,08	0,754754074
RNASE9	14	complement(20556093..20561047)	-	-	#N/D
gene:ENSG000	14	complement(20582892..20609795)	-	-	#N/D
RNASE11	14	complement(20582892..20609884)	-	-	#N/D
RNASE12	14	complement(20590073..20591376)	-	-	#N/D
OR6S1	14	complement(20640696..20641691)	-	-	#N/D
ANG	14	20684177..20698971	2,04	1,50	0,669254265
RNASE4	14	20684560..20701216	1,63	0,75	1,348355011
gene:ENSG000	14	20684587..20700576	0,35	0,26	0,665122135
EDDM3A	14	20745887..20748380	-	-	#N/D
EDDM3B	14	20768404..20770948	-	-	#N/D
RNASE6	14	20781268..20782467	-	-	#N/D
RNASE1	14	complement(20801228..20802855)	-	-	0,188027316
RNASE3	14	20891385..20892348	-	-	0,188027316
RNASE2	14	20955487..20956436	-	-	0,188027316
METTL17	14	20989973..20997035	9,26	11,29	-0,062936083
SLC39A2	14	20999255..21001871	0,01	0,03	-1,478387936
NDRG2	14	complement(21016763..21070872)	1,53	0,74	1,275080957
TPPP2	14	21024109..21036276	-	-	#N/D
RNASE13	14	complement(21032818..21034807)	-	-	0,188027316
RNASE7	14	21042316..21044234	-	-	0,188027316
RNASE8	14	21057822..21058455	-	-	0,188027316
ARHGEF40	14	21070273..21090248	4,64	5,90	-0,124625716
ZNF219	14	complement(21090077..21104722)	0,81	1,70	-0,854116552
TMEM253	14	21098811..21103724	0,02	0,09	-1,983268216
OR5AU1	14	complement(21148370..21159060)	0,00	-	3,967268276
HNRNPC	14	complement(21209136..21269494)	46,27	62,00	-0,199012641
RPGRIP1	14	21280083..21351301	0,00	-	3,068695355

SUPT16H	14	complement(21351476..21384019)	12,07	20,31	-0,527168936
CHD8	14	complement(21385194..21456126)	9,32	10,50	0,051799913
RAB2B	14	complement(21459020..21476960)	5,25	5,68	0,109095257
TOX4	14	21476597..21499175	10,57	7,99	0,627452377
METTL3	14	complement(21498133..21511342)	10,58	12,30	0,005591964
SALL2	14	complement(21521080..21537216)	0,09	0,03	1,740757315
OR10G3	14	complement(21568520..21580076)	-	0,00	-2,89252005
TRAV1-1	14	21621838..21622567	-	-	#N/D
OR10G2	14	complement(21633836..21634940)	-	-	#N/D
TRAV1-2	14	21642889..21643578	-	-	#N/D
OR4E2	14	21653835..21667642	-	-	#N/D
OR4E1	14	complement(21667886..21673818)	-	-	0,188027316
TRAV2	14	21712321..21712843	-	-	#N/D
TRAV3	14	21723713..21724321	-	-	#N/D
TRAV4	14	21736152..21736982	-	-	#N/D
TRAV5	14	21749178..21749705	-	-	#N/D
TRAV6	14	21768489..21769080	-	-	#N/D
TRAV7	14	21782993..21783503	-	-	#N/D
TRAV8-1	14	21797287..21797886	-	-	#N/D
TRAV9-1	14	21811502..21811977	-	-	#N/D
TRAV10	14	21825472..21826075	-	-	#N/D
TRAV12-1	14	21841240..21841774	-	-	#N/D
TRAV8-2	14	21846537..21847221	-	-	#N/D
TRAV8-3	14	21852558..21853006	-	-	#N/D
gene:ENSG000	14	21861353..21862012	-	-	#N/D
TRAV13-1	14	21868839..21869365	-	-	#N/D
TRAV12-2	14	21887857..21888502	-	-	#N/D
TRAV8-4	14	21894433..21895030	-	-	#N/D
TRAV13-2	14	21918188..21918756	-	-	0,188027316
TRAV14DV4	14	21924063..21924651	-	-	#N/D
TRAV9-2	14	21941128..21941657	-	-	#N/D
TRAV12-3	14	21965451..21966061	-	-	#N/D
TRAV8-6	14	21978459..21979120	-	-	0,188027316
TRAV16	14	21990496..21990938	-	-	0,188027316
TRAV17	14	21997539..21998168	-	-	#N/D
TRAV18	14	22003106..22003673	-	-	#N/D
TRAV19	14	22007512..22008181	-	-	0,188027316
TRAV20	14	22040594..22041153	-	-	#N/D
TRAV21	14	22052514..22053056	-	-	#N/D
TRAV22	14	22070557..22071208	-	-	#N/D
TRAV23DV6	14	22086407..22086961	-	-	0,188027316
TRDV1	14	22096032..22096619	-	-	#N/D
TRAV24	14	22105343..22105846	-	-	#N/D
TRAV25	14	22112347..22113031	-	-	#N/D
TRAV26-1	14	22123318..22124285	-	-	#N/D
TRAV8-7	14	22132553..22133034	-	-	#N/D
TRAV27	14	22147995..22148633	-	-	#N/D
TRAV29DV5	14	22163238..22163870	-	-	#N/D

TRAV30	14	22168429..22168988	-	-	0,188027316
TRAV26-2	14	22202583..22203368	-	-	#N/D
TRAV34	14	22207522..22208129	-	-	#N/D
TRAV36DV7	14	22226746..22227254	-	-	#N/D
TRAV38-1	14	22271968..22272563	-	-	#N/D
TRAV38-2DV8	14	22281105..22281748	-	-	#N/D
TRAV39	14	22304054..22304553	-	-	#N/D
TRAV40	14	22314490..22314919	-	-	#N/D
TRAV41	14	22320188..22320691	-	-	0,188027316
TRDV2	14	22422371..22423042	-	-	0,188027316
TRDD1	14	22438547..22438554	-	-	#N/D
TRDD2	14	22439007..22439015	-	-	#N/D
TRDD3	14	22449113..22449125	-	-	#N/D
TRDJ1	14	22450089..22450139	-	-	#N/D
TRDJ4	14	22455249..22455296	-	-	#N/D
TRDJ2	14	22456689..22456742	-	-	#N/D
TRDJ3	14	22459098..22459156	-	-	#N/D
TRDC	14	22462932..22465787	-	0,00	-2,89252005
TRDV3	14	complement(22469041..22469698)	-	-	#N/D
TRAJ61	14	22475316..22475375	-	-	#N/D
TRAJ59	14	22476553..22476606	-	-	#N/D
TRAJ58	14	22477707..22477769	-	-	#N/D
TRAJ57	14	22478872..22478934	-	-	#N/D
TRAJ56	14	22479521..22479582	-	-	#N/D
TRAJ54	14	22482287..22482346	-	-	#N/D
TRAJ53	14	22483004..22483069	-	-	#N/D
TRAJ52	14	22486228..22486296	-	-	#N/D
TRAJ50	14	22488593..22488652	-	-	#N/D
TRAJ49	14	22489488..22489543	-	-	#N/D
TRAJ48	14	22490491..22490553	-	-	#N/D
TRAJ47	14	22492851..22492907	-	-	#N/D
TRAJ46	14	22493403..22493465	-	-	#N/D
TRAJ45	14	22493925..22493990	-	-	#N/D
TRAJ44	14	22494821..22494883	-	-	#N/D
TRAJ43	14	22495913..22495966	-	-	#N/D
TRAJ42	14	22496887..22496952	-	-	#N/D
TRAJ41	14	22497657..22497718	-	-	#N/D
TRAJ40	14	22499689..22499749	-	-	#N/D
TRAJ39	14	22501601..22501663	-	-	#N/D
TRAJ38	14	22502231..22502292	-	-	#N/D
TRAJ37	14	22503750..22503814	-	-	#N/D
TRAJ36	14	22505110..22505167	-	-	#N/D
TRAJ35	14	22506644..22506702	-	-	#N/D
TRAJ34	14	22507666..22507723	-	-	#N/D
TRAJ33	14	22508602..22508658	-	-	#N/D
TRAJ32	14	22509341..22509406	-	-	#N/D
TRAJ31	14	22510968..22511024	-	-	#N/D
TRAJ30	14	22512852..22512908	-	-	#N/D

TRAJ29	14	22513939..22513998	-	-	#N/D
TRAJ28	14	22515623..22515688	-	-	#N/D
TRAJ27	14	22516273..22516331	-	-	#N/D
TRAJ26	14	22518446..22518505	-	-	#N/D
TRAJ25	14	22518812..22518871	-	-	#N/D
TRAJ24	14	22519969..22520031	-	-	#N/D
TRAJ23	14	22520416..22520478	-	-	#N/D
TRAJ22	14	22522040..22522102	-	-	#N/D
TRAJ21	14	22523600..22523654	-	-	#N/D
TRAJ20	14	22524325..22524381	-	-	#N/D
TRAJ19	14	22525263..22525322	-	-	#N/D
TRAJ18	14	22525650..22525715	-	-	#N/D
TRAJ17	14	22526844..22526906	-	-	#N/D
TRAJ16	14	22528527..22528586	-	-	#N/D
TRAJ14	14	22530327..22530378	-	-	#N/D
TRAJ13	14	22531076..22531138	-	-	#N/D
TRAJ12	14	22531939..22531998	-	-	#N/D
TRAJ11	14	22532502..22532561	-	-	#N/D
TRAJ10	14	22533497..22533560	-	-	#N/D
TRAJ9	14	22535554..22535614	-	-	#N/D
TRAJ7	14	22537622..22537680	-	-	#N/D
TRAJ6	14	22539073..22539134	-	-	#N/D
TRAJ5	14	22540247..22540306	-	-	#N/D
TRAJ4	14	22542199..22542261	-	-	#N/D
TRAJ3	14	22543179..22543240	-	-	#N/D
TRAJ2	14	22544071..22544136	-	-	#N/D
TRAJ1	14	22545037..22545098	-	-	#N/D
TRAC	14	22547506..22552156	0,02	0,04	-0,833928617
DAD1	14	complement(22564907..22589224)	65,04	53,32	0,509687586
ABHD4	14	22598290..22612963	5,07	7,38	-0,319494538
OR6J1	14	complement(22630929..22644352)	0,00	0,01	-0,923955849
OXA1L	14	22766522..22773042	13,48	15,16	0,053530761
SLC7A7	14	complement(22773222..22829820)	0,11	0,12	0,103812933
MRPL52	14	22829879..22835037	10,38	15,76	-0,379386461
MMP14	14	22836560..22849041	97,21	87,09	0,381803041
LRP10	14	22871740..22881713	19,31	18,64	0,273670008
REM2	14	22883222..22887678	0,26	0,16	0,98255903
RBM23	14	complement(22893204..22919182)	1,28	2,15	-0,522008141
PRMT5	14	complement(22920525..22929408)	19,09	29,89	-0,424174723
HAUS4	14	complement(22946228..22957161)	7,96	8,41	0,143076596
gene:ENSG000	14	complement(22946270..22982258)	-	-	0,188027316
AJUBA	14	complement(22971177..22982551)	2,91	6,75	-0,99192162
C14orf93	14	complement(22985894..23010166)	1,69	2,01	-0,031508649
gene:ENSG000	14	complement(23005499..23007123)	0,19	0,31	-0,465459939
PSMB5	14	complement(23016543..23035230)	53,75	62,07	0,015509597
gene:ENSG000	14	complement(23023083..23024217)	0,09	0,07	0,686693064
PSMB11	14	23042212..23044060	-	0,01	-2,89252005
CDH24	14	complement(23047062..23057538)	3,74	4,14	0,073729582

ACIN1	14	complement(23058564..23095614)	20,88	22,09	0,141832123
C14orf119	14	23095505..23100456	8,24	8,33	0,205996141
CIROP	14	complement(23099062..23104989)	0,04	0,06	-0,27623233
CEBPE	14	complement(23117036..23120256)	0,03	0,01	1,436225396
SLC7A8	14	complement(23125295..23183674)	1,20	0,77	0,851579695
RNF212B	14	23185316..23273477	-	-	0,188027316
HOMEZ	14	complement(23272422..23299796)	0,44	0,53	-0,034370783
PPP1R3E	14	complement(23295652..23302859)	1,82	1,55	0,456159711
BCL2L2	14	23298790..23311751	7,39	6,55	0,396638559
BCL2L2-PABPN	14	23306833..23326175	4,51	4,71	0,158646159
PABPN1	14	23321457..23326163	26,74	27,91	0,161253763
SLC22A17	14	complement(23346314..23352912)	7,79	3,12	1,544693571
EFS	14	complement(23356403..23365752)	12,75	12,66	0,232825329
IL25	14	23372809..23376403	0,03	-	4,913749003
CMTM5	14	23376773..23379772	0,01	-	3,068695355
MYH6	14	complement(23381982..23408273)	-	-	0,188027316
MYH7	14	complement(23412740..23435660)	-	-	0,188027316
NGDN	14	23469679..23509862	2,39	3,21	-0,205517283
ZFHX2	14	complement(23520857..23556192)	0,04	0,24	-2,486967143
THTPA	14	23555988..23560271	1,79	1,91	0,125663578
AP1G2	14	complement(23559565..23568070)	6,01	8,86	-0,335639728
JPH4	14	complement(23568038..23578790)	0,01	0,02	-1,009277154
DHRS2	14	23630115..23645639	0,07	0,09	-0,144216092
DHRS4	14	23953734..23969279	1,93	2,08	0,116543267
DHRS4L2	14	23969874..24006408	1,89	1,96	0,171722857
CARMIL3	14	24052009..24069729	0,05	0,02	1,875658198
CPNE6	14	24070837..24078100	0,01	-	4,516772761
NRL	14	complement(24078662..24115010)	0,08	0,13	-0,401282968
PCK2	14	24094053..24110598	2,87	12,69	-1,923203148
DCAF11	14	24114195..24125242	7,88	7,51	0,291290247
gene:ENSG000	14	24120956..24131829	0,01	0,05	-1,478387936
FITM1	14	24130659..24132849	0,11	0,04	1,745345563
PSME1	14	24136163..24138967	30,84	36,77	-0,030502137
EMC9	14	complement(24138959..24141591)	6,51	7,11	0,094995263
PSME2	14	complement(24143362..24147570)	16,44	19,39	-0,015010611
RNF31	14	24146683..24160660	8,45	6,71	0,556360512
gene:ENSG000	14	24147548..24166452	2,09	1,97	0,311031037
IRF9	14	24161234..24168043	4,11	1,26	1,930377365
REC8	14	24171853..24180257	1,75	0,66	1,627785125
IPO4	14	complement(24180219..24188869)	14,68	16,35	0,067809894
gene:ENSG000	14	complement(24180395..24190416)	0,86	0,48	1,083713211
TM9SF1	14	complement(24189149..24195687)	12,91	11,91	0,339258724
gene:ENSG000	14	complement(24189157..24213473)	0,18	0,21	-0,008546567
TSSK4	14	24205696..24208362	0,37	0,43	0,015095183
CHMP4A	14	complement(24209615..24213830)	2,04	2,92	-0,295073887
MDP1	14	complement(24213943..24216070)	1,46	3,30	-0,948474763
NEDD8-MDP1	14	complement(24213955..24232352)	-	-	0,188027316
NEDD8	14	complement(24216857..24232367)	36,42	38,73	0,134326183

GMPR2	14	24232422..24239242	9,53	9,82	0,180480144
TINF2	14	complement(24238286..24242663)	5,47	6,52	-0,030156943
TGM1	14	complement(24249114..24264432)	0,41	0,47	0,000831926
RABGGTA	14	complement(24265538..24271611)	5,54	7,07	-0,129284173
DHRS1	14	complement(24290598..24299780)	4,27	2,58	0,949803504
NOP9	14	24299850..24309124	6,75	6,65	0,244516876
CIDEB	14	complement(24305187..24311430)	-	-	0,188027316
LTB4R2	14	24305734..24312053	3,31	3,47	0,151975759
LTB4R	14	24311450..24318036	3,62	3,79	0,157681629
ADCY4	14	complement(24318349..24335093)	0,00	0,01	-0,025382928
RIPK3	14	complement(24336025..24340022)	0,01	0,01	-0,923955849
NFATC4	14	24365673..24379604	6,94	9,15	-0,175220398
NYNRIN	14	24399003..24419283	0,64	0,50	0,581186369
CBLN3	14	complement(24426545..24430954)	0,39	1,13	-1,309826268
KHNYN	14	24429286..24441843	7,65	8,90	0,003439152
SDR39U1	14	complement(24439766..24442905)	3,74	6,22	-0,511470194
CMA1	14	complement(24505353..24508265)	-	-	#N/D
CTSG	14	complement(24573518..24576250)	-	-	0,188027316
GZMH	14	complement(24606480..24609699)	-	-	#N/D
GZMB	14	complement(24630954..24634267)	-	-	#N/D
STXBP6	14	complement(24809454..25050147)	0,00	0,04	-2,850473978
gene:ENSG000	14	25820366..25822741	-	-	#N/D
NOVA1	14	complement(26443090..26598033)	0,20	0,47	-1,019390242
FOXG1	14	28764329..28770277	0,05	0,02	1,170931565
PRKD1	14	complement(29576479..30191898)	1,99	3,79	-0,701676823
gene:ENSG000	14	30370108..30373115	-	-	#N/D
G2E3	14	30559158..30620064	1,12	1,83	-0,486690911
SCFD1	14	30622291..30737694	1,72	1,84	0,126012279
COCH	14	30874514..30895065	1,10	0,04	4,830440182
STRN3	14	complement(30893799..31026401)	2,42	3,80	-0,428776084
AP4S1	14	31025106..31130996	0,12	0,20	-0,483377682
HECTD1	14	complement(31100117..31207804)	10,34	11,07	0,124426455
HEATR5A	14	complement(31291788..31420550)	3,23	3,30	0,188398612
gene:ENSG000	14	complement(31334312..31457441)	1,66	0,30	2,7108357
DTD2	14	complement(31446036..31457506)	0,78	1,89	-1,043616274
GPR33	14	complement(31482875..31488039)	-	0,01	-2,89252005
NUBPL	14	31489956..31861224	1,07	1,52	-0,274605199
ARHGAP5	14	32076114..32159728	1,25	2,56	-0,810304152
AKAP6	14	32329298..32837684	1,84	0,15	3,84699583
gene:ENSG000	14	32879246..32882830	-	-	#N/D
NPAS3	14	32934396..33820863	0,13	0,16	-0,075619623
EGLN3	14	complement(33924227..34462774)	0,58	0,19	1,81429044
SPTSSA	14	complement(34432788..34462240)	3,33	2,03	0,934565599
EAPP	14	complement(34515938..34539704)	3,23	3,95	-0,065016757
gene:ENSG000	14	34541044..34541714	4,29	3,96	0,340238418
SNX6	14	complement(34561093..34630160)	3,22	6,59	-0,811476736
CFL2	14	complement(34709113..34714823)	1,82	3,54	-0,732139664
BAZ1A	14	complement(34752731..34875647)	1,67	4,45	-1,195792453

SRP54	14	34981957..35029686	16,55	11,29	0,774563361
FAM177A1	14	35045117..35116630	2,36	2,09	0,39467842
PPP2R3C	14	complement(35085467..35122517)	0,80	0,89	0,076142514
gene:ENSG000	14	35086035..35088059	0,21	0,27	-0,146595544
PRORP	14	35121846..35277622	1,61	2,30	-0,2908947
gene:ENSG000	14	35122549..35317474	3,12	3,84	-0,076630572
PSMA6	14	35278633..35317493	25,26	36,82	-0,320929441
NFKBIA	14	complement(35401079..35404749)	28,67	32,19	0,055954965
INSM2	14	35534164..35537054	-	0,01	-4,359055975
RALGAPA1	14	complement(35538352..35809304)	1,18	2,21	-0,675851234
BRMS1L	14	35826338..35932325	0,39	0,76	-0,728093577
MBIP	14	complement(36298564..36320637)	2,83	2,87	0,202892393
SFTA3	14	complement(36473207..36521149)	-	-	#N/D
NKX2-1	14	complement(36516392..36521149)	0,29	0,04	3,012989978
NKX2-8	14	complement(36580004..36582614)	0,05	0,06	-0,204080844
PAX9	14	36657568..36679362	1,70	1,05	0,920232947
SLC25A21	14	complement(36677921..37172606)	0,17	0,41	-0,992331441
MIPOL1	14	37197894..37579125	0,17	0,25	-0,326092144
gene:ENSG000	14	37258724..37261129	0,01	0,01	-0,025382928
FOXA1	14	complement(37589552..37596059)	0,35	0,63	-0,637280067
TTC6	14	37595629..38041442	0,01	0,00	2,399873901
SSTR1	14	38207904..38213067	0,39	0,17	1,471656598
CLEC14A	14	complement(38254000..38256093)	-	-	#N/D
SEC23A	14	complement(39031919..39109646)	21,13	16,67	0,564869657
GEMIN2	14	39114285..39136973	1,31	2,29	-0,57820502
TRAPPC6B	14	complement(39147811..39170532)	1,20	1,43	-0,025411378
PNN	14	39175183..39183220	7,27	9,33	-0,13776349
MIA2	14	39230231..39388513	1,11	1,71	-0,40001038
FBXO33	14	complement(39397684..39432466)	2,87	2,51	0,42135779
gene:ENSG000	14	complement(41514972..41515350)	-	-	#N/D
LRFN5	14	41606876..41904549	0,02	0,01	0,947848749
FSCB	14	complement(44504149..44507283)	-	-	#N/D
C14orf28	14	44897275..44907257	0,16	0,25	-0,445635695
KLHL28	14	complement(44924324..45042322)	1,46	0,72	1,237043028
TOGARAM1	14	44962190..45074431	1,63	1,58	0,271551987
PRPF39	14	45084107..45116282	1,80	2,29	-0,125332025
FKBP3	14	complement(45115599..45135319)	1,82	3,92	-0,880585872
FANCM	14	45135930..45200890	0,46	0,57	-0,099051503
MIS18BP1	14	complement(45203190..45253540)	1,38	2,48	-0,627948204
RPL10L	14	complement(46651010..46651781)	-	-	#N/D
MDGA2	14	complement(46840092..47675605)	0,06	0,08	-0,12919867
RPS29	14	complement(49570984..49599164)	7,61	8,13	0,127767661
LRR1	14	49598761..49614672	2,05	3,85	-0,687185945
RPL36AL	14	complement(49618530..49620626)	18,26	22,35	-0,068307688
MGAT2	14	49620799..49623481	5,04	7,78	-0,405181989
DNAAF2	14	complement(49625174..49635244)	0,81	1,65	-0,799080749
POLE2	14	complement(49643555..49688422)	0,81	2,00	-1,086835071
KLHDC1	14	49693105..49753150	0,06	0,09	-0,19821929

KLHDC2	14	49768130..49786385	3,04	3,40	0,065889917
NEMF	14	complement(49782083..49852821)	1,23	2,19	-0,612303735
ARF6	14	49893079..49897054	16,35	16,87	0,178323206
VCPKMT	14	complement(50108632..50116600)	1,72	1,06	0,914053948
SOS2	14	complement(50117130..50231578)	1,16	1,83	-0,435094944
L2HGDH	14	complement(50237563..50312229)	1,11	1,44	-0,146803472
DMAC2L	14	50312324..50335558	0,95	1,08	0,034812878
CDKL1	14	complement(50326265..50416461)	0,60	1,54	-1,1254995
MAP4K5	14	complement(50418501..50561126)	4,26	5,64	-0,182596528
ATL1	14	50532509..50634017	0,01	0,02	-0,446980808
SAV1	14	complement(50632058..50668306)	1,38	3,51	-1,122660023
NIN	14	complement(50719763..50831162)	1,54	3,09	-0,7843095
PYGL	14	complement(50857891..50944483)	5,13	5,38	0,152892518
ABHD12B	14	50872053..50904970	0,01	-	3,967268276
TRIM9	14	complement(50975262..51096061)	0,14	0,23	-0,47160808
TMX1	14	51240162..51257655	2,64	3,35	-0,120121011
FRMD6	14	51489100..51730727	10,10	14,22	-0,271533051
GNG2	14	51826195..51979342	1,44	1,18	0,510099849
RTRAF	14	51989514..52010694	4,02	4,76	-0,020375794
NID2	14	complement(52004809..52069059)	2,18	2,13	0,252364873
PTGDR	14	52267698..52276724	-	-	0,188027316
PTGER2	14	52314305..52328598	1,66	0,48	2,00010634
TXNDC16	14	complement(52430596..52552522)	0,60	0,55	0,350295039
GPR137C	14	52552836..52637713	0,17	0,10	1,022553962
ERO1A	14	complement(52639915..52695900)	7,23	10,54	-0,320827103
PSMC6	14	52707178..52728590	4,81	6,56	-0,225604197
STYX	14	52730166..52774989	2,11	1,84	0,426467254
GNPNAT1	14	complement(52775193..52791668)	4,41	8,09	-0,652223629
FERMT2	14	complement(52857268..52952435)	7,51	10,55	-0,266919339
DDHD1	14	complement(53036745..53153323)	0,86	1,63	-0,703178769
BMP4	14	complement(53949736..53958761)	0,05	0,08	-0,335149681
CDKN3	14	54396849..54420218	7,89	7,82	0,235137808
CNIH1	14	complement(54423561..54441391)	4,16	6,40	-0,397778464
GMFB	14	complement(54474484..54489025)	0,74	1,40	-0,685714205
CGRRF1	14	54509812..54539292	0,54	0,98	-0,632788217
SAMD4A	14	54566117..54793315	8,53	7,30	0,447174795
GCH1	14	complement(54842008..54902826)	0,28	0,83	-1,364662485
WDHD1	14	complement(54938949..55027105)	2,25	2,93	-0,157770258
SOCS4	14	55027230..55049489	1,98	1,89	0,285232887
MAPK1IP1L	14	55051647..55070194	4,20	6,13	-0,321637984
LGALS3	14	55124110..55145423	18,00	21,74	-0,049612521
DLGAP5	14	complement(55148112..55191608)	8,85	11,53	-0,159020221
FBXO34	14	55271344..55370053	3,55	4,13	0,004728941
ATG14	14	complement(55366391..55411830)	2,01	2,30	0,029779381
TBPL2	14	complement(55414210..55456726)	0,02	0,01	1,833201638
KTN1	14	55559072..55701526	5,24	10,28	-0,747812936
PELI2	14	56117814..56301524	0,78	1,60	-0,810488105
TMEM260	14	56488354..56650606	0,47	0,57	-0,05306405

OTX2	14	complement(56799905..56816693)	-	-	#N/D
EXOC5	14	complement(57200507..57268905)	1,34	2,46	-0,655583717
AP5M1	14	57268924..57298742	0,53	1,02	-0,729521772
NAA30	14	57390544..57415906	1,00	1,38	-0,239520781
CCDC198	14	complement(57469300..57493867)	0,01	-	4,516772761
SLC35F4	14	complement(57563920..57982194)	-	0,00	-2,89252005
ARMH4	14	complement(57999735..58298139)	0,01	0,00	2,144183439
ACTR10	14	58200080..58235636	2,77	4,52	-0,484183643
PSMA3	14	58244843..58272012	20,78	31,18	-0,362150339
ARID4A	14	58298504..58373887	0,68	0,74	0,107063464
TOMM20L	14	58395928..58408702	0,02	-	3,068695355
TIMM9	14	complement(58408495..58427531)	3,34	4,99	-0,35868981
KIAA0586	14	58427385..58551297	0,87	1,45	-0,511994233
DACT1	14	58633967..58648321	0,29	0,18	0,921933821
DAAM1	14	59188646..59371405	0,52	0,77	-0,348765727
GPR135	14	complement(59429022..59465380)	0,57	1,03	-0,629088274
L3HYPDH	14	complement(59460363..59484408)	2,30	3,35	-0,320199851
JKAMP	14	59484443..59505410	4,15	5,73	-0,244137366
CCDC175	14	complement(59504539..59576812)	-	-	0,188027316
RTN1	14	complement(59595976..59870776)	0,79	2,64	-1,510234736
LRRC9	14	59919713..60066817	0,00	-	3,967268276
PCNX4	14	60091911..60169133	0,69	1,48	-0,889496478
DHRS7	14	complement(60144119..60169856)	1,97	2,55	-0,148304831
PPM1A	14	60245752..60299087	1,63	2,08	-0,134167902
C14orf39	14	complement(60396469..60515543)	-	-	0,188027316
SIX6	14	60509146..60512850	-	-	0,188027316
SIX1	14	complement(60643421..60658259)	3,94	3,80	0,27475998
SIX4	14	complement(60709539..60724351)	3,77	3,28	0,426337286
MNAT1	14	60734742..60969965	4,28	5,05	-0,014560273
TRMT5	14	complement(60971441..60981170)	1,08	1,93	-0,614321062
SLC38A6	14	60981114..61114316	0,49	0,85	-0,58708225
PRKCH	14	61187559..61550976	2,06	2,90	-0,269471288
TMEM30B	14	complement(61277370..61281761)	0,02	0,01	1,487770062
gene:ENSG000	14	61529128..61657964	0,07	0,52	-2,634741553
HIF1A	14	61695513..61748259	49,71	51,65	0,167705456
SNAPC1	14	61762420..61796428	2,01	4,25	-0,859470971
gene:ENSG000	14	61801713..61803634	0,06	0,13	-0,954535799
SYT16	14	61811974..62112550	0,01	-	6,95890237
gene:ENSG000	14	62075340..62128421	0,03	-	5,480421266
KCNH5	14	complement(62699464..63102037)	0,00	-	3,068695355
RHOJ	14	63204114..63293508	-	-	0,188027316
GPHB5	14	complement(63312835..63318935)	-	-	#N/D
PPP2R5E	14	complement(63371364..63543377)	2,87	3,61	-0,107355074
WDR89	14	complement(63597039..63641871)	1,29	1,56	-0,051990926
SGPP1	14	complement(63684216..63728065)	2,10	2,74	-0,155774839
SYNE2	14	63761899..64226433	4,77	2,17	1,361046541
ESR2	14	complement(64084232..64338112)	0,10	0,07	0,73397893
MTHFD1	14	64388031..64463457	9,71	11,48	-0,018542332

ZBTB25	14	complement(64449106..64505213)	1,10	0,90	0,506292518
AKAP5	14	64465499..64474503	0,05	0,05	0,145728619
ZBTB1	14	64503712..64533690	1,48	1,47	0,236078363
HSPA2	14	64535905..64546173	1,76	0,80	1,36262202
PPP1R36	14	64549920..64589381	0,01	0,04	-1,291882393
PLEKHG3	14	64704102..64750249	2,28	3,95	-0,570210509
SPTB	14	complement(64746283..64879907)	0,05	0,03	1,289809822
CHURC1	14	64914361..64944591	1,03	1,36	-0,178008184
CHURC1-FNTB	14	64914485..65061803	0,62	0,61	0,251213234
GPX2	14	complement(64939152..64942746)	0,29	0,20	0,733826537
RAB15	14	complement(64945814..64973226)	0,55	0,97	-0,586958192
FNTB	14	64986895..65062652	4,06	5,03	-0,086780693
MAX	14	complement(65006174..65102695)	6,41	4,52	0,726792868
FUT8	14	65410592..65744121	1,32	8,45	-2,452189932
CCDC196	14	66411704..66498677	-	-	#N/D
GPHN	14	66507407..67181803	0,08	0,03	1,420083537
GARIN2	14	67189393..67228550	-	-	0,188027316
PALS1	14	67240713..67336061	2,49	2,76	0,076719353
ATP6V1D	14	complement(67294371..67360265)	4,03	3,51	0,424233035
EIF2S1	14	67360328..67386516	7,30	9,05	-0,086983667
PLEK2	14	complement(67386984..67412167)	3,67	4,48	-0,065758451
TMEM229B	14	complement(67447084..67533739)	0,11	0,09	0,565739302
PLEKHH1	14	67533290..67589612	0,24	0,40	-0,513480518
PIGH	14	complement(67581955..67600286)	1,30	1,53	-0,015333739
ARG2	14	67619920..67651708	3,19	3,47	0,103798004
VTI1B	14	complement(67647085..67674820)	4,06	5,14	-0,114862805
gene:ENSG000	14	complement(67659820..67690367)	-	-	0,188027316
RDH11	14	complement(67676800..67695793)	12,46	12,62	0,204718615
RDH12	14	67701886..67734451	0,02	0,03	-0,429837071
ZFYVE26	14	complement(67727374..67816590)	2,00	1,87	0,319757285
RAD51B	14	67819779..68730218	0,67	0,97	-0,296528842
ZFP36L1	14	complement(68787660..68796253)	8,40	13,37	-0,447669577
ACTN1	14	complement(68874128..68979440)	45,08	55,65	-0,080856712
DCAF5	14	complement(69050881..69153150)	4,42	4,90	0,075287178
EXD2	14	69191498..69244020	5,01	5,17	0,175163679
GALNT16	14	69259277..69357033	0,55	1,36	-1,089499816
gene:ENSG000	14	69357010..69359308	0,37	0,13	1,733817544
ERH	14	complement(69380128..69398299)	15,76	26,53	-0,528562636
SLC39A9	14	69398015..69462390	18,55	11,92	0,860818974
PLEKHD1	14	69484692..69531551	0,00	0,01	-1,478387936
CCDC177	14	complement(69569799..69574871)	-	0,02	-5,071123353
SUSD6	14	69611596..69715144	4,84	5,01	0,17219316
SRSF5	14	69726900..69772005	15,43	13,86	0,378461652
SLC10A1	14	complement(69775416..69797241)	0,10	0,13	-0,112641019
SMOC1	14	69854131..70032366	3,12	6,81	-0,901504501
SLC8A3	14	complement(70044215..70189480)	-	-	#N/D
COX16	14	complement(70325081..70416984)	4,01	3,69	0,342021758
SYNJ2BP-COX1	14	complement(70326064..70417074)	3,08	2,88	0,321118631

SYNJ2BP	14	complement(70366499..70417090)	1,82	1,91	0,156953863
ADAM21	14	70417107..70460427	0,26	0,20	0,647112565
ADAM20	14	complement(70522358..70535015)	0,33	0,20	0,940416551
MED6	14	complement(70581257..70600690)	3,45	3,95	0,027316829
TTC9	14	70641916..70675366	-	0,00	-2,89252005
MAP3K9	14	complement(70722526..70809534)	0,07	0,25	-1,661167107
PCNX1	14	70907405..71115382	4,45	5,59	-0,105954318
SIPA1L1	14	71320449..71741229	1,29	1,77	-0,234645174
RGS6	14	71932429..72566530	-	0,01	-4,758582517
DPF3	14	complement(72609034..72894101)	0,37	1,68	-1,945984094
DCAF4	14	72926377..72959703	3,45	7,78	-0,94947838
ZFYVE1	14	complement(72969451..73027131)	3,62	2,68	0,654614444
RBM25	14	73058532..73123899	3,00	3,43	0,028615967
PSEN1	14	73136418..73223691	2,23	3,20	-0,299017482
PAPLN	14	73237497..73274640	0,11	0,15	-0,147075548
NUMB	14	complement(73275107..73458617)	7,78	8,97	0,018958122
HEATR4	14	complement(73478484..73558947)	0,06	0,11	-0,600639664
RIOX1	14	73490933..73493394	1,57	4,31	-1,234389946
ACOT1	14	73537143..73543796	0,08	0,18	-0,916110795
ACOT2	14	73567620..73575658	1,89	3,68	-0,735403154
gene:ENSG000	14	73580694..73582918	0,02	0,10	-1,854949791
ACOT4	14	73591873..73595766	0,11	0,33	-1,33363117
ACOT6	14	73610945..73619888	-	0,01	-2,89252005
DNAL1	14	73644875..73703732	0,60	0,63	0,161777541
PNMA1	14	complement(73711783..73714384)	4,68	8,19	-0,583441436
MIDEAS	14	complement(73715122..73790285)	10,56	10,37	0,250397375
PTGR2	14	73851831..73885726	1,41	2,20	-0,417750745
gene:ENSG000	14	73851971..73932278	0,18	0,29	-0,501052647
ZNF410	14	73886617..73932521	4,51	5,99	-0,187585296
FAM161B	14	complement(73931501..73950414)	0,70	0,54	0,588527472
COQ6	14	73949926..73963670	2,49	2,49	0,225862725
ENTPD5	14	complement(73958010..74019399)	1,02	1,66	-0,483637127
BBOF1	14	74019349..74082863	0,18	0,24	-0,186798546
ALDH6A1	14	complement(74056847..74084492)	3,10	3,40	0,086249855
LIN52	14	74084956..74201493	2,90	2,70	0,324844627
VSX2	14	74239449..74262738	-	-	0,188027316
ABCD4	14	complement(74285269..74303055)	3,33	3,63	0,096757637
VRTN	14	74303069..74360008	-	-	#N/D
SYNDIG1L	14	complement(74405894..74426210)	0,03	0,00	2,805678585
NPC2	14	complement(74476192..74494177)	7,13	6,98	0,253223632
ISCA2	14	74493756..74497106	1,44	2,46	-0,548963653
LTBP2	14	complement(74498183..74612378)	2,08	3,53	-0,542772084
AREL1	14	complement(74653437..74713117)	6,41	7,63	-0,026969705
FCF1	14	74713144..74738620	5,45	5,92	0,103349097
YLPM1	14	74763316..74859435	3,91	6,07	-0,409890159
PROX2	14	complement(74852871..74876154)	0,01	0,07	-2,382179099
DLST	14	74881891..74903743	21,34	23,65	0,074639418
RPS6KL1	14	complement(74903951..74923302)	0,33	0,36	0,068851119

PGF	14	complement(74941834..74955626)	27,65	9,98	1,69345258
EIF2B2	14	75002921..75012366	7,74	5,11	0,822512311
gene:ENSG000	14	complement(75011269..75012851)	0,14	0,07	1,268358442
MLH3	14	complement(75013769..75051532)	0,92	1,82	-0,764064599
ACYP1	14	complement(75053237..75069483)	2,74	1,71	0,906330959
ZC2HC1C	14	75064109..75119502	0,06	0,15	-1,082514907
NEK9	14	complement(75079353..75127344)	5,56	8,22	-0,340362079
TMED10	14	complement(75131469..75176612)	30,75	31,89	0,170507453
FOS	14	75278826..75282230	0,53	0,85	-0,462990329
JDP2	14	75427716..75474111	1,90	2,63	-0,242342315
BATF	14	75522455..75546993	-	-	0,188027316
FLVCR2	14	75578620..75663214	0,03	0,09	-1,42562615
TTLL5	14	75633625..75955079	2,90	2,69	0,333448365
ERG28	14	complement(75649791..75660876)	6,10	3,63	0,973347444
IFT43	14	75902136..76084585	3,81	4,01	0,147146996
TGFB3	14	complement(75958097..75983011)	0,33	0,48	-0,327587783
GPATCH2L	14	76151916..76254342	2,44	2,76	0,040428633
gene:ENSG000	14	76279173..76283103	0,01	0,02	-0,345401666
ESRRB	14	76310712..76501837	0,04	0,14	-1,733006195
VASH1	14	76761468..76783015	1,19	2,60	-0,902557768
ANGEL1	14	complement(76781733..76826246)	3,60	3,79	0,149311087
LRRC74A	14	76826372..76870304	-	-	#N/D
IRF2BPL	14	complement(77024543..77028708)	10,39	12,04	0,010686537
CIPC	14	77098126..77117287	3,63	3,05	0,477071169
gene:ENSG000	14	77098311..77225462	-	0,04	-3,804623889
TMEM63C	14	77116568..77259495	0,00	0,03	-2,94381988
ZDHHC22	14	complement(77131270..77142734)	-	-	0,188027316
NGB	14	complement(77265483..77271206)	-	-	#N/D
POMT2	14	complement(77274956..77320883)	3,04	4,57	-0,365985582
GSTZ1	14	77320996..77331597	3,17	3,57	0,050621235
TMED8	14	complement(77335029..77377094)	3,34	4,97	-0,348445688
SAMD15	14	77376689..77392163	0,05	0,06	-0,242839224
NOXRED1	14	complement(77394021..77423523)	0,04	0,06	-0,322898219
VIPAS39	14	complement(77426675..77457952)	4,79	5,95	-0,089798981
AHSA1	14	77457870..77469472	13,13	20,67	-0,430895248
ISM2	14	complement(77474394..77498816)	0,08	0,12	-0,245074778
SPTLC2	14	complement(77505997..77616637)	2,54	2,64	0,167962256
ALKBH1	14	complement(77672404..77708023)	1,69	2,15	-0,126776295
SLIRP	14	77708071..77761104	4,23	6,51	-0,399383676
SNW1	14	complement(77717599..77761207)	13,13	15,67	-0,032983338
ADCK1	14	77800109..77935014	0,58	0,94	-0,453592696
NRXN3	14	78170373..79868291	-	-	0,188027316
DIO2	14	complement(80197526..80387757)	0,02	0,03	-0,573028908
CEP128	14	complement(80476983..80959517)	0,57	0,98	-0,562122662
TSHR	14	80954989..81146306	0,00	0,00	0,886720911
GTF2A1	14	complement(81175452..81221377)	2,18	3,55	-0,479829307
STON2	14	complement(81260652..81436465)	0,00	0,13	-5,753671789
SEL1L	14	complement(81471547..81533853)	7,39	7,21	0,258675395

FLRT2	14	85530144..85654428	0,50	0,38	0,627235353
GALC	14	complement(87837820..87993665)	-	-	0,188027316
GPR65	14	88005135..88014811	0,00	0,01	-0,923955849
KCNK10	14	complement(88180103..88326907)	-	0,00	-2,89252005
SPATA7	14	88384924..88470350	0,12	0,38	-1,456572761
PTPN21	14	complement(88465778..88555007)	0,86	1,30	-0,374340684
ZC3H14	14	88562970..88627596	1,30	1,93	-0,347463372
EML5	14	complement(88612431..88792953)	0,01	0,01	0,278120973
TTC8	14	88824153..88881078	0,77	1,20	-0,417905481
FOXN3	14	complement(89124871..89619149)	2,09	3,82	-0,648757567
EFCAB11	14	complement(89794669..89954777)	0,28	0,40	-0,271233471
TDP1	14	89954939..90044764	3,07	3,06	0,228411104
KCNK13	14	90061994..90185853	0,02	0,11	-2,005885947
PSMC1	14	90256527..90275429	10,47	17,36	-0,507315366
NRDE2	14	complement(90267860..90331969)	1,45	1,49	0,175390915
CALM1	14	90396502..90408268	11,39	13,53	-0,025825511
TTC7B	14	complement(90524564..90816479)	0,99	1,51	-0,3867977
RPS6KA5	14	complement(90847861..91060641)	0,10	0,08	0,518906705
DGLUCY	14	91060333..91225632	2,91	3,91	-0,203336363
GPR68	14	complement(91232532..91253925)	0,77	0,96	-0,107533486
CCDC88C	14	complement(91271323..91417844)	0,38	0,45	0,007363969
PPP4R3A	14	complement(91457508..91510554)	3,87	5,08	-0,166595532
gene:ENSG000	14	complement(91572444..91575271)	0,02	0,00	1,833201638
CATSPERB	14	complement(91580696..91780707)	-	-	#N/D
TC2N	14	complement(91779746..91867536)	0,02	0,01	0,537167373
FBLN5	14	complement(91869411..91947987)	0,23	0,95	-1,817211588
TRIP11	14	complement(91965991..92040896)	1,70	2,45	-0,302223566
ATXN3	14	complement(92044496..92106621)	1,13	1,23	0,099275319
NDUFB1	14	complement(92116122..92121917)	4,31	4,96	0,019490631
CPSF2	14	92121969..92172145	2,74	4,28	-0,422840263
SLC24A4	14	92322581..92501481	0,01	0,00	0,533811434
RIN3	14	92513781..92688994	1,91	3,09	-0,470358712
gene:ENSG000	14	complement(92619986..92627966)	0,04	0,10	-1,211830422
LGMN	14	complement(92703807..92748679)	8,16	9,68	-0,023380014
GOLGA5	14	92794305..92839947	14,43	11,01	0,613101033
CHGA	14	92923150..92935285	0,03	0,03	0,278120973
ITPK1	14	complement(92936914..93116320)	2,71	5,32	-0,752793671
MOAP1	14	complement(93182199..93184923)	3,42	3,46	0,203892917
LYSET	14	93184951..93188463	1,19	1,44	-0,046052446
gene:ENSG000	14	93184973..93218586	0,03	0,09	-1,291882393
GON7	14	complement(93202894..93207065)	1,00	1,02	0,183829669
UBR7	14	93207241..93229215	6,95	6,50	0,319673555
BTBD7	14	complement(93237550..93333092)	2,64	2,74	0,170173106
UNC79	14	93333219..93707876	0,01	0,00	1,487770062
COX8C	14	93347182..93348356	-	0,02	-2,89252005
PRIMA1	14	complement(93718298..93788485)	0,04	0,01	1,833480694
gene:ENSG000	14	93895762..93897759	-	-	0,188027316
FAM181A	14	93918894..93929608	-	-	0,188027316

ASB2	14	complement(93934166..93976739)	0,01	0,02	-1,21700111
CCDC197	14	93987225..94008863	-	-	0,188027316
OTUB2	14	94026340..94048930	3,82	5,69	-0,352166472
DDX24	14	complement(94048287..94081202)	14,63	15,63	0,127303219
IFI27L1	14	94081301..94103846	5,47	3,65	0,806909531
IFI27	14	94104836..94116695	6,09	0,64	3,466264823
IFI27L2	14	complement(94127779..94130253)	12,72	12,99	0,1932281
PPP4R4	14	94146128..94279734	0,94	0,08	3,708497781
SERPINA10	14	complement(94280460..94293268)	0,18	0,00	6,271989619
SERPINA6	14	complement(94304248..94323389)	0,18	0,13	0,615472278
SERPINA2	14	complement(94364316..94366698)	0,01	-	3,068695355
SERPINA1	14	complement(94376747..94390693)	0,30	0,09	1,942515144
SERPINA11	14	complement(94442464..94452800)	-	-	0,188027316
SERPINA9	14	complement(94462717..94479689)	-	-	#N/D
SERPINA12	14	complement(94487274..94517844)	-	-	#N/D
SERPINA4	14	94561442..94569913	-	-	#N/D
SERPINA5	14	94561442..94593118	0,04	0,01	1,914812876
gene:ENSG000	14	94592058..94624646	-	0,00	-2,89252005
SERPINA3	14	94612384..94624055	0,02	0,10	-2,416176665
GSC	14	complement(94768223..94770113)	1,10	1,11	0,20236868
DICER1	14	complement(95086228..95158010)	2,44	4,76	-0,742991305
CLMN	14	complement(95181940..95319908)	0,73	1,82	-1,093093365
SYNE3	14	complement(95407266..95516650)	2,69	4,66	-0,569662924
GLRX5	14	95533503..95544724	5,79	5,06	0,417227455
TCL1B	14	95686426..95692628	0,01	0,01	-0,01185201
TCL1A	14	complement(95709947..95714196)	0,02	-	4,516772761
TUNAR	14	95866587..95925663	0,02	-	5,88622595
C14orf132	14	96039362..96093971	6,14	4,71	0,606280028
BDKRB2	14	96204679..96244166	3,82	5,35	-0,262401243
gene:ENSG000	14	96204844..96263929	1,74	3,15	-0,629783918
BDKRB1	14	96256210..96268967	16,09	31,24	-0,733795929
ATG2B	14	complement(96279195..96363341)	1,25	2,11	-0,532866959
GSKIP	14	96363452..96387288	0,96	1,23	-0,1450898
AK7	14	96392128..96489427	0,02	0,00	2,805678585
PAPOLA	14	96501433..96567116	8,91	15,01	-0,529601985
VRK1	14	96797304..96954846	0,61	0,99	-0,485855098
gene:ENSG000	14	96992864..96996601	0,02	0,02	0,627075281
gene:ENSG000	14	complement(98916902..98917801)	0,02	0,01	0,886720911
BCL11B	14	complement(99169287..99272197)	0,02	0,01	0,591315826
SETD3	14	complement(99397748..99480889)	7,44	6,70	0,37456622
CCNK	14	99481169..99535044	7,36	7,07	0,279603981
CCDC85C	14	complement(99500190..99604207)	2,45	3,07	-0,1023333
HHIPL1	14	99645129..99680569	0,11	0,11	0,254441097
CYP46A1	14	99684298..99727301	0,04	0,02	0,90516579
EML1	14	99737693..99942060	4,73	5,38	0,035319362
EVL	14	99971449..100144236	2,42	2,57	0,134419698
DEGS2	14	complement(100143957..10015964	0,12	0,12	0,142150013
YY1	14	100238298..100282788	13,13	10,65	0,525179828

SLC25A29	14	complement(100291116..10030654	4,90	2,52	1,182561893
SLC25A47	14	100323339..100330421	-	0,02	-4,359055975
WARS1	14	complement(100333790..10037680	7,84	28,09	-1,617152014
WDR25	14	100376418..100530303	0,87	1,07	-0,077991131
BEGAIN	14	complement(100537147..10058741	0,47	0,59	-0,121701444
DLK1	14	100725705..100738224	-	-	0,188027316
RTL1	14	complement(100879753..10090372	-	-	0,188027316
DIO3	14	101560139..101563452	0,08	0,19	-0,987207374
PPP2R5C	14	101761709..101927989	4,61	5,93	-0,138475464
DYNC1H1	14	101964573..102056443	19,86	27,23	-0,23277368
HSP90AA1	14	complement(102080742..10213969	112,11	#####	-0,494286546
WDR20	14	102139503..102224847	1,90	1,93	0,200593536
MOK	14	complement(102224500..10230519	0,82	0,70	0,45392789
ZNF839	14	102317377..102342702	1,36	3,00	-0,923623406
CINP	14	complement(102341102..10236291	2,81	2,98	0,13483865
TECPR2	14	102362941..102502477	1,31	2,11	-0,465946903
ANKRD9	14	complement(102501767..10250979	3,96	2,86	0,692776552
RCOR1	14	102592649..102730561	5,68	5,50	0,267757185
TRAF3	14	102777449..102911500	3,99	6,28	-0,428882436
AMN	14	102922663..102933596	0,06	0,08	-0,037246625
CDC42BPB	14	complement(102932380..10305754	12,90	15,75	-0,065706359
LBHD2	14	103084210..103090027	-	-	#N/D
EXOC3L4	14	103094725..103110559	0,02	0,07	-1,43066689
TNFAIP2	14	103121476..103137439	28,15	52,14	-0,666223224
EIF5	14	103333544..103345025	3,45	9,28	-1,203488997
gene:ENSG000	14	103334237..103335932	-	-	0,188027316
MARK3	14	103385377..103503831	4,97	5,30	0,131781307
CKB	14	complement(103519667..10352283	18,36	49,04	-1,194418364
TRMT61A	14	103529196..103537073	7,09	5,41	0,612131916
BAG5	14	complement(103556545..10356265	8,18	5,26	0,860293118
KLC1	14	103561896..103714249	2,98	3,80	-0,126321804
COA8	14	103562960..103607523	1,76	2,41	-0,229439827
gene:ENSG000	14	103562962..103685924	0,53	1,42	-1,18359764
XRCC3	14	complement(103697609..10371550	3,94	3,46	0,408713542
ZFYVE21	14	103715730..103733668	5,24	4,75	0,362693504
PPP1R13B	14	complement(103733195..10384757	0,82	1,06	-0,150263136
ATP5MJ	14	complement(103912288..10392826	5,74	6,99	-0,062771365
TDRD9	14	103928456..104052667	-	-	#N/D
RD3L	14	complement(103940426..10394256	-	-	#N/D
ASPG	14	104085686..104115582	0,00	0,00	-0,01185201
KIF26A	14	104136454..104180894	0,92	0,93	0,200121959
TMEM179	14	complement(104474678..10460564	-	-	0,188027316
C14orf180	14	104579764..104590515	-	-	0,188027316
INF2	14	104681146..104722535	9,10	8,43	0,333876524
ADSS1	14	104724174..104747325	1,71	1,52	0,392170435
SIVA1	14	104753147..104768494	10,49	10,37	0,240332006
AKT1	14	complement(104769349..10479575	31,51	30,39	0,274914786
ZBTB42	14	104800596..104804712	0,69	1,33	-0,729118672

gene:ENSG000	14	complement(104815449..10481575	0,74	0,55	0,64694678
CEP170B	14	104865268..104896747	13,28	17,35	-0,162631329
PLD4	14	104924713..104937761	0,05	0,02	1,422471951
AHNAK2	14	complement(104937244..10497837	5,24	6,24	-0,028689602
CLBA1	14	104985775..105010482	1,19	1,76	-0,343461545
CDCA4	14	complement(105009573..10502108	21,95	18,21	0,492325296
GPR132	14	complement(105049395..10506544	0,03	0,18	-2,323071803
gene:ENSG000	14	complement(105110347..10511080	0,09	0,08	0,366665713
JAG2	14	complement(105140982..10516882	1,11	1,02	0,342403023
NUDT14	14	complement(105172938..10518132	2,46	2,58	0,150931073
BRF1	14	complement(105209286..10531558	2,44	2,97	-0,060109819
BTBD6	14	105248533..105251093	4,83	6,13	-0,122989066
PACS2	14	105300563..105398147	3,52	5,21	-0,341294391
gene:ENSG000	14	105370418..105372918	0,75	1,17	-0,41767187
TEX22	14	105398538..105450106	0,21	0,30	-0,276807691
MTA1	14	105419820..105470729	20,13	32,26	-0,45760117
CRIP2	14	105472962..105480162	43,55	34,18	0,572435128
CRIP1	14	105486317..105488947	0,55	0,25	1,376708913
gene:ENSG000	14	105487199..105492267	0,27	0,17	0,829733423
TEDC1	14	105489855..105499575	1,88	3,12	-0,507096397
TMEM121	14	105526583..105530198	1,52	1,22	0,537192082
IGHA2	14	complement(105583731..10558839	-	-	#N/D
IGHE	14	complement(105597691..10560172	-	-	0,188027316
IGHG4	14	complement(105620506..10562606	0,01	0,00	2,616997665
IGHG2	14	complement(105639559..10564479	0,00	-	3,967268276
IGHA1	14	complement(105703995..10570866	-	-	0,188027316
IGHG1	14	complement(105736343..10574307	0,00	0,00	-0,01185201
IGHG3	14	complement(105764503..10577140	-	-	#N/D
IGHD	14	complement(105836765..10584567	-	-	0,188027316
IGHM	14	complement(105851705..10585621	-	-	#N/D
IGHJ6	14	complement(105863198..10586325	-	-	#N/D
IGHJ5	14	complement(105863814..10586386	-	-	#N/D
IGHJ4	14	complement(105864215..10586426	-	-	#N/D
IGHJ3	14	complement(105864587..10586463	-	-	#N/D
IGHJ2	14	complement(105865199..10586525	-	-	#N/D
IGHJ1	14	complement(105865407..10586545	-	-	#N/D
IGHD7-27	14	complement(105865551..10586556	-	-	#N/D
IGHD1-26	14	complement(105881034..10588105	-	-	#N/D
IGHD6-25	14	complement(105881539..10588155	-	-	#N/D
IGHD5-24	14	complement(105883903..10588392	-	-	#N/D
IGHD4-23	14	complement(105884870..10588488	-	-	#N/D
IGHD3-22	14	complement(105886031..10588606	-	-	#N/D
IGHD2-21	14	complement(105888551..10588857	-	-	#N/D
IGHD1-20	14	complement(105891191..10589120	-	-	#N/D
IGHD6-19	14	complement(105891699..10589171	-	-	#N/D
IGHD5-18	14	complement(105893542..10589356	-	-	#N/D
IGHD4-17	14	complement(105894508..10589452	-	-	#N/D
IGHD3-16	14	complement(105895634..10589567	-	-	#N/D

IGHD2-15	14	complement(105897957..10589798	-	-	#N/D
IGHD1-14	14	complement(105900638..10590065	-	-	#N/D
IGHD6-13	14	complement(105901142..10590116	-	-	#N/D
IGHD5-12	14	complement(105902649..10590267	-	-	#N/D
IGHD4-11	14	complement(105903616..10590363	-	-	#N/D
IGHD3-10	14	complement(105904497..10590452	-	-	#N/D
IGHD3-9	14	complement(105904681..10590471	-	-	#N/D
IGHD2-8	14	complement(105907211..10590724	-	-	#N/D
IGHD1-7	14	complement(105909907..10590992	-	-	#N/D
IGHD6-6	14	complement(105910410..10591042	-	-	#N/D
IGHD5-5	14	complement(105912257..10591227	-	-	#N/D
IGHD4-4	14	complement(105913222..10591323	-	-	#N/D
IGHD3-3	14	complement(105914359..10591438	-	-	#N/D
IGHD2-2	14	complement(105916826..10591685	-	-	#N/D
IGHD1-1	14	complement(105919502..10591951	-	-	#N/D
IGHV6-1	14	complement(105939756..10594025	-	-	#N/D
IGHV1-2	14	complement(105986582..10598708	-	0,05	-3,804623889
IGHV1-3	14	complement(106005095..10600557	-	-	#N/D
IGHV4-4	14	complement(106011922..10601242	-	-	#N/D
IGHV7-4-1	14	complement(106025145..10602563	-	-	#N/D
IGHV2-5	14	complement(106037902..10603836	-	-	#N/D
IGHV3-7	14	complement(106062151..10606268	-	-	#N/D
IGHV3-64D	14	complement(106088122..10608857	-	-	#N/D
IGHV5-10-1	14	complement(106107972..10610846	-	-	#N/D
IGHV3-11	14	complement(106116635..10611720	-	-	#N/D
IGHV3-13	14	complement(106129540..10613007	-	-	#N/D
IGHV3-15	14	complement(106153624..10615416	-	-	#N/D
IGHV3-16	14	complement(106165205..10616573	-	-	#N/D
IGHV1-18	14	complement(106184901..10618539	-	-	#N/D
IGHV3-20	14	complement(106210936..10621145	-	-	#N/D
IGHV3-21	14	complement(106235064..10623559	-	-	#N/D
IGHV3-23	14	complement(106268606..10626914	-	-	#N/D
IGHV1-24	14	complement(106276548..10627704	-	-	0,188027316
IGHV2-26	14	complement(106301396..10630186	-	-	#N/D
IGHV4-28	14	complement(106324254..10632476	-	-	#N/D
IGHV3-30	14	complement(106335082..10633561	-	-	#N/D
IGHV4-31	14	complement(106349283..10634979	-	-	#N/D
IGHV3-33	14	complement(106359793..10636032	-	-	#N/D
IGHV4-34	14	complement(106373663..10637414	-	-	#N/D
IGHV3-35	14	complement(106389392..10638985	-	-	#N/D
IGHV3-38	14	complement(106410493..10641102	-	-	#N/D
IGHV4-39	14	complement(106421711..10642221	0,04	-	3,967268276
IGHV3-43	14	complement(106470264..10647080	-	-	#N/D
IGHV1-45	14	complement(106506996..10650749	-	-	#N/D
IGHV1-46	14	complement(106511117..10651185	-	-	#N/D
IGHV3-48	14	complement(106537810..10653834	-	-	#N/D
IGHV3-49	14	complement(106556936..10655747	-	-	#N/D
IGHV5-51	14	complement(106578744..10657923	-	-	#N/D

IGHV8-51-1	14	complement(106583501..10658380	-	-	#N/D
IGHV3-53	14	complement(106592676..10659334	-	-	#N/D
IGHV1-58	14	complement(106622357..10662285	-	-	#N/D
IGHV4-59	14	complement(106627249..10662782	-	-	#N/D
IGHV4-61	14	complement(106639119..10663965	-	-	#N/D
IGHV3-64	14	complement(106657725..10665825	-	-	#N/D
IGHV3-66	14	complement(106675017..10667554	-	-	#N/D
IGHV1-69	14	complement(106714684..10671518	-	-	#N/D
IGHV2-70D	14	complement(106723574..10672409	-	-	#N/D
IGHV1-69-2	14	complement(106737110..10673754	-	-	#N/D
IGHV1-69D	14	complement(106762092..10676258	-	-	#N/D
IGHV2-70	14	complement(106770577..10677102	-	-	#N/D
IGHV3-72	14	complement(106790694..10679123	-	-	0,188027316
IGHV3-73	14	complement(106802694..10680323	-	-	#N/D
IGHV3-74	14	complement(106810442..10681113	-	-	0,188027316
IGHV7-81	14	complement(106874583..10687507	-	-	#N/D
IGHV1OR15-9	15	complement(19964666..19965101)	-	-	#N/D
IGHV3OR15-7	15	complement(19987656..19988117)	-	-	#N/D
IGHD5OR15-5A	15	complement(20003840..20003862)	-	-	#N/D
IGHD4OR15-4A	15	complement(20004797..20004815)	-	-	#N/D
IGHD3OR15-3A	15	complement(20005905..20005935)	-	-	#N/D
IGHD2OR15-2A	15	complement(20008402..20008432)	-	-	#N/D
IGHD1OR15-1A	15	complement(20011153..20011169)	-	-	#N/D
gene:ENSG000	15	20141794..20146430	-	-	0,188027316
GOLGA6L6	15	complement(20531856..20541800)	-	-	0,188027316
gene:ENSG000	15	20658900..20664970	-	-	0,188027316
POTEB2	15	complement(20835372..20866314)	-	-	0,188027316
IGHD5OR15-5B	15	complement(21010494..21010516)	-	-	#N/D
IGHD4OR15-4B	15	complement(21011451..21011469)	-	-	#N/D
IGHD3OR15-3B	15	complement(21012559..21012589)	-	-	#N/D
IGHD2OR15-2B	15	complement(21015048..21015078)	-	-	#N/D
IGHD1OR15-1B	15	complement(21017800..21017816)	-	-	#N/D
gene:ENSG000	15	21148334..21152970	0,09	0,03	1,983427248
POTEB3	15	complement(21405401..21440499)	-	-	0,188027316
LINC02203	15	21552795..21653276	-	-	#N/D
OR4M2B	15	21637909..21638992	-	-	#N/D
OR4N4C	15	21651844..21652968	-	-	#N/D
gene:ENSG000	15	complement(21717808..21718245)	-	-	#N/D
gene:ENSG000	15	complement(21742364..21742799)	-	-	#N/D
POTEB	15	complement(21846329..21877735)	-	-	#N/D
OR4M2-OT1	15	22015233..22095857	-	-	#N/D
OR4M2	15	22070241..22083221	-	-	#N/D
OR4N4	15	22094522..22095862	-	-	#N/D
IGHV1OR15-1	15	complement(22160431..22160868)	-	-	#N/D
IGHV4OR15-8	15	complement(22184967..22185402)	-	-	#N/D
GOLGA6L22	15	22458903..22469226	-	-	#N/D
NIPA1	15	22773063..22829789	2,57	2,63	0,193196781
NIPA2	15	22838644..22869362	4,05	5,05	-0,093877428

CYFIP1	15	complement(22867052..22981063)	11,14	12,89	0,012652427
TUBGCP5	15	complement(22983192..23039572)	2,03	2,80	-0,244090429
GOLGA6L1	15	complement(23127066..23136822)	-	-	#N/D
GOLGA6L26	15	complement(23324535..23334196)	0,00	-	3,068695355
GOLGA8S	15	23354748..23368341	0,01	0,07	-2,114828667
GOLGA6L2	15	complement(23439038..23447243)	-	-	#N/D
MKRN3	15	23565674..23630075	0,47	0,51	0,104518381
MAGEL2	15	complement(23643549..23647867)	-	0,00	-2,89252005
NDN	15	complement(23685400..23687305)	-	-	#N/D
NPAP1	15	24675775..24683393	-	-	0,188027316
SNRPN	15	24823637..24978723	-	-	#N/D
SNURF	15	24954987..24977850	-	-	#N/D
gene:ENSG000	15	24955034..25000276	-	-	#N/D
PWAR5	15	24985053..24988232	-	-	#N/D
gene:ENSG000	15	complement(25091960..25092377)	-	-	#N/D
PWAR1	15	25135642..25138055	-	-	#N/D
gene:ENSG000	15	25255579..25257027	0,01	0,02	-0,923955849
UBE3A	15	complement(25333728..25439051)	1,89	3,01	-0,450915456
ATP10A	15	complement(25677273..25865184)	0,28	0,11	1,596937387
GABRB3	15	complement(26543552..26939539)	0,18	0,46	-1,112940685
GABRA5	15	26866911..26949208	-	-	#N/D
GABRG3	15	26971181..27541984	0,00	-	3,967268276
OCA2	15	complement(27754875..28099315)	0,00	0,01	-0,923955849
HERC2	15	complement(28111040..28322179)	7,44	9,11	-0,069119974
GOLGA6L24	15	complement(28347851..28358084)	-	-	#N/D
GOLGA8F	15	28378621..28392021	0,13	0,22	-0,494699922
GOLGA8G	15	complement(28519611..28533014)	0,27	0,29	0,12911403
GOLGA6L25	15	28553568..28563779	-	-	0,188027316
GOLGA8M	15	complement(28698583..28738384)	0,02	0,02	-0,037246625
GOLGA6L7	15	complement(28841832..28848675)	0,13	0,14	0,12297266
APBA2	15	28884483..29118315	3,68	6,61	-0,621739469
ENTREP2	15	complement(29120252..29570979)	0,28	0,16	0,990289098
NSMCE3	15	complement(29264989..29269822)	1,00	2,10	-0,847978687
TJP1	15	complement(29699367..29968915)	5,33	11,96	-0,943628385
GOLGA8J	15	30082955..30096690	0,00	0,01	-1,877914477
GOLGA8T	15	30135051..30148748	0,04	0,14	-1,492537881
gene:ENSG000	15	complement(30344307..30348236)	0,04	0,02	1,613114315
CHRFAM7A	15	complement(30360566..30393849)	0,50	0,79	-0,434707906
GOLGA8R	15	complement(30400562..30414260)	0,38	0,43	0,07338217
GOLGA8Q	15	30552050..30565684	-	-	0,188027316
GOLGA8H	15	30604028..30617752	0,26	0,29	0,056302299
ARHGAP11B	15	30624494..30649529	0,87	0,31	1,694536122
gene:ENSG000	15	30624548..30773002	1,20	2,55	-0,86671655
FAN1	15	30890559..30943108	1,83	2,70	-0,332083717
MTMR10	15	complement(30938941..30991628)	0,71	1,17	-0,495343082
TRPM1	15	complement(31001065..31161160)	-	-	0,188027316
KLF13	15	31326835..31435665	11,76	8,72	0,654096267
OTUD7A	15	complement(31475398..31870789)	0,01	0,01	-1,121232669

CHRNA7	15	31923438..32173018	0,02	0,03	0,038295137
gene:ENSG000	15	32182551..32186475	0,00	0,01	-1,877914477
GOLGA8K	15	complement(32389599..32403292)	-	0,03	-6,052163584
GOLGA8O	15	complement(32441914..32455634)	0,35	0,51	-0,326960642
GOLGA8N	15	32593456..32607310	0,12	0,13	0,037513468
ARHGAP11A-S(	15	32613860..32697095	0,36	1,56	-1,897080618
ARHGAP11A	15	32615144..32639941	4,90	7,24	-0,340069744
SCG5	15	32641676..32697098	0,02	0,19	-3,263348475
GREM1	15	32718004..32745106	0,41	6,90	-3,845117294
FMN1	15	complement(32765544..33194714)	0,40	1,27	-1,426836532
gene:ENSG000	15	complement(33105758..33106236)	-	0,03	-2,89252005
RYR3	15	33310962..33866121	0,00	0,01	-1,806405564
AVEN	15	complement(33858782..34075155)	1,39	2,43	-0,57672968
CHRM5	15	33968497..34067458	0,00	0,02	-3,171495545
EMC7	15	complement(34084017..34101862)	14,17	12,54	0,400030875
PGBD4	15	34102083..34108686	0,37	0,40	0,099224618
KATNBL1	15	complement(34140674..34210096)	1,46	2,04	-0,253215438
EMC4	15	34225013..34230156	13,13	14,14	0,115592792
SLC12A6	15	complement(34229784..34338060)	1,07	2,49	-0,990594583
NOP10	15	complement(34339159..34343180)	16,95	18,75	0,078001162
NUTM1	15	34343315..34357737	0,06	0,08	-0,154366259
LPCAT4	15	complement(34358633..34367196)	15,49	18,22	-0,010874925
GOLGA8A	15	complement(34378935..34437808)	4,28	5,50	-0,139003238
gene:ENSG000	15	complement(34415450..34420273)	0,20	0,26	-0,164510337
GOLGA8B	15	complement(34525095..34588503)	3,67	5,93	-0,469149403
GJD2	15	complement(34751032..34754998)	-	-	0,188027316
ACTC1	15	complement(34790107..34795589)	0,29	0,04	3,101353686
AQR	15	complement(34851782..34969742)	2,34	2,96	-0,115118598
ZNF770	15	complement(34978341..34988287)	3,00	4,08	-0,21721122
gene:ENSG000	15	34978351..34980084	-	-	#N/D
gene:ENSG000	15	34999902..35000270	-	-	0,188027316
NANOGP8	15	complement(35083410..35085295)	0,01	-	3,068695355
DPH6	15	complement(35217345..35546193)	0,19	0,45	-1,024434481
CDIN1	15	36579626..36810248	0,66	1,68	-1,125072189
MEIS2	15	complement(36889204..37101299)	0,17	0,62	-1,669699863
TMCO5A	15	37921939..37967724	-	-	0,188027316
SPRED1	15	38252836..38357249	3,49	3,16	0,365248982
FAM98B	15	38454127..38487710	1,32	1,79	-0,212126438
RASGRP1	15	complement(38488103..38565575)	0,07	0,14	-0,813297795
THBS1	15	39581079..39599466	6,46	16,02	-1,087453046
FSIP1	15	complement(39588357..39782841)	0,06	0,20	-1,428793978
GPR176	15	complement(39799008..39920936)	7,90	10,89	-0,240569322
EIF2AK4	15	39934115..40035591	6,11	9,12	-0,35593873
SRP14	15	complement(40035690..40039181)	7,77	11,41	-0,331045491
BMF	15	complement(40087890..40108892)	0,61	0,61	0,215306296
BUB1B	15	40161023..40221123	11,02	17,84	-0,472457382
PAK6	15	40217428..40277487	0,07	0,29	-1,838564768
BUB1B-PAK6	15	40218500..40264890	-	-	0,188027316

gene:ENSG000	15	40244925..40247366	0,00	0,07	-3,545596224
gene:ENSG000	15	40258501..40260370	0,01	0,01	0,886720911
PLCB2	15	complement(40278176..40307935)	0,02	-	6,575938476
ANKRD63	15	complement(40278372..40283064)	0,00	-	3,068695355
INAFM2	15	40323692..40326715	6,13	3,65	0,971666125
CCDC9B	15	complement(40331452..40340939)	6,53	11,71	-0,619089473
PHGR1	15	40351033..40356434	0,05	-	3,967268276
DISP2	15	40358219..40378621	0,21	0,09	1,421786909
KNSTRN	15	40382721..40394288	7,35	12,55	-0,548236375
IVD	15	40405485..40435947	2,78	3,67	-0,179634323
BAHD1	15	40439721..40468236	5,74	6,42	0,061938011
CHST14	15	40470984..40473158	11,85	20,79	-0,587282968
CCDC32	15	complement(40528683..40565054)	0,58	0,60	0,170961564
RPUSD2	15	40569299..40574949	2,78	3,28	-0,013615429
KNL1	15	40594020..40664342	1,72	3,00	-0,579086899
RAD51	15	40694774..40732340	2,66	3,64	-0,232503166
RMDN3	15	complement(40735884..40755851)	4,03	4,15	0,182674081
GCHFR	15	40764068..40767708	1,57	2,50	-0,445493888
DNAJC17	15	complement(40765161..40807478)	2,07	1,88	0,363522571
C15orf62	15	40769980..40772449	1,97	1,75	0,395514153
ZFYVE19	15	40807086..40815084	4,15	5,07	-0,065615668
PPP1R14D	15	complement(40815451..40828709)	0,09	-	5,697545031
SPINT1	15	40844018..40858207	0,04	0,02	1,629328643
RHOV	15	complement(40872214..40874234)	0,34	1,09	-1,456422707
VPS18	15	40894450..40903975	5,52	4,19	0,620179122
DLL4	15	40929340..40939073	0,05	0,03	1,046316654
CHAC1	15	40952962..40956512	3,23	4,53	-0,264518022
INO80	15	complement(40978880..41116280)	4,00	7,11	-0,606083671
EXD1	15	complement(41182725..41230757)	0,01	0,01	-0,03031053
CHP1	15	41230839..41281887	12,64	16,62	-0,170984735
gene:ENSG000	15	41284007..41380996	0,83	1,11	-0,205036783
OIP5	15	complement(41309273..41332591)	2,62	4,42	-0,530234102
NUSAP1	15	41320794..41381050	20,00	24,84	-0,089244697
NDUFAF1	15	complement(41387218..41409403)	2,10	2,68	-0,125766172
RTF1	15	41408408..41483563	6,81	8,21	-0,045794003
ITPKA	15	41493393..41503551	0,58	1,26	-0,886193771
LTK	15	complement(41503637..41513887)	0,15	0,17	0,028381884
RPAP1	15	complement(41517176..41544269)	7,69	9,65	-0,104835012
TYRO3	15	41557675..41583589	3,82	5,37	-0,267609582
MGA	15	41621134..41773081	1,60	2,66	-0,512550324
MAPKBP1	15	41774434..41827855	4,54	3,41	0,637877676
JMJD7	15	41828092..41837581	2,24	2,24	0,221264041
JMJD7-PLA2G4	15	41828095..41848155	1,18	0,95	0,540590516
PLA2G4B	15	41837775..41848147	0,91	1,02	0,059543911
SPTBN5	15	complement(41848146..41894053)	0,03	0,09	-1,219934563
EHD4	15	complement(41895933..41972557)	7,06	9,61	-0,221806261
PLA2G4E	15	complement(41981394..42051190)	0,00	0,01	-0,579815015
PLA2G4D	15	complement(42067009..42094562)	-	-	0,188027316

PLA2G4F	15	complement(42139034..42156636)	-	-	0,188027316
VPS39	15	complement(42158701..42208307)	9,67	9,32	0,277092746
TMEM87A	15	complement(42210447..42273534)	3,20	3,76	-0,007897585
GANC	15	42273201..42353666	0,99	1,69	-0,541875268
gene:ENSG000	15	42348103..42412317	0,02	0,05	-0,877094735
CAPN3	15	42359498..42412949	0,03	0,11	-1,414336283
ZNF106	15	complement(42412823..42491141)	4,08	5,76	-0,274874332
SNAP23	15	42491233..42545356	3,08	3,84	-0,097966477
gene:ENSG000	15	complement(42497395..42548786)	0,31	0,23	0,624282904
LRRC57	15	complement(42537820..42548802)	1,07	1,57	-0,331016799
HAUS2	15	42548828..42569994	2,39	3,18	-0,189958197
STARD9	15	42575606..42720998	0,52	0,39	0,639342138
CDAN1	15	complement(42723544..42737128)	2,51	3,52	-0,263408447
TTBK2	15	complement(42738730..42920809)	2,24	2,42	0,109974361
UBR1	15	complement(42942897..43106113)	2,66	2,76	0,173116606
TMEM62	15	43123279..43185144	1,01	3,87	-1,708288538
gene:ENSG000	15	complement(43179715..43206481)	-	-	0,188027316
CCNDBP1	15	43185118..43197177	4,22	4,68	0,07249248
EPB42	15	complement(43197227..43221018)	0,02	0,01	1,487770062
TGM5	15	complement(43232590..43266928)	-	-	0,188027316
TGM7	15	complement(43276271..43302255)	-	-	0,188027316
LCMT2	15	complement(43323649..43330582)	0,43	1,24	-1,29862811
ADAL	15	43330672..43354569	1,54	1,70	0,079186558
ZSCAN29	15	complement(43358172..43371043)	2,22	4,24	-0,709366684
TUBGCP4	15	43369221..43409771	4,17	6,71	-0,461885092
TP53BP1	15	complement(43403061..43510728)	9,26	10,71	0,013474549
MAP1A	15	43510958..43531620	0,46	1,03	-0,935644463
PPIP5K1	15	complement(43533462..43590208)	3,58	4,17	0,00102831
CKMT1B	15	43593054..43604901	-	-	0,188027316
STRC	15	complement(43599563..43618800)	0,34	0,12	1,69888603
gene:ENSG000	15	complement(43614208..43632283)	0,10	0,15	-0,377031134
CATSPER2	15	complement(43628503..43668118)	0,19	0,20	0,187387264
CKMT1A	15	43692886..43699222	-	-	0,188027316
PDIA3	15	43746394..43773279	46,21	48,71	0,146870447
ELL3	15	complement(43772605..43777315)	0,35	0,28	0,543075303
gene:ENSG000	15	complement(43772617..43799133)	-	-	0,188027316
SERF2	15	43777087..43804427	33,55	23,30	0,749133427
SERINC4	15	complement(43794162..43800220)	0,04	-	6,338059249
HYPK	15	43796142..43804427	8,58	2,60	1,946351964
MFAP1	15	complement(43804492..43824690)	7,80	8,38	0,120048201
WDR76	15	43826980..43868412	6,77	8,31	-0,071133485
FRMD5	15	complement(43870761..44195271)	0,21	0,98	-1,987390774
GOLM2	15	44288719..44415758	12,87	17,75	-0,24082231
CTDSPL2	15	44427622..44529038	2,67	3,39	-0,122951752
EIF3J	15	44537125..44563029	3,13	8,14	-1,156193038
SPG11	15	complement(44554818..44663688)	4,03	6,17	-0,391979354
PATL2	15	complement(44665732..44711323)	0,06	0,25	-1,750952677
B2M	15	44711358..44718851	170,52	#####	-0,57113281

TRIM69	15	44728988..44767829	2,00	3,18	-0,443631411
TERB2	15	44956687..44979229	0,01	0,01	0,886720911
SORD	15	45023147..45077185	6,83	9,20	-0,2051861
DUOX2	15	complement(45092650..45114172)	0,02	0,03	-0,27623233
DUOXA2	15	45114326..45118421	0,03	0,02	0,750935198
DUOXA1	15	complement(45117366..45129938)	0,09	0,05	1,003788922
DUOX1	15	45129933..45165576	0,14	0,25	-0,606738764
SHF	15	complement(45167214..45201175)	0,46	0,47	0,19485683
gene:ENSG000	15	complement(45214448..45215044)	-	-	#N/D
SLC28A2	15	45252234..45277846	0,01	0,01	-0,742377908
GATM	15	complement(45361124..45402327)	-	-	0,188027316
AFG2B	15	45402336..45421415	2,34	3,01	-0,139865456
C15orf48	15	45430579..45448761	0,26	3,42	-3,482851436
SLC30A4	15	complement(45479606..45522755)	1,22	1,13	0,335807246
BLOC1S6	15	45587214..45615945	5,38	6,17	0,023767689
gene:ENSG000	15	45587351..45676314	-	-	0,188027316
SQOR	15	45631148..45691281	27,39	20,94	0,610162954
gene:ENSG000	15	complement(47133495..47134122)	0,10	0,29	-1,239733301
SEMA6D	15	47184101..47774228	8,13	4,16	1,190366579
SLC24A5	15	48120990..48142672	-	-	#N/D
MYEF2	15	complement(48134632..48178353)	0,30	0,67	-0,915776923
CTXN2	15	48178122..48203758	0,00	0,00	-0,01185201
SLC12A1	15	48178438..48304078	-	-	0,188027316
DUT	15	48331011..48343373	10,66	12,81	-0,041298512
FBN1	15	complement(48408313..48645721)	65,73	29,08	1,399671104
CEP152	15	complement(48712928..48811146)	4,71	4,41	0,315622554
SHC4	15	complement(48823741..48963919)	0,19	0,25	-0,223240599
EID1	15	48878134..48880173	13,52	12,12	0,379983957
SECISBP2L	15	complement(48988476..49046447)	4,27	4,81	0,0519978
COPS2	15	complement(49106068..49155661)	2,84	4,58	-0,467700413
GALK2	15	49155656..49367869	2,91	2,43	0,488061581
FAM227B	15	complement(49326962..49620929)	0,24	0,16	0,808766485
FGF7	15	49423237..49488775	0,00	0,01	-0,579815015
DTWD1	15	49621037..49656232	0,43	0,59	-0,232515348
ATP8B4	15	complement(49858238..50182817)	0,01	0,01	-0,602143793
SLC27A2	15	50182196..50236385	-	0,02	-4,758582517
HDC	15	complement(50241947..50265965)	-	-	#N/D
GABPB1	15	complement(50275389..50355408)	1,42	3,12	-0,914291961
USP8	15	50424380..50514421	1,25	1,71	-0,226446689
USP50	15	complement(50494018..50546708)	-	0,02	-4,359055975
TRPM7	15	complement(50552473..50686797)	1,62	2,69	-0,509392819
gene:ENSG000	15	complement(50648802..50686717)	1,44	1,36	0,30164755
SPPL2A	15	complement(50702266..50765948)	1,97	4,62	-1,00654553
gene:ENSG000	15	complement(50742702..50743276)	0,19	0,18	0,279559702
AP4E1	15	50908672..51005895	1,42	2,62	-0,66673049
TNFAIP8L3	15	complement(51056598..51105276)	0,66	1,27	-0,719426162
CYP19A1	15	complement(51208057..51338601)	0,10	0,05	1,177515149
GLDN	15	51341655..51408005	0,07	0,86	-3,475909209

DMXL2	15	complement(51447711..51622833)	1,70	2,75	-0,472402968
SCG3	15	51681492..51721026	0,01	0,03	-1,955757882
LYSMD2	15	complement(51723011..51751585)	0,89	2,18	-1,067689606
TMOD2	15	51751597..51816363	0,12	0,34	-1,308453535
TMOD3	15	51829628..51947295	11,06	11,41	0,177549658
LEO1	15	complement(51938025..51971778)	2,58	3,41	-0,178541169
MAPK6	15	51952106..52067375	8,73	9,18	0,149168259
gene:ENSG000	15	52045846..52045899	-	-	#N/D
BCL2L10	15	complement(52109263..52112775)	0,01	0,01	-0,01185201
GNB5	15	complement(52115100..52191392)	2,57	3,13	-0,061313914
MYO5C	15	complement(52192322..52295804)	0,04	0,04	0,016676551
MYO5A	15	complement(52307281..52529132)	2,53	5,17	-0,810238649
ARPP19	15	complement(52547045..52569883)	11,83	15,38	-0,155876048
ATOSA	15	complement(52581317..52709817)	0,72	1,06	-0,323623409
ONECUT1	15	complement(52755053..52791078)	0,00	0,01	-1,291882393
WDR72	15	complement(53513741..53762878)	-	-	0,188027316
gene:ENSG000	15	53910769..53914712	-	-	#N/D
UNC13C	15	53978201..54628707	0,00	-	4,516772761
RSL24D1	15	complement(55180806..55197049)	5,46	10,45	-0,713254994
RAB27A	15	complement(55202966..55319113)	1,32	2,54	-0,717431229
PIGBOS1	15	complement(55317184..55319161)	1,87	3,01	-0,463897356
PIGB	15	55318960..55355648	1,47	2,15	-0,322191535
CCPG1	15	complement(55340032..55408510)	1,70	5,07	-1,348725445
PIERCE2	15	55408495..55418798	0,09	0,26	-1,270895709
DNAAF4	15	complement(55410525..55508234)	0,03	0,13	-1,660236228
PYGO1	15	complement(55538884..55588947)	1,26	2,76	-0,911484808
PRTG	15	complement(55611544..55743152)	0,32	1,16	-1,612872493
NEDD4	15	complement(55826922..55993660)	2,85	4,19	-0,333371475
RFX7	15	complement(56087280..56245082)	2,09	2,59	-0,086022855
TEX9	15	56243971..56445997	0,04	0,14	-1,806415609
MNS1	15	complement(56421544..56465137)	0,12	0,24	-0,824193273
ZNF280D	15	complement(56630176..56734086)	0,76	1,13	-0,344146121
gene:ENSG000	15	complement(56652488..56918571)	0,31	0,81	-1,158455753
gene:ENSG000	15	56795382..56795522	-	-	#N/D
TCF12	15	56918623..57299281	11,64	16,26	-0,258987349
CGNL1	15	57375967..57550717	0,08	0,43	-2,137182362
MYZAP	15	57591904..57685364	1,86	2,43	-0,158676515
GCOM1	15	57591908..57714745	0,90	2,03	-0,940890383
POLR2M	15	57706695..57782762	2,74	4,37	-0,451224933
ALDH1A2	15	complement(57953424..58497866)	0,04	0,06	-0,533146901
AQP9	15	58138169..58185911	0,00	0,02	-2,190455314
LIPC	15	58410569..58569844	0,00	0,01	-0,923955849
ADAM10	15	complement(58588809..58749791)	6,54	9,01	-0,239189517
MINDY2	15	58771192..58861900	1,26	1,25	0,234883716
RNF111	15	58865175..59097419	5,56	6,02	0,106412665
SLTM	15	complement(58879050..58933679)	2,78	5,87	-0,853455822
CCNB2	15	59105126..59125045	25,97	36,63	-0,273102055
MYO1E	15	complement(59132434..59372871)	4,01	7,06	-0,593820045

LDHAL6B	15	59206843..59208588	0,10	0,14	-0,284856676
FAM81A	15	59372693..59523555	0,01	0,14	-3,037280827
GCNT3	15	59594875..59640239	0,05	0,78	-3,698659805
GTF2A2	15	complement(59638062..59657541)	5,15	7,54	-0,327790939
BNIP2	15	complement(59659146..59689339)	4,56	5,50	-0,045627526
FOXB1	15	60004234..60061730	0,02	0,44	-4,035311322
ANXA2	15	complement(60347134..60402883)	348,35	#####	0,484178649
ICE2	15	complement(60419609..60479160)	1,81	3,90	-0,884730017
RORA	15	complement(60488284..61229302)	0,57	0,46	0,546186305
VPS13C	15	complement(61852389..62060473)	2,78	4,52	-0,478798189
C2CD4A	15	62066977..62070917	0,01	0,08	-2,850473978
C2CD4B	15	complement(62163535..62165285)	-	-	0,188027316
gene:ENSG000	15	complement(62257952..62261415)	-	-	#N/D
TLN2	15	62390526..62844631	2,73	4,86	-0,609609671
TPM1	15	63042620..63071915	10,59	14,50	-0,231028264
LACTB	15	63121833..63142061	2,84	5,42	-0,710013053
RPS27L	15	complement(63125872..63158021)	1,85	2,56	-0,240750229
RAB8B	15	63189560..63267776	2,10	3,96	-0,689556737
APH1B	15	63276018..63309126	1,10	2,25	-0,805990653
CA12	15	complement(63321378..63381846)	27,06	43,42	-0,459045941
USP3	15	63504511..63594640	4,75	6,69	-0,270272247
FBXL22	15	63597353..63602428	0,16	0,32	-0,733312865
HERC1	15	complement(63608618..63833948)	6,82	6,28	0,342143868
DAPK2	15	complement(63907036..64072033)	0,35	1,26	-1,609536619
CIAO2A	15	complement(64072565..64094262)	4,59	6,37	-0,248757287
SNX1	15	64094123..64146090	8,82	9,02	0,190675927
SNX22	15	64151715..64157481	2,46	1,92	0,58265546
PPIB	15	complement(64155740..64163134)	175,52	#####	0,45099707
CSNK1G1	15	complement(64165525..64356173)	2,81	3,39	-0,050124569
gene:ENSG000	15	complement(64181180..64381440)	3,17	3,43	0,108605046
PCLAF	15	complement(64364304..64387687)	9,91	8,13	0,508497328
TRIP4	15	64387748..64455303	3,05	3,95	-0,150490209
ZNF609	15	64460578..64686068	8,22	11,42	-0,25099165
OAZ2	15	complement(64687573..64703281)	26,98	32,54	-0,047375525
RBPMS2	15	complement(64739891..64775589)	3,08	7,49	-1,05855704
PIF1	15	complement(64815632..64825668)	4,89	6,84	-0,261213937
PLEKHO2	15	64841883..64868002	2,03	4,61	-0,964420983
gene:ENSG000	15	64841883..64930920	0,06	-	4,913749004
ANKDD1A	15	64911902..64958691	0,35	0,43	-0,061090315
SPG21	15	complement(64963022..64990310)	13,69	20,60	-0,366949158
MTFMT	15	complement(65001512..65029639)	1,33	2,67	-0,778968981
SLC51B	15	65045387..65053397	0,04	0,08	-0,999120035
RASL12	15	complement(65053337..65076690)	0,64	0,87	-0,228595516
KBTBD13	15	65076746..65079948	-	0,00	-2,89252005
UBAP1L	15	complement(65092760..65115200)	0,25	0,56	-0,931058512
PDCD7	15	complement(65117379..65133808)	9,55	11,69	-0,067728447
CLPX	15	complement(65148219..65185342)	5,68	9,65	-0,5418285
CILP	15	complement(65194760..65211473)	0,01	0,00	2,144183439

gene:ENSG000	15	complement(65226472..65226697)	-	-	0,188027316
PARP16	15	complement(65234460..65300618)	2,79	3,56	-0,129922993
IGDCC3	15	complement(65327127..65378002)	-	0,01	-3,804623889
IGDCC4	15	complement(65381484..65422947)	0,01	0,06	-2,587642893
DPP8	15	complement(65442463..65517704)	3,18	3,27	0,183314232
HACD3	15	65530418..65578349	19,69	27,53	-0,260816101
INTS14	15	complement(65578753..65611289)	8,92	14,71	-0,498128676
SLC24A1	15	65611366..65660995	0,54	1,20	-0,916946265
DENND4A	15	complement(65658046..65792293)	1,46	3,41	-0,999417558
RAB11A	15	65726054..65891989	11,67	11,61	0,230270869
MEGF11	15	complement(65895079..66253756)	0,15	0,17	-0,015534853
DIS3L	15	66293217..66333898	2,15	5,98	-1,254287726
TIPIN	15	complement(66336191..66386746)	1,10	2,22	-0,78686172
MAP2K1	15	66386837..66491656	11,23	12,53	0,064528102
SNAPC5	15	complement(66490135..66497780)	1,79	3,19	-0,60917745
RPL4	15	complement(66498015..66524532)	310,40	#####	0,039624559
ZWILCH	15	66504959..66550130	4,83	7,77	-0,462053862
LCTL	15	complement(66547179..66565998)	0,10	0,19	-0,637355692
SMAD6	15	66702236..66782849	7,57	4,14	1,092881955
SMAD3	15	67063763..67195173	76,00	51,42	0,786745576
AAGAB	15	complement(67200667..67255195)	7,65	11,38	-0,349066503
IQCH	15	67254786..67502260	0,37	0,60	-0,49406751
C15orf61	15	67521131..67530146	0,63	1,24	-0,744920895
MAP2K5	15	67542703..67807117	4,06	7,58	-0,678077257
SKOR1	15	67819704..67834582	0,08	0,12	-0,309030447
PIAS1	15	68054309..68198603	3,13	3,83	-0,06779475
gene:ENSG000	15	complement(68184032..68229718)	0,48	0,49	0,177030123
CALML4	15	complement(68190705..68206079)	0,70	0,81	0,019607349
CLN6	15	complement(68206992..68257211)	7,25	10,39	-0,296806342
FEM1B	15	68277745..68295862	6,35	6,50	0,190582121
ITGA11	15	complement(68296532..68432163)	4,36	0,70	2,866060675
CORO2B	15	68578993..68727806	0,78	6,02	-2,721243998
ANP32A	15	complement(68778535..68820897)	29,56	41,13	-0,253606844
SPESP1	15	68818221..68946811	-	-	0,188027316
NOX5_1	15	68930504..69062743	-	-	#N/D
NOX5_2	15	69014695..69062762	0,01	0,07	-2,729265868
GLCE	15	69160584..69272217	3,44	4,96	-0,304174845
PAQR5	15	69298912..69407780	10,02	15,52	-0,408083683
KIF23	15	69414246..69448427	14,36	21,41	-0,353518595
RPLP1	15	69452814..69456205	213,58	#####	-0,188778778
TLE3	15	complement(70047790..70098176)	12,13	13,81	0,03587127
UACA	15	complement(70654554..70763558)	6,57	7,81	-0,026216357
LARP6	15	complement(70829130..70854157)	10,66	15,87	-0,35131301
LRRC49	15	70853239..71053658	0,36	0,78	-0,893131868
THAP10	15	complement(70881342..70892433)	1,11	2,02	-0,641242292
THSD4	15	71096952..71783383	3,20	6,10	-0,705685513
NR2E3	15	71792638..71818259	0,02	0,05	-0,895537122
MYO9A	15	complement(71822291..72118577)	1,62	3,30	-0,797884956

SENP8	15	72114258..72143692	0,25	0,52	-0,852591883
GRAMD2A	15	complement(72159806..72197787)	-	0,01	-4,758582517
PKM	15	complement(72199029..72231819)	396,90	#####	0,172463329
PARP6	15	complement(72241181..72272999)	5,35	9,39	-0,588452563
gene:ENSG000	15	complement(72266746..72319946)	-	-	#N/D
CELF6	15	complement(72284727..72320157)	-	0,01	-4,359055975
gene:ENSG000	15	complement(72284727..72375981)	0,01	0,02	-0,03547153
HEXA	15	complement(72340924..72376420)	4,80	6,39	-0,190755158
TMEM202	15	72398302..72408367	-	-	#N/D
ARIH1	15	72474330..72602987	2,76	4,56	-0,500393056
GOLGA6B	15	72654697..72669599	-	-	0,188027316
HIGD2B	15	complement(72675798..72686182)	0,02	0,06	-1,291882393
BBS4	15	72686179..72738475	1,92	3,63	-0,698733634
ADPGK	15	complement(72751294..72785846)	5,85	11,68	-0,773660038
NEO1	15	73051710..73305205	4,93	1,28	2,167365721
HCN4	15	complement(73319859..73368958)	0,00	-	4,516772761
REC114	15	73443164..73560013	0,01	-	3,068695355
NPTN	15	complement(73560014..73634134)	11,05	15,89	-0,300986053
CD276	15	73683966..73714514	29,10	49,27	-0,536379972
INSYN1	15	complement(73735458..73753351)	0,68	0,74	0,099845646
TBC1D21	15	73873564..73889214	-	-	#N/D
LOXL1	15	73925989..73952137	11,09	23,78	-0,876598715
STOML1	15	complement(73978926..73994622)	1,24	1,95	-0,428677114
PML	15	73994673..74047827	8,36	9,95	-0,028465167
GOLGA6A	15	complement(74069857..74082550)	0,01	0,02	-0,602143793
ISLR2	15	74100311..74138540	0,03	0,07	-0,754394027
ISLR	15	74173710..74176872	0,65	1,34	-0,814748863
STRA6	15	complement(74179466..74212267)	0,08	16,39	-7,458438174
CCDC33	15	74202705..74336472	0,04	0,82	-4,265830582
gene:ENSG000	15	74299503..74304527	-	0,20	-9,017250101
CYP11A1	15	complement(74337759..74367646)	0,21	1,12	-2,210332155
SEMA7A	15	complement(74409289..74433958)	18,24	21,88	-0,039899573
UBL7	15	complement(74445977..74461182)	14,27	29,53	-0,825798389
ARID3B	15	74541206..74598131	2,46	4,36	-0,605211166
CLK3	15	74598500..74645414	4,29	4,12	0,279395725
EDC3	15	complement(74630558..74696292)	13,81	12,19	0,402319172
CYP1A1	15	complement(74719542..74725536)	4,82	5,94	-0,077791085
CYP1A2	15	74748845..74756607	0,02	0,00	2,399873901
CSK	15	74782080..74803197	12,44	28,91	-0,99322312
LMAN1L	15	74812716..74825757	0,01	0,01	-0,923955849
CPLX3	15	74826627..74831802	0,04	0,03	0,438394362
ULK3	15	complement(74836118..74843346)	5,21	9,34	-0,618443139
SCAMP2	15	complement(74843730..74873365)	12,49	19,35	-0,408571246
MPI	15	74890005..74902219	2,15	3,29	-0,395020871
FAM219B	15	complement(74899992..74906883)	4,43	8,69	-0,748271532
COX5A	15	complement(74919791..74938083)	12,43	27,21	-0,907872471
RPP25	15	complement(74954418..74956772)	1,18	6,50	-2,235955358
SCAMP5	15	74957219..75021495	0,69	0,99	-0,292000053

PPCDC	15	75023586..75117462	0,98	2,24	-0,964290526
C15orf39	15	75195643..75212169	3,72	8,60	-0,98466837
GOLGA6C	15	75258334..75273455	0,00	0,01	-1,877914477
GOLGA6D	15	75282835..75295530	-	-	0,188027316
COMMD4	15	75336020..75343224	3,95	5,86	-0,344412823
NEIL1	15	75346955..75357115	0,80	1,74	-0,900528463
MAN2C1	15	complement(75355207..75368612)	10,63	18,04	-0,540890734
SIN3A	15	complement(75369379..75455842)	4,86	7,85	-0,470154711
PTPN9	15	complement(75463251..75579315)	4,26	9,03	-0,860665455
SNUPN	15	complement(75598083..75626469)	2,99	6,55	-0,908629538
IMP3	15	complement(75639085..75648706)	3,90	10,36	-1,187470814
SNX33	15	75647912..75662301	8,18	8,87	0,107352556
CSPG4	15	complement(75674322..75712848)	11,80	43,05	-1,644634851
CIMAP1C	15	75724041..75727688	0,08	0,35	-1,973246408
UBE2Q2	15	75843307..75901078	1,13	2,39	-0,852889925
FBXO22	15	75903876..75942511	0,94	1,70	-0,633780531
NRG4	15	complement(75935969..76059795)	0,03	0,07	-0,905350572
TMEM266	15	76059958..76229121	0,13	0,21	-0,485781181
ETFA	15	complement(76188555..76311730)	15,22	17,08	0,056445671
ISL2	15	76336773..76342475	0,01	1,54	-6,700853513
SCAPER	15	complement(76347904..76905444)	0,87	1,61	-0,664109426
RCN2	15	76931738..76954393	1,90	4,26	-0,94500135
PSTPIP1	15	76993359..77037475	0,02	0,05	-0,802495947
TSPAN3	15	complement(77041404..77083984)	4,92	11,49	-1,001379239
gene:ENSG000	15	complement(77063397..77064910)	0,11	0,26	-1,032283172
PEAK1	15	complement(77100656..77420144)	1,42	3,45	-1,060682907
HMG20A	15	77420412..77485607	6,31	10,31	-0,485948001
gene:ENSG000	15	complement(77484275..77485606)	-	-	0,188027316
LINGO1	15	complement(77613027..77820900)	0,06	0,18	-1,504360346
gene:ENSG000	15	complement(77784610..77785070)	-	-	#N/D
TBC1D2B	15	complement(77984036..78077724)	4,21	9,41	-0,938526974
SH2D7	15	78077808..78104370	0,00	0,02	-1,478387936
CIB2	15	complement(78104606..78131535)	0,86	1,10	-0,122619566
IDH3A	15	78131498..78171945	3,34	6,13	-0,653533027
ACSBG1	15	complement(78167468..78245688)	0,05	0,04	0,678797266
DNAJA4	15	78264086..78282196	0,14	0,09	0,777984703
SKIC8	15	complement(78277835..78299703)	7,39	7,75	0,153669942
gene:ENSG000	15	complement(78339208..78341757)	-	0,01	-2,89252005
CRABP1	15	78340353..78348225	-	-	0,188027316
IREB2	15	78437431..78501453	1,89	4,26	-0,952168107
HYKK	15	78507564..78537372	0,48	0,43	0,381678904
gene:ENSG000	15	78537681..78538946	0,09	0,01	3,257511884
PSMA4	15	78540405..78552417	3,40	10,77	-1,441545602
CHRNA5	15	78565520..78595269	0,13	0,71	-2,213905246
CHRNA3	15	complement(78593052..78621295)	0,01	0,09	-2,436352761
gene:ENSG000	15	complement(78599892..78600013)	-	-	#N/D
CHRNB4	15	complement(78624111..78727754)	-	0,00	-2,89252005
ADAMTS7	15	complement(78759206..78811464)	1,09	1,03	0,298571168

MORF4L1	15	78810487..78898139	23,81	40,22	-0,533067666
gene:ENSG000	15	78906127..78906809	0,44	0,74	-0,5158084
CTSH	15	complement(78921058..78949574)	0,15	0,37	-1,138212777
RASGRF1	15	complement(78959906..79090780)	0,01	0,01	-0,291161992
ANKRD34C	15	79282722..79298239	-	-	0,188027316
TMED3	15	79311112..79427432	4,28	2,52	0,987792262
MINAR1	15	79432336..79472304	0,67	0,44	0,832190981
MTHFS	15	complement(79833585..79897379)	0,37	1,07	-1,296794239
ST20-MTHFS	15	complement(79845150..79923754)	2,01	5,79	-1,302462669
gene:ENSG000	15	complement(79875920..79878179)	0,03	0,05	-0,442604891
BCL2A1	15	complement(79960892..79971196)	0,03	0,98	-4,61367294
gene:ENSG000	15	79989905..79990606	-	-	#N/D
gene:ENSG000	15	80014157..80015707	0,01	-	3,068695355
ZFAND6	15	80059568..80138393	5,88	12,23	-0,83273495
FAH	15	80152490..80186946	5,42	7,74	-0,290594738
CTXND1	15	complement(80195481..80252213)	0,03	0,06	-0,694397966
ARNT2	15	80404350..80597933	2,09	6,16	-1,334702718
ABHD17C	15	80679684..80755621	3,76	5,82	-0,405809842
CEMIP	15	80779343..80951776	1,89	2,18	0,012207247
MESD	15	complement(80946289..80989828)	1,97	3,95	-0,775810122
TLNRD1	15	81000923..81005788	1,48	3,51	-1,022214854
gene:ENSG000	15	81002142..81002279	-	-	#N/D
CFAP161	15	81007033..81149179	0,02	0,01	0,524121557
gene:ENSG000	15	81060953..81063488	0,01	0,01	-0,025382928
IL16	15	81159575..81314058	0,06	0,06	0,055652845
STARD5	15	complement(81309053..81324183)	1,09	1,09	0,227151713
TMC3	15	complement(81331088..81374213)	0,06	0,10	-0,452912276
MEX3B	15	complement(82041778..82046119)	0,32	1,39	-1,918107441
EFL1	15	complement(82130206..82262773)	2,53	7,62	-1,368369732
SAXO2	15	82262810..82284930	0,12	0,12	0,25844112
GOLGA6L10	15	complement(82339993..82349475)	0,17	0,26	-0,438995678
GOLGA6L9	15	82429816..82439153	0,39	1,16	-1,35071792
RPS17	15	complement(82536750..82540459)	106,57	#####	-0,6330351
gene:ENSG000	15	complement(82536788..82573194)	-	0,25	-8,107938261
CPEB1	15	complement(82543201..82648861)	0,49	3,18	-2,47795229
AP3B2	15	complement(82659281..82710112)	0,00	0,04	-2,945723354
gene:ENSG000	15	82738386..82739122	0,28	0,30	0,120130055
FSD2	15	complement(82755362..82806070)	0,27	0,27	0,229166235
WHAMM	15	82809628..82836108	3,26	3,33	0,192922387
HOMER2	15	complement(82836946..82986153)	1,02	3,58	-1,589669461
RAMAC	15	82986210..82991057	2,85	6,25	-0,912834492
C15orf40	15	complement(82988441..83011641)	0,64	1,04	-0,47417078
BTBD1	15	complement(83016423..83067252)	4,60	11,11	-1,050478015
TM6SF1	15	83107572..83144854	0,02	0,06	-1,151227584
HDGFL3	15	complement(83112738..83207875)	0,67	1,79	-1,197368526
BNC1	15	complement(83255884..83284664)	3,16	9,54	-1,369513096
SH3GL3	15	83447228..83618743	0,05	0,57	-3,162896655
ADAMTSL3	15	83654088..84039842	-	0,02	-5,902279478

DNM1P41	15	complement(84166547..84169896)	0,23	0,28	-0,060555629
GOLGA6L4	15	84235773..84245358	0,13	0,41	-1,463046383
ZSCAN2	15	84600986..84627796	0,83	1,49	-0,624268632
WDR73	15	complement(84639285..84654343)	2,38	4,69	-0,753171568
NMB	15	complement(84655129..84658563)	0,44	0,14	1,87991332
SEC11A	15	complement(84669538..84716460)	13,92	23,94	-0,55927518
ZNF592	15	84748592..84806445	6,10	9,09	-0,353235433
ALPK3	15	84817356..84873479	0,03	0,09	-1,388949567
SLC28A1	15	84884654..84945798	-	0,01	-3,804623889
PDE8A	15	84980440..85139145	3,12	7,58	-1,055732253
AKAP13	15	85380571..85749358	3,24	7,87	-1,057274994
KLHL25	15	complement(85759326..85794925)	1,76	2,21	-0,104837876
AGBL1	15	86079871..87049169	-	-	0,188027316
NTRK3	15	complement(87859751..88256791)	0,04	0,13	-1,640981367
MRPL46	15	complement(88459477..88467390)	3,47	8,07	-0,993649272
gene:ENSG000	15	complement(88459501..88546585)	0,13	0,25	-0,744315315
MRPS11	15	88467453..88480776	2,62	5,30	-0,790998583
gene:ENSG000	15	complement(88477871..88478387)	1,31	1,68	-0,128982653
DET1	15	complement(88494440..88546681)	1,17	1,90	-0,48237537
AEN	15	88621337..88632281	8,05	17,40	-0,888127907
ISG20	15	88635670..88656483	0,36	2,62	-2,650382714
ACAN	15	88803436..88875353	0,00	0,00	-1,877914477
HAPLN3	15	complement(88877294..88895597)	1,40	1,11	0,557503009
MFGE8	15	complement(88898683..88913468)	3,29	5,28	-0,460628553
ABHD2	15	89087459..89202355	16,66	14,82	0,391897972
RLBP1	15	complement(89209869..89221614)	-	0,01	-2,89252005
FANCI	15	89243945..89317261	6,94	16,17	-0,996621628
POLG	15	complement(89305198..89334861)	14,66	18,07	-0,078769192
POLGARF	15	complement(89330208..89333809)	-	-	#N/D
gene:ENSG000	15	89358841..89360377	-	-	#N/D
RHCG	15	complement(89471398..89496589)	0,06	0,08	-0,038531947
TICRR	15	89575469..89631056	5,01	6,82	-0,220551435
KIF7	15	complement(89608789..89663086)	5,07	9,20	-0,635049939
PLIN1	15	complement(89664367..89679427)	0,33	0,33	0,222091282
PEX11A	15	complement(89677764..89690783)	1,47	2,63	-0,614880195
WDR93	15	89690811..89743638	0,03	0,06	-0,877094735
MESP1	15	complement(89749875..89751249)	1,15	2,38	-0,820595829
MESP2	15	89760591..89778754	-	0,01	-2,89252005
ANPEP	15	complement(89784895..89815401)	122,13	#####	-0,10223687
AP3S2	15	complement(89830599..89894638)	4,58	7,52	-0,492227503
ARPIN-AP3S2	15	complement(89834308..89912882)	3,60	11,59	-1,465225614
ARPIN	15	complement(89895006..89912952)	0,84	2,02	-1,03746601
gene:ENSG000	15	89950760..89951345	0,02	-	3,068695355
ZNF710	15	90001324..90082206	3,04	6,97	-0,974119272
IDH2	15	complement(90083045..90102477)	16,60	13,84	0,485106542
SEMA4B	15	90160604..90229679	11,32	16,44	-0,314735912
CIB1	15	complement(90229975..90234047)	54,92	51,02	0,329318146
GDPGP1	15	90233808..90245811	0,31	1,15	-1,68514149

gene:ENSG000	15	90234200..90265462	0,03	0,22	-2,422303057
TTLL13	15	90249530..90265482	0,00	0,08	-4,423353916
gene:ENSG000	15	90249556..90272208	0,11	0,27	-1,055535135
NGRN	15	90265659..90275778	18,73	39,50	-0,853084779
gene:ENSG000	15	90266317..90314499	0,02	-	3,068695355
GABARAPL3	15	90347587..90349437	-	-	0,188027316
ZNF774	15	90352284..90369146	0,18	0,61	-1,520157007
IQGAP1	15	90388242..90502239	18,83	33,20	-0,595362922
CRTC3	15	90529923..90645345	3,23	8,61	-1,19174072
BLM	15	90717346..90816166	0,75	2,14	-1,280835381
FURIN	15	90868588..90883564	16,71	37,34	-0,936432165
FES	15	90883695..90895776	0,03	0,04	-0,297455117
MAN2A2	15	90902218..90922584	4,51	10,41	-0,982005985
HDDC3	15	complement(90929968..90935196)	3,63	9,15	-1,10986344
UNC45A	15	90930180..90954093	10,67	18,37	-0,560733465
RCCD1	15	90954881..90963125	4,65	9,72	-0,841208177
PRC1	15	complement(90966040..90995629)	28,77	46,65	-0,474510175
gene:ENSG000	15	complement(90966190..91022566)	4,47	5,82	-0,158254017
VPS33B	15	complement(90998673..91022603)	2,17	3,93	-0,631364239
SV2B	15	91099950..91302565	0,00	-	3,068695355
SLCO3A1	15	91853708..92172435	1,59	4,61	-1,311005746
ST8SIA2	15	92393881..92468728	0,01	-	4,516772761
FAM174B	15	complement(92617448..92809884)	1,61	1,10	0,7662619
CHD2	15	92886203..93027996	3,70	4,63	-0,099551831
RGMA	15	complement(93035271..93089211)	0,09	0,08	0,397346413
MCTP2	15	94231538..94483952	0,85	1,30	-0,38255331
gene:ENSG000	15	complement(95280753..95284249)	-	-	0,188027316
gene:ENSG000	15	complement(96112628..96116051)	0,00	-	3,068695355
gene:ENSG000	15	complement(96294935..96298023)	0,03	0,01	1,655502569
NR2F2	15	96325938..96340263	0,93	2,97	-1,458231337
ARRDC4	15	97960703..97973833	1,33	0,62	1,334777394
IGF1R	15	98648539..98964530	0,83	4,58	-2,243511506
PGPEP1L	15	complement(98968229..99007792)	-	0,02	-3,804623889
SYNM	15	99098217..99135593	1,33	2,39	-0,625240801
TTC23	15	complement(99136323..99251223)	2,29	3,08	-0,205616494
LRRC28	15	99251362..99390729	0,86	1,37	-0,45219154
gene:ENSG000	15	complement(99497812..99498375)	0,06	0,02	1,436225396
MEF2A	15	99565417..99716488	6,44	6,90	0,122116577
LYSMD4	15	complement(99715697..99733561)	0,44	0,86	-0,73653249
ADAMTS17	15	complement(99971437..100342005	0,03	0,15	-2,182763817
CERS3	15	complement(100400395..10054499	0,01	0,04	-2,302004938
LINS1	15	complement(100559369..10060323	0,75	0,93	-0,093108528
ASB7	15	100602589..100651701	1,74	3,00	-0,567083464
gene:ENSG000	15	complement(100782417..10078618	0,17	0,52	-1,35563396
ALDH1A3	15	100877714..100916626	115,24	#####	-0,342214835
LRRK1	15	100919327..101078257	3,68	7,60	-0,823795189
CHSY1	15	complement(101175727..10125204	10,20	14,01	-0,235269505
SELENOS	15	complement(101270817..10127750	6,16	5,83	0,300362461

SNRPA1	15	complement(101281510..10129528	15,41	24,25	-0,430993716
PCSK6	15	complement(101289784..10152520	1,69	2,60	-0,400822957
gene:ENSG000	15	complement(101299656..10130164	0,81	1,42	-0,595863926
TM2D3	15	complement(101621444..10165239	3,47	5,29	-0,385221493
TARS3	15	complement(101653596..10172447	0,65	0,88	-0,221962423
OR4F6	15	101803509..101806887	-	-	0,188027316
OR4F15	15	101812202..101820197	0,01	0,01	-0,01185201
OR4F4	15	complement(101922042..10192311	-	-	#N/D
POLR3K	16	complement(46407..53608)	7,28	10,40	-0,291853814
SNRNP25	16	53828..57669	7,07	9,32	-0,175284007
RHBDF1	16	complement(58059..76355)	9,45	8,49	0,378112489
gene:ENSG000	16	74683..74961	-	-	0,188027316
MPG	16	77007..85851	26,86	22,03	0,508961051
NPRL3	16	complement(84271..138677)	13,57	24,49	-0,628041273
HBZ	16	142728..154503	0,03	0,01	1,436225396
HBM	16	153892..166764	-	-	#N/D
HBA2	16	172876..173710	2,45	1,48	0,949205315
HBA1	16	176680..177522	0,28	1,28	-1,955239165
HBQ1	16	180459..181179	-	-	0,188027316
LUC7L	16	complement(188969..229463)	5,21	5,18	0,23206635
FAM234A	16	234521..272183	19,92	25,40	-0,127804007
RGS11	16	complement(268301..275980)	0,03	0,01	1,487770062
ARHGDIG	16	280450..283010	0,25	0,19	0,675749352
PDIA2	16	283164..287215	0,07	0,02	1,790975958
AXIN1	16	complement(287440..352723)	8,73	14,75	-0,534105472
MRPL28	16	complement(366969..371289)	19,42	22,52	0,00925462
PGAP6	16	complement(370788..387113)	11,23	11,73	0,159101224
NME4	16	396725..410367	37,05	38,59	0,164334575
DECR2	16	401858..412487	5,14	7,34	-0,291068583
RAB11FIP3	16	425649..523011	6,02	8,57	-0,287183511
CAPN15	16	527712..554636	12,05	15,71	-0,160296698
PRR35	16	560394..565529	0,03	0,03	0,438394362
PIGQ	16	566995..584109	4,60	6,90	-0,362493201
NHLRC4	16	567005..569495	0,19	0,09	1,215597416
RAB40C	16	589357..629272	5,69	5,05	0,396519841
WFIKKN1	16	629239..634117	0,60	0,64	0,140630516
METTL26	16	complement(634427..636366)	21,00	23,74	0,045926511
MCRIP2	16	636817..648474	8,55	11,18	-0,163229902
WDR90	16	649311..667833	9,31	8,72	0,317880819
RHOT2	16	668100..674174	30,33	27,81	0,348442997
RHBDL1	16	675671..678268	6,75	4,67	0,754353732
gene:ENSG000	16	678645..679061	0,75	0,81	0,117033586
STUB1	16	680224..682870	22,92	27,00	-0,013262947
JMJD8	16	complement(681670..684528)	8,65	8,06	0,324681872
WDR24	16	complement(684622..690444)	6,79	5,58	0,50643216
FBXL16	16	complement(692498..705801)	0,04	0,08	-0,838652411
gene:ENSG000	16	707650..709816	0,04	0,03	0,7939095
METRN	16	715118..719655	5,85	5,36	0,349826286

ANTKMT	16	720581..722590	3,57	3,76	0,150639925
CCDC78	16	complement(722582..726954)	1,49	2,18	-0,323394968
HAGHL	16	726936..735525	4,43	4,89	0,081256868
CIAO3	16	complement(729760..741329)	4,70	4,13	0,409329682
MSLN	16	760734..768865	0,01	0,09	-2,481072915
gene:ENSG000	16	complement(769119..769443)	-	-	0,188027316
MSLNL	16	complement(769428..782054)	-	-	0,188027316
RPUSD1	16	complement(784974..788397)	6,26	7,62	-0,061362595
CHTF18	16	788046..800737	10,89	11,08	0,197954202
GNG13	16	complement(798041..800734)	0,09	0,13	-0,35396495
LMF1	16	complement(853634..981318)	1,89	1,11	0,993979453
SOX8	16	981444..986979	0,26	0,29	0,066219505
SSTR5	16	1063867..1081454	0,29	0,68	-1,014912941
C1QTNF8	16	complement(1088226..1096711)	0,21	1,08	-2,121842649
CACNA1H	16	1153103..1224169	1,76	1,68	0,283770166
TPSG1	16	complement(1221651..1225793)	-	0,48	-8,415177538
TPSB2	16	complement(1227272..1232757)	0,01	0,01	-0,01185201
TPSAB1	16	1239266..1242554	-	-	0,188027316
TPSD1	16	1256059..1259008	-	-	#N/D
UBE2I	16	1291444..1327018	11,59	14,12	-0,062535667
BAIAP3	16	1333638..1349439	0,58	0,25	1,429084215
TSR3	16	complement(1349240..1351878)	12,23	17,21	-0,268773927
GNPTG	16	1351931..1365737	4,88	4,66	0,289891585
UNKL	16	complement(1363205..1414751)	7,67	9,84	-0,136442698
UQCC4	16	complement(1419752..1420756)	10,76	9,39	0,419491342
PERCC1	16	1431078..1434441	0,02	0,05	-0,820024868
CCDC154	16	complement(1434383..1444556)	0,21	0,13	0,926873464
gene:ENSG000	16	1437154..1439315	0,13	0,14	0,129376454
CLCN7	16	complement(1444934..1475077)	8,39	12,13	-0,30864186
PTX4	16	complement(1485886..1488981)	-	-	0,188027316
TELO2	16	1493344..1510457	8,49	10,59	-0,096476822
IFT140	16	complement(1510427..1612072)	5,36	4,66	0,424857239
TMEM204	16	1528688..1555580	0,15	0,21	-0,185877329
gene:ENSG000	16	1530715..1533301	0,07	0,16	-1,032816922
gene:ENSG000	16	complement(1553655..1554130)	0,27	0,54	-0,768720584
gene:ENSG000	16	complement(1576716..1578371)	0,27	0,17	0,853990683
CRAMP1	16	1612337..1677908	4,44	5,57	-0,10431455
gene:ENSG000	16	1632259..1686715	0,85	0,79	0,344764241
JPT2	16	1678256..1702280	18,48	23,05	-0,09531789
gene:ENSG000	16	1688355..1690536	0,69	0,65	0,320060571
MAPK8IP3	16	1706166..1770351	15,77	18,33	0,006291381
NME3	16	complement(1770320..1771561)	0,86	1,02	-0,02187385
MRPS34	16	complement(1771890..1773150)	26,59	32,34	-0,059311633
EME2	16	1772810..1781708	5,13	3,99	0,586969807
SPSB3	16	complement(1776712..1793700)	18,14	11,83	0,839728606
NUBP2	16	1782932..1789186	20,06	18,77	0,31894973
IGFALS	16	complement(1790413..1794971)	1,10	0,56	1,194761729
HAGH	16	complement(1795620..1827157)	5,95	7,89	-0,182923414

FAHD1	16	1826967..1840207	0,99	1,60	-0,480321391
MEIOB	16	complement(1833986..1884294)	1,62	4,61	-1,290669241
HS3ST6	16	complement(1911475..1918415)	-	-	0,188027316
MSRB1	16	complement(1938229..1943199)	10,80	15,35	-0,285023613
RPL3L	16	complement(1943974..1957606)	0,08	0,07	0,397821164
NDUFB10	16	1959538..1961975	34,10	47,65	-0,259423529
RPS2	16	complement(1962058..1964841)	889,31	#####	0,059918138
RNF151	16	1966823..1968975	0,07	0,11	-0,442604891
TBL3	16	1972053..1982929	10,03	10,03	0,223108584
NOXO1	16	complement(1978917..1984192)	-	-	0,188027316
GFER	16	1984193..1987749	9,61	9,36	0,261738765
SYNGR3	16	1989660..1994275	0,48	0,65	-0,201502419
ZNF598	16	complement(1997654..2009821)	34,84	37,55	0,114869701
NPW	16	2009926..2020755	0,17	0,15	0,404132137
NHERF2	16	2025356..2039026	2,56	5,79	-0,953574561
NTHL1	16	complement(2039815..2047866)	9,97	16,87	-0,534822698
TSC2	16	2047967..2089491	6,16	6,76	0,088219035
PKD1	16	complement(2088708..2135898)	29,00	29,07	0,21975628
RAB26	16	2140803..2154165	1,51	3,10	-0,812582185
TRAF7	16	2155698..2178129	40,34	33,59	0,487631222
CASKIN1	16	complement(2177180..2196605)	0,25	0,21	0,46572062
MLST8	16	2204248..2209453	8,31	12,07	-0,314991708
BRICD5	16	complement(2209253..2210905)	3,64	2,94	0,527513287
PGP	16	complement(2211593..2214840)	8,34	6,54	0,573245684
E4F1	16	2223580..2235742	10,98	9,95	0,365731884
DNASE1L2	16	2235816..2238711	2,50	2,56	0,186251053
ECI1	16	complement(2239402..2252300)	6,71	12,34	-0,656204376
RNPS1	16	complement(2253116..2268397)	41,89	61,26	-0,325299069
ABCA3	16	complement(2275881..2340746)	0,46	0,24	1,151014776
CCNF	16	2429394..2458854	22,95	23,77	0,172761339
TEDC2	16	2460086..2464963	4,08	5,66	-0,248069087
NTN3	16	2471297..2474145	0,48	1,03	-0,882689863
TBC1D24	16	2475051..2509560	3,34	3,80	0,037167825
gene:ENSG000	16	2496032..2520218	0,53	0,17	1,838579345
ATP6V0C	16	2513952..2520218	58,69	50,32	0,445062377
gene:ENSG000	16	2513965..2527955	0,05	0,13	-0,999120035
gene:ENSG000	16	2516658..2517999	2,51	1,28	1,193123986
AMDHD2	16	2520357..2531422	2,59	2,58	0,226182331
CEMP1	16	complement(2530035..2531417)	0,83	1,09	-0,156946502
PDPK1	16	2537979..2603188	5,24	5,16	0,247401832
gene:ENSG000	16	complement(2575628..2577373)	2,36	1,04	1,406475279
gene:ENSG000	16	2578395..2579963	1,51	0,73	1,273310974
gene:ENSG000	16	2592514..2594563	0,45	0,27	0,940416551
gene:ENSG000	16	complement(2620222..2621794)	0,94	0,47	1,222523521
KCTD5	16	2682523..2709030	22,67	18,35	0,528225375
gene:ENSG000	16	2683106..2684764	1,85	0,96	1,165575993
PRSS27	16	complement(2712419..2720551)	0,51	1,22	-1,031513688
SRRM2	16	2752626..2772538	82,56	93,48	0,043902923

ELOB	16	complement(2771414..2777289)	18,23	18,48	0,203632027
PRSS33	16	complement(2783953..2787948)	0,01	0,02	-0,979341556
PRSS41	16	2798470..2805304	-	0,01	-2,89252005
PRSS21	16	2817180..2826304	-	-	#N/D
ZG16B	16	2830253..2839585	0,01	0,20	-4,107069724
PRSS22	16	complement(2852730..2858170)	-	-	0,188027316
FLYWCH2	16	2883213..2899382	20,95	21,33	0,197091442
FLYWCH1	16	2911931..2951208	11,35	15,84	-0,257237266
KREMEN2	16	2964216..2968380	0,32	0,53	-0,480255549
PKMYT1	16	complement(2968024..2980479)	7,46	10,43	-0,259545054
PAQR4	16	2969270..2973484	11,63	10,18	0,414732396
GREP1	16	2988256..3002295	-	0,01	-3,804623889
gene:ENSG000	16	3004132..3004859	-	0,02	-2,89252005
CLDN9	16	3012923..3014505	0,20	0,19	0,292946376
CLDN6	16	complement(3014712..3020071)	0,01	0,04	-1,54862452
TNFRSF12A	16	3018445..3022383	55,30	61,06	0,080112136
HCFC1R1	16	complement(3022625..3024286)	42,80	35,53	0,491772311
THOC6	16	3024027..3027755	25,20	30,59	-0,05648319
BICDL2	16	complement(3027682..3036944)	0,06	0,07	-0,039381237
MMP25	16	3046062..3060726	0,40	0,75	-0,697141801
IL32	16	3065297..3082192	3,20	7,29	-0,962933965
ZSCAN10	16	complement(3088890..3099295)	0,01	-	3,967268276
ZNF205	16	3112560..3120517	2,71	3,40	-0,103547806
ZNF213	16	3129777..3142804	2,84	3,90	-0,231141311
OR1F1	16	3188204..3224779	0,07	0,16	-0,990391258
ZNF200	16	complement(3222325..3236221)	1,06	1,50	-0,275373822
MEFV	16	complement(3242027..3256633)	0,06	0,06	0,097248751
gene:ENSG000	16	3263800..3291029	2,68	1,15	1,442462137
gene:ENSG000	16	3273609..3275807	0,05	0,04	0,822166453
ZNF263	16	3283283..3301401	3,60	3,01	0,482309664
gene:ENSG000	16	3292879..3293403	-	-	0,188027316
TIGD7	16	complement(3298808..3305729)	1,35	0,57	1,477980238
ZNF75A	16	3305406..3318852	0,56	1,13	-0,786528012
OR2C1	16	3355889..3357306	0,01	-	3,068695355
gene:ENSG000	16	3365099..3479550	-	0,07	-4,359055975
ZSCAN32	16	complement(3382081..3401065)	2,13	3,00	-0,268897166
ZNF174	16	3401215..3409364	2,87	4,05	-0,274421689
ZNF597	16	complement(3432414..3443504)	0,33	0,37	0,039173645
NAA60	16	3443649..3486953	9,46	11,17	-0,016368557
C16orf90	16	complement(3493484..3495489)	0,06	0,01	2,144183439
CLUAP1	16	3500976..3539048	1,41	2,34	-0,506260364
gene:ENSG000	16	complement(3533429..3534258)	0,12	-	6,053060169
NLRC3	16	complement(3539033..3577403)	0,22	0,33	-0,39442546
SLX4	16	complement(3581181..3611606)	4,67	5,61	-0,040872463
DNASE1	16	3611728..3680143	1,57	1,62	0,172771903
TRAP1	16	complement(3651639..3717553)	33,78	51,90	-0,396588895
CREBBP	16	complement(3725054..3880713)	4,83	8,50	-0,591308456
ADCY9	16	complement(3953387..4116442)	5,41	7,85	-0,31414027

SRL	16	complement(4189374..4242080)	0,01	0,02	-0,895537122
TFAP4	16	complement(4257186..4273075)	6,42	12,79	-0,770272583
GLIS2	16	4314761..4339597	5,84	8,67	-0,347017127
PAM16	16	complement(4331549..4355607)	10,76	15,47	-0,300429196
CORO7-PAM16	16	complement(4340251..4420494)	0,34	0,53	-0,404141026
gene:ENSG000	16	complement(4346694..4348648)	3,23	2,71	0,475367437
CORO7	16	complement(4354542..4425705)	3,05	4,01	-0,170129754
VASN	16	4371848..4383538	25,19	38,26	-0,379541081
DNAJA3	16	4425805..4456775	23,67	34,79	-0,332882758
NMRAL1	16	complement(4461691..4495763)	2,69	3,76	-0,260948915
HMOX2	16	4474690..4510347	7,87	9,14	0,006572784
CDIP1	16	complement(4510669..4538828)	6,57	8,56	-0,159933147
C16orf96	16	4556340..4600758	0,02	0,05	-1,43066689
UBALD1	16	complement(4608883..4615027)	8,73	8,79	0,213540479
MGRN1	16	4616493..4690974	16,38	14,20	0,429200276
NUDT16L1	16	4693562..4695859	6,43	4,67	0,684320975
ANKS3	16	complement(4696510..4734378)	5,70	3,33	1,001376008
DNAAF8	16	4734344..4749396	0,38	0,20	1,171331558
ZNF500	16	complement(4748239..4767624)	2,83	3,04	0,12158064
SEPTIN12	16	complement(4777606..4788398)	-	-	0,188027316
SMIM22	16	4788397..4796491	0,07	0,07	0,152452079
ROGDI	16	complement(4796968..4802880)	2,12	1,28	0,946604904
GLYR1	16	complement(4803203..4847288)	7,39	12,04	-0,481375808
UBN1	16	4846665..4882401	9,41	8,44	0,379703672
PPL	16	complement(4882507..4960741)	0,19	0,20	0,16658898
SEC14L5	16	4958330..5019157	0,00	0,00	-0,025382928
NAGPA	16	complement(5024844..5034141)	4,64	4,76	0,183549278
ALG1	16	5033702..5087379	1,97	2,26	0,027332099
C16orf89	16	complement(5044122..5066110)	0,01	-	3,068695355
EEF2KMT	16	complement(5084284..5097795)	2,48	5,04	-0,801227978
RBFOX1	16	5239802..7713340	0,00	0,00	0,886720911
gene:ENSG000	16	6037210..6038977	-	-	0,188027316
gene:ENSG000	16	6703883..6705558	-	-	#N/D
TMEM114	16	complement(8537605..8590511)	-	-	0,188027316
METTL22	16	8621683..8649654	3,71	4,08	0,088362901
gene:ENSG000	16	8666762..8667271	0,27	0,28	0,199980087
ABAT	16	8674596..8784575	0,13	0,12	0,341238756
TMEM186	16	complement(8780384..8797642)	5,01	7,34	-0,329501722
PMM2	16	8788823..8862534	15,01	10,37	0,756378082
CARHSP1	16	complement(8852942..8869012)	12,76	10,20	0,54699767
LITAFD	16	8882351..8885351	-	-	0,188027316
USP7	16	complement(8892097..8975328)	10,88	14,71	-0,212070413
HAPSTR1	16	9091644..9121635	8,52	6,54	0,603384382
GRIN2A	16	complement(9753404..10182928)	0,02	0,05	-1,379073191
ATF7IP2	16	10326434..10483638	0,69	0,86	-0,084786768
gene:ENSG000	16	complement(10445309..10449747)	0,39	0,27	0,772542665
EMP2	16	complement(10528422..10580632)	11,00	8,54	0,588455946
TEKT5	16	complement(10627501..10694930)	-	-	0,188027316

NUBP1	16	10743786..10769351	9,47	11,82	-0,097184953
TVP23A	16	complement(10760919..10818794)	0,10	0,13	-0,19460957
CIITA	16	10866222..10943021	0,30	0,23	0,613013498
DEXI	16	complement(10928891..10942468)	5,51	4,69	0,455461536
gene:ENSG000	16	10933903..10936280	0,73	0,45	0,922552392
CLEC16A	16	10944539..11182186	3,51	4,60	-0,165550272
RMI2	16	11249619..11381662	2,72	4,14	-0,382018814
SOCS1	16	complement(11254417..11256204)	0,66	1,48	-0,95104388
TNP2	16	complement(11267748..11269533)	0,01	-	3,068695355
PRM3	16	complement(11273199..11273629)	-	-	#N/D
PRM2	16	complement(11275639..11276480)	-	-	#N/D
PRM1	16	complement(11280841..11281330)	0,05	-	3,967268276
gene:ENSG000	16	complement(11359845..11527247)	0,14	0,10	0,613456203
LITAF	16	complement(11547722..11636381)	11,22	18,05	-0,462809799
SNN	16	11668455..11679152	7,18	8,94	-0,094967693
TXNDC11	16	complement(11679083..11742857)	13,29	10,83	0,51942112
ZC3H7A	16	complement(11750586..11797258)	6,77	6,77	0,222618451
RSL1D1	16	complement(11833850..11851580)	11,41	20,33	-0,610330301
GSPT1	16	complement(11868128..11916082)	18,00	22,92	-0,125918219
NPIPB2	16	complement(11927259..11976643)	1,75	1,71	0,253442086
TNFRSF17	16	11965210..11968068	-	-	0,188027316
SNX29	16	11976734..12574287	4,59	3,52	0,604899726
gene:ENSG000	16	12374355..12376032	0,08	0,08	0,221705268
CPPED1	16	complement(12659799..12803887)	1,67	2,98	-0,609488455
SHISA9	16	12901598..13240416	-	0,00	-2,89252005
ERCC4	16	13920138..13952348	1,05	1,63	-0,402446986
MRTFB	16	14071319..14266773	1,96	2,31	-0,010353408
PARN	16	complement(14435700..14632728)	3,63	6,68	-0,657858536
BFAR	16	14632931..14669236	15,81	17,94	0,040431715
PLA2G10	16	complement(14672548..14694308)	-	0,02	-2,89252005
NPIPA3	16	14703411..14726342	0,26	0,14	1,139303216
NPIPA2	16	14742533..14765462	1,27	1,78	-0,263950131
NOMO1	16	14833721..14896157	40,79	50,53	-0,086070488
NPIPA1	16	14922802..14952060	10,41	14,89	-0,293497216
PDXDC1	16	14974591..15139339	36,58	24,23	0,817498935
NTAN1	16	complement(15037854..15056079)	11,90	9,31	0,577562293
RRN3	16	complement(15060033..15094311)	9,11	12,72	-0,25889988
NPIPA5	16	complement(15363628..15381047)	5,80	6,93	-0,033753945
MPV17L	16	15395754..15413271	0,03	0,00	2,688971416
gene:ENSG000	16	15395754..15515348	-	-	#N/D
BMERB1	16	15434475..15625028	3,87	10,76	-1,250202907
MARF1	16	complement(15594387..15643154)	10,81	7,88	0,678167939
NDE1	16	15643267..15734691	8,55	8,56	0,220787674
MYH11	16	complement(15703135..15858438)	0,11	0,16	-0,322370277
gene:ENSG000	16	complement(15790975..15792717)	-	-	#N/D
CEP20	16	complement(15865719..15888625)	13,92	17,06	-0,070932937
ABCC1	16	15949138..16143257	28,96	31,05	0,122616329
ABCC6	16	complement(16149565..16223522)	0,05	0,06	-0,145997477

NOMO3	16	16232506..16300806	5,48	7,70	-0,268061984
gene:ENSG000	16	16317444..16350590	14,63	15,93	0,100213988
NPIPA7	16	16379140..16393972	1,67	2,74	-0,486538445
gene:ENSG000	16	16760905..16762465	-	-	#N/D
gene:ENSG000	16	complement(17082295..17085049)	-	-	0,188027316
XYLT1	16	complement(17101769..17470960)	7,42	5,37	0,6891475
gene:ENSG000	16	complement(17192863..17194232)	0,12	0,13	0,059935958
gene:ENSG000	16	complement(17227023..17228000)	0,24	0,32	-0,164183768
gene:ENSG000	16	complement(17249674..17251395)	0,13	0,11	0,471230033
gene:ENSG000	16	17961597..17962370	-	-	0,188027316
gene:ENSG000	16	18201501..18201956	-	-	#N/D
NPIPA8	16	complement(18317919..18336736)	4,15	2,81	0,787727977
NPIPA9	16	complement(18358086..18379331)	7,63	12,94	-0,539237216
NOMO2	16	complement(18417325..18562211)	39,69	47,76	-0,043903754
RPS15A	16	complement(18781295..18790383)	62,68	70,87	0,045863706
gene:ENSG000	16	complement(18788063..18801519)	12,29	7,71	0,895758425
ARL6IP1	16	complement(18791669..18801572)	61,06	56,41	0,337180035
SMG1	16	complement(18804860..18926408)	10,90	11,44	0,153310357
TMC7	16	18983934..19063942	1,49	1,90	-0,124734033
COQ7	16	19067614..19080095	3,02	3,95	-0,162644908
ITPRIPL2	16	19113932..19121629	18,57	13,30	0,704954746
SYT17	16	19167971..19268332	1,06	0,55	1,165262941
CLEC19A	16	19285731..19322145	0,00	0,01	-1,478387936
gene:ENSG000	16	complement(19392213..19393468)	0,01	0,05	-2,190455314
TMC5	16	19410496..19499113	0,03	0,04	0,120130055
GDE1	16	complement(19501693..19522123)	11,53	11,80	0,18897328
gene:ENSG000	16	19503861..19504625	-	0,14	-5,734998842
CCP110	16	19523811..19553408	2,52	2,53	0,216190035
VPS35L	16	19555240..19706793	1,20	2,27	-0,694016843
KNOP1	16	complement(19701937..19718235)	3,62	5,77	-0,449715861
IQCK	16	19716456..19858467	0,72	1,42	-0,764561585
GPRC5B	16	complement(19856691..19886167)	0,41	3,28	-2,775814172
GPR139	16	complement(20028239..20073890)	-	-	#N/D
GP2	16	complement(20309574..20327808)	0,01	-	3,967268276
UMOD	16	complement(20333051..20356301)	-	-	0,188027316
PDILT	16	complement(20359175..20404737)	-	-	#N/D
ACSM5	16	20409534..20441336	-	0,00	-2,89252005
ACSM2A	16	20451461..20487669	-	-	#N/D
gene:ENSG000	16	20487702..20489659	-	-	#N/D
ACSM2B	16	complement(20536226..20576427)	-	-	#N/D
ACSM3	16	20610243..20797581	0,19	0,27	-0,317402277
ACSM1	16	complement(20623235..20698890)	-	-	0,188027316
THUMPD1	16	complement(20702816..20742084)	3,39	4,22	-0,095282632
ERI2	16	complement(20780193..20900349)	0,62	1,08	-0,580150755
REXO5	16	20806429..20849665	1,83	3,33	-0,642563754
DCUN1D3	16	complement(20854925..20900358)	6,78	4,89	0,692986824
LYRM1	16	20899868..20925006	2,43	4,06	-0,516628816
DNAH3	16	complement(20933111..21159441)	0,00	0,00	-0,03031053

LDAF1	16	21158377..21180616	3,02	6,06	-0,782594067
ZP2	16	complement(21197450..21214510)	-	-	0,188027316
ANKS4B	16	21233699..21253850	0,01	0,00	2,399873901
CRYM	16	complement(21238874..21303083)	-	-	0,188027316
NPIPB3	16	complement(21402237..21448567)	5,87	9,68	-0,497650708
METTL9	16	21597218..21657471	12,57	17,50	-0,253569949
IGSF6	16	complement(21639550..21652608)	0,23	0,15	0,839565393
OTOA	16	21663968..21761935	0,02	0,00	2,399873901
NPIPB4	16	complement(21834582..21880827)	0,74	0,87	0,002468526
UQCRC2	16	21953288..21983660	39,48	49,29	-0,097019362
PDZD9	16	complement(21983865..22001116)	0,09	0,16	-0,659786428
MOSMO	16	22007638..22087534	2,61	2,83	0,105655893
VWA3A	16	22092538..22156964	0,01	0,01	0,221270597
SDR42E2	16	22162492..22192208	0,03	0,05	-0,581183184
EEF2K	16	22206278..22288738	9,32	8,44	0,366982002
POLR3E	16	22297375..22335101	11,99	14,68	-0,069369572
CDR2	16	complement(22345936..22437165)	16,01	13,01	0,52199729
NPIPB5	16	22479121..22536535	7,74	10,28	-0,186615401
HS3ST2	16	22814162..22916338	-	-	#N/D
USP31	16	complement(23061406..23149452)	2,54	4,62	-0,641796635
gene:ENSG000	16	23061767..23062232	-	-	#N/D
SCNN1G	16	23182745..23216883	-	-	0,188027316
SCNN1B	16	23278231..23381294	-	-	0,188027316
COG7	16	complement(23388493..23453189)	9,18	8,34	0,361188284
RN7SKP23	16	23412380..23416680	0,60	0,29	1,273956174
GGA2	16	complement(23463542..23521995)	10,34	11,85	0,026774796
EARS2	16	complement(23520754..23557731)	10,76	7,50	0,743979458
UBFD1	16	23557721..23574389	24,68	24,33	0,243712366
NDUFAB1	16	complement(23581014..23596316)	10,48	11,95	0,033889882
PALB2	16	complement(23603160..23641321)	2,39	3,58	-0,362496227
DCTN5	16	23641466..23677472	7,57	5,24	0,752506594
PLK1	16	23677656..23690367	57,66	79,90	-0,247543697
ERN2	16	complement(23690310..23713226)	0,03	0,06	-0,892557502
gene:ENSG000	16	complement(23696665..23697314)	0,02	-	3,068695355
gene:ENSG000	16	complement(23753689..23755939)	-	-	#N/D
CHP2	16	23755026..23758935	-	-	0,188027316
PRKCB	16	23835983..24220611	-	-	0,188027316
gene:ENSG000	16	23962951..23963351	-	-	#N/D
gene:ENSG000	16	24098239..24099971	-	-	0,188027316
gene:ENSG000	16	24208105..24208478	-	-	#N/D
CACNG3	16	24256335..24362412	-	-	#N/D
RBBP6	16	24537693..24572863	5,35	7,18	-0,199995153
TNRC6A	16	24610209..24827632	8,81	9,21	0,159162658
SLC5A11	16	24845841..24911628	0,02	0,03	-0,291161992
ARHGAP17	16	complement(24919389..25015666)	9,10	13,36	-0,331327449
LCMT1	16	25111731..25178231	11,60	12,15	0,156017246
AQP8	16	25215731..25228932	-	0,01	-2,89252005
ZKSCAN2	16	complement(25236001..25257845)	0,88	2,54	-1,29638018

HS3ST4	16	25691959..26137685	-	-	0,188027316
C16orf82	16	27066927..27069166	-	-	0,188027316
KDM8	16	27203508..27221768	0,79	1,26	-0,457335203
NSMCE1	16	complement(27224994..27268772)	13,02	19,39	-0,351137688
IL4R	16	27313668..27364778	6,63	14,21	-0,877401993
IL21R	16	27402174..27452042	0,03	0,01	1,520939857
GTF3C1	16	complement(27459555..27549913)	18,90	22,15	-0,005656779
KATNIP	16	27550133..27780344	3,35	4,17	-0,091701449
GSG1L	16	complement(27787528..28063714)	0,00	-	3,967268276
XPO6	16	complement(28097976..28211965)	30,97	39,44	-0,125721981
SBK1	16	28259246..28323849	0,02	0,17	-2,569197718
NPIPB6	16	complement(28342517..28363508)	0,41	0,29	0,718233243
EIF3CL	16	complement(28379579..28403841)	9,73	7,93	0,518357863
NPIPB7	16	complement(28456329..28472336)	0,49	0,56	0,02917873
gene:ENSG000	16	complement(28456371..28492098)	0,16	0,26	-0,477653454
CLN3	16	complement(28474111..28495575)	2,45	2,28	0,32476978
APOBR	16	28494643..28498964	0,01	0,01	-0,429837071
IL27	16	complement(28499362..28512051)	-	-	#N/D
NUPR1	16	complement(28532708..28539008)	2,37	7,96	-1,525327681
SGF29	16	28553915..28591790	3,66	6,92	-0,69582498
SULT1A2	16	complement(28591943..28597050)	-	0,01	-2,89252005
SULT1A1	16	complement(28605196..28610044)	0,17	0,29	-0,53063305
NPIPB8	16	28637654..28658744	0,18	0,07	1,571459608
EIF3C	16	28688558..28735727	88,59	#####	-0,537467328
NPIPB9	16	28751787..28772864	0,05	0,06	0,097868349
ATXN2L	16	28822999..28837237	40,72	56,35	-0,245493144
TUFM	16	complement(28842411..28846348)	77,86	96,07	-0,080081058
SH2B1	16	28846600..28874212	10,44	8,93	0,449254146
ATP2A1	16	28878405..28904466	1,18	0,81	0,766247112
RABEP2	16	complement(28904421..28936526)	2,37	3,24	-0,225058454
CD19	16	28931965..28939342	-	-	0,188027316
NFATC2IP	16	28950807..28967092	5,99	6,26	0,159327453
SPNS1	16	28974221..28984548	17,12	15,37	0,378268893
LAT	16	28984826..28990784	1,13	0,58	1,194999925
gene:ENSG000	16	29204633..29205855	0,04	0,17	-1,997339153
NPIPB11	16	complement(29381310..29406711)	0,70	0,91	-0,148361462
BOLA2-SMG1P	16	complement(29443230..29454651)	6,58	6,56	0,229081034
BOLA2	16	complement(29453590..29454351)	33,31	19,29	1,011013955
SLX1B	16	29454533..29458219	13,33	14,52	0,099585919
SULT1A4	16	29459913..29464966	0,64	1,23	-0,715668296
NPIPB12	16	complement(29483534..29506408)	2,49	3,52	-0,278643635
gene:ENSG000	16	complement(29595130..29596137)	0,81	1,07	-0,167343859
SPN	16	29662979..29670876	0,00	-	3,967268276
QPRT	16	29663279..29698699	0,06	0,07	-0,038353104
C16orf54	16	complement(29742463..29745990)	-	-	0,188027316
ZG16	16	29778256..29782973	0,00	-	3,068695355
KIF22	16	29790727..29805385	23,16	25,46	0,086524664
MAZ	16	29806106..29811164	58,55	68,68	-0,006981084

PRRT2	16	29811382..29815892	3,48	2,49	0,705024543
gene:ENSG000	16	29812261..29820092	0,87	1,74	-0,775655592
PAGR1	16	29816152..29822489	7,88	7,74	0,248676835
gene:ENSG000	16	29817239..29830627	5,37	4,63	0,436081075
MVP	16	29820394..29848039	33,40	26,30	0,567622696
CDIPT	16	complement(29858357..29863414)	26,25	28,64	0,097386811
SEZ6L2	16	complement(29871159..29899550)	13,21	14,75	0,063633898
ASPHD1	16	29900375..29919864	13,55	8,74	0,856063234
KCTD13	16	complement(29905012..29926236)	2,56	3,38	-0,175742591
gene:ENSG000	16	complement(29913144..29915337)	1,53	1,73	0,049828775
TMEM219	16	29940885..29973050	13,30	14,95	0,054236386
TAOK2	16	29973868..29992261	11,07	7,42	0,800717144
HIRIP3	16	complement(29992330..29996074)	4,24	6,67	-0,432581336
INO80E	16	29995715..30005793	13,45	17,36	-0,145463976
DOC2A	16	complement(30005514..30023270)	0,17	0,16	0,24840349
C16orf92	16	30023198..30027736	0,01	-	3,068695355
TLCD3B	16	complement(30024427..30052978)	0,07	0,07	0,091002416
gene:ENSG000	16	30053123..30070414	4,77	4,91	0,180371299
ALDOA	16	30064164..30070457	574,94	#####	1,232437712
PPP4C	16	30075978..30085376	51,05	51,03	0,223522648
TBX6	16	complement(30085793..30091924)	0,29	0,42	-0,32324035
YPEL3	16	complement(30092314..30096915)	1,90	1,21	0,873440082
GDPD3	16	complement(30104810..30113537)	1,54	1,11	0,698389003
MAPK3	16	complement(30114105..30123506)	19,52	15,73	0,534198955
CORO1A	16	30182827..30189076	0,86	0,54	0,899470639
BOLA2B	16	complement(30192932..30194306)	8,70	8,02	0,340087022
SLX1A	16	30193875..30197561	14,07	15,32	0,099463213
SULT1A3	16	30199228..30204310	14,96	15,31	0,190143061
NPIPB13	16	complement(30222897..30255268)	3,88	5,14	-0,182037387
CD2BP2	16	complement(30350773..30355308)	14,98	15,34	0,188568941
TBC1D10B	16	complement(30357102..30370494)	21,04	18,54	0,405463517
MYL11	16	30370934..30377991	0,05	0,05	0,278120973
ZNF48	16	30378106..30400108	1,45	3,32	-0,97625174
SEPTIN1	16	complement(30378135..30395991)	0,60	0,43	0,70050922
gene:ENSG000	16	complement(30379763..30380110)	0,59	0,67	0,038295137
ZNF771	16	30407414..30431108	0,57	0,76	-0,19072917
DCTPP1	16	complement(30423615..30430030)	18,65	22,63	-0,056254547
SEPHS2	16	complement(30443631..30445874)	8,35	14,81	-0,603483095
ITGAL	16	30472658..30523567	0,01	0,02	-1,133870313
ZNF768	16	complement(30524004..30526821)	11,95	19,77	-0,50277679
gene:ENSG000	16	complement(30525923..30534852)	-	0,22	-7,418882588
gene:ENSG000	16	30526918..30528294	0,52	0,95	-0,65318375
ZNF747	16	complement(30530367..30535347)	2,36	4,81	-0,80461017
ZNF764	16	complement(30553764..30558374)	2,65	2,80	0,143270882
ZNF688	16	complement(30569672..30572734)	0,54	1,36	-1,107336659
ZNF785	16	complement(30573740..30585769)	4,09	2,96	0,692708822
ZNF689	16	complement(30602558..30624012)	3,50	5,58	-0,448336403
gene:ENSG000	16	complement(30610211..30610351)	-	-	#N/D

PRR14	16	30650717..30656413	8,33	9,40	0,049325352
FBRS	16	30658431..30670810	22,17	20,12	0,3632107
SRCAP	16	30698209..30741409	7,83	10,98	-0,264902685
gene:ENSG000	16	30704067..30745169	3,91	5,35	-0,229849557
TMEM265	16	30740642..30745196	-	0,01	-2,89252005
PHKG2	16	30748293..30761176	4,75	2,59	1,096016344
CFAP119	16	complement(30757423..30762221)	0,75	0,35	1,314214121
RNF40	16	30761745..30776307	16,77	14,24	0,459299488
gene:ENSG000	16	complement(30773532..30776033)	2,79	1,58	1,043789824
ZNF629	16	complement(30778456..30787205)	5,19	7,22	-0,254494148
BCL7C	16	complement(30833626..30894302)	5,41	5,98	0,077859787
CTF1	16	30896614..30903547	0,03	-	4,913749003
FBXL19	16	30923055..30948783	9,38	9,94	0,140031828
ORAI3	16	30949068..30956461	5,27	4,31	0,513626527
SETD1A	16	30957294..30984664	7,87	8,51	0,109909454
gene:ENSG000	16	complement(30984630..30988270)	0,35	0,54	-0,393361075
HSD3B7	16	30985207..30989147	1,48	2,53	-0,547578234
STX1B	16	complement(30989256..31010638)	0,11	0,21	-0,652949869
STX4	16	31032889..31042975	10,41	8,40	0,533026614
ZNF668	16	complement(31060843..31074240)	4,79	4,96	0,171288512
ZNF646	16	31074422..31084196	8,39	7,21	0,440975886
PRSS53	16	complement(31083437..31089628)	3,27	0,60	2,662403836
gene:ENSG000	16	complement(31083439..31094956)	0,47	0,79	-0,51165561
VKORC1	16	complement(31090842..31095980)	32,15	22,33	0,748942763
gene:ENSG000	16	31093727..31095944	0,70	0,47	0,806271582
BCKDK	16	31106107..31112791	13,94	12,84	0,341124838
KAT8	16	31114489..31131393	6,62	7,46	0,050913969
PRSS8	16	complement(31131433..31135727)	0,24	0,16	0,853990683
PRSS36	16	complement(31138926..31150083)	0,10	0,26	-1,088429322
FUS	16	31180138..31196963	17,03	30,19	-0,60266328
PYCARD	16	complement(31201486..31203450)	0,15	0,24	-0,385430895
TRIM72	16	31214119..31231537	0,01	0,01	0,438394362
PYDC1	16	complement(31215962..31217135)	-	-	0,188027316
ITGAM	16	31259967..31332892	0,10	0,14	-0,29727385
ITGAX	16	31355134..31382999	0,73	0,63	0,435606995
ITGAD	16	31393335..31426505	0,03	0,03	0,415033091
COX6A2	16	complement(31427731..31428360)	-	-	#N/D
ZNF843	16	complement(31432593..31443160)	0,10	0,28	-1,226038592
ARMC5	16	31458080..31467166	2,87	3,06	0,131248886
gene:ENSG000	16	complement(31470204..31472108)	0,84	0,47	1,058776371
TGFB1I1	16	31471585..31477960	11,80	9,38	0,553353676
SLC5A2	16	31483002..31490860	0,26	0,17	0,810092062
RUSF1	16	complement(31489471..31509309)	40,83	31,39	0,602349682
AHSP	16	31527900..31528803	0,02	-	3,068695355
gene:ENSG000	16	complement(31566360..31567000)	-	-	#N/D
KRBOX5	16	31713229..31794869	0,42	0,44	0,164963188
ZNF267	16	31873807..31917357	1,34	1,49	0,071202111
IGHV3OR16-9	16	32066065..32066358	-	-	#N/D

gene:ENSG000	16	32185624..32186311	-	-	0,188027316
TP53TG3D	16	32253286..32255928	-	-	0,188027316
TP53TG3	16	complement(32673528..32676165)	0,12	0,24	-0,736859784
gene:ENSG000	16	32781883..32784138	-	-	0,188027316
gene:ENSG000	16	32786394..32787379	-	-	0,188027316
gene:ENSG000	16	32787962..32788944	-	-	0,188027316
IGHV2OR16-5	16	32847713..32848156	-	-	#N/D
IGHV3OR16-10	16	32995048..32995505	-	-	#N/D
IGHV3OR16-8	16	33009175..33009620	-	-	#N/D
TP53TG3C	16	33194226..33196858	0,11	0,04	1,745345563
TP53TG3E	16	33304306..33306945	0,03	-	5,224730805
TP53TG3B	16	33360841..33363478	0,07	0,15	-0,974683893
TP53TG3F	16	33459612..33462249	-	-	0,188027316
IGHV3OR16-12	16	33802764..33803217	0,02	-	3,068695355
IGHV3OR16-13	16	33827214..33827661	-	-	#N/D
IGHV3OR16-17	16	complement(33844784..33845229)	-	-	#N/D
CCNYL1B	16	complement(34351499..34381110)	-	-	0,188027316
gene:ENSG000	16	34941977..34943880	-	-	0,188027316
gene:ENSG000	16	35148636..35154366	-	-	0,188027316
gene:ENSG000	16	35903117..35904916	-	-	#N/D
SHCBP1	16	complement(46578591..46621379)	17,46	10,32	0,981820022
VPS35	16	complement(46656132..46689518)	13,84	11,20	0,528629731
gene:ENSG000	16	complement(46673443..46673927)	1,02	0,64	0,894599361
ORC6	16	46689643..46698394	14,97	11,12	0,651832046
MYLK3	16	complement(46702282..46790407)	0,05	0,02	1,254956342
C16orf87	16	complement(46796603..46831180)	1,98	1,41	0,714550034
GPT2	16	46884362..46931289	12,56	16,12	-0,136678864
DNAJA2	16	complement(46955362..46973674)	15,95	11,20	0,733130964
NETO2	16	complement(47077703..47143945)	2,82	4,07	-0,305367821
ITFG1	16	complement(47154387..47464149)	3,35	5,10	-0,384653627
gene:ENSG000	16	47218026..47218768	-	-	0,188027316
PHKB	16	47461123..47701523	3,32	5,96	-0,620835992
ABCC12	16	complement(48080882..48156018)	-	0,00	-2,89252005
ABCC11	16	complement(48165773..48247568)	0,04	0,02	0,827824525
LONP2	16	48244300..48363122	10,49	11,92	0,037842807
gene:ENSG000	16	complement(48343755..48346058)	0,70	0,53	0,62316109
SIAH1	16	complement(48356364..48448402)	1,98	2,80	-0,27933631
N4BP1	16	complement(48538726..48620148)	3,21	5,44	-0,53981395
CBLN1	16	complement(49277917..49281838)	0,00	-	3,068695355
gene:ENSG000	16	49297143..49299762	-	-	0,188027316
C16orf78	16	49373804..49399431	-	-	#N/D
ZNF423	16	complement(49487524..49857919)	0,18	0,05	1,964977367
gene:ENSG000	16	49711273..49711914	-	-	0,188027316
gene:ENSG000	16	49900019..49900524	-	-	#N/D
CNEP1R1	16	50024410..50037088	3,34	2,79	0,483544527
HEATR3	16	50065967..50107272	1,30	2,76	-0,865431376
gene:ENSG000	16	50072862..50074986	0,16	0,13	0,49798677
TENT4B	16	50152911..50235310	1,44	1,55	0,109308646

ADCY7	16	50246137..50318135	24,59	24,67	0,218338937
BRD7	16	complement(50313487..50368988)	3,95	5,52	-0,25892934
NKD1	16	50548396..50649249	0,15	0,06	1,490243967
gene:ENSG000	16	50555355..50558719	0,01	0,02	-0,742377908
SNX20	16	complement(50666300..50681353)	-	-	0,188027316
NOD2	16	50693588..50733077	0,06	0,08	-0,294064518
CYLD	16	50742050..50801935	4,82	5,17	0,123858646
SALL1	16	complement(51135982..51152334)	1,81	3,86	-0,868242057
gene:ENSG000	16	51176066..51178898	0,02	0,02	-0,345401666
HNRNPA1L3	16	51553436..51647132	0,50	0,55	0,077486313
gene:ENSG000	16	complement(52081568..52082096)	-	-	#N/D
TOX3	16	complement(52436417..52547802)	-	-	0,188027316
gene:ENSG000	16	complement(52436772..52437474)	-	-	#N/D
CHD9NB	16	complement(53035690..53052897)	0,09	0,18	-0,808768467
CHD9	16	53054991..53329150	7,15	5,86	0,509803729
gene:ENSG000	16	53227146..53227694	-	0,02	-2,89252005
gene:ENSG000	16	53310797..53311989	0,20	0,26	-0,164183768
gene:ENSG000	16	53389886..53390563	-	-	#N/D
gene:ENSG000	16	53391089..53392473	-	-	#N/D
RBL2	16	53433977..53491648	38,07	27,10	0,713438276
gene:ENSG000	16	complement(53478957..53481550)	0,46	0,24	1,123884327
gene:ENSG000	16	complement(53487607..53489943)	0,33	0,27	0,543200523
AKTIP	16	complement(53491040..53504411)	4,52	4,84	0,122505983
RPGRIP1L	16	complement(53598153..53703938)	2,52	1,74	0,758960993
FTO	16	53701692..54158512	13,15	9,05	0,762120349
gene:ENSG000	16	53991012..53994454	0,68	0,60	0,4142419
IRX3	16	complement(54283304..54286787)	6,56	4,98	0,621505443
IRX5	16	54930865..54934485	6,15	3,25	1,14357553
IRX6	16	55324203..55330756	0,02	0,00	2,399873901
MMP2	16	55389700..55506691	55,56	68,88	-0,086898795
gene:ENSG000	16	55486618..55488777	0,19	0,41	-0,877307165
LPCAT2	16	55509072..55586666	0,01	0,01	-0,345401666
CAPNS2	16	55566684..55567687	-	-	0,188027316
SLC6A2	16	55655988..55707645	-	-	0,188027316
gene:ENSG000	16	55704119..55706422	-	-	#N/D
CES1	16	complement(55802851..55833337)	1,57	1,28	0,518141435
CES5A	16	complement(55846154..55956031)	-	-	0,188027316
gene:ENSG000	16	56108980..56109970	-	-	#N/D
GNAO1	16	56189660..56357444	3,26	6,45	-0,760758322
gene:ENSG000	16	56306845..56308335	0,04	0,16	-1,854949791
AMFR	16	complement(56361452..56425545)	96,60	#####	0,118076264
NUDT21	16	complement(56429133..56452199)	36,04	40,86	0,041916787
gene:ENSG000	16	complement(56430556..56501497)	10,33	9,47	0,347380385
OGFOD1	16	56451521..56479104	28,19	29,09	0,177876519
BBS2	16	complement(56465640..56582667)	9,68	11,03	0,034600182
MT4	16	56565073..56568957	-	-	#N/D
MT3	16	56589074..56591088	-	0,01	-2,89252005
MT2A	16	56608584..56609497	#######	#####	0,917162939

MT1E	16	56625475..56627112	10,18	27,03	-1,185755208
MT1M	16	56632659..56633981	1,01	1,62	-0,454224829
MT1A	16	56638666..56640087	4,17	1,18	2,042698141
MT1B	16	56651886..56653204	-	0,40	-6,312013662
MT1F	16	56657731..56660698	0,02	0,06	-0,923955849
MT1G	16	complement(56666730..56668065)	0,74	0,13	2,726985196
MT1H	16	56669814..56671129	-	0,04	-3,804623889
MT1X	16	56682470..56684196	312,46	94,33	1,950914677
NUP93	16	56730118..56850286	19,77	25,46	-0,141897907
SLC12A3	16	56865207..56915850	-	0,01	-4,758582517
HERPUD1	16	56932142..56944864	506,96	49,41	3,582123322
CETP	16	56961923..56983845	0,05	0,37	-2,604455062
NLRC5	16	56989485..57083531	2,76	11,83	-1,878240455
CPNE2	16	57092583..57148369	8,76	19,82	-0,954437622
gene:ENSG000	16	complement(57140896..57144586)	0,44	0,91	-0,82253066
gene:ENSG000	16	57142652..57144936	0,17	0,31	-0,583781189
PSME3IP1	16	complement(57152466..57186116)	35,99	38,61	0,121941737
RSPRY1	16	57186137..57240469	11,54	13,93	-0,048305787
ARL2BP	16	57245259..57253635	55,94	70,08	-0,10184985
PLLP	16	complement(57248547..57284672)	1,77	1,69	0,293302664
CCL22	16	57358783..57366189	0,01	0,03	-1,54862452
CX3CL1	16	57372477..57385044	-	0,02	-5,545746556
CCL17	16	57404767..57416063	-	0,04	-3,804623889
CIAPIN1	16	complement(57428187..57447420)	47,78	55,48	0,007436781
COQ9	16	57447425..57461270	36,68	40,10	0,094467355
POLR2C	16	57462660..57472009	122,94	92,23	0,637732829
DOK4	16	complement(57471922..57487327)	7,44	6,12	0,504611112
CCDC102A	16	complement(57512181..57536571)	2,20	4,05	-0,655331687
ADGRG5	16	57542643..57591681	0,02	0,12	-2,593916734
ADGRG1	16	57610652..57665580	11,92	27,55	-0,985540245
ADGRG3	16	57668277..57689378	0,08	0,34	-1,790280788
DRC7	16	57694793..57731805	0,01	0,04	-1,151227584
KATNB1	16	57735739..57757244	27,43	43,43	-0,439788511
KIFC3	16	complement(57758217..57863053)	47,33	58,90	-0,092313827
CNGB1	16	complement(57882340..57971128)	0,05	0,17	-1,557298283
SPMIP8	16	57976435..57988116	0,29	0,04	3,030543327
ZNF319	16	complement(57994676..58000672)	5,74	12,57	-0,908180107
USB1	16	57999546..58021618	22,13	32,51	-0,331687768
MMP15	16	58025754..58046901	4,90	9,16	-0,679331503
CFAP20	16	complement(58113592..58129381)	55,64	58,09	0,16095891
CSNK2A2	16	complement(58157907..58198106)	37,00	44,12	-0,030816568
CCDC113	16	58231157..58283836	5,20	8,94	-0,559477392
PRSS54	16	complement(58279997..58295047)	0,04	-	5,697545031
GINS3	16	58295080..58406147	9,52	16,65	-0,582856423
NDRG4	16	58462846..58513628	1,31	1,92	-0,332057826
SETD6	16	58515479..58523842	3,09	3,99	-0,145323466
CNOT1	16	complement(58519951..58629885)	102,09	78,02	0,611069483
SLC38A7	16	complement(58665109..58684770)	4,27	4,67	0,094944381

gene:ENSG000	16	complement(58705799..58706297)	0,32	0,36	0,059935958
GOT2	16	complement(58707131..58734342)	130,06	#####	-0,101342579
CDH8	16	complement(61647242..62037035)	-	-	0,188027316
gene:ENSG000	16	62342420..62342557	-	-	#N/D
gene:ENSG000	16	63397707..63397946	-	-	0,188027316
CDH11	16	complement(64943753..65126112)	0,97	2,77	-1,282250903
gene:ENSG000	16	complement(64981123..64983227)	0,03	0,07	-1,019619711
CDH5	16	66366690..66404784	0,94	0,11	3,311244532
BEAN1	16	66427295..66493529	0,64	0,64	0,215547671
gene:ENSG000	16	complement(66469812..66517312)	0,17	0,37	-0,892557502
TK2	16	complement(66508003..66552544)	3,56	4,92	-0,243264101
CKLF	16	66552563..66566251	14,64	17,75	-0,055079463
CKLF-CMTM1	16	66552587..66579135	0,66	1,65	-1,088263655
CMTM1	16	66566393..66579137	1,16	2,47	-0,860482696
CMTM2	16	66579448..66588275	-	0,11	-5,902279478
CMTM3	16	66603874..66613892	9,20	10,00	0,102494469
CMTM4	16	complement(66614750..66696743)	5,48	8,44	-0,400488989
DYNC1LI2	16	complement(66720893..66751609)	22,96	18,61	0,5264088
TERB1	16	complement(66754640..66801620)	0,00	0,02	-1,877914477
NAE1	16	complement(66802875..66873256)	4,76	10,84	-0,964385032
CA7	16	66844414..66854153	0,16	0,18	0,014515821
PDP2	16	66878589..66895754	5,81	4,19	0,695689166
CDH16	16	complement(66908122..66918917)	-	0,03	-5,902279478
RRAD	16	complement(66921685..66925535)	0,75	6,02	-2,773789701
CIAO2B	16	complement(66932065..66934423)	55,75	64,37	0,015826333
CES2	16	66934444..66945096	6,47	9,92	-0,393228389
gene:ENSG000	16	complement(66944660..66945096)	0,57	2,04	-1,618824296
CES3	16	66961245..66975149	0,16	0,64	-1,75064956
CES4A	16	66988589..67010417	0,00	0,01	-0,923955849
gene:ENSG000	16	complement(67006672..67009481)	0,01	0,01	-0,579815015
CBFB	16	67028984..67101058	7,38	9,22	-0,097126101
PHAF1	16	67109941..67148544	7,83	9,25	-0,016829034
B3GNT9	16	complement(67148104..67150998)	17,57	7,31	1,488101288
TRADD	16	complement(67154185..67159909)	5,09	3,78	0,651586112
FBXL8	16	67159932..67164570	2,56	3,19	-0,090792919
gene:ENSG000	16	67163385..67165815	2,93	3,62	-0,079503747
HSF4	16	67164681..67169945	4,55	5,10	0,058442339
NOL3	16	67170154..67175737	5,03	6,32	-0,107950589
MATCAP1	16	complement(67175599..67184040)	5,81	5,38	0,33553751
EXOC3L1	16	complement(67184379..67190185)	0,08	0,11	-0,222756867
E2F4	16	67192155..67198918	39,67	44,16	0,068352464
ELMO3	16	67199111..67204029	3,17	1,51	1,292196025
gene:ENSG000	16	complement(67205175..67206849)	1,74	1,29	0,65724029
TMEM208	16	67227103..67229278	20,58	14,03	0,776168096
FHOD1	16	complement(67229387..67247481)	16,68	17,17	0,180918313
SLC9A5	16	67237683..67272191	2,28	4,03	-0,600318859
PLEKHG4	16	67277510..67289499	8,51	16,73	-0,751762546
KCTD19	16	complement(67289428..67326760)	0,28	0,72	-1,135534734

LRRC36	16	67326798..67385204	0,11	0,50	-1,910903717
TPPP3	16	complement(67389809..67393518)	0,78	1,00	-0,140717889
ZDHHC1	16	complement(67394152..67416833)	2,58	5,09	-0,757229201
HSD11B2	16	67430652..67437553	0,10	0,32	-1,477071699
ATP6V0D1	16	complement(67438014..67481181)	12,15	10,44	0,441731593
AGRP	16	complement(67482571..67483547)	-	0,03	-2,89252005
RIPOR1	16	67518418..67546788	20,25	25,35	-0,101136184
gene:ENSG000	16	complement(67550815..67552935)	0,27	0,34	-0,066675999
CTCF	16	67562467..67639177	5,87	7,27	-0,086381168
CARMIL2	16	67644988..67657569	0,58	2,08	-1,615750979
ACD	16	complement(67657512..67660810)	10,72	15,86	-0,342403353
PARD6A	16	67660946..67662774	0,15	0,31	-0,771743303
ENKD1	16	complement(67662945..67667265)	4,33	5,31	-0,070337146
C16orf86	16	67666814..67668757	0,34	0,33	0,272926936
GFOD2	16	complement(67674531..67719339)	2,83	3,15	0,067519214
RANBP10	16	complement(67723070..67806560)	3,18	3,57	0,055890358
TSNAXIP1	16	67806765..67832148	0,20	0,14	0,694614909
CENPT	16	complement(67828157..67847811)	10,00	7,40	0,656049425
THAP11	16	67842320..67844195	13,00	16,93	-0,157809896
NUTF2	16	67846882..67872567	23,39	24,55	0,153301358
EDC4	16	67873052..67884499	18,19	17,69	0,263210344
NRN1L	16	67884885..67888855	0,35	0,16	1,3196162
PSKH1	16	67893254..67929676	4,52	7,95	-0,590783998
CTRL	16	complement(67927640..67932365)	0,73	0,85	0,013203733
gene:ENSG000	16	complement(67929614..67936017)	0,24	0,41	-0,50374212
PSMB10	16	complement(67934506..67936864)	4,05	5,76	-0,283787479
LCAT	16	complement(67939750..67944131)	3,35	3,09	0,339386134
SLC12A4	16	complement(67943474..67969601)	14,22	11,57	0,519874227
DPEP3	16	complement(67975663..67980549)	-	-	#N/D
DPEP2	16	complement(67987394..68000586)	0,01	0,03	-1,54862452
DUS2	16	67987746..68079320	3,23	5,48	-0,541573304
DPEP2NB	16	complement(68013436..68015911)	0,05	-	5,480421266
DDX28	16	complement(68020916..68023232)	1,81	5,59	-1,402651719
NFATC3	16	68084751..68229259	5,18	6,39	-0,080116281
gene:ENSG000	16	complement(68199841..68200981)	0,06	0,08	-0,253352605
ESRP2	16	complement(68229033..68238102)	0,15	0,27	-0,652949869
PLA2G15	16	68245304..68261058	8,27	7,45	0,373523506
SLC7A6	16	68264516..68301823	5,19	6,84	-0,173177719
SLC7A6OS	16	complement(68284503..68310946)	2,50	2,50	0,224339682
gene:ENSG000	16	complement(68290087..68292790)	1,43	1,68	-0,012312306
PRMT7	16	68310951..68360852	2,18	3,42	-0,425538697
SMPD3	16	complement(68358327..68448508)	0,19	0,33	-0,5545259
gene:ENSG000	16	complement(68367325..68370262)	0,01	0,01	0,366665713
ZFP90	16	68530090..68576072	2,37	3,89	-0,492859555
CDH3	16	68636189..68727468	0,11	0,19	-0,546257624
CDH1	16	68737292..68835537	0,00	0,01	-1,478387936
TANGO6	16	68843531..69085182	2,87	5,32	-0,668590165
HAS3	16	69105653..69118719	2,25	17,43	-2,733039727

CHTF8	16	complement(69118010..69132588)	13,95	14,57	0,160174807
DERPC	16	complement(69118010..69132588)	6,11	10,85	-0,605796254
UTP4	16	69131291..69231130	7,36	13,38	-0,638690234
SNTB2	16	69187147..69309052	9,82	9,43	0,280922884
gene:ENSG000	16	69299682..69322700	0,48	0,32	0,789946792
VPS4A	16	69311336..69326939	21,37	23,29	0,098545101
COG8	16	complement(69320140..69339583)	5,45	7,39	-0,215214092
PDF	16	complement(69326913..69330588)	1,13	1,14	0,205829389
gene:ENSG000	16	complement(69328644..69334871)	2,49	5,05	-0,795484697
gene:ENSG000	16	complement(69335091..69356306)	-	-	0,188027316
gene:ENSG000	16	complement(69335276..69351730)	0,07	0,07	0,278120973
NIP7	16	69337996..69343106	4,91	7,95	-0,472245676
TMED6	16	complement(69343250..69351786)	0,15	0,43	-1,236435222
TERF2	16	complement(69355567..69408571)	5,99	7,40	-0,082597574
CYB5B	16	69398402..69466264	12,41	12,21	0,246378933
NFAT5	16	69565094..69704666	6,65	7,62	0,026695059
NQO1	16	complement(69706996..69726668)	102,93	43,03	1,481297715
NOB1	16	complement(69741871..69754926)	28,46	33,34	-0,005085562
WWP2	16	69762328..69941741	3,24	4,46	-0,23911393
CLEC18A	16	69950705..69964452	0,03	0,10	-1,669701709
PDPR	16	70114332..70162537	11,88	12,13	0,193809318
CLEC18C	16	70173322..70187361	-	-	0,188027316
EXOSC6	16	complement(70246778..70251940)	5,15	7,91	-0,397947193
AARS1	16	complement(70251983..70289707)	49,18	78,16	-0,445277319
DDX19B	16	70289663..70335305	3,25	6,09	-0,684141789
gene:ENSG000	16	70299194..70372582	1,18	0,92	0,58504936
DDX19A	16	70346861..70373383	8,12	9,21	0,041844873
ST3GAL2	16	complement(70375977..70439237)	4,71	6,82	-0,311508885
FCSK	16	70454595..70480274	2,35	3,30	-0,266933707
COG4	16	complement(70480568..70523560)	19,84	17,68	0,389091054
SF3B3	16	70523791..70577670	23,61	35,03	-0,346064473
IL34	16	70579895..70660682	-	0,02	-4,359055975
MTSS2	16	complement(70661204..70686053)	38,73	28,25	0,678197441
gene:ENSG000	16	70661667..70665836	2,52	1,28	1,200157821
VAC14	16	complement(70687439..70801160)	6,10	8,60	-0,27326437
gene:ENSG000	16	70713987..70716890	0,23	0,52	-0,919657005
gene:ENSG000	16	70768356..70768785	0,18	0,15	0,438394362
gene:ENSG000	16	complement(70768967..70770221)	0,22	0,27	-0,039610984
gene:ENSG000	16	70778433..70779145	0,11	0,05	1,15046174
HYDIN	16	complement(70802084..71230722)	0,00	0,00	1,436225396
CMTR2	16	complement(71281389..71289715)	1,09	2,24	-0,816505164
CALB2	16	71358713..71390436	0,94	0,21	2,367888051
TLE7	16	complement(71429980..71442054)	0,01	-	3,068695355
ZNF23	16	complement(71447597..71463095)	0,87	1,90	-0,909407478
gene:ENSG000	16	complement(71447600..71489311)	0,04	-	6,780090526
ZNF19	16	complement(71464555..71565089)	0,05	0,14	-1,247374092
CHST4	16	71525233..71538748	-	-	0,188027316
TAT	16	complement(71565660..71577092)	-	-	#N/D

MARVELD3	16	71626161..71642114	0,02	0,03	-0,489732438
PHLPP2	16	complement(71637835..71724701)	1,19	1,86	-0,41582406
AP1G1	16	complement(71729000..71809201)	10,75	10,16	0,305187239
ATXN1L	16	71808439..71885268	6,59	7,41	0,053757731
IST1	16	71845996..71931199	14,52	15,27	0,150528634
gene:ENSG000	16	71859680..71860676	-	0,08	-5,327865481
ZNF821	16	complement(71859680..71895336)	0,63	0,78	-0,093102665
PKD1L3	16	complement(71929538..72000402)	0,00	0,00	-0,025382928
DHODH	16	72008588..72027664	1,30	1,37	0,145614479
TXNL4B	16	complement(72044289..72094431)	5,26	3,69	0,734776807
HP	16	72054505..72061055	0,04	0,01	2,805678585
HPR	16	72063148..72077246	0,04	0,03	0,533811434
DHX38	16	72093613..72112912	18,81	18,58	0,240978312
PMFBP1	16	complement(72112157..72176878)	0,06	0,05	0,493326738
ZFHX3	16	complement(72782885..73891871)	2,91	4,08	-0,265891632
gene:ENSG000	16	complement(72973374..72973832)	0,14	0,03	2,399873901
gene:ENSG000	16	complement(73005601..73006141)	-	-	0,188027316
gene:ENSG000	16	complement(73015047..73016016)	0,19	0,25	-0,19821929
gene:ENSG000	16	complement(73293866..73294296)	-	-	#N/D
gene:ENSG000	16	74053365..74053965	-	-	#N/D
gene:ENSG000	16	74150520..74151299	-	-	#N/D
PSMD7	16	74296814..74306288	31,88	45,26	-0,282572629
NPIPB15	16	74376306..74392115	4,32	3,57	0,498716084
CLEC18B	16	complement(74408631..74421478)	-	0,05	-5,545746556
GLG1	16	complement(74447427..74607144)	27,85	31,79	0,031947118
RFWD3	16	complement(74621399..74666877)	20,12	24,75	-0,075709937
MLKL	16	complement(74671855..74700960)	2,45	5,42	-0,924628626
FA2H	16	complement(74712955..74774831)	0,03	0,12	-1,909364873
WDR59	16	complement(74871362..75000173)	3,62	5,62	-0,410063336
ZNRF1	16	74999024..75110994	5,07	9,39	-0,667302025
LDHD	16	complement(75111860..75116780)	0,58	0,49	0,469650309
ZFP1	16	75148494..75172236	0,98	1,88	-0,715853118
CTRB2	16	complement(75204103..75207161)	0,02	0,01	0,886720911
CTRB1	16	75218988..75226338	0,20	0,12	0,920688843
BCAR1	16	complement(75228181..75268053)	13,70	11,95	0,420183815
gene:ENSG000	16	complement(75245994..75250077)	2,41	1,95	0,529079292
CFDP1	16	complement(75293698..75433503)	4,05	4,70	0,010896111
gene:ENSG000	16	complement(75412684..75464706)	0,02	0,20	-2,854330803
TMEM170A	16	complement(75443054..75465497)	1,79	2,44	-0,223315754
CHST6	16	complement(75472052..75495445)	0,02	0,02	0,075897498
CHST5	16	complement(75528530..75536108)	0,15	0,27	-0,643677545
gene:ENSG000	16	complement(75528532..75545428)	-	-	0,188027316
TMEM231	16	complement(75536741..75556289)	1,63	3,57	-0,903476887
GABARAPL2	16	75566375..75577881	23,29	21,71	0,324461384
ADAT1	16	complement(75596868..75623300)	4,83	7,87	-0,48174294
KARS1	16	complement(75627474..75648643)	42,14	47,18	0,060002643
TERF2IP	16	75647773..75761872	10,11	11,07	0,093002526
CPHXL2	16	complement(75660227..75677009)	0,06	0,14	-0,916746549

DUXB	16	complement(75693893..75701461)	0,01	-	3,068695355
CPHXL	16	complement(75714131..75726490)	0,02	0,03	-0,579815015
CNTNAP4	16	76277278..76560757	-	-	0,188027316
gene:ENSG000	16	76277288..76819624	-	-	#N/D
gene:ENSG000	16	77104260..77105297	-	-	#N/D
MON1B	16	77190835..77202398	11,03	8,13	0,662521666
SYCE1L	16	77199408..77213215	1,09	0,44	1,540814312
ADAMTS18	16	complement(77247813..77435034)	0,11	0,03	2,361663901
gene:ENSG000	16	77657174..77660570	0,03	0,01	1,893574746
NUDT7	16	77722492..77742260	1,59	1,22	0,600342595
VAT1L	16	77788564..77980107	0,23	0,18	0,597608354
gene:ENSG000	16	77800023..77801714	-	0,01	-2,89252005
CLEC3A	16	78022515..78066761	-	-	0,188027316
WWOX	16	78099400..79212667	1,13	0,98	0,42979439
gene:ENSG000	16	complement(78166717..78168514)	0,04	0,04	0,181652234
gene:ENSG000	16	complement(78525189..78526535)	0,11	0,12	0,067331041
gene:ENSG000	16	complement(78550739..78553296)	0,06	0,08	-0,229465536
gene:ENSG000	16	78872739..78873505	-	0,02	-2,89252005
gene:ENSG000	16	78890231..78892302	-	-	0,188027316
gene:ENSG000	16	complement(78981722..78981843)	-	-	#N/D
gene:ENSG000	16	79016136..79017429	0,04	0,05	-0,034419864
MAF	16	complement(79585843..79600737)	0,01	-	5,224730805
gene:ENSG000	16	complement(79675685..79677715)	0,01	-	3,068695355
DYNLRB2	16	80540734..80550811	-	-	0,188027316
CDYL2	16	complement(80597907..80804598)	0,09	0,04	1,538976189
gene:ENSG000	16	complement(80828736..81006894)	0,10	0,12	0,019012699
CMC2	16	complement(80966448..81020270)	1,98	2,07	0,158557271
gene:ENSG000	16	complement(80982319..80984094)	0,53	0,34	0,86242405
CENPN	16	81006552..81033114	6,97	8,86	-0,124163188
gene:ENSG000	16	complement(81022316..81096284)	0,15	0,22	-0,36833206
ATMIN	16	81035842..81047350	2,49	4,54	-0,644336416
C16orf46	16	complement(81053497..81077267)	-	0,06	-6,052163584
gene:ENSG000	16	complement(81053587..81096296)	0,78	0,92	-0,015330227
GCSH	16	complement(81081945..81096395)	1,35	2,40	-0,607318156
PKD1L2	16	complement(81100875..81220370)	0,04	0,07	-0,533621475
BCO1	16	81238689..81291142	0,00	-	3,068695355
gene:ENSG000	16	complement(81261924..81264091)	0,01	0,01	0,886720911
GAN	16	81314944..81390809	2,53	2,85	0,053094982
CMIP	16	81444808..81711762	15,06	14,88	0,240925405
gene:ENSG000	16	complement(81463892..81464859)	1,37	1,18	0,441845416
gene:ENSG000	16	81517824..81520617	0,68	0,74	0,105737115
gene:ENSG000	16	complement(81617038..81617543)	0,40	0,38	0,299198032
gene:ENSG000	16	81618505..81620563	0,48	0,53	0,073476116
gene:ENSG000	16	81630193..81632038	0,61	0,58	0,301825876
PLCG2	16	81779279..81962685	1,30	1,18	0,365725495
SDR42E1	16	complement(81988855..82011481)	0,01	0,01	0,53887001
HSD17B2	16	82035004..82098534	0,20	-	10,3432491
MPHOSPH6	16	complement(82147798..82170224)	1,53	2,08	-0,218975841

CDH13	16	82626965..83800640	-	0,02	-6,630750384
gene:ENSG000	16	82727474..82727694	-	-	#N/D
HSBP1	16	83719311..83819737	4,52	4,53	0,219712899
MLYCD	16	83899115..83951445	0,54	0,58	0,106659857
OSGIN1	16	83931311..83966332	6,64	1,94	1,995097458
NECAB2	16	83968244..84002776	0,06	0,13	-0,841031413
SLC38A8	16	complement(84009667..84042795)	-	-	#N/D
MBTPS1	16	complement(84053761..84116942)	26,33	37,71	-0,295490715
HSDL1	16	complement(84122141..84145192)	2,78	3,64	-0,169249453
DNAAF1	16	84145287..84178767	0,04	0,01	1,655502569
TAF1C	16	complement(84177847..84187070)	9,98	12,97	-0,154716869
ADAD2	16	84191138..84197168	0,01	0,01	0,886720911
KCNG4	16	complement(84218657..84240012)	-	-	0,188027316
WFDC1	16	84294846..84329844	-	0,03	-4,758582517
ATP2C2	16	84368527..84464187	0,47	0,54	0,00754756
MEAK7	16	complement(84476355..84554033)	12,67	15,83	-0,097363134
COTL1	16	complement(84565596..84618078)	128,29	#####	-0,14107355
KLHL36	16	84648511..84667686	9,59	9,56	0,227623012
USP10	16	84699986..84779922	20,21	29,60	-0,327465508
CRISPLD2	16	84819985..84920768	1,54	0,69	1,368334318
ZDHHC7	16	complement(84974175..85011535)	14,71	19,33	-0,170388249
KIAA0513	16	85027782..85094230	3,38	2,79	0,500785634
CIBAR2	16	complement(85098358..85112472)	0,11	0,01	4,037428367
GSE1	16	85169525..85676204	11,07	11,33	0,190081525
GINS2	16	complement(85676198..85690073)	5,20	5,39	0,170084542
C16orf74	16	complement(85690084..85751129)	4,32	6,54	-0,373674325
EMC8	16	complement(85771758..85799608)	12,40	15,39	-0,088191428
COX4I1	16	85798633..85807068	47,13	55,10	-0,002155267
gene:ENSG000	16	85859521..85862012	-	-	#N/D
IRF8	16	85899116..85922606	-	-	#N/D
gene:ENSG000	16	86035627..86036419	-	-	#N/D
FOXF1	16	86510527..86515422	59,34	34,97	0,985944775
MTHFSD	16	complement(86530178..86555235)	8,73	3,90	1,387797047
FLJ30679	16	86555320..86557299	0,19	0,20	0,139907968
FOXC2	16	86566829..86569728	2,77	3,92	-0,277779515
FOXL1	16	86576368..86583478	0,52	0,46	0,404248759
gene:ENSG000	16	86595189..86597639	0,20	0,25	-0,1026997
C16orf95	16	complement(87083562..87317420)	1,73	2,97	-0,556515379
gene:ENSG000	16	87296949..87297633	0,05	0,08	-0,429837071
gene:ENSG000	16	complement(87302876..87334273)	0,58	0,60	0,172159388
FBXO31	16	complement(87326987..87392142)	6,14	5,22	0,458419422
MAP1LC3B	16	87383953..87404779	14,10	11,78	0,481902857
ZCCHC14	16	complement(87406246..87493024)	7,80	9,62	-0,079812127
JPH3	16	87601835..87698156	2,55	6,42	-1,109417353
KLHDC4	16	complement(87696485..87765992)	1,99	3,83	-0,72478552
SLC7A5	16	complement(87830023..87869507)	168,93	#####	-0,050903786
CA5A	16	complement(87881546..87936580)	-	-	0,188027316
BANP	16	87949244..88077318	4,19	5,31	-0,118857405

ZNF469	16	88382959..88440757	0,57	2,43	-1,877863123
ZFPM1	16	88453280..88537031	0,90	1,22	-0,217427013
ZC3H18	16	88570403..88631964	13,77	18,37	-0,192635822
IL17C	16	88638572..88640468	0,04	0,01	1,833201638
CYBA	16	complement(88643275..88651083)	0,26	0,02	4,044563405
MVD	16	complement(88651935..88663161)	19,89	5,38	2,109005443
SNAI3	16	complement(88677688..88686507)	0,63	0,39	0,929608569
RNF166	16	complement(88696499..88706408)	3,97	5,31	-0,19711851
CTU2	16	88706483..88715396	12,36	8,03	0,845029521
PIEZO1	16	complement(88715338..88785220)	27,63	29,75	0,116058717
CDT1	16	88803789..88809258	23,52	21,96	0,321647871
APRT	16	complement(88809339..88811937)	52,35	53,44	0,193285329
GALNS	16	complement(88813734..88856970)	5,47	5,21	0,29568635
TRAPPC2L	16	88856220..88862686	3,62	3,16	0,422796756
PABPN1L	16	complement(88863333..88866660)	0,06	0,07	-0,036228841
CBFA2T3	16	complement(88874858..88977207)	0,05	0,02	1,420755175
ACSF3	16	89088375..89164121	2,57	3,75	-0,324527871
CDH15	16	89171748..89195492	0,64	0,05	3,760346638
SLC22A31	16	complement(89195761..89201651)	3,77	2,18	1,011699307
ZNF778	16	89217703..89237141	1,72	1,27	0,668607614
ANKRD11	16	complement(89267630..89490561)	14,35	16,64	0,009677673
gene:ENSG000	16	complement(89317046..89418292)	-	-	#N/D
SPG7	16	89490719..89557766	9,16	6,98	0,615873166
RPL13	16	89560677..89564542	175,61	#####	-0,142482519
CPNE7	16	89575758..89597246	7,52	17,78	-1,018379493
DPEP1	16	89613308..89638456	0,02	0,01	0,524121557
CHMP1A	16	complement(89640816..89657738)	15,11	15,79	0,159301212
SPATA33	16	89657740..89671272	1,55	2,07	-0,194722095
CDK10	16	89680737..89696354	9,99	9,78	0,254412454
SPATA2L	16	complement(89696357..89701705)	3,01	2,83	0,311210976
VPS9D1	16	complement(89707134..89720898)	6,85	9,75	-0,286203565
ZNF276	16	89720400..89740925	3,85	4,00	0,166815161
FANCA	16	complement(89726683..89816977)	4,86	5,74	-0,016575074
SPIRE2	16	89818179..89871319	1,73	3,02	-0,582007477
TCF25	16	89873570..89913627	19,99	24,70	-0,082453257
MC1R	16	89912119..89920973	6,86	5,61	0,513895107
gene:ENSG000	16	89919165..89936092	0,10	-	7,56729892
TUBB3	16	89921392..89938761	135,50	11,19	3,820558235
DEF8	16	89948697..89968060	31,01	11,49	1,65594336
DBNDD1	16	complement(90004871..90019890)	16,89	8,60	1,197120002
GAS8	16	90019629..90044975	10,14	3,32	1,832949635
PRDM7	16	complement(90056566..90092072)	0,02	0,01	0,677647514
SCGB1C2	17	137569..139067	0,07	-	4,516772761
DOC2B	17	complement(142789..181650)	0,01	-	4,913749003
RPH3AL	17	complement(212389..386254)	0,56	1,01	-0,629409271
LIAT1	17	410325..431062	0,15	0,18	0,001721572
RFLNB	17	complement(439978..445939)	2,30	0,54	2,306400253
VPS53	17	complement(508503..721717)	2,43	2,48	0,197159158

TLCD3A	17	732412..742968	4,09	5,72	-0,258249708
GEMIN4	17	complement(744421..753999)	7,55	10,21	-0,212705145
GLOD4	17	complement(757097..782294)	9,75	12,50	-0,135206092
MRM3	17	782353..792509	4,54	6,46	-0,284300691
NXN	17	complement(799310..979776)	34,74	11,73	1,789620039
TIMM22	17	997129..1003671	5,00	6,67	-0,192850412
ABR	17	complement(1003519..1229738)	13,40	11,66	0,423693787
BHLHA9	17	1270444..1271815	-	-	0,188027316
TRARG1	17	1279662..1300978	0,01	-	3,967268276
YWHAE	17	complement(1344275..1400222)	198,18	#####	-0,091063599
gene:ENSG000	17	1352070..1352255	0,41	0,49	-0,036228841
CRK	17	complement(1420689..1463162)	14,34	14,20	0,236751894
MYO1C	17	complement(1464186..1492686)	52,04	50,33	0,271267764
INPP5K	17	complement(1494577..1516742)	4,00	4,15	0,170089134
PITPNA	17	complement(1517718..1562792)	11,07	13,74	-0,087886126
SLC43A2	17	complement(1569268..1628886)	0,17	0,39	-0,965438661
SCARF1	17	complement(1633858..1645744)	0,11	0,13	-0,039936706
RILP	17	complement(1646145..1649866)	0,81	0,84	0,169359452
PRPF8	17	complement(1650629..1684867)	83,06	#####	-0,133031612
TLCD2	17	complement(1702816..1710377)	1,07	0,58	1,11269685
WDR81	17	1716523..1738599	2,58	2,23	0,4340715
SERPINF2	17	1742836..1755265	0,05	0,01	2,345408045
SERPINF1	17	1762029..1777565	0,42	0,34	0,535768584
SMYD4	17	complement(1779485..1830634)	3,94	3,80	0,275098857
RPA1	17	1829702..1900082	24,64	25,58	0,168970114
RTN4RL1	17	complement(1934677..2025334)	1,09	3,37	-1,405232118
DPH1	17	2030137..2043898	9,47	9,31	0,247696159
OVCA2	17	2042022..2043425	4,29	6,52	-0,380813452
HIC1	17	2054154..2063241	0,36	0,72	-0,806312772
SMG6	17	complement(2059839..2303785)	3,49	4,65	-0,192983935
gene:ENSG000	17	2072823..2072956	-	-	0,188027316
gene:ENSG000	17	2101542..2101667	-	-	0,188027316
gene:ENSG000	17	2117626..2117772	-	-	#N/D
gene:ENSG000	17	complement(2122342..2122508)	-	-	0,188027316
SMG6-IT1	17	complement(2235713..2236086)	0,09	0,03	1,436225396
gene:ENSG000	17	2255719..2256157	0,12	0,06	1,232079601
SRR	17	2303383..2325260	1,53	2,46	-0,458083487
TSR1	17	complement(2322396..2336657)	16,91	19,23	0,038219796
SGSM2	17	2337498..2381058	11,10	10,87	0,252983967
MNT	17	complement(2384073..2401104)	22,80	10,32	1,366277126
METTL16	17	complement(2405562..2511891)	7,15	8,20	0,025380921
gene:ENSG000	17	complement(2424179..2424412)	-	-	#N/D
gene:ENSG000	17	complement(2460183..2460294)	-	-	#N/D
PAFAH1B1	17	2593183..2685615	11,80	15,13	-0,136147257
gene:ENSG000	17	2639297..2642418	0,23	0,32	-0,243528719
CLUH	17	complement(2689386..2712663)	22,94	29,89	-0,158699933
CCDC92B	17	complement(2720801..2749652)	0,03	0,09	-1,416252183
RAP1GAP2	17	2755705..3037741	9,45	17,12	-0,634802936

OR1D5	17	complement(3062669..3063607)	0,01	0,04	-1,478387936
OR1D2	17	complement(3088484..3104422)	-	-	0,188027316
OR1G1	17	complement(3126610..3127551)	-	-	#N/D
OR1P1	17	complement(3153889..3154882)	-	-	0,188027316
OR1A2	17	3197519..3198448	-	-	0,188027316
OR1A1	17	3207539..3218896	-	-	#N/D
OR3A2	17	complement(3276177..3386317)	-	-	0,188027316
OR3A1	17	complement(3291017..3298360)	-	-	#N/D
OR1R1P	17	3385930..3386874	-	-	#N/D
OR1E1	17	complement(3397104..3398410)	-	-	#N/D
OR3A3	17	3411370..3424070	-	0,01	-4,359055975
OR1E2	17	complement(3432870..3433841)	-	-	#N/D
gene:ENSG000	17	complement(3439923..3441844)	-	-	#N/D
SPATA22	17	complement(3440019..3513852)	-	-	0,188027316
ASPA	17	3472374..3503405	0,00	0,01	-0,579815015
TRPV3	17	complement(3510502..3557812)	0,00	0,12	-5,764259665
TRPV1	17	complement(3565444..3609411)	1,49	1,89	-0,125878908
gene:ENSG000	17	complement(3585149..3636249)	0,27	0,62	-0,953139219
SHPK	17	complement(3608240..3636250)	6,33	3,91	0,917470853
CTNS	17	3636459..3663103	2,23	2,36	0,143808924
TAX1BP3	17	complement(3662895..3668679)	51,14	48,61	0,296265271
P2RX5-TAX1BP	17	complement(3663063..3696194)	0,39	0,57	-0,322664414
EMC6	17	3668812..3669668	13,23	15,68	-0,022452398
P2RX5	17	complement(3672199..3696240)	5,86	10,33	-0,596183908
ITGAE	17	complement(3714628..3801188)	0,65	1,50	-0,992072483
HASPIN	17	3723903..3726699	3,96	8,11	-0,811339244
NCBP3	17	complement(3802158..3846246)	2,56	2,97	0,008770729
CAMKK1	17	complement(3860315..3894891)	5,85	5,42	0,332888693
P2RX1	17	complement(3896592..3916476)	0,00	0,02	-1,877914477
ATP2A3	17	complement(3923870..3964464)	0,98	0,48	1,241934577
ZZEF1	17	complement(4004445..4143030)	8,86	5,79	0,835450794
CYB5D2	17	4143168..4187310	3,42	3,10	0,36620074
ANKFY1	17	complement(4163821..4263995)	5,76	7,04	-0,067889332
UBE2G1	17	complement(4269259..4366675)	6,77	10,34	-0,388816103
SPNS3	17	4433940..4488208	-	0,01	-2,89252005
SPNS2	17	4498881..4539035	1,23	1,02	0,492803279
MYBBP1A	17	complement(4538897..4555386)	20,36	25,95	-0,126774969
GGT6	17	complement(4556927..4560818)	-	0,01	-4,359055975
SMTNL2	17	4583999..4608319	0,00	-	3,068695355
ALOX15	17	complement(4630919..4642294)	0,02	0,04	-0,865575989
PELP1	17	complement(4669774..4704337)	17,78	19,10	0,119809842
ARRB2	17	4710596..4721499	5,64	8,14	-0,306046973
MED11	17	4731428..4733608	1,97	2,34	-0,024648224
CXCL16	17	complement(4733533..4739928)	0,52	1,62	-1,410724157
ZMYND15	17	4739833..4746119	0,07	0,10	-0,428283595
TM4SF5	17	4771884..4783213	-	-	#N/D
VMO1	17	complement(4785285..4786433)	0,09	0,15	-0,392761763
gene:ENSG000	17	4787057..4789162	0,53	0,45	0,455344973

GLTPD2	17	4788964..4790589	0,07	0,09	-0,036800208
PSMB6	17	4796144..4798502	82,04	99,82	-0,05990903
C17orf114	17	complement(4801159..4807013)	0,51	0,27	1,117604237
PLD2	17	4807152..4823434	5,26	5,50	0,156734152
MINK1	17	4833340..4898061	30,09	26,03	0,432634915
CHRNE	17	complement(4897771..4934438)	0,22	0,24	0,070476772
C17orf107	17	4899418..4902934	0,21	0,33	-0,438223471
GP1BA	17	4932277..4935023	2,63	2,08	0,56411416
SLC25A11	17	complement(4937130..4940053)	39,89	42,94	0,116845448
RNF167	17	4940008..4945222	29,95	34,68	0,011363127
PFN1	17	complement(4945652..4949061)	415,18	#####	0,233310424
ENO3	17	4948092..4957131	2,41	6,42	-1,189175776
SPAG7	17	complement(4959226..4967817)	11,78	13,54	0,022836807
CAMTA2	17	complement(4967992..4987675)	11,19	19,37	-0,568491961
INCA1	17	complement(4988130..4997610)	1,51	1,25	0,498233244
KIF1C	17	4997950..5028401	16,74	19,20	0,025136966
SLC52A1	17	complement(5032600..5052009)	0,11	0,09	0,488286468
ZFP3	17	5078467..5096374	0,02	0,01	1,505976878
ZNF232	17	complement(5105269..5123162)	1,00	2,23	-0,939756016
USP6	17	5116032..5175034	0,03	0,03	0,158573612
ZNF594	17	complement(5179535..5191868)	0,49	1,00	-0,793982691
SCIMP	17	complement(5208920..5234860)	0,22	0,19	0,390825535
RABEP1	17	5282265..5386340	2,52	6,06	-1,044543179
NUP88	17	complement(5359668..5420164)	3,99	6,07	-0,380134859
RPAIN	17	5419641..5432877	2,00	2,94	-0,335601584
C1QBP	17	complement(5432777..5448830)	48,14	89,06	-0,664497349
DHX33	17	complement(5440917..5468982)	7,07	10,15	-0,297860756
DERL2	17	complement(5471254..5486811)	5,09	3,57	0,735030329
MIS12	17	5486285..5490814	4,20	4,77	0,039236575
NLRP1	17	complement(5499415..5619424)	1,30	2,79	-0,874800642
gene:ENSG000	17	complement(5499427..5501006)	0,63	0,13	2,501394944
gene:ENSG000	17	5772926..6080792	0,04	1,09	-4,385296617
WSCD1	17	6057807..6124427	0,11	1,81	-3,769997056
AIPL1	17	complement(6393693..6435199)	-	0,01	-3,804623889
PIMREG	17	6444441..6451469	19,75	17,25	0,418346377
PITPNM3	17	complement(6451263..6556555)	1,24	1,71	-0,239053445
KIAA0753	17	complement(6578147..6640711)	2,89	3,05	0,145815678
gene:ENSG000	17	complement(6578147..6640711)	0,54	0,46	0,450006959
TXNDC17	17	6640985..6644541	2,69	3,31	-0,079652264
MED31	17	complement(6643311..6651634)	3,32	2,68	0,534557067
C17orf100	17	6651762..6693202	0,59	1,19	-0,785664219
SLC13A5	17	complement(6684719..6713377)	0,02	-	5,697545031
XAF1	17	6755447..6775647	0,16	-	9,218481149
PRAL	17	complement(6772831..6776116)	0,02	-	5,697545031
FBXO39	17	6776215..6797101	0,01	0,02	-0,979341556
TEKT1	17	complement(6789133..6831761)	-	-	#N/D
ALOX12	17	6996049..7010754	0,41	0,16	1,604765501
RNASEK	17	7012417..7014532	34,73	35,62	0,18669815

RNASEK-C17or	17	7012635..7017520	0,87	0,53	0,937372107
C17orf49	17	7014495..7017525	26,64	25,20	0,303026325
BCL6B	17	7023050..7030290	-	0,03	-6,187931073
SLC16A13	17	7036015..7040117	0,82	1,76	-0,868144078
SLC16A11	17	complement(7041621..7044092)	0,15	0,11	0,644262894
CLEC10A	17	complement(7074537..7080307)	-	-	#N/D
ASGR2	17	complement(7101322..7115700)	-	-	0,188027316
ASGR1	17	complement(7173431..7179564)	0,22	0,15	0,777984703
DLG4	17	complement(7187187..7219836)	8,98	5,99	0,807891559
gene:ENSG000	17	complement(7192295..7192448)	0,84	0,34	1,515286335
ACADVL	17	7217125..7225266	128,65	#####	0,499906373
DVL2	17	complement(7225342..7234517)	11,69	10,94	0,31924159
PHF23	17	complement(7235029..7239722)	10,51	11,30	0,117911763
GABARAP	17	complement(7240008..7242449)	47,03	39,74	0,465955391
gene:ENSG000	17	complement(7240427..7244635)	0,79	0,40	1,202979428
gene:ENSG000	17	7242200..7243814	0,53	0,60	0,034368956
CTDNEP1	17	complement(7243591..7252491)	64,08	66,11	0,177915337
gene:ENSG000	17	complement(7246829..7262089)	-	-	0,188027316
ELP5	17	7251416..7259940	19,72	17,81	0,369569777
CLDN7	17	complement(7259903..7263983)	0,32	0,34	0,139798879
SLC2A4	17	7281718..7288257	0,17	0,12	0,725319184
YBX2	17	complement(7288263..7294639)	1,37	1,54	0,051408266
EIF5A	17	7306999..7312463	502,05	#####	-0,048054176
GPS2	17	complement(7311324..7315564)	6,33	10,14	-0,457177655
gene:ENSG000	17	complement(7312661..7319174)	0,34	0,17	1,190735552
NEURL4	17	complement(7315628..7329393)	13,86	10,08	0,681548201
ACAP1	17	7336529..7351477	0,84	0,38	1,36548508
KCTD11	17	7351889..7354944	5,18	4,92	0,296605872
TMEM95	17	7355123..7357219	0,06	0,07	-0,036228841
TNK1	17	7380534..7389774	0,30	0,18	0,972658575
PLSCR3	17	complement(7389727..7394842)	7,53	6,72	0,387328372
TMEM256-PLS(	17	complement(7389734..7404097)	0,12	0,13	0,173321121
TMEM256	17	complement(7402975..7404097)	13,16	15,57	-0,020199912
NLGN2	17	7404874..7419860	22,02	21,86	0,233340249
SPEM1	17	7420324..7421632	0,01	0,11	-3,021611439
SPEM2	17	7425616..7427568	-	0,02	-3,804623889
SPEM3	17	7428857..7432820	-	0,00	-2,89252005
gene:ENSG000	17	7428861..7436278	-	-	0,188027316
TMEM102	17	7435435..7437679	0,48	0,71	-0,352924543
FGF11	17	7438273..7444937	0,13	0,17	-0,14497129
CHRNB1	17	7445061..7457710	1,06	0,92	0,419660611
ZBTB4	17	complement(7459366..7484263)	20,43	17,54	0,443298752
SLC35G6	17	7481446..7483496	0,28	0,47	-0,514092269
POLR2A	17	7484366..7514616	22,93	27,02	-0,013862991
TNFSF12	17	7548508..7557890	0,40	0,12	1,953666277
TNFSF12-TNFS	17	7549099..7561601	-	-	0,188027316
TNFSF13	17	7558292..7561608	2,05	3,04	-0,34319443
SENP3	17	7561919..7571969	19,32	31,34	-0,47459588

SENP3-EIF4A1	17	7563287..7578715	1,26	1,45	0,019969228
EIF4A1	17	7572824..7579006	172,77	#####	0,080889804
CD68	17	7579491..7582111	35,34	32,38	0,349536993
MPDU1	17	7583529..7592789	11,24	12,29	0,093398857
SOX15	17	complement(7588178..7590094)	0,50	0,67	-0,200560769
FXR2	17	complement(7591230..7614897)	22,93	27,21	-0,023528294
SHBG	17	7613946..7633382	0,01	0,03	-0,979341556
SAT2	17	complement(7626234..7627876)	10,80	12,27	0,039839409
ATP1B2	17	7646627..7657770	0,01	0,01	-0,025382928
TP53	17	complement(7661779..7687546)	-	0,00	-3,804623889
WRAP53	17	7686071..7703502	4,26	4,50	0,144452431
EFNB3	17	7705202..7711372	0,14	0,09	0,824874595
DNAH2	17	7717744..7833742	0,01	0,01	-0,741621346
KDM6B	17	7834217..7854796	18,07	10,13	1,05781057
TMEM88	17	7855066..7856099	1,18	0,92	0,589126283
NAA38	17	complement(7856685..7885238)	24,88	24,46	0,247746137
CYB5D1	17	7857746..7862282	5,56	2,68	1,278380002
gene:ENSG000	17	7858943..7866083	1,54	0,89	1,012304741
CHD3	17	7884796..7912760	33,03	50,03	-0,376156682
RNF227	17	complement(7913339..7916289)	1,02	1,18	0,001341486
KCNAB3	17	complement(7921859..7929856)	0,31	0,51	-0,494075921
gene:ENSG000	17	complement(7925931..7930622)	-	-	0,188027316
TRAPPC1	17	complement(7930345..7932123)	102,36	85,42	0,48419515
CNTROB	17	7932101..7949920	17,76	14,09	0,557248487
GUCY2D	17	8002615..8020342	0,01	0,00	0,886720911
gene:ENSG000	17	complement(8013327..8016378)	-	-	#N/D
ALOX15B	17	8039034..8049134	-	0,01	-4,359055975
ALOX12B	17	complement(8072636..8087716)	-	0,02	-4,359055975
ALOXE3	17	complement(8095900..8119047)	0,04	0,10	-1,124991381
HES7	17	complement(8120592..8124106)	0,22	2,51	-3,299285336
PER1	17	complement(8140472..8156506)	31,35	7,39	2,307927963
gene:ENSG000	17	complement(8150816..8162975)	1,50	1,24	0,498833771
VAMP2	17	complement(8159149..8163546)	31,44	13,68	1,424104164
TMEM107	17	complement(8172457..8176399)	3,92	3,31	0,464455562
BORCS6	17	complement(8188345..8190180)	5,11	4,89	0,285178639
gene:ENSG000	17	8188933..8189688	-	-	0,188027316
AURKB	17	complement(8204733..8210600)	64,53	86,82	-0,204849833
CTC1	17	complement(8224815..8248058)	2,49	3,86	-0,407114362
PFAS	17	8247618..8270491	9,06	18,37	-0,796236309
SLC25A35	17	complement(8287763..8295400)	3,45	4,41	-0,130388489
RANGRF	17	8288654..8290092	9,61	9,28	0,27340033
ARHGEF15	17	8310241..8322514	-	-	#N/D
ODF4	17	8339840..8346048	0,01	-	3,068695355
KRBA2	17	complement(8356902..8376704)	0,71	1,18	-0,502270564
gene:ENSG000	17	complement(8368638..8383187)	0,06	0,27	-1,861438984
RPL26	17	complement(8377516..8383213)	225,63	#####	-0,06223653
RNF222	17	complement(8390702..8397827)	0,14	0,24	-0,524103859
NDEL1	17	8413131..8490411	28,41	16,72	0,988100386

MYH10	17	complement(8474207..8631376)	10,54	12,69	-0,045030036
CCDC42	17	complement(8729935..8745219)	-	-	0,188027316
SPDYE4	17	complement(8747524..8758559)	0,01	-	4,516772761
MFSD6L	17	complement(8797110..8799365)	-	0,01	-2,89252005
PIK3R6	17	complement(8802722..8867677)	0,01	0,00	0,886720911
PIK3R5	17	complement(8878911..8965712)	0,00	0,01	-1,291882393
NTN1	17	9021510..9244000	0,64	1,55	-1,048094017
STX8	17	complement(9250471..9576591)	9,48	11,78	-0,090532359
CFAP52	17	9576627..9643447	0,02	0,02	0,533811434
USP43	17	9644698..9729687	0,40	1,54	-1,712271519
DHRS7C	17	complement(9771434..9791592)	-	-	0,188027316
GSG1L2	17	complement(9800608..9822079)	-	-	0,188027316
GLP2R	17	9822206..9892099	0,25	0,42	-0,533528945
RCVRN	17	complement(9896320..9905271)	-	0,02	-4,359055975
GAS7	17	complement(9910606..10198606)	0,75	0,75	0,219570056
MYH13	17	complement(10300865..10373130)	-	-	0,188027316
MYH8	17	complement(10390322..10421950)	-	-	#N/D
MYH4	17	complement(10443290..10469559)	-	-	#N/D
MYH1	17	complement(10492307..10518542)	-	-	0,188027316
MYH2	17	complement(10521148..10549700)	-	-	0,188027316
MYH3	17	complement(10628526..10657309)	0,14	0,61	-1,935317596
SCO1	17	complement(10672474..10698375)	3,22	5,30	-0,494476525
ADPRM	17	10697594..10711558	2,00	2,44	-0,064452563
TMEM220	17	complement(10699015..10730023)	0,49	0,36	0,681947583
TMEM238L	17	complement(10794908..10804099)	0,03	-	4,913749003
gene:ENSG000	17	complement(10821901..10822243)	-	-	#N/D
PIRT	17	complement(10822470..10838087)	-	-	#N/D
SHISA6	17	11241213..11564063	-	-	0,188027316
DNAH9	17	11598470..11969748	0,00	0,00	-0,345401666
ZNF18	17	complement(11977439..11997475)	1,14	2,45	-0,878290145
MAP2K4	17	12020829..12143830	3,63	5,43	-0,356528118
MYOCD	17	12665890..12768949	0,02	0,08	-1,86514709
ARHGAP44	17	12789498..12991643	2,87	4,63	-0,465943126
gene:ENSG000	17	complement(12973033..12973834)	0,13	0,36	-1,164660879
ELAC2	17	complement(12991612..13018065)	12,63	15,63	-0,084216463
HS3ST3A1	17	complement(13494032..13601929)	0,60	1,47	-1,076246742
COX10	17	14069490..14231736	2,69	3,88	-0,304734865
CDRT15	17	complement(14235673..14236862)	0,90	0,32	1,726896678
HS3ST3B1	17	14301081..14349404	0,29	0,21	0,668987489
PMP22	17	complement(15229773..15272292)	2,06	2,47	-0,038932173
gene:ENSG000	17	15260513..15261841	0,28	0,18	0,914231636
TEKT3	17	complement(15303811..15341632)	0,04	0,07	-0,489732438
gene:ENSG000	17	15365323..15365877	0,02	-	3,068695355
CDRT4	17	complement(15436015..15475728)	3,31	1,71	1,178726455
TVP23C-CDRT4	17	complement(15436021..15563561)	1,32	2,05	-0,40665594
TVP23C	17	complement(15502264..15563595)	0,90	0,67	0,642183031
FBXW10B	17	complement(15565483..15619704)	0,01	0,05	-2,634741553
gene:ENSG000	17	complement(15571491..15651653)	0,03	0,01	2,134539329

TRIM16	17	complement(15627960..15684311)	10,49	10,28	0,253338701
ZNF286A-TBC1	17	15699552..15745940	0,07	0,10	-0,137116903
ZNF286A	17	15699577..15720787	1,12	1,73	-0,408326204
TBC1D26	17	15732247..15746162	0,01	0,03	-0,999120035
ADORA2B	17	15945130..15975746	13,30	19,51	-0,330283245
ZSWIM7	17	complement(15976560..15999717)	1,73	2,46	-0,283467675
TTC19	17	15999784..16045015	3,33	5,61	-0,52922147
NCOR1	17	complement(16029065..16218185)	6,36	9,68	-0,384126184
PIGL	17	16217191..16351797	2,03	1,71	0,466245872
CENPV	17	complement(16342534..16353656)	2,95	11,42	-1,732065539
UBB	17	16380798..16382745	192,12	#####	-0,074500207
TRPV2	17	16415571..16437003	0,77	0,88	0,031021188
LRRC75A	17	complement(16441577..16492193)	40,64	50,53	-0,091119819
ZNF287	17	complement(16546954..16569204)	0,12	0,24	-0,757273144
ZNF624	17	complement(16620734..16653856)	0,18	0,30	-0,536333793
CCDC144A	17	16689537..16777881	0,25	0,49	-0,766347221
gene:ENSG000	17	16690261..16804453	0,54	0,60	0,084979293
TNFRSF13B	17	complement(16929816..16972118)	-	-	0,188027316
MPRIP	17	17042457..17217679	16,44	17,17	0,160855873
gene:ENSG000	17	17181504..17183257	4,15	4,27	0,182936242
PLD6	17	complement(17200995..17206333)	6,54	5,98	0,352166686
gene:ENSG000	17	complement(17202649..17237185)	-	0,18	-7,304021955
FLCN	17	complement(17212212..17237188)	5,40	6,47	-0,038090057
COPS3	17	complement(17246616..17281273)	11,34	17,59	-0,410322515
NT5M	17	17303335..17347663	2,22	4,16	-0,681572516
MED9	17	17476994..17493221	4,86	5,05	0,167536281
RASD1	17	complement(17494437..17496395)	0,32	0,45	-0,269995358
PEMT	17	complement(17505563..17591708)	8,71	10,71	-0,075389007
gene:ENSG000	17	17616043..17617745	0,02	0,03	-0,429837071
RAI1	17	17681458..17811453	8,92	8,14	0,355675648
SREBF1	17	complement(17810399..17837002)	9,98	6,25	0,898891589
TOM1L2	17	complement(17843511..17972422)	6,89	6,71	0,261818671
DRC3	17	17972813..18016889	0,18	0,21	0,013027003
ATPAF2	17	complement(17977409..18039209)	1,40	2,16	-0,39880167
gene:ENSG000	17	complement(18026072..18026771)	1,09	0,74	0,785297892
GID4	17	18039408..18068405	3,10	3,53	0,035932741
DRG2	17	18087892..18107970	6,34	7,70	-0,057369458
gene:ENSG000	17	complement(18107334..18110463)	0,25	0,29	-0,02028187
MYO15A	17	18108756..18179802	0,01	0,01	0,943435191
ALKBH5	17	18183078..18209954	40,13	29,08	0,687761175
LLGL1	17	18225635..18244875	5,87	10,83	-0,660961578
FLII	17	complement(18244815..18258738)	26,03	32,57	-0,100444188
MIEF2	17	18260597..18266552	4,00	3,30	0,497823947
TOP3A	17	complement(18271428..18315007)	7,53	5,17	0,764920893
SMCR8	17	18315293..18328056	5,91	4,59	0,587074669
SHMT1	17	complement(18327873..18363550)	2,98	3,38	0,042620686
EVPLL	17	18377778..18389647	-	-	0,188027316
LGALS9C	17	18476737..18494945	0,23	0,01	4,263220504

TBC1D28	17	complement(18634247..18644427)	0,00	-	3,068695355
FOXO3B	17	complement(18667629..18682314)	0,87	0,96	0,088881087
FBXW10	17	18744026..18779349	0,04	0,03	0,582943981
TVP23B	17	18781111..18806714	4,74	3,82	0,534362213
PRPSAP2	17	18840085..18931287	2,45	4,19	-0,549579502
SLC5A10	17	18950345..19022595	0,16	0,07	1,398337516
FAM83G	17	complement(18968789..19004764)	4,49	10,04	-0,936798752
GRAP	17	complement(19020656..19047011)	0,06	0,11	-0,581183184
gene:ENSG000	17	19030857..19033529	0,06	0,25	-1,893640597
GRAPL	17	19127535..19162274	0,27	0,71	-1,198100856
EPN2	17	19215615..19336715	8,84	7,15	0,528254103
B9D1	17	complement(19334308..19378193)	2,09	1,84	0,410772624
MAPK7	17	19377721..19383544	6,12	3,51	1,024971547
MFAP4	17	complement(19383442..19387190)	2,57	1,97	0,60038776
RNF112	17	19411125..19417276	0,77	0,62	0,546904016
SLC47A1	17	19495385..19579034	2,98	5,17	-0,570145995
ALDH3A2	17	19648136..19685760	13,40	9,15	0,772397659
SLC47A2	17	complement(19678288..19718979)	0,71	0,12	2,832169342
ALDH3A1	17	complement(19737984..19748943)	28,16	6,48	2,342484695
ULK2	17	complement(19770829..19867936)	1,28	2,20	-0,560567614
gene:ENSG000	17	19855747..19856164	0,05	0,15	-1,291882393
AKAP10	17	complement(19904302..19978343)	2,89	4,22	-0,325825852
SPECC1	17	20008865..20319026	1,46	2,46	-0,531052745
LGALS9B	17	complement(20449395..20467539)	0,16	-	7,673595028
CDRT15L2	17	20579724..20580911	-	-	#N/D
USP22	17	complement(20999596..21043760)	62,80	65,17	0,16944938
DHRS7B	17	21123364..21193265	2,44	2,50	0,189621649
TMEM11	17	complement(21197954..21214176)	15,42	15,16	0,247213339
NATD1	17	complement(21238870..21253410)	0,68	0,58	0,44907486
MAP2K3	17	21284672..21315232	17,40	15,68	0,373243833
KCNJ12	17	21376357..21419870	0,39	0,94	-1,051163299
KCNJ18	17	21692523..21704612	-	-	0,188027316
WSB1	17	27294076..27315926	3,89	3,63	0,324887202
KSR1	17	27456448..27626438	0,98	2,73	-1,258048895
gene:ENSG000	17	27623364..27640777	-	-	0,188027316
LGALS9	17	27629798..27649560	0,34	0,26	0,58101812
NOS2	17	complement(27756766..27800529)	0,01	0,00	1,893574746
gene:ENSG000	17	complement(27798806..27893365)	-	-	#N/D
LYRM9	17	complement(27878314..27894752)	0,62	1,01	-0,475304394
NLK	17	28041737..28196381	5,72	4,36	0,614421473
TMEM97	17	28319200..28328685	7,55	7,37	0,258023001
IFT20	17	complement(28328325..28335489)	8,92	6,06	0,780768511
TNFAIP1	17	28335602..28347009	20,12	15,71	0,580305142
POLDIP2	17	complement(28346633..28357527)	26,42	45,42	-0,55850375
TMEM199	17	28357642..28363683	2,18	2,99	-0,230011369
SEBOX	17	complement(28363506..28365199)	0,54	0,49	0,366768448
gene:ENSG000	17	complement(28364288..28368012)	-	-	0,188027316
SARM1	17	28364356..28404049	2,62	3,77	-0,299575102

VTN	17	complement(28367284..28373091)	0,17	0,21	-0,091870672
gene:ENSG000	17	complement(28373673..28374301)	0,41	0,93	-0,942651554
SLC46A1	17	complement(28394642..28407197)	1,94	2,03	0,157060168
RSKR	17	complement(28455752..28614197)	1,95	1,88	0,27476674
SLC13A2	17	28473293..28497781	-	-	#N/D
FOXN1	17	28506243..28538900	0,00	0,04	-3,171495545
UNC119	17	complement(28546708..28552631)	5,13	6,12	-0,031316687
PIGS	17	complement(28553383..28571794)	11,92	14,40	-0,049268201
ALDOC	17	complement(28573115..28576948)	0,58	1,69	-1,322424536
SPAG5	17	complement(28577565..28599025)	32,79	32,07	0,255140244
gene:ENSG000	17	complement(28580012..28614185)	0,68	0,18	2,16386377
BLTP2	17	complement(28614446..28645454)	31,52	33,33	0,14225949
SDF2	17	complement(28648346..28662189)	15,32	13,04	0,455869058
SUPT6H	17	28662198..28702679	16,76	15,82	0,306321915
PROCA1	17	complement(28703197..28711888)	1,37	1,36	0,226903014
RAB34	17	complement(28714281..28718429)	38,36	49,32	-0,139637439
RPL23A	17	28719985..28724359	184,12	#####	-0,003088346
TLCD1	17	complement(28724348..28727935)	2,40	4,27	-0,610874198
NEK8	17	28725897..28743455	0,76	0,64	0,452980705
TRAF4	17	28744011..28750956	12,57	8,74	0,746659833
FAM222B	17	complement(28755980..28855232)	1,94	3,06	-0,433398196
ERAL1	17	28855010..28861061	24,44	32,57	-0,191371956
FLOT2	17	complement(28879335..28897733)	28,19	40,68	-0,305949426
DHRS13	17	complement(28897781..28903079)	2,03	3,94	-0,732661378
PHF12	17	complement(28905250..28951771)	5,96	5,87	0,245050981
PIPOX	17	28950513..29057216	0,05	0,09	-0,708502297
SEZ6	17	complement(28954901..29006440)	0,04	0,13	-1,370061022
MYO18A	17	complement(29071122..29180398)	8,74	7,33	0,475503214
CRYBA1	17	29246859..29254494	0,08	0,06	0,533811434
NUFIP2	17	complement(29255839..29294148)	20,88	16,34	0,576182466
TAOK1	17	29390363..29551903	7,06	8,58	-0,057180524
ABHD15	17	complement(29560547..29567037)	1,45	2,91	-0,779450088
TP53I13	17	29566052..29573157	6,21	8,44	-0,218062773
GIT1	17	complement(29573475..29594054)	13,62	17,54	-0,141185301
ANKRD13B	17	29589769..29614761	4,69	9,36	-0,775025497
CORO6	17	complement(29614756..29622912)	0,69	0,50	0,695592445
SSH2	17	complement(29625938..29930276)	2,09	3,65	-0,578315359
EFCAB5	17	29929200..30108452	0,00	0,01	-2,447197442
NSRP1	17	30115521..30186475	1,51	2,01	-0,191365192
SLC6A4	17	complement(30194319..30236002)	0,02	0,02	0,53887001
BLMH	17	complement(30248203..30292056)	11,36	16,62	-0,325619014
TMIGD1	17	complement(30316333..30334059)	-	-	0,188027316
CPD	17	30378927..30469989	8,14	11,80	-0,312086713
GOSR1	17	30477362..30527592	10,32	9,98	0,271189241
gene:ENSG000	17	30551193..30551802	0,09	0,21	-1,015460095
gene:ENSG000	17	complement(30724982..30725181)	-	-	0,188027316
gene:ENSG000	17	30738182..30740275	0,42	0,52	-0,075187455
CRLF3	17	complement(30769388..30824692)	4,25	5,18	-0,062368125

ATAD5	17	30831966..30895869	1,05	1,80	-0,55208683
TEFM	17	complement(30897336..30906238)	1,24	1,46	-0,010768146
ADAP2	17	30906344..30959322	0,06	0,04	0,78195819
RNF135	17	30970984..30999911	5,20	11,51	-0,921517724
NF1	17	31094927..31382116	6,50	7,86	-0,050993916
OMG	17	complement(31272013..31297539)	0,13	0,19	-0,345671544
EVI2B	17	complement(31303770..31314105)	0,04	0,16	-1,664396418
gene:ENSG000	17	complement(31305213..31318831)	0,03	0,05	-0,742377908
EVI2A	17	complement(31316410..31321749)	0,03	0,13	-1,759313529
RAB11FIP4	17	31391675..31538211	0,19	0,18	0,255293718
gene:ENSG000	17	complement(31533171..31533938)	-	-	0,188027316
COPRS	17	complement(31851871..31859291)	8,80	12,52	-0,285378479
UTP6	17	complement(31860904..31901708)	6,65	6,59	0,23671273
SUZ12	17	31937007..32001038	3,60	6,65	-0,662784548
LRRC37B	17	32007383..32053504	1,27	1,25	0,243880551
gene:ENSG000	17	32051030..32053208	0,97	1,14	-0,010986601
RHOT1	17	32142454..32253374	4,26	6,26	-0,331888496
gene:ENSG000	17	32159311..32160371	0,58	0,48	0,50562527
gene:ENSG000	17	complement(32178946..32180074)	0,31	0,61	-0,765382083
RHBDL3	17	32265832..32324659	0,56	0,47	0,468971896
C17orf75	17	complement(32324565..32350023)	0,98	1,95	-0,769395219
ZNF207	17	32350132..32381885	8,53	8,44	0,238695234
PSMD11	17	32444379..32483319	20,40	37,92	-0,671332459
CDK5R1	17	32486993..32491253	1,78	1,72	0,277155913
MYO1D	17	complement(32492522..32877177)	0,14	0,07	1,135006798
gene:ENSG000	17	complement(32504567..32505644)	-	0,01	-2,89252005
gene:ENSG000	17	complement(32518322..32518934)	-	-	#N/D
H2BN1	17	32895433..32906586	-	-	#N/D
TMEM98	17	32927910..32945106	0,60	0,24	1,553017148
SPACA3	17	32970376..32997877	-	-	#N/D
ASIC2	17	complement(33013087..34174964)	0,00	-	3,068695355
gene:ENSG000	17	complement(33099969..33100319)	-	-	#N/D
gene:ENSG000	17	complement(34000789..34006772)	-	-	#N/D
CCL2	17	34255274..34257208	5,93	92,89	-3,746999221
CCL7	17	34270221..34272242	-	0,17	-6,187931073
CCL11	17	34285742..34288334	-	-	#N/D
CCL8	17	34319435..34321402	-	-	#N/D
CCL13	17	34356480..34358610	-	-	#N/D
CCL1	17	complement(34360328..34363233)	-	-	#N/D
TMEM132E	17	34579487..34639318	-	0,00	-2,89252005
gene:ENSG000	17	34614409..34614809	-	-	#N/D
CCT6B	17	complement(34927859..34981078)	0,09	0,20	-0,900569988
ZNF830	17	34961540..34963777	1,87	2,86	-0,389144733
LIG3	17	34980512..35009743	8,53	7,77	0,356912713
RFFL	17	complement(35005990..35089319)	4,04	6,46	-0,455548891
gene:ENSG000	17	complement(35011349..35121493)	-	0,02	-4,359055975
RAD51D	17	complement(35092221..35121522)	1,28	1,89	-0,33337414
FNDC8	17	35121615..35130732	0,11	0,04	1,735012788

NLE1	17	complement(35128730..35142304)	9,78	9,98	0,193906781
UNC45B	17	35147817..35189345	-	-	0,188027316
SLC35G3	17	complement(35192520..35194393)	-	-	#N/D
SLFN5	17	35243071..35273655	5,38	8,11	-0,370962408
SLFN11	17	complement(35350305..35373701)	17,94	14,89	0,49240065
SLFN12	17	complement(35409465..35433475)	1,78	1,81	0,20135182
SLFN13	17	complement(35435096..35448837)	0,38	0,25	0,829437421
SLFN12L	17	complement(35464249..35537683)	-	0,00	-3,804623889
SLFN14	17	complement(35543985..35560819)	0,01	0,01	0,181652234
PEX12	17	complement(35574795..35578863)	1,22	2,72	-0,93393123
AP2B1	17	35578046..35726413	33,96	49,53	-0,321398559
RASL10B	17	35731639..35743521	0,14	1,00	-2,564332519
GAS2L2	17	complement(35744511..35753239)	-	-	0,188027316
MMP28	17	complement(35756249..35795707)	0,09	0,12	-0,190947973
C17orf50	17	35760887..35765079	-	-	#N/D
TAF15	17	35809482..35864615	8,13	14,37	-0,598559096
HEATR9	17	complement(35854946..35868891)	0,01	0,02	-0,429837071
CCL5	17	complement(35871491..35880793)	0,92	0,51	1,072953083
RDM1	17	complement(35918066..35930773)	1,52	1,17	0,599154687
LYZL6	17	complement(35934518..35943713)	-	-	0,188027316
CCL16	17	complement(35976493..35981497)	0,02	-	4,913749004
CCL14	17	complement(35983288..35987004)	-	-	0,188027316
CCL15-CCL14	17	complement(35983656..36001621)	-	-	0,188027316
CCL15	17	complement(35996440..36001553)	0,02	-	3,967268276
CCL23	17	complement(36013056..36017972)	-	-	#N/D
CCL18	17	36064272..36072032	-	-	#N/D
CCL3	17	complement(36088256..36090169)	-	0,10	-5,327865481
CCL4	17	36103827..36105621	-	-	#N/D
TBC1D3B	17	complement(36165683..36176636)	0,13	0,09	0,739976582
CCL3L3	17	complement(36194869..36196758)	0,17	0,10	0,946003371
CCL4L2	17	36210924..36212878	-	-	0,188027316
TBC1D3I	17	complement(36253456..36264570)	0,25	0,34	-0,191914522
TBC1D3G	17	36323884..36334759	-	0,08	-6,426264264
TBC1D3H	17	complement(36377531..36388435)	0,17	-	7,865233509
TBC1D3F	17	complement(36428618..36439566)	-	0,02	-4,758582517
ZNHIT3	17	36486629..36499310	4,80	5,29	0,08365203
MYO19	17	complement(36495636..36543435)	11,70	13,98	-0,033739476
PIGW	17	36534987..36539310	2,63	2,95	0,058383073
GGNBP2	17	36544912..36589848	6,32	5,77	0,355557964
DHRS11	17	36591879..36600804	2,86	3,01	0,147309902
MRM1	17	36601583..36608964	2,77	4,26	-0,398870739
LHX1	17	36936785..36944612	1,23	2,48	-0,789322476
AATF	17	36948925..37056871	10,78	12,17	0,047196242
ACACA	17	complement(37084992..37406836)	11,48	10,06	0,412470739
C17orf78	17	37375985..37392708	-	0,02	-4,359055975
TADA2A	17	37406886..37479725	1,12	2,40	-0,872552915
DUSP14	17	37489891..37513501	7,57	9,44	-0,095400708
SYNRG	17	complement(37514807..37609472)	3,00	3,55	-0,0195777

DDX52	17	complement(37609739..37643446)	1,83	3,83	-0,841566604
HNF1B	17	complement(37686431..37745059)	-	-	0,188027316
TBC1D3K	17	37924415..37935371	-	-	0,188027316
TBC1D3L	17	complement(37978155..37989060)	2,38	2,75	0,018043246
TBC1D3D	17	38003976..38014908	0,05	0,42	-2,807789431
TBC1D3C	17	complement(38057693..38068634)	0,93	0,62	0,80803142
TBC1D3E	17	38127951..38138868	0,07	-	6,68162262
TBC1D3	17	complement(38181659..38192555)	-	1,03	-10,07851714
MRPL45	17	38297023..38323217	22,56	28,04	-0,090981431
GPR179	17	complement(38324571..38343956)	0,10	0,20	-0,699209726
SOCS7	17	38351844..38405593	10,45	6,88	0,825781772
ARHGAP23	17	38419280..38512385	8,95	10,68	-0,031017584
SRCIN1	17	complement(38530031..38605952)	0,13	0,27	-0,77044263
EPOP	17	complement(38671703..38674957)	2,03	1,88	0,336177275
MLLT6	17	38705273..38729795	15,65	17,57	0,056672897
gene:ENSG000	17	complement(38727833..38728198)	-	-	0,188027316
CISD3	17	38730341..38735605	7,40	9,06	-0,068591174
PCGF2	17	complement(38733898..38749817)	12,65	12,18	0,277803825
PSMB3	17	38752741..38764225	169,38	#####	0,168909684
PIP4K2B	17	complement(38765691..38800126)	14,51	13,63	0,313998776
CWC25	17	complement(38800441..38825355)	6,46	5,27	0,516866052
SPMAP1	17	complement(38835086..38841438)	-	-	0,188027316
RPL23	17	complement(38847860..38853764)	107,43	#####	-0,016592212
LASP1	17	38869859..38921770	60,68	51,14	0,469761992
LASP1NB	17	38925614..38929381	0,09	0,07	0,623580048
FBXO47	17	complement(38936432..38967403)	0,02	0,02	0,278120973
PLXDC1	17	complement(39063313..39154394)	0,01	0,02	0,112279066
ARL5C	17	complement(39156894..39167484)	-	-	#N/D
gene:ENSG000	17	39165073..39167849	0,02	-	5,224730805
CACNB1	17	complement(39173453..39197702)	2,35	3,58	-0,385729863
RPL19	17	39200283..39204840	173,54	#####	-0,226307241
STAC2	17	complement(39210541..39225945)	0,01	-	4,516772761
FBXL20	17	complement(39252663..39402523)	2,88	4,45	-0,404186709
MED1	17	complement(39404285..39451272)	11,69	17,30	-0,342216535
CDK12	17	39461486..39567559	8,49	11,07	-0,159874759
NEUROD2	17	complement(39603536..39609777)	0,01	0,02	-0,979341556
PPP1R1B	17	39626740..39636626	0,02	0,06	-1,326441896
STARD3	17	39637090..39664201	13,27	11,87	0,383318387
TCAP	17	39665349..39666554	1,33	0,51	1,590671473
PNMT	17	39667981..39670475	0,06	0,08	-0,036228841
PGAP3	17	complement(39671122..39696797)	3,10	2,99	0,273294657
ERBB2	17	39687914..39730426	7,61	11,04	-0,313955494
MIEN1	17	complement(39728496..39730532)	17,30	17,32	0,221661883
GRB7	17	39737927..39747291	0,06	0,17	-1,202073017
IKZF3	17	complement(39757718..39864312)	0,01	0,01	1,07890858
ZPBP2	17	39868202..39877896	-	-	#N/D
GSDMB	17	complement(39904595..39919854)	10,91	5,80	1,135498748
ORMDL3	17	complement(39921041..39927601)	18,39	11,95	0,844748868

LRRC3C	17	39927732..39944999	0,02	-	3,967268276
GSDMA	17	39953263..39977768	0,02	0,01	0,921097799
PSMD3	17	39980807..39997959	64,33	90,74	-0,273091137
CSF3	17	40015361..40017813	0,13	52,31	-8,440153669
MED24	17	complement(40019097..40061215)	15,99	19,72	-0,079151899
THRA	17	40058290..40093867	10,52	10,90	0,171943348
NR1D1	17	complement(40092793..40100589)	19,81	10,66	1,117328057
gene:ENSG000	17	complement(40113215..40115442)	0,14	0,43	-1,387171673
MSL1	17	40121971..40136917	10,22	10,93	0,126805204
CASC3	17	40140318..40172171	26,70	31,82	-0,030069265
RAPGEFL1	17	40177010..40195656	0,83	1,27	-0,378516541
WIPF2	17	40219304..40284136	6,89	7,18	0,164764703
CDC6	17	40287879..40304657	9,43	7,46	0,56041025
RARA	17	40309180..40357643	7,26	8,22	0,044238305
gene:ENSG000	17	complement(40348049..40350539)	0,54	0,65	-0,040617209
GJD3	17	complement(40360652..40364737)	0,72	0,93	-0,152522392
TOP2A	17	complement(40388525..40417896)	45,72	65,98	-0,305989482
IGFBP4	17	40443450..40457725	0,57	20,03	-4,916985018
gene:ENSG000	17	complement(40473554..40475289)	-	0,22	-7,575658083
TNS4	17	complement(40475828..40501623)	0,01	0,54	-5,260289222
CCR7	17	complement(40553769..40565472)	-	0,14	-7,525269081
gene:ENSG000	17	complement(40597113..40598585)	-	-	0,188027316
SMARCE1	17	complement(40624962..40648654)	4,46	5,65	-0,115575906
gene:ENSG000	17	complement(40627356..40665141)	0,52	1,23	-1,012595858
KRT222	17	complement(40654665..40665181)	0,00	-	3,068695355
KRT24	17	complement(40697991..40703752)	-	-	#N/D
KRT25	17	complement(40748021..40755331)	-	-	#N/D
KRT26	17	complement(40766238..40772201)	-	-	#N/D
KRT27	17	complement(40776808..40782550)	-	-	#N/D
KRT28	17	complement(40792196..40799959)	-	-	#N/D
KRT10	17	complement(40818117..40822614)	2,28	3,03	-0,18771245
KRT12	17	complement(40861303..40867223)	-	0,01	-3,804623889
KRT20	17	complement(40875889..40885242)	0,01	-	3,068695355
KRT23	17	complement(40922700..40937646)	0,00	0,00	-0,01185201
KRT39	17	complement(40958417..40966948)	-	0,01	-2,89252005
KRT40	17	complement(40977715..40987135)	-	0,01	-3,804623889
KRTAP3-3	17	complement(40993430..40994164)	-	-	#N/D
KRTAP3-2	17	complement(40999193..40999906)	-	-	0,188027316
KRTAP3-1	17	complement(41008521..41019324)	-	-	#N/D
KRTAP1-5	17	complement(41026026..41027208)	0,68	5,00	-2,64551874
KRTAP1-4	17	complement(41029351..41030195)	-	-	#N/D
KRTAP1-3	17	complement(41033884..41034874)	-	-	0,188027316
KRTAP1-1	17	complement(41040541..41041450)	-	0,01	-2,89252005
KRTAP2-1	17	complement(41046541..41047316)	-	-	0,188027316
KRTAP2-2	17	complement(41054498..41055230)	-	0,04	-3,804623889
KRTAP2-3	17	complement(41059240..41060114)	1,07	5,55	-2,146598919
KRTAP2-4	17	complement(41065116..41065879)	-	-	0,188027316
KRTAP4-7	17	41084150..41085144	-	-	0,188027316

KRTAP4-8	17	complement(41096981..41098142)	-	-	0,188027316
KRTAP4-16	17	complement(41101502..41102209)	-	-	0,188027316
KRTAP4-9	17	41105389..41106488	0,02	0,02	-0,025382928
KRTAP4-11	17	complement(41117181..41118373)	-	-	0,188027316
KRTAP4-12	17	complement(41123091..41124182)	-	-	#N/D
KRTAP4-6	17	complement(41139433..41140544)	-	-	0,188027316
KRTAP4-5	17	complement(41148924..41149825)	-	-	0,188027316
KRTAP4-4	17	complement(41159649..41160748)	-	-	#N/D
KRTAP4-3	17	complement(41167231..41168221)	-	-	#N/D
KRTAP4-2	17	complement(41177446..41178221)	-	-	#N/D
KRTAP4-1	17	complement(41184100..41184911)	-	-	#N/D
KRTAP9-1	17	41189887..41190639	-	-	#N/D
KRTAP9-2	17	41226648..41227652	-	-	#N/D
KRTAP9-3	17	41232449..41233454	-	-	#N/D
KRTAP9-8	17	41237999..41239004	-	-	#N/D
KRTAP9-4	17	41249687..41250653	-	-	#N/D
KRTAP9-9	17	41255384..41256364	-	-	#N/D
KRTAP9-6	17	41265378..41265860	-	-	#N/D
KRTAP9-7	17	41275698..41276207	-	-	#N/D
KRTAP29-1	17	complement(41301826..41302851)	-	-	#N/D
KRTAP16-1	17	complement(41307700..41309309)	0,09	0,14	-0,398057975
KRTAP17-1	17	complement(41314912..41315710)	-	0,02	-2,89252005
KRT33A	17	complement(41346092..41350828)	-	-	#N/D
KRT33B	17	complement(41363498..41369813)	-	0,02	-4,359055975
KRT34	17	complement(41377669..41382306)	0,13	3,46	-4,479597928
KRT31	17	complement(41393721..41397608)	-	-	#N/D
KRT37	17	complement(41420547..41424585)	-	-	0,188027316
KRT38	17	complement(41436154..41440983)	-	-	0,188027316
KRT32	17	complement(41459513..41467386)	0,02	0,01	1,436225396
KRT35	17	complement(41476710..41481151)	-	-	0,188027316
KRT36	17	complement(41486136..41492546)	-	-	0,188027316
KRT13	17	complement(41500981..41505705)	0,03	0,01	2,399873901
KRT15	17	complement(41513745..41522529)	1,18	1,34	0,046077442
KRT19	17	complement(41523617..41528308)	0,14	0,31	-0,915873205
KRT9	17	complement(41565836..41572059)	-	-	0,188027316
KRT14	17	complement(41582279..41586895)	-	0,02	-3,804623889
KRT16	17	complement(41609778..41615899)	0,01	0,02	-0,979341556
KRT17	17	complement(41619442..41624842)	-	0,01	-3,804623889
gene:ENSG000	17	41665566..41668031	0,01	0,03	-1,291882393
EIF1	17	41688885..41692668	85,64	#####	-0,097737097
GAST	17	41712331..41715969	-	-	#N/D
HAP1	17	complement(41717742..41734644)	0,00	0,06	-3,74602812
JUP	17	complement(41754604..41786931)	0,75	8,13	-3,210152623
P3H4	17	complement(41801947..41812604)	28,03	26,38	0,310511259
FKBP10	17	41812680..41823213	139,87	94,48	0,789068239
NT5C3B	17	complement(41825057..41836260)	9,06	16,14	-0,609093269
KLHL10	17	41835685..41848384	0,06	0,13	-0,916110795
KLHL11	17	complement(41848518..41865423)	3,94	4,50	0,032985823

ACLY	17	complement(41866917..41930542)	63,51	73,38	0,014654247
ODAD4	17	41930617..41966503	0,12	0,12	0,299673953
CNP	17	41966763..41977740	14,97	15,42	0,180798221
DNAJC7	17	complement(41976421..42021376)	6,13	8,57	-0,260938869
NKIRAS2	17	42011382..42025644	18,22	18,73	0,18289061
ZNF385C	17	complement(42025576..42098479)	0,22	0,34	-0,430778255
C17orf113	17	complement(42038232..42050601)	0,55	0,72	-0,171862735
DHX58	17	complement(42101404..42112714)	1,69	3,26	-0,725500763
KAT2A	17	complement(42113111..42121367)	21,93	33,71	-0,397015524
gene:ENSG000	17	complement(42119674..42154916)	0,04	0,17	-1,686357352
HSPB9	17	42122804..42123352	0,06	0,35	-2,302004938
RAB5C	17	complement(42124978..42155044)	33,08	38,85	-0,008875438
KCNH4	17	complement(42156891..42181142)	0,00	0,00	-0,01185201
HCRT	17	complement(42184060..42185452)	-	-	0,188027316
GHDC	17	complement(42188799..42194513)	1,44	2,23	-0,410371843
STAT5B	17	complement(42199176..42288633)	22,81	13,96	0,931685972
gene:ENSG000	17	42233832..42235469	0,09	0,05	1,165729824
STAT5A	17	42287547..42311943	4,99	4,98	0,227571314
STAT3	17	complement(42313324..42388568)	40,96	40,28	0,247060726
CAVIN1	17	complement(42402449..42423256)	210,11	#####	0,623002333
ATP6V0A1	17	42458844..42522582	5,29	5,53	0,159221787
NAGLU	17	42536241..42544449	12,62	16,82	-0,191064201
HSD17B1	17	42552922..42555214	1,05	1,63	-0,41697753
COASY	17	42561467..42566277	44,91	47,06	0,155737637
MLX	17	42567072..42573239	20,41	17,17	0,472501941
PSMC3IP	17	complement(42572310..42577831)	1,81	2,56	-0,280364104
RETREG3	17	complement(42579513..42610623)	14,34	15,23	0,136036724
gene:ENSG000	17	42598643..42599177	0,12	0,19	-0,442604891
TUBG1	17	42609641..42615238	53,19	51,78	0,261843913
TUBG2	17	42659284..42667006	8,91	10,99	-0,079511461
PLEKHH3	17	complement(42667914..42676994)	9,14	7,96	0,421768531
CCR10	17	complement(42678889..42683917)	0,20	0,61	-1,37730992
CNTNAP1	17	42682531..42699993	2,95	9,76	-1,501594957
EZH1	17	complement(42700275..42745049)	7,83	8,90	0,037762713
RAMP2	17	42758447..42763041	0,59	1,05	-0,624164101
VPS25	17	42773449..42779599	28,92	34,86	-0,046352232
WNK4	17	42780610..42797066	0,31	1,37	-1,930200809
COA3	17	complement(42795147..42798704)	8,51	8,90	0,158814563
CNTD1	17	42798800..42811587	0,30	0,33	0,089046524
BECN1	17	complement(42810134..42833350)	10,61	10,87	0,18776034
PSME3	17	42824385..42843760	13,34	21,76	-0,483150909
AOC2	17	42844580..42850707	1,82	2,32	-0,130690305
AOC3	17	42851184..42858130	0,48	0,49	0,207922016
G6PC1	17	42900797..42914438	0,01	0,01	-0,579815015
AARSD1	17	complement(42950526..42964498)	8,14	8,35	0,186215331
PTGES3L-AARS	17	complement(42950526..42980528)	-	0,09	-6,723060841
PTGES3L	17	complement(42968088..42980433)	0,02	0,10	-1,905028696
RUNDC1	17	42980565..42995142	2,34	3,21	-0,234108177

RPL27	17	42998273..43002959	33,04	51,49	-0,416668894
IFI35	17	43006740..43014456	3,12	5,12	-0,492688011
VAT1	17	complement(43014607..43025123)	71,88	88,91	-0,083634104
RND2	17	43025231..43032041	0,04	0,07	-0,749374493
BRCA1	17	complement(43044295..43170245)	3,58	6,83	-0,708186382
NBR1	17	43170481..43211689	32,30	38,92	-0,045875347
TMEM106A	17	43211835..43220041	2,74	1,53	1,070399646
CCDC200	17	complement(43216941..43305397)	0,50	0,37	0,667787711
gene:ENSG000	17	complement(43360033..43361361)	5,35	3,38	0,886433538
ARL4D	17	43398993..43401137	11,75	15,28	-0,156138493
DHX8	17	43483865..43610338	12,39	13,25	0,125530841
ETV4	17	complement(43527844..43579620)	15,00	32,88	-0,908830294
MEOX1	17	complement(43640389..43661922)	0,05	0,48	-3,082943349
SOST	17	complement(43753738..43758791)	-	0,02	-4,359055975
DUSP3	17	complement(43766125..43778977)	11,79	18,65	-0,438489001
CFAP97D1	17	43780435..43787620	0,03	0,12	-1,65413141
MPP3	17	complement(43800799..43833170)	1,58	3,36	-0,866288669
CD300LG	17	43847148..43863639	0,00	-	3,068695355
MPP2	17	complement(43875357..43909711)	1,81	1,57	0,424325413
PPY	17	complement(43940804..43942476)	-	-	#N/D
PYY	17	complement(43952733..44004469)	0,15	0,02	2,966251165
NAGS	17	44004622..44009068	0,68	0,56	0,511947981
TMEM101	17	complement(44011188..44023946)	16,12	11,34	0,73063582
LSM12	17	complement(44034328..44067619)	25,77	29,13	0,046520604
G6PC3	17	44070620..44082151	13,97	16,64	-0,029247858
HDAC5	17	complement(44076746..44123702)	11,24	8,57	0,614546395
HROB	17	44141906..44162476	6,01	6,92	0,020203872
ASB16	17	44170447..44179084	1,06	0,91	0,439086615
TMUB2	17	44186970..44191929	12,24	9,83	0,539617737
ATXN7L3	17	complement(44191805..44200113)	18,54	21,28	0,024462287
UBTF	17	complement(44205033..44221626)	7,20	10,82	-0,365032867
SLC4A1	17	complement(44248390..44268141)	0,01	0,00	1,436225396
RUNDC3A	17	44308413..44318670	0,19	0,19	0,215355996
SLC25A39	17	complement(44319625..44324870)	43,94	40,46	0,342282469
GRN	17	44345246..44353106	40,68	44,61	0,090074856
FAM171A2	17	complement(44353215..44363853)	1,55	1,07	0,757254746
ITGA2B	17	complement(44372180..44389649)	0,56	0,14	2,207206452
GPATCH8	17	complement(44395281..44503430)	10,53	11,43	0,104938422
FZD2	17	44557484..44561262	7,78	10,56	-0,217414748
MEIOC	17	44656404..44690308	0,06	0,05	0,312310631
CCDC43	17	complement(44673069..44689779)	4,20	4,80	0,029673216
gene:ENSG000	17	44689858..44693340	0,02	0,06	-1,51742441
DBF4B	17	44708608..44752264	4,26	6,51	-0,387852705
ADAM11	17	44758988..44781846	0,24	0,43	-0,60703462
GJC1	17	complement(44798448..44830816)	6,93	8,36	-0,04730084
HIGD1B	17	44846353..44850476	0,22	0,21	0,320475827
EFTUD2	17	complement(44849948..44899445)	23,78	35,43	-0,352050761
CCDC103	17	44899142..44905390	0,68	1,23	-0,625448392

GFAP	17	complement(44903159..44916937)	0,13	0,27	-0,846977025
FAM187A	17	44903433..44905390	-	-	#N/D
KIF18B	17	complement(44924709..44947773)	25,75	32,15	-0,09718583
NMT1	17	44957992..45109016	23,02	25,74	0,061863725
C1QL1	17	complement(44959693..44968303)	4,36	5,17	-0,024371412
DCAKD	17	complement(45023338..45061131)	6,76	5,86	0,429868109
PLCD3	17	complement(45108959..45133354)	6,74	8,16	-0,052412955
ACBD4	17	45132600..45144181	3,13	3,24	0,175542943
HEXIM1	17	45148475..45152099	10,87	11,84	0,099153383
HEXIM2	17	45160700..45170040	3,74	3,27	0,416162815
FMNL1	17	45221444..45247319	3,34	5,18	-0,410530043
SPATA32	17	complement(45254393..45262094)	0,04	0,03	0,438394362
MAP3K14	17	complement(45263119..45317029)	7,79	6,29	0,532178833
ARHGAP27	17	complement(45393902..45434421)	0,06	0,20	-1,635722395
PLEKHM1	17	complement(45435900..45490758)	1,92	2,67	-0,254586099
gene:ENSG000	17	45531577..45533838	0,31	0,52	-0,509032679
gene:ENSG000	17	45592621..45593369	-	-	0,188027316
LINC02210-CR	17	45620344..45835826	0,77	0,98	-0,113540316
CRHR1	17	45784277..45835828	-	-	#N/D
SPPL2C	17	45844881..45847067	-	-	#N/D
MAPT	17	45894527..46028334	0,56	1,21	-0,895682652
STH	17	45999250..45999694	-	0,06	-3,804623889
KANSL1	17	complement(46029916..46225389)	6,35	8,65	-0,222483329
ARL17B	17	complement(46274784..46361766)	0,38	0,34	0,404954294
LRRC37A	17	46292733..46338077	0,21	0,29	-0,238659261
ARL17A	17	complement(46499780..46579695)	2,94	1,45	1,240861715
LRRC37A2	17	46511508..46556910	1,32	1,28	0,262828698
NSF	17	46590669..46757679	7,63	7,73	0,203445763
WNT3	17	complement(46762506..46833154)	1,12	6,28	-2,264230747
WNT9B	17	46833201..46886730	0,00	0,01	-1,54862452
GOSR2	17	46923075..46975524	12,66	6,99	1,080720537
gene:ENSG000	17	46923133..47049932	-	0,23	-6,723060841
RPRML	17	complement(46978156..46979253)	0,09	0,20	-0,944785508
CDC27	17	complement(47117703..47189422)	8,16	12,50	-0,392372701
MYL4	17	47200446..47223679	-	-	0,188027316
ITGB3	17	47253827..47313743	2,60	9,77	-1,684884165
gene:ENSG000	17	47253897..47344292	0,18	0,35	-0,743797071
EFCAB13	17	47323290..47441312	0,18	0,44	-1,092747751
NPEPPS	17	47522942..47624665	9,93	11,95	-0,044610051
KPNB1	17	47649476..47685505	64,15	70,13	0,094333481
TBKBP1	17	47694161..47712052	3,84	4,08	0,138346734
TBX21	17	47733236..47746122	0,05	0,01	2,345408045
OSBPL7	17	complement(47807372..47821803)	10,21	5,09	1,226362274
MRPL10	17	complement(47823272..47831541)	18,75	20,79	0,074058549
LRRC46	17	47831634..47837719	0,47	0,48	0,212841929
SCRN2	17	complement(47837692..47841289)	12,08	7,10	0,989797144
SP6	17	complement(47844908..47855874)	0,09	0,05	1,175134531
SP2	17	47896150..47928957	3,68	3,94	0,124615083

PNPO	17	47941506..47949308	7,94	12,60	-0,443386913
PRR15L	17	complement(47951967..47957883)	0,36	0,36	0,239064919
CDK5RAP3	17	47967810..47981781	21,74	16,01	0,664550011
COPZ2	17	complement(48026167..48038030)	6,86	7,20	0,152387967
NFE2L1	17	48048329..48061545	59,04	#####	-0,637586865
CBX1	17	complement(48070052..48101478)	15,45	23,10	-0,357360302
SNX11	17	48103357..48123601	3,52	3,79	0,116292123
SKAP1	17	complement(48133442..48430275)	0,00	0,01	-0,923955849
HOXB1	17	complement(48528526..48531011)	-	-	0,188027316
HOXB2	17	complement(48540894..48545109)	2,95	4,67	-0,440771535
HOXB3	17	complement(48548870..48604912)	1,81	2,23	-0,078252414
HOXB4	17	complement(48575507..48578350)	3,65	4,49	-0,078683584
HOXB5	17	complement(48591257..48593779)	1,25	3,67	-1,333662337
HOXB6	17	complement(48595751..48604992)	2,49	4,38	-0,590172181
HOXB7	17	complement(48607232..48633572)	3,28	5,79	-0,596351808
HOXB8	17	complement(48611377..48615292)	0,81	2,01	-1,083335566
HOXB9	17	complement(48621156..48626358)	10,78	18,50	-0,556090112
PRAC1	17	complement(48721719..48722518)	0,25	0,34	-0,187130731
PRAC2	17	48723185..48724758	2,95	3,90	-0,181835998
HOXB13	17	complement(48724763..48728750)	1,61	3,49	-0,891791063
TTLL6	17	complement(48762234..48817229)	0,02	0,07	-1,377936944
CALCOCO2	17	48831018..48866522	12,00	14,37	-0,036894155
ATP5MC1	17	48892765..48895871	75,06	61,28	0,515750144
UBE2Z	17	48908407..48929056	32,41	36,10	0,067529764
SNF8	17	complement(48929316..48944842)	9,29	11,61	-0,099358433
GIP	17	complement(48958554..48968596)	0,03	0,09	-1,291882393
IGF2BP1	17	48997385..49056145	2,54	13,94	-2,232438345
B4GALNT2	17	49132460..49176840	0,01	0,02	-1,389560774
GNGT2	17	complement(49202791..49210574)	-	-	0,188027316
ABI3	17	49210411..49223225	0,01	0,02	-0,429837071
PHOSPHO1	17	complement(49223366..49230787)	0,14	0,22	-0,406148568
ZNF652	17	complement(49289206..49362473)	2,35	2,68	0,031534598
PHB1	17	complement(49404049..49414905)	42,97	59,40	-0,244269512
gene:ENSG000	17	complement(49494223..49497800)	0,00	0,03	-2,854330803
NGFR	17	49495293..49515008	0,15	0,13	0,399063122
NXPH3	17	49575871..49583827	0,11	0,04	1,808963148
SPOP	17	complement(49598884..49678163)	5,42	7,05	-0,155673984
SLC35B1	17	complement(49700934..49709014)	16,87	15,35	0,35912162
FAM117A	17	complement(49710332..49789180)	0,94	0,46	1,255202074
KAT7	17	49788624..49835026	5,79	6,27	0,107361694
TAC4	17	complement(49838300..49848069)	0,23	0,16	0,78195819
DLX4	17	49968588..49974959	5,70	3,19	1,05951478
DLX3	17	complement(49990005..49995224)	1,81	0,90	1,229238667
ITGA3	17	50055968..50090481	112,03	74,98	0,802394071
PDK2	17	50094737..50112152	3,06	3,12	0,19620051
SAMD14	17	complement(50110040..50130160)	0,29	0,14	1,289470552
PPP1R9B	17	complement(50133737..50150677)	30,61	24,79	0,527279282
SGCA	17	50164214..50175928	0,01	0,03	-1,291882393

COL1A1	17	complement(50184101..50201632)	262,95	#####	0,366658171
TMEM92	17	50271406..50281485	4,98	3,32	0,807469345
XYLT2	17	50346126..50363138	25,16	20,64	0,508298807
gene:ENSG000	17	complement(50362967..50365232)	0,68	0,87	-0,138739859
MRPL27	17	complement(50367857..50373207)	6,21	6,87	0,078410839
EME1	17	50373220..50381483	9,63	11,84	-0,074433374
LRRC59	17	complement(50375059..50397523)	86,95	66,75	0,604491523
ACSF2	17	50426158..50474845	9,28	10,11	0,099327738
CHAD	17	complement(50464496..50468906)	1,08	1,01	0,314478369
RSAD1	17	50478860..50485974	12,39	13,33	0,117153686
MYCBPAP	17	50508425..50531501	0,05	0,07	-0,269532571
EPN3	17	50532682..50543750	0,11	0,24	-0,854637691
SPATA20	17	50543058..50555852	15,80	15,43	0,256811031
CACNA1G	17	50560715..50627474	0,63	0,19	1,970476991
ABCC3	17	50634777..50692253	5,01	8,58	-0,552935659
ANKRD40	17	complement(50693198..50707914)	13,32	19,53	-0,32948985
LUC7L3	17	50719565..50756219	11,96	8,85	0,657675778
gene:ENSG000	17	50722074..50724224	1,07	0,66	0,918525931
ANKRD40CL	17	complement(50761029..50767557)	0,01	-	3,068695355
WFIKKN2	17	50834650..50842353	0,01	0,00	0,886720911
TOB1	17	complement(50862223..50867978)	7,83	8,31	0,13754149
SPAG9	17	complement(50962174..51120868)	14,13	12,52	0,397861024
NME1	17	51153559..51162428	25,79	38,82	-0,366759502
NME1-NME2	17	51153590..51171744	18,19	12,78	0,731858029
NME2	17	51165435..51171744	173,00	#####	0,146193719
MBTD1	17	complement(51177425..51260163)	0,97	1,89	-0,737979232
gene:ENSG000	17	complement(51183191..51186403)	0,28	0,37	-0,204352175
UTP18	17	51260546..51297936	8,54	11,38	-0,190931647
CA10	17	complement(51630313..52160017)	0,01	-	4,516772761
gene:ENSG000	17	complement(51761260..51763397)	-	-	#N/D
KIF2B	17	53822927..53825193	-	-	#N/D
TOM1L1	17	54899387..54961956	2,13	3,80	-0,612918975
COX11	17	complement(54951902..54968703)	2,83	3,30	0,001644478
gene:ENSG000	17	complement(54956760..54958426)	1,31	1,58	-0,047804001
STXBP4	17	54968727..55173632	0,53	0,65	-0,058118107
HLF	17	55264960..55325187	1,09	0,47	1,429915444
MMD	17	complement(55392622..55421924)	5,48	8,23	-0,362965845
SMIM36	17	complement(55449856..55511452)	-	-	0,188027316
TMEM100	17	complement(55719627..55732121)	0,00	0,02	-1,877914477
PCTP	17	55751051..55842830	2,87	3,44	-0,039265727
ANKFN1	17	55882301..56517016	0,34	0,08	2,265635306
gene:ENSG000	17	56471142..56473647	0,00	0,01	-0,01185201
NOG	17	56593699..56595611	0,28	0,42	-0,34981301
C17orf67	17	complement(56791913..56838773)	0,61	0,97	-0,442622625
DGKE	17	56834107..56869567	0,90	1,12	-0,089013946
TRIM25	17	complement(56836387..56914080)	17,28	17,17	0,232069165
COIL	17	complement(56938199..56961050)	4,78	6,04	-0,112421743
SCPEP1	17	56978129..57006768	10,88	9,94	0,353593051

AKAP1	17	57085092..57121346	5,33	10,21	-0,714810519
MSI2	17	57255851..57684689	3,54	5,11	-0,306661389
gene:ENSG000	17	57277920..57280017	0,25	0,39	-0,454224829
gene:ENSG000	17	57448218..57450291	0,09	0,15	-0,533146901
gene:ENSG000	17	57531769..57532355	0,20	0,24	-0,037899139
CCDC182	17	complement(57744495..57745299)	-	-	#N/D
MRPS23	17	complement(57834781..57850056)	3,47	4,02	0,007303185
CUEDC1	17	complement(57861243..57955412)	7,62	9,45	-0,088627625
VEZF1	17	complement(57971547..57988259)	8,48	11,89	-0,263792616
gene:ENSG000	17	57988180..57988760	0,32	0,27	0,458861392
gene:ENSG000	17	complement(57989038..58007246)	9,13	13,63	-0,353894131
SRSF1	17	complement(58000919..58007346)	18,12	17,71	0,256758158
gene:ENSG000	17	58006652..58009596	1,02	1,15	0,055403717
DYNLL2	17	58083419..58095542	8,34	7,27	0,421659632
gene:ENSG000	17	58095672..58101479	0,90	0,66	0,687030322
OR4D1	17	58148384..58159585	0,00	-	3,967268276
OR4D2	17	58166982..58171411	0,01	-	3,068695355
EPX	17	58192726..58205174	0,05	0,05	0,221705268
MKS1	17	complement(58205441..58219605)	1,43	2,37	-0,501497
LPO	17	58218548..58268518	0,00	0,00	-0,01185201
MPO	17	complement(58269855..58280935)	-	-	#N/D
TSPOAP1	17	complement(58301228..58328795)	0,52	0,01	6,850852299
SUPT4H1	17	complement(58345175..58353093)	15,95	17,36	0,101058367
gene:ENSG000	17	complement(58345206..58417595)	0,01	0,05	-2,453497736
RNF43	17	complement(58353676..58417595)	0,01	0,12	-2,986377451
HSF5	17	complement(58420167..58488408)	0,00	0,01	-0,923955849
MTMR4	17	complement(58489529..58517905)	10,82	10,75	0,233161036
SEPTIN4	17	complement(58520250..58544368)	0,28	0,51	-0,628636428
TEX14	17	complement(58556678..58692055)	0,03	0,04	-0,398057975
IGBP1C	17	complement(58660424..58692045)	-	-	0,188027316
RAD51C	17	58692573..58735611	0,75	1,21	-0,458201874
PPM1E	17	58755854..58985179	0,25	0,33	-0,195415915
TRIM37	17	complement(58982638..59106921)	3,38	6,96	-0,817961548
SKA2	17	complement(59109857..59155260)	3,50	5,06	-0,308448777
PRR11	17	59155732..59206709	23,55	28,34	-0,044023598
gene:ENSG000	17	59197566..59215226	-	-	0,188027316
SMG8	17	59209400..59215239	3,50	6,09	-0,574238647
GDPD1	17	59220467..59275970	0,19	0,45	-1,003496157
YPEL2	17	59331655..59401729	0,61	0,55	0,380308616
gene:ENSG000	17	59362665..59363278	-	-	0,188027316
DHX40	17	59565558..59608345	9,28	12,69	-0,227557522
CLTC	17	59619680..59696956	65,18	56,06	0,440511218
PTRH2	17	complement(59674636..59707626)	5,68	4,19	0,66100589
VMP1	17	59707192..59842255	21,52	24,83	0,016701463
TUBD1	17	complement(59859479..59892945)	1,31	2,18	-0,508667795
RPS6KB1	17	59893046..59950574	2,22	3,34	-0,365738021
gene:ENSG000	17	59940908..59973101	-	0,11	-4,758582517
RNFT1	17	complement(59952240..59964761)	0,55	1,24	-0,934838491

HEATR6	17	complement(60041008..60078922)	3,20	6,98	-0,902162457
CA4	17	60149942..60170899	-	0,01	-3,804623889
USP32	17	complement(60177327..60422470)	7,22	7,96	0,082112142
CHCT1	17	60392429..60431426	0,01	-	3,068695355
APPBP2	17	complement(60443158..60526242)	2,90	3,50	-0,051528565
PPM1D	17	60600193..60666280	0,73	1,10	-0,361429961
BCAS3	17	60677453..61392838	2,76	2,48	0,374512864
TBX2	17	61399843..61409466	1,11	1,75	-0,426967149
TBX4	17	61452404..61485110	0,02	0,01	0,939616118
NACA2	17	complement(61590421..61591219)	-	-	0,188027316
BRIP1	17	complement(61679139..61863559)	0,57	1,02	-0,615166243
INTS2	17	complement(61865367..61928016)	2,33	3,22	-0,24415619
MED13	17	complement(61942605..62065278)	7,05	9,28	-0,173896774
gene:ENSG000	17	complement(62003700..62007518)	0,64	0,64	0,222127678
EFCAB3	17	62343941..62416480	-	-	0,188027316
METTL2A	17	62423875..62453385	2,78	4,35	-0,421490646
TLK2	17	62458658..62615481	2,77	4,55	-0,495191403
MRC2	17	62627670..62693597	59,86	35,04	0,995642292
gene:ENSG000	17	complement(62679403..62681354)	3,08	1,83	0,971832408
MARCHF10	17	complement(62701314..62808344)	0,01	0,04	-1,806405564
TANC2	17	62966235..63427703	3,12	7,25	-0,995794808
CYB561	17	complement(63432304..63446354)	23,85	22,08	0,334114947
ACE	17	63477061..63498380	0,64	0,06	3,668724181
gene:ENSG000	17	63484823..63521848	-	-	0,188027316
KCNH6	17	63523334..63548977	0,03	0,04	-0,297455117
DCAF7	17	63550222..63594286	24,11	22,64	0,313825652
gene:ENSG000	17	63550603..63608347	2,75	3,55	-0,143982144
TACO1	17	63600882..63608365	5,69	10,48	-0,657504775
MAP3K3	17	63622415..63696305	10,19	8,20	0,537203572
STRADA	17	complement(63682336..63741986)	4,89	5,52	0,049716632
LIMD2	17	complement(63695888..63701172)	1,73	2,28	-0,172447159
gene:ENSG000	17	63700847..63702670	0,24	0,39	-0,498171294
gene:ENSG000	17	complement(63702845..63752097)	0,12	-	7,391550995
CCDC47	17	complement(63745255..63776351)	7,67	8,88	0,013127411
DDX42	17	63773603..63819317	18,94	16,65	0,408995139
FTSJ3	17	complement(63819433..63830012)	6,86	7,08	0,177248158
PSMC5	17	63827152..63832026	20,57	29,89	-0,315891117
SMARCD2	17	complement(63832081..63843065)	20,45	24,17	-0,017571379
CSH2	17	complement(63872012..63873766)	-	-	#N/D
GH2	17	complement(63880215..63881944)	-	-	#N/D
CSH1	17	complement(63894909..63896661)	-	-	0,188027316
CSHL1	17	complement(63909597..63911341)	-	-	#N/D
GH1	17	complement(63917200..63918839)	-	-	#N/D
gene:ENSG000	17	complement(63917203..63931385)	-	-	#N/D
CD79B	17	complement(63928738..63932336)	-	0,01	-2,89252005
SCN4A	17	complement(63938554..63972918)	0,00	0,00	1,436225396
PRR29	17	63998351..64004305	0,05	-	6,780090526
ICAM2	17	complement(64002594..64020634)	0,01	0,01	0,886720911

ERN1	17	complement(64039080..64130819)	0,68	1,62	-1,033196782
TEX2	17	complement(64147227..64263260)	2,84	5,10	-0,619714854
PECAM1	17	complement(64319415..64413776)	0,36	0,81	-0,950952889
MILR1	17	64449037..64468643	2,44	3,79	-0,410269216
POLG2	17	complement(64477785..64497054)	2,84	3,06	0,117177498
DDX5	17	complement(64498254..64508199)	25,24	30,56	-0,052817999
CEP95	17	64506865..64542461	2,73	3,19	-0,003561013
SMURF2	17	complement(64542282..64662307)	6,66	8,33	-0,100678129
LRRC37A3	17	complement(64854130..64919480)	0,40	0,78	-0,717903164
GNA13	17	complement(65009289..65056740)	4,96	6,59	-0,186275873
RGS9	17	65100812..65227703	0,88	0,81	0,338849474
AXIN2	17	complement(65528563..65561648)	0,92	1,57	-0,545529597
gene:ENSG000	17	complement(65538102..65641033)	-	-	0,188027316
CEP112	17	complement(65635537..66192133)	0,68	1,09	-0,44231032
APOH	17	complement(66212033..66256525)	-	-	#N/D
PRKCA	17	66302613..66810743	5,64	9,99	-0,600238023
CACNG5	17	66835117..66894751	0,00	0,00	-0,025382928
CACNG4	17	66964707..67033398	0,70	2,11	-1,365880025
CACNG1	17	67044554..67056797	0,02	0,01	0,886720911
HELZ	17	complement(67070444..67245989)	3,97	4,56	0,024092888
PSMD12	17	complement(67337916..67366605)	5,71	8,80	-0,401604687
PITPNC1	17	67377281..67697256	1,81	2,58	-0,284676817
gene:ENSG000	17	67611277..67613822	0,00	0,03	-2,447197442
gene:ENSG000	17	67637774..67638973	0,07	0,09	-0,036800208
NOL11	17	67717931..67744531	6,10	10,79	-0,600691236
BPTF	17	67825503..67984378	4,69	6,85	-0,322385781
gene:ENSG000	17	67955314..67957291	0,17	0,26	-0,406148568
gene:ENSG000	17	67973934..67976072	0,13	0,16	-0,093784646
C17orf58	17	complement(67991099..67996469)	1,05	2,22	-0,854278172
KPNA2	17	68035636..68047364	64,11	95,83	-0,356869533
AMZ2	17	68205481..68257712	9,05	12,53	-0,247085852
ARSG	17	68259182..68422731	2,50	5,46	-0,903928656
SLC16A6	17	complement(68267026..68291267)	1,77	20,27	-3,297846323
WIPI1	17	complement(68420948..68457513)	17,22	13,04	0,624915328
PRKAR1A	17	68511780..68551319	18,68	16,72	0,383117299
FAM20A	17	complement(68535113..68601367)	0,41	0,33	0,520679882
ABCA8	17	complement(68867289..68955392)	0,01	0,00	0,921097799
ABCA9	17	complement(68974488..69060949)	0,01	0,14	-3,319051466
ABCA6	17	complement(69078702..69141895)	0,19	1,24	-2,467244157
ABCA10	17	complement(69147214..69244846)	0,31	2,34	-2,704347973
PRO1804	17	complement(69232963..69235012)	0,01	0,02	-0,579815015
ABCA5	17	complement(69244311..69327244)	0,17	0,39	-0,928609494
MAP2K6	17	69414697..69553865	0,25	0,16	0,846683076
gene:ENSG000	17	69433084..69435533	0,00	0,02	-1,877914477
KCNJ16	17	70053429..70135608	-	0,00	-2,89252005
KCNJ2	17	70168673..70180044	0,91	0,26	2,059123346
SOX9	17	72121020..72126416	10,28	5,65	1,087433574
SLC39A11	17	complement(72645949..73092712)	2,75	2,31	0,474334845

SSTR2	17	73165010..73176633	0,07	0,13	-0,688363716
COG1	17	73193055..73208507	14,41	11,64	0,531605514
VCF1	17	complement(73207353..73236753)	4,80	5,87	-0,066967844
C17orf80	17	73232233..73248959	2,18	3,36	-0,401846767
CPSF4L	17	complement(73248449..73262352)	0,06	0,09	-0,489732438
CDC42EP4	17	complement(73283624..73312005)	17,92	16,63	0,330634694
SDK2	17	complement(73334384..73644445)	0,48	0,05	3,551508706
RPL38	17	74203582..74210655	45,92	54,35	-0,019947275
TTYH2	17	74213571..74262020	0,44	0,47	0,117002276
DNAI2	17	74274234..74314884	-	0,01	-2,89252005
KIF19	17	74326210..74355820	0,00	-	3,068695355
BTBD17	17	complement(74356416..74361868)	-	-	#N/D
GPR142	17	74367458..74372600	-	-	#N/D
GPRC5C	17	74424851..74451653	2,32	3,36	-0,3133587
CD300A	17	74466399..74484794	-	-	0,188027316
CD300LB	17	complement(74521174..74531475)	0,00	0,00	-0,01185201
CD300C	17	complement(74541073..74546115)	0,01	0,02	-0,025382928
CD300H	17	complement(74560701..74567343)	-	0,04	-3,804623889
CD300LD	17	complement(74579365..74592283)	-	-	0,188027316
CD300E	17	complement(74609885..74623738)	0,00	-	3,068695355
RAB37	17	74670578..74747335	0,03	0,02	0,415033091
CD300LF	17	complement(74694311..74712978)	-	-	#N/D
NHERF1	17	74748628..74769353	53,56	27,03	1,209762101
NAT9	17	complement(74770529..74776367)	16,92	10,29	0,940547649
TMEM104	17	74776499..74839753	14,55	5,10	1,736501202
GRIN2C	17	complement(74842023..74861504)	0,18	0,05	2,197986293
FDXR	17	complement(74862497..74873031)	2,35	2,66	0,040311902
FADS6	17	complement(74877302..74893781)	0,01	0,00	0,886720911
USH1G	17	complement(74916083..74923256)	0,01	0,01	0,366665713
OTOP2	17	74922950..74933912	0,01	0,01	0,886720911
OTOP3	17	74935898..74949992	-	-	#N/D
HID1	17	complement(74950742..74973166)	0,00	0,00	-0,01185201
CDR2L	17	74987632..75005800	5,79	9,31	-0,463458234
MRPL58	17	75012670..75021261	10,36	16,89	-0,481987165
KCTD2	17	75032575..75065889	5,36	6,20	0,013522258
ATP5PD	17	complement(75038863..75046985)	45,20	53,47	-0,019067289
SLC16A5	17	75087727..75106162	1,36	5,37	-1,757635611
ARMC7	17	75109952..75130272	1,62	3,57	-0,915067711
NT5C	17	complement(75130225..75131757)	5,11	13,43	-1,169332507
gene:ENSG000	17	75130883..75133043	0,36	1,08	-1,340883926
JPT1	17	complement(75135248..75168281)	19,09	27,07	-0,280832887
SUMO2	17	complement(75165586..75182959)	13,02	16,73	-0,138835925
NUP85	17	75205659..75235758	11,23	10,12	0,372883434
GGA3	17	complement(75236599..75262363)	8,75	12,05	-0,238392592
MRPS7	17	75261674..75266376	8,59	12,47	-0,313722249
MIF4GD	17	complement(75266228..75271231)	4,16	9,64	-0,987161803
SLC25A19	17	complement(75272981..75289510)	2,43	6,34	-1,161649252
GRB2	17	complement(75318076..75405709)	24,77	24,27	0,252112673

TMEM94	17	75441159..75500452	11,31	12,07	0,128574261
CASKIN2	17	complement(75500261..75515537)	4,98	5,32	0,128973637
TSEN54	17	75515944..75524735	3,69	5,88	-0,448864569
LLGL2	17	75525080..75575209	0,21	0,40	-0,705090729
MYO15B	17	75587800..75626849	0,60	0,37	0,937906181
RECQL5	17	complement(75626845..75667189)	5,94	6,62	0,067677298
SMIM5	17	75633434..75641404	0,43	0,58	-0,200345712
SMIM6	17	75646243..75647975	0,38	0,53	-0,271997407
SAP30BP	17	75667251..75708062	5,88	7,68	-0,160905677
ITGB4	17	75721328..75757818	0,64	4,58	-2,625969436
GALK1	17	complement(75751594..75765236)	9,06	10,23	0,04719206
H3-3B	17	complement(75776434..75785893)	38,26	65,15	-0,545076434
UNK	17	75784806..75825799	7,07	9,01	-0,126478741
UNC13D	17	complement(75827225..75844785)	0,17	0,31	-0,598327422
WBP2	17	complement(75845699..75856507)	33,97	42,30	-0,093390644
TRIM47	17	complement(75874164..75878581)	6,11	13,20	-0,887256144
TRIM65	17	complement(75880335..75896951)	11,15	18,74	-0,526232309
MRPL38	17	complement(75898644..75905093)	28,82	35,72	-0,086638555
gene:ENSG000	17	complement(75898645..75930129)	0,13	0,34	-1,119553623
FBF1	17	complement(75909574..75941140)	3,00	4,60	-0,395093347
ACOX1	17	complement(75941507..75979177)	5,59	4,99	0,386049405
TEN1-CDK3	17	75979231..76005999	0,07	0,19	-1,169203102
TEN1	17	75979240..76000586	3,55	4,70	-0,182775426
CDK3	17	76000855..76005999	2,33	2,58	0,079139562
EVPL	17	complement(76004502..76027306)	-	0,01	-4,359055975
SRP68	17	complement(76038775..76072517)	23,95	30,63	-0,13170164
ZACN	17	76071961..76083666	0,30	0,34	0,057286266
GALR2	17	76074781..76077537	-	0,01	-2,89252005
EXOC7	17	complement(76081016..76121576)	8,92	11,60	-0,156918362
gene:ENSG000	17	76090308..76091669	1,85	3,04	-0,492082273
FOXJ1	17	complement(76136333..76141245)	0,01	0,01	-0,025382928
RNF157	17	complement(76142465..76240493)	1,27	2,27	-0,610070907
UBALD2	17	76265348..76271298	24,81	31,87	-0,138201109
QRICH2	17	complement(76274049..76308276)	0,16	0,56	-1,559985896
PRPSAP1	17	complement(76309478..76384521)	4,01	6,07	-0,374172437
SPHK1	17	76376584..76387860	3,67	7,36	-0,780702837
UBE2O	17	complement(76389456..76453152)	16,91	16,50	0,25864968
AANAT	17	76453351..76470117	0,19	0,22	-0,000603959
RHBDF2	17	complement(76470891..76501790)	11,75	10,43	0,394658392
CYGB	17	complement(76527356..76551175)	6,01	2,83	1,309997546
PRCD	17	76527586..76553578	0,28	0,57	-0,810308151
gene:ENSG000	17	complement(76563710..76569671)	33,66	30,75	0,35345215
ST6GALNAC2	17	complement(76565377..76586956)	0,39	0,03	3,976784667
ST6GALNAC1	17	complement(76624761..76643786)	0,02	-	5,224730805
MXRA7	17	complement(76672551..76711004)	7,31	6,84	0,319120825
JMJD6	17	complement(76712832..76726799)	12,24	5,49	1,381060591
METTL23	17	76726830..76733936	5,89	6,57	0,066492699
gene:ENSG000	17	76732978..76738522	0,05	0,22	-1,955757882

SRSF2	17	complement(76734115..76737333)	25,81	36,60	-0,280499503
MFSD11	17	76735865..76781449	2,71	3,02	0,064697867
MGAT5B	17	76868404..76950393	4,60	4,28	0,325068704
gene:ENSG000	17	complement(76966285..76969530)	0,02	0,01	1,232079601
SEC14L1	17	77088749..77217101	17,31	17,81	0,182003045
SEPTIN9	17	77280569..77500596	63,79	50,51	0,559834779
gene:ENSG000	17	77444704..77447167	0,89	0,68	0,608798265
gene:ENSG000	17	77571418..77572516	-	-	#N/D
TNRC6C	17	77958703..78108822	0,98	1,54	-0,42453227
gene:ENSG000	17	78008421..78008855	-	0,09	-4,359055975
TMC6	17	complement(78107397..78132407)	1,72	1,77	0,187873098
TMC8	17	78130770..78142968	0,05	0,08	-0,543107834
C17orf99	17	78146385..78166297	0,04	-	4,913749003
SYNGR2	17	78168581..78173527	34,77	42,13	-0,053900287
TK1	17	complement(78174091..78187233)	75,56	52,08	0,759921668
AFMID	17	78187358..78207702	8,17	7,15	0,41389165
BIRC5	17	78214186..78225636	45,27	51,47	0,038011321
TMEM235	17	78231310..78240987	0,01	0,00	0,886720911
gene:ENSG000	17	complement(78250346..78250932)	0,22	0,07	1,914812876
SOCS3	17	complement(78356778..78360077)	11,90	14,31	-0,043260786
PGS1	17	78378649..78425114	18,02	18,61	0,176348176
DNAH17	17	complement(78423697..78577396)	0,19	0,23	-0,028879182
CYTH1	17	complement(78674048..78782297)	3,42	3,91	0,030853327
gene:ENSG000	17	complement(78691266..78691979)	0,12	0,09	0,627075281
USP36	17	complement(78787381..78841441)	14,76	10,05	0,778205514
TIMP2	17	complement(78852977..78925387)	47,90	51,40	0,121373906
CEP295NL	17	complement(78890579..78903217)	0,45	0,60	-0,199971113
gene:ENSG000	17	78897264..78900152	0,37	0,48	-0,180539264
LGALS3BP	17	complement(78971238..78979947)	59,63	#####	-0,661380129
CANT1	17	complement(78991716..79009867)	20,55	24,15	-0,009917633
C1QTNF1	17	79022814..79049788	7,73	29,42	-1,704614729
ENGASE	17	79074824..79088599	12,33	10,32	0,479748679
RBFOX3	17	complement(79089345..79611051)	0,62	0,22	1,707078117
ENPP7	17	79730943..79742219	0,00	-	3,068695355
CBX2	17	79778148..79787983	5,28	6,44	-0,062679046
CBX8	17	complement(79792132..79801683)	4,81	2,97	0,917352237
CBX4	17	complement(79833156..79839440)	7,76	8,73	0,052284288
TBC1D16	17	complement(79932343..80035872)	13,44	14,78	0,086921772
CCDC40	17	80036632..80100613	0,53	0,67	-0,113870006
GAA	17	80101533..80119881	10,92	8,96	0,50811994
EIF4A3	17	complement(80134369..80147151)	21,57	26,32	-0,063905511
gene:ENSG000	17	80147250..80148596	1,19	1,06	0,39668911
CARD14	17	80169992..80216073	0,59	1,10	-0,665298926
SGSH	17	complement(80206716..80220923)	7,52	10,16	-0,21200376
SLC26A11	17	80219699..80253500	3,37	3,69	0,095321273
RNF213	17	80260852..80398794	11,27	11,97	0,13559996
gene:ENSG000	17	80339898..80342058	0,36	0,67	-0,644113969
ENDOV	17	80415165..80438086	2,78	1,54	1,07499899

gene:ENSG000	17	complement(80463451..80465593)	0,04	0,01	2,616997665
NPTX1	17	complement(80466834..80477843)	8,12	0,42	4,498376388
RPTOR	17	80544819..80966371	28,21	18,08	0,865120074
gene:ENSG000	17	80545075..80545134	-	-	#N/D
CHMP6	17	80991598..81009517	5,96	8,01	-0,204995908
gene:ENSG000	17	81003335..81005132	1,06	0,53	1,207771341
BAIAP2	17	81035122..81117434	1,73	4,03	-0,995624283
AATK	17	complement(81110487..81166221)	0,18	0,39	-0,89946238
PVALEF	17	81165507..81183166	-	-	#N/D
CEP131	17	complement(81189593..81222999)	4,77	3,61	0,624201842
TEPSIN	17	complement(81228277..81239091)	3,22	4,61	-0,292172184
NDUFAF8	17	81239305..81241310	3,79	4,57	-0,047907483
SLC38A10	17	complement(81244811..81295547)	11,07	14,64	-0,179913877
gene:ENSG000	17	81262920..81265401	0,40	0,37	0,368667711
BAHCC1	17	81395457..81466331	7,34	8,72	-0,026695884
gene:ENSG000	17	complement(81395609..81397144)	0,11	0,23	-0,88099304
ACTG1	17	complement(81509413..81523847)	839,45	#####	0,711920993
FSCN2	17	81528377..81537130	0,24	0,40	-0,508145198
FAAP100	17	complement(81539885..81553961)	8,66	8,16	0,308897074
NPLOC4	17	complement(81556887..81648465)	18,02	25,37	-0,27063605
TSPAN10	17	81637171..81648754	0,25	1,22	-2,090930717
PDE6G	17	complement(81650459..81663112)	0,26	0,62	-1,007964297
OXLD1	17	complement(81665036..81666635)	4,92	10,08	-0,811371957
CCDC137	17	81666737..81673904	11,45	10,69	0,322373078
ARL16	17	complement(81681187..81683797)	13,08	10,82	0,496631784
HGS	17	81683326..81703138	22,21	27,03	-0,060257183
MRPL12	17	81703367..81707517	41,56	84,68	-0,803702159
gene:ENSG000	17	81703371..81720539	1,27	1,41	0,071497499
SLC25A10	17	81712236..81721016	7,22	17,51	-1,054667131
GCGR	17	81804132..81814008	0,01	0,04	-1,955757882
MCRIP1	17	complement(81822361..81833302)	6,16	4,56	0,656435672
PPP1R27	17	complement(81833492..81835050)	0,51	0,54	0,160812297
P4HB	17	complement(81843159..81860856)	153,12	96,07	0,895576018
gene:ENSG000	17	81862778..81863102	9,62	5,06	1,14960075
ARHGDIA	17	complement(81867721..81871378)	98,12	#####	-0,08643963
ALYREF	17	complement(81887835..81891586)	38,93	50,07	-0,13975124
ANAPC11	17	81890790..81900991	8,24	8,89	0,11502881
NPB	17	81900745..81902905	0,09	0,55	-2,384082833
PCYT2	17	complement(81900958..81911432)	3,87	2,91	0,634805666
SIRT7	17	complement(81911939..81921323)	4,52	5,65	-0,099077028
MAFG	17	complement(81918270..81927735)	19,24	13,01	0,787637392
PYCR1	17	complement(81932384..81942412)	47,08	34,03	0,691236495
MYADML2	17	complement(81939645..81947233)	0,03	0,05	-0,759769633
NOTUM	17	complement(81952507..81961840)	1,42	0,86	0,958620014
ASPSCR1	17	81976807..82017406	7,82	9,29	-0,024728856
CENPX	17	complement(82018702..82024107)	21,48	17,47	0,521317748
LRRC45	17	82023305..82031151	5,26	4,19	0,54924983
RAC3	17	82031678..82034204	6,37	5,37	0,471150019

DCXR	17	complement(82035136..82037709)	23,71	19,86	0,478194891
gene:ENSG000	17	complement(82035403..82035800)	0,24	0,78	-1,438989101
RFNG	17	complement(82047902..82051831)	11,89	11,36	0,28909212
GPS1	17	82050691..82057470	25,35	29,28	0,015543816
DUS1L	17	complement(82057506..82065887)	14,00	16,22	0,010032816
FASN	17	complement(82078338..82098294)	113,05	60,18	1,132729141
CCDC57	17	complement(82101460..82212878)	1,63	2,46	-0,370176818
SLC16A3	17	82217934..82261129	62,50	39,52	0,884438816
CSNK1D	17	complement(82239023..82273700)	26,33	29,94	0,037321236
gene:ENSG000	17	82249067..82251844	3,74	3,08	0,501945361
CD7	17	complement(82314868..82317608)	0,18	0,05	1,981046522
SECTM1	17	complement(82321024..82334074)	3,27	1,93	0,980375713
TEX19	17	82359247..82363775	1,30	2,05	-0,427569662
gene:ENSG000	17	complement(82362349..82363196)	-	-	0,188027316
UTS2R	17	82371765..82377458	0,08	0,07	0,423394911
OGFOD3	17	complement(82389210..82418637)	4,37	6,15	-0,268140584
HEXD	17	82418318..82442645	1,50	3,43	-0,974081917
CYBC1	17	complement(82440912..82450829)	6,27	7,97	-0,123811797
NARF	17	82458180..82490537	4,74	3,97	0,478927535
gene:ENSG000	17	82462601..82464255	1,63	0,81	1,237430656
FOXK2	17	82519713..82644662	14,96	17,92	-0,037467238
WDR45B	17	complement(82614562..82648553)	32,96	31,32	0,296752331
gene:ENSG000	17	82648849..82650657	0,11	0,11	0,206887575
RAB40B	17	complement(82654973..82698698)	1,02	1,12	0,092419203
FN3KRP	17	82716706..82730328	7,05	7,51	0,132060629
FN3K	17	82735615..82751196	1,87	3,17	-0,535278384
TBCD	17	82752042..82945914	4,68	6,00	-0,13273359
ZNF750	17	complement(82829434..82840022)	0,29	0,24	0,526703266
B3GNTL1	17	complement(82942149..83051809)	1,60	2,39	-0,353478994
METRNL	17	83079609..83095122	16,02	18,88	-0,013660938
gene:ENSG000	17	complement(83132647..83136150)	0,01	-	4,913749003
TUBB8B	18	complement(47221..73545)	-	-	#N/D
USP14	18	158383..214629	7,59	10,87	-0,295292092
THOC1	18	complement(214520..268050)	1,34	3,24	-1,052343828
COLEC12	18	complement(316737..500722)	11,95	27,48	-0,978018773
CETN1	18	580380..582114	-	-	#N/D
CLUL1	18	596988..650334	0,01	0,01	-0,025382928
TYMS	18	657653..673578	26,86	35,10	-0,16301655
ENOSF1	18	complement(670318..712662)	1,14	1,40	-0,067074862
YES1	18	complement(721588..812546)	6,77	8,52	-0,108478876
ADCYAP1	18	904871..912172	-	-	#N/D
METTL4	18	complement(2537525..2571509)	1,61	1,69	0,145629144
NDC80	18	2571557..2616635	3,76	5,22	-0,250781136
SMCHD1	18	2655726..2805017	1,64	2,74	-0,52251364
EMILIN2	18	2847006..2916003	1,09	2,42	-0,934504041
LPIN2	18	complement(2885296..3013144)	5,91	9,65	-0,482714888
MYOM1	18	complement(3066807..3219968)	0,15	0,43	-1,3495968
MYL12A	18	3247481..3256236	61,28	55,53	0,36520684

MYL12B	18	3261479..3278461	38,05	45,23	-0,025983862
TGIF1	18	3411608..3459978	3,28	2,72	0,492075573
DLGAP1	18	complement(3496032..4455307)	0,00	0,00	-0,923955849
AKAIN1	18	complement(5142911..5197503)	0,00	-	3,068695355
ZBTB14	18	complement(5289019..5297053)	1,61	2,24	-0,258823986
EPB41L3	18	complement(5392381..5630700)	-	0,00	-4,359055975
TMEM200C	18	complement(5882072..5895955)	-	-	0,188027316
L3MBTL4	18	complement(5954706..6415237)	0,01	0,00	2,972512804
gene:ENSG000	18	complement(6394847..6398020)	-	-	0,188027316
gene:ENSG000	18	6727053..6728981	-	-	0,188027316
ARHGAP28	18	6729716..6915716	0,06	0,02	1,949409852
LAMA1	18	complement(6941742..7117797)	0,08	0,39	-2,120687061
LRRC30	18	7231089..7232044	-	-	#N/D
PTPRM	18	7566782..8406861	4,52	3,96	0,414488817
RAB12	18	8609437..8639383	5,60	7,70	-0,235252805
MTCL1	18	8705556..8832778	3,40	3,82	0,054008837
NDUFV2	18	9102630..9134345	21,41	20,80	0,264638847
ANKRD12	18	9136228..9285985	0,95	1,09	0,034064554
TWSG1	18	9334767..9402420	7,10	7,37	0,169868468
RALBP1	18	9475009..9538114	5,34	6,27	-0,008273115
PPP4R1	18	complement(9546791..9615240)	4,74	7,38	-0,416398181
RAB31	18	9708275..9862551	17,06	15,75	0,338027246
TXNDC2	18	9885766..9889275	0,09	0,10	0,016676551
VAPA	18	9914016..9960021	4,86	8,34	-0,556929757
APCDD1	18	10454635..10489949	0,01	-	5,480421266
NAPG	18	10525905..10552764	3,98	4,34	0,096242448
PIEZO2	18	complement(10666483..11149569)	4,97	2,52	1,201418261
SLC35G4	18	11609596..11610612	-	-	#N/D
GNAL	18	11689264..11885685	0,42	0,59	-0,269879659
CHMP1B	18	11851413..11854444	8,03	7,75	0,273547787
MPPE1	18	complement(11882622..11908366)	2,30	2,15	0,32188678
IMPA2	18	11981025..12030877	8,14	6,21	0,614075106
ANKRD62	18	12093843..12129764	-	0,00	-2,89252005
gene:ENSG000	18	12199345..12200089	-	-	0,188027316
CIDEA	18	12254361..12277595	-	-	0,188027316
gene:ENSG000	18	12279427..12282739	0,00	-	3,068695355
TUBB6	18	12307669..12344320	19,21	23,33	-0,057109368
AFG3L2	18	complement(12328944..12377227)	9,26	14,75	-0,449220889
PRELID3A	18	12407896..12432238	2,44	2,99	-0,071898666
SPIRE1	18	complement(12446512..12658107)	8,52	10,22	-0,038462526
PSMG2	18	12658043..12728945	3,60	4,35	-0,049459337
CEP76	18	complement(12661833..12702777)	0,38	1,00	-1,186514427
PTPN2	18	complement(12785478..12929643)	1,61	3,39	-0,846461895
SEH1L	18	12947133..12987536	4,09	10,29	-1,10729098
CEP192	18	12991362..13125052	2,86	4,02	-0,26769147
LDLRAD4	18	13217498..13652755	0,72	0,07	3,520931943
C18orf15	18	13239543..13242076	0,09	0,02	2,714155068
gene:ENSG000	18	complement(13279415..13280110)	0,12	-	5,88622595

FAM210A	18	complement(13663347..13726663)	2,25	2,85	-0,114175762
gene:ENSG000	18	complement(13710249..13710484)	0,14	0,16	-0,03031053
RNMT	18	13726660..13764556	5,90	5,26	0,390038976
MC5R	18	13824149..13827323	-	-	0,188027316
MC2R	18	complement(13882044..13915707)	-	-	#N/D
gene:ENSG000	18	14010054..14010917	0,42	0,18	1,452240688
ZNF519	18	complement(14057457..14132490)	0,59	0,58	0,230191639
POTEC	18	complement(14507339..14543600)	0,00	0,00	-0,01185201
ANKRD30B	18	14728272..14854667	0,01	0,01	0,366665713
gene:ENSG000	18	complement(14990707..14993235)	0,01	0,01	0,886720911
ROCK1	18	complement(20944612..21111813)	2,35	3,32	-0,274393543
gene:ENSG000	18	complement(21111059..21111139)	-	-	#N/D
GREB1L	18	21242232..21526112	0,32	0,85	-1,169155714
ESCO1	18	complement(21529284..21600884)	1,18	2,58	-0,905075624
SNRPD1	18	21612314..21633524	2,71	6,34	-1,001283236
ABHD3	18	complement(21650901..21704780)	0,79	2,65	-1,527037694
MIB1	18	21704957..21870953	6,23	9,60	-0,400252484
GATA6	18	22169589..22202528	1,71	4,51	-1,177720282
CTAGE1	18	complement(22413599..22417915)	0,00	-	3,068695355
RBBP8	18	22798261..23026488	3,12	7,44	-1,032061659
CABLES1	18	23134564..23260470	6,17	3,70	0,962152966
TMEM241	18	complement(23197144..23437961)	4,04	6,89	-0,545977188
RIOK3	18	23453287..23486603	6,64	10,13	-0,386882943
RMC1	18	23503496..23531822	5,67	10,66	-0,686936379
NPC1	18	complement(23506184..23586506)	9,84	19,95	-0,796506869
ANKRD29	18	complement(23598926..23662911)	0,47	2,98	-2,446269514
LAMA3	18	23689453..23956222	0,67	3,94	-2,329350642
TTC39C	18	23992773..24135610	0,56	2,27	-1,791912018
gene:ENSG000	18	complement(24135468..24135829)	-	0,07	-3,804623889
CABYR	18	24138987..24161600	0,10	0,19	-0,654216961
OSBPL1A	18	complement(24162045..24397880)	4,11	6,95	-0,534446621
IMPACT	18	24426634..24453531	2,19	6,69	-1,388407252
HRH4	18	24460637..24479961	0,03	0,05	-0,27623233
ZNF521	18	complement(25061924..25352190)	0,29	0,17	0,967979773
gene:ENSG000	18	complement(25200976..25202837)	0,01	-	3,068695355
SS18	18	complement(26016253..26091217)	5,81	12,96	-0,933513485
PSMA8	18	26133852..26193355	-	-	#N/D
TAF4B	18	26226445..26391685	1,06	3,41	-1,463466824
KCTD1	18	complement(26454910..26657401)	1,46	3,60	-1,076460501
AQP4	18	complement(26852043..26865771)	-	0,00	-2,89252005
CHST9	18	complement(26906481..27185308)	0,00	0,02	-2,640348898
CDH2	18	complement(27932879..28177946)	3,88	29,11	-2,684133187
gene:ENSG000	18	30780006..30780424	-	-	#N/D
DSC3	18	complement(30989365..31042815)	-	0,01	-4,758582517
DSC2	18	complement(31058545..31102600)	0,02	-	7,391550995
DSC1	18	complement(31129236..31162856)	-	0,01	-3,804623889
DSG1	18	31318160..31359246	-	-	0,188027316
DSG4	18	31376777..31414912	-	-	0,188027316

DSG3	18	31447741..31478702	-	0,00	-2,89252005
DSG2	18	31497624..31551351	2,65	9,58	-1,628774307
TTR	18	31557009..31598833	-	-	0,188027316
B4GALT6	18	complement(31622246..31685836)	1,60	3,65	-0,961464818
SLC25A52	18	complement(31759584..31760880)	0,02	0,01	0,886720911
TRAPPC8	18	complement(31829197..31953136)	1,52	3,36	-0,92215959
gene:ENSG000	18	31982899..31986087	-	-	#N/D
RNF125	18	32018825..32073219	0,11	0,44	-1,797480649
RNF138	18	32091874..32131561	1,14	3,31	-1,314424782
GAREM1	18	complement(32124877..32470882)	1,05	2,18	-0,833775603
MEP1B	18	32185069..32220404	0,04	0,02	1,505976878
KLHL14	18	complement(32672673..32773023)	0,08	0,31	-1,761117552
CCDC178	18	complement(32937402..33441101)	-	0,00	-2,89252005
ASXL3	18	33578219..33751195	0,01	0,03	-0,841031413
NOL4	18	complement(33851100..34224952)	0,00	0,04	-3,030577401
gene:ENSG000	18	complement(34221007..34221603)	-	-	#N/D
DTNA	18	34493291..34891844	0,15	0,48	-1,465767939
MAPRE2	18	34976928..35143470	4,96	8,89	-0,618913525
gene:ENSG000	18	35082009..35082242	-	-	#N/D
ZNF397	18	35241030..35267133	1,63	3,39	-0,830881124
ZSCAN30	18	complement(35251058..35290245)	2,86	4,74	-0,50385416
gene:ENSG000	18	35257040..35257512	1,16	2,49	-0,873938485
ZNF24	18	complement(35332227..35345482)	4,80	7,37	-0,396157603
ZNF396	18	complement(35366694..35377337)	0,06	0,09	-0,327867903
gene:ENSG000	18	complement(35443871..35497940)	0,07	-	4,516772761
INO80C	18	complement(35452230..35497979)	0,55	1,35	-1,08780254
gene:ENSG000	18	complement(35483431..35483667)	-	0,05	-2,89252005
GALNT1	18	35581117..35711834	4,45	9,67	-0,897607917
gene:ENSG000	18	35972151..35973916	0,15	0,44	-1,35661736
C18orf21	18	35972625..35979286	2,01	5,46	-1,217749304
RPRD1A	18	complement(35984387..36067576)	2,95	4,33	-0,330828337
SLC39A6	18	complement(36108531..36129385)	10,33	23,07	-0,936133211
ELP2	18	36129444..36180557	4,78	7,15	-0,357474062
MOCOS	18	36187497..36272157	2,37	15,35	-2,473937959
FHOD3	18	36297713..36780220	0,06	2,50	-5,167131463
TPGS2	18	complement(36777647..36829216)	4,56	9,12	-0,776924006
KIAA1328	18	36829106..37232172	0,66	0,82	-0,094345212
CELF4	18	complement(37243040..37565827)	0,02	0,02	0,279559702
gene:ENSG000	18	complement(37305295..37306333)	-	-	#N/D
gene:ENSG000	18	complement(37473330..37473977)	0,05	-	4,516772761
gene:ENSG000	18	complement(40062903..40066471)	-	-	#N/D
PIK3C3	18	41955234..42087830	1,05	1,44	-0,234377948
RIT2	18	complement(42743227..43115691)	-	-	#N/D
SYT4	18	complement(43267892..43277535)	-	-	#N/D
gene:ENSG000	18	43499173..43499836	-	-	#N/D
SETBP1	18	44680173..45068510	0,35	0,41	-0,032954962
gene:ENSG000	18	44732575..44733315	0,03	0,02	0,886720911
gene:ENSG000	18	44986109..44989203	-	0,01	-3,804623889

SLC14A2	18	45212995..45683688	0,02	0,01	1,106450337
SLC14A1	18	45687025..45752520	14,18	41,60	-1,329622068
EPG5	18	complement(45800581..45967329)	3,84	6,18	-0,466048514
SIGLEC15	18	45825675..45844094	0,65	0,55	0,461987165
PSTPIP2	18	complement(45983536..46072272)	1,39	3,57	-1,141559343
ATP5F1A	18	complement(46080248..46104334)	37,10	54,69	-0,336868314
HAUS1	18	46104378..46128333	3,82	9,06	-1,022882485
ARK2N	18	46173553..46266992	4,85	6,26	-0,145463303
ARK2C	18	46326809..46463140	0,32	0,13	1,517523335
gene:ENSG000	18	46385707..46387083	0,02	0,05	-1,291882393
LOXHD1	18	complement(46476972..46657220)	-	-	0,188027316
ST8SIA5	18	complement(46667821..46759257)	0,10	0,07	0,768223735
PIAS2	18	complement(46803218..46920160)	1,20	1,96	-0,481579858
gene:ENSG000	18	complement(46915021..46915623)	0,05	0,19	-1,573534033
KATNAL2	18	46917492..47103478	0,59	1,09	-0,666327415
ELOA2	18	complement(47032527..47035621)	0,07	0,08	0,034262941
gene:ENSG000	18	47076117..47076594	0,02	0,11	-1,877914477
HDHD2	18	complement(47107408..47150500)	0,82	2,15	-1,16944633
gene:ENSG000	18	complement(47108188..47176345)	2,54	3,99	-0,432357335
IER3IP1	18	complement(47152834..47176364)	0,84	1,58	-0,682678161
SKOR2	18	complement(47206169..47251660)	-	-	#N/D
gene:ENSG000	18	47501172..47503106	-	-	#N/D
SMAD2	18	complement(47808957..47931146)	1,60	3,03	-0,696982083
gene:ENSG000	18	47866839..47867367	-	0,02	-2,89252005
gene:ENSG000	18	complement(47912207..47912504)	-	-	0,188027316
ZBTB7C	18	complement(48026672..48410752)	0,14	0,10	0,758259226
gene:ENSG000	18	complement(48147634..48150864)	0,04	0,07	-0,741621346
ZBTB7C-AS2	18	48252301..48252837	0,02	0,02	-0,01185201
CTIF	18	48539031..48863217	7,89	13,46	-0,547857497
gene:ENSG000	18	48564795..48568342	0,18	0,34	-0,695640211
gene:ENSG000	18	complement(48661840..48662635)	0,12	0,26	-0,858027988
gene:ENSG000	18	48755693..48756318	0,07	0,33	-1,997339153
SMAD7	18	complement(48919853..48950965)	6,86	8,94	-0,158520666
gene:ENSG000	18	49013106..49013819	0,03	-	3,967268276
DYM	18	complement(49036387..49461347)	3,42	4,95	-0,311323314
C18orf32	18	complement(49477243..49487252)	0,85	0,85	0,213233136
RPL17-C18orf3	18	complement(49481681..49492479)	0,39	2,17	-2,256163251
RPL17	18	complement(49488453..49492508)	144,36	#####	-0,66179421
LIPG	18	49560699..49599185	0,05	0,14	-1,264384767
ACAA2	18	complement(49782164..49813953)	13,55	21,56	-0,446602763
gene:ENSG000	18	complement(49795793..49837623)	-	-	#N/D
MYO5B	18	complement(49822789..50195164)	0,12	1,27	-3,170180067
CFAP53	18	complement(50227193..50266495)	0,18	0,31	-0,510148976
MBD1	18	complement(50266882..50281774)	8,38	10,50	-0,101673282
CXXC1	18	complement(50282343..50287839)	17,21	19,35	0,053537457
SKA1	18	50375040..50394168	3,34	8,19	-1,073335849
MAPK4	18	50560087..50731826	-	0,00	-2,89252005
MRO	18	complement(50795120..50825402)	0,00	0,01	-1,877914477

ME2	18	50879080..50954257	1,27	4,92	-1,729518009
ELAC1	18	50967991..50988121	0,11	0,78	-2,590403017
gene:ENSG000	18	50968019..51058144	-	0,09	-5,902279478
SMAD4	18	51028528..51085045	3,82	6,64	-0,575440727
MEX3C	18	complement(51174550..51218333)	4,99	5,42	0,103260144
DCC	18	52340197..53535899	0,00	0,00	-1,478387936
gene:ENSG000	18	52409042..52409257	-	-	#N/D
MBD2	18	complement(54151606..54224669)	2,93	6,06	-0,824200308
POLI	18	54269517..54321266	0,50	1,10	-0,913333568
STARD6	18	complement(54324358..54357964)	-	-	0,188027316
C18orf54	18	54357906..54385218	0,66	1,48	-0,948475244
DYNAP	18	54587757..54599493	-	-	0,188027316
RAB27B	18	54717860..54895516	0,09	1,85	-4,126176129
CCDC68	18	complement(54901509..54959461)	0,05	0,04	0,426338815
TCF4	18	complement(55222185..55664787)	1,68	2,84	-0,532713137
TXNL1	18	complement(56597209..56651600)	1,06	2,54	-1,032012865
WDR7	18	56651343..57029811	1,49	3,31	-0,928509683
BOD1L2	18	57147087..57150410	-	-	0,188027316
ST8SIA3	18	57352557..57371731	0,00	-	3,068695355
ONECUT2	18	57435374..57491298	0,23	0,29	-0,125054492
FECH	18	complement(57544377..57586732)	1,67	3,60	-0,882844708
NARS1	18	complement(57600656..57622213)	17,25	40,41	-1,00531894
ATP8B1	18	complement(57646426..57803315)	3,48	4,36	-0,102675739
NEDD4L	18	58044226..58401540	1,57	4,35	-1,242664972
ALPK2	18	complement(58481247..58629091)	7,19	7,41	0,179959275
MALT1	18	58671465..58754477	2,71	3,55	-0,167394734
ZNF532	18	58862600..58986480	7,77	8,37	0,115605705
SEC11C	18	59139866..59158832	0,93	1,95	-0,843531182
GRP	18	59220158..59230774	-	-	#N/D
RAX	18	complement(59267035..59274086)	-	-	#N/D
CPLX4	18	complement(59275156..59318649)	-	-	0,188027316
LMAN1	18	complement(59327823..59359265)	42,54	67,68	-0,446942587
CCBE1	18	complement(59430939..59697662)	1,87	4,42	-1,015303291
PMAIP1	18	59899996..59904305	26,57	24,58	0,335169563
MC4R	18	complement(60371062..60372775)	-	0,05	-5,545746556
CDH20	18	61333430..61555779	0,01	0,01	0,921097799
RNF152	18	complement(61808067..61894247)	0,59	0,41	0,772920147
PIGN	18	complement(61905255..62187118)	1,01	1,99	-0,752422834
RELCH	18	62187255..62310249	1,29	2,90	-0,938723815
TNFRSF11A	18	62325287..62391288	0,24	1,14	-2,025967115
ZCCHC2	18	62523025..62587709	0,60	1,65	-1,248765511
gene:ENSG000	18	62551203..62552505	0,04	0,03	0,677647514
PHLPP1	18	62715541..62980433	1,34	4,62	-1,562237354
BCL2	18	complement(63123346..63320128)	0,21	0,97	-2,009639406
KDSR	18	complement(63327726..63367228)	5,06	12,39	-1,068808564
VPS4B	18	complement(63389190..63422483)	2,90	7,28	-1,104934967
SERPINB5	18	63476958..63505085	3,46	8,16	-1,013746551
SERPINB12	18	63542369..63569329	-	-	0,188027316

SERPINB13	18	63586989..63604639	-	-	#N/D
SERPINB4	18	complement(63637259..63644256)	-	-	0,188027316
SERPINB11	18	63647579..63726432	-	-	#N/D
SERPINB3	18	complement(63655197..63661893)	-	-	0,188027316
SERPINB7	18	63752935..63805376	0,02	0,50	-4,078754419
SERPINB2	18	63871692..63903888	-	0,02	-4,758582517
gene:ENSG000	18	63897174..63917490	-	-	0,188027316
SERPINB10	18	63907958..63936111	-	-	#N/D
HMSD	18	63949301..63981774	-	0,01	-3,804623889
SERPINB8	18	63970029..64019779	0,17	0,95	-2,294379016
CDH7	18	65750252..65890337	0,00	-	5,224730805
CDH19	18	complement(66501083..66604138)	-	-	0,188027316
DSEL	18	complement(67506587..67516720)	1,32	1,52	0,022692333
TMX3	18	complement(68673688..68715108)	2,62	5,51	-0,848010787
CCDC102B	18	68715209..69055189	1,08	0,93	0,436214659
DOK6	18	69400888..69849087	0,39	1,32	-1,528006042
gene:ENSG000	18	69659837..69663413	0,03	0,04	0,097868349
CD226	18	complement(69831158..69961803)	0,01	0,07	-2,190618284
RTTN	18	complement(70003031..70205726)	2,99	6,19	-0,826059067
SOCS6	18	70289045..70330199	2,97	5,91	-0,766452446
gene:ENSG000	18	70489630..70491205	-	-	0,188027316
CBLN2	18	complement(72536680..72638521)	-	0,01	-3,804623889
NETO1	18	complement(72742314..72868146)	0,02	0,55	-4,787792412
gene:ENSG000	18	complement(73921939..73922631)	-	-	0,188027316
FBXO15	18	complement(74073353..74147843)	0,05	0,01	2,028855185
TIMM21	18	74148523..74160531	1,83	2,70	-0,336827184
CYB5A	18	complement(74250846..74291973)	0,02	-	5,224730805
C18orf63	18	74315839..74359189	-	-	0,188027316
DIPK1C	18	complement(74434775..74457944)	0,00	0,00	-0,01185201
CNDP2	18	74495816..74523454	3,27	5,54	-0,537828421
CNDP1	18	74534500..74587212	0,03	0,01	2,583287272
ZNF407	18	74597870..75065671	1,09	1,62	-0,348667499
PTGR3	18	complement(75195113..75209139)	3,64	5,72	-0,428319767
TSHZ1	18	75210755..75289950	3,39	5,56	-0,491311901
SMIM21	18	complement(75409476..75427703)	-	-	#N/D
ZNF516	18	complement(76357682..76495242)	2,43	6,97	-1,297810695
gene:ENSG000	18	complement(76385642..76389406)	0,08	0,15	-0,638927618
ZNF236	18	76822557..76972901	2,34	3,67	-0,426197404
MBP	18	complement(76978827..77133683)	0,02	0,05	-1,493828466
gene:ENSG000	18	complement(77002849..77006846)	-	0,00	-2,89252005
gene:ENSG000	18	complement(77108284..77110537)	-	-	0,188027316
GALR1	18	77249848..77277900	-	-	0,188027316
SALL3	18	78979818..79002677	-	-	0,188027316
ATP9B	18	79069285..79378287	0,77	1,51	-0,755250744
gene:ENSG000	18	79085134..79086435	0,10	0,05	1,202745498
gene:ENSG000	18	79144752..79146918	0,01	0,05	-1,955757882
gene:ENSG000	18	79352458..79354567	0,09	0,25	-1,266903375
NFATC1	18	79395856..79529325	1,73	3,08	-0,608702428

gene:ENSG000	18	79420836..79422780	0,14	0,18	-0,093784646
gene:ENSG000	18	79496058..79498348	0,12	0,13	0,136301807
gene:ENSG000	18	complement(79515798..79516057)	0,04	-	3,068695355
CTDP1	18	79679803..79754503	4,29	6,27	-0,325418691
KCNG2	18	79797938..79900184	0,07	0,11	-0,335149681
SLC66A2	18	complement(79902420..79951657)	8,65	10,06	0,005921852
HSBP1L1	18	79964582..79970822	0,26	0,22	0,44246066
TXNL4A	18	complement(79970813..80033949)	4,49	9,10	-0,797614633
gene:ENSG000	18	80034346..80097088	0,16	0,25	-0,438791925
RBFA	18	80034389..80050651	1,65	2,60	-0,431180521
ADNP2	18	80109262..80147523	7,32	10,72	-0,327364663
PARD6G	18	complement(80157232..80247514)	0,79	1,28	-0,47895454
gene:ENSG000	19	68403..69178	5,20	7,19	-0,245375295
gene:ENSG000	19	69167..69972	0,28	0,67	-1,034727217
OR4F17	19	107104..117102	-	0,00	-2,89252005
PLPP2	19	complement(281040..291403)	28,90	28,42	0,2473383
MIER2	19	complement(305573..344815)	15,37	15,32	0,226878262
SPMAP2	19	complement(361747..376026)	0,28	1,06	-1,700405368
C2CD4C	19	complement(405445..409147)	0,06	0,46	-2,710105792
SHC2	19	complement(416589..461033)	0,06	0,13	-0,788818581
CIMAP1D	19	complement(463346..474983)	0,02	0,02	0,524121557
MADCAM1	19	489176..505343	0,51	0,43	0,48223308
TPGS1	19	507497..519654	0,75	0,99	-0,182820249
CDC34	19	531760..542092	21,24	25,72	-0,053170639
GZMM	19	544034..549924	0,07	0,05	0,533811434
BSG	19	571277..583494	83,84	#####	-0,220366979
HCN2	19	589881..617159	0,76	4,79	-2,430987159
POLRMT	19	complement(617221..633537)	14,55	22,12	-0,381075702
FGF22	19	639879..644373	0,08	0,25	-1,404324043
RNF126	19	complement(647526..663233)	10,18	11,93	-0,006492192
FSTL3	19	676392..683392	12,71	11,78	0,332060405
PRSS57	19	complement(685546..695498)	-	0,01	-2,89252005
PALM	19	708935..748329	7,79	9,16	-0,01133891
MISP	19	751112..764318	0,03	0,59	-3,891848962
PTBP1	19	797075..812327	86,92	94,93	0,09588453
PLPPR3	19	complement(812488..821955)	0,03	0,07	-1,022609537
AZU1	19	825097..832018	0,01	0,11	-2,851509423
PRTN3	19	840999..848175	0,27	0,09	1,750568871
ELANE	19	851014..856247	0,20	0,16	0,503684153
CFD	19	859453..867884	0,33	0,87	-1,187729726
MED16	19	complement(867630..893218)	7,99	11,90	-0,351497817
R3HDM4	19	complement(896503..913245)	14,94	20,29	-0,218349507
KISS1R	19	917287..921005	-	0,02	-3,804623889
ARID3A	19	925781..975939	2,55	3,74	-0,328892484
WDR18	19	984332..998438	14,09	13,92	0,240108368
GRIN3B	19	1000419..1009732	0,06	0,15	-0,995745964
TMEM259	19	complement(1009648..1021179)	41,93	35,37	0,468486533
CNN2	19	1026586..1039068	29,45	34,72	-0,014147254

gene:ENSG000	19	complement(1038727..1039064)	-	-	0,188027316
ABCA7	19	1039997..1065572	1,30	1,56	-0,040843878
ARHGAP45	19	1065923..1086628	0,42	0,55	-0,16392398
POLR2E	19	complement(1086574..1095380)	24,21	24,90	0,182174175
GPX4	19	1103982..1106791	27,51	32,12	-0,000595741
SBNO2	19	complement(1107637..1174268)	22,84	26,93	-0,014466847
STK11	19	1177558..1228431	8,80	7,13	0,526590774
CBARP	19	complement(1228287..1238027)	1,39	1,73	-0,093370437
ATP5F1D	19	1241746..1244825	34,06	28,70	0,469960731
MIDN	19	1248553..1259143	21,49	13,91	0,850349544
CIRBP	19	1259384..1274880	9,92	12,88	-0,154094811
FAM174C	19	1275530..1279244	12,25	15,37	-0,103664657
EFNA2	19	1285873..1301431	0,02	0,49	-4,510964225
PWWP3A	19	1354711..1378431	3,61	6,45	-0,612607572
gene:ENSG000	19	complement(1372238..1374364)	3,12	2,67	0,447753878
NDUFS7	19	1383527..1395589	7,35	9,71	-0,179582617
GAMT	19	complement(1397026..1401570)	6,98	10,45	-0,358585597
DAZAP1	19	1407569..1435687	36,76	51,29	-0,257244546
RPS15	19	1438358..1440495	75,51	#####	-0,218423379
APC2	19	1446302..1473244	0,75	0,59	0,580920737
C19orf25	19	complement(1461143..1479219)	3,92	3,55	0,363782566
PCSK4	19	complement(1481428..1490752)	0,97	0,83	0,446792964
REEP6	19	1491166..1497927	2,33	0,67	2,009221303
ADAMTSL5	19	complement(1505022..1513604)	0,49	0,55	0,073879353
PLK5	19	1508023..1536046	0,02	0,01	0,933337975
MEX3D	19	complement(1554672..1568325)	4,60	3,99	0,427235104
MBD3	19	complement(1573596..1592865)	7,69	10,37	-0,208441251
gene:ENSG000	19	1576939..1577086	-	-	#N/D
gene:ENSG000	19	complement(1578339..1605445)	9,03	10,94	-0,054532301
UQCR11	19	complement(1597169..1605473)	11,21	16,13	-0,301825856
TCF3	19	complement(1609291..1652615)	16,78	19,78	-0,014031253
ONECUT3	19	1753506..1780988	0,00	1,39	-7,969369056
ATP8B3	19	complement(1782075..1812276)	1,81	1,90	0,154304452
REXO1	19	complement(1815248..1848483)	6,23	8,16	-0,165282433
KLF16	19	complement(1852399..1863579)	22,02	15,21	0,756719547
ABHD17A	19	complement(1876810..1885496)	8,22	7,04	0,447304106
SCAMP4	19	1905214..1926013	10,10	9,93	0,24760934
ADAT3	19	1905399..1913447	0,29	0,68	-1,014050586
CSNK1G2	19	1941172..1981338	16,33	15,24	0,322959804
BTBD2	19	complement(1985438..2034881)	26,52	32,69	-0,07898514
gene:ENSG000	19	complement(2002580..2003581)	0,18	0,50	-1,230501059
MKNK2	19	complement(2037465..2051244)	43,39	25,55	0,987093551
MOB3A	19	complement(2071036..2096673)	6,69	7,66	0,029207206
IZUMO4	19	2096429..2099593	0,29	0,36	-0,075560925
AP3D1	19	complement(2100988..2164468)	44,15	38,55	0,418948359
DOT1L	19	2163933..2232578	9,84	8,92	0,36402222
PLEKHJ1	19	complement(2230084..2237704)	4,92	5,58	0,04128227
SF3A2	19	2236824..2248655	35,62	34,30	0,277428961

AMH	19	2249309..2252073	0,54	1,21	-0,932623052
JSRP1	19	complement(2252252..2269759)	0,04	0,09	-1,009277154
OAZ1	19	2269509..2273490	181,69	#####	-0,021142964
gene:ENSG000	19	2269525..2341172	0,01	0,01	-0,01185201
PEAK3	19	complement(2274631..2282175)	0,49	0,30	0,933133297
LINGO3	19	complement(2287164..2308154)	0,50	0,14	2,080281941
LSM7	19	complement(2321520..2328611)	12,40	14,96	-0,047431558
SPPL2B	19	2328615..2355095	5,01	4,81	0,280624558
TMPRSS9	19	2360238..2426261	0,24	0,26	0,111266945
TIMM13	19	complement(2425625..2427586)	9,75	14,17	-0,316764811
LMNB2	19	complement(2427638..2456959)	52,39	69,48	-0,184098504
GADD45B	19	2476122..2478259	21,51	17,90	0,488613404
GNG7	19	complement(2511219..2702694)	0,22	0,09	1,463368994
DIRAS1	19	complement(2714567..2721372)	4,84	3,52	0,685169074
gene:ENSG000	19	complement(2717769..2740048)	0,04	0,19	-1,955757882
SLC39A3	19	complement(2732204..2740028)	4,60	3,57	0,589245108
SGTA	19	complement(2754715..2783362)	18,45	17,70	0,283022703
THOP1	19	2785503..2815807	6,47	8,28	-0,132930986
ZNF554	19	2819868..2836735	0,23	0,22	0,31154454
ZNF555	19	2841437..2860471	0,91	0,62	0,768832618
ZNF556	19	2867335..2883445	0,05	0,01	2,303764381
ZNF57	19	2900928..2918473	1,13	0,94	0,48323229
ZNF77	19	complement(2933218..2944971)	1,98	1,90	0,281969059
gene:ENSG000	19	2949035..2950644	1,23	0,85	0,754166448
TLE6	19	2977538..2995179	0,58	0,35	0,933990802
TLE2	19	complement(2997639..3047635)	0,96	1,14	-0,013907101
TLE5	19	complement(3052910..3063107)	57,45	63,15	0,086614023
GNA11	19	3094362..3123999	15,94	13,02	0,515922781
GNA15	19	3136033..3163749	0,01	0,02	-0,579815015
S1PR4	19	3172346..3180332	0,07	0,06	0,468811989
NCLN	19	3185563..3209575	14,34	23,28	-0,476548835
CELF5	19	3224661..3297076	0,01	0,00	0,921097799
NFIC	19	3314403..3469217	25,73	21,55	0,478754675
gene:ENSG000	19	complement(3355554..3355955)	-	-	0,188027316
gene:ENSG000	19	3371395..3371602	0,10	0,12	-0,025382928
SMIM24	19	complement(3473986..3480525)	0,01	-	3,068695355
SMIM44	19	complement(3482110..3483441)	-	-	0,188027316
DOHH	19	complement(3490821..3500674)	3,00	2,92	0,261574841
FZR1	19	3506311..3538334	11,07	10,19	0,342493257
MFSD12	19	complement(3538261..3574290)	4,73	6,76	-0,291961831
TEKTIP1	19	3539171..3544030	0,96	1,05	0,084908503
gene:ENSG000	19	complement(3567278..3567959)	0,09	0,21	-0,895537122
HMG20B	19	3572777..3579088	19,26	20,98	0,099962721
GIPC3	19	3585478..3593541	0,02	0,03	-0,187130731
TBXA2R	19	complement(3594507..3606875)	0,80	1,33	-0,515253271
CACTIN	19	complement(3610645..3626815)	6,64	4,89	0,663813294
PIP5K1C	19	complement(3630183..3700468)	4,22	4,57	0,107115277
TJP3	19	3708362..3750813	0,05	0,06	-0,038823173

APBA3	19	complement(3750772..3761692)	6,16	4,22	0,768995391
MRPL54	19	3762682..3768575	11,48	12,58	0,091554874
RAX2	19	complement(3769089..3772228)	0,04	0,06	-0,297455117
MATK	19	complement(3777970..3802129)	-	-	0,188027316
ZFR2	19	complement(3804024..3869038)	0,00	-	3,967268276
ATCAY	19	3879864..3928082	0,01	0,01	-0,032860829
NMRK2	19	3933069..3942416	0,01	-	3,068695355
DAPK3	19	complement(3958453..3971123)	19,72	12,79	0,848060691
EEF2	19	complement(3976056..3985463)	552,25	#####	0,125368564
PIAS4	19	4007736..4039386	5,72	7,69	-0,203272135
ZBTB7A	19	complement(4043303..4066899)	8,90	9,27	0,163893555
MAP2K2	19	complement(4090321..4124122)	46,01	56,00	-0,06032171
CREB3L3	19	4153631..4173054	-	0,03	-5,071123353
SIRT6	19	complement(4174109..4182566)	5,66	6,37	0,053085041
ANKRD24	19	4182689..4224814	0,05	0,10	-0,915873205
EBI3	19	4229523..4237528	0,18	1,44	-2,793800575
gene:ENSG000	19	complement(4246339..4247358)	0,10	0,04	1,505976878
YJU2	19	4247080..4269088	3,70	5,44	-0,331642612
SHD	19	4279266..4290722	-	-	0,188027316
TMIGD2	19	complement(4292227..4302431)	0,03	-	4,516772761
FSD1	19	4304598..4323843	0,43	0,01	5,970546438
STAP2	19	complement(4324043..4342786)	0,02	0,05	-1,406253396
MPND	19	4343527..4360086	1,86	1,73	0,328624974
SH3GL1	19	complement(4360370..4400547)	29,72	22,03	0,655045518
CHAF1A	19	4402640..4445018	7,28	9,56	-0,170926046
UBXN6	19	complement(4444999..4457794)	20,59	19,27	0,318273731
gene:ENSG000	19	4448810..4450836	1,40	1,48	0,145555082
HDGFL2	19	4472297..4502208	6,74	6,65	0,242532325
PLIN4	19	complement(4502192..4518486)	0,29	0,23	0,50241785
PLIN5	19	complement(4522531..4535224)	0,02	0,00	2,805678585
gene:ENSG000	19	complement(4528427..4540067)	0,01	-	3,068695355
LRG1	19	complement(4536402..4540036)	0,08	-	7,117975732
SEMA6B	19	complement(4542593..4581776)	6,33	12,69	-0,78055638
TNFAIP8L1	19	4639516..4655568	3,90	4,41	0,044204154
MYDGF	19	complement(4641374..4670362)	48,99	33,14	0,78694005
DPP9	19	complement(4674341..4724673)	19,91	20,56	0,17708769
FEM1A	19	4791734..4801273	2,44	4,38	-0,622008767
TICAM1	19	complement(4815932..4831712)	7,22	6,86	0,294959029
PLIN3	19	complement(4838341..4867694)	56,42	38,30	0,781720235
ARRDC5	19	complement(4890437..4902896)	0,01	0,02	-0,025382928
UHRF1	19	4903080..4962154	14,61	19,43	-0,188711533
KDM4B	19	4969113..5153598	4,26	4,79	0,052876876
PTPRS	19	complement(5158495..5340812)	14,41	17,57	-0,063332705
ZNRF4	19	5455417..5456856	-	-	#N/D
TINCR	19	complement(5558167..5578349)	1,12	0,28	2,239001792
SAFB2	19	complement(5586999..5624046)	9,37	5,30	1,04568132
SAFB	19	5623035..5668478	7,55	9,21	-0,064283084
RPL36	19	5674947..5691875	67,77	93,93	-0,24782438

MICOS13	19	complement(5678421..5680516)	17,26	20,00	0,011059525
HSD11B1L	19	5680604..5688523	0,80	0,78	0,257032111
LONP1	19	complement(5691834..5720572)	20,58	32,67	-0,444010073
CATSPERD	19	5720637..5778734	0,01	0,01	1,436225396
PRR22	19	complement(5782960..5784746)	0,20	0,66	-1,499554962
gene:ENSG000	19	complement(5784005..5785619)	-	-	0,188027316
DUS3L	19	complement(5784832..5791225)	1,79	3,89	-0,897214782
NRTN	19	5805067..5828324	0,22	0,15	0,739976582
FUT6	19	complement(5830408..5839722)	0,02	0,13	-2,098998606
FUT3	19	complement(5842888..5851471)	0,04	0,01	2,209934656
FUT5	19	complement(5865826..5870540)	0,06	-	6,461896167
gene:ENSG000	19	complement(5866171..5903787)	0,08	0,07	0,458861392
NDUFA11	19	complement(5891276..5904006)	7,53	8,52	0,045508645
gene:ENSG000	19	5904841..5914707	-	-	0,188027316
VMAC	19	5904872..5910853	2,00	0,76	1,62520289
CAPS	19	5912339..5916211	1,67	0,80	1,296698913
RANBP3	19	complement(5916139..5978142)	7,21	9,83	-0,22391038
RFX2	19	complement(5993164..6199572)	0,91	2,01	-0,920910327
ACSBG2	19	6135247..6193094	0,06	0,06	0,221852695
MLLT1	19	complement(6210381..6279975)	12,36	14,14	0,028175681
gene:ENSG000	19	complement(6263068..6263469)	0,16	0,16	0,221270597
ACER1	19	complement(6306142..6333612)	-	-	0,188027316
CLPP	19	6361531..6370242	9,82	11,06	0,05159589
ALKBH7	19	6372794..6375250	6,35	5,76	0,365175441
PSPN	19	complement(6375148..6379058)	1,36	0,67	1,250078584
GTF2F1	19	complement(6379572..6393981)	23,88	19,15	0,541678163
KHSRP	19	complement(6413102..6424811)	28,59	39,08	-0,227970293
SLC25A41	19	complement(6426037..6433779)	0,17	0,09	1,191340531
SLC25A23	19	complement(6436079..6465203)	2,74	3,48	-0,12052057
CRB3	19	6463777..6467221	-	-	0,188027316
DENND1C	19	complement(6467207..6482557)	0,01	0,01	-0,579815015
TUBB4A	19	complement(6494319..6502848)	0,01	0,81	-6,605155377
TNFSF9	19	6531026..6535924	2,47	4,29	-0,572037692
CD70	19	complement(6583183..6604103)	3,00	9,47	-1,433808503
TNFSF14	19	complement(6661253..6670588)	-	0,01	-4,758582517
C3	19	complement(6677704..6730562)	8,57	43,33	-2,115142773
GPR108	19	complement(6729914..6737580)	11,48	14,19	-0,082989496
TRIP10	19	6737925..6751530	17,96	26,21	-0,322280829
SH2D3A	19	complement(6752160..6767463)	0,07	0,12	-0,494981755
VAV1	19	6772708..6857366	0,30	0,38	-0,106732756
ADGRE1	19	6887566..6940459	2,89	6,20	-0,878712713
gene:ENSG000	19	complement(6894994..6896832)	0,10	0,20	-0,804783029
MBD3L2B	19	complement(7018969..7021431)	-	-	#N/D
MBD3L5	19	7030578..7033011	-	-	#N/D
MBD3L4	19	complement(7037749..7040179)	-	-	#N/D
MBD3L2	19	7049321..7051735	-	-	0,188027316
MBD3L3	19	complement(7056207..7069665)	-	-	0,188027316
ZNF557	19	7069703..7087968	2,79	3,13	0,055278928

INSR	19	complement(7112255..7294414)	7,37	8,13	0,08107159
ARHGEF18	19	7348937..7472485	18,65	13,54	0,685032986
PEX11G	19	complement(7476875..7497449)	0,25	0,38	-0,386551559
SAXO5	19	7492976..7508450	0,11	0,04	1,59409163
gene:ENSG000	19	7507052..7519622	-	0,20	-5,327865481
ZNF358	19	7515292..7521025	20,47	28,86	-0,272246882
MCOLN1	19	7522624..7534009	7,58	6,87	0,365630513
gene:ENSG000	19	7533595..7540059	-	-	#N/D
PNPLA6	19	7534004..7561764	69,09	50,69	0,669858572
CAMSAP3	19	7595863..7618304	0,12	0,05	1,419725363
XAB2	19	complement(7619525..7629545)	80,01	66,17	0,497000924
PET100	19	7629788..7631956	32,12	22,51	0,735920445
gene:ENSG000	19	7629796..7647477	1,95	0,51	2,152687651
PCP2	19	complement(7631611..7633719)	0,62	0,44	0,715831587
STXBP2	19	7636772..7647873	4,06	4,05	0,225671787
RETN	19	7669049..7670455	-	-	#N/D
MCEMP1	19	7677088..7679829	0,05	0,09	-0,609885528
gene:ENSG000	19	7678501..7682854	-	-	#N/D
TRAPPC5	19	7680833..7687703	6,01	6,66	0,073459898
FCER2	19	complement(7688758..7702146)	0,01	-	3,068695355
CLEC4G	19	complement(7728957..7733906)	-	-	#N/D
gene:ENSG000	19	complement(7728958..7745662)	-	0,00	-2,89252005
CD209	19	complement(7739993..7747564)	-	-	0,188027316
CLEC4M	19	7763210..7769605	-	0,01	-2,89252005
EVI5L	19	7830218..7864976	12,15	16,42	-0,211043594
PRR36	19	complement(7868719..7874390)	0,43	0,19	1,383255855
LRRC8E	19	7888505..7902021	5,06	7,89	-0,417388884
MAP2K7	19	7903843..7914478	30,98	30,58	0,24191783
TGFBR3L	19	7914830..7919097	0,11	0,10	0,371589373
SNAPC2	19	7920338..7923250	24,71	21,57	0,419315616
CTXN1	19	complement(7924491..7926135)	5,34	11,13	-0,834877787
TIMM44	19	complement(7926718..7943667)	21,93	28,78	-0,169240862
ELAVL1	19	complement(7958573..8005659)	24,05	31,34	-0,159177602
CCL25	19	8052318..8062660	0,01	-	3,068695355
FBN3	19	complement(8065402..8149592)	-	-	0,188027316
CERS4	19	8206736..8262421	4,40	3,45	0,573039544
CD320	19	complement(8302127..8308358)	27,35	33,53	-0,070762921
gene:ENSG000	19	complement(8308283..8321379)	13,44	14,09	0,155479686
NDUFA7	19	complement(8308768..8321375)	43,05	52,31	-0,058127929
RPS28	19	8321158..8323340	294,24	#####	0,153228101
KANK3	19	complement(8322584..8343262)	0,20	0,24	-0,040279886
ANGPTL4	19	8363289..8374370	102,50	33,09	1,854235128
RAB11B	19	8389981..8404434	72,27	68,09	0,309001789
MARCHF2	19	8413270..8439017	16,45	12,54	0,614380807
gene:ENSG000	19	complement(8444570..8445597)	0,49	0,40	0,512489134
HNRNPM	19	8444767..8489114	11,01	23,41	-0,864589214
PRAM1	19	complement(8490056..8502640)	0,20	0,12	0,99062067
ZNF414	19	complement(8509678..8514167)	6,30	7,83	-0,090336114

MYO1F	19	complement(8520778..8577442)	0,25	0,21	0,463455041
ADAMTS10	19	complement(8580240..8610735)	9,69	8,63	0,390528462
NFILZ	19	8630633..8681151	0,09	0,14	-0,302466337
gene:ENSG000	19	complement(8695721..8696321)	-	-	#N/D
ACTL9	19	complement(8697400..8698795)	-	-	#N/D
OR2Z1	19	8721634..8732160	-	-	#N/D
ZNF558	19	complement(8806170..8832314)	1,59	2,91	-0,647365272
MBD3L1	19	8832377..8843340	-	0,04	-4,359055975
MUC16	19	complement(8848844..9010390)	-	-	0,188027316
OR1M1	19	9087061..9095669	-	-	#N/D
OR7G2	19	complement(9100407..9107475)	-	-	#N/D
OR7G1	19	complement(9114828..9115763)	-	-	#N/D
OR7G3	19	complement(9126012..9126950)	-	-	#N/D
ZNF317	19	9140397..9163424	14,68	18,99	-0,147951597
OR7D2	19	9178979..9188818	0,03	0,00	2,972512804
OR7D4	19	complement(9210276..9219589)	0,01	0,01	0,524121557
OR7E24	19	9247344..9252625	-	-	#N/D
ZNF699	19	complement(9291140..9309838)	2,54	3,37	-0,187398951
ZNF559	19	9323772..9351162	1,11	1,18	0,136274672
ZNF559-ZNF17	19	9324174..9382617	0,40	0,15	1,621107955
ZNF177	19	9363013..9382617	0,77	0,71	0,347062895
ZNF266	19	complement(9412429..9435573)	2,04	3,03	-0,347815575
ZNF560	19	complement(9466355..9498616)	-	0,01	-3,804623889
ZNF426	19	complement(9523223..9538645)	0,03	0,04	0,091002416
ZNF121	19	complement(9560329..9584504)	14,75	19,51	-0,180775017
ZNF561	19	complement(9604680..9621236)	2,59	2,45	0,304423243
ZNF562	19	complement(9641807..9675100)	4,09	4,86	-0,024432175
ZNF846	19	complement(9751993..9793180)	0,06	0,07	-0,039701368
FBXL12	19	complement(9810267..9827816)	12,84	10,18	0,557640352
UBL5	19	9827892..9830115	31,17	32,71	0,153573536
PIN1	19	9835257..9849689	33,11	36,96	0,064611996
OLFM2	19	complement(9853718..9936515)	0,64	1,26	-0,746724475
COL5A3	19	complement(9959561..10010504)	0,12	0,50	-1,803995678
RDH8	19	10013249..10022279	-	0,02	-4,359055975
SHFL	19	10086122..10093252	12,73	9,88	0,588654587
ANGPTL6	19	complement(10092338..10102678)	0,35	0,20	1,031350903
PPAN-P2RY11	19	10106223..10114780	1,00	0,68	0,7835632
PPAN	19	10106362..10112012	21,67	23,37	0,114209358
P2RY11	19	10111693..10115372	6,48	9,69	-0,357900584
EIF3G	19	complement(10115014..10119918)	107,04	#####	0,044304206
DNMT1	19	complement(10133342..10231286)	41,70	53,50	-0,136483458
S1PR2	19	complement(10221433..10231331)	17,03	15,21	0,386316379
MRPL4	19	10251901..10260055	24,43	35,37	-0,310942805
ICAM1	19	10271093..10286615	53,53	86,98	-0,477157868
ICAM4	19	10286955..10288522	0,21	0,13	0,953440461
ICAM5	19	10289952..10296778	2,31	0,22	3,601824612
ZGLP1	19	complement(10304803..10309880)	0,82	0,87	0,134251146
gene:ENSG000	19	complement(10305427..10316009)	0,32	0,44	-0,226320806

FDX2	19	complement(10309916..10316015)	9,32	11,88	-0,127193411
gene:ENSG000	19	complement(10315471..10320678)	2,86	0,76	2,126259879
RAVER1	19	complement(10316212..10333529)	33,60	39,72	-0,018244466
ICAM3	19	complement(10333776..10339661)	8,43	4,61	1,09367016
TYK2	19	complement(10350533..10380608)	33,62	21,21	0,887826421
CDC37	19	complement(10391133..10420121)	76,51	80,06	0,15758084
PDE4A	19	10416773..10469630	12,65	11,88	0,312729151
KEAP1	19	complement(10486125..10503558)	21,30	30,91	-0,314445884
S1PR5	19	complement(10512742..10517931)	0,12	0,18	-0,324169919
ATG4D	19	10543895..10553418	13,59	15,30	0,051853147
KRI1	19	complement(10553085..10566031)	6,89	12,46	-0,632032767
CDKN2D	19	complement(10566460..10569059)	14,40	9,88	0,767134209
AP1M2	19	complement(10572671..10587315)	0,11	0,18	-0,484508196
SLC44A2	19	10602457..10644557	5,41	2,83	1,157466764
ILF3	19	10654304..10692400	30,41	50,51	-0,509019364
QTRT1	19	10701430..10713437	7,13	8,87	-0,090878534
DNM2	19	10718055..10833488	45,37	44,36	0,255375701
TMED1	19	complement(10832067..10836318)	20,36	16,96	0,486420988
C19orf38	19	10836575..10869790	0,38	0,17	1,349428296
CARM1	19	10871553..10923075	26,59	37,67	-0,279406208
YIPF2	19	complement(10922185..10928681)	61,24	32,27	1,147439627
TIMM29	19	10928811..10933535	6,39	5,58	0,420248697
SMARCA4	19	10960932..11079426	15,26	20,30	-0,18848343
LDLR	19	11089418..11133820	64,24	24,92	1,589374812
SPC24	19	complement(11131520..11155807)	28,36	27,57	0,263901802
KANK2	19	complement(11164270..11197791)	13,94	13,49	0,270550093
DOCK6	19	complement(11199295..11262524)	8,81	14,41	-0,485787921
gene:ENSG000	19	11208195..11208702	0,87	0,97	0,069178647
ANGPTL8	19	11237502..11241943	0,23	0,36	-0,415525303
TSPAN16	19	11296139..11326996	0,01	0,02	-0,923955849
RAB3D	19	complement(11322068..11346270)	0,89	0,70	0,572137478
TMEM205	19	complement(11342776..11346518)	38,83	48,82	-0,107438447
CCDC159	19	11344684..11354944	1,91	1,55	0,525800454
PLPPR2	19	11355386..11365698	30,94	19,17	0,913588333
SWSAP1	19	11374666..11376951	2,53	2,00	0,560275762
EPOR	19	complement(11377207..11384342)	7,48	6,75	0,371709801
RGL3	19	complement(11384341..11419328)	1,74	3,76	-0,88768429
ODAD3	19	complement(11420604..11435782)	0,27	0,62	-0,957806429
PRKCSH	19	11435284..11450968	150,00	#####	-0,01614413
ELAVL3	19	complement(11451326..11481046)	0,03	0,03	0,404132137
gene:ENSG000	19	complement(11466240..11505698)	0,10	-	5,697545031
ZNF653	19	complement(11483427..11505839)	2,38	2,64	0,076547372
ECSIT	19	complement(11505929..11529172)	34,34	39,46	0,022254332
CNN1	19	11538767..11550323	0,02	0,17	-3,078441656
ELOF1	19	complement(11551147..11559230)	11,80	10,63	0,373284465
ZNF627	19	11559374..11619161	3,33	4,06	-0,061429353
ACP5	19	complement(11574653..11579993)	0,13	0,12	0,362858235
ZNF823	19	complement(11721265..11739009)	3,59	2,87	0,54664617

ZNF441	19	11767000..11784078	0,64	0,86	-0,208801578
ZNF491	19	11797667..11809622	0,01	0,00	1,833201638
ZNF440	19	11814273..11835216	1,24	1,88	-0,382883968
ZNF439	19	11848726..11883750	0,48	0,52	0,092490369
gene:ENSG000	19	11856032..11858302	0,29	0,24	0,520049574
ZNF69	19	11887782..11914329	0,00	-	3,068695355
ZNF700	19	11925068..11950773	4,39	4,31	0,249091101
gene:ENSG000	19	11925075..11979290	0,07	0,02	2,134539329
ZNF763	19	11965037..11980617	0,06	0,10	-0,619784286
gene:ENSG000	19	11987641..12078106	0,90	0,72	0,535765798
ZNF433	19	complement(12014715..12035741)	0,77	0,92	-0,034163243
gene:ENSG000	19	complement(12017888..12052967)	0,42	-	7,261238359
ZNF878	19	complement(12043805..12052961)	0,38	0,35	0,327978397
ZNF844	19	12064731..12081565	0,26	0,01	4,629129261
gene:ENSG000	19	12075327..12113325	0,68	0,72	0,12633281
ZNF20	19	complement(12092843..12140355)	0,42	0,60	-0,270880586
ZNF625-ZNF20	19	complement(12132117..12156731)	0,85	0,99	0,015388645
ZNF625	19	complement(12142090..12156734)	3,40	2,67	0,572238432
ZNF136	19	12163064..12189871	2,90	2,06	0,715669588
ZNF44	19	complement(12224686..12294883)	1,18	0,98	0,483454947
ZNF563	19	complement(12317477..12333720)	0,47	0,56	-0,017372561
ZNF442	19	complement(12345944..12365905)	0,14	0,17	-0,040487727
ZNF799	19	complement(12390016..12401271)	1,61	1,93	-0,040816457
gene:ENSG000	19	complement(12391949..12441082)	0,26	0,52	-0,770531843
ZNF443	19	complement(12429706..12441021)	1,32	1,28	0,273302679
gene:ENSG000	19	complement(12433103..12484816)	0,31	0,26	0,458861392
ZNF709	19	complement(12461184..12513854)	0,60	0,82	-0,226144615
gene:ENSG000	19	complement(12461559..12551474)	-	-	0,188027316
ZNF564	19	complement(12525373..12551542)	1,51	1,45	0,281013376
gene:ENSG000	19	complement(12525720..12580975)	1,15	1,40	-0,061419394
ZNF490	19	complement(12576100..12640098)	3,10	2,94	0,300615493
ZNF791	19	12610918..12633840	8,07	6,66	0,499886282
gene:ENSG000	19	complement(12643831..12648397)	0,87	0,22	2,228805317
MAN2B1	19	complement(12646511..12666742)	33,42	23,67	0,721066848
gene:ENSG000	19	complement(12664828..12669397)	-	-	#N/D
WDR83	19	12666802..12675832	4,04	3,64	0,376536123
gene:ENSG000	19	complement(12668071..12681834)	4,93	4,89	0,232996661
WDR83OS	19	complement(12668073..12669415)	174,89	#####	0,173072412
DHPS	19	complement(12675717..12681880)	64,55	51,87	0,53854489
GNG14	19	12687998..12688422	1,07	0,61	1,025254957
FBXW9	19	complement(12688053..12696631)	8,56	7,68	0,379327973
TNPO2	19	complement(12699201..12724011)	45,68	52,22	0,030053114
TRIR	19	complement(12730640..12734684)	68,40	88,21	-0,143768319
GET3	19	12737139..12748323	67,22	53,48	0,552810652
BEST2	19	12751702..12758458	0,04	0,01	1,655502569
HOOK2	19	complement(12763003..12872740)	4,19	6,11	-0,322270199
JUNB	19	12791486..12793315	63,52	45,86	0,693088314
PRDX2	19	complement(12796820..12801800)	105,73	#####	0,155663452

THSD8	19	12802031..12813581	0,42	0,25	0,956049759
RNASEH2A	19	12806584..12813640	69,04	68,76	0,228978502
RTBDN	19	complement(12825478..12835428)	0,05	0,08	-0,449191989
MAST1	19	12833951..12874952	0,44	0,99	-0,939793653
DNASE2	19	complement(12875209..12881595)	27,19	41,95	-0,40273813
KLF1	19	complement(12884422..12887201)	0,01	0,02	-0,025382928
GCDH	19	12891128..12915126	3,89	5,24	-0,207129704
SYCE2	19	complement(12898786..12919293)	1,00	0,78	0,580015601
FARSA	19	complement(12922479..12934037)	81,16	78,19	0,276934302
CALR	19	12938578..12944489	#######	#####	0,948959226
RAD23A	19	12945855..12953642	217,58	#####	0,019240324
GADD45GIP1	19	complement(12953119..12957223)	29,26	37,44	-0,132573375
DAND5	19	12965159..12974760	0,35	0,25	0,686982105
NFIX	19	12995475..13098796	123,31	80,45	0,839097797
gene:ENSG000	19	13081966..13082564	2,90	1,64	1,040970984
LYL1	19	complement(13099033..13103161)	0,06	0,04	0,943435191
TRMT1	19	complement(13104902..13117567)	30,42	29,86	0,249885235
NACC1	19	13116862..13141147	74,06	77,40	0,159453316
STX10	19	complement(13144058..13150383)	54,00	48,34	0,382999353
IER2	19	13150411..13154911	57,59	40,51	0,730614963
CACNA1A	19	complement(13206442..13624489)	0,05	0,12	-0,997069195
YJU2B	19	13731760..13763296	23,78	21,34	0,379062664
MRI1	19	13764522..13774282	12,30	11,74	0,29045581
C19orf53	19	13774456..13778773	59,28	59,34	0,221668437
ZSWIM4	19	13795443..13832254	19,07	32,48	-0,544976304
NANOS3	19	13862063..13880757	0,35	0,27	0,589040426
BRME1	19	complement(13882348..13906452)	0,74	0,60	0,517937375
CC2D1A	19	13906201..13930879	12,41	18,42	-0,347375397
PODNL1	19	complement(13931187..13953392)	0,16	0,20	-0,065333608
DCAF15	19	13952509..13961449	16,57	21,68	-0,164838805
RFX1	19	complement(13961530..14007039)	8,07	7,01	0,426378502
RLN3	19	14028148..14031551	0,02	0,04	-0,979341556
IL27RA	19	14031762..14053218	2,87	7,08	-1,077859724
PALM3	19	complement(14053363..14062076)	0,21	0,39	-0,650026009
MISP3	19	14072536..14075062	1,17	0,52	1,404907276
C19orf67	19	complement(14081624..14085875)	0,33	0,19	1,054500315
SAMD1	19	complement(14087851..14091036)	28,98	34,73	-0,038302418
PRKACA	19	complement(14091688..14118084)	15,20	15,74	0,172687932
SMIM46	19	complement(14112324..14112476)	-	-	#N/D
ASF1B	19	complement(14119512..14136613)	31,41	24,30	0,59308095
ADGRL1	19	complement(14147743..14206187)	10,55	14,68	-0,253131847
ADGRE5	19	14380501..14408725	15,88	31,26	-0,753894189
DDX39A	19	complement(14408798..14419383)	60,50	49,64	0,508593697
PKN1	19	14433053..14471867	42,96	91,87	-0,873458048
PTGER1	19	complement(14472466..14475354)	0,07	0,05	0,7939095
GIPC1	19	complement(14477760..14496149)	37,82	47,31	-0,099686084
DNAJB1	19	complement(14514769..14560391)	20,63	20,66	0,221056697
TECR	19	14517085..14565980	111,10	85,92	0,593851506

NDUFB7	19	complement(14566078..14572066)	57,48	69,96	-0,060465002
CLEC17A	19	14583084..14612035	0,00	-	3,068695355
ADGRE3	19	complement(14619117..14690027)	-	-	0,188027316
ZNF333	19	14689801..14733746	1,93	1,49	0,597085615
ADGRE2	19	complement(14732392..14778560)	0,05	0,02	1,365192532
OR7C1	19	complement(14789260..14835376)	0,01	0,00	1,704893826
OR7A5	19	complement(14826241..14835185)	0,00	0,02	-1,478387936
OR7A10	19	complement(14840466..14848922)	-	-	0,188027316
OR7A17	19	complement(14878203..14886132)	0,16	0,15	0,313836442
OR7C2	19	14941489..14942448	0,17	0,28	-0,520458053
SLC1A6	19	complement(14950033..15022990)	0,01	0,00	1,833201638
TEKTL1	19	15010726..15023276	-	-	#N/D
CASP14	19	15049480..15058293	-	0,00	-2,89252005
OR1I1	19	15082211..15092970	0,02	-	6,053060169
SYDE1	19	15107401..15114985	23,99	30,09	-0,103784558
ILVBL	19	complement(15114984..15125786)	45,79	36,32	0,557364397
NOTCH3	19	complement(15159038..15200995)	0,15	0,05	1,920682147
EPHX3	19	complement(15226919..15233435)	0,05	0,17	-1,458497705
BRD4	19	complement(15235519..15332545)	29,98	38,73	-0,14644261
AKAP8	19	complement(15353385..15379815)	11,41	8,88	0,584941514
AKAP8L	19	complement(15380050..15419141)	26,54	26,00	0,252593223
gene:ENSG000	19	complement(15383202..15383832)	0,67	0,82	-0,076435712
gene:ENSG000	19	15398696..15400356	11,24	10,07	0,381931084
WIZ	19	complement(15419978..15449956)	11,49	13,89	-0,051158918
RASAL3	19	complement(15451624..15464544)	-	-	0,188027316
PGLYRP2	19	complement(15468645..15498956)	-	-	0,188027316
CYP4F22	19	15508525..15552317	-	-	0,188027316
CYP4F8	19	15615218..15630639	-	-	0,188027316
CYP4F3	19	15640897..15662825	-	0,00	-2,89252005
CYP4F12	19	15673018..15697174	-	0,01	-3,804623889
OR10H2	19	15728024..15729052	-	-	#N/D
OR10H3	19	15737985..15742343	-	-	#N/D
OR10H5	19	15787661..15800357	-	-	0,188027316
OR10H1	19	complement(15804549..15815664)	-	0,00	-2,89252005
CYP4F2	19	complement(15878023..15898077)	-	-	#N/D
gene:ENSG000	19	15904556..15907234	-	-	#N/D
CYP4F11	19	complement(15912367..15934867)	0,22	0,47	-0,897929491
OR10H4	19	15944299..15950350	-	-	#N/D
gene:ENSG000	19	16019719..16020290	-	-	#N/D
TPM4	19	16067021..16103002	241,48	#####	0,075454886
gene:ENSG000	19	complement(16074292..16077395)	3,19	2,37	0,654168291
RAB8A	19	16111889..16134234	55,68	50,18	0,373136788
HSH2D	19	16134028..16158575	-	0,01	-4,359055975
CIB3	19	complement(16161368..16173525)	0,02	-	3,068695355
FAM32A	19	16185380..16192046	29,06	25,12	0,433489077
AP1M1	19	16197854..16245906	10,21	9,72	0,293866804
KLF2	19	16324826..16328685	1,10	2,05	-0,67150462
EPS15L1	19	complement(16355225..16472085)	6,23	8,50	-0,224603424

gene:ENSG000	19	complement(16356329..16358327)	0,80	0,96	-0,030953579
CALR3	19	complement(16479061..16496167)	0,03	0,02	0,524121557
gene:ENSG000	19	complement(16479067..16628204)	0,03	-	5,480421266
C19orf44	19	16496394..16521352	2,93	3,41	0,00383988
CHERP	19	complement(16517894..16542437)	19,12	24,67	-0,144420733
SLC35E1	19	complement(16549831..16572415)	33,09	16,79	1,201613821
MED26	19	complement(16574907..16629062)	6,37	3,84	0,953509235
gene:ENSG000	19	complement(16577642..16660115)	2,07	0,97	1,312277525
gene:ENSG000	19	16586905..16589409	0,90	0,45	1,205932532
gene:ENSG000	19	complement(16610374..16612203)	1,57	0,49	1,909336915
SMIM7	19	complement(16630751..16660442)	8,24	9,02	0,092095084
TMEM38A	19	16661139..16690023	1,18	0,66	1,05158133
NWD1	19	16719847..16817963	0,01	0,00	1,505976878
SIN3B	19	16829398..16880353	12,85	13,83	0,117066558
F2RL3	19	16888999..16892606	0,33	0,12	1,734493369
CPAMD8	19	complement(16892951..17026815)	0,36	0,15	1,474864132
HAUS8	19	complement(17049729..17075625)	4,69	6,07	-0,149820738
MYO9B	19	17075777..17214537	22,49	21,88	0,262603761
USE1	19	17215346..17219829	3,17	3,92	-0,085154009
OCEL1	19	17226213..17229219	6,73	7,26	0,113080029
NR2F6	19	complement(17231883..17245919)	19,18	20,32	0,139853829
gene:ENSG000	19	complement(17235940..17255448)	-	0,05	-3,804623889
USHBP1	19	complement(17249171..17282786)	0,05	0,03	0,951907113
BABAM1	19	17267376..17281249	34,31	37,42	0,097670553
gene:ENSG000	19	17267418..17282966	0,08	0,18	-0,944785508
ANKLE1	19	17281645..17287646	0,45	0,60	-0,203210727
ABHD8	19	complement(17292131..17310236)	1,71	1,48	0,428482127
MRPL34	19	17292609..17306843	10,98	16,53	-0,366809307
DDA1	19	17309518..17323298	22,60	18,90	0,480822042
ANO8	19	complement(17323223..17334855)	9,26	8,53	0,341637449
GTPBP3	19	17334920..17342731	15,14	17,55	0,009981644
PLVAP	19	complement(17351450..17377342)	0,06	0,05	0,53887001
CCDC194	19	complement(17387196..17394196)	0,18	0,15	0,50082497
BST2	19	complement(17402939..17405630)	106,49	#####	-0,01540999
MVB12A	19	17405722..17433724	26,92	22,19	0,501464377
TMEM221	19	complement(17435509..17448668)	0,35	0,11	1,82631134
gene:ENSG000	19	complement(17436611..17460804)	-	-	#N/D
NXNL1	19	complement(17455425..17460926)	0,02	-	3,967268276
SLC27A1	19	17468769..17506168	8,52	5,64	0,817109605
PGLS	19	17511636..17521288	28,96	35,74	-0,080525184
NIBAN3	19	17523301..17553839	0,02	0,01	1,060045679
COLGALT1	19	17555649..17583162	71,79	#####	-0,346829397
UNC13A	19	complement(17601336..17688365)	0,11	1,97	-3,956094997
gene:ENSG000	19	17622447..17623338	-	0,44	-7,624346382
MAP1S	19	17719242..17734513	17,14	16,60	0,269000477
FCHO1	19	17747718..17788568	1,36	0,82	0,956169083
B3GNT3	19	17794828..17813576	0,05	0,01	2,134539329
INSL3	19	complement(17816512..17821574)	-	-	0,188027316

JAK3	19	complement(17824780..17848071)	0,03	0,05	-0,587156213
RPL18A	19	17859910..17864153	321,83	#####	0,008097903
gene:ENSG000	19	complement(17871273..17871705)	-	-	0,188027316
SLC5A5	19	17871945..17895174	0,01	-	4,516772761
CCDC124	19	17933015..17943991	27,78	36,68	-0,178166683
KCNN1	19	17951293..18000085	0,03	0,06	-0,877094735
ARRDC2	19	18001132..18014102	4,62	7,00	-0,375113187
gene:ENSG000	19	complement(18009440..18009881)	0,27	0,56	-0,81906241
IL12RB1	19	complement(18058995..18098944)	0,01	0,01	-0,03031053
MAST3	19	18097778..18151692	3,28	3,73	0,038119463
gene:ENSG000	19	18153158..18178117	0,19	0,36	-0,697622064
PIK3R2	19	18153163..18170532	22,05	24,07	0,096913531
IFI30	19	18173162..18178117	5,39	9,23	-0,55363978
MPV17L2	19	18193218..18196948	11,56	12,13	0,154203453
RAB3A	19	complement(18196784..18204042)	17,25	4,58	2,136084137
PDE4C_1	19	complement(18207961..18255419)	-	-	0,188027316
PDE4C_2	19	complement(18207965..18248200)	0,33	0,60	-0,656419181
CIST1	19	complement(18249950..18255362)	-	0,02	-2,89252005
IQCN	19	complement(18257098..18274500)	0,16	0,72	-1,914742418
JUND	19	complement(18279694..18281622)	22,19	13,93	0,894050116
LSM4	19	complement(18306236..18323112)	58,21	59,92	0,1811903
PGPEP1	19	18340598..18369950	7,20	7,00	0,263884583
GDF15	19	18374731..18389176	0,27	22,69	-6,18855315
LRRC25	19	complement(18391137..18397622)	-	0,10	-6,89165328
SSBP4	19	18418864..18434562	17,98	20,25	0,05156785
ISYNA1	19	complement(18434388..18438167)	4,48	2,73	0,936100026
ELL	19	complement(18442663..18522116)	14,74	10,52	0,709624621
gene:ENSG000	19	18448275..18448802	0,98	0,39	1,536756392
gene:ENSG000	19	complement(18448275..18448805)	-	-	0,188027316
FKBP8	19	complement(18531751..18544077)	94,02	91,91	0,255729562
KXD1	19	18557762..18569387	25,07	23,55	0,313129219
UBA52	19	18571730..18577550	67,76	67,73	0,223561776
CRLF1	19	complement(18572220..18607741)	25,68	12,15	1,303354806
REX1BD	19	18588685..18592337	15,64	26,62	-0,54397245
TMEM59L	19	18607430..18621039	3,50	1,06	1,940135062
KLHL26	19	18637025..18671721	4,90	5,25	0,122324036
CRTC1	19	18683678..18782333	2,55	3,38	-0,183528284
COMP	19	complement(18782773..18791305)	0,04	-	6,053060169
UPF1	19	18831959..18868230	45,01	39,89	0,397308314
GDF1	19	complement(18868545..18896158)	1,74	2,98	-0,554543156
CERS1	19	complement(18868545..18896727)	5,28	7,66	-0,314539936
COPE	19	complement(18899514..18919387)	186,69	#####	0,524661365
gene:ENSG000	19	complement(18907013..18934397)	0,60	0,19	1,871856751
DDX49	19	18919705..18929189	30,23	32,86	0,102663701
HOMER3	19	complement(18929201..18941261)	22,19	29,84	-0,204259831
SUGP2	19	complement(18990888..19034023)	62,42	60,77	0,261663882
ARMC6	19	19033575..19060311	17,37	21,79	-0,103584138
SLC25A42	19	19063994..19113030	5,16	1,07	2,485578346

TMEM161A	19	complement(19119169..19138513)	20,56	25,93	-0,111591703
MEF2B	19	complement(19145567..19192131)	1,23	0,80	0,848206171
BORCS8-MEF2	19	complement(19145569..19192158)	2,03	2,16	0,132646758
BORCS8	19	complement(19176903..19192591)	4,77	5,59	-0,005547282
RFXANK	19	19192229..19201869	9,30	13,52	-0,316161502
NR2C2AP	19	complement(19201409..19203414)	12,97	15,89	-0,069733282
NCAN	19	19211958..19252233	0,01	0,00	1,487770062
HAPLN4	19	complement(19254756..19262804)	0,01	0,01	-0,032860829
gene:ENSG000	19	19255638..19256141	-	-	#N/D
TM6SF2	19	complement(19264364..19273391)	0,01	-	3,967268276
SUGP1	19	complement(19276018..19320509)	6,44	7,49	0,003591994
MAU2	19	19320829..19358754	13,02	18,43	-0,27837378
GATAD2A	19	19385826..19508932	29,89	45,03	-0,368126764
TSSK6	19	complement(19512418..19515548)	2,62	3,84	-0,328790874
NDUFA13	19	19515736..19529054	58,66	79,60	-0,217272447
gene:ENSG000	19	19516227..19536076	1,74	2,73	-0,422691096
YJEFN3	19	19528861..19537581	4,07	5,42	-0,187927567
CILP2	19	19538248..19546659	0,20	0,24	0,014579522
PBX4	19	complement(19561707..19618693)	2,04	3,04	-0,350706619
LPAR2	19	complement(19623655..19628930)	5,74	4,32	0,630949539
GMIP	19	complement(19629476..19643657)	9,66	8,69	0,376347829
ATP13A1	19	complement(19645198..19663676)	24,31	25,07	0,178505831
ZNF101	19	19668796..19683509	3,25	3,54	0,100293038
ZNF14	19	complement(19710472..19733112)	1,68	2,46	-0,320295516
ZNF506	19	complement(19785839..19821751)	0,33	0,30	0,376508908
ZNF253	19	19865882..19894674	0,30	0,64	-0,838816506
ZNF93	19	19900913..19963464	0,69	0,94	-0,226412472
ZNF682	19	complement(19997058..20039505)	0,50	0,59	-0,017640006
ZNF90	19	20077994..20127076	1,36	2,27	-0,515357883
ZNF486	19	20167214..20200488	4,43	5,73	-0,146128321
gene:ENSG000	19	complement(20207192..20209789)	0,06	0,16	-1,169203102
gene:ENSG000	19	complement(20220597..20222186)	0,12	0,50	-1,874052651
gene:ENSG000	19	20358163..20371960	-	-	0,188027316
ZNF737	19	complement(20535825..20565809)	0,05	0,20	-1,798144948
ZNF626	19	complement(20619939..20661596)	-	0,17	-9,188928145
ZNF66	19	20776304..20809995	0,27	0,28	0,198293473
ZNF85	19	20923222..20950697	0,36	0,73	-0,796970922
gene:ENSG000	19	complement(20926777..20928714)	0,16	0,19	-0,039701368
ZNF430	19	21020634..21060050	0,47	0,47	0,208277754
ZNF714	19	21082159..21125270	1,46	1,38	0,298952143
ZNF431	19	21142024..21196053	0,36	0,46	-0,138282273
ZNF708	19	complement(21291160..21329425)	0,23	0,27	-0,008859828
ZNF738	19	21358930..21388582	0,51	0,50	0,268416624
ZNF493	19	21397119..21427577	0,37	0,35	0,294883912
gene:ENSG000	19	21405159..21491266	-	-	0,188027316
ZNF429	19	21496682..21556270	0,12	0,23	-0,709836428
ZNF100	19	complement(21722771..21767579)	0,35	0,54	-0,391985736
ZNF43	19	complement(21804946..21852125)	0,06	0,13	-0,877307165

ZNF208	19	complement(21932958..22010949)	-	-	0,188027316
gene:ENSG000	19	complement(21965708..21968529)	-	-	#N/D
ZNF257	19	22052430..22091480	0,01	-	4,913749003
ZNF676	19	complement(22179089..22215801)	0,01	0,01	-0,03031053
ZNF729	19	22286441..22317176	-	-	#N/D
ZNF98	19	complement(22391019..22532485)	-	-	0,188027316
ZNF492	19	22634324..22667671	0,01	0,04	-2,752886291
ZNF99	19	complement(22752183..22784151)	-	0,00	-2,89252005
ZNF723	19	22832291..22858667	-	-	0,188027316
ZNF728	19	complement(22974883..23003176)	-	-	#N/D
ZNF730	19	23075210..23147221	0,07	0,13	-0,757909027
ZNF724_1	19	complement(23221599..23250394)	0,98	1,34	-0,232740908
ZNF724_2	19	complement(23222755..23274221)	0,05	0,03	0,627075281
ZNF91	19	complement(23304991..23395471)	0,58	0,93	-0,446259978
ZNF675	19	complement(23525631..23687220)	0,30	0,45	-0,362231519
ZNF681	19	complement(23739195..23758891)	0,00	0,01	-0,03031053
RPSA2	19	23762944..23871162	16,36	15,81	0,272068737
ZNF726	19	23914876..23945159	0,75	0,74	0,245566156
ZNF254	19	24033405..24129968	0,25	0,47	-0,717994233
gene:ENSG000	19	24078481..24080669	0,10	0,09	0,350150203
gene:ENSG000	19	complement(27639300..27639665)	-	-	0,188027316
UQCRFS1	19	complement(29205320..29213151)	15,90	13,08	0,505447198
gene:ENSG000	19	complement(29506966..29509041)	-	-	#N/D
VSTM2B	19	29525994..29564551	-	-	0,188027316
POP4	19	29606283..29617237	3,55	5,73	-0,468906614
PLEKHF1	19	29665459..29675477	3,89	4,21	0,109395412
C19orf12	19	complement(29698937..29715789)	2,76	5,55	-0,783256682
CCNE1	19	29811991..29824312	1,81	3,87	-0,871144869
gene:ENSG000	19	29915862..29917830	0,02	-	4,516772761
URI1	19	29923644..30016612	5,87	10,14	-0,565209776
ZNF536	19	30224891..30713586	-	-	0,188027316
gene:ENSG000	19	30553956..30558058	-	-	0,188027316
TSHZ3	19	complement(31149979..31349436)	2,77	3,70	-0,196778155
gene:ENSG000	19	31364297..31366098	0,66	0,50	0,631211129
ZNF507	19	32345594..32387667	2,74	5,77	-0,851211072
DPY19L3	19	32405543..32485895	1,18	1,50	-0,126377744
PDCD5	19	32581190..32587453	3,84	6,63	-0,56517763
ANKRD27	19	complement(32597006..32676597)	9,99	13,75	-0,238038114
RGS9BP	19	32675848..32678300	0,68	0,65	0,271619478
NUDT19	19	32691821..32713792	5,40	13,53	-1,103067836
TDRD12	19	32719753..32829580	0,01	0,01	1,15046174
SLC7A9	19	complement(32830509..32869767)	0,01	0,02	-0,03031053
CEP89	19	complement(32875925..32971991)	4,03	4,84	-0,043479318
FAAP24	19	32972209..32978229	1,64	3,29	-0,775264669
RHPN2	19	complement(32978592..33064888)	5,31	18,93	-1,610054983
GPATCH1	19	33080899..33130542	3,85	6,68	-0,573933579
WDR88	19	33132090..33175799	0,26	0,44	-0,556655679
LRP3	19	33177603..33208864	11,89	14,28	-0,041813645

SLC7A10	19	complement(33208664..33225850)	0,01	0,03	-1,766505595
CEBPA	19	complement(33299934..33302534)	0,87	3,80	-1,901351192
CEBPG	19	33373685..33382686	8,90	22,05	-1,086242759
PEPD	19	complement(33386950..33521823)	6,11	8,20	-0,201458328
CHST8	19	33621953..33773509	0,00	0,01	-0,923955849
KCTD15	19	33795933..33815763	6,85	10,30	-0,365296257
LSM14A	19	34172504..34229288	14,65	26,64	-0,639406545
GARRE1	19	34254552..34355566	5,03	7,44	-0,341808755
GPI	19	34359480..34402413	113,18	#####	-0,173712756
gene:ENSG000	19	34396315..34409364	0,10	0,16	-0,446980808
PDCD2L	19	34404399..34426168	3,72	10,86	-1,321433015
UBA2	19	34428352..34471251	12,42	28,04	-0,951633063
WTIP	19	34481758..34512304	1,74	2,40	-0,240801683
SCGB2B2	19	complement(34590644..34677159)	0,02	0,13	-2,786768956
gene:ENSG000	19	34645380..34646838	0,03	0,10	-1,462209385
gene:ENSG000	19	complement(34675717..34677581)	0,20	0,77	-1,742926106
ZNF302	19	34677639..34686397	0,30	1,48	-2,097776009
ZNF181	19	34734155..34745378	0,20	0,66	-1,497725927
ZNF599	19	complement(34758073..34773229)	0,60	0,72	-0,052824855
ZNF30	19	34926903..34945170	0,30	0,83	-1,231685829
gene:ENSG000	19	complement(34949038..34951205)	0,05	0,02	1,655502569
ZNF792	19	complement(34956354..34964229)	0,91	0,87	0,301390822
GRAMD1A	19	34994784..35026471	19,80	32,74	-0,502313827
SCN1B	19	35030470..35040449	0,32	1,43	-1,95141949
HPN	19	35040506..35066573	-	-	0,188027316
gene:ENSG000	19	35106510..35124297	0,05	0,01	2,972512804
FXYD3	19	35115823..35124324	0,05	0,12	-0,979460945
LGI4	19	complement(35124513..35142451)	0,10	0,07	0,626665492
gene:ENSG000	19	complement(35137645..35139580)	0,04	0,04	0,181652234
FXYD1	19	35138824..35143109	0,01	0,04	-1,877914477
FXYD7	19	35143250..35154302	-	0,06	-4,758582517
FXYD5	19	35154730..35169881	17,65	24,27	-0,236425903
FAM187B	19	complement(35224800..35228739)	-	-	#N/D
LSR	19	35248330..35267964	0,29	0,57	-0,759171404
USF2	19	35268962..35279821	14,58	22,01	-0,370612211
HAMP	19	35280716..35285143	-	-	0,188027316
MAG	19	35292125..35313807	0,02	0,05	-1,176557791
CD22	19	35319261..35347361	0,63	1,79	-1,290800076
FFAR1	19	35347902..35353864	0,02	0,04	-1,193454645
FFAR3	19	35358460..35360489	-	-	#N/D
GPR42	19	35371068..35372962	-	-	#N/D
FFAR2	19	35443907..35451767	-	-	#N/D
KRTDAP	19	complement(35487324..35495558)	-	-	#N/D
DMKN	19	complement(35497220..35513658)	-	0,02	-5,327865481
SBSN	19	complement(35523367..35528351)	0,01	1,57	-7,744046826
GAPDHS	19	35533455..35545319	0,31	0,71	-0,985414615
TMEM147	19	35545600..35547526	38,05	51,07	-0,201661606
ATP4A	19	complement(35550031..35563658)	0,06	0,05	0,577093021

PMIS2	19	35586161..35587296	-	-	#N/D
HAUS5	19	35612735..35625355	5,01	7,71	-0,398101929
RBM42	19	35629030..35637686	28,15	39,76	-0,275106498
ETV2	19	35641745..35644871	0,23	0,29	-0,101388123
COX6B1	19	35648323..35658782	49,78	67,61	-0,218691033
UPK1A	19	35666517..35678481	-	0,02	-3,804623889
ZBTB32	19	35704558..35717038	0,54	1,19	-0,915448861
KMT2B	19	35717973..35738880	8,49	13,55	-0,451968984
IGFLR1	19	complement(35738801..35742453)	0,59	1,13	-0,726503748
gene:ENSG000	19	complement(35739252..35745432)	0,45	0,27	0,975007463
U2AF1L4	19	complement(35742464..35745445)	2,95	5,39	-0,644309967
PSENEN	19	35745600..35747519	11,85	15,90	-0,201616807
gene:ENSG000	19	35745678..35754519	2,61	2,33	0,38363501
LIN37	19	35748361..35754519	7,79	4,79	0,923660635
HSPB6	19	complement(35754566..35758079)	0,61	0,73	-0,040540615
PROSER3	19	35758143..35771166	5,47	8,88	-0,47539343
ARHGAP33	19	35774532..35788822	3,63	3,86	0,133275821
PRODH2	19	complement(35799988..35813299)	-	-	0,188027316
NPHS1	19	complement(35825372..35869287)	0,00	-	3,068695355
KIRREL2	19	35855861..35867136	-	0,00	-2,89252005
APLP1	19	35867899..35879792	7,02	5,60	0,550790117
NFKBID	19	complement(35887630..35902303)	1,21	2,95	-1,061186213
gene:ENSG000	19	complement(35901561..35903855)	0,01	0,06	-2,272922624
HCST	19	35902529..35904377	0,04	0,16	-1,766505595
TYROBP	19	complement(35904401..35908295)	-	-	#N/D
LRFN3	19	35935358..35946624	2,63	3,67	-0,257028967
SDHAF1	19	35995188..35996312	12,34	11,49	0,327120111
SYNE4	19	complement(36003307..36008813)	0,29	0,49	-0,529470812
ALKBH6	19	complement(36009120..36014239)	2,72	5,00	-0,654606555
CLIP3	19	complement(36014660..36033343)	0,44	0,46	0,165591245
THAP8	19	complement(36034984..36054739)	1,40	1,63	0,005552432
WDR62	19	36054649..36105108	7,14	9,28	-0,155247057
gene:ENSG000	19	36070239..36071070	1,00	0,89	0,392565133
OVOL3	19	36111143..36113711	0,14	0,22	-0,411705719
POLR2I	19	complement(36113709..36115213)	8,30	17,12	-0,821594252
TBCB	19	36114289..36125947	27,06	44,28	-0,487385649
gene:ENSG000	19	36114362..36115146	1,44	3,05	-0,856906871
CAPNS1	19	36139953..36150353	72,28	#####	-0,352042612
COX7A1	19	complement(36150922..36152449)	0,12	0,06	1,232079601
ZNF565	19	complement(36182276..36246257)	0,39	0,46	-0,028546846
ZNF146	19	36214602..36238771	8,61	15,64	-0,638417441
ZFP14	19	complement(36334453..36379201)	0,74	0,49	0,830670616
ZFP82	19	complement(36383120..36418644)	0,16	0,77	-1,996419242
ZNF566	19	complement(36445119..36489902)	0,22	0,64	-1,309996963
ZNF260	19	complement(36510687..36528271)	1,11	2,51	-0,953997441
ZNF529	19	complement(36534774..36605276)	0,70	0,80	0,039347386
ZNF382	19	36604817..36634114	0,22	0,43	-0,712838411
ZNF461	19	complement(36636618..36666853)	0,64	1,06	-0,50701246

ZNF567	19	36687612..36727701	0,58	0,84	-0,299703723
ZNF850	19	complement(36714383..36772825)	0,01	0,02	-0,35396495
ZNF790	19	complement(36817428..36850787)	0,08	0,04	1,059303449
ZNF345	19	36850361..36913029	0,11	0,20	-0,645282042
gene:ENSG000	19	36850858..36998682	-	0,01	-3,804623889
ZNF829	19	complement(36888124..36916291)	0,01	0,00	2,144183439
ZNF568	19	36916316..37005037	0,00	0,00	1,436225396
ZNF420	19	37007857..37130368	0,54	1,05	-0,73861854
gene:ENSG000	19	complement(37093019..37210512)	0,01	0,02	-1,478387936
ZNF585A	19	complement(37106734..37172741)	0,20	0,03	3,173484847
ZNF585B	19	complement(37181579..37218153)	0,02	0,01	2,134539329
ZNF383	19	37217926..37248740	0,50	0,72	-0,303732944
ZNF875	19	37312837..37369365	3,97	5,33	-0,200697464
ZNF527	19	37371061..37393066	0,52	0,78	-0,358720411
gene:ENSG000	19	complement(37401404..37403846)	0,04	0,02	1,505976878
ZNF569	19	complement(37411155..37469275)	0,34	0,54	-0,44844948
ZNF570	19	37467585..37488652	0,09	0,10	0,022375842
ZNF793	19	37506939..37548762	0,01	0,01	1,356372188
ZNF540	19	37551406..37614179	0,04	0,06	-0,377031134
ZNF571	19	complement(37554782..37594792)	0,52	0,61	-0,013262985
ZFP30	19	complement(37613749..37692337)	0,61	1,39	-0,956615543
gene:ENSG000	19	complement(37689865..37719188)	-	-	0,188027316
ZNF607	19	complement(37696371..37719761)	0,31	0,51	-0,494815866
ZNF573	19	complement(37735833..37817300)	0,30	0,37	-0,072176244
WDR87	19	complement(37884823..37906677)	-	-	0,188027316
SIPA1L3	19	37907208..38208369	13,01	19,58	-0,367321814
DPF1	19	complement(38211006..38229714)	1,32	1,37	0,176795241
SPINT2	19	38244035..38292615	0,19	0,59	-1,405513775
PPP1R14A	19	complement(38251237..38256532)	0,01	0,04	-1,877914477
gene:ENSG000	19	38289151..38304910	-	-	0,188027316
YIF1B	19	complement(38303558..38317273)	21,72	17,98	0,495939668
C19orf33	19	38304164..38305006	0,12	52,71	-8,46652619
KCNK6	19	38319845..38332076	8,73	5,91	0,786365844
CATSPERG	19	38335775..38370943	0,05	0,03	1,151360095
PSMD8	19	38374563..38383850	37,93	55,40	-0,32365742
GGN	19	complement(38384267..38388082)	0,69	0,61	0,40800992
SPRED3	19	38388421..38399587	1,68	2,33	-0,246817826
FAM98C	19	38403093..38409088	4,49	4,58	0,196154205
RASGRP4	19	complement(38409051..38426305)	0,10	0,06	1,09950261
RYR1	19	38433691..38595273	0,01	0,01	0,067331041
MAP4K1	19	complement(38587641..38618882)	0,19	0,64	-1,538799644
EIF3K	19	38619082..38636955	56,95	75,34	-0,180704591
ACTN4	19	38647649..38731589	142,97	#####	0,032964912
CAPN12	19	complement(38730187..38769904)	0,01	0,02	-1,291882393
LGALS7	19	complement(38770968..38773517)	-	-	0,188027316
LGALS7B	19	38789200..38791754	-	-	#N/D
LGALS4	19	complement(38801671..38812945)	0,17	0,41	-1,006640124
ECH1	19	complement(38815422..38831841)	49,65	86,06	-0,570435427

gene:ENSG000	19	complement(38817471..38840178)	-	0,03	-3,804623889
HNRNPL	19	complement(38836370..38852347)	132,14	#####	-0,200550323
RINL	19	complement(38867830..38878275)	0,64	0,09	3,06196894
SIRT2	19	complement(38878555..38899862)	8,95	12,19	-0,222116808
NFKBIB	19	38899700..38908893	6,39	7,90	-0,083183714
CCER2	19	complement(38908980..38912186)	0,14	0,19	-0,229465536
SARS2	19	complement(38915266..38930763)	10,42	19,29	-0,664803487
gene:ENSG000	19	complement(38915404..38949855)	-	-	0,188027316
MRPS12	19	38930944..38933168	18,65	25,87	-0,249104426
FBXO17	19	complement(38941401..38975742)	5,44	11,18	-0,81643881
FBXO27	19	complement(38990714..39032785)	0,45	1,45	-1,452880924
ACP7	19	39083913..39111493	0,01	0,00	1,833201638
PAK4	19	39125770..39182816	13,46	20,82	-0,405446282
NCCRP1	19	39196964..39201884	-	-	0,188027316
SYCN	19	complement(39202825..39204263)	-	-	#N/D
IFNL3	19	complement(39243455..39245250)	-	-	0,188027316
IFNL4	19	complement(39246314..39248856)	-	0,01	-2,89252005
IFNL2	19	39268396..39270188	-	-	#N/D
IFNL1	19	39296407..39298673	-	-	0,188027316
LRFN1	19	complement(39306566..39320863)	0,91	2,32	-1,121443338
GMFG	19	complement(39328353..39342372)	0,11	0,07	0,946003371
SAMD4B	19	39342421..39385710	28,46	28,25	0,234016444
PAF1	19	complement(39385629..39391154)	12,72	16,59	-0,159402802
MED29	19	39391303..39400641	8,53	10,76	-0,111801247
ZFP36	19	39406847..39409407	10,07	6,74	0,801287982
PLEKHG2	19	39412669..39428415	4,65	7,21	-0,409315511
RPS16	19	complement(39433137..39435949)	353,84	#####	-0,459082536
SUPT5H	19	39436156..39476670	36,54	39,29	0,1183232
TIMM50	19	39480412..39493785	12,12	22,75	-0,685226087
DLL3	19	39498895..39508481	0,22	1,23	-2,283246444
SELENOV	19	39515139..39520675	-	0,04	-5,071123353
EID2B	19	complement(39530987..39532852)	2,33	2,83	-0,058583892
EID2	19	complement(39538707..39540161)	1,39	4,40	-1,433847635
LGALS13	19	39602524..39607474	-	-	#N/D
LGALS16	19	39655913..39660647	-	-	#N/D
LGALS14	19	39704481..39709444	-	0,01	-2,89252005
CLC	19	complement(39731255..39738029)	-	-	#N/D
LEUTX	19	39776595..39786291	-	-	#N/D
DYRK1B	19	complement(39825350..39834201)	0,63	1,30	-0,827890256
gene:ENSG000	19	complement(39830482..39831956)	-	-	#N/D
FBL	19	complement(39834458..39846379)	86,40	#####	-0,216256761
FCGBP	19	complement(39863323..39934626)	0,05	0,20	-1,760552657
PSMC4	19	39971165..39981764	55,56	77,29	-0,253200109
ZNF546	19	39984134..40021038	0,10	0,51	-2,096083389
ZNF780B	19	complement(40028260..40056231)	0,10	0,23	-0,93760874
ZNF780A	19	complement(40069152..40090943)	0,34	0,87	-1,15179282
MAP3K10	19	40191426..40215575	5,18	10,13	-0,74367458
TTC9B	19	complement(40216058..40218384)	0,05	0,08	-0,345401666

CCNP	19	complement(40222208..40226697)	0,15	0,05	1,750568871
AKT2	19	complement(40230317..40285536)	16,84	20,87	-0,086440147
C19orf47	19	complement(40319536..40348527)	5,62	7,00	-0,094257036
PLD3	19	40348456..40389472	8,70	12,54	-0,303678931
gene:ENSG000	19	40355842..40360606	1,25	1,23	0,238462436
HIPK4	19	complement(40379271..40390181)	0,05	0,09	-0,659786428
PRX	19	complement(40393766..40414793)	1,93	2,17	0,056772607
SERTAD1	19	complement(40421589..40425992)	2,12	3,39	-0,45131737
SERTAD3	19	complement(40440844..40444335)	16,45	11,76	0,70717517
BLVRB	19	complement(40447765..40465764)	22,25	23,45	0,147312021
SPTBN4	19	40466241..40576464	0,08	0,12	-0,406982159
SHKBP1	19	40576853..40591399	33,86	58,85	-0,574594396
LTBP4	19	40592883..40629818	27,90	27,88	0,224027036
NUMBL	19	complement(40665905..40690972)	5,49	8,79	-0,455693755
COQ8B	19	complement(40691514..40725784)	2,14	4,55	-0,868509179
ITPKC	19	40717112..40740860	4,65	7,59	-0,481866714
ACTMAP	19	complement(40740856..40751553)	11,19	13,53	-0,051255828
SNRPA	19	40750637..40765389	57,52	59,60	0,171731729
MIA	19	40771648..40777490	-	-	0,188027316
MIA-RAB4B	19	40771648..40796943	-	0,05	-5,545746556
RAB4B	19	40778216..40796942	9,45	9,82	0,168439588
RAB4B-EGLN2	19	40778242..40808418	0,50	0,28	1,074969351
EGLN2	19	40798996..40808434	8,69	15,10	-0,574823968
gene:ENSG000	19	40801297..40898282	0,09	0,18	-0,698295353
gene:ENSG000	19	40840159..40842039	0,30	0,23	0,598167742
CYP2A6	19	complement(40843541..40850447)	0,01	0,03	-1,877914477
CYP2A7	19	complement(40875439..40882752)	0,01	0,01	0,524121557
CYP2B6	19	40991282..41018398	0,00	0,06	-3,750082345
CYP2A13	19	41088451..41096195	-	-	#N/D
CYP2F1	19	41114432..41128381	0,02	0,04	-0,999120035
CYP2S1	19	41193210..41207539	2,56	15,61	-2,382951397
AXL	19	41219223..41261766	80,24	#####	-0,77516266
HNRNPUL1	19	41262496..41307787	64,46	88,65	-0,236507712
TGFB1	19	complement(41301587..41353922)	63,76	54,20	0,457289678
CCDC97	19	41310172..41324873	4,58	7,53	-0,493956775
gene:ENSG000	19	41350853..41425001	-	-	0,188027316
TMEM91	19	41350911..41384083	2,14	2,76	-0,145192846
B9D2	19	complement(41354417..41364165)	2,27	3,69	-0,474125931
EXOSC5	19	complement(41386371..41397362)	15,18	19,72	-0,15440597
BCKDHA	19	41397808..41425002	21,20	16,95	0,545984864
B3GNT8	19	complement(41425359..41428730)	0,45	0,48	0,133720772
DMAC2	19	complement(41431318..41440717)	14,47	20,21	-0,25924199
ERICH4	19	41443156..41444765	0,02	-	3,967268276
CEACAM21	19	41549518..41586844	-	-	#N/D
gene:ENSG000	19	complement(41551568..41552274)	-	-	#N/D
CEACAM4	19	complement(41619015..41627074)	-	-	#N/D
CEACAM7	19	complement(41673303..41706976)	-	-	#N/D
CEACAM5	19	41708585..41730433	0,00	0,00	-0,01185201

gene:ENSG000	19	41708657..41756737	-	-	#N/D
CEACAM6	19	41750977..41772211	-	-	0,188027316
CEACAM3	19	41796587..41811554	-	-	#N/D
LYPD4	19	complement(41837074..41844697)	-	-	#N/D
DMRTC2	19	41844743..41852333	-	0,00	-2,89252005
RPS19	19	41860255..41872925	133,22	#####	-0,263711748
CD79A	19	41877279..41881372	0,06	0,11	-0,678413358
ARHGEF1	19	41883173..41930150	28,13	38,50	-0,229718639
ERFL	19	complement(41907704..41928449)	2,08	0,55	2,131102031
RABAC1	19	complement(41956681..41959321)	72,13	61,77	0,446758643
gene:ENSG000	19	complement(41956879..41994232)	-	0,03	-5,545746556
ATP1A3	19	complement(41966582..41997497)	0,04	0,20	-2,052125812
GRIK5	19	complement(41998321..42070206)	0,07	0,16	-1,058093626
ZNF574	19	42068477..42081552	6,15	8,44	-0,234319128
POU2F2	19	complement(42086110..42196585)	0,10	0,55	-2,245648838
DEDD2	19	complement(42198598..42220140)	22,85	15,53	0,780094698
gene:ENSG000	19	complement(42215133..42232149)	0,35	-	5,697545031
ZNF526	19	42220292..42228201	22,47	11,52	1,186760259
GSK3A	19	complement(42226225..42242625)	12,86	20,05	-0,417795432
gene:ENSG000	19	complement(42234583..42255132)	-	0,27	-6,426264264
gene:ENSG000	19	42242775..42244973	0,98	1,29	-0,178789693
ERF	19	complement(42247569..42255128)	19,10	31,83	-0,514105428
CIC	19	42268537..42295797	19,80	17,79	0,378140571
PAFAH1B3	19	complement(42297033..42303546)	19,84	24,89	-0,103989681
PRR19	19	42302098..42310814	0,08	0,18	-0,956362269
TMEM145	19	42313309..42325064	0,01	0,06	-2,394315395
MEGF8	19	42325609..42378769	7,98	14,01	-0,588929016
CNFN	19	complement(42387019..42390297)	0,81	0,70	0,437355004
LIPE	19	complement(42401514..42427388)	0,28	0,76	-1,227385891
CXCL17	19	complement(42428278..42442946)	0,19	0,13	0,742017343
CEACAM1	19	complement(42507304..42561234)	0,14	0,30	-0,858020521
CEACAM8	19	complement(42580243..42595055)	0,02	0,03	-0,602143793
PSG3	19	complement(42721638..42740481)	-	0,01	-3,804623889
PSG8	19	complement(42752686..42765678)	-	0,01	-3,804623889
PSG1	19	complement(42866464..42879822)	0,17	0,67	-1,762598096
PSG6	19	complement(42902086..42919563)	0,01	0,11	-4,024922383
PSG7	19	complement(42924132..42937207)	-	0,10	-7,179219424
PSG11	19	complement(43007656..43026474)	0,02	0,03	-0,345401666
PSG2	19	complement(43064209..43083045)	-	0,02	-4,359055975
PSG5	19	complement(43166256..43186536)	0,06	0,04	1,021047459
PSG4	19	complement(43192702..43207299)	0,02	0,03	-0,225481127
PSG9	19	complement(43211791..43269530)	0,32	0,61	-0,721472397
CD177	19	43353686..43363172	0,55	1,78	-1,460374281
TEX101	19	43401496..43418597	-	-	0,188027316
LYPD3	19	complement(43460787..43465608)	2,02	10,41	-2,144518702
PHLDB3	19	complement(43474954..43504935)	3,84	3,43	0,3891517
ETHE1	19	complement(43506719..43527230)	3,97	7,87	-0,762636838
ZNF575	19	43525497..43536130	0,38	0,77	-0,807817794

XRCC1	19	complement(43543311..43580473)	23,36	30,64	-0,168725684
gene:ENSG000	19	complement(43554692..43593036)	0,22	0,10	1,339142659
PINLYP	19	43575801..43583964	0,27	0,28	0,185894001
IRGQ	19	complement(43584367..43596134)	10,18	9,20	0,369464955
ZNF576	19	43596392..43601157	3,75	3,74	0,226395314
SRRM5	19	43596617..43614498	0,41	0,58	-0,286697885
ZNF428	19	complement(43607224..43619629)	3,75	7,52	-0,778253626
CADM4	19	complement(43622368..43639850)	0,24	1,58	-2,51262612
PLAUR	19	complement(43646095..43670547)	17,96	30,53	-0,54193303
IRGC	19	43716076..43720021	0,01	0,01	-0,01185201
SMG9	19	complement(43727983..43754962)	7,44	10,33	-0,249397544
KCNN4	19	complement(43766533..43780976)	4,41	7,81	-0,599867975
LYPD5	19	complement(43785874..43827206)	0,01	0,01	-0,025382928
ZNF283	19	43827321..43852017	0,39	0,37	0,310710371
ZNF404	19	complement(43872365..43901385)	0,11	0,12	0,120130055
gene:ENSG000	19	43891233..43895411	0,22	0,07	2,003165495
ZNF45	19	complement(43912624..43935282)	1,31	2,04	-0,408825148
ZNF221	19	43951223..43967709	0,55	0,70	-0,111460783
ZNF155	19	43967862..43998326	0,03	0,09	-1,177556582
ZNF230	19	44002957..44013924	0,90	0,62	0,750394242
ZNF222	19	44025342..44033110	0,87	0,66	0,621559246
gene:ENSG000	19	44025354..44087318	0,13	-	8,386648631
ZNF223	19	44051367..44067999	0,21	0,22	0,181527665
ZNF284	19	44072159..44089613	0,94	1,03	0,094381918
ZNF224	19	44094361..44109886	1,29	1,63	-0,11493852
ZNF225	19	44112181..44134822	0,44	0,72	-0,506364168
ZNF234	19	44141554..44160313	1,10	1,45	-0,178550656
ZNF226	19	44165073..44178381	1,56	1,29	0,490967329
ZNF227	19	44207547..44237268	1,46	2,07	-0,282498631
ZNF235	19	complement(44228729..44305046)	0,26	0,42	-0,4779301
gene:ENSG000	19	complement(44246478..44248374)	0,06	0,03	0,943435191
ZNF233	19	44259880..44275317	0,12	0,12	0,272926936
ZNF112	19	complement(44326555..44367217)	0,52	0,87	-0,515888389
gene:ENSG000	19	complement(44329695..44401608)	-	0,09	-4,758582517
ZNF285	19	complement(44382298..44401608)	0,08	0,13	-0,498527562
ZNF229	19	complement(44417519..44448578)	-	-	0,188027316
ZNF180	19	complement(44474428..44500524)	0,60	0,95	-0,438533835
CEACAM20	19	complement(44501677..44529788)	-	-	0,188027316
IGSF23	19	44613563..44636781	0,05	0,24	-2,126488637
PVR	19	44643798..44666162	9,19	18,35	-0,774475115
CEACAM19	19	44662278..44684359	0,77	2,44	-1,447999786
gene:ENSG000	19	44664131..44666158	-	-	#N/D
CEACAM16	19	44699151..44710718	0,03	0,07	-0,865575989
BCL3	19	44747705..44760044	10,68	19,66	-0,658025043
CBLC	19	44777869..44800652	-	0,01	-2,89252005
BCAM	19	44809071..44821421	1,51	3,83	-1,115278788
NECTIN2	19	44846175..44889223	14,22	17,82	-0,103036132
TOMM40	19	44890569..44903689	28,15	34,80	-0,082515336

APOE	19	44905791..44909393	0,56	0,63	0,054950745
gene:ENSG000	19	44909375..44914968	0,04	0,06	-0,321745402
APOC1	19	44914247..44919349	0,01	0,04	-1,877914477
APOC4	19	44942237..44945496	-	-	0,188027316
APOC4-APOC2	19	44942238..44949565	0,06	0,05	0,604285378
APOC2	19	44946035..44949565	0,07	0,10	-0,35396495
CLPTM1	19	44954585..44993341	22,46	27,22	-0,054015168
RELB	19	45001461..45038192	4,50	11,06	-1,074577534
CLASRP	19	45039045..45070956	12,36	13,53	0,092019314
ZNF296	19	complement(45071500..45076478)	1,43	1,81	-0,112025605
GEMIN7	19	45079195..45091518	5,33	7,61	-0,291326311
MARK4	19	45079288..45305284	9,80	8,91	0,361314263
PPP1R37	19	45091396..45148077	7,35	6,61	0,37629265
NKPD1	19	complement(45149744..45160982)	0,29	0,28	0,256939155
TRAPPC6A	19	complement(45162928..45178237)	5,65	3,51	0,911682014
BLOC1S3	19	45178784..45216933	8,65	5,27	0,938637381
EXOC3L2	19	complement(45212370..45245407)	-	0,02	-4,758582517
CKM	19	complement(45306413..45322875)	0,11	0,06	1,138491112
KLC3	19	45333434..45351520	0,16	0,13	0,542675196
ERCC2	19	complement(45349837..45370918)	16,08	15,89	0,240658548
PPP1R13L	19	complement(45379638..45406349)	5,55	6,78	-0,065909012
POLR1G	19	45406644..45410737	6,50	5,91	0,359729996
ERCC1	19	complement(45407334..45478828)	16,17	13,49	0,484133375
FOSB	19	45467995..45475179	2,27	1,27	1,066439054
RTN2	19	complement(45485294..45497055)	5,89	2,38	1,529600804
PPM1N	19	45488777..45502513	0,51	0,55	0,120356282
VASP	19	45506579..45526989	67,76	53,55	0,562511851
OPA3	19	complement(45527767..45602212)	4,66	4,43	0,294740705
GPR4	19	complement(45589764..45602212)	0,05	0,03	0,842164604
EML2	19	complement(45606994..45645602)	9,50	11,21	-0,01509278
GIPR	19	45668221..45683722	0,41	0,22	1,125378317
SNRPD2	19	complement(45687460..45692569)	71,41	78,37	0,088929974
QPCTL	19	45692403..45703989	13,44	11,02	0,509418278
FBXO46	19	complement(45710629..45730896)	6,78	8,08	-0,03041896
gene:ENSG000	19	complement(45714387..45717381)	1,99	2,94	-0,334284279
MEIOSIN	19	45733439..45764541	0,03	0,06	-1,019619711
SIX5	19	complement(45764785..45769252)	6,26	8,31	-0,186668812
DMPK	19	complement(45769709..45782552)	11,64	13,16	0,046201408
gene:ENSG000	19	complement(45779437..45785973)	0,75	0,50	0,819537358
DMWD	19	complement(45782947..45792845)	13,13	14,37	0,092082971
RSPH6A	19	complement(45795713..45815308)	0,17	0,14	0,507941025
SYMPK	19	complement(45815410..45863225)	15,43	17,89	0,009555576
FOXA3	19	45863989..45873797	0,01	0,04	-2,447197442
IRF2BP1	19	complement(45883608..45886141)	11,07	15,27	-0,24145701
MYPOP	19	complement(45890023..45902613)	5,09	6,62	-0,155438281
NANOS2	19	complement(45913214..45914778)	0,02	-	4,516772761
NOVA2	19	complement(45933734..45974044)	0,03	0,09	-1,298099036
CCDC61	19	45995461..46021318	3,61	3,21	0,39235088

PGLYRP1	19	complement(46019153..46023053)	0,24	0,11	1,356372188
IGFL4	19	complement(46039182..46077118)	-	0,03	-4,359055975
IGFL3	19	complement(46120067..46124688)	-	-	0,188027316
IGFL2	19	46143106..46161298	-	0,08	-5,327865481
IGFL1	19	46229742..46231243	-	-	#N/D
HIF3A	19	46297042..46343433	0,00	-	3,967268276
PPP5C	19	46347087..46392981	21,24	25,94	-0,065528272
CCDC8	19	complement(46410329..46413564)	2,81	2,87	0,190963616
PNMA8C	19	complement(46424697..46428936)	-	-	0,188027316
PNMA8A	19	complement(46466491..46471563)	0,03	0,00	3,122038495
PNMA8B	19	complement(46486906..46495879)	0,00	0,00	-0,01185201
CALM3	19	46601074..46610782	128,39	#####	0,578906937
PTGIR	19	complement(46620468..46625089)	0,01	0,08	-2,851509423
GNG8	19	complement(46633953..46636541)	0,09	0,08	0,366665713
gene:ENSG000	19	complement(46636810..46639161)	0,11	0,05	1,420755175
DACT3	19	complement(46647551..46661182)	0,62	0,09	2,932289258
PRKD2	19	complement(46674275..46717127)	17,02	13,60	0,547162456
STRN4	19	complement(46719511..46746994)	19,86	28,64	-0,305081441
FKRP	19	46746046..46776988	1,78	4,44	-1,094409435
SLC1A5	19	complement(46774883..46788594)	71,09	87,18	-0,071318464
AP2S1	19	complement(46838136..46850846)	113,66	#####	0,326873626
ARHGAP35	19	46860997..47005077	43,12	30,50	0,722716561
NPAS1	19	47019837..47045775	0,42	2,35	-2,25677816
TMEM160	19	complement(47045909..47048624)	5,88	4,96	0,467218191
ZC3H4	19	complement(47064187..47113776)	3,33	9,87	-1,341930482
SAE1	19	47113274..47210636	38,89	43,97	0,046127287
BBC3	19	complement(47220822..47232766)	4,83	4,61	0,289116444
CCDC9	19	47255980..47273701	15,32	4,77	1,906014202
INAFM1	19	47274885..47275723	20,61	6,08	1,984174163
C5AR1	19	47290023..47322066	0,02	0,02	0,278120973
C5AR2	19	47332175..47347329	0,27	0,01	5,305223746
DHX34	19	47349315..47382704	7,93	11,31	-0,289375653
MEIS3	19	complement(47403124..47419527)	0,30	0,45	-0,352489165
SLC8A2	19	complement(47428017..47471893)	0,01	0,02	-0,602143793
KPTN	19	complement(47475150..47484265)	3,84	4,09	0,13346091
NAPA	19	complement(47487637..47515091)	43,48	31,04	0,709142728
gene:ENSG000	19	complement(47487638..47489519)	5,01	2,13	1,45877703
ZNF541	19	complement(47520690..47573148)	0,00	0,02	-1,54862452
BICRA	19	47608196..47703277	2,29	2,50	0,095967655
gene:ENSG000	19	complement(47661657..47663895)	0,15	0,15	0,212747274
EHD2	19	47713422..47743134	62,66	51,66	0,501451327
NOP53	19	47745546..47757058	47,12	49,20	0,160916179
SELENOW	19	47778677..47784686	30,87	39,98	-0,15007575
TPRX1	19	complement(47801232..47819051)	-	-	#N/D
CRX	19	47819779..47843330	0,03	0,01	2,469244963
TPRX2	19	47859235..47861721	-	-	0,188027316
SULT2A1	19	complement(47870467..47886315)	0,01	-	3,068695355
BSPH1	19	complement(47968046..47992170)	0,03	-	3,967268276

ELSPBP1	19	47994632..48025154	0,02	-	3,967268276
CABP5	19	complement(48029383..48044079)	0,02	-	4,913749004
PLA2G4C	19	complement(48047843..48110817)	2,81	0,74	2,14839346
LIG1	19	complement(48115445..48170603)	13,69	10,53	0,602043807
ZSWIM9	19	48170680..48197620	2,53	2,38	0,307328026
CARD8	19	complement(48180770..48255946)	0,71	0,69	0,273636328
gene:ENSG000	19	complement(48198107..48199720)	0,22	0,25	0,04994166
ZNF114	19	48270081..48287608	0,09	0,02	2,444941163
ODAD1	19	complement(48296457..48321971)	0,36	0,08	2,348426492
EMP3	19	48321509..48330553	39,96	27,56	0,758696559
TMEM143	19	complement(48332356..48364059)	5,33	2,55	1,284013064
SYNGR4	19	48364367..48376377	0,03	0,02	0,524121557
KDELR1	19	complement(48382575..48391551)	183,56	92,75	1,207902423
GRIN2D	19	48393668..48444931	2,01	0,50	2,226535617
GRWD1	19	48445841..48457022	12,87	8,08	0,894954959
KCNJ14	19	48455574..48466980	2,66	1,31	1,248390662
gene:ENSG000	19	48465837..48472431	-	-	0,188027316
CYTH2	19	48469208..48482314	13,34	7,56	1,041488354
LMTK3	19	complement(48485271..48513935)	0,13	0,12	0,367171321
SULT2B1	19	48552172..48599425	0,17	0,14	0,513216292
FAM83E	19	complement(48599961..48615076)	0,09	0,06	0,829733423
SPACA4	19	48606742..48607714	0,02	0,01	0,886720911
RPL18	19	complement(48615328..48619184)	260,99	#####	0,657288733
SPHK2	19	48619291..48630717	3,59	3,08	0,444520012
DBP	19	complement(48630030..48637379)	7,93	2,78	1,735916683
CA11	19	complement(48637946..48646187)	0,78	1,52	-0,740482487
NTN5	19	complement(48661407..48673081)	0,08	0,02	2,233007235
FUT2	19	48695971..48705951	0,97	0,39	1,517319312
MAMSTR	19	complement(48712725..48719725)	1,72	0,76	1,40000245
RASIP1	19	complement(48720585..48740610)	0,14	0,09	0,922447257
IZUMO1	19	complement(48740852..48746909)	0,04	0,04	0,221270597
FUT1	19	complement(48748011..48755390)	0,08	0,50	-2,492365486
FGF21	19	48755524..48758333	0,03	0,59	-3,919206017
BCAT2	19	complement(48795062..48811029)	12,03	11,13	0,335864043
HSD17B14	19	complement(48813018..48836510)	0,05	0,09	-0,698295353
PLEKHA4	19	complement(48837092..48868617)	2,32	1,56	0,802040421
PPP1R15A	19	48872212..48876059	18,29	13,99	0,609836181
TULP2	19	complement(48880965..48898744)	0,01	0,01	-0,923955849
NUCB1	19	48900050..48923473	30,55	17,23	1,04913599
DHDH	19	48933699..48944969	0,05	0,09	-0,509043067
BAX	19	48954815..48961798	18,20	13,11	0,696422449
FTL	19	48965309..48966879	#######	#####	1,456290485
GYS1	19	complement(48968130..48993310)	25,24	12,44	1,243085568
RUVBL2	19	48993562..49015970	56,58	43,43	0,604781719
LHB	19	complement(49015980..49017091)	1,11	0,57	1,186249369
SAXO3	19	complement(49017968..49020549)	0,09	0,07	0,537167373
CGB3	19	complement(49022869..49024333)	0,10	-	5,88622595
gene:ENSG000	19	complement(49022953..49036895)	0,19	0,02	3,601075255

CGB2	19	49031890..49033238	0,02	0,24	-2,94381988
CGB1	19	complement(49035569..49036895)	-	-	0,188027316
CGB5	19	49043848..49045311	0,98	0,56	1,030867436
CGB8	19	complement(49047638..49049111)	7,57	6,50	0,444473099
CGB7	19	complement(49054274..49058860)	0,16	0,16	0,205224342
gene:ENSG000	19	complement(49055793..49065076)	0,06	0,02	1,655502569
NTF4	19	complement(49061066..49065076)	0,43	0,20	1,301444057
KCNA7	19	complement(49067397..49072699)	0,02	0,01	1,704893826
SNRNP70	19	49085419..49108605	35,38	27,47	0,588020348
LIN7B	19	49114324..49118460	3,68	2,25	0,933256286
C19orf73	19	complement(49118402..49119143)	1,22	0,86	0,735478785
PPFIA3	19	49119544..49151026	4,89	1,41	2,014069256
HRC	19	complement(49151198..49155396)	0,13	0,05	1,433404129
TRPM4	19	49157741..49211836	5,87	3,13	1,129088657
SLC6A16	19	complement(49289638..49325215)	0,13	0,04	1,981046522
CD37	19	49335171..49343335	0,74	0,34	1,328171341
TEAD2	19	complement(49340595..49362457)	13,45	9,04	0,796502663
DKKL1	19	49361783..49375116	0,56	0,08	3,046947144
KASH5	19	49388219..49417990	-	-	#N/D
PTH2	19	complement(49422419..49423441)	-	-	#N/D
GFY	19	49423749..49428987	-	-	0,188027316
SLC17A7	19	complement(49429401..49442360)	0,16	0,07	1,475560475
PIH1D1	19	complement(49446298..49453497)	16,13	10,39	0,857870579
ALDH16A1	19	49453225..49471050	7,49	7,03	0,315090435
gene:ENSG000	19	49462752..49486231	-	0,12	-5,327865481
FLT3LG	19	49474207..49486231	1,24	0,94	0,625729881
RPL13A	19	49487510..49493057	460,29	#####	0,56432624
RPS11	19	49496365..49499708	368,15	#####	0,544434992
FCGRT	19	49506816..49526428	3,75	2,00	1,126245259
RCN3	19	49528003..49546962	98,80	43,82	1,395871685
NOSIP	19	complement(49555468..49590262)	8,87	6,30	0,716510058
PRRG2	19	49580646..49591004	0,39	0,24	0,932273602
PRR12	19	49591182..49626439	10,62	8,71	0,508650324
RRAS	19	complement(49635292..49640143)	54,49	20,71	1,618871813
SCAF1	19	49642209..49658642	31,32	19,77	0,88716533
IRF3	19	complement(49659569..49665875)	20,90	10,83	1,171956037
BCL2L12	19	49665142..49673916	8,92	4,96	1,069669568
PRMT1	19	49675786..49689029	57,78	59,10	0,190414326
ADM5	19	49689594..49690575	1,35	1,69	-0,09761566
CPT1C	19	49690898..49713731	0,42	0,07	2,725156265
TSKS	19	complement(49739753..49763306)	0,01	-	3,068695355
AP2A1	19	49767001..49807114	47,36	20,20	1,452334944
FUZ	19	complement(49806866..49817376)	3,47	2,71	0,575825424
MED25	19	49818282..49840383	11,05	7,74	0,735873229
PTOV1	19	49850735..49860744	47,03	29,85	0,878959846
PNKP	19	complement(49859882..49878351)	10,93	6,58	0,95491376
AKT1S1	19	complement(49869033..49878459)	15,07	9,94	0,823381729
TBC1D17	19	49877666..49888750	13,89	8,79	0,883692623

IL4I1	19	complement(49889654..49929539)	0,05	0,33	-2,417501001
NUP62	19	complement(49906825..49929764)	14,78	14,59	0,242375055
gene:ENSG000	19	complement(49909501..49958391)	-	-	#N/D
ATF5	19	49928702..49933935	3,64	4,36	-0,036412736
SIGLEC11	19	complement(49948985..49961172)	0,00	-	3,068695355
SIGLEC16	19	49969600..49975819	0,15	0,06	1,433236741
VRK3	19	complement(49976468..50025946)	3,95	2,38	0,954992432
ZNF473	19	50025714..50053414	3,17	2,29	0,694786456
IZUMO2	19	complement(50152548..50163281)	-	-	0,188027316
MYH14	19	50188186..50310542	0,01	-	4,913749003
KCNC3	19	complement(50311937..50333515)	0,01	-	5,224730805
NR1H2	19	50329653..50383388	18,02	11,23	0,904416582
NAPSA	19	complement(50358472..50365830)	0,01	-	3,068695355
POLD1	19	50384204..50418018	20,29	15,42	0,619061237
gene:ENSG000	19	50415799..50428409	-	-	0,188027316
SPIB	19	50418938..50431314	0,01	0,03	-0,698295353
MYBPC2	19	50432892..50466321	-	-	0,188027316
GARIN5A	19	complement(50466785..50476848)	0,02	0,14	-2,394315395
EMC10	19	50476400..50490871	1,65	2,00	-0,054981018
JOSD2	19	complement(50505998..50511220)	8,47	7,27	0,4440762
ASPDH	19	complement(50511600..50514690)	0,08	0,06	0,537167373
LRRC4B	19	complement(50516892..50568435)	0,29	0,06	2,48867344
SYT3	19	complement(50621307..50639881)	0,02	-	5,697545031
C19orf81	19	50649445..50659310	0,46	0,20	1,409373041
SHANK1	19	complement(50659255..50719802)	2,53	5,13	-0,794600109
CLEC11A	19	50723364..50725708	68,38	28,44	1,488725552
GPR32	19	50770464..50771732	0,02	0,02	-0,025382928
SMIM47	19	complement(50776141..50793142)	0,04	-	4,516772761
ACP4	19	50790415..50795219	0,22	0,09	1,531118931
KLK1	19	complement(50819146..50823787)	-	-	0,188027316
KLK15	19	complement(50825289..50837213)	-	-	0,188027316
KLK3	19	50854915..50860764	-	-	0,188027316
KLK2	19	50861568..50880567	-	-	0,188027316
KLK4	19	complement(50906351..50911395)	-	-	0,188027316
KLK5	19	complement(50943303..50953093)	-	-	0,188027316
KLK6	19	complement(50958631..50969673)	-	-	0,188027316
gene:ENSG000	19	complement(50962197..50962781)	-	-	#N/D
KLK7	19	complement(50976468..50984099)	-	-	0,188027316
KLK8	19	complement(50996007..51002711)	-	-	#N/D
gene:ENSG000	19	complement(50996018..51009581)	-	-	#N/D
KLK9	19	complement(51002508..51009634)	-	-	#N/D
KLK10	19	complement(51012739..51020175)	0,01	0,01	-0,025382928
KLK11	19	complement(51022216..51028039)	-	-	#N/D
KLK12	19	complement(51029092..51035230)	-	-	#N/D
KLK13	19	complement(51055626..51065114)	-	-	0,188027316
KLK14	19	complement(51077495..51084245)	0,18	0,09	1,268358442
CTU1	19	complement(51097606..51108409)	2,70	1,66	0,929360247
SIGLEC9	19	51124906..51136651	-	-	0,188027316

SIGLEC7	19	51142299..51153526	-	-	#N/D
CD33	19	51225064..51243860	0,02	0,06	-1,462209385
SIGLECL1	19	51246348..51269330	-	-	#N/D
IGLON5	19	51311644..51330891	0,11	0,05	1,337990086
VSIG10L	19	complement(51331536..51342139)	0,98	0,60	0,924720131
ETFB	19	complement(51345169..51366388)	13,20	7,21	1,095356744
gene:ENSG000	19	complement(51350373..51368099)	0,20	0,31	-0,411705719
CLDND2	19	complement(51367098..51369003)	1,39	0,53	1,621107955
NKG7	19	complement(51371606..51372701)	0,13	0,10	0,604285378
LIM2	19	complement(51379909..51387974)	-	-	#N/D
C19orf84	19	complement(51388289..51390591)	-	0,02	-3,804623889
SIGLEC10	19	complement(51410020..51417803)	0,15	0,13	0,413995612
SIGLEC8	19	complement(51450847..51458456)	-	0,00	-2,89252005
CEACAM18	19	51478539..51491301	-	-	#N/D
SIGLEC12	19	complement(51491227..51501800)	-	-	#N/D
SIGLEC6	19	complement(51517819..51531856)	0,01	-	4,516772761
ZNF175	19	51571283..51592510	1,20	0,81	0,780884578
SIGLEC5	19	complement(51610960..51630401)	-	0,00	-2,89252005
SIGLEC14	19	complement(51639478..51646825)	-	-	#N/D
SPACA6	19	51688346..51712387	2,33	1,29	1,079554742
HAS1	19	complement(51713112..51723991)	0,01	-	3,967268276
FPR1	19	complement(51745172..51804115)	-	0,01	-2,89252005
FPR2	19	51752026..51770531	-	-	0,188027316
FPR3	19	51795157..51826190	-	0,00	-2,89252005
ZNF577	19	complement(51804816..51890950)	0,12	0,14	-0,053855849
ZNF649	19	complement(51889235..51905040)	0,43	0,63	-0,331743296
ZNF613	19	51927147..51948759	0,41	0,43	0,15645538
ZNF350	19	complement(51964340..51986856)	1,04	0,48	1,333484954
ZNF615	19	complement(51991332..52008230)	0,53	0,58	0,075704237
ZNF614	19	complement(52012765..52030240)	0,36	0,34	0,311798674
ZNF432	19	complement(52031378..52095738)	1,37	0,84	0,930530916
ZNF841	19	complement(52064465..52095765)	3,84	1,78	1,330006875
ZNF616	19	complement(52113091..52139938)	0,77	0,75	0,269748392
ZNF836	19	complement(52153864..52171643)	1,26	0,42	1,805681932
PPP2R1A	19	52170936..52229518	41,17	25,78	0,898662952
ZNF766	19	52269587..52296046	2,14	1,05	1,245320383
ZNF480	19	52297169..52325922	0,30	0,31	0,15645538
ZNF610	19	52336243..52367778	0,04	-	6,338059249
ZNF880	19	52369917..52385795	-	-	0,188027316
ZNF528	19	52397849..52418412	0,70	0,06	3,68424131
ZNF534	19	52429148..52452315	0,01	-	4,516772761
ZNF578	19	52453553..52516882	0,17	0,14	0,563633166
ZNF808	19	52527652..52564464	1,40	0,60	1,437001795
ZNF701	19	52555822..52587174	0,63	0,38	0,967103993
ZNF83	19	complement(52594060..52690559)	2,32	1,71	0,663717196
gene:ENSG000	19	complement(52650447..52690496)	1,37	1,87	-0,2246651
ZNF611	19	complement(52702813..52735073)	1,36	1,18	0,426641668
ZNF600	19	complement(52749942..52786807)	0,37	0,40	0,104509533

ZNF28	19	complement(52797408..52857600)	0,61	0,56	0,338452795
ZNF468	19	complement(52838010..52857649)	0,95	0,90	0,296603898
ZNF320	19	complement(52860851..52897693)	0,58	0,46	0,551823894
ZNF888	19	complement(52904415..52923470)	0,54	0,43	0,536792187
ZNF816-ZNF32	19	complement(52928475..52962823)	0,31	0,48	-0,392435783
ZNF816	19	complement(52949379..52962911)	0,45	0,55	-0,050975961
ERVV-1	19	53013921..53016123	-	-	0,188027316
ERVV-2	19	53044740..53051680	-	0,01	-4,359055975
ZNF160	19	complement(53066606..53103434)	1,81	1,21	0,799653096
ZNF415	19	complement(53107879..53133077)	-	-	0,188027316
ZNF347	19	complement(53124072..53159075)	0,21	0,00	6,267736412
ZNF665	19	complement(53159213..53193386)	0,01	0,01	0,366665713
ZNF677	19	complement(53235381..53254898)	0,00	0,01	-1,478387936
VN1R2	19	53258292..53261837	0,02	0,01	0,886720911
VN1R4	19	complement(53266676..53267723)	-	-	#N/D
FAM90A27P	19	53277462..53284947	-	-	#N/D
ZNF845	19	53333749..53356906	0,66	0,77	-0,003912066
ZNF525	19	53365693..53392217	0,27	0,18	0,801457266
ZNF765	19	53389793..53430413	0,56	0,51	0,354878707
ZNF761	19	53431984..53461862	0,85	0,75	0,411203768
ZNF813	19	53467733..53496255	-	0,00	-2,89252005
ZNF331	19	53519527..53580269	1,55	1,46	0,305948178
DPRX	19	53600777..53637014	-	-	#N/D
NLRP12	19	complement(53793741..53824403)	0,00	-	3,068695355
MYADM	19	53864763..53876435	43,94	31,96	0,682301353
PRKCG	19	53879190..53907652	0,02	0,07	-1,599259329
CACNG7	19	53909278..53943950	-	0,01	-3,804623889
CACNG8	19	53962937..53990215	0,84	1,00	-0,034540514
CACNG6	19	53991637..54012666	0,21	0,31	-0,361316456
VSTM1	19	complement(54040825..54063953)	-	-	0,188027316
TARM1	19	complement(54069895..54081365)	0,01	-	3,068695355
OSCAR	19	complement(54094668..54102692)	0,36	0,44	-0,060834107
NDUFA3	19	54102728..54109257	7,78	6,06	0,582214427
TFPT	19	complement(54107020..54115657)	9,03	4,56	1,208942733
PRPF31	19	54115410..54131719	15,19	11,42	0,634825926
CNOT3	19	54137749..54155681	13,83	6,70	1,268941522
LENG1	19	complement(54155161..54159721)	3,08	2,18	0,716728063
TMC4	19	complement(54160108..54173250)	0,31	0,31	0,233642345
MBOAT7	19	complement(54173412..54189882)	20,22	15,32	0,623181706
TSEN34	19	54189938..54194536	11,77	8,24	0,736953195
RPS9	19	54200809..54249003	65,18	64,27	0,243275682
LILRB3	19	complement(54216278..54223506)	0,25	0,31	-0,06002136
LILRA6	19	complement(54236590..54242790)	0,09	0,04	1,365192532
LILRB5	19	complement(54249421..54257301)	-	0,00	-2,89252005
LILRB2	19	complement(54273812..54281184)	-	-	0,188027316
LILRA5	19	complement(54307070..54313166)	-	-	#N/D
LILRA4	19	complement(54333185..54339162)	-	-	0,188027316
LAIR1	19	complement(54351384..54370558)	0,01	0,00	0,524121557

TTYH1	19	54415219..54436904	0,15	0,18	-0,081545845
LENG8	19	54449190..54462016	60,13	30,86	1,18520289
LENG9	19	complement(54461732..54463778)	1,33	1,58	-0,029261555
CDC42EP5	19	complement(54465026..54473296)	3,27	1,23	1,635748447
LAIR2	19	54497879..54510687	-	-	0,188027316
LILRA2	19	54572920..54590287	-	-	#N/D
LILRA1	19	54593582..54602381	-	-	#N/D
LILRB1	19	54617158..54638022	-	-	0,188027316
LILRB4	19	54643889..54670359	-	-	#N/D
KIR3DL3	19	54724442..54736632	-	-	#N/D
KIR2DL3	19	54738513..54753052	-	-	0,188027316
KIR2DL1	19	54769793..54784322	-	-	#N/D
KIR2DL4	19	54803610..54814517	-	-	#N/D
KIR3DL1	19	54816468..54830778	-	-	#N/D
KIR2DS4	19	54832676..54848569	-	-	#N/D
KIR3DL2	19	54850443..54867207	-	-	#N/D
FCAR	19	54874235..54891420	0,02	0,01	1,15046174
NCR1	19	54906148..54916140	0,01	-	3,967268276
NLRP7	19	complement(54923509..54966312)	-	0,00	-2,89252005
NLRP2	19	54953130..55001142	-	-	0,188027316
GP6	19	complement(55013705..55038264)	0,19	0,01	4,393997427
RDH13	19	complement(55039108..55071291)	1,57	1,26	0,533213216
EPS8L1	19	55072020..55087923	0,12	0,07	0,987802155
PPP1R12C	19	complement(55090914..55117637)	9,90	9,35	0,306812639
TNNT1	19	complement(55132698..55149206)	3,09	10,26	-1,509126673
TNNI3	19	complement(55151767..55157773)	0,49	0,15	1,897091915
gene:ENSG000	19	complement(55154757..55160671)	0,02	-	3,967268276
DNAAF3	19	complement(55158661..55166722)	2,31	1,22	1,146175624
SYT5	19	complement(55171196..55180289)	0,14	0,14	0,151849832
PTPRH	19	complement(55181247..55209506)	1,31	2,46	-0,68694528
TMEM86B	19	complement(55226638..55228784)	4,29	1,95	1,358153023
PPP6R1	19	complement(55229779..55259017)	42,12	27,09	0,859613149
HSPBP1	19	complement(55262223..55280381)	12,86	15,05	-0,003558887
BRSK1	19	55282072..55312562	0,40	0,99	-1,075080309
TMEM150B	19	complement(55312801..55334048)	-	0,05	-4,758582517
KMT5C	19	55339853..55348121	5,91	4,06	0,765819775
COX6B2	19	complement(55349306..55354719)	0,42	0,41	0,238196852
gene:ENSG000	19	complement(55352301..55359516)	0,01	0,05	-1,983268216
GARIN5B	19	complement(55354908..55363260)	0,02	0,04	-0,759769633
IL11	19	complement(55364382..55370463)	2,14	2,17	0,204325329
TMEM190	19	55376826..55378246	0,27	0,34	-0,130997631
TMEM238	19	complement(55379244..55384292)	0,25	0,47	-0,678357384
RPL28	19	55385932..55403250	51,65	37,89	0,670053001
UBE2S	19	complement(55399745..55407788)	121,09	70,12	1,011263953
SHISA7	19	complement(55428740..55443300)	0,06	0,03	0,954435988
ISOC2	19	complement(55452985..55462343)	22,81	11,89	1,163272322
C19orf85	19	complement(55463001..55464751)	0,05	0,03	0,933337975
gene:ENSG000	19	complement(55467438..55467934)	-	-	0,188027316

ZNF628	19	55476617..55484487	2,59	2,33	0,376932477
NAT14	19	55485188..55487568	13,63	7,73	1,042116708
SSC5D	19	55488404..55519099	0,68	0,12	2,732763399
SBK2	19	complement(55528611..55537133)	0,03	0,09	-1,213543126
SBK3	19	complement(55540656..55545543)	0,55	0,26	1,287000481
ZNF579	19	complement(55576774..55580848)	3,89	2,98	0,611129318
FIZ1	19	complement(55591376..55601970)	1,98	1,78	0,37721508
ZNF524	19	55600022..55603138	4,00	3,31	0,498848316
ZNF865	19	55605647..55617269	5,05	2,15	1,452734599
ZNF784	19	complement(55620741..55624566)	1,49	1,16	0,588295312
ZNF580	19	55635016..55643470	5,55	3,81	0,765850427
ZNF581	19	55635459..55645623	15,69	6,75	1,440415274
CCDC106	19	55641062..55653161	2,14	1,70	0,551508953
U2AF2	19	55654146..55674716	35,47	46,32	-0,161794016
EPN1	19	55675226..55709858	8,44	6,17	0,676392794
NLRP9	19	complement(55708438..55738402)	-	-	0,188027316
RFPL4A	19	55759097..55763421	-	0,01	-2,89252005
RFPL4AL1	19	55769118..55773420	0,01	-	3,068695355
NLRP11	19	complement(55785397..55836800)	-	0,01	-4,359055975
NLRP4	19	55836540..55881855	-	-	#N/D
NLRP13	19	complement(55891699..55932336)	-	-	#N/D
NLRP8	19	55947832..55988629	-	-	#N/D
NLRP5	19	55999726..56061810	-	-	0,188027316
ZNF787	19	complement(56087366..56121295)	7,82	6,41	0,508869003
gene:ENSG000	19	complement(56127236..56127466)	-	-	0,188027316
ZNF444	19	56132599..56160893	5,59	3,88	0,749261181
gene:ENSG000	19	56149817..56150490	0,70	0,58	0,510367793
GALP	19	56176008..56185775	-	-	0,188027316
ZSCAN5B	19	complement(56189686..56197815)	0,01	-	3,967268276
ZSCAN5C	19	56202273..56209461	-	-	0,188027316
ZSCAN5A	19	complement(56219670..56368383)	0,26	0,37	-0,311296333
EDDM13	19	56272748..56310454	0,06	0,11	-0,609885528
ZNF582	19	complement(56375846..56393538)	0,00	-	3,068695355
ZNF583	19	56397966..56436035	0,05	0,10	-0,797852434
ZNF667	19	complement(56439325..56478065)	-	-	0,188027316
gene:ENSG000	19	complement(56480397..56483977)	-	-	0,188027316
ZNF471	19	56507850..56530221	-	-	0,188027316
ZFP28	19	56538948..56556808	0,45	-	10,97272781
ZNF470	19	56567468..56588911	0,08	-	8,66956442
ZNF71	19	56595300..56626481	2,02	1,79	0,399754217
SMIM17	19	56643159..56657247	0,06	0,01	2,787439322
ZNF835	19	complement(56661980..56671783)	-	-	0,188027316
ZIM2	19	complement(56774547..56840731)	-	-	#N/D
PEG3	19	complement(56810077..56840728)	-	-	#N/D
USP29	19	57119138..57131926	0,00	-	3,068695355
ZIM3	19	complement(57134096..57145202)	-	0,01	-3,804623889
DUXA	19	complement(57154021..57167485)	-	-	#N/D
ZNF264	19	57191500..57222846	3,76	2,24	0,969678127

AURKC	19	57231009..57235548	0,87	0,19	2,438446364
ZNF805	19	57240632..57262728	4,52	1,81	1,54568881
ZNF460	19	57280051..57294069	16,21	10,01	0,919395498
gene:ENSG000	19	complement(57293083..57294469)	3,33	2,13	0,867375484
ZNF543	19	57320472..57330770	1,98	1,86	0,312842273
ZNF304	19	57351271..57359898	3,44	1,69	1,242406071
ZNF547	19	57363477..57379565	0,48	0,42	0,401408031
TRAPPC2B	19	57363551..57365405	2,66	1,03	1,58490372
gene:ENSG000	19	57363566..57398584	-	-	0,188027316
ZNF548	19	57389850..57402992	2,27	1,04	1,347938136
gene:ENSG000	19	57389884..57419994	-	-	0,188027316
gene:ENSG000	19	57394183..57438457	1,35	1,54	0,027655372
ZNF17	19	57411161..57421934	1,51	0,92	0,936354809
ZNF749	19	57435325..57447101	2,48	1,70	0,765612567
gene:ENSG000	19	complement(57437970..57477536)	0,08	-	5,224730805
VN1R1	19	complement(57454790..57457140)	0,44	0,31	0,735634202
ZNF772	19	complement(57466663..57477570)	0,72	1,03	-0,293112573
ZNF419	19	57487711..57496098	1,20	0,81	0,786230891
gene:ENSG000	19	57487718..57517559	0,24	0,30	-0,068174358
ZNF773	19	57499915..57518404	0,68	0,70	0,192366591
ZNF549	19	57527325..57557542	1,33	0,57	1,435721421
ZNF550	19	complement(57535257..57559863)	3,99	1,46	1,679798125
ZNF416	19	complement(57571566..57578911)	1,30	1,31	0,209973323
ZIK1	19	57578456..57593890	0,04	0,01	1,699751701
ZNF530	19	57599885..57612722	1,43	0,53	1,647773858
ZNF134	19	57614233..57624724	2,96	1,77	0,965163133
ZNF211	19	57630395..57644041	1,06	0,71	0,80404459
ZSCAN4	19	57668935..57679152	-	-	0,188027316
ZNF551	19	57681969..57717301	0,80	0,42	1,138807709
gene:ENSG000	19	57682045..57753575	0,53	-	7,772593938
ZNF154	19	complement(57696275..57709204)	0,12	0,07	1,069204515
ZNF671	19	complement(57719751..57727624)	0,58	0,60	0,179001275
ZNF776	19	57746815..57758148	1,89	1,34	0,716438116
ZNF586	19	57769655..57819939	1,69	1,12	0,812349414
ZNF552	19	complement(57803841..57814913)	1,32	0,83	0,891578803
gene:ENSG000	19	57819719..57858941	6,18	2,70	1,414835582
ZNF587B	19	57819721..57846238	2,00	0,96	1,271233676
ZNF814	19	complement(57848731..57889074)	1,16	0,54	1,319485298
ZNF587	19	57849859..57865117	14,80	5,59	1,628108829
gene:ENSG000	19	complement(57876765..57916591)	0,01	-	3,068695355
ZNF417	19	complement(57900296..57916610)	4,01	1,47	1,668935971
ZNF418	19	complement(57921882..57935402)	0,03	-	6,20258586
ZNF256	19	complement(57940833..57947706)	0,53	-	9,615152313
C19orf18	19	complement(57958437..57974534)	0,01	-	3,068695355
ZNF606	19	complement(57977076..58003349)	0,19	0,14	0,623318671
ZSCAN1	19	58034025..58054631	-	0,01	-2,89252005
ZNF135	19	58059239..58086310	0,00	-	3,068695355
ZSCAN18	19	complement(58083838..58118427)	-	0,00	-2,89252005

ZNF329	19	complement(58126248..58155110)	1,34	1,01	0,632736133
ZNF274	19	58183029..58213562	1,38	1,87	-0,219721482
ZNF544	19	58228594..58277495	0,21	0,20	0,29338804
gene:ENSG000	19	58228914..58315183	1,60	1,16	0,683645476
ZNF8	19	58278955..58302791	3,90	2,54	0,843129314
ERVK3-1	19	58305374..58316812	3,91	2,31	0,983952196
ZSCAN22	19	58326994..58342332	1,99	1,22	0,925697414
A1BG	19	complement(58345178..58353492)	2,54	0,22	3,772274966
gene:ENSG000	19	58350795..58351720	1,87	0,81	1,430129332
ZNF497	19	complement(58354357..58362848)	1,70	1,41	0,494497469
ZNF837	19	complement(58367618..58381030)	0,26	0,25	0,317146527
RPS5	19	58386400..58394806	180,91	#####	0,701564325
RNF225	19	58395656..58397079	0,10	0,24	-1,030971428
ZNF584	19	58401504..58431736	2,25	1,80	0,548195212
gene:ENSG000	19	58418560..58419310	0,22	0,22	0,165798947
ZNF132	19	complement(58432814..58440153)	0,11	0,20	-0,575538021
ZNF324B	19	58451611..58457833	1,47	1,23	0,487501826
ZNF324	19	58467056..58475436	3,52	1,70	1,274671906
ZNF446	19	58474017..58481230	1,74	1,52	0,4134697
SLC27A5	19	complement(58479512..58512413)	1,13	0,89	0,56174816
ZBTB45	19	complement(58513530..58538911)	2,66	2,80	0,152325225
TRIM28	19	58544064..58550722	89,68	67,75	0,627610677
CHMP2A	19	complement(58551452..58555105)	15,89	9,91	0,90480087
UBE2M	19	complement(58555712..58558954)	58,78	39,76	0,787031697
MZF1	19	complement(58561931..58573575)	4,80	2,42	1,210977212
DEFB125	20	87250..97094	-	-	#N/D
DEFB126	20	142590..145751	-	-	0,188027316
DEFB127	20	157454..159163	-	-	#N/D
DEFB128	20	complement(187853..189711)	-	-	#N/D
DEFB129	20	227258..229886	-	-	#N/D
DEFB132	20	257724..261096	-	-	#N/D
C20orf96	20	complement(270863..290778)	2,95	3,67	-0,088694181
ZCCHC3	20	297570..300321	4,25	6,33	-0,351801109
SOX12	20	325552..330224	10,27	12,03	-0,004126413
NRSN2	20	346782..359660	7,19	7,03	0,255090136
TRIB3	20	362835..397559	8,99	24,82	-1,242572076
RBCK1	20	407498..432139	11,19	12,87	0,020295084
TBC1D20	20	complement(423596..462566)	9,46	7,03	0,65150723
CSNK2A1	20	complement(472498..543835)	5,64	8,11	-0,300989934
TCF15	20	complement(604257..610309)	0,04	-	4,913749004
SRXN1	20	complement(646615..653200)	18,56	19,30	0,166665868
gene:ENSG000	20	complement(646626..675800)	-	-	#N/D
SCRT2	20	complement(661596..675802)	-	-	#N/D
SLC52A3	20	complement(760080..776015)	-	0,00	-2,89252005
FAM110A	20	833715..857463	2,75	3,06	0,069259785
ANGPT4	20	complement(869900..916334)	0,06	0,10	-0,360253908
RSPO4	20	complement(958452..1002311)	1,76	2,90	-0,499246694
PSMF1	20	1113240..1189415	4,46	5,48	-0,073295348

TMEM74B	20	complement(1180561..1185415)	0,13	0,04	1,887651853
C20orf202	20	1203454..1209076	0,06	0,03	1,422471951
RAD21L1	20	1226044..1296421	0,21	0,21	0,198282257
SNPH	20	1266280..1309328	5,46	4,02	0,664961359
SDCBP2	20	complement(1309909..1329139)	1,23	0,43	1,715458647
gene:ENSG000	20	complement(1317571..1393096)	0,19	0,05	2,209934656
gene:ENSG000	20	1320710..1324367	0,08	0,02	1,833525048
FKBP1A	20	complement(1368977..1393164)	45,21	34,08	0,630808718
NSFL1C	20	complement(1442162..1473842)	11,64	15,63	-0,202415424
SIRPB2	20	complement(1470741..1491587)	0,00	0,01	-1,877914477
SIRPD	20	complement(1534251..1557705)	-	-	#N/D
gene:ENSG000	20	complement(1540144..1620009)	-	-	#N/D
SIRPB1	20	complement(1561385..1620061)	0,03	0,10	-1,617776405
SIRPG	20	complement(1629152..1657779)	-	0,03	-5,071123353
SIRPA	20	1894167..1940592	7,66	6,08	0,556192181
PDYN	20	complement(1978757..1994285)	-	-	#N/D
STK35	20	2101827..2177038	7,38	6,26	0,459039664
gene:ENSG000	20	2207328..2341059	-	-	#N/D
TGM3	20	2296001..2341079	-	-	0,188027316
TGM6	20	2380901..2432753	-	-	0,188027316
SNRPB	20	complement(2461634..2470853)	83,74	93,57	0,062917876
gene:ENSG000	20	complement(2467212..2508907)	0,10	0,05	1,339142659
ZNF343	20	complement(2481817..2524702)	2,28	2,50	0,089425732
TMC2	20	2536573..2643580	0,06	0,20	-1,387619608
NOP56	20	2652593..2658393	13,15	25,60	-0,737602363
IDH3B	20	complement(2658395..2664219)	21,18	26,41	-0,095614739
EBF4	20	2692780..2760108	0,21	0,32	-0,357589173
CPXM1	20	complement(2794074..2800627)	2,09	2,38	0,039603733
gene:ENSG000	20	2814968..2818066	-	0,06	-5,734998842
C20orf141	20	2814987..2815833	-	0,15	-5,902279478
TMEM239	20	2816302..2820284	0,01	0,03	-2,123038518
PCED1A	20	complement(2835314..2841190)	9,14	11,08	-0,055467346
VPS16	20	2840703..2866732	6,93	7,46	0,116758664
PTPRA	20	2873420..3039076	6,58	9,75	-0,344642015
GNRH2	20	3043622..3045747	0,05	0,20	-1,700542576
MRPS26	20	3046052..3048250	15,70	23,91	-0,383870571
OXT	20	3071620..3072517	-	-	#N/D
AVP	20	complement(3082556..3084724)	-	-	#N/D
UBOX5	20	complement(3107573..3160196)	3,40	2,95	0,426868714
FASTKD5	20	complement(3146519..3159865)	2,85	2,82	0,23800197
LZTS3	20	complement(3162617..3173549)	1,22	1,01	0,493775171
DDRGK1	20	complement(3190350..3204685)	5,64	7,87	-0,256495424
ITPA	20	3208868..3223870	7,61	10,54	-0,246082318
SLC4A11	20	complement(3227417..3239559)	0,46	0,74	-0,457559564
DNAAF9	20	complement(3249306..3407669)	0,96	1,61	-0,520467953
ATRN	20	3471018..3651118	3,81	4,48	-0,0135898
GFRA4	20	complement(3659248..3663399)	-	-	0,188027316
ADAM33	20	complement(3667965..3682246)	3,56	2,47	0,751309942

SIGLEC1	20	complement(3686970..3712600)	0,03	0,01	2,132400326
HSPA12B	20	3732685..3753111	0,14	0,14	0,23645756
ADISSP	20	complement(3753508..3767781)	20,61	24,58	-0,030825894
SPEF1	20	complement(3777504..3781448)	0,12	0,07	1,117604237
CENPB	20	complement(3783851..3786740)	35,43	31,26	0,4038263
CDC25B	20	3786772..3806121	29,89	39,59	-0,182766986
AP5S1	20	3820524..3828838	2,48	2,86	0,016315797
MAVS	20	3846799..3876123	8,97	10,32	0,020366403
PANK2	20	3888839..3929887	2,09	2,38	0,034717331
RNF24	20	complement(3927309..4015558)	9,66	12,47	-0,145095195
SMOX	20	4120980..4187747	12,73	14,75	0,010497373
ADRA1D	20	complement(4220630..4249287)	0,27	0,33	-0,059764962
PRNP	20	4686350..4701590	25,09	36,76	-0,32790564
PRND	20	4721909..4728460	-	0,01	-4,359055975
RASSF2	20	complement(4780023..4823608)	1,07	1,89	-0,588618262
SLC23A2	20	complement(4852356..5010293)	5,66	6,45	0,034598947
TMEM230	20	complement(5068232..5113087)	12,01	18,34	-0,388103435
PCNA	20	complement(5114953..5126626)	25,58	56,02	-0,907692024
CDS2	20	5126879..5197887	5,57	7,50	-0,204958125
PROKR2	20	complement(5299218..5316954)	0,00	0,00	0,886720911
GPCPD1	20	complement(5544439..5611006)	3,45	7,19	-0,834840663
SHLD1	20	5750393..5864395	0,41	0,96	-1,004158593
CHGB	20	5911510..5925353	0,01	0,05	-1,723418138
TRMT6	20	complement(5937228..5950558)	1,62	2,43	-0,365900897
MCM8	20	5950652..5998977	4,32	4,42	0,192701769
gene:ENSG000	20	5950949..6039856	0,02	0,05	-0,791308264
CRLS1	20	6006093..6040053	1,73	2,03	-0,005434516
LRRN4	20	complement(6040546..6054060)	0,17	0,12	0,75245664
FERMT1	20	complement(6074845..6123214)	0,47	0,54	0,022566356
BMP2	20	6767686..6780246	3,44	0,76	2,409554521
HAO1	20	complement(7882985..7940458)	0,03	0,01	2,144183439
TMX4	20	complement(7977346..8019805)	10,29	12,74	-0,084417986
PLCB1	20	8077251..8968360	0,35	0,61	-0,560477059
PLCB4	20	9067825..9504593	0,22	0,37	-0,568617253
LAMP5	20	9514358..9530524	-	-	0,188027316
PAK5	20	complement(9537370..9839076)	-	-	#N/D
ANKEF1	20	9986126..10058303	0,53	1,80	-1,534537227
SNAP25	20	10172395..10308258	0,13	0,45	-1,533422976
MKKS	20	complement(10401009..10434222)	1,08	3,02	-1,254594007
gene:ENSG000	20	complement(10413520..10434222)	0,42	0,81	-0,706992932
gene:ENSG000	20	complement(10413708..10434222)	0,59	1,68	-1,291457985
SLX4IP	20	10435305..10636829	0,88	1,80	-0,807326698
JAG1	20	complement(10637684..10673999)	8,55	11,73	-0,233001731
BTBD3	20	11890723..11926609	4,05	9,08	-0,940590692
SPTLC3	20	13008972..13169103	0,86	0,35	1,493384751
ISM1	20	13221274..13300651	0,02	0,21	-3,322038906
TASP1	20	complement(13389392..13638932)	1,84	2,08	0,041044528
ESF1	20	complement(13714322..13784919)	0,78	2,38	-1,385312849

NDUFAF5	20	13785007..13821580	1,03	1,91	-0,661106167
SEL1L2	20	complement(13849247..13996443)	-	-	0,188027316
MACROD2	20	13995369..16053197	0,04	0,00	4,110837235
FLRT3	20	complement(14322985..14337614)	0,01	-	5,480421266
KIF16B	20	complement(16272104..16573448)	1,01	1,72	-0,54971667
SNRPB2	20	16729961..16742564	6,68	7,85	-0,009510212
OTOR	20	16748358..16770062	-	-	#N/D
PCSK2	20	17226107..17484578	-	-	#N/D
BFSP1	20	complement(17493905..17569220)	0,45	0,28	0,906256729
DSTN	20	17570075..17609919	12,22	12,71	0,166484192
RRBP1	20	complement(17613678..17682295)	30,78	20,13	0,83561534
BANF2	20	17693672..17735871	-	0,01	-2,89252005
SNX5	20	complement(17941597..17968980)	10,13	11,42	0,049585833
OVOL2	20	complement(17956979..18059188)	-	-	#N/D
MGME1	20	17969018..17991122	6,48	8,36	-0,144683923
KAT14	20	18137228..18188387	1,48	2,72	-0,654679196
PET117	20	18137863..18143169	0,57	0,78	-0,217759334
ZNF133	20	18288283..18316996	2,72	2,32	0,450870498
DZANK1	20	complement(18383367..18467281)	0,09	0,08	0,352372652
POLR3F	20	18466878..18484648	0,71	1,12	-0,443524287
RBBP9	20	complement(18486540..18497225)	3,08	4,26	-0,246403086
SEC23B	20	18507520..18561415	12,93	15,69	-0,055765612
SMIM26	20	18567347..18569563	7,86	8,40	0,127251497
gene:ENSG000	20	18567453..18744216	6,61	11,25	-0,544366268
DTD1	20	18587942..18766644	1,08	2,09	-0,732808242
SCP2D1	20	18813783..18814378	-	-	#N/D
SLC24A3	20	19212642..19722926	-	0,01	-3,804623889
RIN2	20	19757606..20002457	4,74	9,36	-0,75825114
NAA20	20	20017310..20033655	9,37	10,58	0,047580721
CRNKL1	20	complement(20034368..20056046)	2,17	3,18	-0,326166851
CFAP61	20	20052514..20360703	0,03	0,04	-0,29959007
gene:ENSG000	20	20363691..20366502	-	-	#N/D
INSM1	20	20368104..20370949	-	0,00	-2,89252005
RALGAPA2	20	complement(20389530..20712644)	2,18	2,04	0,319219137
KIZ	20	21125983..21246622	1,08	1,53	-0,270886309
XRN2	20	21303331..21389825	14,89	19,45	-0,162539841
NKX2-4	20	complement(21395365..21397526)	-	-	#N/D
NKX2-2	20	complement(21511017..21514064)	3,26	4,46	-0,230064479
PAX1	20	21705659..21718481	0,03	-	6,68162262
FOXA2	20	complement(22580998..22585455)	1,29	2,55	-0,759646329
SSTR4	20	23035312..23039237	-	-	0,188027316
THBD	20	complement(23045633..23049672)	2,54	0,86	1,780475733
gene:ENSG000	20	complement(23071547..23072722)	-	-	#N/D
CD93	20	complement(23079360..23086324)	-	-	0,188027316
gene:ENSG000	20	23148086..23149939	0,19	1,03	-2,244576619
NXT1	20	23350791..23354771	18,99	13,69	0,694746384
GZF1	20	23362182..23373062	4,82	2,91	0,950214149
NAPB	20	complement(23374519..23421519)	1,18	1,13	0,292907112

CSTL1	20	23426104..23444930	-	-	#N/D
CST11	20	complement(23450403..23452876)	-	-	#N/D
CST8	20	23491101..23496010	-	-	#N/D
CST9L	20	complement(23564732..23568484)	-	-	#N/D
CST9	20	complement(23602410..23605917)	-	-	0,188027316
CST3	20	complement(23626706..23638473)	11,12	13,45	-0,050900461
CST4	20	complement(23685640..23689038)	0,36	0,99	-1,224267722
CST1	20	complement(23747562..23751268)	1,10	5,14	-1,997295826
CST2	20	complement(23823769..23826729)	-	0,12	-5,545746556
CST5	20	complement(23875934..23879748)	-	-	0,188027316
GGTLC1	20	complement(23985050..23988779)	-	-	0,188027316
SYNDIG1	20	24469629..24666616	0,03	-	5,224730805
CST7	20	24949269..24959928	0,04	0,03	0,524121557
APMAP	20	complement(24962925..24992751)	18,93	32,22	-0,544315371
ACSS1	20	complement(25006230..25058980)	0,07	0,03	1,459442463
VSX1	20	complement(25070885..25082141)	-	-	0,188027316
ENTPD6	20	25195693..25228075	9,53	11,52	-0,049398708
gene:ENSG000	20	complement(25226448..25226735)	-	-	#N/D
PYGB	20	25248085..25298012	32,53	35,60	0,093016638
ABHD12	20	complement(25294742..25390835)	10,16	9,30	0,350252547
GINS1	20	25391008..25452700	5,51	5,16	0,318616836
NINL	20	complement(25451594..25585531)	1,30	2,94	-0,954839066
NANP	20	complement(25612935..25624014)	1,89	1,83	0,267013902
ZNF337	20	complement(25673195..25696853)	2,88	3,11	0,113159747
DEFB115	20	31257664..31259632	-	-	#N/D
DEFB116	20	complement(31303212..31308585)	-	-	#N/D
DEFB118	20	31368601..31373923	-	-	#N/D
DEFB119	20	complement(31377164..31390590)	-	-	#N/D
DEFB121	20	complement(31404845..31412838)	-	-	#N/D
DEFB123	20	31440632..31450257	-	-	#N/D
DEFB124	20	complement(31465471..31476757)	-	-	#N/D
REM1	20	31475288..31484895	-	-	#N/D
HM13	20	31514428..31577923	23,47	15,72	0,801104063
MCTS2	20	31547379..31548081	4,89	2,89	0,981387915
ID1	20	31605283..31606515	56,81	57,75	0,199236792
COX4I2	20	31637912..31645006	-	-	#N/D
BCL2L1	20	complement(31664452..31723989)	17,16	17,41	0,20228468
TPX2	20	31739271..31801805	83,61	81,08	0,267369141
MYLK2	20	31819308..31834689	0,05	0,13	-1,130751077
FOXS1	20	complement(31844303..31845604)	0,04	0,07	-0,509043067
DUSP15	20	complement(31847637..31870664)	0,06	0,08	-0,189986357
TTLL9	20	31870634..31945000	0,04	0,03	0,350150203
PDRG1	20	complement(31944337..31952046)	9,04	9,16	0,203967386
XKR7	20	31968151..32003387	-	0,00	-3,804623889
CCM2L	20	32010438..32032180	-	0,00	-2,89252005
HCK	20	32052197..32101856	-	-	0,188027316
TM9SF4	20	32109714..32167258	39,10	29,89	0,61027682
PLAGL2	20	complement(32192504..32207743)	5,56	7,06	-0,120717483

POFUT1	20	32207855..32238658	28,15	27,47	0,258209486
KIF3B	20	32277651..32335011	9,37	6,46	0,7591396
ASXL1	20	32358330..32439319	21,25	18,33	0,435976299
NOL4L	20	complement(32443059..32585333)	3,28	3,13	0,291761797
C20orf203	20	complement(32631625..32673941)	0,16	0,02	3,17991591
gene:ENSG000	20	complement(32632183..32743567)	0,12	0,07	1,09830913
COMMD7	20	complement(32702699..32743467)	15,69	16,36	0,16281331
DNMT3B	20	32762385..32809359	3,50	3,81	0,096986979
MAPRE1	20	32819954..32850405	52,17	47,32	0,363946576
EFCAB8	20	32858923..32961845	0,02	0,01	1,106450337
SUN5	20	complement(32983773..33004433)	-	-	#N/D
BPIFB2	20	33007704..33023703	-	-	0,188027316
BPIFB6	20	33031648..33044047	-	-	#N/D
BPIFB3	20	33053903..33073847	-	-	#N/D
BPIFB4	20	33079643..33111751	-	-	0,188027316
BPIFA2	20	33161768..33181412	-	-	0,188027316
BPIFA3	20	33217310..33227806	-	-	#N/D
BPIFA1	20	33235995..33243311	-	-	#N/D
BPIFB1	20	33273480..33309871	-	-	0,188027316
CDK5RAP1	20	complement(33358839..33401561)	14,99	12,53	0,481643167
SNTA1	20	complement(33407957..33443763)	7,37	5,55	0,633130164
CBFA2T2	20	33490075..33650036	7,05	4,71	0,80303267
NECAB3	20	complement(33657087..33674463)	7,69	6,64	0,435094239
C20orf144	20	33662327..33665619	0,31	0,22	0,689783732
ACTL10	20	33666943..33668525	0,64	1,20	-0,684872237
E2F1	20	complement(33675477..33686385)	30,73	22,71	0,659592382
PXMP4	20	complement(33702758..33720319)	2,16	0,92	1,457152115
ZNF341	20	33731657..33792269	2,75	2,65	0,273976028
CHMP4B	20	33811348..33854366	27,89	27,11	0,263825336
RALY	20	33993646..34108308	16,54	15,25	0,340350868
EIF2S2	20	complement(34088309..34112243)	38,73	39,63	0,190046352
ASIP	20	34194569..34269344	0,11	0,18	-0,446980808
AHCY	20	complement(34280268..34311802)	81,34	76,06	0,320068209
ITCH	20	34363235..34511773	8,38	6,88	0,507746303
gene:ENSG000	20	34363271..34540923	5,77	5,41	0,313690353
DYNLRB1	20	34516395..34540958	28,77	26,74	0,328629905
MAP1LC3A	20	34546854..34560345	3,73	1,94	1,160565308
PIGU	20	complement(34560542..34698790)	31,57	25,71	0,519181133
NCOA6	20	complement(34689097..34825651)	6,19	8,48	-0,230579224
TP53INP2	20	34704339..34713439	3,35	0,81	2,280475808
GGT7	20	complement(34844720..34872856)	4,20	1,95	1,328426783
ACSS2	20	34872146..34927962	12,66	16,36	-0,146790824
GSS	20	complement(34928432..34956027)	17,57	24,11	-0,233219627
MYH7B	20	34955810..35002437	0,74	0,57	0,588123224
TRPC4AP	20	complement(35002404..35092807)	50,03	42,34	0,463705303
EDEM2	20	complement(35115364..35147336)	24,36	22,00	0,369634749
PROCR	20	35172072..35216240	15,31	17,79	0,005994808
MMP24OS	20	complement(35201745..35278131)	29,58	16,58	1,058524231

MMP24	20	35226690..35276998	1,73	1,21	0,736868569
gene:ENSG000	20	complement(35262727..35264187)	2,64	1,89	0,707948476
gene:ENSG000	20	complement(35267885..35280043)	1,49	1,25	0,475830586
EIF6	20	complement(35278907..35284985)	81,48	87,27	0,12400746
FAM83C	20	complement(35285731..35292425)	0,07	0,07	0,416777345
UQCC1	20	complement(35302566..35412031)	13,33	10,86	0,518077971
GDF5	20	complement(35433347..35454746)	0,10	0,05	1,212352745
CEP250	20	35455164..35519280	3,71	4,28	0,015837884
C20orf173	20	complement(35523186..35529652)	0,01	-	3,068695355
ERGIC3	20	35542038..35557634	123,54	81,58	0,821690741
SPAG4	20	35615829..35621094	2,59	1,07	1,504964329
CPNE1	20	complement(35626031..35664956)	98,25	77,77	0,560300218
gene:ENSG000	20	complement(35632340..35674544)	0,01	-	3,068695355
RBM12	20	complement(35648925..35664956)	15,22	11,69	0,604471518
NFS1	20	complement(35668052..35699355)	10,86	13,15	-0,052235387
ROMO1	20	35699272..35700984	52,25	39,23	0,636655856
RBM39	20	complement(35701347..35742312)	21,28	19,96	0,315242977
PHF20	20	35771974..35950370	14,41	12,97	0,374906512
SCAND1	20	complement(35953617..35959472)	6,39	7,17	0,05501259
CNBD2	20	35954564..36030700	0,31	0,01	4,750359389
EPB41L1	20	36091504..36232799	5,17	3,77	0,680288698
AAR2	20	36236131..36270918	9,87	10,76	0,09910284
DLGAP4	20	36306336..36528637	25,97	17,97	0,754594115
MYL9	20	36541497..36551447	0,55	0,01	6,026971921
TGIF2	20	36573488..36593950	9,03	11,03	-0,065365085
TGIF2-RAB5IF	20	36574553..36612384	6,38	2,65	1,488725558
RAB5IF	20	36605779..36612557	40,06	35,13	0,412365375
SLA2	20	complement(36612318..36646196)	0,33	0,32	0,280867888
NDRG3	20	complement(36651766..36746090)	32,98	20,06	0,94041533
DSN1	20	complement(36751791..36773818)	13,77	10,73	0,582872419
MTCL2	20	complement(36777447..36863538)	17,62	17,15	0,262648407
TLDC2	20	36876121..36894235	0,15	0,11	0,733826537
SAMHD1	20	complement(36890229..36951893)	0,95	1,41	-0,346352023
RBL1	20	complement(36996349..37095997)	1,71	3,07	-0,619126184
MROH8	20	complement(37101226..37179588)	0,13	0,12	0,373447863
RPN2	20	37178410..37241619	116,58	#####	0,347976372
GHRH	20	complement(37251086..37261819)	-	-	#N/D
MANBAL	20	37289649..37317260	26,15	26,07	0,227365352
SRC	20	37344685..37406050	13,36	15,86	-0,02386673
BLCAP	20	complement(37492472..37527931)	24,76	10,81	1,418452086
NNAT	20	37521206..37523690	2,49	0,83	1,810285499
CTNNBL1	20	37694030..37872129	17,04	17,87	0,155183598
VSTM2L	20	37903111..37945350	0,33	0,97	-1,345551907
TTI1	20	complement(37983007..38033461)	13,14	14,03	0,128297378
RPRD1B	20	38033725..38127780	7,63	7,31	0,284236689
TGM2	20	complement(38127385..38166578)	0,62	9,03	-3,630776015
KIAA1755	20	complement(38210503..38260772)	0,03	0,06	-0,694397966
BPI	20	38304150..38337505	0,01	-	3,068695355

LBP	20	38346482..38377013	0,01	0,04	-1,54862452
RALGAPB	20	38472816..38578859	12,01	13,02	0,106631875
ADIG	20	38581195..38588463	-	0,04	-4,359055975
ARHGAP40	20	38601809..38651035	0,01	0,01	-0,025382928
SLC32A1	20	38724486..38729372	-	-	#N/D
ACTR5	20	38748460..38772520	2,78	2,54	0,355928024
PPP1R16B	20	38805697..38923024	0,01	0,02	-1,462209385
FAM83D	20	38926312..38953106	31,95	32,57	0,194983161
DHX35	20	38962299..39039723	12,11	10,02	0,495882773
MAFB	20	complement(40685848..40689236)	2,41	0,94	1,588042944
TOP1	20	41028822..41124487	15,43	16,29	0,144376985
PLCG1	20	41136960..41196801	26,17	17,94	0,768224441
ZHX3	20	complement(41178448..41317731)	12,31	8,86	0,697804466
LPIN3	20	41340821..41360582	7,54	4,97	0,825104112
EMILIN3	20	complement(41359962..41366818)	0,19	0,06	1,770514209
CHD6	20	complement(41402083..41618384)	8,19	7,46	0,357336979
PTPRT	20	complement(42072752..43189970)	-	0,00	-2,89252005
SRSF6	20	43457893..43466046	19,89	15,60	0,573768327
gene:ENSG000	20	43458054..43541881	0,02	-	5,88622595
L3MBTL1	20	43489442..43550954	0,50	0,03	4,382695912
gene:ENSG000	20	43540171..43569498	-	-	0,188027316
SGK2	20	43558968..43588237	0,03	0,02	0,415033091
IFT52	20	43590937..43647299	11,93	12,27	0,182997192
MYBL2	20	43667019..43716495	48,97	61,73	-0,111012771
GTSF1L	20	complement(43726164..43727002)	0,03	0,05	-0,579815015
TOX2	20	43914852..44069616	3,33	14,86	-1,935212559
JPH2	20	complement(44106590..44187188)	3,55	2,44	0,765823574
OSER1	20	complement(44195939..44210771)	19,29	18,40	0,291117206
GDAP1L1	20	44247099..44280947	0,07	0,11	-0,321745402
FITM2	20	complement(44302840..44311202)	6,65	4,13	0,90941527
R3HDML	20	44337043..44351238	-	-	#N/D
HNF4A	20	44355699..44432845	0,00	0,01	-0,429837071
TTPAL	20	44475874..44494603	3,34	4,68	-0,264942676
SERINC3	20	complement(44496221..44522085)	63,71	44,52	0,740162342
PKIG	20	44531785..44624247	27,40	16,17	0,984372511
ADA	20	complement(44584896..44652252)	10,09	10,96	0,103680611
CCN5	20	44714844..44728509	0,14	0,41	-1,368811311
KCNK15	20	44745865..44752313	0,00	0,01	-0,01185201
RIMS4	20	complement(44751808..44810546)	0,04	0,11	-1,235426299
YWHAB	20	44885702..44908532	63,97	61,72	0,274833145
PABPC1L	20	44910060..44959035	3,82	9,48	-1,086342212
TOMM34	20	complement(44942130..44960397)	70,11	85,24	-0,058874276
STK4	20	44966494..45080021	7,96	8,00	0,217168499
KCNS1	20	complement(45091214..45101127)	0,28	0,15	1,176787171
WFDC5	20	complement(45109452..45115174)	-	-	0,188027316
WFDC12	20	complement(45123425..45124465)	0,08	-	5,480421266
PI3	20	45174902..45176544	0,18	8,22	-5,297753976
SEMG1	20	45207033..45209768	-	-	#N/D

SEMG2	20	45221373..45224458	-	-	#N/D
SLPI	20	complement(45252239..45254564)	28,75	17,14	0,969036499
MATN4	20	complement(45293445..45308529)	0,03	0,04	-0,036228841
RBPJL	20	45306840..45317824	0,01	0,01	-0,025382928
SDC4	20	complement(45325288..45348424)	128,73	89,98	0,739789964
SYS1	20	45361937..45376798	7,83	5,97	0,615987166
SYS1-DBNDD2	20	45363200..45410610	0,93	0,87	0,323207414
TP53TG5	20	complement(45372557..45407889)	1,03	0,49	1,296341826
DBNDD2	20	45406057..45410610	0,68	3,68	-2,206572962
PIGT	20	45416084..45456934	13,71	12,43	0,36454421
WFDC2	20	45469753..45481532	0,04	0,02	1,232079601
SPINT3	20	complement(45512461..45515622)	-	-	#N/D
WFDC6	20	complement(45534196..45539482)	-	-	#N/D
EPPIN-WFDC6	20	complement(45534196..45547662)	-	-	0,188027316
EPPIN	20	complement(45540626..45547752)	-	-	0,188027316
WFDC8	20	complement(45551153..45579326)	-	-	0,188027316
WFDC9	20	complement(45607939..45631284)	-	-	#N/D
WFDC10A	20	45629739..45631196	-	-	#N/D
WFDC11	20	complement(45648563..45670270)	-	-	#N/D
WFDC10B	20	complement(45684651..45705019)	-	0,02	-2,89252005
WFDC13	20	45702038..45708817	0,01	-	3,068695355
SPINT4	20	45722347..45725830	-	-	0,188027316
WFDC3	20	complement(45747944..45791932)	0,39	0,18	1,330592333
DNTTIP1	20	45791954..45811427	22,11	16,63	0,633947097
UBE2C	20	45812576..45816957	133,15	#####	0,229930055
TNNC2	20	complement(45823214..45833745)	0,04	0,03	0,524121557
SNX21	20	45833799..45843276	9,85	8,91	0,367743487
ACOT8	20	complement(45841721..45857405)	12,07	7,40	0,928836663
ZSWIM3	20	45857614..45879122	2,04	2,16	0,140069133
ZSWIM1	20	45881227..45885266	7,07	6,91	0,256228941
SPATA25	20	complement(45886491..45887622)	0,84	1,12	-0,182018073
NEURL2	20	complement(45888625..45891208)	0,30	0,71	-1,015741867
CTSA	20	45890144..45898949	19,96	13,58	0,779448404
PLTP	20	complement(45898621..45912155)	18,52	17,49	0,305293896
PCIF1	20	45934683..45948023	24,55	22,07	0,376738153
ZNF335	20	complement(45948660..45972203)	11,36	8,11	0,709648737
MMP9	20	46008908..46016561	0,06	0,02	2,134539329
SLC12A5	20	46021690..46060150	0,22	0,20	0,394373192
NCOA5	20	complement(46060991..46089962)	2,21	4,98	-0,947150182
CD40	20	46118271..46129863	0,01	-	3,068695355
CDH22	20	complement(46173739..46308498)	0,00	-	3,068695355
SLC35C2	20	complement(46345980..46364458)	12,53	10,40	0,492456692
ELMO2	20	complement(46366050..46432985)	7,60	6,56	0,435929171
ZNF334	20	complement(46499630..46513559)	-	-	0,188027316
OCSTAMP	20	complement(46540946..46550654)	-	-	#N/D
SLC13A3	20	complement(46557823..46684467)	0,01	0,01	-0,03031053
TP53RK	20	complement(46684365..46689444)	4,54	4,18	0,341164377
SLC2A10	20	46709649..46736347	23,27	11,65	1,220757912

EYA2	20	46894624..47188844	1,01	1,13	0,065231498
ZMYND8	20	complement(47209214..47356889)	8,29	15,38	-0,667873557
NCOA3	20	47501887..47656877	11,21	7,66	0,772805988
SULF2	20	complement(47656197..47786616)	5,30	3,56	0,796450218
PREX1	20	complement(48624252..48827999)	14,69	23,74	-0,469772388
ARFGEF2	20	48921711..49036693	15,22	9,99	0,830651918
CSE1L	20	49046246..49096960	55,03	72,58	-0,176320474
STAU1	20	complement(49113339..49188367)	42,44	40,77	0,280977058
DDX27	20	49219347..49244077	11,62	13,70	-0,015118616
ZNFX1	20	complement(49237946..49278426)	20,78	19,34	0,326894926
KCNB1	20	complement(49293394..49484297)	2,56	1,24	1,263688898
PTGIS	20	complement(49503874..49568137)	0,01	0,02	-1,326441896
B4GALT5	20	complement(49632945..49713878)	16,30	25,50	-0,422535829
SLC9A8	20	49812713..49892242	2,67	4,72	-0,600315241
SPATA2	20	complement(49903391..49915529)	9,83	6,55	0,808336939
RNF114	20	49936336..49953885	58,17	42,54	0,674546838
SNAI1	20	49982980..49988886	22,37	10,40	1,327798102
UBE2V1	20	complement(50081124..50115959)	32,07	30,08	0,315367936
PEDS1-UBE2V1	20	complement(50081124..50153637)	9,54	6,68	0,737019519
PEDS1	20	complement(50118254..50153734)	10,21	8,04	0,569060334
CEBPB	20	50190830..50192668	44,09	75,62	-0,555386421
PTPN1	20	50510321..50585241	30,51	28,54	0,319362277
RIPOR3	20	complement(50586108..50691542)	6,76	11,62	-0,559337412
PARD6B	20	50731580..50756795	1,89	3,75	-0,765392085
BCAS4	20	50794894..50877177	6,97	12,22	-0,586631178
ADNP	20	complement(50888916..50931437)	15,53	12,74	0,508681404
DPM1	20	complement(50934867..50959140)	6,03	7,11	-0,01491304
MOCS3	20	50958818..50963929	1,57	2,36	-0,368147213
KCNG1	20	complement(51003656..51023107)	7,56	29,60	-1,746075022
gene:ENSG000	20	51098138..51098845	-	-	#N/D
NFATC2	20	complement(51386957..51562831)	0,13	0,61	-2,019452102
ATP9A	20	complement(51596514..51768390)	23,11	14,42	0,903256265
SALL4	20	complement(51782331..51802521)	0,72	0,06	3,813850873
ZFP64	20	complement(52051663..52204308)	4,10	5,99	-0,322336076
TSHZ2	20	52972358..53495330	-	0,02	-6,630750384
ZNF217	20	complement(53567071..53609907)	35,52	22,41	0,887373524
BCAS1	20	complement(53936777..54070594)	0,00	0,01	-1,877914477
CYP24A1	20	complement(54153446..54173986)	1,86	2,10	0,046467363
PFDN4	20	54208087..54228052	0,95	2,50	-1,167722408
DOK5	20	54475593..54651169	0,29	-	8,562959663
CBLN4	20	complement(55997357..56005519)	-	-	#N/D
MC3R	20	56248732..56249815	-	-	0,188027316
FAM210B	20	56358974..56368663	7,09	3,59	1,205931387
AURKA	20	complement(56369389..56392337)	57,72	57,94	0,217685031
CSTF1	20	56392371..56406362	11,71	9,62	0,506899091
CASS4	20	56412112..56460387	0,01	0,03	-1,573534033
RTF2	20	56468585..56519449	52,17	39,47	0,625524131
GCNT7	20	complement(56491492..56525925)	0,29	0,17	1,008480854

FAM209A	20	56517187..56526152	0,08	0,10	-0,034419864
gene:ENSG000	20	56522252..56523499	0,19	0,09	1,237558708
FAM209B	20	56533246..56536520	0,36	0,21	1,015592606
TFAP2C	20	56629306..56639283	12,01	14,53	-0,051189884
gene:ENSG000	20	57161822..57163775	0,02	0,01	0,921097799
BMP7	20	complement(57168753..57266641)	7,89	4,86	0,919996738
SPO11	20	57329803..57343994	-	-	#N/D
RAE1	20	57351223..57379211	14,42	16,18	0,05683964
RBM38	20	57391396..57409333	6,92	9,76	-0,272851633
HMGB1P1	20	complement(57487959..57495366)	0,05	0,06	-0,037246625
CTCFL	20	complement(57495966..57525652)	0,00	-	3,068695355
PCK1	20	57561080..57568121	0,05	0,01	2,38125567
ZBP1	20	complement(57603846..57620576)	-	-	0,188027316
PMEPA1	20	complement(57648392..57711536)	11,29	6,31	1,061956509
CIMIP1	20	58150902..58161150	-	-	0,188027316
ANKRD60	20	complement(58216126..58228653)	0,00	0,02	-2,190455314
RAB22A	20	58309715..58367507	7,00	4,47	0,869167505
VAPB	20	58389229..58451101	6,04	6,31	0,160949109
APCDD1L	20	complement(58459101..58515399)	0,06	0,09	-0,502447039
STX16	20	58651272..58679526	37,09	31,73	0,448230838
STX16-NPEPL1	20	58651434..58715410	0,24	0,43	-0,637146972
NPEPL1	20	58689131..58719238	14,57	20,18	-0,246744366
GNAS	20	58839718..58911192	109,71	79,43	0,688940524
NELFCD	20	58981208..58995133	37,65	45,12	-0,038008187
CTSZ	20	complement(58985686..59008238)	13,32	14,95	0,05665892
TUBB1	20	59019429..59026654	0,28	0,31	0,080319862
ATP5F1E	20	complement(59025475..59032345)	9,28	8,68	0,320316657
PRELID3B	20	complement(59033145..59042809)	18,28	24,92	-0,224021017
ZNF831	20	59123381..59259113	-	-	#N/D
EDN3	20	59300443..59325992	-	-	#N/D
PHACTR3	20	59577509..59847711	0,04	-	6,872264984
SYCP2	20	complement(59863564..59933655)	0,15	0,04	2,043691548
FAM217B	20	59933764..59948680	1,24	1,31	0,145139744
PPP1R3D	20	complement(59936663..59940305)	2,98	2,57	0,435077089
CDH26	20	59958423..60034011	0,01	0,01	0,533811434
CDH4	20	61252261..61940617	0,23	0,29	-0,137691739
TAF4	20	complement(61953469..62065881)	3,37	4,16	-0,079003998
LSM14B	20	62122461..62135374	19,98	19,70	0,243610396
PSMA7	20	complement(62136733..62143440)	112,27	#####	0,24471518
SS18L1	20	62143769..62182514	6,78	4,86	0,702098519
MTG2	20	62183028..62203568	10,36	10,18	0,247512882
HRH3	20	complement(62214960..62220278)	0,01	0,01	-0,025382928
OSBPL2	20	62231922..62296213	13,32	7,10	1,129810922
ADRM1	20	62302093..62308862	43,41	60,09	-0,246026971
LAMA5	20	complement(62307955..62367312)	26,19	29,70	0,041505818
RPS21	20	62387103..62388520	311,92	#####	0,390062152
CABLES2	20	complement(62388634..62407285)	5,06	4,83	0,291021199
RBBP8NL	20	complement(62410237..62427539)	0,08	0,01	3,198733396

GATA5	20	complement(62463497..62475995)	0,65	1,19	-0,65251544
SLCO4A1	20	62642503..62685785	32,46	28,65	0,40305898
NTSR1	20	62708836..62762771	0,75	1,01	-0,210617952
MRGBP	20	62796473..62801729	21,11	20,47	0,267249723
OGFR	20	62804835..62814000	21,76	18,49	0,457676687
COL9A3	20	62816244..62841159	0,21	0,55	-1,204671607
TCFL5	20	complement(62841005..62861822)	6,27	6,33	0,209409823
DIDO1	20	complement(62877738..62937952)	15,96	9,85	0,920094755
GID8	20	62938147..62948475	13,60	11,08	0,51941575
SLC17A9	20	62952707..62969585	0,02	-	5,697545031
BHLHE23	20	complement(63005927..63007035)	-	-	#N/D
YTHDF1	20	complement(63195429..63216139)	34,15	23,96	0,734422345
BIRC7	20	63235883..63240495	1,43	0,64	1,377421325
NKAIN4	20	complement(63240784..63272694)	0,10	0,06	1,035876212
ARFGAP1	20	63272785..63289790	93,62	41,98	1,380043596
COL20A1	20	63293186..63334851	0,02	0,02	0,14012467
CHRNA4	20	complement(63343223..63378401)	0,04	0,00	4,31100363
KCNQ2	20	complement(63400208..63472677)	0,00	0,00	-0,03031053
EEF1A2	20	complement(63488013..63499239)	141,27	#####	-0,339236828
PPDPF	20	63520765..63522206	56,63	58,99	0,16426177
PTK6	20	complement(63528001..63537376)	0,49	0,80	-0,481960157
SRMS	20	complement(63538489..63547749)	0,16	0,14	0,381776192
FNDC11	20	63547891..63556695	0,36	0,42	0,007429927
HELZ2	20	complement(63558086..63574239)	13,37	6,97	1,162795533
GMEB2	20	complement(63587602..63627101)	10,35	7,19	0,74936748
STMN3	20	complement(63639705..63657682)	26,17	26,23	0,219954644
RTEL1	20	63657810..63696253	8,81	8,37	0,297022931
RTEL1-TNFRSF6	20	63659300..63698684	0,67	0,68	0,198377868
TNFRSF6B	20	63696652..63698684	1,76	2,04	0,006566787
ARFRP1	20	complement(63698642..63708025)	10,43	12,63	-0,052761016
ZGPAT	20	63707465..63736142	8,55	9,19	0,118104594
gene:ENSG000	20	63708864..63739103	6,89	4,84	0,732333833
LIME1	20	63736283..63739103	2,66	2,94	0,077385601
gene:ENSG000	20	63738270..63740398	-	-	#N/D
SLC2A4RG	20	63739776..63744050	42,27	42,32	0,221465234
ZBTB46	20	complement(63743668..63832038)	2,02	2,04	0,212730593
C20orf181	20	complement(63843436..63843915)	-	0,03	-2,89252005
ABHD16B	20	63861498..63862988	2,94	2,49	0,462978177
TPD52L2	20	63865228..63891545	68,89	61,08	0,396522028
DNAJC5	20	63895126..63936031	20,46	16,87	0,501870736
UCKL1	20	complement(63939829..63956416)	11,77	14,92	-0,118792545
ZNF512B	20	complement(63956704..63969930)	12,49	13,86	0,07235993
SAMD10	20	complement(63974116..63980008)	0,31	0,51	-0,465415148
PRPF6	20	63981132..64033100	41,95	29,54	0,728774823
C20orf204	20	64034344..64039962	0,47	0,45	0,306808767
SOX18	20	complement(64047582..64049639)	0,57	1,02	-0,624849504
TCEA2	20	64049836..64072347	16,49	13,27	0,536920993
RGS19	20	complement(64073181..64079988)	10,75	10,43	0,266517402

OPRL1	20	64080082..64100643	1,76	0,92	1,159410824
LKAAEAR1	20	complement(64083376..64084398)	0,11	0,10	0,319286298
MYT1	20	64102394..64242253	0,03	0,04	-0,587156213
NPBWR2	20	complement(64103802..64107565)	-	0,01	-3,804623889
PCMTD2	20	64255695..64304820	2,16	2,53	-0,003643048
gene:ENSG000	21	5011799..5017145	0,26	0,61	-0,972288434
gene:ENSG000	21	5022531..5036771	0,35	0,96	-1,244931234
gene:ENSG000	21	5073458..5087867	0,38	0,40	0,158643948
gene:ENSG000	21	complement(5116341..5128369)	4,22	1,88	1,387426797
gene:ENSG000	21	complement(5130871..5154658)	0,78	0,36	1,324994734
gene:ENSG000	21	complement(5155499..5163695)	0,74	0,26	1,763132321
gene:ENSG000	21	complement(5703182..5705637)	0,00	0,06	-3,307263034
gene:ENSG000	21	complement(5972924..5973383)	0,05	-	3,967268276
gene:ENSG000	21	6008604..6008810	0,63	0,31	1,202745498
gene:ENSG000	21	6060340..6076305	0,15	0,29	-0,736311047
gene:ENSG000	21	complement(6084381..6091407)	0,61	0,78	-0,125246362
gene:ENSG000	21	6111131..6123778	0,37	0,53	-0,320439533
gene:ENSG000	21	complement(6444869..6467513)	1,48	1,42	0,283474508
gene:ENSG000	21	complement(6484623..6499248)	4,65	1,64	1,72526953
gene:ENSG000	21	6499203..6564489	0,04	0,05	-0,024464877
gene:ENSG000	21	6897291..6899280	0,08	0,05	0,857064368
gene:ENSG000	21	complement(7135334..7137789)	0,02	0,03	-0,345401666
gene:ENSG000	21	7744951..7758630	0,36	0,24	0,832257945
gene:ENSG000	21	complement(7769588..7770548)	0,24	0,13	1,021047459
gene:ENSG000	21	complement(7788703..7793954)	0,01	0,01	-0,034419864
gene:ENSG000	21	complement(7816677..7829632)	0,00	0,01	-1,009277154
gene:ENSG000	21	complement(8101251..8103706)	0,00	0,03	-2,190455314
gene:ENSG000	21	8235354..8235832	1,33	11,06	-2,830833081
gene:ENSG000	21	8237649..8237979	-	0,04	-2,89252005
gene:ENSG000	21	8238859..8239092	0,46	24,01	-5,458461489
gene:ENSG000	21	8240736..8241047	-	2,03	-8,329674778
gene:ENSG000	21	8417757..8418235	1,19	10,22	-2,871446139
gene:ENSG000	21	8420675..8421003	0,03	1,46	-5,045026341
gene:ENSG000	21	8421883..8422116	0,55	22,63	-5,113584213
gene:ENSG000	21	8423760..8424071	0,03	1,54	-5,045026341
gene:ENSG000	21	8462320..8462798	1,40	11,11	-2,766500481
gene:ENSG000	21	8465254..8465586	0,52	11,78	-4,270597713
gene:ENSG000	21	8466466..8466699	0,74	24,40	-4,811858907
gene:ENSG000	21	8468320..8468631	0,03	1,41	-4,923495304
gene:ENSG000	21	complement(10028361..10029855)	0,02	0,01	1,436225396
TPTE	21	10521553..10606140	-	0,00	-2,89252005
IGHV1OR21-1	21	complement(10649400..10649835)	-	-	#N/D
POTED	21	13609777..13645823	0,00	-	3,068695355
gene:ENSG000	21	complement(13679300..13681138)	0,30	0,08	2,062918685
LIPI	21	complement(14108813..14210955)	-	-	0,188027316
RBM11	21	14216130..14228372	0,11	-	7,1913846
HSPA13	21	complement(14371115..14383484)	10,28	9,94	0,270911999
SAMSN1	21	complement(14485228..14658821)	-	0,01	-4,359055975

NRIP1	21	complement(14961235..15065936)	2,11	9,14	-1,894275986
gene:ENSG000	21	15050189..15052379	0,14	0,63	-1,918380459
gene:ENSG000	21	15346652..15346738	-	-	#N/D
USP25	21	15729982..15880064	2,67	3,55	-0,186986441
gene:ENSG000	21	16525919..16527019	-	0,07	-5,327865481
CXADR	21	17513043..17593579	0,06	0,08	-0,16144003
BTG3	21	complement(17593653..17612945)	11,93	8,84	0,654544608
gene:ENSG000	21	17705408..17707455	-	-	#N/D
C21orf91	21	complement(17788974..17819386)	0,36	0,50	-0,233224374
CHODL	21	17901263..18267373	0,05	-	6,780090526
TMPRSS15	21	complement(18269116..18485879)	-	0,00	-2,89252005
gene:ENSG000	21	18917213..18917429	-	-	#N/D
NCAM2	21	20998409..21543329	0,07	0,02	2,117205505
gene:ENSG000	21	25371827..25372043	-	-	#N/D
MRPL39	21	complement(25585656..25607517)	8,45	9,83	0,005939853
JAM2	21	25639258..25717562	0,20	0,28	-0,246685965
ATP5PF	21	complement(25716503..25735673)	10,15	9,70	0,287776295
GABPA	21	25734570..25772460	2,12	1,99	0,312011114
APP	21	complement(25880535..26171128)	64,87	72,23	0,067901762
CYYR1	21	complement(26466209..26573286)	0,22	0,46	-0,822107491
ADAMTS1	21	complement(26835755..26845409)	7,21	3,28	1,357132651
ADAMTS5	21	complement(26917922..26967088)	0,08	0,05	0,917515581
N6AMT1	21	complement(28872191..28885371)	1,18	1,56	-0,187889332
LTN1	21	complement(28928144..28992956)	4,60	3,38	0,66559095
RWDD2B	21	complement(29004384..29019360)	2,67	3,19	-0,035118894
USP16	21	29024629..29054488	3,15	3,22	0,195534305
CCT8	21	complement(29055805..29073797)	48,81	44,53	0,35538563
MAP3K7CL	21	29077221..29175889	0,14	0,18	-0,13298786
BACH1	21	29194071..29630751	8,49	6,71	0,562435592
GRIK1	21	complement(29536933..29940033)	0,02	0,02	-0,03814481
CLDN17	21	complement(30165565..30166805)	-	-	#N/D
CLDN8	21	complement(30214006..30216097)	-	-	#N/D
KRTAP24-1	21	complement(30281309..30282950)	-	-	#N/D
KRTAP25-1	21	complement(30289145..30289514)	-	-	#N/D
KRTAP26-1	21	complement(30319124..30320315)	-	-	#N/D
KRTAP27-1	21	complement(30337013..30337694)	-	-	#N/D
KRTAP23-1	21	complement(30348399..30348606)	-	-	#N/D
KRTAP13-2	21	complement(30371390..30372271)	-	-	#N/D
KRTAP13-1	21	30396031..30396822	-	-	#N/D
KRTAP13-3	21	complement(30425380..30425926)	-	-	#N/D
KRTAP13-4	21	30430220..30431026	-	-	#N/D
KRTAP15-1	21	30440275..30440869	-	-	#N/D
KRTAP19-1	21	complement(30479706..30480367)	-	-	#N/D
KRTAP19-2	21	complement(30487043..30487436)	-	-	#N/D
KRTAP19-3	21	complement(30491462..30492013)	-	-	#N/D
KRTAP19-4	21	complement(30496824..30497165)	-	-	#N/D
KRTAP19-5	21	complement(30501657..30502141)	-	-	#N/D
KRTAP19-6	21	complement(30541535..30541864)	-	-	#N/D

KRTAP19-7	21	complement(30560875..30561314)	-	-	#N/D
KRTAP22-2	21	complement(30590105..30590397)	-	-	#N/D
KRTAP6-3	21	30592440..30593075	-	-	#N/D
KRTAP6-2	21	complement(30598590..30598902)	-	-	#N/D
KRTAP22-1	21	30601087..30601382	-	-	#N/D
KRTAP6-1	21	complement(30613431..30613930)	-	-	#N/D
KRTAP20-1	21	30616425..30616699	-	-	#N/D
KRTAP20-4	21	30620627..30620850	-	-	#N/D
KRTAP20-2	21	30635206..30635619	-	-	0,188027316
KRTAP20-3	21	30642864..30643136	-	-	#N/D
KRTAP21-3	21	complement(30718525..30718777)	-	-	0,188027316
KRTAP21-2	21	complement(30746794..30747259)	-	-	#N/D
KRTAP21-1	21	complement(30755015..30755428)	-	-	#N/D
KRTAP8-1	21	complement(30812697..30813274)	-	-	#N/D
KRTAP7-1	21	complement(30829039..30829759)	-	-	#N/D
KRTAP11-1	21	complement(30880644..30881580)	-	-	#N/D
KRTAP19-8	21	complement(31038159..31038476)	-	-	#N/D
TIAM1	21	complement(31118416..31559977)	2,64	1,44	1,097857095
SOD1	21	31659666..31668931	100,33	93,28	0,328088934
SCAF4	21	complement(31671000..31732118)	10,49	9,56	0,356886321
HUNK	21	31873020..32044633	0,27	0,36	-0,18502577
MIS18A	21	complement(32268228..32279049)	8,37	6,77	0,529673786
MRAP	21	32291813..32314784	0,04	0,01	2,144183439
URB1	21	complement(32311018..32393012)	11,20	7,19	0,863142312
CFAP298-TCP1	21	complement(32402511..32612865)	0,30	0,25	0,530620596
EVA1C	21	32412006..32515397	1,06	2,59	-1,065185665
TCP10L	21	complement(32497967..32587373)	0,02	-	6,053060169
CFAP298	21	complement(32592079..32612603)	2,18	2,12	0,263878715
SYNJ1	21	complement(32628759..32728040)	2,42	1,08	1,383481553
PAXBP1	21	complement(32733899..32771792)	1,43	2,07	-0,309164713
gene:ENSG000	21	complement(32790673..32791504)	-	-	#N/D
C21orf62	21	complement(32790673..32813743)	0,02	0,02	-0,253352605
OLIG2	21	33025935..33029196	3,03	0,80	2,142545655
OLIG1	21	33070141..33072413	-	0,02	-4,359055975
IFNAR2	21	33205282..33265675	1,97	2,72	-0,24308076
IFNAR2-IL10RB	21	33229892..33297160	0,20	0,18	0,32138223
IL10RB	21	33266367..33311420	3,31	3,58	0,109551023
IFNAR1	21	33324387..33359864	1,94	2,43	-0,100794337
IFNGR2	21	33403413..33479348	25,37	18,24	0,699048042
TMEM50B	21	complement(33432485..33479974)	4,39	1,53	1,747670921
DNAJC28	21	complement(33488055..33491716)	0,06	0,07	-0,037899139
GART	21	complement(33503931..33543491)	16,06	23,00	-0,295007372
SON	21	33543038..33577481	34,35	26,09	0,619890754
DONSON	21	complement(33559542..33588706)	5,43	3,65	0,795731873
gene:ENSG000	21	complement(33584687..33931607)	1,24	0,78	0,889799888
CRYZL1	21	complement(33589341..33643926)	1,94	1,72	0,398653306
ITSN1	21	33642400..33899861	1,15	1,41	-0,077528187
ATP5PO	21	complement(33903453..33915814)	71,67	58,81	0,508264583

MRPS6	21	34073224..34143034	58,44	17,93	1,927899875
SLC5A3	21	34073578..34106260	22,17	4,57	2,502550799
KCNE2	21	34364006..34371381	0,49	0,17	1,727689981
SMIM11	21	34375480..34416961	1,39	1,02	0,67207079
C21orf140	21	complement(34400112..34401072)	0,25	0,15	0,951907113
SMIM34	21	complement(34418715..34423951)	-	-	0,188027316
KCNE1	21	complement(34446688..34512214)	0,01	-	4,913749004
gene:ENSG000	21	complement(34511122..34614913)	0,18	0,12	0,800439495
RCAN1	21	complement(34513142..34615113)	1,90	2,15	0,041982474
CLIC6	21	34668994..34718227	-	-	0,188027316
RUNX1	21	complement(34787801..36004667)	3,54	3,39	0,284888307
SETD4	21	complement(36034541..36079389)	1,98	1,89	0,294583551
CBR1	21	36069941..36073166	11,57	10,53	0,358678656
CBR3	21	36135079..36146562	3,18	4,77	-0,36452727
DOP1B	21	36156782..36294274	1,01	1,59	-0,433559698
MORC3	21	36320189..36386148	1,75	2,23	-0,123288853
CHAF1B	21	36385392..36419015	4,10	4,56	0,069377894
CLDN14	21	complement(36460621..36576569)	0,02	0,07	-1,389560774
gene:ENSG000	21	36485867..36487760	0,06	0,16	-1,092937864
SIM2	21	36699115..36749917	3,74	3,06	0,510398803
HLCS	21	complement(36748626..36990236)	1,99	2,17	0,095941155
RIPPLY3	21	37006150..37019662	-	0,01	-2,89252005
PIGP	21	complement(37062846..37073170)	1,18	0,89	0,641647067
TTC3	21	37073226..37203112	10,90	7,66	0,732462471
VPS26C	21	complement(37223420..37267919)	11,69	8,30	0,716917438
DYRK1A	21	37365573..37526358	3,03	1,98	0,836425619
KCNJ6	21	complement(37607373..38121345)	0,00	0,00	-0,923955849
KCNJ15	21	38155549..38307357	0,68	0,17	2,218221041
ERG	21	complement(38380027..38661780)	0,53	0,52	0,251034444
ETS2	21	38805183..38824955	15,48	12,39	0,544676448
gene:ENSG000	21	complement(39023000..39023835)	-	0,02	-2,89252005
PSMG1	21	complement(39174769..39183488)	5,23	6,78	-0,150751228
BRWD1	21	complement(39184176..39321559)	2,63	1,91	0,689118407
HMGN1	21	complement(39342315..39349647)	25,22	16,79	0,810109856
GET1	21	39377698..39428528	2,60	1,47	1,046354412
GET1-SH3BGR	21	39380332..39515506	0,41	0,52	-0,144636264
LCA5L	21	complement(39405728..39445805)	0,08	0,10	-0,199777109
SH3BGR	21	39445855..39515506	0,05	0,35	-2,491858398
B3GALT5	21	39556442..39673137	0,39	0,40	0,183922086
IGSF5	21	39745183..39802081	0,10	0,07	0,827824525
PCP4	21	39867438..39929397	-	-	#N/D
DSCAM	21	complement(40010999..40847158)	0,00	-	4,516772761
BACE2	21	41167801..41282530	7,40	8,01	0,107931206
FAM3B	21	41304212..41357727	0,01	-	3,068695355
MX2	21	41361999..41409393	0,02	0,04	-1,022609537
MX1	21	41420020..41470071	1,32	0,66	1,228156835
TMPRSS2	21	complement(41464300..41531116)	0,01	-	4,913749003
RIPK4	21	complement(41739369..41767089)	0,67	0,25	1,662267222

PRDM15	21	complement(41798225..41879482)	2,83	1,80	0,872667747
C2CD2	21	complement(41885112..41954018)	17,19	8,64	1,215556409
ZBTB21	21	complement(41986831..42010387)	9,10	5,42	0,971871612
UMODL1	21	42062959..42143453	0,05	0,05	0,221971569
ABCG1	21	42199689..42297244	0,70	1,20	-0,558575596
TFF3	21	complement(42311667..42315409)	-	-	0,188027316
TFF2	21	complement(42346357..42350997)	-	-	0,188027316
TFF1	21	complement(42362282..42366535)	-	-	0,188027316
TMPRSS3	21	complement(42371887..42396091)	0,02	2,14	-6,677511721
UBASH3A	21	42403447..42447684	0,01	0,09	-3,524780994
RSPH1	21	complement(42472486..42496246)	0,02	0,02	-0,025382928
SLC37A1	21	42496008..42581440	1,83	1,53	0,485113875
PDE9A	21	42653621..42775509	0,77	0,02	5,243881806
WDR4	21	complement(42843094..42879568)	11,32	8,89	0,572170909
NDUFV3	21	42879644..42913304	5,66	3,25	1,025311072
ERVH48-1	21	complement(42916803..42925630)	0,50	0,31	0,891913789
PKNOX1	21	42974510..43033931	3,81	2,62	0,764891339
CBS	21	complement(43053191..43076943)	12,43	12,97	0,162368433
U2AF1	21	complement(43092956..43107570)	14,21	8,96	0,88823226
CRYAA	21	43169008..43172805	-	-	0,188027316
SIK1	21	complement(43414483..43427131)	1,53	2,35	-0,395790104
HSF2BP	21	complement(43529186..43659488)	0,84	0,91	0,112274194
H2BC12L	21	43565182..43565648	-	0,14	-5,071123353
RRP1B	21	43659560..43696079	14,73	13,55	0,343084123
PDXK	21	43719094..43762307	19,34	11,30	0,999101804
CSTB	21	complement(43772511..43776330)	6,33	5,26	0,48843867
RRP1	21	43789513..43805293	5,55	4,91	0,399732901
AGPAT3	21	43865223..43987592	14,09	10,99	0,582092461
gene:ENSG000	21	43939919..43959673	0,12	0,20	-0,509043067
TRAPPC10	21	44012309..44106552	6,99	4,37	0,899755714
PWP2	21	44107373..44131181	11,61	11,95	0,180884485
GATD3	21	44133610..44210114	3,91	3,55	0,361224738
ICOSLG	21	complement(44215382..44241446)	0,99	2,45	-1,086359454
DNMT3L	21	complement(44246339..44262216)	-	0,01	-2,89252005
AIRE	21	44285838..44298648	-	0,03	-5,545746556
PFKL	21	44300051..44327376	48,90	28,42	1,006130003
CFAP410	21	complement(44328944..44339402)	5,09	3,44	0,786870208
TRPM2	21	44350163..44443081	0,10	0,09	0,472865408
LRRC3	21	44455510..44462196	3,58	0,35	3,585222701
TSPEAR	21	complement(44497893..44711572)	0,05	0,05	0,206887575
KRTAP10-1	21	complement(44538981..44540195)	-	-	#N/D
KRTAP10-2	21	complement(44550357..44551505)	-	-	#N/D
KRTAP10-3	21	complement(44557790..44558795)	-	-	#N/D
KRTAP10-4	21	44573729..44575371	-	-	#N/D
KRTAP10-5	21	complement(44579455..44580604)	-	-	0,188027316
KRTAP10-6	21	complement(44591268..44592505)	-	-	#N/D
KRTAP10-7	21	44600597..44602174	-	-	#N/D
KRTAP10-8	21	44612079..44612954	-	-	#N/D

KRTAP10-9	21	44627093..44628378	-	-	#N/D
KRTAP10-10	21	44637356..44638455	-	-	#N/D
KRTAP10-11	21	44646414..44647650	-	-	#N/D
KRTAP12-4	21	complement(44654213..44654659)	-	-	0,188027316
KRTAP12-3	21	44657932..44658341	-	-	#N/D
KRTAP12-2	21	complement(44666189..44666927)	-	-	#N/D
KRTAP12-1	21	complement(44681576..44682163)	-	-	#N/D
KRTAP10-12	21	44697172..44698044	-	-	#N/D
UBE2G2	21	complement(44768580..44801826)	16,14	10,33	0,867144713
SUMO3	21	complement(44805617..44818779)	28,16	14,04	1,22689068
PTTG1IP	21	complement(44849585..44873903)	91,46	49,58	1,106339332
ITGB2	21	complement(44885953..44931989)	0,34	0,72	-0,862662313
SLX9	21	44940025..44976973	8,88	9,88	0,069902415
ADARB1	21	45073853..45226560	1,77	3,51	-0,762911661
POFUT2	21	complement(45263928..45287898)	39,87	12,82	1,860117956
COL18A1	21	45405165..45513720	27,82	14,33	1,180285882
SLC19A1	21	complement(45493572..45573365)	6,54	5,00	0,612362272
PCBP3	21	45643694..45942454	10,91	8,04	0,66275141
COL6A1	21	45981770..46005050	185,52	#####	1,007395608
COL6A2	21	46098112..46132848	224,02	#####	1,173154819
FTCD	21	complement(46136160..46155579)	0,37	0,07	2,564100328
SPATC1L	21	complement(46161148..46184476)	8,62	3,76	1,420900817
LSS	21	complement(46188141..46228824)	19,07	6,69	1,733559516
MCM3AP	21	complement(46235133..46286297)	18,45	11,67	0,883696812
YBEY	21	46286342..46297751	3,08	2,58	0,4769081
C21orf58	21	complement(46300181..46323875)	11,25	5,12	1,35747995
PCNT	21	46324124..46445769	10,99	5,17	1,310341981
gene:ENSG000	21	46326288..46326491	0,11	-	3,967268276
DIP2A	21	46458891..46570015	8,45	4,45	1,14976534
S100B	21	complement(46598604..46605208)	-	-	0,188027316
PRMT2	21	46635595..46665124	11,17	8,18	0,672231069
OR11H1	22	15528192..15529139	-	-	#N/D
POTEH	22	15690026..15721631	0,01	-	3,967268276
CCT8L2	22	complement(16590751..16592810)	-	0,02	-4,359055975
XKR3	22	complement(16783412..16825411)	-	0,01	-2,89252005
GAB4	22	complement(16961936..17008222)	-	-	0,188027316
IL17RA	22	17084954..17115693	2,77	2,80	0,20773661
TMEM121B	22	complement(17116297..17121367)	0,06	0,05	0,37304645
HDHD5	22	complement(17137511..17165287)	22,00	19,16	0,423040818
ADA2	22	complement(17178790..17258235)	0,02	0,02	0,238747802
CECR9	22	17329034..17329232	-	-	#N/D
CECR2	22	17359949..17558151	0,01	0,45	-5,709831398
SLC25A18	22	17563450..17590995	0,18	0,14	0,523111673
ATP6V1E1	22	complement(17592136..17628749)	48,31	35,93	0,650321973
BCL2L13	22	17628855..17730855	8,46	7,93	0,317072618
BID	22	complement(17734138..17774770)	10,85	13,42	-0,083288858
MICAL3	22	complement(17787649..18024561)	6,41	4,77	0,648008501
PEX26	22	18077923..18105396	2,92	2,28	0,579735838

gene:ENSG000	22	18077989..18131720	0,44	0,30	0,808516731
TUBA8	22	18110100..18146683	0,01	0,10	-2,671637508
USP18	22	18149843..18177397	1,25	1,68	-0,204973109
GGTLC3	22	complement(18516344..18518161)	-	0,05	-4,758582517
TMEM191B	22	18527802..18530573	0,28	0,05	2,775224447
RIMBP3	22	complement(18605815..18611919)	0,43	0,18	1,507494262
FAM246B	22	18633984..18634682	-	-	#N/D
DGCR6	22	18906028..18914238	0,64	0,34	1,164836292
gene:ENSG000	22	18906238..18947732	0,41	0,22	1,148408482
PRODH	22	complement(18912777..18936553)	0,03	0,01	2,616997665
FAM246C	22	19029524..19031242	0,07	0,03	1,391449417
DGCR2	22	complement(19036282..19122454)	12,29	8,43	0,767295971
ESS2	22	complement(19130279..19144684)	3,31	1,96	0,980330262
TSSK2	22	19131308..19132622	-	-	#N/D
GSC2	22	complement(19146993..19150292)	0,00	-	3,068695355
SLC25A1	22	complement(19175581..19178739)	55,16	23,77	1,437487185
CLTCL1	22	complement(19179473..19291719)	0,64	1,68	-1,17083236
HIRA	22	complement(19330698..19447450)	5,13	6,70	-0,16203397
C22orf39	22	complement(19351368..19447711)	3,28	2,57	0,574247434
MRPL40	22	19432545..19436075	13,67	15,56	0,036124011
UFD1	22	complement(19449911..19479202)	14,74	10,57	0,703389379
CDC45	22	19479457..19520612	7,89	8,88	0,053524127
CLDN5	22	complement(19523024..19527545)	0,19	0,23	-0,08753198
SEPTIN5	22	19714503..19724224	12,41	7,29	0,990315404
gene:ENSG000	22	19717220..19724772	3,21	2,46	0,609215045
GP1BB	22	19723539..19724771	-	-	0,188027316
TBX1	22	19756703..19783593	3,37	3,54	0,155223194
GNB1L	22	complement(19783223..19854939)	2,72	1,17	1,437338211
RTL10	22	complement(19846138..19854896)	13,29	5,30	1,549034254
TXNRD2	22	complement(19875517..19941820)	3,43	6,17	-0,623045547
COMT	22	19941371..19969975	11,72	12,02	0,186736249
ARVCF	22	complement(19969896..20016823)	0,72	0,62	0,439091167
TANGO2	22	20017014..20067164	5,06	4,88	0,274125664
DGCR8	22	20080221..20111877	8,10	6,02	0,651288181
TRMT2A	22	complement(20111875..20117392)	18,72	7,62	1,519516312
RANBP1	22	20115938..20127355	24,73	25,36	0,186795718
ZDHHC8	22	20129456..20148007	10,69	8,61	0,536606533
CCDC188	22	complement(20148416..20151055)	0,57	0,16	2,066792244
RTN4R	22	complement(20241415..20283246)	1,67	2,11	-0,115571055
DGCR6L	22	complement(20314238..20320080)	10,00	7,93	0,557135244
ZNF74	22	20394115..20408461	1,46	2,48	-0,544929479
SCARF2	22	complement(20424584..20437826)	19,02	6,66	1,73638484
gene:ENSG000	22	complement(20429241..20446620)	0,33	0,12	1,675491804
KLHL22	22	complement(20441519..20495844)	7,21	4,99	0,752750778
MED15	22	20495913..20587632	20,05	13,48	0,795914975
PI4KA	22	complement(20707691..20859417)	15,20	11,10	0,676930322
SERPIND1	22	20774113..20787720	0,21	0,08	1,536271194
SNAP29	22	20859007..20891214	4,79	3,46	0,691384893

CRKL	22	20917407..20953747	27,27	16,88	0,915145622
AIFM3	22	20965108..20981360	0,02	0,08	-1,594046895
LZTR1	22	20982269..20999032	10,40	8,61	0,496880312
THAP7	22	complement(20999104..21002196)	6,03	4,55	0,62936664
TUBA3FP	22	complement(21008193..21014292)	0,01	0,04	-2,447197442
gene:ENSG000	22	complement(21008247..21032572)	-	-	0,188027316
P2RX6	22	21009808..21028013	0,14	0,13	0,310048643
SLC7A4	22	complement(21028718..21032840)	-	0,01	-2,89252005
LRRC74B	22	21045946..21064168	-	-	0,188027316
FAM246A	22	complement(21360601..21361299)	-	0,02	-2,89252005
RIMBP3B	22	21383751..21389478	0,20	0,12	0,957580276
HIC2	22	21417371..21451463	4,19	1,50	1,703461433
TMEM191C	22	21466423..21471269	0,23	0,07	1,917530063
RIMBP3C	22	complement(21545666..21551461)	0,02	0,01	1,255976028
UBE2L3	22	21549447..21624034	25,68	18,16	0,722845372
YDJC	22	complement(21628089..21630064)	10,97	13,07	-0,029751441
CCDC116	22	21632716..21637329	0,15	0,06	1,641580129
SDF2L1	22	21642302..21644299	30,19	5,27	2,73999938
PPIL2	22	21666000..21700015	6,46	4,20	0,843979544
YPEL1	22	complement(21697536..21735794)	0,48	0,22	1,340683417
MAPK1	22	complement(21759657..21867680)	22,53	19,55	0,427628486
PPM1F	22	complement(21919425..21952848)	18,74	11,12	0,976448463
TOP3B	22	complement(21957025..21982813)	6,45	4,21	0,837814068
IGLV4-69	22	22030934..22031472	-	-	#N/D
IGLV8-61	22	22098700..22099212	-	-	#N/D
IGLV4-60	22	22162199..22162681	-	-	#N/D
IGLV6-57	22	22195799..22196276	-	-	#N/D
IGLV11-55	22	22201663..22202161	-	-	#N/D
IGLV10-54	22	22214794..22215270	-	-	#N/D
VPREB1	22	22244780..22245515	-	-	#N/D
IGLV5-52	22	22318727..22319226	2,13	2,02	0,302881715
IGLV1-51	22	22322472..22322969	0,19	0,47	-1,026620426
IGLV1-50	22	22327300..22327814	0,04	0,03	0,886720911
IGLV9-49	22	22343187..22343732	-	-	#N/D
IGLV5-48	22	22352940..22353433	-	-	#N/D
IGLV1-47	22	22357739..22358260	-	-	#N/D
IGLV7-46	22	22369614..22370087	-	-	#N/D
IGLV5-45	22	22375986..22376505	-	-	#N/D
IGLV1-44	22	22380766..22381347	-	-	#N/D
IGLV7-43	22	22395018..22395489	-	-	#N/D
IGLV1-40	22	22409766..22410282	-	-	#N/D
IGLV5-37	22	22427540..22428035	-	-	0,188027316
IGLV1-36	22	22431958..22432465	-	-	#N/D
ZNF280B	22	complement(22484421..22508742)	1,84	2,10	0,027700625
ZNF280A	22	complement(22513736..22520270)	0,55	0,05	3,538123115
PRAME	22	complement(22547701..22559361)	33,07	36,98	0,061995487
IGLV2-33	22	22588155..22588688	-	-	#N/D
IGLV3-32	22	22594528..22595056	-	-	0,188027316

GGTLC2	22	22644614..22647898	-	0,01	-2,89252005
IGLV3-27	22	22668288..22668806	-	-	#N/D
IGLV3-25	22	22686726..22687271	-	-	#N/D
IGLV2-23	22	22697789..22698407	-	-	#N/D
IGLV3-22	22	22704265..22704822	-	-	#N/D
IGLV3-21	22	22711689..22713203	-	-	#N/D
IGLV3-19	22	22720623..22721145	-	0,05	-3,804623889
IGLV2-18	22	22734607..22735089	-	-	#N/D
IGLV3-16	22	22747383..22747921	-	-	#N/D
IGLV2-14	22	22758700..22759218	-	-	#N/D
IGLV3-12	22	22771824..22772582	-	-	#N/D
IGLV2-11	22	22792491..22793007	-	-	#N/D
IGLV3-10	22	22811747..22812281	-	-	#N/D
IGLV3-9	22	22819010..22819756	-	-	#N/D
IGLV2-8	22	22822658..22823289	-	-	#N/D
IGLV4-3	22	22871507..22872035	-	-	#N/D
IGLV3-1	22	22880706..22881396	-	-	#N/D
IGLL5	22	22887780..22896111	-	-	#N/D
IGLJ1	22	22893692..22893818	-	-	#N/D
IGLC1	22	22895375..22895834	-	-	#N/D
IGLJ2	22	22899481..22899655	-	-	#N/D
IGLC2	22	22900976..22901437	-	-	#N/D
IGLJ3	22	22904850..22905025	-	-	#N/D
IGLC3	22	22906342..22906803	-	-	0,188027316
IGLJ4	22	22910574..22910606	-	-	#N/D
IGLJ5	22	22914237..22914308	-	-	#N/D
IGLJ6	22	22918132..22918201	-	-	#N/D
IGLJ7	22	22921390..22921435	-	-	#N/D
IGLC7	22	22922594..22923034	-	-	#N/D
RSPH14	22	complement(23059415..23145021)	0,06	0,03	1,202745498
GNAZ	22	23070519..23125032	5,76	5,04	0,413312936
RAB36	22	23145366..23165663	0,03	0,02	0,632074084
BCR	22	23179704..23318037	12,69	7,91	0,906126336
IGLL1	22	complement(23573125..23580302)	-	-	0,188027316
DRICH1	22	complement(23608452..23632321)	0,02	0,01	1,436225396
RGL4	22	23688136..23699176	0,96	0,56	0,999430131
ZNF70	22	complement(23738682..23751112)	3,65	1,90	1,164103928
VPREB3	22	complement(23752743..23754425)	0,04	0,07	-0,579815015
C22orf15	22	23762990..23765863	0,04	-	4,913749003
CHCHD10	22	complement(23765834..23767972)	-	-	0,188027316
MMP11	22	23768226..23784316	0,65	1,71	-1,175911074
SMARCB1	22	23786931..23838009	8,92	9,38	0,150540501
DERL3	22	complement(23834503..23839128)	0,51	-	10,16338221
SLC2A11	22	23856703..23886312	0,29	0,52	-0,635910878
gene:ENSG000	22	23862188..23895223	-	0,35	-7,112521263
MIF	22	23894383..23895227	358,57	#####	0,756356825
GSTT2B	22	complement(23957414..23961195)	7,25	4,85	0,803084387
DDTL	22	23966888..23972556	2,07	1,70	0,506420713

DDT	22	complement(23970542..23980525)	19,67	13,40	0,777471684
GSTT2	22	23980058..23983915	0,49	0,56	0,041983265
GSTT4	22	complement(23998401..24005453)	-	-	0,188027316
CABIN1	22	24011192..24178628	13,39	9,19	0,766865128
SUSD2	22	24181487..24189106	0,19	0,44	-0,972759529
GGT5	22	complement(24219654..24245108)	0,06	1,44	-4,463349103
SPECC1L	22	24270817..24417739	7,83	7,33	0,318633694
SPECC1L-ADOI	22	24270898..24442356	0,36	0,04	3,259148054
ADORA2A	22	24417879..24442357	0,88	1,69	-0,721743253
UPB1	22	24494107..24528390	0,08	0,06	0,734548152
GUCD1	22	complement(24540423..24555935)	23,92	14,51	0,944020112
SNRPD3	22	24555958..24582052	10,99	7,72	0,732418218
gene:ENSG000	22	24556007..24628987	5,83	4,78	0,508464169
LRRC75B	22	complement(24585620..24593208)	1,37	1,98	-0,303665598
GGT1	22	24594811..24629005	0,46	0,63	-0,208600391
PIWIL3	22	complement(24719034..24774720)	-	-	0,188027316
SGSM1	22	24806169..24927578	0,01	0,00	2,144183439
LHFPL7	22	complement(24935098..24946695)	-	-	#N/D
KIAA1671	22	24952716..25197448	4,68	7,10	-0,379145592
CRYBB3	22	25199858..25207359	0,01	0,11	-2,665078517
CRYBB2	22	25212564..25231870	0,27	-	7,56729892
GRK3	22	25564675..25729294	0,23	0,81	-1,625014113
MYO18B	22	25742144..26031045	0,04	0,05	-0,08687842
SEZ6L	22	26169462..26383597	-	-	0,188027316
ASPHD2	22	26429260..26445015	1,01	2,09	-0,831422796
HPS4	22	complement(26443107..26483931)	3,72	4,12	0,076384794
SRRD	22	26483877..26494658	3,47	3,24	0,321192069
TFIP11	22	complement(26491225..26512505)	7,15	4,81	0,793415156
TPST2	22	complement(26521996..26596717)	1,46	1,34	0,346230387
CRYBB1	22	complement(26599278..26618027)	-	-	0,188027316
CRYBA4	22	26621963..26630669	-	0,03	-3,804623889
MN1	22	complement(27748277..27801756)	2,31	2,54	0,085909785
PITPNB	22	complement(27851669..27920134)	10,91	9,57	0,412570711
TTC28	22	complement(27978014..28679840)	2,87	3,59	-0,10024872
CHEK2	22	complement(28687743..28742422)	4,84	4,59	0,298865382
HSCB	22	28742039..28757515	3,84	3,49	0,358420992
CCDC117	22	28772674..28789301	5,01	4,55	0,362944279
XBP1	22	complement(28794555..28800597)	63,99	39,59	0,91576957
ZNRF3	22	28883572..29057488	1,51	1,81	-0,040848881
C22orf31	22	complement(29058672..29061831)	0,01	0,08	-2,447197442
KREMEN1	22	29073035..29168333	7,90	3,50	1,397319296
EMID1	22	29205896..29259597	0,47	0,21	1,37609024
RHBDD3	22	complement(29259872..29268209)	4,80	5,19	0,110342414
EWSR1	22	29268009..29300525	42,58	46,83	0,085990787
GAS2L1	22	29306618..29312787	5,10	5,30	0,168527455
RASL10A	22	complement(29312933..29319679)	0,11	0,12	0,129542238
AP1B1	22	complement(29327680..29388583)	28,68	21,03	0,670340938
RFPL1	22	29438583..29442455	0,01	-	3,967268276

NEFH	22	29480218..29491390	0,03	0,03	0,221705268
THOC5	22	complement(29505879..29555216)	6,29	6,55	0,165291267
NIPSNAP1	22	complement(29554808..29581327)	46,43	37,33	0,53787192
NF2	22	29603553..29698598	19,91	16,37	0,505375678
CABP7	22	29720003..29731833	0,14	0,07	1,176191113
ZMAT5	22	complement(29730956..29767011)	9,95	8,43	0,462804237
UQCR10	22	29767369..29770413	35,48	24,94	0,731561801
ASCC2	22	complement(29788609..29838304)	16,00	12,84	0,54036215
MTMR3	22	29883169..30030868	6,56	5,36	0,514278808
HORMAD2	22	30080464..30177075	-	-	0,188027316
LIF	22	complement(30240453..30246759)	48,42	39,26	0,525644002
OSM	22	complement(30262829..30266851)	0,10	0,05	1,027668938
gene:ENSG000	22	complement(30275215..30276951)	0,66	0,18	2,109702112
CASTOR1	22	complement(30285117..30289622)	7,60	3,77	1,233954918
gene:ENSG000	22	complement(30285238..30299482)	1,41	0,16	3,323562426
TBC1D10A	22	complement(30291990..30326947)	16,45	8,87	1,113464621
SF3A1	22	complement(30331988..30356919)	32,32	24,67	0,613111979
CCDC157	22	30356635..30378673	0,53	0,40	0,609453916
RNF215	22	complement(30368811..30421771)	3,40	2,36	0,750807662
SEC14L2	22	30396941..30425303	4,77	1,92	1,535079535
gene:ENSG000	22	30409255..30428990	0,33	0,04	3,095898225
MTFP1	22	30425623..30429054	10,00	10,29	0,181705618
SEC14L3	22	complement(30447959..30472017)	-	0,00	-2,89252005
SEC14L4	22	complement(30488902..30505711)	0,39	0,68	-0,588975201
SEC14L6	22	complement(30522799..30546741)	0,01	-	4,516772761
GAL3ST1	22	complement(30554635..30574665)	0,10	0,03	1,990169618
PES1	22	complement(30576625..30607083)	43,03	25,66	0,969265591
TCN2	22	30607003..30627271	0,03	0,03	0,221270597
SLC35E4	22	30635781..30669016	2,06	1,28	0,909411174
DUSP18	22	complement(30652051..30667887)	1,36	1,14	0,477285406
OSBP2	22	30693782..30907824	1,48	2,06	-0,25619926
MORC2	22	complement(30925130..30968774)	9,76	8,22	0,470111659
TUG1	22	30969245..30979395	22,06	11,82	1,123613967
SMTN	22	31064105..31104757	29,04	14,20	1,255706006
SELENOM	22	complement(31104772..31120069)	30,64	16,69	1,099449009
INPP5J	22	31122731..31134697	0,04	0,24	-2,20944432
PLA2G3	22	complement(31134807..31140508)	-	-	0,188027316
RNF185	22	31160182..31207019	9,92	5,05	1,197451059
LIMK2	22	31212239..31280080	1,31	1,28	0,249926179
PIK3IP1	22	complement(31281594..31292534)	0,40	0,11	2,129788017
PATZ1	22	complement(31325804..31346346)	3,32	5,01	-0,372678111
DRG1	22	31399604..31528740	19,55	12,47	0,871905927
EIF4ENIF1	22	complement(31436977..31496108)	7,42	4,48	0,950627202
SFI1	22	31488688..31618588	3,66	3,35	0,34951832
PISD	22	complement(31618491..31662221)	12,08	11,29	0,319451594
PRR14L	22	complement(31676256..31750140)	6,08	5,09	0,48021956
DEPDC5	22	31753867..31908033	1,29	1,20	0,332554495
gene:ENSG000	22	31754899..31956243	-	-	#N/D

YWHAH	22	31944522..31957603	36,04	26,05	0,69126359
SLC5A1	22	32043261..32113029	-	-	0,188027316
C22orf42	22	complement(32149006..32159322)	-	-	0,188027316
RFPL2	22	complement(32190435..32205073)	0,02	-	4,913749004
SLC5A4	22	complement(32218464..32255347)	0,06	-	6,338059249
RFPL3	22	32354885..32361161	0,06	0,02	2,060408965
RTCB	22	complement(32387582..32412248)	31,29	21,64	0,75529289
BPIFC	22	complement(32413845..32464484)	-	0,01	-2,89252005
FBXO7	22	32474676..32498829	22,29	21,10	0,302801939
SYN3	22	complement(32507820..33058381)	0,15	0,07	1,297140247
TIMP3	22	32801705..32863041	0,62	11,53	-3,994973139
LARGE1	22	complement(33162226..33922841)	0,61	0,24	1,549518305
ISX	22	35066136..35087387	-	-	0,188027316
HMGXB4	22	35257452..35295807	9,27	7,89	0,456023699
TOM1	22	35299275..35347992	15,93	7,45	1,31973013
HMOX1	22	35380361..35394214	1,58	5,50	-1,574668219
MCM5	22	35400134..35425431	19,86	19,92	0,219155706
RASD2	22	35540831..35553999	0,45	0,77	-0,559850664
MB	22	complement(35606764..35637951)	0,01	0,06	-3,171495545
APOL6	22	35648446..35668404	2,06	3,69	-0,617262779
APOL5	22	35717872..35729483	0,01	-	3,068695355
RBFOX2	22	complement(35738736..36028824)	20,92	11,89	1,03776724
gene:ENSG000	22	complement(36137430..36139350)	0,46	0,07	2,978290249
APOL3	22	complement(36140329..36166177)	0,99	0,33	1,810393909
APOL4	22	complement(36189124..36204840)	0,03	0,01	2,209934656
APOL2	22	complement(36226209..36239954)	23,18	7,76	1,801110614
APOL1	22	36253071..36267530	0,19	0,36	-0,672999711
MYH9	22	complement(36281280..36388010)	118,56	#####	0,423237656
TXN2	22	complement(36467046..36481640)	26,47	18,10	0,770993087
FOXRED2	22	complement(36487190..36507221)	7,81	7,12	0,357978601
EIF3D	22	complement(36510855..36529184)	70,93	49,18	0,75126124
CACNG2	22	complement(36560857..36703752)	-	-	#N/D
IFT27	22	complement(36758202..36776256)	5,04	3,71	0,667119565
PVALB	22	complement(36800684..36819479)	-	-	#N/D
NCF4	22	36860988..36878017	-	-	0,188027316
CSF2RB	22	36913628..36940439	0,00	0,01	-0,923955849
CIMIP4	22	complement(36991120..37007851)	0,01	-	3,068695355
TST	22	complement(37010859..37020183)	2,35	2,75	-0,00148232
MPST	22	37019635..37029821	12,35	7,97	0,854768947
KCTD17	22	37051739..37063390	5,27	4,25	0,532948891
TMPRSS6	22	complement(37065436..37109713)	0,10	0,07	0,782123294
IL2RB	22	complement(37118666..37175118)	0,03	0,00	3,69954316
C1QTNF6	22	complement(37180166..37199385)	6,21	4,36	0,733128139
SSTR3	22	complement(37204237..37212477)	-	0,00	-2,89252005
RAC2	22	complement(37225270..37259594)	0,13	0,18	-0,202234428
CYTH4	22	37282027..37315341	0,11	0,04	1,573693064
ELFN2	22	complement(37367960..37427479)	1,21	1,65	-0,223286151
MFNG	22	complement(37469063..37486393)	0,35	0,24	0,760732555

CARD10	22	complement(37490362..37519542)	4,51	4,51	0,223020636
CDC42EP1	22	37560480..37569405	40,45	40,22	0,231233009
LGALS2	22	complement(37570248..37582616)	0,14	0,08	1,07890858
GGA1	22	37608474..37633564	10,71	9,63	0,376855414
SH3BP1	22	37634654..37656117	0,28	0,50	-0,630042773
gene:ENSG000	22	37639693..37666932	-	-	0,188027316
PDXP	22	37658723..37666932	21,76	10,21	1,314604175
LGALS1	22	37675636..37679802	#######	#####	0,982335002
NOL12	22	37681673..37693476	11,62	6,87	0,980815003
gene:ENSG000	22	37686414..37726503	-	-	0,188027316
TRIOBP	22	37697048..37776556	10,63	6,77	0,87485384
H1-0	22	37805229..37807432	2,04	0,92	1,364249412
GCAT	22	37807905..37817176	5,76	5,97	0,171259436
GALR3	22	37823382..37825485	0,12	0,02	2,583287272
ANKRD54	22	complement(37830855..37849327)	9,06	5,24	1,011758925
EIF3L	22	37848868..37889407	49,18	28,98	0,98595245
MICALL1	22	37905657..37942822	13,50	15,83	-0,006208919
gene:ENSG000	22	37943050..37944898	5,89	6,18	0,152992384
C22orf23	22	complement(37943050..37953669)	0,55	0,67	-0,065230444
POLR2F	22	37952607..38041915	1,98	1,37	0,756260144
SOX10	22	complement(37970686..37987422)	0,23	0,09	1,525325262
PICK1	22	38056311..38075701	1,08	1,51	-0,263814782
SLC16A8	22	complement(38078134..38084184)	0,04	0,05	-0,036800208
BAIAP2L2	22	complement(38084652..38111015)	2,35	1,50	0,871452745
PLA2G6	22	complement(38111495..38214778)	2,04	2,22	0,10383539
MAFF	22	38200767..38216507	22,48	14,95	0,811408267
TMEM184B	22	complement(38219291..38273010)	56,61	29,18	1,179152329
CSNK1E	22	complement(38290691..38318084)	34,76	23,91	0,762782893
TPTEP2-CSNK1	22	complement(38291918..38398522)	2,19	1,78	0,525991431
KCNJ4	22	complement(38426327..38455199)	0,09	0,44	-2,072191516
KDELR3	22	38468078..38483447	36,34	11,87	1,836620219
DDX17	22	complement(38483438..38507660)	76,91	49,74	0,851692108
DMC1	22	complement(38518948..38570204)	0,16	0,02	3,487291825
FAM227A	22	complement(38578118..38656629)	0,50	0,25	1,206188217
CBY1	22	38656636..38673854	5,03	4,21	0,479537713
TOMM22	22	38681957..38685421	25,23	21,86	0,430158833
JOSD1	22	complement(38685543..38701556)	13,61	16,70	-0,072411391
GTPBP1	22	38705742..38738299	22,89	12,62	1,081923794
SUN2	22	complement(38733290..38794143)	50,78	34,98	0,760727664
DNAL4	22	complement(38778508..38794198)	7,78	5,66	0,682257763
NPTXR	22	complement(38818452..38844028)	3,75	3,42	0,356716066
CBX6	22	complement(38861422..38872249)	17,15	15,01	0,416111729
APOBEC3A	22	38952741..38963184	0,01	0,03	-1,478387936
APOBEC3B	22	38982347..38992804	10,43	10,52	0,210241909
APOBEC3C	22	39014257..39020352	26,05	14,67	1,051896267
gene:ENSG000	22	39014363..39033276	-	-	0,188027316
APOBEC3D	22	39021113..39033277	0,20	0,17	0,45565389
APOBEC3F	22	39040604..39055972	1,48	0,66	1,395535212

APOBEC3G	22	39077067..39087743	-	-	0,188027316
APOBEC3H	22	39097224..39104067	0,09	0,03	1,655502569
CBX7	22	complement(39120167..39152680)	3,76	2,48	0,818964504
PDGFB	22	complement(39223359..39244982)	2,26	3,52	-0,415580165
RPL3	22	complement(39312882..39320389)	502,21	#####	0,308990631
SYNGR1	22	39349925..39385575	4,96	2,45	1,2435903
gene:ENSG000	22	39379610..39380015	1,09	0,38	1,721196444
TAB1	22	39399778..39437060	10,07	7,21	0,70438604
MGAT3	22	39457012..39492194	0,02	0,18	-2,867218167
MIEF1	22	39499432..39518132	14,43	10,90	0,628305866
MIURF	22	39504231..39504443	-	-	#N/D
ATF4	22	39519695..39522683	150,50	#####	0,638009685
RPS19BP1	22	complement(39529093..39532761)	19,50	15,12	0,590090322
CACNA1I	22	39570753..39689735	0,05	0,05	0,30164755
ENTHD1	22	complement(39743044..39893864)	-	-	#N/D
GRAP2	22	39901084..39973721	0,01	0,00	1,436225396
TMA7B	22	39960397..39964905	0,01	-	3,068695355
FAM83F	22	39994954..40043534	0,01	0,00	1,264197729
TNRC6B	22	40044817..40335808	3,66	3,47	0,29887855
ADSL	22	40346461..40390463	5,51	7,96	-0,306630595
gene:ENSG000	22	40346529..40410054	5,51	3,08	1,063620124
SGSM3	22	40370591..40410289	20,88	9,64	1,338009711
MRTFA	22	complement(40410281..40636719)	6,02	4,57	0,622434487
MCHR1	22	40679273..40682812	0,02	0,01	1,833201638
SLC25A17	22	complement(40769630..40819399)	6,17	4,59	0,650145056
ST13	22	complement(40824535..40856639)	29,78	29,33	0,245266773
XPNPEP3	22	40857077..40932815	7,98	5,14	0,85604265
DNAJB7	22	complement(40859549..40862113)	0,37	0,22	1,006639413
RBX1	22	40951347..40973309	25,07	17,12	0,773080482
EP300	22	41092510..41180077	9,35	8,08	0,434472462
L3MBTL2	22	41205282..41231271	6,81	5,61	0,502678409
CHADL	22	complement(41229510..41240931)	0,07	0,05	0,582943981
RANGAP1	22	complement(41244779..41286187)	61,37	47,27	0,59961869
ZC3H7B	22	41301525..41360147	25,90	23,28	0,376459655
TEF	22	41367333..41399326	10,26	3,73	1,684105702
TOB2	22	complement(41433494..41446801)	19,15	10,30	1,117553232
ACO2	22	41447830..41529273	22,11	18,46	0,4834213
PHF5A	22	complement(41459717..41468692)	22,44	15,00	0,803770313
POLR3H	22	complement(41525799..41544606)	7,99	5,11	0,86674353
CSDC2	22	41561010..41577741	0,26	0,12	1,370153711
PMM1	22	complement(41576900..41589871)	19,46	8,80	1,367785553
DESI1	22	complement(41598028..41621043)	13,06	17,91	-0,232333789
XRCC6	22	41621163..41664048	103,07	97,74	0,299671313
SNU13	22	complement(41673933..41690504)	38,62	30,07	0,584100543
MEI1	22	41699503..41799456	0,02	0,02	0,7939095
CCDC134	22	41800679..41832164	8,37	4,61	1,084446942
SREBF2	22	41833105..41907306	39,97	17,73	1,395967085
SHISA8	22	complement(41909543..41915074)	0,09	0,01	3,791717619

TNFRSF13C	22	complement(41922032..41926806)	1,20	0,87	0,689294976
CENPM	22	complement(41938737..41947152)	9,65	7,89	0,512414691
SMIM45	22	41947194..41958939	0,39	0,20	1,218208617
SEPTIN3	22	41969443..41998221	0,09	0,10	0,114031707
WBP2NL	22	41998725..42058456	0,13	0,00	5,63950394
NAGA	22	complement(42058334..42070842)	8,11	5,34	0,826441239
PHETA2	22	42074248..42079438	0,07	0,05	0,632074084
SMDT1	22	42079691..42084284	5,51	4,54	0,502395993
NDUFA6	22	complement(42085526..42090884)	8,40	7,14	0,457788532
CYP2D6	22	complement(42126499..42130865)	0,53	0,56	0,149294061
CYP2D7	22	complement(42140203..42149500)	0,72	0,86	-0,040596617
TCF20	22	complement(42160013..42343616)	13,69	10,34	0,628798896
NFAM1	22	complement(42380407..42432403)	0,02	0,02	-0,187130731
RRP7A	22	complement(42508344..42519796)	10,54	8,72	0,497425502
SERHL2	22	42553617..42574382	1,28	1,07	0,475053092
POLDIP3	22	complement(42583721..42614962)	46,12	23,83	1,175806481
gene:ENSG000	22	complement(42583836..42627688)	0,05	0,02	1,338212201
CYB5R3	22	complement(42615730..42720870)	45,79	36,33	0,556928402
ATP5MGL	22	complement(42639803..42640601)	0,36	0,32	0,390808973
A4GALT	22	complement(42692121..42721298)	4,12	4,11	0,224827833
ARFGAP3	22	complement(42796502..42858106)	31,56	15,58	1,241354317
PACSIN2	22	complement(42835412..43015149)	14,74	12,40	0,472556629
TTLL1	22	complement(43039516..43089419)	2,83	4,67	-0,500674213
BIK	22	43110750..43129712	0,20	0,45	-0,908663233
MCAT	22	complement(43132209..43143398)	2,51	3,08	-0,071567263
TSPO	22	43151547..43163242	77,41	50,02	0,8531678
TTLL12	22	complement(43166622..43187134)	20,44	24,56	-0,041961928
SCUBE1	22	complement(43197280..43343372)	0,03	0,03	0,00728348
MPPED1	22	43411196..43507848	-	-	0,188027316
EFCAB6	22	complement(43528744..43812337)	0,04	0,03	0,800439495
SULT4A1	22	complement(43824509..43862513)	0,00	-	3,068695355
PNPLA5	22	complement(43879678..43892013)	-	-	#N/D
PNPLA3	22	43923792..43964488	6,49	3,59	1,074621683
SAMM50	22	43955442..44010531	17,55	14,75	0,474270975
PARVB	22	43999211..44172939	6,50	7,20	0,076150097
PARVG	22	44172956..44219533	-	0,02	-5,327865481
gene:ENSG000	22	complement(44183031..44187739)	0,00	-	3,967268276
SHISAL1	22	complement(44243665..44312951)	3,44	3,79	0,083036928
gene:ENSG000	22	complement(44486295..44486914)	0,43	0,21	1,270871069
RTL6	22	complement(44492583..44498233)	12,69	13,32	0,152765228
PRR5	22	44668547..44737681	1,69	1,59	0,317683573
PRR5-ARHGAP8	22	44702233..44862706	0,68	0,15	2,431725661
ARHGAP8	22	44752558..44862788	0,99	0,12	3,263568501
PHF21B	22	complement(44881162..45010005)	0,01	-	3,967268276
NUP50	22	45163925..45188017	8,99	7,66	0,455182429
KIAA0930	22	complement(45192244..45240894)	19,68	20,83	0,141189961
UPK3A	22	45284949..45295874	-	-	#N/D
FAM118A	22	45308968..45341955	2,46	2,24	0,355272611

SMC1B	22	complement(45344063..45413619)	0,05	0,02	1,781479726
RIBC2	22	45413693..45432509	0,79	0,64	0,517314746
FBLN1	22	45502238..45601135	28,31	23,33	0,501983801
ATXN10	22	45671798..45845307	23,27	20,10	0,434853676
WNT7B	22	complement(45920366..45977162)	20,12	17,89	0,392636119
gene:ENSG000	22	46006616..46010777	0,63	0,62	0,259083661
PPARA	22	46150521..46243755	5,85	2,60	1,390961112
CDPF1	22	complement(46244011..46250311)	4,69	3,93	0,478897532
PKDREJ	22	complement(46255663..46263343)	0,31	0,36	-0,001047266
TTC38	22	46267961..46294008	3,55	3,29	0,333289972
GTSE1	22	46296870..46330810	19,32	16,46	0,454609318
TRMU	22	46330875..46357340	4,94	4,17	0,465863645
CELSR1	22	complement(46360834..46537620)	5,41	7,29	-0,207718134
GRAMD4	22	46576012..46679790	4,77	3,53	0,655363694
CERK	22	complement(46684410..46738252)	17,03	10,27	0,953142643
TBC1D22A	22	46762617..47175699	4,40	3,40	0,595680148
gene:ENSG000	22	complement(47115838..47117217)	0,40	0,18	1,377908836
gene:ENSG000	22	47259366..47261007	0,01	-	3,967268276
TAFA5	22	48489553..48850912	0,76	-	10,62076298
BRD1	22	complement(49773283..49827873)	8,45	6,24	0,660365772
ZBED4	22	49853844..49890080	7,73	7,48	0,26984012
ALG12	22	complement(49900229..49918438)	10,42	3,97	1,616047285
CRELD2	22	49918167..49927540	16,34	4,62	2,045720759
PIM3	22	49960768..49964072	46,21	30,50	0,822458815
IL17REL	22	complement(49991811..50012765)	0,13	0,02	2,788856269
TTLL8	22	complement(50018575..50058298)	0,00	0,02	-2,190455314
MLC1	22	complement(50059391..50085426)	0,04	0,02	0,842164604
MOV10L1	22	50090006..50161690	0,38	0,45	-0,010515746
PANX2	22	50170731..50180295	8,30	5,20	0,897002224
TRABD	22	50185913..50199598	21,37	16,77	0,572901416
SELENOO	22	50201011..50217616	9,87	6,88	0,742800713
TUBGCP6	22	complement(50217689..50245023)	11,75	6,43	1,093525541
HDAC10	22	complement(50245183..50251405)	10,90	5,41	1,232177848
MAPK12	22	complement(50245450..50261716)	15,43	15,18	0,246683354
MAPK11	22	complement(50263713..50270767)	13,30	10,09	0,621015113
PLXNB2	22	complement(50274979..50307646)	31,58	24,41	0,594701802
DENND6B	22	complement(50309030..50327012)	0,78	0,24	1,926995002
PPP6R2	22	50343304..50445090	9,26	6,95	0,638459511
SBF1	22	complement(50443219..50483923)	26,35	22,29	0,464907548
ADM2	22	50481543..50486440	5,29	5,25	0,233161025
MIOX	22	50486784..50490648	0,05	0,12	-1,111619727
LMF2	22	complement(50502949..50507702)	32,53	13,61	1,479605822
NCAPH2	22	50508224..50524780	13,82	9,28	0,797429578
SCO2	22	complement(50523568..50526461)	0,62	1,12	-0,630678011
TYMP	22	complement(50525752..50530032)	2,30	1,46	0,880374781
CIMAP1B	22	complement(50529710..50532506)	1,53	1,34	0,410112597
KLHDC7B	22	50545899..50551023	0,02	0,02	0,247752455
SYCE3	22	complement(50551112..50562919)	0,02	0,03	-0,01185201

CPT1B	22	complement(50568861..50578465)	3,92	3,61	0,339076593
CHKB-CPT1B	22	complement(50568869..50582965)	2,48	2,40	0,271578502
CHKB	22	complement(50578959..50601455)	8,95	6,14	0,76608575
MAPK8IP2	22	50600793..50613981	0,08	0,06	0,739071628
ARSA	22	complement(50622754..50628173)	5,69	4,12	0,689094669
SHANK3	22	50674408..50733212	3,58	2,28	0,871424139
ACR	22	50738196..50745339	0,19	0,04	2,414444359
RABL2B	22	complement(50767501..50783667)	2,04	1,98	0,262403102
PLCXD1_1	X	276322..303356	3,04	3,26	0,125565582
GTPBP6_1	X	complement(304529..318796)	6,50	10,92	-0,524043319
PPP2R3B_1	X	complement(333933..386955)	3,39	5,62	-0,507710605
SHOX_1	X	624344..659411	-	0,00	-2,89252005
CRLF2_1	X	complement(1190490..1212723)	1,30	4,68	-1,62311972
CSF2RA_1	X	1268800..1310381	0,02	0,04	-0,865575989
IL3RA_1	X	1336616..1382689	0,57	0,48	0,450467606
SLC25A6_1	X	complement(1386152..1392113)	195,48	#####	0,085296812
ASMTL_1	X	complement(1403139..1453762)	34,16	20,01	0,994441286
P2RY8_1	X	complement(1462581..1537185)	0,05	0,02	1,59409163
AKAP17A_1	X	1591604..1602520	30,41	16,06	1,144025874
ASMT_1	X	1615059..1643081	0,05	0,10	-0,895537122
DHRSX_1	X	complement(2219506..2502805)	4,37	6,94	-0,44468734
ZBED1_1	X	complement(2486414..2500976)	8,57	12,26	-0,293318988
CD99_1	X	2691187..2741309	118,58	#####	0,44609147
XG	X	2752040..2816500	-	-	0,188027316
GYG2	X	2828822..2882818	2,75	1,65	0,963822795
ARSD	X	complement(2903972..2929349)	0,98	2,67	-1,219484537
ARSL	X	complement(2934045..2968475)	0,06	0,25	-1,880274666
ARSH	X	3006546..3034111	-	-	0,188027316
ARSF	X	3041471..3112727	0,01	-	3,967268276
MXRA5	X	complement(3308565..3346652)	0,03	0,00	2,905460354
PRKX	X	complement(3604340..3713649)	2,25	3,38	-0,362075611
NLGN4X	X	complement(5840637..6228867)	-	-	0,188027316
VCX3A	X	complement(6533618..6535118)	-	-	#N/D
PUDP	X	complement(6667865..7148158)	4,05	10,28	-1,119349364
STS	X	7147237..7804358	0,39	1,08	-1,254339034
gene:ENSG000	X	7489659..7491199	-	-	#N/D
VCX	X	7842262..7844143	-	-	0,188027316
PNPLA4	X	complement(7898247..7927739)	-	-	0,188027316
VCX2	X	complement(8169944..8171267)	-	-	#N/D
VCX3B	X	8464830..8466510	-	-	0,188027316
ANOS1	X	complement(8528874..8732137)	-	-	0,188027315
FAM9A	X	complement(8790795..8801383)	-	-	0,188027316
FAM9B	X	complement(9024232..9295682)	-	0,00	-2,89252005
TBL1X	X	9463320..9741037	4,98	12,98	-1,159634659
GPR143	X	complement(9725346..9786297)	0,02	0,04	-0,742377908
SHROOM2	X	9786429..9949443	0,11	0,47	-1,90834396
CLDN34	X	9967358..9968352	0,01	-	3,068695355
WWC3	X	10015254..10144474	6,90	14,90	-0,887338667

CLCN4	X	10156975..10237660	0,59	2,55	-1,882687145
MID1	X	complement(10445310..10833654)	2,92	6,26	-0,877818968
gene:ENSG000	X	complement(10566888..10576955)	0,81	0,16	2,55167748
HCCS	X	11111301..11123086	6,07	14,02	-0,983373068
ARHGAP6	X	complement(11117651..11665920)	0,18	0,24	-0,171730508
AMELX	X	11293413..11300761	0,01	0,02	-0,01185201
MSL3	X	11758159..11775772	2,91	4,62	-0,440902079
FRMPD4	X	11822423..12724523	0,00	0,03	-3,399978463
PRPS2	X	12791355..12824222	13,26	24,73	-0,676397383
TLR7	X	12867072..12890361	-	0,00	-2,89252005
TLR8	X	12906620..12923169	-	-	0,188027316
TMSB4X	X	12975110..12977227	244,93	#####	0,332290071
FAM9C	X	complement(13035617..13044682)	-	0,00	-3,804623889
ATXN3L	X	complement(13318647..13320053)	-	-	#N/D
EGFL6	X	13569601..13633575	-	0,01	-2,89252005
TCEANC	X	13653112..13681964	0,18	0,39	-0,87863123
RAB9A	X	13689128..13710504	3,94	6,57	-0,515746935
TRAPPC2	X	complement(13712244..13734635)	3,51	5,09	-0,314887385
OFD1	X	13734743..13777955	0,86	2,11	-1,077305834
GPM6B	X	complement(13770939..13938638)	0,05	0,20	-1,70613675
GEMIN8	X	complement(14006726..14029893)	2,03	3,92	-0,728050495
GLRA2	X	14529298..14731812	0,00	0,03	-2,854330803
FANCB	X	complement(14690388..14873255)	0,70	1,25	-0,623335918
MOSPD2	X	14873421..14922327	2,46	3,96	-0,464001208
ASB9	X	complement(15235288..15270467)	-	0,45	-8,923087847
ASB11	X	complement(15281697..15315640)	-	0,00	-2,89252005
PIGA	X	complement(15319452..15335554)	2,15	2,28	0,139008234
VEGFD	X	complement(15345596..15384413)	0,05	0,03	0,7939095
PIR	X	complement(15384799..15493564)	11,41	20,59	-0,628439772
BMX	X	15464246..15556529	-	-	#N/D
ACE2	X	complement(15494566..15607236)	-	0,00	-2,89252005
gene:ENSG000	X	complement(15561047..15675608)	0,04	0,13	-1,651825528
CLTRN	X	complement(15627318..15675012)	0,02	-	4,516772761
CA5B	X	15688830..15788411	3,67	4,76	-0,152043076
ZRSR2	X	15790156..15830694	0,63	0,79	-0,106225486
AP1S2	X	complement(15825806..15854931)	0,80	2,04	-1,136236444
GRPR	X	16123565..16153518	0,26	0,05	2,654340506
MAGEB17	X	16167481..16171464	0,03	0,01	1,833201638
CTPS2	X	complement(16587999..16712936)	3,69	8,10	-0,908972411
S100G	X	16650158..16654670	-	-	0,188027316
SYAP1	X	16719612..16765340	5,34	8,23	-0,400268124
TXLNG	X	16786432..16844519	3,44	9,39	-1,225371138
RBBP7	X	complement(16839283..16870362)	38,07	52,90	-0,251709168
REPS2	X	16946658..17153272	0,31	0,53	-0,556939259
NHS	X	17375200..17735994	0,61	1,33	-0,910243686
SCML1	X	17737449..17754988	8,43	9,11	0,111253194
RAI2	X	complement(17800049..17861337)	-	0,01	-4,359055975
BEND2	X	complement(18162931..18220886)	-	-	#N/D

SCML2	X	complement(18239313..18354688)	2,23	5,65	-1,120007781
CDKL5	X	18425583..18653629	0,54	0,92	-0,544971493
RS1	X	complement(18639688..18672108)	-	0,00	-2,89252005
PPEF1	X	18675909..18827921	0,28	0,51	-0,650488772
PHKA2	X	complement(18892298..18984114)	7,56	10,65	-0,271860777
ADGRG2	X	complement(18989307..19122637)	0,04	0,13	-1,61577177
PDHA1	X	19343893..19361718	18,68	32,37	-0,570515151
MAP3K15	X	complement(19360056..19515508)	0,04	0,45	-3,208463594
SH3KBP1	X	complement(19533977..19887600)	17,82	42,10	-1,017573943
BCLAF3	X	complement(19912860..19991061)	0,29	0,73	-1,135981152
MAP7D2	X	complement(20006713..20116907)	0,02	0,02	0,181652234
EIF1AX	X	complement(20124525..20141838)	3,61	10,58	-1,326560682
RPS6KA3	X	complement(20149911..20267519)	4,86	12,01	-1,080889863
CNKSR2	X	21372801..21654695	0,14	0,01	3,582258603
KLHL34	X	complement(21654690..21658330)	-	-	0,188027316
SMPX	X	complement(21705978..21758116)	-	-	#N/D
MBTPS2	X	21839617..21885423	5,40	9,99	-0,665774719
YY2	X	21855987..21858740	1,37	2,03	-0,344359508
SMS	X	21940709..21994837	111,83	#####	0,087144206
PHEX	X	22032325..22494713	0,10	0,47	-2,023786604
CBLL2	X	22272913..22274461	-	0,01	-2,89252005
DDX53	X	22999960..23003589	-	0,00	-2,89252005
PTCHD1	X	23334849..23404374	0,00	0,02	-2,453497736
PRDX4	X	23664262..23686397	39,45	50,77	-0,140961984
ACOT9	X	complement(23701055..23766475)	7,42	14,04	-0,697869286
SAT1	X	23783173..23786210	76,49	79,30	0,170990932
gene:ENSG000	X	23783278..23783367	-	-	#N/D
APOO	X	complement(23833353..23907938)	8,63	14,68	-0,542903802
CXorf58	X	23908006..23939509	0,01	0,03	-0,979341556
KLHL15	X	complement(23983716..24027186)	3,75	3,57	0,293794924
EIF2S3	X	24054946..24078810	81,88	#####	-0,454297269
ZFX	X	24149173..24216255	3,18	4,80	-0,371237994
SUPT20HL2	X	complement(24308210..24314069)	-	0,01	-4,359055975
SUPT20HL1	X	24360639..24367839	-	0,00	-2,89252005
PDK3	X	24465244..24550466	2,08	1,45	0,742068465
PCYT1B	X	complement(24558087..24672677)	0,02	0,82	-5,184843359
POLA1	X	24693873..24996986	2,95	7,85	-1,190322001
ARX	X	complement(25003694..25016420)	-	-	0,188027316
MAGEB18	X	26138343..26140736	-	-	#N/D
MAGEB6B	X	26160601..26162410	-	-	#N/D
MAGEB6	X	26192440..26195646	-	0,01	-3,804623889
MAGEB5	X	26216169..26218270	-	-	#N/D
PPP4R3C	X	complement(27460207..27463341)	-	-	0,188027316
DCAF8L2	X	27590344..27749942	-	-	0,188027316
MAGEB10	X	27807990..27823014	-	-	#N/D
DCAF8L1	X	complement(27977992..27981449)	-	-	0,188027316
IL1RAPL1	X	28587446..29956718	0,43	0,01	5,083055962
MAGEB2	X	30215563..30220089	-	-	0,188027316

MAGEB3	X	30230657..30237495	-	-	#N/D
MAGEB4	X	30242000..30244187	-	-	#N/D
MAGEB1	X	30243715..30252040	-	-	#N/D
NR0B1	X	complement(30304206..30309390)	0,10	-	7,117975732
TASL	X	complement(30558809..30577766)	-	-	#N/D
GK	X	30653359..30731462	0,69	1,00	-0,319801321
TAB3	X	complement(30827442..30975084)	0,78	1,19	-0,377508189
FTHL17	X	complement(31071233..31072041)	-	-	#N/D
DMD	X	complement(31097677..33339609)	0,02	0,05	-1,174927118
FAM47A	X	complement(34129752..34132314)	-	-	0,188027316
TMEM47	X	complement(34627075..34657285)	1,50	0,05	4,9951515
FAM47B	X	34942796..34944915	-	-	#N/D
MAGEB16	X	35798342..35803772	-	-	0,188027316
CFAP47	X	35919734..36385317	-	0,00	-2,89252005
H2AL1Q	X	36719829..36720573	-	-	#N/D
FAM47C	X	37008366..37011664	-	-	#N/D
PRRG1	X	37349309..37457292	0,89	0,97	0,089700651
gene:ENSG000	X	37349330..38687674	0,23	0,10	1,457610729
LANCL3	X	37571569..37684463	0,12	0,04	1,700853862
XK	X	37685791..37732130	0,16	0,52	-1,446998698
CYBB	X	37780018..37813461	0,02	0,05	-1,026620426
DYNLT3	X	complement(37836757..37847571)	2,28	1,31	1,019202948
H2AP	X	37990779..37991314	0,06	-	4,516772761
H2AL3	X	37994272..37994904	0,02	-	3,068695355
SYTL5	X	38006553..38128819	1,17	0,05	4,664156134
SRPX	X	complement(38149336..38220924)	8,14	19,77	-1,058279007
RPGR	X	complement(38269163..38327544)	0,28	0,49	-0,58708225
OTC	X	38327598..38422908	-	-	0,188027316
TSPAN7	X	38561542..38688920	0,05	-	6,338059249
MID1IP1	X	38801440..38806537	3,44	4,68	-0,222795735
BCOR	X	complement(40049815..40177329)	5,41	6,59	-0,060615429
ATP6AP2	X	40579372..40606848	8,62	18,78	-0,901300816
MPC1L	X	40623428..40624136	-	-	0,188027316
CXorf38	X	complement(40626921..40647561)	7,71	4,92	0,871073033
MED14	X	complement(40648305..40735858)	5,43	5,56	0,186980787
USP9X	X	41085445..41236579	6,95	8,84	-0,122517771
DDX3X	X	41333348..41364472	6,53	8,93	-0,228234664
NYX	X	41447343..41475710	-	-	0,188027316
CASK	X	complement(41514934..41923554)	1,32	2,03	-0,4002961
GPR34	X	41688973..41697275	0,01	0,01	-0,025382928
GPR82	X	41724181..41730130	0,04	0,04	0,085937779
gene:ENSG000	X	42285130..42285925	0,03	0,07	-0,979341556
PPP1R2C	X	complement(42777366..42778163)	-	-	#N/D
MAOA	X	43654907..43746817	0,03	0,07	-1,137357921
MAOB	X	complement(43766610..43882450)	-	-	0,188027316
NDP	X	complement(43948776..43973395)	0,08	0,02	2,028855185
EFHC2	X	complement(44147872..44343672)	-	-	0,188027316
FUNDC1	X	complement(44523639..44542859)	2,36	3,24	-0,233279551

DUSP21	X	44844021..44844888	-	-	0,188027316
KDM6A	X	44873188..45112779	2,75	2,97	0,109458276
DIPK2B	X	complement(45148373..45200901)	0,00	-	3,068695355
KRBOX4	X	46447292..46497422	1,13	1,21	0,130101431
ZNF674	X	complement(46497725..46545457)	0,39	0,49	-0,087925615
CHST7	X	46573765..46598496	3,02	2,98	0,243364289
SLC9A7	X	complement(46599251..46759118)	9,40	7,49	0,551882579
RP2	X	46837043..46882358	1,81	1,44	0,548637109
JADE3	X	46912276..47061242	1,64	3,10	-0,698357537
RGN	X	47078355..47093314	0,00	-	3,068695355
NDUFB11	X	complement(47142071..47145466)	11,05	13,41	-0,056814157
RBM10	X	47145221..47186813	16,83	17,62	0,156779768
UBA1	X	47190861..47215128	38,39	37,94	0,240225828
CDK16	X	47217860..47229997	19,95	20,39	0,191334834
USP11	X	47232866..47248328	5,76	14,03	-1,061174178
ZNF157	X	47370578..47414498	-	-	#N/D
ZNF41	X	complement(47445178..47483222)	0,63	1,02	-0,476723002
ARAF	X	47561205..47571908	15,97	16,37	0,18773816
SYN1	X	complement(47571901..47619857)	0,07	0,07	0,030621608
TIMP1	X	47582408..47586789	51,00	43,20	0,462366556
CFP	X	complement(47623172..47630305)	0,01	0,01	-0,025382928
ELK1	X	complement(47635521..47650604)	14,57	10,56	0,686772325
UXT	X	complement(47651796..47659180)	20,69	24,00	0,008614911
ZNF81	X	47836902..48002561	0,28	0,36	-0,128478493
ZNF182	X	complement(47974851..48003989)	0,57	1,05	-0,662981565
ZNF630	X	complement(47983356..48071658)	-	-	0,188027316
SPACA5	X	48004336..48009733	-	-	#N/D
SPACA5B	X	48130626..48132620	-	0,02	-2,89252005
SSX5	X	complement(48186220..48196795)	-	-	0,188027316
SSX1	X	48255392..48267444	-	-	0,188027316
SSX3	X	complement(48346427..48356707)	-	-	#N/D
SSX4	X	48383516..48393347	-	-	#N/D
SSX4B	X	complement(48402082..48411960)	-	-	#N/D
SLC38A5	X	complement(48458537..48470260)	0,06	0,06	0,3726069
FTSJ1	X	48476021..48486364	10,24	10,99	0,120685533
PORCN	X	48508959..48520814	2,86	4,44	-0,414144476
EBP	X	48521799..48528716	16,25	13,57	0,483233782
gene:ENSG000	X	48521806..48545469	0,20	0,25	-0,039657841
TBC1D25	X	48539714..48562609	4,28	4,02	0,314110234
RBM3	X	48574449..48581162	5,06	7,32	-0,309016116
gene:ENSG000	X	48580741..48581165	-	-	#N/D
WDR13	X	48590042..48608869	3,96	4,95	-0,101698548
WAS	X	48676596..48691431	0,07	0,01	2,644301309
SUV39H1	X	48695554..48709016	5,86	7,95	-0,218195363
GLOD5	X	48761747..48773648	-	-	#N/D
GATA1	X	48786540..48794311	-	0,01	-2,89252005
HDAC6	X	48801377..48824982	7,75	5,99	0,595025533
ERAS	X	48826513..48830138	0,08	-	6,20258586

PCSK1N	X	complement(48831096..48835610)	-	-	0,188027316
PQBP1	X	48890197..48903402	4,73	5,73	-0,052757528
TIMM17B	X	complement(48893447..48898143)	8,54	9,79	0,02665694
SLC35A2	X	complement(48903180..48911958)	8,94	5,47	0,931464426
PIM2	X	complement(48913182..48919024)	2,56	3,03	-0,020189829
OTUD5	X	complement(48922024..48958386)	11,22	11,24	0,219811785
gene:ENSG000	X	48939992..48940808	0,94	0,82	0,407898783
KCND1	X	complement(48961378..48971844)	0,14	0,10	0,676777903
GRIPAP1	X	complement(48973720..49002265)	1,88	2,09	0,067266488
TFE3	X	complement(49028726..49043410)	20,87	15,45	0,656926461
CCDC120	X	49053572..49069857	0,21	0,12	1,066422375
PRAF2	X	complement(49071161..49074002)	9,68	6,12	0,883669235
gene:ENSG000	X	complement(49071470..49079887)	-	0,06	-5,545746556
WDR45	X	complement(49074433..49101170)	2,28	3,18	-0,261662758
GPKOW	X	complement(49113407..49123735)	9,78	8,29	0,462465384
MAGIX	X	49162564..49168774	0,13	0,19	-0,35601371
gene:ENSG000	X	complement(49165576..49165972)	-	0,03	-2,89252005
PLP2	X	49171898..49175235	58,03	56,10	0,271917714
PRICKLE3	X	complement(49174802..49186528)	2,41	2,91	-0,052856867
SYP	X	complement(49187815..49200218)	0,11	0,19	-0,540888101
CACNA1F	X	complement(49205063..49233371)	0,00	0,00	-0,923955849
CCDC22	X	49235470..49250520	5,15	6,04	-0,006442495
FOXP3	X	complement(49250438..49264800)	0,17	0,13	0,524667622
gene:ENSG000	X	complement(49258343..49270477)	0,05	-	4,516772761
PPP1R3F	X	49269793..49301461	1,97	1,84	0,324495672
GAGE10	X	49303646..49319841	-	-	0,188027316
GAGE12J	X	49322037..49329384	-	-	#N/D
GAGE13	X	49331582..49338949	-	-	#N/D
GAGE2E	X	49341192..49345922	-	-	0,188027316
GAGE12B	X	49529869..49529985	-	-	#N/D
GAGE12C	X	49532177..49539538	-	-	#N/D
GAGE12D	X	49541733..49549094	-	-	#N/D
GAGE12F	X	49551278..49568218	-	-	0,188027316
GAGE12E	X	49551289..49558645	-	-	#N/D
GAGE12G	X	49570400..49577754	-	-	0,188027316
GAGE12H	X	49579949..49587301	-	-	#N/D
GAGE2A	X	49589496..49596824	-	-	0,188027316
GAGE1	X	49599020..49608538	-	-	0,188027316
PAGE1	X	complement(49687447..49695984)	-	-	0,188027316
PAGE4	X	49793653..49834264	-	-	#N/D
USP27X	X	49879484..49882558	0,19	0,35	-0,615316658
CLCN5	X	49922596..50099235	0,36	0,44	-0,062985168
AKAP4	X	complement(50190777..50201007)	-	-	#N/D
CCNB3	X	50202713..50351914	0,03	0,02	0,591315826
DGKK	X	complement(50365409..50470825)	-	-	#N/D
SHROOM4	X	complement(50586796..50814302)	0,03	0,00	5,367297184
BMP15	X	50910735..50916641	-	-	#N/D
NUDT10	X	51332231..51337525	-	-	0,188027316

EZHIP	X	51406948..51408843	-	-	0,188027316
NUDT11	X	complement(51490011..51496592)	0,14	0,02	3,225510647
CENPVL3	X	complement(51617020..51618912)	-	-	#N/D
CENPVL2	X	complement(51681212..51682831)	-	-	#N/D
CENPVL1	X	51710512..51712131	-	-	#N/D
GSPT2	X	51743442..51746232	0,54	1,03	-0,701491885
MAGED1	X	51803007..51902354	91,71	37,50	1,513401533
MAGED4B	X	complement(52061827..52069219)	2,98	1,31	1,408893984
MAGED4	X	52184876..52192268	5,60	1,53	2,093632216
XAGE2	X	52369021..52375680	-	-	0,188027316
XAGE1A	X	52495808..52500812	-	-	0,188027316
XAGE1B	X	complement(52512077..52520803)	-	-	0,188027316
SSX7	X	complement(52644061..52654900)	-	-	#N/D
SSX2	X	complement(52696896..52707178)	-	-	0,188027316
SSX2B	X	52751132..52790305	-	-	0,188027316
SPANXN5	X	complement(52796144..52797427)	-	-	#N/D
XAGE5	X	52811287..52818301	-	-	#N/D
XAGE3	X	complement(52862525..52868083)	-	-	0,188027316
FAM156B	X	52891306..52908560	5,79	3,02	1,16065971
FAM156A	X	complement(52926402..52995472)	2,49	2,99	-0,040858135
GPR173	X	53048789..53080615	2,04	1,43	0,737941822
TSPYL2	X	53082367..53088540	4,31	4,03	0,319482936
KANTR	X	53094142..53170914	0,07	0,08	0,070803356
KDM5C	X	complement(53176283..53225422)	23,28	20,46	0,409019745
IQSEC2	X	complement(53225828..53321350)	2,19	2,01	0,351807678
SMC1A	X	complement(53374149..53422728)	6,85	8,52	-0,092304049
RIBC1	X	53422690..53431120	0,12	0,10	0,478416905
HSD17B10	X	complement(53431258..53434370)	17,44	21,90	-0,105525919
HUWE1	X	complement(53532096..53686752)	22,39	27,88	-0,093545755
PHF8	X	complement(53936676..54048958)	2,33	3,26	-0,263131449
FAM120C	X	complement(54068324..54183281)	0,87	0,88	0,196457856
WNK3	X	complement(54192823..54358642)	0,01	0,01	0,7939095
TSR2	X	54440404..54448032	4,09	4,14	0,205644577
FGD1	X	complement(54445454..54496234)	4,64	6,09	-0,16921329
GNL3L	X	54530183..54621521	4,06	6,12	-0,370079367
ITIH6	X	complement(54748918..54798255)	-	-	0,188027316
MAGED2	X	54807599..54816015	49,75	27,66	1,069801975
TRO	X	54920462..54931431	0,28	0,17	0,948410847
PFKFB1	X	complement(54932961..54998534)	0,00	0,01	-0,923955849
APEX2	X	55000363..55009057	6,55	6,80	0,169350605
ALAS2	X	complement(55009055..55030977)	0,03	0,04	-0,291161992
PAGE2B	X	55075030..55078909	0,08	-	4,913749003
PAGE2	X	55089018..55092842	-	-	0,188027316
VCF2	X	complement(55143102..55161310)	0,53	0,49	0,340614699
PAGE5	X	55220346..55224108	-	-	0,188027316
PAGE3	X	complement(55258415..55264916)	-	-	0,188027316
MAGEH1	X	55452127..55453566	4,49	0,79	2,724279838
USP51	X	complement(55484616..55489848)	0,00	-	3,068695355

FOXR2	X	55623400..55626192	-	-	#N/D
RRAGB	X	55717749..55758774	0,86	0,75	0,425754619
KLF8	X	56232356..56291531	-	-	0,188027316
UBQLN2	X	56563627..56567868	4,40	5,85	-0,188016713
NBDY	X	56729241..56819179	4,23	5,58	-0,176599445
SPIN3	X	complement(56818298..56995827)	0,19	0,01	5,331851588
SPIN2B	X	complement(57118551..57121548)	2,60	2,06	0,562192364
SPIN2A	X	complement(57134530..57137523)	0,39	0,57	-0,298558898
FAAH2	X	57286706..57489193	0,02	0,01	0,524121557
ZXDB	X	57592011..57597477	1,04	1,49	-0,298308742
NLRP2B	X	complement(57677067..57680260)	-	-	0,188027316
ZXDA	X	complement(57905430..57910458)	0,36	0,48	-0,185776561
SPIN4	X	complement(63347228..63351332)	1,32	1,40	0,136260652
ARHGEF9	X	complement(63634967..63809274)	0,58	0,60	0,176804928
gene:ENSG000	X	complement(63754485..63754664)	-	-	#N/D
AMER1	X	complement(64185117..64205708)	1,81	1,86	0,179975312
ASB12	X	complement(64224194..64230607)	-	-	0,188027316
MTMR8	X	complement(64268081..64395452)	-	-	0,188027316
ZC4H2	X	complement(64915802..65034713)	0,04	0,10	-1,177989845
ZC3H12B	X	65034788..65507887	0,04	0,08	-0,594488045
LAS1L	X	complement(65438549..65534810)	4,35	4,39	0,211913644
MSN	X	65588377..65741931	135,46	#####	0,587271021
VSIG4	X	complement(66021738..66040125)	-	-	#N/D
HEPH	X	66162671..66268867	0,06	0,01	3,158420371
EDA2R	X	complement(66595637..66639298)	-	-	0,188027316
AR	X	67544021..67730619	0,88	1,17	-0,18163104
OPHN1	X	complement(67949349..68433913)	0,44	0,73	-0,499186389
YIPF6	X	68498562..68537282	2,72	2,71	0,228141901
STARD8	X	68647666..68725842	1,84	3,54	-0,717186418
EFNB1	X	68829021..68842160	5,29	2,08	1,568102819
PJA1	X	complement(69160851..69165521)	2,98	2,45	0,504132797
NALF2	X	69504326..69532508	-	-	#N/D
EDA	X	69616067..70039472	0,01	0,01	-0,253352605
AWAT2	X	complement(70040542..70049938)	0,00	-	3,068695355
OTUD6A	X	70062457..70064179	0,01	0,01	0,886720911
IGBP1	X	70133447..70166324	8,34	8,83	0,141229201
DGAT2L6	X	70177483..70205704	0,01	-	3,068695355
AWAT1	X	70234655..70240659	0,02	-	3,967268276
P2RY4	X	complement(70258166..70260204)	0,04	0,02	1,15046174
ARR3	X	70268305..70281840	0,02	0,01	1,436225396
PDZD11	X	complement(70281118..70290514)	4,57	4,37	0,288825394
RAB41	X	70282093..70285002	0,10	0,06	0,946003371
KIF4A	X	70290104..70420886	8,06	10,09	-0,099447631
GDPD2	X	70423031..70433390	0,04	0,03	0,943435191
DLG3	X	70444835..70505490	0,40	0,46	0,02847241
TEX11	X	complement(70528940..70908711)	-	-	#N/D
SLC7A3	X	complement(70925579..70931125)	-	-	#N/D
SNX12	X	complement(71056332..71073426)	10,55	10,49	0,231279747

FOXO4	X	71095851..71103532	1,61	0,99	0,919671714
CXorf65	X	complement(71103889..71106788)	-	-	0,188027316
gene:ENSG000	X	complement(71103987..71111575)	-	-	0,188027316
IL2RG	X	complement(71107404..71112108)	-	-	0,188027316
MED12	X	71118543..71144103	2,34	2,79	-0,029378891
NLGN3	X	71144821..71175255	0,05	0,02	1,330592333
GJB1	X	71212811..71225516	0,01	0,00	1,833201638
ZMYM3	X	complement(71239624..71255146)	9,39	7,76	0,498537788
NONO	X	71254814..71301522	35,22	39,04	0,074664218
ITGB1BP2	X	71301750..71305371	0,25	0,32	-0,117683659
TAF1	X	71366136..71532374	2,54	2,51	0,238364742
OGT	X	71533087..71575892	13,53	14,09	0,164683852
GCNA	X	71578437..71613583	0,06	0,17	-1,244895376
CXCR3	X	complement(71615916..71618511)	-	0,03	-5,071123353
gene:ENSG000	X	71667542..71671527	-	-	0,188027316
CXorf49	X	complement(71714371..71718285)	-	-	#N/D
CXorf49B	X	71763349..71767204	-	-	#N/D
NHSL2	X	71910845..72161750	0,03	0,38	-3,626672233
RTL5	X	complement(72127110..72131915)	0,72	0,86	-0,027238545
PIN4	X	72181353..72302926	0,26	0,23	0,411264476
ERCC6L	X	complement(72204657..72239027)	2,21	3,36	-0,385589468
RPS4X	X	complement(72255679..72277248)	156,99	#####	-0,113633994
CITED1	X	complement(72301638..72307187)	0,02	0,08	-1,462209385
gene:ENSG000	X	complement(72301691..72572843)	0,22	0,26	0,031833993
HDAC8	X	complement(72329516..72573101)	0,39	0,53	-0,225309667
PHKA1	X	complement(72578814..72714319)	1,31	3,09	-1,011494884
DMRTC1B	X	72776890..72848803	-	-	0,188027316
FAM236B	X	complement(72781865..72782660)	-	-	#N/D
FAM236D	X	complement(72807425..72808210)	-	-	#N/D
DMRTC1	X	complement(72872025..72943837)	-	-	#N/D
FAM236C	X	72912615..72913401	-	-	#N/D
FAM236A	X	72938163..72938958	-	-	#N/D
PABPC1L2B	X	73002939..73006106	-	-	#N/D
PABPC1L2A	X	complement(73077276..73079512)	-	-	0,188027316
NAP1L2	X	complement(73212299..73214851)	-	0,04	-5,545746556
CDX4	X	73447053..73455171	-	-	#N/D
CHIC1	X	73563197..73687111	0,33	0,40	-0,04748005
gene:ENSG000	X	complement(74122134..74122725)	0,13	0,35	-1,213543126
ZCCHC13	X	74304180..74305034	-	-	#N/D
SLC16A2	X	74421493..74533917	7,21	5,67	0,568908486
RLIM	X	complement(74582976..74614624)	5,93	5,38	0,364888547
NEXMIF	X	complement(74732856..74925472)	-	-	#N/D
ABCB7	X	complement(75051048..75156732)	2,31	2,49	0,116614873
UPRT	X	75156388..75304885	0,57	0,56	0,25304989
ZDHHC15	X	complement(75368427..75523502)	-	0,00	-2,89252005
MAGEE2	X	complement(75782987..75785254)	-	-	0,188027316
PBDC1	X	76173040..76178314	1,21	2,67	-0,923167798
MAGEE1	X	76427710..76431342	-	-	0,188027316

FGF16	X	77447389..77457278	-	-	0,188027316
ATRX	X	complement(77504880..77786233)	1,54	1,94	-0,108101178
MAGT1	X	complement(77825747..77899271)	6,11	5,51	0,371338694
COX7B	X	77899440..77907376	5,63	7,04	-0,099933389
ATP7A	X	77910690..78050395	0,52	0,49	0,32005908
PGK1	X	77910739..78129295	70,43	47,21	0,800233733
PGAM4	X	complement(77967949..77969638)	0,08	-	6,461896167
TAF9B	X	complement(78129748..78139650)	1,62	2,30	-0,279512965
CYSLTR1	X	complement(78271468..78327691)	-	-	0,188027316
RTL3	X	complement(78656068..78659328)	0,06	0,00	3,69954316
LPAR4	X	78747709..78758714	-	0,00	-2,89252005
P2RY10	X	78945332..78963727	-	-	#N/D
GPR174	X	79144688..79175318	-	-	0,188027316
ITM2A	X	complement(79360384..79367667)	-	-	#N/D
TBX22	X	80014753..80031774	-	-	#N/D
TENT5D	X	80335504..80445311	-	-	#N/D
BRWD3	X	complement(80669503..80809877)	0,84	0,99	-0,010480395
HMGN5	X	complement(81113699..81201913)	0,01	0,01	0,886720911
SH3BGRL	X	81202102..81298547	0,90	0,65	0,692196201
gene:ENSG000	X	complement(83506023..83509214)	-	-	#N/D
POU3F4	X	83508290..83512127	-	-	0,188027316
CYLC1	X	83861126..83886699	-	-	#N/D
RPS6KA6	X	complement(84058346..84206356)	-	-	0,188027316
HDX	X	complement(84317874..84502479)	0,11	-	8,816015151
APOOL	X	85003877..85093315	2,19	2,98	-0,224122345
SATL1	X	complement(85092284..85243911)	-	-	#N/D
ZNF711	X	85243991..85273362	-	-	#N/D
POF1B	X	complement(85277396..85379717)	-	-	#N/D
CHM	X	complement(85861180..86047561)	0,54	0,85	-0,412822722
DACH2	X	86148451..86832604	-	-	#N/D
KLHL4	X	87517409..87670050	0,02	-	6,68162262
CPXCR1	X	88747225..88754785	-	-	0,188027316
TGIF2LX	X	89921908..89922883	-	-	0,188027316
PABPC5	X	91434595..91438584	-	-	0,188027316
PCDH11X	X	91779261..92623230	-	-	0,188027316
NAP1L3	X	complement(93670930..93673578)	-	-	0,188027316
FAM133A	X	93674013..93712274	-	0,01	-3,804623889
DIAPH2	X	96684712..97604997	0,72	1,17	-0,472117059
RPA4	X	96883908..96885467	0,03	0,09	-1,151227584
PCDH19	X	complement(100291644..10041027	0,00	-	3,967268276
TNMD	X	100584936..100599885	-	0,01	-2,89252005
TSPAN6	X	complement(100627108..10063999	1,10	1,42	-0,154899895
SRPX2	X	100644195..100675788	2,83	2,82	0,227751835
SYTL4	X	complement(100671783..10073212	0,02	0,03	-0,182572847
CSTF2	X	100820391..100841520	3,32	5,51	-0,508269794
NOX1	X	complement(100843324..10087435	0,02	-	4,913749003
XKRX	X	complement(100913445..10092943	0,56	0,67	-0,032431803
ARL13A	X	100969708..100990831	0,13	0,13	0,129542238

TRMT2B	X	complement(101009346..10105211	3,30	4,85	-0,331162368
TMEM35A	X	101078879..101096367	0,01	0,02	-1,478387936
CENPI	X	101098188..101166126	1,75	1,75	0,224945406
DRP2	X	101219769..101264502	0,02	0,14	-2,418053091
TAF7L	X	complement(101268253..10129305	-	-	0,188027316
TIMM8A	X	complement(101345661..10134874	1,25	1,28	0,191355731
BTK	X	complement(101349338..10139079	0,00	0,01	-0,923955849
RPL36A	X	101390824..101396155	13,74	19,20	-0,259745596
RPL36A-HNRNI	X	101391011..101412297	18,43	27,01	-0,328343611
GLA	X	complement(101393273..10140801	3,35	4,44	-0,18313931
HNRNPH2	X	101408222..101414133	3,05	3,48	0,03432152
ARMCX4	X	101418287..101533459	0,00	0,00	-0,01185201
ARMCX1	X	101550547..101554700	5,39	2,61	1,266613916
ARMCX6	X	complement(101615118..10161800	6,31	5,00	0,558791373
ARMCX3	X	101622797..101627843	5,71	2,32	1,521630487
ARMCX2	X	complement(101655281..10165985	7,86	5,99	0,61547158
ZMAT1	X	complement(101882288..10193203	-	-	0,188027316
TCEAL2	X	102125679..102140426	-	-	0,188027316
TCEAL6	X	complement(102140476..10214297	-	-	#N/D
BEX5	X	complement(102153708..10215597	-	-	0,188027316
TCP11X1	X	102215298..102229532	-	-	#N/D
NXF2	X	102247167..102326722	-	-	0,188027316
NXF2B	X	complement(102360395..10244000	-	-	0,188027316
gene:ENSG000	X	complement(102360395..10247181	-	-	0,188027316
TCP11X2	X	complement(102456862..10247184	-	-	#N/D
TMSB15A	X	complement(102513682..10251673	-	-	0,188027316
ARMCX5	X	102599168..102604159	0,55	0,83	-0,374014175
GPRASP1	X	102651092..102659083	0,03	0,08	-1,371968249
GPRASP2	X	102712445..102717733	0,50	0,89	-0,60005682
ARMCX5-GPRA	X	102712495..102753530	0,08	0,08	0,075032032
GPRASP3	X	102720688..102753540	0,16	0,16	0,250457617
RAB40AL	X	102937272..102938300	0,09	0,10	0,130034011
BEX1	X	complement(103062651..10306417	-	-	0,188027316
NXF3	X	complement(103075810..10309314	-	-	0,188027316
BEX4	X	103215108..103217246	0,04	-	5,224730805
TCEAL8	X	complement(103252995..10325519	4,14	2,20	1,133252229
TCEAL5	X	complement(103273691..10327675	-	-	#N/D
BEX2	X	complement(103309346..10331100	0,03	0,05	-0,429837071
TCEAL7	X	103330229..103332326	-	-	#N/D
TCEAL9	X	103356489..103358462	1,03	0,62	0,965913343
BEX3	X	103376395..103378164	12,73	22,29	-0,584535289
RAB40A	X	complement(103499130..10351948	0,11	0,47	-1,894541678
TCEAL4	X	103576231..103587729	2,04	2,74	-0,20261108
gene:ENSG000	X	103596608..103598904	0,04	0,05	-0,204080844
TCEAL3	X	103607451..103629690	0,89	0,57	0,872672272
TCEAL1	X	103628704..103630953	1,07	1,79	-0,515932806
MORF4L2	X	complement(103675496..10368815	66,91	73,96	0,07859709
TMEM31	X	103710909..103714032	0,24	0,16	0,78195819

PLP1	X	103773718..103792619	0,00	0,12	-4,965153582
RAB9B	X	complement(103822327..10383225	0,03	0,03	-0,187130731
TMSB15B	X	103918896..103966712	0,39	0,12	1,944026192
H2BW1	X	complement(104011147..10401370	-	-	#N/D
H2BW2	X	104039956..104042454	-	-	0,188027316
TMSB15C	X	complement(104063871..10407623	0,26	0,05	2,530033546
SLC25A53	X	complement(104099214..10415700	0,06	0,07	0,081543383
ZCCHC18	X	104112131..104115842	0,03	0,13	-1,656886498
FAM199X	X	104166453..104195902	2,12	2,22	0,156180726
ESX1	X	complement(104250038..10425493	-	0,17	-7,042589436
IL1RAPL2	X	104566199..105767829	0,05	0,06	-0,242839224
TEX13A	X	complement(105218929..10522069	-	-	#N/D
NRK	X	105822539..105958610	-	-	0,188027316
SERPINA7	X	complement(106032435..10603872	-	-	#N/D
PWWP3B	X	106168305..106208956	-	-	0,188027316
RADX	X	106611930..106679439	0,00	-	3,068695355
RNF128	X	106693794..106797016	-	-	0,188027316
TBC1D8B	X	106802673..106876150	0,91	0,20	2,433193592
MORC4	X	complement(106813871..10700021	1,91	2,69	-0,269299409
RIPPLY1	X	complement(106900063..10690334	0,03	-	4,516772761
CLDN2	X	106900164..106930861	0,14	0,07	1,165619831
RBM41	X	complement(107052095..10711882	0,24	0,38	-0,41085473
NUP62CL	X	complement(107123427..10720643	0,05	0,03	1,106450337
DNAAF6	X	107206611..107244247	-	0,01	-2,89252005
FRMPD3	X	107449652..107605255	0,12	0,00	6,271989619
PRPS1	X	107628428..107651993	20,92	13,88	0,814638748
TSC22D3	X	complement(107713221..10777734	0,90	0,97	0,117354804
NCBP2L	X	107774899..107795829	-	-	#N/D
MID2	X	107825755..107931637	-	-	0,188027316
TEX13B	X	complement(107980864..10798237	-	-	0,188027316
VSIG1	X	108044970..108079184	0,02	-	5,697545031
PSMD10	X	complement(108084207..10809154	12,76	12,24	0,2822813
ATG4A	X	108091668..108154671	1,07	1,39	-0,151127083
COL4A6	X	complement(108155607..10843949	1,50	0,37	2,256290636
COL4A5	X	108439838..108697545	11,62	3,77	1,847120027
IRS4	X	complement(108719946..10873656	-	0,00	-2,89252005
GUCY2F	X	complement(109372906..10948208	-	0,01	-3,804623889
NXT2	X	109535781..109544698	0,69	0,78	0,048951881
KCNE5	X	complement(109623700..10962517	0,20	0,28	-0,283656182
ACSL4	X	complement(109624244..10973340	5,43	7,74	-0,28722197
TMEM164	X	110002631..110182734	4,60	6,75	-0,328268132
AMMECR1	X	complement(110194186..11044031	2,56	2,51	0,251814496
GNG5B	X	complement(110346103..11044037	-	-	#N/D
RTL9	X	110358848..110456334	0,03	0,05	-0,548994131
CRIPTO3	X	110520896..110523021	-	-	0,188027316
CHRDL1	X	complement(110673856..11079581	-	0,00	-2,89252005
PAK3	X	110944285..111227361	0,00	0,02	-2,097518818
CAPN6	X	complement(111245099..11127048	-	-	0,188027316

DCX	X	complement(111293779..11141242	-	-	#N/D
SERTM2	X	111511645..111522399	-	-	#N/D
ALG13	X	111665811..111760649	1,01	1,36	-0,207754566
TRPC5	X	complement(111768011..11208277	0,01	0,01	0,728122296
TRPC5OS	X	111876051..111903990	-	-	0,188027316
RTL4	X	112083016..112457514	0,01	-	5,224730805
LHFPL1	X	complement(112630648..11268005	0,03	0,01	1,833201638
AMOT	X	complement(112774503..11284083	0,60	0,19	1,898154171
HTR2C	X	114584078..114910061	-	0,00	-2,89252005
IL13RA2	X	complement(115003975..11501997	0,96	2,73	-1,288289949
LRCH2	X	complement(115110616..11523409	0,04	0,27	-2,546797201
RBMXL3	X	115189410..115192868	-	-	0,188027316
LUZP4	X	115289715..115307563	-	-	#N/D
PLS3	X	115561174..115650861	20,59	23,79	0,014158636
AGTR2	X	116170744..116174974	-	-	0,188027316
SLC6A14	X	116436606..116461458	-	-	0,188027316
CT83	X	complement(116461686..11646297	-	-	0,188027316
gene:ENSG000	X	complement(117894632..11789568	-	-	#N/D
KLHL13	X	complement(117897813..11811734	0,17	0,18	0,131706468
WDR44	X	118346073..118449961	1,37	1,56	0,039714871
DOCK11	X	118495815..118686163	1,19	2,11	-0,599978828
IL13RA1	X	118727133..118794535	6,46	10,41	-0,466192258
ZCCHC12	X	118823824..118826968	0,01	0,01	-0,025382928
LONRF3	X	118974614..119018355	0,80	3,01	-1,6849592
CT47C1	X	119072993..119076765	-	-	#N/D
KIAA1210	X	complement(119078635..11915057	0,00	0,00	-0,01185201
PGRMC1	X	119236245..119244466	14,62	49,36	-1,53225639
SLC25A43	X	119399336..119454478	3,45	12,68	-1,652930617
SLC25A5	X	119468422..119471396	77,82	#####	-1,06400603
UBE2A	X	119474802..119591083	4,47	13,90	-1,414101419
STEEP1	X	complement(119538149..11956540	2,79	6,73	-1,049921132
NKRF	X	complement(119588337..11960644	2,05	5,75	-1,262530063
SEPTIN6	X	complement(119615724..11969337	1,16	1,27	0,096643158
gene:ENSG000	X	119693568..119696059	0,01	0,02	-0,979341556
SOWAHD	X	119758588..119760202	0,06	0,19	-1,438989101
RPL39	X	complement(119786504..11979163	249,16	#####	-1,098531813
UPF3B	X	complement(119834022..11985299	0,96	3,20	-1,511894954
RNF113A	X	complement(119870475..11987173	3,86	7,75	-0,782074033
NDUFA1	X	119871832..119876662	22,31	50,04	-0,942211235
AKAP14	X	119895837..119920716	-	-	0,188027316
NKAP	X	complement(119920672..11994375	1,02	2,31	-0,953520292
RHOXF1P3	X	119942834..119992838	0,28	0,55	-0,77624812
RHOXF2B	X	complement(120072264..12007769	-	-	#N/D
RHOXF1	X	complement(120109051..12024526	0,12	0,18	-0,399845206
RHOXF2	X	120158613..120165630	0,04	0,01	1,655502569
ZBTB33	X	120250752..120258398	1,99	5,20	-1,162653529
TMEM255A	X	complement(120258650..12031155	0,01	0,04	-1,586291974
ATP1B4	X	120362085..120383249	0,01	-	4,516772761

LAMP2	X	complement(120426148..12046936	2,71	10,77	-1,769056482
CUL4B	X	complement(120505920..12060407	0,72	1,07	-0,347236367
MCTS1	X	120594010..120621159	1,29	1,72	-0,188909556
C1GALT1C1	X	complement(120625674..12063005	1,06	2,16	-0,802921708
CT47B1	X	complement(120872607..12087586	-	-	#N/D
gene:ENSG000	X	complement(120877496..12087892	-	-	#N/D
CT47A12	X	complement(120930250..12093230	-	-	#N/D
CT47A11	X	complement(120933840..12093715	-	-	#N/D
CT47A10	X	complement(120938701..12094202	-	-	#N/D
CT47A9	X	complement(120943561..12094688	-	-	#N/D
CT47A8	X	complement(120948422..12095174	-	-	#N/D
CT47A7	X	complement(120953282..12095660	-	-	#N/D
CT47A6	X	complement(120958165..12096148	-	-	#N/D
CT47A5	X	complement(120963026..12096634	-	-	#N/D
CT47A4	X	complement(120967886..12097120	-	-	#N/D
CT47A3	X	complement(120972746..12097606	-	-	#N/D
CT47A2	X	complement(120977606..12098092	-	-	#N/D
CT47A1	X	complement(120982476..12098576	-	-	#N/D
GLUD2	X	121047610..121050094	0,08	0,02	2,493547104
gene:ENSG000	X	122242008..122242616	-	-	#N/D
GRIA3	X	123184153..123490915	-	-	0,188027316
THOC2	X	complement(123600561..12373305	2,38	3,39	-0,289634108
XIAP	X	123859712..123913972	1,95	2,43	-0,098925026
STAG2	X	123960212..124422664	2,92	4,29	-0,331596736
SH2D1A	X	124227868..124373197	-	-	0,188027316
TEX13D	X	124246249..124336863	0,01	-	4,516772761
TENM1	X	complement(124375903..12520431	0,96	0,00	7,830426668
TEX13C	X	125319871..125325216	0,02	-	6,338059249
gene:ENSG000	X	complement(126004446..12600611	-	-	#N/D
DCAF12L2	X	complement(126163499..12616628	0,56	-	10,03963904
DCAF12L1	X	complement(126549383..12655281	-	-	#N/D
PRR32	X	126819729..126821786	-	-	#N/D
gene:ENSG000	X	127392760..127394771	-	-	#N/D
ACTRT1	X	complement(128050962..12805240	-	-	#N/D
SMARCA1	X	complement(129446501..12952350	7,50	6,55	0,4184717
OCRL	X	129539849..129592561	6,00	6,05	0,212413188
APLN	X	complement(129645259..12965495	0,08	0,32	-1,712332243
XPNPEP2	X	129738949..129769536	-	-	#N/D
SASH3	X	129779949..129795201	0,07	0,03	1,524737598
ZDHHC9	X	complement(129803288..12984390	6,42	5,19	0,531057168
UTP14A	X	129906121..129929761	1,51	3,44	-0,967579944
BCORL1	X	129981107..130058071	2,30	2,35	0,19198912
ELF4	X	complement(130063955..13011071	6,00	7,29	-0,058107446
AIFM1	X	complement(130124666..13016587	2,62	3,77	-0,303591536
RAB33A	X	130171962..130184870	0,02	0,10	-1,766505595
ZNF280C	X	complement(130202707..13026889	0,44	1,11	-1,118571231
SLC25A14	X	130339888..130373361	0,96	1,36	-0,27182429
GPR119	X	complement(130379449..13038567	-	-	0,188027316

RBMX2	X	130401987..130413656	2,29	2,67	0,004702185
ENOX2	X	complement(130622325..13090323	0,45	1,00	-0,9389321
ARHGAP36	X	131058346..131089885	-	-	#N/D
IGSF1	X	complement(131273506..13157889	0,00	-	4,516772761
OR13H1	X	131544074..131545000	-	-	0,188027316
STK26	X	132023302..132075943	0,59	0,00	7,294558138
FRMD7	X	complement(132076990..13212802	-	-	0,188027316
RAP2C	X	complement(132203024..13221948	1,56	1,65	0,139379191
MBNL3	X	complement(132369320..13248996	0,18	0,24	-0,189233445
HS6ST2	X	complement(132626015..13296139	1,59	4,04	-1,122060078
USP26	X	complement(133023168..13309710	-	-	#N/D
TFDP3	X	complement(133216662..13321835	-	-	#N/D
GPC4	X	complement(133300103..13341548	2,49	0,08	5,161284341
GPC3	X	complement(133535745..13398710	0,01	-	4,516772761
GPC3-AS1	X	133897443..133897934	-	-	#N/D
CCDC160	X	134237047..134246842	-	-	0,188027316
PHF6	X	134373288..134428791	1,30	1,73	-0,188094489
HPRT1	X	134460165..134520513	5,02	8,20	-0,4843597
PLAC1	X	complement(134565838..13476432	0,08	0,62	-2,771289281
PABIR2	X	complement(134769566..13479723	4,41	4,95	0,05689099
PABIR3	X	134796395..134854835	0,16	0,24	-0,380545918
MOSPD1	X	complement(134887632..13491525	0,73	1,84	-1,110858381
SMIM10	X	134990991..134992473	0,99	1,72	-0,574787675
RTL8B	X	complement(135020513..13502254	-	-	0,188027316
RTL8C	X	135032355..135033546	18,79	14,19	0,628424412
RTL8A	X	complement(135050932..13505219	11,05	11,56	0,158126552
SMIM10L2B	X	complement(135094985..13509871	0,09	0,01	2,844467704
ETDB	X	complement(135118955..13512052	-	0,02	-2,89252005
CT55	X	complement(135156540..13517139	-	-	0,188027316
ZNF75D	X	complement(135248920..13534410	0,86	0,70	0,520102435
ETDA	X	135252061..135253583	-	-	0,188027316
ETDC	X	135309480..135309659	-	-	#N/D
ZNF449	X	135344796..135363413	0,67	0,63	0,317510208
SMIM10L2A	X	135421944..135428075	0,21	0,00	6,466319701
INTS6L	X	135520659..135582510	0,46	0,52	0,057536495
CT45A1	X	135713639..135723539	-	-	#N/D
CT45A3	X	complement(135759846..13576819	-	-	#N/D
CT45A5	X	complement(135775852..13578550	-	-	0,188027316
CT45A6	X	complement(135794685..13580278	-	-	#N/D
CT45A2	X	complement(135811668..13582001	-	-	#N/D
CT45A7	X	complement(135829229..13583728	-	-	#N/D
CT45A8	X	complement(135846497..13585453	-	-	#N/D
CT45A9	X	complement(135863418..13587181	-	-	#N/D
CT45A10	X	complement(135881063..13589344	-	-	0,188027316
SAGE1	X	135890182..135913062	-	-	#N/D
MMGT1	X	complement(135960588..13597402	1,82	2,14	-0,015844728
SLC9A6	X	135973841..136047269	1,11	0,99	0,384993004
FHL1	X	136146702..136211359	10,37	4,97	1,284242592

MAP7D3	X	complement(136213220..13625648	2,11	3,21	-0,385094078
ADGRG4	X	136300963..136416890	-	0,01	-5,734998842
BRS3	X	136487947..136493780	0,02	0,00	1,833201638
HTATSF1	X	136497079..136512346	3,91	5,82	-0,351243273
VGLL1	X	136532215..136556807	-	-	0,188027316
CD40LG	X	136648158..136660390	-	-	#N/D
ARHGEF6	X	complement(136665547..13678093	1,21	1,73	-0,295634
RBMX	X	complement(136848004..13688076	17,85	22,81	-0,131209004
GPR101	X	complement(137023929..13703399	-	-	0,188027316
ZIC3	X	137566127..137577691	-	-	#N/D
FGF13	X	complement(138614727..13922277	0,12	0,04	1,90963935
F9	X	139530739..139563459	-	-	#N/D
MCF2	X	complement(139581770..13970821	-	0,00	-2,89252005
ATP11C	X	complement(139726346..13994527	0,82	2,17	-1,180034486
CXorf66	X	complement(139955728..13996552	0,01	-	3,068695355
HAPSTR2	X	140091678..140092911	-	-	0,188027316
SOX3	X	complement(140502985..14050506	-	-	#N/D
SPANXB1	X	141002594..141003706	-	-	0,188027316
LDOC1	X	complement(141111605..14117712	4,52	3,36	0,647612406
SPANXC	X	complement(141241463..14124251	-	-	0,188027316
SPANXA1	X	complement(141583674..14158473	-	-	0,188027316
SPANXA2	X	141589708..141590762	0,11	-	4,913749003
SPANXD	X	complement(141697411..14169873	-	-	0,188027316
MAGEC3	X	141838316..141897832	0,02	0,01	0,366665713
MAGEC1	X	141903894..141909374	-	-	0,188027316
MAGEC2	X	complement(142202342..14220529	-	-	0,188027316
SPANXN4	X	143025928..143034702	-	-	#N/D
SPANXN3	X	complement(143508735..14351747	-	-	0,188027316
SLITRK4	X	complement(143622790..14363610	0,31	0,01	5,660561538
SPANXN2	X	complement(143711955..14372075	-	-	#N/D
UBE2NL	X	143884071..143885255	-	-	0,188027316
SPANXN1	X	145247503..145256208	-	-	#N/D
gene:ENSG000	X	145797375..145798439	-	-	#N/D
SLITRK2	X	145817829..145829856	0,03	0,00	4,593047663
CXorf51B	X	146809771..146810411	-	-	#N/D
CXorf51A	X	complement(146814106..14681474	-	-	0,188027316
FMR1	X	147911919..147951125	0,98	1,58	-0,471163724
FMR1NB	X	147981337..148026665	-	0,01	-2,89252005
AFF2	X	148500617..149000663	-	-	0,188027316
gene:ENSG000	X	complement(149414877..14941550	-	-	#N/D
IDS	X	complement(149476988..14952109	2,01	2,97	-0,342399008
gene:ENSG000	X	complement(149477104..14954092	0,95	1,01	0,120546093
EOLA1	X	149540355..149550510	1,94	1,22	0,894831625
HSFX3	X	complement(149548210..14954992	-	-	0,188027316
MAGEA9B	X	complement(149581653..14958745	-	0,02	-4,359055975
HSFX2	X	complement(149592512..14959531	0,15	-	7,621425725
TMEM185A	X	complement(149596556..14963191	3,12	3,14	0,213874209
MAGEA11	X	149688228..149717268	-	-	0,188027316

HSFX1	X	149774068..149776867	-	0,20	-7,624346382
MAGEA9	X	149781930..149787737	-	-	#N/D
MAGEA8	X	149881141..149885835	0,01	-	3,967268276
EOLA2	X	complement(149929527..14993870	8,80	5,17	0,991629607
HSFX4	X	149929573..149931287	0,45	0,26	1,012337294
MAMLD1	X	150361422..150514178	0,37	0,76	-0,79705942
MTM1	X	150568417..150673143	0,64	0,89	-0,257323793
MTMR1	X	150692971..150765108	1,97	3,21	-0,483933552
CD99L2	X	complement(150766336..15089881	6,09	8,54	-0,264564788
HMGB3	X	150980509..150990771	3,38	4,33	-0,135351944
GPR50	X	151176584..151181465	-	0,01	-2,89252005
VMA21	X	151396515..151409364	3,15	4,47	-0,279594265
PASD1	X	151563675..151676739	-	-	#N/D
PRRG3	X	151694607..151705924	-	-	0,188027316
FATE1	X	151716035..151723194	-	-	#N/D
CNGA2	X	151734746..151745564	-	-	#N/D
MAGEA4	X	151912495..151925171	-	-	#N/D
GABRE	X	complement(151953124..15197468	2,81	5,09	-0,631483987
MAGEA10	X	complement(152133310..15213857	0,02	0,02	0,533811434
GABRA3	X	complement(152166234..15245131	0,11	0,14	-0,183917926
GABRQ	X	152637895..152657542	0,13	0,74	-2,266139978
MAGEA3	X	152698767..152702347	-	-	0,188027316
CSAG2	X	complement(152708261..15272166	-	-	#N/D
MAGEA2B	X	152714586..152718607	-	-	0,188027316
CSAG1	X	complement(152727484..15273373	-	-	0,188027316
MAGEA12	X	152733757..152737669	-	-	0,188027316
MAGEA2	X	complement(152749863..15275388	-	-	0,188027316
CSAG3	X	152753921..152760222	0,03	0,06	-0,979341556
MAGEA6	X	complement(152766136..15276971	-	-	0,188027316
CETN2	X	complement(152826994..15283075	6,25	6,50	0,167198841
NSDHL	X	152830967..152869729	6,76	4,90	0,685666067
ZNF185	X	152898067..152973481	0,21	0,45	-0,857618368
PNMA5	X	complement(152988824..15299411	-	-	0,188027316
PNMA3	X	153056409..153060467	-	-	#N/D
PNMA6A	X	153072454..153075019	0,00	-	3,068695355
MAGEA1	X	153179284..153183880	-	-	0,188027316
PWWP4	X	153266152..153279145	-	-	#N/D
PNMA6F	X	complement(153317680..15332176	-	-	0,188027316
ZNF275	X	153334147..153360110	3,16	2,68	0,458848237
PNMA6E	X	complement(153395639..15340139	-	-	0,188027316
ZFP92	X	153411472..153426481	-	-	0,188027316
TREX2	X	complement(153444473..15347058	0,22	0,26	0,015095183
HAUS7	X	complement(153447668..15349551	4,82	4,69	0,262067767
BGN	X	153494980..153509546	205,31	#####	1,133227533
ATP2B3	X	153517642..153582939	0,05	0,01	2,378273779
CCNQ	X	complement(153587925..15360004	3,38	4,47	-0,182031198
DUSP9	X	153642492..153651326	2,83	2,04	0,697397216
PNCK	X	complement(153669733..15368901	0,12	0,08	0,744800911

SLC6A8	X	153687926..153696588	12,71	16,53	-0,156711515
BCAP31	X	complement(153700492..15372456	30,52	32,65	0,125750011
ABCD1	X	153724856..153744755	6,74	10,63	-0,435312523
PLXNB3	X	153764196..153779346	1,16	0,89	0,606167128
SRPK3	X	153776412..153785732	0,24	0,31	-0,14447004
IDH3G	X	complement(153785766..15379451	13,87	9,59	0,756255289
SSR4	X	153793516..153798499	17,80	18,04	0,203727201
PDZD4	X	complement(153802166..15383056	0,08	0,11	-0,12963645
L1CAM	X	complement(153861514..15388617	0,01	0,79	-6,792237455
gene:ENSG000	X	complement(153872221..15390922	-	-	#N/D
AVPR2	X	153902531..153907166	0,00	0,05	-2,854330803
ARHGAP4	X	complement(153907367..15393499	0,12	0,13	0,100290568
NAA10	X	complement(153929225..15393508	8,41	11,63	-0,244627803
RENBP	X	complement(153935269..15394468	0,01	0,02	-0,923955849
HCFC1	X	complement(153947557..15397181	19,04	20,56	0,112340586
TMEM187	X	153972754..153983194	1,80	1,96	0,104773211
IRAK1	X	complement(154010506..15401990	27,56	39,38	-0,29177935
MECP2	X	complement(154021573..15413710	3,96	3,76	0,297914792
OPN1LW	X	154144243..154159032	-	-	#N/D
OPN1MW	X	154182596..154196861	-	-	0,188027316
OPN1MW2	X	154219756..154233286	-	-	#N/D
OPN1MW3	X	154257582..154271090	-	-	0,188027316
TEX28	X	complement(154271265..15429585	-	-	0,188027316
TKTL1	X	154295795..154330350	0,00	-	3,068695355
FLNA	X	complement(154348524..15437463	194,58	#####	0,450253715
EMD	X	154379273..154381574	23,68	24,12	0,196461975
RPL10	X	154389955..154409168	134,78	#####	0,104047493
DNASE1L1	X	complement(154401236..15441211	5,56	2,60	1,320665172
TAFAZZIN	X	154411524..154421726	5,98	7,78	-0,155334895
ATP6AP1	X	154428633..154436516	8,22	9,38	0,033247211
GDI1	X	154436913..154443467	36,65	32,62	0,391041022
FAM50A	X	154444141..154450654	9,07	11,57	-0,128316118
PLXNA3	X	154458281..154477779	2,27	1,74	0,608290753
LAGE3	X	complement(154477775..15447928	5,89	7,06	-0,038085709
UBL4A	X	complement(154483717..15448661	11,21	16,61	-0,344050548
SLC10A3	X	complement(154487306..15449069	6,82	5,06	0,652028021
FAM3A	X	complement(154506159..15451624	8,39	8,39	0,224427074
G6PD	X	complement(154517825..15454757	16,15	9,07	1,054509197
IKBKG	X	154541199..154565046	4,91	5,45	0,072080084
CTAG1A	X	154585133..154586816	-	-	#N/D
CTAG1B	X	complement(154617609..15461928	0,04	-	4,913749003
CTAG2	X	complement(154651972..15465357	-	-	#N/D
GAB3	X	complement(154675249..15475158	0,04	0,10	-1,220681718
DKC1	X	154762742..154777689	7,50	11,78	-0,428682772
MPP1	X	complement(154778684..15482100	6,48	5,21	0,537948374
SMIM9	X	complement(154823348..15483466	-	-	#N/D
F8	X	complement(154835788..15502694	0,12	0,22	-0,636725087
H2AB1	X	154884972..154885558	0,04	0,02	0,886720911

F8A1	X	154886355..154888061	2,05	5,25	-1,135241228
FUNDC2	X	155025980..155060304	1,51	1,55	0,185054372
gene:ENSG000	X	complement(155061622..15506551	0,07	0,11	-0,429837071
CMC4	X	complement(155061622..15507113	1,62	1,90	-0,011139812
MTCP1	X	complement(155064034..15514793	0,48	0,61	-0,122914325
BRCC3	X	155071420..155123077	1,10	2,12	-0,71751515
VBP1	X	155197007..155239841	3,74	4,69	-0,1043642
RAB39B	X	complement(155258235..15526449	0,03	0,11	-1,561099539
CLIC2	X	complement(155276211..15533465	0,00	0,00	-0,01185201
H2AB2	X	155380709..155381299	-	0,02	-2,89252005
F8A2	X	155382095..155383801	0,55	1,10	-0,776578791
F8A3	X	complement(155456914..15545862	0,57	0,71	-0,087244559
H2AB3	X	complement(155459415..15546000	-	0,04	-3,804623889
TMLHE	X	complement(155489011..15571909	1,65	2,08	-0,116545699
SPRY3	X	155612572..155782459	0,10	0,18	-0,624736487
VAMP7_1	X	155881345..155943769	1,33	1,68	-0,106640443
IL9R_1	X	155997696..156010817	0,11	0,09	0,572773295
WASH6P_1	X	156020826..156025710	8,65	10,37	-0,038349722
PLCXD1_2	Y	276322..303356	2,94	3,09	0,148890546
GTPBP6_2	Y	complement(304529..318796)	6,22	11,36	-0,646511915
PPP2R3B_2	Y	complement(333933..386955)	3,28	5,60	-0,54877285
SHOX_2	Y	624344..659411	-	-	0,188027316
CRLF2_2	Y	complement(1190490..1212723)	1,35	4,53	-1,523562646
CSF2RA_2	Y	1268800..1310381	0,01	0,05	-2,408690112
IL3RA_2	Y	1336616..1382689	0,43	0,42	0,239104838
SLC25A6_2	Y	complement(1386152..1392113)	193,33	#####	0,056551718
ASMTL_2	Y	complement(1403139..1453762)	33,46	20,81	0,908505704
P2RY8_2	Y	complement(1462581..1537185)	0,05	0,02	1,38420186
AKAP17A_2	Y	1591604..1602520	30,02	16,33	1,101627714
ASMT_2	Y	1615059..1643081	0,02	0,06	-1,54862452
DHRSX_2	Y	complement(2219506..2502805)	4,41	7,42	-0,527397798
ZBED1_2	Y	complement(2486414..2500976)	8,64	12,03	-0,253675828
CD99_2	Y	2691187..2741309	119,65	#####	0,475711097
SRY	Y	complement(2786855..2787682)	-	-	#N/D
RPS4Y1	Y	2841602..2932000	388,02	#####	0,441552955
ZFY	Y	2935281..2982506	1,25	1,86	-0,35303279
TGIF2LY	Y	3579067..3580041	-	-	#N/D
PCDH11Y	Y	5000226..5742224	0,00	0,01	-2,123038518
TSPY2	Y	6246223..6249019	-	-	#N/D
AMELY	Y	complement(6865918..6911752)	-	-	#N/D
TBL1Y	Y	6910686..7101970	-	-	#N/D
TSPY4	Y	9337464..9340284	-	-	#N/D
TSPY8	Y	9357797..9360599	-	-	#N/D
TSPY3	Y	9398421..9401223	-	-	#N/D
TSPY1	Y	9466955..9469749	-	-	#N/D
TSPY9	Y	9487267..9490081	-	-	0,188027316
TSPY10	Y	9527880..9530682	-	-	#N/D
USP9Y	Y	12537650..12860839	3,27	2,92	0,386418525

DDX3Y	Y	12904108..12920478	11,45	9,45	0,499083261
UTY	Y	complement(13234577..13480673)	1,97	1,79	0,361096519
TMSB4Y	Y	13703899..13706024	0,03	0,01	1,833201638
VCY	Y	complement(13985772..13986473)	-	-	#N/D
VCY1B	Y	14056227..14056958	-	-	#N/D
NLGN4Y	Y	14522573..14845654	1,75	1,82	0,168310935
CDY2B	Y	complement(17878260..17880220)	-	-	#N/D
CDY2A	Y	18025787..18027746	-	-	#N/D
HSFY1	Y	18546691..18588963	-	-	0,188027316
HSFY2	Y	complement(18731440..18773735)	-	-	0,188027316
KDM5D	Y	complement(19703865..19744939)	4,59	5,40	-0,009185664
EIF1AY	Y	20575776..20593154	2,11	3,99	-0,697061273
RPS4Y2	Y	20756108..20781032	-	-	#N/D
RBMY1B	Y	21511338..21525786	-	-	#N/D
RBMY1A1	Y	21534879..21549326	0,01	-	3,068695355
RBMY1D	Y	complement(21880076..21894526)	-	-	0,188027316
RBMY1E	Y	complement(21903618..21918042)	-	-	0,188027316
PRY2	Y	complement(22071756..22096007)	-	-	#N/D
RBMY1F	Y	complement(22168542..22182982)	-	-	#N/D
RBMY1J	Y	22403410..22417881	-	-	#N/D
PRY	Y	22490397..22514637	-	0,01	-2,89252005
BPY2	Y	22973819..23005465	-	-	#N/D
DAZ1	Y	complement(23129355..23199010)	0,00	-	3,068695355
DAZ2	Y	23219447..23291356	-	-	#N/D
CDY1B	Y	complement(24045229..24048019)	-	-	#N/D
BPY2B	Y	24607560..24639207	-	-	#N/D
DAZ3	Y	complement(24763069..24813492)	-	-	#N/D
DAZ4	Y	24833843..24907040	-	-	#N/D
BPY2C	Y	complement(25030901..25062548)	-	-	#N/D
CDY1	Y	25622117..25624902	-	-	#N/D
VAMP7_2	Y	57067865..57130289	1,26	1,85	-0,328006621
IL9R_2	Y	57184216..57197337	0,05	0,11	-0,81906241
WASH6P_2	Y	57207346..57212230	8,44	10,41	-0,078816778
MT-ND1	MT	3307..4262	767,97	#####	-0,983368387
MT-ND2	MT	4470..5511	736,94	#####	-1,385699922
MT-CO1	MT	5904..7445	#######	#####	-0,309452639
MT-CO2	MT	7586..8269	#######	#####	-0,71117725
MT-ATP8	MT	8366..8572	7,66	63,80	-2,834212537
MT-ATP6	MT	8527..9207	589,76	#####	-1,169188091
MT-CO3	MT	9207..9990	932,05	#####	-1,054093468
MT-ND3	MT	10059..10404	163,51	#####	-0,925267932
MT-ND4L	MT	10470..10766	791,40	#####	-0,008894774
MT-ND4	MT	10760..12137	#######	#####	-0,171053238
MT-ND5	MT	12337..14148	698,77	#####	0,046050349
MT-ND6	MT	complement(14149..14673)	208,19	#####	-0,400643865
MT-CYB	MT	14747..15887	581,18	#####	-1,080344488

Ratio	P-value	FDR p-value	ENSEMBL
#N/D	#N/D	#N/D	ENSG00000186092
0,07	0,313280	0,425353	ENSG00000284733
0,13	0,46	0,56	ENSG00000284662
1,43	0,00	0,00	ENSG00000187634
1,16	0,07	0,12	ENSG00000188976
1,29	0,00	0,01	ENSG00000187961
0,98	0,82	0,89	ENSG00000187583
0,79	0,19	0,28	ENSG00000187642
0,81	0,05	0,10	ENSG00000188290
1,33	0,00	0,00	ENSG00000187608
1,37	0,00	0,00	ENSG00000188157
0,08	0,00	0,00	ENSG00000237330
1,04	0,67	0,76	ENSG00000131591
15,64	0,33	0,44	ENSG00000162571
3,46	0,05	0,10	ENSG00000186891
1,29	0,46	0,56	ENSG00000186827
1,06	0,47	0,58	ENSG00000078808
0,92	0,35	0,46	ENSG00000176022
3,51	0,00	0,00	ENSG00000184163
0,91	0,25	0,35	ENSG00000160087
0,77	0,01	0,02	ENSG00000162572
1,44	0,00	0,00	ENSG00000131584
1,64	0,00	0,00	ENSG00000169972
1,29	0,00	0,00	ENSG00000127054
0,92	0,30	0,41	ENSG00000224051
1,37	0,00	0,00	ENSG00000169962
1,25	0,00	0,01	ENSG00000107404
1,58	0,00	0,00	ENSG00000162576
1,07	0,42	0,54	ENSG00000175756
1,23	0,01	0,02	ENSG00000221978
0,94	0,47	0,57	ENSG00000242485
0,98	0,97	0,99	ENSG00000235098
0,40	0,12	0,19	ENSG00000205116
1,79	0,00	0,00	ENSG00000179403
1,28	0,27	0,37	ENSG00000215915
1,79	0,00	0,00	ENSG00000160072
1,33	0,00	0,00	ENSG00000197785
0,90	0,45	0,56	ENSG00000205090
1,23	0,01	0,03	ENSG00000160075
0,57	0,00	0,00	ENSG00000228594
1,55	0,00	0,00	ENSG00000197530
0,85	0,41	0,53	ENSG00000189409
1,08	0,36	0,47	ENSG00000248333
0,93	0,35	0,47	ENSG00000189339
1,28	0,00	0,01	ENSG00000008128

0,88	0,12	0,20	ENSG00000008130
1,25	0,01	0,01	ENSG00000078369
2,87	0,04	0,08	ENSG00000169885
7,60	0,00	0,00	ENSG00000178821
0,53	0,58	0,68	ENSG00000142609
5,28	0,10	0,17	ENSG00000187730
1,74	0,00	0,00	ENSG00000067606
1,14	0,12	0,19	ENSG00000162585
1,16	0,06	0,11	ENSG00000157933
1,20	0,06	0,10	ENSG00000116151
2,16	0,00	0,00	ENSG00000157916
0,99	0,93	0,98	ENSG00000157911
1,55	0,00	0,01	ENSG00000149527
1,47	0,00	0,00	ENSG00000157881
1,14	0,97	0,98	ENSG00000197921
21,48	0,00	0,00	ENSG00000157873
0,74	0,69	0,78	ENSG00000225931
1,11	0,24	0,35	ENSG00000157870
1,14	0,97	0,98	ENSG00000142606
0,82	0,09	0,15	ENSG00000215912
#N/D	#N/D	#N/D	ENSG00000169717
3,49	0,00	0,00	ENSG00000142611
3,82	0,01	0,02	ENSG00000279839
0,14	0,00	0,00	ENSG00000130762
1,14	0,09	0,16	ENSG00000162591
1,90	0,00	0,00	ENSG00000158109
1,10	0,27	0,38	ENSG00000116213
0,93	0,41	0,53	ENSG00000078900
0,98	0,97	0,99	ENSG00000162592
1,45	0,56	0,67	ENSG00000235169
1,14	0,10	0,17	ENSG00000130764
0,89	0,14	0,22	ENSG00000116198
1,17	0,10	0,17	ENSG00000169598
1,61	0,00	0,00	ENSG00000198912
602,97	0,02	0,04	ENSG00000196581
1,48	0,00	0,00	ENSG00000131697
0,78	0,00	0,01	ENSG00000069424
4,89	0,00	0,00	ENSG00000116254
3,46	0,05	0,10	ENSG00000285629
0,93	0,34	0,45	ENSG00000116251
0,93	0,43	0,55	ENSG00000158286
1,01	0,87	0,94	ENSG00000116237
0,13	0,46	#N/D	ENSG00000173673
2,50	0,00	0,00	ENSG00000158292
0,46	0,00	0,00	ENSG00000097021
0,27	0,00	0,00	ENSG00000069812
0,92	0,81	0,88	ENSG00000187017
2,03	0,00	0,00	ENSG00000215788

1,54	0,00	0,00	ENSG00000171680
0,63	0,00	0,00	ENSG00000162408
0,75	0,50	0,61	ENSG00000173662
1,20	0,05	0,09	ENSG00000204859
0,98	0,83	0,90	ENSG00000162413
1,25	0,01	0,01	ENSG00000116273
1,04	0,65	0,74	ENSG00000041988
0,68	0,00	0,00	ENSG00000007923
1,16	0,09	0,15	ENSG00000171735
1,30	0,00	0,00	ENSG00000049245
1,39	0,00	0,00	ENSG00000049246
0,14	0,05	0,09	ENSG00000049247
0,08	0,00	0,00	ENSG00000049249
1,07	0,41	0,53	ENSG00000116288
1,50	0,00	0,00	ENSG00000116285
0,68	0,01	0,02	ENSG00000162426
0,67	0,00	0,00	ENSG00000142599
1,52	0,00	0,00	ENSG00000074800
0,07	0,31	#N/D	ENSG00000131686
1,14	0,97	0,98	ENSG00000197241
2,90	0,00	0,00	ENSG00000142583
1,07	0,39	0,51	ENSG00000180758
0,80	0,01	0,02	ENSG00000049239
0,65	0,00	0,00	ENSG00000171621
0,79	0,01	0,02	ENSG00000171612
0,73	0,00	0,00	ENSG00000188807
0,59	0,00	0,00	ENSG00000171608
0,77	0,00	0,00	ENSG00000171603
0,51	0,42	0,54	ENSG00000280113
1,49	0,00	0,00	ENSG00000178585
1,03	0,72	0,81	ENSG00000162441
0,74	0,00	0,01	ENSG00000173614
1,17	0,54	0,65	ENSG00000162444
1,10	0,25	0,35	ENSG00000130939
0,78	0,00	0,01	ENSG00000054523
0,75	0,00	0,00	ENSG00000142657
0,53	0,00	0,01	ENSG00000251503
0,47	0,00	0,00	ENSG00000175279
0,93	0,81	0,89	ENSG00000241563
0,65	0,00	0,00	ENSG00000160049
0,94	0,44	0,56	ENSG00000142655
0,95	0,64	0,74	ENSG00000130940
0,13	0,46	#N/D	ENSG00000175262
1,04	0,64	0,73	ENSG00000120948
0,72	0,03	0,05	ENSG00000009724
0,96	0,57	0,67	ENSG00000116649
0,82	0,02	0,03	ENSG00000171824
1,14	0,10	0,17	ENSG00000198793

5,28	0,00	0,00	ENSG00000171819
1,00	0,96	0,98	ENSG00000120942
1,66	0,29	0,40	ENSG00000204624
1,57	0,00	0,00	ENSG00000116661
0,95	0,55	0,66	ENSG00000132879
0,89	0,17	0,26	ENSG00000116670
0,89	0,24	0,35	ENSG00000116663
0,37	0,00	0,00	ENSG00000162490
0,69	0,00	0,00	ENSG00000177674
22,89	0,26	0,37	ENSG00000215910
1,01	0,94	0,98	ENSG00000177000
2,67	0,00	0,00	ENSG00000011021
1,06	0,85	0,92	ENSG00000175206
1,14	0,97	0,98	ENSG00000120937
1,41	0,00	0,00	ENSG00000116685
1,45	0,00	0,00	ENSG00000083444
1,02	0,84	0,91	ENSG00000116688
0,77	0,00	0,01	ENSG00000116691
0,14	0,00	0,00	ENSG00000120949
0,48	0,00	0,00	ENSG00000028137
1,14	0,10	0,17	ENSG00000048707
0,47	0,00	0,00	ENSG00000162496
0,13	0,46	#N/D	ENSG00000204518
#N/D	#N/D	#N/D	ENSG00000188984
1,14	0,97	#N/D	ENSG00000157330
22,89	0,26	0,37	ENSG00000116726
#N/D	#N/D	#N/D	ENSG00000116721
#N/D	#N/D	#N/D	ENSG00000239810
1,14	0,97	#N/D	ENSG00000179172
#N/D	#N/D	#N/D	ENSG00000120952
#N/D	#N/D	#N/D	ENSG00000243073
#N/D	#N/D	#N/D	ENSG00000187545
#N/D	#N/D	#N/D	ENSG00000204510
#N/D	#N/D	#N/D	ENSG00000232423
#N/D	#N/D	#N/D	ENSG00000274764
#N/D	#N/D	#N/D	ENSG00000277058
1,14	0,97	#N/D	ENSG00000229571
#N/D	#N/D	#N/D	ENSG00000275774
#N/D	#N/D	#N/D	ENSG00000280267
#N/D	#N/D	#N/D	ENSG00000179412
#N/D	#N/D	#N/D	ENSG00000204505
#N/D	#N/D	#N/D	ENSG00000279169
#N/D	#N/D	#N/D	ENSG00000279804
#N/D	#N/D	#N/D	ENSG00000270601
#N/D	#N/D	#N/D	ENSG00000182330
#N/D	#N/D	#N/D	ENSG00000237700
#N/D	#N/D	#N/D	ENSG00000204501
#N/D	#N/D	#N/D	ENSG00000204481

#N/D	#N/D	#N/D	ENSG00000204480
#N/D	#N/D	#N/D	ENSG00000204479
1,14	0,97	#N/D	ENSG00000204478
0,01	0,04	0,07	ENSG00000162494
0,54	0,00	0,00	ENSG00000162493
1,08	0,37	0,48	ENSG00000116731
0,30	0,00	0,00	ENSG00000189337
0,39	0,00	0,00	ENSG00000171729
0,62	0,03	0,06	ENSG00000142621
0,82	0,01	0,02	ENSG00000142634
1,81	0,18	0,27	ENSG00000162438
37,39	0,19	0,29	ENSG00000142615
8,39	0,46	0,56	ENSG00000215704
0,90	0,25	0,35	ENSG00000132906
1,06	0,51	0,61	ENSG00000116138
0,29	0,00	0,00	ENSG00000116771
1,07	0,41	0,52	ENSG00000197312
0,65	0,00	0,00	ENSG00000215695
0,94	0,48	0,58	ENSG00000116786
0,26	0,00	0,00	ENSG00000162461
0,14	0,01	0,02	ENSG00000162460
0,66	0,00	0,00	ENSG00000162458
0,51	0,00	0,00	ENSG00000233954
0,84	0,03	0,05	ENSG00000065526
1,08	0,34	0,45	ENSG00000116809
0,99	1,00	#N/D	ENSG00000183888
8,39	0,46	0,56	ENSG00000173641
0,13	0,46	0,56	ENSG00000186510
1,92	0,23	0,34	ENSG00000184908
0,57	0,00	0,00	ENSG00000185519
0,94	0,42	0,54	ENSG00000142627
1,00	1,00	1,00	ENSG00000142632
0,53	0,00	0,00	ENSG00000132881
0,93	0,39	0,51	ENSG00000037637
1,54	0,00	0,00	ENSG00000055070
1,19	0,70	0,79	ENSG00000187144
1,47	0,00	0,00	ENSG00000157191
0,65	0,00	0,00	ENSG00000219481
0,70	0,00	0,00	ENSG00000058453
1,07	0,39	0,51	ENSG00000117122
0,90	0,20	0,30	ENSG00000159363
#N/D	#N/D	#N/D	ENSG00000288636
0,98	0,80	0,88	ENSG00000117118
1,80	0,08	0,14	ENSG00000117115
0,62	0,20	0,30	ENSG00000142623
1,27	0,00	0,01	ENSG00000142619
1,14	0,97	0,98	ENSG00000159339
8,39	0,46	#N/D	ENSG00000276747

0,86	0,07	0,12	ENSG00000179051
0,60	0,00	0,00	ENSG00000074964
0,98	0,99	0,99	ENSG00000117148
#N/D	#N/D	#N/D	ENSG00000117154
#N/D	#N/D	#N/D	ENSG00000280222
4,97	0,00	0,01	ENSG00000179023
#N/D	#N/D	#N/D	ENSG00000009709
15,64	0,33	0,44	ENSG00000179002
1,72	0,00	0,00	ENSG00000159423
0,87	0,10	0,16	ENSG00000169991
1,56	0,00	0,00	ENSG00000127481
0,84	0,03	0,05	ENSG00000127463
0,90	0,23	0,33	ENSG00000053372
0,63	0,09	0,16	ENSG00000211454
1,08	0,77	0,85	ENSG00000162482
1,27	0,00	0,01	ENSG00000053371
0,75	0,00	0,00	ENSG00000040487
1,18	0,03	0,06	ENSG00000077549
0,95	0,57	0,67	ENSG00000173436
0,83	0,02	0,05	ENSG00000158747
1,27	0,21	0,31	ENSG00000270136
0,34	0,00	0,00	ENSG00000158748
0,58	0,00	0,00	ENSG00000162542
1,14	0,97	#N/D	ENSG00000178828
1,41	0,00	0,00	ENSG00000169914
1,14	0,97	#N/D	ENSG00000188784
0,98	0,99	0,99	ENSG00000188257
0,24	0,00	0,00	ENSG00000127472
51,90	0,15	0,24	ENSG00000117215
15,64	0,33	#N/D	ENSG00000158786
30,14	0,22	0,32	ENSG00000187980
5,63	0,01	0,01	ENSG00000162543
8,39	0,46	0,56	ENSG00000158816
2,10	0,00	0,00	ENSG00000162545
0,99	0,91	0,97	ENSG00000090432
3,47	0,00	0,00	ENSG00000183114
0,25	0,00	0,00	ENSG00000158825
0,82	0,02	0,03	ENSG00000158828
1,06	0,47	0,57	ENSG00000244038
1,01	0,95	0,98	ENSG00000117245
0,67	0,00	0,00	ENSG00000189410
1,33	0,00	0,00	ENSG00000127483
0,88	0,11	0,18	ENSG00000075151
0,73	0,00	0,00	ENSG00000117298
0,98	0,79	0,86	ENSG00000142794
0,13	0,00	0,00	ENSG00000162551
#N/D	#N/D	#N/D	ENSG00000289715
0,59	0,00	0,00	ENSG00000076864

1,14	0,12	0,19	ENSG00000090686
1,15	0,22	0,32	ENSG00000187942
1,14	0,10	0,17	ENSG00000142798
8,39	0,46	0,56	ENSG00000219073
0,13	0,46	#N/D	ENSG00000142789
0,95	0,53	0,63	ENSG00000289694
0,87	0,09	0,16	ENSG00000070831
0,64	0,00	0,00	ENSG00000162552
#N/D	#N/D	#N/D	ENSG00000279625
1,06	0,49	0,59	ENSG00000184677
0,67	0,65	0,74	ENSG00000070886
#N/D	#N/D	#N/D	ENSG00000173372
#N/D	#N/D	#N/D	ENSG00000289692
#N/D	#N/D	#N/D	ENSG00000159189
#N/D	#N/D	#N/D	ENSG00000173369
0,89	0,16	0,25	ENSG00000133216
#N/D	#N/D	#N/D	ENSG00000215906
1,21	0,77	0,85	ENSG00000227868
0,68	0,00	0,00	ENSG00000004487
1,07	0,41	0,52	ENSG00000169641
#N/D	#N/D	#N/D	ENSG00000293565
0,17	0,00	0,00	ENSG00000179546
0,72	0,00	0,00	ENSG00000125944
0,85	0,06	0,11	ENSG00000125945
0,72	0,04	0,07	ENSG00000204219
0,87	0,08	0,14	ENSG00000088280
0,23	0,00	0,00	ENSG00000007968
2,25	0,00	0,00	ENSG00000117318
1,03	0,70	0,79	ENSG00000142676
0,95	0,53	0,64	ENSG00000011007
1,00	0,96	0,98	ENSG00000057757
1,57	0,00	0,00	ENSG00000011009
0,90	0,21	0,32	ENSG00000117308
1,43	0,00	0,00	ENSG00000117305
1,13	0,26	0,37	ENSG00000179163
0,50	0,25	0,36	ENSG00000188822
1,16	0,08	0,14	ENSG00000189266
0,66	0,00	0,00	ENSG00000188529
0,03	0,00	0,00	ENSG00000142661
0,58	0,00	0,00	ENSG00000142677
0,37	0,00	0,00	ENSG00000185436
0,30	0,01	0,01	ENSG00000158055
0,72	0,00	0,00	ENSG00000001460
1,16	0,06	0,11	ENSG00000001461
0,98	0,84	0,91	ENSG00000117602
0,99	1,00	1,00	ENSG00000184454
1,21	0,02	0,04	ENSG00000133226
0,70	0,00	0,00	ENSG00000169504

0,88	0,11	0,18	ENSG00000020633
0,74	0,00	0,01	ENSG00000117614
1,44	0,00	0,00	ENSG00000117616
0,84	0,38	0,49	ENSG00000187010
0,62	0,00	0,00	ENSG00000183726
3,22	0,00	0,00	ENSG00000188672
1,06	0,51	0,62	ENSG00000204178
0,77	0,00	0,01	ENSG00000157978
1,04	0,88	0,94	ENSG00000117643
0,73	0,00	0,00	ENSG00000162430
1,14	0,97	#N/D	ENSG00000255054
1,25	0,01	0,02	ENSG00000117640
0,58	0,00	0,00	ENSG00000127423
0,64	0,00	0,00	ENSG00000182749
0,84	0,03	0,06	ENSG00000117632
0,79	0,02	0,04	ENSG00000158006
0,57	0,24	0,35	ENSG00000158008
2,15	0,20	0,30	ENSG00000158014
1,14	0,97	0,98	ENSG00000158022
0,59	0,00	0,00	ENSG00000175087
2,05	0,10	0,16	ENSG00000197245
0,99	1,00	1,00	ENSG00000282872
0,89	0,43	0,54	ENSG00000236782
1,17	0,10	0,17	ENSG00000142684
0,48	0,00	0,00	ENSG00000142675
0,07	0,31	0,43	ENSG00000188782
1,26	0,00	0,01	ENSG00000130695
1,38	0,00	0,00	ENSG00000142669
0,88	0,20	0,30	ENSG00000158062
8,39	0,46	0,56	ENSG00000169442
0,14	0,05	0,09	ENSG00000176092
#N/D	#N/D	#N/D	ENSG00000176083
0,97	0,95	0,98	ENSG00000131914
2,15	0,00	0,00	ENSG00000117682
1,07	0,43	0,55	ENSG00000198830
1,17	0,06	0,11	ENSG00000117676
0,82	0,01	0,03	ENSG00000117713
0,63	0,00	0,00	ENSG00000060642
0,85	0,06	0,10	ENSG00000204160
0,23	0,00	0,00	ENSG00000175793
1,21	0,02	0,04	ENSG00000142751
0,80	0,02	0,05	ENSG00000198746
0,82	0,01	0,03	ENSG00000090273
1,14	0,97	#N/D	ENSG00000131910
0,62	0,39	0,51	ENSG00000175707
0,33	0,00	0,00	ENSG00000253368
0,54	0,00	0,00	ENSG00000158246
1,07	0,40	0,51	ENSG00000090020

1,16	0,08	0,13	ENSG00000142784
1,44	0,00	0,00	ENSG00000186501
0,71	0,22	0,32	ENSG00000142765
1,37	0,00	0,00	ENSG00000142733
0,42	0,08	0,14	ENSG00000142748
1,14	0,97	#N/D	ENSG00000174950
1,48	0,00	0,00	ENSG00000181773
1,41	0,00	0,00	ENSG00000158195
1,66	0,00	0,00	ENSG00000126705
0,79	0,72	0,80	ENSG00000000938
2,63	0,00	0,00	ENSG00000126709
1,10	0,32	0,43	ENSG00000009780
1,38	0,00	0,00	ENSG00000117758
0,58	0,00	0,00	ENSG00000117751
1,51	0,00	0,00	ENSG00000130775
0,78	0,00	0,01	ENSG00000117748
0,07	0,00	0,00	ENSG00000130768
0,63	0,00	0,00	ENSG00000158156
1,09	0,30	0,41	ENSG00000158161
0,53	0,18	0,27	ENSG00000169403
1,02	0,83	0,90	ENSG00000126698
0,68	0,00	0,00	ENSG00000130770
1,17	0,06	0,11	ENSG00000130766
0,74	0,00	0,00	ENSG00000130772
0,64	0,00	0,00	ENSG00000204138
0,56	0,00	0,00	ENSG00000180198
0,56	0,00	0,01	ENSG00000279443
1,07	0,53	0,63	ENSG00000180098
1,03	0,76	0,84	ENSG00000120656
0,29	0,00	0,00	ENSG00000188060
1,13	0,17	0,26	ENSG00000162419
0,85	0,04	0,08	ENSG00000198492
1,60	0,51	0,62	ENSG00000116329
0,57	0,00	0,00	ENSG00000159023
0,97	0,70	0,79	ENSG00000253304
1,00	0,95	0,98	ENSG00000116350
0,86	0,10	0,17	ENSG00000116353
1,80	0,00	0,00	ENSG00000060656
#N/D	#N/D	#N/D	ENSG00000289710
0,92	0,66	0,75	ENSG00000162510
2,28	0,00	0,00	ENSG00000162511
1,87	0,00	0,00	ENSG00000162512
0,94	0,44	0,56	ENSG00000134644
0,42	0,00	0,00	ENSG00000084628
0,87	0,08	0,14	ENSG00000060688
1,35	0,00	0,00	ENSG00000121766
1,65	0,00	0,00	ENSG00000121769
0,57	0,00	0,00	ENSG00000168528

0,13	0,00	0,00	ENSG00000142910
0,36	0,00	0,00	ENSG00000121764
1,08	0,35	0,46	ENSG00000162517
0,51	0,00	0,00	ENSG00000084636
0,66	0,00	0,00	ENSG00000121753
0,55	0,00	0,00	ENSG00000134668
0,77	0,00	0,00	ENSG00000184007
#N/D	#N/D	#N/D	ENSG00000288678
0,92	0,29	0,40	ENSG00000121774
0,89	0,18	0,27	ENSG00000121775
1,12	0,15	0,24	ENSG00000025800
1,21	0,02	0,04	ENSG00000084652
0,65	0,00	0,00	ENSG00000160050
0,94	0,57	0,67	ENSG00000160051
0,70	0,06	0,11	ENSG00000222046
0,84	0,07	0,12	ENSG00000160055
1,17	0,05	0,10	ENSG00000084623
1,01	0,91	0,97	ENSG00000183615
0,13	0,46	0,56	ENSG00000182866
0,82	0,01	0,03	ENSG00000116478
1,36	0,00	0,00	ENSG00000175130
1,83	0,00	0,00	ENSG00000162526
1,90	0,00	0,00	ENSG00000225828
1,45	0,00	0,00	ENSG00000160058
0,07	0,31	0,43	ENSG00000254553
6,99	0,05	0,10	ENSG00000273274
1,17	0,10	0,16	ENSG00000160062
1,06	0,57	0,67	ENSG00000176261
1,19	0,03	0,05	ENSG00000162521
0,63	0,00	0,00	ENSG00000162520
0,68	0,00	0,00	ENSG00000162522
0,69	0,00	0,00	ENSG00000134684
0,87	0,10	0,17	ENSG00000116497
0,70	0,07	0,12	ENSG00000160097
0,84	0,59	0,69	ENSG00000121905
0,75	0,00	0,00	ENSG00000121900
0,91	0,26	0,37	ENSG00000116514
0,23	0,05	0,10	ENSG00000278966
1,17	0,05	0,09	ENSG00000004455
1,68	0,00	0,00	ENSG00000142920
1,03	0,90	0,96	ENSG00000278997
1,01	0,96	0,98	ENSG00000116525
0,68	0,07	0,13	ENSG00000279179
0,58	0,00	0,00	ENSG00000160094
1,14	0,97	0,98	ENSG00000184389
1,27	0,00	0,01	ENSG00000134686
1,18	0,05	0,10	ENSG00000121903
30,41	0,00	0,00	ENSG00000121904

8,39	0,46	#N/D	ENSG00000176256
8,39	0,46	#N/D	ENSG00000142698
0,87	0,10	0,17	ENSG00000163866
2,07	0,12	0,19	ENSG00000189280
6,99	0,05	0,10	ENSG00000189433
6,15	0,00	0,00	ENSG00000188910
1,80	0,08	0,14	ENSG00000187513
0,57	0,36	0,47	ENSG00000116544
1,22	0,16	0,25	ENSG00000284773
0,89	0,29	0,40	ENSG00000243749
1,06	0,52	0,62	ENSG00000271741
0,84	0,08	0,14	ENSG00000163867
0,54	0,00	0,00	ENSG00000197056
0,60	0,00	0,00	ENSG00000116560
0,75	0,00	0,00	ENSG00000146463
0,86	0,06	0,11	ENSG00000142687
2,15	0,00	0,00	ENSG00000020129
1,82	0,00	0,01	ENSG00000116819
0,81	0,01	0,02	ENSG00000126067
0,83	0,04	0,07	ENSG00000142686
0,85	0,06	0,11	ENSG00000092853
0,76	0,00	0,01	ENSG00000134698
1,02	0,84	0,91	ENSG00000092847
1,02	0,84	0,91	ENSG00000126070
0,11	0,03	0,05	ENSG00000092850
1,26	0,01	0,02	ENSG00000116863
0,25	0,00	0,00	ENSG00000171812
1,12	0,18	0,28	ENSG00000054116
1,06	0,43	0,55	ENSG00000116871
0,98	0,75	0,83	ENSG00000054118
0,80	0,02	0,03	ENSG00000214193
2,80	0,00	0,00	ENSG00000142694
1,17	0,06	0,11	ENSG00000196182
1,13	0,17	0,26	ENSG00000181817
1,57	0,00	0,00	ENSG00000116885
0,92	0,31	0,42	ENSG00000116898
0,53	0,58	0,68	ENSG00000119535
1,14	0,97	#N/D	ENSG00000163873
0,41	0,00	0,00	ENSG00000163874
0,97	0,73	0,81	ENSG00000163875
1,56	0,00	0,00	ENSG00000163877
1,86	0,00	0,01	ENSG00000163879
1,65	0,00	0,00	ENSG00000134697
3,56	0,23	0,34	ENSG00000169218
1,19	0,04	0,07	ENSG00000116922
0,88	0,13	0,21	ENSG00000134690
0,74	0,69	0,78	ENSG00000183317
0,79	0,01	0,02	ENSG00000185090

1,24	0,01	0,02	ENSG00000196449
1,09	0,31	0,42	ENSG00000197982
1,36	0,00	0,00	ENSG00000188786
0,64	0,00	0,00	ENSG00000204084
1,05	0,57	0,68	ENSG00000183431
1,13	0,17	0,26	ENSG00000183386
0,95	0,52	0,62	ENSG00000183520
0,60	0,47	0,58	ENSG00000185668
0,82	0,03	0,06	ENSG00000116954
0,88	0,18	0,28	ENSG00000214114
0,70	0,55	0,65	ENSG00000274944
0,27	0,00	0,00	ENSG00000131233
1,25	0,13	0,21	ENSG00000158315
0,94	0,43	0,55	ENSG00000174574
0,90	0,24	0,35	ENSG00000168653
0,93	0,34	0,45	ENSG00000127603
0,48	0,00	0,00	ENSG00000183682
1,07	0,39	0,50	ENSG00000090621
1,63	0,00	0,01	ENSG00000163909
3,19	0,01	0,03	ENSG00000116981
0,78	0,61	0,70	ENSG00000116983
1,24	0,01	0,02	ENSG00000084072
1,01	0,94	0,98	ENSG00000116985
1,08	0,79	0,86	ENSG00000198754
0,74	0,00	0,01	ENSG00000043514
0,14	0,00	0,00	ENSG00000116990
2,07	0,00	0,00	ENSG00000168389
1,20	0,02	0,04	ENSG00000131236
0,85	0,04	0,08	ENSG00000131238
1,10	0,25	0,36	ENSG00000117000
8,39	0,46	#N/D	ENSG00000188800
0,87	0,10	0,17	ENSG00000084073
1,29	0,74	0,82	ENSG00000049089
2,04	0,00	0,00	ENSG00000084070
0,60	0,00	0,00	ENSG00000187801
0,66	0,51	0,62	ENSG00000279667
0,38	0,00	0,00	ENSG00000187815
0,44	0,00	0,00	ENSG00000164002
1,27	0,13	0,21	ENSG00000117010
1,29	0,00	0,01	ENSG00000117016
0,79	0,00	0,01	ENSG00000066136
1,41	0,39	0,50	ENSG00000117013
0,51	0,00	0,00	ENSG00000179862
0,87	0,08	0,14	ENSG00000171793
0,88	0,18	0,27	ENSG00000171790
0,66	0,00	0,00	ENSG00000010803
1,31	0,15	0,24	ENSG00000204060
0,82	0,74	0,82	ENSG00000127129

0,48	0,00	0,00	ENSG00000127124
#N/D	#N/D	#N/D	ENSG00000284895
#N/D	#N/D	#N/D	ENSG00000044012
#N/D	#N/D	#N/D	ENSG00000197273
0,86	0,06	0,10	ENSG00000198815
0,02	0,12	0,19	ENSG00000177181
0,98	0,97	0,99	ENSG00000066185
0,51	0,00	0,00	ENSG00000127125
0,43	0,00	0,00	ENSG00000186409
0,91	0,27	0,38	ENSG00000171960
0,87	0,08	0,15	ENSG00000065978
0,98	0,97	0,98	ENSG00000164007
1,58	0,00	0,00	ENSG00000117385
1,45	0,00	0,00	ENSG00000164008
0,83	0,35	0,46	ENSG00000283580
3,64	0,00	0,00	ENSG00000274386
0,80	0,12	0,20	ENSG00000177868
0,70	0,00	0,00	ENSG00000164010
0,61	0,00	0,00	ENSG00000164011
1,21	0,02	0,04	ENSG00000117394
1,14	0,97	0,98	ENSG00000186973
0,90	0,19	0,29	ENSG00000117395
0,74	0,30	0,42	ENSG00000243710
30,14	0,22	0,32	ENSG00000179178
1,85	0,60	0,69	ENSG00000253313
0,92	0,74	0,82	ENSG00000066056
0,49	0,10	0,17	ENSG00000117400
1,09	0,29	0,40	ENSG00000117399
1,16	0,06	0,11	ENSG00000066322
1,62	0,00	0,00	ENSG00000159479
1,05	0,51	0,61	ENSG00000198198
0,42	0,00	0,00	ENSG00000178922
0,31	0,00	0,00	ENSG00000142949
0,91	0,28	0,39	ENSG00000066135
1,14	0,97	0,98	ENSG00000284989
0,83	0,03	0,05	ENSG00000126091
0,49	0,00	0,00	ENSG00000117407
1,08	0,34	0,45	ENSG00000117408
1,80	0,00	0,00	ENSG00000132768
1,44	0,00	0,00	ENSG00000117410
1,22	0,01	0,03	ENSG00000117411
1,09	0,39	0,51	ENSG00000159214
0,51	0,00	0,00	ENSG00000196517
0,91	0,53	0,63	ENSG00000171872
0,98	0,97	0,98	ENSG00000283039
0,84	0,06	0,12	ENSG00000178028
0,95	0,53	0,63	ENSG00000117419
0,67	0,00	0,00	ENSG00000187147

0,91	0,35	0,46	ENSG00000126106
2,15	0,20	0,30	ENSG00000198520
1,03	0,75	0,83	ENSG00000142945
0,96	0,64	0,73	ENSG00000142937
1,61	0,36	0,47	ENSG00000142959
1,32	0,00	0,01	ENSG00000173846
1,24	0,22	0,32	ENSG00000188396
0,90	0,25	0,36	ENSG00000222009
0,60	0,00	0,01	ENSG00000117425
1,02	0,83	0,90	ENSG00000070785
1,53	0,00	0,00	ENSG00000126107
0,98	0,80	0,87	ENSG00000126088
3,88	0,00	0,00	ENSG00000162415
0,43	0,00	0,00	ENSG00000186603
0,39	0,00	0,00	ENSG00000132781
1,14	0,97	0,98	ENSG00000288208
0,56	0,00	0,00	ENSG00000132773
1,40	0,00	0,01	ENSG00000070759
1,09	0,49	0,59	ENSG00000280670
0,83	0,04	0,07	ENSG00000132763
1,39	0,00	0,00	ENSG00000117450
1,42	0,00	0,00	ENSG00000117448
0,82	0,02	0,03	ENSG00000132780
0,43	0,00	0,00	ENSG00000159588
0,97	0,67	0,76	ENSG00000159592
0,79	0,01	0,02	ENSG00000159596
0,84	0,08	0,14	ENSG00000197429
0,90	0,21	0,31	ENSG00000086015
0,89	0,39	0,51	ENSG00000117461
1,14	0,97	0,98	ENSG00000278139
0,80	0,35	0,46	ENSG00000117472
#N/D	#N/D	#N/D	ENSG00000250719
1,17	0,05	0,10	ENSG00000085998
2,84	0,11	0,19	ENSG00000171357
1,31	0,00	0,00	ENSG00000085999
0,91	0,25	0,36	ENSG00000132128
1,07	0,40	0,52	ENSG00000173660
1,00	0,96	0,98	ENSG00000117481
0,80	0,23	0,34	ENSG00000117480
1,98	0,00	0,00	ENSG00000197587
1,85	0,60	0,69	ENSG00000282881
0,13	0,46	#N/D	ENSG00000162456
0,81	0,04	0,07	ENSG00000079277
1,14	0,14	0,23	ENSG00000142961
0,80	0,01	0,02	ENSG00000123472
1,25	0,54	0,64	ENSG00000186118
1,47	0,00	0,00	ENSG00000159658
#N/D	#N/D	#N/D	ENSG00000142973

#N/D	#N/D	#N/D	ENSG00000187048
1,85	0,60	0,69	ENSG00000186377
1,14	0,97	#N/D	ENSG00000186160
0,03	0,17	0,26	ENSG00000162365
0,16	0,00	0,00	ENSG00000162366
8,39	0,46	0,56	ENSG00000162367
1,10	0,26	0,37	ENSG00000123473
0,81	0,01	0,03	ENSG00000162368
4,42	0,15	0,24	ENSG00000186790
0,70	0,02	0,04	ENSG00000186564
2,70	0,00	0,00	ENSG00000269113
1,14	0,97	0,98	ENSG00000279096
66,40	0,13	0,21	ENSG00000117834
#N/D	#N/D	#N/D	ENSG00000279214
5,37	0,00	0,00	ENSG00000132122
1,14	0,97	#N/D	ENSG00000186094
#N/D	#N/D	#N/D	ENSG00000162373
#N/D	#N/D	#N/D	ENSG00000279324
1,14	0,97	#N/D	ENSG00000162374
0,03	0,17	0,26	ENSG00000142700
0,84	0,03	0,06	ENSG00000185104
1,24	0,01	0,03	ENSG00000123080
1,14	0,97	#N/D	ENSG00000204006
1,14	0,11	0,19	ENSG00000123091
0,24	0,00	0,00	ENSG00000085831
0,68	0,00	0,00	ENSG00000085832
0,57	0,00	0,00	ENSG00000117859
0,79	0,00	0,01	ENSG00000078618
1,08	0,39	0,50	ENSG00000169213
0,67	0,00	0,00	ENSG00000117862
0,01	0,06	0,11	ENSG00000285839
0,82	0,04	0,07	ENSG00000198841
0,49	0,00	0,00	ENSG00000134717
0,77	0,00	0,01	ENSG00000157077
0,99	0,90	0,97	ENSG00000154222
1,16	0,08	0,14	ENSG00000085840
1,47	0,00	0,00	ENSG00000134748
0,67	0,00	0,00	ENSG00000134744
1,23	0,03	0,06	ENSG00000116157
1,14	0,97	#N/D	ENSG00000182183
0,70	0,00	0,00	ENSG00000162377
1,01	0,87	0,94	ENSG00000162378
0,72	0,00	0,00	ENSG00000203995
0,81	0,02	0,05	ENSG00000121310
0,92	0,34	0,45	ENSG00000116171
0,98	0,99	0,99	ENSG00000174348
0,31	0,04	0,07	ENSG00000162383
0,56	0,00	0,00	ENSG00000157184

0,98	0,83	0,90	ENSG00000162384
0,75	0,01	0,02	ENSG00000162385
1,59	0,00	0,00	ENSG00000157193
#N/D	#N/D	#N/D	ENSG00000293253
1,14	0,97	#N/D	ENSG00000143006
1,00	0,97	0,99	ENSG00000174332
0,75	0,00	0,00	ENSG00000058804
0,89	0,22	0,32	ENSG00000058799
1,44	0,06	0,11	ENSG00000211452
0,73	0,00	0,01	ENSG00000081870
1,12	0,17	0,26	ENSG00000116212
0,13	0,46	#N/D	ENSG00000203985
1,15	0,09	0,15	ENSG00000116209
1,53	0,05	0,10	ENSG00000280378
1,16	0,09	0,16	ENSG00000116205
0,76	0,59	0,69	ENSG00000256407
7,85	0,04	0,07	ENSG00000157211
1,15	0,14	0,22	ENSG00000215883
1,15	0,07	0,13	ENSG00000116221
1,61	0,00	0,00	ENSG00000157216
0,46	0,00	0,00	ENSG00000162390
0,51	0,42	0,54	ENSG00000162391
7,85	0,04	0,07	ENSG00000184313
2,87	0,04	0,08	ENSG00000271723
0,57	0,00	0,00	ENSG00000243725
0,68	0,00	0,00	ENSG00000162396
1,14	0,97	0,98	ENSG00000006555
5,99	0,02	0,03	ENSG00000162398
1,30	0,00	0,00	ENSG00000116133
0,05	0,24	0,35	ENSG00000143001
8,39	0,46	0,56	ENSG00000162399
1,85	0,60	0,69	ENSG00000169174
0,73	0,00	0,00	ENSG00000162402
0,55	0,16	0,25	ENSG00000284686
0,58	0,00	0,00	ENSG00000162407
0,42	0,00	0,00	ENSG00000162409
1,14	0,97	0,98	ENSG00000187889
#N/D	#N/D	#N/D	ENSG00000157131
#N/D	#N/D	#N/D	ENSG00000021852
#N/D	#N/D	#N/D	ENSG00000173406
0,62	0,00	0,00	ENSG00000162600
0,54	0,22	0,32	ENSG00000184292
1,16	0,08	0,13	ENSG00000162601
1,12	0,15	0,23	ENSG00000177606
0,93	0,48	0,59	ENSG00000172456
6,99	0,05	0,10	ENSG00000134709
32,87	0,00	0,00	ENSG00000134716
1,14	0,97	#N/D	ENSG00000162598

0,32	0,00	0,00	ENSG00000162599
1,09	0,38	0,50	ENSG00000162604
0,95	0,54	0,64	ENSG00000132849
22,89	0,26	0,37	ENSG00000240563
8,39	0,46	0,56	ENSG00000132854
0,60	0,00	0,00	ENSG00000162607
0,59	0,00	0,00	ENSG00000116641
0,04	0,20	0,30	ENSG00000132855
0,23	0,05	0,10	ENSG00000278967
0,88	0,26	0,37	ENSG00000125703
0,24	0,00	0,00	ENSG00000187140
0,40	0,00	0,00	ENSG00000088035
0,98	0,89	0,95	ENSG00000142856
0,45	0,00	0,00	ENSG00000203965
0,81	0,01	0,02	ENSG00000079739
0,09	0,00	0,00	ENSG00000185483
#N/D	#N/D	#N/D	ENSG00000177414
1,64	0,00	0,00	ENSG00000158966
0,74	0,00	0,00	ENSG00000162437
0,81	0,01	0,02	ENSG00000162434
0,66	0,00	0,00	ENSG00000162433
0,25	0,00	0,00	ENSG00000116675
0,95	0,54	0,64	ENSG00000213625
0,97	0,71	0,79	ENSG00000116678
0,88	0,13	0,21	ENSG00000184588
0,98	0,98	0,99	ENSG00000118473
1,14	0,97	#N/D	ENSG00000152760
#N/D	#N/D	#N/D	ENSG00000172410
0,92	0,81	0,88	ENSG00000152763
0,82	0,03	0,07	ENSG00000198160
1,16	0,07	0,13	ENSG00000116704
#N/D	#N/D	#N/D	ENSG00000203963
1,14	0,97	#N/D	ENSG00000162594
0,81	0,30	0,42	ENSG00000081985
0,67	0,00	0,00	ENSG00000142864
0,42	0,00	0,00	ENSG00000116717
0,97	0,73	0,81	ENSG00000172380
0,72	0,41	0,52	ENSG00000162595
1,19	0,03	0,06	ENSG00000116729
7,85	0,04	0,07	ENSG00000116745
0,58	0,00	0,00	ENSG00000024526
0,63	0,24	0,35	ENSG00000033122
0,48	0,00	0,00	ENSG00000066557
0,90	0,21	0,31	ENSG00000116754
0,88	0,14	0,23	ENSG00000118454
1,31	0,15	0,23	ENSG00000116761
356,44	0,03	0,06	ENSG00000050628
1,34	0,00	0,00	ENSG00000132485

1,16	0,41	0,52	ENSG00000172260
#N/D	#N/D	#N/D	ENSG00000280317
1,37	0,21	0,32	ENSG00000162620
4,41	0,00	0,00	ENSG00000259030
0,85	0,17	0,26	ENSG00000254685
102,65	0,09	0,16	ENSG00000116783
8,39	0,46	#N/D	ENSG00000162621
15,64	0,33	0,44	ENSG00000178965
1,12	0,21	0,31	ENSG00000116791
1,04	0,65	0,74	ENSG00000162623
38,82	0,00	0,00	ENSG00000162624
22,42	0,00	0,00	ENSG00000137968
0,84	0,04	0,08	ENSG00000117054
0,66	0,00	0,00	ENSG00000137955
0,22	0,01	0,01	ENSG00000057468
#N/D	#N/D	#N/D	ENSG00000154007
3,72	0,00	0,00	ENSG00000184005
1,14	0,97	0,98	ENSG00000117069
0,59	0,00	0,00	ENSG00000142892
1,35	0,00	0,00	ENSG00000154027
0,70	0,00	0,00	ENSG00000036549
0,70	0,00	0,00	ENSG00000077254
1,21	0,02	0,05	ENSG00000180488
0,67	0,01	0,01	ENSG00000162614
1,14	0,11	0,19	ENSG00000162613
1,04	0,72	0,81	ENSG00000162616
0,04	0,20	0,30	ENSG00000137960
3,74	0,00	0,00	ENSG00000122420
3,92	0,00	0,00	ENSG00000137959
1,48	0,00	0,00	ENSG00000137965
1,81	0,00	0,00	ENSG00000162618
0,33	0,00	0,00	ENSG00000117114
0,51	0,00	0,00	ENSG00000137941
0,91	0,31	0,42	ENSG00000142875
0,28	0,00	0,00	ENSG00000203943
0,04	0,20	0,30	ENSG00000137976
0,62	0,00	0,00	ENSG00000117133
0,98	0,77	0,85	ENSG00000174021
0,49	0,01	0,03	ENSG00000122432
1,24	0,02	0,03	ENSG00000117151
1,30	0,00	0,01	ENSG00000117155
2,95	0,00	0,00	ENSG00000171517
1,29	0,02	0,03	ENSG00000153898
2,39	0,01	0,01	ENSG00000162643
0,76	0,02	0,03	ENSG00000055732
0,95	0,77	0,85	ENSG00000280099
0,76	0,01	0,02	ENSG00000097096
1,14	0,17	0,26	ENSG00000162642

1,30	0,00	0,01	ENSG00000142867
0,58	0,00	0,00	ENSG00000153904
1,33	0,00	0,00	ENSG00000142871
0,91	0,30	0,42	ENSG00000117174
0,69	0,26	0,36	ENSG00000171502
1,08	0,46	0,56	ENSG00000122417
0,66	0,51	0,62	ENSG00000137975
#N/D	#N/D	#N/D	ENSG00000016490
#N/D	#N/D	#N/D	ENSG00000016602
1,25	0,01	0,02	ENSG00000097033
0,91	0,27	0,37	ENSG00000183291
0,84	0,04	0,08	ENSG00000153936
0,88	0,78	0,86	ENSG00000267561
1,07	0,42	0,54	ENSG00000143013
1,07	0,59	0,69	ENSG00000279778
0,80	0,01	0,01	ENSG00000065243
1,23	0,03	0,05	ENSG00000137947
0,70	0,00	0,00	ENSG00000137944
1,15	0,14	0,23	ENSG00000213516
1,66	0,00	0,00	ENSG00000117226
0,97	0,70	0,79	ENSG00000117228
2,52	0,00	0,00	ENSG00000162645
1,14	0,97	#N/D	ENSG00000213512
4,88	0,00	0,00	ENSG00000162654
3,21	0,00	0,00	ENSG00000154451
8,39	0,46	0,56	ENSG00000183347
0,29	0,00	0,00	ENSG00000197147
#N/D	#N/D	#N/D	ENSG00000288629
1,07	0,46	0,57	ENSG00000171488
0,05	0,24	0,35	ENSG00000271949
0,34	0,00	0,00	ENSG00000171492
0,49	0,00	0,00	ENSG00000162664
0,29	0,12	0,19	ENSG00000143032
0,71	0,00	0,00	ENSG00000122482
8,39	0,46	0,56	ENSG00000162669
0,68	0,00	0,00	ENSG00000097046
0,52	0,00	0,00	ENSG00000069702
1,14	0,97	0,98	ENSG00000137948
102,65	0,09	0,16	ENSG00000172031
#N/D	#N/D	#N/D	ENSG00000230667
0,61	0,00	0,00	ENSG00000189195
1,14	0,97	#N/D	ENSG00000203910
0,36	0,00	0,00	ENSG00000174842
0,46	0,00	0,00	ENSG00000122484
0,06	0,00	0,00	ENSG00000162676
0,78	0,00	0,01	ENSG00000067208
0,99	0,91	0,97	ENSG00000122406
0,78	0,01	0,02	ENSG00000154511

0,70	0,00	0,00	ENSG00000143033
0,71	0,00	0,00	ENSG00000117500
1,09	0,36	0,47	ENSG00000122483
0,82	0,02	0,03	ENSG00000117505
1,05	0,56	0,66	ENSG00000137942
1,05	0,56	0,66	ENSG00000137936
0,74	0,00	0,00	ENSG00000067334
0,60	0,00	0,00	ENSG00000023909
0,13	0,00	0,00	ENSG00000198691
1,42	0,00	0,00	ENSG00000137962
0,73	0,00	0,00	ENSG00000117528
1,20	0,08	0,14	ENSG00000117525
0,29	0,00	0,01	ENSG00000143036
1,11	0,22	0,32	ENSG00000117519
0,86	0,15	0,23	ENSG00000172339
0,04	0,00	0,00	ENSG00000152078
0,27	0,00	0,00	ENSG00000271092
0,70	0,02	0,04	ENSG00000122481
0,69	0,00	0,00	ENSG00000117569
1,92	0,00	0,00	ENSG00000188641
0,05	0,24	0,35	ENSG00000280040
0,94	0,48	0,58	ENSG00000162627
0,07	0,00	0,01	ENSG00000117598
0,23	0,00	0,00	ENSG00000117600
0,02	0,00	0,00	ENSG00000099260
0,80	0,02	0,04	ENSG00000156869
0,66	0,00	0,00	ENSG00000162688
1,19	0,07	0,12	ENSG00000117620
0,96	0,70	0,79	ENSG00000283761
1,10	0,28	0,39	ENSG00000156875
0,60	0,00	0,00	ENSG00000156876
0,49	0,00	0,00	ENSG00000122435
0,78	0,61	0,70	ENSG00000122477
0,74	0,00	0,00	ENSG00000137992
0,73	0,00	0,00	ENSG00000137996
0,33	0,00	0,00	ENSG00000079335
0,53	0,58	0,68	ENSG00000181656
1,14	0,97	0,98	ENSG00000162692
0,66	0,00	0,00	ENSG00000162694
1,06	0,51	0,61	ENSG00000162695
0,56	0,00	0,00	ENSG00000117543
0,05	0,00	0,00	ENSG00000170989
#N/D	#N/D	#N/D	ENSG00000118733
12,13	0,01	0,02	ENSG00000060718
1,84	0,00	0,00	ENSG00000185946
0,47	0,00	0,00	ENSG00000240038
#N/D	#N/D	#N/D	ENSG00000243480
#N/D	#N/D	#N/D	ENSG00000237763

1,14	0,97	#N/D	ENSG00000174876
#N/D	#N/D	#N/D	ENSG00000187733
0,26	0,00	0,00	ENSG00000198890
0,11	0,00	0,00	ENSG00000162631
0,81	0,63	0,73	ENSG00000134215
0,79	0,00	0,01	ENSG00000085491
0,20	0,00	0,00	ENSG00000196427
0,27	0,00	0,00	ENSG00000186086
0,91	0,25	0,36	ENSG00000162636
0,35	0,00	0,00	ENSG00000162639
0,82	0,02	0,04	ENSG00000134186
0,29	0,02	0,03	ENSG00000143107
0,58	0,00	0,00	ENSG00000116266
0,62	0,08	0,14	ENSG00000162641
1,04	0,60	0,69	ENSG00000121957
1,10	0,22	0,33	ENSG00000121940
1,11	0,22	0,32	ENSG00000085433
1,69	0,00	0,00	ENSG00000197780
1,45	0,00	0,00	ENSG00000215717
0,13	0,46	0,56	ENSG00000179902
0,36	0,00	0,00	ENSG00000116299
0,50	0,00	0,00	ENSG00000031698
0,44	0,00	0,00	ENSG00000143126
0,93	0,39	0,50	ENSG00000134222
1,14	0,97	0,98	ENSG00000221986
0,08	0,00	0,00	ENSG00000134243
0,89	0,17	0,26	ENSG00000143106
0,41	0,10	0,16	ENSG00000143028
0,63	0,00	0,00	ENSG00000162650
2,00	0,00	0,00	ENSG00000174151
1,49	0,13	0,21	ENSG00000181754
0,32	0,00	0,00	ENSG00000156097
1,22	0,01	0,03	ENSG00000065135
0,58	0,01	0,01	ENSG00000134183
2,63	0,00	0,00	ENSG00000116337
1,22	0,02	0,03	ENSG00000168765
2,64	0,00	0,00	ENSG00000213366
1,37	0,00	0,00	ENSG00000134184
4,30	0,00	0,00	ENSG00000134201
2,71	0,00	0,00	ENSG00000134202
0,20	0,00	0,01	ENSG00000198758
1,33	0,00	0,00	ENSG00000184371
1,16	0,06	0,11	ENSG00000168710
0,79	0,00	0,01	ENSG00000143093
0,86	0,07	0,13	ENSG00000156150
0,54	0,00	0,00	ENSG00000186150
0,49	0,00	0,00	ENSG00000197106
1,26	0,01	0,01	ENSG00000116396

1,77	0,00	0,00	ENSG00000162775
0,22	0,00	0,00	ENSG00000168679
1,06	0,48	0,58	ENSG00000134248
3,56	0,23	0,34	ENSG00000143125
#N/D	#N/D	#N/D	ENSG00000143105
1,14	0,97	0,98	ENSG00000177301
0,07	0,31	0,43	ENSG00000177272
22,89	0,26	0,37	ENSG00000143119
0,49	0,00	0,00	ENSG00000121931
1,02	0,81	0,88	ENSG00000156171
0,82	0,02	0,04	ENSG00000134255
3,50	0,00	0,00	ENSG00000162777
0,66	0,00	0,01	ENSG00000064886
#N/D	#N/D	#N/D	ENSG00000134216
1,85	0,60	0,69	ENSG00000173947
3,86	0,00	0,00	ENSG00000085465
0,73	0,00	0,00	ENSG00000116455
0,95	0,56	0,66	ENSG00000116459
0,24	0,00	0,00	ENSG00000143110
1,14	0,97	#N/D	ENSG00000121933
1,14	0,97	#N/D	ENSG00000282608
1,21	0,03	0,05	ENSG00000116473
1,00	0,99	0,99	ENSG00000197852
0,66	0,00	0,00	ENSG00000064703
1,64	0,17	0,26	ENSG00000171385
0,92	0,34	0,45	ENSG00000143079
0,47	0,00	0,00	ENSG00000134245
0,56	0,00	0,00	ENSG00000007341
1,00	0,97	0,99	ENSG00000116489
0,98	0,82	0,89	ENSG00000155363
1,55	0,00	0,00	ENSG00000155366
2,22	0,24	0,35	ENSG00000271810
1,70	0,00	0,00	ENSG00000155367
0,49	0,02	0,03	ENSG00000184599
1,05	0,58	0,68	ENSG00000155380
1,36	0,00	0,00	ENSG00000198799
1,97	0,00	0,00	ENSG00000081026
0,44	0,00	0,00	ENSG00000116793
1,17	0,07	0,12	ENSG00000081019
0,14	0,00	0,00	ENSG00000134242
3,25	0,02	0,04	ENSG00000188761
1,79	0,00	0,00	ENSG00000134262
1,18	0,05	0,09	ENSG00000118655
1,10	0,23	0,34	ENSG00000163349
0,21	0,00	0,00	ENSG00000116774
1,14	0,97	#N/D	ENSG00000134207
0,60	0,00	0,00	ENSG00000197323
0,73	0,00	0,00	ENSG00000116752

6,54	0,00	0,00	ENSG00000175984
#N/D	#N/D	#N/D	ENSG00000116748
1,17	0,06	0,10	ENSG00000213281
0,88	0,10	0,17	ENSG00000009307
1,03	0,76	0,84	ENSG00000052723
#N/D	#N/D	#N/D	ENSG00000198765
1,14	0,97	#N/D	ENSG00000134200
3,26	0,13	0,21	ENSG00000134198
0,18	0,00	0,00	ENSG00000134259
0,59	0,00	0,00	ENSG00000173218
0,07	0,31	0,43	ENSG00000118729
1,14	0,97	#N/D	ENSG00000177551
0,23	0,00	0,00	ENSG00000163393
0,13	0,00	0,00	ENSG00000173212
1,25	0,00	0,01	ENSG00000163399
0,46	0,00	0,00	ENSG00000116815
0,66	0,00	0,00	ENSG00000143061
8,39	0,46	#N/D	ENSG00000116824
0,64	0,00	0,00	ENSG00000134247
0,42	0,00	0,00	ENSG00000134256
0,83	0,02	0,04	ENSG00000116830
1,00	0,97	0,99	ENSG00000134253
0,07	0,31	0,43	ENSG00000134258
0,99	0,88	0,95	ENSG00000198162
0,50	0,32	0,43	ENSG00000279513
13,59	0,00	0,00	ENSG00000183508
1,25	0,01	0,02	ENSG00000196505
0,87	0,09	0,15	ENSG00000065183
1,77	0,26	0,37	ENSG00000155761
0,93	0,35	0,46	ENSG00000092607
0,92	0,34	0,45	ENSG00000116874
8,39	0,46	#N/D	ENSG00000116882
#N/D	#N/D	#N/D	ENSG00000203859
1,14	0,97	#N/D	ENSG00000203857
1,01	0,93	0,98	ENSG00000143067
0,41	0,00	0,00	ENSG00000092621
1,14	0,97	0,98	ENSG00000134240
0,07	0,31	#N/D	ENSG00000134193
#N/D	#N/D	#N/D	ENSG00000134249
0,83	0,02	0,04	ENSG00000134250
1,17	0,06	0,10	ENSG00000265808
1,00	0,99	1,00	ENSG00000270231
1,71	0,00	0,00	ENSG00000286106
0,81	0,02	0,04	ENSG00000273136
0,04	0,20	0,30	ENSG00000263353
0,78	0,05	0,09	ENSG00000281741
0,85	0,05	0,09	ENSG00000188610
1,18	0,04	0,07	ENSG00000171943

0,62	0,09	0,15	ENSG00000273213
1,24	0,01	0,03	ENSG00000263513
#N/D	#N/D	#N/D	ENSG00000271567
1,25	0,01	0,01	ENSG00000266338
1,14	0,97	#N/D	ENSG00000279782
1,38	0,00	0,00	ENSG00000196369
1,21	0,02	0,05	ENSG00000215784
1,53	0,00	0,00	ENSG00000281571
#N/D	#N/D	#N/D	ENSG00000289549
1,25	0,01	0,03	ENSG00000162825
0,08	0,00	0,00	ENSG00000286620
1,12	0,20	0,30	ENSG00000117262
1,31	0,18	0,27	ENSG00000174827
1,69	0,01	0,01	ENSG00000117281
1,79	0,00	0,00	ENSG00000265491
0,93	0,37	0,49	ENSG00000186141
1,59	0,00	0,00	ENSG00000186364
2,07	0,00	0,00	ENSG00000131788
30,14	0,22	0,32	ENSG00000198483
4,20	0,00	0,00	ENSG00000143127
1,65	0,00	0,00	ENSG00000131779
14,71	0,01	0,01	ENSG00000289565
1,17	0,05	0,10	ENSG00000265241
#N/D	#N/D	#N/D	ENSG00000280778
1,46	0,00	0,00	ENSG00000271601
0,74	0,05	0,08	ENSG00000272031
1,14	0,22	0,32	ENSG00000121851
67,31	-	-	ENSG00000265972
22,89	0,26	#N/D	ENSG00000168509
1,01	0,90	0,96	ENSG00000271425
1,15	0,10	0,17	ENSG00000264343
1,14	0,97	#N/D	ENSG00000270339
1,10	0,29	0,40	ENSG00000268043
1,47	0,00	0,00	ENSG00000131791
2,84	0,00	0,00	ENSG00000131781
1,30	0,00	0,00	ENSG00000131778
1,72	0,00	0,00	ENSG00000116128
#N/D	#N/D	#N/D	ENSG00000288626
1,30	0,00	0,01	ENSG00000162836
0,13	0,46	#N/D	ENSG00000265107
#N/D	#N/D	#N/D	ENSG00000121634
1,10	0,31	0,43	ENSG00000188092
1,12	0,20	0,30	ENSG00000263956
1,18	0,11	0,19	ENSG00000280649
0,13	0,46	0,56	ENSG00000236334
0,97	0,75	0,83	ENSG00000270629
1,20	0,03	0,06	ENSG00000286019
0,44	0,00	0,00	ENSG00000177144

1,69	0,00	0,00	ENSG00000178104
0,95	0,53	0,63	ENSG00000269713
1,17	0,07	0,12	ENSG00000286219
1,76	0,00	0,00	ENSG00000286185
1,12	0,17	0,27	ENSG00000271383
22,89	0,26	0,37	ENSG00000288867
1,14	0,97	0,98	ENSG00000150337
0,29	0,00	0,00	ENSG00000203814
0,24	0,00	0,00	ENSG00000183598
0,30	0,00	0,00	ENSG00000270882
0,01	0,00	0,00	ENSG00000203811
0,41	0,00	0,00	ENSG00000288825
0,79	0,01	0,02	ENSG00000288859
0,53	0,00	0,00	ENSG00000203852
0,26	0,00	0,00	ENSG00000270276
0,57	0,00	0,00	ENSG00000184678
0,60	0,00	0,00	ENSG00000184260
0,51	0,00	0,00	ENSG00000184270
0,81	0,04	0,08	ENSG00000178096
0,51	0,08	0,14	ENSG00000159164
1,30	0,00	0,00	ENSG00000143368
0,80	0,03	0,05	ENSG00000014914
1,46	0,00	0,00	ENSG00000264522
1,54	0,00	0,00	ENSG00000136631
0,40	0,00	0,00	ENSG00000023902
0,79	0,01	0,01	ENSG00000143401
2,32	0,00	0,00	ENSG00000118298
1,42	0,00	0,00	ENSG00000117362
1,67	0,00	0,00	ENSG00000118292
15,64	0,33	0,44	ENSG00000159208
1,46	0,00	0,00	ENSG00000266472
1,77	0,00	0,00	ENSG00000117360
0,90	0,23	0,33	ENSG00000163125
1,25	0,01	0,01	ENSG00000143374
1,56	0,00	0,00	ENSG00000143369
0,80	0,01	0,02	ENSG00000143382
1,33	0,00	0,00	ENSG00000143384
1,57	0,00	0,00	ENSG00000143420
2,51	0,00	0,00	ENSG00000143457
1,14	0,97	0,98	ENSG00000143452
0,55	0,00	0,00	ENSG00000163131
0,92	0,48	0,58	ENSG00000143387
1,37	0,00	0,00	ENSG00000143437
1,14	0,97	#N/D	ENSG00000283324
1,15	0,09	0,16	ENSG00000143379
1,44	0,00	0,00	ENSG00000143418
0,59	0,01	0,01	ENSG00000143412
0,64	0,00	0,00	ENSG00000143409

1,33	0,00	0,00	ENSG00000143363
1,77	0,00	0,00	ENSG00000163141
0,87	0,24	0,34	ENSG00000143443
1,39	0,00	0,00	ENSG00000197622
0,42	0,00	0,00	ENSG00000213190
0,57	0,00	0,00	ENSG00000143458
0,89	0,24	0,34	ENSG00000143434
1,00	0,99	0,99	ENSG00000163154
1,21	0,03	0,06	ENSG00000163156
1,75	0,00	0,00	ENSG00000163155
1,29	0,74	0,82	ENSG00000163157
1,52	0,00	0,00	ENSG00000163159
1,41	0,00	0,00	ENSG00000143398
1,11	0,21	0,31	ENSG00000159352
1,72	0,00	0,00	ENSG00000143373
1,65	0,00	0,00	ENSG00000143393
0,81	0,01	0,02	ENSG00000143390
44,64	0,17	0,26	ENSG00000143416
1,24	0,01	0,02	ENSG00000159377
1,17	0,05	0,09	ENSG00000143442
0,04	0,00	0,00	ENSG00000143375
0,77	0,00	0,01	ENSG00000143367
1,09	0,31	0,42	ENSG00000143376
1,03	0,89	0,96	ENSG00000159409
0,99	1,00	1,00	ENSG00000178796
0,93	0,37	0,49	ENSG00000143436
1,29	0,18	0,28	ENSG00000143450
1,30	0,00	0,01	ENSG00000182134
0,98	0,99	0,99	ENSG00000213171
0,89	0,51	0,62	ENSG00000143365
0,13	0,46	0,56	ENSG00000225556
0,07	0,31	0,43	ENSG00000196407
0,42	0,00	0,00	ENSG00000159445
2,45	0,00	0,00	ENSG00000197747
0,97	0,73	0,82	ENSG00000163191
0,13	0,46	#N/D	ENSG00000182898
22,89	0,26	0,37	ENSG00000159450
1,14	0,97	0,98	ENSG00000215853
0,09	0,01	0,03	ENSG00000197915
0,02	0,15	0,23	ENSG00000143631
1,14	0,97	0,98	ENSG00000143520
1,14	0,97	0,98	ENSG00000143536
1,14	0,97	0,98	ENSG00000186207
1,14	0,97	#N/D	ENSG00000169509
#N/D	#N/D	#N/D	ENSG00000185966
0,13	0,46	#N/D	ENSG00000163202
#N/D	#N/D	#N/D	ENSG00000244057
#N/D	#N/D	#N/D	ENSG00000187238

#N/D	#N/D	#N/D	ENSG00000185962
#N/D	#N/D	#N/D	ENSG00000187223
#N/D	#N/D	#N/D	ENSG00000187180
#N/D	#N/D	#N/D	ENSG00000159455
0,13	0,46	#N/D	ENSG00000187173
#N/D	#N/D	#N/D	ENSG00000187170
0,05	0,24	0,35	ENSG00000198854
0,02	0,12	0,19	ENSG00000203786
1,14	0,97	#N/D	ENSG00000240386
#N/D	#N/D	#N/D	ENSG00000186226
#N/D	#N/D	#N/D	ENSG00000172155
1,14	0,97	#N/D	ENSG00000197084
#N/D	#N/D	#N/D	ENSG00000196734
#N/D	#N/D	#N/D	ENSG00000186844
#N/D	#N/D	#N/D	ENSG00000235942
#N/D	#N/D	#N/D	ENSG00000285946
#N/D	#N/D	#N/D	ENSG00000163206
0,07	0,31	0,43	ENSG00000163207
#N/D	#N/D	#N/D	ENSG00000283227
#N/D	#N/D	#N/D	ENSG00000184148
#N/D	#N/D	#N/D	ENSG00000169474
#N/D	#N/D	#N/D	ENSG00000163209
#N/D	#N/D	#N/D	ENSG00000169469
1,14	0,97	#N/D	ENSG00000163216
#N/D	#N/D	#N/D	ENSG00000241794
#N/D	#N/D	#N/D	ENSG00000196805
0,13	0,46	#N/D	ENSG00000203785
1,14	0,97	#N/D	ENSG00000244094
#N/D	#N/D	#N/D	ENSG00000159516
#N/D	#N/D	#N/D	ENSG00000203784
#N/D	#N/D	#N/D	ENSG00000203783
#N/D	#N/D	#N/D	ENSG00000203782
1,14	0,97	0,98	ENSG00000159527
#N/D	#N/D	#N/D	ENSG00000163218
1,14	0,97	#N/D	ENSG00000163220
#N/D	#N/D	#N/D	ENSG00000163221
1,14	0,97	#N/D	ENSG00000143546
1,14	0,97	#N/D	ENSG00000184330
0,13	0,46	#N/D	ENSG00000143556
3,16	0,00	0,00	ENSG00000197956
1,09	0,85	0,92	ENSG00000196420
0,95	0,57	0,67	ENSG00000196154
1,06	0,57	0,67	ENSG00000188015
0,63	0,00	0,00	ENSG00000196754
1,07	0,42	0,54	ENSG00000188643
1,93	0,11	0,18	ENSG00000189334
1,33	0,00	0,00	ENSG00000189171
1,90	0,00	0,00	ENSG00000160678

0,85	0,04	0,08	ENSG00000160679
1,08	0,38	0,49	ENSG00000143553
1,37	0,00	0,00	ENSG00000143621
0,44	0,01	0,01	ENSG00000169418
1,58	0,00	0,00	ENSG00000143624
1,06	0,54	0,65	ENSG00000143554
1,40	0,00	0,00	ENSG00000143614
1,23	0,01	0,02	ENSG00000198837
1,40	0,00	0,00	ENSG00000160741
1,64	0,00	0,00	ENSG00000143570
138,91	0,07	0,13	ENSG00000285779
1,13	0,23	0,33	ENSG00000143578
1,49	0,00	0,00	ENSG00000143543
2,03	0,00	0,00	ENSG00000285641
1,57	0,00	0,00	ENSG00000143545
1,08	0,34	0,45	ENSG00000177954
2,78	0,01	0,02	ENSG00000143552
1,50	0,00	0,00	ENSG00000143549
1,14	0,97	0,98	ENSG00000163263
1,33	0,00	0,00	ENSG00000143612
1,28	0,00	0,01	ENSG00000143569
1,23	0,01	0,03	ENSG00000143575
1,26	0,58	0,68	ENSG00000143595
1,76	0,00	0,00	ENSG00000143515
2,56	0,00	0,00	ENSG00000160712
5,63	0,01	0,01	ENSG00000169291
0,04	0,20	0,30	ENSG00000163239
1,32	0,00	0,00	ENSG00000160714
1,30	0,05	0,09	ENSG00000160716
1,50	0,00	0,00	ENSG00000160710
8,11	0,00	0,00	ENSG00000143603
1,77	0,00	0,00	ENSG00000163344
2,53	0,00	0,00	ENSG00000163346
1,55	0,00	0,00	ENSG00000163348
2,66	0,00	0,00	ENSG00000160691
1,30	0,00	0,00	ENSG00000173207
1,40	0,00	0,00	ENSG00000160688
1,94	0,03	0,05	ENSG00000163352
1,51	0,00	0,00	ENSG00000160685
1,85	0,00	0,00	ENSG00000163354
0,86	0,49	0,59	ENSG00000163357
1,41	0,00	0,00	ENSG00000143537
1,90	0,00	0,00	ENSG00000243364
5,30	0,00	0,00	ENSG00000251246
1,67	0,00	0,00	ENSG00000143590
1,56	0,00	0,00	ENSG00000169242
1,59	0,00	0,00	ENSG00000169241
2,68	0,00	0,00	ENSG00000179085

1,32	0,00	0,00	ENSG00000163463
1,41	0,02	0,04	ENSG00000273088
0,95	0,77	0,85	ENSG00000163462
1,30	0,02	0,05	ENSG00000185499
1,47	0,00	0,00	ENSG00000169231
1,42	0,00	0,00	ENSG00000173171
1,31	0,00	0,00	ENSG00000177628
1,11	0,20	0,30	ENSG00000160767
1,86	0,00	0,00	ENSG00000116521
1,35	0,00	0,00	ENSG00000176444
1,43	0,00	0,00	ENSG00000143630
1,72	0,20	0,30	ENSG00000143627
2,00	0,00	0,00	ENSG00000160752
1,54	0,00	0,00	ENSG00000160753
1,28	0,00	0,01	ENSG00000116539
2,37	0,00	0,00	ENSG00000125459
0,96	0,68	0,77	ENSG00000287839
1,42	0,00	0,00	ENSG00000163374
1,22	0,01	0,03	ENSG00000132676
1,82	0,00	0,00	ENSG00000116580
1,17	0,05	0,09	ENSG00000132718
1,80	0,00	0,00	ENSG00000143622
1,79	0,00	0,00	ENSG00000132680
1,03	0,89	0,95	ENSG00000173080
1,10	0,25	0,36	ENSG00000116584
2,24	0,00	0,00	ENSG00000163479
1,00	0,95	0,98	ENSG00000160803
1,50	0,00	0,00	ENSG00000116586
0,41	0,27	0,38	ENSG00000132698
1,62	0,00	0,00	ENSG00000254726
2,81	0,00	0,00	ENSG00000160789
2,68	0,00	0,00	ENSG00000196189
2,77	0,00	0,00	ENSG00000160785
1,18	0,16	0,25	ENSG00000260238
1,42	0,00	0,00	ENSG00000160783
131,66	0,08	0,13	ENSG00000242252
1,39	0,00	0,00	ENSG00000160781
1,47	0,00	0,00	ENSG00000198952
1,58	0,00	0,00	ENSG00000163472
13,03	0,00	0,00	ENSG00000198715
0,36	0,08	0,14	ENSG00000189030
0,98	0,80	0,88	ENSG00000163468
0,62	0,11	0,19	ENSG00000163467
0,13	0,46	0,56	ENSG00000132677
2,63	0,00	0,00	ENSG00000116604
0,86	0,07	0,12	ENSG00000183856
1,89	0,45	0,56	ENSG00000187862
1,10	0,26	0,37	ENSG00000163382

0,82	0,04	0,07	ENSG00000160818
4,70	0,01	0,03	ENSG00000132702
0,48	0,00	0,00	ENSG00000132692
0,60	0,26	0,37	ENSG00000132688
2,05	0,00	0,00	ENSG00000143320
1,50	0,00	0,00	ENSG00000143319
1,00	0,99	0,99	ENSG00000143303
1,17	0,07	0,12	ENSG00000143314
1,22	0,01	0,03	ENSG00000143321
1,27	0,00	0,01	ENSG00000143294
0,07	0,00	0,00	ENSG00000027869
0,41	0,00	0,00	ENSG00000198400
0,82	0,74	0,82	ENSG00000027644
0,48	0,00	0,00	ENSG00000187800
1,60	0,51	0,62	ENSG00000160838
0,92	0,30	0,41	ENSG00000132694
1,14	0,97	#N/D	ENSG00000253831
2,18	0,00	0,00	ENSG00000117036
1,14	0,97	#N/D	ENSG00000143297
1,14	0,97	#N/D	ENSG00000163518
1,14	0,97	0,98	ENSG00000160856
#N/D	#N/D	#N/D	ENSG00000132704
#N/D	#N/D	#N/D	ENSG00000163534
#N/D	#N/D	#N/D	ENSG00000073754
1,40	0,00	0,00	ENSG00000183853
1,14	0,97	#N/D	ENSG00000288460
14,59	0,00	0,00	ENSG00000158473
#N/D	#N/D	#N/D	ENSG00000158477
#N/D	#N/D	#N/D	ENSG00000158481
#N/D	#N/D	#N/D	ENSG00000158485
#N/D	#N/D	#N/D	ENSG00000158488
#N/D	#N/D	#N/D	ENSG00000186306
#N/D	#N/D	#N/D	ENSG00000180708
1,14	0,97	0,98	ENSG00000173285
#N/D	#N/D	#N/D	ENSG00000198965
#N/D	#N/D	#N/D	ENSG00000197532
#N/D	#N/D	#N/D	ENSG00000186440
#N/D	#N/D	#N/D	ENSG00000279111
#N/D	#N/D	#N/D	ENSG00000198967
8,39	0,46	#N/D	ENSG00000163554
#N/D	#N/D	#N/D	ENSG00000196171
1,14	0,97	#N/D	ENSG00000203757
1,14	0,97	#N/D	ENSG00000197403
#N/D	#N/D	#N/D	ENSG00000180433
#N/D	#N/D	#N/D	ENSG00000188340
1,14	0,97	#N/D	ENSG00000163563
8,39	0,46	0,56	ENSG00000163564
0,60	0,00	0,00	ENSG00000163565

0,04	0,20	0,30	ENSG00000289721
1,14	0,97	0,98	ENSG00000163568
1,14	0,97	#N/D	ENSG00000162706
1,14	0,97	#N/D	ENSG00000213088
#N/D	#N/D	#N/D	ENSG00000179639
1,14	0,97	#N/D	ENSG00000196266
#N/D	#N/D	#N/D	ENSG00000249730
#N/D	#N/D	#N/D	ENSG00000196184
#N/D	#N/D	#N/D	ENSG00000184155
1,14	0,97	0,98	ENSG00000132703
#N/D	#N/D	#N/D	ENSG00000132693
1,95	0,00	0,00	ENSG00000158716
#N/D	#N/D	#N/D	ENSG00000181036
0,07	0,01	0,01	ENSG00000158714
2,19	0,00	0,00	ENSG00000256029
2,56	0,05	0,09	ENSG00000243284
1,99	0,00	0,00	ENSG00000213085
1,12	0,77	0,85	ENSG00000279430
1,57	0,00	0,00	ENSG00000158710
0,15	0,00	0,00	ENSG00000085552
2,53	0,17	0,26	ENSG00000162723
0,50	0,32	0,43	ENSG00000177807
1,25	0,03	0,06	ENSG00000143315
3,99	0,00	0,00	ENSG00000162728
2,13	0,00	0,00	ENSG00000162729
0,01	0,05	0,10	ENSG00000018625
0,13	0,46	0,56	ENSG00000132681
1,14	0,97	0,98	ENSG00000143318
1,31	0,00	0,00	ENSG00000162734
1,00	0,97	0,98	ENSG00000132716
1,26	0,27	0,38	ENSG00000258465
1,14	0,11	0,19	ENSG00000162735
1,81	0,00	0,00	ENSG00000122218
1,11	0,18	0,28	ENSG00000162736
2,22	0,00	0,00	ENSG00000171786
30,13	0,00	0,00	ENSG00000162738
#N/D	#N/D	#N/D	ENSG00000162739
15,64	0,33	0,44	ENSG00000066294
#N/D	#N/D	#N/D	ENSG00000117090
#N/D	#N/D	#N/D	ENSG00000117091
0,13	0,46	0,56	ENSG00000026751
1,14	0,97	#N/D	ENSG00000122224
#N/D	#N/D	#N/D	ENSG00000122223
#N/D	#N/D	#N/D	ENSG00000179914
0,04	0,20	0,30	ENSG00000158764
0,76	0,01	0,01	ENSG00000158769
1,14	0,38	0,49	ENSG00000270149
0,98	0,97	0,99	ENSG00000215845

1,62	0,00	0,00	ENSG00000158773
0,69	0,00	0,01	ENSG00000186517
0,26	0,00	0,00	ENSG00000143217
1,23	0,56	0,66	ENSG00000162755
1,06	0,52	0,62	ENSG00000143256
1,48	0,00	0,00	ENSG00000158793
0,90	0,21	0,31	ENSG00000158796
1,21	0,03	0,06	ENSG00000143222
1,17	0,05	0,10	ENSG00000143258
1,64	0,00	0,00	ENSG00000143224
1,54	0,00	0,00	ENSG00000158850
2,05	0,00	0,00	ENSG00000158859
1,45	0,00	0,00	ENSG00000158864
0,97	0,96	0,98	ENSG00000158869
1,14	0,97	#N/D	ENSG00000158874
1,39	0,00	0,00	ENSG00000158882
2,33	0,00	0,00	ENSG00000143257
1,14	0,97	#N/D	ENSG00000248485
0,71	0,16	0,25	ENSG00000158887
1,32	0,00	0,00	ENSG00000143252
3,48	0,00	0,00	ENSG00000188931
6,62	0,00	0,00	ENSG00000143226
1,29	0,28	0,38	ENSG00000173110
0,04	0,20	0,30	ENSG00000203747
0,13	0,46	#N/D	ENSG00000289768
1,14	0,97	#N/D	ENSG00000244682
8,39	0,46	#N/D	ENSG00000162747
0,07	0,31	0,43	ENSG00000072694
0,12	0,00	0,00	ENSG00000132185
0,31	0,00	0,00	ENSG00000162746
0,68	0,00	0,00	ENSG00000081721
0,92	0,28	0,38	ENSG00000118217
1,43	0,08	0,14	ENSG00000162745
1,05	0,73	0,82	ENSG00000198929
0,12	0,00	0,00	ENSG00000254706
1,07	0,88	0,94	ENSG00000171722
0,99	0,90	0,96	ENSG00000239887
0,02	0,12	0,19	ENSG00000198574
1,50	0,00	0,00	ENSG00000152332
1,84	0,00	0,00	ENSG00000117143
0,96	0,61	0,71	ENSG00000162733
3,34	0,00	0,00	ENSG00000132196
0,49	0,20	0,30	ENSG00000185860
0,52	0,00	0,00	ENSG00000117152
1,18	0,26	0,36	ENSG00000143248
0,73	0,00	0,00	ENSG00000143228
0,55	0,00	0,00	ENSG00000185630
#N/D	#N/D	#N/D	ENSG00000162761

1,14	0,97	0,98	ENSG00000143171
1,14	0,97	#N/D	ENSG00000162763
1,42	0,00	0,00	ENSG00000143198
1,32	0,00	0,00	ENSG00000143149
1,57	0,00	0,00	ENSG00000143183
0,67	0,00	0,00	ENSG00000143179
0,39	0,01	0,03	ENSG00000188859
1,16	0,07	0,13	ENSG00000143157
1,29	0,00	0,01	ENSG00000152382
0,53	0,58	0,68	ENSG00000143195
1,14	0,97	0,98	ENSG00000143194
0,98	0,99	0,99	ENSG00000143167
1,14	0,97	#N/D	ENSG00000198842
1,16	0,08	0,13	ENSG00000143190
0,89	0,79	0,87	ENSG00000198821
0,68	0,00	0,00	ENSG00000143162
0,74	0,57	0,67	ENSG00000198771
1,35	0,00	0,00	ENSG00000197965
1,13	0,75	0,83	ENSG00000143199
1,21	0,03	0,06	ENSG00000143158
1,08	0,37	0,49	ENSG00000143164
1,04	0,60	0,69	ENSG00000143147
1,07	0,44	0,56	ENSG00000143155
1,35	0,00	0,00	ENSG00000213064
1,65	0,00	0,00	ENSG00000143178
1,14	0,97	0,98	ENSG00000143185
8,39	0,46	0,56	ENSG00000143184
2,20	0,00	0,01	ENSG00000143196
1,36	0,00	0,00	ENSG00000143153
1,25	0,66	0,75	ENSG00000289466
1,24	0,03	0,05	ENSG00000143156
2,41	0,00	0,00	ENSG00000117475
2,68	0,06	0,11	ENSG00000117477
0,80	0,02	0,04	ENSG00000117479
0,05	0,24	0,35	ENSG00000198734
22,89	0,26	0,37	ENSG00000174175
0,94	0,49	0,59	ENSG00000000460
0,09	0,00	0,00	ENSG00000188404
0,13	0,46	0,56	ENSG00000007908
0,58	0,00	0,00	ENSG00000171806
0,97	0,75	0,83	ENSG00000000457
1,32	0,00	0,01	ENSG00000075945
0,13	0,46	0,56	ENSG00000203740
1,25	0,02	0,05	ENSG00000120370
3,44	0,01	0,03	ENSG00000116132
#N/D	#N/D	#N/D	ENSG00000117501
8,39	0,46	0,56	ENSG00000007933
#N/D	#N/D	#N/D	ENSG00000094963

1,14	0,97	#N/D	ENSG00000010932
1,24	0,39	0,50	ENSG00000076258
1,17	0,05	0,10	ENSG00000117523
0,05	0,24	0,35	ENSG00000283683
1,14	0,97	0,98	ENSG00000034971
1,40	0,00	0,00	ENSG00000117533
1,09	0,29	0,40	ENSG00000010165
1,45	0,03	0,05	ENSG00000197959
1,25	0,01	0,02	ENSG00000135845
0,27	0,22	0,32	ENSG00000180999
2,49	0,00	0,00	ENSG00000094975
1,14	0,97	#N/D	ENSG00000117560
#N/D	#N/D	#N/D	ENSG00000279061
0,55	0,28	0,39	ENSG00000120337
1,11	0,46	0,57	ENSG00000117586
1,13	0,11	0,19	ENSG00000117592
1,14	0,97	0,98	ENSG00000162753
1,14	0,97	0,98	ENSG00000285777
0,50	0,32	0,43	ENSG00000183831
#N/D	#N/D	#N/D	ENSG00000232113
1,31	0,00	0,01	ENSG00000076321
1,31	0,00	0,01	ENSG00000120334
1,03	0,75	0,83	ENSG00000117593
1,33	0,00	0,00	ENSG00000185278
0,99	1,00	1,00	ENSG00000117601
1,11	0,20	0,30	ENSG00000135870
#N/D	#N/D	#N/D	ENSG00000293562
1,13	0,18	0,27	ENSG00000152061
0,52	0,03	0,07	ENSG00000203737
0,75	0,00	0,00	ENSG00000116161
1,45	0,00	0,00	ENSG00000120333
2,84	0,11	0,19	ENSG00000120332
1,19	0,10	0,17	ENSG00000235750
8,39	0,46	#N/D	ENSG00000116147
1,29	0,00	0,01	ENSG00000143207
0,02	0,00	0,00	ENSG00000116183
0,07	0,31	0,43	ENSG00000152092
1,93	0,19	0,29	ENSG00000198797
4,39	0,00	0,00	ENSG00000120341
1,95	0,00	0,00	ENSG00000075391
0,05	0,24	0,35	ENSG00000188585
2,62	0,00	0,01	ENSG00000240021
1,37	0,00	0,00	ENSG00000116191
1,83	0,00	0,00	ENSG00000116194
1,60	0,00	0,00	ENSG00000116199
1,32	0,00	0,00	ENSG00000186283
1,43	0,00	0,00	ENSG00000143322
1,08	0,36	0,48	ENSG00000057252

0,80	0,62	0,72	ENSG00000162779
1,14	0,97	#N/D	ENSG00000116218
1,14	0,97	0,98	ENSG00000162782
1,14	0,97	#N/D	ENSG00000143340
1,48	0,00	0,00	ENSG00000169905
1,07	0,42	0,54	ENSG00000143337
#N/D	#N/D	#N/D	ENSG00000293564
1,11	0,20	0,30	ENSG00000135837
1,07	0,41	0,52	ENSG00000116260
0,51	0,00	0,00	ENSG00000121454
1,06	0,50	0,60	ENSG00000230124
1,54	0,00	0,00	ENSG00000143324
1,10	0,29	0,40	ENSG00000135835
1,13	0,16	0,25	ENSG00000135823
0,94	0,44	0,56	ENSG00000153029
0,88	0,14	0,22	ENSG00000162783
1,57	0,09	0,15	ENSG00000198216
#N/D	#N/D	#N/D	ENSG00000179930
1,87	0,00	0,00	ENSG00000135821
0,50	0,32	0,43	ENSG00000203730
1,14	0,97	#N/D	ENSG00000121446
1,18	0,12	0,19	ENSG00000135828
0,32	0,00	0,01	ENSG00000143333
0,07	0,31	#N/D	ENSG00000135824
0,29	0,00	0,00	ENSG00000135838
0,72	0,00	0,00	ENSG00000135829
#N/D	#N/D	#N/D	ENSG00000157060
1,18	0,04	0,08	ENSG00000135862
0,40	0,00	0,00	ENSG00000058085
0,47	0,00	0,00	ENSG00000157064
1,14	0,09	0,16	ENSG00000116698
0,05	0,00	0,00	ENSG00000116701
1,07	0,41	0,52	ENSG00000162704
1,00	0,96	0,98	ENSG00000143344
2,24	0,10	0,17	ENSG00000173627
0,48	0,00	0,00	ENSG00000198756
0,77	0,00	0,01	ENSG00000198860
1,04	0,68	0,77	ENSG00000116667
1,32	0,00	0,00	ENSG00000116406
0,30	0,00	0,00	ENSG00000135842
0,94	0,49	0,59	ENSG00000121481
0,75	0,00	0,00	ENSG00000121486
0,82	0,16	0,25	ENSG00000116668
1,19	0,42	0,54	ENSG00000279838
2,19	0,00	0,00	ENSG00000116679
1,14	0,97	#N/D	ENSG00000279401
1,61	0,00	0,00	ENSG00000143341
1,89	0,45	0,56	ENSG00000116690

0,97	0,74	0,83	ENSG00000047410
1,35	0,00	0,00	ENSG00000157181
1,44	0,68	0,77	ENSG00000116703
1,09	0,27	0,37	ENSG00000073756
0,76	0,00	0,00	ENSG00000116711
0,05	0,24	0,35	ENSG00000162670
0,98	0,99	0,99	ENSG00000150681
1,14	0,97	0,98	ENSG00000253148
0,99	1,00	1,00	ENSG00000090104
0,13	0,46	#N/D	ENSG00000127074
0,50	0,00	0,00	ENSG00000116741
1,16	0,10	0,17	ENSG00000116750
1,46	0,00	0,00	ENSG00000116747
0,74	0,01	0,02	ENSG00000023572
1,17	0,06	0,11	ENSG00000134371
1,90	0,01	0,03	ENSG00000162630
0,23	0,00	0,00	ENSG00000162687
2,62	0,00	0,00	ENSG00000000971
2,96	0,00	0,00	ENSG00000289697
1,14	0,97	0,98	ENSG00000116785
1,14	0,97	#N/D	ENSG00000244414
#N/D	#N/D	#N/D	ENSG00000134365
1,14	0,97	#N/D	ENSG00000080910
#N/D	#N/D	#N/D	ENSG00000134389
1,14	0,97	#N/D	ENSG00000143278
0,73	0,00	0,00	ENSG00000066279
2,10	0,00	0,00	ENSG00000177888
0,24	0,00	0,01	ENSG00000134376
0,90	0,29	0,40	ENSG00000213047
0,49	0,04	0,08	ENSG00000203724
0,49	0,10	0,17	ENSG00000143355
0,88	0,15	0,23	ENSG00000151414
1,14	0,97	0,98	ENSG00000151418
22,89	0,26	0,37	ENSG00000081237
0,54	0,13	0,21	ENSG00000116833
0,96	0,62	0,72	ENSG00000162702
0,80	0,01	0,02	ENSG00000118193
1,00	0,99	1,00	ENSG00000118197
1,11	0,21	0,31	ENSG00000118200
0,07	0,31	0,43	ENSG00000170128
0,41	0,00	0,00	ENSG00000163362
0,69	0,00	0,00	ENSG00000116852
0,99	1,00	1,00	ENSG00000081248
0,45	0,01	0,01	ENSG00000232237
1,91	0,00	0,00	ENSG00000116857
0,03	0,00	0,00	ENSG00000163395
0,38	0,14	0,23	ENSG00000081277
#N/D	#N/D	#N/D	ENSG00000118194

0,02	0,10	0,17	ENSG00000159166
15,64	0,33	0,44	ENSG00000159173
0,71	0,00	0,00	ENSG00000174307
0,93	0,34	0,45	ENSG00000159176
2,14	0,00	0,00	ENSG00000134369
1,14	0,09	0,16	ENSG00000198700
2,11	0,00	0,00	ENSG00000198892
0,89	0,61	0,71	ENSG00000163431
2,06	0,00	0,00	ENSG00000134375
1,33	0,00	0,00	ENSG00000176393
0,04	0,00	0,00	ENSG00000163435
0,83	0,24	0,35	ENSG00000170075
1,13	0,14	0,22	ENSG00000143862
0,21	0,04	0,07	ENSG00000143851
0,98	0,98	0,99	ENSG00000133067
1,31	0,00	0,00	ENSG00000077152
1,55	0,00	0,00	ENSG00000077157
1,52	0,02	0,05	ENSG00000143858
1,59	0,00	0,00	ENSG00000117139
0,95	0,66	0,75	ENSG00000183155
1,02	0,83	0,90	ENSG00000117153
1,77	0,00	0,00	ENSG00000159346
1,25	0,01	0,03	ENSG00000159348
1,51	0,00	0,00	ENSG00000163444
5,84	0,00	0,00	ENSG00000143847
22,89	0,26	0,37	ENSG00000122180
5,73	0,00	0,00	ENSG00000163485
2,60	0,00	0,00	ENSG00000133055
1,25	0,01	0,02	ENSG00000133048
0,99	1,00	1,00	ENSG00000133063
1,05	0,65	0,74	ENSG00000159388
0,02	0,15	0,23	ENSG00000122176
0,42	0,08	0,14	ENSG00000188783
0,04	0,20	0,30	ENSG00000188770
1,76	0,00	0,00	ENSG00000058668
0,43	0,22	0,32	ENSG00000122188
2,10	0,00	0,00	ENSG00000257315
1,01	0,86	0,93	ENSG00000058673
#N/D	#N/D	#N/D	ENSG00000288644
1,15	0,13	0,21	ENSG00000182004
1,43	0,00	0,00	ENSG00000143842
2,58	0,00	0,00	ENSG00000143845
0,99	1,00	#N/D	ENSG00000143839
8,39	0,46	0,56	ENSG00000170498
1,14	0,97	#N/D	ENSG00000174567
0,85	0,51	0,61	ENSG00000143850
1,93	0,00	0,00	ENSG00000158615
1,09	0,33	0,44	ENSG00000133056

1,08	0,35	0,46	ENSG00000198625
1,04	0,77	0,85	ENSG00000170382
0,88	0,20	0,29	ENSG00000163531
4,21	0,00	0,00	ENSG00000184144
2,67	0,00	0,00	ENSG00000174529
1,50	0,00	0,00	ENSG00000117222
1,39	0,00	0,00	ENSG00000133059
1,44	0,00	0,00	ENSG00000133069
2,79	0,00	0,00	ENSG00000163545
1,14	0,97	0,98	ENSG00000162873
4,52	0,00	0,00	ENSG00000186007
1,99	0,00	0,00	ENSG00000281406
1,33	0,00	0,01	ENSG00000117266
1,39	0,09	0,16	ENSG00000174514
1,25	0,00	0,01	ENSG00000158711
0,91	0,25	0,36	ENSG00000158715
1,18	0,03	0,07	ENSG00000069275
0,97	0,72	0,81	ENSG00000117280
1,16	0,07	0,12	ENSG00000133065
1,64	0,06	0,10	ENSG00000162877
0,14	0,00	0,00	ENSG00000174502
0,24	0,00	0,00	ENSG00000276600
5,54	0,02	0,04	ENSG00000196188
8,39	0,46	#N/D	ENSG00000263961
1,92	0,28	0,39	ENSG00000198049
1,11	0,23	0,34	ENSG00000196550
1,30	0,00	0,00	ENSG00000266028
0,70	0,00	0,00	ENSG00000263528
1,38	0,00	0,00	ENSG00000266094
1,06	0,85	0,92	ENSG00000279946
1,66	0,00	0,00	ENSG00000143486
1,01	0,93	0,98	ENSG00000143479
1,43	0,00	0,00	ENSG00000162889
0,04	0,20	0,30	ENSG00000136634
1,14	0,97	#N/D	ENSG00000142224
0,01	0,06	0,11	ENSG00000162891
0,09	0,00	0,00	ENSG00000162892
0,33	0,00	0,00	ENSG00000162894
0,14	0,05	0,09	ENSG00000162896
1,29	0,74	0,82	ENSG00000162897
0,57	0,01	0,02	ENSG00000182795
0,58	0,00	0,00	ENSG00000123836
1,15	0,09	0,15	ENSG00000180667
2,71	0,36	0,48	ENSG00000123843
8,42	0,00	0,00	ENSG00000123838
0,97	0,68	0,77	ENSG00000196352
0,97	0,93	0,98	ENSG00000117322
0,13	0,46	0,56	ENSG00000203710

37,39	0,19	0,29	ENSG00000197721
1,09	0,30	0,41	ENSG00000117335
0,85	0,60	0,69	ENSG00000174059
0,62	0,00	0,00	ENSG00000076356
0,49	0,10	0,17	ENSG00000008118
3,36	0,00	0,00	ENSG00000196878
203,28	0,00	0,00	ENSG00000123689
0,10	0,02	0,04	ENSG00000117594
5,28	0,10	0,17	ENSG00000009790
1,37	0,00	0,00	ENSG00000162757
0,49	0,10	0,17	ENSG00000289700
0,34	0,01	0,01	ENSG00000117595
0,75	0,00	0,00	ENSG00000117597
1,05	0,58	0,68	ENSG00000143469
0,96	0,72	0,81	ENSG00000082497
0,92	0,43	0,55	ENSG00000054392
0,51	0,08	0,14	ENSG00000143473
0,33	0,02	0,04	ENSG00000279333
1,14	0,97	#N/D	ENSG00000283952
1,09	0,36	0,47	ENSG00000117625
0,74	0,00	0,00	ENSG00000082512
1,14	0,97	#N/D	ENSG00000198570
1,20	0,03	0,07	ENSG00000170385
1,13	0,14	0,23	ENSG00000117650
1,26	0,00	0,01	ENSG00000123684
0,94	0,45	0,56	ENSG00000143493
1,30	0,00	0,00	ENSG00000143476
1,39	0,00	0,00	ENSG00000066027
1,15	0,11	0,19	ENSG00000065600
1,61	0,00	0,00	ENSG00000117691
3,02	0,00	0,00	ENSG00000162772
0,01	0,07	0,12	ENSG00000162771
0,49	0,00	0,00	ENSG00000123685
1,07	0,46	0,56	ENSG00000117697
0,71	0,00	0,00	ENSG00000203705
8,39	0,46	0,56	ENSG00000185523
0,89	0,22	0,32	ENSG00000162769
1,41	0,02	0,05	ENSG00000143494
0,71	0,00	0,00	ENSG00000174606
1,50	0,00	0,00	ENSG00000136643
0,98	0,97	0,98	ENSG00000117707
1,48	0,00	0,00	ENSG00000143499
2,04	0,00	0,00	ENSG00000152104
0,80	0,01	0,01	ENSG00000117724
0,01	0,09	0,16	ENSG00000082482
1,01	0,91	0,97	ENSG00000136636
1,01	0,98	0,99	ENSG00000042781
37,39	0,19	0,29	ENSG00000196482

1,06	0,53	0,63	ENSG00000092978
0,56	0,03	0,07	ENSG00000162814
0,60	0,00	0,00	ENSG00000067533
0,66	0,00	0,00	ENSG00000092969
0,52	0,00	0,00	ENSG00000143353
2,71	0,36	0,48	ENSG00000215817
0,99	1,00	#N/D	ENSG00000196660
0,67	0,00	0,00	ENSG00000136628
1,13	0,15	0,24	ENSG00000162813
0,84	0,02	0,05	ENSG00000067704
1,00	0,97	0,99	ENSG00000118873
0,02	0,11	0,18	ENSG00000116141
15,64	0,33	0,44	ENSG00000162817
0,34	0,00	0,00	ENSG00000117791
0,13	0,46	0,56	ENSG00000186205
1,14	0,97	0,98	ENSG00000286231
0,73	0,00	0,01	ENSG00000136630
0,82	0,02	0,05	ENSG00000143507
1,56	0,00	0,00	ENSG00000143512
0,59	0,00	0,00	ENSG00000143498
1,12	0,17	0,26	ENSG00000154305
1,15	0,08	0,13	ENSG00000186063
0,84	0,04	0,07	ENSG00000162819
0,13	0,46	0,56	ENSG00000197520
0,60	0,00	0,00	ENSG00000154309
1,02	0,91	0,97	ENSG00000187554
0,94	0,75	0,83	ENSG00000143502
1,14	0,97	0,98	ENSG00000178395
1,44	0,68	0,77	ENSG00000203697
1,06	0,43	0,55	ENSG00000162909
0,57	0,00	0,00	ENSG00000143514
0,63	0,00	0,00	ENSG00000143756
0,88	0,12	0,20	ENSG00000143753
0,84	0,05	0,09	ENSG00000143748
0,67	0,00	0,00	ENSG00000143771
1,22	0,01	0,03	ENSG00000162923
0,06	0,00	0,00	ENSG00000143786
0,36	0,00	0,00	ENSG00000185842
0,74	0,00	0,00	ENSG00000143815
1,88	0,00	0,00	ENSG00000154380
1,18	0,04	0,08	ENSG00000143742
2,12	0,00	0,00	ENSG00000143819
1,13	0,14	0,23	ENSG00000196187
1,29	0,56	0,67	ENSG00000243709
66,40	0,13	0,21	ENSG00000255835
0,95	0,54	0,65	ENSG00000143811
#N/D	#N/D	#N/D	ENSG00000143768
1,55	0,00	0,00	ENSG00000143751

1,03	0,71	0,79	ENSG00000163041
1,82	0,00	0,00	ENSG00000182827
0,70	0,55	0,65	ENSG00000185155
0,63	0,00	0,00	ENSG00000183814
0,66	0,00	0,00	ENSG00000143799
0,79	0,72	0,80	ENSG00000203685
0,97	0,68	0,77	ENSG00000143772
0,38	0,00	0,00	ENSG00000143801
0,44	0,00	0,00	ENSG00000288674
0,67	0,00	0,00	ENSG00000163050
0,58	0,00	0,00	ENSG00000143776
1,90	0,00	0,00	ENSG00000181450
1,12	0,16	0,26	ENSG00000143740
0,86	0,07	0,13	ENSG00000081692
#N/D	#N/D	#N/D	ENSG00000185888
1,05	0,67	0,76	ENSG00000143816
2,30	0,12	0,19	ENSG00000154342
1,19	0,03	0,06	ENSG00000143761
1,35	0,00	0,00	ENSG00000143793
1,56	0,00	0,00	ENSG00000162910
1,59	0,03	0,05	ENSG00000280157
2,01	0,00	0,00	ENSG00000143774
3,29	0,00	0,00	ENSG00000198835
1,14	0,14	0,23	ENSG00000181873
1,37	0,00	0,00	ENSG00000154358
1,76	0,00	0,00	ENSG00000154370
1,53	0,00	0,00	ENSG00000162931
0,88	0,78	0,86	ENSG00000168148
0,48	0,00	0,00	ENSG00000181218
0,36	0,00	0,00	ENSG00000196890
0,64	0,14	0,23	ENSG00000279306
0,74	0,00	0,00	ENSG00000168159
0,86	0,19	0,29	ENSG00000116574
1,26	0,01	0,02	ENSG00000168118
1,38	0,00	0,00	ENSG00000154429
1,85	0,60	0,69	ENSG00000143632
1,05	0,57	0,68	ENSG00000069248
0,82	0,02	0,05	ENSG00000135776
0,99	0,89	0,96	ENSG00000135801
0,82	0,02	0,03	ENSG00000135763
1,12	0,17	0,26	ENSG00000143641
0,45	0,00	0,00	ENSG00000177614
0,91	0,25	0,36	ENSG00000135775
1,42	0,08	0,14	ENSG00000135744
8,39	0,46	#N/D	ENSG00000135773
1,38	0,00	0,00	ENSG00000119280
0,68	0,00	0,00	ENSG00000143643
1,30	0,00	0,01	ENSG00000173409

0,54	0,00	0,00	ENSG00000182118
0,51	0,42	0,54	ENSG00000119283
0,62	0,00	0,00	ENSG00000143633
0,76	0,00	0,00	ENSG00000116906
1,39	0,00	0,00	ENSG00000116903
0,83	0,04	0,08	ENSG00000010072
0,76	0,00	0,00	ENSG00000135766
0,65	0,00	0,00	ENSG00000287856
1,09	0,32	0,43	ENSG00000116918
2,73	0,00	0,00	ENSG00000270106
1,05	0,60	0,70	ENSG00000162946
1,51	0,00	0,00	ENSG00000116991
0,73	0,06	0,10	ENSG00000212916
0,72	0,00	0,00	ENSG00000135778
0,47	0,00	0,00	ENSG00000135749
0,79	0,01	0,02	ENSG00000143674
1,06	0,55	0,66	ENSG00000135750
1,89	0,00	0,00	ENSG00000183780
0,73	0,01	0,02	ENSG00000168275
0,72	0,00	0,00	ENSG00000059588
1,32	0,00	0,00	ENSG00000168264
1,00	0,95	0,98	ENSG00000173726
0,72	0,00	0,00	ENSG00000188739
1,13	0,14	0,23	ENSG00000054267
0,74	0,00	0,00	ENSG00000152904
0,96	0,68	0,77	ENSG00000285053
0,66	0,00	0,00	ENSG00000284770
0,50	0,00	0,00	ENSG00000162885
0,54	0,00	0,00	ENSG00000168243
0,50	0,00	0,00	ENSG00000143669
0,76	0,00	0,00	ENSG00000116962
1,00	0,99	0,99	ENSG00000077585
1,07	0,54	0,64	ENSG00000086619
1,58	0,01	0,02	ENSG00000186197
0,79	0,01	0,01	ENSG00000116977
0,65	0,00	0,00	ENSG00000119285
0,17	0,00	0,00	ENSG00000077522
0,83	0,02	0,04	ENSG00000116984
3,56	0,23	0,34	ENSG00000244020
0,35	0,00	0,00	ENSG00000198626
1,14	0,97	#N/D	ENSG00000116996
0,37	0,01	0,01	ENSG00000133019
0,76	0,00	0,00	ENSG00000155816
1,64	0,17	0,26	ENSG00000180875
0,98	0,97	0,99	ENSG00000182901
0,98	0,84	0,91	ENSG00000091483
0,49	0,20	0,30	ENSG00000117009
0,80	0,02	0,04	ENSG00000054277

0,88	0,15	0,24	ENSG00000203668
0,99	1,00	1,00	ENSG00000162843
0,92	0,34	0,45	ENSG00000174371
0,13	0,46	#N/D	ENSG00000196289
4,08	0,03	0,05	ENSG00000197769
1,14	0,97	0,98	ENSG00000180287
0,75	0,00	0,00	ENSG00000143702
0,64	0,00	0,00	ENSG00000054282
0,82	0,01	0,03	ENSG00000117020
1,74	0,00	0,00	ENSG00000179456
1,14	0,97	#N/D	ENSG00000279774
0,03	0,17	0,26	ENSG00000173728
0,64	0,00	0,00	ENSG00000035687
15,64	0,33	0,44	ENSG00000179397
0,97	0,76	0,84	ENSG00000121644
0,13	0,46	0,56	ENSG00000284188
1,52	0,00	0,00	ENSG00000203667
0,69	0,00	0,00	ENSG00000153187
0,35	0,00	0,00	ENSG00000203666
0,74	0,10	0,17	ENSG00000162849
0,95	0,54	0,64	ENSG00000185420
1,41	0,00	0,00	ENSG00000162851
0,95	0,54	0,64	ENSG00000162852
0,40	0,00	0,00	ENSG00000143653
0,72	0,00	0,00	ENSG00000153207
0,99	0,93	0,98	ENSG00000197472
1,06	0,78	0,86	ENSG00000135747
0,49	0,00	0,00	ENSG00000277462
0,91	0,35	0,47	ENSG00000188295
1,03	0,80	0,87	ENSG00000196418
0,13	0,46	0,56	ENSG00000197617
1,11	0,19	0,29	ENSG00000162714
0,21	0,00	0,00	ENSG00000162711
0,67	0,65	0,74	ENSG00000177535
0,13	0,46	#N/D	ENSG00000169224
#N/D	#N/D	#N/D	ENSG00000196242
#N/D	#N/D	#N/D	ENSG00000177489
#N/D	#N/D	#N/D	ENSG00000177476
#N/D	#N/D	#N/D	ENSG00000198452
#N/D	#N/D	#N/D	ENSG00000197437
#N/D	#N/D	#N/D	ENSG00000169214
#N/D	#N/D	#N/D	ENSG00000239395
#N/D	#N/D	#N/D	ENSG00000241128
#N/D	#N/D	#N/D	ENSG00000153230
1,99	0,00	0,00	ENSG00000221888
1,14	0,97	#N/D	ENSG00000228336
#N/D	#N/D	#N/D	ENSG00000196772
#N/D	#N/D	#N/D	ENSG00000197591

0,76	0,00	0,01	ENSG00000162722
0,65	0,00	0,01	ENSG00000238243
0,61	0,14	0,23	ENSG00000177462
#N/D	#N/D	#N/D	ENSG00000177275
1,91	0,35	0,47	ENSG00000279263
1,60	0,51	0,62	ENSG00000187080
6,13	0,07	0,13	ENSG00000197454
1,44	0,68	0,77	ENSG00000203663
30,14	0,22	0,32	ENSG00000198128
6,85	0,00	0,00	ENSG00000196071
0,02	0,15	0,23	ENSG00000162727
0,13	0,46	0,56	ENSG00000198601
0,65	0,35	0,47	ENSG00000228198
0,01	0,05	0,10	ENSG00000171180
0,50	0,32	0,43	ENSG00000177212
1,14	0,97	#N/D	ENSG00000177201
#N/D	#N/D	#N/D	ENSG00000177186
#N/D	#N/D	#N/D	ENSG00000177174
22,89	0,26	0,37	ENSG00000196944
1,85	0,60	0,69	ENSG00000198104
#N/D	#N/D	#N/D	ENSG00000175143
#N/D	#N/D	#N/D	ENSG00000227152
#N/D	#N/D	#N/D	ENSG00000196240
#N/D	#N/D	#N/D	ENSG00000196539
#N/D	#N/D	#N/D	ENSG00000203661
8,39	0,46	0,56	ENSG00000188558
1,14	0,97	#N/D	ENSG00000182783
#N/D	#N/D	#N/D	ENSG00000183310
#N/D	#N/D	#N/D	ENSG00000184022
#N/D	#N/D	#N/D	ENSG00000279301
#N/D	#N/D	#N/D	ENSG00000177151
1,14	0,97	#N/D	ENSG00000187701
1,14	0,97	0,98	ENSG00000189181
1,14	0,97	0,98	ENSG00000286015
15,64	0,33	#N/D	ENSG00000259823
1,28	0,00	0,01	ENSG00000175137
1,05	0,56	0,66	ENSG00000171161
1,00	0,99	1,00	ENSG00000171163
0,73	0,03	0,06	ENSG00000185220
1,14	0,97	0,98	ENSG00000184731
1,35	0,00	0,01	ENSG00000035115
1,14	0,11	0,18	ENSG00000143727
0,60	0,47	0,58	ENSG00000189292
0,83	0,04	0,08	ENSG00000151353
22,89	0,26	0,37	ENSG00000280360
15,64	0,33	0,44	ENSG00000172554
0,05	0,24	0,35	ENSG00000115705
0,98	0,76	0,84	ENSG00000130508

2,80	0,20	0,30	ENSG00000186487
0,82	0,02	0,04	ENSG00000032389
0,89	0,15	0,24	ENSG00000171853
1,05	0,59	0,69	ENSG00000182551
0,24	0,00	0,00	ENSG00000286905
1,15	0,09	0,15	ENSG00000171865
1,01	0,86	0,93	ENSG00000171863
1,79	0,10	0,17	ENSG00000118004
1,14	0,97	#N/D	ENSG00000151360
15,64	0,33	0,44	ENSG00000214866
5.323,33	0,00	0,00	ENSG00000176887
2,00	0,00	0,00	ENSG00000134326
33,78	0,00	0,00	ENSG00000134321
1,67	0,00	0,00	ENSG00000151692
2,40	0,00	0,00	ENSG00000115738
1,13	0,15	0,23	ENSG00000134313
0,79	0,00	0,01	ENSG00000143797
0,68	0,00	0,00	ENSG00000151693
0,97	0,69	0,78	ENSG00000119185
0,77	0,00	0,00	ENSG00000119203
1,81	0,00	0,00	ENSG00000134330
1,19	0,04	0,07	ENSG00000151694
0,94	0,45	0,56	ENSG00000134308
0,64	0,00	0,00	ENSG00000115750
0,53	0,00	0,00	ENSG00000134317
1,69	0,00	0,00	ENSG00000172059
1,40	0,01	0,01	ENSG00000205795
1,24	0,01	0,02	ENSG00000171848
1,28	0,00	0,01	ENSG00000115756
0,61	0,00	0,00	ENSG00000115758
0,82	0,01	0,03	ENSG00000115761
2,91	0,00	0,00	ENSG00000143882
1,42	0,00	0,00	ENSG00000143870
1,10	0,39	0,50	ENSG00000162975
0,50	0,32	0,43	ENSG00000150873
1,54	0,00	0,00	ENSG00000162976
1,09	0,29	0,40	ENSG00000134318
0,61	0,00	0,00	ENSG00000169016
2,08	0,00	0,00	ENSG00000196208
#N/D	#N/D	#N/D	ENSG00000169006
3,27	0,00	0,00	ENSG00000134324
6,18	0,00	0,00	ENSG00000071575
0,39	0,00	0,00	ENSG00000162981
1,17	0,05	0,09	ENSG00000151779
0,79	0,00	0,01	ENSG00000079785
0,18	0,10	0,16	ENSG00000134323
1,14	0,97	#N/D	ENSG00000279663
5,14	0,00	0,00	ENSG00000197872

0,46	0,11	0,19	ENSG00000214842
3,65	0,00	0,00	ENSG00000163032
0,62	0,00	0,00	ENSG00000163029
0,48	0,00	0,00	ENSG00000178295
0,13	0,46	#N/D	ENSG00000151379
1,04	0,66	0,75	ENSG00000170745
0,70	0,00	0,00	ENSG00000240857
#N/D	#N/D	#N/D	ENSG00000250741
0,37	0,04	0,07	ENSG00000185013
0,22	0,00	0,00	ENSG00000143867
0,97	0,82	0,89	ENSG00000183891
0,39	0,00	0,00	ENSG00000118965
18,99	0,00	0,01	ENSG00000132031
0,97	0,74	0,82	ENSG00000068697
0,72	0,00	0,00	ENSG00000115884
0,78	0,00	0,01	ENSG00000055917
1,35	0,00	0,00	ENSG00000143878
1,10	0,25	0,36	ENSG00000118960
0,74	0,06	0,11	ENSG00000143869
0,82	0,04	0,07	ENSG00000118961
1,14	0,97	#N/D	ENSG00000084674
#N/D	#N/D	#N/D	ENSG00000280390
#N/D	#N/D	#N/D	ENSG00000218819
0,49	0,00	0,00	ENSG00000119771
0,26	0,08	0,14	ENSG00000279526
1,16	0,11	0,18	ENSG00000119778
0,61	0,00	0,00	ENSG00000173960
1,20	0,51	0,62	ENSG00000205639
0,91	0,28	0,39	ENSG00000163026
0,56	0,02	0,05	ENSG00000119782
0,70	0,00	0,00	ENSG00000115128
0,77	0,16	0,25	ENSG00000219626
1,27	0,11	0,19	ENSG00000115129
0,54	0,05	0,10	ENSG00000176732
1,26	0,58	0,68	ENSG00000276087
3,15	0,08	0,14	ENSG00000186453
0,66	0,00	0,00	ENSG00000198399
0,71	0,00	0,00	ENSG00000084676
0,93	0,44	0,56	ENSG00000184924
1,19	0,04	0,07	ENSG00000138092
0,69	0,00	0,00	ENSG00000138031
1,26	0,03	0,06	ENSG00000115137
0,62	0,00	0,00	ENSG00000084710
#N/D	#N/D	#N/D	ENSG00000115138
1,41	0,00	0,00	ENSG00000119772
0,96	0,66	0,75	ENSG00000138101
0,82	0,02	0,04	ENSG00000143970
0,80	0,01	0,02	ENSG00000084731

0,74	0,00	0,00	ENSG00000084733
0,55	0,00	0,00	ENSG00000157833
1,30	0,00	0,00	ENSG00000084754
1,40	0,00	0,00	ENSG00000138029
1,13	0,50	0,60	ENSG00000173567
0,91	0,28	0,38	ENSG00000138018
1,44	0,68	0,77	ENSG00000157856
0,22	0,14	0,22	ENSG00000115155
8,39	0,46	0,56	ENSG00000173557
15,64	0,33	#N/D	ENSG00000157884
2,66	0,00	0,00	ENSG00000171303
1,33	0,00	0,00	ENSG00000213699
1,04	0,61	0,71	ENSG00000115163
8,39	0,46	0,56	ENSG00000157851
0,54	0,00	0,00	ENSG00000084764
2,09	0,00	0,00	ENSG00000119777
1,22	0,01	0,03	ENSG00000084693
1,36	0,00	0,00	ENSG00000228474
0,85	0,04	0,08	ENSG00000138080
0,83	0,17	0,26	ENSG00000138030
1,33	0,00	0,01	ENSG00000138028
3,92	0,00	0,00	ENSG00000143994
1,81	0,00	0,00	ENSG00000138073
0,05	0,24	#N/D	ENSG00000186143
3,26	0,13	0,21	ENSG00000163792
0,43	0,00	0,00	ENSG00000138074
0,85	0,04	0,08	ENSG00000138085
0,92	0,28	0,38	ENSG00000084774
0,99	0,96	0,98	ENSG00000115194
0,13	0,46	0,56	ENSG00000163793
0,62	0,39	0,51	ENSG00000138100
1,19	0,34	0,45	ENSG00000163794
1,33	0,00	0,00	ENSG00000115204
1,14	0,10	0,17	ENSG00000115207
0,95	0,55	0,66	ENSG00000115211
1,03	0,73	0,81	ENSG00000115234
2,14	0,00	0,00	ENSG00000163795
0,83	0,02	0,04	ENSG00000115241
1,01	0,88	0,95	ENSG00000115216
1,35	0,15	0,23	ENSG00000157992
1,23	0,01	0,03	ENSG00000138002
2,47	0,00	0,00	ENSG00000115226
0,85	0,60	0,70	ENSG00000084734
0,50	0,00	0,00	ENSG00000221843
1,29	0,00	0,00	ENSG00000243943
0,50	0,00	0,00	ENSG00000176714
0,76	0,00	0,00	ENSG00000198522
0,76	0,00	0,00	ENSG00000119760

0,87	0,12	0,19	ENSG00000163798
0,74	0,01	0,01	ENSG00000243147
0,64	0,00	0,01	ENSG00000171174
0,56	0,00	0,00	ENSG00000158019
1,13	0,14	0,22	ENSG00000075426
0,32	0,00	0,00	ENSG00000163803
0,80	0,01	0,01	ENSG00000213639
0,14	0,05	0,09	ENSG00000163806
0,75	0,00	0,01	ENSG00000171103
0,80	0,01	0,01	ENSG00000163811
0,90	0,29	0,40	ENSG00000189350
0,26	0,08	0,14	ENSG00000179270
0,66	0,00	0,00	ENSG00000115295
2,97	0,00	0,00	ENSG00000171094
1,79	0,00	0,00	ENSG00000119801
2,40	0,00	0,00	ENSG00000213626
#N/D	#N/D	#N/D	ENSG00000280154
0,71	0,00	0,00	ENSG00000172954
0,02	0,11	0,18	ENSG00000278908
#N/D	#N/D	#N/D	ENSG00000162949
1,79	0,00	0,00	ENSG00000158089
211,42	0,05	0,09	ENSG00000214711
3,30	0,00	0,00	ENSG00000013016
0,96	0,76	0,84	ENSG00000158125
1,14	0,97	#N/D	ENSG00000277893
0,67	0,00	0,00	ENSG00000162959
0,86	0,11	0,18	ENSG00000162961
0,70	0,00	0,00	ENSG00000021574
0,61	0,00	0,00	ENSG00000152683
0,40	0,00	0,01	ENSG00000091106
0,73	0,00	0,00	ENSG00000119820
1,26	0,00	0,01	ENSG00000115760
1,94	0,01	0,02	ENSG00000279544
0,86	0,09	0,16	ENSG00000018699
0,28	0,00	0,00	ENSG00000049323
5,05	0,00	0,00	ENSG00000152689
1,65	0,00	0,00	ENSG00000119812
1,25	0,01	0,01	ENSG00000150938
0,88	0,13	0,20	ENSG00000171055
3,26	0,00	0,00	ENSG00000205221
0,95	0,54	0,64	ENSG00000115808
#N/D	#N/D	#N/D	ENSG00000279519
1,27	0,01	0,01	ENSG00000008869
1,06	0,52	0,63	ENSG00000152133
1,01	0,88	0,95	ENSG00000055332
0,13	0,46	#N/D	ENSG00000138068
0,97	0,74	0,82	ENSG00000218739
0,86	0,07	0,12	ENSG00000115816

0,92	0,36	0,47	ENSG00000003509
0,81	0,01	0,03	ENSG00000115825
2,61	0,03	0,06	ENSG00000115828
0,63	0,00	0,00	ENSG00000163171
0,45	0,01	0,02	ENSG00000115841
0,65	0,00	0,00	ENSG00000138061
0,23	0,05	0,10	ENSG00000280276
0,82	0,02	0,04	ENSG00000119787
1,13	0,14	0,22	ENSG00000143889
1,35	0,00	0,01	ENSG00000143891
1,05	0,56	0,67	ENSG00000115875
1,01	0,93	0,98	ENSG00000152147
0,71	0,00	0,00	ENSG00000163214
1,27	0,19	0,29	ENSG00000188010
0,70	0,39	0,51	ENSG00000214694
0,69	0,00	0,00	ENSG00000115904
0,29	0,12	0,19	ENSG00000205111
0,54	0,00	0,00	ENSG00000011566
0,70	0,06	0,11	ENSG00000152154
0,50	0,00	0,00	ENSG00000138050
1,15	0,38	0,49	ENSG00000183023
0,79	0,02	0,04	ENSG00000162878
0,72	0,00	0,00	ENSG00000143924
1,44	0,00	0,00	ENSG00000115944
0,55	0,04	0,07	ENSG00000171126
0,59	0,00	0,00	ENSG00000057935
0,09	0,00	0,00	ENSG00000162881
0,05	0,24	0,35	ENSG00000162882
1,04	0,64	0,74	ENSG00000152518
0,74	0,00	0,00	ENSG00000115970
2,08	0,00	0,00	ENSG00000152527
0,16	0,00	0,00	ENSG00000223658
0,58	0,00	0,00	ENSG00000138036
0,27	0,22	0,32	ENSG00000138075
0,49	0,08	0,15	ENSG00000143921
0,68	0,00	0,00	ENSG00000138095
0,84	0,07	0,12	ENSG00000138032
1,68	0,40	0,52	ENSG00000285542
3,13	0,00	0,00	ENSG00000138079
1,09	0,29	0,40	ENSG00000138078
0,76	0,03	0,05	ENSG00000143919
8,39	0,46	#N/D	ENSG00000278957
1,29	0,74	0,82	ENSG00000138083
0,58	0,00	0,00	ENSG00000170577
0,60	0,00	0,00	ENSG00000068784
0,69	0,00	0,00	ENSG00000171132
1,95	0,00	0,00	ENSG00000116016
0,05	0,24	#N/D	ENSG00000284701

0,46	0,00	0,00	ENSG00000250565
0,80	0,01	0,02	ENSG00000119729
1,24	0,03	0,05	ENSG00000151665
1,82	0,00	0,00	ENSG00000119878
1,83	0,02	0,04	ENSG00000279254
1,43	0,00	0,00	ENSG00000171150
1,78	0,00	0,00	ENSG00000180398
1,39	0,00	0,00	ENSG00000068724
0,19	0,03	0,05	ENSG00000239605
3,56	0,23	0,34	ENSG00000273269
0,60	0,00	0,00	ENSG00000143933
0,98	0,97	0,98	ENSG00000119888
1,16	0,07	0,13	ENSG00000095002
0,82	0,13	0,21	ENSG00000184261
0,95	0,50	0,60	ENSG00000116062
0,87	0,09	0,16	ENSG00000138081
0,88	0,16	0,24	ENSG00000170802
0,58	0,00	0,00	ENSG00000162869
1,24	0,02	0,04	ENSG00000243244
0,65	0,35	0,47	ENSG00000068781
1,14	0,97	#N/D	ENSG00000242441
0,07	0,31	#N/D	ENSG00000279956
8,39	0,46	0,56	ENSG00000138039
#N/D	#N/D	#N/D	ENSG00000170820
8,39	0,46	#N/D	ENSG00000179915
1,00	0,99	1,00	ENSG00000115239
0,39	0,00	0,00	ENSG00000143942
1,22	0,02	0,04	ENSG00000068912
0,94	0,68	0,77	ENSG00000119737
1,25	0,00	0,01	ENSG00000068878
0,72	0,01	0,03	ENSG00000170634
1,29	0,74	0,82	ENSG00000178021
1,14	0,97	0,98	ENSG00000177994
0,66	0,00	0,00	ENSG00000115306
0,49	0,00	0,00	ENSG00000214595
0,90	0,17	0,26	ENSG00000115310
0,59	0,00	0,00	ENSG00000162994
0,79	0,00	0,01	ENSG00000143947
0,68	0,00	0,00	ENSG00000085760
0,78	0,00	0,01	ENSG00000115355
0,97	0,75	0,83	ENSG00000163001
0,73	0,00	0,00	ENSG00000275052
0,50	0,00	0,00	ENSG00000138035
0,07	0,00	0,00	ENSG00000115380
8,39	0,46	0,56	ENSG00000055813
0,47	0,00	0,00	ENSG00000028116
0,60	0,00	0,00	ENSG00000115392
1,45	0,48	0,58	ENSG00000119866

0,74	0,00	0,00	ENSG00000115421
0,56	0,00	0,00	ENSG00000162924
0,83	0,14	0,22	ENSG00000162927
1,28	0,00	0,01	ENSG00000162928
0,35	0,00	0,00	ENSG00000162929
0,72	0,02	0,04	ENSG00000237651
0,83	0,02	0,04	ENSG00000115464
0,67	0,00	0,00	ENSG00000082898
0,64	0,00	0,00	ENSG00000170264
0,86	0,05	0,10	ENSG00000115484
0,95	0,58	0,68	ENSG00000173163
0,93	0,43	0,55	ENSG00000170340
1,08	0,66	0,75	ENSG00000186889
0,71	0,00	0,00	ENSG00000115504
0,95	0,68	0,77	ENSG00000115507
0,94	0,56	0,67	ENSG00000143951
5,32	0,01	0,02	ENSG00000279485
0,99	0,90	0,96	ENSG00000014641
0,89	0,18	0,28	ENSG00000169764
0,58	0,00	0,00	ENSG00000143952
0,52	0,00	0,00	ENSG00000197329
0,87	0,13	0,21	ENSG00000119862
1,06	0,51	0,61	ENSG00000119844
0,89	0,14	0,23	ENSG00000179833
0,38	0,00	0,00	ENSG00000115902
0,67	0,00	0,00	ENSG00000011523
1,30	0,00	0,00	ENSG00000138069
0,97	0,73	0,82	ENSG00000138071
0,92	0,32	0,43	ENSG00000198369
0,05	0,24	#N/D	ENSG00000280257
0,43	0,00	0,00	ENSG00000143995
0,43	0,00	0,00	ENSG00000143971
0,78	0,02	0,05	ENSG00000197223
0,84	0,07	0,12	ENSG00000243667
0,92	0,59	0,69	ENSG00000273398
0,80	0,01	0,02	ENSG00000115946
0,94	0,44	0,55	ENSG00000221823
0,71	0,00	0,00	ENSG00000119865
0,29	0,12	0,19	ENSG00000115956
0,50	0,00	0,00	ENSG00000204923
0,39	0,00	0,00	ENSG00000169621
0,13	0,46	0,56	ENSG00000169618
0,13	0,00	0,00	ENSG00000163219
0,04	0,20	0,30	ENSG00000163217
0,13	0,46	0,56	ENSG00000183607
1,14	0,97	#N/D	ENSG00000169605
0,87	0,09	0,15	ENSG00000169604
1,67	0,00	0,00	ENSG00000198380

0,66	0,00	0,00	ENSG00000169599
0,85	0,04	0,08	ENSG00000115977
0,59	0,00	0,00	ENSG00000196975
1,06	0,60	0,70	ENSG00000087338
0,97	0,72	0,81	ENSG00000124380
1,10	0,28	0,39	ENSG00000059728
0,73	0,12	0,19	ENSG00000244617
1,03	0,67	0,76	ENSG00000169564
0,73	0,00	0,00	ENSG00000115998
0,85	0,07	0,12	ENSG00000116001
0,71	0,00	0,00	ENSG00000116005
0,59	0,00	0,00	ENSG00000143977
0,78	0,00	0,01	ENSG00000035141
0,56	0,00	0,00	ENSG00000163235
0,41	0,00	0,00	ENSG00000075340
#N/D	#N/D	#N/D	ENSG00000183733
4,42	0,15	0,24	ENSG00000152672
2,53	0,17	0,26	ENSG00000116031
0,84	0,19	0,29	ENSG00000116035
1,60	0,01	0,02	ENSG00000116039
0,60	0,00	0,00	ENSG00000258881
1,13	0,13	0,21	ENSG00000144043
3,42	0,00	0,00	ENSG00000144031
2,06	0,00	0,00	ENSG00000124357
2,03	0,00	0,00	ENSG00000124370
1,17	0,09	0,16	ENSG00000124383
4,23	0,00	0,00	ENSG00000124374
0,74	0,00	0,00	ENSG00000075292
0,70	0,01	0,02	ENSG00000135636
0,65	0,00	0,00	ENSG00000003137
1,06	0,50	0,61	ENSG00000144036
0,63	0,00	0,00	ENSG00000116096
0,98	0,99	0,99	ENSG00000135638
0,81	0,04	0,07	ENSG00000144040
1,52	0,00	0,00	ENSG00000135631
0,98	0,97	0,98	ENSG00000214513
1,05	0,57	0,67	ENSG00000135632
2,19	0,00	0,00	ENSG00000135617
0,79	0,00	0,01	ENSG00000135624
0,97	0,70	0,79	ENSG00000163013
1,14	0,97	0,98	ENSG00000135625
1,10	0,25	0,36	ENSG00000116127
8,39	0,46	#N/D	ENSG00000144035
0,13	0,46	#N/D	ENSG00000204872
0,91	0,50	0,61	ENSG00000144034
2,74	0,00	0,00	ENSG00000280037
1,22	0,04	0,07	ENSG00000144048
2,35	0,30	0,41	ENSG00000187833

1,00	0,97	0,98	ENSG00000124356
0,43	0,22	0,32	ENSG00000163017
0,92	0,32	0,43	ENSG00000114956
0,78	0,00	0,01	ENSG00000187605
8,39	0,46	0,56	ENSG00000279181
0,73	0,00	0,00	ENSG00000163170
0,81	0,01	0,02	ENSG00000114978
0,48	0,00	0,00	ENSG00000065911
#N/D	#N/D	#N/D	ENSG00000279201
0,98	0,97	0,98	ENSG00000264324
1,27	0,17	0,26	ENSG00000188687
1,01	0,86	0,93	ENSG00000204843
3,44	0,01	0,03	ENSG00000159239
0,74	0,02	0,03	ENSG00000284308
0,85	0,06	0,10	ENSG00000005448
0,74	0,00	0,00	ENSG00000114993
1,27	0,01	0,03	ENSG00000115274
1,76	0,00	0,00	ENSG00000274049
1,61	0,00	0,00	ENSG00000239779
1,12	0,16	0,25	ENSG00000115275
1,00	0,97	0,98	ENSG00000204822
1,03	0,79	0,86	ENSG00000135637
0,90	0,23	0,33	ENSG00000115282
0,94	0,65	0,74	ENSG00000179528
1,06	0,50	0,60	ENSG00000115289
1,73	0,32	0,44	ENSG00000115297
2,56	0,00	0,01	ENSG00000144045
1,13	0,12	0,20	ENSG00000115307
1,04	0,62	0,71	ENSG00000115317
0,81	0,08	0,14	ENSG00000115318
0,70	0,00	0,00	ENSG00000115325
1,44	0,15	0,24	ENSG00000159374
0,99	0,93	0,98	ENSG00000135622
1,91	0,00	0,00	ENSG00000159399
1,35	0,00	0,00	ENSG00000115350
1,99	0,00	0,00	ENSG00000115353
2,13	0,00	0,00	ENSG00000115363
0,91	0,26	0,37	ENSG00000115364
0,63	0,00	0,00	ENSG00000005436
15,64	0,33	0,44	ENSG00000176204
#N/D	#N/D	#N/D	ENSG00000143954
#N/D	#N/D	#N/D	ENSG00000172023
#N/D	#N/D	#N/D	ENSG00000115386
#N/D	#N/D	#N/D	ENSG00000172016
0,29	0,12	0,19	ENSG00000066032
8,39	0,46	#N/D	ENSG00000162951
0,99	0,86	0,92	ENSG00000163541
6,13	0,07	0,13	ENSG00000115423

0,23	0,00	0,00	ENSG00000186854
1,69	0,00	0,00	ENSG00000034510
1,11	0,20	0,30	ENSG00000176407
1,67	0,00	0,00	ENSG00000152284
1,10	0,22	0,33	ENSG00000152291
1,32	0,00	0,00	ENSG00000042445
1,07	0,46	0,56	ENSG00000115459
0,84	0,04	0,07	ENSG00000042493
0,62	0,03	0,06	ENSG00000152292
1,80	0,00	0,00	ENSG00000168906
1,91	0,00	0,00	ENSG00000115486
0,87	0,57	0,67	ENSG00000118640
0,79	0,01	0,03	ENSG00000168899
1,26	0,01	0,02	ENSG00000168894
0,80	0,04	0,08	ENSG00000168890
0,86	0,07	0,13	ENSG00000168883
0,93	0,36	0,47	ENSG00000168887
0,14	0,00	0,00	ENSG00000168878
0,27	0,22	0,32	ENSG00000115523
0,50	0,00	0,00	ENSG00000168874
1,37	0,00	0,01	ENSG00000115525
0,75	0,00	0,00	ENSG00000068654
0,62	0,00	0,00	ENSG00000132300
0,90	0,18	0,28	ENSG00000132305
0,83	0,03	0,06	ENSG00000132313
1,16	0,63	0,73	ENSG00000068615
1,15	0,10	0,17	ENSG00000115548
0,99	0,92	0,98	ENSG00000115561
0,46	0,00	0,00	ENSG00000249884
0,79	0,01	0,02	ENSG00000239305
0,85	0,04	0,08	ENSG00000153561
1,14	0,97	0,98	ENSG00000153563
1,14	0,97	#N/D	ENSG00000172116
1,89	0,45	0,56	ENSG00000187627
0,97	0,94	0,98	ENSG00000183281
#N/D	#N/D	#N/D	ENSG00000233999
0,80	0,44	0,56	ENSG00000125551
0,15	0,00	0,00	ENSG00000185304
0,36	0,00	0,00	ENSG00000172086
1,14	0,97	0,98	ENSG00000115593
#N/D	#N/D	#N/D	ENSG00000163586
1,83	0,00	0,00	ENSG00000144115
0,99	1,00	1,00	ENSG00000214336
#N/D	#N/D	#N/D	ENSG00000172073
1,94	0,00	0,00	ENSG00000172071
0,71	0,00	0,00	ENSG00000153574
1,14	0,97	#N/D	ENSG00000211592
#N/D	#N/D	#N/D	ENSG00000211593

#N/D	#N/D	#N/D	ENSG00000211594
#N/D	#N/D	#N/D	ENSG00000211595
#N/D	#N/D	#N/D	ENSG00000211596
#N/D	#N/D	#N/D	ENSG00000211597
#N/D	#N/D	#N/D	ENSG00000211598
#N/D	#N/D	#N/D	ENSG00000211599
#N/D	#N/D	#N/D	ENSG00000243466
#N/D	#N/D	#N/D	ENSG00000239855
#N/D	#N/D	#N/D	ENSG00000243063
#N/D	#N/D	#N/D	ENSG00000240671
#N/D	#N/D	#N/D	ENSG00000241755
#N/D	#N/D	#N/D	ENSG00000241351
#N/D	#N/D	#N/D	ENSG00000243290
#N/D	#N/D	#N/D	ENSG00000244437
#N/D	#N/D	#N/D	ENSG00000240864
#N/D	#N/D	#N/D	ENSG00000240382
#N/D	#N/D	#N/D	ENSG00000239951
#N/D	#N/D	#N/D	ENSG00000211611
#N/D	#N/D	#N/D	ENSG00000241294
#N/D	#N/D	#N/D	ENSG00000244575
#N/D	#N/D	#N/D	ENSG00000244116
8,39	0,46	#N/D	ENSG00000243238
#N/D	#N/D	#N/D	ENSG00000242076
#N/D	#N/D	#N/D	ENSG00000239862
#N/D	#N/D	#N/D	ENSG00000242371
#N/D	#N/D	#N/D	ENSG00000273962
#N/D	#N/D	#N/D	ENSG00000251039
1,14	0,97	#N/D	ENSG00000251546
#N/D	#N/D	#N/D	ENSG00000250036
#N/D	#N/D	#N/D	ENSG00000239975
1,14	0,97	#N/D	ENSG00000239571
#N/D	#N/D	#N/D	ENSG00000243264
#N/D	#N/D	#N/D	ENSG00000242534
#N/D	#N/D	#N/D	ENSG00000211623
#N/D	#N/D	#N/D	ENSG00000241566
#N/D	#N/D	#N/D	ENSG00000225523
#N/D	#N/D	#N/D	ENSG00000211625
#N/D	#N/D	#N/D	ENSG00000211626
#N/D	#N/D	#N/D	ENSG00000242766
#N/D	#N/D	#N/D	ENSG00000241244
#N/D	#N/D	#N/D	ENSG00000224041
#N/D	#N/D	#N/D	ENSG00000276566
#N/D	#N/D	#N/D	ENSG00000278857
#N/D	#N/D	#N/D	ENSG00000211632
#N/D	#N/D	#N/D	ENSG00000211633
#N/D	#N/D	#N/D	ENSG00000242580
1,14	0,97	#N/D	ENSG00000239819
#N/D	#N/D	#N/D	ENSG00000228325

0,99	1,00	1,00	ENSG00000259916
2,07	0,01	0,01	ENSG00000163060
51,90	0,15	0,24	ENSG00000172005
1,05	0,53	0,64	ENSG00000144029
0,00	0,02	0,03	ENSG00000289685
1,06	0,52	0,62	ENSG00000144026
0,96	0,70	0,79	ENSG00000275111
0,41	0,00	0,00	ENSG00000233757
2,23	0,00	0,00	ENSG00000155066
0,89	0,24	0,34	ENSG00000115041
1,12	0,16	0,25	ENSG00000115042
1,14	0,97	#N/D	ENSG00000144010
8,39	0,46	#N/D	ENSG00000144015
0,82	0,03	0,06	ENSG00000174501
2,80	0,20	0,30	ENSG00000186281
9,56	0,02	0,05	ENSG00000274286
1,50	0,17	0,26	ENSG00000188886
0,61	0,00	0,00	ENSG00000158050
0,71	0,00	0,00	ENSG00000084090
1,24	0,01	0,02	ENSG00000135956
0,89	0,15	0,24	ENSG00000144021
1,01	0,93	0,98	ENSG00000144028
0,81	0,02	0,05	ENSG00000198885
0,85	0,05	0,10	ENSG00000121152
0,56	0,20	0,30	ENSG00000163121
1,32	0,00	0,01	ENSG00000196843
0,89	0,16	0,25	ENSG00000114982
2,13	0,05	0,10	ENSG00000249715
0,80	0,01	0,02	ENSG00000114988
1,44	0,00	0,00	ENSG00000158158
1,39	0,00	0,00	ENSG00000168763
1,21	0,14	0,22	ENSG00000163126
1,03	0,76	0,84	ENSG00000213337
0,84	0,04	0,07	ENSG00000168758
0,13	0,46	#N/D	ENSG00000168754
0,50	0,00	0,00	ENSG00000144199
0,41	0,27	0,38	ENSG00000279791
0,77	0,00	0,01	ENSG00000135976
1,01	0,94	0,98	ENSG00000196912
0,81	0,01	0,02	ENSG00000135940
1,32	0,00	0,00	ENSG00000115073
1,33	0,54	0,64	ENSG00000228486
1,14	0,97	0,98	ENSG00000115085
0,80	0,01	0,02	ENSG00000075568
0,01	0,09	0,16	ENSG00000168658
0,13	0,46	0,56	ENSG00000144191
0,90	0,20	0,30	ENSG00000040933
0,83	0,06	0,11	ENSG00000183513

0,94	0,50	0,60	ENSG00000115446
4,39	0,00	0,00	ENSG00000071073
1,14	0,97	0,98	ENSG00000196872
0,84	0,58	0,68	ENSG00000135951
0,72	0,15	0,24	ENSG00000241962
0,49	0,20	0,30	ENSG00000273045
0,63	0,01	0,02	ENSG00000144182
0,16	0,07	0,12	ENSG00000273155
1,00	0,97	0,99	ENSG00000158411
0,82	0,02	0,04	ENSG00000185414
0,13	0,46	0,56	ENSG00000185674
0,38	0,00	0,00	ENSG00000144214
1,16	0,12	0,20	ENSG00000115514
0,94	0,48	0,58	ENSG00000158417
0,96	0,62	0,71	ENSG00000135945
0,42	0,00	0,00	ENSG00000144218
0,21	0,00	0,00	ENSG00000170500
0,48	0,00	0,00	ENSG00000115526
#N/D	#N/D	#N/D	ENSG00000204640
0,66	0,00	0,00	ENSG00000115539
0,20	0,00	0,00	ENSG00000170485
0,92	0,29	0,40	ENSG00000071082
1,44	0,00	0,00	ENSG00000204634
0,69	0,00	0,00	ENSG00000158435
0,78	0,00	0,01	ENSG00000163162
0,08	0,00	0,00	ENSG00000175874
1,32	0,44	0,56	ENSG00000196460
0,50	0,00	0,00	ENSG00000071054
2,71	0,36	0,48	ENSG00000115590
1,18	0,06	0,10	ENSG00000115594
1,14	0,97	0,98	ENSG00000115598
1,14	0,97	0,98	ENSG00000115602
3,33	0,03	0,06	ENSG00000115604
1,14	0,97	0,98	ENSG00000115607
1,14	0,97	0,98	ENSG00000180251
66,40	0,13	0,21	ENSG00000115616
0,74	0,01	0,02	ENSG00000135953
0,88	0,35	0,46	ENSG00000170417
#N/D	#N/D	#N/D	ENSG00000279904
0,12	0,00	0,00	ENSG00000198914
0,67	0,00	0,00	ENSG00000135972
1,14	0,97	#N/D	ENSG00000135973
0,57	0,00	0,00	ENSG00000135966
1,20	0,04	0,08	ENSG00000135974
1,07	0,39	0,50	ENSG00000115641
0,77	0,00	0,01	ENSG00000071051
#N/D	#N/D	#N/D	ENSG00000119147
0,95	0,55	0,65	ENSG00000115652

0,55	0,04	0,07	ENSG00000153165
15,64	0,33	#N/D	ENSG00000254126
69,56	0,00	0,00	ENSG00000144057
1,16	0,32	0,43	ENSG00000196862
1,14	0,97	#N/D	ENSG00000115665
0,53	0,58	0,68	ENSG00000196228
0,01	0,04	0,08	ENSG00000198203
2,71	0,36	0,48	ENSG00000198075
1,09	0,30	0,41	ENSG00000135968
0,80	0,01	0,02	ENSG00000169756
0,77	0,00	0,00	ENSG00000153201
0,87	0,25	0,36	ENSG00000163006
0,97	0,94	0,98	ENSG00000135960
0,49	0,00	0,00	ENSG00000172985
0,94	0,48	0,59	ENSG00000186522
1,06	0,52	0,62	ENSG00000198142
0,40	0,00	0,00	ENSG00000015568
1,77	0,00	0,01	ENSG00000256977
5,28	0,10	0,17	ENSG00000284337
4,71	0,00	0,00	ENSG00000144063
0,95	0,66	0,76	ENSG00000144061
0,74	0,00	0,00	ENSG00000175701
0,13	0,46	0,56	ENSG00000257207
0,04	0,00	0,00	ENSG00000256671
1,90	0,00	0,00	ENSG00000183054
0,83	0,02	0,04	ENSG00000169679
1,17	0,80	0,87	ENSG00000153093
0,87	0,20	0,30	ENSG00000153094
#N/D	#N/D	#N/D	ENSG00000293584
0,74	0,00	0,00	ENSG00000153107
1,32	0,05	0,10	ENSG00000153208
1,06	0,49	0,60	ENSG00000153214
0,26	0,08	0,14	ENSG00000144152
1,14	0,97	0,98	ENSG00000278962
0,61	0,00	0,00	ENSG00000144161
1,04	0,67	0,76	ENSG00000188177
0,56	0,00	0,00	ENSG00000169629
0,61	0,00	0,00	ENSG00000114999
0,88	0,12	0,20	ENSG00000125630
0,90	0,23	0,33	ENSG00000125611
1,45	0,00	0,00	ENSG00000144136
1,77	0,00	0,00	ENSG00000144130
0,67	0,00	0,00	ENSG00000169607
0,02	0,00	0,00	ENSG00000115008
0,21	0,00	0,00	ENSG00000125538
1,79	0,01	0,02	ENSG00000280228
3,46	0,05	0,10	ENSG00000125571
1,29	0,63	0,73	ENSG00000136688

1,14	0,97	#N/D	ENSG00000136694
#N/D	#N/D	#N/D	ENSG00000136696
1,14	0,97	#N/D	ENSG00000136695
#N/D	#N/D	#N/D	ENSG00000136697
1,03	0,93	0,98	ENSG00000136689
1,16	0,19	0,29	ENSG00000125637
0,83	0,05	0,10	ENSG00000125618
0,62	0,39	0,51	ENSG00000231292
0,90	0,34	0,45	ENSG00000136682
1,11	0,84	0,91	ENSG00000184492
1,55	0,09	0,16	ENSG00000279267
2,97	0,00	0,00	ENSG00000144134
1,16	0,07	0,12	ENSG00000115084
0,84	0,03	0,06	ENSG00000115091
0,67	0,65	0,74	ENSG00000279957
#N/D	#N/D	#N/D	ENSG00000175497
1,07	0,42	0,53	ENSG00000088205
0,86	0,07	0,13	ENSG00000125633
0,61	0,01	0,02	ENSG00000279227
1,41	0,00	0,00	ENSG00000125629
1,87	0,00	0,00	ENSG00000163064
1,14	0,97	#N/D	ENSG00000019169
15,64	0,33	0,44	ENSG00000144119
2,00	0,00	0,00	ENSG00000115107
1,22	0,12	0,20	ENSG00000186132
1,36	0,00	0,00	ENSG00000155368
1,40	0,22	0,32	ENSG00000171227
8,39	0,46	0,56	ENSG00000080293
1,14	0,97	0,98	ENSG00000163075
0,47	0,00	0,00	ENSG00000144120
0,58	0,00	0,00	ENSG00000088179
0,76	0,00	0,01	ENSG00000115109
0,93	0,39	0,51	ENSG00000226479
1,44	0,00	0,00	ENSG00000144118
1,13	0,75	0,83	ENSG00000163083
1,14	0,97	#N/D	ENSG00000280409
0,55	0,00	0,00	ENSG00000074047
0,93	0,50	0,60	ENSG00000115112
0,98	0,84	0,91	ENSG00000074054
1,92	0,23	0,34	ENSG00000279721
0,87	0,12	0,20	ENSG00000155438
0,95	0,55	0,65	ENSG00000211460
#N/D	#N/D	#N/D	ENSG00000279874
#N/D	#N/D	#N/D	ENSG00000155052
1,13	0,15	0,24	ENSG00000136732
#N/D	#N/D	#N/D	ENSG00000237524
0,66	0,00	0,00	ENSG00000136717
0,82	0,06	0,11	ENSG00000186684

0,98	0,77	0,85	ENSG00000163161
1,15	0,09	0,15	ENSG00000169967
1,26	0,36	0,47	ENSG00000115718
0,94	0,42	0,54	ENSG00000163166
6,07	0,00	0,00	ENSG00000169994
0,64	0,06	0,10	ENSG00000072163
0,53	0,58	0,68	ENSG00000144230
0,59	0,00	0,00	ENSG00000136709
1,14	0,97	0,98	ENSG00000173349
0,76	0,00	0,00	ENSG00000144231
1,11	0,19	0,29	ENSG00000144233
1,20	0,03	0,05	ENSG00000136715
0,85	0,04	0,07	ENSG00000136731
1,83	0,00	0,00	ENSG00000136720
1,14	0,97	#N/D	ENSG00000222014
1,54	0,00	0,00	ENSG00000196604
1,32	0,06	0,10	ENSG00000152076
1,35	0,00	0,00	ENSG00000136699
1,04	0,67	0,76	ENSG00000152082
1,05	0,91	0,97	ENSG00000152086
1,11	0,25	0,36	ENSG00000136710
1,15	0,08	0,14	ENSG00000136718
1,35	0,00	0,00	ENSG00000072135
2,28	0,00	0,00	ENSG00000196834
#N/D	#N/D	#N/D	ENSG00000152093
#N/D	#N/D	#N/D	ENSG00000136698
0,32	0,00	0,01	ENSG00000222038
0,07	0,31	#N/D	ENSG00000173302
1,14	0,97	#N/D	ENSG00000178171
0,37	0,00	0,00	ENSG00000136002
1,83	0,00	0,00	ENSG00000284479
1,14	0,11	0,18	ENSG00000152102
1,29	0,00	0,00	ENSG00000115762
2,20	0,00	0,00	ENSG00000188219
1,14	0,97	#N/D	ENSG00000233087
1,03	0,74	0,82	ENSG00000173272
0,63	0,04	0,07	ENSG00000075886
1,02	0,90	0,96	ENSG00000163040
#N/D	#N/D	#N/D	ENSG00000163046
0,27	0,00	0,00	ENSG00000183840
1,44	0,02	0,05	ENSG00000150551
0,52	0,00	0,00	ENSG00000176771
8,39	0,46	0,56	ENSG00000279512
1,15	0,08	0,14	ENSG00000152127
0,66	0,42	0,54	ENSG00000152128
22,89	0,26	0,37	ENSG00000153086
0,99	0,90	0,96	ENSG00000082258
2,22	0,24	0,35	ENSG00000176601

0,83	0,02	0,04	ENSG00000115839
0,68	0,00	0,00	ENSG00000121988
0,74	0,00	0,00	ENSG00000048991
0,85	0,05	0,09	ENSG00000144224
0,02	0,11	0,18	ENSG00000115850
0,75	0,00	0,00	ENSG00000076003
1,16	0,07	0,13	ENSG00000115866
5,63	0,01	0,01	ENSG00000121966
#N/D	#N/D	#N/D	ENSG00000279024
1,14	0,97	0,98	ENSG00000144229
0,38	0,14	0,23	ENSG00000150540
0,90	0,27	0,38	ENSG00000144228
0,07	0,01	0,02	ENSG00000144227
0,15	0,00	0,00	ENSG00000168702
0,43	0,00	0,00	ENSG00000115919
1,14	0,97	0,98	ENSG00000075884
0,82	0,06	0,10	ENSG00000121964
0,24	0,00	0,00	ENSG00000169554
0,22	0,00	0,00	ENSG00000279166
#N/D	#N/D	#N/D	ENSG00000279876
0,56	0,00	0,00	ENSG00000121989
0,56	0,00	0,00	ENSG00000115947
1,34	0,00	0,00	ENSG00000204406
1,14	0,97	0,98	ENSG00000281469
8,39	0,46	#N/D	ENSG00000281772
0,58	0,00	0,00	ENSG00000135999
0,65	0,00	0,00	ENSG00000168280
30,14	0,22	0,32	ENSG00000150556
0,30	0,00	0,00	ENSG00000187123
1,13	0,13	0,21	ENSG00000168288
0,65	0,00	0,00	ENSG00000115963
1,05	0,70	0,79	ENSG00000184898
0,28	0,00	0,00	ENSG00000123609
0,30	0,00	0,00	ENSG00000123610
0,82	0,01	0,03	ENSG00000080345
0,36	0,02	0,05	ENSG00000183091
0,98	0,84	0,91	ENSG00000162980
15,64	0,33	0,44	ENSG00000283228
1,60	0,51	0,62	ENSG00000182389
1,19	0,05	0,08	ENSG00000115145
0,62	0,00	0,00	ENSG00000157827
0,72	0,00	0,00	ENSG00000196504
0,81	0,01	0,03	ENSG00000177917
#N/D	#N/D	#N/D	ENSG00000177519
835,00	0,01	0,03	ENSG00000144278
8,39	0,46	#N/D	ENSG00000162989
2,91	0,00	0,00	ENSG00000153234
0,77	0,00	0,00	ENSG00000115159

1,47	0,00	0,00	ENSG00000136542
1,14	0,97	0,98	ENSG00000136541
1,14	0,97	0,98	ENSG00000115165
3,04	0,02	0,04	ENSG00000123612
1,27	0,00	0,01	ENSG00000115170
1,14	0,97	#N/D	ENSG00000007001
0,70	0,55	0,65	ENSG00000153237
0,67	0,00	0,00	ENSG00000144283
1,05	0,91	0,97	ENSG00000163331
0,83	0,02	0,04	ENSG00000115183
0,51	0,00	0,00	ENSG00000196151
0,77	0,00	0,01	ENSG00000123636
0,85	0,05	0,10	ENSG00000136536
0,95	0,60	0,70	ENSG00000241399
1,14	0,97	0,98	ENSG00000248672
51,90	0,15	0,24	ENSG00000054219
1,17	0,10	0,16	ENSG00000153246
0,21	0,04	0,07	ENSG00000115221
1,12	0,15	0,24	ENSG00000153250
#N/D	#N/D	#N/D	ENSG00000279844
0,67	0,00	0,00	ENSG00000136560
0,66	0,00	0,00	ENSG00000115233
0,50	0,25	0,36	ENSG00000136535
0,51	0,42	0,54	ENSG00000144290
1,14	0,97	0,98	ENSG00000280105
0,73	0,11	0,18	ENSG00000197635
1,14	0,97	#N/D	ENSG00000115263
0,39	0,00	0,00	ENSG00000078098
1,50	0,00	0,00	ENSG00000115267
4,70	0,00	0,00	ENSG00000115271
#N/D	#N/D	#N/D	ENSG00000184611
0,68	0,00	0,00	ENSG00000182263
0,27	0,00	0,00	ENSG00000115290
2,96	0,00	0,00	ENSG00000082438
0,13	0,46	#N/D	ENSG00000169507
8,54	0,00	0,00	ENSG00000153253
0,35	0,00	0,01	ENSG00000136531
0,58	0,00	0,00	ENSG00000178662
1,45	0,00	0,00	ENSG00000115339
0,74	0,00	0,00	ENSG00000123607
0,13	0,46	0,56	ENSG00000144285
0,06	0,00	0,00	ENSG00000169432
1,14	0,97	#N/D	ENSG00000136546
1,14	0,97	0,98	ENSG00000163092
0,11	0,00	0,00	ENSG00000172318
0,61	0,00	0,00	ENSG00000198648
0,84	0,04	0,07	ENSG00000172292
1,25	0,27	0,38	ENSG00000163072

0,56	0,00	0,00	ENSG00000152253
#N/D	#N/D	#N/D	ENSG00000152254
0,41	0,27	0,38	ENSG00000073734
0,98	0,97	0,98	ENSG00000073737
8,39	0,46	0,56	ENSG00000081479
0,78	0,01	0,03	ENSG00000163093
1,14	0,97	0,98	ENSG00000251569
0,78	0,53	0,63	ENSG00000239474
0,71	0,00	0,00	ENSG00000138399
0,95	0,59	0,69	ENSG00000138398
0,86	0,76	0,84	ENSG00000154479
0,51	0,05	0,10	ENSG00000144362
0,37	0,00	0,00	ENSG00000213160
0,54	0,00	0,00	ENSG00000138385
0,77	0,00	0,01	ENSG00000138382
0,96	0,64	0,73	ENSG00000144357
0,03	0,17	0,26	ENSG00000071909
0,46	0,00	0,00	ENSG00000204335
0,67	0,04	0,08	ENSG00000204334
0,66	0,09	0,15	ENSG00000128683
1,23	0,01	0,02	ENSG00000115806
0,56	0,00	0,00	ENSG00000198586
0,58	0,00	0,00	ENSG00000123600
0,71	0,00	0,00	ENSG00000115827
1,82	0,00	0,00	ENSG00000071967
1,05	0,54	0,64	ENSG00000077380
0,54	0,00	0,00	ENSG00000115840
0,81	0,02	0,04	ENSG00000128708
0,57	0,00	0,00	ENSG00000172878
0,86	0,12	0,20	ENSG00000144355
0,82	0,09	0,15	ENSG00000115844
1,79	0,00	0,00	ENSG00000091409
1,14	0,97	#N/D	ENSG00000278924
2,36	0,00	0,00	ENSG00000152256
1,07	0,62	0,71	ENSG00000091428
1,23	0,01	0,02	ENSG00000091436
0,56	0,00	0,00	ENSG00000144354
1,13	0,14	0,23	ENSG00000172845
0,90	0,21	0,31	ENSG00000138430
2,00	0,00	0,01	ENSG00000280414
1,07	0,74	0,82	ENSG00000217236
1,19	0,07	0,13	ENSG00000138433
0,70	0,00	0,00	ENSG00000144306
3,14	0,00	0,00	ENSG00000163328
1,14	0,97	0,98	ENSG00000279884
1,03	0,73	0,82	ENSG00000115935
1,14	0,97	0,98	ENSG00000138435
0,48	0,00	0,00	ENSG00000128656

1,16	0,07	0,12	ENSG00000115966
0,91	0,26	0,37	ENSG00000154518
1,28	0,00	0,01	ENSG00000144320
5,28	0,10	0,17	ENSG00000174279
1,19	0,31	0,43	ENSG00000128714
2,53	0,03	0,05	ENSG00000170178
22,96	0,00	0,00	ENSG00000128713
21,21	0,00	0,00	ENSG00000128710
1,58	0,00	0,00	ENSG00000128709
1,03	0,78	0,86	ENSG00000175879
1,56	0,15	0,24	ENSG00000128652
0,43	0,00	0,00	ENSG00000170166
4,62	0,00	0,00	ENSG00000128645
0,02	0,11	0,18	ENSG00000279160
0,49	0,00	0,00	ENSG00000128654
0,34	0,18	0,27	ENSG00000279205
0,81	0,01	0,02	ENSG00000170144
1,17	0,05	0,09	ENSG00000116044
0,44	0,00	0,00	ENSG00000018510
0,57	0,00	0,00	ENSG00000196659
0,91	0,45	0,56	ENSG00000197557
6,86	0,00	0,00	ENSG00000128655
0,67	0,00	0,00	ENSG00000155636
0,31	0,00	0,00	ENSG00000079156
0,84	0,04	0,08	ENSG00000180228
0,48	0,00	0,00	ENSG00000204311
1,16	0,14	0,22	ENSG00000079150
1,01	0,94	0,98	ENSG00000116095
0,62	0,00	0,00	ENSG00000155657
0,88	0,64	0,73	ENSG00000279598
1,17	0,80	0,87	ENSG00000163492
0,96	0,63	0,73	ENSG00000187231
0,07	0,00	0,00	ENSG00000144331
0,59	0,00	0,00	ENSG00000163510
#N/D	#N/D	#N/D	ENSG00000279559
1,62	0,00	0,00	ENSG00000170035
0,69	0,00	0,00	ENSG00000115232
37,39	0,19	0,29	ENSG00000188452
1,14	0,97	#N/D	ENSG00000162992
1,93	0,00	0,00	ENSG00000138434
1,15	0,74	0,82	ENSG00000150722
3,56	0,23	0,34	ENSG00000115252
0,63	0,00	0,00	ENSG00000077232
0,67	0,32	0,43	ENSG00000162998
0,70	0,00	0,00	ENSG00000061676
0,79	0,09	0,15	ENSG00000162999
0,55	0,00	0,00	ENSG00000163002
0,74	0,00	0,01	ENSG00000170396

0,77	0,37	0,49	ENSG00000188738
0,69	0,00	0,00	ENSG00000065548
1,02	0,82	0,89	ENSG00000138448
0,68	0,00	0,00	ENSG00000144369
0,13	0,46	#N/D	ENSG00000163012
0,78	0,61	0,70	ENSG00000064989
0,61	0,00	0,00	ENSG00000003436
1,14	0,19	0,28	ENSG00000144366
0,64	0,00	0,00	ENSG00000168542
1,44	0,00	0,00	ENSG00000204262
0,66	0,00	0,00	ENSG00000115368
1,32	0,22	0,32	ENSG00000138449
0,65	0,00	0,00	ENSG00000138381
1,14	0,97	0,98	ENSG00000286053
1,14	0,97	0,98	ENSG00000286165
0,97	0,88	0,94	ENSG00000151687
0,58	0,00	0,00	ENSG00000128694
0,92	0,33	0,45	ENSG00000128699
0,67	0,00	0,00	ENSG00000064933
0,77	0,15	0,24	ENSG00000187699
2,22	0,24	0,35	ENSG00000138379
0,89	0,23	0,34	ENSG00000198130
0,72	0,00	0,00	ENSG00000151689
0,60	0,00	0,00	ENSG00000151690
0,49	0,00	0,00	ENSG00000189362
0,77	0,00	0,00	ENSG00000138386
1,30	0,00	0,00	ENSG00000115419
1,34	0,00	0,00	ENSG00000115415
0,66	0,02	0,04	ENSG00000138378
0,98	0,99	0,99	ENSG00000280083
0,86	0,05	0,10	ENSG00000128641
0,85	0,05	0,09	ENSG00000173559
0,48	0,00	0,00	ENSG00000168497
0,02	0,00	0,00	ENSG00000144339
1,25	0,01	0,02	ENSG00000196950
0,50	0,01	0,02	ENSG00000118997
0,96	0,65	0,75	ENSG00000081320
0,43	0,00	0,00	ENSG00000138411
0,76	0,02	0,04	ENSG00000144395
0,74	0,00	0,00	ENSG00000119041
0,67	0,65	0,74	ENSG00000187944
8,39	0,46	0,56	ENSG00000280176
0,70	0,00	0,00	ENSG00000197121
0,90	0,30	0,41	ENSG00000065413
1,24	0,01	0,02	ENSG00000115524
1,35	0,00	0,00	ENSG00000115520
0,73	0,00	0,00	ENSG00000144381
0,70	0,00	0,00	ENSG00000115541

1,05	0,63	0,72	ENSG00000270757
0,69	0,00	0,00	ENSG00000115540
2,75	0,00	0,00	ENSG00000162944
0,64	0,00	0,00	ENSG00000247626
0,07	0,31	0,43	ENSG00000152430
2,71	0,36	0,48	ENSG00000115896
0,78	0,00	0,01	ENSG00000119042
1,78	0,01	0,02	ENSG00000226124
1,10	0,32	0,43	ENSG00000178074
0,83	0,07	0,13	ENSG00000162971
0,65	0,00	0,00	ENSG00000162972
1,10	0,25	0,36	ENSG00000196141
0,63	0,00	0,00	ENSG00000155729
0,82	0,03	0,06	ENSG00000163535
0,41	0,00	0,00	ENSG00000138356
1,38	0,00	0,00	ENSG00000082153
1,37	0,00	0,00	ENSG00000013441
0,97	0,73	0,82	ENSG00000240344
0,86	0,08	0,14	ENSG00000196290
0,78	0,00	0,01	ENSG00000115942
2,29	0,00	0,00	ENSG00000155744
1,11	0,27	0,38	ENSG00000119013
0,83	0,02	0,05	ENSG00000003402
0,65	0,00	0,00	ENSG00000003400
0,43	0,00	0,00	ENSG00000064012
0,18	0,00	0,00	ENSG00000155749
0,64	0,00	0,00	ENSG00000115993
0,53	0,00	0,00	ENSG00000082146
0,07	0,31	0,43	ENSG00000155754
0,68	0,00	0,00	ENSG00000155755
0,57	0,00	0,00	ENSG00000082126
0,95	0,54	0,64	ENSG00000003393
0,54	0,01	0,01	ENSG00000138395
0,62	0,00	0,00	ENSG00000155760
0,13	0,46	0,56	ENSG00000182329
0,80	0,01	0,02	ENSG00000116030
0,76	0,00	0,00	ENSG00000055044
1,15	0,10	0,16	ENSG00000204217
#N/D	#N/D	#N/D	ENSG00000289490
0,60	0,00	0,00	ENSG00000138439
0,35	0,00	0,00	ENSG00000163596
0,63	0,00	0,00	ENSG00000138442
0,55	0,00	0,00	ENSG00000138380
1,18	0,06	0,11	ENSG00000144426
0,54	0,00	0,00	ENSG00000119004
1,13	0,13	0,21	ENSG00000138443
1,22	0,01	0,03	ENSG00000173166
0,98	0,97	0,99	ENSG00000178562

1,14	0,97	#N/D	ENSG00000163599
1,14	0,97	#N/D	ENSG00000163600
0,24	0,00	0,00	ENSG00000116117
0,13	0,00	0,00	ENSG00000118257
0,77	0,00	0,01	ENSG00000114933
0,96	0,65	0,75	ENSG00000023228
0,86	0,06	0,11	ENSG00000114942
0,05	0,00	0,00	ENSG00000183671
0,72	0,00	0,00	ENSG00000204186
0,61	0,00	0,00	ENSG00000114948
#N/D	#N/D	#N/D	ENSG00000235118
#N/D	#N/D	#N/D	ENSG00000232125
5,54	0,02	0,04	ENSG00000138400
0,62	0,00	0,00	ENSG00000118246
1,85	0,60	0,69	ENSG00000144410
1,49	0,00	0,00	ENSG00000118263
0,98	0,82	0,89	ENSG00000118260
0,93	0,48	0,58	ENSG00000144401
1,33	0,00	0,00	ENSG00000163249
1,11	0,28	0,39	ENSG00000163251
0,82	0,02	0,04	ENSG00000178385
1,14	0,97	#N/D	ENSG00000118231
0,13	0,46	#N/D	ENSG00000163254
#N/D	#N/D	#N/D	ENSG00000182187
#N/D	#N/D	#N/D	ENSG00000168582
#N/D	#N/D	#N/D	ENSG00000188674
0,74	0,00	0,00	ENSG00000138413
1,36	0,00	0,00	ENSG00000115020
0,05	0,24	0,35	ENSG00000144407
0,70	0,01	0,03	ENSG00000078018
0,54	0,22	0,32	ENSG00000144406
0,80	0,01	0,02	ENSG00000197713
0,63	0,00	0,00	ENSG00000144445
0,29	0,03	0,05	ENSG00000115361
1,14	0,97	0,98	ENSG00000168530
0,97	0,67	0,76	ENSG00000115365
0,56	0,00	0,00	ENSG00000021826
0,22	0,00	0,00	ENSG00000178568
2,84	0,00	0,00	ENSG00000030419
0,90	0,37	0,48	ENSG00000144451
1,89	0,45	0,56	ENSG00000174453
0,77	0,00	0,01	ENSG00000138376
0,20	0,00	0,00	ENSG00000144452
0,87	0,09	0,16	ENSG00000138363
1,41	0,00	0,00	ENSG00000115414
0,53	0,00	0,00	ENSG00000118242
1,16	0,14	0,22	ENSG00000115425
0,61	0,04	0,08	ENSG00000163449

1,04	0,64	0,73	ENSG00000079246
1,43	0,00	0,00	ENSG00000279348
0,04	0,00	0,00	ENSG00000144583
0,82	0,02	0,04	ENSG00000138375
0,94	0,43	0,55	ENSG00000197756
0,32	0,00	0,00	ENSG00000115457
69,38	-	-	ENSG00000115461
8,39	0,46	#N/D	ENSG00000118245
0,82	0,01	0,02	ENSG00000079308
#N/D	#N/D	#N/D	ENSG00000188282
1,14	0,97	#N/D	ENSG00000180871
8,39	0,46	#N/D	ENSG00000163464
1,07	0,42	0,54	ENSG00000163466
2,01	0,00	0,01	ENSG00000179921
1,07	0,42	0,54	ENSG00000127837
1,05	0,56	0,66	ENSG00000127838
1,27	0,00	0,01	ENSG00000135926
1,14	0,97	0,98	ENSG00000158428
1,14	0,42	0,53	ENSG00000018280
1,19	0,03	0,06	ENSG00000144579
1,31	0,35	0,46	ENSG00000127831
1,37	0,00	0,00	ENSG00000135913
1,17	0,06	0,10	ENSG00000144580
0,97	0,91	0,97	ENSG00000115556
1,03	0,70	0,79	ENSG00000115568
0,86	0,08	0,13	ENSG00000074582
1,11	0,24	0,35	ENSG00000163481
1,07	0,41	0,52	ENSG00000163482
0,69	0,00	0,00	ENSG00000135912
0,89	0,23	0,33	ENSG00000135929
#N/D	#N/D	#N/D	ENSG00000115592
8,74	0,00	0,00	ENSG00000115596
0,51	0,42	0,54	ENSG00000135925
0,13	0,00	0,00	ENSG00000171450
0,07	0,31	0,43	ENSG00000163497
8,39	0,46	#N/D	ENSG00000163499
2,71	0,36	0,48	ENSG00000181378
0,27	0,22	0,32	ENSG00000163501
0,88	0,16	0,25	ENSG00000187736
1,62	0,12	0,19	ENSG00000280537
0,92	0,74	0,82	ENSG00000213901
0,87	0,11	0,19	ENSG00000115649
1,28	0,00	0,01	ENSG00000144567
1,44	0,00	0,00	ENSG00000158552
1,58	0,00	0,00	ENSG00000115657
1,33	0,00	0,01	ENSG00000284820
1,09	0,29	0,41	ENSG00000198925
1,69	0,00	0,00	ENSG00000163516

0,94	0,58	0,68	ENSG00000163521
1,08	0,37	0,49	ENSG00000115661
1,06	0,48	0,58	ENSG00000127824
0,60	0,47	0,58	ENSG00000243910
1,62	0,00	0,00	ENSG00000135924
2,71	0,36	0,48	ENSG00000054356
#N/D	#N/D	#N/D	ENSG00000182698
0,77	0,00	0,00	ENSG00000123992
0,98	0,93	0,98	ENSG00000175084
1,14	0,97	#N/D	ENSG00000280323
0,79	0,00	0,01	ENSG00000072195
0,70	0,55	0,65	ENSG00000286143
1,28	0,28	0,39	ENSG00000286095
2,26	0,00	0,00	ENSG00000144591
4,13	0,00	0,00	ENSG00000072182
1,64	0,00	0,00	ENSG00000123989
1,34	0,02	0,05	ENSG00000188760
1,11	0,21	0,32	ENSG00000124006
0,93	0,54	0,64	ENSG00000123999
1,23	0,02	0,03	ENSG00000144589
1,07	0,42	0,54	ENSG00000114923
0,55	0,00	0,00	ENSG00000116106
0,84	0,06	0,11	ENSG00000135903
0,01	0,07	0,13	ENSG00000288658
0,19	0,00	0,00	ENSG00000163082
0,90	0,19	0,29	ENSG00000116120
22,89	0,26	0,37	ENSG00000124003
1,80	0,00	0,00	ENSG00000123983
7,02	0,00	0,00	ENSG00000152049
0,13	0,00	0,00	ENSG00000171951
0,51	0,00	0,00	ENSG00000152056
0,49	0,00	0,00	ENSG00000286239
0,90	0,19	0,29	ENSG00000085449
0,94	0,46	0,56	ENSG00000135900
0,95	0,49	0,60	ENSG00000135919
2,71	0,36	0,48	ENSG00000124019
0,72	0,00	0,00	ENSG00000036257
#N/D	#N/D	#N/D	ENSG00000283428
0,55	0,00	0,00	ENSG00000135905
0,26	0,08	0,14	ENSG00000144460
1,14	0,97	#N/D	ENSG00000279015
0,95	0,53	0,64	ENSG00000169047
0,76	0,00	0,00	ENSG00000144468
0,20	0,00	0,00	ENSG00000081052
0,38	0,00	0,00	ENSG00000169031
0,99	0,93	0,98	ENSG00000168958
0,76	0,59	0,69	ENSG00000168955
#N/D	#N/D	#N/D	ENSG00000284629

0,72	0,00	0,00	ENSG00000173744
#N/D	#N/D	#N/D	ENSG00000284643
#N/D	#N/D	#N/D	ENSG00000284622
#N/D	#N/D	#N/D	ENSG00000284704
#N/D	#N/D	#N/D	ENSG00000284631
#N/D	#N/D	#N/D	ENSG00000284667
0,06	0,00	0,00	ENSG00000135917
#N/D	#N/D	#N/D	ENSG00000284725
#N/D	#N/D	#N/D	ENSG00000284718
#N/D	#N/D	#N/D	ENSG00000284635
0,29	0,03	0,05	ENSG00000115009
0,02	0,11	0,18	ENSG00000123977
0,50	0,25	0,36	ENSG00000153820
0,47	0,00	0,00	ENSG00000153823
2,36	0,00	0,00	ENSG00000187957
1,08	0,32	0,43	ENSG00000153827
1,50	0,00	0,00	ENSG00000153832
2,32	0,03	0,06	ENSG00000163053
2,09	0,00	0,00	ENSG00000135899
0,73	0,37	0,48	ENSG00000079263
1,05	0,57	0,68	ENSG00000185404
1,84	0,12	0,20	ENSG00000279964
1,17	0,05	0,10	ENSG00000067066
1,53	0,00	0,00	ENSG00000135932
1,27	0,00	0,01	ENSG00000135916
1,74	0,00	0,00	ENSG00000135898
#N/D	#N/D	#N/D	ENSG00000173699
0,83	0,10	0,17	ENSG00000204128
0,77	0,00	0,00	ENSG00000173692
0,81	0,41	0,53	ENSG00000135914
1,50	0,00	0,00	ENSG00000135931
1,57	0,01	0,02	ENSG00000156966
0,60	0,00	0,00	ENSG00000115053
1,93	0,13	0,21	ENSG00000171596
0,53	0,58	0,68	ENSG00000177673
1,91	0,00	0,00	ENSG00000187514
0,79	0,01	0,02	ENSG00000156973
1,02	0,77	0,85	ENSG00000144524
1,29	0,74	0,82	ENSG00000163273
0,90	0,20	0,30	ENSG00000144535
0,98	0,99	0,99	ENSG00000163283
0,53	0,58	0,68	ENSG00000163286
1,14	0,97	#N/D	ENSG00000163295
1,14	0,97	#N/D	ENSG00000171551
1,14	0,97	#N/D	ENSG00000237412
0,62	0,08	0,14	ENSG00000135902
1,86	0,09	0,15	ENSG00000196811
1,24	0,02	0,03	ENSG00000221944

1,28	0,00	0,01	ENSG00000135930
6,38	0,00	0,00	ENSG00000115468
1,32	0,00	0,00	ENSG00000204120
1,71	0,00	0,01	ENSG00000115474
1,26	0,07	0,12	ENSG00000182600
1,02	0,84	0,91	ENSG00000066248
0,04	0,20	0,30	ENSG00000115488
0,84	0,75	0,83	ENSG00000168918
1,17	0,06	0,11	ENSG00000085978
#N/D	#N/D	#N/D	ENSG00000130561
0,83	0,02	0,04	ENSG00000077044
0,69	0,00	0,00	ENSG00000085982
1,14	0,97	0,98	ENSG00000242366
1,14	0,97	0,98	ENSG00000242515
#N/D	#N/D	#N/D	ENSG00000241119
4,90	0,00	0,00	ENSG00000244122
1,42	0,34	0,45	ENSG00000167165
0,02	0,15	0,23	ENSG00000288705
0,03	0,17	0,26	ENSG00000244474
1,60	0,51	0,62	ENSG00000288702
0,33	0,00	0,00	ENSG00000241635
1,44	0,68	0,77	ENSG00000185038
1,39	0,00	0,00	ENSG00000123485
4,52	0,00	0,00	ENSG00000144481
#N/D	#N/D	#N/D	ENSG00000072080
#N/D	#N/D	#N/D	ENSG00000279218
1,30	0,00	0,00	ENSG00000188042
1,01	0,94	0,98	ENSG00000130147
0,84	0,04	0,07	ENSG00000157985
0,73	0,08	0,13	ENSG00000168505
0,34	0,10	0,16	ENSG00000182177
4,42	0,15	0,24	ENSG00000132321
0,54	0,00	0,00	ENSG00000144476
1,45	0,00	0,00	ENSG00000198612
1,58	0,00	0,00	ENSG00000163359
1,93	0,19	0,29	ENSG00000115648
15,64	0,33	#N/D	ENSG00000071677
0,44	0,05	0,10	ENSG00000124839
0,87	0,10	0,16	ENSG00000124831
0,54	0,10	0,18	ENSG00000177483
2,69	0,00	0,00	ENSG00000132329
0,94	0,45	0,56	ENSG00000184182
1,04	0,89	0,96	ENSG00000258984
0,41	0,00	0,00	ENSG00000132330
0,31	0,04	0,07	ENSG00000144488
0,09	0,00	0,00	ENSG00000168427
0,70	0,00	0,00	ENSG00000178752
0,89	0,22	0,32	ENSG00000132323

1,11	0,52	0,62	ENSG00000144485
0,96	0,61	0,71	ENSG00000132326
0,64	0,00	0,00	ENSG00000204104
1,01	0,90	0,96	ENSG00000065802
0,97	0,76	0,84	ENSG00000233608
1,03	0,72	0,81	ENSG00000068024
1,14	0,11	0,18	ENSG00000130414
1,14	0,97	#N/D	ENSG00000182083
#N/D	#N/D	#N/D	ENSG00000178586
1,46	0,00	0,00	ENSG00000172428
15,64	0,33	#N/D	ENSG00000178602
1,25	0,01	0,01	ENSG00000063660
0,93	0,43	0,55	ENSG00000144504
1,04	0,73	0,82	ENSG00000188542
1,43	0,00	0,00	ENSG00000142327
1,25	0,01	0,02	ENSG00000142330
1,49	0,01	0,03	ENSG00000178623
0,13	0,46	#N/D	ENSG00000185176
#N/D	#N/D	#N/D	ENSG00000184945
0,16	0,07	0,12	ENSG00000130294
7,85	0,04	0,07	ENSG00000172482
1,57	0,32	0,43	ENSG00000172478
0,17	0,00	0,00	ENSG00000226321
1,86	0,00	0,00	ENSG00000162804
1,09	0,33	0,45	ENSG00000122085
1,11	0,22	0,32	ENSG00000115687
1,07	0,42	0,53	ENSG00000115685
1,47	0,01	0,02	ENSG00000146205
1,51	0,00	0,00	ENSG00000115677
1,37	0,00	0,00	ENSG00000168385
1,17	0,05	0,10	ENSG00000006607
1,16	0,06	0,11	ENSG00000115694
0,60	0,00	0,00	ENSG00000176720
0,87	0,08	0,14	ENSG00000176946
1,47	0,00	0,00	ENSG00000168397
1,35	0,00	0,00	ENSG00000168393
1,72	0,00	0,00	ENSG00000168395
1,14	0,11	0,19	ENSG00000180902
1,92	0,00	0,00	ENSG00000154252
1,20	0,51	0,62	ENSG00000204099
0,98	0,99	0,99	ENSG00000188389
0,05	0,24	0,35	ENSG00000188011
37,39	0,19	0,29	ENSG00000216921
5,44	0,00	0,00	ENSG00000280119
4.554,73	0,00	0,01	ENSG00000134121
30,14	0,22	0,32	ENSG00000134115
6,13	0,07	0,13	ENSG00000144619
15,64	0,33	#N/D	ENSG00000091181

1,11	0,28	0,39	ENSG00000072756
0,89	0,23	0,34	ENSG00000113851
1,23	0,03	0,05	ENSG00000144455
15,64	0,33	0,44	ENSG00000175928
1,03	0,83	0,90	ENSG00000170364
2,10	0,00	0,00	ENSG00000150995
3,53	0,00	0,00	ENSG00000134107
0,85	0,08	0,14	ENSG00000134108
1,42	0,00	0,00	ENSG00000134109
8,39	0,46	#N/D	ENSG00000196277
2,00	0,00	0,00	ENSG00000071282
0,72	0,32	0,43	ENSG00000125046
1,21	0,77	0,85	ENSG00000182533
0,82	0,35	0,46	ENSG00000180914
1,05	0,55	0,65	ENSG00000070950
0,87	0,30	0,41	ENSG00000196220
0,84	0,04	0,08	ENSG00000134077
1,31	0,00	0,00	ENSG00000168137
0,00	0,03	0,06	ENSG00000156959
1,06	0,49	0,59	ENSG00000163719
1,44	0,68	0,77	ENSG00000144550
1,38	0,00	0,00	ENSG00000156983
1,22	0,04	0,07	ENSG00000114026
0,89	0,34	0,45	ENSG00000134072
0,93	0,35	0,46	ENSG00000171148
1,11	0,21	0,31	ENSG00000241553
2,10	0,00	0,00	ENSG00000250151
1,24	0,01	0,03	ENSG00000214021
0,68	0,00	0,00	ENSG00000156990
5,28	0,10	0,17	ENSG00000187288
0,84	0,04	0,08	ENSG00000171135
1,51	0,05	0,09	ENSG00000163701
1,56	0,00	0,00	ENSG00000163702
10,08	0,00	0,00	ENSG00000288550
2,57	0,00	0,00	ENSG00000163703
0,57	0,00	0,00	ENSG00000163704
1,16	0,09	0,15	ENSG00000125037
0,73	0,00	0,00	ENSG00000144554
0,11	0,03	0,05	ENSG00000163705
1,56	0,00	0,00	ENSG00000254999
0,80	0,01	0,01	ENSG00000134086
0,55	0,00	0,00	ENSG00000134070
1,20	0,03	0,06	ENSG00000157014
#N/D	#N/D	#N/D	ENSG00000289763
1,46	0,00	0,00	ENSG00000272410
1,12	0,64	0,74	ENSG00000157017
1,53	0,00	0,00	ENSG00000157020
0,07	0,00	0,01	ENSG00000157087

0,05	0,00	0,00	ENSG00000132164
0,07	0,31	0,43	ENSG00000157103
0,56	0,00	0,00	ENSG00000196639
0,78	0,00	0,01	ENSG00000197548
1,93	0,00	0,00	ENSG00000144560
0,89	0,28	0,39	ENSG00000144559
0,14	0,05	0,09	ENSG00000157152
0,38	0,00	0,00	ENSG00000157150
0,69	0,00	0,00	ENSG00000132170
0,47	0,00	0,00	ENSG00000154743
0,86	0,76	0,84	ENSG00000225526
1,32	0,00	0,00	ENSG00000075975
1,12	0,15	0,23	ENSG00000132155
4,03	0,00	0,00	ENSG00000088726
1,81	0,00	0,00	ENSG00000144712
1,14	0,97	#N/D	ENSG00000289809
1,07	0,41	0,52	ENSG00000144713
0,99	0,92	0,98	ENSG00000144711
0,34	0,00	0,00	ENSG00000132182
1,19	0,07	0,13	ENSG00000163517
0,65	0,00	0,00	ENSG00000163520
#N/D	#N/D	#N/D	ENSG00000154764
0,70	0,00	0,00	ENSG00000163528
0,73	0,00	0,00	ENSG00000170876
1,14	0,97	0,98	ENSG00000268279
0,70	0,00	0,00	ENSG00000154767
1,42	0,00	0,00	ENSG00000170860
2,81	0,00	0,00	ENSG00000131389
1,17	0,58	0,68	ENSG00000144596
1,08	0,42	0,54	ENSG00000154781
0,04	0,20	0,30	ENSG00000131379
8,39	0,46	0,56	ENSG00000154783
0,97	0,69	0,78	ENSG00000177463
1,01	0,94	0,98	ENSG00000131368
1,25	0,01	0,02	ENSG00000131381
0,88	0,13	0,21	ENSG00000131375
0,76	0,00	0,01	ENSG00000131370
0,96	0,64	0,73	ENSG00000206562
1,10	0,26	0,37	ENSG00000144597
1,14	0,97	0,98	ENSG00000293553
0,48	0,01	0,03	ENSG00000206561
0,87	0,15	0,24	ENSG00000131373
1,02	0,83	0,90	ENSG00000169814
0,56	0,00	0,00	ENSG00000206560
0,71	0,35	0,46	ENSG00000279465
0,04	0,00	0,00	ENSG00000131386
1,08	0,45	0,56	ENSG00000154813
1,04	0,66	0,76	ENSG00000154814

1,98	0,00	0,00	ENSG00000131378
0,13	0,46	#N/D	ENSG00000092345
0,54	0,00	0,00	ENSG00000154822
1,13	0,13	0,21	ENSG00000131374
4.511,22	0,00	0,01	ENSG00000182568
1,14	0,97	#N/D	ENSG00000183960
0,82	0,74	0,82	ENSG00000163576
1,12	0,20	0,30	ENSG00000144566
0,66	0,42	0,54	ENSG00000183977
0,95	0,62	0,72	ENSG00000114166
0,74	0,00	0,00	ENSG00000129810
0,33	0,00	0,00	ENSG00000151789
0,51	0,00	0,00	ENSG00000182247
0,66	0,00	0,00	ENSG00000170142
0,66	0,00	0,00	ENSG00000197885
1,02	0,80	0,87	ENSG00000174748
0,72	0,00	0,00	ENSG00000174738
1,08	0,42	0,54	ENSG00000151090
1,20	0,11	0,18	ENSG00000077092
0,82	0,01	0,03	ENSG00000077097
0,67	0,00	0,00	ENSG00000151092
0,47	0,00	0,00	ENSG00000151093
1,14	0,97	#N/D	ENSG00000179796
1,45	0,03	0,05	ENSG00000163491
3,67	0,00	0,00	ENSG00000033867
1,03	0,90	0,96	ENSG00000163508
0,76	0,04	0,08	ENSG00000187118
0,63	0,00	0,00	ENSG00000163512
1,20	0,59	0,68	ENSG00000206559
1,08	0,82	0,89	ENSG00000283563
1,29	0,00	0,01	ENSG00000144642
0,63	0,00	0,00	ENSG00000163513
8,39	0,46	0,56	ENSG00000144644
0,79	0,00	0,01	ENSG00000163527
0,63	0,00	0,00	ENSG00000144645
8,39	0,46	0,56	ENSG00000197385
0,54	0,00	0,00	ENSG00000152642
0,72	0,04	0,08	ENSG00000170293
0,39	0,00	0,00	ENSG00000153551
0,93	0,40	0,51	ENSG00000091317
0,84	0,04	0,08	ENSG00000144635
0,69	0,00	0,00	ENSG00000182973
15,64	0,33	0,44	ENSG00000206557
15,64	0,33	0,44	ENSG00000183813
0,90	0,21	0,31	ENSG00000170266
0,88	0,20	0,30	ENSG00000188167
1,11	0,18	0,28	ENSG00000170275
0,51	0,00	0,00	ENSG00000173705

1,42	0,00	0,00	ENSG00000153558
0,95	0,52	0,62	ENSG00000153560
0,50	0,00	0,00	ENSG00000163539
0,89	0,16	0,25	ENSG00000170248
37,39	0,19	0,29	ENSG00000172995
0,38	0,00	0,00	ENSG00000144681
1,14	0,97	0,98	ENSG00000163673
0,13	0,46	0,56	ENSG00000281100
0,63	0,00	0,00	ENSG00000168016
1,43	0,00	0,00	ENSG00000178567
0,75	0,00	0,00	ENSG00000076242
0,67	0,00	0,00	ENSG00000093167
0,97	0,70	0,78	ENSG00000144674
1,07	0,79	0,87	ENSG00000144668
1,21	0,02	0,04	ENSG00000144677
1,20	0,26	0,37	ENSG00000136059
1,98	0,00	0,00	ENSG00000187091
1,17	0,43	0,54	ENSG00000008226
1,20	0,05	0,09	ENSG00000060971
0,49	0,00	0,00	ENSG00000172936
1,02	0,81	0,89	ENSG00000172939
0,70	0,15	0,24	ENSG00000172940
0,86	0,76	0,84	ENSG00000144671
0,61	0,00	0,00	ENSG00000093217
0,66	0,00	0,00	ENSG00000114739
0,67	0,00	0,00	ENSG00000157036
3,56	0,00	0,00	ENSG00000183873
#N/D	#N/D	#N/D	ENSG00000185313
15,64	0,33	0,44	ENSG00000168356
0,78	0,01	0,01	ENSG00000114742
1,30	0,00	0,00	ENSG00000114745
1,30	0,04	0,08	ENSG00000168026
2,44	0,00	0,00	ENSG00000144655
0,02	0,11	0,18	ENSG00000168334
#N/D	#N/D	#N/D	ENSG00000168329
1,14	0,97	#N/D	ENSG00000179934
0,97	0,77	0,85	ENSG00000144659
1,37	0,00	0,00	ENSG00000168028
0,74	0,69	0,78	ENSG00000168314
0,54	0,22	0,32	ENSG00000170011
0,96	0,64	0,73	ENSG00000114784
#N/D	#N/D	#N/D	ENSG00000291315
0,41	0,27	0,38	ENSG00000168032
0,93	0,35	0,46	ENSG00000188846
0,70	0,00	0,00	ENSG00000177873
1,11	0,62	0,71	ENSG00000177842
0,83	0,03	0,06	ENSG00000172888
1,00	0,95	0,98	ENSG00000168036

0,89	0,33	0,44	ENSG00000168038
0,88	0,11	0,18	ENSG00000182606
0,30	0,01	0,01	ENSG00000187094
1,14	0,97	0,98	ENSG00000157093
4,42	0,15	0,24	ENSG00000114812
0,99	0,92	0,98	ENSG00000093183
0,97	0,75	0,84	ENSG00000008324
1,33	0,00	0,00	ENSG00000114857
0,93	0,42	0,54	ENSG00000114853
30,14	0,22	0,32	ENSG00000157119
1,14	0,97	#N/D	ENSG00000010282
1,14	0,97	#N/D	ENSG00000244607
0,95	0,68	0,77	ENSG00000280571
0,78	0,01	0,01	ENSG00000181061
4,17	0,06	0,11	ENSG00000144648
1,14	0,97	#N/D	ENSG00000273291
#N/D	#N/D	#N/D	ENSG00000290317
0,13	0,46	#N/D	ENSG00000180432
1,14	0,97	0,98	ENSG00000182983
1,14	0,97	0,98	ENSG00000240747
2,34	0,06	0,11	ENSG00000144649
1,02	0,82	0,89	ENSG00000144647
1,40	0,00	0,00	ENSG00000163788
1,13	0,15	0,23	ENSG00000160746
0,76	0,01	0,02	ENSG00000011198
0,39	0,00	0,00	ENSG00000280435
1,14	0,97	#N/D	ENSG00000173769
0,74	0,00	0,01	ENSG00000179152
1,16	0,07	0,13	ENSG00000185219
0,90	0,32	0,43	ENSG00000178917
1,17	0,80	0,87	ENSG00000196345
0,15	0,01	0,02	ENSG00000144792
0,82	0,03	0,06	ENSG00000186448
0,89	0,27	0,38	ENSG00000169981
1,14	0,97	0,98	ENSG00000196653
1,14	0,97	0,98	ENSG00000186446
0,85	0,07	0,12	ENSG00000163807
0,84	0,05	0,10	ENSG00000163808
0,64	0,00	0,00	ENSG00000169964
0,62	0,39	0,51	ENSG00000163810
0,90	0,23	0,34	ENSG00000163812
0,79	0,01	0,03	ENSG00000075914
4,93	0,00	0,00	ENSG00000163815
0,80	0,05	0,10	ENSG00000163814
1,11	0,21	0,31	ENSG00000249992
0,83	0,02	0,05	ENSG00000011376
1,43	0,00	0,00	ENSG00000144791
1,01	0,87	0,93	ENSG00000211456

6,13	0,07	0,13	ENSG00000279017
1,14	0,97	0,98	ENSG00000163817
0,72	0,00	0,01	ENSG00000163818
2,35	0,30	0,41	ENSG00000173585
1,09	0,30	0,42	ENSG00000163820
1,75	0,00	0,00	ENSG00000172215
1,14	0,97	#N/D	ENSG00000173578
1,14	0,97	0,98	ENSG00000183625
1,14	0,97	0,98	ENSG00000163823
1,14	0,97	0,98	ENSG00000121807
1,14	0,97	0,98	ENSG00000160791
0,15	0,00	0,00	ENSG00000121797
0,00	0,01	0,02	ENSG00000012223
0,12	0,03	0,07	ENSG00000163825
0,13	0,46	0,56	ENSG00000163827
1,14	0,97	0,98	ENSG00000241186
#N/D	#N/D	#N/D	ENSG00000283877
1,14	0,97	#N/D	ENSG00000283473
1,30	0,00	0,01	ENSG00000178038
1,14	0,97	0,98	ENSG00000181585
4,42	0,15	0,24	ENSG00000206549
0,98	0,96	0,98	ENSG00000283706
0,40	0,12	0,19	ENSG00000160808
0,29	0,00	0,00	ENSG00000160801
1,22	0,03	0,06	ENSG00000160799
0,57	0,00	0,00	ENSG00000160796
0,73	0,00	0,00	ENSG00000181555
1,02	0,85	0,92	ENSG00000088727
0,81	0,02	0,04	ENSG00000114648
0,69	0,03	0,06	ENSG00000280173
1,11	0,19	0,28	ENSG00000076201
1,03	0,76	0,84	ENSG00000114650
0,81	0,02	0,04	ENSG00000163832
0,68	0,03	0,05	ENSG00000114646
0,67	0,00	0,00	ENSG00000173473
0,83	0,02	0,04	ENSG00000132153
1,13	0,13	0,22	ENSG00000047849
0,89	0,18	0,27	ENSG00000164045
#N/D	#N/D	#N/D	ENSG00000164047
1,13	0,21	0,32	ENSG00000164048
0,75	0,00	0,01	ENSG00000172113
#N/D	#N/D	#N/D	ENSG00000229453
1,85	0,60	0,69	ENSG00000164049
1,00	0,99	0,99	ENSG00000164050
1,05	0,63	0,73	ENSG00000164051
0,93	0,51	0,62	ENSG00000232112
0,78	0,01	0,02	ENSG00000164053
2,12	0,00	0,00	ENSG00000213689

0,98	0,82	0,89	ENSG00000164054
2,31	0,00	0,00	ENSG00000114268
0,82	0,25	0,36	ENSG00000145040
0,94	0,50	0,61	ENSG00000114270
1,41	0,00	0,00	ENSG00000010256
1,85	0,60	0,69	ENSG00000183396
1,69	0,00	0,00	ENSG00000225697
0,87	0,12	0,19	ENSG00000008300
1,58	0,00	0,00	ENSG00000213672
1,01	0,95	0,98	ENSG00000068745
0,91	0,23	0,33	ENSG00000114302
1,85	0,00	0,00	ENSG00000178537
0,82	0,02	0,04	ENSG00000177479
0,19	0,01	0,02	ENSG00000178467
1,32	0,00	0,00	ENSG00000178252
1,11	0,24	0,35	ENSG00000178149
1,03	0,73	0,81	ENSG00000178057
0,93	0,35	0,46	ENSG00000178035
1,56	0,00	0,00	ENSG00000290315
0,90	0,18	0,27	ENSG00000198218
1,53	0,00	0,00	ENSG00000172053
0,98	0,79	0,86	ENSG00000172046
1,78	0,00	0,00	ENSG00000172037
0,88	0,16	0,25	ENSG00000177352
1,62	0,00	0,00	ENSG00000185909
0,99	1,00	#N/D	ENSG00000236980
15,64	0,33	0,44	ENSG00000173421
0,66	0,01	0,02	ENSG00000188315
1,10	0,25	0,36	ENSG00000114316
0,82	0,01	0,03	ENSG00000233276
1,14	0,11	0,19	ENSG00000290318
1,41	0,00	0,00	ENSG00000067560
2,81	0,00	0,00	ENSG00000145022
1,18	0,07	0,13	ENSG00000283189
1,43	0,00	0,00	ENSG00000145020
1,12	0,21	0,31	ENSG00000145029
0,96	0,58	0,68	ENSG00000173402
0,57	0,00	0,00	ENSG00000164061
1,08	0,38	0,49	ENSG00000164062
0,54	0,00	0,00	ENSG00000173531
0,87	0,09	0,15	ENSG00000164068
0,82	0,31	0,43	ENSG00000176020
1,99	0,00	0,00	ENSG00000173540
1,07	0,43	0,55	ENSG00000176095
2,28	0,01	0,03	ENSG00000187492
1,00	1,00	1,00	ENSG00000185614
2,64	0,00	0,00	ENSG00000182179
0,79	0,02	0,04	ENSG00000183763

0,07	0,31	0,43	ENSG00000164076
2,83	0,00	0,00	ENSG00000164078
0,93	0,48	0,58	ENSG00000164077
0,87	0,09	0,16	ENSG00000004534
1,04	0,64	0,73	ENSG00000003756
1,93	0,00	0,00	ENSG00000001617
0,99	1,00	1,00	ENSG00000114349
1,21	0,68	0,77	ENSG00000188338
1,74	0,00	0,00	ENSG00000114353
0,68	0,00	0,00	ENSG00000012171
1,40	0,05	0,09	ENSG00000179564
0,84	0,03	0,06	ENSG00000214706
0,77	0,03	0,06	ENSG00000186792
1,50	0,00	0,00	ENSG00000243477
1,36	0,09	0,16	ENSG00000114378
1,63	0,00	0,00	ENSG00000068001
1,45	0,00	0,00	ENSG00000114383
1,74	0,00	0,00	ENSG00000068028
2,68	0,00	0,00	ENSG00000004838
3,21	0,00	0,00	ENSG00000114388
2,49	0,00	0,00	ENSG00000114395
7,31	0,00	0,00	ENSG00000272104
1,01	0,86	0,93	ENSG00000126062
13,50	0,00	0,00	ENSG00000007402
1,15	0,13	0,21	ENSG00000088543
1,82	0,00	0,00	ENSG00000114735
4,35	0,00	0,00	ENSG00000114737
0,68	0,00	0,00	ENSG00000114738
0,48	0,00	0,00	ENSG00000088538
2,48	0,00	0,00	ENSG00000145050
1,01	0,90	0,97	ENSG00000259956
1,07	0,43	0,55	ENSG00000145041
0,84	0,03	0,06	ENSG00000164080
0,88	0,14	0,23	ENSG00000164081
15,64	0,33	0,44	ENSG00000164082
#N/D	#N/D	#N/D	ENSG00000214686
#N/D	#N/D	#N/D	ENSG00000285749
#N/D	#N/D	#N/D	ENSG00000229972
#N/D	#N/D	#N/D	ENSG00000184345
1,14	0,97	#N/D	ENSG00000214681
8,39	0,46	#N/D	ENSG00000173389
0,99	0,93	0,98	ENSG00000114767
0,57	0,00	0,00	ENSG00000041880
1,50	0,17	0,26	ENSG00000280422
1,52	0,21	0,31	ENSG00000180929
1,29	0,00	0,01	ENSG00000090097
1,43	0,00	0,00	ENSG00000114779
0,68	0,00	0,01	ENSG00000248487

0,70	0,00	0,01	ENSG00000114786
0,78	0,01	0,02	ENSG00000243989
0,93	0,36	0,47	ENSG00000162244
1,23	0,01	0,03	ENSG00000164086
0,76	0,00	0,00	ENSG00000164087
1,05	0,58	0,68	ENSG00000023330
3,06	0,03	0,06	ENSG00000280003
1,71	0,01	0,02	ENSG00000239732
1,14	0,97	0,98	ENSG00000173366
1,18	0,04	0,08	ENSG00000247596
1,23	0,02	0,03	ENSG00000164088
0,96	0,63	0,73	ENSG00000164091
0,97	0,77	0,85	ENSG00000168237
0,99	0,94	0,98	ENSG00000114841
1,20	0,02	0,05	ENSG00000163930
0,67	0,00	0,01	ENSG00000010318
0,97	0,95	0,98	ENSG00000010319
0,31	0,04	0,07	ENSG00000114854
1,43	0,00	0,00	ENSG00000010322
4,62	0,00	0,00	ENSG00000010327
1,37	0,00	0,00	ENSG00000168268
2,08	0,00	0,00	ENSG00000168273
0,70	0,00	0,00	ENSG00000163939
1,17	0,06	0,10	ENSG00000163938
1,06	0,49	0,59	ENSG00000016864
0,92	0,32	0,44	ENSG00000114902
0,75	0,00	0,00	ENSG00000114904
0,13	0,46	0,56	ENSG00000055957
8,39	0,46	0,56	ENSG00000162267
0,53	0,58	0,68	ENSG00000055955
1,24	0,24	0,34	ENSG00000243696
2,02	0,05	0,09	ENSG00000272573
80,90	0,11	0,18	ENSG00000248592
1,24	0,01	0,03	ENSG00000213533
2,88	0,03	0,05	ENSG00000279144
0,59	0,00	0,00	ENSG00000163935
1,42	0,30	0,42	ENSG00000272305
0,43	0,22	0,32	ENSG00000280417
0,92	0,33	0,44	ENSG00000163933
0,93	0,43	0,55	ENSG00000163932
1,62	0,00	0,00	ENSG00000163931
1,19	0,04	0,08	ENSG00000272886
5,68	0,00	0,00	ENSG00000157388
0,72	0,00	0,00	ENSG00000016391
1,05	0,69	0,78	ENSG00000056736
1,35	0,00	0,00	ENSG00000113812
1,78	0,00	0,00	ENSG00000113811
0,53	0,00	0,01	ENSG00000157445

1,14	0,97	#N/D	ENSG00000144771
0,95	0,56	0,67	ENSG00000114251
0,60	0,47	0,58	ENSG00000187672
0,80	0,05	0,10	ENSG00000180376
0,64	0,00	0,00	ENSG00000163946
0,84	0,08	0,14	ENSG00000163947
1,31	0,32	0,43	ENSG00000186451
2,47	0,00	0,00	ENSG00000144730
0,60	0,09	0,15	ENSG00000163666
0,68	0,00	0,00	ENSG00000157500
1,13	0,71	0,80	ENSG00000239388
0,14	0,01	0,02	ENSG00000174844
1,14	0,13	0,21	ENSG00000174840
2,39	0,00	0,00	ENSG00000168374
0,96	0,62	0,72	ENSG00000174839
1,02	0,77	0,85	ENSG00000163681
1,11	0,19	0,29	ENSG00000136068
#N/D	#N/D	#N/D	ENSG00000163687
0,64	0,00	0,00	ENSG00000163686
0,77	0,00	0,01	ENSG00000163684
1,73	0,00	0,00	ENSG00000255154
0,67	0,00	0,00	ENSG00000168297
1,03	0,75	0,83	ENSG00000168291
0,58	0,00	0,00	ENSG00000168301
0,37	0,00	0,00	ENSG00000168306
7,44	0,00	0,00	ENSG00000168309
2,35	0,30	0,41	ENSG00000198643
0,77	0,04	0,07	ENSG00000163689
7,79	0,00	0,00	ENSG00000189283
0,51	0,00	0,00	ENSG00000144724
1,14	0,97	0,98	ENSG00000114405
#N/D	#N/D	#N/D	ENSG00000153266
0,92	0,81	0,88	ENSG00000163618
1,14	0,97	0,98	ENSG00000163630
0,13	0,46	0,56	ENSG00000188817
0,23	0,02	0,04	ENSG00000163632
0,60	0,00	0,00	ENSG00000163634
0,92	0,30	0,42	ENSG00000163635
0,67	0,00	0,00	ENSG00000163636
1,45	0,00	0,00	ENSG00000163637
22,69	0,00	0,00	ENSG00000163638
0,79	0,02	0,04	ENSG00000151276
1,01	0,95	0,98	ENSG00000144741
0,21	0,00	0,00	ENSG00000144749
0,41	0,00	0,00	ENSG00000163376
0,87	0,10	0,17	ENSG00000172340
0,98	0,99	0,99	ENSG00000183662
#N/D	#N/D	#N/D	ENSG00000163377

0,66	0,00	0,00	ENSG00000163378
1,61	0,00	0,00	ENSG00000144747
0,87	0,11	0,18	ENSG00000144744
1,19	0,04	0,07	ENSG00000144746
1,29	0,74	0,82	ENSG00000163380
0,29	0,00	0,00	ENSG00000114541
2,40	0,00	0,00	ENSG00000187098
#N/D	#N/D	#N/D	ENSG00000242120
0,80	0,01	0,01	ENSG00000114861
1,51	0,19	0,28	ENSG00000285708
1,10	0,28	0,39	ENSG00000163412
0,83	0,06	0,10	ENSG00000170837
37,39	0,19	0,29	ENSG00000163421
0,71	0,00	0,00	ENSG00000163602
0,96	0,70	0,78	ENSG00000144736
0,59	0,00	0,00	ENSG00000172986
0,81	0,02	0,03	ENSG00000163605
1,06	0,71	0,79	ENSG00000255423
1,23	0,02	0,04	ENSG00000121440
0,81	0,30	0,42	ENSG00000278934
1.458,58	0,01	0,02	ENSG00000113805
8,39	0,46	0,56	ENSG00000172969
1,91	0,35	0,47	ENSG00000227124
0,50	0,00	0,00	ENSG00000185008
1,01	0,94	0,98	ENSG00000169855
1,32	0,00	0,00	ENSG00000114480
0,03	0,00	0,00	ENSG00000175161
0,80	0,01	0,03	ENSG00000206538
0,78	0,01	0,01	ENSG00000083937
1,14	0,97	0,98	ENSG00000064835
#N/D	#N/D	#N/D	ENSG00000179097
0,83	0,03	0,06	ENSG00000163320
#N/D	#N/D	#N/D	ENSG00000288654
0,88	0,23	0,33	ENSG00000175105
0,89	0,27	0,38	ENSG00000179021
1,14	0,97	#N/D	ENSG00000283434
9,67	0,00	0,00	ENSG00000044524
0,83	0,12	0,20	ENSG00000184500
0,76	0,01	0,01	ENSG00000169379
0,05	0,24	0,35	ENSG00000178750
0,71	0,00	0,01	ENSG00000178700
1,03	0,76	0,84	ENSG00000178694
0,56	0,00	0,00	ENSG00000080224
1,18	0,18	0,28	ENSG00000113966
0,90	0,23	0,34	ENSG00000080200
0,93	0,37	0,48	ENSG00000170854
1,14	0,97	#N/D	ENSG00000183185
#N/D	#N/D	#N/D	ENSG00000213439

#N/D	#N/D	#N/D	ENSG00000196578
#N/D	#N/D	#N/D	ENSG00000231192
1,14	0,97	#N/D	ENSG00000236032
#N/D	#N/D	#N/D	ENSG00000233412
#N/D	#N/D	#N/D	ENSG00000230301
#N/D	#N/D	#N/D	ENSG00000197938
1,14	0,97	#N/D	ENSG00000232535
#N/D	#N/D	#N/D	ENSG00000196098
#N/D	#N/D	#N/D	ENSG00000206536
1,14	0,97	#N/D	ENSG00000232382
15,64	0,33	0,44	ENSG00000231861
1,10	0,23	0,34	ENSG00000080822
1,14	0,97	#N/D	ENSG00000285635
95,40	0,10	0,16	ENSG00000154165
1,08	0,36	0,47	ENSG00000080819
1,22	0,08	0,13	ENSG00000064225
1,28	0,00	0,01	ENSG00000057019
1,48	0,00	0,00	ENSG00000144810
0,65	0,00	0,00	ENSG00000184220
1,08	0,47	0,58	ENSG00000168386
1,84	0,00	0,00	ENSG00000036054
0,90	0,20	0,30	ENSG00000114021
1,31	0,00	0,00	ENSG00000154174
0,82	0,11	0,18	ENSG00000206535
1,53	0,00	0,00	ENSG00000181458
1,91	0,00	0,00	ENSG00000144820
1,65	0,00	0,00	ENSG00000114354
0,02	0,00	0,00	ENSG00000154175
0,88	0,70	0,78	ENSG00000081148
1,16	0,13	0,21	ENSG00000138468
0,67	0,00	0,00	ENSG00000174173
0,78	0,00	0,01	ENSG00000081154
2,35	0,00	0,00	ENSG00000066422
1,28	0,00	0,01	ENSG00000114391
1,46	0,00	0,00	ENSG00000293268
1,33	0,00	0,00	ENSG00000182504
0,51	0,00	0,00	ENSG00000144815
0,61	0,00	0,00	ENSG00000144802
1,54	0,44	0,56	ENSG00000170044
1,60	0,00	0,00	ENSG00000170017
1,70	0,00	0,00	ENSG00000114423
1,14	0,97	#N/D	ENSG00000138483
1,04	0,62	0,72	ENSG00000114439
1,35	0,00	0,00	ENSG00000196776
0,94	0,52	0,62	ENSG00000114446
15,64	0,33	0,44	ENSG00000114455
3,87	0,00	0,00	ENSG00000144821
0,83	0,03	0,06	ENSG00000163507

0,86	0,10	0,17	ENSG00000198919
1,14	0,97	#N/D	ENSG00000163515
#N/D	#N/D	#N/D	ENSG00000163519
#N/D	#N/D	#N/D	ENSG00000138472
#N/D	#N/D	#N/D	ENSG00000114487
#N/D	#N/D	#N/D	ENSG00000241224
1,14	0,97	#N/D	ENSG00000163530
0,07	0,31	#N/D	ENSG00000121570
1,17	0,08	0,13	ENSG00000177707
0,27	0,22	0,32	ENSG00000153283
3,72	0,09	0,15	ENSG00000177494
0,87	0,15	0,24	ENSG00000240891
0,18	0,00	0,00	ENSG00000144824
1,10	0,24	0,35	ENSG00000144827
0,99	1,00	1,00	ENSG00000144834
15,64	0,33	0,44	ENSG00000176040
0,68	0,00	0,00	ENSG00000114529
0,97	0,86	0,93	ENSG00000174500
0,02	0,15	0,23	ENSG00000172139
8,39	0,46	0,56	ENSG00000091972
30,14	0,22	0,32	ENSG00000186265
1,17	0,72	0,81	ENSG00000279349
1,20	0,03	0,06	ENSG00000144848
1,02	0,80	0,87	ENSG00000138459
0,96	0,58	0,68	ENSG00000091986
1,14	0,97	#N/D	ENSG00000206531
3,33	0,03	0,06	ENSG00000163606
0,69	0,00	0,00	ENSG00000163607
1,32	0,00	0,00	ENSG00000163608
1,70	0,00	0,00	ENSG00000144857
0,18	0,00	0,00	ENSG00000206530
0,81	0,05	0,09	ENSG00000285943
1,70	0,00	0,00	ENSG00000163611
3,03	0,00	0,00	ENSG00000072858
1,00	0,96	0,98	ENSG00000176542
0,98	0,80	0,87	ENSG00000121579
1,87	0,00	0,00	ENSG00000114573
1,28	0,05	0,09	ENSG00000178075
1,04	0,69	0,78	ENSG00000184307
1,30	0,01	0,03	ENSG00000163617
0,97	0,72	0,81	ENSG00000151576
2,71	0,36	0,48	ENSG00000151577
30,14	0,22	0,32	ENSG00000174255
0,74	0,69	0,78	ENSG00000181847
1,34	0,00	0,00	ENSG00000181722
0,17	0,00	0,00	ENSG00000172020
1,15	0,44	0,56	ENSG00000185565
30,14	0,22	0,32	ENSG00000144847

8,39	0,46	#N/D	ENSG00000163424
#N/D	#N/D	#N/D	ENSG00000251012
0,67	0,65	0,74	ENSG00000114638
1,39	0,00	0,00	ENSG00000121578
1,22	0,01	0,03	ENSG00000031081
2,13	0,00	0,00	ENSG00000176142
0,48	0,00	0,00	ENSG00000163389
0,96	0,62	0,72	ENSG00000113845
1,68	0,40	0,52	ENSG00000121594
0,01	0,09	0,15	ENSG00000144843
0,03	0,17	0,26	ENSG00000144837
0,09	0,00	0,00	ENSG00000121577
1,25	0,03	0,06	ENSG00000138495
3,79	0,00	0,00	ENSG00000183833
1,14	0,97	#N/D	ENSG00000285585
8,39	0,46	0,56	ENSG00000144852
0,84	0,03	0,05	ENSG00000082701
0,57	0,00	0,00	ENSG00000175697
1,02	0,86	0,93	ENSG00000163428
0,85	0,04	0,07	ENSG00000163430
1,34	0,00	0,00	ENSG00000065518
1,36	0,60	0,70	ENSG00000113924
1,23	0,02	0,03	ENSG00000144840
0,92	0,33	0,44	ENSG00000153767
0,25	0,00	0,00	ENSG00000145087
0,97	0,70	0,78	ENSG00000051341
0,67	0,65	0,74	ENSG00000186103
1,85	0,60	#N/D	ENSG00000163833
0,21	0,00	0,00	ENSG00000180353
1,25	0,01	0,01	ENSG00000173230
0,88	0,17	0,26	ENSG00000173226
8,90	0,00	0,00	ENSG00000145088
6,21	0,00	0,00	ENSG00000163406
#N/D	#N/D	#N/D	ENSG00000145103
#N/D	#N/D	#N/D	ENSG00000114013
1,14	0,97	#N/D	ENSG00000036828
0,01	0,05	0,10	ENSG00000121552
0,88	0,20	0,30	ENSG00000160124
1,81	0,00	0,00	ENSG00000114023
1,44	0,00	0,00	ENSG00000196981
1,22	0,01	0,03	ENSG00000114030
1,50	0,00	0,00	ENSG00000138496
1,18	0,04	0,08	ENSG00000163840
0,79	0,72	0,80	ENSG00000173200
1,15	0,09	0,16	ENSG00000173193
0,76	0,03	0,06	ENSG00000169087
0,97	0,77	0,85	ENSG00000138463
0,13	0,46	#N/D	ENSG00000082684

1,17	0,06	0,11	ENSG00000065485
1,98	0,00	0,00	ENSG00000121542
4,03	0,00	0,00	ENSG00000173175
0,85	0,05	0,10	ENSG00000206527
0,93	0,37	0,48	ENSG00000065534
0,76	0,00	0,00	ENSG00000175455
1,85	0,60	0,69	ENSG00000065371
3,77	0,00	0,00	ENSG00000160145
1,04	0,62	0,72	ENSG00000114491
1,57	0,00	0,00	ENSG00000082781
1,11	0,56	0,67	ENSG00000280042
0,26	0,00	0,01	ENSG00000173702
1,36	0,00	0,00	ENSG00000173706
0,99	0,94	0,98	ENSG00000221955
1,17	0,06	0,11	ENSG00000163848
0,47	0,00	0,00	ENSG00000114520
1,10	0,27	0,38	ENSG00000144909
3,56	0,23	0,34	ENSG00000114547
1,36	0,00	0,00	ENSG00000114544
6,56	0,00	0,00	ENSG00000144908
0,31	0,00	0,00	ENSG00000163884
0,60	0,47	0,58	ENSG00000163885
0,97	0,89	0,96	ENSG00000279328
1,69	0,00	0,00	ENSG00000070476
3,56	0,23	0,34	ENSG00000159650
#N/D	#N/D	#N/D	ENSG00000180767
0,13	0,46	#N/D	ENSG00000180697
1,12	0,22	0,32	ENSG00000197763
1,32	0,00	0,00	ENSG00000159685
1,43	0,27	0,38	ENSG00000280053
0,88	0,11	0,18	ENSG00000114554
#N/D	#N/D	#N/D	ENSG00000214324
30,14	0,22	0,32	ENSG00000239620
1,53	0,00	0,00	ENSG00000163870
1,15	0,08	0,14	ENSG00000073111
0,89	0,15	0,23	ENSG00000114631
1,39	0,00	0,00	ENSG00000114626
1,59	0,00	0,00	ENSG00000074416
2,35	0,30	0,41	ENSG00000187715
2,36	0,00	0,00	ENSG00000058262
1,02	0,77	0,85	ENSG00000175792
0,82	0,03	0,07	ENSG00000132394
1,36	0,60	0,70	ENSG00000179407
0,83	0,04	0,07	ENSG00000179348
1,55	0,00	0,00	ENSG00000163902
1,05	0,56	0,66	ENSG00000075785
1,26	0,00	0,01	ENSG00000177646
1,87	0,00	0,00	ENSG00000114656

#N/D	#N/D	#N/D	ENSG00000279507
0,98	0,97	0,98	ENSG00000114654
1,14	0,97	#N/D	ENSG00000169704
3,15	0,00	0,00	ENSG00000172780
1,97	0,00	0,00	ENSG00000261796
1,36	0,00	0,00	ENSG00000240682
1,24	0,01	0,02	ENSG00000169714
2,07	0,00	0,00	ENSG00000181789
1,16	0,08	0,13	ENSG00000183624
0,61	0,00	0,00	ENSG00000184897
1,23	0,56	0,66	ENSG00000172771
1,48	0,00	0,00	ENSG00000163913
1,63	0,00	0,00	ENSG00000129071
1,14	0,97	#N/D	ENSG00000163914
#N/D	#N/D	#N/D	ENSG00000178804
1,15	0,08	0,14	ENSG00000004399
1,01	0,92	0,98	ENSG00000172765
2,71	0,36	0,48	ENSG00000170893
1,21	0,77	0,85	ENSG00000251287
#N/D	#N/D	#N/D	ENSG00000172752
1,16	0,56	0,66	ENSG00000206384
1,29	0,00	0,01	ENSG00000196455
0,87	0,08	0,13	ENSG00000017260
1,11	0,78	0,86	ENSG00000284095
1,25	0,03	0,06	ENSG00000034533
0,95	0,69	0,78	ENSG00000114670
1,75	0,00	0,00	ENSG00000198585
0,79	0,00	0,01	ENSG00000114686
1,66	0,29	0,40	ENSG00000196353
4,87	0,00	0,00	ENSG00000014257
1,47	0,00	0,00	ENSG00000138246
1,14	0,12	0,19	ENSG00000240303
1,01	0,95	0,98	ENSG00000274810
2,10	0,00	0,00	ENSG00000129048
1,22	0,03	0,06	ENSG00000081307
1,58	0,00	0,00	ENSG00000113971
3,39	0,00	0,00	ENSG00000144868
15,64	0,33	#N/D	ENSG00000170819
1,21	0,02	0,04	ENSG00000091527
1,35	0,00	0,00	ENSG00000163781
1,69	0,01	0,02	ENSG00000091513
2,02	0,00	0,00	ENSG00000144867
2,09	0,00	0,00	ENSG00000154917
0,66	0,10	0,16	ENSG00000174640
1,19	0,03	0,07	ENSG00000163785
1,29	0,00	0,00	ENSG00000114019
1,45	0,00	0,00	ENSG00000129055
0,93	0,46	0,56	ENSG00000182923

0,78	0,61	0,70	ENSG00000174611
1,83	0,04	0,08	ENSG00000154928
1,80	0,00	0,00	ENSG00000073711
1,06	0,47	0,58	ENSG00000174579
0,94	0,42	0,54	ENSG00000114054
1,28	0,00	0,01	ENSG00000118007
0,77	0,01	0,01	ENSG00000168917
1,45	0,00	0,00	ENSG00000158092
0,88	0,16	0,25	ENSG00000174564
#N/D	#N/D	#N/D	ENSG00000168875
1,10	0,59	0,69	ENSG00000066405
1,08	0,39	0,51	ENSG00000158163
1,61	0,36	0,47	ENSG00000118017
0,71	0,00	0,00	ENSG00000138231
0,72	0,00	0,00	ENSG00000114098
0,89	0,65	0,74	ENSG00000181322
1,07	0,42	0,54	ENSG00000158186
0,72	0,01	0,02	ENSG00000158220
0,54	0,02	0,03	ENSG00000280399
0,80	0,01	0,03	ENSG00000114107
0,36	0,00	0,00	ENSG00000158234
1,14	0,12	0,19	ENSG00000051382
0,36	0,35	0,46	ENSG00000183770
0,19	0,00	0,01	ENSG00000206262
0,07	0,31	0,43	ENSG00000206260
1,09	0,27	0,38	ENSG00000175110
1,14	0,97	#N/D	ENSG00000184814
1,14	0,97	#N/D	ENSG00000233701
1,46	0,00	0,00	ENSG00000184432
3,56	0,23	0,34	ENSG00000114113
1,61	0,36	0,47	ENSG00000114115
5,01	0,00	0,00	ENSG00000163864
10,55	0,00	0,00	ENSG00000158258
#N/D	#N/D	#N/D	ENSG00000155890
0,70	0,00	0,00	ENSG00000114120
2,68	0,00	0,01	ENSG00000175093
0,64	0,00	0,00	ENSG00000155893
0,61	0,00	0,00	ENSG00000177311
0,69	0,00	0,00	ENSG00000155903
1,00	0,99	0,99	ENSG00000114125
1,14	0,97	#N/D	ENSG00000285558
1,27	0,41	0,53	ENSG00000114124
0,87	0,08	0,14	ENSG00000069849
0,98	0,97	0,99	ENSG00000279147
0,51	0,00	0,00	ENSG00000114126
0,63	0,00	0,00	ENSG00000175066
0,76	0,00	0,00	ENSG00000114127
0,68	0,00	0,00	ENSG00000175054

0,41	0,00	0,00	ENSG00000120756
0,65	0,00	0,00	ENSG00000144935
1,01	0,94	0,98	ENSG00000163710
1,35	0,03	0,07	ENSG00000188582
0,49	0,00	0,00	ENSG00000163714
0,12	0,00	0,00	ENSG00000175040
1,01	0,94	0,98	ENSG00000181804
0,87	0,14	0,22	ENSG00000181744
22,89	0,26	0,37	ENSG00000280397
1,23	0,01	0,02	ENSG00000152952
2,99	0,00	0,00	ENSG00000114698
2,80	0,20	0,30	ENSG00000163746
2,20	0,08	0,14	ENSG00000279320
1,05	0,60	0,70	ENSG00000188313
1,14	0,97	#N/D	ENSG00000231213
1,06	0,58	0,68	ENSG00000174963
0,83	0,03	0,05	ENSG00000152977
0,43	0,00	0,00	ENSG00000144891
#N/D	#N/D	#N/D	ENSG00000153002
0,04	0,20	0,30	ENSG00000163751
0,77	0,00	0,00	ENSG00000163754
0,78	0,00	0,00	ENSG00000071794
1,25	0,01	0,02	ENSG00000163755
2,56	0,00	0,00	ENSG00000047457
122,71	0,00	0,00	ENSG00000163762
1,43	0,00	0,00	ENSG00000169908
0,22	0,14	0,22	ENSG00000169903
1,56	0,00	0,00	ENSG00000018408
0,69	0,00	0,00	ENSG00000114744
0,07	0,31	#N/D	ENSG00000206199
0,80	0,01	0,02	ENSG00000082996
0,77	0,00	0,00	ENSG00000070087
1,60	0,00	0,00	ENSG00000196428
1,26	0,00	0,01	ENSG00000120742
1,07	0,39	0,50	ENSG00000144895
0,75	0,00	0,00	ENSG00000198843
3,72	0,09	0,15	ENSG00000163645
1,58	0,00	0,00	ENSG00000181788
0,67	0,65	0,74	ENSG00000214237
1,14	0,97	#N/D	ENSG00000260234
0,13	0,46	#N/D	ENSG00000163646
7,64	0,00	0,00	ENSG00000144893
30,14	0,22	0,32	ENSG00000174946
8,39	0,46	0,56	ENSG00000174944
196,91	0,05	0,10	ENSG00000138271
22,89	0,26	0,37	ENSG00000181631
8,39	0,46	0,56	ENSG00000169313
5,08	0,00	0,00	ENSG00000152580

0,53	0,58	0,68	ENSG00000197953
73,65	0,12	0,19	ENSG00000114771
18,29	0,00	0,00	ENSG00000198829
1,72	0,00	0,00	ENSG00000152601
0,18	0,00	0,00	ENSG00000169860
1,04	0,67	0,76	ENSG00000181467
0,39	0,00	0,00	ENSG00000114790
0,67	0,00	0,00	ENSG00000174953
#N/D	#N/D	#N/D	ENSG00000279305
1,14	0,97	#N/D	ENSG00000174948
0,47	0,00	0,00	ENSG00000196549
8,39	0,46	#N/D	ENSG00000240045
0,22	0,00	0,00	ENSG00000114805
0,58	0,00	0,00	ENSG00000174928
3,16	0,00	0,00	ENSG00000284952
1,45	0,00	0,00	ENSG00000169359
0,84	0,03	0,06	ENSG00000163655
0,70	0,00	0,00	ENSG00000169282
0,85	0,04	0,08	ENSG00000114850
1,58	0,00	0,00	ENSG00000163659
0,46	0,00	0,00	ENSG00000197980
1,16	0,07	0,12	ENSG00000163660
0,14	0,00	0,00	ENSG00000197415
0,28	0,00	0,00	ENSG00000163661
0,54	0,00	0,00	ENSG00000168779
0,61	0,00	0,00	ENSG00000174891
0,74	0,00	0,00	ENSG00000178053
0,68	0,00	0,00	ENSG00000168827
2,35	0,30	0,41	ENSG00000079257
1,09	0,80	0,87	ENSG00000118849
1,13	0,17	0,26	ENSG00000118855
4,44	0,00	0,00	ENSG00000279311
1,15	0,21	0,31	ENSG00000283154
1,29	0,63	0,73	ENSG00000214216
1,18	0,08	0,14	ENSG00000151967
0,63	0,00	0,00	ENSG00000168811
0,90	0,62	0,72	ENSG00000180044
0,02	0,10	0,17	ENSG00000248710
0,62	0,00	0,00	ENSG00000068885
0,85	0,04	0,08	ENSG00000113810
1,56	0,00	0,00	ENSG00000213186
0,69	0,00	0,00	ENSG00000186432
0,07	0,31	0,43	ENSG00000179674
0,18	0,00	0,00	ENSG00000163590
0,76	0,01	0,01	ENSG00000169255
0,78	0,00	0,01	ENSG00000169251
0,26	0,08	0,14	ENSG00000196542
#N/D	#N/D	#N/D	ENSG00000182447

#N/D	#N/D	#N/D	ENSG00000090402
0,09	0,00	0,00	ENSG00000121871
0,01	0,00	0,00	ENSG00000114200
0,99	1,00	1,00	ENSG00000169064
0,07	0,31	#N/D	ENSG00000114204
1,09	0,80	0,87	ENSG00000174776
0,77	0,01	0,01	ENSG00000114209
5,86	0,00	0,00	ENSG00000163536
1,97	0,00	0,00	ENSG00000173905
0,21	0,00	0,00	ENSG00000085276
0,86	0,41	0,53	ENSG00000184378
1,23	0,02	0,04	ENSG00000085274
0,67	0,00	0,01	ENSG00000171757
0,25	0,01	0,03	ENSG00000188306
15,64	0,33	#N/D	ENSG00000114248
#N/D	#N/D	#N/D	ENSG00000187033
0,93	0,38	0,49	ENSG00000008952
0,29	0,00	0,00	ENSG00000173890
1,11	0,21	0,31	ENSG00000173889
0,73	0,00	0,00	ENSG00000163558
0,76	0,00	0,00	ENSG00000136603
0,59	0,00	0,00	ENSG00000013297
0,90	0,79	0,87	ENSG00000285218
1,14	0,97	0,98	ENSG00000013293
0,50	0,00	0,00	ENSG00000163584
0,71	0,00	0,00	ENSG00000163577
0,13	0,46	0,56	ENSG00000163581
0,25	0,00	0,00	ENSG00000154310
0,07	0,00	0,00	ENSG00000279673
0,50	0,00	0,00	ENSG00000075651
0,07	0,31	0,43	ENSG00000186329
0,92	0,30	0,41	ENSG00000075420
0,87	0,48	0,58	ENSG00000279862
1,14	0,97	0,98	ENSG00000121853
0,72	0,19	0,29	ENSG00000121858
1,41	0,00	0,00	ENSG00000144959
0,68	0,00	0,00	ENSG00000114346
1,14	0,97	#N/D	ENSG00000144962
0,17	0,00	0,00	ENSG00000169760
1,65	0,00	0,00	ENSG00000177694
0,92	0,31	0,42	ENSG00000177565
95,40	0,10	0,16	ENSG00000197584
0,04	0,20	0,30	ENSG00000275163
1,16	0,07	0,13	ENSG00000172667
0,74	0,00	0,00	ENSG00000121879
0,94	0,71	0,80	ENSG00000171121
0,83	0,03	0,06	ENSG00000121864
1,09	0,30	0,42	ENSG00000171109

1,12	0,15	0,24	ENSG00000114450
0,81	0,01	0,03	ENSG00000136518
0,76	0,00	0,00	ENSG00000136522
0,81	0,01	0,02	ENSG00000136521
0,61	0,00	0,00	ENSG00000288698
0,63	0,00	0,00	ENSG00000058056
0,09	0,00	0,00	ENSG00000114757
1,23	0,02	0,03	ENSG00000163728
0,98	0,97	0,98	ENSG00000284862
0,92	0,30	0,41	ENSG00000114416
0,93	0,47	0,57	ENSG00000205981
15,64	0,33	0,44	ENSG00000181449
0,81	0,01	0,02	ENSG00000058063
0,64	0,00	0,00	ENSG00000043093
0,72	0,00	0,00	ENSG00000078070
0,07	0,00	0,00	ENSG00000078081
0,18	0,00	0,00	ENSG00000053524
0,74	0,00	0,00	ENSG00000176597
1,14	0,97	0,98	ENSG00000172578
0,94	0,49	0,59	ENSG00000114796
0,75	0,00	0,00	ENSG00000163872
0,25	0,00	0,00	ENSG00000283765
1,52	0,09	0,15	ENSG00000180834
0,86	0,07	0,12	ENSG00000175193
0,66	0,00	0,00	ENSG00000114770
#N/D	#N/D	#N/D	ENSG00000186090
#N/D	#N/D	#N/D	ENSG00000178084
0,98	0,99	0,99	ENSG00000186038
0,82	0,01	0,03	ENSG00000145191
0,89	0,14	0,22	ENSG00000161202
0,90	0,20	0,30	ENSG00000161203
0,96	0,62	0,72	ENSG00000161204
1,76	0,00	0,00	ENSG00000145198
0,75	0,00	0,00	ENSG00000214160
1,40	0,00	0,00	ENSG00000284753
2,80	0,20	0,30	ENSG00000284917
0,64	0,00	0,00	ENSG00000163888
5,28	0,10	0,17	ENSG00000145194
0,64	0,00	0,00	ENSG00000175166
0,82	0,01	0,03	ENSG00000114867
0,93	0,42	0,54	ENSG00000175182
0,60	0,00	0,00	ENSG00000114859
0,84	0,04	0,08	ENSG00000163882
0,12	0,03	0,07	ENSG00000090534
0,13	0,00	0,00	ENSG00000090539
2,67	0,00	0,00	ENSG00000182580
0,63	0,00	0,00	ENSG00000177383
0,69	0,00	0,00	ENSG00000156931

1,14	0,97	0,98	ENSG00000187068
0,82	0,05	0,09	ENSG00000113790
1,29	0,00	0,01	ENSG00000073803
0,64	0,00	0,00	ENSG00000163900
0,08	0,00	0,00	ENSG00000163898
1,08	0,38	0,50	ENSG00000163904
0,70	0,00	0,00	ENSG00000073792
0,90	0,19	0,29	ENSG00000136527
0,91	0,25	0,35	ENSG00000244405
0,61	0,00	0,00	ENSG00000058866
0,56	0,04	0,08	ENSG00000213139
0,91	0,33	0,44	ENSG00000113838
1,42	0,00	0,00	ENSG00000090520
#N/D	#N/D	#N/D	ENSG00000283149
1,14	0,97	#N/D	ENSG00000145192
#N/D	#N/D	#N/D	ENSG00000090512
#N/D	#N/D	#N/D	ENSG00000113905
#N/D	#N/D	#N/D	ENSG00000113889
1,09	0,28	0,38	ENSG00000156976
0,72	0,00	0,00	ENSG00000163918
0,13	0,46	#N/D	ENSG00000181092
0,47	0,00	0,00	ENSG00000073849
0,78	0,01	0,03	ENSG00000163923
0,13	0,46	0,56	ENSG00000175077
0,82	0,40	0,52	ENSG00000127241
1,54	0,44	0,56	ENSG00000136514
0,07	0,31	0,43	ENSG00000280270
#N/D	#N/D	#N/D	ENSG00000157005
22,89	0,26	0,37	ENSG00000198471
0,74	0,00	0,00	ENSG00000113916
0,81	0,01	0,02	ENSG00000145012
1,03	0,87	0,93	ENSG00000279891
0,19	0,00	0,00	ENSG00000188001
0,04	0,00	0,00	ENSG00000073282
1,73	0,00	0,00	ENSG00000090530
0,20	0,00	0,00	ENSG00000163347
0,80	0,50	0,60	ENSG00000113946
#N/D	#N/D	#N/D	ENSG00000198398
0,87	0,12	0,20	ENSG00000196083
4,42	0,15	0,24	ENSG00000205835
1,44	0,68	0,77	ENSG00000188729
0,60	0,09	0,15	ENSG00000188958
0,89	0,17	0,26	ENSG00000152492
1,14	0,97	#N/D	ENSG00000253548
0,29	0,00	0,00	ENSG00000114279
0,43	0,00	0,00	ENSG00000180611
0,12	0,00	0,00	ENSG00000127252
0,07	0,31	0,43	ENSG00000187527

0,44	0,17	0,26	ENSG00000127249
0,53	0,00	0,00	ENSG00000198836
0,56	0,00	0,00	ENSG00000114315
0,98	0,99	0,99	ENSG00000178772
0,73	0,49	0,59	ENSG00000172061
0,99	1,00	1,00	ENSG00000178732
1,00	0,97	0,99	ENSG00000133657
1,11	0,24	0,35	ENSG00000145014
0,86	0,07	0,13	ENSG00000041802
0,46	0,00	0,00	ENSG00000185112
0,76	0,00	0,00	ENSG00000173950
1,13	0,14	0,22	ENSG00000114331
0,87	0,11	0,18	ENSG00000184203
2,80	0,20	0,30	ENSG00000189058
0,70	0,21	0,31	ENSG00000176945
1,29	0,74	0,82	ENSG00000145113
1,16	0,07	0,12	ENSG00000061938
0,02	0,13	0,21	ENSG00000289747
0,39	0,00	0,00	ENSG00000072274
0,99	1,00	1,00	ENSG00000163958
1,94	0,01	0,01	ENSG00000163959
1,02	0,84	0,91	ENSG00000161217
3,46	0,00	0,00	ENSG00000272741
0,87	0,28	0,39	ENSG00000213123
196,91	0,05	0,10	ENSG00000273331
0,44	0,00	0,00	ENSG00000145107
1,02	0,77	0,85	ENSG00000163960
1,52	0,00	0,00	ENSG00000163961
0,26	0,08	0,14	ENSG00000214097
0,99	0,89	0,95	ENSG00000185798
0,83	0,02	0,05	ENSG00000174013
1,36	0,60	0,70	ENSG00000174004
0,80	0,01	0,02	ENSG00000163964
0,83	0,17	0,26	ENSG00000174007
0,84	0,03	0,05	ENSG00000180370
1,20	0,02	0,05	ENSG00000119231
1,05	0,56	0,66	ENSG00000114503
0,95	0,56	0,67	ENSG00000270170
1,07	0,53	0,64	ENSG00000119227
0,96	0,65	0,74	ENSG00000163975
0,62	0,00	0,00	ENSG00000075711
0,16	0,00	0,00	ENSG00000161267
0,78	0,00	0,01	ENSG00000145016
0,81	0,01	0,02	ENSG00000122068
0,46	0,00	0,00	ENSG00000186001
0,91	0,46	0,56	ENSG00000114473
1,08	0,33	0,45	ENSG00000182899
0,63	0,00	0,00	ENSG00000185621

0,30	0,00	0,00	ENSG00000272602
1,12	0,30	0,41	ENSG00000250312
0,98	0,99	0,99	ENSG00000186777
1,73	0,00	0,00	ENSG00000131127
0,59	0,00	0,00	ENSG00000182903
1,05	0,56	0,66	ENSG00000174227
66,40	0,13	0,21	ENSG00000273238
1,11	0,84	0,91	ENSG00000133256
1,04	0,65	0,74	ENSG00000169020
0,53	0,00	0,00	ENSG00000215375
0,42	0,01	0,03	ENSG00000169026
1,31	0,00	0,00	ENSG00000185619
3,70	0,00	0,01	ENSG00000168993
1,38	0,00	0,00	ENSG00000178950
1,31	0,01	0,01	ENSG00000127419
1,42	0,00	0,00	ENSG00000145214
0,58	0,00	0,00	ENSG00000145217
1,32	0,01	0,02	ENSG00000127415
2,09	0,00	0,00	ENSG00000127418
22,89	0,26	0,37	ENSG00000178222
1,67	0,00	0,00	ENSG00000159674
1,33	0,00	0,00	ENSG00000159692
0,87	0,11	0,19	ENSG00000090316
1,29	0,00	0,00	ENSG00000163945
8,39	0,46	#N/D	ENSG00000235608
0,56	0,00	0,00	ENSG00000174137
1,25	0,01	0,02	ENSG00000163950
1,14	0,09	0,16	ENSG00000013810
1,46	0,00	0,00	ENSG00000168936
1,58	0,01	0,01	ENSG00000068078
0,76	0,00	0,00	ENSG00000168924
0,88	0,12	0,20	ENSG00000109685
0,99	0,88	0,95	ENSG00000185049
0,63	0,00	0,00	ENSG00000243449
0,40	0,00	0,00	ENSG00000185818
0,17	0,00	0,00	ENSG00000130997
1,37	0,18	0,28	ENSG00000290263
1,03	0,82	0,89	ENSG00000214367
1,56	0,00	0,00	ENSG00000123933
1,65	0,00	0,00	ENSG00000159733
0,13	0,46	0,56	ENSG00000206113
1,00	0,96	0,98	ENSG00000063978
1,14	0,97	0,98	ENSG00000290180
1,03	0,71	0,80	ENSG00000125386
0,84	0,05	0,10	ENSG00000168884
1,20	0,03	0,05	ENSG00000087266
0,90	0,20	0,30	ENSG00000087274
1,20	0,03	0,05	ENSG00000109736

0,75	0,00	0,00	ENSG00000087269
1,12	0,44	0,56	ENSG00000125388
1,33	0,00	0,00	ENSG00000197386
0,61	0,11	0,18	ENSG00000188981
1,28	0,00	0,01	ENSG00000159788
3,49	0,00	0,00	ENSG00000109758
1,90	0,01	0,03	ENSG00000175920
1,14	0,11	0,19	ENSG00000163956
2,24	0,00	0,00	ENSG00000184160
2,71	0,36	0,48	ENSG00000163982
0,76	0,01	0,01	ENSG00000132406
0,50	0,00	0,00	ENSG00000145220
1,54	0,00	0,00	ENSG00000168826
2,35	0,01	0,02	ENSG00000168824
0,77	0,01	0,03	ENSG00000168818
1,26	0,49	0,59	ENSG00000280310
1,05	0,64	0,73	ENSG00000163132
#N/D	#N/D	#N/D	ENSG00000170891
8,02	0,00	0,00	ENSG00000152953
1,14	0,97	0,98	ENSG00000173040
0,47	0,00	0,00	ENSG00000072840
0,42	0,00	0,00	ENSG00000072832
1,14	0,97	0,98	ENSG00000181215
0,99	1,00	1,00	ENSG00000152969
#N/D	#N/D	#N/D	ENSG00000284684
1,35	0,00	0,00	ENSG00000109501
0,47	0,00	0,00	ENSG00000074211
1,09	0,32	0,43	ENSG00000013288
0,89	0,16	0,25	ENSG00000179010
1,15	0,15	0,24	ENSG00000170846
0,99	1,00	1,00	ENSG00000279859
0,66	0,42	0,54	ENSG00000163993
1,14	0,97	0,98	ENSG00000282742
0,58	0,00	0,00	ENSG00000178988
0,64	0,00	0,00	ENSG00000186222
0,76	0,00	0,00	ENSG00000170871
0,75	0,00	0,00	ENSG00000132405
0,97	0,95	0,98	ENSG00000173013
0,92	0,37	0,49	ENSG00000173011
0,93	0,37	0,48	ENSG00000109519
0,69	0,02	0,05	ENSG00000184985
0,02	0,12	0,19	ENSG00000178597
0,72	0,00	0,00	ENSG00000196526
0,81	0,31	0,43	ENSG00000163995
0,99	0,93	0,98	ENSG00000125089
0,13	0,46	0,56	ENSG00000170801
0,88	0,20	0,30	ENSG00000087008
0,91	0,31	0,42	ENSG00000155275

0,13	0,00	0,00	ENSG00000155269
0,81	0,39	0,51	ENSG00000109625
1,68	0,00	0,00	ENSG00000215612
0,13	0,46	#N/D	ENSG00000229924
1,14	0,97	#N/D	ENSG00000231396
0,02	0,13	0,21	ENSG00000233136
#N/D	#N/D	#N/D	ENSG00000227551
1,14	0,97	#N/D	ENSG00000232399
#N/D	#N/D	#N/D	ENSG00000223569
#N/D	#N/D	#N/D	ENSG00000249104
1,14	0,97	0,98	ENSG00000250844
#N/D	#N/D	#N/D	ENSG00000248920
#N/D	#N/D	#N/D	ENSG00000250745
#N/D	#N/D	#N/D	ENSG00000249811
#N/D	#N/D	#N/D	ENSG00000248933
#N/D	#N/D	#N/D	ENSG00000250913
#N/D	#N/D	#N/D	ENSG00000232264
#N/D	#N/D	#N/D	ENSG00000230430
#N/D	#N/D	#N/D	ENSG00000229579
#N/D	#N/D	#N/D	ENSG00000227140
#N/D	#N/D	#N/D	ENSG00000235780
#N/D	#N/D	#N/D	ENSG00000231051
#N/D	#N/D	#N/D	ENSG00000231637
#N/D	#N/D	#N/D	ENSG00000228856
#N/D	#N/D	#N/D	ENSG00000186146
0,07	0,31	0,43	ENSG00000109667
#N/D	#N/D	#N/D	ENSG00000169676
0,94	0,44	0,56	ENSG00000071127
0,70	0,00	0,00	ENSG00000178163
15,64	0,33	0,44	ENSG00000109684
3,36	0,00	0,00	ENSG00000002587
0,58	0,00	0,00	ENSG00000157869
1,06	0,73	0,82	ENSG00000109705
0,98	0,83	0,90	ENSG00000038219
1,38	0,00	0,00	ENSG00000137449
0,83	0,64	0,74	ENSG00000163145
3,76	0,00	0,00	ENSG00000048342
0,89	0,19	0,29	ENSG00000118564
0,91	0,38	0,50	ENSG00000237765
0,45	0,01	0,01	ENSG00000109743
0,36	0,35	0,46	ENSG00000004468
3,78	0,00	0,00	ENSG00000137440
#N/D	#N/D	#N/D	ENSG00000137441
0,13	0,46	#N/D	ENSG00000007062
0,55	0,00	0,00	ENSG00000169762
1,92	0,00	0,00	ENSG00000169744
0,82	0,03	0,05	ENSG00000151552
#N/D	#N/D	#N/D	ENSG00000249581

1,00	0,96	0,98	ENSG00000002549
1,65	0,00	0,00	ENSG00000118579
1,57	0,21	0,32	ENSG00000047662
1,33	0,00	0,00	ENSG00000163257
0,74	0,00	0,00	ENSG00000109805
0,89	0,21	0,31	ENSG00000178177
#N/D	#N/D	#N/D	ENSG00000279918
#N/D	#N/D	#N/D	ENSG00000279379
1,20	0,03	0,05	ENSG00000145147
0,67	0,00	0,00	ENSG00000163138
1,14	0,97	0,98	ENSG00000185774
1,11	0,19	0,29	ENSG00000152990
1,14	0,97	#N/D	ENSG00000249948
2,32	0,00	0,00	ENSG00000109819
1,13	0,14	0,23	ENSG00000109606
0,78	0,53	0,63	ENSG00000109610
1,22	0,02	0,04	ENSG00000181982
0,08	0,00	0,00	ENSG00000153012
0,77	0,01	0,03	ENSG00000109618
0,97	0,74	0,82	ENSG00000038210
0,69	0,00	0,00	ENSG00000168228
0,67	0,00	0,00	ENSG00000053900
8,39	0,46	0,56	ENSG00000157765
0,32	0,00	0,00	ENSG00000091490
0,81	0,04	0,08	ENSG00000250317
1,65	0,00	0,00	ENSG00000168214
15,64	0,33	0,44	ENSG00000163394
0,81	0,11	0,19	ENSG00000109680
0,70	0,00	0,00	ENSG00000109689
1,38	0,05	0,08	ENSG00000169851
0,31	0,00	0,00	ENSG00000047365
1,14	0,97	0,98	ENSG00000197057
2,62	0,10	0,16	ENSG00000174145
0,81	0,45	0,56	ENSG00000154274
1,32	0,00	0,00	ENSG00000181826
0,82	0,02	0,04	ENSG00000169299
0,65	0,26	0,37	ENSG00000279386
0,97	0,67	0,76	ENSG00000065882
1,44	0,68	0,77	ENSG00000250254
#N/D	#N/D	#N/D	ENSG00000280015
1,25	0,01	0,02	ENSG00000109787
8,39	0,46	0,56	ENSG00000174123
0,12	0,00	0,00	ENSG00000174125
0,77	0,08	0,14	ENSG00000174130
1,70	0,00	0,00	ENSG00000197712
0,33	0,00	0,00	ENSG00000121895
0,69	0,00	0,00	ENSG00000109790
0,48	0,00	0,00	ENSG00000157796

0,89	0,14	0,23	ENSG00000035928
0,56	0,01	0,02	ENSG00000134962
0,75	0,00	0,00	ENSG00000163682
0,67	0,00	0,00	ENSG00000121897
2,14	0,00	0,00	ENSG00000109814
2,18	0,00	0,00	ENSG00000163683
0,76	0,00	0,00	ENSG00000078140
0,69	0,00	0,00	ENSG00000121892
0,61	0,00	0,00	ENSG00000078177
0,98	0,97	0,98	ENSG00000168421
0,02	0,11	0,18	ENSG00000174343
0,78	0,13	0,21	ENSG00000163694
1,89	0,45	0,56	ENSG00000179299
0,38	0,00	0,00	ENSG00000163697
#N/D	#N/D	#N/D	ENSG00000289761
0,34	0,00	0,00	ENSG00000154277
1,88	0,00	0,00	ENSG00000064042
#N/D	#N/D	#N/D	ENSG00000109132
0,69	0,00	0,00	ENSG00000109133
0,73	0,26	0,37	ENSG00000182308
0,84	0,05	0,09	ENSG00000014824
0,39	0,00	0,00	ENSG00000188848
0,91	0,50	0,60	ENSG00000178343
2,41	0,00	0,00	ENSG00000124406
1,03	0,93	0,98	ENSG00000215203
5,28	0,10	0,17	ENSG00000183783
1,14	0,97	0,98	ENSG00000177752
0,28	0,00	0,00	ENSG00000151806
0,51	0,00	0,00	ENSG00000163281
0,01	0,09	0,15	ENSG00000163285
1,56	0,00	0,00	ENSG00000151834
1,14	0,97	0,98	ENSG00000170516
1,14	0,97	0,98	ENSG00000109158
28,14	0,00	0,00	ENSG00000163288
0,94	0,58	0,68	ENSG00000169019
1,28	0,01	0,01	ENSG00000145246
0,58	0,12	0,20	ENSG00000145244
1,08	0,38	0,49	ENSG00000170448
0,27	0,00	0,00	ENSG00000163293
1,21	0,77	0,85	ENSG00000198515
0,11	0,03	0,05	ENSG00000074966
0,39	0,00	0,00	ENSG00000135605
1,10	0,25	0,36	ENSG00000109171
0,90	0,79	0,87	ENSG00000145248
0,02	0,13	0,21	ENSG00000182223
0,88	0,13	0,21	ENSG00000075539
0,64	0,00	0,00	ENSG00000109180
0,13	0,46	0,56	ENSG00000145247

1,14	0,97	#N/D	ENSG00000109182
1,14	0,97	#N/D	ENSG00000280043
4,76	0,00	0,00	ENSG00000109184
1,45	0,37	0,48	ENSG00000188993
2,89	0,00	0,00	ENSG00000163069
2,35	0,30	0,41	ENSG00000163071
3,12	0,00	0,00	ENSG00000109189
12,99	0,01	0,02	ENSG00000226887
7,57	0,00	0,00	ENSG00000128045
9,79	0,00	0,00	ENSG00000184178
5,03	0,00	0,00	ENSG00000145216
7,47	0,00	0,00	ENSG00000282278
2,70	0,00	0,00	ENSG00000072201
4,01	0,00	0,00	ENSG00000109220
1,14	0,97	0,98	ENSG00000180613
19,58	0,00	0,00	ENSG00000134853
2,90	0,00	0,00	ENSG00000157404
5,90	0,00	0,00	ENSG00000128052
1,10	0,33	0,44	ENSG00000128039
0,97	0,91	0,97	ENSG00000288695
0,83	0,03	0,06	ENSG00000134851
0,89	0,17	0,27	ENSG00000134852
0,07	0,31	0,43	ENSG00000163440
2,21	0,00	0,00	ENSG00000109255
1,14	0,97	0,98	ENSG00000250821
1,13	0,16	0,25	ENSG00000090989
0,69	0,00	0,00	ENSG00000174799
0,26	0,00	0,00	ENSG00000109265
0,76	0,01	0,02	ENSG00000157426
1,03	0,72	0,81	ENSG00000128059
1,00	0,97	0,98	ENSG00000128050
0,83	0,03	0,05	ENSG00000174780
0,02	0,15	0,23	ENSG00000196503
1,14	0,97	0,98	ENSG00000249693
1,40	0,44	0,56	ENSG00000171476
0,02	0,13	0,21	ENSG00000280285
1,14	0,97	#N/D	ENSG00000128040
1,11	0,20	0,30	ENSG00000084093
1,18	0,06	0,11	ENSG00000084092
0,98	0,85	0,92	ENSG00000047315
1,75	0,00	0,00	ENSG00000163453
73,65	0,12	0,19	ENSG00000150471
#N/D	#N/D	#N/D	ENSG00000205678
5,31	0,00	0,00	ENSG00000145242
0,86	0,10	0,16	ENSG00000145241
1,14	0,97	0,98	ENSG00000035720
0,88	0,69	0,78	ENSG00000279464
0,59	0,00	0,00	ENSG00000033178

0,56	0,20	0,30	ENSG00000109163
0,69	0,45	0,56	ENSG00000153802
0,50	0,32	0,43	ENSG00000187054
#N/D	#N/D	#N/D	ENSG00000198092
1,14	0,97	#N/D	ENSG00000185873
0,89	0,18	0,27	ENSG00000083896
1,14	0,97	#N/D	ENSG00000087128
#N/D	#N/D	#N/D	ENSG00000197888
#N/D	#N/D	#N/D	ENSG00000196620
1,14	0,97	#N/D	ENSG00000109181
#N/D	#N/D	#N/D	ENSG00000135220
1,14	0,97	0,98	ENSG00000171234
#N/D	#N/D	#N/D	ENSG00000213759
#N/D	#N/D	#N/D	ENSG00000135226
22,89	0,26	#N/D	ENSG00000156096
15,64	0,33	#N/D	ENSG00000271271
1,14	0,97	#N/D	ENSG00000173610
5,56	0,00	0,00	ENSG00000173597
#N/D	#N/D	#N/D	ENSG00000284695
8,39	0,46	0,56	ENSG00000109193
#N/D	#N/D	#N/D	ENSG00000126545
#N/D	#N/D	#N/D	ENSG00000135222
8,39	0,46	#N/D	ENSG00000126549
#N/D	#N/D	#N/D	ENSG00000205649
#N/D	#N/D	#N/D	ENSG00000126550
#N/D	#N/D	#N/D	ENSG00000187533
#N/D	#N/D	#N/D	ENSG00000109205
2,80	0,20	0,30	ENSG00000181617
#N/D	#N/D	#N/D	ENSG00000171209
1,14	0,97	#N/D	ENSG00000145309
#N/D	#N/D	#N/D	ENSG00000109208
#N/D	#N/D	#N/D	ENSG00000171201
1,14	0,97	#N/D	ENSG00000171199
1,14	0,97	#N/D	ENSG00000171195
0,20	0,00	0,00	ENSG00000187689
0,07	0,31	#N/D	ENSG00000178522
1,14	0,97	0,98	ENSG00000132464
8,39	0,46	0,56	ENSG00000132465
0,79	0,01	0,03	ENSG00000132467
0,91	0,34	0,46	ENSG00000018189
0,87	0,10	0,17	ENSG00000132463
1,14	0,14	0,22	ENSG00000173542
0,88	0,16	0,25	ENSG00000156136
0,41	0,01	0,02	ENSG00000080493
#N/D	#N/D	#N/D	ENSG00000145321
1,14	0,97	0,98	ENSG00000056291
1,37	0,00	0,00	ENSG00000156140
0,88	0,16	0,26	ENSG00000163626

0,90	0,21	0,31	ENSG00000132466
0,03	0,17	0,26	ENSG00000163631
0,23	0,00	0,00	ENSG00000081051
#N/D	#N/D	#N/D	ENSG00000079557
0,07	0,31	0,43	ENSG00000169435
0,35	0,00	0,00	ENSG00000169429
1,14	0,97	0,98	ENSG00000124875
4,62	0,00	0,00	ENSG00000109272
0,12	0,00	0,00	ENSG00000163739
15,64	0,33	0,44	ENSG00000163737
22,89	0,26	0,37	ENSG00000288796
#N/D	#N/D	#N/D	ENSG00000163736
0,13	0,46	0,56	ENSG00000163735
0,12	0,00	0,00	ENSG00000163734
0,02	0,00	0,00	ENSG00000081041
0,36	0,00	0,00	ENSG00000163738
0,62	0,39	0,51	ENSG00000182585
613,85	0,00	0,00	ENSG00000124882
0,36	0,08	0,14	ENSG00000109321
0,01	0,05	0,10	ENSG00000174808
0,15	0,01	0,02	ENSG00000169116
0,46	0,00	0,00	ENSG00000163743
0,69	0,00	0,00	ENSG00000174796
3,56	0,23	0,34	ENSG00000174792
8,39	0,46	0,56	ENSG00000138769
1,01	0,91	0,97	ENSG00000138757
0,91	0,25	0,35	ENSG00000138768
8,39	0,46	0,56	ENSG00000156194
0,93	0,47	0,58	ENSG00000138744
0,54	0,00	0,00	ENSG00000198301
1,14	0,97	#N/D	ENSG00000138755
8,39	0,46	0,56	ENSG00000156219
3,01	0,00	0,00	ENSG00000169245
1,93	0,16	0,25	ENSG00000169248
0,60	0,00	0,00	ENSG00000138750
0,81	0,01	0,02	ENSG00000138760
0,22	0,14	0,22	ENSG00000189157
0,57	0,24	0,35	ENSG00000272414
1,09	0,45	0,56	ENSG00000118804
1,14	0,97	0,98	ENSG00000163749
1,97	0,00	0,00	ENSG00000138771
1,44	0,68	0,77	ENSG00000186212
1,01	0,88	0,94	ENSG00000138758
1,11	0,18	0,28	ENSG00000118816
1,82	0,00	0,00	ENSG00000138764
1,14	0,97	#N/D	ENSG00000156234
1,42	0,00	0,00	ENSG00000138767
0,52	0,00	0,00	ENSG00000169288

0,32	0,00	0,00	ENSG00000138759
0,53	0,58	0,68	ENSG00000138772
0,45	0,00	0,00	ENSG00000138756
0,56	0,00	0,00	ENSG00000163291
0,07	0,31	0,43	ENSG00000156269
1,14	0,97	#N/D	ENSG00000196475
1,65	0,00	0,00	ENSG00000163297
1,20	0,03	0,07	ENSG00000152784
1,66	0,00	0,00	ENSG00000138675
1,79	0,22	0,32	ENSG00000197826
22,89	0,26	0,37	ENSG00000152785
2,53	0,17	0,26	ENSG00000138669
30,14	0,22	0,32	ENSG00000138670
0,64	0,00	0,00	ENSG00000138668
0,82	0,02	0,03	ENSG00000152795
0,92	0,32	0,43	ENSG00000145293
0,54	0,00	0,00	ENSG00000249242
1,16	0,10	0,16	ENSG00000145284
1,82	0,00	0,00	ENSG00000138674
0,50	0,00	0,00	ENSG00000168152
0,91	0,29	0,40	ENSG00000189308
0,97	0,69	0,78	ENSG00000138663
2,71	0,36	0,48	ENSG00000145287
0,59	0,00	0,00	ENSG00000173085
0,19	0,00	0,00	ENSG00000173083
0,53	0,00	0,00	ENSG00000163312
1,20	0,12	0,19	ENSG00000163319
0,75	0,03	0,06	ENSG00000163322
1,56	0,00	0,00	ENSG00000138678
1,76	0,00	0,00	ENSG00000163623
0,95	0,69	0,78	ENSG00000163624
1,00	0,95	0,98	ENSG00000163625
1,71	0,00	0,00	ENSG00000138639
2,70	0,00	0,00	ENSG00000109339
0,60	0,00	0,00	ENSG00000163629
1,14	0,97	0,98	ENSG00000145283
0,05	0,00	0,00	ENSG00000163633
0,28	0,01	0,02	ENSG00000285458
0,46	0,00	0,00	ENSG00000172493
0,66	0,00	0,00	ENSG00000145332
0,67	0,65	0,74	ENSG00000170509
0,81	0,02	0,05	ENSG00000198189
0,79	0,02	0,04	ENSG00000170502
#N/D	#N/D	#N/D	ENSG00000272856
0,03	0,17	0,26	ENSG00000152583
1,14	0,97	#N/D	ENSG00000152591
1,14	0,97	#N/D	ENSG00000152592
#N/D	#N/D	#N/D	ENSG00000029559

1,14	0,97	#N/D	ENSG00000152595
0,13	0,46	0,56	ENSG00000118785
0,78	0,01	0,02	ENSG00000118762
0,53	0,00	0,00	ENSG00000118777
1,37	0,00	0,00	ENSG00000163644
0,39	0,00	0,00	ENSG00000138642
0,50	0,00	0,00	ENSG00000138646
0,92	0,37	0,48	ENSG00000255072
0,91	0,34	0,45	ENSG00000145337
1,14	0,46	0,57	ENSG00000287542
1,28	0,00	0,01	ENSG00000138641
0,53	0,00	0,00	ENSG00000177432
1,15	0,14	0,22	ENSG00000138640
0,62	0,00	0,00	ENSG00000180346
0,31	0,00	0,00	ENSG00000185477
0,23	0,00	0,00	ENSG00000145335
22,89	0,26	#N/D	ENSG00000138722
7,00	0,00	0,00	ENSG00000184305
#N/D	#N/D	#N/D	ENSG00000279013
#N/D	#N/D	#N/D	ENSG00000280056
0,13	0,01	0,01	ENSG00000152208
0,02	0,10	0,17	ENSG00000172238
0,46	0,00	0,00	ENSG00000163104
0,07	0,31	0,43	ENSG00000163106
0,38	0,00	0,00	ENSG00000163110
0,11	0,00	0,00	ENSG00000138696
0,26	0,00	0,00	ENSG00000182168
#N/D	#N/D	#N/D	ENSG00000163114
1,44	0,68	0,77	ENSG00000163116
0,94	0,48	0,58	ENSG00000138698
1,60	0,00	0,00	ENSG00000168785
0,89	0,19	0,28	ENSG00000151247
0,88	0,12	0,19	ENSG00000164024
1,00	0,96	0,98	ENSG00000197894
1,14	0,97	0,98	ENSG00000198099
1,14	0,97	0,98	ENSG00000172955
#N/D	#N/D	#N/D	ENSG00000187758
1,14	0,97	#N/D	ENSG00000196616
1,14	0,97	0,98	ENSG00000248144
#N/D	#N/D	#N/D	ENSG00000196344
1,14	0,97	#N/D	ENSG00000138813
1,17	0,32	0,43	ENSG00000145331
0,09	0,01	0,03	ENSG00000138823
1,14	0,97	0,98	ENSG00000248713
2,71	0,36	0,48	ENSG00000070190
#N/D	#N/D	#N/D	ENSG00000279913
1,44	0,00	0,00	ENSG00000109270
0,77	0,01	0,02	ENSG00000164031

1,14	0,97	0,98	ENSG00000279098
1,09	0,31	0,42	ENSG00000164032
2,07	0,03	0,06	ENSG00000145358
#N/D	#N/D	#N/D	ENSG00000164035
1,07	0,41	0,53	ENSG00000138814
0,82	0,74	0,82	ENSG00000153064
0,49	0,00	0,00	ENSG00000138821
0,45	0,00	0,00	ENSG00000109320
0,78	0,01	0,02	ENSG00000109323
0,82	0,01	0,03	ENSG00000109332
0,73	0,00	0,00	ENSG00000145354
0,98	0,97	0,99	ENSG00000164037
0,78	0,01	0,02	ENSG00000164038
0,75	0,01	0,03	ENSG00000164039
0,51	0,00	0,00	ENSG00000138778
8,39	0,46	0,56	ENSG00000169836
0,70	0,02	0,04	ENSG00000168772
1,08	0,39	0,51	ENSG00000168769
0,73	0,00	0,00	ENSG00000138777
0,99	1,00	1,00	ENSG00000236699
1,06	0,52	0,62	ENSG00000138785
0,67	0,00	0,00	ENSG00000138780
0,13	0,46	0,56	ENSG00000168743
0,78	0,01	0,01	ENSG00000145348
1,00	0,97	0,99	ENSG00000164022
1,14	0,97	#N/D	ENSG00000250298
1,26	0,10	0,16	ENSG00000155011
1,17	0,06	0,10	ENSG00000138801
0,79	0,01	0,02	ENSG00000164023
0,92	0,51	0,62	ENSG00000155016
0,88	0,13	0,21	ENSG00000138796
0,42	0,00	0,00	ENSG00000138795
1,16	0,07	0,12	ENSG00000109475
1,46	0,00	0,00	ENSG00000198856
8,39	0,46	0,56	ENSG00000164089
8,39	0,46	0,56	ENSG00000188517
0,88	0,13	0,21	ENSG00000138802
0,63	0,00	0,00	ENSG00000005059
0,60	0,00	0,00	ENSG00000138794
0,96	0,69	0,78	ENSG00000123739
0,69	0,33	0,44	ENSG00000285330
1,08	0,87	0,93	ENSG00000205403
0,73	0,01	0,02	ENSG00000109534
0,60	0,12	0,19	ENSG00000180245
1,45	0,37	0,48	ENSG00000183423
0,83	0,23	0,33	ENSG00000138798
0,53	0,00	0,00	ENSG00000170522
8,39	0,46	0,56	ENSG00000138792

1,92	0,00	0,00	ENSG00000164093
0,50	0,00	0,00	ENSG00000174749
0,50	0,00	0,00	ENSG00000138660
1,01	0,94	0,98	ENSG00000145365
0,60	0,00	0,00	ENSG00000073331
0,01	0,00	0,00	ENSG00000178403
0,92	0,37	0,48	ENSG00000138658
0,77	0,01	0,03	ENSG00000174720
0,13	0,00	0,00	ENSG00000145362
1,02	0,83	0,90	ENSG00000145349
0,82	0,02	0,04	ENSG00000180801
1.886,38	0,01	0,01	ENSG00000174607
#N/D	#N/D	#N/D	ENSG00000138653
0,89	0,54	0,64	ENSG00000174599
10,42	0,02	0,04	ENSG00000164100
2,84	0,00	0,00	ENSG00000164099
0,68	0,00	0,00	ENSG00000145388
1,53	0,00	0,00	ENSG00000150961
0,35	0,00	0,00	ENSG00000172403
0,07	0,31	0,43	ENSG00000172399
0,47	0,00	0,00	ENSG00000145390
2,92	0,00	0,00	ENSG00000164096
8,39	0,46	0,56	ENSG00000145384
9,15	0,00	0,00	ENSG00000138735
0,93	0,37	0,48	ENSG00000164109
0,58	0,00	0,00	ENSG00000138738
0,09	0,00	0,00	ENSG00000173376
0,22	0,00	0,00	ENSG00000050730
0,37	0,00	0,00	ENSG00000186867
1,26	0,00	0,01	ENSG00000164111
0,94	0,82	0,89	ENSG00000164112
0,95	0,52	0,63	ENSG00000123737
0,74	0,00	0,00	ENSG00000145386
0,69	0,00	0,00	ENSG00000138686
0,65	0,04	0,07	ENSG00000138741
1,05	0,51	0,62	ENSG00000138688
#N/D	#N/D	#N/D	ENSG00000164113
#N/D	#N/D	#N/D	ENSG00000109471
22,89	0,26	#N/D	ENSG00000138684
0,73	0,01	0,02	ENSG00000181004
0,99	0,94	0,98	ENSG00000138685
1,32	0,00	0,01	ENSG00000170917
0,94	0,47	0,58	ENSG00000145375
0,25	0,00	0,00	ENSG00000164056
0,72	0,00	0,00	ENSG00000151458
1,83	0,00	0,00	ENSG00000196159
0,73	0,00	0,01	ENSG00000164066
#N/D	#N/D	#N/D	ENSG00000151475

0,13	0,00	0,00	ENSG00000164070
0,58	0,00	0,00	ENSG00000142731
0,94	0,58	0,68	ENSG00000164073
0,82	0,06	0,10	ENSG00000164074
1,09	0,35	0,47	ENSG00000138709
1,29	0,00	0,01	ENSG00000164040
0,67	0,00	0,00	ENSG00000077684
0,77	0,01	0,02	ENSG00000151466
1,15	0,32	0,43	ENSG00000151470
#N/D	#N/D	#N/D	ENSG00000280059
0,51	0,00	0,00	ENSG00000138650
1,61	0,00	0,00	ENSG00000254535
#N/D	#N/D	#N/D	ENSG00000279749
3,40	0,00	0,00	ENSG00000189184
#N/D	#N/D	#N/D	ENSG00000280352
1,46	0,00	0,00	ENSG00000151012
1,35	0,00	0,00	ENSG00000151014
1,12	0,20	0,30	ENSG00000109381
2,87	0,00	0,00	ENSG00000137463
0,82	0,04	0,07	ENSG00000109390
0,65	0,00	0,00	ENSG00000164134
1,44	0,00	0,00	ENSG00000172007
0,86	0,07	0,12	ENSG00000145391
0,60	0,00	0,00	ENSG00000085871
0,55	0,00	0,00	ENSG00000196782
0,85	0,06	0,12	ENSG00000153130
0,20	0,00	0,00	ENSG00000153132
1,14	0,97	#N/D	ENSG00000205301
1,14	0,97	#N/D	ENSG00000280262
0,89	0,25	0,36	ENSG00000179387
1,14	0,97	#N/D	ENSG00000109424
1,08	0,36	0,48	ENSG00000109436
0,61	0,00	0,00	ENSG00000170153
0,89	0,20	0,30	ENSG00000109445
0,80	0,14	0,22	ENSG00000164136
0,20	0,00	0,00	ENSG00000109452
0,72	0,00	0,00	ENSG00000170185
0,93	0,57	0,67	ENSG00000109458
0,64	0,00	0,00	ENSG00000153147
#N/D	#N/D	#N/D	ENSG00000183090
0,97	0,94	0,98	ENSG00000197465
#N/D	#N/D	#N/D	ENSG00000250361
1,14	0,97	#N/D	ENSG00000170180
1,16	0,63	0,73	ENSG00000285713
0,03	0,00	0,00	ENSG00000164161
0,81	0,05	0,10	ENSG00000164162
0,62	0,00	0,00	ENSG00000164163
0,81	0,01	0,02	ENSG00000164164

1,14	0,97	#N/D	ENSG00000278943
0,40	0,00	0,00	ENSG00000170365
0,84	0,18	0,27	ENSG00000151611
1,14	0,97	#N/D	ENSG00000237136
0,68	0,00	0,00	ENSG00000151612
0,58	0,17	0,26	ENSG00000279481
0,66	0,00	0,00	ENSG00000164167
1,14	0,97	0,98	ENSG00000279845
0,05	0,24	0,35	ENSG00000250673
1,07	0,57	0,67	ENSG00000120519
#N/D	#N/D	#N/D	ENSG00000151615
0,98	0,97	0,98	ENSG00000137473
#N/D	#N/D	#N/D	ENSG00000279376
1,49	0,04	0,07	ENSG00000151617
3,29	0,01	0,01	ENSG00000280219
0,92	0,37	0,49	ENSG00000164168
0,57	0,00	0,00	ENSG00000164169
0,85	0,08	0,13	ENSG00000071205
0,50	0,00	0,00	ENSG00000151623
0,13	0,46	#N/D	ENSG00000279016
1,14	0,97	0,98	ENSG00000234828
1,41	0,00	0,00	ENSG00000170390
0,77	0,00	0,00	ENSG00000198589
0,35	0,00	0,00	ENSG00000181541
1,13	0,13	0,21	ENSG00000145425
0,89	0,16	0,25	ENSG00000109686
2,88	0,03	0,05	ENSG00000189099
1,51	0,00	0,00	ENSG00000164142
1,04	0,68	0,77	ENSG00000059691
0,98	0,88	0,95	ENSG00000109670
0,70	0,00	0,00	ENSG00000170006
0,11	0,03	0,05	ENSG00000169989
1,47	0,00	0,00	ENSG00000164144
0,35	0,00	0,00	ENSG00000137460
0,61	0,00	0,00	ENSG00000109654
0,33	0,00	0,00	ENSG00000288637
0,42	0,00	0,00	ENSG00000121211
0,44	0,00	0,00	ENSG00000121210
1,08	0,59	0,69	ENSG00000137462
0,41	0,00	0,00	ENSG00000145428
0,13	0,46	#N/D	ENSG00000145423
2,02	0,00	0,00	ENSG00000197410
0,68	0,00	0,00	ENSG00000171566
0,13	0,46	0,56	ENSG00000171564
1,14	0,97	#N/D	ENSG00000171560
1,14	0,97	0,98	ENSG00000171557
0,61	0,14	0,23	ENSG00000121207
0,34	0,18	0,27	ENSG00000151962

8,39	0,46	0,56	ENSG00000185149
0,73	0,00	0,00	ENSG00000164114
0,04	0,00	0,00	ENSG00000164116
0,86	0,11	0,19	ENSG00000061918
1,14	0,97	#N/D	ENSG00000256394
0,04	0,00	0,00	ENSG00000151790
0,86	0,31	0,42	ENSG00000256043
1,02	0,82	0,90	ENSG00000145431
1,14	0,97	0,98	ENSG00000280284
8,39	0,46	0,56	ENSG00000278880
0,24	0,00	0,00	ENSG00000109738
1,14	0,97	0,98	ENSG00000120251
#N/D	#N/D	#N/D	ENSG00000279703
#N/D	#N/D	#N/D	ENSG00000279994
#N/D	#N/D	#N/D	ENSG00000279139
#N/D	#N/D	#N/D	ENSG00000280140
0,38	0,00	0,00	ENSG00000164125
0,59	0,00	0,00	ENSG00000164124
233,17	0,05	0,09	ENSG00000171509
3,90	0,00	0,00	ENSG00000205208
4,33	0,00	0,00	ENSG00000171503
9,25	0,00	0,00	ENSG00000171497
1,02	0,86	0,93	ENSG00000052795
1,14	0,97	0,98	ENSG00000164123
0,86	0,07	0,13	ENSG00000109756
16,92	0,00	0,00	ENSG00000168843
0,77	0,00	0,01	ENSG00000145414
0,97	0,94	0,98	ENSG00000164128
1,14	0,97	0,98	ENSG00000164129
15,64	0,33	0,44	ENSG00000151005
0,56	0,00	0,00	ENSG00000198498
0,96	0,71	0,80	ENSG00000145416
1,14	0,97	0,98	ENSG00000248771
#N/D	#N/D	#N/D	ENSG00000248329
0,14	0,05	0,09	ENSG00000183439
#N/D	#N/D	#N/D	ENSG00000176979
0,40	0,12	0,19	ENSG00000250374
0,96	0,60	0,70	ENSG00000170088
0,60	0,00	0,00	ENSG00000109466
1,09	0,80	0,87	ENSG00000229894
5,15	0,00	0,00	ENSG00000052802
2,02	0,00	0,00	ENSG00000109472
0,85	0,55	0,65	ENSG00000038295
4,51	0,00	0,00	ENSG00000196104
1,85	0,60	0,69	ENSG00000109511
0,93	0,36	0,47	ENSG00000137628
0,50	0,00	0,00	ENSG00000181381
1,25	0,00	0,01	ENSG00000129116

0,83	0,58	0,68	ENSG00000279384
1,00	0,97	0,98	ENSG00000145439
0,70	0,00	0,00	ENSG00000154447
0,61	0,00	0,00	ENSG00000137601
0,53	0,00	0,00	ENSG00000109572
0,61	0,00	0,00	ENSG00000056050
0,47	0,00	0,00	ENSG00000198948
0,69	0,00	0,01	ENSG00000109576
0,50	0,00	0,01	ENSG00000174473
1,50	0,00	0,00	ENSG00000109586
1,60	0,00	0,00	ENSG00000164104
0,76	0,14	0,22	ENSG00000164105
1,59	0,18	0,27	ENSG00000164106
0,37	0,00	0,00	ENSG00000164107
1,28	0,01	0,02	ENSG00000164117
1,59	0,00	0,00	ENSG00000164118
3,56	0,23	0,34	ENSG00000164120
15,64	0,33	0,44	ENSG00000145451
1,14	0,97	0,98	ENSG00000168594
0,03	0,00	0,00	ENSG00000150625
0,39	0,00	0,00	ENSG00000150627
0,34	0,10	0,16	ENSG00000150628
8,39	0,46	0,56	ENSG00000164122
1,11	0,20	0,30	ENSG00000129128
2,97	0,00	0,00	ENSG00000150630
1,95	0,00	0,00	ENSG00000109674
1,02	0,86	0,93	ENSG00000038002
0,40	0,00	0,00	ENSG00000218336
0,76	0,00	0,00	ENSG00000129187
1,02	0,77	0,85	ENSG00000151718
#N/D	#N/D	#N/D	ENSG00000177300
1,25	0,66	0,75	ENSG00000185758
1,15	0,13	0,21	ENSG00000168564
1,45	0,01	0,02	ENSG00000168556
0,69	0,00	0,00	ENSG00000182552
0,89	0,16	0,25	ENSG00000168538
1,35	0,01	0,02	ENSG00000173320
0,62	0,33	0,44	ENSG00000283355
0,53	0,58	0,68	ENSG00000164303
0,86	0,07	0,13	ENSG00000168310
0,83	0,05	0,09	ENSG00000164305
0,46	0,00	0,00	ENSG00000164306
0,68	0,00	0,00	ENSG00000151725
0,62	0,00	0,00	ENSG00000151726
#N/D	#N/D	#N/D	ENSG00000279460
#N/D	#N/D	#N/D	ENSG00000187821
0,95	0,60	0,70	ENSG00000151729
0,61	0,00	0,00	ENSG00000164323

0,85	0,09	0,16	ENSG00000109762
0,70	0,06	0,10	ENSG00000109771
2,17	0,00	0,00	ENSG00000186352
1,38	0,00	0,00	ENSG00000109775
6,13	0,07	0,13	ENSG00000205129
22,89	0,26	0,37	ENSG00000168491
0,41	0,00	0,00	ENSG00000154553
0,18	0,00	0,00	ENSG00000154556
1,06	0,71	0,79	ENSG00000164342
1,62	0,00	0,01	ENSG00000109794
0,37	0,01	0,01	ENSG00000279943
0,33	0,00	0,00	ENSG00000145476
0,13	0,46	0,56	ENSG00000290316
1,14	0,97	0,98	ENSG00000164344
1,14	0,97	0,98	ENSG00000088926
0,34	0,10	0,16	ENSG00000272297
1,85	0,60	0,69	ENSG00000168412
0,77	0,00	0,00	ENSG00000083857
1,85	0,60	0,69	ENSG00000179059
0,01	0,09	0,16	ENSG00000179046
1,14	0,97	#N/D	ENSG00000184108
0,77	0,04	0,08	ENSG00000278974
0,98	0,82	0,89	ENSG00000109536
1,14	0,97	0,98	ENSG00000205097
0,03	0,17	0,26	ENSG00000260596
8,12	0,00	0,00	ENSG00000153404
0,92	0,81	0,89	ENSG00000185028
0,88	0,14	0,22	ENSG00000164366
1,37	0,01	0,03	ENSG00000286001
1,16	0,06	0,10	ENSG00000073578
0,98	0,81	0,88	ENSG00000249915
1,04	0,73	0,81	ENSG00000288622
0,93	0,38	0,49	ENSG00000063438
0,62	0,00	0,00	ENSG00000180104
0,56	0,00	0,00	ENSG00000066230
0,63	0,00	0,00	ENSG00000112877
0,51	0,00	0,00	ENSG00000171368
0,63	0,00	0,00	ENSG00000206077
0,21	0,00	0,00	ENSG00000188818
0,66	0,00	0,00	ENSG00000028310
0,60	0,00	0,00	ENSG00000071539
0,84	0,04	0,07	ENSG00000145506
0,83	0,02	0,04	ENSG00000113504
15,64	0,33	0,44	ENSG00000174358
15,64	0,33	#N/D	ENSG00000164363
0,77	0,05	0,10	ENSG00000164362
0,67	0,00	0,00	ENSG00000049656
0,05	0,24	#N/D	ENSG00000279908

0,41	0,27	0,38	ENSG00000142319
1,01	0,92	0,98	ENSG00000153395
0,63	0,00	0,00	ENSG00000171421
0,54	0,00	0,00	ENSG00000145494
#N/D	#N/D	#N/D	ENSG00000279222
44,64	0,17	0,26	ENSG00000113430
0,47	0,00	0,00	ENSG00000170561
0,46	0,00	0,00	ENSG00000170549
0,02	0,00	0,00	ENSG00000145536
0,57	0,00	0,00	ENSG00000164151
#N/D	#N/D	#N/D	ENSG00000278913
0,82	0,02	0,04	ENSG00000133398
22,89	0,26	0,37	ENSG00000215218
0,69	0,00	0,00	ENSG00000037474
0,82	0,03	0,05	ENSG00000145545
0,88	0,12	0,20	ENSG00000112941
22,89	0,26	0,37	ENSG00000078295
1,14	0,97	0,98	ENSG00000215217
0,54	0,00	0,00	ENSG00000124275
0,56	0,00	0,00	ENSG00000124279
#N/D	#N/D	#N/D	ENSG00000280338
#N/D	#N/D	#N/D	ENSG00000280251
1,92	0,23	0,34	ENSG00000279075
#N/D	#N/D	#N/D	ENSG00000279097
#N/D	#N/D	#N/D	ENSG00000279264
#N/D	#N/D	#N/D	ENSG00000279002
#N/D	#N/D	#N/D	ENSG00000280373
#N/D	#N/D	#N/D	ENSG00000279125
#N/D	#N/D	#N/D	ENSG00000280438
#N/D	#N/D	#N/D	ENSG00000278877
#N/D	#N/D	#N/D	ENSG00000280185
#N/D	#N/D	#N/D	ENSG00000279635
1,73	0,00	0,00	ENSG00000112902
1,44	0,68	0,77	ENSG00000279698
0,57	0,36	0,47	ENSG00000169777
#N/D	#N/D	#N/D	ENSG00000279011
0,05	0,24	#N/D	ENSG00000279755
1,14	0,97	#N/D	ENSG00000278874
1,14	0,97	#N/D	ENSG00000280016
0,53	0,58	0,68	ENSG00000280449
0,07	0,31	#N/D	ENSG00000280368
0,46	0,00	0,00	ENSG00000150756
0,43	0,00	0,00	ENSG00000150753
0,78	0,00	0,01	ENSG00000164237
0,18	0,10	0,16	ENSG00000280369
0,72	0,00	0,00	ENSG00000145495
1,74	0,17	0,26	ENSG00000145491
#N/D	#N/D	#N/D	ENSG00000280017

1,14	0,97	0,98	ENSG00000279450
0,27	0,00	0,00	ENSG00000164236
8,39	0,46	0,56	ENSG00000279979
0,74	0,69	0,78	ENSG00000278865
1,12	0,14	0,22	ENSG00000112977
2,66	0,00	0,01	ENSG00000169862
0,67	0,00	0,00	ENSG00000039139
0,70	0,00	0,00	ENSG00000038382
0,35	0,00	0,00	ENSG00000145569
0,70	0,00	0,00	ENSG00000154124
0,69	0,00	0,00	ENSG00000154122
1,79	0,00	0,00	ENSG00000183580
#N/D	#N/D	#N/D	ENSG00000183654
0,87	0,09	0,15	ENSG00000173545
0,04	0,00	0,00	ENSG00000154153
0,81	0,01	0,02	ENSG00000145555
0,42	0,00	0,00	ENSG00000176788
#N/D	#N/D	#N/D	ENSG00000249662
#N/D	#N/D	#N/D	ENSG00000268799
#N/D	#N/D	#N/D	ENSG00000284373
#N/D	#N/D	#N/D	ENSG00000284439
#N/D	#N/D	#N/D	ENSG00000284283
#N/D	#N/D	#N/D	ENSG00000284234
#N/D	#N/D	#N/D	ENSG00000284042
#N/D	#N/D	#N/D	ENSG00000284465
#N/D	#N/D	#N/D	ENSG00000283967
#N/D	#N/D	#N/D	ENSG00000283988
#N/D	#N/D	#N/D	ENSG00000284356
0,53	0,58	0,68	ENSG00000283740
0,03	0,17	0,26	ENSG00000249156
1,14	0,97	#N/D	ENSG00000283776
#N/D	#N/D	#N/D	ENSG00000250782
1,14	0,97	#N/D	ENSG00000269466
0,25	0,00	0,00	ENSG00000145526
0,30	0,07	0,12	ENSG00000154162
#N/D	#N/D	#N/D	ENSG00000164256
0,19	0,00	0,00	ENSG00000040731
#N/D	#N/D	#N/D	ENSG00000279671
#N/D	#N/D	#N/D	ENSG00000279531
#N/D	#N/D	#N/D	ENSG00000279869
0,13	0,46	#N/D	ENSG00000113100
1,14	0,97	#N/D	ENSG00000280305
3,24	0,00	0,00	ENSG00000113361
0,52	0,00	0,00	ENSG00000113360
0,38	0,00	0,00	ENSG00000082213
0,53	0,03	0,06	ENSG00000279923
0,57	0,00	0,00	ENSG00000133401
0,69	0,00	0,00	ENSG00000113384

0,39	0,00	0,00	ENSG00000150712
0,46	0,00	0,00	ENSG00000056097
0,34	0,00	0,00	ENSG00000113387
0,06	0,00	0,00	ENSG00000113389
0,39	0,00	0,00	ENSG00000113407
0,23	0,00	0,00	ENSG00000151388
1,14	0,97	#N/D	ENSG00000182631
0,11	0,03	0,05	ENSG00000164175
0,30	0,00	0,00	ENSG00000242110
0,41	0,00	0,00	ENSG00000273294
0,67	0,32	0,43	ENSG00000289791
1,15	0,61	0,71	ENSG00000082196
15,64	0,33	0,44	ENSG00000278900
1,14	0,97	#N/D	ENSG00000279995
0,55	0,00	0,00	ENSG00000039560
0,78	0,22	0,32	ENSG00000205838
1,10	0,22	0,33	ENSG00000113456
0,66	0,00	0,00	ENSG00000113460
0,61	0,00	0,00	ENSG00000168724
0,13	0,46	#N/D	ENSG00000113492
0,18	0,00	0,00	ENSG00000113494
0,77	0,16	0,25	ENSG00000152582
0,70	0,00	0,00	ENSG00000168685
#N/D	#N/D	#N/D	ENSG00000279737
0,04	0,20	0,30	ENSG00000152611
1,14	0,97	#N/D	ENSG00000145626
1,45	0,42	0,53	ENSG00000168671
0,60	0,00	0,00	ENSG00000164187
0,46	0,00	0,00	ENSG00000145604
0,68	0,00	0,00	ENSG00000152620
0,88	0,70	0,78	ENSG00000164188
0,11	0,00	0,00	ENSG00000079215
0,54	0,00	0,00	ENSG00000164190
0,40	0,00	0,00	ENSG00000197603
0,53	0,00	0,00	ENSG00000113569
0,54	0,00	0,00	ENSG00000082068
0,26	0,00	0,00	ENSG00000168621
0,29	0,12	0,19	ENSG00000164318
0,37	0,00	0,00	ENSG00000113594
0,40	0,00	0,00	ENSG00000145623
0,49	0,00	0,00	ENSG00000164327
0,01	0,09	0,15	ENSG00000082074
0,13	0,46	#N/D	ENSG00000113600
0,85	0,04	0,08	ENSG00000153071
0,72	0,00	0,00	ENSG00000113638
0,71	0,00	0,01	ENSG00000171522
0,59	0,00	0,00	ENSG00000132356
0,66	0,00	0,00	ENSG00000145592

0,50	0,00	0,00	ENSG00000132357
22,89	0,26	0,37	ENSG00000112936
1,14	0,97	#N/D	ENSG00000171495
1,14	0,97	0,98	ENSG00000039537
15,71	0,00	0,00	ENSG00000182836
0,29	0,00	0,00	ENSG00000083720
0,52	0,00	0,00	ENSG00000205765
0,26	0,00	0,00	ENSG00000151876
0,57	0,00	0,00	ENSG00000112964
2,09	0,00	0,00	ENSG00000198865
1,11	0,70	0,79	ENSG00000250722
0,48	0,00	0,00	ENSG00000177721
0,64	0,00	0,00	ENSG00000172262
0,09	0,00	0,00	ENSG00000177453
3,71	0,00	0,00	ENSG00000112972
12,50	0,00	0,00	ENSG00000151882
0,35	0,00	0,00	ENSG00000151881
0,39	0,00	0,00	ENSG00000172244
0,49	0,00	0,00	ENSG00000172239
1,35	0,21	0,31	ENSG00000279557
0,46	0,00	0,00	ENSG00000112992
0,13	0,46	0,56	ENSG00000070193
0,42	0,00	0,00	ENSG00000112996
1,21	0,68	0,77	ENSG00000164588
1,45	0,00	0,00	ENSG00000170571
0,59	0,00	0,00	ENSG00000151883
2,59	0,00	0,00	ENSG00000016082
#N/D	#N/D	#N/D	ENSG00000279470
1,20	0,03	0,05	ENSG00000152684
1,07	0,48	0,58	ENSG00000213949
2,40	0,00	0,00	ENSG00000164171
1,08	0,38	0,49	ENSG00000164172
0,63	0,00	0,00	ENSG00000134363
1,36	0,00	0,00	ENSG00000164258
1,18	0,21	0,31	ENSG00000185305
#N/D	#N/D	#N/D	ENSG00000279638
37,39	0,19	0,29	ENSG00000169271
1,60	0,00	0,00	ENSG00000178996
15,64	0,33	0,44	ENSG00000280159
0,07	0,00	0,00	ENSG00000164283
#N/D	#N/D	#N/D	ENSG00000113088
8,39	0,46	#N/D	ENSG00000145649
5,28	0,10	0,17	ENSG00000164287
1,41	0,00	0,00	ENSG00000164294
0,24	0,00	0,00	ENSG00000234602
0,62	0,00	0,00	ENSG00000152669
1,49	0,00	0,00	ENSG00000067248
1,16	0,08	0,14	ENSG00000039123

1,46	0,00	0,00	ENSG00000067113
0,66	0,51	0,62	ENSG00000279883
0,91	0,38	0,49	ENSG00000177058
1,14	0,97	0,98	ENSG00000152670
0,64	0,00	0,00	ENSG00000164509
0,88	0,10	0,16	ENSG00000134352
0,20	0,00	0,00	ENSG00000164512
0,89	0,22	0,32	ENSG00000095015
0,58	0,00	0,00	ENSG00000155542
1,21	0,05	0,09	ENSG00000155545
1,21	0,02	0,05	ENSG00000062194
0,17	0,00	0,00	ENSG00000169067
0,62	0,00	0,00	ENSG00000145632
#N/D	#N/D	#N/D	ENSG00000175857
#N/D	#N/D	#N/D	ENSG00000152932
1,23	0,03	0,05	ENSG00000113448
0,93	0,39	0,50	ENSG00000035499
0,30	0,00	0,00	ENSG00000164181
0,95	0,61	0,70	ENSG00000049167
1,20	0,12	0,20	ENSG00000164182
1,32	0,33	0,45	ENSG00000279985
1,24	0,02	0,05	ENSG00000188725
1,67	0,00	0,00	ENSG00000130449
1,00	1,00	1,00	ENSG00000068796
1,14	0,97	0,98	ENSG00000288643
1,04	0,67	0,77	ENSG00000086189
0,98	0,79	0,87	ENSG00000086200
0,72	0,41	0,52	ENSG00000186105
1,14	0,97	#N/D	ENSG00000178394
15,64	0,33	0,44	ENSG00000164197
#N/D	#N/D	#N/D	ENSG00000186479
22,89	0,26	0,37	ENSG00000145642
1,15	0,13	0,21	ENSG00000153006
1,23	0,02	0,05	ENSG00000153015
0,20	0,00	0,00	ENSG00000049192
1,38	0,00	0,01	ENSG00000123219
1,16	0,12	0,20	ENSG00000113593
1,97	0,00	0,00	ENSG00000113595
1,09	0,42	0,54	ENSG00000113597
0,67	0,11	0,19	ENSG00000253251
1,37	0,00	0,00	ENSG00000197860
1,52	0,00	0,00	ENSG00000123213
1,30	0,00	0,00	ENSG00000112851
1,09	0,30	0,42	ENSG00000153914
0,82	0,03	0,06	ENSG00000069020
8,39	0,46	0,56	ENSG00000134061
1,57	0,00	0,00	ENSG00000145675
1,81	0,00	0,00	ENSG00000145740

1,32	0,00	0,00	ENSG00000134057
0,93	0,67	0,76	ENSG00000280187
1,65	0,00	0,00	ENSG00000153044
0,99	0,93	0,98	ENSG00000134056
1,13	0,15	0,24	ENSG00000134058
1,42	0,02	0,03	ENSG00000183323
0,76	0,07	0,13	ENSG00000085231
1,66	0,00	0,00	ENSG00000273841
0,97	0,71	0,80	ENSG00000152942
0,65	0,30	0,41	ENSG00000152939
0,14	0,00	0,00	ENSG00000197822
1,05	0,61	0,70	ENSG00000183474
1,05	0,62	0,72	ENSG00000205572
4,02	0,00	0,00	ENSG00000205571
0,88	0,48	0,58	ENSG00000172058
1,52	0,00	0,00	ENSG00000172062
1,51	0,00	0,00	ENSG00000249437
1,06	0,61	0,70	ENSG00000145736
0,92	0,36	0,48	ENSG00000145734
0,85	0,05	0,08	ENSG00000131844
#N/D	#N/D	#N/D	ENSG00000164326
1,50	0,00	0,00	ENSG00000131711
1,27	0,00	0,01	ENSG00000113048
0,99	0,88	0,94	ENSG00000049883
1,03	0,93	0,98	ENSG00000178175
1,10	0,25	0,36	ENSG00000083312
0,98	0,82	0,89	ENSG00000157107
0,24	0,00	0,00	ENSG00000157111
#N/D	#N/D	#N/D	ENSG00000164325
0,78	0,01	0,01	ENSG00000251493
1,01	0,86	0,93	ENSG00000145741
0,89	0,24	0,34	ENSG00000164331
0,96	0,66	0,75	ENSG00000164338
0,90	0,21	0,31	ENSG00000214944
0,50	0,00	0,00	ENSG00000171617
1,22	0,02	0,03	ENSG00000049860
1,18	0,05	0,10	ENSG00000164347
1,50	0,00	0,00	ENSG00000164346
0,63	0,00	0,00	ENSG00000198780
8,71	0,03	0,06	ENSG00000176928
0,13	0,46	0,56	ENSG00000145700
5,42	0,00	0,00	ENSG00000113161
1,01	0,94	0,98	ENSG00000113163
1,36	0,00	0,00	ENSG00000122008
0,05	0,24	0,35	ENSG00000189045
0,94	0,50	0,60	ENSG00000152359
0,13	0,46	0,56	ENSG00000122012
3,07	0,00	0,00	ENSG00000145703

0,18	0,00	0,00	ENSG00000164220
4,32	0,00	0,00	ENSG00000181104
0,49	0,00	0,00	ENSG00000164251
1,14	0,97	0,98	ENSG00000171643
1,14	0,97	0,98	ENSG00000145708
1,30	0,00	0,01	ENSG00000164252
0,00	0,01	0,02	ENSG00000285000
0,42	0,00	0,00	ENSG00000132846
0,37	0,00	0,00	ENSG00000284762
0,55	0,00	0,00	ENSG00000113231
1,53	0,00	0,00	ENSG00000164253
3,56	0,23	0,34	ENSG00000171540
1,07	0,42	0,54	ENSG00000171530
1,48	0,00	0,00	ENSG00000132842
0,96	0,66	0,76	ENSG00000085365
1,05	0,58	0,68	ENSG00000145685
1,23	0,02	0,04	ENSG00000113273
0,08	0,00	0,00	ENSG00000132837
0,53	0,58	0,68	ENSG00000132840
0,09	0,01	0,03	ENSG00000145692
1,03	0,70	0,79	ENSG00000152409
0,81	0,02	0,05	ENSG00000152413
1,38	0,00	0,00	ENSG00000164329
1,01	0,94	0,98	ENSG00000164309
1,26	0,01	0,01	ENSG00000177034
1,03	0,74	0,82	ENSG00000113296
2,56	0,00	0,00	ENSG00000164300
#N/D	#N/D	#N/D	ENSG00000164299
1,14	0,14	0,22	ENSG00000039319
1,27	0,52	0,63	ENSG00000152380
0,05	0,24	0,35	ENSG00000189127
1,50	0,00	0,00	ENSG00000228716
0,86	0,08	0,15	ENSG00000113318
1,20	0,03	0,06	ENSG00000113319
1,36	0,47	0,57	ENSG00000131730
0,94	0,52	0,62	ENSG00000131732
1,14	0,97	0,98	ENSG00000172497
0,37	0,00	0,00	ENSG00000145687
0,68	0,00	0,00	ENSG00000152348
1,17	0,04	0,08	ENSG00000186468
2,08	0,00	0,00	ENSG00000174695
0,81	0,06	0,10	ENSG00000152422
0,57	0,00	0,00	ENSG00000038427
1,14	0,97	#N/D	ENSG00000145681
4,70	0,00	0,00	ENSG00000164176
#N/D	#N/D	#N/D	ENSG00000280009
1,04	0,65	0,74	ENSG00000127184
1,48	0,00	0,00	ENSG00000145715

1,13	0,16	0,25	ENSG00000134480
1,27	0,01	0,02	ENSG00000164180
2,20	0,00	0,00	ENSG00000081189
0,58	0,00	0,00	ENSG00000153140
0,67	0,00	0,00	ENSG00000176055
0,69	0,00	0,00	ENSG00000113356
2,32	0,00	0,00	ENSG00000176018
1,52	0,39	0,50	ENSG00000164199
1,49	0,00	0,00	ENSG00000113369
11,26	0,00	0,00	ENSG00000175745
30,14	0,22	0,32	ENSG00000278905
1,28	0,01	0,01	ENSG00000113391
0,79	0,25	0,36	ENSG00000248483
0,60	0,01	0,03	ENSG00000185261
1,12	0,26	0,37	ENSG00000133302
0,03	0,00	0,00	ENSG00000175471
1,14	0,97	#N/D	ENSG00000153347
1,10	0,22	0,33	ENSG00000198677
1,68	0,00	0,00	ENSG00000164291
0,99	1,00	1,00	ENSG00000178015
0,59	0,00	0,01	ENSG00000175449
0,62	0,33	0,44	ENSG00000145757
2,48	0,00	0,00	ENSG00000164292
3,63	0,00	0,00	ENSG00000173221
1,90	0,00	0,00	ENSG00000118985
0,32	0,00	0,00	ENSG00000175426
0,94	0,46	0,57	ENSG00000153113
0,66	0,00	0,00	ENSG00000164307
1,16	0,20	0,30	ENSG00000164308
1,03	0,73	0,82	ENSG00000113441
0,13	0,46	0,56	ENSG00000145721
1,15	0,14	0,22	ENSG00000058729
1,51	0,00	0,00	ENSG00000174136
0,73	0,00	0,00	ENSG00000153922
1,35	0,00	0,01	ENSG00000174132
4,08	0,00	0,00	ENSG00000113532
0,09	0,00	0,00	ENSG00000173930
#N/D	#N/D	#N/D	ENSG00000205359
1,53	0,00	0,00	ENSG00000145730
0,87	0,27	0,38	ENSG00000145723
1,23	0,02	0,03	ENSG00000145725
0,99	0,94	0,98	ENSG00000181751
1,38	0,00	0,00	ENSG00000112874
#N/D	#N/D	#N/D	ENSG00000278958
4,53	0,00	0,00	ENSG00000184349
1,40	0,00	0,00	ENSG00000145743
1,19	0,04	0,07	ENSG00000151422
2,72	0,00	0,00	ENSG00000198961

0,83	0,02	0,05	ENSG00000112893
1,44	0,68	0,77	ENSG00000186952
1,05	0,56	0,66	ENSG00000164209
0,44	0,00	0,00	ENSG00000145777
1,24	0,01	0,02	ENSG00000134987
0,65	0,00	0,00	ENSG00000152495
4,14	0,00	0,00	ENSG00000164211
2,35	0,00	0,00	ENSG00000134986
1,18	0,21	0,31	ENSG00000129595
1,41	0,00	0,00	ENSG00000134982
4,80	0,00	0,00	ENSG00000258864
1,22	0,02	0,05	ENSG00000153037
1,29	0,00	0,01	ENSG00000129625
1,66	0,00	0,00	ENSG00000172795
0,65	0,00	0,00	ENSG00000171444
1,60	0,51	0,62	ENSG00000212122
0,84	0,05	0,09	ENSG00000047188
0,21	0,00	0,00	ENSG00000080709
0,38	0,00	0,00	ENSG00000152503
1,10	0,32	0,43	ENSG00000164219
0,72	0,02	0,05	ENSG00000164221
1,43	0,00	0,00	ENSG00000145780
2,29	0,00	0,00	ENSG00000243414
2,17	0,00	0,00	ENSG00000251201
1,84	0,00	0,00	ENSG00000134970
1,14	0,97	0,98	ENSG00000129596
1,42	0,00	0,00	ENSG00000145782
1,06	0,50	0,60	ENSG00000177879
2,16	0,00	0,00	ENSG00000172901
0,02	0,11	0,18	ENSG00000268223
0,94	0,48	0,58	ENSG00000145781
2,23	0,00	0,00	ENSG00000092421
1,47	0,00	0,00	ENSG00000169570
0,85	0,05	0,09	ENSG00000172869
1,46	0,00	0,00	ENSG00000145779
1,22	0,01	0,03	ENSG00000133835
22,89	0,26	0,37	ENSG00000164334
7,81	0,00	0,00	ENSG00000184838
#N/D	#N/D	#N/D	ENSG00000279860
#N/D	#N/D	#N/D	ENSG00000181867
1,05	0,60	0,69	ENSG00000151304
2,50	0,00	0,00	ENSG00000113083
1,46	0,13	0,21	ENSG00000164185
1,14	0,97	0,98	ENSG00000250803
1,83	0,00	0,00	ENSG00000064692
1,01	0,90	0,96	ENSG00000205302
0,81	0,02	0,05	ENSG00000064652
1,58	0,00	0,00	ENSG00000168938

2,67	0,00	0,00	ENSG00000061455
0,66	0,00	0,00	ENSG00000168944
0,99	1,00	1,00	ENSG00000279772
1,03	0,77	0,85	ENSG00000151292
2,58	0,00	0,00	ENSG00000168916
1,08	0,41	0,52	ENSG00000155324
0,54	0,06	0,12	ENSG00000279118
1,36	0,00	0,00	ENSG00000164904
1,17	0,06	0,11	ENSG00000164902
#N/D	#N/D	#N/D	ENSG00000196900
1,28	0,00	0,01	ENSG00000113368
1,04	0,79	0,87	ENSG00000173926
1,09	0,47	0,57	ENSG00000164241
1,14	0,97	0,98	ENSG00000279446
0,07	0,31	0,43	ENSG00000145794
2,90	0,00	0,00	ENSG00000164244
#N/D	#N/D	#N/D	ENSG00000205279
0,13	0,46	0,56	ENSG00000230561
1,15	0,11	0,18	ENSG00000064651
1,36	0,00	0,00	ENSG00000138829
2.053,15	0,01	0,01	ENSG00000113396
1,14	0,17	0,26	ENSG00000066583
1.581,84	0,01	0,02	ENSG00000145808
6,13	0,07	0,13	ENSG00000186367
1,54	0,00	0,00	ENSG00000198108
1,14	0,11	0,18	ENSG00000169567
0,90	0,22	0,32	ENSG00000186687
0,93	0,41	0,53	ENSG00000158985
#N/D	#N/D	#N/D	ENSG00000279370
1,07	0,42	0,54	ENSG00000158987
0,00	0,02	0,04	ENSG00000273217
1,43	0,00	0,00	ENSG00000217128
0,51	0,42	0,54	ENSG00000279584
#N/D	#N/D	#N/D	ENSG00000239642
#N/D	#N/D	#N/D	ENSG00000281938
1,14	0,97	0,98	ENSG00000164398
#N/D	#N/D	#N/D	ENSG00000164399
0,05	0,00	0,00	ENSG00000164400
1,27	0,00	0,01	ENSG00000072682
0,95	0,53	0,64	ENSG00000131435
0,46	0,00	0,00	ENSG00000197208
1,02	0,85	0,92	ENSG00000197375
1,32	0,00	0,00	ENSG00000283782
1,80	0,00	0,00	ENSG00000125347
1,14	0,97	0,98	ENSG00000113525
0,57	0,00	0,00	ENSG00000113522
1,14	0,97	0,98	ENSG00000169194
15,64	0,33	#N/D	ENSG00000113520

1,11	0,25	0,36	ENSG00000131437
1,01	0,97	0,98	ENSG00000205089
1,65	0,00	0,00	ENSG00000164402
0,42	0,03	0,06	ENSG00000198944
0,57	0,00	0,00	ENSG00000164403
1,35	0,06	0,11	ENSG00000164404
1,30	0,00	0,00	ENSG00000164405
1,29	0,42	0,54	ENSG00000164406
1,21	0,02	0,03	ENSG00000072364
1,06	0,56	0,66	ENSG00000155329
1,02	0,77	0,85	ENSG00000170606
1,26	0,58	0,68	ENSG00000279691
0,91	0,73	0,81	ENSG00000053108
#N/D	#N/D	#N/D	ENSG00000280104
#N/D	#N/D	#N/D	ENSG00000279197
1,37	0,00	0,00	ENSG00000113583
1,34	0,00	0,00	ENSG00000213585
0,85	0,06	0,11	ENSG00000081059
1,23	0,01	0,02	ENSG00000113558
0,78	0,00	0,01	ENSG00000272772
1,00	0,96	0,98	ENSG00000113575
0,71	0,12	0,20	ENSG00000273345
0,93	0,70	0,79	ENSG00000006837
0,96	0,64	0,73	ENSG00000119048
1,45	0,37	0,48	ENSG00000279469
1,25	0,01	0,02	ENSG00000237190
1,29	0,74	0,82	ENSG00000280420
0,78	0,00	0,01	ENSG00000043143
2,18	0,00	0,00	ENSG00000152700
2,49	0,00	0,00	ENSG00000113615
1,48	0,00	0,00	ENSG00000164615
1,25	0,01	0,02	ENSG00000145833
1,43	0,00	0,00	ENSG00000181904
1,35	0,00	0,00	ENSG00000113621
0,69	0,00	0,00	ENSG00000132570
1,02	0,94	0,98	ENSG00000279799
0,43	0,01	0,02	ENSG00000152705
3,08	0,00	0,00	ENSG00000069011
1,25	0,01	0,01	ENSG00000113648
#N/D	#N/D	#N/D	ENSG00000251380
#N/D	#N/D	#N/D	ENSG00000255833
0,13	0,46	0,56	ENSG00000145832
#N/D	#N/D	#N/D	ENSG00000181965
15,64	0,33	0,44	ENSG00000145824
#N/D	#N/D	#N/D	ENSG00000145839
30,14	0,22	0,32	ENSG00000145826
2,77	0,00	0,00	ENSG00000120708
1,54	0,00	0,00	ENSG00000113658

0,63	0,13	0,22	ENSG00000271824
0,02	0,12	0,19	ENSG00000069018
1,05	0,56	0,67	ENSG00000152377
0,22	0,00	0,00	ENSG00000146021
0,96	0,57	0,67	ENSG00000177733
0,24	0,01	0,02	ENSG00000120729
0,71	0,18	0,27	ENSG00000078795
1,26	0,01	0,03	ENSG00000031003
1,85	0,60	0,69	ENSG00000061492
1,14	0,97	#N/D	ENSG00000112981
1,11	0,20	0,30	ENSG00000112983
1,89	0,00	0,00	ENSG00000112984
1,42	0,00	0,00	ENSG00000094880
0,98	0,97	0,99	ENSG00000146013
1,10	0,27	0,37	ENSG00000158402
1,28	0,00	0,01	ENSG00000120709
1,07	0,39	0,50	ENSG00000120733
0,23	0,00	0,00	ENSG00000132563
1,22	0,04	0,07	ENSG00000120738
1,25	0,01	0,01	ENSG00000120705
0,70	0,00	0,00	ENSG00000113013
1,09	0,27	0,38	ENSG00000044115
1,26	0,16	0,24	ENSG00000146006
1,57	0,00	0,00	ENSG00000120725
0,74	0,00	0,00	ENSG00000280987
0,88	0,10	0,17	ENSG00000015479
0,94	0,49	0,59	ENSG00000120727
1,04	0,91	0,97	ENSG00000170482
4,03	0,01	0,03	ENSG00000170476
1,84	0,00	0,00	ENSG00000228672
1,44	0,00	0,00	ENSG00000170469
9,91	0,00	0,00	ENSG00000170464
30,14	0,22	0,32	ENSG00000279686
6,02	0,00	0,00	ENSG00000249751
0,67	0,65	0,74	ENSG00000283288
1,60	0,00	0,00	ENSG00000184584
1,11	0,21	0,31	ENSG00000131508
3,51	0,00	0,00	ENSG00000171604
3,42	0,00	0,00	ENSG00000146005
1,14	0,20	0,30	ENSG00000158458
1,61	0,00	0,00	ENSG00000185129
2,55	0,00	0,00	ENSG00000182700
1,14	0,14	0,23	ENSG00000120306
1,48	0,00	0,00	ENSG00000113068
0,71	0,00	0,00	ENSG00000113070
0,50	0,32	0,43	ENSG00000113073
2,01	0,00	0,00	ENSG00000131503
0,63	0,00	0,00	ENSG00000254996

2,32	0,00	0,00	ENSG00000213523
0,34	0,00	0,00	ENSG00000243056
1,42	0,00	0,00	ENSG00000113108
1,57	0,00	0,00	ENSG00000293600
1,47	0,00	0,00	ENSG00000176087
3,05	0,00	0,00	ENSG00000170458
1,60	0,00	0,00	ENSG00000131495
0,76	0,00	0,01	ENSG00000113119
1,38	0,00	0,00	ENSG00000113141
2,23	0,00	0,00	ENSG00000120314
0,54	0,10	0,18	ENSG00000256453
1,40	0,00	0,00	ENSG00000170445
1,24	0,01	0,02	ENSG00000112855
1,69	0,00	0,00	ENSG00000146007
0,99	1,00	1,00	ENSG00000204970
0,51	0,42	0,54	ENSG00000204969
0,01	0,08	0,14	ENSG00000255408
1,85	0,60	0,69	ENSG00000204967
0,98	0,98	0,99	ENSG00000204965
0,99	1,00	#N/D	ENSG00000081842
0,05	0,24	0,35	ENSG00000204963
1,14	0,97	#N/D	ENSG00000204962
1,14	0,97	0,98	ENSG00000204961
30,14	0,22	0,32	ENSG00000250120
1,14	0,97	#N/D	ENSG00000249158
1,14	0,97	#N/D	ENSG00000251664
1,14	0,97	0,98	ENSG00000239389
1,14	0,97	0,98	ENSG00000248383
4,76	0,00	0,00	ENSG00000243232
0,05	0,24	0,35	ENSG00000171815
13,08	0,00	0,00	ENSG00000112852
4,42	0,15	0,24	ENSG00000113205
1,14	0,97	0,98	ENSG00000081818
0,13	0,46	0,56	ENSG00000113209
0,53	0,58	0,68	ENSG00000113211
0,05	0,24	0,35	ENSG00000113212
3,15	0,08	0,14	ENSG00000120322
0,97	0,92	0,98	ENSG00000272674
0,65	0,30	0,41	ENSG00000177839
0,33	0,06	0,10	ENSG00000120324
0,19	0,01	0,02	ENSG00000197479
1,85	0,60	0,69	ENSG00000120328
0,06	0,00	0,00	ENSG00000187372
0,41	0,00	0,00	ENSG00000120327
1,14	0,97	#N/D	ENSG00000279983
0,04	0,00	0,00	ENSG00000113248
1,37	0,00	0,00	ENSG00000178913
1,14	0,97	0,98	ENSG00000120329

0,72	0,28	0,40	ENSG00000204956
0,33	0,02	0,04	ENSG00000081853
0,02	0,15	0,23	ENSG00000254245
3,22	0,00	0,00	ENSG00000254221
0,79	0,22	0,32	ENSG00000262576
0,89	0,25	0,36	ENSG00000253910
1,91	0,00	0,00	ENSG00000253485
1,55	0,01	0,03	ENSG00000262209
1,49	0,03	0,06	ENSG00000253731
1,94	0,00	0,01	ENSG00000253537
2,79	0,00	0,00	ENSG00000253953
1,98	0,01	0,02	ENSG00000253767
1,21	0,62	0,72	ENSG00000276547
0,10	0,00	0,00	ENSG00000261934
0,84	0,58	0,68	ENSG00000253305
0,36	0,00	0,00	ENSG00000253846
0,28	0,00	0,00	ENSG00000254122
0,93	0,75	0,83	ENSG00000253873
2,11	0,17	0,26	ENSG00000253159
2,10	0,00	0,00	ENSG00000240184
3,48	0,00	0,00	ENSG00000242419
2,91	0,00	0,00	ENSG00000240764
1,58	0,00	0,00	ENSG00000131504
1,51	0,00	0,00	ENSG00000171720
1,09	0,34	0,45	ENSG00000164620
1,89	0,00	0,00	ENSG00000197948
0,79	0,00	0,01	ENSG00000120318
0,06	0,00	0,00	ENSG00000156453
1,65	0,00	0,00	ENSG00000081791
2,95	0,00	0,00	ENSG00000113555
0,91	0,25	0,35	ENSG00000013561
1,51	0,00	0,00	ENSG00000113552
1,06	0,47	0,57	ENSG00000131507
1,54	0,02	0,03	ENSG00000280047
2,20	0,00	0,00	ENSG00000187678
1,22	0,30	0,41	ENSG00000113578
0,55	0,00	0,00	ENSG00000145819
1,92	0,28	0,39	ENSG00000285366
2,26	0,00	0,00	ENSG00000113580
1,14	0,97	0,98	ENSG00000279679
0,13	0,46	0,56	ENSG00000279130
#N/D	#N/D	#N/D	ENSG00000158497
2,32	0,00	0,00	ENSG00000145817
1,91	0,00	0,01	ENSG00000183775
0,91	0,35	0,47	ENSG00000186314
0,13	0,46	0,56	ENSG00000204928
0,25	0,00	0,00	ENSG00000156463
0,32	0,00	0,00	ENSG00000173261

0,94	0,42	0,53	ENSG00000133706
1,31	0,00	0,00	ENSG00000275740
1,11	0,22	0,32	ENSG00000091009
1,33	0,54	0,64	ENSG00000091010
1,19	0,03	0,06	ENSG00000113649
0,45	0,14	0,22	ENSG00000173250
15,64	0,33	0,44	ENSG00000156475
2,09	0,00	0,00	ENSG00000169302
2,19	0,00	0,00	ENSG00000113657
0,13	0,46	0,56	ENSG00000176049
0,04	0,20	0,30	ENSG00000164266
1,14	0,97	#N/D	ENSG00000164265
0,68	0,03	0,06	ENSG00000178776
1,14	0,97	0,98	ENSG00000133710
#N/D	#N/D	#N/D	ENSG00000196800
37,39	0,19	0,29	ENSG00000178172
1,14	0,97	#N/D	ENSG00000248109
3,26	0,13	0,21	ENSG00000214510
#N/D	#N/D	#N/D	ENSG00000145879
#N/D	#N/D	#N/D	ENSG00000204909
1,25	0,01	0,02	ENSG00000145868
59,15	0,14	0,22	ENSG00000164270
1,26	0,01	0,02	ENSG00000169252
0,83	0,05	0,09	ENSG00000169247
0,85	0,04	0,07	ENSG00000173210
0,81	0,01	0,03	ENSG00000157510
1,30	0,00	0,00	ENSG00000164284
1,38	0,00	0,00	ENSG00000145882
8,39	0,46	0,56	ENSG00000127743
1,25	0,00	0,01	ENSG00000113712
2,32	0,00	0,00	ENSG00000183111
0,58	0,00	0,00	ENSG00000155846
0,79	0,36	0,47	ENSG00000132915
1,60	0,00	0,00	ENSG00000155850
0,79	0,04	0,07	ENSG00000164296
1,53	0,00	0,00	ENSG00000113716
1,34	0,10	0,17	ENSG00000182578
1,12	0,19	0,29	ENSG00000113721
1,14	0,97	#N/D	ENSG00000113722
2,35	0,30	0,41	ENSG00000011083
4,57	0,00	0,01	ENSG00000070808
14,66	0,00	0,00	ENSG00000183876
0,73	0,00	0,00	ENSG00000070814
0,68	0,19	0,29	ENSG00000019582
1,11	0,17	0,26	ENSG00000164587
1,34	0,00	0,00	ENSG00000070614
0,76	0,00	0,00	ENSG00000171992
0,31	0,00	0,00	ENSG00000164591

1,26	0,00	0,01	ENSG00000086589
1,22	0,01	0,03	ENSG00000132912
2,23	0,00	0,00	ENSG00000256235
1,54	0,44	0,56	ENSG00000237693
2,71	0,36	0,48	ENSG00000145908
0,20	0,00	0,00	ENSG00000211445
0,80	0,01	0,01	ENSG00000145901
2,78	0,00	0,00	ENSG00000197043
0,41	0,00	0,00	ENSG00000198624
1,93	0,00	0,00	ENSG00000196743
8,39	0,46	0,56	ENSG00000186334
4,42	0,15	0,24	ENSG00000186335
1,25	0,01	0,03	ENSG00000123643
2,04	0,01	0,03	ENSG00000086570
3,05	0,00	0,00	ENSG00000113140
1,14	0,12	0,20	ENSG00000177556
1,12	0,14	0,22	ENSG00000145907
0,62	0,15	0,24	ENSG00000145888
15,64	0,33	0,44	ENSG00000132911
1,14	0,97	0,98	ENSG00000155511
1,14	0,14	0,22	ENSG00000055147
1,49	0,00	0,00	ENSG00000037749
0,50	0,00	0,00	ENSG00000164574
1,06	0,52	0,62	ENSG00000164576
7,65	0,00	0,00	ENSG00000113196
1,24	0,01	0,01	ENSG00000155506
1,55	0,00	0,00	ENSG00000170271
1,68	0,00	0,00	ENSG00000155508
1,11	0,19	0,28	ENSG00000082516
1,36	0,00	0,00	ENSG00000082515
1,13	0,82	0,89	ENSG00000226650
225,92	0,05	0,09	ENSG00000170624
#N/D	#N/D	#N/D	ENSG00000231989
#N/D	#N/D	#N/D	ENSG00000145850
1,14	0,97	#N/D	ENSG00000113249
2,15	0,20	0,30	ENSG00000135077
1,36	0,00	0,01	ENSG00000155868
1,14	0,97	0,98	ENSG00000113263
#N/D	#N/D	#N/D	ENSG00000170613
0,16	0,00	0,00	ENSG00000055163
0,93	0,74	0,83	ENSG00000285868
0,07	0,31	#N/D	ENSG00000172568
1,87	0,00	0,00	ENSG00000135074
1,96	0,00	0,00	ENSG00000172548
0,72	0,19	0,29	ENSG00000039600
1,14	0,97	#N/D	ENSG00000187658
0,84	0,06	0,11	ENSG00000113272
1,95	0,00	0,00	ENSG00000155858

1,76	0,00	0,00	ENSG00000113282
0,81	0,25	0,36	ENSG00000164330
0,05	0,24	0,35	ENSG00000279204
1,49	0,00	0,00	ENSG00000145860
0,70	0,00	0,00	ENSG00000164332
1,14	0,97	0,98	ENSG00000113302
0,56	0,00	0,00	ENSG00000170214
1,05	0,52	0,62	ENSG00000113312
1,40	0,00	0,00	ENSG00000170234
0,53	0,00	0,00	ENSG00000170231
0,84	0,09	0,16	ENSG00000135083
0,37	0,00	0,00	ENSG00000145861
1,60	0,00	0,00	ENSG00000221886
1,31	0,00	0,00	ENSG00000164609
1,66	0,00	0,00	ENSG00000164611
0,05	0,00	0,01	ENSG00000118322
#N/D	#N/D	#N/D	ENSG00000145864
#N/D	#N/D	#N/D	ENSG00000145863
1,14	0,97	#N/D	ENSG00000022355
#N/D	#N/D	#N/D	ENSG00000113327
1,55	0,00	0,00	ENSG00000113328
0,98	0,85	0,92	ENSG00000170584
0,45	0,00	0,00	ENSG00000072571
1,10	0,26	0,37	ENSG00000038274
0,21	0,00	0,00	ENSG00000145934
0,31	0,01	0,03	ENSG00000279739
0,32	0,00	0,00	ENSG00000113645
1,70	0,00	0,00	ENSG00000113643
0,56	0,07	0,12	ENSG00000188573
1,62	0,00	0,00	ENSG00000120137
0,39	0,00	0,00	ENSG00000184347
0,13	0,46	0,56	ENSG00000279752
0,76	0,00	0,00	ENSG00000040275
2,26	0,00	0,00	ENSG00000134516
0,72	0,00	0,00	ENSG00000204767
1,14	0,97	#N/D	ENSG00000168269
#N/D	#N/D	#N/D	ENSG00000234511
1,14	0,97	0,98	ENSG00000043462
1,14	0,97	0,98	ENSG00000182132
0,67	0,65	0,74	ENSG00000145936
1,14	0,97	0,98	ENSG00000094755
0,94	0,53	0,63	ENSG00000204764
1,14	0,97	0,98	ENSG00000164438
1,14	0,10	0,17	ENSG00000181163
0,55	0,09	0,16	ENSG00000156427
#N/D	#N/D	#N/D	ENSG00000185662
1,32	0,00	0,00	ENSG00000072803
1,11	0,19	0,29	ENSG00000072786

0,13	0,46	0,56	ENSG00000214360
1,06	0,49	0,59	ENSG00000168246
1,75	0,00	0,00	ENSG00000174705
#N/D	#N/D	#N/D	ENSG00000280318
2,54	0,00	0,00	ENSG00000214357
1,04	0,67	0,76	ENSG00000120129
1,36	0,00	0,00	ENSG00000113719
0,96	0,67	0,76	ENSG00000037241
1,02	0,78	0,86	ENSG00000113732
1,68	0,00	0,00	ENSG00000164463
1,64	0,00	0,00	ENSG00000113734
1,06	0,59	0,69	ENSG00000183072
1,58	0,00	0,00	ENSG00000113739
1,32	0,00	0,00	ENSG00000145919
1,99	0,00	0,00	ENSG00000113742
1,14	0,97	#N/D	ENSG00000185056
22,89	0,26	0,37	ENSG00000170091
1,64	0,00	0,00	ENSG00000120149
1,14	0,97	#N/D	ENSG00000184845
1,94	0,00	0,00	ENSG00000164466
0,26	0,08	0,14	ENSG00000113749
1,24	0,50	0,61	ENSG00000145920
6,13	0,07	0,13	ENSG00000280068
1,37	0,00	0,00	ENSG00000051596
2,71	0,36	0,48	ENSG00000289731
1,12	0,16	0,25	ENSG00000170085
1,53	0,00	0,00	ENSG00000122203
0,78	0,17	0,26	ENSG00000175414
1,05	0,59	0,69	ENSG00000048162
1,50	0,00	0,00	ENSG00000146066
1,20	0,03	0,06	ENSG00000175416
#N/D	#N/D	#N/D	ENSG00000280139
1,45	0,00	0,00	ENSG00000113194
1,08	0,37	0,48	ENSG00000146083
0,92	0,64	0,74	ENSG00000074276
1,08	0,34	0,45	ENSG00000169258
0,99	0,93	0,98	ENSG00000074317
1,44	0,68	0,77	ENSG00000175766
1,79	0,00	0,00	ENSG00000048140
17,06	0,00	0,00	ENSG00000113763
0,74	0,69	0,78	ENSG00000160883
1,55	0,00	0,00	ENSG00000087206
1,13	0,17	0,26	ENSG00000113761
1,95	0,00	0,00	ENSG00000160867
1,03	0,68	0,77	ENSG00000165671
1,47	0,00	0,00	ENSG00000169228
2,29	0,00	0,00	ENSG00000213347
1,52	0,00	0,00	ENSG00000169230

1,64	0,00	0,00	ENSG00000169223
2,33	0,00	0,00	ENSG00000169220
1,89	0,45	0,56	ENSG00000131183
#N/D	#N/D	#N/D	ENSG00000196570
1,58	0,00	0,00	ENSG00000131187
1,26	0,00	0,01	ENSG00000198055
1,35	0,00	0,01	ENSG00000131188
1,32	0,00	0,00	ENSG00000113758
1,46	0,00	0,00	ENSG00000279821
1,21	0,02	0,04	ENSG00000196923
0,33	0,00	0,00	ENSG00000146094
2,08	0,00	0,00	ENSG00000183258
1,69	0,00	0,00	ENSG00000146067
2,32	0,00	0,00	ENSG00000184840
1,81	0,00	0,00	ENSG00000027847
3,06	0,03	0,06	ENSG00000170074
0,60	0,47	0,58	ENSG00000175325
2,26	0,02	0,04	ENSG00000204677
0,52	0,00	0,00	ENSG00000145911
1,04	0,68	0,77	ENSG00000145916
1,70	0,00	0,00	ENSG00000145912
1,14	0,97	#N/D	ENSG00000244234
0,96	0,58	0,68	ENSG00000197451
1,43	0,00	0,00	ENSG00000175309
24,03	0,00	0,00	ENSG00000050767
1,37	0,00	0,01	ENSG00000113240
8,39	0,46	0,56	ENSG00000285891
1,14	0,97	0,98	ENSG00000285978
0,51	0,00	0,00	ENSG00000169131
0,83	0,10	0,17	ENSG00000178338
0,40	0,02	0,05	ENSG00000198939
0,99	1,00	1,00	ENSG00000178187
0,03	0,17	0,26	ENSG00000113262
0,87	0,36	0,47	ENSG00000234284
1,60	0,00	0,00	ENSG00000177932
1,29	0,00	0,01	ENSG00000087116
#N/D	#N/D	#N/D	ENSG00000251545
1,19	0,04	0,08	ENSG00000176783
0,93	0,33	0,44	ENSG00000169045
1,14	0,97	#N/D	ENSG00000204661
0,01	0,09	0,16	ENSG00000204659
1,37	0,00	0,00	ENSG00000127022
1,26	0,00	0,01	ENSG00000161021
1,17	0,80	0,87	ENSG00000213316
1,15	0,08	0,14	ENSG00000161013
1,39	0,00	0,00	ENSG00000161011
1,63	0,00	0,00	ENSG00000161010
1,40	0,00	0,00	ENSG00000197226

0,84	0,04	0,07	ENSG00000113269
8,39	0,46	0,56	ENSG00000278965
0,91	0,80	0,88	ENSG00000146090
1,04	0,61	0,70	ENSG00000050748
0,48	0,00	0,00	ENSG00000131459
1,58	0,00	0,00	ENSG00000113300
#N/D	#N/D	#N/D	ENSG00000161055
1,15	0,35	0,46	ENSG00000037280
#N/D	#N/D	#N/D	ENSG00000174339
2,12	0,00	0,00	ENSG00000131446
0,51	0,00	0,00	ENSG00000280161
1,13	0,16	0,26	ENSG00000196670
#N/D	#N/D	#N/D	ENSG00000113303
#N/D	#N/D	#N/D	ENSG00000168903
1,51	0,12	0,20	ENSG00000165810
1,14	0,97	#N/D	ENSG00000185372
0,04	0,20	0,30	ENSG00000182613
0,80	0,02	0,04	ENSG00000146054
1,46	0,00	0,00	ENSG00000146063
1,27	0,00	0,01	ENSG00000204628
1,79	0,00	0,00	ENSG00000183718
30,14	0,22	0,32	ENSG00000230178
0,76	0,00	0,01	ENSG00000112679
2,15	0,06	0,11	ENSG00000137265
1,06	0,48	0,58	ENSG00000112685
0,74	0,34	0,45	ENSG00000188996
0,23	0,00	0,00	ENSG00000164379
0,57	0,00	0,00	ENSG00000137273
0,42	0,00	0,00	ENSG00000054598
0,87	0,16	0,25	ENSG00000112699
51,90	0,15	0,24	ENSG00000280423
0,38	0,03	0,06	ENSG00000145949
0,65	0,00	0,00	ENSG00000124535
0,45	0,00	0,00	ENSG00000021355
0,04	0,20	0,30	ENSG00000170542
0,69	0,00	0,00	ENSG00000124570
0,75	0,00	0,01	ENSG00000124588
0,71	0,00	0,00	ENSG00000137275
0,74	0,02	0,04	ENSG00000137274
0,82	0,02	0,05	ENSG00000137267
0,05	0,00	0,00	ENSG00000137285
1,34	0,00	0,00	ENSG00000180822
0,38	0,00	0,00	ENSG00000137266
0,88	0,13	0,21	ENSG00000168994
0,98	0,99	0,99	ENSG00000145945
1,25	0,42	0,54	ENSG00000279926
0,85	0,06	0,11	ENSG00000112739
0,05	0,24	0,35	ENSG00000145975

0,78	0,00	0,01	ENSG00000198721
0,68	0,00	0,00	ENSG00000153046
0,68	0,01	0,01	ENSG00000124787
1,82	0,00	0,01	ENSG00000219607
1,28	0,00	0,01	ENSG00000214113
2,03	0,00	0,00	ENSG00000145982
6,52	0,00	0,00	ENSG00000124785
1,14	0,97	0,98	ENSG00000124491
1,14	0,97	#N/D	ENSG00000112799
0,23	0,00	0,01	ENSG00000279368
2,83	0,00	0,00	ENSG00000124782
0,91	0,25	0,36	ENSG00000124783
13,65	0,00	0,00	ENSG00000164304
0,92	0,33	0,44	ENSG00000124784
43,27	0,00	0,00	ENSG00000096696
0,89	0,20	0,30	ENSG00000168566
0,69	0,00	0,00	ENSG00000153162
1,38	0,00	0,00	ENSG00000239264
0,54	0,00	0,00	ENSG00000259040
1,13	0,17	0,26	ENSG00000188428
88,15	0,10	0,17	ENSG00000265818
1,07	0,47	0,57	ENSG00000124802
1,38	0,00	0,00	ENSG00000124786
2,50	0,00	0,00	ENSG00000137203
1,00	0,96	0,98	ENSG00000111846
0,19	0,00	0,00	ENSG00000137434
0,79	0,01	0,02	ENSG00000111845
0,87	0,09	0,15	ENSG00000111843
1,29	0,00	0,01	ENSG00000137210
1,93	0,08	0,14	ENSG00000272162
1,32	0,11	0,18	ENSG00000111837
0,97	0,95	0,98	ENSG00000124827
0,38	0,00	0,00	ENSG00000153157
0,77	0,00	0,00	ENSG00000197977
0,58	0,00	0,00	ENSG00000224531
0,85	0,27	0,38	ENSG00000244476
3,07	0,00	0,00	ENSG00000111859
0,54	0,00	0,00	ENSG00000205269
0,49	0,00	0,00	ENSG00000111863
0,69	0,00	0,00	ENSG00000095951
0,25	0,00	0,00	ENSG00000078401
0,92	0,53	0,64	ENSG00000112137
0,93	0,37	0,48	ENSG00000145979
0,28	0,00	0,00	ENSG00000145990
0,54	0,00	0,00	ENSG00000124523
0,64	0,00	0,00	ENSG00000225921
0,85	0,05	0,09	ENSG00000010017
0,87	0,10	0,17	ENSG00000050393

0,83	0,04	0,07	ENSG00000180537
0,38	0,00	0,00	ENSG00000112149
0,95	0,52	0,63	ENSG00000008083
0,68	0,00	0,00	ENSG00000047579
1,35	0,00	0,01	ENSG00000007944
0,77	0,02	0,04	ENSG00000137198
1,46	0,00	0,00	ENSG00000124788
#N/D	#N/D	#N/D	ENSG00000288708
0,13	0,46	#N/D	ENSG00000230873
1,11	0,32	0,43	ENSG00000112183
0,58	0,00	0,00	ENSG00000112186
1,58	0,00	0,00	ENSG00000137414
0,95	0,53	0,64	ENSG00000124789
0,86	0,06	0,11	ENSG00000137177
4,20	0,00	0,01	ENSG00000187566
0,90	0,29	0,41	ENSG00000137364
0,67	0,00	0,00	ENSG00000165097
0,71	0,00	0,00	ENSG00000124795
0,72	0,00	0,00	ENSG00000137393
5,77	0,00	0,00	ENSG00000172201
2,64	0,00	0,00	ENSG00000172197
0,92	0,30	0,42	ENSG00000112242
0,81	0,02	0,04	ENSG00000145996
1,16	0,08	0,14	ENSG00000124766
#N/D	#N/D	#N/D	ENSG00000280443
#N/D	#N/D	#N/D	ENSG00000172179
#N/D	#N/D	#N/D	ENSG00000112273
0,13	0,46	#N/D	ENSG00000152954
0,13	0,46	0,56	ENSG00000146038
0,65	0,00	0,00	ENSG00000124532
1,03	0,94	0,98	ENSG00000112293
15,64	0,33	0,44	ENSG00000112294
0,09	0,00	0,00	ENSG00000137261
0,64	0,00	0,00	ENSG00000111802
0,80	0,02	0,04	ENSG00000112304
1,10	0,23	0,33	ENSG00000112308
0,76	0,00	0,01	ENSG00000112312
1,14	0,97	0,98	ENSG00000260286
1,14	0,97	#N/D	ENSG00000282804
0,76	0,33	0,44	ENSG00000111913
0,60	0,00	0,00	ENSG00000079691
#N/D	#N/D	#N/D	ENSG00000079689
#N/D	#N/D	#N/D	ENSG00000290217
1,14	0,97	#N/D	ENSG00000164508
1,14	0,97	0,98	ENSG00000146047
#N/D	#N/D	#N/D	ENSG00000146039
#N/D	#N/D	#N/D	ENSG00000124568
#N/D	#N/D	#N/D	ENSG00000124564

1,14	0,97	#N/D	ENSG00000112337
1,85	0,00	0,00	ENSG00000112343
1,45	0,48	0,58	ENSG00000124610
0,40	0,00	0,00	ENSG00000275714
0,64	0,00	0,00	ENSG00000278637
0,44	0,00	0,00	ENSG00000278705
0,45	0,00	0,00	ENSG00000286522
0,23	0,00	0,00	ENSG00000278463
0,25	0,00	0,00	ENSG00000276410
0,38	0,00	0,00	ENSG00000287080
0,34	0,00	0,00	ENSG00000187837
0,62	0,00	0,00	ENSG00000180596
0,84	0,07	0,13	ENSG00000010704
0,41	0,00	0,00	ENSG00000197061
0,01	0,04	0,08	ENSG00000187475
0,66	0,00	0,00	ENSG00000291336
0,38	0,00	0,00	ENSG00000180573
0,39	0,00	0,00	ENSG00000168298
10.101,70	0,00	0,00	ENSG00000291338
0,56	0,00	0,00	ENSG00000158373
0,64	0,00	0,00	ENSG00000274290
0,48	0,00	0,00	ENSG00000277157
0,37	0,00	0,00	ENSG00000282988
1,18	0,14	0,22	ENSG00000197409
0,13	0,00	0,00	ENSG00000196866
0,36	0,00	0,00	ENSG00000277224
0,59	0,00	0,00	ENSG00000276966
0,33	0,00	0,00	ENSG00000273802
0,24	0,00	0,00	ENSG00000277075
0,44	0,00	0,00	ENSG00000274750
0,25	0,00	0,00	ENSG00000124575
#N/D	#N/D	#N/D	ENSG00000274618
#N/D	#N/D	#N/D	ENSG00000275663
0,65	0,00	0,00	ENSG00000277775
0,41	0,00	0,00	ENSG00000275713
0,43	0,00	0,00	ENSG00000273983
0,49	0,00	0,00	ENSG00000278588
0,41	0,00	0,00	ENSG00000158406
0,53	0,00	0,00	ENSG00000186470
1,21	0,09	0,15	ENSG00000124508
0,82	0,02	0,04	ENSG00000026950
0,80	0,02	0,04	ENSG00000111801
1,04	0,65	0,75	ENSG00000112763
0,13	0,46	#N/D	ENSG00000124557
0,76	0,00	0,00	ENSG00000182952
1,03	0,78	0,86	ENSG00000146109
1,36	0,00	0,00	ENSG00000181315
0,37	0,00	0,00	ENSG00000124635

0,46	0,00	0,00	ENSG00000196787
0,19	0,00	0,00	ENSG00000276180
0,55	0,00	0,00	ENSG00000197903
0,38	0,00	0,00	ENSG00000274997
0,76	0,03	0,07	ENSG00000112812
8,39	0,46	0,56	ENSG00000158553
0,64	0,00	0,00	ENSG00000124613
4,09	0,00	0,00	ENSG00000096654
1,38	0,00	0,00	ENSG00000185130
1,15	0,09	0,15	ENSG00000196747
1,01	0,92	0,98	ENSG00000278828
1,01	0,87	0,94	ENSG00000276368
0,34	0,00	0,00	ENSG00000273703
1,26	0,01	0,01	ENSG00000197238
1,14	0,10	0,16	ENSG00000273542
1,31	0,01	0,02	ENSG00000275221
3,59	0,00	0,00	ENSG00000233822
0,46	0,00	0,00	ENSG00000276903
1,02	0,80	0,88	ENSG00000184357
1,11	0,20	0,30	ENSG00000275379
0,88	0,41	0,52	ENSG00000275126
0,49	0,00	0,00	ENSG00000197153
1,71	0,00	0,00	ENSG00000278677
2,23	0,00	0,00	ENSG00000274641
30,14	0,22	0,32	ENSG00000168131
1,14	0,97	0,98	ENSG00000124657
1,14	0,97	#N/D	ENSG00000182477
1,23	0,48	0,59	ENSG00000197279
1,20	0,45	0,56	ENSG00000196812
1,13	0,14	0,23	ENSG00000198315
1,02	0,89	0,96	ENSG00000226314
0,63	0,07	0,13	ENSG00000280107
0,82	0,05	0,10	ENSG00000137185
1,04	0,76	0,84	ENSG00000187626
1,14	0,97	#N/D	ENSG00000189134
0,63	0,00	0,00	ENSG00000197062
0,28	0,04	0,08	ENSG00000276302
0,65	0,00	0,00	ENSG00000137338
4,88	0,00	0,00	ENSG00000235109
0,85	0,13	0,22	ENSG00000189298
0,60	0,00	0,00	ENSG00000158691
1,94	0,00	0,00	ENSG00000187987
#N/D	#N/D	#N/D	ENSG00000198704
1,14	0,97	#N/D	ENSG00000224586
0,30	0,00	0,00	ENSG00000232040
1,17	0,06	0,10	ENSG00000204713
0,36	0,00	0,00	ENSG00000197935
#N/D	#N/D	#N/D	ENSG00000204704

#N/D	#N/D	#N/D	ENSG00000204703
#N/D	#N/D	#N/D	ENSG00000204702
#N/D	#N/D	#N/D	ENSG00000204701
#N/D	#N/D	#N/D	ENSG00000204700
1,14	0,97	#N/D	ENSG00000204695
#N/D	#N/D	#N/D	ENSG00000243729
#N/D	#N/D	#N/D	ENSG00000112462
8,39	0,46	#N/D	ENSG00000280236
#N/D	#N/D	#N/D	ENSG00000251608
#N/D	#N/D	#N/D	ENSG00000204694
#N/D	#N/D	#N/D	ENSG00000206474
8,39	0,46	#N/D	ENSG00000204688
#N/D	#N/D	#N/D	ENSG00000204687
1,14	0,97	0,98	ENSG00000237988
1,14	0,97	#N/D	ENSG00000213886
0,68	0,00	0,00	ENSG00000204681
30,14	0,22	0,32	ENSG00000204657
30,14	0,22	0,32	ENSG00000204655
0,13	0,46	#N/D	ENSG00000204644
0,26	0,00	0,00	ENSG00000204642
0,13	0,46	0,56	ENSG00000204632
0,75	0,00	0,00	ENSG00000206503
1,01	0,93	0,98	ENSG00000066379
1,55	0,00	0,00	ENSG00000204619
4,18	0,00	0,00	ENSG00000204618
0,74	0,69	0,78	ENSG00000204616
1,14	0,97	#N/D	ENSG00000204614
0,13	0,46	#N/D	ENSG00000204613
#N/D	#N/D	#N/D	ENSG00000204610
0,75	0,00	0,00	ENSG00000234127
0,67	0,00	0,00	ENSG00000280128
0,99	0,94	0,98	ENSG00000204599
1,14	0,97	0,98	ENSG00000248167
0,98	0,80	0,87	ENSG00000241370
0,79	0,00	0,01	ENSG00000204592
0,97	0,73	0,82	ENSG00000204590
0,79	0,01	0,02	ENSG00000204576
0,92	0,32	0,43	ENSG00000204574
0,46	0,00	0,00	ENSG00000204569
1,00	0,99	0,99	ENSG00000204568
1,61	0,00	0,00	ENSG00000137343
0,90	0,23	0,34	ENSG00000204564
0,91	0,24	0,35	ENSG00000204560
2,42	0,00	0,00	ENSG00000146112
1,26	0,01	0,02	ENSG00000137404
0,80	0,00	0,01	ENSG00000137337
1,14	0,11	0,18	ENSG00000196230
0,96	0,65	0,74	ENSG00000137312

1,05	0,53	0,64	ENSG00000137331
1,49	0,00	0,00	ENSG00000204580
1,05	0,61	0,70	ENSG00000213780
0,91	0,24	0,35	ENSG00000137411
#N/D	#N/D	#N/D	ENSG00000196260
1,14	0,97	0,98	ENSG00000168631
8,39	0,46	#N/D	ENSG00000204544
0,13	0,46	0,56	ENSG00000261272
1,14	0,97	0,98	ENSG00000204542
2,72	0,04	0,07	ENSG00000204540
0,94	0,82	0,89	ENSG00000204539
2,84	0,11	0,19	ENSG00000204538
1,57	0,00	0,00	ENSG00000204536
1,90	0,00	0,00	ENSG00000137310
1,38	0,04	0,08	ENSG00000204531
0,57	0,00	0,00	ENSG00000204525
0,23	0,00	0,00	ENSG00000234745
1,52	0,00	0,00	ENSG00000204520
0,76	0,00	0,00	ENSG00000204516
1,23	0,56	0,66	ENSG00000204511
0,69	0,00	0,00	ENSG00000198563
0,51	0,00	0,00	ENSG00000254870
1,37	0,17	0,27	ENSG00000213760
1,22	0,03	0,05	ENSG00000204498
1,60	0,51	0,62	ENSG00000226979
0,36	0,35	0,46	ENSG00000232810
10,42	0,02	0,04	ENSG00000227507
1,73	0,32	0,44	ENSG00000204482
0,13	0,46	0,56	ENSG00000204475
#N/D	#N/D	#N/D	ENSG00000204472
0,83	0,02	0,05	ENSG00000204469
#N/D	#N/D	#N/D	ENSG00000291302
#N/D	#N/D	#N/D	ENSG00000289282
1,16	0,07	0,12	ENSG00000204463
2,25	0,00	0,00	ENSG00000204444
1,15	0,12	0,20	ENSG00000204439
0,81	0,02	0,05	ENSG00000204438
1,05	0,59	0,69	ENSG00000204435
1,89	0,00	0,00	ENSG00000263020
1,63	0,00	0,00	ENSG00000240053
0,22	0,00	0,01	ENSG00000204428
0,82	0,04	0,07	ENSG00000204427
#N/D	#N/D	#N/D	ENSG00000204424
1,14	0,97	#N/D	ENSG00000250641
1,14	0,97	0,98	ENSG00000244355
2,23	0,02	0,04	ENSG00000204420
1,14	0,97	0,98	ENSG00000204421
1,60	0,00	0,00	ENSG00000213722

1,20	0,02	0,05	ENSG00000213719
0,71	0,00	0,00	ENSG00000204410
1,25	0,03	0,05	ENSG00000255152
0,18	0,00	0,00	ENSG00000228727
0,56	0,01	0,01	ENSG00000204396
1,21	0,02	0,04	ENSG00000204394
1,12	0,19	0,29	ENSG00000204392
0,54	0,00	0,00	ENSG00000204390
1,07	0,40	0,52	ENSG00000204389
1,11	0,22	0,32	ENSG00000204388
1,50	0,00	0,00	ENSG00000204386
1,38	0,51	0,62	ENSG00000204385
0,80	0,01	0,01	ENSG00000204371
1,49	0,04	0,08	ENSG00000166278
0,99	0,91	0,97	ENSG00000204366
1,14	0,97	#N/D	ENSG00000244255
0,26	0,00	0,00	ENSG00000243649
1,53	0,00	0,00	ENSG00000204356
1,73	0,00	0,00	ENSG00000204351
1,12	0,21	0,31	ENSG00000204348
2,01	0,00	0,00	ENSG00000204344
1,14	0,97	0,98	ENSG00000244731
1,81	0,00	0,00	ENSG00000224389
0,82	0,29	0,40	ENSG00000231852
0,53	0,00	0,00	ENSG00000168477
1,20	0,03	0,05	ENSG00000213676
1,01	0,92	0,98	ENSG00000204315
0,68	0,01	0,03	ENSG00000204314
1,13	0,75	0,83	ENSG00000285085
0,61	0,00	0,00	ENSG00000221988
0,99	0,92	0,98	ENSG00000258388
1,43	0,12	0,20	ENSG00000241404
1,04	0,60	0,70	ENSG00000204310
1,50	0,00	0,00	ENSG00000204308
1,79	0,00	0,00	ENSG00000204305
0,90	0,19	0,28	ENSG00000204304
1,26	0,13	0,21	ENSG00000213654
0,89	0,65	0,74	ENSG00000204301
0,13	0,46	0,56	ENSG00000204296
1,14	0,97	#N/D	ENSG00000204290
1,14	0,97	0,98	ENSG00000204287
1,14	0,97	0,98	ENSG00000198502
1,14	0,97	0,98	ENSG00000196126
1,14	0,97	0,98	ENSG00000196735
1,14	0,97	0,98	ENSG00000179344
1,14	0,97	0,98	ENSG00000237541
1,14	0,97	0,98	ENSG00000232629
1,51	0,00	0,00	ENSG00000241106

0,67	0,00	0,00	ENSG00000250264
0,60	0,00	0,00	ENSG00000204267
0,46	0,00	0,00	ENSG00000204264
0,37	0,00	0,00	ENSG00000240065
0,66	0,00	0,00	ENSG00000168394
2,98	0,00	0,00	ENSG00000242574
0,17	0,02	0,04	ENSG00000248993
1,42	0,00	0,00	ENSG00000204257
2,39	0,00	0,00	ENSG00000204256
1,21	0,77	0,85	ENSG00000204252
2,14	0,00	0,00	ENSG00000231389
1,70	0,00	0,00	ENSG00000223865
2,81	0,00	0,00	ENSG00000204248
1,27	0,00	0,01	ENSG00000204231
1,70	0,00	0,00	ENSG00000112473
1,30	0,11	0,18	ENSG00000204228
1,13	0,13	0,21	ENSG00000204227
0,88	0,12	0,20	ENSG00000223501
0,98	0,81	0,88	ENSG00000231500
0,45	0,00	0,00	ENSG00000235863
1,19	0,03	0,06	ENSG00000227057
1,13	0,15	0,23	ENSG00000204220
1,29	0,00	0,00	ENSG00000237441
1,26	0,00	0,01	ENSG00000231925
0,92	0,34	0,45	ENSG00000236104
0,81	0,01	0,03	ENSG00000204209
1,14	0,97	#N/D	ENSG00000285064
37,39	0,19	0,29	ENSG00000286920
1,50	0,00	0,00	ENSG00000237649
2,02	0,00	0,00	ENSG00000112511
1,80	0,00	0,00	ENSG00000112514
1,03	0,71	0,80	ENSG00000197283
1,01	0,89	0,96	ENSG00000213588
1,32	0,00	0,00	ENSG00000030110
0,66	0,00	0,00	ENSG00000096433
1,06	0,53	0,63	ENSG00000137288
1,14	0,97	0,98	ENSG00000161896
1,04	0,60	0,70	ENSG00000161904
1,14	0,97	#N/D	ENSG00000096395
2,15	0,20	0,30	ENSG00000124493
0,95	0,54	0,65	ENSG00000137309
0,68	0,00	0,00	ENSG00000186577
0,89	0,14	0,23	ENSG00000272325
0,57	0,00	0,00	ENSG00000270800
1,05	0,56	0,66	ENSG00000124614
3,83	0,00	0,00	ENSG00000124507
0,79	0,54	0,64	ENSG00000124664
1,27	0,00	0,01	ENSG00000196821

3,31	0,00	0,00	ENSG00000124562
2,05	0,00	0,00	ENSG00000065060
4,06	0,00	0,00	ENSG00000064995
3,53	0,00	0,00	ENSG00000064999
0,88	0,60	0,69	ENSG00000124678
1,69	0,00	0,00	ENSG00000146197
1,12	0,18	0,28	ENSG00000065029
0,35	0,03	0,05	ENSG00000023892
1,04	0,63	0,73	ENSG00000112033
0,80	0,01	0,02	ENSG00000112039
0,96	0,63	0,73	ENSG00000198755
1,07	0,40	0,52	ENSG00000007866
0,28	0,00	0,00	ENSG00000112041
0,62	0,00	0,00	ENSG00000096060
0,82	0,47	0,57	ENSG00000157343
4,74	0,00	0,01	ENSG00000196748
1,14	0,97	#N/D	ENSG00000204140
#N/D	#N/D	#N/D	ENSG00000137392
0,89	0,25	0,36	ENSG00000197753
0,64	0,00	0,00	ENSG00000096063
0,40	0,12	0,19	ENSG00000112053
0,99	0,92	0,98	ENSG00000112062
0,83	0,03	0,06	ENSG00000156711
1,47	0,00	0,00	ENSG00000096070
1,29	0,56	0,67	ENSG00000180316
8,39	0,46	0,56	ENSG00000189325
0,65	0,26	0,37	ENSG00000010030
30,14	0,22	0,32	ENSG00000179165
0,90	0,20	0,30	ENSG00000112078
0,80	0,01	0,02	ENSG00000112079
0,67	0,00	0,00	ENSG00000112081
3,10	0,00	0,00	ENSG00000124762
15,64	0,33	0,44	ENSG00000255587
5,40	0,00	0,00	ENSG00000124772
0,91	0,29	0,40	ENSG00000137168
1,49	0,00	0,00	ENSG00000198663
0,98	0,98	0,99	ENSG00000164530
1,24	0,01	0,02	ENSG00000137409
0,13	0,46	0,56	ENSG00000146192
1,10	0,25	0,36	ENSG00000137193
0,62	0,17	0,27	ENSG00000172738
2,56	0,05	0,09	ENSG00000286105
1,04	0,65	0,75	ENSG00000065491
0,71	0,00	0,00	ENSG00000112130
1,08	0,37	0,49	ENSG00000137200
1,43	0,00	0,00	ENSG00000198937
0,76	0,59	0,69	ENSG00000279942
1,51	0,00	0,00	ENSG00000112139

0,88	0,12	0,20	ENSG00000156639
1,18	0,06	0,11	ENSG00000183826
3,05	0,00	0,00	ENSG00000124767
1.255,55	0,01	0,02	ENSG00000124721
0,51	0,42	0,54	ENSG00000112164
2,90	0,00	0,00	ENSG00000112167
0,46	0,00	0,00	ENSG00000164626
1,14	0,97	#N/D	ENSG00000124780
#N/D	#N/D	#N/D	ENSG00000095981
0,82	0,74	0,82	ENSG00000164627
28,31	0,00	0,00	ENSG00000146122
22,89	0,26	0,37	ENSG00000281969
1,19	0,17	0,26	ENSG00000124615
1,14	0,97	#N/D	ENSG00000156564
0,62	0,01	0,01	ENSG00000124602
0,57	0,00	0,00	ENSG00000124596
0,19	0,03	0,05	ENSG00000112212
0,54	0,01	0,02	ENSG00000124701
2,02	0,00	0,00	ENSG00000001167
0,53	0,58	0,68	ENSG00000161911
#N/D	#N/D	#N/D	ENSG00000095970
1,14	0,97	#N/D	ENSG00000112195
0,13	0,46	#N/D	ENSG00000188056
0,41	0,10	0,16	ENSG00000124731
#N/D	#N/D	#N/D	ENSG00000096264
#N/D	#N/D	#N/D	ENSG00000279284
1,14	0,97	#N/D	ENSG00000280371
0,75	0,00	0,00	ENSG00000137166
0,13	0,46	0,56	ENSG00000112559
1,31	0,01	0,03	ENSG00000112561
0,13	0,46	0,56	ENSG00000096088
0,57	0,00	0,00	ENSG00000137218
0,90	0,44	0,56	ENSG00000124593
0,44	0,00	0,01	ENSG00000278224
1,02	0,77	0,85	ENSG00000214736
1,57	0,00	0,00	ENSG00000164663
0,94	0,60	0,70	ENSG00000288721
1,26	0,01	0,02	ENSG00000124641
0,87	0,09	0,16	ENSG00000112578
1,69	0,00	0,00	ENSG00000112576
0,81	0,01	0,03	ENSG00000137413
0,37	0,00	0,00	ENSG00000188112
1,14	0,97	#N/D	ENSG00000287363
#N/D	#N/D	#N/D	ENSG00000290147
1,21	0,77	0,85	ENSG00000048545
0,86	0,28	0,39	ENSG00000112599
0,81	0,01	0,03	ENSG00000048544
0,63	0,00	0,00	ENSG00000124496

1,18	0,05	0,10	ENSG00000024048
0,28	0,01	0,02	ENSG00000112619
0,87	0,16	0,25	ENSG00000124659
0,68	0,00	0,00	ENSG00000112624
0,52	0,00	0,00	ENSG00000146223
1,10	0,58	0,68	ENSG00000221821
#N/D	#N/D	#N/D	ENSG00000171611
0,85	0,05	0,09	ENSG00000137161
1,32	0,44	0,56	ENSG00000124713
0,99	0,91	0,97	ENSG00000124587
1,27	0,00	0,01	ENSG00000112640
1,02	0,80	0,88	ENSG00000124733
1,15	0,09	0,16	ENSG00000124702
1,06	0,50	0,61	ENSG00000124541
1,96	0,00	0,00	ENSG00000044090
1,41	0,00	0,00	ENSG00000137171
486,95	0,02	0,05	ENSG00000288564
1,19	0,03	0,07	ENSG00000112651
0,82	0,01	0,03	ENSG00000112655
0,82	0,02	0,03	ENSG00000112658
0,92	0,32	0,43	ENSG00000112659
0,93	0,46	0,56	ENSG00000112667
8,39	0,46	0,56	ENSG00000146216
#N/D	#N/D	#N/D	ENSG00000137204
#N/D	#N/D	#N/D	ENSG00000146215
0,72	0,00	0,00	ENSG00000171467
0,84	0,05	0,09	ENSG00000124574
0,79	0,48	0,58	ENSG00000171462
0,81	0,02	0,04	ENSG00000137221
16,92	0,00	0,00	ENSG00000204052
0,95	0,58	0,68	ENSG00000171453
1,91	0,00	0,00	ENSG00000137207
0,66	0,00	0,00	ENSG00000124571
1,48	0,00	0,00	ENSG00000170734
0,69	0,00	0,00	ENSG00000172432
0,67	0,00	0,00	ENSG00000124688
0,79	0,13	0,22	ENSG00000172426
0,89	0,20	0,30	ENSG00000096080
1,02	0,76	0,85	ENSG00000112715
4,76	0,00	0,00	ENSG00000180992
6,78	0,00	0,00	ENSG00000137216
218,67	0,05	0,09	ENSG00000137225
6,13	0,07	0,13	ENSG00000262179
3,98	0,00	0,00	ENSG00000112759
4,79	0,00	0,00	ENSG00000096384
10,88	0,00	0,00	ENSG00000157593
3,94	0,00	0,00	ENSG00000146232
7,34	0,00	0,00	ENSG00000178233

4,75	0,00	0,00	ENSG00000272442
16,42	0,00	0,01	ENSG00000146221
4,58	0,00	0,00	ENSG00000124608
22,89	0,26	0,37	ENSG00000249481
4,39	0,00	0,00	ENSG00000096401
#N/D	#N/D	#N/D	ENSG00000279076
0,16	0,07	0,12	ENSG00000196284
0,17	0,00	0,00	ENSG00000124813
0,13	0,46	0,56	ENSG00000112782
1,12	0,72	0,81	ENSG00000001561
1,14	0,97	0,98	ENSG00000112796
0,22	0,00	0,00	ENSG00000172348
0,38	0,00	0,00	ENSG00000146233
0,93	0,52	0,62	ENSG00000153291
0,53	0,18	0,27	ENSG00000180113
0,07	0,31	0,43	ENSG00000146070
1,14	0,97	#N/D	ENSG00000230062
8,39	0,46	0,56	ENSG00000112818
8,39	0,46	0,56	ENSG00000069122
0,80	0,49	0,59	ENSG00000153292
1,23	0,01	0,02	ENSG00000146072
0,88	0,16	0,25	ENSG00000198087
0,01	0,07	0,12	ENSG00000153294
15,64	0,33	#N/D	ENSG00000124818
1,14	0,97	0,98	ENSG00000244694
1,73	0,00	0,00	ENSG00000146085
0,60	0,00	0,00	ENSG00000031691
1,19	0,07	0,12	ENSG00000203972
0,37	0,00	0,00	ENSG00000197261
1,14	0,97	#N/D	ENSG00000112077
#N/D	#N/D	#N/D	ENSG00000124490
1,14	0,97	#N/D	ENSG00000279127
1,14	0,97	#N/D	ENSG00000096006
0,13	0,46	#N/D	ENSG00000170950
#N/D	#N/D	#N/D	ENSG00000124812
#N/D	#N/D	#N/D	ENSG00000177684
#N/D	#N/D	#N/D	ENSG00000214642
#N/D	#N/D	#N/D	ENSG00000203970
#N/D	#N/D	#N/D	ENSG00000180872
#N/D	#N/D	#N/D	ENSG00000008197
#N/D	#N/D	#N/D	ENSG00000008196
2,80	0,20	0,30	ENSG00000170927
#N/D	#N/D	#N/D	ENSG00000112115
#N/D	#N/D	#N/D	ENSG00000112116
1,74	0,00	0,00	ENSG00000112118
1,23	0,01	0,03	ENSG00000170915
2,37	0,00	0,00	ENSG00000096093
2,45	0,00	0,00	ENSG00000065308

2,59	0,00	0,00	ENSG00000096092
0,03	0,17	0,26	ENSG00000244067
0,13	0,46	0,56	ENSG00000243955
#N/D	#N/D	#N/D	ENSG00000182793
#N/D	#N/D	#N/D	ENSG00000174156
1,42	0,00	0,01	ENSG00000170899
1,06	0,54	0,64	ENSG00000112144
#N/D	#N/D	#N/D	ENSG00000288614
0,91	0,32	0,43	ENSG00000112146
#N/D	#N/D	#N/D	ENSG00000288646
1,60	0,51	0,62	ENSG00000137270
1,51	0,00	0,00	ENSG00000012660
0,81	0,02	0,05	ENSG00000001084
0,98	0,99	0,99	ENSG00000124743
0,70	0,00	0,00	ENSG00000137269
0,36	0,35	0,46	ENSG00000146147
1,14	0,97	#N/D	ENSG00000137251
0,13	0,46	0,56	ENSG00000168143
1,14	0,97	#N/D	ENSG00000137252
1,14	0,97	#N/D	ENSG00000187871
#N/D	#N/D	#N/D	ENSG00000146151
1,14	0,97	#N/D	ENSG00000112175
2,99	0,00	0,01	ENSG00000124749
0,82	0,01	0,03	ENSG00000151914
1,17	0,20	0,30	ENSG00000151917
0,68	0,01	0,01	ENSG00000168116
1,02	0,80	0,87	ENSG00000112200
0,51	0,00	0,00	ENSG00000112208
0,56	0,00	0,00	ENSG00000112210
0,92	0,33	0,44	ENSG00000146143
#N/D	#N/D	#N/D	ENSG00000112232
0,67	0,65	0,74	ENSG00000198225
1,14	0,97	0,98	ENSG00000146166
1,07	0,42	0,53	ENSG00000112245
1,23	0,03	0,06	ENSG00000285976
0,82	0,02	0,03	ENSG00000118482
5,61	0,00	0,00	ENSG00000188107
1,52	0,21	0,31	ENSG00000214558
0,33	0,00	0,00	ENSG00000135298
#N/D	#N/D	#N/D	ENSG00000288712
1,45	0,00	0,00	ENSG00000168216
0,68	0,14	0,22	ENSG00000082293
#N/D	#N/D	#N/D	ENSG00000112280
0,67	0,00	0,00	ENSG00000082269
1,00	0,98	0,99	ENSG00000154079
2,11	0,00	0,00	ENSG00000112305
1,10	0,74	0,82	ENSG00000112309
0,70	0,00	0,00	ENSG00000119900

1,06	0,70	0,79	ENSG00000279289
35,72	0,00	0,00	ENSG00000079841
0,47	0,00	0,00	ENSG00000185760
0,03	0,17	0,26	ENSG00000243501
204,17	0,05	0,10	ENSG00000256980
1,05	0,65	0,74	ENSG00000135314
#N/D	#N/D	#N/D	ENSG00000203909
8,39	0,46	0,56	ENSG00000203908
0,55	0,16	0,25	ENSG00000203907
0,13	0,46	0,56	ENSG00000080007
0,82	0,02	0,05	ENSG00000164430
1,15	0,10	0,17	ENSG00000135297
1,29	0,00	0,00	ENSG00000156508
2,02	0,00	0,00	ENSG00000119899
1,43	0,00	0,00	ENSG00000156535
15,64	0,33	0,44	ENSG00000279790
7,72	0,00	0,00	ENSG00000111799
1,14	0,10	0,17	ENSG00000112695
1,01	0,94	0,98	ENSG00000112697
0,46	0,00	0,01	ENSG00000118407
0,74	0,00	0,00	ENSG00000112701
1,02	0,78	0,85	ENSG00000196586
0,13	0,46	0,56	ENSG00000112706
1,14	0,97	0,98	ENSG00000135312
0,75	0,35	0,46	ENSG00000269964
0,61	0,02	0,04	ENSG00000146243
0,80	0,01	0,02	ENSG00000146247
1,65	0,00	0,00	ENSG00000118418
1,44	0,00	0,00	ENSG00000135338
1,81	0,18	0,27	ENSG00000279659
0,51	0,00	0,00	ENSG00000198478
1,14	0,51	0,62	ENSG00000118402
0,91	0,24	0,34	ENSG00000112742
1,29	0,01	0,02	ENSG00000083123
0,22	0,14	0,22	ENSG00000279022
0,85	0,09	0,16	ENSG00000112773
1,02	0,80	0,88	ENSG00000005700
1,24	0,01	0,02	ENSG00000146242
0,54	0,00	0,00	ENSG00000118420
1,17	0,09	0,16	ENSG00000083097
1,53	0,00	0,00	ENSG00000013375
0,82	0,05	0,10	ENSG00000013392
0,78	0,00	0,01	ENSG00000065833
1,14	0,97	0,98	ENSG00000146250
6,71	0,00	0,00	ENSG00000065609
2,71	0,36	0,48	ENSG00000203877
0,82	0,03	0,06	ENSG00000065615
3,79	0,00	0,00	ENSG00000135324

1,06	0,74	0,82	ENSG00000135315
0,62	0,00	0,00	ENSG00000112837
1,39	0,00	0,00	ENSG00000135318
3,56	0,02	0,05	ENSG00000280232
0,78	0,00	0,01	ENSG00000135317
1,02	0,92	0,98	ENSG00000271793
0,78	0,00	0,01	ENSG00000135316
#N/D	#N/D	#N/D	ENSG00000168830
#N/D	#N/D	#N/D	ENSG00000135346
1,53	0,00	0,00	ENSG00000188994
0,40	0,12	0,19	ENSG00000164411
0,63	0,01	0,01	ENSG00000111850
0,62	0,20	0,30	ENSG00000203872
0,45	0,14	0,22	ENSG00000279616
3,26	0,13	0,21	ENSG00000272514
0,03	0,17	0,26	ENSG00000213204
0,78	0,03	0,06	ENSG00000164414
1,05	0,56	0,66	ENSG00000146282
0,84	0,05	0,09	ENSG00000135336
0,76	0,00	0,00	ENSG00000135334
#N/D	#N/D	#N/D	ENSG00000118434
0,66	0,51	0,62	ENSG00000118432
22,89	0,26	#N/D	ENSG00000279565
1,12	0,20	0,30	ENSG00000111880
1,11	0,84	0,91	ENSG00000280277
1,15	0,10	0,17	ENSG00000146278
1,83	0,15	0,24	ENSG00000154548
0,45	0,00	0,00	ENSG00000146281
1,14	0,97	#N/D	ENSG00000146276
1,15	0,33	0,45	ENSG00000111886
1,02	0,79	0,87	ENSG00000198833
2,24	0,00	0,00	ENSG00000025039
1,92	0,00	0,00	ENSG00000135299
0,79	0,01	0,02	ENSG00000083099
0,80	0,01	0,02	ENSG00000112159
0,96	0,62	0,71	ENSG00000118412
#N/D	#N/D	#N/D	ENSG00000135355
0,75	0,07	0,12	ENSG00000112182
1,08	0,33	0,44	ENSG00000135341
2,31	0,01	0,03	ENSG00000135333
#N/D	#N/D	#N/D	ENSG00000280451
#N/D	#N/D	#N/D	ENSG00000279403
1,03	0,77	0,85	ENSG00000172469
1,14	0,97	#N/D	ENSG00000172461
1,13	0,16	0,26	ENSG00000014123
#N/D	#N/D	#N/D	ENSG00000112214
181,85	0,00	0,00	ENSG00000112218
0,46	0,00	0,00	ENSG00000123545

0,13	0,46	0,56	ENSG00000186231
0,70	0,00	0,00	ENSG00000146263
4,76	0,00	0,00	ENSG00000184486
0,91	0,25	0,35	ENSG00000112234
0,68	0,00	0,01	ENSG00000146267
1,09	0,38	0,49	ENSG00000132423
1,03	0,75	0,84	ENSG00000132424
0,77	0,01	0,01	ENSG00000123552
0,61	0,03	0,05	ENSG00000279170
1,06	0,53	0,64	ENSG00000112237
0,98	0,99	0,99	ENSG00000112238
8,39	0,46	#N/D	ENSG00000152034
3,56	0,00	0,00	ENSG00000112246
1,02	0,81	0,89	ENSG00000112249
1,43	0,01	0,01	ENSG00000164418
1,36	0,00	0,00	ENSG00000085382
0,09	0,01	0,03	ENSG00000187772
0,57	0,00	0,00	ENSG00000112276
0,49	0,00	0,00	ENSG00000132429
1,12	0,17	0,26	ENSG00000085377
0,22	0,00	0,00	ENSG00000057657
0,81	0,01	0,03	ENSG00000057663
0,92	0,34	0,45	ENSG00000112297
0,68	0,00	0,00	ENSG00000130347
0,64	0,00	0,00	ENSG00000130348
0,07	0,00	0,00	ENSG00000272398
0,67	0,00	0,01	ENSG00000130349
0,85	0,09	0,16	ENSG00000178409
1,11	0,23	0,34	ENSG00000164494
2,23	0,00	0,00	ENSG00000112320
#N/D	#N/D	#N/D	ENSG00000280135
#N/D	#N/D	#N/D	ENSG00000146285
1,12	0,16	0,25	ENSG00000025796
0,86	0,09	0,15	ENSG00000081087
1,54	0,44	0,56	ENSG00000112333
1,14	0,97	#N/D	ENSG00000279398
0,83	0,02	0,05	ENSG00000112335
0,75	0,00	0,01	ENSG00000135537
0,76	0,59	0,69	ENSG00000279498
1,43	0,00	0,00	ENSG00000118689
1,39	0,01	0,03	ENSG00000118690
0,84	0,16	0,25	ENSG00000080546
0,87	0,11	0,19	ENSG00000183137
1,07	0,44	0,56	ENSG00000135535
0,57	0,01	0,03	ENSG00000185250
0,79	0,02	0,03	ENSG00000135587
0,90	0,20	0,30	ENSG00000135596
0,82	0,03	0,06	ENSG00000112365

0,72	0,10	0,17	ENSG00000155085
0,82	0,03	0,06	ENSG00000112367
#N/D	#N/D	#N/D	ENSG00000146360
0,67	0,00	0,00	ENSG00000112290
0,91	0,29	0,40	ENSG00000168438
2,62	0,10	0,16	ENSG00000053328
1,25	0,21	0,32	ENSG00000203797
#N/D	#N/D	#N/D	ENSG00000004809
0,81	0,02	0,03	ENSG00000155111
0,88	0,13	0,20	ENSG00000123505
0,43	0,00	0,00	ENSG00000155115
0,69	0,00	0,00	ENSG00000197498
0,49	0,01	0,03	ENSG00000112394
1,16	0,08	0,15	ENSG00000173214
0,73	0,00	0,00	ENSG00000009413
0,59	0,00	0,00	ENSG00000056972
0,16	0,00	0,00	ENSG00000010810
0,13	0,46	0,56	ENSG00000112761
0,30	0,00	0,00	ENSG00000074935
1,08	0,48	0,58	ENSG00000203778
1,26	0,00	0,01	ENSG00000112769
0,13	0,46	0,56	ENSG00000281613
0,29	0,12	0,19	ENSG00000251258
0,51	0,00	0,00	ENSG00000277443
0,77	0,00	0,00	ENSG00000196591
5,94	0,00	0,01	ENSG00000249853
0,70	0,02	0,05	ENSG00000111816
1,18	0,08	0,14	ENSG00000178425
1,52	0,13	0,21	ENSG00000123500
0,97	0,74	0,82	ENSG00000187189
1,00	0,96	0,98	ENSG00000111817
0,86	0,07	0,12	ENSG00000189241
0,97	0,91	0,97	ENSG00000285446
3,56	0,23	0,34	ENSG00000188820
1,14	0,97	0,98	ENSG00000173626
0,36	0,00	0,00	ENSG00000178033
0,05	0,24	0,35	ENSG00000164451
0,85	0,07	0,12	ENSG00000111832
0,01	0,09	0,15	ENSG00000111834
0,77	0,01	0,02	ENSG00000153975
0,60	0,00	0,00	ENSG00000196911
0,98	0,98	0,99	ENSG00000183807
1,14	0,97	0,98	ENSG00000173612
#N/D	#N/D	#N/D	ENSG00000185002
15,64	0,33	0,44	ENSG00000170162
8,17	0,00	0,00	ENSG00000047936
#N/D	#N/D	#N/D	ENSG00000282218
1,27	0,01	0,01	ENSG00000164465

0,83	0,03	0,06	ENSG00000047932
1,56	0,00	0,00	ENSG00000153989
3,56	0,23	0,34	ENSG00000196376
0,85	0,10	0,17	ENSG00000111860
0,44	0,05	0,10	ENSG00000198523
0,43	0,00	0,00	ENSG00000111877
0,70	0,00	0,00	ENSG00000111875
0,24	0,00	0,00	ENSG00000111879
0,32	0,00	0,00	ENSG00000111885
0,69	0,00	0,00	ENSG00000146350
0,26	0,00	0,00	ENSG00000152661
0,95	0,54	0,65	ENSG00000025156
1,50	0,00	0,00	ENSG00000279453
1,16	0,06	0,12	ENSG00000111897
1,92	0,23	0,34	ENSG00000135549
0,85	0,51	0,61	ENSG00000279114
1,14	0,97	#N/D	ENSG00000164434
0,64	0,06	0,11	ENSG00000172594
8,39	0,46	#N/D	ENSG00000146352
1,14	0,97	#N/D	ENSG00000186439
1,68	0,40	0,52	ENSG00000188580
1,16	0,09	0,16	ENSG00000146373
1,54	0,11	0,19	ENSG00000111907
0,69	0,00	0,00	ENSG00000111906
22,89	0,26	0,37	ENSG00000135547
0,40	0,00	0,00	ENSG00000111912
0,78	0,01	0,01	ENSG00000111911
0,60	0,00	0,00	ENSG00000066651
1,19	0,05	0,10	ENSG00000203760
0,13	0,46	0,56	ENSG00000146374
0,95	0,58	0,68	ENSG00000118518
0,89	0,22	0,32	ENSG00000093144
0,73	0,37	0,48	ENSG00000189367
0,24	0,00	0,00	ENSG00000255330
0,36	0,00	0,00	ENSG00000214338
0,03	0,17	0,26	ENSG00000184530
1,14	0,97	#N/D	ENSG00000172673
0,97	0,69	0,78	ENSG00000152894
1,08	0,36	0,48	ENSG00000196569
1,31	0,00	0,01	ENSG00000146376
3,18	0,00	0,00	ENSG00000203756
0,99	0,91	0,97	ENSG00000198945
0,67	0,65	0,74	ENSG00000164483
0,33	0,00	0,00	ENSG00000164484
0,51	0,42	0,54	ENSG00000256162
0,70	0,00	0,00	ENSG00000079819
0,32	0,00	0,00	ENSG00000118507
1,29	0,74	0,82	ENSG00000118520

1,07	0,45	0,56	ENSG00000112282
0,89	0,79	0,87	ENSG00000154269
1,14	0,97	0,98	ENSG00000180658
22,89	0,26	0,37	ENSG00000236761
0,62	0,00	0,00	ENSG00000197594
1,06	0,48	0,59	ENSG00000118523
0,36	0,35	0,46	ENSG00000280155
0,79	0,08	0,14	ENSG00000079931
0,87	0,11	0,19	ENSG00000079950
#N/D	#N/D	#N/D	ENSG00000237110
#N/D	#N/D	#N/D	ENSG00000146385
1,14	0,97	#N/D	ENSG00000146383
#N/D	#N/D	#N/D	ENSG00000135569
#N/D	#N/D	#N/D	ENSG00000146378
0,07	0,31	0,43	ENSG00000146399
0,11	0,00	0,00	ENSG00000112299
0,01	0,07	0,12	ENSG00000112303
0,33	0,00	0,00	ENSG00000146409
0,80	0,01	0,01	ENSG00000112306
8,39	0,46	0,56	ENSG00000279960
1,13	0,15	0,23	ENSG00000112319
0,64	0,00	0,00	ENSG00000118526
0,68	0,00	0,00	ENSG00000028839
#N/D	#N/D	#N/D	ENSG00000288529
0,72	0,00	0,01	ENSG00000146411
0,66	0,00	0,00	ENSG00000118515
1,97	0,00	0,01	ENSG00000118514
1,20	0,03	0,05	ENSG00000112339
0,21	0,00	0,00	ENSG00000118513
0,77	0,00	0,01	ENSG00000135541
0,27	0,00	0,00	ENSG00000171408
0,88	0,17	0,27	ENSG00000146410
0,94	0,47	0,58	ENSG00000029363
0,11	0,00	0,00	ENSG00000135525
1,02	0,83	0,90	ENSG00000197442
0,80	0,06	0,11	ENSG00000112357
2,53	0,03	0,05	ENSG00000182747
0,01	0,05	0,10	ENSG00000016402
#N/D	#N/D	#N/D	ENSG00000164485
0,57	0,00	0,00	ENSG00000027697
#N/D	#N/D	#N/D	ENSG00000177468
0,46	0,00	0,00	ENSG00000118503
1,17	0,05	0,09	ENSG00000112378
0,13	0,00	0,00	ENSG00000112379
1,11	0,84	0,91	ENSG00000254440
1,14	0,97	#N/D	ENSG00000262543
0,85	0,06	0,10	ENSG00000051620
0,87	0,10	0,16	ENSG00000135540

0,93	0,43	0,55	ENSG00000024862
1,70	0,10	0,17	ENSG00000203734
0,44	0,00	0,00	ENSG00000135597
0,63	0,00	0,00	ENSG00000146386
1,10	0,24	0,34	ENSG00000112406
0,36	0,02	0,05	ENSG00000164440
2,94	0,00	0,00	ENSG00000164442
0,53	0,18	0,27	ENSG00000135577
0,13	0,46	#N/D	ENSG00000203733
0,81	0,01	0,03	ENSG00000009844
0,42	0,00	0,00	ENSG00000112414
0,80	0,01	0,01	ENSG00000010818
0,75	0,00	0,00	ENSG00000146416
0,58	0,00	0,00	ENSG00000189007
0,55	0,00	0,00	ENSG00000034693
0,66	0,00	0,00	ENSG00000001036
0,55	0,00	0,00	ENSG00000112419
0,04	0,20	0,30	ENSG00000257065
0,72	0,00	0,00	ENSG00000135521
#N/D	#N/D	#N/D	ENSG00000280148
1,14	0,97	#N/D	ENSG00000118491
0,72	0,00	0,00	ENSG00000118495
1,32	0,00	0,00	ENSG00000169976
0,06	0,00	0,00	ENSG00000135604
1,21	0,02	0,05	ENSG00000152818
0,51	0,00	0,00	ENSG00000112425
1,47	0,02	0,04	ENSG00000280184
0,78	0,00	0,01	ENSG00000118496
0,90	0,20	0,30	ENSG00000146414
0,28	0,01	0,02	ENSG00000152822
0,83	0,03	0,06	ENSG00000118508
1,14	0,97	#N/D	ENSG00000118492
0,76	0,00	0,00	ENSG00000164506
1,61	0,00	0,00	ENSG00000203727
0,60	0,00	0,00	ENSG00000111961
1,28	0,00	0,01	ENSG00000111962
0,76	0,00	0,00	ENSG00000055208
2,01	0,04	0,08	ENSG00000177688
0,64	0,05	0,09	ENSG00000178199
0,83	0,05	0,09	ENSG00000131013
0,77	0,00	0,01	ENSG00000055211
0,79	0,01	0,02	ENSG00000186625
0,95	0,54	0,64	ENSG00000131023
1,11	0,19	0,29	ENSG00000120253
1,24	0,01	0,02	ENSG00000120265
1,28	0,02	0,05	ENSG00000285991
1,78	0,00	0,00	ENSG00000120256
0,69	0,00	0,00	ENSG00000164520

1,06	0,65	0,75	ENSG00000203722
0,88	0,17	0,26	ENSG00000131015
0,28	0,00	0,00	ENSG00000111981
0,11	0,00	0,00	ENSG00000155918
0,63	0,00	0,00	ENSG00000131019
0,10	0,02	0,04	ENSG00000198729
0,29	0,03	0,05	ENSG00000009765
0,26	0,00	0,00	ENSG00000120278
0,71	0,00	0,00	ENSG00000120254
0,12	0,00	0,00	ENSG00000131016
0,79	0,01	0,02	ENSG00000181472
1,01	0,92	0,98	ENSG00000155906
0,77	0,01	0,01	ENSG00000146476
1,25	0,54	0,64	ENSG00000120262
0,27	0,00	0,00	ENSG00000091831
0,58	0,00	0,00	ENSG00000131018
1,14	0,97	0,98	ENSG00000120279
0,13	0,46	0,56	ENSG00000146469
0,92	0,34	0,45	ENSG00000112029
0,79	0,01	0,02	ENSG00000112031
1,36	0,00	0,00	ENSG00000091844
1,91	0,35	0,47	ENSG00000112038
1,14	0,97	0,98	ENSG00000074706
1,14	0,97	0,98	ENSG00000288520
1,01	0,88	0,95	ENSG00000153721
0,90	0,19	0,29	ENSG00000213079
1,91	0,00	0,00	ENSG00000146426
0,99	0,88	0,94	ENSG00000029639
0,72	0,06	0,11	ENSG00000171217
1,14	0,97	#N/D	ENSG00000074771
0,72	0,00	0,00	ENSG00000049618
0,70	0,00	0,00	ENSG00000215712
0,89	0,20	0,29	ENSG00000175048
1,32	0,00	0,00	ENSG00000130340
0,90	0,19	0,29	ENSG00000078269
0,51	0,00	0,00	ENSG00000122335
0,70	0,00	0,00	ENSG00000272047
0,91	0,25	0,36	ENSG00000130338
0,93	0,35	0,46	ENSG00000146433
1,36	0,00	0,00	ENSG00000146425
0,87	0,12	0,20	ENSG00000164674
0,66	0,00	0,00	ENSG00000092820
1,16	0,10	0,17	ENSG00000130363
6,99	0,05	0,10	ENSG00000164691
5,08	0,00	0,00	ENSG00000164694
0,84	0,02	0,05	ENSG00000291237
0,79	0,00	0,01	ENSG00000146457
1,97	0,00	0,00	ENSG00000120437

0,94	0,43	0,55	ENSG00000120438
1,35	0,00	0,00	ENSG00000112110
0,16	0,07	0,12	ENSG00000146453
1,60	0,51	0,62	ENSG00000130368
0,98	0,79	0,87	ENSG00000197081
0,44	0,04	0,08	ENSG00000175003
1,14	0,97	0,98	ENSG00000112499
0,21	0,04	0,07	ENSG00000146477
1,14	0,97	0,98	ENSG00000198670
0,13	0,46	0,56	ENSG00000122194
1,14	0,12	0,19	ENSG00000085511
0,90	0,22	0,32	ENSG00000026652
0,99	0,96	0,98	ENSG00000185345
0,68	0,28	0,39	ENSG00000112530
1,02	0,79	0,87	ENSG00000112531
0,07	0,31	0,43	ENSG00000112539
0,81	0,01	0,03	ENSG00000112541
3,56	0,23	0,34	ENSG00000164458
2,03	0,07	0,12	ENSG00000176381
0,99	0,87	0,94	ENSG00000198818
1,31	0,01	0,02	ENSG00000060762
0,86	0,76	0,84	ENSG00000280057
0,51	0,00	0,00	ENSG00000071242
0,67	0,02	0,04	ENSG00000235272
0,74	0,69	0,78	ENSG00000249141
1,08	0,42	0,54	ENSG00000026297
0,85	0,06	0,11	ENSG00000213066
0,49	0,00	0,00	ENSG00000272980
0,54	0,22	0,32	ENSG00000112486
1,14	0,97	0,98	ENSG00000120436
1,14	0,97	0,98	ENSG00000112494
51,90	0,15	0,24	ENSG00000120440
0,82	0,01	0,03	ENSG00000130396
1,45	0,56	0,67	ENSG00000125337
0,13	0,46	0,56	ENSG00000153303
0,13	0,46	0,56	ENSG00000164488
363,69	0,03	0,06	ENSG00000112562
1,14	0,97	0,98	ENSG00000280665
1,14	0,97	#N/D	ENSG00000279810
0,97	0,68	0,77	ENSG00000186340
0,39	0,00	0,00	ENSG00000285733
1,32	0,00	0,00	ENSG00000184465
0,56	0,00	0,00	ENSG00000185127
0,69	0,00	0,00	ENSG00000130024
1,20	0,64	0,73	ENSG00000184786
0,75	0,00	0,01	ENSG00000130023
0,50	0,00	0,00	ENSG00000198719
1,15	0,09	0,15	ENSG00000112584

0,88	0,13	0,21	ENSG00000008018
0,96	0,64	0,74	ENSG00000112592
0,78	0,00	0,01	ENSG00000071994
1,99	0,00	0,00	ENSG00000177706
2,03	0,00	0,00	ENSG00000249852
2,71	0,36	0,48	ENSG00000248767
0,32	0,00	0,00	ENSG00000197461
0,31	0,00	0,00	ENSG00000188191
0,72	0,00	0,00	ENSG00000164818
1,20	0,03	0,05	ENSG00000164828
1,33	0,00	0,00	ENSG00000239857
0,57	0,00	0,00	ENSG00000105963
0,99	0,95	0,98	ENSG00000240230
1,75	0,03	0,06	ENSG00000073067
1,02	0,83	0,90	ENSG00000146540
1,21	0,28	0,39	ENSG00000164849
1,00	0,99	1,00	ENSG00000164850
1,12	0,20	0,30	ENSG00000178381
0,02	0,10	0,17	ENSG00000164853
1,61	0,00	0,00	ENSG00000164877
1,04	0,58	0,68	ENSG00000164880
1,23	0,01	0,02	ENSG00000198517
0,44	0,00	0,00	ENSG00000164855
0,75	0,00	0,00	ENSG00000157778
0,25	0,00	0,00	ENSG00000225968
0,75	0,00	0,00	ENSG00000002822
1,76	0,00	0,00	ENSG00000286192
0,60	0,00	0,00	ENSG00000122687
0,94	0,48	0,58	ENSG00000106268
0,88	0,13	0,21	ENSG00000106266
1,08	0,35	0,46	ENSG00000106263
0,98	0,86	0,92	ENSG00000136213
8,39	0,46	#N/D	ENSG00000275572
0,49	0,00	0,00	ENSG00000106003
0,86	0,06	0,11	ENSG00000106009
0,94	0,49	0,59	ENSG00000106012
1,30	0,00	0,00	ENSG00000136295
1,71	0,00	0,00	ENSG00000174945
1,31	0,00	0,00	ENSG00000146535
1,14	0,97	0,98	ENSG00000198286
1,28	0,01	0,02	ENSG00000146555
1,43	0,00	0,00	ENSG00000164916
1,79	0,00	0,00	ENSG00000242802
1,38	0,02	0,04	ENSG00000157927
1,91	0,35	0,47	ENSG00000218823
#N/D	#N/D	#N/D	ENSG00000136297
1,15	0,23	0,34	ENSG00000272968
0,91	0,29	0,40	ENSG00000146587

0,80	0,01	0,02	ENSG00000157954
1,92	0,00	0,00	ENSG00000164638
1,30	0,00	0,00	ENSG00000182095
1,29	0,00	0,00	ENSG00000155034
1,11	0,19	0,29	ENSG00000075624
1,83	0,00	0,00	ENSG00000075618
1,04	0,60	0,69	ENSG00000011275
8,39	0,46	#N/D	ENSG00000122543
0,77	0,00	0,01	ENSG00000122674
0,11	0,03	0,05	ENSG00000155026
0,60	0,00	0,00	ENSG00000122512
0,48	0,00	0,00	ENSG00000106305
1,21	0,02	0,04	ENSG00000086232
1,79	0,00	0,00	ENSG00000157999
0,88	0,16	0,25	ENSG00000106346
0,96	0,59	0,69	ENSG00000008256
1,36	0,00	0,00	ENSG00000286075
1,23	0,03	0,06	ENSG00000178397
0,88	0,12	0,19	ENSG00000136238
0,75	0,00	0,00	ENSG00000164535
1,69	0,00	0,00	ENSG00000136240
1,93	0,08	0,14	ENSG00000215045
0,71	0,00	0,00	ENSG00000136247
0,89	0,17	0,26	ENSG00000146576
0,68	0,00	0,00	ENSG00000236609
1,07	0,42	0,54	ENSG00000205903
1,33	0,00	0,00	ENSG00000164631
4,08	0,03	0,05	ENSG00000169402
0,82	0,02	0,04	ENSG00000146574
1,12	0,22	0,32	ENSG00000106392
0,05	0,24	0,35	ENSG00000215018
0,68	0,00	0,00	ENSG00000164654
0,92	0,35	0,47	ENSG00000106399
0,99	0,92	0,98	ENSG00000219545
0,64	0,00	0,00	ENSG00000106415
34,42	0,00	0,00	ENSG00000003147
#N/D	#N/D	#N/D	ENSG00000122584
1,02	0,77	0,85	ENSG00000189043
0,71	0,00	0,00	ENSG00000106443
1,77	0,26	0,37	ENSG00000005108
0,58	0,00	0,00	ENSG00000106460
0,98	0,85	0,92	ENSG00000146530
#N/D	#N/D	#N/D	ENSG00000226690
11,74	0,00	0,00	ENSG00000006747
0,82	0,03	0,07	ENSG00000122644
0,61	0,00	0,00	ENSG00000006468
1,85	0,60	0,69	ENSG00000136267
1,14	0,97	0,98	ENSG00000187546

8,39	0,46	#N/D	ENSG00000106511
37,39	0,19	0,29	ENSG00000214960
0,13	0,46	0,56	ENSG00000171243
1,14	0,97	#N/D	ENSG00000205858
1,00	0,96	0,98	ENSG00000106524
0,74	0,00	0,00	ENSG00000136261
2,10	0,00	0,00	ENSG00000106537
15,64	0,33	0,44	ENSG00000106541
#N/D	#N/D	#N/D	ENSG00000173467
0,94	0,43	0,55	ENSG00000106546
0,11	0,00	0,00	ENSG00000283321
0,64	0,00	0,00	ENSG00000071189
0,63	0,06	0,11	ENSG00000279048
#N/D	#N/D	#N/D	ENSG00000280340
#N/D	#N/D	#N/D	ENSG00000229937
0,25	0,00	0,00	ENSG00000048052
0,68	0,00	0,00	ENSG00000122691
#N/D	#N/D	#N/D	ENSG00000146618
0,82	0,03	0,05	ENSG00000105849
0,13	0,46	#N/D	ENSG00000173452
0,53	0,58	0,68	ENSG00000183742
0,14	0,00	0,00	ENSG00000105855
2,80	0,20	0,30	ENSG00000004846
2,04	0,00	0,00	ENSG00000164651
1,10	0,31	0,43	ENSG00000105866
0,54	0,01	0,02	ENSG00000105877
0,73	0,00	0,00	ENSG00000164649
0,36	0,00	0,00	ENSG00000136237
0,67	0,00	0,00	ENSG00000105889
0,02	0,00	0,00	ENSG00000136244
1,09	0,29	0,41	ENSG00000196683
0,65	0,00	0,00	ENSG00000122591
1,29	0,00	0,01	ENSG00000122550
0,64	0,00	0,00	ENSG00000136243
0,45	0,00	0,00	ENSG00000136235
1,03	0,76	0,84	ENSG00000156928
1,25	0,01	0,01	ENSG00000136231
0,94	0,52	0,63	ENSG00000164548
0,61	0,00	0,01	ENSG00000169193
6,25	0,00	0,01	ENSG00000188732
8,39	0,46	0,56	ENSG00000196335
1,14	0,97	0,98	ENSG00000122585
0,72	0,00	0,00	ENSG00000105926
0,42	0,00	0,00	ENSG00000105928
0,35	0,00	0,00	ENSG00000070882
1,58	0,00	0,00	ENSG00000172115
0,46	0,00	0,00	ENSG00000153790
#N/D	#N/D	#N/D	ENSG00000105954

0,46	0,00	0,00	ENSG00000050344
0,88	0,10	0,17	ENSG00000122566
0,72	0,00	0,00	ENSG00000122565
0,58	0,00	0,00	ENSG00000086300
1,14	0,97	#N/D	ENSG00000280255
0,92	0,32	0,44	ENSG00000005020
0,91	0,38	0,50	ENSG00000105991
2,01	0,00	0,00	ENSG00000105996
0,92	0,53	0,64	ENSG00000105997
1,25	0,07	0,13	ENSG00000197576
0,67	0,00	0,01	ENSG00000106004
0,77	0,06	0,11	ENSG00000106006
0,54	0,00	0,00	ENSG00000122592
1,53	0,00	0,00	ENSG00000078399
0,98	0,98	0,99	ENSG00000257184
0,96	0,68	0,77	ENSG00000253293
0,86	0,21	0,31	ENSG00000005073
1,97	0,00	0,00	ENSG00000106031
22,89	0,26	0,37	ENSG00000106038
0,13	0,46	#N/D	ENSG00000279655
#N/D	#N/D	#N/D	ENSG00000279429
0,94	0,45	0,56	ENSG00000106049
0,96	0,66	0,75	ENSG00000106052
0,70	0,00	0,00	ENSG00000153814
0,54	0,00	0,00	ENSG00000146592
1,43	0,06	0,11	ENSG00000255690
0,23	0,00	0,00	ENSG00000106066
0,46	0,00	0,00	ENSG00000106069
0,16	0,07	0,12	ENSG00000176532
0,63	0,28	0,39	ENSG00000122574
1,04	0,64	0,74	ENSG00000136193
1,40	0,00	0,00	ENSG00000106080
0,92	0,30	0,41	ENSG00000106086
0,77	0,00	0,01	ENSG00000180354
0,76	0,03	0,06	ENSG00000180233
0,01	0,09	0,16	ENSG00000281593
0,66	0,00	0,00	ENSG00000106100
0,76	0,00	0,01	ENSG00000006625
0,57	0,24	0,35	ENSG00000281039
0,66	0,00	0,00	ENSG00000106105
0,67	0,65	0,74	ENSG00000106113
1,93	0,13	0,21	ENSG00000241644
1,14	0,97	0,98	ENSG00000254959
0,87	0,34	0,45	ENSG00000106125
1,14	0,97	0,98	ENSG00000250424
3,21	0,00	0,00	ENSG00000240583
1,14	0,97	#N/D	ENSG00000106128
0,17	0,00	0,01	ENSG00000078549

#N/D	#N/D	#N/D	ENSG00000164600
1,14	0,97	0,98	ENSG00000180347
1,14	0,97	#N/D	ENSG00000106341
2,38	0,00	0,00	ENSG00000154678
0,62	0,00	0,00	ENSG00000106355
0,94	0,43	0,55	ENSG00000105778
0,89	0,15	0,24	ENSG00000170852
0,83	0,02	0,04	ENSG00000122642
0,28	0,00	0,00	ENSG00000122643
1,08	0,47	0,57	ENSG00000164610
1,20	0,04	0,08	ENSG00000122507
0,26	0,08	0,14	ENSG00000164619
8,39	0,46	#N/D	ENSG00000187258
1,33	0,00	0,00	ENSG00000173852
0,93	0,50	0,61	ENSG00000164532
0,75	0,00	0,00	ENSG00000122557
0,69	0,00	0,00	ENSG00000122545
1,53	0,00	0,00	ENSG00000122547
0,98	0,86	0,93	ENSG00000164542
1,22	0,01	0,03	ENSG00000011426
0,22	0,00	0,00	ENSG00000136250
2,24	0,10	0,17	ENSG00000155849
1,14	0,97	#N/D	ENSG00000187037
1,14	0,97	0,98	ENSG00000290149
#N/D	#N/D	#N/D	ENSG00000086288
0,27	0,22	0,32	ENSG00000106483
0,44	0,00	0,00	ENSG00000086289
0,85	0,06	0,10	ENSG00000010270
1,85	0,60	0,69	ENSG00000227191
#N/D	#N/D	#N/D	ENSG00000211687
#N/D	#N/D	#N/D	ENSG00000211688
0,13	0,46	0,56	ENSG00000211689
#N/D	#N/D	#N/D	ENSG00000289746
#N/D	#N/D	#N/D	ENSG00000211690
#N/D	#N/D	#N/D	ENSG00000211691
#N/D	#N/D	#N/D	ENSG00000211692
#N/D	#N/D	#N/D	ENSG00000211693
#N/D	#N/D	#N/D	ENSG00000211694
1,14	0,97	#N/D	ENSG00000211695
#N/D	#N/D	#N/D	ENSG00000211696
0,03	0,17	0,26	ENSG00000211697
1,85	0,60	#N/D	ENSG00000211698
1,44	0,68	0,77	ENSG00000211699
0,13	0,46	#N/D	ENSG00000233306
0,53	0,58	0,68	ENSG00000211701
0,99	0,96	0,98	ENSG00000078053
0,88	0,14	0,22	ENSG00000006715
#N/D	#N/D	#N/D	ENSG00000106536

0,87	0,32	0,44	ENSG00000241127
0,94	0,46	0,56	ENSG00000006451
0,84	0,04	0,07	ENSG00000065883
0,93	0,43	0,54	ENSG00000168303
0,70	0,02	0,04	ENSG00000175600
0,00	0,02	0,04	ENSG00000122641
0,57	0,00	0,00	ENSG00000106571
0,36	0,00	0,00	ENSG00000136197
0,52	0,00	0,00	ENSG00000256646
0,92	0,29	0,40	ENSG00000106588
1,09	0,34	0,46	ENSG00000106591
0,52	0,00	0,00	ENSG00000002746
1,11	0,21	0,31	ENSG00000164543
0,90	0,18	0,28	ENSG00000106603
1,08	0,34	0,46	ENSG00000106605
1,05	0,59	0,69	ENSG00000062582
0,01	0,07	0,12	ENSG00000270617
0,94	0,46	0,56	ENSG00000106608
0,39	0,00	0,00	ENSG00000078967
1,10	0,47	0,57	ENSG00000136206
1,36	0,00	0,00	ENSG00000136279
15,64	0,33	0,44	ENSG00000164708
1,06	0,53	0,63	ENSG00000122678
0,86	0,05	0,09	ENSG00000106624
1,03	0,72	0,81	ENSG00000106628
#N/D	#N/D	#N/D	ENSG00000106631
0,99	1,00	1,00	ENSG00000106633
1,65	0,00	0,00	ENSG00000106636
2,60	0,00	0,00	ENSG00000058404
0,66	0,51	0,62	ENSG00000279853
1,20	0,03	0,05	ENSG00000015676
4,32	0,00	0,00	ENSG00000015520
0,96	0,60	0,70	ENSG00000136271
1,13	0,12	0,19	ENSG00000158604
0,76	0,00	0,00	ENSG00000105953
0,72	0,00	0,00	ENSG00000122515
1,15	0,08	0,14	ENSG00000196262
1,27	0,00	0,01	ENSG00000105968
1,16	0,07	0,12	ENSG00000146676
0,12	0,00	0,01	ENSG00000136286
1,09	0,34	0,45	ENSG00000136280
0,09	0,00	0,00	ENSG00000136274
0,69	0,00	0,00	ENSG00000136270
1,14	0,97	#N/D	ENSG00000122679
3,86	0,00	0,00	ENSG00000164742
0,02	0,13	0,21	ENSG00000283247
0,03	0,17	0,26	ENSG00000146678
4,59	0,00	0,00	ENSG00000146674

1,05	0,51	0,61	ENSG00000136205
0,84	0,50	0,60	ENSG00000158683
0,93	0,41	0,52	ENSG00000136273
0,27	0,22	0,32	ENSG00000164744
0,60	0,09	0,15	ENSG00000164746
0,16	0,00	0,00	ENSG00000183696
3,84	0,00	0,00	ENSG00000179869
#N/D	#N/D	#N/D	ENSG00000279578
#N/D	#N/D	#N/D	ENSG00000218305
1,14	0,97	#N/D	ENSG00000279104
37,39	0,19	0,29	ENSG00000188730
#N/D	#N/D	#N/D	ENSG00000042813
#N/D	#N/D	#N/D	ENSG00000164500
1,14	0,97	#N/D	ENSG00000185811
0,52	0,00	0,00	ENSG00000132436
1,14	0,97	#N/D	ENSG00000132437
0,37	0,00	0,00	ENSG00000106070
1,92	0,28	0,39	ENSG00000106078
#N/D	#N/D	#N/D	ENSG00000221900
#N/D	#N/D	#N/D	ENSG00000170419
1,45	0,00	0,00	ENSG00000132432
1,16	0,07	0,12	ENSG00000146648
0,59	0,00	0,00	ENSG00000132434
1,01	0,89	0,95	ENSG00000154978
1,14	0,97	#N/D	ENSG00000154997
0,88	0,69	0,78	ENSG00000249773
2,10	0,04	0,07	ENSG00000178665
0,90	0,20	0,30	ENSG00000146729
0,90	0,26	0,36	ENSG00000239789
0,63	0,00	0,00	ENSG00000146733
0,81	0,01	0,02	ENSG00000146731
1,16	0,07	0,13	ENSG00000129103
1,17	0,32	0,43	ENSG00000164776
0,94	0,41	0,52	ENSG00000106153
#N/D	#N/D	#N/D	ENSG00000185290
8,39	0,46	0,56	ENSG00000185177
#N/D	#N/D	#N/D	ENSG00000280225
15,64	0,33	0,44	ENSG00000182111
1,14	0,97	0,98	ENSG00000241149
0,13	0,46	0,56	ENSG00000214652
#N/D	#N/D	#N/D	ENSG00000223614
0,53	0,58	0,68	ENSG00000197123
0,92	0,33	0,45	ENSG00000234444
0,68	0,00	0,00	ENSG00000173041
0,69	0,00	0,00	ENSG00000196247
0,88	0,43	0,55	ENSG00000197008
1,17	0,26	0,37	ENSG00000198039
0,93	0,48	0,58	ENSG00000152926

1,12	0,26	0,36	ENSG00000213462
1,20	0,18	0,27	ENSG00000146757
0,98	0,81	0,89	ENSG00000196715
1,61	0,00	0,00	ENSG00000169919
1,24	0,02	0,04	ENSG00000126522
0,35	0,01	0,01	ENSG00000249319
0,91	0,28	0,39	ENSG00000241258
0,82	0,02	0,04	ENSG00000169902
0,28	0,04	0,08	ENSG00000279785
2,42	0,00	0,00	ENSG00000243335
0,50	0,00	0,00	ENSG00000284461
1,03	0,72	0,81	ENSG00000154710
1,28	0,00	0,01	ENSG00000106609
0,84	0,05	0,09	ENSG00000126524
0,91	0,34	0,45	ENSG00000198874
1,22	0,36	0,48	ENSG00000230358
2,76	0,00	0,00	ENSG00000158321
22,89	0,26	0,37	ENSG00000185274
8,39	0,46	0,56	ENSG00000183166
0,97	0,89	0,96	ENSG00000277149
0,99	0,86	0,93	ENSG00000196313
8,39	0,46	0,56	ENSG00000155428
0,07	0,01	0,01	ENSG00000273520
0,11	0,00	0,01	ENSG00000275976
0,15	0,00	0,00	ENSG00000262461
0,88	0,40	0,52	ENSG00000274570
0,65	0,00	0,00	ENSG00000130305
0,13	0,46	#N/D	ENSG00000146755
0,98	0,97	0,98	ENSG00000077800
2,24	0,00	0,01	ENSG00000188763
0,86	0,05	0,10	ENSG00000009954
0,68	0,00	0,00	ENSG00000106635
1,02	0,77	0,85	ENSG00000106638
0,44	0,02	0,03	ENSG00000009950
1,39	0,02	0,04	ENSG00000176428
1,20	0,07	0,13	ENSG00000176410
0,91	0,24	0,34	ENSG00000071462
0,76	0,00	0,00	ENSG00000106089
0,63	0,00	0,00	ENSG00000106077
0,98	0,99	0,99	ENSG00000165215
0,70	0,02	0,04	ENSG00000189143
0,43	0,01	0,02	ENSG00000165171
0,04	0,20	0,30	ENSG00000175877
0,87	0,77	0,85	ENSG00000049540
0,66	0,00	0,00	ENSG00000106683
1,13	0,13	0,21	ENSG00000106682
0,91	0,54	0,65	ENSG00000086730
1,12	0,18	0,27	ENSG00000049541

1,07	0,42	0,54	ENSG00000106665
0,95	0,49	0,59	ENSG00000006704
#N/D	#N/D	#N/D	ENSG00000279005
0,87	0,08	0,14	ENSG00000263001
0,05	0,24	0,35	ENSG00000158517
0,99	0,91	0,97	ENSG00000289346
2,63	0,00	0,00	ENSG00000196275
1,28	0,23	0,34	ENSG00000184616
1,55	0,00	0,00	ENSG00000274070
0,94	0,49	0,59	ENSG00000274523
0,70	0,00	0,01	ENSG00000174428
1,14	0,97	0,98	ENSG00000286038
138,91	0,07	0,13	ENSG00000286137
131,66	0,08	0,13	ENSG00000286014
0,92	0,46	0,56	ENSG00000178809
1,01	0,93	0,98	ENSG00000272391
1,95	0,00	0,00	ENSG00000170092
0,96	0,65	0,74	ENSG00000127946
0,80	0,55	0,65	ENSG00000279996
1,01	0,93	0,98	ENSG00000006606
1,35	0,35	0,46	ENSG00000106178
0,89	0,17	0,26	ENSG00000005486
0,75	0,00	0,00	ENSG00000127948
1,80	0,00	0,00	ENSG00000189077
1,00	0,99	0,99	ENSG00000127952
1,06	0,72	0,81	ENSG00000280388
0,90	0,18	0,28	ENSG00000146701
1,14	0,17	0,27	ENSG00000177679
0,88	0,10	0,17	ENSG00000106211
0,95	0,49	0,60	ENSG00000170027
1,14	0,69	0,78	ENSG00000146700
0,18	0,00	0,00	ENSG00000188372
0,58	0,00	0,00	ENSG00000091073
0,27	0,00	0,00	ENSG00000243566
0,89	0,53	0,64	ENSG00000185040
0,95	0,68	0,77	ENSG00000146707
1,14	0,97	0,98	ENSG00000186645
1,43	0,14	0,23	ENSG00000205482
0,52	0,01	0,02	ENSG00000135205
1,32	0,44	0,56	ENSG00000127951
0,44	0,00	0,00	ENSG00000186088
0,94	0,45	0,56	ENSG00000127947
1,03	0,73	0,82	ENSG00000187257
1,04	0,73	0,82	ENSG00000135211
1,00	1,00	1,00	ENSG00000006576
0,93	0,61	0,71	ENSG00000187391
1,14	0,97	#N/D	ENSG00000281008
#N/D	#N/D	#N/D	ENSG00000280958

1,47	0,00	0,00	ENSG00000127955
1,69	0,24	0,34	ENSG00000135218
#N/D	#N/D	#N/D	ENSG00000214415
1,15	0,10	0,16	ENSG00000075223
#N/D	#N/D	#N/D	ENSG00000279326
2,88	0,00	0,00	ENSG00000019991
0,27	0,00	0,00	ENSG00000153956
0,27	0,00	0,00	ENSG00000186472
0,53	0,09	0,15	ENSG00000170381
#N/D	#N/D	#N/D	ENSG00000285398
1,66	0,00	0,00	ENSG00000075213
1,23	0,10	0,18	ENSG00000280325
13,67	0,00	0,00	ENSG00000153993
1,14	0,97	0,98	ENSG00000198822
1,22	0,27	0,38	ENSG00000164659
0,89	0,17	0,26	ENSG00000135164
1,46	0,00	0,00	ENSG00000135185
1,55	0,00	0,00	ENSG00000005469
2,73	0,00	0,00	ENSG00000005471
0,43	0,00	0,00	ENSG00000085563
0,68	0,10	0,16	ENSG00000105784
0,88	0,21	0,31	ENSG00000075303
0,63	0,00	0,00	ENSG00000006634
0,39	0,00	0,00	ENSG00000008277
1,02	0,78	0,86	ENSG00000075142
0,67	0,65	0,74	ENSG00000127954
1,14	0,97	0,98	ENSG00000182348
#N/D	#N/D	#N/D	ENSG00000164645
1,04	0,67	0,77	ENSG00000164647
1,63	0,00	0,00	ENSG00000157214
1,04	0,81	0,89	ENSG00000105792
1,44	0,68	0,77	ENSG00000283267
0,59	0,00	0,00	ENSG00000105793
1,14	0,97	#N/D	ENSG00000280440
0,64	0,00	0,00	ENSG00000157224
0,93	0,44	0,56	ENSG00000058091
1,14	0,97	0,98	ENSG00000251154
1,14	0,15	0,24	ENSG00000157240
0,88	0,29	0,40	ENSG00000127989
0,87	0,11	0,19	ENSG00000127914
1,51	0,00	0,00	ENSG00000001630
1,14	0,97	#N/D	ENSG00000285772
0,32	0,00	0,00	ENSG00000289027
0,99	1,00	1,00	ENSG00000240720
1,14	0,97	0,98	ENSG00000285953
0,72	0,00	0,00	ENSG00000001631
0,84	0,04	0,08	ENSG00000001629
0,83	0,03	0,07	ENSG00000157259

0,65	0,07	0,13	ENSG00000242950
0,49	0,00	0,00	ENSG00000127980
0,86	0,13	0,22	ENSG00000127993
0,81	0,07	0,13	ENSG00000234545
1,07	0,40	0,51	ENSG00000105810
0,55	0,00	0,00	ENSG00000205413
0,78	0,03	0,06	ENSG00000177409
15,64	0,33	0,44	ENSG00000188175
0,94	0,54	0,64	ENSG00000004766
1,14	0,97	0,98	ENSG00000004948
15,64	0,33	#N/D	ENSG00000127928
0,26	0,00	0,00	ENSG00000105825
1,41	0,01	0,02	ENSG00000127920
1,50	0,30	0,42	ENSG00000278959
1,27	0,01	0,02	ENSG00000105829
283,97	-	-	ENSG00000164692
0,51	0,00	0,00	ENSG00000127995
0,62	0,00	0,00	ENSG00000127990
1,08	0,78	0,86	ENSG00000242265
0,36	0,00	0,00	ENSG00000158528
0,07	0,31	0,43	ENSG00000005421
1,14	0,97	0,98	ENSG00000105852
0,64	0,00	0,00	ENSG00000105854
1,14	0,97	0,98	ENSG00000005981
1,14	0,97	0,98	ENSG00000279525
1,48	0,00	0,00	ENSG00000004799
1,97	0,00	0,01	ENSG00000158560
1,29	0,63	0,73	ENSG00000279067
0,46	0,00	0,00	ENSG00000004864
1,02	0,88	0,95	ENSG00000127922
12,13	0,01	0,02	ENSG00000006377
30,14	0,22	0,32	ENSG00000105880
1,40	0,01	0,03	ENSG00000196636
#N/D	#N/D	#N/D	ENSG00000006128
0,28	0,00	0,00	ENSG00000070669
#N/D	#N/D	#N/D	ENSG00000135175
0,90	0,22	0,32	ENSG00000164715
5,79	0,00	0,00	ENSG00000180535
1,09	0,33	0,44	ENSG00000205356
0,90	0,21	0,31	ENSG00000164713
1,04	0,66	0,75	ENSG00000006453
6,56	0,00	0,00	ENSG00000106236
4,08	0,00	0,00	ENSG00000166448
0,92	0,33	0,44	ENSG00000196367
1,06	0,47	0,57	ENSG00000198742
2,35	0,30	0,41	ENSG00000185467
1,34	0,01	0,01	ENSG00000284292
1,03	0,71	0,80	ENSG00000241685

0,98	0,81	0,89	ENSG00000130429
1,01	0,86	0,93	ENSG00000106244
1,08	0,40	0,51	ENSG00000106245
0,89	0,23	0,33	ENSG00000106246
0,55	0,00	0,00	ENSG00000248919
0,92	0,37	0,49	ENSG00000160917
0,90	0,20	0,30	ENSG00000241468
1,54	0,00	0,00	ENSG00000198556
1,45	0,00	0,00	ENSG00000160908
1,08	0,36	0,47	ENSG00000196652
0,92	0,56	0,66	ENSG00000221909
0,81	0,01	0,03	ENSG00000197343
59,15	0,14	0,22	ENSG00000272647
0,44	0,00	0,00	ENSG00000244219
0,87	0,15	0,23	ENSG00000197037
0,50	0,02	0,04	ENSG00000106258
0,27	0,22	0,32	ENSG00000282301
1,14	0,97	0,98	ENSG00000160870
1,14	0,97	0,98	ENSG00000160868
1,14	0,97	#N/D	ENSG00000021461
1,14	0,97	0,98	ENSG00000244623
1,58	0,00	0,00	ENSG00000146833
0,13	0,46	0,56	ENSG00000176402
0,18	0,10	0,16	ENSG00000160862
1,26	0,00	0,01	ENSG00000106261
0,84	0,13	0,22	ENSG00000166529
1,23	0,02	0,04	ENSG00000166526
0,95	0,53	0,64	ENSG00000168090
0,79	0,00	0,01	ENSG00000166508
0,91	0,33	0,44	ENSG00000221838
0,87	0,09	0,15	ENSG00000106290
1,02	0,83	0,90	ENSG00000166997
0,48	0,00	0,00	ENSG00000214309
1,21	0,03	0,07	ENSG00000188186
0,79	0,02	0,04	ENSG00000146826
3,51	0,00	0,00	ENSG00000197093
2,59	0,00	0,00	ENSG00000213420
1,35	0,05	0,10	ENSG00000066923
0,99	0,95	0,98	ENSG00000292277
0,02	0,13	0,21	ENSG00000213413
0,66	0,02	0,04	ENSG00000214300
0,74	0,00	0,00	ENSG00000121716
0,25	0,01	0,03	ENSG00000085514
0,28	0,01	0,02	ENSG00000289690
0,60	0,00	0,00	ENSG00000078487
1,03	0,68	0,77	ENSG00000146834
1,07	0,47	0,58	ENSG00000160813
2,66	0,00	0,00	ENSG00000185955

1,44	0,00	0,00	ENSG00000166925
#N/D	#N/D	#N/D	ENSG00000289760
0,43	0,00	0,00	ENSG00000166924
0,70	0,00	0,00	ENSG00000106351
1,44	0,02	0,04	ENSG00000205307
1,03	0,73	0,82	ENSG00000077454
1,51	0,02	0,04	ENSG00000106336
0,93	0,38	0,50	ENSG00000106333
1,68	0,00	0,00	ENSG00000106330
0,27	0,00	0,00	ENSG00000106327
1,14	0,97	#N/D	ENSG00000077080
1,54	0,00	0,00	ENSG00000172354
1,10	0,22	0,32	ENSG00000146830
1,27	0,01	0,01	ENSG00000172336
1,29	0,74	0,82	ENSG00000130427
0,34	0,18	0,27	ENSG00000146839
1,09	0,27	0,38	ENSG00000196411
1,16	0,12	0,19	ENSG00000146828
1,25	0,01	0,01	ENSG00000087077
1,15	0,09	0,16	ENSG00000087087
0,75	0,15	0,24	ENSG00000176125
0,31	0,00	0,00	ENSG00000087085
0,05	0,24	0,35	ENSG00000169894
0,36	0,35	0,46	ENSG00000205277
1,14	0,97	#N/D	ENSG00000169876
1,33	0,00	0,00	ENSG00000169871
0,65	0,00	0,00	ENSG00000106366
1,22	0,02	0,03	ENSG00000106367
0,33	0,00	0,00	ENSG00000128564
0,75	0,10	0,16	ENSG00000167011
8,39	0,46	0,56	ENSG00000106384
1,10	0,23	0,33	ENSG00000106397
1,47	0,00	0,00	ENSG00000106400
0,84	0,07	0,13	ENSG00000106404
1,15	0,10	0,17	ENSG00000214253
1,10	0,36	0,47	ENSG00000128581
1,89	0,45	0,56	ENSG00000160963
8,39	0,46	#N/D	ENSG00000106436
1,04	0,65	0,74	ENSG00000257923
0,37	0,00	0,00	ENSG00000160999
1,33	0,36	0,47	ENSG00000260097
1,44	0,00	0,00	ENSG00000128563
1,67	0,00	0,00	ENSG00000160991
0,80	0,04	0,08	ENSG00000160993
0,75	0,00	0,01	ENSG00000161036
1,11	0,27	0,37	ENSG00000005075
3,27	0,00	0,00	ENSG00000170667
1,35	0,00	0,00	ENSG00000168255

#N/D	#N/D	#N/D	ENSG00000284981
6,52	0,00	0,00	ENSG00000270249
0,94	0,54	0,64	ENSG00000205238
1,13	0,15	0,24	ENSG00000285437
3,18	0,00	0,00	ENSG00000105808
1,40	0,00	0,01	ENSG00000205236
1,24	0,01	0,02	ENSG00000267368
1,07	0,48	0,58	ENSG00000267645
1,04	0,82	0,89	ENSG00000173678
1,34	0,00	0,01	ENSG00000228049
0,96	0,77	0,85	ENSG00000222011
0,77	0,33	0,44	ENSG00000161040
0,21	0,04	0,07	ENSG00000128606
0,85	0,07	0,12	ENSG00000170632
1,08	0,42	0,54	ENSG00000161048
1,91	0,35	0,47	ENSG00000279482
0,78	0,00	0,01	ENSG00000105819
1,03	0,73	0,81	ENSG00000105821
0,88	0,13	0,21	ENSG00000161057
1,93	0,16	0,25	ENSG00000170615
16,00	0,00	0,00	ENSG00000189056
0,40	0,00	0,00	ENSG00000164815
1,14	0,97	#N/D	ENSG00000187416
1,15	0,09	0,16	ENSG00000005483
#N/D	#N/D	#N/D	ENSG00000289360
#N/D	#N/D	#N/D	ENSG00000288914
0,75	0,00	0,00	ENSG00000135250
0,49	0,00	0,00	ENSG00000091127
1,50	0,00	0,00	ENSG00000135249
2,34	0,02	0,03	ENSG00000185055
0,69	0,00	0,00	ENSG00000146776
0,60	0,12	0,19	ENSG00000128536
#N/D	#N/D	#N/D	ENSG00000280439
1,08	0,39	0,50	ENSG00000008282
0,45	0,00	0,00	ENSG00000105835
0,84	0,06	0,11	ENSG00000253276
8,39	0,46	0,56	ENSG00000105851
0,35	0,00	0,00	ENSG00000005249
1,66	0,00	0,00	ENSG00000105856
0,72	0,00	0,00	ENSG00000164597
0,97	0,95	0,98	ENSG00000172209
0,26	0,00	0,00	ENSG00000105865
0,86	0,12	0,19	ENSG00000288558
0,51	0,00	0,00	ENSG00000075790
0,59	0,01	0,02	ENSG00000091137
0,90	0,26	0,36	ENSG00000105879
0,13	0,46	#N/D	ENSG00000091138
0,82	0,02	0,04	ENSG00000091140

1,32	0,00	0,00	ENSG00000091136
3,66	0,00	0,00	ENSG00000091128
0,08	0,00	0,00	ENSG00000091129
0,92	0,36	0,47	ENSG00000135241
0,91	0,32	0,43	ENSG00000177683
2,07	0,00	0,00	ENSG00000128590
0,13	0,46	0,56	ENSG00000279043
0,75	0,03	0,06	ENSG00000184903
0,09	0,01	0,03	ENSG00000173114
0,69	0,00	0,00	ENSG00000128512
0,86	0,14	0,22	ENSG00000198839
0,49	0,00	0,00	ENSG00000006652
0,93	0,58	0,68	ENSG00000288640
0,97	0,96	0,98	ENSG00000288634
0,33	0,06	0,10	ENSG00000181016
0,74	0,00	0,01	ENSG00000146802
1,04	0,81	0,88	ENSG00000164603
0,51	0,42	0,54	ENSG00000279288
0,42	0,00	0,00	ENSG00000164604
1,17	0,08	0,13	ENSG00000214194
#N/D	#N/D	#N/D	ENSG00000278894
#N/D	#N/D	#N/D	ENSG00000154415
0,44	0,00	0,01	ENSG00000128573
0,95	0,55	0,65	ENSG00000135272
1,14	0,97	#N/D	ENSG00000105967
1,31	0,22	0,33	ENSG00000279086
1,00	0,97	0,99	ENSG00000135269
3,16	0,00	0,00	ENSG00000105971
3,03	0,00	0,00	ENSG00000105974
0,63	0,00	0,00	ENSG00000105976
0,74	0,00	0,00	ENSG00000198898
0,67	0,00	0,00	ENSG00000004866
0,51	0,42	0,54	ENSG00000288635
22,89	0,26	#N/D	ENSG00000105989
1,14	0,97	#N/D	ENSG00000001626
#N/D	#N/D	#N/D	ENSG00000154438
0,15	0,00	0,00	ENSG00000077063
0,88	0,18	0,27	ENSG00000128534
1,14	0,97	0,98	ENSG00000106013
0,01	0,06	0,11	ENSG00000184408
0,83	0,14	0,22	ENSG00000106025
0,58	0,00	0,00	ENSG00000071243
2,57	0,00	0,00	ENSG00000106034
5,06	0,00	0,00	ENSG00000002745
1,28	0,01	0,01	ENSG00000196937
0,27	0,22	0,32	ENSG00000106278
0,56	0,00	0,00	ENSG00000008311
1,14	0,97	0,98	ENSG00000128610

5,14	0,00	0,00	ENSG00000081803
1,14	0,97	0,98	ENSG00000188050
15,64	0,33	0,44	ENSG00000235631
#N/D	#N/D	#N/D	ENSG00000128519
0,99	1,00	1,00	ENSG00000081800
2,87	0,04	0,08	ENSG00000164675
0,86	0,22	0,32	ENSG00000128609
8,39	0,46	#N/D	ENSG00000146809
0,13	0,46	0,56	ENSG00000170807
1,00	0,98	0,99	ENSG00000106299
1,14	0,97	0,98	ENSG00000106302
#N/D	#N/D	#N/D	ENSG00000106304
#N/D	#N/D	#N/D	ENSG00000234224
#N/D	#N/D	#N/D	ENSG00000230268
0,05	0,24	0,35	ENSG00000279419
1,54	0,00	0,00	ENSG00000170775
0,82	0,03	0,06	ENSG00000128513
8,39	0,46	0,56	ENSG00000179603
0,84	0,07	0,13	ENSG00000048405
0,85	0,60	0,70	ENSG00000279265
1,20	0,08	0,14	ENSG00000280347
1,13	0,17	0,26	ENSG00000179562
0,83	0,02	0,04	ENSG00000004059
1,85	0,60	0,69	ENSG00000106328
0,13	0,46	#N/D	ENSG00000106331
1,31	0,00	0,00	ENSG00000197157
2,11	0,00	0,00	ENSG00000128594
2,72	0,04	0,07	ENSG00000174697
0,90	0,20	0,30	ENSG00000106344
1,73	0,32	0,44	ENSG00000224940
1,31	0,00	0,00	ENSG00000106348
3,23	0,00	0,00	ENSG00000135245
0,89	0,18	0,27	ENSG00000165055
0,20	0,00	0,00	ENSG00000205085
0,07	0,31	0,43	ENSG00000135248
1,28	0,00	0,01	ENSG00000128595
0,65	0,26	0,37	ENSG00000128617
0,84	0,36	0,47	ENSG00000128596
1,71	0,00	0,00	ENSG00000128591
0,07	0,31	0,43	ENSG00000135253
1,14	0,11	0,18	ENSG00000128524
1,59	0,03	0,06	ENSG00000272899
0,53	0,06	0,11	ENSG00000128604
2,87	0,04	0,08	ENSG00000275106
0,95	0,50	0,60	ENSG00000064419
1,74	0,00	0,00	ENSG00000158457
0,74	0,00	0,00	ENSG00000128602
1,03	0,77	0,85	ENSG00000158467

0,50	0,00	0,00	ENSG00000128578
0,14	0,00	0,00	ENSG00000240204
0,70	0,00	0,00	ENSG00000106459
1,35	0,00	0,00	ENSG00000186591
0,79	0,01	0,02	ENSG00000091732
1,66	0,00	0,00	ENSG00000128607
0,69	0,00	0,00	ENSG00000146842
1,14	0,97	#N/D	ENSG00000165120
1,14	0,97	0,98	ENSG00000158516
0,59	0,00	0,00	ENSG00000128510
0,22	0,14	0,22	ENSG00000158525
0,07	0,31	0,43	ENSG00000091704
0,78	0,01	0,01	ENSG00000106477
0,84	0,75	0,83	ENSG00000106484
0,85	0,05	0,10	ENSG00000158623
1,14	0,97	#N/D	ENSG00000213265
0,02	0,12	0,19	ENSG00000266265
0,89	0,18	0,27	ENSG00000128585
0,43	0,00	0,00	ENSG00000128567
4,62	0,00	0,00	ENSG00000221866
0,62	0,00	0,00	ENSG00000106554
1,05	0,53	0,63	ENSG00000131558
1,08	0,87	0,93	ENSG00000155530
0,75	0,00	0,00	ENSG00000205060
0,85	0,04	0,08	ENSG00000085662
8,51	0,00	0,00	ENSG00000198074
1,17	0,55	0,66	ENSG00000227471
0,78	0,01	0,03	ENSG00000172331
0,45	0,00	0,00	ENSG00000122786
1,61	0,26	0,37	ENSG00000146856
0,62	0,00	0,00	ENSG00000122783
0,76	0,00	0,01	ENSG00000146859
1,39	0,00	0,00	ENSG00000105875
#N/D	#N/D	#N/D	ENSG00000146857
0,93	0,43	0,55	ENSG00000080802
0,78	0,00	0,01	ENSG00000155561
1,06	0,49	0,60	ENSG00000243317
0,89	0,65	0,74	ENSG00000164707
0,51	0,00	0,00	ENSG00000189320
1,13	0,14	0,22	ENSG00000105887
3,56	0,23	0,34	ENSG00000181072
0,91	0,56	0,66	ENSG00000105894
0,43	0,00	0,01	ENSG00000157680
1,25	0,01	0,01	ENSG00000182158
#N/D	#N/D	#N/D	ENSG00000122787
0,96	0,69	0,78	ENSG00000122779
1,14	0,97	0,98	ENSG00000157703
0,18	0,10	0,16	ENSG00000105929

51,90	0,15	0,24	ENSG00000214128
0,43	0,00	0,00	ENSG00000122778
1,10	0,34	0,45	ENSG00000146858
2,22	0,00	0,00	ENSG00000105939
0,77	0,02	0,03	ENSG00000105948
1,23	0,02	0,03	ENSG00000157741
2,68	0,00	0,00	ENSG00000164898
0,93	0,42	0,54	ENSG00000146963
1,43	0,00	0,00	ENSG00000269955
1,14	0,97	0,98	ENSG00000188883
5,08	0,03	0,06	ENSG00000236279
1,65	0,00	0,00	ENSG00000064393
3,21	0,00	0,00	ENSG00000059377
0,66	0,00	0,00	ENSG00000059378
0,40	0,00	0,00	ENSG00000006459
1,26	0,01	0,02	ENSG00000157800
2,71	0,36	0,48	ENSG00000146955
1,08	0,37	0,48	ENSG00000133606
0,74	0,00	0,00	ENSG00000146966
0,73	0,00	0,00	ENSG00000133597
1,17	0,08	0,13	ENSG00000090266
1,10	0,27	0,38	ENSG00000157764
0,58	0,00	0,00	ENSG00000090263
0,84	0,75	0,83	ENSG00000261115
0,81	0,02	0,04	ENSG00000006530
0,72	0,00	0,00	ENSG00000257093
0,53	0,58	0,68	ENSG00000214102
0,76	0,00	0,01	ENSG00000106028
0,74	0,34	0,45	ENSG00000127362
1,00	1,00	1,00	ENSG00000127364
0,52	0,04	0,08	ENSG00000127366
3,56	0,23	0,34	ENSG00000165076
1,14	0,97	0,98	ENSG00000237621
0,03	0,17	0,26	ENSG00000257335
#N/D	#N/D	#N/D	ENSG00000258083
0,13	0,46	0,56	ENSG00000258227
#N/D	#N/D	#N/D	ENSG00000257138
0,07	0,31	0,43	ENSG00000257743
#N/D	#N/D	#N/D	ENSG00000258223
#N/D	#N/D	#N/D	ENSG00000226660
#N/D	#N/D	#N/D	ENSG00000237702
#N/D	#N/D	#N/D	ENSG00000211710
#N/D	#N/D	#N/D	ENSG00000211734
#N/D	#N/D	#N/D	ENSG00000211706
#N/D	#N/D	#N/D	ENSG00000211707
#N/D	#N/D	#N/D	ENSG00000211745
#N/D	#N/D	#N/D	ENSG00000283063
8,39	0,46	#N/D	ENSG00000282939

#N/D	#N/D	#N/D	ENSG00000211713
1,85	0,60	0,69	ENSG00000211714
1,14	0,97	#N/D	ENSG00000211715
#N/D	#N/D	#N/D	ENSG00000211716
#N/D	#N/D	#N/D	ENSG00000211717
#N/D	#N/D	#N/D	ENSG00000211720
#N/D	#N/D	#N/D	ENSG00000229769
1,14	0,97	0,98	ENSG00000211721
1,14	0,97	#N/D	ENSG00000253409
1,14	0,97	0,98	ENSG00000230099
1,14	0,97	#N/D	ENSG00000211724
1,14	0,97	#N/D	ENSG00000211725
#N/D	#N/D	#N/D	ENSG00000253188
#N/D	#N/D	#N/D	ENSG00000211727
1,14	0,97	#N/D	ENSG00000211728
#N/D	#N/D	#N/D	ENSG00000253534
1,14	0,97	#N/D	ENSG00000253291
#N/D	#N/D	#N/D	ENSG00000211731
1,14	0,97	0,98	ENSG00000278030
1,14	0,97	0,98	ENSG00000276405
1,14	0,97	0,98	ENSG00000275791
0,27	0,22	0,32	ENSG00000276597
37,39	0,19	0,29	ENSG00000274752
2,86	0,07	0,12	ENSG00000276953
1,14	0,97	0,98	ENSG00000275158
1,14	0,97	0,98	ENSG00000275743
#N/D	#N/D	#N/D	ENSG00000276819
#N/D	#N/D	#N/D	ENSG00000275243
#N/D	#N/D	#N/D	ENSG00000277880
#N/D	#N/D	#N/D	ENSG00000276557
#N/D	#N/D	#N/D	ENSG00000211746
#N/D	#N/D	#N/D	ENSG00000211747
#N/D	#N/D	#N/D	ENSG00000211749
#N/D	#N/D	#N/D	ENSG00000211750
1,14	0,97	#N/D	ENSG00000282499
#N/D	#N/D	#N/D	ENSG00000211752
#N/D	#N/D	#N/D	ENSG00000211753
#N/D	#N/D	#N/D	ENSG00000232869
1,14	0,97	0,98	ENSG00000204983
1,14	0,97	0,98	ENSG00000275896
#N/D	#N/D	#N/D	ENSG00000282431
#N/D	#N/D	#N/D	ENSG00000282320
#N/D	#N/D	#N/D	ENSG00000282420
#N/D	#N/D	#N/D	ENSG00000282133
#N/D	#N/D	#N/D	ENSG00000281958
#N/D	#N/D	#N/D	ENSG00000282173
#N/D	#N/D	#N/D	ENSG00000282780
1,14	0,97	0,98	ENSG00000211751

#N/D	#N/D	#N/D	ENSG00000292271
1,14	0,97	0,98	ENSG00000211764
#N/D	#N/D	#N/D	ENSG00000211765
#N/D	#N/D	#N/D	ENSG00000211766
1,14	0,97	#N/D	ENSG00000211767
#N/D	#N/D	#N/D	ENSG00000211768
#N/D	#N/D	#N/D	ENSG00000211769
#N/D	#N/D	#N/D	ENSG00000211770
#N/D	#N/D	#N/D	ENSG00000211771
1,14	0,97	0,98	ENSG00000211772
1,14	0,97	0,98	ENSG00000237254
7,38	0,00	0,00	ENSG00000106123
1,14	0,97	#N/D	ENSG00000165125
#N/D	#N/D	#N/D	ENSG00000127412
0,07	0,31	#N/D	ENSG00000165131
1,93	0,16	0,25	ENSG00000197993
22,89	0,26	0,37	ENSG00000179468
0,13	0,46	0,56	ENSG00000225781
#N/D	#N/D	#N/D	ENSG00000159763
0,03	0,17	0,26	ENSG00000236398
0,13	0,46	#N/D	ENSG00000221937
#N/D	#N/D	#N/D	ENSG00000279223
0,83	0,05	0,09	ENSG00000197448
1,39	0,24	0,35	ENSG00000178826
1,27	0,01	0,01	ENSG00000106144
1,14	0,97	0,98	ENSG00000188037
1,35	0,01	0,02	ENSG00000159784
0,96	0,63	0,73	ENSG00000159840
2,61	0,03	0,06	ENSG00000146904
8,39	0,46	0,56	ENSG00000185899
1,14	0,97	0,98	ENSG00000221855
1,14	0,97	0,98	ENSG00000176510
2,02	0,06	0,11	ENSG00000271079
4,49	0,00	0,00	ENSG00000170379
3,41	0,00	0,00	ENSG00000283528
0,07	0,31	0,43	ENSG00000271321
1,29	0,00	0,01	ENSG00000198420
1,14	0,97	#N/D	ENSG00000221910
0,13	0,46	0,56	ENSG00000213215
8,39	0,46	#N/D	ENSG00000221813
9,56	0,02	0,05	ENSG00000221836
15,64	0,33	0,44	ENSG00000221933
0,53	0,58	0,68	ENSG00000221858
1,14	0,97	#N/D	ENSG00000221989
0,99	1,00	#N/D	ENSG00000221938
0,28	0,00	0,00	ENSG00000288784
1,00	0,99	1,00	ENSG00000213214
0,51	0,04	0,08	ENSG00000212807

0,56	0,01	0,03	ENSG00000243896
7,43	0,00	0,00	ENSG00000289604
0,84	0,54	0,64	ENSG00000221970
0,98	0,78	0,86	ENSG00000050327
1,14	0,97	#N/D	ENSG00000106410
0,80	0,17	0,26	ENSG00000196511
#N/D	#N/D	#N/D	ENSG00000280144
2,71	0,36	0,48	ENSG00000174469
1,14	0,97	0,98	ENSG00000170279
0,73	0,00	0,00	ENSG00000055130
0,77	0,00	0,01	ENSG00000106462
2,05	0,00	0,00	ENSG00000155660
0,52	0,00	0,00	ENSG00000197362
0,83	0,34	0,45	ENSG00000204947
1,07	0,43	0,55	ENSG00000197024
0,98	0,85	0,92	ENSG00000170265
1,29	0,01	0,01	ENSG00000170260
0,90	0,19	0,29	ENSG00000204946
0,82	0,03	0,05	ENSG00000196453
0,96	0,63	0,73	ENSG00000181220
0,83	0,05	0,09	ENSG00000133619
0,21	0,00	0,00	ENSG00000181444
1,29	0,00	0,01	ENSG00000106479
1,20	0,51	0,62	ENSG00000280149
0,52	0,00	0,00	ENSG00000171130
1,14	0,97	0,98	ENSG00000279536
1,85	0,60	0,69	ENSG00000106526
0,07	0,31	0,43	ENSG00000127399
1,14	0,97	0,98	ENSG00000106538
0,57	0,00	0,00	ENSG00000214022
0,97	0,79	0,86	ENSG00000196456
1,10	0,38	0,50	ENSG00000284691
0,67	0,65	0,74	ENSG00000171115
#N/D	#N/D	#N/D	ENSG00000179144
0,23	0,05	0,10	ENSG00000133574
1,14	0,97	#N/D	ENSG00000133561
0,45	0,01	0,02	ENSG00000106560
22,89	0,26	0,37	ENSG00000213203
0,05	0,24	#N/D	ENSG00000281887
8,39	0,46	0,56	ENSG00000196329
0,13	0,46	#N/D	ENSG00000106565
#N/D	#N/D	#N/D	ENSG00000002933
#N/D	#N/D	#N/D	ENSG00000002726
2,88	0,02	0,03	ENSG00000055118
1,91	0,35	0,47	ENSG00000164867
0,66	0,42	0,54	ENSG00000181652
1,18	0,05	0,09	ENSG00000197150
0,92	0,44	0,56	ENSG00000213199

1,25	0,02	0,04	ENSG00000164885
0,90	0,18	0,27	ENSG00000164889
#N/D	#N/D	#N/D	ENSG00000288608
1,25	0,01	0,02	ENSG00000164896
1,10	0,27	0,37	ENSG00000164897
1,24	0,01	0,02	ENSG00000133612
0,69	0,45	0,56	ENSG00000164900
0,13	0,46	#N/D	ENSG00000146926
0,36	0,35	0,46	ENSG00000278685
0,98	0,98	0,99	ENSG00000285480
0,89	0,23	0,33	ENSG00000285292
0,86	0,07	0,13	ENSG00000033050
1,48	0,00	0,00	ENSG00000033100
1,20	0,05	0,09	ENSG00000082014
0,88	0,13	0,21	ENSG00000013374
0,57	0,36	0,47	ENSG00000187260
4,42	0,15	0,24	ENSG00000127377
0,72	0,00	0,00	ENSG00000106615
0,62	0,00	0,00	ENSG00000106617
#N/D	#N/D	#N/D	ENSG00000106648
1,22	0,02	0,04	ENSG00000178234
1,21	0,02	0,04	ENSG00000055609
0,69	0,00	0,00	ENSG00000196584
0,73	0,01	0,02	ENSG00000133627
8,39	0,46	0,56	ENSG00000130226
0,72	0,00	0,00	ENSG00000157212
#N/D	#N/D	#N/D	ENSG00000157219
0,40	0,01	0,03	ENSG00000278998
6,47	0,00	0,00	ENSG00000186480
0,93	0,54	0,64	ENSG00000164778
8,39	0,46	#N/D	ENSG00000146910
1,84	0,04	0,07	ENSG00000283128
1,07	0,43	0,54	ENSG00000184863
0,05	0,24	0,35	ENSG00000164690
0,73	0,02	0,04	ENSG00000105982
0,70	0,00	0,00	ENSG00000105983
0,94	0,50	0,60	ENSG00000146909
0,44	0,00	0,00	ENSG00000130675
0,93	0,37	0,49	ENSG00000009335
0,86	0,06	0,11	ENSG00000105993
3,26	0,13	0,21	ENSG00000280266
0,94	0,56	0,66	ENSG00000155093
1,06	0,46	0,56	ENSG00000146918
1,40	0,00	0,00	ENSG00000117868
0,92	0,43	0,55	ENSG00000126870
1,14	0,97	#N/D	ENSG00000106018
2,71	0,36	0,48	ENSG00000176269
1,21	0,25	0,36	ENSG00000172748

0,79	0,01	0,02	ENSG00000147364
5,21	0,00	0,01	ENSG00000279852
3,90	0,00	0,00	ENSG00000180190
1,22	0,04	0,08	ENSG00000104714
0,26	0,08	0,14	ENSG00000198010
1,89	0,00	0,00	ENSG00000182372
3,20	0,00	0,00	ENSG00000283239
1,35	0,00	0,00	ENSG00000104728
1,15	0,12	0,20	ENSG00000176595
1,69	0,06	0,10	ENSG00000036448
0,13	0,46	#N/D	ENSG00000183117
1,05	0,56	0,66	ENSG00000147316
0,92	0,69	0,78	ENSG00000091879
0,77	0,00	0,00	ENSG00000155189
1,88	0,00	0,00	ENSG00000275591
#N/D	#N/D	#N/D	ENSG00000164825
#N/D	#N/D	#N/D	ENSG00000164822
#N/D	#N/D	#N/D	ENSG00000164821
#N/D	#N/D	#N/D	ENSG00000206047
#N/D	#N/D	#N/D	ENSG00000240247
#N/D	#N/D	#N/D	ENSG00000239839
#N/D	#N/D	#N/D	ENSG00000164816
8,39	0,46	0,56	ENSG00000230045
#N/D	#N/D	#N/D	ENSG00000233132
8,39	0,46	#N/D	ENSG00000223885
22,89	0,26	0,37	ENSG00000215373
3,26	0,13	0,21	ENSG00000233295
22,89	0,26	0,37	ENSG00000206034
0,66	0,51	0,62	ENSG00000230549
1,14	0,97	#N/D	ENSG00000236125
1,14	0,97	#N/D	ENSG00000215372
#N/D	#N/D	#N/D	ENSG00000215371
#N/D	#N/D	#N/D	ENSG00000177257
#N/D	#N/D	#N/D	ENSG00000177243
#N/D	#N/D	#N/D	ENSG00000164871
#N/D	#N/D	#N/D	ENSG00000177023
#N/D	#N/D	#N/D	ENSG00000187082
#N/D	#N/D	#N/D	ENSG00000186599
#N/D	#N/D	#N/D	ENSG00000198129
1,14	0,97	#N/D	ENSG00000255251
#N/D	#N/D	#N/D	ENSG00000285975
#N/D	#N/D	#N/D	ENSG00000285687
15,64	0,33	#N/D	ENSG00000285765
#N/D	#N/D	#N/D	ENSG00000285814
#N/D	#N/D	#N/D	ENSG00000285913
#N/D	#N/D	#N/D	ENSG00000285620
#N/D	#N/D	#N/D	ENSG00000285937
#N/D	#N/D	#N/D	ENSG00000285720

#N/D	#N/D	#N/D	ENSG00000285657
#N/D	#N/D	#N/D	ENSG00000285607
0,73	0,49	0,59	ENSG00000285950
#N/D	#N/D	#N/D	ENSG00000255378
#N/D	#N/D	#N/D	ENSG00000186572
1,14	0,97	#N/D	ENSG00000186562
#N/D	#N/D	#N/D	ENSG00000186579
#N/D	#N/D	#N/D	ENSG00000176782
#N/D	#N/D	#N/D	ENSG00000178287
#N/D	#N/D	#N/D	ENSG00000176797
#N/D	#N/D	#N/D	ENSG00000171711
#N/D	#N/D	#N/D	ENSG00000215356
#N/D	#N/D	#N/D	ENSG00000237038
1,14	0,97	#N/D	ENSG00000225327
8,39	0,46	0,56	ENSG00000205989
51,90	0,15	0,24	ENSG00000233115
1,14	0,97	#N/D	ENSG00000215354
#N/D	#N/D	#N/D	ENSG00000254229
0,46	0,00	0,00	ENSG00000275342
0,98	0,92	0,98	ENSG00000253958
1,04	0,59	0,69	ENSG00000147324
1,11	0,23	0,33	ENSG00000104626
1,11	0,22	0,32	ENSG00000173281
0,88	0,11	0,18	ENSG00000173273
1,16	0,14	0,22	ENSG00000175806
1,89	0,45	0,56	ENSG00000253649
1,14	0,97	0,98	ENSG00000184647
0,26	0,00	0,00	ENSG00000183638
0,15	0,01	0,02	ENSG00000171060
0,32	0,00	0,00	ENSG00000171056
0,18	0,00	0,00	ENSG00000258724
0,85	0,16	0,25	ENSG00000254093
0,99	1,00	1,00	ENSG00000280294
1,25	0,10	0,17	ENSG00000171044
1,03	0,71	0,79	ENSG00000104643
2,88	0,03	0,05	ENSG00000177710
0,21	0,00	0,00	ENSG00000154319
0,14	0,05	0,09	ENSG00000136573
18,75	0,00	0,00	ENSG00000136574
0,44	0,00	0,00	ENSG00000154328
2,31	0,00	0,00	ENSG00000079459
1,39	0,00	0,00	ENSG00000164733
#N/D	#N/D	#N/D	ENSG00000205884
#N/D	#N/D	#N/D	ENSG00000205883
#N/D	#N/D	#N/D	ENSG00000205882
1,14	0,97	#N/D	ENSG00000233050
1,14	0,97	0,98	ENSG00000215343
0,21	0,00	0,00	ENSG00000226430

0,11	0,00	0,00	ENSG00000223443
8,39	0,46	0,56	ENSG00000254866
0,72	0,00	0,00	ENSG00000186523
#N/D	#N/D	#N/D	ENSG00000232948
0,56	0,00	0,00	ENSG00000145002
0,57	0,00	0,00	ENSG00000154359
0,31	0,00	0,00	ENSG00000250305
0,52	0,00	0,00	ENSG00000164741
0,97	0,93	0,98	ENSG00000164743
1,73	0,32	0,44	ENSG00000185053
0,85	0,05	0,09	ENSG00000104723
0,62	0,10	0,17	ENSG00000038945
0,13	0,46	0,56	ENSG00000078579
0,69	0,06	0,11	ENSG00000155970
0,56	0,00	0,00	ENSG00000104219
0,78	0,00	0,01	ENSG00000198791
0,70	0,00	0,00	ENSG00000155975
1,17	0,44	0,56	ENSG00000003987
0,22	0,00	0,00	ENSG00000003989
0,02	0,10	0,17	ENSG00000279271
8,39	0,46	#N/D	ENSG00000280453
0,13	0,46	0,56	ENSG00000279903
0,23	0,00	0,00	ENSG00000104213
0,36	0,00	0,00	ENSG00000129422
1,14	0,97	#N/D	ENSG00000104760
0,77	0,00	0,00	ENSG00000078674
0,58	0,00	0,00	ENSG00000104763
0,56	0,01	0,03	ENSG00000171428
0,13	0,46	0,56	ENSG00000156006
0,85	0,04	0,08	ENSG00000156011
1,44	0,20	0,30	ENSG00000278886
#N/D	#N/D	#N/D	ENSG00000279919
0,45	0,00	0,00	ENSG00000104611
8,39	0,46	0,56	ENSG00000279858
0,09	0,00	0,00	ENSG00000147408
0,96	0,62	0,71	ENSG00000104613
0,07	0,31	#N/D	ENSG00000175445
#N/D	#N/D	#N/D	ENSG00000036565
0,81	0,01	0,02	ENSG00000147416
4,22	0,00	0,00	ENSG00000061337
#N/D	#N/D	#N/D	ENSG00000280215
20,21	0,00	0,00	ENSG00000168546
0,13	0,46	0,56	ENSG00000147443
1,09	0,26	0,37	ENSG00000130227
22,89	0,26	0,37	ENSG00000158806
0,74	0,69	0,78	ENSG00000158815
0,56	0,00	0,00	ENSG00000158856
1,12	0,18	0,27	ENSG00000158863

1,11	0,26	0,37	ENSG00000275074
0,28	0,00	0,00	ENSG00000168453
#N/D	#N/D	#N/D	ENSG00000288677
0,94	0,44	0,56	ENSG00000168476
0,84	0,65	0,75	ENSG00000168481
0,22	0,14	0,22	ENSG00000168484
0,72	0,00	0,00	ENSG00000168487
0,47	0,00	0,00	ENSG00000168490
2,06	0,00	0,00	ENSG00000168495
0,63	0,01	0,03	ENSG00000197181
0,80	0,00	0,01	ENSG00000104635
0,72	0,00	0,00	ENSG00000120910
0,87	0,07	0,13	ENSG00000120896
1,11	0,21	0,32	ENSG00000120913
1,43	0,12	0,20	ENSG00000248235
1,02	0,87	0,93	ENSG00000241852
1,08	0,34	0,46	ENSG00000158941
1,08	0,40	0,52	ENSG00000147439
0,34	0,00	0,01	ENSG00000179388
1,85	0,60	0,69	ENSG00000134020
1,03	0,71	0,80	ENSG00000008853
1,07	0,37	0,48	ENSG00000120889
0,79	0,13	0,21	ENSG00000284956
0,64	0,00	0,01	ENSG00000173535
1,45	0,56	0,67	ENSG00000173530
0,43	0,00	0,00	ENSG00000104689
0,87	0,09	0,15	ENSG00000147457
0,63	0,00	0,00	ENSG00000104679
2,17	0,00	0,00	ENSG00000134013
0,71	0,00	0,00	ENSG00000197217
0,96	0,58	0,68	ENSG00000147454
0,18	0,00	0,00	ENSG00000167034
1,14	0,97	#N/D	ENSG00000180053
0,31	0,00	0,00	ENSG00000159167
1,72	0,20	0,30	ENSG00000042980
1,14	0,97	#N/D	ENSG00000134028
1,14	0,97	#N/D	ENSG00000069206
1,57	0,32	0,43	ENSG00000104722
0,44	0,02	0,04	ENSG00000277586
0,84	0,02	0,05	ENSG00000147459
1,41	0,01	0,03	ENSG00000147437
0,97	0,74	0,82	ENSG00000104756
1,01	0,92	0,98	ENSG00000184661
2,84	0,11	0,19	ENSG00000221818
0,73	0,00	0,00	ENSG00000221914
1,73	0,00	0,00	ENSG00000104765
0,34	0,00	0,00	ENSG00000240694
1,37	0,00	0,00	ENSG00000092964

8,39	0,46	0,56	ENSG00000120907
0,99	1,00	1,00	ENSG00000015592
1,08	0,34	0,45	ENSG00000104228
0,87	0,24	0,34	ENSG00000120899
0,99	1,00	1,00	ENSG00000120903
1,10	0,38	0,50	ENSG00000120915
0,44	0,00	0,00	ENSG00000120885
1,16	0,07	0,12	ENSG00000168077
1,53	0,04	0,07	ENSG00000279302
0,61	0,00	0,00	ENSG00000147419
0,54	0,00	0,00	ENSG00000171320
0,52	0,00	0,00	ENSG00000168078
0,14	0,00	0,00	ENSG00000168079
0,13	0,46	0,56	ENSG00000189233
0,85	0,06	0,11	ENSG00000134014
8,39	0,46	0,56	ENSG00000168081
1,66	0,00	0,00	ENSG00000186918
0,53	0,00	0,00	ENSG00000214050
0,16	0,00	0,01	ENSG00000104290
0,87	0,09	0,16	ENSG00000012232
0,52	0,00	0,00	ENSG00000104299
1,08	0,36	0,48	ENSG00000147421
0,44	0,00	0,00	ENSG00000197892
0,89	0,14	0,22	ENSG00000120875
0,82	0,74	0,82	ENSG00000280147
0,85	0,04	0,08	ENSG00000133872
0,66	0,00	0,00	ENSG00000104660
0,97	0,95	0,98	ENSG00000177669
0,79	0,01	0,02	ENSG00000104671
0,58	0,00	0,00	ENSG00000157110
0,49	0,00	0,00	ENSG00000279041
0,94	0,50	0,60	ENSG00000197265
1,68	0,40	0,52	ENSG00000253457
1,07	0,42	0,54	ENSG00000104687
1,15	0,16	0,25	ENSG00000104691
0,84	0,03	0,06	ENSG00000104695
0,05	0,24	0,35	ENSG00000133863
0,61	0,23	0,33	ENSG00000172733
0,50	0,00	0,00	ENSG00000165392
0,14	0,00	0,00	ENSG00000157168
#N/D	#N/D	#N/D	ENSG00000286131
0,79	0,01	0,03	ENSG00000172728
0,93	0,42	0,54	ENSG00000129696
0,66	0,00	0,00	ENSG00000198042
0,80	0,03	0,05	ENSG00000133874
22,89	0,26	0,37	ENSG00000133878
1,14	0,97	0,98	ENSG00000156687
#N/D	#N/D	#N/D	ENSG00000279099

1,14	0,97	0,98	ENSG00000215262
0,89	0,24	0,35	ENSG00000183779
1,11	0,21	0,31	ENSG00000147475
0,82	0,02	0,05	ENSG00000147471
0,43	0,00	0,00	ENSG00000020181
2,65	0,00	0,00	ENSG00000104221
0,76	0,00	0,00	ENSG00000156675
1,14	0,97	0,98	ENSG00000280064
#N/D	#N/D	#N/D	ENSG00000169154
1,14	0,97	#N/D	ENSG00000285880
1,14	0,97	#N/D	ENSG00000188778
0,70	0,00	0,00	ENSG00000187840
0,81	0,01	0,03	ENSG00000129691
0,71	0,28	0,38	ENSG00000147465
1,18	0,08	0,14	ENSG00000175324
1,15	0,11	0,18	ENSG00000156735
0,87	0,08	0,14	ENSG00000085788
1,82	0,00	0,00	ENSG00000147535
0,98	0,79	0,87	ENSG00000147548
0,50	0,00	0,00	ENSG00000165046
0,71	0,00	0,00	ENSG00000077782
0,94	0,42	0,54	ENSG00000147526
0,69	0,00	0,00	ENSG00000169499
0,36	0,35	0,46	ENSG00000169495
1,09	0,33	0,45	ENSG00000169490
1,49	0,00	0,00	ENSG00000168615
0,94	0,82	0,89	ENSG00000197140
#N/D	#N/D	#N/D	ENSG00000168619
#N/D	#N/D	#N/D	ENSG00000104755
0,06	0,00	0,00	ENSG00000131203
0,02	0,15	0,23	ENSG00000188676
0,12	0,00	0,00	ENSG00000176907
0,99	1,00	1,00	ENSG00000165061
2,02	0,00	0,00	ENSG00000104332
1,00	0,98	0,99	ENSG00000147533
1,60	0,00	0,00	ENSG00000147536
1,15	0,08	0,14	ENSG00000158669
0,07	0,31	#N/D	ENSG00000165066
0,25	0,00	0,00	ENSG00000029534
0,78	0,00	0,01	ENSG00000083168
0,55	0,00	0,00	ENSG00000070718
0,23	0,00	0,00	ENSG00000104368
0,81	0,01	0,03	ENSG00000104365
0,62	0,00	0,00	ENSG00000070501
0,13	0,46	#N/D	ENSG00000104371
0,77	0,00	0,00	ENSG00000078668
0,98	0,86	0,93	ENSG00000168575
0,56	0,00	0,00	ENSG00000176209

#N/D	#N/D	#N/D	ENSG00000147432
1,14	0,97	0,98	ENSG00000147434
0,93	0,47	0,57	ENSG00000131931
0,65	0,00	0,00	ENSG00000120925
1,26	0,00	0,01	ENSG00000168172
#N/D	#N/D	#N/D	ENSG00000254673
0,92	0,35	0,46	ENSG00000168522
1,15	0,09	0,16	ENSG00000185900
0,84	0,04	0,07	ENSG00000165102
1,14	0,97	#N/D	ENSG00000188877
0,89	0,18	0,27	ENSG00000164808
0,46	0,00	0,00	ENSG00000221869
0,76	0,00	0,00	ENSG00000253729
0,91	0,22	0,33	ENSG00000104738
0,69	0,00	0,00	ENSG00000169139
0,04	0,20	0,30	ENSG00000279881
#N/D	#N/D	#N/D	ENSG00000034239
0,74	0,00	0,01	ENSG00000019549
1,14	0,97	0,98	ENSG00000168333
8,39	0,46	#N/D	ENSG00000147481
0,44	0,04	0,08	ENSG00000147485
1,41	0,00	0,00	ENSG00000168300
0,56	0,07	0,12	ENSG00000147488
8,39	0,46	0,56	ENSG00000196711
0,60	0,00	0,00	ENSG00000023287
37,39	0,19	0,29	ENSG00000288611
44,64	0,17	0,26	ENSG00000082556
0,87	0,10	0,17	ENSG00000047249
1,07	0,66	0,75	ENSG00000147509
0,58	0,00	0,00	ENSG00000187735
0,69	0,00	0,00	ENSG00000120992
0,72	0,00	0,00	ENSG00000137547
0,50	0,00	0,00	ENSG00000164736
1,21	0,77	0,85	ENSG00000104237
2,22	0,24	0,35	ENSG00000206579
0,79	0,01	0,02	ENSG00000167904
0,49	0,00	0,00	ENSG00000137574
0,48	0,00	0,00	ENSG00000254087
0,99	0,85	0,92	ENSG00000008988
#N/D	#N/D	#N/D	ENSG00000172680
1,52	0,00	0,00	ENSG00000181690
2,13	0,00	0,00	ENSG00000170791
1,14	0,97	0,98	ENSG00000170786
#N/D	#N/D	#N/D	ENSG00000181195
1,27	0,00	0,01	ENSG00000104331
0,85	0,14	0,22	ENSG00000169122
0,42	0,00	0,00	ENSG00000215114
0,13	0,46	#N/D	ENSG00000167910

0,77	0,00	0,00	ENSG00000137575
0,58	0,00	0,00	ENSG00000035681
0,75	0,01	0,03	ENSG00000198846
8,39	0,46	0,56	ENSG00000178538
0,97	0,73	0,81	ENSG00000104388
1,70	0,00	0,00	ENSG00000171316
1,19	0,52	0,63	ENSG00000177182
1,42	0,00	0,00	ENSG00000198363
2,46	0,00	0,00	ENSG00000185942
0,98	0,84	0,91	ENSG00000137563
2,82	0,00	0,01	ENSG00000137561
1,15	0,10	0,17	ENSG00000185728
#N/D	#N/D	#N/D	ENSG00000180828
1,14	0,97	#N/D	ENSG00000172817
0,87	0,10	0,17	ENSG00000104442
1,01	0,94	0,98	ENSG00000066855
0,90	0,23	0,33	ENSG00000205268
12,83	0,00	0,00	ENSG00000147570
1,29	0,51	0,61	ENSG00000147573
3,56	0,23	0,34	ENSG00000147571
0,72	0,00	0,00	ENSG00000179041
1,14	0,97	0,98	ENSG00000285655
0,01	0,07	0,13	ENSG00000285791
1,66	0,03	0,06	ENSG00000147576
1,33	0,15	0,24	ENSG00000169085
0,96	0,67	0,76	ENSG00000185697
1,19	0,04	0,07	ENSG00000175073
1,43	0,02	0,03	ENSG00000288602
0,71	0,01	0,02	ENSG00000104205
0,75	0,06	0,10	ENSG00000178460
1,14	0,97	#N/D	ENSG00000261787
0,13	0,46	#N/D	ENSG00000178125
1,08	0,36	0,47	ENSG00000121022
0,73	0,00	0,00	ENSG00000104218
0,94	0,42	0,53	ENSG00000066777
0,05	0,00	0,00	ENSG00000165078
#N/D	#N/D	#N/D	ENSG00000046889
0,57	0,36	0,47	ENSG00000165084
0,10	0,00	0,00	ENSG00000137573
0,12	0,00	0,00	ENSG00000137571
#N/D	#N/D	#N/D	ENSG00000280393
1,14	0,97	#N/D	ENSG00000147596
0,95	0,56	0,66	ENSG00000140396
0,97	0,69	0,77	ENSG00000067167
0,73	0,01	0,02	ENSG00000147592
2,35	0,30	0,41	ENSG00000221947
0,55	0,00	0,00	ENSG00000104313
0,94	0,58	0,68	ENSG00000178860

2,05	0,00	0,00	ENSG00000104321
1,14	0,97	#N/D	ENSG00000182674
0,75	0,00	0,01	ENSG00000147601
0,63	0,28	0,39	ENSG00000164764
#N/D	#N/D	#N/D	ENSG00000274443
0,90	0,18	0,27	ENSG00000147604
0,74	0,00	0,01	ENSG00000121039
0,50	0,00	0,00	ENSG00000040341
0,68	0,00	0,01	ENSG00000258677
0,90	0,27	0,38	ENSG00000104343
0,94	0,45	0,56	ENSG00000154582
0,73	0,00	0,01	ENSG00000175606
0,22	0,00	0,00	ENSG00000154589
0,07	0,00	0,00	ENSG00000104369
0,29	0,00	0,00	ENSG00000104381
0,03	0,17	0,26	ENSG00000137558
0,68	0,00	0,00	ENSG00000121005
0,25	0,00	0,00	ENSG00000164749
0,79	0,00	0,01	ENSG00000091656
1,22	0,02	0,04	ENSG00000164751
0,36	0,00	0,00	ENSG00000171033
0,52	0,00	0,00	ENSG00000104427
0,51	0,00	0,00	ENSG00000104432
1,14	0,97	#N/D	ENSG00000104435
1,27	0,03	0,06	ENSG00000164683
0,94	0,49	0,59	ENSG00000147586
0,09	0,01	0,03	ENSG00000276418
0,34	0,00	0,00	ENSG00000076554
0,83	0,04	0,08	ENSG00000205189
1,29	0,00	0,01	ENSG00000164684
1,17	0,16	0,25	ENSG00000076641
17,46	0,00	0,00	ENSG00000164687
1,14	0,97	#N/D	ENSG00000147588
#N/D	#N/D	#N/D	ENSG00000205186
0,01	0,08	0,14	ENSG00000170323
1,14	0,97	#N/D	ENSG00000197416
0,61	0,00	0,00	ENSG00000133731
15,64	0,33	0,44	ENSG00000253598
0,52	0,01	0,03	ENSG00000280193
0,81	0,02	0,05	ENSG00000104231
1,14	0,97	0,98	ENSG00000164695
0,85	0,18	0,27	ENSG00000104497
0,13	0,46	#N/D	ENSG00000184672
0,49	0,00	0,00	ENSG00000133739
0,73	0,00	0,00	ENSG00000133740
0,79	0,01	0,03	ENSG00000176731
0,83	0,16	0,25	ENSG00000185015
0,27	0,22	0,32	ENSG00000133742

1,60	0,51	0,62	ENSG00000164879
0,71	0,01	0,03	ENSG00000104267
0,22	0,00	0,00	ENSG00000147614
8,39	0,46	#N/D	ENSG00000147613
#N/D	#N/D	#N/D	ENSG00000164893
0,78	0,00	0,01	ENSG00000123124
1,08	0,35	0,46	ENSG00000176623
0,98	0,82	0,89	ENSG00000085719
0,14	0,05	0,09	ENSG00000170289
0,07	0,31	#N/D	ENSG00000176571
#N/D	#N/D	#N/D	ENSG00000176566
0,59	0,00	0,00	ENSG00000156103
0,79	0,01	0,01	ENSG00000104312
0,76	0,00	0,01	ENSG00000164823
0,53	0,00	0,00	ENSG00000104320
1,08	0,43	0,55	ENSG00000104325
0,51	0,42	0,54	ENSG00000104327
0,72	0,00	0,00	ENSG00000180694
0,90	0,72	0,80	ENSG00000123119
0,57	0,00	0,00	ENSG00000253250
0,80	0,02	0,04	ENSG00000155099
0,80	0,05	0,09	ENSG00000155100
0,28	0,01	0,02	ENSG00000214954
0,50	0,32	0,43	ENSG00000147606
0,07	0,31	0,43	ENSG00000079102
1,21	0,03	0,07	ENSG00000205133
0,73	0,00	0,00	ENSG00000188343
1,07	0,43	0,55	ENSG00000183808
0,85	0,31	0,43	ENSG00000279331
0,59	0,00	0,00	ENSG00000164953
0,61	0,00	0,00	ENSG00000164951
0,78	0,61	0,70	ENSG00000079112
0,86	0,18	0,28	ENSG00000164949
0,91	0,33	0,44	ENSG00000197275
0,97	0,83	0,90	ENSG00000265817
1,23	0,01	0,02	ENSG00000164944
0,00	0,02	0,04	ENSG00000104413
0,71	0,00	0,00	ENSG00000156162
0,84	0,04	0,07	ENSG00000164941
0,71	0,01	0,02	ENSG00000175305
0,58	0,00	0,00	ENSG00000156170
3,28	0,00	0,00	ENSG00000164938
1,25	0,04	0,08	ENSG00000175895
0,93	0,56	0,66	ENSG00000156172
3,98	0,00	0,00	ENSG00000156466
0,71	0,00	0,00	ENSG00000156467
0,53	0,00	0,00	ENSG00000156469
1,03	0,74	0,82	ENSG00000156471

0,35	0,00	0,00	ENSG00000169439
0,91	0,35	0,47	ENSG00000104324
0,13	0,46	0,56	ENSG00000180543
0,91	0,24	0,34	ENSG00000147649
0,81	0,01	0,02	ENSG00000104341
0,76	0,03	0,06	ENSG00000132561
0,85	0,04	0,08	ENSG00000156482
1,14	0,97	0,98	ENSG00000177459
0,83	0,10	0,17	ENSG00000132541
1,00	0,99	1,00	ENSG00000104356
1,39	0,01	0,01	ENSG00000104361
0,85	0,08	0,14	ENSG00000104375
#N/D	#N/D	#N/D	ENSG00000278914
2,47	0,04	0,08	ENSG00000156486
1,20	0,38	0,49	ENSG00000164920
0,78	0,00	0,01	ENSG00000132549
0,96	0,65	0,74	ENSG00000164919
1,14	0,97	0,98	ENSG00000132554
0,56	0,00	0,00	ENSG00000156509
1,10	0,28	0,38	ENSG00000147669
0,28	0,00	0,00	ENSG00000104450
1,11	0,23	0,34	ENSG00000034677
0,66	0,00	0,00	ENSG00000186106
4,08	0,03	0,05	ENSG00000174226
0,85	0,04	0,08	ENSG00000070756
0,91	0,22	0,32	ENSG00000164924
0,79	0,01	0,01	ENSG00000120963
1,14	0,97	0,98	ENSG00000083307
1,14	0,97	0,98	ENSG00000104490
0,94	0,50	0,60	ENSG00000048392
1,51	0,00	0,00	ENSG00000104517
1,14	0,97	#N/D	ENSG00000155087
2,09	0,00	0,00	ENSG00000155090
0,76	0,00	0,00	ENSG00000155096
#N/D	#N/D	#N/D	ENSG00000289653
0,73	0,00	0,00	ENSG00000155097
1,33	0,54	0,64	ENSG00000164929
0,60	0,00	0,00	ENSG00000164930
0,97	0,92	0,98	ENSG00000164932
0,79	0,01	0,01	ENSG00000164933
0,02	0,11	0,18	ENSG00000285982
1,29	0,00	0,01	ENSG00000164934
8,39	0,46	0,56	ENSG00000176406
0,13	0,46	0,56	ENSG00000147647
#N/D	#N/D	#N/D	ENSG00000164935
0,02	0,15	0,23	ENSG00000279138
0,62	0,00	0,00	ENSG00000147650
1,00	0,98	0,99	ENSG00000169946

1,06	0,49	0,60	ENSG00000164830
2,62	0,10	0,16	ENSG00000174429
1,26	0,21	0,31	ENSG00000154188
2,80	0,20	0,30	ENSG00000147655
1,18	0,04	0,07	ENSG00000104408
1,82	0,00	0,00	ENSG00000104412
15,44	0,00	0,00	ENSG00000164841
2,20	0,08	0,14	ENSG00000174417
1,75	0,00	0,00	ENSG00000120526
1,55	0,00	0,00	ENSG00000120533
0,37	0,00	0,00	ENSG00000205038
0,71	0,00	0,00	ENSG00000147654
1,14	0,97	0,98	ENSG00000289767
0,10	0,00	0,00	ENSG00000147642
0,16	0,00	0,01	ENSG00000164794
0,56	0,02	0,05	ENSG00000164796
1,23	0,01	0,02	ENSG00000104447
1,42	0,00	0,00	ENSG00000147677
1,31	0,00	0,00	ENSG00000147679
1,02	0,77	0,85	ENSG00000164754
4,70	0,01	0,03	ENSG00000205002
22,89	0,26	0,37	ENSG00000164756
0,89	0,26	0,37	ENSG00000164758
1,81	0,00	0,00	ENSG00000182197
0,83	0,11	0,18	ENSG00000177570
8,01	0,00	0,00	ENSG00000164761
22,51	0,00	0,00	ENSG00000184374
0,98	0,99	0,99	ENSG00000147676
2,71	0,00	0,00	ENSG00000136999
0,43	0,00	0,00	ENSG00000136960
1,28	0,00	0,01	ENSG00000064313
1,02	0,85	0,92	ENSG00000136982
0,55	0,01	0,02	ENSG00000279347
4,69	0,00	0,00	ENSG00000155792
1,36	0,47	0,57	ENSG00000187955
1,50	0,00	0,00	ENSG00000172172
0,96	0,63	0,73	ENSG00000172167
1,63	0,00	0,00	ENSG00000172164
0,53	0,00	0,00	ENSG00000170961
0,42	0,00	0,00	ENSG00000178764
1,37	0,00	0,00	ENSG00000136986
0,87	0,12	0,19	ENSG00000156787
0,07	0,00	0,00	ENSG00000147689
1,11	0,28	0,38	ENSG00000189376
1,78	0,00	0,00	ENSG00000259305
1,41	0,00	0,00	ENSG00000165156
0,85	0,04	0,08	ENSG00000156802
1,73	0,00	0,00	ENSG00000156795

0,46	0,00	0,00	ENSG00000156804
1,06	0,69	0,78	ENSG00000175946
0,05	0,24	0,35	ENSG00000104537
1,41	0,00	0,00	ENSG00000176853
0,07	0,00	0,00	ENSG00000214814
1,01	0,88	0,95	ENSG00000164983
0,89	0,20	0,30	ENSG00000183665
0,82	0,03	0,05	ENSG00000170881
0,45	0,00	0,00	ENSG00000147687
0,83	0,03	0,05	ENSG00000147684
0,95	0,65	0,74	ENSG00000170873
1,14	0,97	0,98	ENSG00000180938
2,88	0,00	0,00	ENSG00000104549
0,81	0,01	0,02	ENSG00000164961
1,37	0,00	0,00	ENSG00000156831
0,67	0,00	0,00	ENSG00000173334
1,86	0,00	0,00	ENSG00000168672
1,97	0,00	0,01	ENSG00000212993
0,53	0,00	0,00	ENSG00000136997
37,39	0,19	0,29	ENSG00000147697
0,61	0,00	0,00	ENSG00000153310
1,13	0,13	0,22	ENSG00000153317
13,28	0,00	0,00	ENSG00000155897
1,56	0,00	0,00	ENSG00000132294
#N/D	#N/D	#N/D	ENSG00000253117
0,05	0,24	0,35	ENSG00000258417
1,85	0,60	#N/D	ENSG00000132297
0,26	0,00	0,00	ENSG00000184156
2,45	0,00	0,00	ENSG00000129295
0,42	0,00	0,01	ENSG00000165071
0,91	0,29	0,40	ENSG00000129292
0,75	0,11	0,18	ENSG00000042832
0,04	0,00	0,00	ENSG00000155926
0,09	0,00	0,00	ENSG00000104415
10,16	0,00	0,00	ENSG00000104419
3,49	0,00	0,00	ENSG00000008513
1,58	0,00	0,00	ENSG00000066827
0,27	0,22	0,32	ENSG00000278898
0,67	0,27	0,38	ENSG00000279518
7,56	0,00	0,00	ENSG00000131773
1,14	0,97	0,98	ENSG00000147724
1,30	0,03	0,07	ENSG00000169436
0,05	0,24	0,35	ENSG00000169427
1,43	0,00	0,00	ENSG00000167632
2,35	0,30	0,41	ENSG00000250733
1,13	0,14	0,22	ENSG00000104472
1,25	0,00	0,01	ENSG00000123908
1,63	0,02	0,04	ENSG00000279766

1,54	0,12	0,20	ENSG00000280326
1,37	0,00	0,00	ENSG00000169398
1,04	0,82	0,89	ENSG00000279786
1,49	0,00	0,00	ENSG00000105339
0,72	0,00	0,00	ENSG00000022567
1,21	0,62	0,72	ENSG00000280035
1,91	0,35	0,47	ENSG00000204882
0,75	0,00	0,00	ENSG00000184489
2,86	0,07	0,12	ENSG00000226807
#N/D	#N/D	#N/D	ENSG00000226490
0,89	0,22	0,32	ENSG00000171045
2,98	0,00	0,00	ENSG00000181790
0,29	0,00	0,00	ENSG00000198576
1,77	0,00	0,00	ENSG00000234616
0,77	0,08	0,13	ENSG00000167653
0,85	0,04	0,08	ENSG00000160886
1,45	0,00	0,00	ENSG00000130193
4,76	0,00	0,00	ENSG00000126233
5,28	0,10	0,17	ENSG00000197353
5,45	0,00	0,00	ENSG00000284505
1,14	0,97	#N/D	ENSG00000283992
15,25	0,00	0,00	ENSG00000180155
1,14	0,97	#N/D	ENSG00000167656
#N/D	#N/D	#N/D	ENSG00000104499
15,64	0,33	#N/D	ENSG00000160882
1,14	0,97	#N/D	ENSG00000179142
1,54	0,00	0,00	ENSG00000160932
0,74	0,57	0,67	ENSG00000291309
#N/D	#N/D	#N/D	ENSG00000261667
1,14	0,97	#N/D	ENSG00000176956
0,94	0,76	0,84	ENSG00000277494
0,90	0,21	0,31	ENSG00000181638
1,58	0,00	0,00	ENSG00000264668
1,39	0,00	0,00	ENSG00000250571
1,13	0,19	0,28	ENSG00000185730
1,22	0,02	0,04	ENSG00000184428
2,38	0,00	0,00	ENSG00000158106
0,19	0,00	0,00	ENSG00000182759
1,46	0,00	0,00	ENSG00000014164
2,20	0,00	0,00	ENSG00000104518
0,93	0,52	0,63	ENSG00000204839
1,38	0,00	0,01	ENSG00000147813
2,21	0,00	0,00	ENSG00000279605
1,95	0,00	0,00	ENSG00000104529
1,48	0,00	0,00	ENSG00000179886
1,76	0,00	0,00	ENSG00000104524
2,13	0,00	0,00	ENSG00000104522
1,20	0,02	0,05	ENSG00000183309

1,92	0,00	0,00	ENSG00000181135
2,22	0,24	0,35	ENSG00000255181
1,27	0,35	0,46	ENSG00000181085
0,95	0,54	0,64	ENSG00000180921
0,81	0,20	0,30	ENSG00000203499
0,99	0,88	0,95	ENSG00000180900
1,29	0,00	0,00	ENSG00000179950
2,41	0,00	0,00	ENSG00000185189
29,89	0,00	0,00	ENSG00000261150
1,93	0,00	0,00	ENSG00000178209
1,50	0,00	0,00	ENSG00000178685
1,34	0,00	0,00	ENSG00000178719
0,76	0,24	0,35	ENSG00000186583
1,09	0,31	0,43	ENSG00000178814
1,59	0,00	0,00	ENSG00000290230
1,43	0,00	0,00	ENSG00000178896
2,39	0,00	0,00	ENSG00000197858
1,31	0,00	0,00	ENSG00000179091
1,57	0,00	0,00	ENSG00000179526
1,36	0,00	0,00	ENSG00000179632
1,88	0,00	0,00	ENSG00000179698
0,84	0,04	0,08	ENSG00000235173
2,24	0,00	0,00	ENSG00000179832
0,80	0,01	0,02	ENSG00000261236
1,94	0,02	0,05	ENSG00000260428
1,16	0,06	0,12	ENSG00000185122
1,39	0,00	0,00	ENSG00000185000
0,55	0,28	0,39	ENSG00000261678
1,42	0,00	0,00	ENSG00000185803
0,66	0,42	0,54	ENSG00000261587
0,35	0,00	0,01	ENSG00000271698
0,96	0,66	0,75	ENSG00000182325
1,20	0,17	0,26	ENSG00000173137
1,26	0,01	0,01	ENSG00000071894
1,09	0,47	0,58	ENSG00000147804
1,27	0,01	0,01	ENSG00000160948
0,68	0,00	0,00	ENSG00000160949
1,16	0,16	0,25	ENSG00000187954
1,77	0,00	0,00	ENSG00000291316
1,15	0,19	0,29	ENSG00000291317
1,33	0,00	0,00	ENSG00000167702
1,24	0,41	0,52	ENSG00000160973
1,09	0,34	0,45	ENSG00000160972
0,50	0,02	0,04	ENSG00000167701
1,13	0,20	0,30	ENSG00000167700
1,54	0,00	0,00	ENSG00000160957
1,44	0,00	0,00	ENSG00000160959
0,17	0,00	0,00	ENSG00000254402

1,11	0,27	0,37	ENSG00000213563
0,62	0,00	0,00	ENSG00000147799
1,21	0,04	0,08	ENSG00000198169
0,75	0,03	0,07	ENSG00000196378
0,97	0,73	0,82	ENSG00000161016
0,64	0,00	0,00	ENSG00000197363
1,30	0,00	0,01	ENSG00000147789
1,34	0,00	0,00	ENSG00000170619
1,29	0,01	0,02	ENSG00000196150
1,28	0,02	0,04	ENSG00000170631
1,43	0,00	0,00	ENSG00000182307
0,91	0,27	0,38	ENSG00000181404
0,18	0,00	0,00	ENSG00000170122
0,91	0,36	0,47	ENSG00000172785
0,36	0,00	0,00	ENSG00000107099
0,75	0,00	0,00	ENSG00000107104
0,34	0,00	0,00	ENSG00000137090
0,78	0,09	0,15	ENSG00000064218
1,44	0,68	0,77	ENSG00000173253
1,60	0,00	0,00	ENSG00000080503
1,22	0,05	0,10	ENSG00000147852
0,91	0,73	0,81	ENSG00000168263
0,70	0,00	0,00	ENSG00000080608
0,98	0,86	0,93	ENSG00000080298
0,97	0,68	0,77	ENSG00000107249
1,01	0,91	0,97	ENSG00000106688
1,26	0,58	0,68	ENSG00000106686
0,45	0,00	0,00	ENSG00000205808
1,18	0,13	0,20	ENSG00000106993
0,80	0,01	0,02	ENSG00000147853
0,72	0,00	0,00	ENSG00000120158
0,75	0,01	0,02	ENSG00000096968
1,14	0,97	0,98	ENSG00000120210
8,39	0,46	0,56	ENSG00000120211
1,14	0,97	0,98	ENSG00000107014
1,14	0,97	#N/D	ENSG00000107018
0,74	0,00	0,01	ENSG00000107020
0,80	0,29	0,40	ENSG00000120217
0,23	0,00	0,00	ENSG00000197646
1,19	0,04	0,08	ENSG00000107036
0,53	0,00	0,00	ENSG00000099219
0,62	0,00	0,00	ENSG00000183354
0,41	0,04	0,07	ENSG00000120215
0,95	0,55	0,65	ENSG00000137040
0,36	0,35	0,46	ENSG00000137033
1,14	0,97	0,98	ENSG00000170777
1,05	0,54	0,64	ENSG00000147854
0,62	0,05	0,10	ENSG00000178445

1,06	0,51	0,62	ENSG00000107077
0,99	0,95	0,98	ENSG00000137038
1,14	0,97	#N/D	ENSG00000153707
17,33	0,00	0,00	ENSG00000107165
1,15	0,10	0,17	ENSG00000153714
4,65	0,00	0,00	ENSG00000107186
1,07	0,42	0,54	ENSG00000147862
1,33	0,00	0,00	ENSG00000175893
#N/D	#N/D	#N/D	ENSG00000147869
1,80	0,00	0,00	ENSG00000164946
1,85	0,00	0,00	ENSG00000155158
1,77	0,00	0,00	ENSG00000164975
0,76	0,00	0,00	ENSG00000164985
0,58	0,00	0,00	ENSG00000164989
3,29	0,00	0,00	ENSG00000173068
1,91	0,00	0,00	ENSG00000044459
1,14	0,97	#N/D	ENSG00000107295
1,62	0,00	0,00	ENSG00000178031
2,13	0,02	0,04	ENSG00000155875
2,39	0,00	0,00	ENSG00000155876
1,08	0,34	0,45	ENSG00000147874
5,20	0,00	0,00	ENSG00000147872
1,12	0,19	0,28	ENSG00000137145
1,20	0,02	0,04	ENSG00000137154
1,38	0,13	0,22	ENSG00000177076
1,14	0,97	#N/D	ENSG00000155886
1,36	0,00	0,00	ENSG00000171843
1,61	0,00	0,00	ENSG00000188352
1,02	0,85	0,91	ENSG00000188921
0,07	0,31	0,43	ENSG00000171855
8,39	0,46	0,56	ENSG00000177047
1,14	0,97	#N/D	ENSG00000137080
#N/D	#N/D	#N/D	ENSG00000236637
#N/D	#N/D	#N/D	ENSG00000214042
#N/D	#N/D	#N/D	ENSG00000186803
15,64	0,33	#N/D	ENSG00000147885
#N/D	#N/D	#N/D	ENSG00000234829
8,39	0,46	#N/D	ENSG00000228083
30,14	0,22	0,32	ENSG00000147873
2,29	0,00	0,00	ENSG00000198642
#N/D	#N/D	#N/D	ENSG00000120235
0,05	0,24	0,35	ENSG00000233816
#N/D	#N/D	#N/D	ENSG00000188379
0,18	0,10	0,16	ENSG00000120242
3,50	0,00	0,00	ENSG00000197919
2,44	0,00	0,00	ENSG00000184995
4,98	0,00	0,00	ENSG00000099810
#N/D	#N/D	#N/D	ENSG00000279670

#N/D	#N/D	#N/D	ENSG00000147889
#N/D	#N/D	#N/D	ENSG00000147883
#N/D	#N/D	#N/D	ENSG00000176399
0,53	0,58	0,68	ENSG00000107105
#N/D	#N/D	#N/D	ENSG00000205442
0,93	0,64	0,73	ENSG00000198680
#N/D	#N/D	#N/D	ENSG00000279256
1,15	0,29	0,40	ENSG00000120159
1,93	0,00	0,00	ENSG00000137055
1,19	0,21	0,31	ENSG00000096872
30,14	0,22	0,32	ENSG00000184434
0,27	0,22	0,32	ENSG00000120156
#N/D	#N/D	#N/D	ENSG00000120160
4,03	0,00	0,00	ENSG00000120162
8,39	0,46	0,56	ENSG00000147896
1,36	0,01	0,02	ENSG00000147894
0,13	0,46	0,56	ENSG00000174482
0,75	0,00	0,00	ENSG00000122729
1,16	0,08	0,15	ENSG00000107201
1,43	0,13	0,21	ENSG00000288684
0,76	0,00	0,01	ENSG00000197579
1,18	0,19	0,28	ENSG00000235453
1,16	0,11	0,19	ENSG00000165264
1,14	0,97	0,98	ENSG00000122728
0,02	0,12	0,19	ENSG00000188133
0,93	0,42	0,54	ENSG00000137074
1,11	0,21	0,31	ENSG00000086061
1,02	0,84	0,91	ENSG00000122692
1,21	0,02	0,03	ENSG00000086062
1,14	0,97	0,98	ENSG00000122711
0,63	0,00	0,00	ENSG00000107262
1,19	0,04	0,07	ENSG00000086065
1,21	0,02	0,05	ENSG00000086102
1,45	0,56	0,67	ENSG00000165269
0,65	0,00	0,01	ENSG00000165272
1,73	0,00	0,00	ENSG00000165271
0,23	0,00	0,00	ENSG00000230453
1,44	0,68	0,77	ENSG00000205274
1,14	0,97	0,98	ENSG00000010438
1,77	0,00	0,00	ENSG00000107341
1,17	0,07	0,13	ENSG00000137073
1,03	0,69	0,78	ENSG00000198876
0,90	0,21	0,31	ENSG00000165006
1,05	0,59	0,69	ENSG00000186638
1,11	0,29	0,41	ENSG00000164978
1,38	0,02	0,04	ENSG00000164976
1,68	0,40	0,52	ENSG00000164972
1,26	0,01	0,01	ENSG00000164970

0,38	0,07	0,12	ENSG00000122735
0,39	0,01	0,02	ENSG00000168913
14,71	0,01	0,01	ENSG00000122756
0,48	0,00	0,00	ENSG00000164967
1,53	0,00	0,00	ENSG00000137100
1,08	0,87	0,93	ENSG00000205143
0,78	0,00	0,01	ENSG00000147955
1,05	0,63	0,72	ENSG00000213930
2,52	0,00	0,00	ENSG00000258728
3,22	0,00	0,00	ENSG00000137070
0,67	0,65	0,74	ENSG00000213927
1,16	0,32	0,44	ENSG00000187186
3,56	0,23	0,34	ENSG00000172724
2,35	0,30	0,41	ENSG00000137077
3,72	0,00	0,00	ENSG00000205108
15,64	0,33	0,44	ENSG00000187791
2,10	0,00	0,01	ENSG00000122733
2,00	0,00	0,00	ENSG00000137094
1,02	0,95	0,98	ENSG00000279489
0,92	0,62	0,71	ENSG00000279608
0,54	0,00	0,00	ENSG00000174038
1,42	0,00	0,00	ENSG00000165280
0,13	0,46	#N/D	ENSG00000288699
1,02	0,85	0,92	ENSG00000221829
1,06	0,49	0,59	ENSG00000165282
0,83	0,02	0,04	ENSG00000165283
2,96	0,00	0,00	ENSG00000005238
1,15	0,09	0,16	ENSG00000198722
0,98	0,99	0,99	ENSG00000279083
1,13	0,12	0,20	ENSG00000198853
1,35	0,12	0,20	ENSG00000215187
1,34	0,00	0,00	ENSG00000107140
3,88	0,00	0,00	ENSG00000137101
1,15	0,74	0,82	ENSG00000137078
2,15	0,00	0,00	ENSG00000159884
0,69	0,00	0,00	ENSG00000137135
8,47	0,00	0,00	ENSG00000107159
1,33	0,00	0,00	ENSG00000198467
1,51	0,00	0,00	ENSG00000137076
2,41	0,00	0,00	ENSG00000107175
1,15	0,08	0,14	ENSG00000070610
1,28	0,00	0,01	ENSG00000107185
1,14	0,97	0,98	ENSG00000215183
0,88	0,10	0,17	ENSG00000159899
1,92	0,00	0,00	ENSG00000137098
0,70	0,00	0,00	ENSG00000137133
51,90	0,15	0,24	ENSG00000285645
1,33	0,00	0,00	ENSG00000137103

0,33	0,00	0,00	ENSG00000204930
13,85	0,01	0,02	ENSG00000168828
1,62	0,00	0,00	ENSG00000196196
2,41	0,00	0,00	ENSG00000235387
30,14	0,22	0,32	ENSG00000278889
#N/D	#N/D	#N/D	ENSG00000243641
0,85	0,05	0,09	ENSG00000122707
0,33	0,00	0,00	ENSG00000122694
0,24	0,00	0,01	ENSG00000185972
0,89	0,16	0,25	ENSG00000122705
0,85	0,04	0,08	ENSG00000159921
1,01	0,87	0,94	ENSG00000137075
0,72	0,00	0,00	ENSG00000165304
4,28	0,00	0,00	ENSG00000196092
1,07	0,45	0,56	ENSG00000147905
0,95	0,57	0,67	ENSG00000137106
2,52	0,00	0,00	ENSG00000168795
1,29	0,00	0,01	ENSG00000137054
0,71	0,00	0,00	ENSG00000147912
0,32	0,00	0,01	ENSG00000256966
1,09	0,33	0,44	ENSG00000175768
1,41	0,15	0,23	ENSG00000255872
0,34	0,18	0,27	ENSG00000070601
2,20	0,00	0,00	ENSG00000165275
0,81	0,05	0,09	ENSG00000107371
0,94	0,44	0,56	ENSG00000122741
1,12	0,24	0,34	ENSG00000122696
1,33	0,00	0,00	ENSG00000107338
0,67	0,00	0,00	ENSG00000137124
3,11	0,00	0,00	ENSG00000137142
0,46	0,00	0,00	ENSG00000180071
#N/D	#N/D	#N/D	ENSG00000283329
1,82	0,00	0,00	ENSG00000106714
#N/D	#N/D	#N/D	ENSG00000204849
0,68	0,02	0,05	ENSG00000280077
0,95	0,82	0,90	ENSG00000279456
0,66	0,51	0,62	ENSG00000273514
0,59	0,00	0,00	ENSG00000215126
0,71	0,01	0,03	ENSG00000279561
1,18	0,05	0,09	ENSG00000154529
1,14	0,97	#N/D	ENSG00000185775
1,14	0,97	#N/D	ENSG00000276581
#N/D	#N/D	#N/D	ENSG00000276040
2,78	0,00	0,00	ENSG00000283378
2,02	0,00	0,00	ENSG00000284471
1,04	0,91	0,97	ENSG00000204779
0,93	0,50	0,61	ENSG00000147996
0,02	0,12	0,19	ENSG00000184659

0,56	0,00	0,00	ENSG00000274349
#N/D	#N/D	#N/D	ENSG00000275969
1,33	0,36	0,47	ENSG00000260691
1,20	0,11	0,18	ENSG00000196873
1,14	0,51	0,62	ENSG00000279706
0,02	0,15	0,23	ENSG00000187559
0,46	0,00	0,00	ENSG00000154330
#N/D	#N/D	#N/D	ENSG00000181778
1,14	0,97	0,98	ENSG00000107242
0,45	0,00	0,00	ENSG00000187866
#N/D	#N/D	#N/D	ENSG00000165059
1,28	0,03	0,07	ENSG00000285130
0,58	0,00	0,00	ENSG00000165060
0,58	0,00	0,00	ENSG00000119139
182,41	0,06	0,10	ENSG00000135063
7,70	0,00	0,00	ENSG00000107282
1,39	0,00	0,00	ENSG00000188647
0,13	0,46	#N/D	ENSG00000204711
2,65	0,02	0,04	ENSG00000165072
0,84	0,05	0,10	ENSG00000198887
1,08	0,35	0,46	ENSG00000119138
4,42	0,15	0,24	ENSG00000083067
1,01	0,88	0,95	ENSG00000135048
1,21	0,07	0,13	ENSG00000107362
1,20	0,13	0,20	ENSG00000155621
1,14	0,97	#N/D	ENSG00000204669
0,05	0,24	0,35	ENSG00000119125
0,78	0,00	0,01	ENSG00000107372
4,42	0,15	0,24	ENSG00000165091
0,11	0,03	0,05	ENSG00000165092
0,91	0,25	0,35	ENSG00000135046
7,56	0,00	0,00	ENSG00000198963
4,42	0,15	0,24	ENSG00000119121
0,91	0,38	0,49	ENSG00000135045
1,16	0,13	0,21	ENSG00000156017
0,99	0,91	0,97	ENSG00000106733
1,78	0,00	0,00	ENSG00000134996
0,29	0,00	0,00	ENSG00000099139
0,52	0,00	0,00	ENSG00000135002
0,32	0,00	0,00	ENSG00000187210
8,36	0,00	0,00	ENSG00000106772
1,14	0,97	#N/D	ENSG00000204612
0,69	0,00	0,00	ENSG00000197969
0,74	0,69	0,78	ENSG00000156049
0,96	0,63	0,73	ENSG00000156052
0,63	0,00	0,00	ENSG00000148019
0,20	0,00	0,00	ENSG00000135069
1,02	0,83	0,90	ENSG00000106829

#N/D	#N/D	#N/D	ENSG00000279715
0,49	0,00	0,00	ENSG00000196781
1,14	0,97	0,98	ENSG00000189357
1,14	0,97	0,98	ENSG00000186788
1,14	0,97	0,98	ENSG00000214929
1,14	0,97	#N/D	ENSG00000278988
0,29	0,00	0,00	ENSG00000165105
0,28	0,00	0,00	ENSG00000172159
0,66	0,07	0,12	ENSG00000148057
0,76	0,00	0,00	ENSG00000135018
0,52	0,00	0,00	ENSG00000165113
0,43	0,00	0,00	ENSG00000165115
0,59	0,00	0,00	ENSG00000165118
0,74	0,00	0,00	ENSG00000165119
0,72	0,00	0,00	ENSG00000178966
0,08	0,01	0,02	ENSG00000197506
2,71	0,36	0,48	ENSG00000148053
0,42	0,00	0,00	ENSG00000135049
0,94	0,47	0,57	ENSG00000135040
0,44	0,00	0,00	ENSG00000135052
1,14	0,62	0,72	ENSG00000187753
0,72	0,00	0,00	ENSG00000135070
0,86	0,07	0,12	ENSG00000083223
0,59	0,01	0,02	ENSG00000180447
0,52	0,00	0,00	ENSG00000196730
0,54	0,00	0,00	ENSG00000135047
1,14	0,97	#N/D	ENSG00000177992
15,64	0,33	0,44	ENSG00000230246
#N/D	#N/D	#N/D	ENSG00000283205
1,23	0,04	0,08	ENSG00000156345
1,14	0,97	#N/D	ENSG00000177910
1,14	0,97	#N/D	ENSG00000279154
1,06	0,45	0,56	ENSG00000106723
2,14	0,00	0,01	ENSG00000130045
1,47	0,00	0,00	ENSG00000213694
0,22	0,00	0,00	ENSG00000148082
0,90	0,22	0,32	ENSG00000123975
0,63	0,00	0,00	ENSG00000187742
0,47	0,00	0,00	ENSG00000187764
88,15	0,10	0,17	ENSG00000130222
#N/D	#N/D	#N/D	ENSG00000279503
0,53	0,58	#N/D	ENSG00000165023
980,02	0,01	0,03	ENSG00000165025
0,83	0,08	0,13	ENSG00000148090
1,44	0,00	0,00	ENSG00000165030
90,99	0,00	0,00	ENSG00000169071
1,06	0,48	0,58	ENSG00000090054
0,64	0,00	0,00	ENSG00000196305

0,74	0,00	0,00	ENSG00000198000
1,19	0,06	0,10	ENSG00000188312
1,81	0,18	0,27	ENSG00000106809
0,41	0,10	0,16	ENSG00000127083
0,27	0,22	0,32	ENSG00000106819
0,27	0,00	0,00	ENSG00000106823
1,41	0,00	0,00	ENSG00000127080
1,15	0,08	0,14	ENSG00000185963
0,99	0,88	0,94	ENSG00000127081
1,79	0,22	0,32	ENSG00000127084
0,13	0,46	0,56	ENSG00000157303
1,93	0,00	0,00	ENSG00000165233
1,33	0,00	0,00	ENSG00000131669
0,66	0,00	0,00	ENSG00000165238
0,70	0,00	0,00	ENSG00000188938
1,00	0,96	0,98	ENSG00000048828
0,88	0,13	0,21	ENSG00000197724
0,86	0,07	0,13	ENSG00000131668
0,59	0,00	0,00	ENSG00000158079
1,20	0,07	0,12	ENSG00000175787
2,72	0,04	0,07	ENSG00000130950
0,94	0,46	0,56	ENSG00000148110
0,36	0,35	0,46	ENSG00000130957
0,98	0,99	0,99	ENSG00000165140
0,75	0,00	0,00	ENSG00000148120
0,98	0,80	0,88	ENSG00000158169
1,11	0,30	0,42	ENSG00000185920
0,77	0,00	0,01	ENSG00000182150
1,91	0,35	0,47	ENSG00000130948
1,14	0,97	0,98	ENSG00000285269
0,77	0,01	0,02	ENSG00000130958
1,26	0,01	0,03	ENSG00000165244
1,31	0,00	0,01	ENSG00000130956
0,73	0,00	0,00	ENSG00000081377
0,84	0,05	0,09	ENSG00000158122
0,62	0,00	0,00	ENSG00000081386
0,57	0,00	0,00	ENSG00000196597
1,39	0,01	0,02	ENSG00000188152
4,66	0,00	0,00	ENSG00000136943
0,99	0,95	0,98	ENSG00000197816
0,90	0,22	0,32	ENSG00000196116
0,37	0,00	0,00	ENSG00000136842
0,61	0,00	0,00	ENSG00000136925
0,80	0,01	0,02	ENSG00000136937
0,84	0,29	0,40	ENSG00000136936
0,51	0,42	0,54	ENSG00000178919
0,55	0,00	0,00	ENSG00000136932
#N/D	#N/D	#N/D	ENSG00000136929

0,62	0,00	0,00	ENSG00000136938
1,19	0,03	0,06	ENSG00000095380
0,74	0,00	0,00	ENSG00000106785
1,18	0,23	0,33	ENSG00000106789
1,08	0,40	0,52	ENSG00000095383
2,08	0,14	0,22	ENSG00000136928
0,63	0,00	0,00	ENSG00000165138
0,28	0,00	0,00	ENSG00000119514
0,21	0,00	0,00	ENSG00000204291
0,55	0,00	0,00	ENSG00000106799
1,99	0,00	0,00	ENSG00000119523
1,53	0,00	0,00	ENSG00000106803
0,93	0,75	0,83	ENSG00000119508
0,62	0,00	0,00	ENSG00000136874
1,06	0,46	0,56	ENSG00000023318
0,70	0,00	0,00	ENSG00000119509
0,91	0,25	0,35	ENSG00000136891
1,04	0,66	0,75	ENSG00000066697
0,54	0,00	0,00	ENSG00000251349
0,50	0,00	0,00	ENSG00000241697
0,54	0,00	0,00	ENSG00000170681
0,07	0,31	#N/D	ENSG00000148123
0,73	0,33	0,44	ENSG00000136881
0,80	0,02	0,05	ENSG00000136897
0,70	0,00	0,00	ENSG00000136870
1,60	0,51	0,62	ENSG00000136872
0,72	0,00	0,00	ENSG00000165152
1,33	0,00	0,00	ENSG00000155827
1,84	0,00	0,00	ENSG00000198785
0,98	0,99	0,99	ENSG00000188386
#N/D	#N/D	#N/D	ENSG00000155833
0,72	0,00	0,00	ENSG00000136824
#N/D	#N/D	#N/D	ENSG00000186881
#N/D	#N/D	#N/D	ENSG00000148136
1,14	0,97	#N/D	ENSG00000204246
#N/D	#N/D	#N/D	ENSG00000186943
#N/D	#N/D	#N/D	ENSG00000277556
#N/D	#N/D	#N/D	ENSG00000276119
#N/D	#N/D	#N/D	ENSG00000136839
1,14	0,97	#N/D	ENSG00000179055
1,15	0,14	0,22	ENSG00000136783
0,90	0,62	0,72	ENSG00000165028
0,23	0,00	0,00	ENSG00000165029
0,74	0,00	0,00	ENSG00000070214
1,01	0,94	0,98	ENSG00000106701
0,79	0,01	0,01	ENSG00000106692
8,39	0,46	0,56	ENSG00000279609
0,07	0,31	0,43	ENSG00000186051

0,37	0,00	0,00	ENSG00000095209
0,53	0,00	0,00	ENSG00000148143
0,74	0,00	0,00	ENSG00000119318
0,71	0,00	0,00	ENSG00000136826
37,39	0,19	0,29	ENSG00000148156
0,98	0,99	0,99	ENSG00000187003
1,17	0,05	0,09	ENSG00000070061
0,83	0,07	0,13	ENSG00000119328
1,09	0,29	0,40	ENSG00000119326
1,47	0,00	0,00	ENSG00000106771
1,85	0,60	0,69	ENSG00000260230
0,43	0,00	0,00	ENSG00000095203
0,71	0,00	0,00	ENSG00000070159
0,63	0,00	0,00	ENSG00000157654
1,45	0,37	0,48	ENSG00000188959
1,13	0,14	0,22	ENSG00000136810
1,14	0,97	#N/D	ENSG00000204193
0,72	0,00	0,00	ENSG00000165124
2,14	0,00	0,00	ENSG00000030304
1,54	0,00	0,00	ENSG00000198121
0,07	0,31	0,43	ENSG00000171133
0,84	0,03	0,07	ENSG00000136813
0,94	0,71	0,80	ENSG00000173258
0,83	0,03	0,07	ENSG00000106853
1,84	0,00	0,00	ENSG00000059769
1,18	0,16	0,25	ENSG00000244115
0,86	0,09	0,16	ENSG00000242616
1,61	0,36	0,47	ENSG00000165181
0,43	0,00	0,00	ENSG00000148154
0,51	0,00	0,00	ENSG00000106868
0,86	0,06	0,12	ENSG00000119314
0,70	0,00	0,00	ENSG00000119471
0,70	0,00	0,00	ENSG00000165185
1,20	0,04	0,07	ENSG00000148153
0,92	0,32	0,43	ENSG00000148158
0,85	0,60	0,70	ENSG00000119457
4,53	0,00	0,00	ENSG00000228623
1,06	0,70	0,79	ENSG00000136866
1,84	0,01	0,03	ENSG00000136867
1,16	0,08	0,14	ENSG00000119321
1,72	0,00	0,00	ENSG00000136868
1,03	0,75	0,83	ENSG00000176386
1,00	0,98	0,99	ENSG00000136875
0,99	1,00	1,00	ENSG00000165188
0,65	0,00	0,00	ENSG00000148225
0,07	0,31	0,43	ENSG00000119411
0,17	0,00	0,00	ENSG00000119431
1,27	0,01	0,01	ENSG00000148218

1,70	0,00	0,00	ENSG00000148229
0,88	0,64	0,73	ENSG00000157653
1,46	0,00	0,00	ENSG00000138835
1,54	0,44	0,56	ENSG00000282897
0,81	0,01	0,02	ENSG00000157657
0,16	0,07	0,12	ENSG00000106927
1,44	0,68	0,77	ENSG00000136883
1,11	0,22	0,32	ENSG00000196739
1,14	0,97	#N/D	ENSG00000229314
0,07	0,31	#N/D	ENSG00000228278
0,35	0,00	0,00	ENSG00000106948
1,69	0,00	0,00	ENSG00000095397
0,85	0,06	0,10	ENSG00000136888
0,71	0,00	0,00	ENSG00000157693
30,14	0,22	0,32	ENSG00000230054
0,36	0,35	0,46	ENSG00000230601
0,45	0,00	0,00	ENSG00000181634
0,88	0,78	0,86	ENSG00000106952
1,58	0,00	0,00	ENSG00000041982
0,03	0,00	0,00	ENSG00000182752
0,65	0,00	0,00	ENSG00000148219
0,78	0,00	0,01	ENSG00000119401
0,75	0,50	0,61	ENSG00000285082
0,68	0,00	0,00	ENSG00000136869
1,14	0,97	#N/D	ENSG00000078725
0,76	0,00	0,00	ENSG00000136861
0,93	0,44	0,56	ENSG00000106780
1,02	0,85	0,92	ENSG00000119402
0,26	0,00	0,00	ENSG00000214654
0,91	0,26	0,37	ENSG00000095261
0,75	0,00	0,00	ENSG00000119403
0,14	0,00	0,00	ENSG00000056558
0,67	0,01	0,02	ENSG00000106804
0,52	0,00	0,00	ENSG00000119397
1,19	0,03	0,06	ENSG00000119396
3,58	0,00	0,00	ENSG00000148180
0,68	0,00	0,00	ENSG00000148175
6,99	0,05	0,10	ENSG00000204136
1,47	0,00	0,00	ENSG00000136848
0,77	0,00	0,00	ENSG00000175764
1,05	0,57	0,68	ENSG00000119421
8,39	0,46	0,56	ENSG00000185681
1,36	0,07	0,13	ENSG00000106852
0,92	0,34	0,46	ENSG00000119446
0,93	0,39	0,51	ENSG00000148187
0,98	0,96	0,98	ENSG00000095303
0,07	0,31	0,43	ENSG00000136834
#N/D	#N/D	#N/D	ENSG00000197233

#N/D	#N/D	#N/D	ENSG00000239590
#N/D	#N/D	#N/D	ENSG00000171505
#N/D	#N/D	#N/D	ENSG00000171501
0,67	0,65	0,74	ENSG00000171496
1,14	0,97	#N/D	ENSG00000165202
8,39	0,46	0,56	ENSG00000280094
#N/D	#N/D	#N/D	ENSG00000173679
#N/D	#N/D	#N/D	ENSG00000171481
15,64	0,33	0,44	ENSG00000136939
1,14	0,97	#N/D	ENSG00000171459
1,44	0,68	0,77	ENSG00000148215
1,51	0,00	0,00	ENSG00000136940
2,35	0,30	0,41	ENSG00000165204
1,06	0,46	0,57	ENSG00000056586
0,64	0,00	0,00	ENSG00000186130
1,14	0,17	0,26	ENSG00000171448
0,93	0,36	0,47	ENSG00000011454
1,69	0,03	0,05	ENSG00000188394
1,06	0,59	0,69	ENSG00000165209
0,63	0,05	0,09	ENSG00000148204
0,80	0,01	0,01	ENSG00000119522
1,18	0,14	0,23	ENSG00000106689
1,47	0,00	0,00	ENSG00000119408
0,80	0,01	0,01	ENSG00000136930
#N/D	#N/D	#N/D	ENSG00000180264
0,99	1,00	1,00	ENSG00000136931
0,33	0,00	0,00	ENSG00000148200
0,90	0,21	0,31	ENSG00000185585
0,86	0,56	0,66	ENSG00000136918
0,98	0,79	0,86	ENSG00000136942
0,86	0,09	0,16	ENSG00000136950
0,82	0,02	0,04	ENSG00000136935
1,12	0,20	0,30	ENSG00000173611
0,78	0,00	0,01	ENSG00000119414
0,73	0,00	0,00	ENSG00000136933
4,04	0,00	0,00	ENSG00000044574
1,17	0,05	0,09	ENSG00000165219
0,95	0,53	0,64	ENSG00000119487
0,61	0,00	0,00	ENSG00000167081
0,42	0,00	0,00	ENSG00000196814
1,14	0,97	0,98	ENSG00000279137
0,98	0,82	0,89	ENSG00000136944
1,26	0,01	0,01	ENSG00000169155
1,35	0,00	0,00	ENSG00000177125
0,90	0,30	0,41	ENSG00000136828
0,45	0,00	0,00	ENSG00000136859
0,84	0,12	0,20	ENSG00000136895
1,06	0,56	0,66	ENSG00000136856

1,16	0,22	0,32	ENSG00000196152
0,74	0,00	0,00	ENSG00000197958
1,28	0,00	0,01	ENSG00000148356
1,49	0,00	0,00	ENSG00000136830
0,96	0,64	0,73	ENSG00000136854
1,03	0,78	0,85	ENSG00000187024
1,52	0,00	0,00	ENSG00000160401
0,62	0,39	0,51	ENSG00000167094
0,69	0,00	0,00	ENSG00000160404
1,39	0,15	0,23	ENSG00000095370
1,22	0,01	0,03	ENSG00000136807
0,92	0,31	0,43	ENSG00000136877
1,13	0,14	0,22	ENSG00000106991
1,12	0,20	0,30	ENSG00000106992
0,87	0,10	0,17	ENSG00000257524
1,79	0,00	0,00	ENSG00000160408
0,96	0,63	0,72	ENSG00000136840
1,19	0,11	0,19	ENSG00000167103
1,18	0,05	0,09	ENSG00000136908
0,83	0,02	0,05	ENSG00000167106
0,93	0,45	0,56	ENSG00000171169
1,26	0,01	0,02	ENSG00000148339
0,70	0,00	0,00	ENSG00000148334
0,00	0,00	0,00	ENSG00000148346
0,61	0,00	0,00	ENSG00000171159
0,81	0,01	0,02	ENSG00000148337
2,19	0,00	0,00	ENSG00000106976
2,01	0,00	0,00	ENSG00000167110
1,96	0,00	0,00	ENSG00000175854
0,88	0,12	0,20	ENSG00000167112
1,16	0,08	0,15	ENSG00000167113
0,63	0,00	0,00	ENSG00000167114
1,27	0,00	0,01	ENSG00000167118
1,11	0,19	0,29	ENSG00000167123
1,27	0,00	0,01	ENSG00000136811
1,12	0,16	0,26	ENSG00000119392
0,64	0,00	0,00	ENSG00000197694
0,89	0,16	0,25	ENSG00000119333
0,75	0,00	0,00	ENSG00000119335
0,44	0,00	0,00	ENSG00000160447
1,50	0,00	0,00	ENSG00000160446
1,00	0,98	0,99	ENSG00000160445
0,96	0,62	0,72	ENSG00000107021
0,72	0,00	0,00	ENSG00000167136
0,90	0,23	0,33	ENSG00000198917
1,51	0,00	0,00	ENSG00000286112
1,48	0,00	0,00	ENSG00000171097
1,92	0,00	0,00	ENSG00000136802

1,07	0,82	0,90	ENSG00000175287
0,13	0,46	#N/D	ENSG00000251184
0,74	0,00	0,00	ENSG00000175283
0,60	0,00	0,00	ENSG00000095319
0,81	0,01	0,02	ENSG00000148341
1,56	0,00	0,00	ENSG00000148343
1,08	0,39	0,50	ENSG00000167130
1,18	0,04	0,08	ENSG00000095321
0,82	0,01	0,02	ENSG00000119383
3,96	0,00	0,00	ENSG00000235007
0,70	0,00	0,00	ENSG00000188483
1,02	0,80	0,88	ENSG00000148335
0,13	0,46	0,56	ENSG00000179058
1,13	0,13	0,21	ENSG00000148331
0,68	0,00	0,00	ENSG00000167157
0,05	0,00	0,00	ENSG00000148344
0,93	0,43	0,55	ENSG00000136816
0,62	0,00	0,00	ENSG00000136827
0,85	0,05	0,10	ENSG00000136819
1,00	0,99	1,00	ENSG00000136878
0,65	0,00	0,00	ENSG00000187239
1,25	0,01	0,01	ENSG00000148358
0,84	0,03	0,05	ENSG00000107130
0,86	0,25	0,36	ENSG00000148357
1,12	0,17	0,26	ENSG00000130707
0,94	0,46	0,57	ENSG00000107164
0,57	0,01	0,01	ENSG00000130711
0,77	0,00	0,00	ENSG00000130713
0,83	0,02	0,04	ENSG00000097007
#N/D	#N/D	#N/D	ENSG00000288570
0,59	0,00	0,01	ENSG00000188710
1,47	0,00	0,00	ENSG00000130720
0,28	0,00	0,00	ENSG00000050555
4,17	0,00	0,00	ENSG00000126878
1,29	0,00	0,00	ENSG00000126883
0,55	0,00	0,00	ENSG00000126882
0,60	0,00	0,00	ENSG00000160539
0,90	0,17	0,27	ENSG00000288701
#N/D	#N/D	#N/D	ENSG00000291303
#N/D	#N/D	#N/D	ENSG00000288841
0,66	0,00	0,00	ENSG00000130714
1,28	0,01	0,01	ENSG00000130717
1,60	0,51	0,62	ENSG00000283526
0,87	0,10	0,16	ENSG00000107263
0,79	0,00	0,01	ENSG00000160563
0,33	0,00	0,00	ENSG00000196358
1,32	0,00	0,00	ENSG00000107290
1,22	0,03	0,07	ENSG00000125482

22,89	0,26	0,37	ENSG00000188523
0,60	0,47	0,58	ENSG00000125492
0,72	0,00	0,00	ENSG00000125485
0,94	0,44	0,55	ENSG00000125484
2,55	0,01	0,02	ENSG00000165695
1,88	0,00	0,00	ENSG00000165698
1,28	0,00	0,01	ENSG00000165699
22,89	0,26	0,37	ENSG00000165702
1,06	0,49	0,60	ENSG00000148308
0,70	0,02	0,05	ENSG00000170835
1,10	0,25	0,35	ENSG00000160271
0,45	0,00	0,00	ENSG00000285245
0,70	0,00	0,00	ENSG00000148288
#N/D	#N/D	#N/D	ENSG00000171102
#N/D	#N/D	#N/D	ENSG00000175164
1,34	0,00	0,00	ENSG00000148296
1,04	0,66	0,75	ENSG00000148297
1,16	0,06	0,11	ENSG00000148303
0,91	0,31	0,43	ENSG00000148290
0,44	0,00	0,00	ENSG00000148291
1,53	0,00	0,00	ENSG00000148248
1,17	0,30	0,42	ENSG00000198870
0,86	0,08	0,14	ENSG00000148300
0,96	0,67	0,76	ENSG00000160323
2,46	0,00	0,00	ENSG00000160325
0,13	0,00	0,00	ENSG00000160326
#N/D	#N/D	#N/D	ENSG00000187616
3,16	0,01	0,02	ENSG00000197859
#N/D	#N/D	#N/D	ENSG00000196990
1,14	0,97	0,98	ENSG00000123454
1,14	0,97	0,98	ENSG00000123453
1,03	0,76	0,84	ENSG00000160293
0,79	0,01	0,01	ENSG00000235106
0,89	0,14	0,22	ENSG00000169925
0,70	0,00	0,00	ENSG00000196363
22,89	0,26	0,37	ENSG00000279929
1,25	0,01	0,01	ENSG00000186350
1,24	0,01	0,02	ENSG00000130635
1,14	0,97	#N/D	ENSG00000160339
0,99	1,00	1,00	ENSG00000085265
#N/D	#N/D	#N/D	ENSG00000279935
1,62	0,00	0,00	ENSG00000130558
0,69	0,00	0,00	ENSG00000196422
1,58	0,00	0,01	ENSG00000160345
0,65	0,00	0,00	ENSG00000122140
8,39	0,46	0,56	ENSG00000160349
#N/D	#N/D	#N/D	ENSG00000122136
0,03	0,17	0,26	ENSG00000122133

1,14	0,97	#N/D	ENSG00000236543
#N/D	#N/D	#N/D	ENSG00000204007
1,14	0,97	#N/D	ENSG00000148386
#N/D	#N/D	#N/D	ENSG00000165643
2,85	0,00	0,00	ENSG00000107147
1,16	0,07	0,12	ENSG00000130559
0,91	0,29	0,41	ENSG00000130560
0,87	0,08	0,14	ENSG00000148411
1,15	0,08	0,14	ENSG00000238227
8,39	0,46	0,56	ENSG00000107187
0,85	0,05	0,09	ENSG00000165661
0,66	0,51	0,62	ENSG00000260220
#N/D	#N/D	#N/D	ENSG00000279813
1,48	0,00	0,00	ENSG00000160360
1,74	0,00	0,00	ENSG00000213221
0,67	0,00	0,01	ENSG00000289701
2,80	0,20	0,30	ENSG00000187796
1,03	0,75	0,84	ENSG00000165684
0,72	0,00	0,00	ENSG00000165689
1,20	0,02	0,05	ENSG00000165688
0,67	0,00	0,00	ENSG00000148384
2,02	0,00	0,00	ENSG00000148396
0,83	0,03	0,05	ENSG00000148400
1,00	0,99	0,99	ENSG00000172889
1,38	0,00	0,00	ENSG00000169692
0,59	0,00	0,00	ENSG00000165716
37,39	0,19	0,29	ENSG00000187922
#N/D	#N/D	#N/D	ENSG00000204003
1,14	0,97	#N/D	ENSG00000267206
#N/D	#N/D	#N/D	ENSG00000204001
#N/D	#N/D	#N/D	ENSG00000177984
1,52	0,00	0,00	ENSG00000244187
0,39	0,00	0,01	ENSG00000272896
1,45	0,06	0,10	ENSG00000213213
1,00	0,99	0,99	ENSG00000196642
1,99	0,00	0,00	ENSG00000232434
1,35	0,00	0,00	ENSG00000054148
2,59	0,00	0,00	ENSG00000177943
1,43	0,00	0,00	ENSG00000107223
1,01	0,91	0,97	ENSG00000127191
1,58	0,00	0,00	ENSG00000159069
1,29	0,42	0,54	ENSG00000176919
1,44	0,68	0,77	ENSG00000184925
0,99	1,00	1,00	ENSG00000107317
#N/D	#N/D	#N/D	ENSG00000284341
15,64	0,33	0,44	ENSG00000214402
0,90	0,25	0,36	ENSG00000148362
0,58	0,09	0,16	ENSG00000169583

1,13	0,14	0,22	ENSG00000107331
0,35	0,05	0,09	ENSG00000180549
59,15	0,14	0,22	ENSG00000279073
1,09	0,30	0,42	ENSG00000107281
0,82	0,74	0,82	ENSG00000054179
0,79	0,00	0,01	ENSG00000186193
0,94	0,44	0,56	ENSG00000197355
1,23	0,01	0,02	ENSG00000177239
1,08	0,35	0,46	ENSG00000176978
1,29	0,63	0,73	ENSG00000176884
1,14	0,97	0,98	ENSG00000184709
1,14	0,97	0,98	ENSG00000261793
#N/D	#N/D	#N/D	ENSG00000185863
1,24	0,01	0,02	ENSG00000176248
0,88	0,13	0,21	ENSG00000176101
1,13	0,15	0,24	ENSG00000176058
0,87	0,09	0,15	ENSG00000187713
0,95	0,55	0,66	ENSG00000188566
1,14	0,97	0,98	ENSG00000284976
0,52	0,00	0,00	ENSG00000212864
0,20	0,00	0,00	ENSG00000197191
0,46	0,00	0,00	ENSG00000233198
0,64	0,01	0,01	ENSG00000198569
0,99	0,89	0,96	ENSG00000188229
0,47	0,02	0,05	ENSG00000188163
0,95	0,76	0,84	ENSG00000197768
0,94	0,48	0,59	ENSG00000188986
0,19	0,00	0,00	ENSG00000198113
0,29	0,00	0,00	ENSG00000198435
1,38	0,00	0,00	ENSG00000187609
1,34	0,43	0,54	ENSG00000188747
1,44	0,16	0,25	ENSG00000188833
0,99	0,91	0,97	ENSG00000165802
1,31	0,08	0,14	ENSG00000130653
1,05	0,60	0,69	ENSG00000182154
2,13	0,00	0,00	ENSG00000148399
0,92	0,32	0,43	ENSG00000165724
1,04	0,68	0,77	ENSG00000197070
0,87	0,08	0,14	ENSG00000181090
0,79	0,72	0,80	ENSG00000148408
6,99	0,05	0,10	ENSG00000261456
0,79	0,01	0,02	ENSG00000015171
1,32	0,00	0,00	ENSG00000151240
0,89	0,14	0,22	ENSG00000107929
0,95	0,55	0,65	ENSG00000107937
1,14	0,97	#N/D	ENSG00000148377
5,73	0,00	0,00	ENSG00000067064
1,56	0,00	0,00	ENSG00000047056

22,89	0,26	0,37	ENSG00000185736
1,34	0,00	0,00	ENSG00000067057
0,82	0,01	0,03	ENSG00000107959
1,68	0,00	0,00	ENSG00000067082
0,49	0,16	0,25	ENSG00000165568
2,74	0,00	0,00	ENSG00000187134
3,68	0,00	0,00	ENSG00000151632
1,88	0,00	0,00	ENSG00000196139
0,57	0,36	0,47	ENSG00000264006
0,98	0,97	0,99	ENSG00000198610
#N/D	#N/D	#N/D	ENSG00000280450
#N/D	#N/D	#N/D	ENSG00000178473
0,16	0,00	0,01	ENSG00000178462
0,81	0,01	0,03	ENSG00000173848
1,14	0,97	#N/D	ENSG00000178372
1,14	0,97	#N/D	ENSG00000178363
0,30	0,00	0,00	ENSG00000196372
1,07	0,43	0,55	ENSG00000108021
1,04	0,65	0,74	ENSG00000057608
1,13	0,29	0,41	ENSG00000134461
0,96	0,59	0,69	ENSG00000134452
0,52	0,00	0,00	ENSG00000134470
8,39	0,46	#N/D	ENSG00000134460
0,77	0,00	0,01	ENSG00000134453
0,79	0,00	0,01	ENSG00000170525
0,02	0,12	0,19	ENSG00000065675
0,89	0,41	0,53	ENSG00000198879
0,13	0,46	0,56	ENSG00000278982
0,27	0,22	0,32	ENSG00000123243
8,39	0,46	0,56	ENSG00000151655
1,27	0,03	0,06	ENSG00000151657
0,90	0,20	0,30	ENSG00000165629
0,80	0,03	0,05	ENSG00000165632
28,42	0,00	0,00	ENSG00000107485
#N/D	#N/D	#N/D	ENSG00000279575
0,24	0,00	0,00	ENSG00000048740
#N/D	#N/D	#N/D	ENSG00000280086
0,39	0,00	0,00	ENSG00000148429
0,85	0,08	0,14	ENSG00000134463
0,09	0,00	0,00	ENSG00000148426
1,42	0,00	0,00	ENSG00000151461
0,86	0,06	0,11	ENSG00000181192
0,82	0,04	0,08	ENSG00000065665
0,91	0,29	0,40	ENSG00000165609
0,67	0,00	0,00	ENSG00000151465
0,92	0,32	0,43	ENSG00000183049
0,64	0,00	0,00	ENSG00000151468
0,75	0,00	0,00	ENSG00000123240

0,79	0,01	0,02	ENSG00000065328
0,36	0,35	0,46	ENSG00000165623
0,52	0,00	0,00	ENSG00000107537
0,77	0,00	0,00	ENSG00000086475
0,76	0,02	0,04	ENSG00000165626
1,11	0,29	0,41	ENSG00000165630
0,53	0,00	0,00	ENSG00000151474
0,67	0,00	0,00	ENSG00000065809
0,85	0,66	0,75	ENSG00000185267
0,93	0,38	0,49	ENSG00000284024
0,94	0,54	0,65	ENSG00000187522
0,76	0,00	0,01	ENSG00000152455
1,03	0,72	0,80	ENSG00000152457
8,39	0,46	0,56	ENSG00000197889
0,21	0,00	0,01	ENSG00000152463
0,72	0,02	0,05	ENSG00000176244
0,90	0,33	0,44	ENSG00000152464
1,32	0,00	0,00	ENSG00000152465
0,98	0,85	0,92	ENSG00000148468
2,29	0,00	0,00	ENSG00000077943
0,65	0,00	0,00	ENSG00000148481
0,51	0,00	0,00	ENSG00000165983
0,44	0,00	0,00	ENSG00000165985
1,26	0,01	0,01	ENSG00000148484
0,44	0,00	0,00	ENSG00000107611
0,45	0,00	0,00	ENSG00000107614
1,96	0,00	0,00	ENSG00000026025
0,22	0,14	0,22	ENSG00000148488
0,59	0,00	0,00	ENSG00000165996
0,97	0,76	0,84	ENSG00000136738
0,27	0,02	0,04	ENSG00000148483
1,14	0,97	0,98	ENSG00000260314
1,14	0,97	#N/D	ENSG00000148482
0,69	0,03	0,05	ENSG00000165995
0,72	0,01	0,01	ENSG00000241058
0,87	0,12	0,19	ENSG00000165997
0,13	0,46	0,56	ENSG00000204740
71,13	0,00	0,00	ENSG00000120594
0,50	0,25	0,36	ENSG00000078114
0,54	0,00	0,00	ENSG00000180592
0,78	0,00	0,01	ENSG00000078403
0,71	0,00	0,00	ENSG00000136770
1,14	0,97	0,98	ENSG00000223601
0,35	0,05	0,09	ENSG00000279631
0,64	0,00	0,00	ENSG00000148444
0,57	0,00	0,00	ENSG00000269897
0,34	0,00	0,00	ENSG00000168283
0,07	0,31	0,43	ENSG00000077327

0,74	0,00	0,00	ENSG00000150867
0,83	0,64	0,74	ENSG00000279819
#N/D	#N/D	#N/D	ENSG00000165309
1,10	0,32	0,43	ENSG00000148450
1,14	0,97	#N/D	ENSG00000168267
22,89	0,26	0,37	ENSG00000179133
1,27	0,03	0,06	ENSG00000165312
1,02	0,84	0,91	ENSG00000120549
1,46	0,26	0,36	ENSG00000279029
1,22	0,02	0,03	ENSG00000107863
2,47	0,04	0,08	ENSG00000099256
0,70	0,55	0,65	ENSG00000151023
0,53	0,00	0,00	ENSG00000185875
8,39	0,46	0,56	ENSG00000151025
#N/D	#N/D	#N/D	ENSG00000095777
1,14	0,97	#N/D	ENSG00000136750
22,89	0,26	0,37	ENSG00000077420
0,60	0,47	0,58	ENSG00000279212
0,61	0,00	0,00	ENSG00000148459
0,98	0,77	0,85	ENSG00000136754
0,66	0,00	0,00	ENSG00000107890
1,00	0,98	0,99	ENSG00000136758
1,06	0,53	0,63	ENSG00000120539
0,91	0,28	0,39	ENSG00000107897
1,14	0,97	#N/D	ENSG00000182077
1,61	0,00	0,00	ENSG00000099246
0,09	0,00	0,00	ENSG00000150051
0,32	0,00	0,00	ENSG00000169126
0,23	0,00	0,00	ENSG00000150054
0,75	0,00	0,00	ENSG00000095787
3,03	0,00	0,00	ENSG00000095739
#N/D	#N/D	#N/D	ENSG00000120563
1,32	0,00	0,00	ENSG00000197321
2,09	0,00	0,00	ENSG00000165757
0,69	0,00	0,00	ENSG00000107951
0,25	0,00	0,00	ENSG00000107968
#N/D	#N/D	#N/D	ENSG00000151033
1,13	0,23	0,33	ENSG00000183621
1,10	0,22	0,32	ENSG00000148516
0,98	0,83	0,90	ENSG00000165322
0,73	0,00	0,00	ENSG00000170759
0,70	0,00	0,00	ENSG00000120616
0,43	0,00	0,00	ENSG00000216937
1,11	0,19	0,28	ENSG00000150093
0,84	0,03	0,06	ENSG00000099250
0,70	0,00	0,00	ENSG00000148498
0,76	0,00	0,00	ENSG00000108094
0,45	0,00	0,00	ENSG00000095794

0,66	0,00	0,00	ENSG00000108100
0,99	1,00	1,00	ENSG00000177291
0,41	0,00	0,00	ENSG00000177283
1,14	0,97	0,98	ENSG00000148513
1,09	0,39	0,50	ENSG00000198105
0,78	0,02	0,03	ENSG00000175395
0,85	0,09	0,15	ENSG00000189180
0,55	0,00	0,00	ENSG00000075407
5,46	0,00	0,00	ENSG00000283930
0,35	0,00	0,00	ENSG00000196693
0,87	0,10	0,17	ENSG00000165733
0,86	0,26	0,37	ENSG00000165731
0,86	0,09	0,16	ENSG00000169826
1,91	0,01	0,01	ENSG00000198915
#N/D	#N/D	#N/D	ENSG00000150201
0,91	0,26	0,37	ENSG00000169813
0,68	0,16	0,25	ENSG00000243660
0,35	0,00	0,00	ENSG00000196793
0,64	0,01	0,02	ENSG00000198298
0,85	0,10	0,17	ENSG00000169740
0,20	0,00	0,00	ENSG00000107562
22,89	0,26	0,37	ENSG00000187783
1,15	0,21	0,31	ENSG00000107551
2,54	0,00	0,00	ENSG00000165507
0,59	0,00	0,00	ENSG00000165512
15,64	0,33	0,44	ENSG00000256574
8,39	0,46	0,56	ENSG00000012779
1,12	0,22	0,32	ENSG00000165406
0,67	0,01	0,01	ENSG00000172671
0,74	0,00	0,00	ENSG00000172661
1,00	0,99	0,99	ENSG00000188234
0,86	0,08	0,15	ENSG00000265354
1,07	0,39	0,51	ENSG00000266412
8,39	0,46	0,56	ENSG00000263639
2,71	0,36	0,48	ENSG00000274209
0,03	0,00	0,00	ENSG00000264230
0,18	0,10	0,16	ENSG00000204174
8,71	0,03	0,06	ENSG00000204175
1,40	0,08	0,14	ENSG00000204176
1,14	0,97	0,98	ENSG00000204179
0,99	1,00	#N/D	ENSG00000266524
1,14	0,97	#N/D	ENSG00000263761
22,89	0,26	0,37	ENSG00000265203
1,17	0,33	0,44	ENSG00000265763
0,12	0,00	0,01	ENSG00000265190
#N/D	#N/D	#N/D	ENSG00000189090
0,82	0,06	0,11	ENSG00000204172
1,59	0,00	0,00	ENSG00000277758

1,14	0,97	0,98	ENSG00000264717
#N/D	#N/D	#N/D	ENSG00000276430
0,97	0,94	0,98	ENSG00000170324
0,79	0,01	0,02	ENSG00000107643
1,04	0,88	0,94	ENSG00000279822
1,18	0,08	0,14	ENSG00000128805
3,26	0,13	0,21	ENSG00000128815
#N/D	#N/D	#N/D	ENSG00000165383
1,34	0,00	0,00	ENSG00000165633
#N/D	#N/D	#N/D	ENSG00000172538
1,14	0,97	#N/D	ENSG00000204161
#N/D	#N/D	#N/D	ENSG00000177354
0,01	0,06	0,11	ENSG00000165606
0,70	0,00	0,00	ENSG00000225830
#N/D	#N/D	#N/D	ENSG00000070748
#N/D	#N/D	#N/D	ENSG00000187714
1,14	0,97	#N/D	ENSG00000178645
0,36	0,35	0,46	ENSG00000197444
0,76	0,00	0,01	ENSG00000227345
1,21	0,07	0,12	ENSG00000204152
0,78	0,02	0,04	ENSG00000178440
0,97	0,77	0,85	ENSG00000204149
0,81	0,02	0,04	ENSG00000099290
0,82	0,35	0,47	ENSG00000188611
0,56	0,00	0,00	ENSG00000198964
1,17	0,57	0,68	ENSG00000279863
0,74	0,01	0,02	ENSG00000204147
3,26	0,13	0,21	ENSG00000148584
29,57	0,00	0,00	ENSG00000185532
1,08	0,37	0,49	ENSG00000177613
2,82	0,00	0,00	ENSG00000107984
0,13	0,46	0,56	ENSG00000165471
1,14	0,97	#N/D	ENSG00000150275
0,90	0,24	0,34	ENSG00000122952
0,88	0,26	0,37	ENSG00000151151
0,72	0,00	0,00	ENSG00000122873
0,52	0,00	0,00	ENSG00000072401
0,39	0,00	0,00	ENSG00000108064
1,43	0,00	0,00	ENSG00000122870
0,13	0,46	#N/D	ENSG00000165443
0,07	0,00	0,01	ENSG00000148541
0,32	0,00	0,00	ENSG00000165449
1,14	0,97	#N/D	ENSG00000227877
0,77	0,00	0,00	ENSG00000108091
0,20	0,00	0,00	ENSG00000151150
0,91	0,23	0,33	ENSG00000170312
0,47	0,00	0,00	ENSG00000072422
15,64	0,33	#N/D	ENSG00000196932

0,53	0,58	0,68	ENSG00000183346
2,17	0,00	0,00	ENSG00000150347
0,00	0,01	0,02	ENSG00000182010
0,08	0,00	0,00	ENSG00000138311
0,04	0,20	0,30	ENSG00000285837
0,76	0,00	0,01	ENSG00000181915
0,38	0,03	0,06	ENSG00000122877
1,07	0,48	0,59	ENSG00000148572
0,66	0,00	0,00	ENSG00000171988
0,96	0,66	0,75	ENSG00000165476
1,89	0,45	0,56	ENSG00000183230
#N/D	#N/D	#N/D	ENSG00000198739
#N/D	#N/D	#N/D	ENSG00000289325
0,82	0,30	0,41	ENSG00000108176
0,91	0,31	0,42	ENSG00000096717
1,23	0,01	0,03	ENSG00000148634
0,46	0,00	0,00	ENSG00000138347
0,07	0,31	0,43	ENSG00000179774
1,22	0,14	0,23	ENSG00000108187
0,74	0,00	0,00	ENSG00000096746
0,98	0,84	0,91	ENSG00000204130
0,68	0,00	0,00	ENSG00000138346
1,07	0,56	0,66	ENSG00000122912
0,96	0,74	0,82	ENSG00000138336
0,80	0,01	0,02	ENSG00000060339
0,30	0,01	0,01	ENSG00000165730
1,16	0,09	0,15	ENSG00000107625
0,75	0,00	0,00	ENSG00000165732
1,01	0,93	0,98	ENSG00000198954
0,70	0,02	0,04	ENSG00000122862
0,49	0,00	0,00	ENSG00000122958
0,64	0,00	0,00	ENSG00000156502
0,54	0,00	0,00	ENSG00000156510
1,18	0,04	0,07	ENSG00000156515
1,05	0,86	0,93	ENSG00000075073
0,30	0,00	0,00	ENSG00000099282
#N/D	#N/D	#N/D	ENSG00000122859
0,50	0,00	0,00	ENSG00000171224
1,75	0,00	0,00	ENSG00000197467
0,91	0,35	0,46	ENSG00000099284
0,52	0,00	0,00	ENSG00000042286
0,65	0,00	0,00	ENSG00000156521
1,34	0,00	0,00	ENSG00000079332
0,86	0,07	0,12	ENSG00000180817
0,51	0,42	0,54	ENSG00000148734
0,79	0,01	0,02	ENSG00000172731
1,54	0,00	0,00	ENSG00000148730
1,58	0,01	0,02	ENSG00000156574

1,71	0,00	0,01	ENSG00000280401
0,54	0,00	0,00	ENSG00000107719
0,67	0,65	0,74	ENSG00000180644
0,96	0,68	0,77	ENSG00000138316
#N/D	#N/D	#N/D	ENSG00000166220
0,49	0,00	0,00	ENSG00000166224
0,55	0,00	0,00	ENSG00000166228
0,21	0,00	0,00	ENSG00000107731
0,72	0,02	0,03	ENSG00000198246
1,15	0,74	0,82	ENSG00000279406
1,21	0,44	0,56	ENSG00000107736
37,39	0,19	0,29	ENSG00000214688
0,79	0,01	0,02	ENSG00000107738
0,94	0,41	0,53	ENSG00000197746
1,09	0,29	0,40	ENSG00000122863
0,32	0,00	0,00	ENSG00000107742
0,88	0,16	0,26	ENSG00000138303
0,81	0,02	0,03	ENSG00000166295
0,77	0,00	0,00	ENSG00000168209
0,83	0,03	0,06	ENSG00000148719
0,84	0,04	0,07	ENSG00000107745
0,79	0,01	0,01	ENSG00000156026
0,13	0,46	0,56	ENSG00000280166
0,28	0,04	0,08	ENSG00000279502
1,14	0,97	0,98	ENSG00000138315
0,05	0,24	0,35	ENSG00000138308
1,85	0,00	0,00	ENSG00000122884
0,81	0,36	0,47	ENSG00000166321
1,10	0,26	0,37	ENSG00000122882
1,13	0,22	0,32	ENSG00000138286
0,94	0,46	0,57	ENSG00000213551
0,76	0,00	0,00	ENSG00000182180
0,12	0,00	0,00	ENSG00000156042
0,82	0,02	0,04	ENSG00000138279
0,71	0,05	0,10	ENSG00000166343
0,74	0,00	0,00	ENSG00000107758
0,57	0,00	0,00	ENSG00000166348
1,14	0,97	#N/D	ENSG00000177791
0,17	0,00	0,00	ENSG00000166317
0,68	0,00	0,00	ENSG00000172650
1,89	0,00	0,00	ENSG00000279088
1,85	0,00	0,00	ENSG00000176986
2,63	0,00	0,00	ENSG00000279689
1,11	0,25	0,36	ENSG00000196968
0,87	0,23	0,33	ENSG00000172586
2,04	0,00	0,00	ENSG00000214655
5,54	0,02	0,04	ENSG00000280238
1,76	0,00	0,00	ENSG00000272916

0,65	0,00	0,00	ENSG00000166507
0,99	0,92	0,98	ENSG00000148660
0,77	0,00	0,00	ENSG00000122861
0,95	0,54	0,64	ENSG00000035403
0,85	0,06	0,10	ENSG00000185009
0,91	0,26	0,37	ENSG00000156110
1,01	0,91	0,97	ENSG00000156650
1,14	0,97	#N/D	ENSG00000188716
0,07	0,31	0,43	ENSG00000079393
15,64	0,33	0,44	ENSG00000293542
1,14	0,97	0,98	ENSG00000293543
1,26	0,01	0,01	ENSG00000156671
1,25	0,01	0,01	ENSG00000165637
1,18	0,25	0,36	ENSG00000165644
1,92	0,00	0,00	ENSG00000165655
0,51	0,00	0,00	ENSG00000148655
0,30	0,01	0,01	ENSG00000279814
8,39	0,46	#N/D	ENSG00000278992
1,10	0,26	0,36	ENSG00000156113
1,10	0,24	0,34	ENSG00000151208
1,19	0,03	0,07	ENSG00000148606
0,95	0,56	0,66	ENSG00000138326
1,37	0,00	0,00	ENSG00000108175
0,81	0,01	0,02	ENSG00000108179
1,15	0,09	0,16	ENSG00000165424
1,43	0,22	0,32	ENSG00000253626
8,71	0,03	0,06	ENSG00000185303
22,89	0,26	#N/D	ENSG00000122852
0,87	0,20	0,30	ENSG00000280355
0,82	0,19	0,28	ENSG00000188199
0,54	0,00	0,00	ENSG00000228570
8,39	0,46	0,56	ENSG00000133661
0,91	0,39	0,50	ENSG00000133678
1,50	0,30	0,42	ENSG00000279399
0,79	0,31	0,43	ENSG00000189129
1,11	0,18	0,28	ENSG00000122359
1,14	0,97	#N/D	ENSG00000279359
0,13	0,46	#N/D	ENSG00000151224
1,14	0,97	#N/D	ENSG00000170788
1,14	0,97	0,98	ENSG00000133665
0,16	0,00	0,00	ENSG00000122378
0,84	0,03	0,06	ENSG00000108219
3,77	0,04	0,07	ENSG00000178217
#N/D	#N/D	#N/D	ENSG00000185737
0,87	0,08	0,14	ENSG00000165678
#N/D	#N/D	#N/D	ENSG00000188373
1,14	0,97	#N/D	ENSG00000148600
#N/D	#N/D	#N/D	ENSG00000204033

#N/D	#N/D	#N/D	ENSG00000148604
#N/D	#N/D	#N/D	ENSG00000148602
0,88	0,13	0,21	ENSG00000107771
#N/D	#N/D	#N/D	ENSG00000279910
1,14	0,97	#N/D	ENSG00000280134
13,67	0,00	0,00	ENSG00000182771
0,98	0,82	0,89	ENSG00000062650
1,14	0,97	#N/D	ENSG00000122375
1,14	0,97	0,98	ENSG00000289258
1,21	0,50	0,61	ENSG00000122367
0,70	0,00	0,00	ENSG00000107779
1,36	0,00	0,01	ENSG00000173269
0,98	0,97	0,98	ENSG00000281974
0,80	0,05	0,09	ENSG00000173267
1,79	0,00	0,00	ENSG00000148671
1,20	0,64	0,73	ENSG00000188100
0,99	0,88	0,94	ENSG00000148672
0,73	0,00	0,00	ENSG00000122376
0,77	0,03	0,06	ENSG00000184923
0,38	0,00	0,00	ENSG00000214562
0,64	0,00	0,00	ENSG00000107789
1,36	0,00	0,00	ENSG00000198682
0,63	0,00	0,00	ENSG00000138138
0,75	0,44	0,55	ENSG00000227268
0,97	0,77	0,85	ENSG00000171862
#N/D	#N/D	#N/D	ENSG00000289051
27,86	0,00	0,00	ENSG00000184719
1,14	0,97	#N/D	ENSG00000204022
#N/D	#N/D	#N/D	ENSG00000182333
1,14	0,97	#N/D	ENSG00000204021
0,05	0,24	0,35	ENSG00000204020
#N/D	#N/D	#N/D	ENSG00000173239
0,49	0,08	0,15	ENSG00000152766
0,79	0,01	0,02	ENSG00000138134
0,97	0,79	0,87	ENSG00000107796
0,89	0,22	0,32	ENSG00000026103
1,02	0,95	0,98	ENSG00000138135
0,95	0,59	0,69	ENSG00000107798
1,05	0,59	0,69	ENSG00000119922
0,58	0,00	0,00	ENSG00000119917
0,13	0,46	0,56	ENSG00000204010
1,37	0,00	0,00	ENSG00000185745
0,52	0,00	0,00	ENSG00000152778
0,02	0,12	0,19	ENSG00000152779
0,40	0,00	0,00	ENSG00000152782
0,63	0,00	0,00	ENSG00000138182
0,98	0,85	0,92	ENSG00000148680
0,89	0,18	0,28	ENSG00000148688

0,01	0,09	0,15	ENSG00000148677
0,44	0,00	0,00	ENSG00000180628
0,40	0,00	0,00	ENSG00000165338
0,56	0,00	0,00	ENSG00000119938
0,94	0,42	0,54	ENSG00000107854
0,61	0,06	0,11	ENSG00000174721
0,99	0,93	0,98	ENSG00000095564
1,04	0,81	0,89	ENSG00000107864
0,89	0,19	0,29	ENSG00000198060
0,73	0,00	0,00	ENSG00000119912
0,87	0,10	0,17	ENSG00000138160
1,06	0,58	0,68	ENSG00000152804
0,56	0,00	0,00	ENSG00000138190
1,38	0,51	0,62	ENSG00000187553
44,64	0,17	0,26	ENSG00000095596
0,94	0,41	0,53	ENSG00000138119
0,63	0,00	0,00	ENSG00000138180
1,14	0,97	0,98	ENSG00000186188
1,04	0,86	0,93	ENSG00000138207
3,56	0,02	0,05	ENSG00000095464
0,95	0,77	0,85	ENSG00000148690
8,39	0,46	0,56	ENSG00000108231
0,71	0,01	0,01	ENSG00000176273
0,82	0,02	0,04	ENSG00000138193
0,60	0,00	0,00	ENSG00000173145
0,72	0,01	0,03	ENSG00000108239
0,76	0,00	0,00	ENSG00000119969
8,39	0,46	0,56	ENSG00000108242
1,14	0,97	#N/D	ENSG00000276490
0,07	0,31	0,43	ENSG00000165841
1,14	0,97	0,98	ENSG00000138109
1,14	0,97	0,98	ENSG00000138115
1,14	0,97	0,98	ENSG00000173124
2,18	0,00	0,00	ENSG00000107438
0,37	0,00	0,00	ENSG00000095637
1,81	0,00	0,00	ENSG00000059573
1,14	0,13	0,22	ENSG00000119977
0,46	0,00	0,00	ENSG00000138185
#N/D	#N/D	#N/D	ENSG00000270099
0,87	0,77	0,85	ENSG00000188649
0,76	0,01	0,02	ENSG00000107443
0,41	0,00	0,00	ENSG00000177853
1,14	0,97	0,98	ENSG00000095585
#N/D	#N/D	#N/D	ENSG00000107447
#N/D	#N/D	#N/D	ENSG00000197430
0,59	0,31	0,42	ENSG00000095587
1,09	0,30	0,41	ENSG00000077147
0,50	0,01	0,02	ENSG00000155629

0,70	0,00	0,00	ENSG00000196233
1,08	0,75	0,83	ENSG00000187122
0,10	0,00	0,00	ENSG00000269891
1,43	0,00	0,00	ENSG00000213390
0,54	0,00	0,00	ENSG00000165879
1,22	0,04	0,08	ENSG00000181274
1,37	0,00	0,00	ENSG00000052749
1,86	0,00	0,00	ENSG00000171314
1,14	0,18	0,27	ENSG00000171311
1,70	0,00	0,00	ENSG00000171307
1,08	0,36	0,47	ENSG00000155229
1,65	0,00	0,00	ENSG00000165886
0,67	0,65	0,74	ENSG00000165887
0,48	0,00	0,00	ENSG00000241935
0,79	0,17	0,26	ENSG00000249967
0,33	0,01	0,03	ENSG00000203942
0,98	0,84	0,91	ENSG00000171160
1,41	0,00	0,00	ENSG00000155252
0,78	0,01	0,02	ENSG00000119986
0,94	0,48	0,58	ENSG00000155254
1,29	0,00	0,01	ENSG00000155256
0,98	0,99	0,99	ENSG00000120057
0,43	0,00	0,00	ENSG00000155265
0,01	0,06	0,10	ENSG00000095713
0,78	0,02	0,03	ENSG00000166024
0,93	0,81	0,89	ENSG00000138131
0,11	0,00	0,00	ENSG00000119943
0,87	0,10	0,18	ENSG00000107521
1,14	0,97	0,98	ENSG00000289758
1,14	0,97	0,98	ENSG00000172987
30,14	0,22	0,32	ENSG00000119946
0,91	0,26	0,37	ENSG00000120053
1,29	0,74	0,82	ENSG00000119919
1,17	0,08	0,14	ENSG00000155287
0,92	0,73	0,82	ENSG00000285932
1,52	0,00	0,00	ENSG00000198018
1,05	0,57	0,67	ENSG00000119929
1,26	0,01	0,01	ENSG00000014919
0,69	0,00	0,00	ENSG00000023839
1,28	0,00	0,01	ENSG00000107554
8,39	0,46	#N/D	ENSG00000120054
0,89	0,18	0,27	ENSG00000107566
0,95	0,58	0,68	ENSG00000213341
1,06	0,47	0,58	ENSG00000095485
0,61	0,00	0,00	ENSG00000196072
1,14	0,97	#N/D	ENSG00000107593
2,23	0,00	0,00	ENSG00000099194
1,61	0,36	0,47	ENSG00000075290

1,24	0,02	0,04	ENSG00000075826
1,07	0,67	0,76	ENSG00000255339
0,84	0,04	0,08	ENSG00000166136
1,05	0,55	0,66	ENSG00000166135
0,97	0,95	0,98	ENSG00000075891
0,99	0,91	0,98	ENSG00000119906
1,09	0,31	0,43	ENSG00000055950
0,79	0,05	0,09	ENSG00000095539
0,57	0,00	0,00	ENSG00000107815
1,02	0,76	0,84	ENSG00000107816
1,76	0,00	0,00	ENSG00000186862
1,42	0,00	0,00	ENSG00000107819
0,96	0,61	0,71	ENSG00000107821
0,13	0,46	#N/D	ENSG00000107807
30,14	0,22	0,32	ENSG00000138136
1,13	0,15	0,24	ENSG00000166167
1,33	0,01	0,01	ENSG00000166171
0,83	0,03	0,06	ENSG00000166169
0,68	0,00	0,00	ENSG00000107829
0,74	0,69	0,78	ENSG00000107831
0,79	0,01	0,02	ENSG00000107833
1,05	0,58	0,68	ENSG00000198408
1,00	0,98	0,99	ENSG00000120049
0,76	0,00	0,01	ENSG00000120029
0,72	0,00	0,00	ENSG00000166189
1,09	0,32	0,43	ENSG00000198728
0,96	0,66	0,75	ENSG00000148840
0,89	0,16	0,25	ENSG00000166197
1,83	0,15	0,24	ENSG00000119915
2,15	0,20	0,30	ENSG00000107859
1,86	0,00	0,00	ENSG00000107862
0,74	0,00	0,00	ENSG00000077150
0,97	0,90	0,96	ENSG00000059915
1,07	0,59	0,69	ENSG00000107872
0,90	0,21	0,31	ENSG00000107874
0,46	0,00	0,01	ENSG00000120055
0,78	0,03	0,06	ENSG00000138111
1,10	0,22	0,32	ENSG00000138107
1,06	0,46	0,57	ENSG00000107882
0,75	0,00	0,00	ENSG00000171206
1,38	0,00	0,00	ENSG00000138175
0,83	0,18	0,28	ENSG00000156398
1,02	0,85	0,92	ENSG00000166272
0,13	0,46	#N/D	ENSG00000148795
0,59	0,00	0,00	ENSG00000166275
0,76	0,59	0,69	ENSG00000270316
1,14	0,97	0,98	ENSG00000214435
0,90	0,27	0,38	ENSG00000148842

0,78	0,00	0,01	ENSG00000076685
0,13	0,46	#N/D	ENSG00000235376
1,14	0,97	#N/D	ENSG00000148798
0,42	0,00	0,00	ENSG00000156374
1,05	0,65	0,74	ENSG00000148835
0,98	0,79	0,87	ENSG00000173915
1,07	0,40	0,52	ENSG00000148843
1,77	0,00	0,00	ENSG00000138172
0,62	0,20	0,30	ENSG00000185933
0,94	0,82	0,89	ENSG00000183128
0,37	0,01	0,03	ENSG00000107954
1,01	0,90	0,96	ENSG00000107957
0,98	0,83	0,90	ENSG00000107960
0,76	0,00	0,00	ENSG00000065613
0,71	0,00	0,01	ENSG00000065618
0,76	0,04	0,08	ENSG00000156384
0,74	0,57	0,67	ENSG00000197748
1,02	0,80	0,87	ENSG00000148834
0,82	0,26	0,37	ENSG00000065621
0,83	0,03	0,06	ENSG00000148841
0,54	0,01	0,03	ENSG00000120051
1,14	0,97	0,98	ENSG00000156395
15,64	0,33	0,44	ENSG00000108018
1,03	0,69	0,78	ENSG00000108039
0,86	0,08	0,13	ENSG00000148700
1,08	0,36	0,47	ENSG00000119950
1,03	0,72	0,80	ENSG00000119953
0,33	0,00	0,00	ENSG00000138166
0,52	0,00	0,00	ENSG00000108055
0,27	0,00	0,00	ENSG00000203867
0,78	0,01	0,02	ENSG00000150593
0,66	0,00	0,00	ENSG00000214413
0,94	0,48	0,58	ENSG00000108061
8,39	0,46	#N/D	ENSG00000150594
0,70	0,00	0,00	ENSG00000119927
0,04	0,20	0,30	ENSG00000119913
0,43	0,00	0,00	ENSG00000197142
0,75	0,00	0,00	ENSG00000023041
0,80	0,02	0,04	ENSG00000151532
0,45	0,14	0,22	ENSG00000279315
0,90	0,23	0,33	ENSG00000148737
1,14	0,97	#N/D	ENSG00000148702
0,13	0,46	#N/D	ENSG00000197893
1,07	0,46	0,56	ENSG00000165806
1,14	0,97	0,98	ENSG00000148735
0,47	0,00	0,00	ENSG00000198924
0,90	0,21	0,32	ENSG00000196865
0,70	0,04	0,08	ENSG00000043591

1,67	0,00	0,00	ENSG00000165813
0,67	0,65	0,74	ENSG00000095627
2,22	0,24	0,35	ENSG00000165816
2,12	0,00	0,00	ENSG00000169129
2,71	0,36	0,48	ENSG00000279796
1,44	0,00	0,00	ENSG00000099204
1,10	0,26	0,37	ENSG00000151553
0,78	0,01	0,02	ENSG00000165832
5,34	0,00	0,00	ENSG00000107518
0,02	0,00	0,00	ENSG00000151892
1,14	0,97	#N/D	ENSG00000182645
0,13	0,46	#N/D	ENSG00000203837
#N/D	#N/D	#N/D	ENSG00000175535
#N/D	#N/D	#N/D	ENSG00000187021
1,14	0,97	#N/D	ENSG00000266200
1,14	0,97	#N/D	ENSG00000165863
0,44	0,00	0,00	ENSG00000165868
2,12	0,02	0,03	ENSG00000188316
0,64	0,00	0,00	ENSG00000187164
0,53	0,01	0,02	ENSG00000148704
1,14	0,97	#N/D	ENSG00000186795
22,89	0,26	0,37	ENSG00000165646
0,97	0,69	0,78	ENSG00000165650
1,65	0,01	0,03	ENSG00000170370
0,55	0,00	0,00	ENSG00000107560
1,07	0,44	0,55	ENSG00000165669
8,39	0,46	0,56	ENSG00000119973
1,28	0,00	0,01	ENSG00000151893
0,71	0,03	0,06	ENSG00000188613
0,75	0,00	0,00	ENSG00000107581
0,56	0,00	0,00	ENSG00000119979
0,50	0,00	0,00	ENSG00000183605
0,76	0,00	0,00	ENSG00000165672
1,64	0,00	0,00	ENSG00000198873
0,73	0,00	0,00	ENSG00000148908
0,92	0,31	0,42	ENSG00000151923
1,21	0,02	0,05	ENSG00000151929
0,75	0,00	0,00	ENSG00000198825
0,83	0,02	0,05	ENSG00000197771
1,44	0,00	0,00	ENSG00000107651
0,39	0,00	0,00	ENSG00000203805
0,80	0,01	0,02	ENSG00000120008
12,14	0,00	0,00	ENSG00000066468
0,58	0,00	0,00	ENSG00000107669
0,61	0,00	0,00	ENSG00000107672
0,52	0,00	0,00	ENSG00000138162
1,14	0,97	0,98	ENSG00000138152
0,78	0,01	0,01	ENSG00000107679

0,51	0,42	0,54	ENSG00000254636
2,44	0,00	0,00	ENSG00000166033
0,53	0,00	0,00	ENSG00000187908
#N/D	#N/D	#N/D	ENSG00000286135
#N/D	#N/D	#N/D	ENSG00000183559
1,45	0,56	0,67	ENSG00000138161
0,59	0,07	0,13	ENSG00000286088
1,18	0,18	0,27	ENSG00000213185
1,14	0,97	#N/D	ENSG00000203795
1,33	0,01	0,02	ENSG00000119965
0,77	0,15	0,23	ENSG00000179988
1,33	0,00	0,01	ENSG00000095574
0,66	0,00	0,00	ENSG00000196177
0,69	0,00	0,00	ENSG00000188620
3,56	0,23	0,34	ENSG00000188816
0,91	0,25	0,36	ENSG00000154473
0,01	0,07	0,12	ENSG00000154478
0,22	0,00	0,00	ENSG00000121898
0,65	0,00	0,00	ENSG00000182022
1,08	0,37	0,48	ENSG00000065154
0,70	0,39	0,51	ENSG00000229544
1,34	0,00	0,01	ENSG00000107902
0,61	0,00	0,01	ENSG00000258539
0,81	0,01	0,02	ENSG00000189319
0,97	0,75	0,83	ENSG00000203791
0,88	0,18	0,27	ENSG00000165660
1,21	0,02	0,04	ENSG00000019995
1,14	0,11	0,18	ENSG00000175029
0,02	0,11	0,18	ENSG00000175018
0,91	0,27	0,37	ENSG00000107938
0,35	0,03	0,05	ENSG00000154485
0,74	0,00	0,00	ENSG00000188690
0,84	0,04	0,08	ENSG00000107949
0,74	0,00	0,00	ENSG00000089876
1,34	0,32	0,44	ENSG00000203780
0,59	0,00	0,00	ENSG00000148848
0,95	0,77	0,85	ENSG00000154493
0,82	0,01	0,03	ENSG00000150760
1,04	0,86	0,92	ENSG00000188916
#N/D	#N/D	#N/D	ENSG00000214285
#N/D	#N/D	#N/D	ENSG00000279725
1,14	0,97	#N/D	ENSG00000186766
1,14	0,97	#N/D	ENSG00000180745
0,17	0,00	0,00	ENSG00000132334
1,01	0,88	0,94	ENSG00000148773
1,72	0,00	0,00	ENSG00000170430
0,53	0,00	0,00	ENSG00000108001
1,27	0,41	0,53	ENSG00000237489

0,83	0,03	0,05	ENSG00000108010
#N/D	#N/D	#N/D	ENSG00000176769
#N/D	#N/D	#N/D	ENSG00000280446
1,11	0,23	0,33	ENSG00000175470
2,40	0,00	0,00	ENSG00000176171
2,88	0,03	0,05	ENSG00000279982
0,89	0,49	0,59	ENSG00000188385
1,26	0,03	0,06	ENSG00000151640
2,93	0,00	0,00	ENSG00000165752
1,30	0,02	0,05	ENSG00000148814
1,10	0,31	0,43	ENSG00000171813
1,43	0,00	0,00	ENSG00000068383
2,39	0,14	0,22	ENSG00000148826
59,15	0,14	0,22	ENSG00000171811
1,14	0,97	#N/D	ENSG00000197177
1,95	0,00	0,00	ENSG00000171798
#N/D	#N/D	#N/D	ENSG00000171794
22,89	0,26	0,37	ENSG00000151650
1,25	0,05	0,10	ENSG00000151651
1,42	0,00	0,00	ENSG00000130640
0,85	0,09	0,15	ENSG00000198546
#N/D	#N/D	#N/D	ENSG00000283496
6,90	0,01	0,02	ENSG00000130643
1,64	0,06	0,10	ENSG00000165828
1,12	0,27	0,37	ENSG00000148803
1,18	0,04	0,08	ENSG00000127884
0,07	0,31	0,43	ENSG00000148832
124,41	0,08	0,14	ENSG00000254536
0,69	0,00	0,00	ENSG00000148824
0,76	0,09	0,15	ENSG00000203772
1,94	0,00	0,00	ENSG00000214279
2,65	0,00	0,00	ENSG00000130649
0,57	0,36	0,47	ENSG00000171772
8,39	0,46	0,56	ENSG00000225899
2,63	0,00	0,00	ENSG00000177951
1,14	0,97	#N/D	ENSG00000188076
4,66	0,00	0,00	ENSG00000177947
1,64	0,00	0,00	ENSG00000177963
1,20	0,05	0,09	ENSG00000142082
1,43	0,00	0,00	ENSG00000185627
1,29	0,74	0,82	ENSG00000174885
1,32	0,00	0,00	ENSG00000142102
1,11	0,84	0,91	ENSG00000206013
2,05	0,00	0,00	ENSG00000185201
#N/D	#N/D	#N/D	ENSG00000288681
3,88	0,00	0,00	ENSG00000185885
2,04	0,00	0,00	ENSG00000142089
2,80	0,00	0,00	ENSG00000182272

0,91	0,30	0,41	ENSG00000184363
1,72	0,00	0,00	ENSG00000185187
0,59	0,21	0,32	ENSG00000185101
1,52	0,00	0,00	ENSG00000174915
1,65	0,00	0,00	ENSG00000023191
1,70	0,00	0,00	ENSG00000174775
1,05	0,78	0,86	ENSG00000161328
1,17	0,20	0,30	ENSG00000185522
2,62	0,00	0,00	ENSG00000099849
1,72	0,00	0,00	ENSG00000070047
4,13	0,00	0,00	ENSG00000185507
2,43	0,00	0,00	ENSG00000099834
3,95	0,00	0,00	ENSG00000070031
2,00	0,00	0,00	ENSG00000069696
1,37	0,00	0,00	ENSG00000177030
1,33	0,00	0,01	ENSG00000177106
2,06	0,00	0,00	ENSG00000177042
1,49	0,00	0,00	ENSG00000177156
1,89	0,00	0,00	ENSG00000177225
0,94	0,58	0,68	ENSG00000279672
0,74	0,08	0,14	ENSG00000184524
1,84	0,00	0,00	ENSG00000177542
2,04	0,00	0,00	ENSG00000288675
1,76	0,00	0,00	ENSG00000177595
1,49	0,00	0,00	ENSG00000177600
1,70	0,00	0,00	ENSG00000177666
0,89	0,35	0,47	ENSG00000177685
1,84	0,00	0,00	ENSG00000177697
1,84	0,00	0,00	ENSG00000177700
2,18	0,00	0,00	ENSG00000214063
1,86	0,00	0,00	ENSG00000177830
1,32	0,00	0,00	ENSG00000183020
0,79	0,20	0,30	ENSG00000184956
0,34	0,10	0,16	ENSG00000198788
0,05	0,00	0,00	ENSG00000215182
2,25	0,00	0,00	ENSG00000117983
1,44	0,00	0,00	ENSG00000078902
0,73	0,00	0,00	ENSG00000174672
1,32	0,00	0,01	ENSG00000182208
2,42	0,00	0,00	ENSG00000184545
1,61	0,26	0,37	ENSG00000205869
1,14	0,97	#N/D	ENSG00000205867
#N/D	#N/D	#N/D	ENSG00000196224
1,14	0,97	#N/D	ENSG00000241598
#N/D	#N/D	#N/D	ENSG00000185940
#N/D	#N/D	#N/D	ENSG00000205864
11,80	0,00	0,00	ENSG00000244242
5,97	0,00	0,00	ENSG00000250644

1,51	0,00	0,00	ENSG00000117984
1,14	0,97	#N/D	ENSG00000149043
#N/D	#N/D	#N/D	ENSG00000130598
0,99	0,91	0,97	ENSG00000130592
0,41	0,01	0,02	ENSG00000283787
9,56	0,02	0,05	ENSG00000130595
1,49	0,00	0,00	ENSG00000214026
1,05	0,50	0,61	ENSG00000167244
1,14	0,97	#N/D	ENSG00000284779
#N/D	#N/D	#N/D	ENSG00000129965
#N/D	#N/D	#N/D	ENSG00000254647
1,14	0,97	0,98	ENSG00000180176
1,14	0,97	0,98	ENSG00000183734
0,91	0,58	0,68	ENSG00000110665
1,36	0,04	0,08	ENSG00000064201
1,94	0,00	0,00	ENSG00000110651
2,07	0,00	0,00	ENSG00000184281
2,49	0,00	0,01	ENSG00000070985
18,99	0,00	0,01	ENSG00000053918
1,19	0,24	0,34	ENSG00000129757
0,70	0,00	0,00	ENSG00000110628
1,42	0,00	0,00	ENSG00000181649
1,17	0,06	0,10	ENSG00000205531
1,01	0,95	0,98	ENSG00000110619
1,48	0,00	0,00	ENSG00000021762
#N/D	#N/D	#N/D	ENSG00000182170
#N/D	#N/D	#N/D	ENSG00000184350
0,64	0,00	0,00	ENSG00000005801
0,22	0,14	0,22	ENSG00000167311
1,14	0,97	0,98	ENSG00000129744
0,33	0,06	0,10	ENSG00000129749
1,06	0,44	0,55	ENSG00000110713
1,44	0,00	0,00	ENSG00000148985
1,28	0,00	0,01	ENSG00000177105
0,93	0,39	0,51	ENSG00000167323
1,08	0,36	0,47	ENSG00000167325
#N/D	#N/D	#N/D	ENSG00000284018
#N/D	#N/D	#N/D	ENSG00000284306
#N/D	#N/D	#N/D	ENSG00000283873
#N/D	#N/D	#N/D	ENSG00000284546
#N/D	#N/D	#N/D	ENSG00000284438
1,14	0,97	#N/D	ENSG00000221996
0,96	0,70	0,78	ENSG00000132109
1,14	0,97	#N/D	ENSG00000181963
1,14	0,97	#N/D	ENSG00000196778
#N/D	#N/D	#N/D	ENSG00000197790
0,74	0,69	0,78	ENSG00000226288
0,66	0,51	0,62	ENSG00000232268

0,92	0,39	0,50	ENSG00000167333
#N/D	#N/D	#N/D	ENSG00000197428
1,14	0,97	#N/D	ENSG00000180785
30,14	0,22	0,32	ENSG00000167332
1,14	0,97	#N/D	ENSG00000197674
0,56	0,20	0,30	ENSG00000167346
#N/D	#N/D	#N/D	ENSG00000280021
#N/D	#N/D	#N/D	ENSG00000279270
#N/D	#N/D	#N/D	ENSG00000176925
#N/D	#N/D	#N/D	ENSG00000176922
#N/D	#N/D	#N/D	ENSG00000176900
0,43	0,22	0,32	ENSG00000176895
1,11	0,84	0,91	ENSG00000176893
0,67	0,65	0,74	ENSG00000278870
0,51	0,42	0,54	ENSG00000205497
0,98	0,99	0,99	ENSG00000205496
0,59	0,21	0,32	ENSG00000176798
0,13	0,46	0,56	ENSG00000205495
#N/D	#N/D	#N/D	ENSG00000176787
0,07	0,31	#N/D	ENSG00000273085
1,14	0,97	#N/D	ENSG00000171944
1,14	0,97	#N/D	ENSG00000182070
1,14	0,97	#N/D	ENSG00000176742
#N/D	#N/D	#N/D	ENSG00000285498
8,39	0,46	0,56	ENSG00000244734
#N/D	#N/D	#N/D	ENSG00000223609
22,89	0,26	0,37	ENSG00000213934
1,14	0,97	0,98	ENSG00000284931
3,56	0,23	0,34	ENSG00000196565
1,45	0,56	0,67	ENSG00000239920
0,07	0,31	0,43	ENSG00000213931
0,07	0,31	0,43	ENSG00000183251
0,05	0,24	0,35	ENSG00000167355
#N/D	#N/D	#N/D	ENSG00000279012
#N/D	#N/D	#N/D	ENSG00000176239
0,60	0,47	0,58	ENSG00000184698
0,05	0,24	0,35	ENSG00000167360
2,71	0,36	0,48	ENSG00000167359
2,71	0,36	0,48	ENSG00000187918
0,98	0,99	0,99	ENSG00000181609
0,98	0,98	0,99	ENSG00000175520
0,80	0,55	0,65	ENSG00000175518
0,99	1,00	#N/D	ENSG00000181616
4,42	0,15	0,24	ENSG00000187747
0,61	0,00	0,00	ENSG00000121236
0,75	0,17	0,26	ENSG00000258588
0,86	0,18	0,27	ENSG00000258659
1,90	0,00	0,00	ENSG00000132256

1,29	0,51	0,61	ENSG00000132274
#N/D	#N/D	#N/D	ENSG00000181109
#N/D	#N/D	#N/D	ENSG00000181023
8,39	0,46	#N/D	ENSG00000181074
#N/D	#N/D	#N/D	ENSG00000181017
#N/D	#N/D	#N/D	ENSG00000181009
1,14	0,97	0,98	ENSG00000181001
#N/D	#N/D	#N/D	ENSG00000180988
#N/D	#N/D	#N/D	ENSG00000205409
#N/D	#N/D	#N/D	ENSG00000183269
#N/D	#N/D	#N/D	ENSG00000180974
#N/D	#N/D	#N/D	ENSG00000277932
#N/D	#N/D	#N/D	ENSG00000184478
#N/D	#N/D	#N/D	ENSG00000188691
8,39	0,46	#N/D	ENSG00000183313
#N/D	#N/D	#N/D	ENSG00000183389
1,14	0,97	#N/D	ENSG00000180934
1,14	0,97	#N/D	ENSG00000180919
1,14	0,97	#N/D	ENSG00000255307
1,91	0,35	0,47	ENSG00000175485
2,88	0,00	0,00	ENSG00000180878
2,33	0,00	0,00	ENSG00000051009
2,71	0,36	0,48	ENSG00000132259
8,39	0,46	#N/D	ENSG00000110148
0,94	0,50	0,60	ENSG00000170955
1,26	0,00	0,01	ENSG00000166311
1,44	0,00	0,00	ENSG00000166313
1,61	0,36	0,47	ENSG00000110169
1,24	0,02	0,04	ENSG00000110171
2,14	0,00	0,00	ENSG00000132254
1,48	0,22	0,32	ENSG00000283977
2,13	0,00	0,00	ENSG00000132286
2,10	0,00	0,00	ENSG00000179532
1,24	0,01	0,02	ENSG00000132275
1,34	0,00	0,00	ENSG00000166333
1,10	0,25	0,35	ENSG00000166337
1,74	0,00	0,00	ENSG00000166340
2,76	0,00	0,00	ENSG00000166341
1,46	0,00	0,00	ENSG00000158042
0,13	0,46	0,56	ENSG00000188124
0,79	0,72	0,80	ENSG00000279486
3,56	0,23	0,34	ENSG00000184933
8,39	0,46	0,56	ENSG00000166363
2,24	0,10	0,17	ENSG00000170790
0,36	0,35	0,46	ENSG00000170782
0,79	0,72	0,80	ENSG00000166368
1,14	0,97	0,98	ENSG00000178358
1,43	0,00	0,00	ENSG00000149054

1,68	0,08	0,14	ENSG00000149050
1,37	0,18	0,28	ENSG00000158077
0,98	0,99	0,99	ENSG00000170748
6,99	0,05	0,10	ENSG00000170743
1,29	0,56	0,67	ENSG00000183801
0,83	0,12	0,20	ENSG00000166387
0,37	0,00	0,00	ENSG00000166394
0,13	0,46	0,56	ENSG00000183378
1,14	0,97	0,98	ENSG00000183303
1,14	0,97	0,98	ENSG00000182334
1,14	0,97	0,98	ENSG00000279000
0,07	0,31	0,43	ENSG00000170683
0,02	0,12	0,19	ENSG00000182261
1,14	0,10	0,17	ENSG00000175390
1,99	0,00	0,00	ENSG00000166402
8,39	0,46	#N/D	ENSG00000279391
1,00	0,99	0,99	ENSG00000166405
0,79	0,48	0,58	ENSG00000166407
0,13	0,46	0,56	ENSG00000130413
1,20	0,04	0,07	ENSG00000166436
1,02	0,85	0,91	ENSG00000166441
0,58	0,00	0,00	ENSG00000166444
0,91	0,26	0,37	ENSG00000166452
0,61	0,16	0,25	ENSG00000176029
8,39	0,46	0,56	ENSG00000176009
1,01	0,93	0,98	ENSG00000175348
0,95	0,52	0,62	ENSG00000175352
0,55	0,09	0,16	ENSG00000175356
0,94	0,48	0,58	ENSG00000184014
1,98	0,00	0,00	ENSG00000166471
0,76	0,00	0,00	ENSG00000205339
1,53	0,00	0,00	ENSG00000166478
1,10	0,27	0,37	ENSG00000166483
1,09	0,29	0,40	ENSG00000133789
1,06	0,47	0,57	ENSG00000133812
3,29	0,00	0,00	ENSG00000148926
0,80	0,01	0,02	ENSG00000133805
1,10	0,26	0,37	ENSG00000110315
2,24	0,10	0,17	ENSG00000133800
2,77	0,00	0,00	ENSG00000072952
1,00	0,96	0,98	ENSG00000198730
0,76	0,00	0,00	ENSG00000110321
0,81	0,02	0,04	ENSG00000236287
1,11	0,22	0,32	ENSG00000110328
0,83	0,58	0,68	ENSG00000254598
1,14	0,97	#N/D	ENSG00000280288
0,70	0,00	0,00	ENSG00000170242
1,10	0,24	0,35	ENSG00000050165

0,98	0,77	0,85	ENSG00000133816
0,72	0,00	0,00	ENSG00000197702
0,92	0,30	0,41	ENSG00000187079
0,24	0,00	0,00	ENSG00000189431
1,07	0,45	0,56	ENSG00000133794
0,62	0,00	0,00	ENSG00000148925
#N/D	#N/D	#N/D	ENSG00000152266
0,67	0,00	0,00	ENSG00000197601
0,53	0,01	0,01	ENSG00000262655
1,23	0,02	0,04	ENSG00000133818
1,27	0,00	0,01	ENSG00000129083
0,57	0,24	0,35	ENSG00000256206
0,76	0,00	0,00	ENSG00000129084
0,98	0,98	0,99	ENSG00000152270
0,51	0,00	0,00	ENSG00000186104
0,02	0,15	0,23	ENSG00000175868
1,14	0,97	#N/D	ENSG00000110680
1,33	0,04	0,07	ENSG00000188487
11,28	0,01	0,03	ENSG00000110693
0,67	0,00	0,00	ENSG00000110696
0,04	0,00	0,00	ENSG00000166689
0,93	0,35	0,46	ENSG00000110700
0,85	0,05	0,09	ENSG00000011405
0,92	0,38	0,49	ENSG00000070081
0,11	0,00	0,00	ENSG00000188211
0,43	0,22	0,32	ENSG00000187486
22,89	0,26	0,37	ENSG00000006071
1,14	0,97	#N/D	ENSG00000006611
22,89	0,26	#N/D	ENSG00000188162
#N/D	#N/D	#N/D	ENSG00000129152
0,31	0,01	0,02	ENSG00000129159
1,20	0,08	0,13	ENSG00000129158
0,18	0,10	0,16	ENSG00000129167
0,96	0,63	0,73	ENSG00000166788
0,13	0,46	0,56	ENSG00000179826
1,14	0,97	0,98	ENSG00000179817
0,02	0,15	0,23	ENSG00000148965
1,14	0,97	0,98	ENSG00000255071
0,06	0,00	0,00	ENSG00000134339
0,01	0,00	0,00	ENSG00000173432
1,00	0,96	0,98	ENSG00000110756
0,75	0,00	0,00	ENSG00000110768
2,65	0,00	0,00	ENSG00000134333
1,14	0,97	0,98	ENSG00000166796
1,45	0,48	0,58	ENSG00000166800
0,95	0,57	0,68	ENSG00000074319
0,85	0,09	0,15	ENSG00000151116
0,56	0,07	0,12	ENSG00000247595

0,28	0,04	0,08	ENSG00000279837
3,15	0,00	0,00	ENSG00000179119
0,81	0,25	0,36	ENSG00000151117
8,39	0,46	0,56	ENSG00000179057
1,14	0,97	#N/D	ENSG00000110786
#N/D	#N/D	#N/D	ENSG00000170255
#N/D	#N/D	#N/D	ENSG00000183695
0,66	0,00	0,00	ENSG00000177054
1,14	0,97	#N/D	ENSG00000129170
0,47	0,00	0,00	ENSG00000129173
0,93	0,36	0,47	ENSG00000166833
0,41	0,27	0,38	ENSG00000279266
#N/D	#N/D	#N/D	ENSG00000109851
37,39	0,19	0,29	ENSG00000109854
0,44	0,00	0,00	ENSG00000185238
#N/D	#N/D	#N/D	ENSG00000165970
#N/D	#N/D	#N/D	ENSG00000165973
1,14	0,97	#N/D	ENSG00000171714
#N/D	#N/D	#N/D	ENSG00000091664
0,87	0,18	0,28	ENSG00000183161
1,89	0,45	0,56	ENSG00000148935
0,92	0,52	0,62	ENSG00000198168
1,14	0,97	#N/D	ENSG00000255359
0,05	0,24	0,35	ENSG00000187398
1,14	0,97	#N/D	ENSG00000134343
1,14	0,97	0,98	ENSG00000169550
1,14	0,97	0,98	ENSG00000148942
1,55	0,00	0,01	ENSG00000176971
0,99	1,00	1,00	ENSG00000129151
0,79	0,02	0,04	ENSG00000109881
0,86	0,09	0,15	ENSG00000205213
1,14	0,13	0,21	ENSG00000148943
0,96	0,63	0,72	ENSG00000176697
0,72	0,00	0,00	ENSG00000121621
0,75	0,00	0,00	ENSG00000169519
#N/D	#N/D	#N/D	ENSG00000182255
1,14	0,97	#N/D	ENSG00000131808
0,92	0,43	0,55	ENSG00000152219
8,39	0,46	0,56	ENSG00000066382
51,90	0,15	0,24	ENSG00000170959
1,47	0,00	0,00	ENSG00000170946
0,85	0,25	0,36	ENSG00000148950
0,96	0,70	0,79	ENSG00000109911
1,34	0,00	0,00	ENSG00000007372
0,57	0,00	0,00	ENSG00000285283
0,93	0,35	0,46	ENSG00000049449
1,14	0,97	0,98	ENSG00000184937
1,04	0,61	0,71	ENSG00000149100

0,18	0,10	0,16	ENSG00000186714
1,30	0,21	0,31	ENSG00000135378
0,80	0,01	0,01	ENSG00000060749
0,79	0,13	0,21	ENSG00000121690
0,58	0,00	0,00	ENSG00000176148
0,65	0,00	0,00	ENSG00000176102
1,88	0,00	0,00	ENSG00000110422
0,82	0,02	0,05	ENSG00000110427
0,73	0,33	0,44	ENSG00000205177
3,74	0,00	0,00	ENSG00000284969
3,16	0,00	0,00	ENSG00000085063
1,58	0,00	0,00	ENSG00000110429
2,66	0,00	0,01	ENSG00000135363
1,46	0,00	0,00	ENSG00000135387
1,16	0,07	0,12	ENSG00000135372
0,52	0,00	0,00	ENSG00000166016
1,55	0,00	0,00	ENSG00000121691
0,13	0,46	#N/D	ENSG00000135374
0,01	0,00	0,00	ENSG00000135373
1,22	0,03	0,05	ENSG00000149089
1,55	0,00	0,00	ENSG00000110435
2,11	0,00	0,00	ENSG00000026508
0,55	0,08	0,14	ENSG00000110436
0,59	0,11	0,18	ENSG00000149090
0,40	0,00	0,00	ENSG00000179431
1,32	0,00	0,00	ENSG00000166326
0,58	0,00	0,00	ENSG00000179241
0,30	0,00	0,01	ENSG00000280321
2,03	0,00	0,00	ENSG00000110442
0,43	0,00	0,00	ENSG00000135362
2,10	0,00	0,00	ENSG00000175104
15,64	0,33	#N/D	ENSG00000280331
4,38	0,00	0,00	ENSG00000166349
22,89	0,26	0,37	ENSG00000175097
3,41	0,00	0,00	ENSG00000166352
#N/D	#N/D	#N/D	ENSG00000279004
8,39	0,46	#N/D	ENSG00000279675
1,14	0,97	0,98	ENSG00000148948
1,16	0,06	0,11	ENSG00000166181
0,96	0,61	0,70	ENSG00000052841
1,15	0,10	0,17	ENSG00000149084
1,18	0,08	0,14	ENSG00000166199
22,89	0,26	0,37	ENSG00000187479
1,14	0,97	#N/D	ENSG00000205126
0,75	0,03	0,06	ENSG00000110455
1,49	0,00	0,00	ENSG00000151348
1,44	0,00	0,00	ENSG00000052850
2,62	0,00	0,00	ENSG00000085117

1,14	0,22	0,32	ENSG00000157570
2,54	0,00	0,00	ENSG00000175274
1,22	0,06	0,10	ENSG00000019485
20,71	0,00	0,00	ENSG00000019505
74,81	0,00	0,00	ENSG00000175264
1,35	0,00	0,00	ENSG00000181830
3,05	0,00	0,00	ENSG00000121671
0,88	0,18	0,28	ENSG00000121653
0,99	1,00	1,00	ENSG00000234776
1,67	0,00	0,00	ENSG00000121680
1,89	0,45	0,56	ENSG00000165905
1,62	0,00	0,00	ENSG00000135365
1,18	0,04	0,07	ENSG00000157613
1,39	0,00	0,00	ENSG00000149091
0,99	0,91	0,97	ENSG00000110492
0,52	0,01	0,02	ENSG00000180720
1,51	0,00	0,00	ENSG00000110497
1,35	0,02	0,04	ENSG00000180423
1,94	0,00	0,00	ENSG00000175224
2,76	0,00	0,00	ENSG00000175220
2,20	0,00	0,00	ENSG00000175213
0,74	0,69	0,78	ENSG00000180210
1,25	0,01	0,01	ENSG00000175216
1,77	0,00	0,00	ENSG00000134569
1,80	0,00	0,00	ENSG00000149179
1,40	0,00	0,00	ENSG00000149182
1,16	0,08	0,14	ENSG00000165912
1,69	0,00	0,00	ENSG00000134574
0,98	0,83	0,90	ENSG00000134575
1,57	0,00	0,00	ENSG00000025434
1,64	0,00	0,00	ENSG00000110514
0,13	0,46	0,56	ENSG00000134571
1,49	0,11	0,18	ENSG00000066336
2,31	0,00	0,00	ENSG00000165915
1,53	0,00	0,00	ENSG00000165916
0,99	1,00	1,00	ENSG00000165917
1,29	0,00	0,00	ENSG00000149187
1,64	0,00	0,00	ENSG00000213619
1,13	0,17	0,26	ENSG00000110536
2,18	0,00	0,00	ENSG00000123444
1,85	0,60	0,69	ENSG00000196666
37,39	0,19	0,29	ENSG00000172247
1,34	0,00	0,00	ENSG00000109919
1,02	0,95	0,98	ENSG00000165923
1,51	0,00	0,00	ENSG00000109920
1,34	0,00	0,00	ENSG00000030066
0,94	0,44	0,56	ENSG00000149177
1,14	0,97	#N/D	ENSG00000175619

#N/D	#N/D	#N/D	ENSG00000172208
#N/D	#N/D	#N/D	ENSG00000176567
#N/D	#N/D	#N/D	ENSG00000176555
1,14	0,97	#N/D	ENSG00000176547
#N/D	#N/D	#N/D	ENSG00000176540
#N/D	#N/D	#N/D	ENSG00000237388
#N/D	#N/D	#N/D	ENSG00000220948
#N/D	#N/D	#N/D	ENSG00000182053
1,14	0,97	#N/D	ENSG00000214891
#N/D	#N/D	#N/D	ENSG00000086205
#N/D	#N/D	#N/D	ENSG00000258817
#N/D	#N/D	#N/D	ENSG00000221954
#N/D	#N/D	#N/D	ENSG00000260811
#N/D	#N/D	#N/D	ENSG00000185926
#N/D	#N/D	#N/D	ENSG00000225997
#N/D	#N/D	#N/D	ENSG00000221840
8,39	0,46	0,56	ENSG00000150244
#N/D	#N/D	#N/D	ENSG00000181961
#N/D	#N/D	#N/D	ENSG00000181958
#N/D	#N/D	#N/D	ENSG00000181939
#N/D	#N/D	#N/D	ENSG00000279514
#N/D	#N/D	#N/D	ENSG00000172188
#N/D	#N/D	#N/D	ENSG00000181927
#N/D	#N/D	#N/D	ENSG00000174982
#N/D	#N/D	#N/D	ENSG00000181903
#N/D	#N/D	#N/D	ENSG00000186886
#N/D	#N/D	#N/D	ENSG00000279761
#N/D	#N/D	#N/D	ENSG00000186113
#N/D	#N/D	#N/D	ENSG00000279395
#N/D	#N/D	#N/D	ENSG00000186119
#N/D	#N/D	#N/D	ENSG00000205030
#N/D	#N/D	#N/D	ENSG00000205029
1,14	0,97	#N/D	ENSG00000124900
#N/D	#N/D	#N/D	ENSG00000187612
#N/D	#N/D	#N/D	ENSG00000167825
#N/D	#N/D	#N/D	ENSG00000174970
#N/D	#N/D	#N/D	ENSG00000149133
1,14	0,97	#N/D	ENSG00000181785
#N/D	#N/D	#N/D	ENSG00000172154
#N/D	#N/D	#N/D	ENSG00000181767
#N/D	#N/D	#N/D	ENSG00000181761
8,39	0,46	#N/D	ENSG00000167822
#N/D	#N/D	#N/D	ENSG00000181752
#N/D	#N/D	#N/D	ENSG00000174957
#N/D	#N/D	#N/D	ENSG00000254658
#N/D	#N/D	#N/D	ENSG00000181718
#N/D	#N/D	#N/D	ENSG00000172489
#N/D	#N/D	#N/D	ENSG00000181698

8,39	0,46	#N/D	ENSG00000181693
#N/D	#N/D	#N/D	ENSG00000280314
#N/D	#N/D	#N/D	ENSG00000150261
#N/D	#N/D	#N/D	ENSG00000172487
#N/D	#N/D	#N/D	ENSG00000172199
#N/D	#N/D	#N/D	ENSG00000272987
#N/D	#N/D	#N/D	ENSG00000279961
1,14	0,97	#N/D	ENSG00000284732
#N/D	#N/D	#N/D	ENSG00000150269
#N/D	#N/D	#N/D	ENSG00000174937
#N/D	#N/D	#N/D	ENSG00000181371
#N/D	#N/D	#N/D	ENSG00000255223
#N/D	#N/D	#N/D	ENSG00000254834
#N/D	#N/D	#N/D	ENSG00000255012
#N/D	#N/D	#N/D	ENSG00000172464
#N/D	#N/D	#N/D	ENSG00000172459
1,14	0,97	#N/D	ENSG00000174914
#N/D	#N/D	#N/D	ENSG00000172457
#N/D	#N/D	#N/D	ENSG00000241356
#N/D	#N/D	#N/D	ENSG00000181282
#N/D	#N/D	#N/D	ENSG00000181273
1,14	0,97	0,98	ENSG00000183908
22,89	0,26	0,37	ENSG00000134817
1,61	0,00	0,00	ENSG00000149115
1,31	0,00	0,00	ENSG00000149136
15,64	0,33	0,44	ENSG00000109991
1,14	0,97	0,98	ENSG00000156575
0,60	0,47	0,58	ENSG00000186652
15,64	0,33	0,44	ENSG00000254979
0,59	0,00	0,00	ENSG00000134802
1,10	0,33	0,44	ENSG00000186907
1,61	0,00	0,00	ENSG00000149150
1,46	0,00	0,00	ENSG00000134809
8,27	0,00	0,01	ENSG00000214872
1,28	0,00	0,01	ENSG00000156587
269,42	0,04	0,08	ENSG00000149131
1,19	0,13	0,21	ENSG00000166793
1,74	0,00	0,00	ENSG00000172409
1,48	0,00	0,00	ENSG00000156599
1,81	0,00	0,00	ENSG00000156603
1,65	0,00	0,00	ENSG00000213593
1,93	0,00	0,00	ENSG00000288534
1,10	0,26	0,36	ENSG00000211450
5,54	0,02	0,04	ENSG00000254732
2,94	0,00	0,00	ENSG00000233436
2,02	0,00	0,00	ENSG00000198561
#N/D	#N/D	#N/D	ENSG00000186509
#N/D	#N/D	#N/D	ENSG00000279051

1,14	0,97	#N/D	ENSG00000172377
8,39	0,46	#N/D	ENSG00000186513
#N/D	#N/D	#N/D	ENSG00000197887
1,14	0,97	#N/D	ENSG00000280204
#N/D	#N/D	#N/D	ENSG00000180475
#N/D	#N/D	#N/D	ENSG00000172772
1,14	0,97	#N/D	ENSG00000197786
#N/D	#N/D	#N/D	ENSG00000172769
1,14	0,97	#N/D	ENSG00000172365
0,99	1,00	1,00	ENSG00000172362
1,44	0,68	0,77	ENSG00000198283
1,52	0,00	0,00	ENSG00000110031
1,58	0,00	0,00	ENSG00000186660
0,90	0,59	0,69	ENSG00000255073
1,03	0,86	0,93	ENSG00000242689
1,38	0,30	0,41	ENSG00000280010
0,07	0,31	0,43	ENSG00000149124
0,44	0,05	0,10	ENSG00000156689
0,46	0,00	0,01	ENSG00000166840
0,13	0,46	0,56	ENSG00000255151
0,88	0,35	0,46	ENSG00000189057
2,75	0,00	0,00	ENSG00000166801
8,09	0,00	0,00	ENSG00000110042
5,54	0,00	0,00	ENSG00000197629
1,13	0,82	0,89	ENSG00000176495
0,13	0,46	#N/D	ENSG00000172324
8,39	0,46	0,56	ENSG00000172320
#N/D	#N/D	#N/D	ENSG00000166884
15,64	0,33	0,44	ENSG00000254466
#N/D	#N/D	#N/D	ENSG00000176200
15,64	0,33	0,44	ENSG00000172742
2,76	0,00	0,00	ENSG00000110048
1,34	0,00	0,00	ENSG00000166889
1,14	0,97	#N/D	ENSG00000172289
0,85	0,08	0,13	ENSG00000166900
1,24	0,02	0,04	ENSG00000166902
1,14	0,97	0,98	ENSG00000134812
1,14	0,97	0,98	ENSG00000134827
1,14	0,97	0,98	ENSG00000285231
#N/D	#N/D	#N/D	ENSG00000284873
#N/D	#N/D	#N/D	ENSG00000285010
#N/D	#N/D	#N/D	ENSG00000255393
1,14	0,97	#N/D	ENSG00000149507
#N/D	#N/D	#N/D	ENSG00000149516
0,13	0,46	#N/D	ENSG00000149534
#N/D	#N/D	#N/D	ENSG00000110077
1,14	0,97	0,98	ENSG00000110079
0,28	0,04	0,08	ENSG00000214787

1,14	0,97	#N/D	ENSG00000166926
1,14	0,97	#N/D	ENSG00000166927
#N/D	#N/D	#N/D	ENSG00000166928
#N/D	#N/D	#N/D	ENSG00000166930
#N/D	#N/D	#N/D	ENSG00000156738
#N/D	#N/D	#N/D	ENSG00000071203
#N/D	#N/D	#N/D	ENSG00000204979
#N/D	#N/D	#N/D	ENSG00000166959
#N/D	#N/D	#N/D	ENSG00000214782
0,55	0,28	0,39	ENSG00000166961
0,13	0,46	#N/D	ENSG00000172689
1,09	0,32	0,43	ENSG00000110104
2,15	0,00	0,00	ENSG00000183134
1,11	0,61	0,71	ENSG00000149506
0,99	0,87	0,94	ENSG00000110107
0,90	0,21	0,31	ENSG00000110108
0,62	0,00	0,00	ENSG00000006118
0,60	0,26	0,37	ENSG00000110446
8,39	0,46	#N/D	ENSG00000013725
3,16	0,00	0,00	ENSG00000110448
1,29	0,00	0,01	ENSG00000167987
1,94	0,01	0,01	ENSG00000279549
0,13	0,46	0,56	ENSG00000229859
1,14	0,97	#N/D	ENSG00000229183
1,29	0,74	0,82	ENSG00000256713
1,58	0,00	0,01	ENSG00000167992
1,99	0,00	0,00	ENSG00000167986
1,08	0,39	0,51	ENSG00000149476
1,59	0,00	0,00	ENSG00000162144
0,41	0,00	0,00	ENSG00000279246
1,29	0,00	0,01	ENSG00000149483
1,05	0,67	0,76	ENSG00000187049
1,58	0,00	0,00	ENSG00000149532
1,74	0,06	0,11	ENSG00000279632
4,65	0,00	0,00	ENSG00000256591
2,01	0,00	0,00	ENSG00000167985
6,17	0,00	0,00	ENSG00000162148
1,24	0,50	0,61	ENSG00000204950
1,60	0,00	0,00	ENSG00000011347
2,42	0,00	0,00	ENSG00000279878
0,69	0,00	0,00	ENSG00000134780
2,38	0,00	0,00	ENSG00000124920
2,08	0,00	0,00	ENSG00000134825
2,27	0,00	0,00	ENSG00000168496
3,98	0,00	0,00	ENSG00000134824
1,42	0,00	0,00	ENSG00000149485
0,62	0,00	0,00	ENSG00000221968
0,56	0,00	0,00	ENSG00000167994

0,27	0,00	0,00	ENSG00000167995
0,28	0,00	0,00	ENSG00000167996
1,06	0,82	0,89	ENSG00000279491
1,57	0,00	0,00	ENSG00000149503
#N/D	#N/D	#N/D	ENSG00000168515
#N/D	#N/D	#N/D	ENSG00000124939
0,13	0,46	#N/D	ENSG00000124935
8,39	0,46	#N/D	ENSG00000110484
#N/D	#N/D	#N/D	ENSG00000197745
1,13	0,58	0,68	ENSG00000162174
15,64	0,33	0,44	ENSG00000149021
2,54	0,00	0,00	ENSG00000124942
1,57	0,00	0,00	ENSG00000254772
2,36	0,00	0,00	ENSG00000255508
2,08	0,00	0,00	ENSG00000149016
1,31	0,00	0,00	ENSG00000149480
1,34	0,00	0,00	ENSG00000149499
1,90	0,00	0,00	ENSG00000149489
2,47	0,00	0,00	ENSG00000149541
1,64	0,00	0,00	ENSG00000089597
1,36	0,00	0,00	ENSG00000185085
37,39	0,19	0,29	ENSG00000255432
0,83	0,04	0,08	ENSG00000278615
1,06	0,59	0,69	ENSG00000162194
0,84	0,10	0,17	ENSG00000214756
0,93	0,46	0,57	ENSG00000204922
1,63	0,00	0,00	ENSG00000162191
1,36	0,60	0,70	ENSG00000177363
2,99	0,00	0,00	ENSG00000168000
1,90	0,00	0,00	ENSG00000234857
1,69	0,00	0,00	ENSG00000162188
1,88	0,00	0,00	ENSG00000214753
1,60	0,00	0,00	ENSG00000162222
2,05	0,00	0,00	ENSG00000185670
1,36	0,00	0,00	ENSG00000168002
1,16	0,15	0,23	ENSG00000162227
1,58	0,00	0,00	ENSG00000168569
1,44	0,00	0,00	ENSG00000185475
1,64	0,00	0,00	ENSG00000162231
2,14	0,00	0,00	ENSG00000162236
2,58	0,00	0,01	ENSG00000283268
0,86	0,07	0,13	ENSG00000133316
1,21	0,02	0,04	ENSG00000168003
3,99	0,00	0,01	ENSG00000168539
#N/D	#N/D	#N/D	ENSG00000197901
#N/D	#N/D	#N/D	ENSG00000149452
#N/D	#N/D	#N/D	ENSG00000197658
1,14	0,97	0,98	ENSG00000196600

#N/D	#N/D	#N/D	ENSG00000184999
0,99	1,00	#N/D	ENSG00000149742
1,14	0,97	0,98	ENSG00000168004
0,50	0,25	0,36	ENSG00000133317
1,56	0,00	0,00	ENSG00000133321
0,98	0,99	0,99	ENSG00000133328
1,67	0,00	0,00	ENSG00000176485
2,00	0,00	0,00	ENSG00000184743
1,14	0,11	0,19	ENSG00000133318
1,25	0,02	0,05	ENSG00000188070
0,99	0,93	0,98	ENSG00000168005
1,25	0,01	0,02	ENSG00000072518
0,67	0,03	0,06	ENSG00000167771
1,48	0,00	0,00	ENSG00000110583
1,93	0,00	0,00	ENSG00000176340
1,39	0,00	0,00	ENSG00000167770
0,96	0,67	0,76	ENSG00000133315
0,27	0,00	0,00	ENSG00000126500
1,26	0,00	0,01	ENSG00000168439
2,13	0,00	0,00	ENSG00000149781
1,73	0,00	0,00	ENSG00000149743
2,16	0,00	0,00	ENSG00000149761
2,13	0,00	0,00	ENSG00000110011
1,92	0,00	0,00	ENSG00000173511
1,71	0,00	0,00	ENSG00000173486
3,35	0,00	0,00	ENSG00000286264
1,83	0,00	0,00	ENSG00000173457
1,57	0,00	0,00	ENSG00000149782
1,57	0,00	0,00	ENSG00000002330
1,38	0,00	0,00	ENSG00000173264
1,26	0,58	0,68	ENSG00000182450
2,24	0,10	0,17	ENSG00000219435
1,61	0,00	0,00	ENSG00000173153
1,33	0,00	0,00	ENSG00000173113
1,77	0,00	0,00	ENSG00000126432
1,56	0,00	0,01	ENSG00000168071
1,26	0,01	0,01	ENSG00000162302
1,89	0,45	0,56	ENSG00000168065
8,39	0,46	#N/D	ENSG00000197891
52,00	0,00	0,00	ENSG00000110076
0,36	0,35	0,46	ENSG00000068831
8,27	0,00	0,01	ENSG00000068976
0,92	0,31	0,43	ENSG00000168066
1,94	0,00	0,00	ENSG00000168067
1,14	0,11	0,18	ENSG00000133895
1,19	0,19	0,28	ENSG00000171219
1,77	0,00	0,00	ENSG00000110047
1,34	0,00	0,00	ENSG00000110046

3,01	0,00	0,00	ENSG00000068971
4,95	0,00	0,00	ENSG00000149735
22,89	0,26	0,37	ENSG00000168070
0,92	0,74	0,82	ENSG00000168062
1,45	0,00	0,00	ENSG00000213465
0,00	0,01	0,03	ENSG00000273003
0,74	0,00	0,00	ENSG00000110025
1,80	0,00	0,00	ENSG00000168061
4,22	0,00	0,00	ENSG00000168060
2,10	0,00	0,00	ENSG00000146670
2,84	0,00	0,00	ENSG00000162300
6,66	0,00	0,00	ENSG00000187066
1,75	0,00	0,00	ENSG00000149823
2,04	0,00	0,00	ENSG00000149809
1,36	0,01	0,01	ENSG00000174276
1,68	0,08	0,14	ENSG00000278952
1,62	0,00	0,00	ENSG00000149806
3,64	0,00	0,00	ENSG00000162298
1,74	0,00	0,00	ENSG00000149792
8,39	0,46	0,56	ENSG00000204710
1,77	0,00	0,00	ENSG00000014216
1,26	0,01	0,01	ENSG00000014138
0,76	0,00	0,01	ENSG00000285816
0,50	0,00	0,00	ENSG00000149798
0,92	0,33	0,44	ENSG00000133884
0,46	0,02	0,03	ENSG00000173825
0,67	0,00	0,00	ENSG00000162241
3,01	0,00	0,00	ENSG00000126391
1,47	0,00	0,00	ENSG00000142186
1,54	0,00	0,00	ENSG00000168056
#N/D	#N/D	#N/D	ENSG00000279093
1,76	0,00	0,00	ENSG00000173465
1,72	0,00	0,00	ENSG00000176973
1,65	0,00	0,00	ENSG00000173442
0,94	0,68	0,77	ENSG00000173338
1,60	0,00	0,00	ENSG00000173327
1,47	0,00	0,00	ENSG00000197136
0,64	0,00	0,00	ENSG00000213445
1,40	0,00	0,00	ENSG00000173039
1,55	0,00	0,00	ENSG00000172977
2,14	0,00	0,00	ENSG00000172922
1,16	0,08	0,14	ENSG00000254470
1,89	0,45	0,56	ENSG00000172818
1,68	0,00	0,00	ENSG00000172757
1,31	0,30	0,41	ENSG00000172803
1,22	0,02	0,04	ENSG00000172732
2,68	0,00	0,00	ENSG00000172638
2,17	0,00	0,00	ENSG00000172543

1,22	0,02	0,03	ENSG00000172500
1,52	0,00	0,00	ENSG00000175602
2,52	0,00	0,00	ENSG00000175592
1,79	0,00	0,00	ENSG00000175573
2,08	0,00	0,00	ENSG00000175550
0,36	0,35	0,46	ENSG00000175513
1,79	0,00	0,00	ENSG00000175467
2,64	0,00	0,00	ENSG00000175376
1,41	0,00	0,00	ENSG00000175334
0,36	0,35	0,46	ENSG00000175315
2,19	0,00	0,00	ENSG00000175294
3,05	0,00	0,00	ENSG00000175229
1,89	0,00	0,00	ENSG00000087365
1,47	0,00	0,00	ENSG00000175115
1,16	0,06	0,11	ENSG00000174996
2,37	0,00	0,00	ENSG00000174903
3,45	0,00	0,00	ENSG00000174871
2,53	0,00	0,00	ENSG00000174851
2,89	0,01	0,02	ENSG00000179292
0,78	0,61	0,70	ENSG00000174807
1,55	0,00	0,00	ENSG00000174791
1,39	0,00	0,00	ENSG00000174744
1,45	0,00	0,00	ENSG00000174684
0,79	0,54	0,64	ENSG00000174669
8,39	0,46	#N/D	ENSG00000174576
1,26	0,01	0,02	ENSG00000174547
1,80	0,00	0,00	ENSG00000174516
1,40	0,00	0,00	ENSG00000254986
4,34	0,00	0,00	ENSG00000256349
1,51	0,00	0,00	ENSG00000174483
2,55	0,00	0,00	ENSG00000174165
73,65	0,12	0,19	ENSG00000248746
8.890,79	0,00	0,00	ENSG00000174080
3,20	0,00	0,00	ENSG00000182791
1,69	0,00	0,00	ENSG00000173992
1,25	0,01	0,02	ENSG00000239306
2,03	0,00	0,00	ENSG00000248643
1,52	0,00	0,00	ENSG00000173933
2,24	0,00	0,00	ENSG00000173914
0,37	0,00	0,00	ENSG00000173898
1,09	0,44	0,55	ENSG00000173715
1,97	0,00	0,00	ENSG00000173653
1,56	0,00	0,00	ENSG00000173599
1,35	0,00	0,00	ENSG00000173621
0,39	0,00	0,00	ENSG00000173237
0,84	0,46	0,56	ENSG00000173227
1,32	0,00	0,00	ENSG00000173156
2,76	0,00	0,00	ENSG00000173120

1,44	0,00	0,00	ENSG00000173020
1,85	0,00	0,00	ENSG00000172932
1,88	0,00	0,00	ENSG00000172830
2,71	0,00	0,00	ENSG00000172613
1,65	0,00	0,00	ENSG00000175482
552,21	0,02	0,04	ENSG00000256514
1,06	0,55	0,66	ENSG00000175505
1,52	0,00	0,00	ENSG00000172531
2,66	0,00	0,01	ENSG00000175463
1,46	0,09	0,16	ENSG00000172508
1,56	0,00	0,00	ENSG00000175634
1,14	0,97	0,98	ENSG00000213402
1,71	0,00	0,00	ENSG00000172725
73,65	0,12	0,19	ENSG00000175514
71,39	0,00	0,00	ENSG00000175544
1,24	0,02	0,04	ENSG00000172663
1,58	0,00	0,00	ENSG00000110711
1,78	0,00	0,00	ENSG00000110697
3,76	0,00	0,00	ENSG00000167797
#N/D	#N/D	#N/D	ENSG00000167791
1,30	0,00	0,00	ENSG00000084207
1,50	0,00	0,00	ENSG00000167792
1,63	0,00	0,00	ENSG00000167799
6,99	0,05	0,10	ENSG00000167800
0,36	0,35	0,46	ENSG00000132744
#N/D	#N/D	#N/D	ENSG00000132746
2,04	0,00	0,00	ENSG00000110057
0,90	0,21	0,31	ENSG00000006534
1,42	0,00	0,00	ENSG00000110717
1,94	0,00	0,00	ENSG00000110719
1,40	0,00	0,00	ENSG00000110721
1,62	0,00	0,00	ENSG00000110066
2,04	0,00	0,00	ENSG00000171067
1,92	0,00	0,00	ENSG00000162337
1,03	0,68	0,77	ENSG00000110075
1,14	0,97	0,98	ENSG00000069482
8,39	0,46	0,56	ENSG00000132749
1,10	0,22	0,32	ENSG00000110090
1,13	0,16	0,25	ENSG00000197345
1,29	0,00	0,01	ENSG00000132740
0,13	0,46	#N/D	ENSG00000172938
2,32	0,01	0,02	ENSG00000172935
1,31	0,01	0,01	ENSG00000162341
1,48	0,06	0,10	ENSG00000287725
3,48	0,00	0,00	ENSG00000284713
0,98	0,90	0,96	ENSG00000172927
1,80	0,00	0,00	ENSG00000110092
1,31	0,00	0,01	ENSG00000149716

0,38	0,00	0,01	ENSG00000162344
#N/D	#N/D	#N/D	ENSG00000075388
1,14	0,97	#N/D	ENSG00000186895
3,05	0,00	0,00	ENSG00000131620
1,04	0,68	0,77	ENSG00000168040
0,96	0,62	0,72	ENSG00000131626
1,61	0,00	0,00	ENSG00000085733
0,54	0,00	0,00	ENSG00000162105
1,14	0,97	0,98	ENSG00000279459
#N/D	#N/D	#N/D	ENSG00000280089
3,16	0,00	0,00	ENSG00000172893
1,91	0,00	0,00	ENSG00000172890
1,14	0,97	#N/D	ENSG00000244411
0,07	0,31	0,43	ENSG00000241233
1,14	0,97	0,98	ENSG00000254997
0,13	0,46	#N/D	ENSG00000204572
#N/D	#N/D	#N/D	ENSG00000204571
1,92	0,00	0,00	ENSG00000289716
#N/D	#N/D	#N/D	ENSG00000184276
1,92	0,00	0,00	ENSG00000254469
#N/D	#N/D	#N/D	ENSG00000225805
2,02	0,00	0,00	ENSG00000137522
2,16	0,00	0,00	ENSG00000137496
1,39	0,00	0,00	ENSG00000137497
1,62	0,01	0,01	ENSG00000284922
1,47	0,00	0,00	ENSG00000184154
1,18	0,05	0,09	ENSG00000149357
1,14	0,97	0,98	ENSG00000284844
1,29	0,00	0,01	ENSG00000110200
0,16	0,07	0,12	ENSG00000110203
1,14	0,97	0,98	ENSG00000110195
8,39	0,46	#N/D	ENSG00000165457
1,77	0,00	0,00	ENSG00000165458
1,13	0,66	0,76	ENSG00000165462
1,19	0,03	0,06	ENSG00000162129
0,26	0,00	0,00	ENSG00000186642
1,22	0,02	0,04	ENSG00000186635
1,02	0,81	0,88	ENSG00000214530
1,46	0,00	0,00	ENSG00000168010
1,33	0,00	0,00	ENSG00000137478
0,07	0,00	0,00	ENSG00000175591
0,28	0,00	0,00	ENSG00000171631
1,79	0,00	0,00	ENSG00000110237
2,18	0,00	0,00	ENSG00000054967
2,35	0,00	0,00	ENSG00000054965
0,62	0,39	0,51	ENSG00000021300
1,64	0,00	0,00	ENSG00000175582
1,35	0,00	0,00	ENSG00000175581

1,28	0,00	0,01	ENSG00000181924
1,49	0,00	0,00	ENSG00000175575
0,89	0,71	0,80	ENSG00000187726
4,31	0,00	0,00	ENSG00000175567
0,97	0,93	0,98	ENSG00000175564
1,52	0,00	0,00	ENSG00000168014
1,46	0,00	0,00	ENSG00000214517
9,04	0,00	0,00	ENSG00000280269
1,62	0,12	0,19	ENSG00000149380
2,28	0,00	0,00	ENSG00000165434
0,04	0,20	0,30	ENSG00000175538
0,41	0,00	0,00	ENSG00000175536
1,68	0,00	0,00	ENSG00000077514
15,64	0,33	0,44	ENSG00000054938
0,02	0,11	0,18	ENSG00000279353
1,52	0,00	0,00	ENSG00000166439
1,34	0,00	0,00	ENSG00000166435
1,87	0,06	0,12	ENSG00000278879
1,45	0,00	0,00	ENSG00000118363
1,41	0,00	0,00	ENSG00000162139
0,99	1,00	1,00	ENSG00000171561
0,07	0,00	0,00	ENSG00000137491
167,91	0,06	0,11	ENSG00000261594
1,48	0,00	0,00	ENSG00000137486
1,14	0,97	0,98	ENSG00000279117
1,57	0,00	0,00	ENSG00000149273
1,01	0,92	0,98	ENSG00000149243
1,48	0,00	0,00	ENSG00000158555
1,55	0,00	0,00	ENSG00000149257
2,17	0,07	0,13	ENSG00000171533
1,14	0,97	#N/D	ENSG00000166391
0,41	0,00	0,00	ENSG00000062282
1,52	0,00	0,00	ENSG00000198382
0,60	0,47	0,58	ENSG00000085741
0,83	0,03	0,06	ENSG00000137492
1,28	0,01	0,02	ENSG00000179240
1,93	0,00	0,00	ENSG00000158636
1,91	0,35	0,47	ENSG00000137507
1,63	0,00	0,00	ENSG00000182704
0,84	0,05	0,09	ENSG00000078124
0,98	0,96	0,98	ENSG00000198488
2,70	0,00	0,00	ENSG00000149260
2,30	0,12	0,19	ENSG00000254550
2,76	0,00	0,00	ENSG00000137474
#N/D	#N/D	#N/D	ENSG00000178795
1,31	0,00	0,00	ENSG00000149269
0,95	0,54	0,65	ENSG00000074201
0,63	0,03	0,06	ENSG00000178301

1,03	0,76	0,84	ENSG00000048649
2,35	0,00	0,00	ENSG00000087884
1,87	0,00	0,00	ENSG00000149262
0,42	0,00	0,00	ENSG00000151364
1,10	0,60	0,70	ENSG00000259112
1,38	0,51	0,62	ENSG00000151365
1,31	0,00	0,01	ENSG00000151366
1,09	0,32	0,43	ENSG00000159063
1,46	0,00	0,00	ENSG00000188997
2,02	0,00	0,00	ENSG00000118369
0,56	0,00	0,00	ENSG00000033327
1,25	0,01	0,03	ENSG00000137513
0,36	0,00	0,00	ENSG00000149256
#N/D	#N/D	#N/D	ENSG00000279295
8,39	0,46	0,56	ENSG00000182103
1,52	0,00	0,00	ENSG00000137509
1,06	0,53	0,63	ENSG00000165490
1,72	0,00	0,00	ENSG00000137502
0,87	0,77	0,85	ENSG00000279900
0,89	0,16	0,24	ENSG00000165494
2,06	0,00	0,00	ENSG00000137494
0,83	0,06	0,11	ENSG00000137500
1,41	0,02	0,03	ENSG00000150672
0,80	0,03	0,06	ENSG00000171204
1,57	0,00	0,00	ENSG00000171202
1,85	0,00	0,00	ENSG00000137504
2,35	0,30	0,41	ENSG00000179071
4,79	0,00	0,00	ENSG00000137501
1,91	0,01	0,02	ENSG00000279742
1,14	0,97	0,98	ENSG00000150676
1,52	0,00	0,00	ENSG00000073921
1,08	0,41	0,53	ENSG00000074266
1,35	0,00	0,00	ENSG00000149196
2,05	0,02	0,04	ENSG00000149201
3,56	0,23	0,34	ENSG00000278989
0,89	0,34	0,46	ENSG00000151376
2,05	0,00	0,00	ENSG00000150687
[truncated: 408,904 more chars]
